# Genome analysis of the rice coral *Montipora capitata*

**DOI:** 10.1038/s41598-019-39274-3

**Published:** 2019-02-22

**Authors:** Alexander Shumaker, Hollie M. Putnam, Huan Qiu, Dana C. Price, Ehud Zelzion, Arye Harel, Nicole E. Wagner, Ruth D. Gates, Hwan Su Yoon, Debashish Bhattacharya

**Affiliations:** 1 Department of Biochemistry and Microbiology, Rutgers University, New Brunswick, NJ 08901 USA; 2 Department of Biological Sciences, University of Rhode Island, Kingston, RI 02881 USA; 3 Department of Ecology, Evolution and Natural Resources, Rutgers University, New Brunswick, NJ 08901 USA; 4 Department of Plant Biology, Rutgers University, New Brunswick, NJ 08901 USA; 5 Department of Vegetable and Field Crop Research, Institute of Plant Sciences, Volcani Center, ARO, Rishon LeZion, 7505101 Israel; 6 Hawai’i Institute of Marine Biology, Kāneohe, HI 96744 USA; 7 Department of Biological Sciences, Sungkyunkwan University, Suwon, 16419 Korea

## Abstract

Corals comprise a biomineralizing cnidarian, dinoflagellate algal symbionts, and associated microbiome of prokaryotes and viruses. Ongoing efforts to conserve coral reefs by identifying the major stress response pathways and thereby laying the foundation to select resistant genotypes rely on a robust genomic foundation. Here we generated and analyzed a high quality long-read based ~886 Mbp nuclear genome assembly and transcriptome data from the dominant rice coral, *Montipora capitata* from Hawai’i. Our work provides insights into the architecture of coral genomes and shows how they differ in size and gene inventory, putatively due to population size variation. We describe a recent example of foreign gene acquisition via a bacterial gene transfer agent and illustrate the major pathways of stress response that can be used to predict regulatory components of the transcriptional networks in *M*. *capitata*. These genomic resources provide insights into the adaptive potential of these sessile, long-lived species in both natural and human influenced environments and facilitate functional and population genomic studies aimed at Hawaiian reef restoration and conservation.

## Introduction

Coral reef ecosystems are ‘hotspots’ of marine biodiversity that are driven by complex biological interactions. These reefs generate immense productivity and economic value^1^ but are being pushed towards the brink of collapse by anthropogenic influences^2–4^. Recent mass bleaching and coral mortality on the Australian Great Barrier Reef (GBR) and worldwide has intensified the call to arms to expand knowledge at the cellular level to address the potential for acclimatization and adaptation through genetic, epigenetic, and symbiotic mechanisms^5^. Furthermore, the rate and extent of global reef loss have heralded a shift in management thinking to aggressive human intervention strategies to conserve and restore reefs to functional states^6,7^. Approaches such as assisted evolution, assisted gene flow^7^, and synthetic biology^8,9^ require genomic resources to inform and interpret mechanistic understanding, yet these resources, while growing^5,10^, are still relatively scarce. Currently, the majority of our understanding of coral genomic architecture comes from the cosmopolitan species *Acropora digitifera*^11^, *Stylophora pistillata*^10^, and *Pocillopora damicornis*^12^, and a handful of coral genomes at various stages of completion and availability (e.g., *Montastrea*, *Orbicella*, *Seriatopora*^5,10,13^). There remains, however, a dearth of genomic information across taxa ranging from environmentally susceptible to more resistant, and from resilient species that can inform us of natural adaptive potential and its utility in assisted evolution approaches.

**Figure 1:**
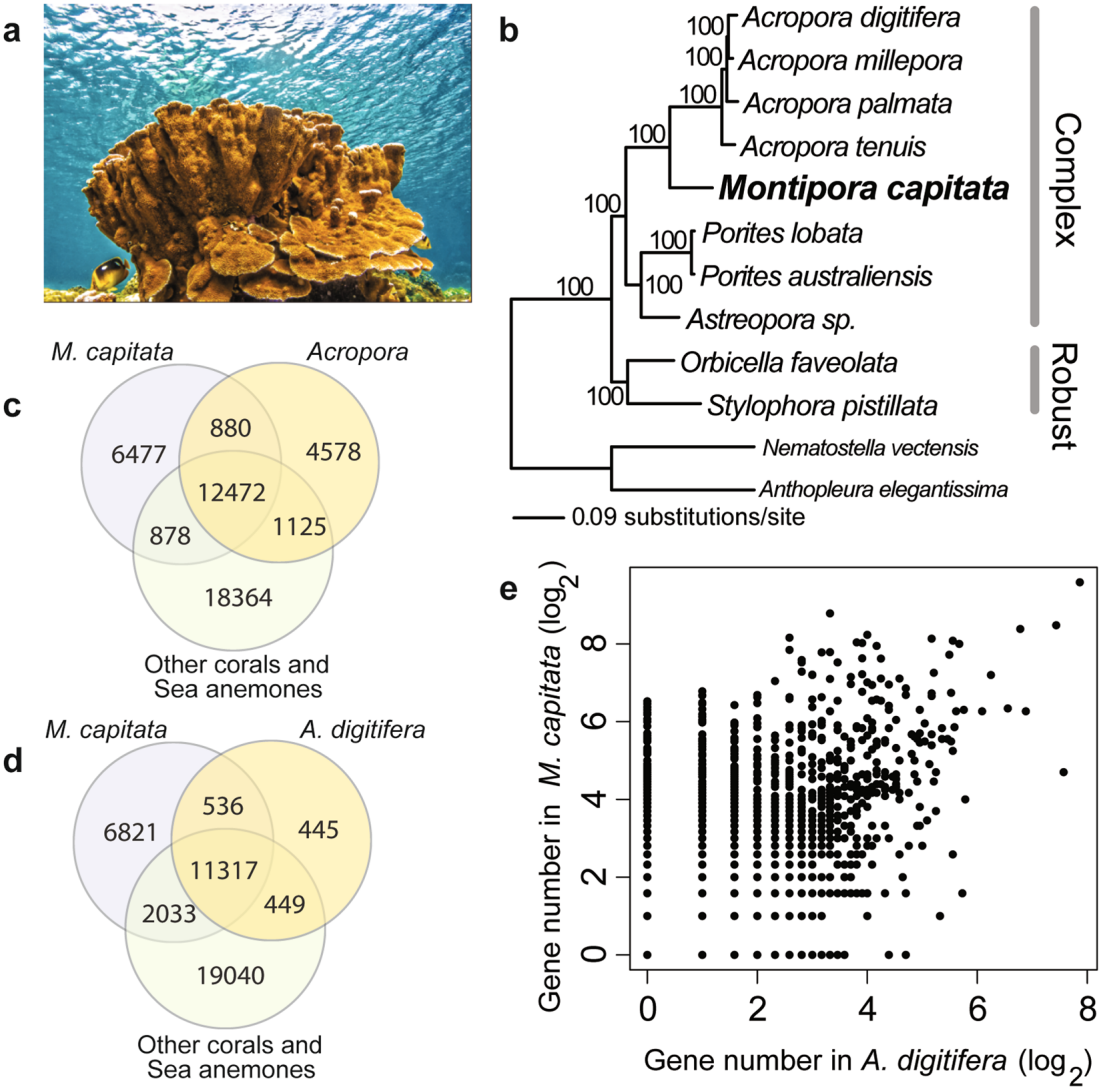
Genomic study of the rice coral *Montipora capitata*. (**a**) *M*. *capitata* colony photographed at Wai'ōpae, Southeast Hawai’i Island, that is ca. 0.25 m wide and of a similar height. Image provided by John Burns. (**b**) Maximum likelihood tree of 12 anthozoan species. The branch support values were estimated using 100 bootstrap replicates. (**c**) Venn diagram of coral gene families across *M*. *capitata*, *Acropora* species, and outgroups. (**d**) Venn diagram of coral gene families across *M*. *capitata*, *A*. *digitifera*, and outgroups. (**e**) Scatterplot of gene numbers in shared gene families between *M*. *capitata* and *A*. *digitifera*.

Additional resilience and restoration considerations include examining how low diversity reefs (e.g., in Hawai’i) may differ from well-connected sites with cosmopolitan species (e.g., GBR and the Coral Triangle). In light of this issue, we have targeted the environmentally robust rice coral, *Montipora capitata* (Fig. 1a), which is endemic to the Northwest and Main Hawaiian Islands. *M*. *capitata* is a broadcast spawning coral and dominant reef builder in lagoon and fringing reef sites throughout the archipelago, thus this species contributes substantially to ecosystem performance, goods, and services. Analysis of population genetic structure in *M*. *capitata* substantiates the existence of sexual reproduction, yet there are strong population disjunctions and high local recruitment^14^. Examination of stress tolerance of *M*. *capitata* populations reveals low sensitivity to ocean acidification and thermal stressors relative to other corals^15,16^. This species provides therefore an ideal opportunity to characterize the nuclear genome of a locally restricted coral to learn how genomic architecture and stress response differ between endemic and cosmopolitan lineages (i.e., *A*. *digitifera*).

## Results and Discussion

### Analysis of genome completeness and the predicted gene inventory

We isolated total sperm bundle DNA gathered from a single *M*. *capitata* colony located in a fringing reef near the Hawai’i Institute of Marine biology (HIMB, see Methods and)^17^. This DNA is predicted to represent a single diploid genotype although the gametes differ from each other due to independent meiotic recombination events between the two haploid somatic genomes. A library made from this DNA was sequenced with 60 “single molecule, real-time” (SMRT) cells using the PacBio platform, resulting in ca. 49 Gbp of data (6.6 million reads). We also generated 22.8 Gbp of Illumina HiSeq data to correct the long reads. The corrected PacBio sequences were assembled using phase-aware FALCON-Unzip^18^, resulting in a ~886 Mbp draft primary genome assembly comprising 3,043 contigs (N50 = 540,623 bp). The haplotigs, comprising the diverged regions of the coral genome, totaled ~312 Mbp in 3,411 contigs (N50 = 201,029 bp). To validate that the PacBio assembly represents a single diploid genotype, we aligned all Illumina reads (requiring that 95% of the read had a minimum of 95% identity) to the *M*. *capitata* primary assembly and ran a SNP analysis using the CLC Genomics Workbench. This procedure identified 1,088,903 high quality SNPs from non-repeated regions of the primary genome assembly. The distribution of allele frequencies at the SNP sites (Supplementary Fig. S1) resembles a classic diploid distribution, in which the peak is centered around the allele frequency of 0.5.

Nonetheless, because the *M*. *capitata* genome assembly is nearly double the size of some other sequenced corals (e.g., *A*. *digitifera*, ca. 448 Mbp, *Stylophora pistillata*, ca. 401 Mbp; see below) we inspected the assembly size. First, we used self-BLASTn to determine if the primary assembly was purged of all haplotigs. This analysis showed limited regions of high (>99%) DNA identify on different contigs (largest is 42 kbp), indicating that the primary assembly is largely free of haplotig data. Second, we again mapped the Illumina reads to the PacBio assembly, but this time at the higher stringency of 98% identity, resulting in 93.8% success, with the contigs having a uniform coverage of 23x. This information was used to infer genome size by dividing the sum of all base pairs of mapped Illumina reads (21,356,890,318 bp) by the average coverage (23x). This estimate of the *M*. *capitata* genome assembly size is 928,560,448 bp which is roughly comparable to the PacBio result. Third, use of the Illumina data to generate an independent assembly and its analysis also supports the larger genome size of *M*. *capitata* (for details, see Supplementary data). Interestingly, analysis of the genome of the coral *Platygyra daedalea* revealed a size of ca. 800 Mbp^19^ and our recent PacBio primary assembly of sperm DNA from a single Hawaiian *Porites compressa* colony is ca. 751 Mbp (DB, HMP, HSY unpublished data). We also inspected various *k*-mer spectra using the high-quality Illumina reads under the expectation of diploidy (http://kmergenie.bx.psu.edu) (Supplementary Fig. S2). These spectra using the predicted best *k* = 41 suggested a haploid genome size of ca. 523 Mbp. However, the impact of repetitive elements (described above) on this *k*-mer-based estimate likely explains the larger actual assembly size. These analyses suggest that, in spite of the inherent uncertainties associated with estimating genome size when large amounts of repeated DNA are present, the *M*. *capitata* haploid genome size we report is likely to be accurate and in line with data from other corals sequenced using long-read technology. In light of these insights, we used 978 conserved metazoan core genes as markers in a BUSCO analysis^20^ of the *M*. *capitata* primary assembly. This returned 887 (>90%) complete and 12 (1.2%) partial gene models, suggesting a relatively complete genome.

A total of 63,229 protein-coding genes were predicted using a combination of ab initio, evidence-based, and homology-based gene predictions (Table 1). Among them, 56,586 genes (89.4%, comprising 14,230 gene families [see details below]) share homology with at least one of the 11 other coral or sea anemone taxa in the phylogeny (Fig. 1b) (see below). Using proteins from three stony coral genomes (*A*. *digitifera*, *O*. *faveolata*, and *S*. *pistillata*) as reference, a majority of the *M*. *capitata* proteins showed completeness as indicated by the high alignment coverage against their closest homologs (Supplementary Fig. S3a). The 6,643 *M*. *capitata*–specific genes (10.6%, comprising 6,477 gene families, Fig. 1c) showed highly similar codon usage patterns to the core genes (Supplementary Fig. S3b). These results suggest an overall high quality of the predicted *M*. *capitata* gene models.

**Table 1:** Statistics for seven anthozoan genome assemblies.

Species	Genome size	Protein coding genes	Intron number	GC%
*Montipora capitata*	885,704,498	63,229	226,369	39.6%
*Acropora digitifera* ^a^	447,497,157	26,060	151,291	40.5%
*Orbicella faveolata* ^b^	485,548,939	25,916	157,289	41.9%
*Stylophora pistillata* ^c^	401,120,318	24,833	173,798	39.7%
*Pocillopora damicornis* ^d^	234,350,878	25,422	150,008	36.4%
*Exaiptasia pallida* ^e^	256,132,296	22,119	148,612	29.8%
*Nematostella vectensis* ^f^	297,398,056	24,773	106,993	40.6%

^a^NCBI Assembly name: Adig_1.1; ^b^NCBI Assembly name: ofav_dov_v1; ^c^NCBI Assembly name: GCA_002571385.1; ^d^NCBI Assembly name: GCA_003704095.1; ^e^NCBI Assembly name: *Aiptasia* genome 1.1; ^f^EnsemblMetazoa version 53.

To understand the basis of *M*. *capitata* gene inventory expansion when compared to *A*. *digitifera*, we studied orthologous gene families using a database of 12 anthozoan species (Fig. 1b). This analysis showed that comparable numbers of lineage-specific gene gains and losses are found between *M*. *capitata* (6,477 gains and 1,125 losses) and *Acropora* species (4,578 gains and 878 losses) (Fig. 1c). When *A*. *digitifera* was used for gene family enumeration (instead of all 5 *Acropora* species), we found a loss of 2,033 gene families in *A*. *digitifera* that predates the *Montipora*-*Acropora* split from other corals and sea anemones, compared to 449 gene family losses in *M*. *capitata* (Fig. 1d). Regarding the 11,853 gene families that are shared between *M*. *capitata* and *A*. *digitifera* (Fig. 1d), *M*. *capitata* has larger gene family sizes (Fig. 1e) and nearly twice as many genes (47,522) as *A*. *digitifera* (24,619). Similar results were obtained when *M*. *capitata* was compared to *Orbicella faveolata* and *Stylophora pistillata* (Supplementary Fig. S4) This observation explains the larger gene inventory in the *M*. *capitata* primary assembly. The top 20 gene families with the greatest size expansion in this species, when compared to three other coral species are listed in Table 2 and Supplementary Table S1. Many gene families are annotated as uncharacterized thus their functions are unknown. The remaining gene families are frequently associated with nucleotide processing functions such as polyprotein, DNA polymerase, and transposase. This result is consistent with the growth of *M*. *capitata* gene functions that are utilized by transposable elements (TEs) to increase their copy numbers in the genome using a ‘copy-and-paste’ mechanism via an RNA intermediate. Considering core eukaryotic genes that mapped to PFAM^21^ and KEGG pathways^22^, larger gene numbers were identified in *M*. *capitata* when compared to other stony corals (Supplementary Fig. S5). Gene family expansion also occurred in *M*. *capitata* lineage-specific genes, leading to 44 gene families with ≥2 members (284 genes) and 6,433 singletons. Given that orphan genes often result from gene duplication followed by accelerated evolution^23^, this result is likely an underestimate of the impact of gene duplications on the provenance of lineage-specific *M*. *capitata* genes.

**Table 2:** The top 20 gene families (orthogroups, OGs) that show the greatest expansion in the *M*. *capitata* genome assembly when compared to *A*. *digitifera*.

Representative gene	OG	*M*. *capitata* Count	*A*. *digitifera* Count	Fold change	Annotation
g11509.t1	OG0000083	92	1	92	5-hydroxytryptamine receptor 1-like
g10335.t1	OG0000184	88	1	88	Transposon polyprotein*
Monca.adi2mcaRNA34736_R8	OG0000188	83	1	83	Retrovirus-related Pol polyprotein*
Monca.adi2mcaRNA22274_R1	OG0000273	79	1	79	Integrator complex subunit 3-like*
Monca.adi2mcaRNA32317_R1	OG0000247	75	1	75	Uncharacterized protein
Monca.adi2mcaRNA782_R1	OG0000264	75	1	75	Uncharacterized protein
g10303.t1	OG0000239	75	1	75	Hypothetical protein
Monca.adi2mcaRNA17438_R1	OG0000263	73	1	73	Transposon Tf2–6 polyprotein*
Monca.adi2mcaRNA28536_R2	OG0000270	67	1	67	Uncharacterized protein
g10743.t1	OG0000298	67	1	67	Zinc finger CCHC domain
Monca.adi2mcaRNA13434_R2	OG0000427	64	1	64	Tcb2 transposase*
Monca.adi2mcaRNA20397_R5	OG0000369	59	1	59	ATP-binding cassette sub-family b
Monca.adi2mcaRNA20797_R1	OG0000159	110	2	55	Sentrin-specific protease 3
g1002.t1	OG0000442	52	1	52	Protein sidekick-2
Monca.adi2mcaRNA12306_R4	OG0000127	102	2	51	RNA-directed DNA polymerase*
g12976.t1	OG0000602	50	1	50	Uncharacterized protein
g13456.t1	OG0000465	48	1	48	Uncharacterized protein
Monca.adi2mcaRNA15668_R1	OG0000016	286	6	47	RNA-directed DNA polymerase*
Monca.adi2mcaRNA36640_R7	OG0000548	47	1	47	Uncharacterized protein
Monca.adi2mcaRNA27782_R0	OG0000452	46	1	46	Uncharacterized protein

Finally, to determine if the *M*. *capitata* genome may have undergone whole genome duplication (WGD) as a possible explanation for the larger gene inventory in this species, we used Cd-hit^24^ to query all predicted proteins against the full proteome to see if a cluster size of 2 (or another number) was predominant. This analysis, done at three different protein identity levels (90%, 70%, and 50% identity), all requiring 70% minimum query coverage to exclude heterologous sequences demonstrates that the dominant cluster size in *M*. *capitata* is one (Supplementary Fig. S6), providing an argument against WGD.

**Figure 2:**
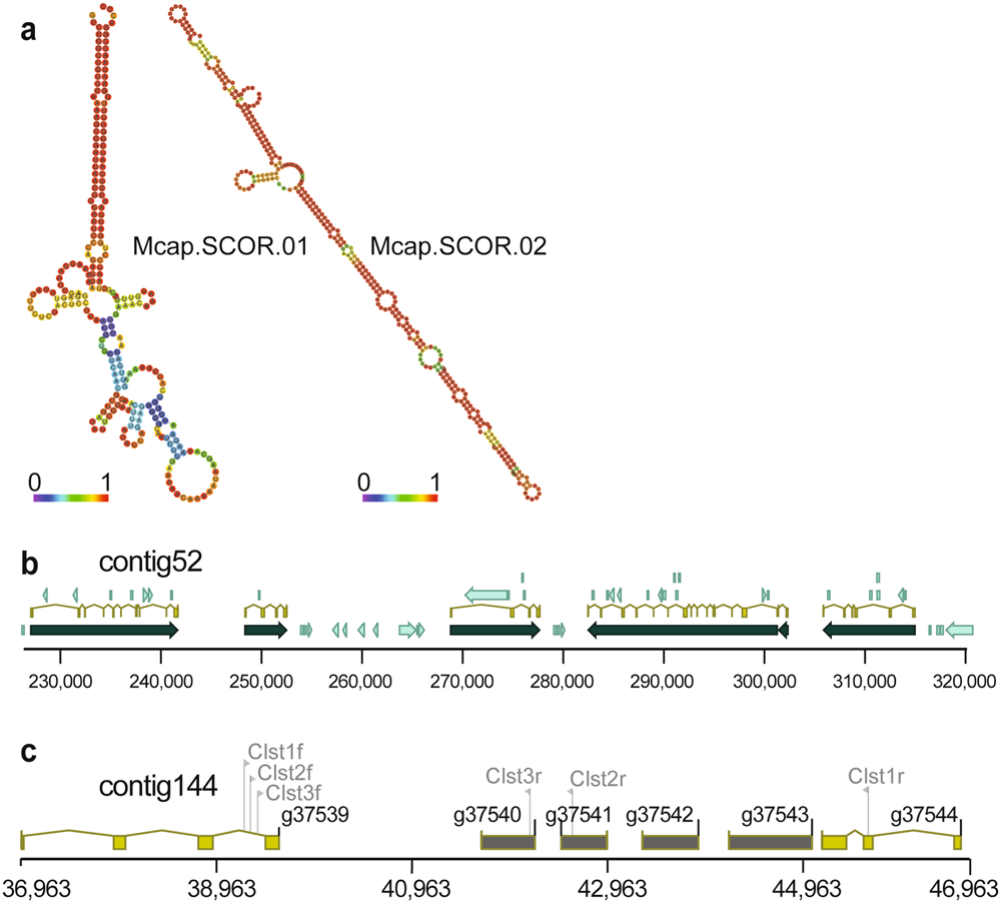
Evolutionary analysis of coral genomes. (**a**) Predicted secondary structures of two typical SCORs in the *M*. *capitata* genome (Methods). These were initially identified in transcriptomic data. The predicted minimum free energy (MFE) secondary structures are shown, with the colors corresponding to base-pairing probabilities (red is more stable). For unpaired regions, the color denotes the probability of being unpaired. Mcap.SCOR.01 is described in the text, whereas Mcap.SCOR02, a 572 bp long repeat has 9,758 copies accounting for 4Mbp of the *M*. *capitata* genome. This repeat is found 4,582 times in the intron regions of 4,035 genes, and 5,176 copies are in intergenic regions. These target genes include ras-specific guanine nucleotide-releasing factor, centrosome-associated protein, indole-3-acetaldehyde oxidase, and a G-protein coupled receptor. (**b**) Example of a *M*. *capitata* genome contig showing its complex structure. Genes are in dark green, intron-exon structures are in yellow, and intron and UTR-encoded SCORs are in light green. (**c**) Location of the bacterium-derived 4-gene cluster in *M*. *capitata* genome contig144. The coral (animal) genes and HGT candidates are shown in light brown and dark grey filled boxes, respectively.

Beyond gene family growth, the major reason for genome size increase in *M*. *capitata* is the massive expansion of repeats. De novo repeat-family identification, done using RepeatModeler turned up 2,239 repeat families that account for 46% (408,047,463 bp) of the *M*. *capitata* primary genome assembly. A total of 1,684 of these families were classified as unknown repeats, ranging in length from 100 bp to 14.6 kbp. Most significantly, we found an extensive collection of highly conserved, anciently derived, Scleractinia COral-specific (i.e., absent from all other eukaryotes and prokaryotes) Repeat families (SCORs) that are present in the genomes of studied corals (20 species)^25^. SCORs form complex secondary structures (Fig. 2a) and are located in untranslated regions and introns, but most abundant in intergenic DNA. For example, Mcap.SCOR01 (Fig. 2a) is 240 bp in length and present in 5,502 copies, summing to 1.2 Mbp in the *M*. *capitata* genome. About one-half of these copies (2,846) are inserted in the intronic regions of 2,277 genes that include a variety of functions such as dynein heavy chain 2, axonemal-like, acetyl-CoA carboxylase, sodium bicarbonate transporter, F-box DNA helicase 1. The remainder of the hits is in intergenic regions. SCORs have undergone frequent duplication and degradation, suggesting a ‘boom and bust’ cycle of invasion and loss^25^. We speculate that due to the high sequence identities of a small fraction of the shared family members (most are too diverged to be compared) across anciently diverged corals, physical association with genes, and dynamic evolution, some SCORs might have adaptive functions in coral^25^. No correlation was found however between the differentially expressed (DE) genes reported in this study (under heat and *p*CO_2_ conditions described below) and the composition of SCORs in these genes. In addition, we used whole genome bisulfite sequencing to address the hypothesis that methylated DNA was preferentially associated with SCORs and would act to silence TEs. These preliminary data do not however support this hypothesis because methylated CpGs are represented by <1% of the SCOR-encoding bases (Supplementary data).

In the *M*. *capitata* genome, SCORs have spread throughout most genic regions, making gene prediction very challenging (e.g., Fig. 2b). Furthermore, SCORs are uniformly distributed in intergenic and genic regions (Supplementary Table S2) and the analysis of SCOR prevalence with respect to intron length per gene shows a strong positive correlation between these parameters (Supplementary Fig. S7). This observation suggests that the spread of SCORs in *M*. *capitata* contributes to genome size expansion. As explanation for the latter, we propose that the spread of repeats and TEs in this coral reflects a population bottleneck at the Hawaiian site. *M*. *capitata* is an endemic species and previous analyses show low pelagic larval recruitment across the Hawaiian Archipelago, with most sites having limited genetic diversity (>90% self-recruitment;)^14^. In a previous study of *M*. *capitata* at the same Kāneohe Bay, O’ahu, Hawai’i site^17^, we found evidence for a major and a minor (introgressed from *M*. *flabellata*) mitochondrial haplotype, incomplete rDNA repeat homogenization, but little to no sequence variation among single-copy nuclear genes in 5 studied colonies. These multiple sources of genome data provided no evidence of nuclear gene chimerism and were consistent with a recently diverged population that retained (i.e., not yet homogenized by concerted evolution) ancestral rRNA genotypes. As is widely appreciated, population size is an important factor in genome size growth or shrinkage. The drift-barrier hypothesis for mutation-rate evolution^26^ predicts that effective population size and genetic drift govern the strength of selection on trait evolution. Under this hypothesis, smaller populations undergo greater genetic drift and therefore, elevated genome-wide mutation rates (e.g., base substitutions, insertions, deletions, invasions by repeat elements). In the case of *M*. *capitata*, this appears to manifest itself in the spread of mobile repeats such as SCORs^25^. In contrast, larger more cosmopolitan populations, such as found with *A*. *digitifera* are presumably under stronger selection leading to more efficient removal of deleterious mutations^27^. Analysis of plant genomes supports this view with TE-derived genome size growth occurring in these taxa independent of polyploidization^28,29^.

### Coral phylogeny and evidence of recent horizontal gene transfer

Using 211 single-copy orthologous genes (total of 54,795 aligned amino acids) that are conserved across 10 corals and two sea anemone species, we built a phylogeny that shows 100% bootstrap support for all interior nodes (Fig. 1b). This tree is consistent with a coral species tree previously built using a smaller dataset^5^ and places *M*. *capitata* within the complex corals as sister to *Acropora* species, all of which form a sister clade to robust corals^30^. Using this reference phylogeny as a framework, we searched for recent horizontal gene transfers (HGTs) in the *M*. *capitata* genome that have occurred since its split from *Acropora* species. Analysis of phylogenomic data (see Methods) resulted surprisingly, in the identification of a single candidate, a bacterium-derived 4-gene cluster (g37540–37543) in genome contig144 (Fig. 2c). The HGT candidates are flanked on both sides by sequences of eukaryotic (metazoan) origin and the bacterial genes are putatively of proteobacterial provenance (Supplementary Fig. S8). The absence of spliceosomal introns in these transferred genes (Fig. 2c) and their physical clustering is consistent with a recent transfer into the *M*. *capitata* genome. This hypothesis was validated using PCR amplification followed by sequencing of the resulting products that span the coral-bacterial gene boundaries (Fig. 2c). Mapping of the Illumina paired-end (75 × 75 bp) reads to contig144 showed uniform coverage across the regions encoding the HGTs, arguing against an assembly artifact (Supplementary Fig. S9). A search against the whole genome sequence (WGS) assembly available from 17 Cnidaria taxa using NCBI BLAST turned up no significant hits to these four genes. We tested if the HGT candidates are expressed under the different temperature and *p*CO_2_ conditions used here (see below) and found that all four genes are expressed, albeit at low levels (Supplementary Table S3a). It is unclear if these recently acquired HGTs have recruited regulatory elements for gene expression and are involved in important functions in the host. Nonetheless, these results provide direct evidence of HGT in corals, that, over their long evolutionary history has contributed adaptive traits such as protection from UVR and stress from reactive species^5^, many of which are lineage-specific, as demonstrated here.

Regarding the mechanism of bacterial gene integration, a likely vector is a gene transfer agent (GTA). GTAs are phage-like genetic elements produced by some prokaryotes that can drive HGT of random DNA segments from the host cell to a recipient, usually from the same population^31^. GTAs package host DNA 4–14 kbp in size, with the best studied GTA in *Rhodobacter capsulatus* transferring ca. 4 kbp fragments^32^. The bacterial region in the genome of *M*. *capitata* bears the hallmarks of a GTA transfer because: (1) it is about 4 kbp in size (Fig. 2c); (2) the donor appears to be a *Pseudovibrio* sp. (or *Jannaschia* sp.; see Supplementary Fig. S8) that are alpha-proteobacteria known to harbor GTAs^31^; (3) *Pseudovibrio* species are associated with sessile marine taxa such as sponges and corals^33^, and may form mutualisms with these lineages, providing antimicrobials as defense against predation and disease^34^; and (4) *Pseudovibrio* species encode type IV secretions systems that are able to deliver factors (DNA or proteins) from the cytoplasm of the donor to the recipient cell^35^. This first evidence of a putative GTA-derived region in a coral genome likely involved a bacterial symbiont that had long-term residency in the coral holobiont. The 4-gene bacterial cluster in *M*. *capitata* has no significant hits in the data available at NCBI.

### Comparison of protein divergence in *M*. *capitata* and *A*. *digitifera*

**Figure 3:**
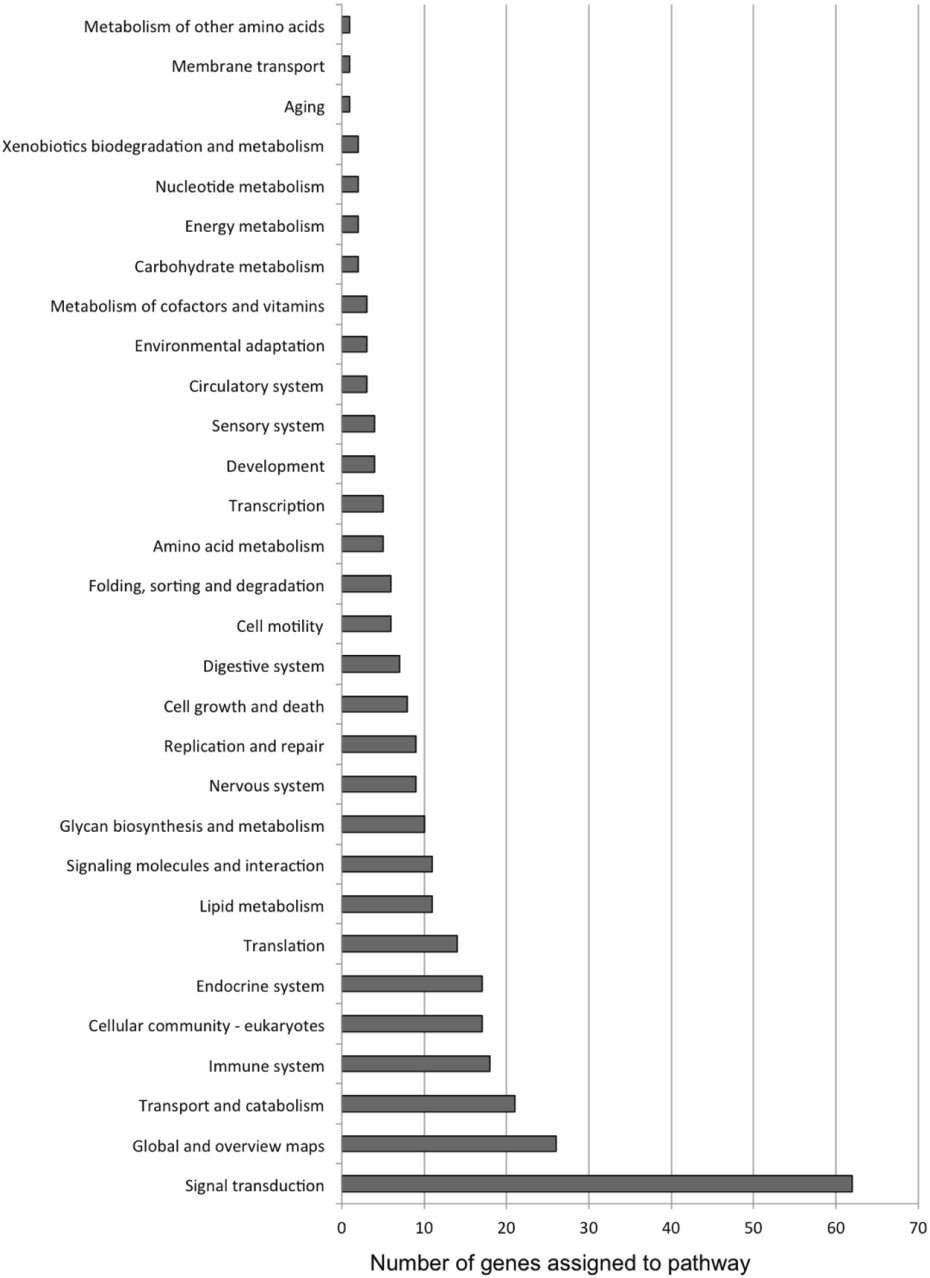
Results of selection analysis. Distribution of the top 10% of genes under diversifying selection in the *M*. *capitata* - *A*. *digitifera* comparison with respect to placement in KEGG pathways.

We tested if particular *M*. *capitata* genes are under diversifying selection when compared to *A*. *digitifera*. Specifically, we hypothesized that stress responses such as signaling pathways or genes integral to symbiosome (compartment that houses the algal symbiont) formation may be targets of natural selection when comparing an endemic and a cosmopolitan lineage. For this approach, we calculated pairwise dN values for 12,196 single-copy ortholog groups derived from the coral protein data. From this list, the top 1,220 (10%) proteins with the highest dN values were selected and annotated prior to Gene Ontology (GO) assignment and placement in broader KEGG pathway maps (Supplementary Table S4). Examination of the KEGG data (Fig. 3) reveals that by far the largest number of KO terms from the fast-evolving set (23.5% by number, excluding “Global and overview maps”) are assigned to pathways of “Signal transduction”. Specifically, the interconnected phosphatidylinositol 3′-kinase (PI3K)-Akt, Rap1, Ras, and MAPK signaling pathways contained the most members (Supplementary Fig. S10A–D). The PI3K-Akt pathway phosphorylate substrates involved in apoptosis, protein synthesis, metabolism, and the cell cycle^36^ and may thus have a role in dinoflagellate symbiont selectivity, maintenance, and breakdown. Both the Ras and Rap1 pathways rely on GTPases that, when bound to GTP, trigger various signaling cascades. Ras is predominantly associated with cell proliferation, differentiation, and cytoskeletal organization often via PI3K effectors, whereas Rap1 is associated with cell-cell and cell-matrix interactions and also regulates MAP kinases (MAPK; itself a signaling molecule integral to the cell cycle). It should be noted that many of the proteins/KO terms composing these four pathways are shared (i.e., K02583, K04362, K05089, K05093) and thus it is perhaps more appropriate to consider them constituents of a meta-signaling pathway resulting in cellular differentiation.

Both Notch and Wnt signaling pathways (Supplementary Fig. S10E,F) regulate cell identity and differentiation, and are likely to have a role in the immune response^37^; The Wnt pathway is a broadly conserved signaling cascade that regulates progenitor cell proliferation during development, including innate immune cells. Of note, both the Frizzled (FZD) receptor and LRP-5/6 (LRP) cell surface receptors that bind Wnt protein and initiate the pathway are present in our test set. Notch signaling, in addition to cell identity, regulates processes such as apoptosis and wound regeneration^37^. Both innate immunity and apoptosis are vital pathways governing post-phagocytic recognition of symbiont acquisition^38^. Reminiscent of the Wnt pathway, our test set contained proteins encoding the ligands that physically bind Notch and initiate the pathway, but few of the remaining downstream enzymes.

The “Transport and catabolism” class is comprised predominantly of KO terms assigned to the *Endocytosis* and *Lysosome* pathways (Supplementary Fig. S10G,H). Both pathways are strongly integrated into dinoflagellate symbiont selection and uptake, because *Symbiodinium* cells are acquired via phagocytosis and persist in the symbiosome (a fused phagosome/lysosome structure). Among the individual proteins in the set are integral lysosomal membrane components (LAMP/LIMP) and the Niemann-Pick type C1 protein (NPC1), which has recently been shown to localize to the symbiosome in anthozoans^39^. Additionally, we find that both caveolin-1 (a structural component of membrane invaginations known as caveolae) and the Src kinase that targets cav1 are represented. To our knowledge, the role of caveolae in the symbiosis has not yet been explored, however significant up-regulation of both NPC1 and caveolin in symbiotic vs. aposymbiotic *Aiptasia* larvae was previously reported^40^.

The “Immune system” class contained multiple proteins assigned to the *Nod-like receptor signaling pathway* (Supplementary Fig. S10I). Nod-like receptors (NLRs), including Nod1 and Nod2, function in peptidoglycan recognition of invasive bacteria as part of the innate immune system. Notably, our test set included a homolog of SGT1, which activates Nod1 directly^41^. The immune system, and specifically the Nod-like receptor complex, has been implicated in the establishment of a functional coral symbiosis^42^ and thus protein evolution among pathway constituents (coupled with immune cascades initiated by *Signal transduction* pathways above) may reflect a diverging cellular response to and recognition of different algal endosymbionts.

Examination of the *RNA transport* pathway (Supplementary Fig. S10J) shows that 3/7 nuclear pore complex (NPC) cytoplasmic filament subunits are present in the test set. The NPC filaments are hypothesized to interact with RNA-protein cargo crossing the nuclear pore^43^. Additionally, RNaseP and TGS1 enzymes are present that are responsible for 5′ end modification of tRNAs and snRNAs, respectively. These modifications may be commensurate with any modifications to the NPC filaments. We also note that the *Circadian rhythm* pathway (Supplementary Fig. S10K) contains a single yet important homolog of the CLOCK gene that encodes a transcription factor central to regulation of the circadian clock. Both the coral and algal symbionts exhibit complex diel rhythms with regard to calcification and reproduction (in the host) and photosynthesis and cell division (in the alga), however the mechanisms responsible for any synchronization of circadian rhythm between the two remain unknown^44^. Additionally, the timing of gamete release and/or coral broadcast spawning is critical and varies with coral population and environmental conditions^45^ and thus CLOCK may play an integral role in divergent coral circadian phenotypes.

The Fisher’s exact test identified ten over-represented gene ontology (GO) terms in our test set (Supplementary Table S4): *proteinaceous extracellular matrix*, *growth factor activity*, *collagen trimer*, *RNA polymerase I transcription factor complex*, *synaptonemal complex assembly*, *response to pheromone*, *copper ion transmembrane transporter activity*, *copper ion transmembrane transport*, *polynucleotide 5′-hydroxyl-kinase activity* and *cell-matrix adhesion*. Three of these terms (*proteinaceous extracellular matrix*, *collagen trimer*, *cell-matrix adhesion*) share a common set of proteins that implicate constituents of the extracellular matrix. Similarly, both terms involving copper ion transport contain the same two Cu^2+^ transporters. Exposure to elevated levels of Cu leads to oxidative stress in scleractinian corals^46^ and thus modification of the transport system may reflect adaptation to changing ocean chemistry. The *growth factor activity* ontology contained multiple proteins annotated as “balbiani ring 3-like isoform”, however, this was likely an artifact resulting from C-terminal spacing of cysteine residues because these proteins encode *vascular endothelial growth factor* (VEGF) domains via the NCBI Conserved Domain Database.

### Transcriptome analysis of the *M*. *capitata* stress response

A detailed accounting of the results of the *p*CO_2_ RNA-Seq analysis is presented in the Supplementary data (see also Supplementary Table S3). Briefly, the sampled *M*. *capitata* colonies and colony fragments had a period of recovery and acclimation in flow-through tanks located at HIMB, and then were exposed to either ambient temperature and ambient *p*CO_2_ (ATAC; 27.4 °C, ~472 µatm), ambient temperature and high *p*CO_2_ (ATHC; 27.8 °C, ~823 µatm), or high temperature and ambient *p*CO_2_ (HTAC; 29.8 °C, ~376 µatm). We performed RNA-Seq on three biological replicate samples collected after one and six hours of exposure to each treatment and initially used DESeq2 to identify DE transcripts in comparisons between each stress condition and the control. The results show that thermal challenge elicits a larger transcriptional response (e.g., in comparison to the ATAC control after six hours of exposure, we identified 100 DE transcripts in the ATHC samples and 1,542 in the HTAC samples) compared to the high *p*CO_2_ condition. With regard to the acute thermal challenge, a total of 55 transcripts with BLASTx hits to known proteins were differentially expressed in HTAC/ATAC comparisons at both time points. Among these are homologs of various transcription factors (e.g., *AP-1* and *c-Fos*, *MafB*, *Traf3*), transcriptional regulators—particularly those involved in regulating NF-κB activation and activity (*Tnip3*, *Sik1*), heat shock proteins and co-chaperones, proteins involved in regulation of cell proliferation (*Tob1*, *Btg2*), and proteins involved in calcium sensing and calcium homeostasis (*Calumenin*, *Cml19*).

Among the transcripts up-regulated after one hour of exposure in the HTAC treatment are homologs of proteins involved in membrane lipid metabolism, arachidonic acid biogenesis, and the production of various signaling molecules (e.g., leukotrienes, eicosanoids) via arachidonic acid metabolic pathways (*cPLA2*, *Alox5*). A number of transcription factors are up-regulated, including homologs of *CrebH* and *Xbp1* which are known to induce the expression of acute phase response (APR) and unfolded protein response (UPR) genes in the endoplasmic reticulum^47,48^. Among the down-regulated transcripts are homologs of proteins that may be involved in regulation of intracellular trafficking and transport (*Ift172*, *Kif3a*, *Klhl20*), autophagy, apoptosis (*Casp3*), NF-κB signaling and control of cell differentiation and cell cycle progression (*Ankrd52*, *Msx-2*, *Rit1*). Together, the post-one-hour snapshot of the *M*. *capitata* response to acute thermal challenge highlights the differential expression of metabolic pathways that form signaling molecules, regulation of activation and activity of major stress-responsive transcription factors such as pro-inflammatory NF-κB, up-regulation of components of the unfolded protein response and down-regulation of mediators of cell growth and development.

**Figure 4:**
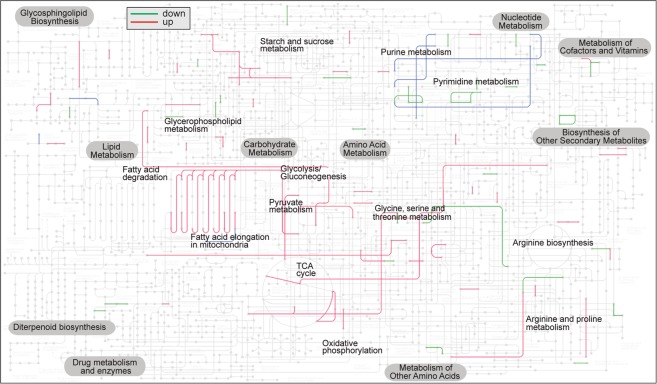
The distribution of significantly up- and down-regulated genes in the HTAC 6 hr treatment when mapped on the KEGG global metabolic pathway. The green and red lines show genes that were significantly down- and up-regulated, respectively. The blue lines indicate genes that show both types of effects, likely due to the existence of gene paralogs with differential expression. Image created using KEGG Mapper^80^ at the KEGG website^22^.

Among the up-regulated transcripts in the HTAC group after six hours of exposure includes genes in the global KEGG metabolic map that are involved in fatty acid and amino acid metabolism (Fig. 4). More specifically, GO enrichment analysis reveals overrepresentation of GO terms of the Biological Process (BP) category associated with *transcriptional regulation* (GO:0016192), *positive regulation of proteasomal ubiquitin-dependent protein catabolic process* (GO:0032436) and *protein ubiquitination involved in ubiquitin-dependent protein catabolic process* (GO:0042787), *regulation of immune response* (GO:0050776), *mitogen-activated protein kinase* (*MAPK*) *cascade* (GO:0000165) (Supplementary Fig. S11), *autophagy* (GO:0006914) and *regulation of apoptotic process* (GO:0042981) (Supplementary Fig. S12). Overrepresented BP GOs among the down-regulated DEGs are associated with *DNA replication* (GO:0006260). After six hours of thermal challenge, *M*. *capitata* appears to shift resources from unfolded protein recuperation to autophagy and protein degradation. Up-regulation was observed for homologs of proteins involved in autophagy and autophagosome formation (*Atg2a*, *Dram2*), lysosome-associated cathepsins (*CtzL/Z*), and a number of ubiquitin-protein ligases that may target proteins for degradation *via* ubiquitination.

Prolonged exposure to experimental stress conditions, in particular high temperature, may “flip the switch” between cell survival and apoptotic cell death pathways in *M*. *capitata*. The differential expression analysis reveals upregulation of multiple genes after six hours of heat stress that are associated with MAPK activity (Supplemental Table S4, Supplementary Fig. S11). The role of MAPK pathway in sensing and coping with environmental stress is well-known in eukaryotes (e.g., coping with heat, cold, oxidative, UV, osmotic, and dehydration mediated stresses in plants^49)^. Specific examples include transgenic tobacco plants that express a constitutively active MAPKKK (activator of the MAPK pathway), resulting in enhanced heat tolerance^50^. Deletion of (hog1) MAPK in *Saccharomyces cerevisiae* results in slow recovery from heat stress^51^ and there is higher mortality in mutants of the (PMK-1) MAPK-pathway under heat stress in *Caenorhabditis elegans*^52^. Considering the important role of the MAPK pathway in regulating the response to heat stress across many domains of life, it is likely that a similar role is played in corals. Homologs of several regulators of apoptosis (e.g., *apoptosis-inducing factor 2*, *Bax*) were up-regulated. *Bax* can stimulate the release of cytochrome C from the mitochondria and contribute to the activation of *caspase-3*^53^. Several homologs of eukaryotic translation initiation factors (eIFs) were up-regulated. However, a homolog of *Eif2ak3*, which inactivates *eIF2* by phosphorylation — and thus contributes to global down-regulation of protein synthesis – was also up-regulated. DNA and amino acid synthesis appear to have been impaired as well, as homologs of both subunits of ribonucleoside-diphosphate reductase (*Rrm1* and *Rrm2b*), as well as a homolog of *dihydrofolate reductase*, were identified among the down-regulated transcripts. Finally, a homolog of *caspase-3*, an effector of apoptosis in the execution phase was up-regulated. Notably, we identified in the comparison between HTAC and ATAC treatments after six hours of exposure is the number of differentially-expressed transcripts that are homologous to genes derived from mobile elements (e.g., *Jockey/pol*, *Tigd4*, *Gin1*, *Pgbd4*). Of 15 transcripts with BLASTx hits to these elements, nine were up-regulated and six were down-regulated. A homolog of the piRNA biogenesis protein *Exd1*, thought to function as an RNA-binding adapter in the PIWI-EXD1-Tdrd12 (PET) complex which mediates piRNA biogenesis and may act to suppress transposon transcripts^54^, is down-regulated. Another example at the 6 h time-point of the HTAC treatment is the 2.62-fold up-regulation of gene adi2mcaRNA5483_R6 that encodes a putative RNA-directed DNA polymerase. Also notable were the up-regulation (3.74-fold) of a potential toxin component in the ATHC treatment at the 6 h time-point (i.e., PI-stichtotoxin-She2a; a secreted aspartic-acid type endopeptidase) that carries a strong secretory signal (gene adi2mcaRNA20544_R0; *P* = 0.983, TargetP 1.1). The potential contributions of toxins and TE proteins to climate change-associated stress conditions remain to be investigated.

**Figure 5:**
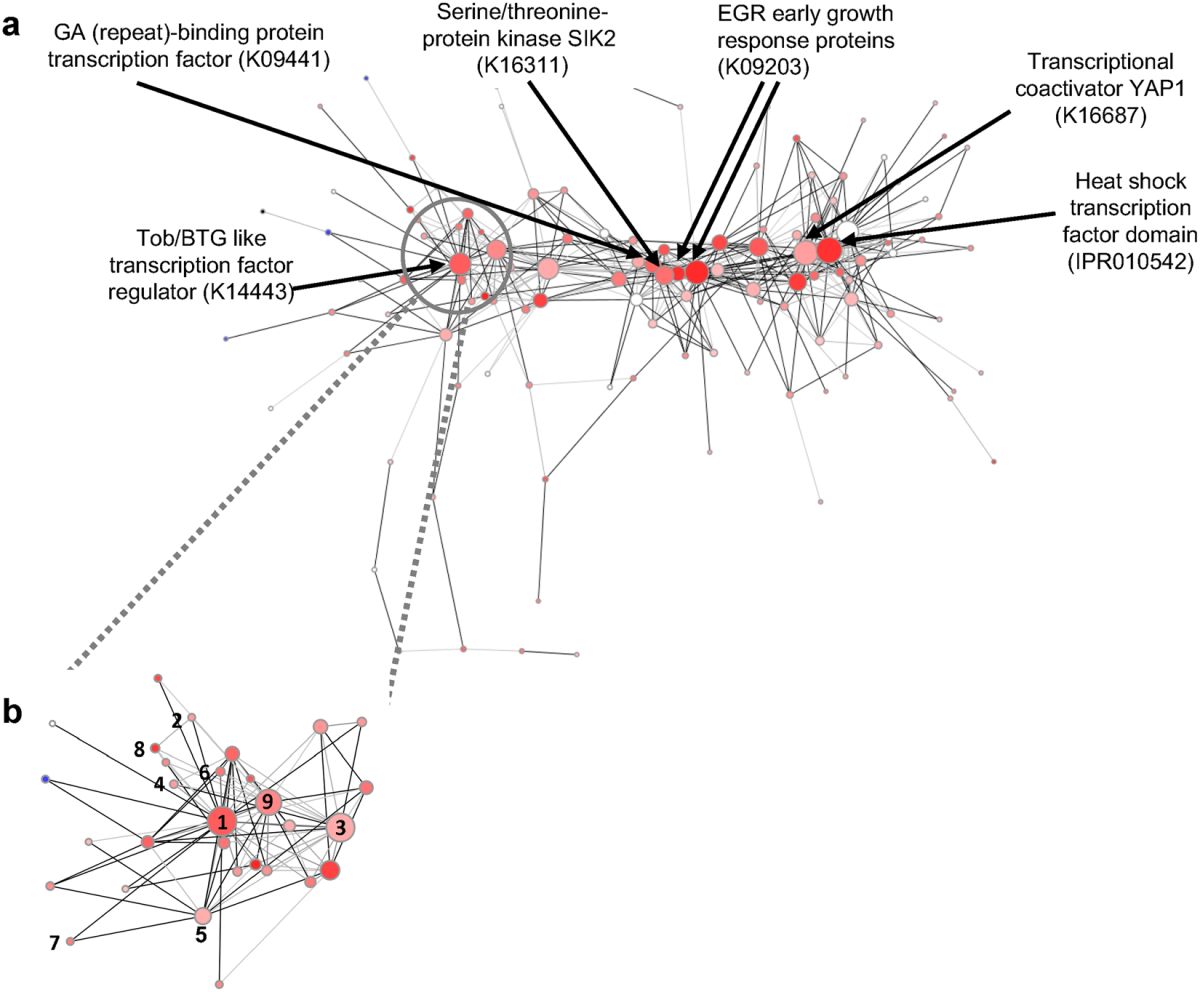
Network analysis in *M*. *capitata* of gene co-expression under heat stress. (**a**) Network topology of one WGCNA module (significantly enriched with up-regulated genes following 1 hr heat stress over 1 hr ambient control, *p*-value < 0.05, Fisher’s exact test, Methods). Each node represents a gene, and edges represent correlations between them. Major hubs (annotated nodes) are identified by the top 10% of degree and betweenness values in the network. Node size is directly correlated with degree (number of adjacent neighbors). Dark to light connections indicate high to low co-expression coefficients, respectively. Node color is correlated with the DESeq2 characterized heat 1 hr vs. ambient 1 hr contrast of significantly differentially expressed genes (i.e., gradient of dark red to light pink indicate highest to lowest over expression after 1 hr heat exposure; similarly, the gradient of dark blue to light blue indicates under expression). (**b**) Enlargement of the network region containing Tob1/Tob2 like transcription factor regulators. Dark to light red/blue nodes indicate high to low over/under expression, respectively, after 1 h of heat exposure. Legend for network hubs: 1. BTG/Tob- transcription factor regulators (K14443); 2. Metal regulated homodimeric repressors (IPR011991); 3. Zinc finger (Znf) domain participating in binding DNA, RNA, protein and/or lipid substrates (IPR013087); 4. Cysteine- serine-rich nuclear proteins (CSRNP) - transcription activator (K17494); 5. cAMP response element-binding protein (CREB)- transcription factor (K09050); 6. Rab30 (GTPases) regulators of intracellular membrane trafficking (K07917); 7. RAN- GTPase involved in nucleocytoplasmic transport of proteins and RNA through the nuclear pore complex (K07936); 8. Cytosolic phospholipase A2 (PLA2G4)- initiating lipid-based regulation of immune response, and other intracellular pathways (K16342). 9. Proteinase inhibitor I35 domain (IPR001820). All node annotations depicted in both (A and B) panels were generated by identifying significantly enriched (*p*-value < 0.05, Fisher’s exact test) KEGG orthologs (indicated by Kxxxxx) or InterPro domains (indicated by IPR0xxxxx).

Next, we used weighted gene co-expression network analysis (WGCNA^55^; see Supplemental data for methods) to identify gene modules that co-participate in the heat stress (ATAC vs. HTAC) response and their major regulatory components. Analysis of the RNA-Seq data identified 10 co-expression modules, seven of which were found to contain a significant enrichment (see module preservation in the Methods) of KEGG orthologs, InterPro domains, and GO terms (Supplementary Table S5). Network topology analysis was done to identify regulatory points, or hubs in the network. This approach assessed network parameters of centrality such as degree (i.e., number of connections of one node with other nodes) and betweenness (i.e., connectivity of a node between other nodes-pairs that are not connected [capacity to act as a link]) to identify transcriptional hubs that may act as regulatory components of the transcriptional networks. Our results with regard to 1 h of heat stress, when compared to the ambient control, identified a module of genes (green set, Supplementary Fig. S13) that putatively act as a master regulatory network in *M*. *capitata* (Fig. 5a). Enriched with significantly over-expressed genes at 1 h of heat stress (*p*-value < 0.05, Fisher’s exact test, see *Identification of significant modules* in the Supplementary data), this module contains several hubs of regulatory elements that are primarily involved in transcription. One of these hubs contains a paralog of the mammalian Tob1/Tob2 family (indicated with 1 in Fig. 5b) known to interact with other proteins, and in turn to regulate transcription factors, and (poly-A) deadenylation-mediated mRNA turnover^56^. Interestingly, this hub contains additional transcription factors, in addition to components governing other means of regulation such as intracellular membrane trafficking, nucleocytoplasmic transport of proteins and RNA, lipid-based regulation, and protein inhibition (indicated by 6, 7, 8, and 9 respectively in Fig. 5b). Some members of this hub (approximately 20%) are unannotated. Their membership in this module suggests a potential role as regulators of the early heat stress response. Similarly, in the turquoise module, which is enriched with significantly down-regulated genes following 6 h of heat stress (*p*-value < 0.05, Fisher’s exact test, see *Identification of significant modules* in the Supplementary data), our analysis demonstrates a highly connected protein hub containing a S-adenosyl-L-methionine-dependent (SAM) methyl transferase domain (Supplementary Fig. S14). SAM-binding methyltransferases utilize the ubiquitous methyl donor SAM as a cofactor to methylate proteins, small molecules, lipids, and nucleic acids, thereby regulating a variety of processes. This protein is connected with proteins containing domains involved in cationic amino acid transport, the binding and potential regulation of RNA, Ca^2+^-dependent membrane-targeting, suggesting a potential direct (or indirect) down-regulation of these functions by this methyltransferase. These findings open new avenues for future studies of the role of methylation in the coral heat-stress response.

## Conclusions

Corals are immensely complex meta-organisms in which the domains of life converge (i.e., animal, algal symbiont [eukaryotic], the bacterial and archaeal microbiome, and virome) but knowledge about their interactions is incomplete across the many different taxa under study. The major goal of this work was to generate a high-quality genome assembly of the ecologically important species *M*. *capitata* that will serve as a platform for studying low diversity reefs at multiple ecological and genetic levels. Analysis of the genome data suggests that its major features (e.g., genome size, repeat content) likely reflect a population bottleneck at the Kāneohe Bay site. The finding of recent putative GTA-derived HGT in this species provides direct evidence that the prokaryotic microbiome can contribute to the coral germline. Analysis of protein divergence between the endemic *M*. *capitata* and the ubiquitous *A*. *digitifera* shows that diversifying selection has had the greatest impact (i.e., 23.5% of KO terms in the fast-evolving set) on signal transduction pathways, followed by genes involved in transport and catabolism. We interpret these results, as well as the list of over-represented GO-terms as indicating the wide breadth of selective constraints that need to be considered when interpreting the evolutionary trajectories of these two divergent (i.e., an endemic and a cosmopolitan) coral species.

Genes that underwent diversifying selection (Fig. 3, Supplementary Fig. S10) are not numerically prominent targets of DE under heat or pH stress. Our analysis showed that only 96/1,220 fast-evolving genes show DE (e.g., heat 1 h: 21 up-regulated, 8 down-regulated; heat 6 h: 56 up-regulated, 15 down-regulated). These results suggest that when comparing the data on a gene-by-gene level, diversifying selection and differential gene expression under the tested conditions are not strongly correlated in *M*. *capitata*. However, the fact that many of the same conserved pathways are impacted by both processes (e.g., MAPK and RAS signaling and lysosome) suggests that these are targets for selection in these divergent coral lineages. The DE work also indicates that 6 h of heat stress (i.e., 2 °C increase) can trigger the apoptotic cascade in the coral and is likely to be relevant to bleaching events from projected ocean warming in the coming years. Future studies should also focus on transcriptional responses to sub-bleaching perturbations with the sampling of more time points at shorter intervals to improve resolution of the dynamics of the ecologically-relevant *M*. *capitata* heat stress response. Overall, with ongoing studies of microbiome dynamics in *M*. *capitata* in response to coral bleaching and expanded methylome and stress response analyses, we hope ultimately to erect a model that integrates these lines of evidence to foster better understanding and conservation of these important coral reef ecosystem engineers.

## Materials and Methods

### Preparation of DNA

*M*. *capitata* colony 628 from Kāneohe Bay, O’ahu Hawai’i was sampled on June 5, 2016 (Special Activity Permit 2015–17). We collected a mixture of egg sperm bundles immediately upon their release and the sperm fraction was removed by pipetting to a new tube and was cleaned by a series of three rinse-and-spin steps, with samples rinsed with 0.2 µm filtered seawater and centrifuged at 13,000 rpm for 3 min and snap frozen in liquid nitrogen. Sperm bundles were ground to a powder in liquid nitrogen and DNA was extracted with the Qiagen Genomic -tip 100/G kit according to the manufacturers’ instructions. DNA concentrations were measured on a Qubit instrument and the sample sent to the DNA Link Sequencing Lab^57^ for sequencing on a PacBio RS II instrument. These data were assembled using FALCON-Unzip (done by DNA Link). The options used for this assembly are as follows: length_cutoff = 12000, length_cutoff_pr = 8000, falcon_sense_option = –output_multi–min_idt0.70–min_cov4–max_n_read200–n_core24, overlap_filtering_setting = –max_diff60–max_cov60–min_cov2–n_core24.

### Prediction of protein-coding genes

The *M*. *capitata* RNA-Seq data (see below and Supplementary data for details) derived from different temperature treatments was mapped to the *M*. *capitata* genome assembly using STAR^58^. The *M*. *capitata* genome assembly and the RNA-Seq mapping result were used for *ab initio* and evidence-based gene prediction using Braker with the default setting^59^. After the annotation of repetitive elements, we re-run the *ab initio* gene prediction using Augustus^60^ with the Braker-derived HMM matrix (*M*. *capitata*-specific parameters), the information of intron-exon boundary (supported by ≥10 reads in RNA-Seq mapping data) and coordinates of repetitive elements. Gene models with in-frame stop codons and those encoding coding sequences with atypical codon usage were discarded. We also carried out homology-based gene prediction using *Acropora digitifera* gene models as guidance. *A*. *digitifera* gene models annotated by the NCBI eukaryotic genome annotation pipeline from NCBI Genome database were downloaded, and the proteins were mapped to the *Montipora capitata* genome assembly using tBLASTn (*e*-value = 1e^−20^). The *A*. *digitifera* gene structure information, tBLASTn outputs, and RNA-Seq mapping results were used for homology-based gene prediction with the Gene Model Mapper (GeMoMa)^61^. Finally, the two sets of gene models were merged with priority given to Augustus-derived gene models. GeMoMa-derived gene models were used when two or more non-overlapping Augustus gene models overlapped with the same GeMoMa gene models. In these cases, the Augustus gene models (likely partial predictions) were replaced with the corresponding longer GeMoMa gene models. When single gene models corresponding to the same locus were found, the GeMoMa gene model was used only when its completeness (with respect to *A*. *digitifera* homologs) was >5% higher than the corresponding Augustus gene model. Finally, the GeMoMa gene models without overlapping Augustus gene models were added to the predicted gene set. The resulting proteins were filtered against the repeat library (*e*-value = 1e^−20^) resulted from RepeatModeler, resulting in 63,229 gene models (Table 1).

### Repeat identification and genome masking

Repeats were identified using RepeatModeler^62^ and classified using the latest Repbase version^63^. To eliminate any repeat redundancies the identified repeat sequences were clustered using Cd-hit^24^ with a cutoff of 85% identity. The remaining repeat sequences that were classified as “unknown” were compared against the NCBI non-redundant protein database using BLASTX (*e*-value < 10^−5^), any repeat sequence with a hit was discarded from the final repeat set. The predicted repeats were masked from the *M*. *capitata* genome using RepeatMasker^64^ under default parameters.

### Construction of the coral super-gene phylogeny

We collected proteomes from 12 anthozoan species (Fig. 1b). For each species, the highly similar sequences (identity >95%) were removed using Cd-hit. The resulting proteomes were used to build orthologous gene families with OrthoFinder v1.1.8^65^ under the default settings. For each single-copy orthologous gene family, the corresponding sequences were retrieved and aligned using MUSCLE v3.8.31 under the default settings^66^. The alignments were then trimmed using TrimAl (version 1.4) in automated mode (-automated)^67^ and then ‘polished’ with T-COFFEE (version 9.03) to remove poorly aligned residues (conservation score ≤5) among the aligned blocks. We also removed columns with ≥30% missing data and partial sequences with ≥50% missing data. The resulting 211 single-gene alignments (length ≥120 amino acids) were concatenated into a super-alignment for phylogeny construction. The maximum likelihood tree was built using RAxML version 7.2.8 under the LG + Γ + F model. The supporting values were estimated using 100 bootstrap replicates^68^.

### *M*. *capitata* lineage-specific HGTs

We searched the *M*. *capitata* protein sequences using UBLAST with an *e*-value cut-off (=1e^−05^) against a comprehensive local protein database that comprises NCBI RefSeq database and proteins derived from genomes or transcriptomes of 20 coral species (Ref58 + Coral)^5^. Sequences with a top-hit from corals or any other metazoan species were removed. The remaining sequences were subjected to our phylogenomic pipeline to produce phylogenetic tree for each query following a similar procedure described in a previous study^5^. Briefly, the *M*. *capitata* protein sequences were used as query to search against the “Ref58 + Coral” database^5^ using BLASTp (*e*-value = 1e^−5^). Up to 1000 top hits (query-hit identity ≥30%) were recorded. Representative sequences were then selected from the BLASTp outputs (sorted by bit-score by default) in a “first-come-first-served manner” with no more than 6 sequences for each phylum. The BLASTp hits were then re-sorted according to query-hit identity in a descending order among those with query-hit alignment length (≥200 amino acids). And a second set of representative sequences was generated. The two sets of representative sequences were then combined and aligned using MUSCLE version 3.8.31 under default settings and trimmed using TrimAl version 1.4 in an automated mode (-automated1). Alignment positions with ≥50% gaps were discarded. The resulting alignments were used for phylogenetic tree building using FastTree version 2.1.7^69^ under the ‘WAG + CAT’ model with four rounds of minimum-evolution SPR moves (-spr 4) and exhaustive ML nearest-neighbor interchanges (-mlacc 2 -slownni). The resulting trees were then manually examined to screen genes that were likely derived from non-metazoan sources.

### PCR validation of HGT cluster presence in the genome

In order to determine whether the observed four-gene cluster of potential HGT-derived bacterial genes was an artifact or assembly or a result of contamination, several primer pairs were designed to target and amplify (1) the segment of the genomic contig that contains the cluster lying between the flanking eukaryotic genes (Clst1), (2) a portion of the Clst1 product extending from one of the flanking eukaryotic genes to the g37541 gene within the cluster (Clst2), and (3) a portion of the Clst1 product extending from the flanking eukaryotic genes to the g37540 gene within the cluster (Clst3). Primer sequences are provided in the Supplemental data. Amplified products were sent to GENEWIZ (South Plainfield, NJ, USA) for Sanger sequencing. After inspection of the waveforms and editing for quality, sequences were aligned with the genome using CLC Genomics Workbench^70^.

### Test of selection

It has been shown that the common metric for natural selection, the Ka/Ks (or dN/dS) ratio, that normalizes the non-synonymous substitution rate (dN) by the background mutation (or synonymous) substitution rate (dS) is unreliable when applied to closely-related organisms^71^. They however found that dN alone remains stable for quantifying fast and slowly-evolving proteins. This is predominantly due to the different algorithms used to estimate dS in a maximum-likelihood framework and can also be heavily influenced by sequence composition^71^ and by segregating polymorphisms at the population level (neutral and slightly deleterious) that have yet to be fixed or purged from the genome, post-divergence^72^. Because even small stochastic variation in synonymous substitution rates coupled with artifacts in dS calculation can have a disproportionately large influence on the selection signature^73^, we used Ka (dN) as our primary metric of protein evolution in the gene-by-gene comparisons.

Single-copy ortholog groups (OGs; i.e., containing a single representative from both taxa) were identified using OrthoFinder within the predicted proteomes of *M*. *capitata* (this work) and *A*. *digitifera*^74^. The two sequences from each OG were aligned using MAFFT^75^, and the corresponding nucleotide CDS codons were then aligned using TranslatorX^76^ with the protein alignment as a guide. Any sites containing gaps in either species were removed from the codon alignment, and the KaKs Calculator v2.0^77^ was used to calculate Ka, Ks and Ka/Ks (or dN, dS and dN/dS) values for each alignment under model averaging (i.e., averaging parameters across all candidate substitution models). The top 10% of proteins ranked descending by Ka value were selected and annotated (using the *A*. *digitifera* protein as a query) against the KEGG automatic annotation server^78^ and additionally with BLAST2GO^79^. To test for functional enrichment, a Fisher’s exact test was performed in BLAST2GO using the GO terms present in the set of fast-evolving proteins as a test set, and those present in the remainder of the single-copy orthologs as a reference. GO terms with a single test *p*-value < 0.05 were considered significant and retained. KEGG pathway maps^80^ created using the test set as input were manually examined for the presence of multiple members or particular proteins and/or enzymes proximal to each other that may indicate a focus of protein evolution or divergence.

### Transcriptome analysis

*Sample Collection:* Coral branch fragments (~6 cm^2^) were collected June 2016 from *M*. *capitata* colonies (one per colony) from the fringing reefs in Kāneohe Bay (latitude 21.429782, longitude -157.792586) under SAP 2017–28. Fragments were each separately placed in seawater-filled bags and transported immediately to the HIMB, where they were placed in a 1,300 L common garden tank with flowing seawater^15^. Coral nubbins were formed by attaching the broken skeleton fragments to plastic frag plugs, using non-toxic hot glue. Nubbins were acclimated for 15 days under ambient environmental conditions including natural diurnal fluctuations in pH (NBS 8.02 ± 0.01, light 141 ± 14µmol quanta m^−2^ s^−1^), and temperature (26.68 ± 0.02 °C). These values represent mean ± sem from measurements logged in the tanks every 15 min.

#### Experimental Design

Following the recovery and acclimation period, multiple branch fragments were randomly allocated to replicate treatment tanks. Tanks were assigned, in triplicate, to one of three conditions: Ambient Temperature Ambient *p*CO_2_ (ATAC; 27.4 °C, ~472 µatm), Ambient Temperature High *p*CO_2_ (ATHC; 27.8 °C, ~823 µatm), and High Temperature Ambient *p*CO_2_ (HTAC; 29.8 °C, ~376 µatm). The treatments are designed to allow for fluctuations that mimic those observed in the surrounding bay. The treatment and control conditions were maintained using a pH-stat CO_2_ injection system^15^. pH probes from the pH-stat CO_2_ injection system were calibrated weekly (NBS scale). Carbonate chemistry was assessed with direct measurements of pH (total scale), total alkalinity, temperature, and salinity. Total alkalinity samples were quantified through open cell potentiometric titrations^81^ and assessed against certified reference materials (CRMs; A. Dickson Laboratory, UCSD; values on average <1% different from TA CRMs); From these measurements, carbonate parameters were calculated using the seacarb package (v3.0.11)^82^. At 0 minutes of treatment exposure, a fragment was removed from each of the ATAC replicate tanks, placed in a sterile Whirlpak and immediately snap-frozen in liquid nitrogen. At 60 and 360 minutes of treatment exposure, a fragment was removed from each of the ATAC, ATHC, and HTAC replicate tanks to be immediately snap-frozen in liquid nitrogen. Frozen samples were shipped in a liquid nitrogen charged dry shipper to the Bhattacharya Laboratory at Rutgers University-New Brunswick for extraction, library preparation, and sequencing.

#### DNA/RNA Extraction and Library Preparation

Frozen coral branch fragments were fractured using a flame-sterilized hammer and chisel. To homogenize the samples, fractured coral pieces were placed in 2 mL Eppendorf tubes with 600 µL Buffer RLT Plus (Qiagen) lysis buffer and ~100 µL 0.5 mm zirconia/silica beads (BioSpec Products) and vortexed for 5 minutes. Cell debris and coral skeletal fragments were pelleted by centrifugation and the lysate was removed for RNA extraction using the AllPrep DNA/RNA/miRNA Universal Kit (Qiagen). Complementary DNA (cDNA) libraries were prepared from RNA extracts using the TruSeq RNA Sample Prep Kit v2 (Illumina).

#### Sequencing and Processing of Sequence Data

A total of 21 cDNA libraries were sequenced on an Illumina MiSeq platform using 75 × 75 paired-end cycle kits. A total of 487,890,058 sequence reads from 21 cDNA libraries were imported into CLC Genomics Workbench 8.5.1^70^, wherein reads were trimmed for quality and any contaminating Illumina adaptor sequences removed. After trimming, a total of 423,573,416 reads remained (371,925,330 paired reads and 51,648,086 orphan reads).

#### Analysis of Differential Gene Expression

In order to increase the number of uniquely-mapping reads when mapping the libraries to the predicted protein-coding gene models, Cd-hit was used to cluster the 64,351 gene models at a similarity threshold of 0.97. The resulting 51,424 gene models were imported into CLC Genomics Workbench 8.5.1 and used as the reference for mapping the 423,573,416 trimmed reads using the RNA-Seq Analysis tool. A count matrix, recording the number of unique reads mapping to each of the 51,424 gene models per library, was constructed. Differential expression analyses were conducted with the DESeq. 2 package^83^ in R^84^. For each of the 21 libraries a “Group” designation was assigned, combining treatment and time point information (ex: samples taken after 1 hour of exposure to the ATHC treatment were designated “ATHC_1”), and the design formula of the DESeqDataSet was designated “~Group”. Results were extracted (alpha = 0.05) for the following comparisons: (1) ATHC_1 vs. ATAC_1, (2) ATHC_6 vs. ATAC_6, (3) HTAC_1 vs. ATAC_1, and (4) HTAC_6 vs. ATAC_6. Gene models were considered to be differentially expressed in a comparison if the model (i) had an FDR-adjusted p-value < 0.05 and (ii) had a Log2FoldChange estimate |*x*| ≥ 1.

#### Characterization of uncharacterized DEGs using TargetP and SignalP

Differentially expressed predicted gene models with no BLASTx best hits, or best hits to uncharacterized or hypothetical proteins, as well as those with no hits at all, were investigated using TargetP^85^ which assigns subcellular localization predictions by searching for targeting signals within amino acid sequences. Sequences were submitted to TargetP using the Non-Plant “Organism Group” criteria and the default “winner-takes-all” option for cutoffs. TargetP assigns a “reliability class” (RC) descriptor for its predictions ranging from 1 to 5, with 1 representing the strongest predictions. When analyzing the results of the TargetP predictions, only those sequences with predictions having RC ≤ 3 were considered. Sequences that had a TargetP prediction of “secretory pathway” or “other” were analyzed using SignalP^86^ for a more detailed prediction of secretory signals.

#### Gene Ontology Enrichment Analysis

Nucleotide sequences and associated BLASTx results were imported into Blast2GO. The Mapping tool in Blast2GO was used to map Gene Ontology (GO) terms to the gene models. GO terms were assigned to 10,441 of the 51,424 gene models. GO enrichment analyses for the gene models exhibiting differential expression between treatments at each time point were performed using Fisher’s Exact Test as implemented by Blast2GO using an FDR threshold of 0.05. The full set of 51,424 was used as the background set for set for these analyses.

## Supplementary information

Supplementary Information

## Data Availability

The genome data and analyses cited in this work are available at http://cyanophora.rutgers.edu/montipora and the *M*. *capitata* PacBio reads, Illumina HiSeq genomic reads, and RNA-Seq libraries are available via NCBI BioProject PRJNA509219.
