# Supplementary material for: Genome analysis of the rice coral Montipora capitata
Source: Sci Rep. 2019 Feb 22;9:2571. doi: 10.1038/s41598-019-39274-3 (PMC6385260; doi:10.1038/s41598-019-39274-3)
Supplement: Supplementary file 1 — Supplementary Information [file 41598_2019_39274_MOESM1_ESM.pdf]

Supplementary Information

Genome analysis of the rice coral *Montipora capitata*

Running title:

Rice coral genome

Authors:

Alexander Shumaker,[1†] Hollie M. Putnam,[2†] Huan Qiu,[3†] Dana C. Price,[4] Ehud Zelzion,[3] Arye Harel,[5] Nicole Wagner,[3] Ruth D. Gates,[6] Hwan Su Yoon,[7] *Debashish Bhattacharya[1]

Affiliations:

[1]Department of Biochemistry and Microbiology, Rutgers University, New Brunswick, NJ 08901, USA.

[2]Department of Biological Sciences, University of Rhode Island, Kingston, RI 02881, USA.

[3]Department of Ecology, Evolution and Natural Resources, Rutgers University, New Brunswick, NJ 08901, USA.

[4]Department of Plant Biology, Rutgers University, New Brunswick, NJ 08901, USA.

[5]Department of Vegetable and Field Crop Research, Institute of Plant Sciences, Volcani Center, ARO, Rishon LeZion 7505101, Israel.

[6]Hawai'i Institute of Marine Biology, Kāneohe, HI 96744, USA.

[7]Department of Biological Sciences, Sungkyunkwan University, Suwon 16419 Korea.

[†]Equal contribution.

Corresponding author:

Debashish Bhattacharya

Rutgers University, Department of Biochemistry and Microbiology

59 Dudley Road, 102 Foran Hall

New Brunswick, NJ 08901, USA

Tel: +1 (848) 932-6218

Fax: +1 (732) 932-8965

Email: d.bhattacharya@rutgers.edu

Supplementary Data

Genome analysis of the rice coral *Montipora capitata*

Alexander Shumaker,[1†] Hollie M. Putnam,[2†] Huan Qiu,[3†] Dana C. Price,[4] Ehud Zelzion,[3] Arye Harel,[5] Nicole Wagner,[3] Ruth D. Gates,[6] Hwan Su Yoon,[7] Debashish Bhattacharya[1]

Illumina data-based genome size estimation

Assembly of the high-quality Illumina data alone using the CLC Genomic Workbench (default parameters) resulted in 404 K contigs, summing to ca. 520 Mbp (N50 = 3,273 bp; maximum scaffold size = 87 kbp). To explore the discrepancy in assembly sizes between the two platforms, we mapped the Illumina reads at the same stringency to the Illumina assembly, and only 65% were recovered. The largest 300 Illumina contigs had an average coverage of ca. 26x, and analysis of all contigs with an arbitrary value of >40x coverage (32,236 contigs) had an accumulated length of 24 Mbp. Given 26x coverage of the largest, repeat-free regions, we used this standard across all contigs with >40x coverage (i.e., 432 contigs had >1000x coverage, with the highest being >42 K) to approximate repeat contribution to the *M. capitata* genome. This resulted in the original 24 Mbp, representing repeated DNA that could not be properly assembled, summing to 120 Mbp. The combined unique and repeated data generated using the Illumina data now summed to 640 Mbp. Given that the 93.8% of these short-read data mapped to the PacBio assembly but only 65% to the Illumina assembly, this suggests that 35% (8.1 Gbp) of the Illumina reads were not present in the native assembly. This 35%, under the assumption of 26x coverage would comprise ca. 320 Mbp, which, when added to the 640 Mbp is close to the size of the PacBio assembly of 886 Mbp. These calculations suggest that the size of the *M. capitata* Illumina-based assembly is likely to be an under-estimate, due to the well-known inability of short-read data to account for highly repeated regions.

Analysis of PFAM and KEGG annotations

The database of HMMs built for protein families was downloaded from PFAM (release 32.0, https://pfam.xfam.org/). PFAM domains in coral proteins were searched using pfam_scan.pl script under the default settings (version 1.6, ftp://ftp.ebi.ac.uk/pub/databases/Pfam/Tools/). Hits (e-value <= $1e^{-5}$) were removed and the remaining domains were used for counting. KEGG pathway genes were searched using KAAS online server (https://www.genome.jp/kegg/kaas/) [1]

with BLAST against the ‘eukaryotes’ database. The single-direction best hit for queries were retrieved.

Codon usage analysis

The codon count data from the *M. capitata* coding sequences were subjected to within-group correspondence analysis [2,3] implemented as 'dudi.coa' and 'within' functions in the ADE-4 R package [4]. Only long, coding sequences (≥225 nucleotides) were included in the analysis. The coordinates of the input sequences at the first and second axes were recorded and used for the plots.

PCR validation of HGT cluster in the M. capitata genome

To determine whether the four-gene cluster of bacterium-derived genes on genomic contig144 was an artifact of assembly or the result of contamination, a pair of primers (Clst1f: 5’-CAATGTGCCGATGTAATGTGACTG-3’; Clst1r: 5’-AAATGTCCTGAAGATACCCTGTCC-3’) was designed to target segments within the two eukaryotic genes (g37539 and g37544) flanking the cluster. A second set of primers (Clst2f: 5’-ACCCGCTGTGCTACATTACTTT -3’; Clst2r: 5’-AGTGCCTTCAGATACATTCTCTC -3’) was designed to target one of the flanking eukaryotic genes (g37539) and one of the genes within the cluster (g37541). The Clst1 amplified product was used as a template for PCR amplification using the Clst2 primer pair. Gel electrophoresis was used to confirm that the Clst1 and Clst2 PCR products were of the expected lengths (~6.5 kb and ~3.3 kb, respectively). Amplified products were sent to GENEWIZ (South Plainfield, NJ, USA) for Sanger sequencing. Following inspection of the waveforms and editing for quality, sequences were aligned with the genome using CLC Genomics Workbench.

RNA-Seq output

Transcriptional response to elevated pCO_2

Two transcripts sharing homology with carbonic anhydrase 2 (Ca2) were up-regulated after one hour of exposure to the ATHC treatment (Table S3b). Increased expression of *Ca2* may be required to maintain control of intracellular concentrations of CO_2 and bicarbonate, some of which may be provided to the endosymbiont as photosynthetic substrate in exchange for energy from photosynthates translocated to the host. It may otherwise be a mechanism for maintaining

intracellular acid-base homeostasis; consumption of CO_2 by the *Symbiodinium* has been suggested as a mechanism for controlling intracellular pH in the host cell [5]. Curiously, no carbonic anhydrase homologs were identified among the DE transcripts in the comparison between the ATHC and ATAC treatments after six hours of exposure. Exposure to high pCO_2 conditions appears to impact cell growth, cell cycle progression, cell differentiation and proliferation. Homologs of several proteins involved in regulation of cell migration and proliferation, such as *Trim59* and *Fgf10*, were up-regulated. Homologs of *Irf1* and *Brca1*, potential promoters of cell cycle arrest, are up-regulated. Induction of cell cycle arrest may allow cells time to repair DNA damage and cell defects before proliferation.

Immune signaling and inflammatory responses appear to be impacted as well. A homolog of the cytokine-activated transcription factor *Stat5b* is up-regulated while two homologs of lysophosphatidlycholine acetyltransferase 2 (*Lpcat2*) are down-regulated; the latter is involved in the activation of platelet-activating factor (PAF) via reversible conversion from lyso-PA. PAF interacts with its receptor to mediate downstream inflammatory processes, and a recent metabolomic study of corals demonstrated decreased lyso-PAF/PAF ratios in damaged coral tissues following competitive interactions with algae, suggesting that PAF is an important component of the coral's response to inflammation and tissue damage [6], and down-regulation of *Lpcat2* may indicate regulatory control of this ratio.

After six hours of ATHC exposure two phospholipase homologs (basic phospholipase A2 DE-1 and phospholipase D1) are up-regulated. These enzymes may be involved in intracellular signaling, as products of their activity—such as arachidonic acid, choline, and phosphatidic acid—are signaling molecules or precursors of signaling molecules. Phospholipases have also been found in venom—in fact, the phospholipase with which the *M. capitata* gene model shared homology was found in snake venom. It is interesting that another potential toxin, a homolog of a protease inhibitor (*PI-stichotoxin-She2a*) isolated from the sun anemone *Stichodactyla helianthus*, is also up-regulated. The *M. capitata* phospholipase A2 homolog found differentially expressed here has homology to secretory phospholipase A2, and submission to SignalP 4.0 revealed a predicted secretory signal. Up-regulation of these potential venom components may be a defense mechanism, or it may be indicative of increased metabolic demand to be addressed in part by predation. If it is not a component of the coral's toxic arsenal, this phospholipase A2 homolog may be involved in bacterial defense.

Transcriptional responses common to both high temperature and high pCO2

We identified differential expression of a number of regulatory elements relatively early (~one hour of exposure) in the response of *M. capitata* to both the ATHC and HTAC treatments. Homologs of known developmental and stress response transcription factors including heat shock factor 1 (*Hsf1*), Krüppel-like factor 5 (*Klf5*), thyrotroph embryonic factor (*Tef*) and its chicken homolog vitellogenin-binding protein (*Vbp*) were up-regulated. Several of these factors regulate diverse developmental process including cell proliferation and survival, though their precise function in corals is not thoroughly understood. Up-regulation of protein homologs potentially involved in signaling (*Wnt-1*) and biogenesis of protein synthesis machinery (*Nop56*) suggest that the coral is preparing to mount a larger response to stress conditions, while upregulation of a carbonic anhydrase may increase intracellular CO2 concentration, potentially to provide to the *Symbiodinium* for photosynthetic carbon fixation. Translocation of photosynthates to the host may provide the energy required to mount the stress response.

After six hours of exposure, up-regulation of homologs of regulators of transcription factor NF-kB (*Nod2*, *Nlrp12*) may be indicative of onset of inflammatory and/or immune responses. Transcripts sharing homology with conserved regulatory elements of the coral circadian machinery exhibited differential expression after six hours of exposure to either treatment, perhaps indicative of stress-induced disturbance of the coral circadian cycle. Two cryptochrome homologs (*Cry1*/*2*) were up-regulated, whereas a *Clock* homolog was down-regulated. Down-regulation of the Clock homolog is interesting, because a transcript with a Blastx hit to neuronal PAS domain-containing protein 2 (*Npas2*) is up-regulated after six hours in the HTAC treatment. *Npas2* has been implicated as a *Clock* paralog in mammals, but its role in corals has not been investigated.

Alterations in glycogen metabolism appear to be a response to both treatments, because two regulatory subunits that recruit protein phosphatase 1 to glycogen (*Pppr3b*/*d*) to regulate the activity of glycogen synthase and glycogen phosphorylase are up-regulated in both treatments. One homolog of cytochrome P450 1A1 (*Cyp1a1*), a protein implicated in xenobiotic metabolism, detoxification, and generation of active signaling molecules, is up-regulated following high pCO2 exposure but down-regulated following high temperature exposure. This differential response between treatments is intriguing in light of the fact that nine other transcripts sharing

homology to various P450 cytochromes were identified among the transcripts up-regulated after six hours of thermal challenge.

Differential expression after 1 hour of ATHC treatment: In the comparison of the ATHC and ATAC treatments following 1 hour of treatment exposure there were 108 gene models identified as being differentially expressed (73 up-regulated, 35 down-regulated). About 49% of these gene models (53: 32 up-regulated, 21 down-regulated) had no BLASTx hits, or had best hits to hypothetical or uncharacterized proteins. There were no significant enrichments for BP or MF GO terms among either the up-regulated or down-regulated gene models in this comparison.

Among the 50 most up-regulated gene models (Log2FoldChange 1.87 to 26.02) were potential homologs of molecular chaperones (e.g., DnaJ protein homolog 1, heat shock 70 kDa protein 1 isoform X2), transcription factors (ex: early growth response protein 1 isoform X2, Krüppel-like factor 5, paired box protein Pax-3-B, signal transducer and activator of transcription 5B isoform X1, thyrotroph embryonic factor, transcription factor VBP), proteins involved in apoptosis or autophagy (lactadherin, TBC1 domain family member 17), and carbon fixation (carbonic anhydrase). 25 of the 50 most up-regulated gene models had no BLASTx hits or had best hits to hypothetical or uncharacterized proteins. Among the 35 down-regulated gene models (Log2FoldChange -23.49 to -1.66) were sequences involved in protein ubiquitination (E3 ubiquitin-protein ligase TRIM71, F-box/LRR-repeat protein 20) in immune response and injury response (lysophosphatidylcholine acyltransferase 2, nucleotide-binding oligomerization domain-containing protein 2, snaclec coagulation factor IX-binding protein subunit A), in apoptotic processes (XK-related protein 8), metabolism (indoleamine 2,3-dioxygenase 2, NADP-specific glutamate dehydrogenase) and axonal development and growth (noelin isoform X1). In total, 21 of the 34 down-regulated gene models had no BLASTx hits or had best hits to hypothetical or uncharacterized proteins.

TargetP sub-cellular localization predictions with a RC ≤ 3 were reported for 6 gene models in this set (5 up-regulated, 1 down-regulated): 2 “secretory” and 4 “other”. Of the two gene models with TargetP “secretory” classification, only one was predicted to have a signal peptide sequence by SignalP (see Table S3c).

Differential expression after 6 hours of ATHC treatment: In the comparison of the ATHC and ATAC treatments following 6 hours of exposure, there were 100 gene models identified as being differentially expressed (69 up-regulated, 31 down-regulated. Of these, 15 were also identified as being differentially expressed in the same treatments after 1 hour of exposure. About 56% of these gene models (56; 38 up-regulated, 18 down-regulated) had not BLASTx hits, or had best hits to hypothetical or uncharacterized proteins. No GO term enrichments were observed for the 69 up-regulated gene models. The 31 down-regulated gene models were enriched for MF GO terms associated with mannosyl-oligosaccharide glucosidase activity (GO:0004573) and ATP-dependent peptidase activity (GO:0004176).

Among the 50 most up-regulated gene models (Log2FoldChange 2.51 to 35.91) are potential homologs of transcription factors (ETS translocation variant 4, thyrotroph embryonic factor), proteins involved in protein degradation and modification (BTB/POZ domain-containing protein Atg08455, F-box/LRR-repeat protein 20, furin-like protease kpc-1, protein phosphatase 1 regulatory subunit 3D), proteins involved in apoptosis (XK-related protein 8, GRAM domain-containing protein 4), and transposable elements (Ty3-G Gag-Pol polyprotein). 33 of the 50 most up-regulated gene models had no BLASTx hits or had best hits to hypothetical or uncharacterized proteins. Among the 31 down-regulated genes (Log2FoldChange -46.08 to -1.37) are potential homologs of proteins involved in tRNA maturation (ribonuclease P protein subunit p40 isoform X1), formation of calcium carbonate (perlucin isoform X2), degradation of DMSP and ROS scavenging (dimethylsulfonioproprionate lyase 7), cell migration (protein scribble homolog) and profileration (ras-related and estrogen-regulated growth inhibitor), immune response (uromodulin isoform X1) and transporters (monoxcarboxylate transporter 10). 18 of 31 down-regulated gene models had no BLASTx hits or had best hits to hypothetical or uncharacterized proteins.

TargetP sub-cellular localization predictions with a RC ≤ 3 were reported for 42 gene models in this set (28 up-regulated, 14 down-regulated) with Blastx best hits to uncharacterized or hypothetical proteins, or for which there was no Blastx best hit: 16 “secretory”, 2 “mitochondrial” and 24 “other”. Of the 16 gene models with TargetP “secretory” classification, 8 were predicted to have a signal peptide sequence by SignalP (see Table S3c).

Differential expression after 1 hour of HTAC treatment: In the comparison of the HTAC and ATAC treatments following 1 hour of exposure, there were 306 predicted gene models identified as being differentially expressed (240 up-regulated, 66 down-regulated). About 41% of these (125 predicted gene models; 95 up-regulated, 30 down-regulated) either had no BLASTx hits or had best hits to hypothetical or uncharacterized proteins. GO enrichment analyses for Biological Process (BP) and Molecular Function (MF) terms were performed using Fisher's Exact Test in Blast2GO, corrected for FDR <0.05. No significant enrichments were discovered among the predicted gene models down-regulated between the HTAC and ATAC treatments after 1 hour of exposure. Significant enrichments for MF terms were discovered among the up-regulated gene models, associated with unfolded protein binding (GO:0051082).

Among the 50 most highly up-regulated gene (Log2FoldChange 3.31 to 27.20) are potential homologs of molecular chaperones (ex: heat shock 70 kDa protein 1 isoform X2, small heat shock protein, DnaJ protein homolog 1) transcription factors (ex: Early growth response protein 1 isoform X2, forkhead box protein O, heat shock factor protein 1, Krüppel-like factor 5, paired box proteins D2 and Pax-3-B, TNF receptor-associated factor 3, transcription factor AP-1, transcription factor VBP), as well as potential homologs of proteins involved in responses to oxidative stress (ex: glutathione-S-transferase omega 1), cellular signaling and synthesis of signaling molecules (ex: cannabinoid receptor 1, cytosolic phospholipase A2, GABA type B receptor subunit 1), and transport (ex: chloride intracellular channel protein 2, major facilitator superfamily domain-containing protein 12 isoform X2). Twenty-two of the 50 most up-regulated gene models had either no BLASTx best hits or had best hits to uncharacterized or hypothetical proteins.

Among the 50 most down-regulated gene (Log2FoldChange -23.16 to -1.31) are potential homologs of proteins involved in regulation of autophagy and apoptosis (ex: protein prune homolog 2, transmembrane protein 192, kelch-like protein 20), metabolism (ex: 5-formyltetrahydrofolate cyclo-ligase, cysteine synthase 1, D-amino-acid oxidase, mannose-6-phosphate isomerase isoform X1, NADP-specific glutamate dehydrogenase, and phtiocerol synthesis polyketide synthase), transcription factors and transcriptional regulators (e.g., homeobox protein protein MSX-2, thyrotroph embryonic factor) and proteins involved in intracellular transport and cilia formation (e.g., intraflagellar transport protein 172, kinesin-like

protein KIF3A). Twenty-seven of the 50 most down-regulated gene models had either no BLASTx best hits or had best hits to uncharacterized or hypothetical proteins.

TargetP sub-cellular localization predictions with a RC ≤ 3 were reported for 92 gene models in this set (71 up-regulated, 21 down-regulated) with Blastx best hits to uncharacterized or hypothetical proteins, or for which there was no Blastx best hit: 25 “secretory”, 5 “mitochondrial” and 62 “other”. Of the 25 gene models with TargetP “secretory” classification, 16 were predicted to have a signal peptide sequence by SignalP (see Table S3c).

Differential expression after 6 hours of HTAC treatment: In the comparison of the HTAC and ATAC treatments following 6 hours of treatment exposure there were 1541 predicted gene models differentially expressed (1059 up-regulated, 482 down-regulated). A total of 92 of these gene models were also found to be differentially expressed in the comparison between treatments after 1 hour of exposure. About 35% of these gene models (539; 366 up-regulated, 173 down-regulated) either had no BLASTx hits or had best hits to hypothetical or uncharacterized proteins. GO enrichment analyses for BP and MF terms were performed using Fisher’s Exact Test in Blast2GO, corrected for FDR <0.05. The 1059 up-regulated gene models were enriched for BP terms associated with, among other things, apoptosis and autophagy (GO:0042981, GO:0006914), protein degradation or modification (GO:0032436, GO:0042787, GO:0046777, GO:0006952), transport of drugs and organic substances (GO:0015893, GO:0071702), regulation of immune and stress responses (GO:0050776, GO:0080134, GO: 0002285, GO:0006952), regulation of cell growth, development and differentiation (GO:0045596, GO:0048468, GO:0030097, GO:0016043), and metabolism and regulation of metabolic processes (GO:0031324, GO:0010605, GO:0006629). The up-regulated genes were enriched for MF terms associated with protein kinase activity (GO:0004672), oxidoreductase, glutathione transferase and protein disulfide oxidoreductase activity (GO:0016671, GO:0004364, GO:0015035), regulatory region DNA binding and transcription factor activity (GO:0000975, GO:0003700), electron carrier activity (GO:0009055), and binding of ATP, receptors, enzymes and transition metals (GO:0005524, GO;0005102, GO:0019899, GO:0046914). The 482 down-regulated gene models were enriched for BP terms associated with ion homeostasis and transport (GO:0055080, GO:0006812, GO:0034220, GO:0098771), carboxylic acid transport

(GO:0046942), DNA replication (GO:0006260), and single-organism metabolic process (GO:0044710).

Among the 50 most up-regulated gene models (Log2FoldChange 6.32 to 32.48) are potential homologs of molecular chaperones (heat shock 70 kDa protein 1 isoform X2, small heat shock protein), proteins involved in apoptotic processes (XK-related protein 8, XIAP-associated factor 1), cell cycle progression or cell development (protein lethal(2)essential for life, zinc finger and BTB domain-containing protein 17, neuroligin, X-linked), protein degradation (BTB/POZ domain-containing protein Atg08455), transposable elements (piggyBac transposable element-derived protein 4), and proteins involved in regeneration of pigment (retinol dehydrogenase 8). 35 of the 50 most up-regulated gene models either had no BLASTx hits or had best hits to hypothetical or uncharacterized proteins.

Among the 50 most down-regulated gene models (Log2FoldChange -23.79 to -3.89) are potential homologs of transporters and mediators of transport (multidrug resistance-associated protein 4, probable cationic amino acid transporter isoform X1, monocarboxylate transporter 10, folate receptor beta), metabolism (phosphatidylserine decarboxylase proenzyme 1 isoform X2, probable thiopurine S-methyltransferase, histamine N-methyltransferase, acetylserotonin O-methyltransferase), proteins involved in induction of apoptosis (TNF ligand superfamily member 10), cysteine and glutathione homeostasis (gamma-glutamyltranspeptidase 1), immune response (deleted in malignant brain tumors 1 protein), and extracellular glycoproteins (spondin 1). A total of 29 of the 50 most down-regulated gene models had either no BLASTx hits or had best hits to hypothetical or uncharacterized proteins.

TargetP sub-cellular localization predictions with a RC ≤ 3 were reported for 394 gene models in this set (263 up-regulated, 131 down-regulated) with Blastx best hits to uncharacterized or hypothetical proteins, or for which there was no Blastx best hit: 100 “secretory”, 29 “mitochondrial” and 265 “other”. Of the 100 gene models with TargetP “secretory” classification, 70 were predicted to have a signal peptide sequence by SignalP (see Table S3c).

Differential expression common to both the ATHC and HTAC treatments: A total of 40 gene models (37 up-regulated, 3 down-regulated) were identified as differentially expressed in both the ATHC vs. ATAC and HTAC vs. ATAC comparisons after 1 hour of treatment. Among the

up-regulated gene models were potential homologs of transcription factors (early growth response protein 1 isoform X2, Krüppel-like factor 5, heat shock factor protein 1 isoform X1, paired box protein Pax-3-B, thyrotroph embryonic factor, and transcription factor VBP), molecular chaperones (heat shock 70 kDa protein 1 isoform X2, DnaJ protein homolog 1, DnaJ homolog subfamily B member 1/5), degradation of extracellular matrix (matrix metalloproteinase 24), DNA repair (DNA ligase 1), signaling (protein Wnt-1), lipid metabolism and membrane maintenance (outer membrane protein Blc), cell adhesion (sushi, von Willebrand factor type A, EGF and pentraxin domain-containing protein 1 isoform X2), and CO2 cycling (carbonic anhydrase 2). Only one of the 3 down-regulated gene models in this set had a best hit in a BLASTx search, to a NADP-specific glutamate dehydrogenase.

Among the up-regulated gene models in this set, 16 had either no BLASTx hit or had hits to hypothetical and uncharacterized proteins. 15 of these gene models had TargetP sub-cellular localization predictions with an RC <3; 3 “secretory” and 12 “other”. Of the 3 gene models assigned a “secretory” prediction, 2 were predicted by SignalP to possess a signal peptide. Among the down-regulated gene models in this set, 2 had either no BLASTx hit or had hits to hypothetical or uncharacterized proteins. These 2 gene models had TargetP sub-cellular localization predictions with an RC <3; 1 “secretory” and 1 “other”. The single gene model with a “secretory” prediction was predicted by SignalP to possess a signal peptide (see Table S3c).

A total of 61 gene models (48 up-regulated, 12 down-regulated, 1 mixed) were differentially expressed in both the ATHC vs. ATAC and HTAC vs. ATAC comparisons after 6 hours of treatment exposure. Among the up-regulated gene models are potential homologs of ubiquitin ligases or components thereof (BTB/POZ domain-containing protein At4g08455, Fbox/LRR-repeat protein 20), proteins involved in regulation of activation of NF-kB (NACHT, LRR and PYD domains-containing protein 12 isoform X2, nucleotide-binding oligomerization domain-containing protein 2 isoform X1), transcription factors (thyrotroph embryonic factor, transcription factor VBP), mediators of apoptotic processes (GRAM domain-containing protein 4, XK-related protein 8), regulatory subunits of protein phosphatases (protein phosphatase 1 regulatory subunit 3B/D), potential mediators of circadian processes (cryptochrome-1, cryptochrome CRY2), cation channels (transient receptor potential cation channel subfamily A member 1 homolog), proteins involved in protein activation and modification (furin-like protease kpc-1), and proteins derived from transposable elements (Ty3-G Gag-Pol polyprotein). 9 of these

gene models were also identified as differentially expressed in the same comparisons after 1 hour of treatment exposure (2 with no BLASTx, 3 with hits to hypothetical or uncharacterized proteins, a LON peptidase N-terminal domain and RING finger protein 3, cryptochrome-1, and thyrotroph embryonic factor). Among the few down-regulated gene models in this set are potential homologs of proteins involved in circadian processes (circadian locomoter output cycles protein kaput), transporters (monocarboxylate transporter 10), extracellular glycoproteins (spondin-1), and proteins with potential roles in cell proliferation (Ras-related and estrogen-regulated growth inhibitor). One gene model, a potential homolog of cytochrome P450 1A1, was up-regulated after 6 hours in the ATHC treatment but down-regulated after 6 hours of the HTAC treatment.

Among the up regulated gene models in this set, 30 had either no BLASTx hit or had hits to hypothetical and uncharacterized proteins. 22 of these gene models had TargetP sub cellular localization predictions with an RC <3; 9 “secretory”, 2 “mitochondria” and 12 “other”. Of the 9 gene models assigned a “secretory” prediction, 4 were predicted by SignalP to possess a signal peptide. Among the down-regulated gene models in this set, 5 had either no BLASTx hit or had hits to hypothetical or uncharacterized proteins. These 5 gene models had TargetP sub cellular localization predictions with an RC <3; 1 “secretory” and 4 “other”. The single gene model witha “secretory” prediction was not predicted by SignalP to possess a signal peptide (see Table S3c).

Generation of co-expression networks

The limma [7] and edgeR [8] R packages were used for preprocessing and normalization of the read counts. DESeq2 was used to identify DE genes that did not have low counts (according to the rule of <10 counts in more than 90% of samples) and were used in this analysis. Library sizes were scaled using the calcNormFactors function based on TMM normalization (limma), log-CPM with prior 3 was used for further analysis (as recommended in the limma User's Guide). Weighted correlation network analysis (WGCNA, R/package [9]) was used to generate expression networks from these normalized read counts. The authors appreciate that using a limited set of genes (i.e., DE genes, as opposed to all genes that do not have low counts) as input for the co-expression network analysis may introduce some bias, and may essentially result in fewer modules. However, this approach enabled us to identify few significant modules, with significantly enriched annotations (see below, *Identification of significant modules*) allowing us

to shed light on the underlying mechanisms governing the coral stress response. The "pickSoftThreshold" function was used to find the soft thresholds power of 16 based on network topology (resulting in scale-free topology fit index threshold > 0.88). Modules of co-expressed genes were identified using above soft thresholds power, minimal module size of 30 genes, and minKMEtoStay of 0.5 to minimize mis-assignment of genes to the module eigengene (which corresponds to the first principal component of a given module).

Identification of significant modules: For all identified modules, we used the modulePreservation function to validate that modules identified by WGCNA were significantly above randomly generated modules[10]. This function was designed to calculate preservation statistics between independent data sets, however it can be used to identify modules that are significantly above random levels (personal correspondence, Dr. Peter Langfelder, University of California, USA). The single data set was supplied both as reference and as a test set, and modules with a quality statistic of Z score > 10 were regarded as highly preserved [Table S5 [10]]. In order to identify significant differences (p-value < 0.05) between expression levels of treatments pairs we have used the Tukey-Kramer HSD test, based on the normalized expression values of each treatment. Another means of establishing significance for the module expression pattern was to identify significantly enriched (p-value < 0.05, Fisher's exact test, Methods) DESeq2-identified DE genes. Only modules that were significantly preserved (Z-summary score >10) and contained a significant expression pattern according to at least one of these two methods were considered for further analysis. To decipher the role of gene modules in the stress response, we identified significantly enriched (using Fisher's exact test, p-value < 0.05) KEGG orthologs, InterPro domains (identified using InterProScan [11]), and GO functions in each module (Table S5). Enrichment for these terms (and of DESeq2-identified DE genes) was calculated using the ratio: (SITM/ AITM) / (SIODG/ AIODG), where SITM denotes count of Selected ID in Tested Module; AITM denotes count of All IDs in Tested Module; SIODG denotes count of Selected ID in all Other DE Gene; AIODG denotes counts of All IDs in in all Other DE Gene. To calculate the enrichment of DE genes in each contrast (e.g., enrichment of "Heat1 vs. Heat6"), counts of DESeq2-identified DE genes was used instead of counts of IDs described above (and the contrast was used instead of selected ID). Significance of these terms enrichment was calculated using Fisher's exact test [12].

Identification and visualization of hubs: To highlight genes with a central role in expression and their comprising hubs, we characterized genes in modules using standard network parameters of centrality [i.e., degree, and betweenness, calculated using igraph/R package [13], major hubs were identified by inspecting nodes annotated with the top 10% of degree and betweenness values in the network. To inspect the network topology, modules were exported to Cytoscape [14], using the exportNetworkToCytoscape function (WGCNA, R/package), following setting of the network layout using the force-directed algorithm) [15].

Methylome methods

In order to determine the coral methylome (genome-wide DNA methylation), DNA was extracted as described in *DNA/RNA Extraction and Library Preparation* section. Note this will result in extraction of both host and *Symbiodinium* DNA simultaneously. Two DNA samples from the Time 0 Ambient condition and 6h high temperature condition was quantified using a Nanodrop spectrophotometer and quality controlled by running on a 0.7% agarose gel for 2h and 70V. Approximately 375ng of high molecular weight DNA for each sample was sent to the Epigenomics Core Facility at Weill Cornell Medical College for whole genome bisulfite sequencing (WGBS). Sequencing library preparation was completed according to manufacturer's instructions in the Illumina TruSeq DNA methylation kit reference guide (#15066014 v01 August 2016) with the addition of a Lambda phage spike for bisulfite conversion efficiency and the quality-controlled library was sequenced on one lane of Illumina HiSeq2500 using Paired End Read Clustering and 2 x 100 sequencing cycles.

Sequencing of the samples produced 177,168,330 paired end reads with a mean quality score of ~37. Sequences were mapped against the bisulfite genome of the lambda phage using bowtie2 (v2.1.0 x) within Bismark (v0.18.2, x) revealing a bisulfite conversion rate of ~97%. Sequences were trimmed using cutadapt (v1.11 with Python 2.7.0) for a quality score of 30 and -m -21 to remove sequencing adapters and short reads. The reads were mapped to the *M. capitata* genome (-q --score-min L,0,-0.6 --ignore-quals --no-mixed --no-discordant --dovetail –maxins 500, Option '--directional' specified (default mode): alignments to complementary strands (CTOT, CTOB) were ignored (i.e. not performed), resulted in 63.2% mapping) following Bismark genome preparation. The data were de-duplicated in Bismark (using the command

deduplicate_bismark), which retained 98.76% of the total mapped. Methylation calls were completed following filtering for coverage of 5 for each position. The location of methylation was determined with respect to intergenic regions, gene bodies (introns, exons), and SCORs using bedtools intersect with the genome feature files.

Preliminary description of the *M. capitata* methylome

Bisulfite sequencing of two samples resulted in sequence information for 80% of the CpG in the genome and 58.9% after filtering for 5x coverage, which was used for the comparative analyses. The sparse methylation common in invertebrates was also seen in *M. capitata*, with CpG methylation accounting for only 0.06% of the total genome and 18.8% of CpGs. In keeping with the intergenic expansion in this species (see above), the majority of CpG methylation was found to be in the intergenic regions (54.8%) and a minority of the genic regions (45.1%). Within the genic regions, 80.3% of methylation occurred in introns and 19.7% in exons, similar to the finding in the coral *S. pistillata* [16]. With the existing data, we sought to address the hypothesis that methylated DNA was preferentially associated with SCORs and would act to silence TEs. This does not however appear to be the case in *M. capitata* because methylated CpGs are represented by <1% of the SCOR-encoding bases. It will be critical however to explicitly test these hypotheses of genome-epigenome interaction in a well replicated, high coverage fashion. This should include examining the linkage between DNA methylation and differential gene expression within the same sample, and the connection, if any, between DNA methylation and the expanded gene sets, or enriched ontologies within *M. capitata*.

References

1 Moriya, Y., Itoh, M., Okuda, S., Yoshizawa, A. C. & Kanehisa, M. KAAS: an automatic genome annotation and pathway reconstruction server. *Nucleic Acids Res* **35**, W182-185, doi:10.1093/nar/gkm321 (2007).

2 Benzécri, J. P. Analyse de l'inertie intra-classe par l'analyse d'un tableau des correspondances. *Les Cahiers de l'Analyse des Données* **8**, 351-358 (1983).

3 Charif, D., Thioulouse, J., Lobry, J. R. & Perrière, G. Online synonymous codon usage analyses with the ade4 and seqinR packages. *Bioinformatics* **21**, 545-547, doi:10.1093/bioinformatics/bti037 (2005).

4 Thioulouse, J., Chessel, D., Dolédec, S. & Olivier, J.-M. ADE-4: a multivariate analysis and graphical display software. *Journal of Statistics and Computing* **7**, 75-83 (1997).

5 Laurent, J., Tambutté, S., Tambutté, É., Allemand, D. & Venn, A. The influence of photosynthesis on host intracellular pH in scleractinian corals. *J Exp Biol* **216**, 1398-1404, doi:10.1242/jeb.082081 (2013).

6 Quinn, R. A. *et al.* Metabolomics of reef benthic interactions reveals a bioactive lipid involved in coral defence. *Proc Biol Sci* **283**, doi:10.1098/rspb.2016.0469 (2016).

7 Ritchie, M. E. *et al.* limma powers differential expression analyses for RNA-sequencing and microarray studies. *Nucleic Acids Res* **43**, e47, doi:10.1093/nar/gkv007 (2015).

8 Robinson, M. D., McCarthy, D. J. & Smyth, G. K. edgeR: a Bioconductor package for differential expression analysis of digital gene expression data. *Bioinformatics* **26**, 139-140, doi:10.1093/bioinformatics/btp616 (2010).

9 Langfelder, P. & Horvath, S. WGCNA: an R package for weighted correlation network analysis. *BMC Bioinformatics* **9**, 559, doi:10.1186/1471-2105-9-559 (2008).

10 Langfelder, P., Luo, R., Oldham, M. C. & Horvath, S. Is my network module preserved and reproducible? *PLoS Comput Biol* **7**, e1001057, doi:10.1371/journal.pcbi.1001057 (2011).

11 Jones, P. *et al.* InterProScan 5: genome-scale protein function classification. *Bioinformatics* **30**, 1236-1240, doi:10.1093/bioinformatics/btu031 (2014).

12 Rivals, I., Personnaz, L., Taing, L. & Potier, M. C. Enrichment or depletion of a GO category within a class of genes: which test? *Bioinformatics* **23**, 401-407, doi:10.1093/bioinformatics/btl633 (2007).

13 Csardi, G. & Nepusz, T. The Igraph software package for complex network research. *InterJournal 2006, Complex Systems* **1695** (2006).

14 Smoot, M. E., Ono, K., Ruscheinski, J., Wang, P. L. & Ideker, T. Cytoscape 2.8: new features for data integration and network visualization. *Bioinformatics* **27**, 431-432, doi:10.1093/bioinformatics/btq675 (2011).

15 Fruchterman, T. M. J. & Reingold, E. M. Graph Drawing by Force-Directed Placement. *Software-Practice & Experience* **21**, 1129-1164, doi:DOI 10.1002/spe.4380211102 (1991).

16 Liew, Y. J. *et al.* Epigenome-associated phenotypic acclimatization to ocean acidification in a reef-building coral. *Sci Adv* **4**, eaar8028, doi:10.1126/sciadv.aar8028 (2018).

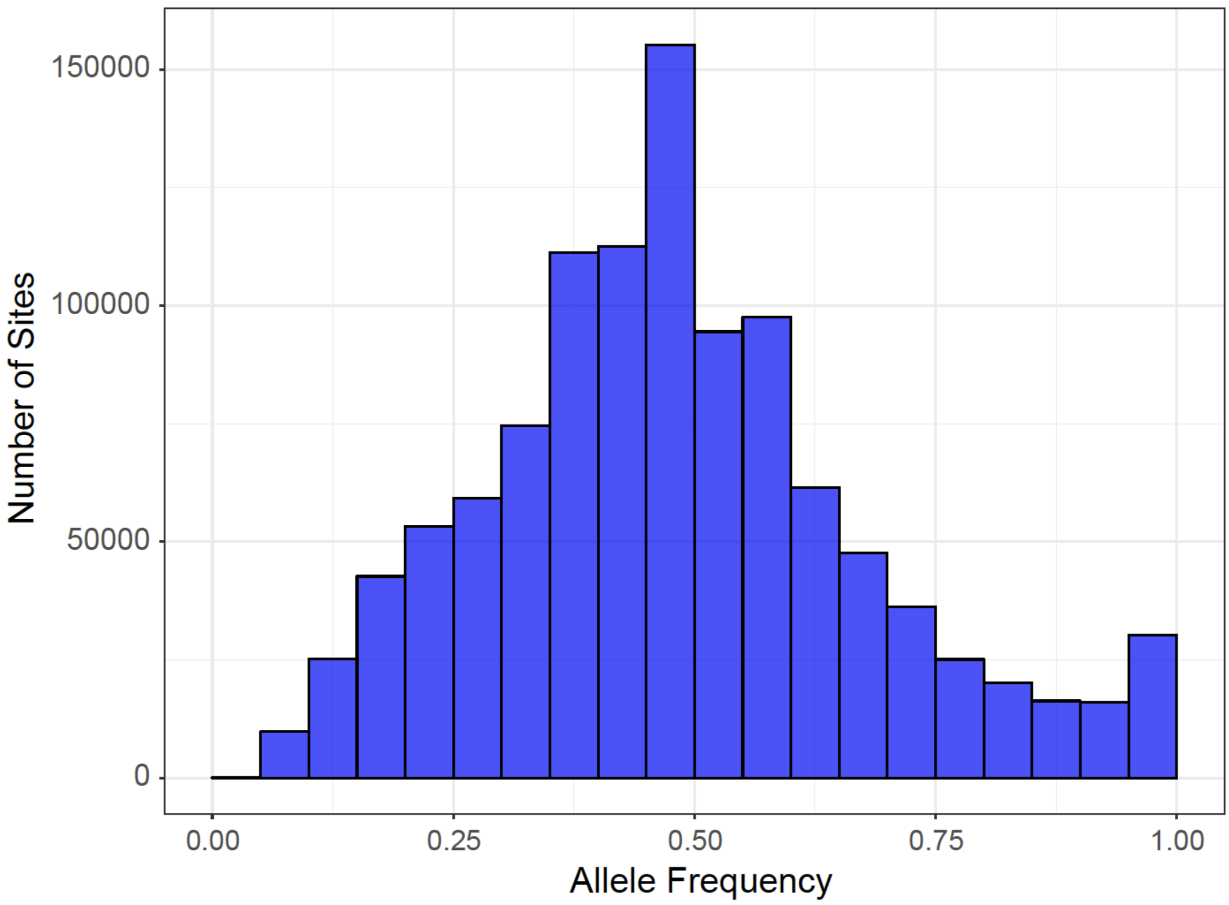

Figure S1. SNP frequency distribution in the *M. capitata* PacBio genome assembly. The allele frequency distribution of 1,088,903 SNP sites from non-repeated regions of the coral genome. The distribution follows the classical allele frequency distribution of a diploid tissue, verifying that the assembly represents a single diploid genotype although the DNA was collected form gametes that differ from each other due to independent meiotic recombination events between the two haploid somatic genomes.

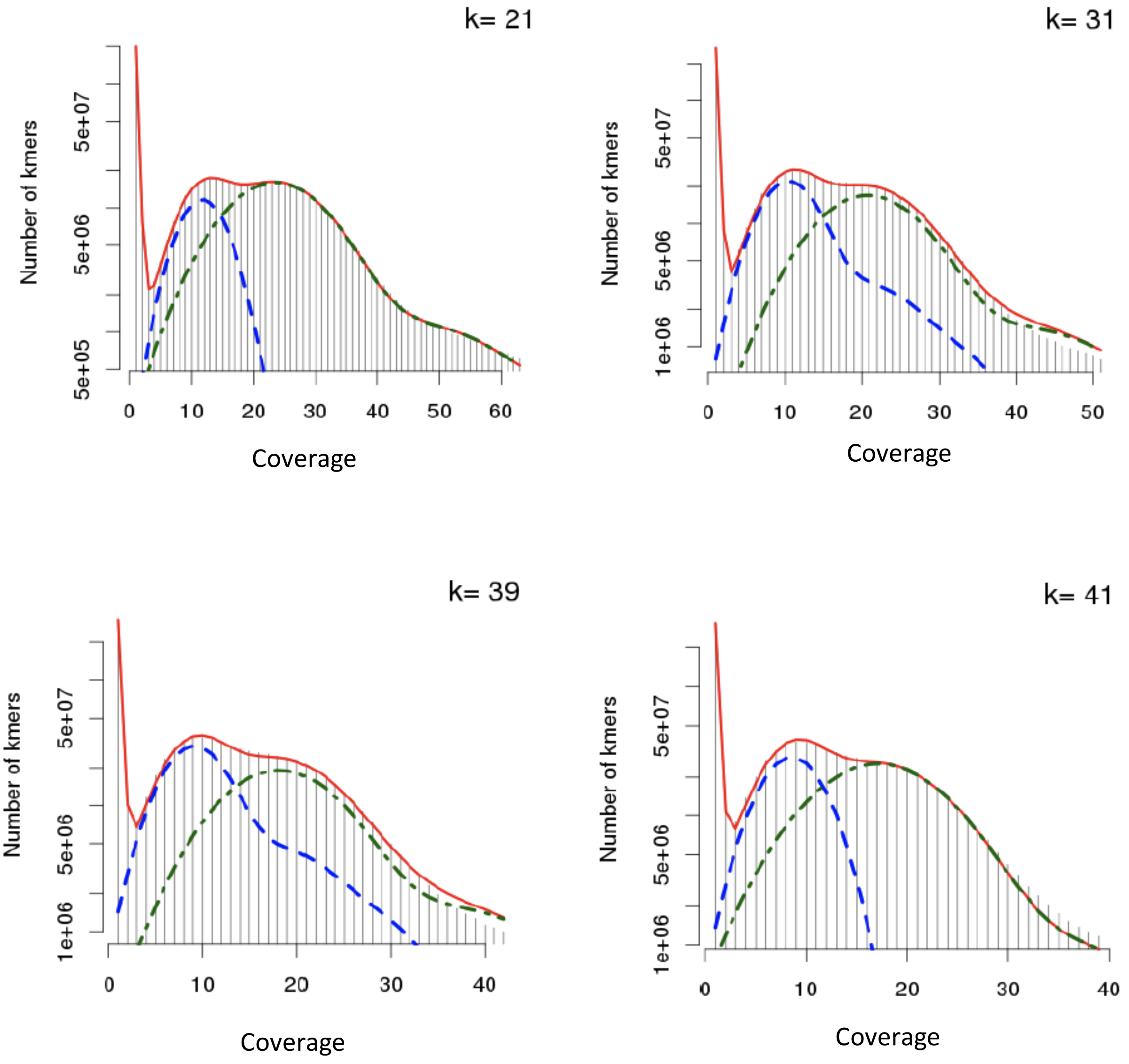

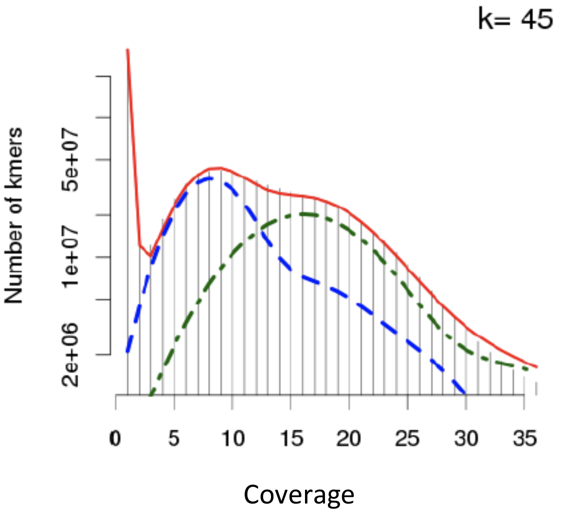

Figure S2. ***K*-mer Analysis of *Montipora capitata* Illumina data.** *K*-mer analysis of Illumina reads from *M. capitata* using a variety of spectra under the expectation of a diploid genome (http://kmergenie.bx.psu.edu). The predicted best fit is $k = 41$. The red lines represent the fit of the complete statistical model of the histogram (erroneous *k*-mers + genomic *k*-mers). The blue line represents the heterozygous *k*-mers, whereas the green line represents the homozygous *k*-mers. The peak at ca. ½ the *k*-mer coverage suggests that this coral is a diploid with moderate heterozygosity.

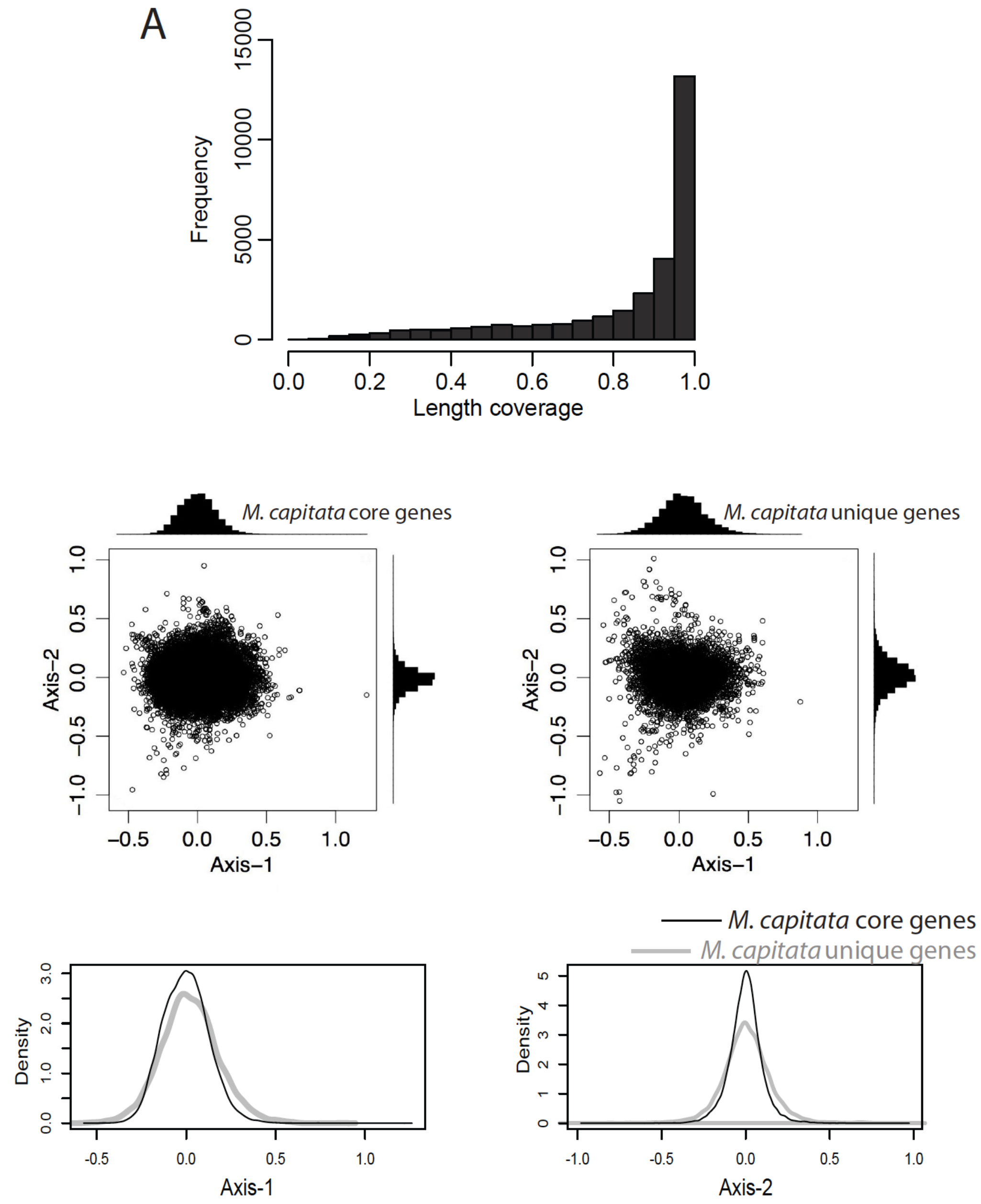

Figure S3. ***M. capitata*** **gene completeness and codon usage pattern.** A) Length coverage of *M. capitata* proteins against the homologs in other corals. The *M. capitata* proteins were used as queries to search using BLASTp (e-value = 1e^{-10}) against the combined protein database comprising high quality proteomes from *Acropora digitifera*, *Orbicella faveolata*, and *Stylophora pistillata*. Following the removal of potential unspecific hits (<100 aa in length and <30% in identity), the alignment coverage of the top hits was included in the plot. The long tail presumably represents residual unspecific hits (e.g., to distant homologs in superfamilies or ubiquitous domains). B) Codon usage pattern of *M. capitata* core genes and lineage-specific genes. The positions of *M. capitata* genes along the major axes (axis 1 and axis 2) were plotted. Both gene categories showed homogeneous codon usage (upper panel). The density distributions of the two gene categories are largely the same along axis 1 and axis 2 (lower panel).

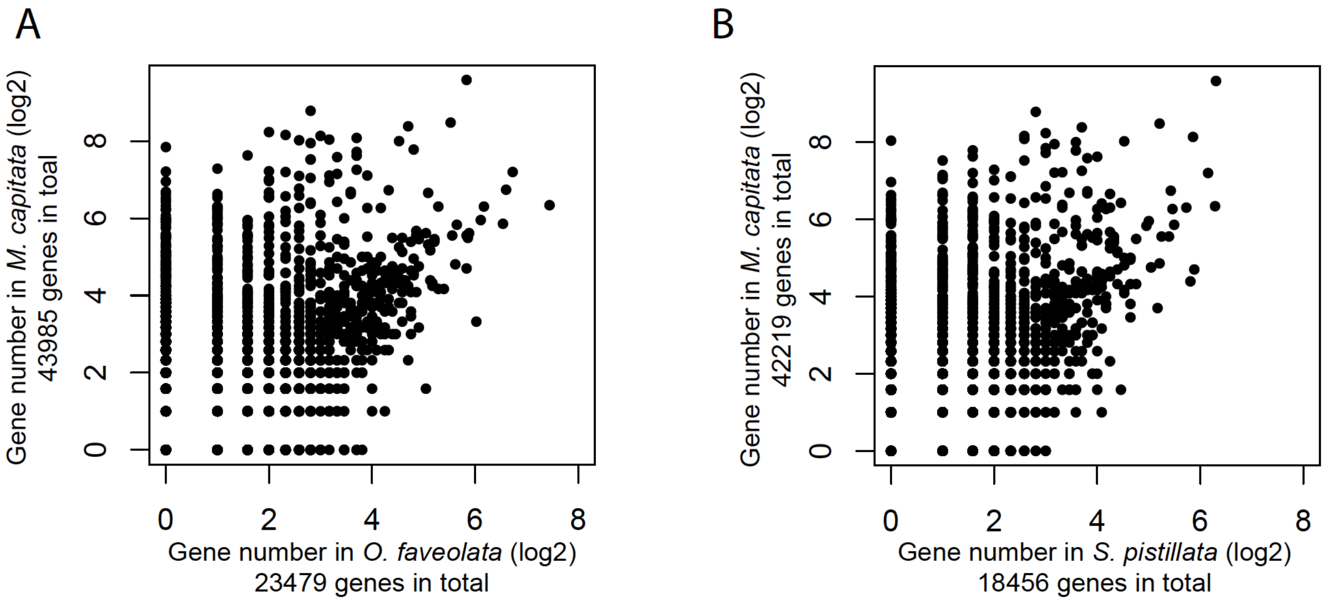

Figure S4. Size comparison of shared gene families between two coral species. A) Scatterplot of gene numbers in shared gene families between *M. capitata* and *O. faveolata*. B) Scatterplot of gene numbers in shared gene families between *M. capitata* and *S. pistillata*.

A

Species	KEGG	Ratio*	PFAM	Ratio*
Montipora capitata	16994	100%	50435	100%
Acropora digitifera	13001	131%	32829	153%
Orbicella faveolata	14486	117%	35770	141%
Stylophora pistillata	11961	142%	40431	125%

* *M. capitata* / each coral taxon

B

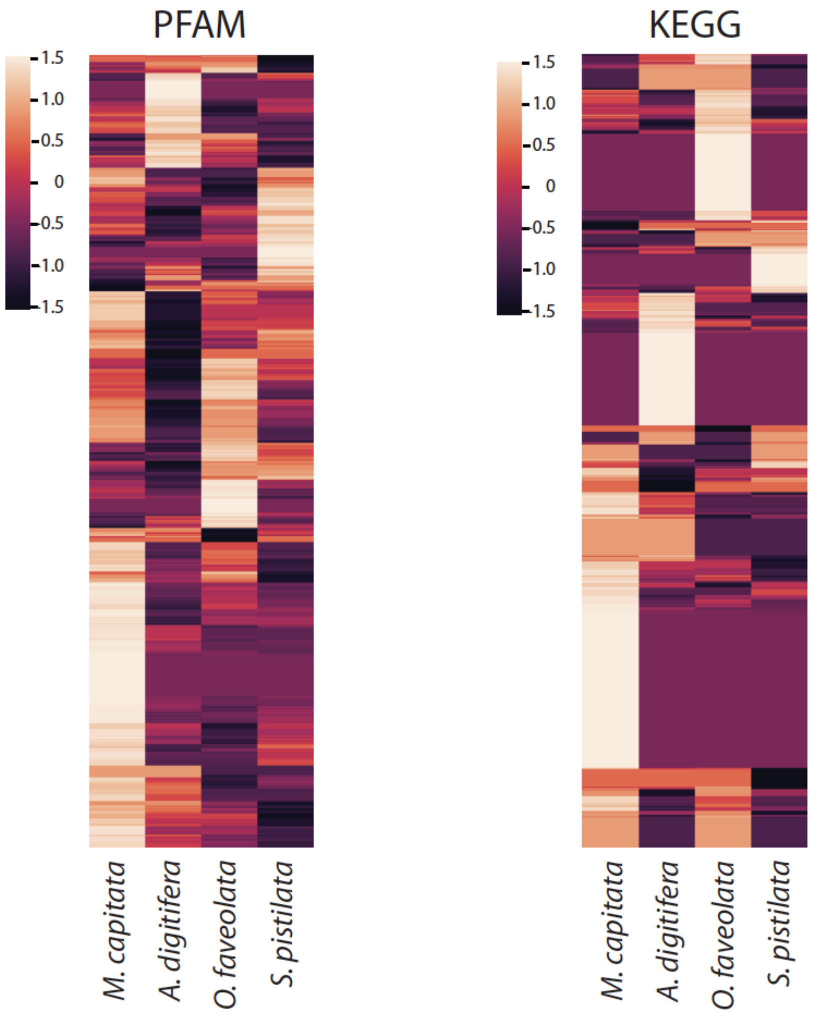

Figure S5. Larger core eukaryotic gene inventory in *M. capitata* than in other corals. A) Total KEGG pathway gene and PFAM domain counts in four stony coral species. B) PFAM and KEGG domain analysis. PFAM domains with counts ≤2 in all species were excluded. The remaining 1,746 PFAM domains with variable counts among the 4 species were included in the heatmap. KEGG pathways with counts ≤3 in all species were excluded. The remaining 3,349 KEGG pathways with variable counts among the 4 species were included in the heatmap. The lighter colors indicate high gene numbers and darker colors, low gene numbers.

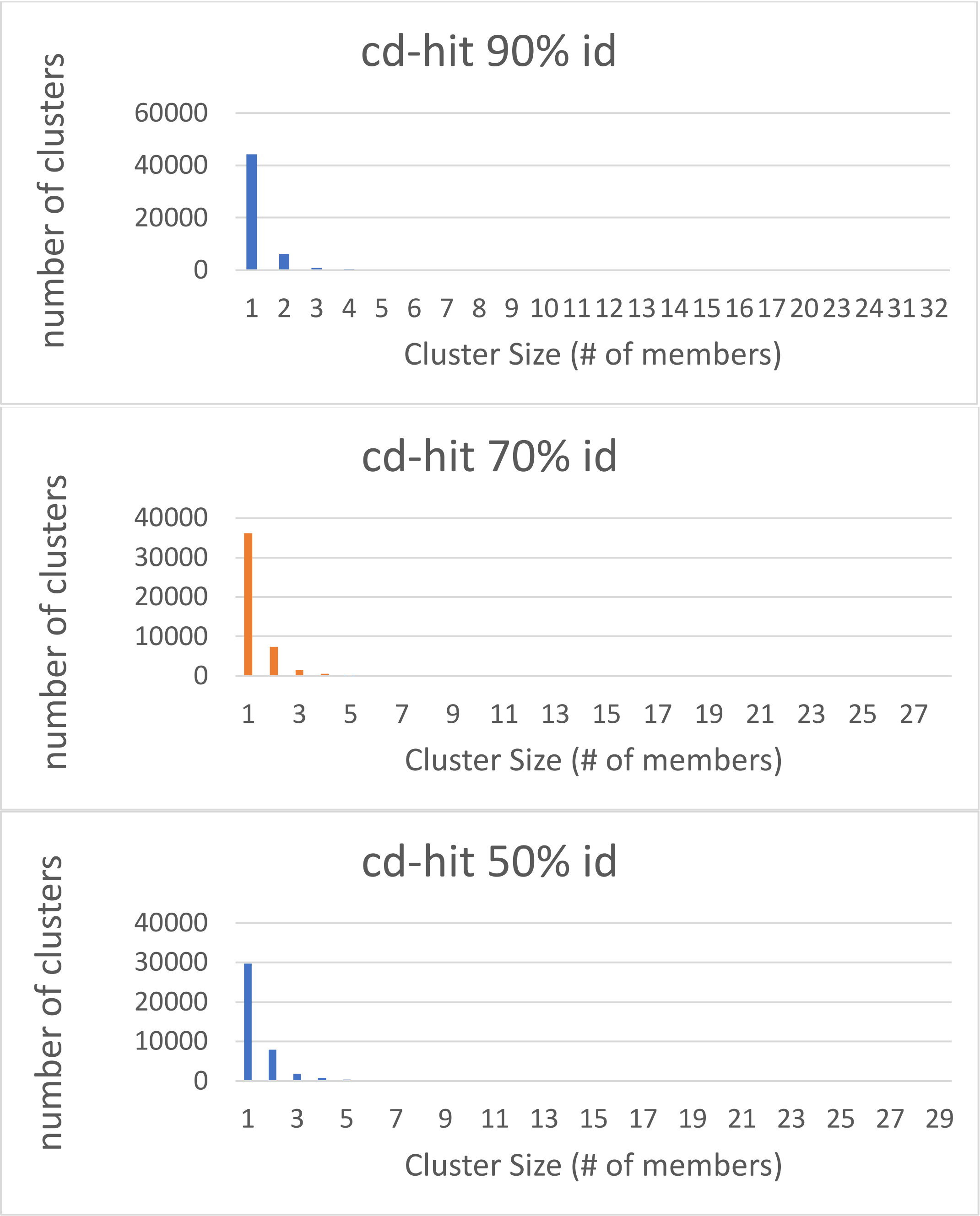

Figure S6. Cd-hit analysis of the predicted proteome of *M. capitata*. Three different protein identity (id) stringencies were used for each query protein analysis, all requiring a minimum of 70% query coverage to avoid comparing conserved domains in heterologous sequences.

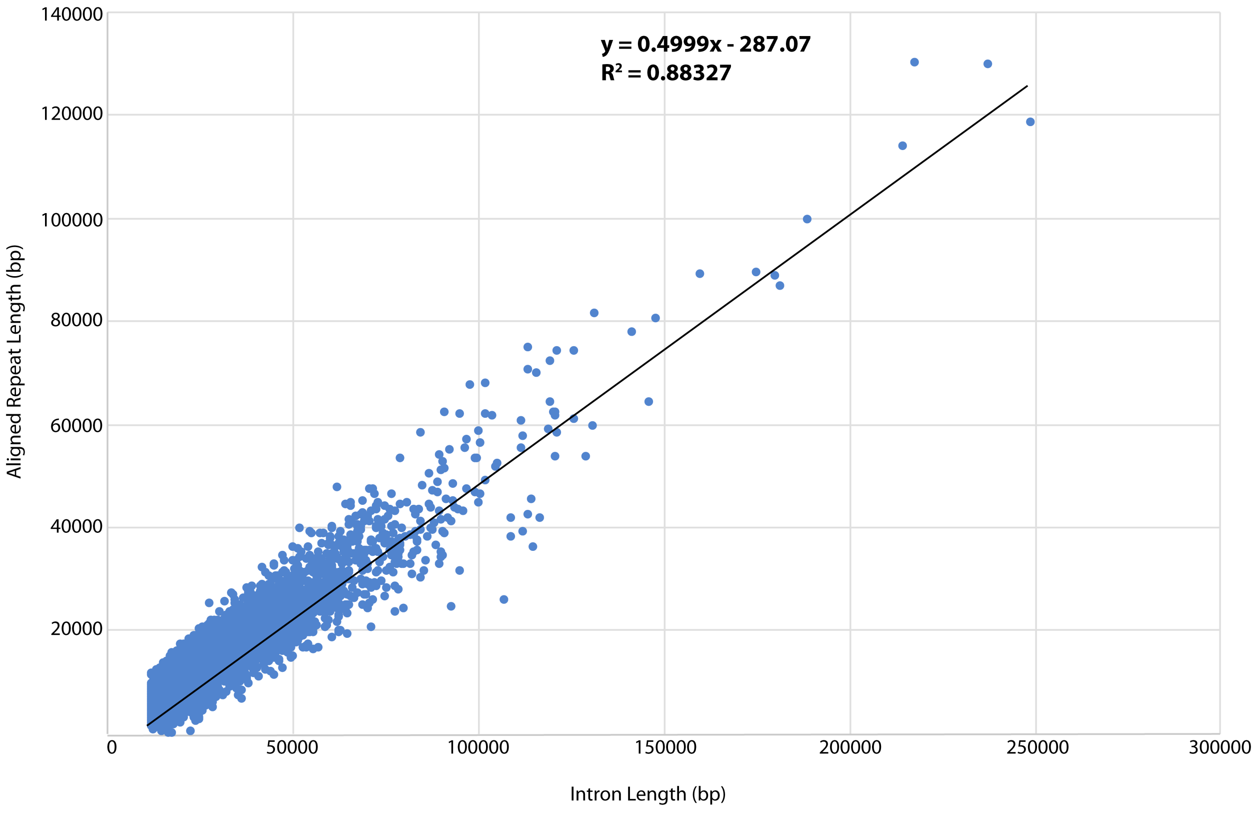

Figure S7. Scatterplot of 9,000 *M. capitata* genes. The x-axis represents the total intron length (in bp) of each gene and the y-axis denote the accumulated length (in bp) of repeats aligned to intronic regions in each studied gene.

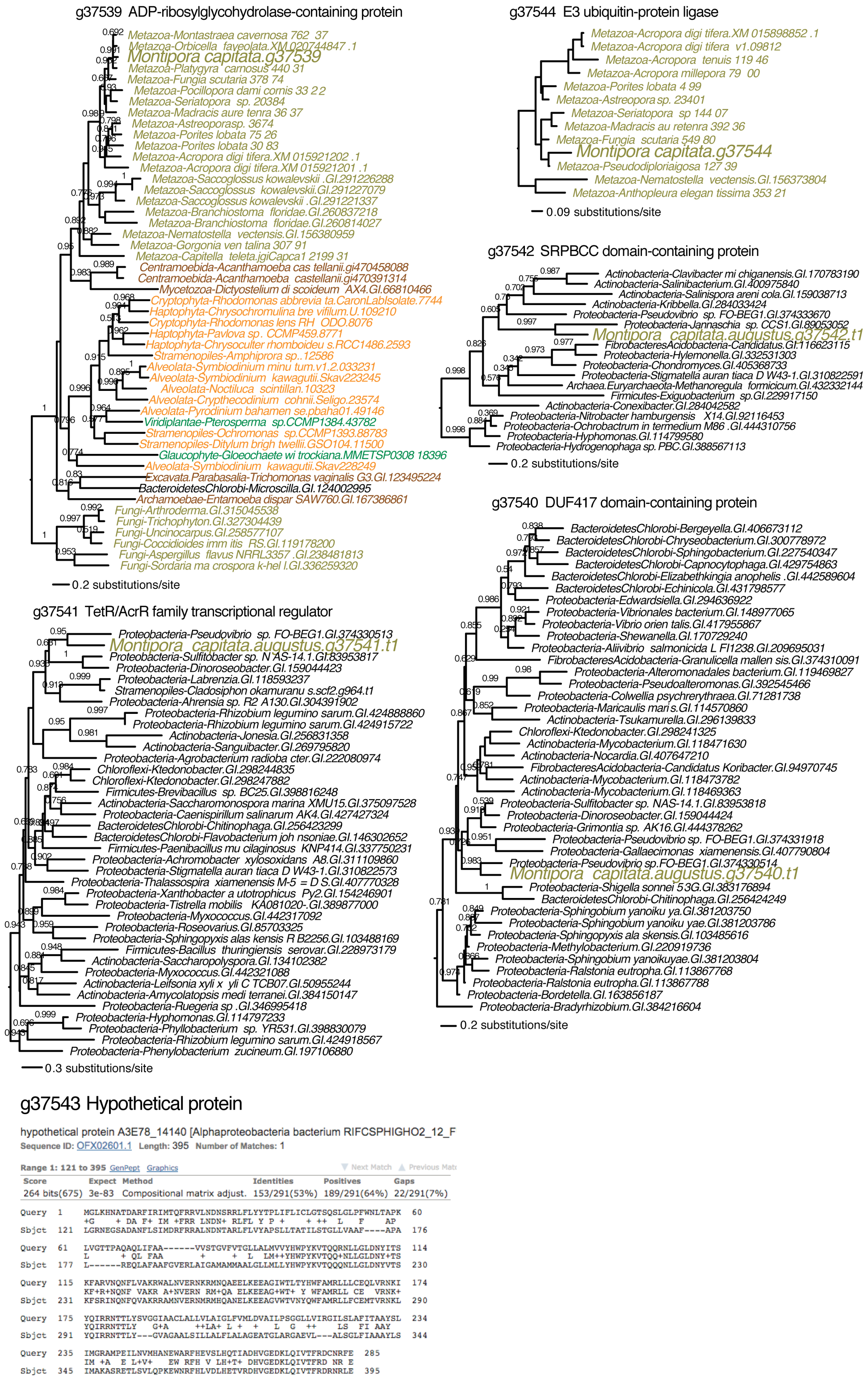

Figure S8. HGT in *M. capitata*. Phylogenetic trees for three of the HGT candidates and the flanking coral genes. Branch support (larger than 0.5) are local support values estimated using the Shimodaira-Hasegawa test (Shimodaira and Hasegawa, 1999). Below is shown the HGT candidate (g37543) that does not have sufficient homologs in NCBI GenBank for tree building. Therefore, the alignment with the top hit is provided to demonstrate its bacterial origin.

References

Shimodaira H, Hasegawa M. 1999. Multiple comparisons of log-likelihoods with applications to phylogenetic inference. *Mol Biol Evol*. 16(8):1114.

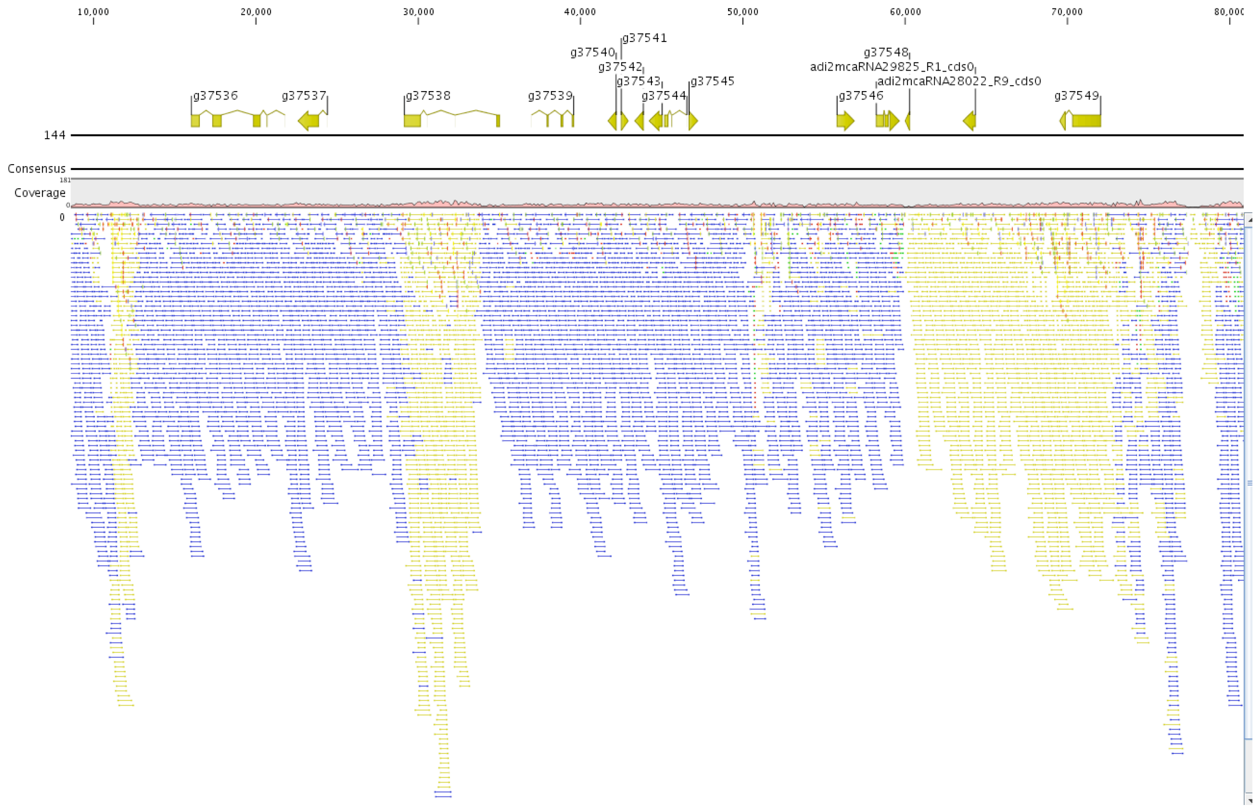

Figure S9. Mapping of Ilumina HiSeq reads to contig 144. These results demonstrate that the genomic region encompassing the HGTs has uniform coverage and is therefore unlikely to result from an assembly artifact. Paired-end reads (100 x 100 bp) are in blue, non-unique reads are in yellow and comprise SCORs that are widespread in the *M. capitata* genome. SNPs in individual reads (most or all at low frequency and therefore likely to be sequencing errors) are shown as hash marks.

A

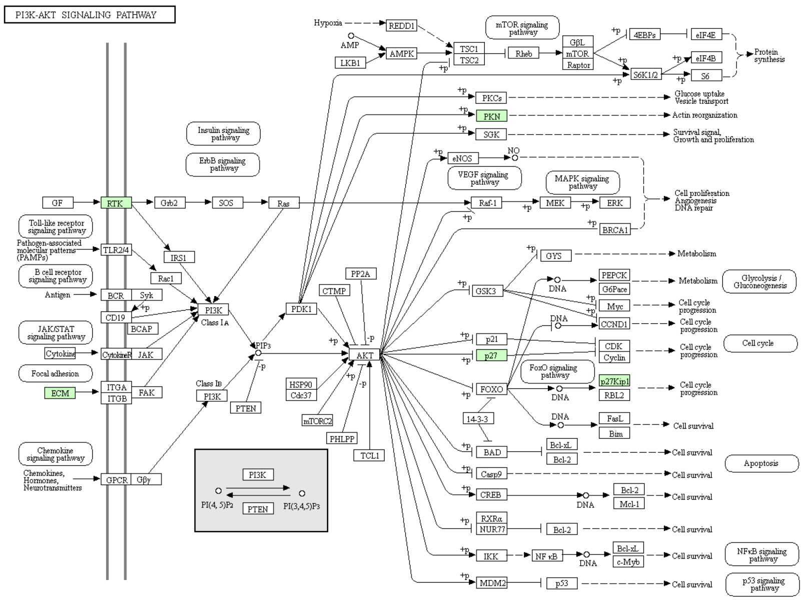

B

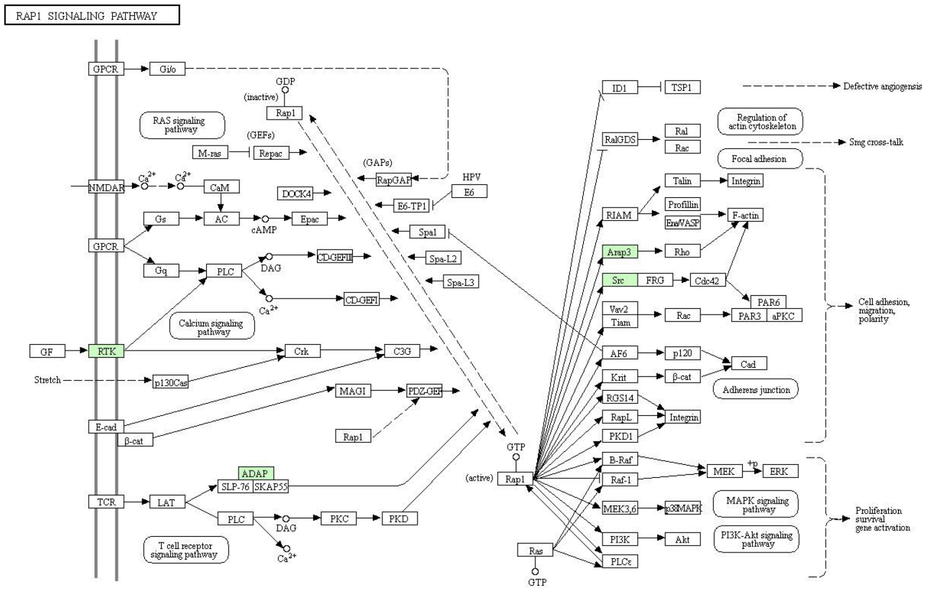

C

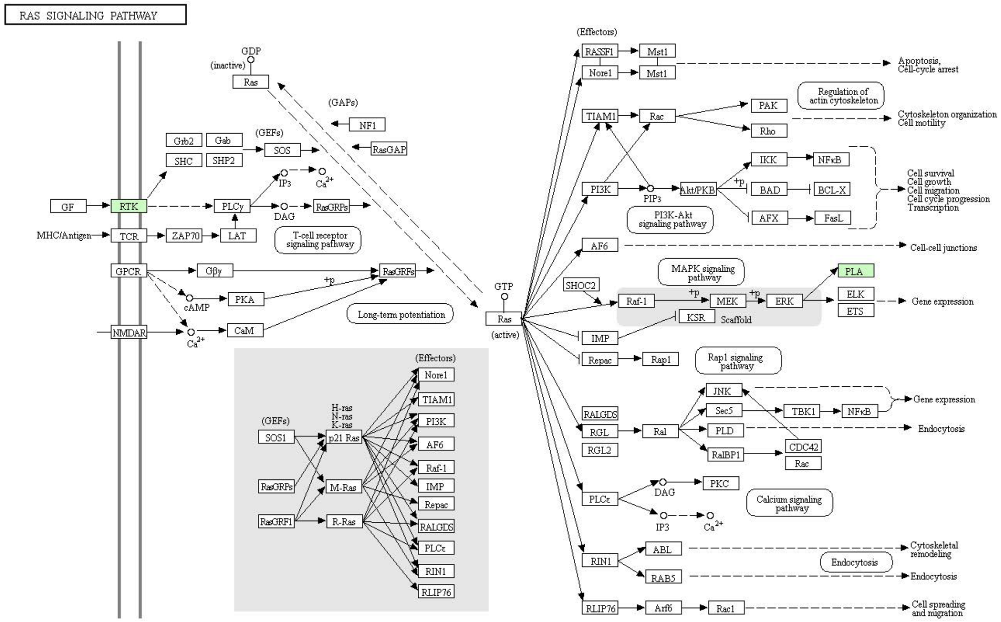

(continued)

D

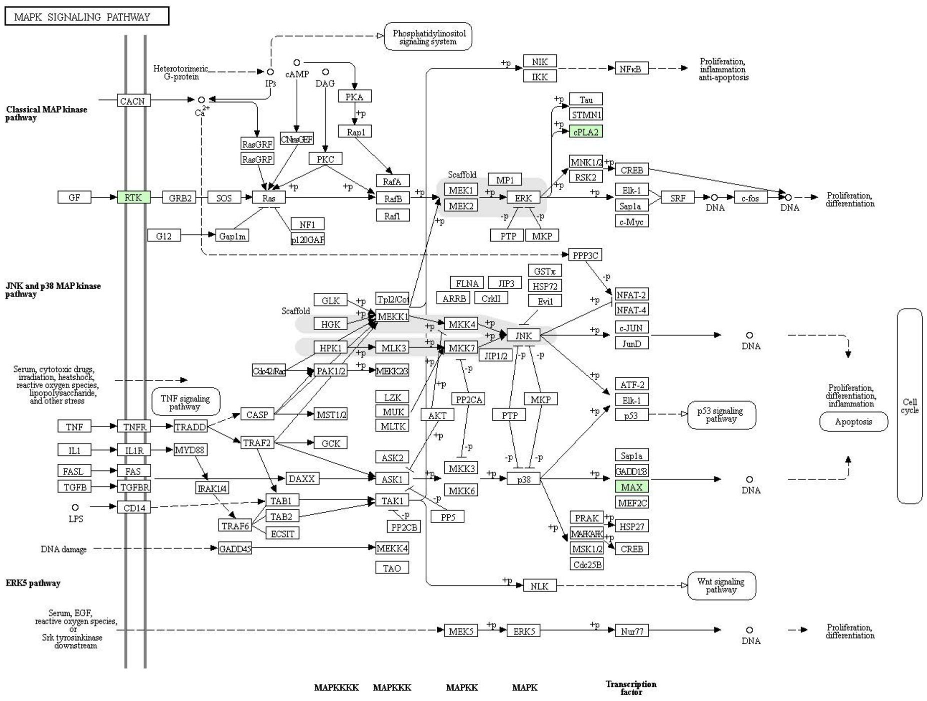
MAPK SIGNALING PATHWAY
Classical MAP kinase pathway
JNK and p38 MAP kinase pathway
ERK5 pathway
Phosphatidylinositol signaling system
Heterotrimeric G-protein
CACN
GF
RTK
GRB2
SOS
Ras
RafB
Raf1
MEK1
MEK2
ERK
MP1
PKA
Rap1
PKC
RasGRF
RasGRP
NF1
G12
Gap1m
PTP
MKP
NIK
IKK
NFκB
Proliferation, inflammation anti-apoptosis
Tau
STMN1
cPLA2
MNK1/2
RSK2
CREB
Elk-1
Sap1a
c-Myc
SRF
c-fos
DNA
Proliferation, differentiation
PPP3C
NFAT-2
NFAT-4
c-JUN
JunD
GLK
HGK
HPK1
MEKK1
MLK3
MKK4
MKK7
JNK
JIP1/2
FLNA
JIP3
ARRB
CrkII
GSTπ
HSP72
Evil
TNF signaling pathway
TNF
TNFR
TRADD
CASP
MST1/2
TRAF2
GCK
IL1
IL1R
MYD88
FASL
FAS
TGFB
TGFBR
LPS
CD14
IRAK1/4
DAXX
TAB1
TAB2
TRAF6
ECSIT
ASK2
ASK1
TAK1
MKK3
MKK6
p38
PP2CA
PP2CB
PP5
AKT
LZK
MUK
MLTK
PAK1/2
MEKK2/3
ATF-2
Elk-1
p53
p53 signaling pathway
Sap1a
GADD153
MAX
MEF2C
PRAK
MAPKAPK
MSK1/2
HSP27
CREB
Cdc25B
DNA damage
GADD45
MEKK4
TAO
NLK
Wnt signaling pathway
Proliferation, differentiation, inflammation
Apoptosis
Cell cycle
Serum, EGF, reactive oxygen species, or Src tyrosinkinase downstream
MEK5
ERK5
Nur77
MAPKKKK
MAPKKK
MAPKK
MAPK
Transcription factor

E

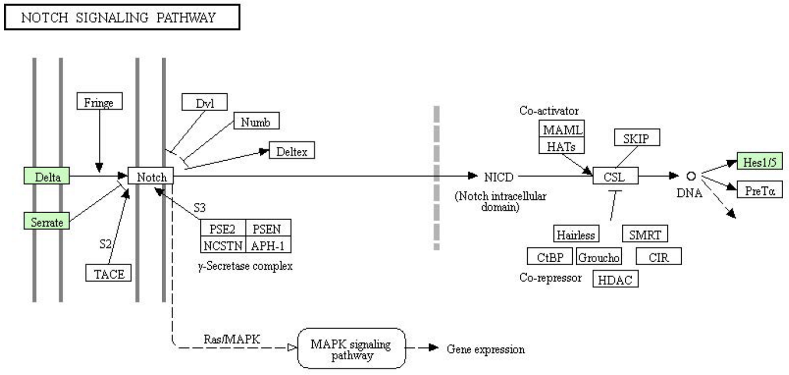
NOTCH SIGNALING PATHWAY
Fringe
Dvl
Numb
Deltex
Delta
Serrate
Notch
S2
S3
TACE
PSE2
PSEN
NCSTN
APH-1
γ-Secretase complex
NICD
(Notch intracellular domain)
Co-activator
MAML
HATs
SKIP
CSL
DNA
Hes1/5
PreTα
Hairless
SMRT
CtBP
Groucho
CIR
Co-repressor
HDAC
Ras/MAPK
MAPK signaling pathway
Gene expression

F

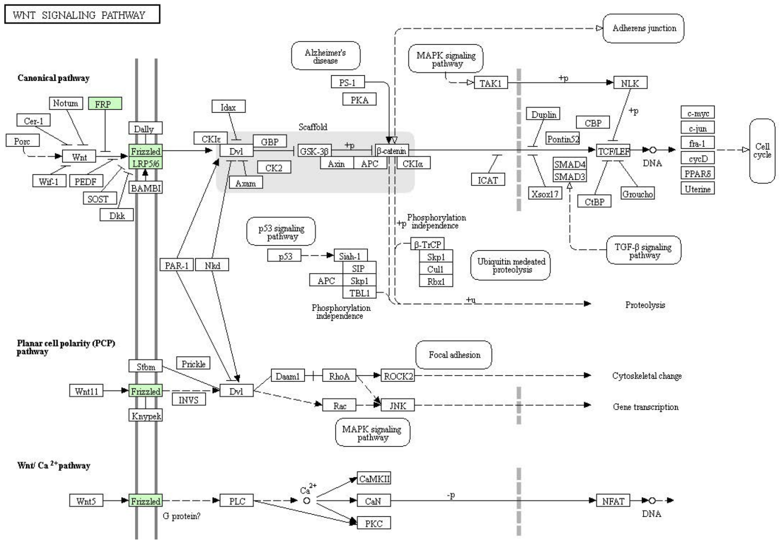
WNT SIGNALING PATHWAY
Canonical pathway
Notum
FRP
Cer-1
Porc
Wnt
Wif-1
PEDF
SOST
Dkk
Dally
Frizzled
LRP5/6
BAMBI
Idax
CKIε
Dvl
GBP
GSK-3β
CK2
Axin
APC
Azam
Scaffold
β-catenin
CKIα
PS-1
PKA
Alzheimer's disease
MAPK signaling pathway
TAK1
NLK
Adherens junction
Duplin
CBP
Pontin52
TCF/LEF
SMAD4
SMAD3
ICAT
Xsox17
CtBP
Groucho
TGF-β signaling pathway
c-myc
c-jun
fra-1
cycD
PPARδ
Uterine
DNA
Cell cycle
p53 signaling pathway
p53
Siah-1
SIP
APC
Skp1
TBL1
β-TrCP
Cul1
Rbx1
Phosphorylation independence
Ubiquitin mediated proteolysis
Proteolysis
PAR-1
Nkd
Planar cell polarity (PCP) pathway
Stbm
Prickle
Wnt11
Knypek
INVS
Daam1
RhoA
ROCK2
Rac
JNK
Focal adhesion
Cytoskeletal change
Gene transcription
Wnt/ Ca2+ pathway
Wnt5
PLC
CaMKII
CaN
PKC
NFAT
G protein?

G

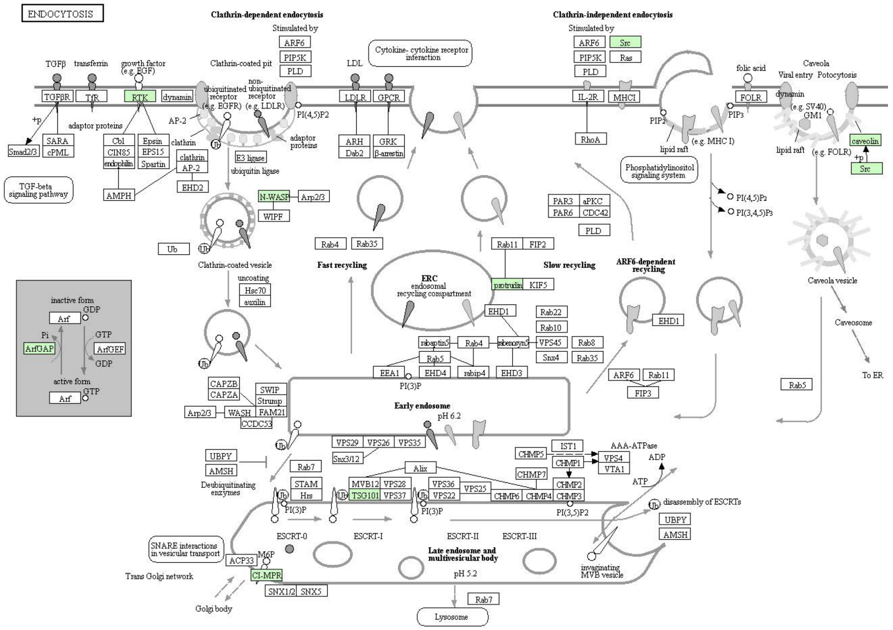
ENDOCYTOSIS
Clathrin-dependent endocytosis
Clathrin-independent endocytosis
Stimulated by
ARF6
PIP5K
PLD
Src
Ras
Cytokine- cytokine receptor interaction
TGFβ
transferrin
growth factor (e.g. EGF)
Clathrin-coated pit
LDL
TGFBR
TfR
RTK
dynamin
LDLR
GPCR
IL-2R
MHCI
FOLR
caveolin
Smad2/3
SARA
cPML
TGF-beta signaling pathway
Cbl
CIN85
endophilin
Epsin
EPS15
Spartin
clathrin
AP-2
EHD2
AMPH
ARH
Dab2
GRK
β-arrestin
RhoA
Phosphatidylinositol signaling system
N-WASP
Arp2/3
WIPF
Ub
Clathrin-coated vesicle
uncoating
Hsc70
auxilin
Rab4
Rab35
Fast recycling
ERC
endosomal recycling compartment
Rab11
FIP2
Slow recycling
ARF6-dependent recycling
protrudin
KIF5
PAR3
aPKC
PAR6
CDC42
EHD1
Rab22
Rab10
Rab8
Rab35
VPS45
Snx4
ARF6
Rab11
FIP3
EEA1
EHD4
Rab5
EHD3
PI(3)P
Early endosome
pH 6.2
CAPZB
CAPZA
SWIP
Strump
WASH
FAM21
CCDC53
Arp2/3
VPS29
VPS26
VPS35
Snx3/12
Rab7
UBPY
AMSH
Deubiquitinating enzymes
STAM
Hrs
MVB12
TSG101
VPS28
VPS37
Alix
VPS36
VPS22
VPS25
CHMP6
CHMP4
CHMP2
CHMP3
CHMP5
CHMP1
IST1
VPS4
VTA1
AAA-ATPase
ADP
ATP
disassembly of ESCRTs
UBPY
AMSH
ESCRT-0
ESCRT-I
ESCRT-II
ESCRT-III
PI(3,5)P2
Late endosome and multivesicular body
pH 5.2
invaginating MVB vesicle
SNARE interactions in vesicular transport
Trans Golgi network
ACP33
M6P
CI-MPR
SNX1/2
SNX5
Golgi body
Rab7
Lysosome
inactive form
Arf
GDP
Pi
ArfGAP
GTP
ArfGEF
active form
Caveola
Viral entry
Potocytosis
folic acid
dynamin
(e.g. SV40)
GM1
lipid raft
(e.g. FOLR)
(e.g. MHC I)
PIP3
Caveola vesicle
Caveosome
Rab5
To ER

H

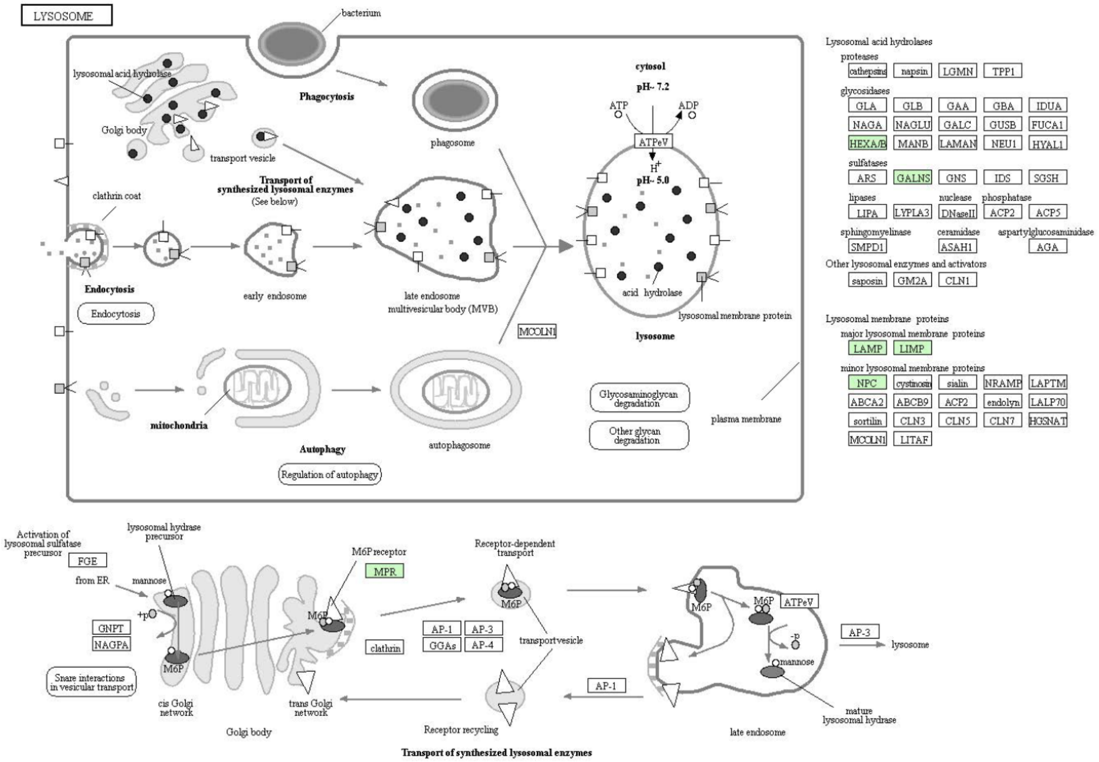
LYSOSOME
bacterium
lysosomal acid hydrolase
Golgi body
transport vesicle
Phagocytosis
phagosome
Transport of synthesized lysosomal enzymes
(See below)
clathrin coat
Endocytosis
early endosome
late endosome multivesicular body (MVB)
cytosol
pH~ 7.2
ATP
ADP
ATPeV
pH~ 5.0
acid hydrolase
lysosomal membrane protein
lysosome
MCOLN1
mitochondria
Autophagy
autophagosome
Regulation of autophagy
Glycosaminoglycan degradation
Other glycan degradation
plasma membrane
Lysosomal acid hydrolases
proteases
cathepsins
napsin
LGMN
TPP1
glycosidases
GLA
GLB
GAA
GBA
IDUA
NAGA
NAGLU
GALC
GUSB
FUCA1
HEXA/B
MANB
LAMAN
NEU1
HYAL1
sulfatases
ARS
GALNS
GNS
IDS
SGSH
lipases
nuclease
phosphatase
LIPA
LYPLA3
DNaseII
ACP2
ACP5
sphingomyelinase
ceramidase
aspartylglucosaminidase
SMPD1
ASAH1
AGA
Other lysosomal enzymes and activators
saposin
GM2A
CLN1
Lysosomal membrane proteins
major lysosomal membrane proteins
LAMP
LIMP
minor lysosomal membrane proteins
NPC
cystinosin
sialin
NRAMP
LAPTM
ABCA2
ABCB9
ACP2
endolyn
LALP70
sortilin
CLN3
CLN5
CLN7
HGSNAT
MCOLN1
LITAF
Activation of lysosomal sulfatase precursor
FGE
lysosomal hydrase precursor
from ER
mannose
GNPT
NAGPA
M6P
Snare interactions in vesicular transport
cis Golgi network
Golgi body
M6P receptor
MPR
clathrin
trans Golgi network
Receptor-dependent transport
AP-1
AP-3
GGAs
AP-4
transport vesicle
Receptor recycling
late endosome
mannose
mature lysosomal hydrase
lysosome
Transport of synthesized lysosomal enzymes

I

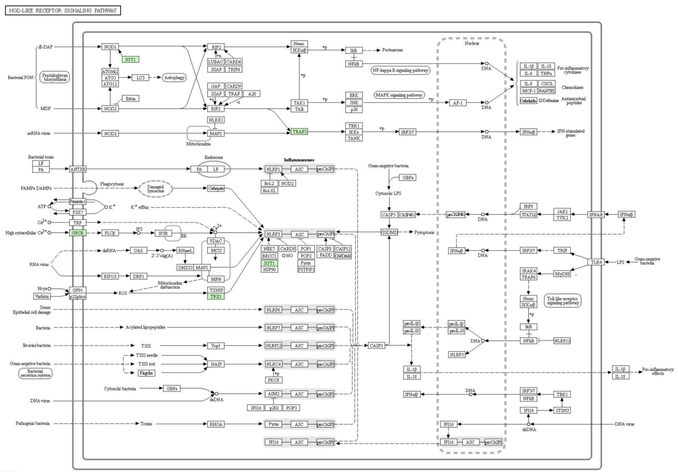
NOD-LIKE RECEPTOR SIGNALING PATHWAY

J

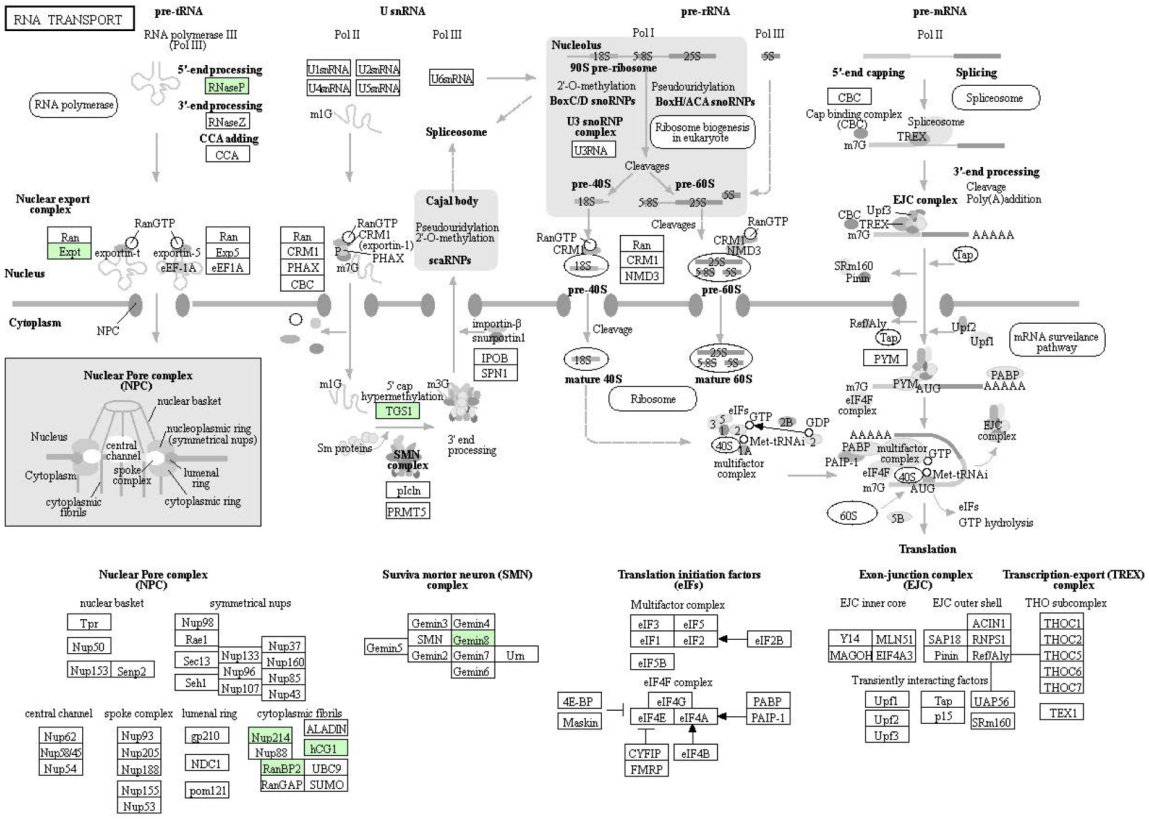

K

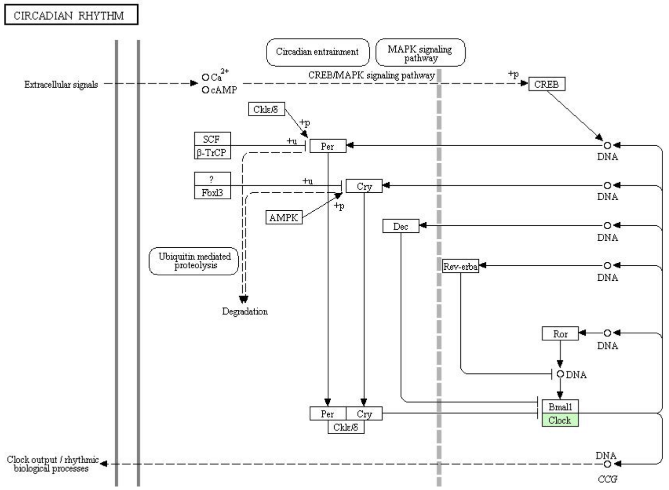

Figure S10 (above 4 pages). Mapping of genes that show evidence of positive selection on different KEGG pathways. Each pathway is shown individually with genes under diversifying selection in the green boxes. Pathways include (A) PI3K-AKT Signaling, (B) Rap1 Signaling, (C) Ras Signaling, (D) MAPK Signaling, (E) Notch Signaling, (F) Wnt Signaling, (G) Endocytosis, (H) Lysosome, (I) NOD-like Receptor Signaling, (J) RNA Transport, and (K) Circadian Rhythm.

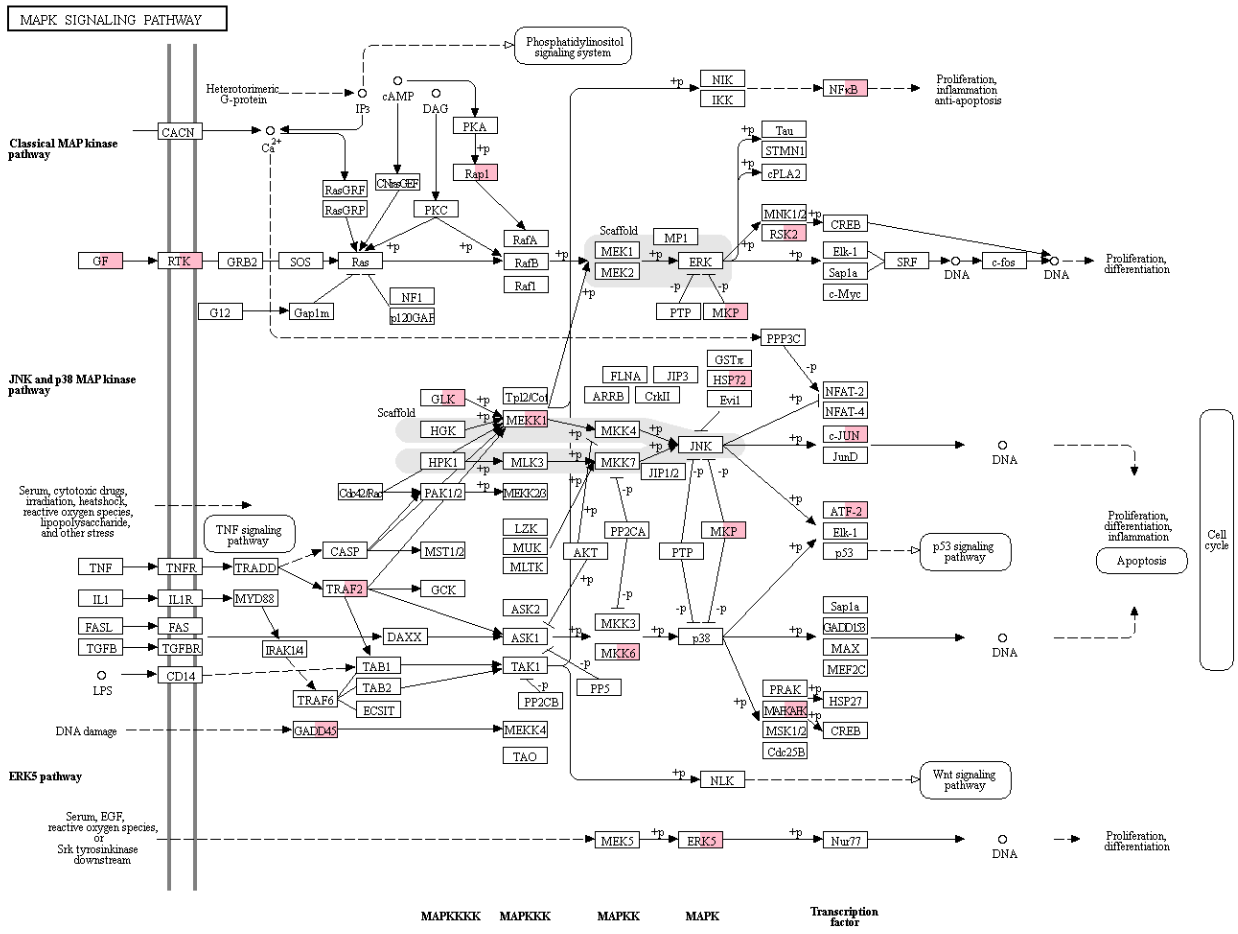

Figure S11. KEGG pathway analysis of genes that are significantly up- or down-regulated in the MAPK signaling pathway. In this figure, genes that are up-regulated after 6hr HTAC are shown in the red boxes.

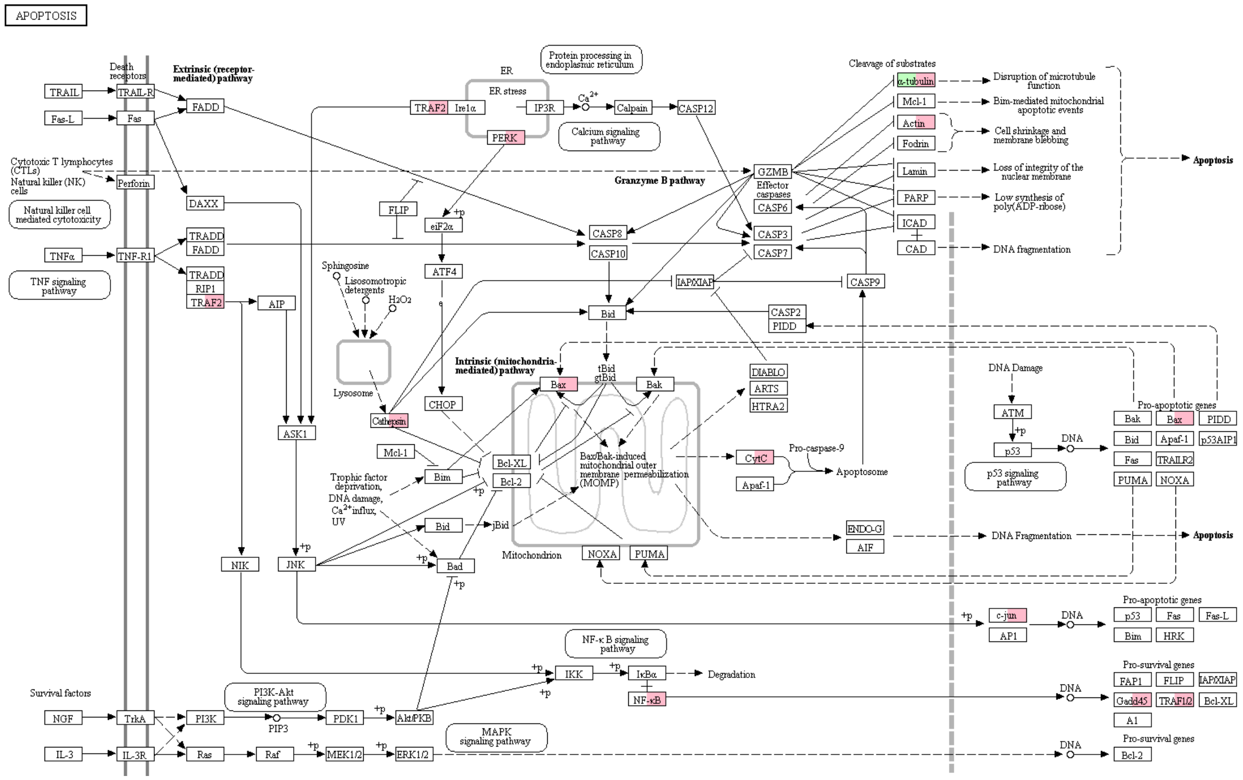

Figure S12. KEGG pathway analysis of genes that are significantly up- or down-regulated in the apoptosis pathway. In this figure, genes that are up-regulated after 6hr HTAC are shown in the red boxes and down-regulated in the green box.

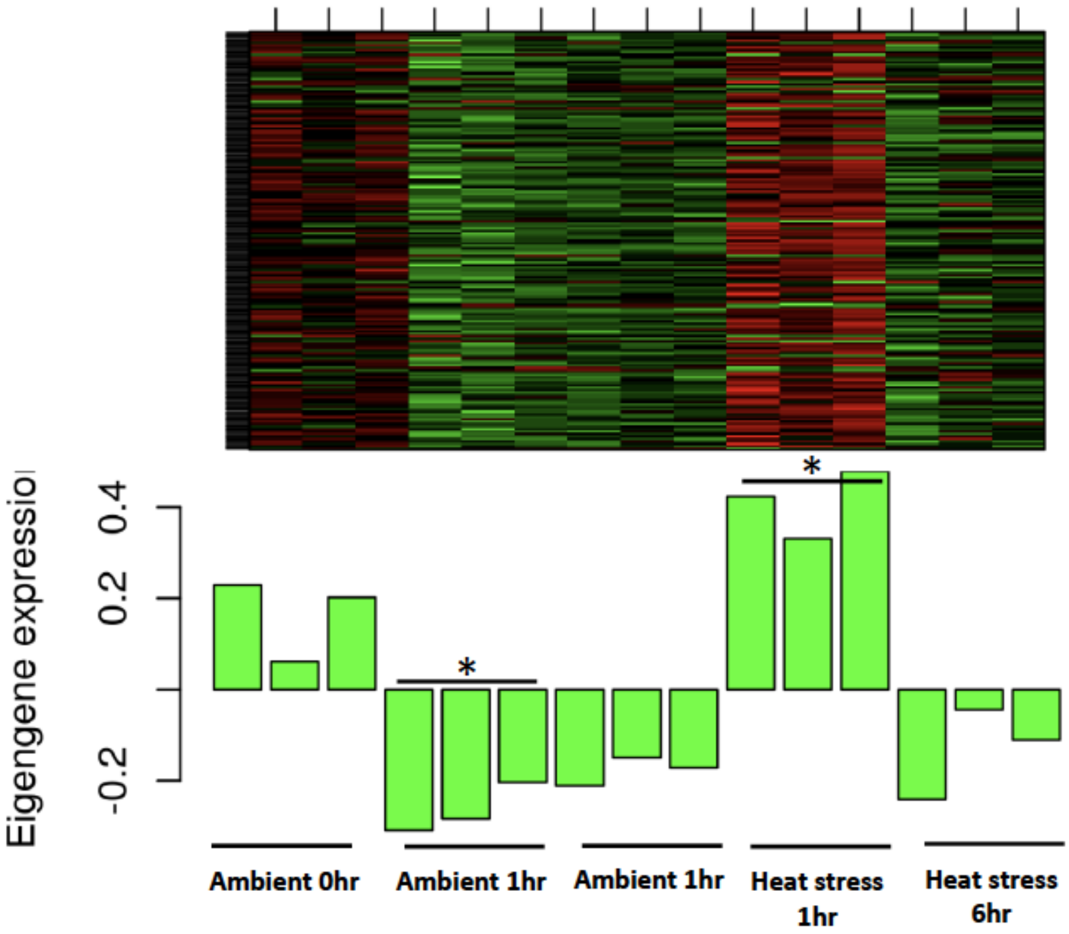

Figure S13. Analysis of the network green module. Bottom - eigengene expression values (y-axis), showing significant enrichment at 1hr [Fisher's exact test, *p*<0.05, Methods] of up-regulated gene contrasts (heat stress 1hr vs. ambient 1hr)] identified by DESeq2 as being significant (Table S9). Top - corresponding heatmap of module genes (rows) versus the same RNA-Seq treatment replicates (columns). Note that the module eigengene takes on high values in treatments (barplot columns) in which many module genes are up-regulated (red color in the heatmap).

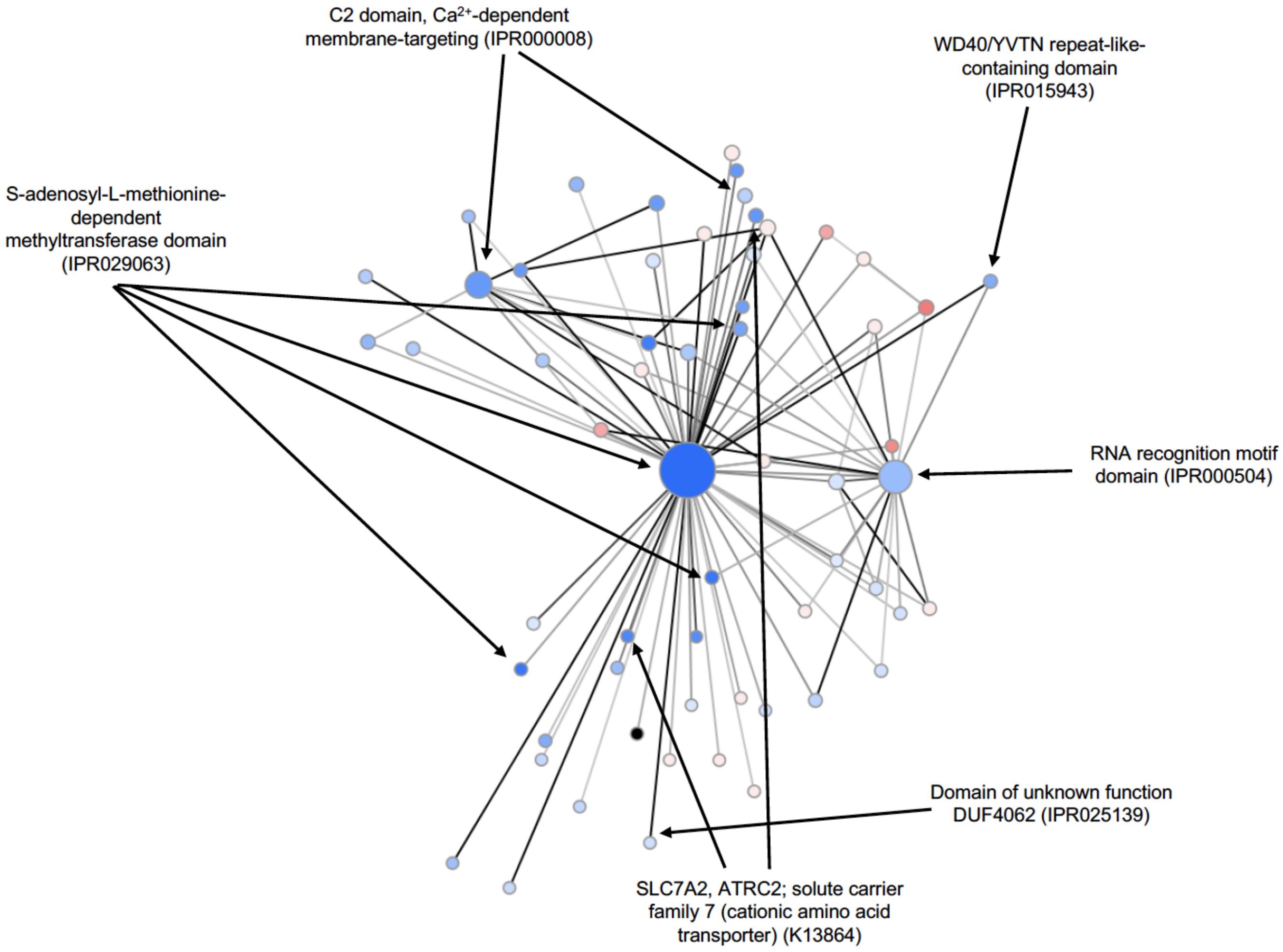

Figure S14. WGCNA analysis of the *M. capitata* RNA-Seq data. Network comprising significant enrichment of down-regulated genes following 6h of heat stress over 6hr ambient (control, p-value < 0.05, Fisher's exact test, Methods). All node annotations depicted in this figure were generated by identifying significantly enriched (p-value < 0.05, Fisher's exact test) KEGG orthologs (indicated by Kxxxxx) or InterPro domains (indicated by IPR0xxxxx).

Table S1. The top 20 gene families that show the greatest expansion in the *M. capitata* genome assembly when compared to *O. faveolata* (above) and the top 20 gene families that show the greatest expansion in the *M. capitata* genome assembly when compared to *S. pistillata* (below).

Representative gene	Orthogroup	*M. capitata* Count	*O. faveolata* Count	Fold change	Annotation
Monca.adi2mcaRNA13435_R6	OG0000030	230	1	230	RNA-directed DNA polymerase
Monca.adi2mcaRNA17760_R2	OG0000070	148	1	148	RNA-directed DNA polymerase
Monca.adi2mcaRNA12696_R7	OG0000104	124	1	124	ATP-dependent DNA helicase RecQ-like
Monca.adi2mcaRNA12308_R7	OG0000090	103	1	103	ATP-dependent DNA helicase Q1
Monca.adi2mcaRNA10592_R8	OG0000189	99	1	99	tigger transposable element-derived protein
Monca.augustus.g11509.t1	OG0000083	92	1	92	5-hydroxytryptamine receptor 1-lik
Monca.adi2mcaRNA12884_R1	OG0000210	89	1	89	uncharacterized protein
Monca.adi2mcaRNA14552_R6	OG0000172	86	1	86	Uncharacterized protein
Monca.adi2mcaRNA28123_R0	OG0000246	80	1	80	Uncharacterized protein
Monca.adi2mcaRNA15798_R3	OG0000079	156	2	78	uncharacterized protein
Monca.adi2mcaRNA14136_R0	OG0000014	301	4	75	52 kDa repressor of the inhibitor of the protein kinase
Monca.adi2mcaRNA15585_R8	OG0000288	75	1	75	Uncharacterized protein
Monca.adi2mcaRNA32317_R1	OG0000247	75	1	75	Uncharacterized protein
Monca.augustus.g10303.t1	OG0000239	75	1	75	Uncharacterized protein
Monca.adi2mcaRNA28536_R2	OG0000270	67	1	67	Uncharacterized protein
Monca.adi2mcaRNA13435_R1	OG0000037	198	3	66	RNA-directed DNA polymerase
Monca.augustus.g10557.t1	OG0000454	64	1	64	Uncharacterized protein
Monca.adi2mcaRNA11304_R0	OG0000076	63	1	63	RNA-directed DNA polymerase
Monca.adi2mcaRNA13417_R3	OG0000008	441	7	63	Uncharacterized protein
Monca.adi2mcaRNA16418_R0	OG0000311	63	1	63	Uncharacterized protein

Representative gene	Orthogroup	*M. capitata* Count	*S. pistillata* Count	Fold change	Annotation
Monca.adi2mcaRNA14585_R0	OG0000023	263	1	263	Uncharacterized protein
Monca.adi2mcaRNA17449_R7	OG0000093	125	1	125	P2X purinoceptor 7-like
Monca.adi2mcaRNA10592_R8	OG0000189	99	1	99	tigger transposable element-derived protein
Monca.adi2mcaRNA1598_R7	OG0000044	184	2	92	Uncharacterized protein
Monca.augustus.g11509.t1	OG0000083	92	1	92	5-hydroxytryptamine receptor 1-like
Monca.adi2mcaRNA9616_R2	OG0000208	88	1	88	Retrovirus-related Pol polyprotein
Monca.adi2mcaRNA34736_R8	OG0000188	83	1	83	Retrovirus-related Pol polyprotein
Monca.adi2mcaRNA12307_R0	OG0000185	77	1	77	Uncharacterized protein
Monca.adi2mcaRNA15585_R8	OG0000288	75	1	75	Uncharacterized protein
Monca.adi2mcaRNA10499_R0	OG0000035	221	3	73	Uncharacterized protein
Monca.adi2mcaRNA17438_R1	OG0000263	73	1	73	Uncharacterized protein
Monca.adi2mcaRNA17251_R1	OG0000066	142	2	71	Uncharacterized protein
Monca.adi2mcaRNA10989_R2	OG0000087	137	2	68	Mitogen-activated protein kinase

Monca.adi2mcaRNA28070_R1	OG0000258	68	1	68	Uncharacterized protein
Monca.adi2mcaRNA10392_R8	OG0000081	132	2	66	Uncharacterized protein
Monca.adi2mcaRNA13435_R1	OG0000037	198	3	66	RNA-directed DNA polymerase
Monca.augustus.g10833.t1	OG0000231	65	1	65	ATP-dependent DNA helicase RecQ
Monca.augustus.g11407.t1	OG0000431	65	1	65	Endonuclease-reverse transcriptase
Monca.adi2mcaRNA36639_R0	OG0000233	64	1	64	Uncharacterized protein
Monca.adi2mcaRNA13417_R3	OG0000008	441	7	63	Uncharacterized protein

Table S2. The total number of base pairs (bp) in different regions of the *M. capitata* genome.

	Total	Repeats	Percent Repeats
**Genome**	885,704,498	408,047,463	46.07%
**Intergenic**	444,311,938	250,433,791	56.36%
**Genic**	441,392,560	157,613,672	35.71%
**Introns**	337,760,784	157,218,910	46.55%
**Coding DNA sequence (CDS)**	103,631,776	394,762	0.38%

Table S3. RNA-Seq experimental conditions and gene expression data.

(A) Gene expression levels in the region of contig 144 that encodes the four HGT candidates that are derived from a bacterial source; (D) the temperature conditions across treatment tanks at HIMB for the 1h and 6h exposures.
(B) The list of DE genes in M. capitata under the conditions used in this work. Blastx, KEGG, and Blast2GO assignments for the DE genes are also shown;
(C) the list of DE genes in M. capitata under aforementioned conditions for which there were either no Blastx best hit, or for which the best hit was a hypothetical or uncharacterized protein. In addition to Blastx, KEGG and Blast2GO assignments, the TargetP and SignalP output are provided for DE genes with a TargetP RC designation ≤ 3;
(D) the temperature conditions across treatment tanks at HIMB for the 1h and 6h exposures.

Contig144		CLC Genomics Workbench, RNA-Seq Analysis Tool, Mean Unique Counts													BestHits_BLASTx	
**GeneOrder**	**CDS**	**ATAC_0**	**ATAC_1**	**ATHC_1**	**HTAC_1**	**ATAC_6**	**ATHC_6**	**HTAC_6**	**MeanCounts_AllLibraries**	**St.Dev**	**QuerLen**	**SubLen**	**% Identity**	**AlignLen**	**BestMatch_No.**	**BestMatch_Description**
1	augustus.g37535.t1	2.000	12.000	12.000	1.667	2.000	1.333	1.333	4.619	5.049	570	618	77.1	620	>XP_015763771	PREDICTED: uncharacterized protein LOC107342775 [Acropora digitifera].
2	augustus.g37536.t1	5.667	11.667	21.667	0.000	0.000	0.000	8.667	6.810	8.044	589	629	77.6	505	>XP_020611931	beta-1,4-N-acetylgalactosaminyltransferase 3-like [Orbicella faveolata].
3	augustus.g37537.t1	35.333	92.000	106.667	0.000	0.000	0.000	104.333	48.333	51.028	462	456	72.6	446	>XP_020632959	transient receptor potential cation channel subfamily A member 1-like [Orbicella faveolata].
4	augustus.g37538.t1	1.333	2.667	0.667	0.000	0.000	0.000	2.667	1.048	1.208	456	246	39.5	129	>XP_001629094	predicted protein [Nematostella vectensis].
5	augustus.g37539.t1	3.333	13.000	23.667	0.000	0.000	0.000	4.667	6.381	8.916	157	469	86.5	156	>XP_020600506	uncharacterized protein LOC110039705 [Orbicella faveolata].
6	augustus.g37540.t1	1.000	0.000	1.667	0.000	0.000	0.000	0.000	0.381	0.678	185	166	58.5	164	>WP_014284837	membrane protein [Pseudovibrio sp. FO-BEG1].
7	augustus.g37541.t1	0.333	0.333	0.667	0.000	0.000	0.000	0.000	0.190	0.262	159	193	44.9	156	>WP_019960938	TetR family transcriptional regulator [Woodsholea maritima].
8	augustus.g37542.t1	0.667	0.667	0.667	0.000	0.000	0.000	0.000	0.286	0.356	194	160	27.4	157	>OGN73568	ATPase [Chloroflexi bacterium GWB2_54_36].
9	augustus.g37543.t1	1.333	0.000	1.667	0.000	0.000	0.000	1.667	0.667	0.839	286	395	52.9	291	>OFX02601	hypothetical protein A3E78_14140 [Alphaproteobacteria bacterium RIFCSPHIGHO2_12_FULL_63_12].
10	augustus.g37544.t1	0.333	0.333	2.667	0.000	0.000	0.000	0.667	0.571	0.957	148	326	87.5	56	>XP_020630661	probable E3 ubiquitin-protein ligase RNF144A-B isoform X1 [Orbicella faveolata].
11	augustus.g37545.t1	1.333	0.333	1.667	0.000	0.000	0.000	0.000	0.476	0.716	194	347	34.9	192	>XP_020620978	uncharacterized protein LOC110058664 [Orbicella faveolata].
13	adi2mcaRNA28022_R9	6.333	2.333	5.000	1.667	1.333	1.667	2.667	3.000	1.915	466	276	75.6	275	>XP_015772606	PREDICTED: uncharacterized protein K02A2.6-like [Acropora digitifera].
15	adi2mcaRNA29825_R1	3.000	0.000	0.667	0.667	0.667	1.667	4.667	1.619	1.660	272	279	79.7	271	>XP_015774249	PREDICTED: uncharacterized protein LOC107352430 [Acropora digitifera].
17	augustus.g37550.t1	1.000	0.000	0.333	2.000	1.000	1.000	0.667	0.857	0.634	359	300	26.3	297	>XP_020607210	uncharacterized protein LOC110045894 [Orbicella faveolata].
18	adi2mcaRNA30107_R3	0.333	1.667	0.000	0.000	0.333	1.667	6.333	1.476	2.260	1765	2000	56.4	2002	>XP_015757880	PREDICTED: ankyrin repeat domain-containing protein 50-like isoform X1 [Acropora digitifera].
19	augustus.g37555.t1	373.333	674.333	354.333	399.333	406.000	353.667	555.667	445.238	122.573	1561	1318	71.3	1321	>XP_015757883	PREDICTED: DNA repair protein RAD50-like isoform X1 [Acropora digitifera].
20	augustus.g37556.t1	8.000	16.000	10.333	20.333	14.000	13.333	16.000	14.000	4.041	n/a	n/a	n/a	n/a	n/a	n/a
21	augustus.g37557.t1	2.667	3.667	6.333	19.333	14.667	8.667	6.000	8.762	6.100	184	149	65.6	122	>XP_015757894	PREDICTED: uncharacterized protein LOC107337279 [Acropora digitifera].
22	augustus.g37558.t1	0.000	0.000	0.000	1.667	3.333	7.667	0.000	1.810	2.879	158	545	37.4	163	>XP_001619242	hypothetical protein NEMVEDRAFT_v1g224366 [Nematostella vectensis].
23	augustus.g37559.t1	34.667	32.667	33.333	87.333	47.000	39.667	39.333	44.857	19.371	146	149	66.2	136	>XP_015757894	PREDICTED: uncharacterized protein LOC107337279 [Acropora digitifera].
24	augustus.g37560.t1	8.000	8.667	11.000	17.667	10.667	6.667	8.333	10.143	3.646	n/a	n/a	n/a	n/a	n/a	n/a
25	adi2mcaRNA10633_R0	0.333	0.000	0.333	0.667	0.000	0.333	0.000	0.238	0.252	475	495	85.7	495	>XP_015756847	PREDICTED: uncharacterized protein K02A2.6-like [Acropora digitifera].
26	augustus.g37564.t1	0.000	0.000	0.000	0.333	0.000	0.000	0.000	0.048	0.126	136	389	68.5	127	>KXJ07802	hypothetical protein AC249_AIPGENE8511 [Exaiptasia pallida].
27	augustus.g37565.t1	0.000	0.000	0.000	0.000	0.333	0.000	0.000	0.048	0.126	n/a	n/a	n/a	n/a	n/a	n/a
28	adi2mcaRNA11747_R1	148.333	95.000	119.000	109.000	80.333	107.000	34.000	98.952	35.560	247	260	34.9	232	>XP_015757899	PREDICTED: uncharacterized protein LOC107337285 [Acropora digitifera].
29	adi2mcaRNA11746_R1	684.333	310.667	489.000	441.333	360.000	350.000	218.667	407.714	150.011	3539	3613	52.9	3619	>XP_015757898	PREDICTED: uncharacterized protein LOC107337284 [Acropora digitifera].
30	augustus.g37570.t1	20.333	4.333	9.667	11.667	7.333	6.667	5.667	9.381	5.417	392	3224	55.4	287	>XP_020627018	uncharacterized protein LOC110064321 [Orbicella faveolata].
31	augustus.g37572.t1	0.000	0.000	0.000	0.000	0.333	0.000	0.000	0.048	0.126	n/a	n/a	n/a	n/a	n/a	n/a
32	augustus.g37573.t1	0.000	0.000	0.000	0.000	0.000	0.000	0.000	0.000	0.000	n/a	n/a	n/a	n/a	n/a	n/a
33	augustus.g37574.t1	9.000	2.667	13.667	4.000	3.667	5.667	3.667	6.048	3.955	n/a	n/a	n/a	n/a	n/a	n/a
34	augustus.g37575.t1	13.000	11.000	7.667	8.333	5.333	7.333	1.000	7.667	3.873	142	861	45.6	136	>XP_015766876	PREDICTED: uncharacterized protein LOC107345652 [Acropora digitifera].
35	augustus.g37576.t1	4.000	6.000	3.000	3.333	3.000	2.333	2.333	3.429	1.272	270	154	89.4	142	>XP_015780861	PREDICTED: uncharacterized protein LOC107358790 [Acropora digitifera].
36	adi2mcaRNA30722_R8	2.000	2.667	1.333	2.333	2.333	1.000	3.333	2.143	0.790	377	407	85.8	408	>XP_015775014	PREDICTED: putative nuclease HARBI1 [Acropora digitifera].

Immediate flanking coral genes
Bacterium-derived potential HGT

a

SequenceID	ATHC1vsATAC1 Log2FoldChange	ATHC1vsATAC1 pAdj	HTAC1vsATAC1 Log2FoldChange	HTAC1vsATAC1 pAdj	ATHC6vsATAC6 Log2FoldChange	ATHC6vsATAC6 pAdj	HTAC6vsATAC6 Log2FoldChange	HTAC6vsATAC6 pAdj	Blastx Best Hit	AccessionNo	% Identity	ExpectValue	KEGG KO Term	#GO	GO IDs	GO Names	Enzyme Code	Enzyme Name	InterPro IDs	InterPro GO IDs	InterPro GO Names
adi2mcaRNA10072_R0	n/a	n/a	n/a	n/a	n/a	n/a	1.356467308	0.014701801	PREDICTED: protocadherin-like protein [Acropora digitifera].	XP_015756343	75.9	2.70E-306	K16506	n/a	n/a	n/a	n/a	n/a	n/a	n/a	n/a
adi2mcaRNA10278_R1	n/a	n/a	n/a	n/a	n/a	n/a	-1.39963612	0.025858241	PREDICTED: methyltransferase-like protein 2 [Acropora digitifera].	XP_015756492	61.4	4.50E-115	n/a	n/a	n/a	n/a	n/a	n/a	n/a	n/a	n/a
adi2mcaRNA10631_R0	n/a	n/a	n/a	n/a	n/a	n/a	1.376099208	0.014153797	PREDICTED: fibroblast growth factor receptor 3-like [Acropora digitifera].	XP_015756845	60.3	1.60E-188	K05094	n/a	n/a	n/a	n/a	n/a	n/a	n/a	n/a
adi2mcaRNA10726_R0	n/a	n/a	2.923696264	0.000555389	n/a	n/a	n/a	n/a	PREDICTED: SCO-spondin-like [Acropora digitifera].	XP_015756907	61.3	0.00E+00	n/a	n/a	n/a	n/a	n/a	n/a	n/a	n/a	n/a
adi2mcaRNA10726_R3	n/a	n/a	n/a	n/a	n/a	n/a	1.108095222	8.50E-05	Coadhesin [Exaiptasia pallida].	KXJ21074	47.4	2.40E-197	n/a	n/a	n/a	n/a	n/a	n/a	n/a	n/a	n/a
adi2mcaRNA10795_R1	n/a	n/a	n/a	n/a	n/a	n/a	-2.428192343	0.005248239	PREDICTED: L-galactose dehydrogenase-like [Acropora digitifera].	XP_015757013	86.1	1.20E-173	n/a	2	F:GO:001649	F:oxidoreduct	n/a		IPR020471	(IF:GO:001649	F:oxidoreductase activity; P:oxidation-reduct[illegible]
adi2mcaRNA1099_R0	n/a	n/a	n/a	n/a	n/a	n/a	1.881422747	0.030977867	PREDICTED: adhesion G protein-coupled receptor L4-like [Acropora digitifera].	XP_015757727	61.9	4.50E-239	n/a	n/a	n/a	n/a	n/a	n/a	n/a	n/a	n/a
adi2mcaRNA11091_R0	n/a	n/a	n/a	n/a	n/a	n/a	2.229318604	1.56E-10	PREDICTED: low-density lipoprotein receptor-related protein 4-like [Acropora digitifera].	XP_015757208	41	1.60E-70	n/a	n/a	n/a	n/a	n/a	n/a	n/a	n/a	n/a
adi2mcaRNA11158_R0	n/a	n/a	n/a	n/a	n/a	n/a	1.776998689	9.28E-08	E3 ubiquitin-protein ligase RNF19A-like isoform X2 [Orbicella faveolata].	XP_020631035	89.8	3.40E-282	K11972	10	C:GO:000581	C:centrosome	n/a		IPR001841	(IF:GO:000484	F:ubiquitin-protein transferase activity; P:pro[illegible]
adi2mcaRNA11174_R0	n/a	n/a	n/a	n/a	n/a	n/a	1.078103268	0.002625854	PREDICTED: metabotropic glutamate receptor 3-like [Acropora digitifera].	XP_015757359	75.9	0.00E+00	n/a	11	F:GO:000164	F:group II men	n/a		IPR000337	(IF:GO:000493	F:G-protein coupled receptor activity; P:G-pr[illegible]
adi2mcaRNA11316_R0	n/a	n/a	n/a	n/a	n/a	n/a	-3.489941863	0.01777476	PREDICTED: uncharacterized protein LOC107336922 isoform X2 [Acropora digitifera].	XP_015757495	49.8	8.40E-50	n/a	n/a	n/a	n/a	n/a	n/a	n/a	n/a	n/a
adi2mcaRNA11417_R3	n/a	n/a	n/a	n/a	n/a	n/a	2.397489299	0.017576093	PREDICTED: uncharacterized protein K02A2.6-like [Acropora digitifera].	XP_015757563	90.2	0.00E+00	n/a	n/a	n/a	n/a	n/a	n/a	n/a	n/a	n/a
adi2mcaRNA11417_R9	n/a	n/a	n/a	n/a	n/a	n/a	1.320078149	0.023982573	PREDICTED: uncharacterized protein K02A2.6-like [Acropora digitifera].	XP_015757563	93.2	0.00E+00	n/a	n/a	n/a	n/a	n/a	n/a	n/a	n/a	n/a
adi2mcaRNA11437_R1	n/a	n/a	n/a	n/a	n/a	n/a	-1.040188986	0.035535155	PREDICTED: uncharacterized protein LOC107336976 [Acropora digitifera].	XP_015757568	56.1	4.10E-294	n/a	6	P:GO:005098	P:detection of	n/a		IPR001024	(IF:GO:000551	F:protein binding
adi2mcaRNA11453_R0	n/a	n/a	n/a	n/a	n/a	n/a	2.674216199	5.97E-11	PREDICTED: cytochrome b561 and DOMON domain-containing protein At3g25290-like [Acropora digi[illegible]	XP_015757575	71	8.90E-185	n/a	3	C:GO:001602	C:membrane;	n/a		IPR006593	(IF:GO:000551	F:protein binding
adi2mcaRNA116_R0	n/a	n/a	n/a	n/a	n/a	n/a	1.021888092	0.026977612	PREDICTED: caspase-3-like isoform X2 [Acropora digitifera].	XP_015762449	62.7	3.20E-125	n/a	n/a	n/a	n/a	n/a	n/a	n/a	n/a	n/a
adi2mcaRNA11727_R1	n/a	n/a	n/a	n/a	n/a	n/a	1.383044508	0.042921591	PREDICTED: DNA repair protein RAD50-like isoform X1 [Acropora digitifera].	XP_015757883	71.1	3.90E-253	K10866	n/a	n/a	n/a	n/a	n/a	n/a	n/a	n/a
adi2mcaRNA11949_R0	n/a	n/a	n/a	n/a	n/a	n/a	1.522832673	7.01E-15	solute carrier family 26 member alpha [Stylophora pistillata].	AJQ31793	66.3	6.70E-225	K14704	3	F:GO:001510	F:inorganic an	n/a		IPR002645	(IP:GO:000827	P:sulfate transport; F:secondary active sulfate[illegible]
adi2mcaRNA12029_R0	n/a	n/a	n/a	n/a	n/a	n/a	1.340263475	0.003046133	PREDICTED: uncharacterized protein LOC107337462 [Acropora digitifera].	XP_015758104	45.6	9.10E-49	n/a	n/a	n/a	n/a	n/a	n/a	n/a	n/a	n/a
adi2mcaRNA12219_R1	n/a	n/a	n/a	n/a	n/a	n/a	2.299898766	0.000738816	probable cubilin [Orbicella faveolata].	XP_020616735	49.9	3.90E-104	n/a	n/a	n/a	n/a	n/a	n/a	n/a	n/a	n/a
adi2mcaRNA12220_R1	n/a	n/a	n/a	n/a	n/a	n/a	1.776398545	0.003692826	PREDICTED: TNF receptor-associated factor 3-like [Acropora digitifera].	XP_015758361	69.5	3.80E-230	K03174	4	F:GO:000551	F:protein bind	n/a		IPR002083	(IF:GO:000827	F:zinc ion binding; F:protein binding; F:meta[illegible]
adi2mcaRNA12268_R6	n/a	n/a	n/a	n/a	n/a	n/a	1.68161779	0.048501244	PREDICTED: uncharacterized protein LOC107337644 [Acropora digitifera].	XP_015758342	47.3	5.40E-103	n/a	n/a	n/a	n/a	n/a	n/a	n/a	n/a	n/a
adi2mcaRNA12268_R7	n/a	n/a	n/a	n/a	n/a	n/a	2.147443413	0.000303394	PREDICTED: uncharacterized protein LOC107337644 [Acropora digitifera].	XP_015758342	45.9	5.40E-92	n/a	n/a	n/a	n/a	n/a	n/a	n/a	n/a	n/a
adi2mcaRNA12268_R9	n/a	n/a	n/a	n/a	n/a	n/a	1.362682224	0.00069464	PREDICTED: uncharacterized protein LOC107337644 [Acropora digitifera].	XP_015758342	46.5	3.80E-81	n/a	n/a	n/a	n/a	n/a	n/a	n/a	n/a	n/a
adi2mcaRNA12272_R0	n/a	n/a	n/a	n/a	n/a	n/a	3.550411157	5.82E-06	PREDICTED: uncharacterized protein LOC107337647 [Acropora digitifera].	XP_015758345	59.6	4.20E-275	K05126	n/a	n/a	n/a	n/a	n/a	n/a	n/a	n/a
adi2mcaRNA12316_R2	n/a	n/a	n/a	n/a	n/a	n/a	1.453397117	0.013274591	PREDICTED: uncharacterized protein LOC107337721 [Acropora digitifera].	XP_015758434	79.9	0.00E+00	n/a	n/a	n/a	n/a	n/a	n/a	n/a	n/a	n/a
adi2mcaRNA12344_R0	n/a	n/a	2.29039313	0.029289229	n/a	n/a	n/a	n/a	PREDICTED: mitochondrial coenzyme A transporter SLC25A42-like [Acropora digitifera].	XP_015758439	84	1.70E-85	K15085	13	F:GO:001521	F:purine nucle	n/a		n/a	n/a	n/a
adi2mcaRNA12355_R2	n/a	n/a	n/a	n/a	n/a	n/a	1.911670177	2.35E-08	PREDICTED: uncharacterized protein LOC107337766 [Acropora digitifera].	XP_015758484	56.3	2.60E-45	n/a	n/a	n/a	n/a	n/a	n/a	n/a	n/a	n/a
adi2mcaRNA12356_R8	n/a	n/a	n/a	n/a	n/a	n/a	2.356523954	0.001737988	PREDICTED: uncharacterized protein LOC107337765 [Acropora digitifera].	XP_015758483	55.3	5.40E-145	n/a	n/a	n/a	n/a	n/a	n/a	n/a	n/a	n/a
adi2mcaRNA12403_R0	n/a	n/a	n/a	n/a	n/a	n/a	1.249919583	5.00E-06	PREDICTED: nucleolar protein 14-like [Acropora digitifera].	XP_015758520	50.9	1.90E-219	K14766	10	C:GO:003204	C:small-subun	n/a		IPR007276	(IC:GO:003204	C:small-subunit processome
adi2mcaRNA12454_R0	n/a	n/a	n/a	n/a	n/a	n/a	2.965093073	0.030050064	fez family zinc finger protein 1-like [Orbicella faveolata]	XP_020623834	172	8.00E-154	n/a	n/a	n/a	n/a	n/a	n/a	n/a	n/a	n/a
adi2mcaRNA12676_R1	n/a	n/a	n/a	n/a	n/a	n/a	1.770681068	0.038137588	PREDICTED: acyl-CoA synthetase family member 2, mitochondrial-like [Acropora digitifera].	XP_015758793	81	3.90E-126	K00666	2	F:GO:000382	F:catalytic act	n/a		G3DSA:3.40.	F:GO:000382	F:catalytic activity; P:metabolic process
adi2mcaRNA12974_R0	n/a	n/a	n/a	n/a	n/a	n/a	1.075553463	0.037093852	PREDICTED: cyclin-G-associated kinase-like [Acropora digitifera].	XP_015758990	71.9	0.00E+00	K08855	11	F:GO:000552	F:ATP bindin	n/a		IPR000719	(IF:GO:000552	F:ATP binding; F:protein kinase activity; P:p[illegible]
adi2mcaRNA13008_R0	n/a	n/a	1.392997054	0.001077357	n/a	n/a	n/a	n/a	PREDICTED: uncharacterized protein LOC107338364 [Acropora digitifera].	XP_015759084	90.4	7.90E-300	n/a	n/a	n/a	n/a	n/a	n/a	n/a	n/a	n/a
[illegible]	[illegible]	[illegible]	[illegible]	[illegible]	[illegible]	[illegible]	[illegible]	[illegible]	[illegible]	[illegible]	[illegible]	[illegible]	[illegible]	[illegible]	[illegible]	[illegible]	[illegible]	[illegible]	[illegible]	[illegible]	[illegible]

augustus.g1172.t1	n/a	n/a	n/a	n/a	n/a	n/a	-2.162682083	0.03866554	pol-like protein [Ciona intestinalis].	BAC82626	22.9	5.00E-12	n/a	n/a	n/a	n/a	n/a	n/a	n/a	n/a	n/a
augustus.g11739.t1	2.910825945	0.036094348	4.787534371	5.17E-08	n/a	n/a	2.213211685	0.025858241	PREDICTED: early growth response protein 1-like isoform X2 [Acropora digitifera].	XP_015753321	85.4	4.90E-166	K09203	n/a	n/a	n/a	n/a	n/a	n/a	n/a	n/a
augustus.g11876.t1	n/a	n/a	n/a	n/a	n/a	n/a	-1.743621188	0.011450819	PREDICTED: carbohydrate sulfotransferase 1-like [Acropora digitifera].	XP_015751175	70.7	2.90E-166	K01022	n/a	n/a	n/a	n/a	n/a	n/a	n/a	n/a
augustus.g11919.t1	n/a	n/a	n/a	n/a	n/a	n/a	3.969922452	0.01110059	catalase-like isoform X2 [Orbicella faveolata].	XP_020604902	80.7	5.40E-126	K03781	n/a	n/a	n/a	n/a	n/a	n/a	n/a	n/a
augustus.g11940.t1	n/a	n/a	-5.758674267	0.036154113	n/a	n/a	n/a	n/a	PREDICTED: olfactomedin-like protein 2B [Acropora digitifera].	XP_015765467	62.7	4.00E-112	n/a	n/a	n/a	n/a	n/a	n/a	n/a	n/a	n/a
augustus.g11956.t1	n/a	n/a	n/a	n/a	n/a	n/a	1.824107629	0.000165254	PREDICTED: uncharacterized protein LOC107346841 [Acropora digitifera].	XP_015768172	64.3	4.00E-94	n/a	1	F:GO:000550	F:calcium ion	n/a	n/a	IPR002048 (S	F:GO:000551	F:protein binding; F:calcium ion binding
augustus.g11957.t1	n/a	n/a	1.981007809	0.044050328	n/a	n/a	n/a	n/a	PREDICTED: uncharacterized protein LOC107346863 [Acropora digitifera].	XP_015768204	78.1	1.90E-179	n/a	n/a	n/a	n/a	n/a	n/a	n/a	n/a	n/a
augustus.g12042.t1	n/a	n/a	n/a	n/a	n/a	n/a	2.007363291	5.21E-05	n/a	n/a	n/a	n/a	n/a	n/a	n/a	n/a	n/a	n/a	n/a	n/a	n/a
augustus.g12057.t1	n/a	n/a	n/a	n/a	n/a	n/a	1.143449133	0.000729306	dnaJ homolog subfamily B member 14-like [Orbicella faveolata].	XP_020625391	69.9	8.80E-114	K09518	n/a	n/a	n/a	n/a	n/a	n/a	n/a	n/a
augustus.g12065.t1	n/a	n/a	n/a	n/a	n/a	n/a	-1.915843017	1.99E-07	RNA-binding protein with multiple splicing-like isoform X2 [Orbicella faveolata].	XP_020616085	65.3	1.40E-75	n/a	2	F:GO:000016	F:nucleotide b	n/a	n/a	IPR000504 (S	F:GO:000367	F:nucleic acid binding
augustus.g12073.t1	n/a	n/a	n/a	n/a	n/a	n/a	-1.249342269	0.023113642	dihydrofolate reductase-like [Orbicella faveolata].	XP_020608530	65.7	1.30E-65	K00287	1	P:GO:004471	P:single-organ	n/a	n/a	IPR012259 (I	F:GO:005064	F:NADP binding; P:nucleotide biosynthetic p
augustus.g12348.t1	n/a	n/a	-6.800689258	0.028549062	n/a	n/a	n/a	n/a	cysteine synthase 1-like [Orbicella faveolata].	XP_020626202	85.6	5.60E-188	K01738	3	F:GO:003017	F:pyridoxal p	n/a	n/a	IPR001926 (I	no GO terms	no GO terms
augustus.g12352.t1	n/a	n/a	n/a	n/a	n/a	n/a	1.846876858	0.014743423	uncharacterized protein LOC110064862 isoform X2 [Orbicella faveolata].	XP_020627624	61.1	7.00E-244	n/a	1	P:GO:000716	P:signal transd	n/a	n/a	IPR001478 (S	F:GO:000551	F:protein binding; P:signal transduction
augustus.g12399.t1	n/a	n/a	n/a	n/a	n/a	n/a	1.005167493	0.009084237	PREDICTED: AF4/FMR2 family member 4-like [Acropora digitifera].	XP_015771642	64.3	1.30E-150	K15195	n/a	n/a	n/a	n/a	n/a	n/a	n/a	n/a
augustus.g1244.t1	-5.901487462	0.035978018	n/a	n/a	n/a	n/a	n/a	n/a	PREDICTED: uncharacterized protein LOC107339408 isoform X1 [Acropora digitifera].	XP_015760172	72.1	7.70E-258	n/a	2	F:GO:000484	F:ubiquitin-pro	n/a	n/a	IPR000569 (S	F:GO:000484	F:ubiquitin-protein transferase activity
augustus.g12474.t1	n/a	n/a	n/a	n/a	n/a	n/a	1.31763158	0.010616731	PREDICTED: orphan steroid hormone receptor 2-like [Acropora digitifera].	XP_015763888	81.1	3.40E-180	n/a	n/a	n/a	n/a	n/a	n/a	n/a	n/a	n/a
augustus.g12667.t1	n/a	n/a	n/a	n/a	-6.076720419	8.81E-05	n/a	n/a	uncharacterized protein LOC110043570 [Orbicella faveolata].	XP_020604698	87.5	1.30E-133	n/a	n/a	n/a	n/a	n/a	n/a	n/a	n/a	n/a
augustus.g12804.t1	-21.48080164	0.002325054	n/a	n/a	n/a	n/a	n/a	n/a	uncharacterized protein LOC110062459 [Orbicella faveolata].	XP_020625043	63.6	1.10E-80	n/a	n/a	n/a	n/a	n/a	n/a	n/a	n/a	n/a
augustus.g12900.t1	n/a	n/a	-2.398091813	0.03531431	n/a	n/a	n/a	n/a	LOW QUALITY PROTEIN: uncharacterized protein LOC110044084 [Orbicella faveolata].	XP_020605262	54.7	1.00E-161	n/a	n/a	n/a	n/a	n/a	n/a	n/a	n/a	n/a
augustus.g12922.t1	-23.49284398	5.59E-09	n/a	n/a	n/a	n/a	n/a	n/a	PREDICTED: noelin-like isoform X1 [Acropora digitifera].	XP_015756162	75	1.70E-140	n/a	n/a	n/a	n/a	n/a	n/a	n/a	n/a	n/a
augustus.g12998.t1	n/a	n/a	n/a	n/a	n/a	n/a	-1.426291595	0.014106238	transcription factor E2F7-like [Orbicella faveolata].	XP_020628356	67.7	1.20E-203	K09391	n/a	n/a	n/a	n/a	n/a	n/a	n/a	n/a
augustus.g13046.t1	n/a	n/a	n/a	n/a	n/a	n/a	-2.356223465	0.021773065	2-epi-5-epi-valiolone synthase [Exaiptasia pallida].	KXJ07711	66.8	0.00E+00	n/a	2	F:GO:000817	F:O-methyltra	n/a	n/a	G3DSA:1.20.	F:GO:000817	F:O-methyltransferase activity
augustus.g13081.t1	n/a	n/a	n/a	n/a	n/a	n/a	3.14808662	2.85E-26	LOW QUALITY PROTEIN: uncharacterized protein LOC110062558 [Orbicella faveolata].	XP_020625147	81.5	2.90E-179	n/a	3	P:GO:005508	P:transmembr	n/a	n/a	G3DSA:1.20.	P:GO:005508	P:transmembrane transport; C:integral compo
augustus.g13085.t1	n/a	n/a	n/a	n/a	n/a	n/a	-1.263573556	0.020515132	hypothetical protein AC249_AIPGENE608 [Exaiptasia pallida].	KXJ07972	45.3	1.30E-89	n/a	n/a	n/a	n/a	n/a	n/a	n/a	n/a	n/a
augustus.g13101.t1	n/a	n/a	n/a	n/a	n/a	n/a	1.181745152	6.40E-06	upstream stimulatory factor 1-like isoform X1 [Orbicella faveolata].	XP_020617357	90.9	5.50E-183	K09106	n/a	n/a	n/a	n/a	n/a	n/a	n/a	n/a
augustus.g13104.t1	n/a	n/a	n/a	n/a	n/a	n/a	1.822536512	6.90E-08	PREDICTED: protein maelstrom 2-like [Acropora digitifera].	XP_015764549	77.2	1.00E-209	K18411	6	P:GO:001062	P:negative reg	n/a	n/a	IPR009071 (P	GO:00609	P:regulation of gene silencing by miRNA
augustus.g13166.t1	n/a	n/a	n/a	n/a	3.38466441	0.010126503	n/a	n/a	PREDICTED: phosphatidylinositol phosphatase PTPRQ-like isoform X5 [Acropora digitifera].	XP_015777612	56.2	0.00E+00	K16353	n/a	n/a	n/a	n/a	n/a	n/a	n/a	n/a
augustus.g1319.t1	n/a	n/a	n/a	n/a	n/a	n/a	-1.639818903	0.008353578	PREDICTED: acylphosphatase-1-like [Acropora digitifera].	XP_015749362	80.4	4.70E-38	K01512	1	F:GO:000399	F:acylphosph	EC:3.6.1.7	Acylphosphat	IPR020456 (I	F:GO:000399	F:acylphosphatase activity
augustus.g1328.t1	n/a	n/a	n/a	n/a	n/a	n/a	-3.32545133	0.001522521	PREDICTED: uncharacterized protein LOC107333256 [Acropora digitifera].	XP_015753522	61.9	0.00E+00	n/a	2	C:GO:000585	C:cytoskeleton	n/a	n/a	IPR013783 (I	no GO terms	no GO terms
augustus.g1329.t1	n/a	n/a	n/a	n/a	n/a	n/a	-3.780518329	0.002380829	n/a	n/a	n/a	n/a	n/a	n/a	n/a	n/a	n/a	n/a	n/a	n/a	n/a
augustus.g13472.t1	n/a	n/a	n/a	n/a	n/a	n/a	1.600428615	1.71E-09	sushi, von Willebrand factor type A, EGF and pentraxin domain-containing protein 1-like isoform X1 [Or	XP_020628105.1	50	6.00E-105	n/a	n/a	n/a	n/a	n/a	n/a	n/a	n/a	n/a
augustus.g13699.t1	n/a	n/a	n/a	n/a	n/a	n/a	3.186611328	5.34E-06	n/a	n/a	n/a	n/a	n/a	n/a	n/a	n/a	n/a	n/a	n/a	n/a	n/a
augustus.g13703.t1	n/a	n/a	n/a	n/a	n/a	n/a	2.655588677	0.000961679	n/a	n/a	n/a	n/a	n/a	n/a	n/a	n/a	n/a	n/a	n/a	n/a	n/a
augustus.g13845.t1	n/a	n/a	n/a	n/a	n/a	n/a	1.02770738	0.034870109	interferon-related developmental regulator 1-like [Orbicella faveolata].	XP_020629462	73.2	1.70E-164	n/a	1	C:GO:000563	C:nucleus	n/a	n/a	IPR006921 (I	F:GO:000548	F:binding
augustus.g1386.t1	n/a	n/a	n/a	n/a	n/a	n/a	1.016358583	0.011094235	PREDICTED: putative diacylglycerol O-acyltransferase MT1809 isoform X2 [Acropora digitifera].	XP_015765008	58.9	3.20E-167	n/a	n/a	n/a	n/a	n/a	n/a	n/a	n/a	n/a
augustus.g13905.t1	n/a	n/a	n/a	n/a	6.688941993	0.048276311	6.961125875	0.003033229	PREDICTED: uncharacterized protein LOC107335421 [Acropora digitifera].	XP_015755935	49.1	1.50E-36	n/a	n/a	n/a	n/a	n/a	n/a	n/a	n/a	n/a
augustus.g1394.t1	n/a	n/a	n/a	n/a	n/a	n/a	2.008900283	4.64E-05	PREDICTED: uncharacterized protein LOC107343912 [Acropora digitifera].	XP_015764990	84	2.10E-256	n/a	n/a	n/a	n/a	n/a	n/a	n/a	n/a	n/a
augustus.g13995.t1	n/a	n/a	n/a	n/a	n/a	n/a	1.11917423	0.00031773	PREDICTED: phospholipase A-2-activating protein-like [Acropora digitifera].	XP_015765131	75.2	1.70E-274	K14018	4	P:GO:006500	P:biological re	n/a	n/a	IPR001680 (S	F:GO:000551	F:protein binding; F:binding
augustus.g13996.t1	-4.089347558	0.000253163	n/a	n/a	n/a	n/a	n/a	n/a	PREDICTED: E3 ubiquitin-protein ligase TRIM71-like [Acropora digitifera].	XP_015759158	59.9	8.90E-153	K12035	n/a	n/a	n/a	n/a	n/a	n/a	n/a	n/a
augustus.g14000.t1	n/a	n/a	n/a	n/a	n/a	n/a	-3.739958637	7.45E-05	A disintegrin and metalloproteinase with thrombospondin motifs 6-like [Orbicella faveolata].	XP_020626465	63.3	6.10E-155	K08621	18	F:GO:000827	F:zinc ion bin	n/a	n/a	IPR013273 (I	F:GO:000827	F:zinc ion binding; F:metalloendopeptidase a
augustus.g1409.t1	n/a	n/a	n/a	n/a	n/a	n/a	1.977855722	0.027393412	PREDICTED: uncharacterized protein LOC107356209 [Acropora digitifera].	XP_015778332	53.2	3.90E-33	n/a	n/a	n/a	n/a	n/a	n/a	n/a	n/a	n/a
augustus.g14107.t1	n/a	n/a	2.238247452	1.10E-08	n/a	n/a	1.037041546	0.025076355	PREDICTED: calcium-binding protein CML19-like [Acropora digitifera].	XP_015755487	90.6	3.40E-78	n/a	n/a	n/a	n/a	n/a	n/a	n/a	n/a	n/a
augustus.g14113.t1	2.744663803	0.036161583	n/a	n/a	n/a	n/a	n/a	n/a	PREDICTED: probable calcium-binding protein CML15 [Acropora digitifera].	XP_015775478	75.9	2.70E-70	n/a	n/a	n/a	n/a	n/a	n/a	n/a	n/a	n/a
augustus.g14164.t1	n/a	n/a	n/a	n/a	n/a	n/a	-1.270642327	0.00044904	metalloendopeptidase OMA1, mitochondrial-like [Orbicella faveolata].	XP_020609988	71.1	1.10E-133	n/a	5	P:GO:004423	P:primary me	n/a	n/a	IPR001915 (I	P:GO:000650	P:proteolysis; F:metalloendopeptidase activity
augustus.g14186.t1	n/a	n/a	n/a	n/a	n/a	n/a	-1.95277448	0.025016369	PREDICTED: allene oxide synthase-lipoxygenase protein-like [Acropora digitifera].	XP_015771173	72.7	0.00E+00	K00461	2	F:GO:001649	F:oxidoreduct	n/a	n/a	IPR001885 (I	F:GO:001670	F:oxidoreductase activity, acting on single do
augustus.g1419.t1	n/a	n/a	n/a	n/a	n/a	n/a	-4.355878971	0.035183784	n/a	n/a	n/a	n/a	n/a	n/a	n/a	n/a	n/a	n/a	n/a	n/a	n/a
augustus.g14262.t1	n/a	n/a	n/a	n/a	n/a	n/a	1.992656435	1.44E-06	uncharacterized protein LOC110066365 isoform X2 [Orbicella faveolata].	XP_020629254	26.8	3.80E-17	n/a	n/a	n/a	n/a	n/a	n/a	n/a	n/a	n/a
augustus.g14289.t1	-3.958980499	0.03183138	n/a	n/a	n/a	n/a	n/a	n/a	E3 ubiquitin-protein ligase TRIM71-like [Orbicella faveolata].	XP_020625543	50.3	2.40E-44	K12035	3	F:GO:000827	F:zinc ion bin	n/a	n/a	IPR001841 (S	F:GO:000827	F:zinc ion binding; C:intracellular
augustus.g14392.t1	n/a	n/a	n/a	n/a	n/a	n/a	1.926369287	0.00135661	PREDICTED: probable cysteine desulfurase [Acropora digitifera].	XP_015752250	83	0.00E+00	n/a	n/a	n/a	n/a	n/a	n/a	n/a	n/a	n/a
augustus.g14554.t1	n/a	n/a	n/a	n/a	n/a	n/a	2.72993954	0.005895074	PREDICTED: PHD finger protein 20-like protein 1 [Acropora digitifera].	XP_015757337	83.3	1.00E-50	K18402	n/a	n/a	n/a	n/a	n/a	n/a	n/a	n/a
augustus.g14630.t1	n/a	n/a	n/a	n/a	n/a	n/a	-21.59850569	5.78E-05	tumor necrosis factor ligand superfamily member 10-like [Orbicella faveolata].	XP_020627738	58	1.30E-45	n/a	4	P:GO:000695	P:immune resp	n/a	n/a	n/a	n/a	n/a
augustus.g14635.t1	n/a	n/a	n/a	n/a	n/a	n/a	-2.582362811	0.022277808	galectin [Pocillopora damicornis].	AMZ03553	62.9	2.70E-84	n/a	1	F:GO:003024	F:carbohydrat	n/a	n/a	IPR000922 (I	F:GO:003024	F:carbohydrate binding
augustus.g14644.t1	n/a	n/a	3.223656309	1.07E-09	n/a	n/a	n/a	n/a	hypothetical protein AC249_AIPGENE20382 [Exaiptasia pallida].	KXJ18229	54.2	4.70E-146	n/a	n/a	n/a	n/a	n/a	n/a	n/a	n/a	n/a
augustus.g14646.t1	3.507730331	0.036509811	n/a	n/a	n/a	n/a	n/a	n/a	PREDICTED: uncharacterized protein LOC107351155 [Acropora digitifera].	XP_015772918	40.7	4.10E-07	n/a	n/a	n/a	n/a	n/a	n/a	n/a	n/a	n/a

augustus.g29921.t1	n/a	n/a	n/a	n/a	n/a	n/a	2.029306629	0.004668612	uncharacterized protein LOC110057701 [Orbicella faveolata].	XP_020619948	68.5	3.40E-253	n/a	n/a	n/a	n/a	n/a	n/a	n/a	n/a	n/a
augustus.g29927.t1	n/a	n/a	n/a	n/a	n/a	n/a	-1.598649285	0.007048566	tryptophan 2,3-dioxygenase-like [Orbicella faveolata].	XP_020619942	79.1	4.00E-187	K00453	n/a	n/a	n/a	n/a	n/a	n/a	n/a	n/a
augustus.g2997.t1	n/a	n/a	n/a	n/a	n/a	n/a	1.764084493	0.004683327	predicted protein [Nematostella vectensis].	XP_001641535	70.8	2.80E-202	K08955	6	F:GO:000552	F:ATP bindin	EC:3.4.24; EI	Acting on pe	n/a	n/a	n/a
augustus.g2998.t1	n/a	n/a	n/a	n/a	n/a	n/a	-1.025467851	0.014171931	PREDICTED: solute carrier family 12 member 2-like [Acropora digitifera].	XP_015748716	77.5	0.00E+00	K10951	5	P:GO:001569	P:ammonium	n/a	n/a	n/a	n/a	n/a
augustus.g30027.t1	n/a	n/a	1.061162091	0.016234546	n/a	n/a	n/a	n/a	uncharacterized protein LOC110050460 [Orbicella faveolata].	XP_020612043	77.8	0.00E+00	K02295	2	F:GO:005108	F:unfolded pr	n/a	n/a	n/a	n/a	n/a
augustus.g30033.t1	n/a	n/a	n/a	n/a	n/a	n/a	-1.009314455	0.020214421	PREDICTED: pathogen-related protein-like [Acropora digitifera].	XP_015767419	80.2	6.00E-96	n/a	n/a	n/a	n/a	n/a	n/a	n/a	n/a	n/a
augustus.g30050.t1	n/a	n/a	-1.000110387	0.009422432	n/a	n/a	1.284378646	2.06E-05	uncharacterized protein LOC110058304 [Orbicella faveolata].	XP_020620594	60.8	3.80E-118	n/a	n/a	n/a	n/a	n/a	n/a	n/a	n/a	n/a
augustus.g3006.t1	n/a	n/a	n/a	n/a	n/a	n/a	1.703098427	0.02711767	deleted in lung and esophageal cancer protein 1-like [Orbicella faveolata].	XP_020611459	64.5	0.00E+00	n/a	2	C:GO:000573	C:cytoplasm;	n/a	n/a	IPR013783 (G	P:GO:000828	P:negative regulation of cell proliferation
augustus.g30140.t1	n/a	n/a	n/a	n/a	n/a	n/a	2.730762605	4.26E-06	uncharacterized protein LOC110058996 [Orbicella faveolata].	XP_020621318	52.4	1.20E-33	K16339	n/a	n/a	n/a	n/a	n/a	n/a	n/a	n/a
augustus.g30144.t1	n/a	n/a	2.102581553	0.005234029	n/a	n/a	2.221180002	0.000761664	n/a	n/a	n/a	n/a	n/a	n/a	n/a	n/a	n/a	n/a	n/a	n/a	n/a
augustus.g30147.t1	n/a	n/a	n/a	n/a	n/a	n/a	2.322740822	2.77E-22	PREDICTED: cyclic AMP-responsive element-binding protein 3-like protein 3-B isoform X1 [Acropora d	XP_015751395	69.2	3.80E-123	K09048	6	F:GO:000370	F:transcription	n/a	n/a	IPR004827 (IF	F:GO:000370	F:transcription factor activity, sequence-specific DNA binding; F:cAMP response element bi
augustus.g30184.t1	n/a	n/a	n/a	n/a	n/a	n/a	-2.006714799	2.34E-05	PREDICTED: uncharacterized protein LOC107349253 [Acropora digitifera].	XP_015770847	75	3.30E-71	n/a	n/a	n/a	n/a	n/a	n/a	n/a	n/a	n/a
augustus.g30192.t1	n/a	n/a	n/a	n/a	n/a	n/a	1.180596282	2.00E-06	secretory carrier-associated membrane protein 2-like isoform X2 [Orbicella faveolata].	XP_020631877	72.7	1.30E-113	K19995	3	C:GO:001602	C:membrane;	n/a	n/a	IPR007273 (IP	P:GO:001503	P:protein transport; C:integral component of membrane
augustus.g30204.t1	n/a	n/a	n/a	n/a	n/a	n/a	2.258248731	0.031709887	PREDICTED: aristaless-related homeobox protein-like isoform X1 [Acropora digitifera].	XP_015767208	86.5	1.60E-118	n/a	n/a	n/a	n/a	n/a	n/a	n/a	n/a	n/a
augustus.g30301.t1	n/a	n/a	-1.125452907	0.041303941	n/a	n/a	n/a	n/a	EKC/KEOPS complex subunit Lage3-like [Orbicella faveolata].	XP_020626699	74.2	1.10E-25	K15902	9	C:GO:000578	C:endoplasmi	n/a	n/a	n/a	n/a	n/a
augustus.g3047.t1	n/a	n/a	2.230737768	0.00090918	n/a	n/a	n/a	n/a	PREDICTED: arf-GAP with coiled-coil, ANK repeat and PH domain-containing protein 2-like [Acropora	XP_015779878	84.7	1.30E-90	n/a	2	F:GO:001990	F:protein kina	n/a	n/a	n/a	n/a	n/a
augustus.g30474.t1	n/a	n/a	n/a	n/a	n/a	n/a	1.653637577	0.008433262	uncharacterized protein LOC110054837 [Orbicella faveolata].	XP_020616855	58.3	4.30E-25	n/a	n/a	n/a	n/a	n/a	n/a	n/a	n/a	n/a
augustus.g30477.t1	n/a	n/a	n/a	n/a	-3.592429813	8.21E-07	-4.229769951	9.34E-10	uncharacterized protein LOC110054836 [Orbicella faveolata].	XP_020616854	62	3.60E-34	n/a	n/a	n/a	n/a	n/a	n/a	n/a	n/a	n/a
augustus.g30478.t1	n/a	n/a	n/a	n/a	-1.979320945	0.003461913	-2.147303048	8.28E-05	uncharacterized protein LOC110054836 [Orbicella faveolata].	XP_020616854	62.2	1.20E-34	n/a	n/a	n/a	n/a	n/a	n/a	n/a	n/a	n/a
augustus.g30586.t1	n/a	n/a	-1.143812043	0.048217687	n/a	n/a	n/a	n/a	Protein farnesyltransferase/geranylgeranyltransferase type-1 subunit alpha [Exaiptasia pallida].	KXJ18390	71.8	3.60E-77	K05955	n/a	n/a	n/a	n/a	n/a	n/a	n/a	n/a
augustus.g30623.t1	n/a	n/a	-6.799837762	0.029923009	7.851838022	0.01545207	n/a	n/a	PREDICTED: phthiocerol synthesis polyketide synthase type I PpsC-like [Acropora digitifera].	XP_015754310	61.1	0.00E+00	n/a	n/a	n/a	n/a	n/a	n/a	n/a	n/a	n/a
augustus.g3072.t1	n/a	n/a	n/a	n/a	n/a	n/a	1.931605743	0.001081628	PREDICTED: multiple epidermal growth factor-like domains protein 8 [Acropora digitifera].	XP_015766427	74.4	5.20E-91	n/a	3	F:GO:000550	F:calcium ion	n/a	n/a	n/a	n/a	n/a
augustus.g30752.t1	n/a	n/a	1.788367455	0.032544157	n/a	n/a	n/a	n/a	potassium channel subfamily K member 15-like [Orbicella faveolata].	XP_020603093	52.2	2.90E-73	n/a	4	C:GO:001602	C:membrane;	n/a	n/a	n/a	n/a	n/a
augustus.g30911.t1	n/a	n/a	n/a	n/a	7.955967147	0.023584578	6.77966613	0.014106238	PREDICTED: uncharacterized protein LOC107349372 isoform X1 [Acropora digitifera].	XP_015771004	85.1	3.70E-265	n/a	n/a	n/a	n/a	n/a	n/a	n/a	n/a	n/a
augustus.g3095.t1	n/a	n/a	n/a	n/a	n/a	n/a	1.578634092	3.97E-05	n/a	n/a	n/a	n/a	n/a	n/a	n/a	n/a	n/a	n/a	n/a	n/a	n/a
augustus.g3109.t1	n/a	n/a	n/a	n/a	n/a	n/a	-1.527700499	0.022235745	PREDICTED: TWiK family of potassium channels protein 18-like [Acropora digitifera].	XP_015753403	86	1.20E-185	K05323	3	P:GO:006500	P:biological	n/a	n/a	n/a	n/a	n/a
augustus.g31177.t1	n/a	n/a	n/a	n/a	n/a	n/a	-1.369219151	0.044525051	PREDICTED: mitochondrial genome maintenance protein MGM101-like [Acropora digitifera].	XP_015779945	81.7	2.30E-110	n/a	6	F:GO:000367	F:DNA bindin	n/a	n/a	n/a	n/a	n/a
augustus.g31183.t1	n/a	n/a	n/a	n/a	n/a	n/a	1.003399454	0.002392005	NEDD4-like E3 ubiquitin-protein ligase WWP1 [Exaiptasia pallida].	KXJ10517	79	0.00E+00	K05633	1	F:GO:000382	F:catalytic act	n/a	n/a	n/a	n/a	n/a
augustus.g31186.t1	n/a	n/a	n/a	n/a	n/a	n/a	1.510319213	0.001084419	PREDICTED: metal tolerance protein 11-like [Acropora digitifera].	XP_015779992	73.9	4.20E-163	n/a	1	P:GO:000681	P:transport	n/a	n/a	n/a	n/a	n/a
augustus.g31223.t1	n/a	n/a	2.346214494	0.001263027	n/a	n/a	2.586625871	3.84E-05	phosphoenolpyruvate carboxykinase, cytosolic [GTP]-like [Orbicella faveolata].	XP_020610165	84.7	0.00E+00	K01596	5	F:GO:000552	F:GTP bindin	EC:4.1.1.32	Phosphoenol	IPR035077 (IF	F:GO:000552	F:GTP binding; F:phosphoenolpyruvate carboxykinase activity; P:gluconeogenesis; F:purine
augustus.g31346.t1	n/a	n/a	n/a	n/a	n/a	n/a	-1.388985564	0.005691306	PREDICTED: probable G-protein coupled receptor 157 [Acropora digitifera].	XP_015756891	68.6	7.10E-102	n/a	7	F:GO:000493	F:G-protein co	n/a	n/a	n/a	n/a	n/a
augustus.g31425.t1	n/a	n/a	n/a	n/a	n/a	n/a	-1.43336412	0.048501244	PREDICTED: uncharacterized protein LOC107339358 [Acropora digitifera].	XP_015760116	55.7	9.40E-51	n/a	n/a	n/a	n/a	n/a	n/a	n/a	n/a	n/a
augustus.g31430.t1	n/a	n/a	n/a	n/a	27.01938609	3.62E-13	25.9054557	9.84E-13	hypothetical protein CAPTEDRAFT_81373, partial [Capitella teleta].	ELU08736	38.1	1.10E-13	n/a	n/a	n/a	n/a	n/a	n/a	n/a	n/a	n/a
augustus.g31440.t1	n/a	n/a	n/a	n/a	n/a	n/a	2.005181637	1.75E-15	PREDICTED: next to BRCA1 gene 1 protein-like isoform X1 [Acropora digitifera].	XP_015769400	62.2	2.20E-297	K17987	1	C:GO:000444	C:cytoplasmic	n/a	n/a	n/a	n/a	n/a
augustus.g31744.t1	n/a	n/a	n/a	n/a	n/a	n/a	1.249266015	0.029144107	PREDICTED: DIS3-like exonuclease 2 [Acropora digitifera].	XP_015752721	74.4	0.00E+00	K18758	11	C:GO:004322	C:intracellular	n/a	n/a	n/a	n/a	n/a
augustus.g31747.t1	n/a	n/a	n/a	n/a	n/a	n/a	1.030982274	0.005564556	PREDICTED: numb-like protein isoform X2 [Acropora digitifera].	XP_015761300	68.4	9.10E-179	n/a	10	P:GO:000740	P:neuroblast p	n/a	n/a	n/a	n/a	n/a
augustus.g31777.t1	n/a	n/a	n/a	n/a	n/a	n/a	-1.641643337	0.030050064	PREDICTED: egl nine homolog 3-like [Acropora digitifera].	XP_015762813	75.5	4.20E-164	K09592	2	F:GO:001649	F:oxidoreduct	n/a	n/a	n/a	n/a	n/a
augustus.g31809.t1	n/a	n/a	2.620111258	0.006423154	n/a	n/a	n/a	n/a	PREDICTED: uncharacterized protein LOC107341866 [Acropora digitifera].	XP_015762828	30.7	2.50E-09	n/a	n/a	n/a	n/a	n/a	n/a	n/a	n/a	n/a
augustus.g31818.t1	n/a	n/a	n/a	n/a	n/a	n/a	-6.63176959	0.000827446	uncharacterized protein LOC110064896 [Orbicella faveolata].	XP_020627660	32.1	2.40E-07	n/a	n/a	n/a	n/a	n/a	n/a	n/a	n/a	n/a
augustus.g31829.t1	n/a	n/a	n/a	n/a	n/a	n/a	3.934622158	1.82E-14	PREDICTED: tumor necrosis factor receptor superfamily member 16-like [Acropora digitifera].	XP_015763742	35.6	2.80E-67	n/a	n/a	n/a	n/a	n/a	n/a	n/a	n/a	n/a
augustus.g31830.t1	n/a	n/a	n/a	n/a	n/a	n/a	4.676312788	6.61E-07	PREDICTED: tumor necrosis factor receptor superfamily member 16-like [Acropora digitifera].	XP_015763742	31.7	5.20E-59	n/a	n/a	n/a	n/a	n/a	n/a	n/a	n/a	n/a
augustus.g31831.t1	n/a	n/a	n/a	n/a	n/a	n/a	4.658339952	0.00512636	PREDICTED: tumor necrosis factor receptor superfamily member 16-like [Acropora digitifera].	XP_015763743	39.8	5.40E-45	n/a	n/a	n/a	n/a	n/a	n/a	n/a	n/a	n/a
augustus.g31852.t1	n/a	n/a	n/a	n/a	n/a	n/a	1.36942189	0.00037309	integrin alpha-V-like [Orbicella faveolata].	XP_020611470	54.1	8.50E-276	K06487	3	P:GO:004885	P:anatomical	n/a	n/a	n/a	n/a	n/a
augustus.g31882.t1	n/a	n/a	n/a	n/a	n/a	n/a	2.87234316	0.015408352	parathyroid hormone/parathyroid hormone-related peptide receptor-like isoform X2 [Orbicella faveolata].	XP_020611402	41.9	3.80E-136	K04579	n/a	n/a	n/a	n/a	n/a	n/a	n/a	n/a
augustus.g31923.t1	n/a	n/a	n/a	n/a	n/a	n/a	-1.066441195	0.007997823	phosphatidylinositol N-acetylglucosaminyltransferase subunit A-like [Orbicella faveolata].	XP_020610937	81.2	4.40E-208	K03857	3	P:GO:000650	P:GPI anchor	n/a	n/a	n/a	n/a	n/a
augustus.g31936.t1	n/a	n/a	n/a	n/a	n/a	n/a	2.253959588	1.57E-05	n/a	n/a	n/a	n/a	n/a	n/a	n/a	n/a	n/a	n/a	n/a	n/a	n/a
augustus.g31964.t1	10.31526512	0.040388838	n/a	n/a	n/a	n/a	n/a	n/a	PREDICTED: GFP-like fluorescent chromoprotein FP506 [Acropora digitifera].	XP_015757116	74.8	8.50E-99	n/a	n/a	n/a	n/a	n/a	n/a	n/a	n/a	n/a
augustus.g32012.t1	n/a	n/a	n/a	n/a	n/a	n/a	-1.187851122	0.003411461	PREDICTED: probable serine/threonine-protein kinase irlF [Acropora digitifera]	XP_015774498.1	54	7.00E-75	n/a	n/a	n/a	n/a	n/a	n/a	n/a	n/a	n/a
augustus.g32052.t1	n/a	n/a	n/a	n/a	n/a	n/a	-1.681992598	4.20E-06	PREDICTED: valacyclovir hydrolase-like [Acropora digitifera].	XP_015768695	80.1	1.50E-129	K18399	3	P:GO:000652	P:cellular ami	n/a	n/a	n/a	n/a	n/a
augustus.g32111.t1	n/a	n/a	1.759612089	0.043853792	n/a	n/a	n/a	n/a	PREDICTED: protein phosphatase 1 regulatory subunit 27-like [Acropora digitifera].	XP_015753693	70.5	1.90E-51	K17566	n/a	n/a	n/a	n/a	n/a	n/a	n/a	n/a
augustus.g32116.t1	n/a	n/a	n/a	n/a	1.460304411	2.72E-12	1.503867271	9.11E-14	PREDICTED: transcription factor VBP-like [Acropora digitifera].	XP_015768917	81.6	6.00E-79	n/a	n/a	n/a	n/a	n/a	n/a	n/a	n/a	n/a
augustus.g32119.t1	n/a	n/a	n/a	n/a	n/a	n/a	1.834345373	1.35E-05	PREDICTED: SEC14 cytosolic factor-like isoform X1 [Acropora digitifera].	XP_015768905	86.1	2.40E-210	n/a	1	C:GO:004446	C:cell part	n/a	n/a	n/a	n/a	n/a
augustus.g3212.t1	n/a	n/a	n/a	n/a	n/a	n/a	1.407863783	0.008978608	PREDICTED: uncharacterized protein LOC107356778 [Acropora digitifera].	XP_015778890	48.3	2.10E-20	n/a	n/a	n/a	n/a	n/a	n/a	n/a	n/a	n/a
augustus.g32139.t1	n/a	n/a	-1.538473298	0.000131159	n/a	n/a	n/a	n/a	PREDICTED: uncharacterized protein LOC107347456 [Acropora digitifera].	XP_015768869	72.4	1.10E-143	n/a	n/a	n/a	n/a	n/a	n/a	n/a	n/a	n/a
augustus.g3215.t1	-9.077003957	0.041529385	n/a	n/a	n/a	n/a	n/a	n/a	n/a	n/a	n/a	n/a	n/a	n/a	n/a	n/a	n/a	n/a	n/a	n/a	n/a
augustus.g32152.t1	n/a	n/a	n/a	n/a	n/a	n/a	2.614035916	1.03E-12	cathepsin L1-like [Orbicella faveolata].	XP_020632264	76.7	2.00E-134	K01365	4	C:GO:000561	C:extracellula	EC:3.4.22	Acting on pe	IPR000668 (IF	P:GO:000650	P:proteolysis; F:cysteine-type peptidase activity
augustus.g32202.t1	n/a	n/a	1.915523369	0.000606793	n/a	n/a	1.451346133	0.010123611	PREDICTED: cysteine/serine-rich nuclear protein 1-like [Acropora digitifera].	XP_015761354	79.8	8.60E-87	K17494	n/a	n/a	n/a	n/a	n/a	n/a	n/a	n/a
augustus.g32273.t1	n/a	n/a	n/a	n/a	n/a	n/a	4.737500856	0.000745718	PREDICTED: uncharacterized protein LOC107338323 [Acropora digitifera].	XP_015759045	33	4.30E-72	n/a	n/a	n/a	n/a	n/a	n/a	n/a	n/a	n/a
augustus.g32300.t1	n/a	n/a	3.081150434	6.06E-09	n/a	n/a	1.896039676	0.000907811	PREDICTED: protein BTG2-like [Acropora digitifera].	XP_015759486	79.8	1.10E-80	K14443	n/a	n/a	n/a	n/a	n/a	n/a	n/a	n/a
augustus.g32320.t1	n/a	n/a	1.575995873	0.029788377	n/a	n/a	n/a	n/a	PREDICTED: thiosulfate sulfurtransferase/rhodanese-like domain-containing protein 1 [Acropora digitifer	XP_015759652	70.9	2.80E-62	n/a	n/a	n/a	n/a	n/a	n/a	n/a	n/a	n/a
augustus.g32325.t1	n/a	n/a	n/a	n/a	n/a	n/a	-1.145371346	0.000203804	PREDICTED: neurocalcin homolog [Acropora digitifera].	XP_015759752	93.9	5.60E-91	n/a	2	F:GO:004687	F:metal ion bin	n/a	n/a	n/a	n/a	n/a
augustus.g32331.t1	n/a	n/a	n/a	n/a	n/a	n/a	1.238958332	0.031648213	PREDICTED: diphosphoinositol polyphosphate phosphohydrolase 1-like isoform X1 [Acropora digitifera	XP_015759764	93.9	5.00E-76	K07766	n/a	n/a	n/a	n/a	n/a	n/a	n/a	n/a
augustus.g32336.t1	n/a	n/a	n/a	n/a	n/a	n/a	1.038735839	0.004711637	A disintegrin and metalloproteinase with thrombospondin motifs 16-like [Orbicella faveolata].	XP_020608406	53.5	2.10E-303	n/a	n/a	n/a	n/a	n/a	n/a	n/a	n/a	n/a
augustus.g32393.t1	n/a	n/a	n/a	n/a	n/a	n/a	1.336371291	0.00926419	uncharacterized protein LOC110041724 [Orbicella faveolata].	XP_020602697	60.2	1.70E-119	n/a	3	F:GO:000550	F:calcium ion	n/a	n/a	n/a	n/a	n/a
augustus.g32415.t1	n/a	n/a	n/a	n/a	n/a	n/a	-4.817113882	0.000842399	PREDICTED: histamine N-methyltransferase-like [Acropora digitifera].	XP_015764997	66.4	1.60E-41	n/a	n/a	n/a	n/a	n/a	n/a	n/a	n/a	n/a
augustus.g32416.t1	n/a	n/a	n/a	n/a	n/a	n/a	-3.064660759	0.00182725	PREDICTED: histamine N-methyltransferase-like [Acropora digitifera].	XP_015764997	69.1	1.00E-104	K00546	n/a	n/a	n/a	n/a	n/a	n/a	n/a	n/a
augustus.g32436.t1	n/a	n/a	-1.135757371	0.012410861	n/a	n/a	n/a	n/a	transmembrane protein 198-like [Orbicella faveolata].	XP_020602030	58.2	7.60E-104	n/a	7	P:GO:000752	P:neuromusc	n/a	n/a	n/a	n/a	n/a
augustus.g32470.t1	n/a	n/a	n/a	n/a	n/a	n/a	2.633451896	6.62E-05	PREDICTED: stromal interaction molecule 1-like [Acropora digitifera].	XP_015778612	72.9	6.90E-218	K16059	11	P:GO:000211	P:store-operat	n/a	n/a	n/a	n/a	n/a
augustus.g32471.t1	n/a	n/a	1.472248375	0.043853792	n/a	n/a	1.504930915	0.010334126	protein transport protein sec31-like [Orbicella faveolata].	XP_020604574	55.4	1.40E-101	n/a	2	C:GO:004322	C:intracellular	n/a	n/a	n/a	n/a	n/a
augustus.g32488.t1	n/a	n/a	n/a	n/a	n/a	n/a	3.503629214	0.042210437	PREDICTED: uncharacterized protein LOC107331434 isoform X1 [Acropora digitifera].	XP_015751499	57.6	3.10E-51	n/a	n/a	n/a	n/a	n/a	n/a	n/a	n/a	n/a
augustus.g32544.t1	n/a	n/a	n/a	n/a	n/a	n/a	-1.271828993	0.037956852	PREDICTED: LOW QUALITY PROTEIN: stress response protein NST1-like [Acropora digitifera].	XP_015777076	63	4.50E-39	n/a	n/a	n/a	n/a	n/a	n/a	n/a	n/a	n/a
augustus.g32745.t1	n/a	n/a	-1.86798147	0.015691048	n/a	n/a	n/a	n/a	histamine H2 receptor-like [Orbicella faveolata].	XP_020606032	70.2	5.20E-118	n/a	n/a	n/a	n/a	n/a	n/a	n/a	n/a	n/a
augustus.g32767.t1	n/a	n/a	n/a	n/a	n/a	n/a	1.550631303	4.59E-09	PREDICTED: diacylglycerol kinase zeta-like [Acropora digitifera].	XP_015770938	74.3	0.00E+00	K00901	n/a	n/a	n/a	n/a	n/a	n/a	n/a	n/a
augustus.g32792.t1	n/a	n/a	1.254507685	0.019025281	n/a	n/a	n/a	n/a	PREDICTED: cyclic AMP-responsive element-binding protein 3-like protein 3-A [Acropora digitifera].	XP_015755420	75.8	6.30E-104	K09048	5	F:GO:000552	F:GTP bindin	EC:4.1.1.32	Phosphoenol	IPR035077 (IF	F:GO:000552	F:GTP binding; F:phosphoenolpyruvate carboxykinase activity; P:gluconeogenesis; F:purine
augustus.g32794.t1	n/a	n/a	n/a	n/a	n/a	n/a	-1.037928885	0.0018767	G1/S-specific cyclin-D2-like [Orbicella faveolata].	XP_020616594	88.3	3.10E-143	K10151	5	P:GO:000193	P:positive reg	n/a	n/a	n/a	n/a	n/a
augustus.g32819.t1	n/a	n/a	n/a	n/a	n/a	n/a	-6.620332923	0.017576093	piggyBac transposable element-derived protein 4-like isoform X1 [Orbicella faveolata].	XP_020619609	67.9	5.30E-216	n/a	n/a	n/a	n/a	n/a	n/a	n/a	n/a	n/a
augustus.g329.t1	n/a	n/a	n/a	n/a	n/a	n/a	2.496248479	0.045258809	n/a	n/a	n/a	n/a	n/a	n/a	n/a	n/a	n/a	n/a	n/a	n/a	n/a
augustus.g32944.t1	n/a	n/a	n/a	n/a	n/a	n/a	1.329973478	0.000842384	uncharacterized protein LOC110051556 [Orbicella faveolata].	XP_020613252	75.4	7.70E-183	K07282	n/a	n/a	n/a	n/a	n/a	n/a	n/a	n/a
augustus.g32962.t1	n/a	n/a	n/a	n/a	n/a	n/a	1.26236498	0.009930791	atrial natriuretic peptide receptor 1-like [Orbicella faveolata].	XP_020613286	60.6	0.00E+00	K12323	13	P:GO:000618	P:cGMP biosyn	n/a	n/a	n/a	n/a	n/a
augustus.g33008.t1	n/a	n/a	n/a	n/a	n/a	n/a	-4.24817764	0.010334126	PREDICTED: neuronal acetylcholine receptor subunit alpha-10-like [Acropora digitifera].	XP_015779537	74.2	1.20E-220	n/a	n/a	n/a	n/a	n/a	n/a	n/a	n/a	n/a
augustus.g33049.t1	n/a	n/a	n/a	n/a	n/a	n/a	1.994702857	0.004183143	hypothetical protein NEMVEDRAFT_v1g225049, partial [Nematostella vectensis].	XP_001618519	42.5	7.30E-12	n/a	n/a	n/a	n/a	n/a	n/a	n/a	n/a	n/a
augustus.g3314.t1	n/a	n/a	n/a	n/a	n/a	n/a	1.233817642	0.00609215	PREDICTED: 3-hydroxy-3-methylglutaryl-coenzyme A reductase-like [Acropora digitifera].	XP_015765211	78.6	0.00E+00	K00021	9	P:GO:190136	P:organic cyc	n/a	n/a	n/a	n/a	n/a
augustus.g33148.t1	1.428217745	0.039278482	2.257965935	7.49E-07	n/a	n/a	n/a	n/a	protein Wnt-1-like [Orbicella faveolata].	XP_020610577	63.7	7.00E-126	K00182	n/a	n/a	n/a	n/a	n/a	n/a	n/a	n/a
augustus.g33153.t1	n/a	n/a	n/a	n/a	n/a	n/a	-1.615475886	0.011598131	PREDICTED: RNA-directed DNA polymerase from mobile element jockey-like [Acropora digitifera].	XP_015747852	72.7	6.10E-70	n/a	n/a	n/a	n/a	n/a	n/a	n/a	n/a	n/a
augustus.g33166.t1	n/a	n/a	n/a	n/a	n/a	n/a	1.608841085	0.024372809	n/a	n/a	n/a	n/a	n/a	n/a	n/a	n/a	n/a	n/a	n/a	n/a	n/a
augustus.g33188.t1	n/a	n/a	n/a	n/a	n/a	n/a	1.02820367	0.004462499	ATPase family AAA domain-containing protein 3-like [Orbicella faveolata].	XP_020616979	87.1	2.00E-232	K17681	2	F:GO:000016	F:nucleotide b	n/a	n/a	n/a	n/a	n/a
augustus.g33193.t1	n/a	n/a	n/a	n/a	n/a	n/a	-4.074119276	4.44E-05	PREDICTED: uncharacterized protein LOC107332179 [Acropora digitifera].	XP_015752387	70.7	8.40E-113	n/a	n/a	n/a	n/a	n/a	n/a	n/a	n/a	n/a
augustus.g33195.t1	n/a	n/a	n/a	n/a	n/a	n/a	-3.929940281	0.043002708	PREDICTED: uncharacterized protein LOC107332179 [Acropora digitifera].	XP_015752387	71.8	4.70E-116	n/a	n/a	n/a	n/a	n/a	n/a	n/a	n/a	n/a
augustus.g33223.t1	n/a	n/a	n/a	n/a	n/a	n/a	-1.218572836	0.000741634	DNA helicase MCM8-like [Orbicella faveolata].	XP_020618820	61	3.30E-77	K10737	10	F:GO:000016	F:nucleotide b	n/a	n/a	n/a	n/a	n/a
augustus.g33244.t1	n/a	n/a	1.187001886	6.33E-07	n/a	n/a	1.461330559	1.52E-11	heat shock protein HSP 90-beta-like [Orbicella faveolata].	XP_020618369	93.3	0.00E+00	K04079	9	C:GO:000563	C:nucleus; F:	n/a	n/a	n/a	n/a	n/a
augustus.g33295.t1	n/a	n/a	n/a	n/a	n/a	n/a	1.402092901	0.000697789	serine/threonine-protein kinase D3-like [Orbicella faveolata].	XP_020608855	84.1	0.00E+00	K06070	9	F:GO:000016	F:nucleotide b	EC:2.7.11	Transferring p	IPR020454 (IF	F:GO:000552	F:ATP binding; F:protein kinase activity; P:protein phosphorylation; P:intracellular signal tra
augustus.g33396.t1	n/a	n/a	n/a	n/a	n/a	n/a	1.424629934	0.016388359	PREDICTED: retinoid-inducible serine carboxypeptidase-like [Acropora digitifera].	XP_015762490	78.5	3.70E-191	K09646	1	F:GO:001678	F:hydrolase a	n/a	n/a	n/a	n/a	n/a
augustus.g33399.t1	n/a	n/a	-3.739405379	0.029289229	n/a	n/a	n/a	n/a	PREDICTED: kinesin-like protein KIF3A, partial [Acropora digitifera].	XP_015762418	77.9	0.00E+00	n/a	n/a	n/a	n/a	n/a	n/a	n/a	n/a	n/a
augustus.g33512.t1	n/a	n/a	n/a	n/a	n/a	n/a	3.238558242	0.011450819	PREDICTED: LOW QUALITY PROTEIN: sortilin-related receptor-like [Acropora digitifera].	XP_015748796	69.8	5.20E-250	n/a	5	C:GO:001602	C:membrane;	n/a	n/a	n/a	n/a	n/a
augustus.g33517.t1	n/a	n/a	3.64187613	6.47E-06	n/a	n/a	2.707667531	0.000820698	PREDICTED: uncharacterized protein LOC107343973 [Acropora digitifera].	XP_015765058	29.5	1.70E-68	n/a	n/a	n/a	n/a	n/a	n/a	n/a	n/a	n/a
augustus.g33531.t1	n/a	n/a	n/a	n/a	n/a	n/a	5.102313681	0.02188232	PREDICTED: trace amine-associated receptor 8b-like [Acropora digitifera].	XP_015749244	65.8	2.70E-98	n/a	n/a	n/a	n/a	n/a	n/a	n/a	n/a	n/a
augustus.g33535.t1	n/a	n/a	1.918864632	0.020478647	n/a	n/a	n/a	n/a	PREDICTED: uncharacterized protein LOC107328997 isoform X1 [Acropora digitifera].	XP_015749226	80.3	2.40E-80	K20232	n/a	n/a	n/a	n/a	n/a	n/a	n/a	n/a
augustus.g33599.t1	n/a	n/a	5.424443991	0.000113034	n/a	n/a	n/a	n/a	PREDICTED: chloride intracellular channel protein 2-like [Acropora digitifera].	XP_015759980	82.6	3.40E-103	K05022	n/a	n/a	n/a	n/a	n/a	n/a	n/a	n/a
augustus.g33626.t1	n/a	n/a	n/a	n/a	n/a	n/a	-1.446715785	0.048155419	PREDICTED: utrophin-like [Acropora digitifera].	XP_015775378	60.7	6.60E-296	K10366	4	F:GO:000377	F:actin bindin	n/a	n/a	n/a	n/a	n/a
augustus.g33658.t1	n/a	n/a	n/a	n/a	n/a	n/a	1.960036625	1.25E-09	PREDICTED: [illegible]-1-like [Acropora digitifera].	XP_015756107	76	1.40E-74	K17905	6	F:GO:000016	F:nucleotide b	n/a	n/a	n/a	n/a	n/a
augustus.g33749.t1	n/a	n/a	n/a	n/a	n/a	n/a	-1.478687127	0.009289171	p53 and DNA damage-regulated protein 1-like [Orbicella faveolata].	XP_020625336	70.5	4.20E-42	n/a	3	C:GO:001627	C:prefoldin co	n/a	n/a	n/a	n/a	n/a
augustus.g33767.t1	n/a	n/a	n/a	n/a	n/a	n/a	-3.030637899	0.013729974	PREDICTED: stimulated by retinoic acid gene 6 protein homolog isoform X3 [Acropora digitifera].	XP_015765168	52.4	1.20E-47	n/a	n/a	n/a	n/a	n/a	n/a	n/a	n/a	n/a
augustus.g33796.t1	n/a	n/a	1.27390645	0.005358578	n/a	n/a	2.245315739	1.17E-10	PREDICTED: mitogen-activated protein kinase 4-like [Acropora digitifera].	XP_015755188	81.4	1.10E-236	K06855	1	F:GO:001630	F:kinase activ	n/a	n/a	n/a	n/a	n/a
augustus.g33874.t1	n/a	n/a	n/a	n/a	n/a	n/a	-3.45937028	0.001737961	uncharacterized protein LOC110064325 [Orbicella faveolata].	XP_020627028	61.2	1.80E-90	n/a	n/a	n/a	n/a	n/a	n/a	n/a	n/a	n/a
augustus.g33875.t1	n/a	n/a	n/a	n/a	n/a	n/a	-2.823488002	0.002152861	uncharacterized protein LOC110064316 [Orbicella faveolata].	XP_020627016	47.2	6.00E-65	n/a	n/a	n/a	n/a	n/a	n/a	n/a	n/a	n/a
augustus.g33924.t1	n/a	n/a	n/a	n/a	n/a	n/a	-1.399433329	0.039221268	Katanin p60 ATPase-containing subunit A-like 2 [Exaiptasia pallida]	KXJ28774.1	42	2.00E-54	n/a	n/a	n/a	n/a	n/a	n/a	n/a	n/a	n/a
augustus.g33950.t1	n/a	n/a	n/a	n/a	n/a	n/a	1.824361052	7.68E-07	PREDICTED: myocyte-specific enhancer factor 2A-like isoform X1 [Acropora digitifera].	XP_015763926	83.7	1.00E-202	K09260	8	F:GO:000367	F:DNA bindin	n/a	n/a	n/a	n/a	n/a
augustus.g33957.t1	n/a	n/a	1.652929895	0.046723241	n/a	n/a	1.614530838	0.016322778	calumenin-A-like isoform X1 [Stylophora pistillata]	XP_022797263.1	40	2.00E-47	n/a	3	P:GO:000716	P:signal transd	n/a	n/a	n/a	n/a	n/a
augustus.g33958.t1	n/a	n/a	2.807137157	0.001341673	n/a	n/a	2.185564648	0.008881778	calumenin-like [Orbicella faveolata].	XP_020621477	55.5	1.40E-85	n/a	1	F:GO:000550	F:calcium ion	n/a	n/a	n/a	n/a	n/a
augustus.g33968.t1	n/a	n/a	n/a	n/a	n/a	n/a	2.47252331	0.024114174	n/a	n/a	n/a	n/a	n/a	n/a	n/a	n/a	n/a	n/a	n/a	n/a	n/a
augustus.g33989.t1	n/a	n/a	n/a	n/a	n/a	n/a	-2.891895198	0.000207639	PREDICTED: putative histone-lysine N-methyltransferase PRDM6 isoform X1 [Acropora digitifera].	XP_015754032	67.9	4.80E-142	K20795	n/a	n/a	n/a	n/a	n/a	n/a	n/a	n/a
augustus.g3411.t1	n/a	n/a	n/a	n/a	n/a	n/a	1.892748713	0.045747621	PREDICTED: uncharacterized protein LOC107338465 [Acropora digitifera].	XP_015759189	63.2	4.30E-95	n/a	n/a	n/a	n/a	n/a	n/a	n/a	n/a	n/a
augustus.g34164.t1	n/a	n/a	n/a	n/a	n/a	n/a	2.497831652	0.00135661	uncharacterized protein LOC110055983 [Orbicella faveolata].	XP_020618080	39.5	2.10E-37	n/a	n/a	n/a	n/a	n/a	n/a	n/a	n/a	n/a
augustus.g34384.t1	n/a	n/a	n/a	n/a	n/a	n/a	1.552109751	0.012717573	PREDICTED: iron-sulfur cluster assembly 1 homolog, mitochondrial-like [Acropora digitifera].	XP_015778293	87.8	7.30E-61	K22063	7	C:GO:000575	C:mitochondr	n/a	n/a	n/a	n/a	n/a
augustus.g34399.t1	n/a	n/a	2.756496502	0.001235072	n/a	n/a	2.405594073	0.002625854	PREDICTED: ras-related protein Rap-2a-like [Acropora digitifera].	XP_015757214	86.3	7.10E-97	K07841	n/a	n/a	n/a	n/a	n/a	n/a	n/a	n/a
augustus.g34401.t1	n/a	n/a	3.415333338	4.77E-15	n/a	n/a	3.486040814	2.44E-16	PREDICTED: transcription factor AP-1-like [Acropora digitifera].	XP_015757213	78.1	3.90E-126	K04448	8	F:GO:000367	F:DNA bindin	n/a	n/a	n/a	n/a	n/a
augustus.g34408.t1	n/a	n/a	n/a	n/a	n/a	n/a	-1.015624645	0.035923597	PREDICTED: uncharacterized protein LOC107340729 [Acropora digitifera].	XP_015761593	83.1	0.00E+00	n/a	n/a	n/a	n/a	n/a	n/a	n/a	n/a	n/a
augustus.g34436.t1	n/a	n/a	-1.032020146	0.00541774	n/a	n/a	n/a	n/a	kelch-like protein 20 [Orbicella faveolata].	XP_020616476	92.2	4.30E-224	n/a	9	C:GO:000563	C:nucleus; F:	n/a	n/a	n/a	n/a	n/a
augustus.g34451.t1	n/a	n/a	n/a	n/a	n/a	n/a	1.121842774	0.044762487	proteasome-associated protein ECM29 homolog [Orbicella faveolata].	XP_020604939	62	0.00E+00	K11886	2	F:GO:003294	F:protein com	n/a	n/a	n/a	n/a	n/a
augustus.g34502.t1	n/a	n/a	n/a	n/a	n/a	n/a	2.02763315	0.041838061	uncharacterized protein LOC110064730 [Orbicella faveolata].	XP_020627485	73	5.00E-216	n/a	n/a	n/a	n/a	n/a	n/a	n/a	n/a	n/a
augustus.g34603.t1	n/a	n/a	n/a	n/a	n/a	n/a	1.157712227	0.006985317	3-ketoacyl-CoA thiolase, mitochondrial-like [Orbicella faveolata].	XP_020605842	89.9	1.20E-201	K07508	7	C:GO:000700	C:extracellula	n/a	n/a	n/a	n/a	n/a
augustus.g34620.t1	n/a	n/a	2.475700198	0.003577723	n/a	n/a	n/a	n/a	PREDICTED: multidrug resistance-associated protein 4-like [Acropora digitifera].	XP_015763824	75.2	0.00E+00	n/a	n/a	n/a	n/a	n/a	n/a	n/a	n/a	n/a
augustus.g34624.t1	n/a	n/a	n/a	n/a	n/a	n/a	1.080999457	0.048007644	PREDICTED: osmotic avoidance abnormal protein 3-like [Acropora digitifera].	XP_015763790	85.9	0.00E+00	K20198	n/a	n/a	n/a	n/a	n/a	n/a	n/a	n/a
augustus.g34627.t1	n/a	n/a	n/a	n/a	n/a	n/a	3.890300283	3.62E-06	n/a	n/a	n/a	n/a	n/a	n/a	n/a	n/a	n/a	n/a	n/a	n/a	n/a
augustus.g34628.t1	n/a	n/a	n/a	n/a	n/a	n/a	4.322235611	5.51E-23	PREDICTED: AN1-type zinc finger protein 2B-like isoform X1 [Acropora digitifera].	XP_015763793	81.6	1.10E-70	n/a	2	F:GO:000827	F:zinc ion bin	n/a	n/a	n/a	n/a	n/a
augustus.g34629.t1	n/a	n/a	n/a	n/a	n/a	n/a	1.148866273	2.10E-06	Isocitrate dehydrogenase [NADP] cytoplasmic-like [Stylophora pistillata]	XP_022800770	89	3.30E-202	K00031	16	F:GO:004280	F:protein hon	EC:1.1.1.42	Isocitrate deh	IPR024084 (IF	F:GO:001661	F:oxidoreductase activity, acting on the CH-OH group of donors, NAD or NADP as accepto
augustus.g34656.t1	n/a	n/a	n/a	n/a	n/a	n/a	3.338054848	0.009554228	Centrosomal protein of 290 kDa [Exaiptasia pallida].	KXJ10827	39.3	4.60E-35	n/a	n/a	n/a	n/a	n/a	n/a	n/a	n/a	n/a
augustus.g3477.t1	n/a	n/a	n/a	n/a	n/a	n/a	2.543020737	0.042921591	PREDICTED: uncharacterized protein LOC107348635 [Acropora digitifera].	XP_015770173	83.4	2.10E-88	n/a	n/a	n/a	n/a	n/a	n/a	n/a	n/a	n/a
augustus.g34785.t1	n/a	n/a	1.69262251	0.000263784	n/a	n/a	n/a	n/a	PREDICTED: early endosome antigen 1-like isoform X3 [Acropora digitifera].	XP_015767528.1	57	0	n/a	n/a	n/a	n/a	n/a	n/a	n/a	n/a	n/a
augustus.g34805.t1	n/a	n/a	1.389118386	3.80E-08	n/a	n/a	n/a	n/a	mRNA decay activator protein ZFP36L1-like [Orbicella faveolata].	XP_020603722	78.8	1.10E-45	K18753	n/a	n/a	n/a	n/a	n/a	n/a	n/a	n/a

augustus.g43430.t1	n/a	n/a	2.802184775	4.25E-05	n/a	n/a	n/a	n/a	PREDICTED: uncharacterized protein LOC107334854 [Acropora digitifera].	XP_015755303	51.5	2.10E-112	n/a	n/a
augustus.g43690.t1	n/a	n/a	22.12807074	0.00700553	21.87939675	0.021227623	22.24164674	0.001638622	uncharacterized protein LOC110053458 [Orbicella faveolata].	XP_020615341	27.3	2.20E-39	n/a	n/a
augustus.g43730.t1	n/a	n/a	n/a	n/a	n/a	n/a	-2.022620416	0.044762487	calcium/calmodulin-dependent protein kinase type II alpha chain-like [Orbicella faveolata].	XP_020609534	85.1	2.10E-156	K04515	n/a
augustus.g4378.t1	n/a	n/a	n/a	n/a	-8.832139779	0.002976616	n/a	n/a	PREDICTED: uromodulin-like isoform X1 [Acropora digitifera].	XP_015760281	60	4.80E-42	n/a	n/a
augustus.g4389.t1	n/a	n/a	n/a	n/a	n/a	n/a	1.112868813	0.014165232	E3 ubiquitin-protein ligase RNF31-like isoform X3 [Orbicella faveolata].	XP_020631211	55	0.00E+00	K11974	n/a
augustus.g43928.t1	n/a	n/a	n/a	n/a	n/a	n/a	-1.891553345	0.000216975	n/a	n/a	n/a	n/a	n/a	n/a
augustus.g43951.t1	n/a	n/a	4.125891365	0.001212833	n/a	n/a	3.044930689	0.013591718	paired box D2 protein [Acropora digitifera].	BAQ19097	79.4	2.30E-140	K09381	1
augustus.g44012.t1	n/a	n/a	n/a	n/a	n/a	n/a	1.095292559	0.034922955	cytosolic carboxypeptidase 6-like [Orbicella faveolata].	XP_020620718	87.6	5.40E-249	n/a	n/a
augustus.g44091.t1	n/a	n/a	n/a	n/a	n/a	n/a	4.21119213	0.007586817	PREDICTED: uncharacterized protein LOC107338305 [Acropora digitifera].	XP_015759022	39.6	7.50E-96	K06619	1
augustus.g44120.t1	n/a	n/a	n/a	n/a	n/a	n/a	-1.913579175	0.044706321	PREDICTED: uncharacterized protein LOC107338313 [Acropora digitifera].	XP_015759033	63	7.70E-148	n/a	n/a
augustus.g44178.t1	n/a	n/a	n/a	n/a	n/a	n/a	-4.269274263	0.029686964	PREDICTED: multidrug resistance-associated protein 4-like [Acropora digitifera].	XP_015780908	79.1	2.10E-210	n/a	n/a
augustus.g44179.t1	n/a	n/a	n/a	n/a	n/a	n/a	-2.655318319	0.015014366	multidrug resistance-associated protein 4-like [Orbicella faveolata].	XP_020601175	49.5	2.60E-83	K05673	n/a
augustus.g4426.t1	n/a	n/a	n/a	n/a	n/a	n/a	-3.79119672	0.00159818	PREDICTED: uncharacterized protein LOC107344490 isoform X1 [Acropora digitifera].	XP_015765638	52.2	1.00E-208	n/a	n/a
augustus.g44327.t1	n/a	n/a	n/a	n/a	n/a	n/a	3.727130691	0.006949247	uncharacterized protein LOC110056377 [Orbicella faveolata].	XP_020618503	46.3	3.50E-208	n/a	3
augustus.g44402.t1	n/a	n/a	n/a	n/a	1.990519071	2.57E-05	1.171096255	0.021118903	PREDICTED: transient receptor potential cation channel subfamily A member 1 homolog [Acropora digit	XP_015765476	69.7	4.10E-272	n/a	n/a
augustus.g44404.t1	n/a	n/a	n/a	n/a	n/a	n/a	-1.4723352	4.56E-05	quinone oxidoreductase PIG3-like [Orbicella faveolata].	XP_020622355	73.3	1.10E-139	K10133	3
augustus.g44420.t1	n/a	n/a	n/a	n/a	n/a	n/a	3.422813937	0.000230386	uncharacterized protein LOC110059901 [Orbicella faveolata].	XP_020622276	62.5	3.40E-12	n/a	n/a
augustus.g44503.t1	n/a	n/a	n/a	n/a	n/a	n/a	5.075401072	0.030977867	hypothetical protein AC249_AIPGENE16794 [Exaiptasia pallida].	KXJ09427	34.3	3.90E-170	n/a	4
augustus.g44550.t1	n/a	n/a	1.651141722	0.010697324	3.138357947	2.21E-06	4.360353235	2.68E-14	PREDICTED: F-box/LRR-repeat protein 20-like [Acropora digitifera].	XP_015759581	75.2	5.10E-185	n/a	5
augustus.g44563.t1	n/a	n/a	n/a	n/a	n/a	n/a	3.395320037	7.14E-16	hepatocyte growth factor-regulated tyrosine kinase substrate-like [Orbicella faveolata].	XP_020608686	72.9	6.20E-180	K12182	5
augustus.g4459.t1	n/a	n/a	-2.83475297	0.032441093	n/a	n/a	n/a	n/a	hypothetical protein AC249_AIPGENE16139 [Exaiptasia pallida].	KXJ16672	34.4	8.60E-213	K08592	2
augustus.g44594.t1	n/a	n/a	n/a	n/a	n/a	n/a	-2.168887276	0.033896262	steroid 17-alpha-hydroxylase/17,20 lyase-like [Orbicella faveolata].	XP_020609020	63.5	1.10E-189	n/a	n/a
augustus.g44595.t1	n/a	n/a	n/a	n/a	n/a	n/a	-1.578099807	0.042788808	PREDICTED: riboflavin-binding protein-like [Acropora digitifera].	XP_015764670	66.7	2.30E-94	K13649	n/a
augustus.g44608.t1	n/a	n/a	n/a	n/a	n/a	n/a	-1.206940605	0.003163597	calmodulin-like protein, partial [Acropora millepora].	ACY07618	81.4	6.30E-19	K02183	12
augustus.g44609.t1	n/a	n/a	n/a	n/a	n/a	n/a	1.603856081	0.043745873	n/a	n/a	n/a	n/a	n/a	n/a
augustus.g44633.t1	n/a	n/a	n/a	n/a	n/a	n/a	2.554631755	7.09E-05	PREDICTED: activated CDC42 kinase 1-like [Acropora digitifera]	XP_015776163.1	84	3.00E-122	n/a	n/a
augustus.g4468.t1	n/a	n/a	1.263359973	0.001256592	n/a	n/a	4.278450788	4.32E-49	PREDICTED: uncharacterized protein LOC107349868 [Acropora digitifera].	XP_015771546	65	0.00E+00	n/a	3
augustus.g44703.t1	n/a	n/a	n/a	n/a	n/a	n/a	1.882000707	0.031007512	n/a	n/a	n/a	n/a	n/a	n/a
augustus.g44723.t1	n/a	n/a	n/a	n/a	n/a	n/a	2.023594595	0.03942526	uncharacterized protein LOC110048052 isoform X1 [Orbicella faveolata].	XP_020609478	67.6	6.20E-84	K15074	n/a
augustus.g44729.t1	n/a	n/a	n/a	n/a	n/a	n/a	1.02195712	0.043977131	glutathione S-transferase-like isoform X1 [Orbicella faveolata].	XP_020609412	79.9	5.10E-127	n/a	2
augustus.g44730.t1	n/a	n/a	n/a	n/a	n/a	n/a	2.561992059	2.17E-19	ubiquitin-protein ligase E3C-like [Orbicella faveolata].	XP_020609438	80.1	0.00E+00	K10589	6
augustus.g44731.t1	n/a	n/a	n/a	n/a	n/a	n/a	2.106457502	2.94E-06	ubiquitin-protein ligase E3C-like [Orbicella faveolata].	XP_020609438	90.3	8.70E-43	K10589	3
augustus.g44792.t1	n/a	n/a	n/a	n/a	n/a	n/a	1.660927023	0.017777521	uncharacterized protein LOC110051686 isoform X2 [Orbicella faveolata].	XP_020613404	52.3	2.60E-141	K09445	1
augustus.g44797.t1	n/a	n/a	n/a	n/a	n/a	n/a	1.267259053	2.51E-06	PREDICTED: transmembrane protein 198-B-like [Acropora digitifera].	XP_015754849	75.2	2.30E-129	n/a	n/a
augustus.g44838.t1	n/a	n/a	n/a	n/a	n/a	n/a	1.058905147	0.01773976	headcase protein homolog [Orbicella faveolata].	XP_020620132	86.4	4.20E-216	n/a	n/a
augustus.g44867.t1	n/a	n/a	n/a	n/a	n/a	n/a	2.022632751	1.75E-06	PREDICTED: tyrosine-protein kinase BTK-like [Acropora digitifera].	XP_015752489	53.9	2.80E-141	K07364	n/a
augustus.g45088.t1	n/a	n/a	n/a	n/a	n/a	n/a	2.277119136	0.000528917	PREDICTED: RNA-directed DNA polymerase from mobile element jockey-like [Acropora digitifera].	XP_015768399	54.5	2.30E-33	n/a	n/a
augustus.g45178.t1	n/a	n/a	2.70811799	2.62E-05	n/a	n/a	2.567711736	0.000107348	n/a	n/a	n/a	n/a	n/a	n/a
augustus.g45179.t1	1.453540067	0.013876226	2.552037307	2.74E-10	n/a	n/a	1.934516584	2.68E-06	PREDICTED: uncharacterized protein LOC107351698 [Acropora digitifera].	XP_015773472	63.1	0.00E+00	K09228	n/a
augustus.g45188.t1	n/a	n/a	n/a	n/a	n/a	n/a	1.117943435	0.025995442	PREDICTED: fibroblast growth factor receptor 4-like [Acropora digitifera].	XP_015758535	61.5	0.00E+00	K05094	n/a
augustus.g45208.t1	n/a	n/a	n/a	n/a	n/a	n/a	-1.208650656	0.002794048	PREDICTED: erythroid differentiation-related factor 1-like [Acropora digitifera].	XP_015758533	71.4	0.00E+00	n/a	n/a
augustus.g45219.t1	1.277538965	0.013379875	1.163173457	0.014873413	n/a	n/a	n/a	n/a	nucleolar protein 56-like [Orbicella faveolata].	XP_020623790	90.5	5.90E-222	K14564	n/a
augustus.g45220.t1	n/a	n/a	n/a	n/a	n/a	n/a	-1.130222309	0.013427204	STAGA complex 65 subunit gamma-like [Orbicella faveolata].	XP_020623812	83.4	3.30E-132	K11316	n/a
augustus.g45229.t1	n/a	n/a	-1.047403464	0.010974874	n/a	n/a	n/a	n/a	PREDICTED: dopamine receptor 2-like [Acropora digitifera].	XP_015758564	76.1	2.40E-159	n/a	4
augustus.g45264.t1	n/a	n/a	n/a	n/a	n/a	n/a	-1.771299074	0.004385034	methylated-DNA--protein-cysteine methyltransferase-like [Orbicella faveolata].	XP_020623885	62.4	7.70E-55	K00567	13
augustus.g45290.t1	n/a	n/a	n/a	n/a	n/a	n/a	1.991433213	7.36E-06	PREDICTED: high mobility group nucleosome-binding domain-containing protein 5-like [Acropora digit	XP_015747327	45.8	2.30E-33	n/a	n/a
augustus.g45305.t1	n/a	n/a	n/a	n/a	n/a	n/a	3.44115754	1.45E-14	PREDICTED: tumor necrosis factor receptor superfamily member 4-like isoform X2 [Acropora digitifera]	XP_015761851	42.9	4.80E-72	n/a	n/a
augustus.g45335.t1	n/a	n/a	n/a	n/a	n/a	n/a	4.055375049	0.024042816	n/a	n/a	n/a	n/a	K10352	n/a
augustus.g45338.t1	n/a	n/a	n/a	n/a	n/a	n/a	-1.002158883	0.004353259	PREDICTED: early endosome antigen 1-like isoform X2 [Acropora digitifera].	XP_015761832	59.4	1.70E-58	K12478	1
augustus.g45391.t1	n/a	n/a	n/a	n/a	n/a	n/a	3.311706158	8.33E-07	PREDICTED: hematopoietic prostaglandin D synthase-like isoform X1 [Acropora digitifera].	XP_015757626	67.7	2.20E-80	K04097	n/a
augustus.g45508.t1	n/a	n/a	n/a	n/a	n/a	n/a	-1.006350612	0.04487857	uncharacterized protein LOC110050239 isoform X1 [Orbicella faveolata].	XP_020611800	82.7	3.30E-152	n/a	6
augustus.g4551.t1	n/a	n/a	n/a	n/a	n/a	n/a	-1.575168559	0.047152954	n/a	n/a	n/a	n/a	K15295	n/a
augustus.g45510.t1	n/a	n/a	n/a	n/a	n/a	n/a	2.339966688	3.71E-06	growth arrest and DNA damage-inducible protein GADD45 beta-like [Orbicella faveolata].	XP_020611869	73	1.10E-54	K04402	n/a
augustus.g45538.t1	n/a	n/a	n/a	n/a	n/a	n/a	1.245736329	0.003494539	PREDICTED: malate synthase-like isoform X1 [Acropora digitifera].	XP_015757443	86.2	1.20E-290	K01638	n/a
augustus.g45554.t1	1.309307828	0.004435025	1.627859779	8.56E-06	2.775346785	3.24E-17	3.719321838	8.55E-33	PREDICTED: LON peptidase N-terminal domain and RING finger protein 3-like [Acropora digitifera].	XP_015769251	64.2	3.50E-244	n/a	4
augustus.g45665.t1	n/a	n/a	n/a	n/a	n/a	n/a	1.282563609	0.001368327	putative insulin-like peptide receptor [Orbicella faveolata].	XP_020600867	57.1	4.80E-88	K05087	2
augustus.g45671.t1	n/a	n/a	n/a	n/a	n/a	n/a	1.276248691	0.001978317	predicted protein, partial [Nematostella vectensis].	XP_001639714	51.1	1.00E-60	n/a	4
augustus.g457.t1	n/a	n/a	n/a	n/a	n/a	n/a	1.645196252	5.26E-08	PREDICTED: tolloid-like protein 2 isoform X2 [Acropora digitifera].	XP_015779687	59.8	1.70E-129	K14616	n/a
augustus.g45706.t1	n/a	n/a	1.130613272	0.015221367	n/a	n/a	n/a	n/a	n/a	n/a	n/a	n/a	n/a	3
augustus.g45721.t1	n/a	n/a	2.110157044	2.73E-05	n/a	n/a	n/a	n/a	PREDICTED: carbohydrate sulfotransferase 11-like [Acropora digitifera].	XP_015764887	67.4	1.40E-123	K01017	n/a
augustus.g45769.t1	n/a	n/a	1.666371992	0.010697324	n/a	n/a	n/a	n/a	PREDICTED: smoothelin-like protein 1 [Acropora digitifera].	XP_015762997	76.2	3.80E-228	n/a	n/a
augustus.g45842.t1	n/a	n/a	n/a	n/a	n/a	n/a	-1.407272749	0.001986643	PREDICTED: short transient receptor potential channel 3-like isoform X1 [Acropora digitifera].	XP_015765945	70	5.00E-297	n/a	n/a

[illegible]

augustus.g60042.t1	n/a	n/a	n/a	n/a	n/a	n/a	1.608823056	4.21E-05	uncharacterized protein LOC110066933 [Orbicella faveolata].	XP_020629849	87.4	2.10E-219	n/a
augustus.g60043.t1	n/a	n/a	n/a	n/a	n/a	n/a	1.358136746	3.41E-05	PREDICTED: interaptin-like [Acropora digitifera].	XP_015758804	49.8	1.00E-93	K19946
augustus.g60074.t1	2.775524708	1.12E-28	3.107345718	7.55E-37	6.293965841	3.45E-85	7.023494892	6.93E-107	PREDICTED: thyrotroph embryonic factor-like [Acropora digitifera].	XP_015747987	73.1	1.20E-91	K09057
augustus.g60119.t1	n/a	n/a	n/a	n/a	n/a	n/a	-4.316818142	0.000178714	PREDICTED: uncharacterized protein LOC107353686 isoform X4 [Acropora digitifera].	XP_015775518	56.5	0.00E+00	K07904
augustus.g60154.t1	n/a	n/a	2.202842456	0.002594994	n/a	n/a	1.534034958	0.03432989	PREDICTED: SPRY domain-containing SOCS box protein 1-like isoform X1 [Acropora digitifera].	XP_015760530	90.4	7.50E-170	K10343
augustus.g60331.t1	n/a	n/a	n/a	n/a	n/a	n/a	-2.68757794	0.002295526	PREDICTED: DDB1- and CUL4-associated factor 5-like [Acropora digitifera].	XP_015755345	64.9	2.00E-242	K11800
augustus.g60345.t1	n/a	n/a	n/a	n/a	n/a	n/a	1.061313258	0.029152609	delta-like protein [Euphyllia ancora].	AKR17061	56.3	2.40E-111	K06051
augustus.g60346.t1	n/a	n/a	n/a	n/a	n/a	n/a	1.046378145	9.02E-05	calcium-independent protein kinase C-like [Orbicella faveolata].	XP_020604227	81.6	0.00E+00	K18050
augustus.g60350.t1	n/a	n/a	n/a	n/a	n/a	n/a	1.761358468	2.33E-12	PREDICTED: cell wall protein AWA1-like [Acropora digitifera].	XP_015751213	56.2	7.60E-100	n/a
augustus.g60356.t1	n/a	n/a	n/a	n/a	n/a	n/a	1.116708062	0.012133237	PREDICTED: heat shock 70 kDa protein 13-like [Acropora digitifera].	XP_015751141	88.8	6.30E-220	K09491
augustus.g60451.t1	n/a	n/a	n/a	n/a	n/a	n/a	1.866748625	1.41E-07	E3 ubiquitin-protein ligase RNF4-like [Orbicella faveolata].	XP_020619157	45.2	7.30E-54	n/a
augustus.g60476.t1	n/a	n/a	n/a	n/a	n/a	n/a	6.17257057	1.96E-05	PREDICTED: poly [ADP-ribose] polymerase 14-like [Acropora digitifera].	XP_015776502	61.5	0.00E+00	K15261
augustus.g60502.t1	n/a	n/a	n/a	n/a	3.798949964	7.45E-11	3.31876988	9.26E-09	protein phosphatase 1 regulatory subunit 3D-like [Orbicella faveolata].	XP_020631339	56.5	4.80E-49	K07189
augustus.g60582.t1	n/a	n/a	n/a	n/a	n/a	n/a	-1.226524144	0.005102056	PREDICTED: uncharacterized protein LOC107341272 [Acropora digitifera].	XP_015762159	44.1	7.00E-24	n/a
augustus.g60646.t1	n/a	n/a	n/a	n/a	n/a	n/a	1.22234399	0.030050064	sodium bicarbonate cotransporter 3-like isoform X2 [Orbicella faveolata].	XP_020624364	78.2	0.00E+00	K13861
augustus.g60725.t1	n/a	n/a	n/a	n/a	n/a	n/a	-1.309407867	0.00028148	glycine dehydrogenase (decarboxylating), mitochondrial-like [Orbicella faveolata].	XP_020624693	75.4	0.00E+00	K00281
augustus.g60790.t1	n/a	n/a	n/a	n/a	n/a	n/a	-3.725692943	0.040980788	PREDICTED: uncharacterized protein LOC107351542 [Acropora digitifera].	XP_015773320	53.8	1.10E-111	K17533
augustus.g6087.t1	n/a	n/a	n/a	n/a	n/a	n/a	1.991002955	0.016353683	monocarboxylate transporter 10-like [Orbicella faveolata].	XP_020604263	66	1.20E-144	n/a
augustus.g60891.t1	n/a	n/a	1.609969063	0.007016719	n/a	n/a	n/a	n/a	n/a	n/a	n/a	n/a	n/a
augustus.g60897.t1	n/a	n/a	n/a	n/a	n/a	n/a	1.519921523	0.034756864	protein fem-1 homolog C-like [Orbicella faveolata].	XP_020632770	53.7	5.90E-63	n/a
augustus.g60902.t1	n/a	n/a	n/a	n/a	n/a	n/a	-3.249212277	0.019471766	collagen triple helix repeat-containing protein 1-like [Orbicella faveolata].	XP_020618704	58.3	4.10E-56	n/a
augustus.g60904.t1	n/a	n/a	n/a	n/a	n/a	n/a	2.42500981	5.40E-15	PREDICTED: protein patched homolog 1-like, partial [Acropora digitifera].	XP_015768314	74.7	0.00E+00	n/a
augustus.g60906.t1	n/a	n/a	n/a	n/a	n/a	n/a	2.663331744	5.99E-18	ATP-binding cassette sub-family A member 1-like [Orbicella faveolata].	XP_020625798	67.6	0.00E+00	K05641
augustus.g60907.t1	n/a	n/a	n/a	n/a	n/a	n/a	1.291027505	0.009289171	PREDICTED: F-box/WD repeat-containing protein 9-like [Acropora digitifera].	XP_015769483	74.7	8.50E-179	K10265
augustus.g60912.t1	n/a	n/a	n/a	n/a	n/a	n/a	-1.053591088	0.011976292	PREDICTED: uncharacterized protein LOC107348074 [Acropora digitifera].	XP_015769569	88.6	1.10E-151	n/a
augustus.g60913.t1	n/a	n/a	n/a	n/a	n/a	n/a	-1.096100133	0.00236945	PREDICTED: fukutin-like isoform X2 [Acropora digitifera].	XP_015769500	70.3	4.40E-195	K19872
augustus.g60997.t1	1.729097207	0.004435025	3.263728773	4.09E-14	n/a	n/a	n/a	n/a	PREDICTED: matrix metalloproteinase-24-like [Acropora digitifera].	XP_015771447	73.1	2.10E-193	K07763
augustus.g61027.t1	n/a	n/a	n/a	n/a	5.853343293	0.030955219	5.490509607	0.007276345	PREDICTED: uncharacterized protein LOC107353173 [Acropora digitifera].	XP_015774979	62.3	3.10E-60	n/a
augustus.g61028.t1	n/a	n/a	n/a	n/a	n/a	n/a	1.5066232	0.024710118	PREDICTED: AN1-type zinc finger protein 6-like [Acropora digitifera].	XP_015774982	93.7	1.90E-40	n/a
augustus.g61114.t1	-2.051666312	0.004577138	n/a	n/a	n/a	n/a	n/a	n/a	n/a	n/a	n/a	n/a	n/a
augustus.g61128.t1	n/a	n/a	n/a	n/a	n/a	n/a	1.014569238	0.000456481	PREDICTED: transcriptional repressor p66-beta-like [Acropora digitifera].	XP_015760859	73.6	6.00E-186	n/a
augustus.g61141.t1	n/a	n/a	n/a	n/a	n/a	n/a	1.380849686	1.83E-09	PREDICTED: LOW QUALITY PROTEIN: low-density lipoprotein receptor-related protein 4-like [Acropo	XP_015780521	73.4	9.10E-213	n/a
augustus.g61441.t1	n/a	n/a	n/a	n/a	n/a	n/a	-3.640248737	0.041221265	uncharacterized protein LOC110062006 isoform X1 [Orbicella faveolata].	XP_020624541	74.5	2.10E-93	n/a
augustus.g61573.t1	n/a	n/a	n/a	n/a	n/a	n/a	1.031379813	0.038888112	PREDICTED: embryonic stem cell-specific 5-hydroxymethylcytosine-binding protein-like isoform X1 [A	XP_015756305	78.2	3.90E-148	n/a
augustus.g61660.t1	n/a	n/a	n/a	n/a	n/a	n/a	-3.000166923	0.002938869	multidrug resistance-associated protein 4-like [Orbicella faveolata].	XP_020607897	61.2	0.00E+00	n/a
augustus.g61706.t1	n/a	n/a	n/a	n/a	n/a	n/a	1.840412501	0.000247629	PREDICTED: uncharacterized protein LOC107336709 isoform X2 [Acropora digitifera].	XP_015757261	47.4	2.20E-46	n/a
augustus.g61765.t1	n/a	n/a	n/a	n/a	n/a	n/a	3.764274497	0.01811554	Caltractin [Exaiptasia pallida].	KXJ28037	46.9	1.20E-79	n/a
augustus.g61766.t1	n/a	n/a	2.037764241	0.046098146	n/a	n/a	n/a	n/a	PREDICTED: uncharacterized protein LOC107339100 [Acropora digitifera].	XP_015759828	75	5.70E-38	n/a
augustus.g61783.t1	n/a	n/a	n/a	n/a	n/a	n/a	4.770257811	0.043976697	heat shock transcription factor, X-linked-like [Orbicella faveolata].	XP_020617237	51.1	1.10E-69	n/a
augustus.g61794.t1	n/a	n/a	n/a	n/a	n/a	n/a	1.407159682	0.014693559	PREDICTED: transmembrane and TPR repeat-containing protein 2-like [Acropora digitifera].	XP_015756741	62.2	3.50E-230	n/a
augustus.g61867.t1	n/a	n/a	n/a	n/a	n/a	n/a	-1.041237027	0.006818446	PACRG-like protein [Orbicella faveolata].	XP_020606993	86.2	3.60E-90	n/a
augustus.g61875.t1	n/a	n/a	n/a	n/a	n/a	n/a	-1.026544121	0.032653528	protein DJ-like [Orbicella faveolata].	XP_020630296	86.2	2.50E-84	n/a
augustus.g61950.t1	n/a	n/a	1.070809482	0.041321876	n/a	n/a	n/a	n/a	Krueppel-like factor 5 [Orbicella faveolata].	XP_020619816	78.3	6.60E-113	K09207
augustus.g61953.t1	n/a	n/a	n/a	n/a	n/a	n/a	1.397854046	1.93E-06	TBC1 domain family member 1 [Exaiptasia pallida].	KXJ23926	46.5	9.60E-277	K17902
augustus.g61996.t1	n/a	n/a	n/a	n/a	n/a	n/a	-1.58285681	0.010849065	PCNA-associated factor-like [Orbicella faveolata].	XP_020628848	75	3.40E-18	n/a
augustus.g62052.t1	-5.937664738	0.008305377	-5.637045934	0.003684746	n/a	n/a	n/a	n/a	uncharacterized protein LOC110060551 isoform X1 [Orbicella faveolata].	XP_020622997	64.4	0.00E+00	n/a
augustus.g62089.t1	n/a	n/a	-2.342894957	0.01719565	n/a	n/a	-2.597125025	0.001317802	uncharacterized protein LOC110047759 [Orbicella faveolata].	XP_020609165	55.2	7.80E-23	n/a
augustus.g62090.t1	n/a	n/a	2.123517796	0.04175215	n/a	n/a	n/a	n/a	PREDICTED: uncharacterized protein LOC107354720 [Acropora digitifera].	XP_015776692	69.8	2.80E-11	n/a
augustus.g62147.t1	n/a	n/a	n/a	n/a	n/a	n/a	1.219382037	0.002845797	mitochondrial intermembrane space import and assembly protein 40-like [Orbicella faveolata].	XP_020627523	91.5	1.10E-54	K17782
augustus.g62182.t1	n/a	n/a	n/a	n/a	n/a	n/a	-1.281649173	0.011976292	PREDICTED: golgin subfamily A member 6-like protein 22 [Acropora digitifera].	XP_015759860	63.4	2.80E-213	n/a
augustus.g62260.t1	n/a	n/a	n/a	n/a	n/a	n/a	1.49092292	8.68E-07	TBC1 domain family member 10A-like [Orbicella faveolata].	XP_020616342	79.2	1.80E-164	K19944
augustus.g62470.t1	n/a	n/a	n/a	n/a	n/a	n/a	1.037596333	7.89E-05	oxysterol-binding protein-related protein 1-like [Orbicella faveolata].	XP_020604214	79.2	7.70E-288	K20174
augustus.g62599.t1	n/a	n/a	1.014331445	0.002822895	n/a	n/a	n/a	n/a	PREDICTED: allatostatin-A receptor-like [Acropora digitifera].	XP_015752050	76.9	1.60E-147	n/a
augustus.g62605.t1	n/a	n/a	n/a	n/a	n/a	n/a	1.440523619	3.17E-07	predicted protein [Nematostella vectensis].	XP_001632039	86.6	0.00E+00	K03006
augustus.g62655.t1	n/a	n/a	n/a	n/a	n/a	n/a	2.188009677	0.014452614	PREDICTED: tetratricopeptide repeat protein 38-like [Acropora digitifera].	XP_015763421	79.8	1.30E-215	n/a
augustus.g62660.t1	n/a	n/a	n/a	n/a	n/a	n/a	2.069857631	2.60E-11	predicted protein [Nematostella vectensis].	XP_001630467	50.6	2.60E-123	n/a
augustus.g62717.t1	n/a	n/a	1.20982996	0.013160206	n/a	n/a	n/a	n/a	PREDICTED: RING finger protein 44-like [Acropora digitifera].	XP_015775721	85.6	4.50E-148	K19041
augustus.g62726.t1	n/a	n/a	n/a	n/a	n/a	n/a	1.521306616	2.91E-09	ribosomal protein S6 kinase alpha-3-like isoform X2 [Orbicella faveolata].	XP_020627341	93.6	0.00E+00	K04373
augustus.g6273.t1	n/a	n/a	n/a	n/a	n/a	n/a	3.613788338	1.46E-29	PREDICTED: ABC transporter G family member 26-like [Acropora digitifera].	XP_015754524	63.2	2.80E-255	n/a
augustus.g62779.t1	n/a	n/a	n/a	n/a	n/a	n/a	-1.176156091	3.76E-06	PREDICTED: glycerol-3-phosphate dehydrogenase [NAD(+)], cytoplasmic-like isoform X1 [Acropora di	XP_015772267	94.1	1.30E-219	K00006
augustus.g62783.t1	n/a	n/a	1.621253069	0.000146922	n/a	n/a	1.300743288	0.001946232	PREDICTED: juxtaposed with another zinc finger protein 1-like [Acropora digitifera].	XP_015772232	94.2	7.50E-108	K19495
augustus.g62786.t1	n/a	n/a	n/a	n/a	n/a	n/a	2.024948526	4.96E-11	tax1-binding protein 1 homolog B-like [Orbicella faveolata].	XP_020623097	62.1	5.10E-209	K21347
augustus.g62874.t1	n/a	n/a	n/a	n/a	n/a	n/a	1.651563246	0.00812431	DNA-binding protein RFX6-like isoform X1 [Orbicella faveolata].	XP_020608877	81.5	0.00E+00	K19521
augustus.g62977.t1	n/a	n/a	n/a	n/a	n/a	n/a	1.601733505	0.033037762	uncharacterized protein LOC110050596 [Orbicella faveolata].	XP_020612179	38.7	1.60E-99	n/a
augustus.g62992.t1	n/a	n/a	n/a	n/a	n/a	n/a	2.354182845	0.000127958	PREDICTED: fibronectin type III domain-containing protein [Acropora digitifera].	XP_015778146	68.7	1.20E-26	n/a
augustus.g63031.t1	n/a	n/a	2.448644004	0.005373103	n/a	n/a	n/a	n/a	hypothetical protein AC249_AIPGENE10081 [Exaiptasia pallida].	KXJ16402	43.3	1.10E-293	n/a
augustus.g63048.t1	n/a	n/a	n/a	n/a	n/a	n/a	-1.923458516	1.17E-05	PREDICTED: bis(5'-adenosyl)-triphosphatase enpp4-like [Acropora digitifera].	XP_015766471	74.4	7.80E-222	n/a
augustus.g63070.t1	6.76005108	0.002100082	n/a	n/a	n/a	n/a	n/a	n/a	PREDICTED: uncharacterized protein LOC106816918 [Priapulus caudatus].	XP_014677047	41.3	7.30E-74	n/a
augustus.g6317.t1	n/a	n/a	-1.62059429	0.002243228	n/a	n/a	n/a	n/a	PREDICTED: GTP-binding protein Rit1-like [Acropora digitifera].	XP_015766375	96.2	2.30E-106	K07832
augustus.g6318.t1	n/a	n/a	-1.180119599	0.031920644	n/a	n/a	n/a	n/a	uncharacterized protein C3orf38 homolog [Orbicella faveolata].	XP_020614624	72.8	1.50E-119	n/a
augustus.g63271.t1	n/a	n/a	n/a	n/a	n/a	n/a	1.44577484	0.006660802	PREDICTED: tubulin polyglutamylase TTLL6-like [Acropora digitifera].	XP_015777644	66.1	1.10E-250	K16600
augustus.g63297.t1	n/a	n/a	n/a	n/a	n/a	n/a	4.388171532	7.75E-07	ras-like GTP-binding protein RHO isoform X1 [Orbicella faveolata].	XP_020631639	81.2	7.50E-87	K04513
augustus.g63333.t1	n/a	n/a	n/a	n/a	n/a	n/a	3.061233374	8.92E-08	PREDICTED: uncharacterized protein LOC107331434 isoform X1 [Acropora digitifera].	XP_015751499	36.9	4.60E-45	n/a
augustus.g63372.t1	n/a	n/a	n/a	n/a	n/a	n/a	2.214407705	2.88E-06	n/a	n/a	n/a	n/a	n/a
augustus.g63380.t1	n/a	n/a	n/a	n/a	4.415769741	0.004561063	4.183277056	0.001045129	PREDICTED: uncharacterized protein LOC107335421 [Acropora digitifera].	XP_015755935	50.9	9.60E-39	n/a
augustus.g63390.t1	n/a	n/a	n/a	n/a	n/a	n/a	-1.618582216	0.045747621	PREDICTED: protein BTN1-like [Acropora digitifera].	XP_015755877	87.8	6.60E-201	n/a
augustus.g63433.t1	n/a	n/a	n/a	n/a	n/a	n/a	7.209478538	0.00228852	PREDICTED: uncharacterized protein LOC107352444 [Acropora digitifera].	XP_015774265	63.7	7.90E-29	n/a
augustus.g6346.t1	n/a	n/a	n/a	n/a	n/a	n/a	1.188546355	0.005334894	PREDICTED: LOW QUALITY PROTEIN: kinesin-like protein klp-3 [Acropora digitifera].	XP_015756922	79.1	0.00E+00	K10406
augustus.g63467.t1	n/a	n/a	n/a	n/a	n/a	n/a	3.243801915	0.014983422	PREDICTED: protein giant-like [Acropora digitifera].	XP_015762804	42.3	5.30E-29	K09056
augustus.g6347.t1	1.090709746	0.038851532	1.204018284	0.003577723	5.606894979	6.18E-39	6.249402134	1.83E-49	n/a	n/a	n/a	n/a	n/a
augustus.g63475.t1	n/a	n/a	n/a	n/a	n/a	n/a	-4.482649779	0.007421634	uncharacterized protein LOC110043121 [Orbicella faveolata].	XP_020604196	70.9	6.70E-142	n/a
augustus.g63511.t1	n/a	n/a	n/a	n/a	n/a	n/a	1.120288171	0.031007512	TBC1 domain family member 25-like [Orbicella faveolata].	XP_020615641	75	2.20E-252	n/a
augustus.g63588.t1	-20.99400498	0.005109307	n/a	n/a	22.89923375	0.000667808	n/a	n/a	PREDICTED: uncharacterized protein LOC107352627 [Acropora digitifera].	XP_015774422	80.5	1.90E-297	n/a
augustus.g63613.t1	n/a	n/a	3.783246053	0.014257989	n/a	n/a	n/a	n/a	n/a	n/a	n/a	n/a	n/a
augustus.g6365.t1	n/a	n/a	n/a	n/a	n/a	n/a	1.586049447	0.000578788	PREDICTED: transmembrane protein 184B-like [Acropora digitifera].	XP_015750908	90	5.40E-216	n/a
augustus.g63832.t1	n/a	n/a	n/a	n/a	n/a	n/a	1.934576076	0.010722964	PREDICTED: kelch-like protein diablo isoform X1 [Acropora digitifera].	XP_015752682	83.4	1.50E-166	n/a
augustus.g6412.t1	n/a	n/a	n/a	n/a	n/a	n/a	-1.284189351	0.005248239	PREDICTED: guanidinoacetate N-methyltransferase A-like isoform X1 [Acropora digitifera].	XP_015757244	87.1	4.40E-119	K00542
augustus.g64207.t1	n/a	n/a	n/a	n/a	n/a	n/a	1.198222201	0.016086219	PREDICTED: zinc finger protein 474-like [Acropora digitifera].	XP_015765758	68.5	2.00E-180	n/a
augustus.g64233.t1	n/a	n/a	4.504367896	0.003684746	n/a	n/a	n/a	n/a	PREDICTED: major facilitator superfamily domain-containing protein 12-like isoform X2 [Acropora digi	XP_015754089	52.5	6.70E-182	n/a
augustus.g64271.t1	n/a	n/a	n/a	n/a	5.409826827	0.000546096	n/a	n/a	n/a	n/a	n/a	n/a	n/a
augustus.g64316.t1	n/a	n/a	n/a	n/a	n/a	n/a	1.321525978	0.03308548	probable ATP-dependent RNA helicase DDX58 [Orbicella faveolata].	XP_020611351	67.9	0.00E+00	n/a
augustus.g64357.t1	n/a	n/a	n/a	n/a	n/a	n/a	1.114820292	0.000562557	PREDICTED: dnaJ homolog subfamily A member 2-like [Acropora digitifera].	XP_015770572	88.2	2.60E-207	K09503
augustus.g64402.t1	n/a	n/a	2.139566495	0.004581918	n/a	n/a	n/a	n/a	PREDICTED: ras-related and estrogen-regulated growth inhibitor-like isoform X1 [Acropora digitifera].	XP_015754810	85.6	1.90E-127	K07855
augustus.g64406.t1	n/a	n/a	n/a	n/a	-2.340089883	0.041557727	-2.170141468	0.009498916	ras-related and estrogen-regulated growth inhibitor-like [Orbicella faveolata].	XP_020614308	75.9	8.90E-102	K07855
augustus.g64427.t1	n/a	n/a	n/a	n/a	n/a	n/a	1.195759619	0.001212421	max dimerization protein 1-like [Orbicella faveolata].	XP_020607626	91.4	2.90E-81	K09114
augustus.g64474.t1	n/a	n/a	1.099037963	0.021468562	n/a	n/a	n/a	n/a	PREDICTED: arachidonate 5-lipoxygenase-like [Acropora digitifera].	XP_015771914	92.6	0.00E+00	K00461
augustus.g64484.t1	n/a	n/a	n/a	n/a	n/a	n/a	-1.443576664	0.000950865	tubulin alpha-1C chain-like [Orbicella faveolata].	XP_020625030	96.4	3.50E-258	K07374
augustus.g64488.t1	n/a	n/a	n/a	n/a	n/a	n/a	-1.303087438	0.02734931	PREDICTED: LOW QUALITY PROTEIN: nicotinate-nucleotide pyrophosphorylase [carboxylating]-like [	XP_015771920	81.7	7.90E-145	K00767
augustus.g64510.t1	n/a	n/a	n/a	n/a	n/a	n/a	1.563515462	4.88E-09	PREDICTED: zinc transporter ZIP14-like isoform X1 [Acropora digitifera].	XP_015754552	53.6	1.40E-206	K14718
[illegible]	[illegible]	[illegible]	[illegible]	[illegible]	[illegible]	[illegible]	[illegible]	[illegible]	[illegible]	[illegible]	[illegible]	[illegible]	[illegible]

	DESeq2 Output								Blastx Output			
	ATHC1vsATAC1		HTAC1vsATAC1		ATHC6vsATAC6		HTAC6vsATAC6					
SequenceID	Log2FoldChange	pAdj	Log2FoldChange	pAdj	Log2FoldChange	pAdj	Log2FoldChange	pAdj	Blastx Best Hit	AccessionNo	% Identity	Expect Value
adi2mcaRNA11316_R0	n/a	n/a	n/a	n/a	n/a	n/a	-3.489941863	0.01777476	PREDICTED: uncharacterized protein LOC107336922 isoform X2 [Acropora digitifera].	XP_015757495	49.8	8.40E-50
adi2mcaRNA11417_R3	n/a	n/a	n/a	n/a	n/a	n/a	2.397489299	0.017576093	PREDICTED: uncharacterized protein K02A2.6-like [Acropora digitifera].	XP_015757563	90.2	0.00E+00
adi2mcaRNA11417_R9	n/a	n/a	n/a	n/a	n/a	n/a	1.320078149	0.023982573	PREDICTED: uncharacterized protein K02A2.6-like [Acropora digitifera].	XP_015757563	93.2	0.00E+00
adi2mcaRNA11437_R1	n/a	n/a	n/a	n/a	n/a	n/a	-1.040188986	0.035535155	PREDICTED: uncharacterized protein LOC107336976 [Acropora digitifera].	XP_015757568	56.1	4.10E-294
adi2mcaRNA12268_R6	n/a	n/a	n/a	n/a	n/a	n/a	1.68161779	0.048501244	PREDICTED: uncharacterized protein LOC107337644 [Acropora digitifera].	XP_015758342	47.3	5.40E-103
adi2mcaRNA12268_R9	n/a	n/a	n/a	n/a	n/a	n/a	1.362682224	0.00069464	PREDICTED: uncharacterized protein LOC107337644 [Acropora digitifera].	XP_015758342	46.5	3.80E-81
adi2mcaRNA12272_R0	n/a	n/a	n/a	n/a	n/a	n/a	3.550411157	5.82E-06	PREDICTED: uncharacterized protein LOC107337647 [Acropora digitifera].	XP_015758345	59.6	4.20E-275
adi2mcaRNA12316_R2	n/a	n/a	n/a	n/a	n/a	n/a	1.453397117	0.013274591	PREDICTED: uncharacterized protein LOC107337721 [Acropora digitifera].	XP_015758434	79.9	0.00E+00
adi2mcaRNA12355_R2	n/a	n/a	n/a	n/a	n/a	n/a	1.911670177	2.35E-08	PREDICTED: uncharacterized protein LOC107337766 [Acropora digitifera].	XP_015758484	56.3	2.60E-45
adi2mcaRNA12454_R0	n/a	n/a	n/a	n/a	n/a	n/a	2.965093073	0.030050064	PREDICTED: uncharacterized protein LOC107337811 [Acropora digitifera].	XP_015758534	45.1	1.30E-105
adi2mcaRNA13096_R2	n/a	n/a	n/a	n/a	n/a	n/a	-2.877213583	3.13E-06	uncharacterized protein LOC110062965 [Orbicella faveolata].	XP_020625568	77.9	7.80E-252
adi2mcaRNA13977_R3	n/a	n/a	3.536621027	4.25E-05	n/a	n/a	5.931652842	1.97E-15	PREDICTED: uncharacterized protein LOC107339221 isoform X2 [Acropora digitifera].	XP_015759968	59.9	7.20E-79
adi2mcaRNA14437_R2	n/a	n/a	n/a	n/a	n/a	n/a	5.487984422	3.79E-13	PREDICTED: uncharacterized protein LOC107339511 [Acropora digitifera].	XP_015760302	50.8	0.00E+00
adi2mcaRNA14447_R9	n/a	n/a	n/a	n/a	n/a	n/a	1.363861247	0.000479073	PREDICTED: uncharacterized protein LOC107339570 [Acropora digitifera].	XP_015760377	46.7	4.00E-305
adi2mcaRNA15643_R1	n/a	n/a	n/a	n/a	n/a	n/a	1.16263097	0.00141076	n/a	n/a	n/a	n/a
adi2mcaRNA15678_R2	n/a	n/a	n/a	n/a	n/a	n/a	1.209958863	0.003341668	PREDICTED: uncharacterized protein LOC107340633 [Acropora digitifera].	XP_015761485	72.3	1.40E-108
adi2mcaRNA1571_R3	n/a	n/a	n/a	n/a	n/a	n/a	1.493745609	0.002847886	n/a	n/a	n/a	n/a
adi2mcaRNA17088_R3	n/a	n/a	n/a	n/a	n/a	n/a	2.344392576	0.03141441	PREDICTED: uncharacterized protein LOC107341759 [Acropora digitifera].	XP_015762706	69.6	5.50E-159
adi2mcaRNA17126_R1	n/a	n/a	n/a	n/a	n/a	n/a	1.359082298	3.01E-05	n/a	n/a	n/a	n/a
adi2mcaRNA17743_R1	n/a	n/a	n/a	n/a	n/a	n/a	-3.121048206	0.010382042	PREDICTED: uncharacterized protein LOC107342327 [Acropora digitifera].	XP_015763297	64.2	7.80E-114
adi2mcaRNA18203_R3	n/a	n/a	n/a	n/a	n/a	n/a	1.782390158	0.036447846	PREDICTED: uncharacterized protein LOC107342736 [Acropora digitifera].	XP_015763714	74	1.80E-09
adi2mcaRNA18203_R8	n/a	n/a	n/a	n/a	n/a	n/a	1.832013275	2.67E-06	PREDICTED: uncharacterized protein LOC107342736 [Acropora digitifera].	XP_015763714	82.1	2.50E-08
adi2mcaRNA18782_R5	n/a	n/a	n/a	n/a	n/a	n/a	1.422109233	0.003462036	PREDICTED: uncharacterized protein LOC107343216 [Acropora digitifera].	XP_015764269	62.1	6.70E-114
adi2mcaRNA18830_R0	n/a	n/a	n/a	n/a	n/a	n/a	1.479535297	0.001806835	PREDICTED: uncharacterized protein LOC107343290 [Acropora digitifera].	XP_015764344	85	0.00E+00
adi2mcaRNA19051_R8	n/a	n/a	n/a	n/a	n/a	n/a	-1.751604236	0.000939912	PREDICTED: uncharacterized protein LOC107343477, partial [Acropora digitifera].	XP_015764531	47.2	4.00E-53
adi2mcaRNA19534_R0	n/a	n/a	n/a	n/a	n/a	n/a	2.702528113	1.66E-05	PREDICTED: uncharacterized protein LOC107343845 isoform X2 [Acropora digitifera].	XP_015764919	46.8	2.30E-102
adi2mcaRNA2052_R1	n/a	n/a	n/a	n/a	n/a	n/a	1.175409657	0.034086104	uncharacterized protein LOC110060447 [Orbicella faveolata].	XP_020622887	71.4	2.50E-75
adi2mcaRNA21592_R0	n/a	n/a	n/a	n/a	n/a	n/a	1.616029822	0.035133802	PREDICTED: uncharacterized protein LOC107345619 isoform X1 [Acropora digitifera].	XP_015766847	42.1	8.70E-08
adi2mcaRNA21790_R0	-7.276959311	0.010431318	-5.979530983	0.022137823	n/a	n/a	5.977712014	0.005569653	PREDICTED: uncharacterized protein LOC107345791 [Acropora digitifera].	XP_015767027	61	0.00E+00
adi2mcaRNA22574_R1	n/a	n/a	n/a	n/a	n/a	n/a	1.390427831	0.04169384	uncharacterized protein LOC110068603 [Orbicella faveolata].	XP_020631660	39	1.70E-37
adi2mcaRNA23092_R9	n/a	n/a	n/a	n/a	n/a	n/a	-3.195394021	0.014857407	uncharacterized protein LOC110045599 [Orbicella faveolata].	XP_020606857	62.5	2.90E-130
adi2mcaRNA23205_R9	n/a	n/a	n/a	n/a	3.176262595	0.040249816	n/a	n/a	PREDICTED: uncharacterized protein LOC107346956 [Acropora digitifera].	XP_015768311	66.4	2.10E-90
adi2mcaRNA23300_R1	n/a	n/a	n/a	n/a	n/a	n/a	-4.301088976	4.50E-07	uncharacterized protein LOC110058568 [Orbicella faveolata].	XP_020620878	55.8	1.20E-71
adi2mcaRNA23330_R2	n/a	n/a	n/a	n/a	n/a	n/a	1.383897272	0.002554483	PREDICTED: uncharacterized protein LOC107347082 [Acropora digitifera].	XP_015768438	63.8	0.00E+00
adi2mcaRNA24278_R1	n/a	n/a	n/a	n/a	n/a	n/a	-1.699106407	0.001592877	PREDICTED: uncharacterized protein LOC107347831 [Acropora digitifera].	XP_015769290	74.9	9.40E-90
adi2mcaRNA24313_R2	n/a	n/a	n/a	n/a	19.35507178	8.57E-06	21.37951352	3.98E-08	PREDICTED: uncharacterized protein LOC107331445 [Acropora digitifera].	XP_015751521	41.6	3.40E-135
adi2mcaRNA24325_R1	n/a	n/a	n/a	n/a	n/a	n/a	2.829834985	0.00613959	PREDICTED: uncharacterized protein LOC107347846 [Acropora digitifera].	XP_015769306	60.5	5.70E-266
adi2mcaRNA24388_R0	n/a	n/a	n/a	n/a	n/a	n/a	1.087490436	0.000222038	PREDICTED: uncharacterized protein LOC107347927 [Acropora digitifera].	XP_015769405	74.2	0.00E+00
adi2mcaRNA24402_R0	n/a	n/a	n/a	n/a	n/a	n/a	2.200234416	1.04E-06	PREDICTED: uncharacterized protein LOC107347925 [Acropora digitifera].	XP_015769402	40.7	1.30E-125
adi2mcaRNA24544_R0	n/a	n/a	n/a	n/a	n/a	n/a	2.264123586	0.034698062	PREDICTED: uncharacterized protein KIAA0895-like [Acropora digitifera].	XP_015769523	79	7.90E-196
adi2mcaRNA24767_R3	n/a	n/a	n/a	n/a	n/a	n/a	-1.120179392	0.004484424	PREDICTED: uncharacterized protein LOC107348226 [Acropora digitifera].	XP_015769738	70.7	0.00E+00
adi2mcaRNA2527_R0	n/a	n/a	n/a	n/a	n/a	n/a	1.925704543	0.003031	uncharacterized protein LOC110062166, partial [Orbicella faveolata].	XP_020624685	54.5	4.70E-137
adi2mcaRNA25674_R1	n/a	n/a	n/a	n/a	n/a	n/a	1.244495149	0.001095897	PREDICTED: uncharacterized protein LOC107348928 isoform X1 [Acropora digitifera].	XP_015770501	52.1	2.20E-130
adi2mcaRNA26194_R7	n/a	n/a	1.275182036	3.29E-05	n/a	n/a	1.308887754	3.19E-06	PREDICTED: uncharacterized protein K02A2.6-like [Acropora digitifera].	XP_015770983	77.7	0.00E+00
adi2mcaRNA26837_R1	n/a	0.004435025	4.716943536	0.002853704	n/a	n/a	n/a	n/a	PREDICTED: uncharacterized protein LOC107349886 isoform X4 [Acropora digitifera].	XP_015771564	51.8	0.00E+00
adi2mcaRNA2707_R0	n/a	n/a	n/a	n/a	n/a	n/a	1.629931912	1.40E-11	PREDICTED: uncharacterized protein LOC107350039 isoform X1 [Acropora digitifera].	XP_015771739	75	0.00E+00
adi2mcaRNA27095_R1	n/a	n/a	n/a	n/a	n/a	n/a	-1.359885739	0.003907071	PREDICTED: uncharacterized protein LOC107350112 [Acropora digitifera].	XP_015771827	80	1.80E-243
adi2mcaRNA27289_R1	n/a	n/a	n/a	n/a	n/a	n/a	2.724007083	0.000740171	PREDICTED: uncharacterized protein LOC107350246 [Acropora digitifera].	XP_015771954	70.8	1.30E-143
adi2mcaRNA27676_R8	n/a	n/a	n/a	n/a	n/a	n/a	1.206551273	2.66E-05	PREDICTED: uncharacterized protein LOC107350589 isoform X2 [Acropora digitifera].	XP_015772318	41.7	8.50E-149
adi2mcaRNA2771_R0	n/a	n/a	n/a	n/a	n/a	n/a	4.043951436	0.014933111	PREDICTED: uncharacterized protein LOC107350816 isoform X1 [Acropora digitifera].	XP_015772554	73.2	9.40E-79
adi2mcaRNA28534_R0	n/a	n/a	n/a	n/a	n/a	n/a	2.187129698	0.001368862	PREDICTED: uncharacterized protein K02A2.6-like [Acropora digitifera].	XP_015773068	81.1	3.70E-279
adi2mcaRNA28536_R2	n/a	n/a	n/a	n/a	n/a	n/a	2.233257403	0.000564818	PREDICTED: uncharacterized protein LOC107351301 [Acropora digitifera].	XP_015773076	70.7	1.90E-46
adi2mcaRNA28940_R9	n/a	n/a	n/a	n/a	3.228796643	0.021753282	3.071223844	0.004111062	uncharacterized protein LOC110063612 [Orbicella faveolata].	XP_020626262	65.1	1.30E-88
adi2mcaRNA29396_R0	n/a	n/a	n/a	n/a	n/a	n/a	3.14628742	2.87E-11	PREDICTED: uncharacterized protein LOC107352037 [Acropora digitifera].	XP_015773825	40	1.60E-79
adi2mcaRNA29984_R6	n/a	n/a	n/a	n/a	n/a	n/a	1.809878715	0.021373007	n/a	n/a	n/a	n/a
adi2mcaRNA30791_R0	n/a	n/a	n/a	n/a	n/a	n/a	1.326473865	6.96E-06	PREDICTED: uncharacterized protein LOC107353273 [Acropora digitifera].	XP_015775087	67	6.10E-151
adi2mcaRNA31092_R7	n/a	n/a	n/a	n/a	n/a	n/a	2.849617883	0.000266172	PREDICTED: uncharacterized protein LOC107353532 [Acropora digitifera].	XP_015775335	60.2	4.20E-47
adi2mcaRNA31525_R2	n/a	n/a	n/a	n/a	n/a	n/a	1.594247574	1.42E-05	PREDICTED: uncharacterized protein DDB_G0271670-like isoform X1 [Acropora digitifera].	XP_015775750	60.3	2.00E-37
adi2mcaRNA31527_R7	n/a	n/a	n/a	n/a	n/a	n/a	1.284984738	0.042921591	PREDICTED: uncharacterized protein LOC107353873 isoform X1 [Acropora digitifera].	XP_015775748	44.4	7.50E-146
adi2mcaRNA31538_R0	n/a	n/a	n/a	n/a	n/a	n/a	2.265429793	0.023751793	PREDICTED: uncharacterized protein LOC107353887 [Acropora digitifera].	XP_015775762	67.1	1.70E-89
adi2mcaRNA31559_R0	n/a	n/a	n/a	n/a	n/a	n/a	1.30545103	0.013163515	PREDICTED: uncharacterized protein LOC107353909 [Acropora digitifera].	XP_015775787	47.5	0.00E+00
adi2mcaRNA31989_R3	n/a	n/a	n/a	n/a	n/a	n/a	1.878488936	0.036447846	PREDICTED: uncharacterized protein LOC107327586 isoform X2 [Acropora digitifera].	XP_015747817	53.5	7.10E-86
[illegible]	[illegible]	[illegible]	[illegible]	[illegible]	[illegible]	[illegible]	[illegible]	[illegible]	[illegible]	[illegible]	[illegible]	[illegible]

augustus.g54400.t1	n/a	n/a	1.310928395	3.90E-05	n/a	n/a	n/a	n/a	uncharacterized protein LOC110047797 [Orbicella faveolata].	XP_020609197	76.5
augustus.g54909.t1	n/a	n/a	n/a	n/a	n/a	n/a	4.139537096	6.23E-08	PREDICTED: uncharacterized protein LOC107331806, partial [Acropora digitifera].	XP_015751937	62.1
augustus.g54910.t1	n/a	n/a	n/a	n/a	n/a	n/a	2.41366925	0.0350766	PREDICTED: uncharacterized protein LOC107331806, partial [Acropora digitifera].	XP_015751937	60.5
augustus.g54946.t1	n/a	n/a	n/a	n/a	n/a	n/a	-1.885553876	0.004305286	PREDICTED: uncharacterized protein LOC107352889 [Acropora digitifera].	XP_015774696	62.7
augustus.g54976.t1	n/a	n/a	n/a	n/a	n/a	n/a	1.41494271	0.039020701	uncharacterized protein LOC110046083 [Orbicella faveolata].	XP_020607413	57.8
augustus.g54979.t1	n/a	n/a	n/a	n/a	n/a	n/a	1.993482316	4.19E-14	PREDICTED: uncharacterized protein LOC107355947 [Acropora digitifera].	XP_015778047	52.7
augustus.g55135.t1	n/a	n/a	n/a	n/a	n/a	n/a	1.950567861	0.000914434	uncharacterized protein LOC110047052 isoform X4 [Orbicella faveolata].	XP_020608445	42.9
augustus.g55286.t1	n/a	n/a	n/a	n/a	n/a	n/a	1.907550198	0.005166944	uncharacterized protein LOC110040221 [Orbicella faveolata].	XP_020601085	38.7
augustus.g55428.t1	n/a	n/a	n/a	n/a	n/a	n/a	-1.753830601	0.001592877	hypothetical protein AC249_AIPGENE26984 [Exaiptasia pallida].	KXJ29425	66
augustus.g55481.t1	n/a	n/a	n/a	n/a	4.653666971	0.023366833	n/a	n/a	uncharacterized protein LOC110041271, partial [Orbicella faveolata].	XP_020602214	69.3
augustus.g55546.t1	n/a	n/a	3.869295101	0.036552144	n/a	n/a	n/a	n/a	PREDICTED: uncharacterized protein LOC107355139 [Acropora digitifera].	XP_015777145	63
augustus.g55639.t1	n/a	n/a	n/a	n/a	n/a	n/a	-1.870226019	2.50E-07	PREDICTED: uncharacterized protein LOC107337478 [Acropora digitifera].	XP_015758124	56.2
augustus.g5571.t1	n/a	n/a	n/a	n/a	n/a	n/a	1.554629346	0.005360509	n/a	n/a	n/a
augustus.g55712.t1	n/a	n/a	n/a	n/a	n/a	n/a	2.333343723	0.049423956	uncharacterized protein C4orf45 homolog [Orbicella faveolata].	XP_020629295	74.6
augustus.g55852.t1	n/a	n/a	-1.723120087	0.027581094	n/a	n/a	n/a	n/a	PREDICTED: uncharacterized protein LOC107347066 isoform X1 [Acropora digitifera].	XP_015768415	44.1
augustus.g55868.t1	n/a	n/a	n/a	n/a	n/a	n/a	-2.701587106	0.014550545	PREDICTED: uncharacterized protein LOC107357809 [Acropora digitifera].	XP_015779933	93.4
augustus.g55963.t1	n/a	n/a	n/a	n/a	n/a	n/a	5.900504604	2.31E-12	uncharacterized protein LOC110067417 isoform X3 [Orbicella faveolata].	XP_020630401	46.3
augustus.g55984.t1	n/a	n/a	n/a	n/a	n/a	n/a	-1.538802308	0.022321246	hypothetical protein [Gilvimarinus polysaccharolyticus].	WP_049722681	35.8
augustus.g5614.t1	n/a	n/a	n/a	n/a	n/a	n/a	-2.165532691	0.000248939	hypothetical protein NEMVEDRAFT_v1g222427 [Nematostella vectensis].	XP_001621044	69.1
augustus.g56153.t1	n/a	n/a	n/a	n/a	n/a	n/a	1.123074399	0.009036679	PREDICTED: uncharacterized protein LOC107344017 isoform X2 [Acropora digitifera].	XP_015765102	43.3
augustus.g56492.t1	n/a	n/a	n/a	n/a	n/a	n/a	-2.117368046	0.019588755	PREDICTED: uncharacterized protein LOC107336613 [Acropora digitifera].	XP_015757162	64.2
augustus.g56845.t1	n/a	n/a	n/a	n/a	n/a	n/a	-2.227730751	0.00126489	uncharacterized protein LOC110066382 [Orbicella faveolata].	XP_020629268	49.5
augustus.g57157.t1	n/a	n/a	n/a	n/a	n/a	n/a	-1.345400954	0.036467331	n/a	n/a	n/a
augustus.g57225.t1	n/a	n/a	-6.848726494	0.027185306	n/a	n/a	n/a	n/a	n/a	n/a	n/a
augustus.g58316.t1	n/a	n/a	n/a	n/a	35.91277592	1.54E-14	32.47624076	1.86E-12	PREDICTED: uncharacterized protein LOC107336256 [Acropora digitifera].	XP_015756810	74.8
augustus.g58392.t1	n/a	n/a	n/a	n/a	n/a	n/a	1.115702206	0.034640649	PREDICTED: uncharacterized protein LOC107350452 [Acropora digitifera].	XP_015772165	78.7
augustus.g58508.t1	n/a	n/a	n/a	n/a	n/a	n/a	-1.073124964	0.000753214	PREDICTED: uncharacterized protein LOC107340577 isoform X2 [Acropora digitifera].	XP_015761424	53.7
augustus.g58538.t1	n/a	n/a	n/a	n/a	n/a	n/a	4.915318046	3.66E-05	n/a	n/a	n/a
augustus.g58613.t1	n/a	n/a	2.482850335	0.003369687	n/a	n/a	n/a	n/a	n/a	n/a	n/a
augustus.g58663.t1	n/a	n/a	n/a	n/a	n/a	n/a	6.506301602	0.003907071	PREDICTED: uncharacterized protein LOC107345799 [Acropora digitifera].	XP_015767034	62
augustus.g58834.t1	n/a	n/a	n/a	n/a	n/a	n/a	-6.037906221	0.023603834	uncharacterized protein LOC110041904 [Orbicella faveolata].	XP_020602893	46.1
augustus.g58863.t1	n/a	n/a	2.37289729	0.002542162	n/a	n/a	1.573196796	0.043976697	PREDICTED: uncharacterized protein LOC107345411 [Acropora digitifera].	XP_015766599	62
augustus.g594.t1	n/a	n/a	1.444290959	0.043843388	n/a	n/a	n/a	n/a	PREDICTED: uncharacterized protein LOC107337760 [Acropora digitifera].	XP_015758476	77.2
augustus.g59463.t1	n/a	n/a	n/a	n/a	n/a	n/a	-7.13317657	0.000561725	hypothetical protein AC249_AIPGENE17291 [Exaiptasia pallida].	KXJ10011	69.4
augustus.g59670.t1	n/a	0.002080881	n/a	n/a	n/a	n/a	n/a	n/a	uncharacterized protein LOC110055113 isoform X2 [Orbicella faveolata].	XP_020617153	59.3
augustus.g59731.t1	n/a	n/a	n/a	n/a	n/a	n/a	2.970620076	0.042700347	Uncharacterized protein K02A2.6 [Exaiptasia pallida].	KXJ17167	61.1
augustus.g59938.t1	n/a	n/a	n/a	n/a	n/a	n/a	-1.831260333	0.018021582	n/a	n/a	n/a
augustus.g60042.t1	n/a	n/a	n/a	n/a	n/a	n/a	1.608823056	4.21E-05	uncharacterized protein LOC110066933 [Orbicella faveolata].	XP_020629849	87.4
augustus.g60119.t1	n/a	n/a	n/a	n/a	n/a	n/a	-4.316818142	0.000178714	PREDICTED: uncharacterized protein LOC107353686 isoform X4 [Acropora digitifera].	XP_015775518	56.5
augustus.g60582.t1	n/a	n/a	n/a	n/a	n/a	n/a	-1.226524144	0.005102056	PREDICTED: uncharacterized protein LOC107341272 [Acropora digitifera].	XP_015762159	44.1
augustus.g60790.t1	n/a	n/a	n/a	n/a	n/a	n/a	-3.725692943	0.040980788	PREDICTED: uncharacterized protein LOC107351542 [Acropora digitifera].	XP_015773320	53.8
augustus.g60891.t1	n/a	n/a	1.609969063	0.007016719	n/a	n/a	n/a	n/a	n/a	n/a	n/a
augustus.g60912.t1	n/a	n/a	n/a	n/a	n/a	n/a	-1.053591088	0.011976292	PREDICTED: uncharacterized protein LOC107348074 [Acropora digitifera].	XP_015769569	88.6
augustus.g61027.t1	n/a	n/a	n/a	n/a	5.853343293	0.030955219	5.490509607	0.007276345	PREDICTED: uncharacterized protein LOC107353173 [Acropora digitifera].	XP_015774979	62.3
augustus.g61114.t1	n/a	0.004577138	n/a	n/a	n/a	n/a	n/a	n/a	n/a	n/a	n/a
augustus.g61706.t1	n/a	n/a	n/a	n/a	n/a	n/a	1.840412501	0.000247629	PREDICTED: uncharacterized protein LOC107336709 isoform X2 [Acropora digitifera].	XP_015757261	47.4
augustus.g62052.t1	n/a	0.008305377	-5.637045934	0.003684746	n/a	n/a	n/a	n/a	uncharacterized protein LOC110060551 isoform X1 [Orbicella faveolata].	XP_020622997	64.4
augustus.g62089.t1	n/a	n/a	-2.342894957	0.01719565	n/a	n/a	-2.597125025	0.001317802	uncharacterized protein LOC110047759 [Orbicella faveolata].	XP_020609165	55.2
augustus.g62090.t1	n/a	n/a	2.123517796	0.04175215	n/a	n/a	n/a	n/a	PREDICTED: uncharacterized protein LOC107354720 [Acropora digitifera].	XP_015776692	69.8
augustus.g62977.t1	n/a	n/a	n/a	n/a	n/a	n/a	1.601733505	0.033037762	uncharacterized protein LOC110050596 [Orbicella faveolata].	XP_020612179	38.7
augustus.g63031.t1	n/a	n/a	2.448644004	0.005373103	n/a	n/a	n/a	n/a	hypothetical protein AC249_AIPGENE10081 [Exaiptasia pallida].	KXJ16402	43.3
augustus.g63070.t1	n/a	0.002100082	n/a	n/a	n/a	n/a	n/a	n/a	PREDICTED: uncharacterized protein LOC106816918 [Priapulus caudatus].	XP_014677047	41.3
augustus.g6318.t1	n/a	n/a	-1.180119599	0.031920644	n/a	n/a	n/a	n/a	uncharacterized protein C3orf38 homolog [Orbicella faveolata].	XP_020614624	72.8
augustus.g63372.t1	n/a	n/a	n/a	n/a	n/a	n/a	2.214407705	2.88E-06	n/a	n/a	n/a
augustus.g63380.t1	n/a	n/a	n/a	n/a	4.415769741	0.004561063	4.183277056	0.001045129	PREDICTED: uncharacterized protein LOC107335421 [Acropora digitifera].	XP_015755935	50.9
augustus.g6347.t1	1.090709746	0.038851532	1.204018284	0.003577723	5.606894979	6.18E-39	6.249402134	1.83E-49	n/a	n/a	n/a
augustus.g63475.t1	n/a	n/a	n/a	n/a	n/a	n/a	-4.482649779	0.007421634	uncharacterized protein LOC110043121 [Orbicella faveolata].	XP_020604196	70.9
augustus.g63588.t1	n/a	0.005109307	n/a	n/a	22.89923375	0.000667808	n/a	n/a	PREDICTED: uncharacterized protein LOC107352627 [Acropora digitifera].	XP_015774422	80.5
augustus.g64271.t1	n/a	n/a	n/a	n/a	5.409826827	0.000546096	n/a	n/a	n/a	n/a	n/a
augustus.g64662.t1	n/a	n/a	n/a	n/a	n/a	n/a	-5.743996602	0.004244204	PREDICTED: uncharacterized protein LOC107355036 [Acropora digitifera].	XP_015777038	56.9
augustus.g64756.t1	n/a	n/a	n/a	n/a	n/a	n/a	2.777820196	0.001737903	Uncharacterized protein K02A2.6 [Exaiptasia pallida].	KXJ17167	66.3
augustus.g64798.t1	n/a	0.010385758	n/a	n/a	n/a	n/a	n/a	n/a	PREDICTED: uncharacterized protein LOC107339408 isoform X1 [Acropora digitifera].	XP_015760172	92.7
augustus.g64957.t1	n/a	n/a	n/a	n/a	n/a	n/a	2.028769149	5.28E-07	PREDICTED: uncharacterized protein K02A2.6-like [Acropora digitifera].	XP_015778408	61.1
augustus.g64963.t1	n/a	n/a	n/a	n/a	n/a	n/a	2.296734123	0.003374319	uncharacterized protein LOC110043447 [Orbicella faveolata].	XP_020604556	45.4
augustus.g65285.t1	n/a	n/a	n/a	n/a	n/a	n/a	2.39246734	0.011564862	hypothetical protein AC249_AIPGENE8873 [Exaiptasia pallida].	KXJ21423	49.3
augustus.g65382.t1	n/a	n/a	3.93015725	0.006179017	n/a	n/a	n/a	n/a	PREDICTED: uncharacterized protein LOC107349711 [Acropora digitifera].	XP_015771383	74.8
augustus.g6543.t1	n/a	n/a	n/a	n/a	n/a	n/a	-1.387451739	0.003907071	n/a	n/a	n/a
augustus.g66104.t1	n/a	n/a	n/a	n/a	n/a	n/a	5.049769608	0.01705339	PREDICTED: uncharacterized protein LOC107349369 isoform X1 [Acropora digitifera].	XP_015770999	69.1
augustus.g66116.t1	n/a	n/a	2.205923855	0.044548323	n/a	n/a	3.707437809	1.03E-06	PREDICTED: uncharacterized protein LOC107339511 [Acropora digitifera].	XP_015760302	56
augustus.g66176.t1	n/a	n/a	n/a	n/a	n/a	n/a	1.410826281	6.29E-05	PREDICTED: uncharacterized protein LOC107334767 isoform X1 [Acropora digitifera].	XP_015755223	63.4
augustus.g66760.t1	n/a	n/a	n/a	n/a	-21.19176309	3.49E-07	n/a	n/a	PREDICTED: uncharacterized protein LOC107347066 isoform X1 [Acropora digitifera].	XP_015768415	44.6
augustus.g6677.t1	n/a	n/a	n/a	n/a	n/a	n/a	2.533076253	5.84E-05	uncharacterized protein LOC110053951 [Orbicella faveolata].	XP_020615931	55
augustus.g66885.t1	n/a	n/a	n/a	n/a	n/a	n/a	5.59239947	1.24E-05	n/a	n/a	n/a
augustus.g67045.t1	n/a	n/a	1.602050533	0.0011549	n/a	n/a	1.915995941	5.40E-06	PREDICTED: uncharacterized protein LOC107353619 [Acropora digitifera].	XP_015775440	73.9
augustus.g6729.t1	n/a	n/a	n/a	n/a	n/a	n/a	2.452225067	0.038137588	uncharacterized protein LOC110060862 [Orbicella faveolata].	XP_020623331	50.1
augustus.g67292.t1	n/a	n/a	n/a	n/a	n/a	n/a	-3.448626736	0.02832932	uncharacterized protein LOC110040103 [Orbicella faveolata].	XP_020600952	54.1
augustus.g67505.t1	n/a	n/a	n/a	n/a	n/a	n/a	-1.221388887	0.028621716	n/a	n/a	n/a
augustus.g67588.t1	n/a	n/a	n/a	n/a	n/a	n/a	1.564692447	0.014098878	hypothetical protein AC249_AIPGENE3237 [Exaiptasia pallida].	KXJ10339	30.4
augustus.g67778.t1	n/a	n/a	1.066805222	0.034001516	n/a	n/a	n/a	n/a	uncharacterized protein LOC110068761 isoform X1 [Orbicella faveolata].	XP_020631826	61.3
augustus.g67839.t1	n/a	0.048640771	n/a	n/a	n/a	n/a	n/a	n/a	n/a	n/a	n/a
augustus.g67922.t1	n/a	n/a	n/a	n/a	n/a	n/a	1.390594758	0.007554808	uncharacterized protein LOC110044793 [Orbicella faveolata].	XP_020606039	75.5
augustus.g67932.t1	2.764043406	2.05E-20	2.985066278	1.97E-24	2.315592453	4.92E-22	2.90344522	1.43E-36	PREDICTED: uncharacterized protein LOC107336247 [Acropora digitifera].	XP_015756799	63
augustus.g68009.t1	n/a	n/a	n/a	n/a	n/a	n/a	-1.246947337	0.018063012	PREDICTED: uncharacterized protein LOC107335893 [Acropora digitifera].	XP_015756433	55.1
augustus.g68640.t1	n/a	n/a	n/a	n/a	n/a	n/a	2.068728428	0.016448298	PREDICTED: uncharacterized protein LOC107352156 [Acropora digitifera].	XP_015773985	46.8
augustus.g68735.t1	n/a	7.96E-13	n/a	n/a	n/a	n/a	n/a	n/a	n/a	n/a	n/a
augustus.g68893.t1	n/a	n/a	2.675810888	3.11E-05	n/a	n/a	n/a	n/a	PREDICTED: uncharacterized protein LOC107348045 isoform X1 [Acropora digitifera].	XP_015769531	72.2
augustus.g69690.t1	n/a	n/a	-2.204204418	0.003684746	n/a	n/a	n/a	n/a	uncharacterized protein LOC110046761 [Orbicella faveolata].	XP_020608126	51.5
augustus.g69814.t1	n/a	n/a	n/a	n/a	n/a	n/a	-1.694429927	0.033639034	PREDICTED: uncharacterized protein LOC107327612 [Acropora digitifera].	XP_015747841	65.4
augustus.g6990.t1	n/a	n/a	n/a	n/a	n/a	n/a	8.237542565	0.00553255	hypothetical protein AC249_AIPGENE10767 [Exaiptasia pallida].	KXJ08542	55.8
augustus.g69963.t1	n/a	0.000522683	n/a	n/a	n/a	n/a	n/a	n/a	PREDICTED: uncharacterized protein LOC107327932 [Acropora digitifera].	XP_015748140	72.1
augustus.g70041.t1	n/a	n/a	n/a	n/a	-10.03066875	2.12E-08	-6.50347339	4.60E-07	uncharacterized protein LOC110040364 [Orbicella faveolata].	XP_020601248	34.2
augustus.g70042.t1	n/a	n/a	n/a	n/a	n/a	n/a	-4.08143659	0.004385034	uncharacterized protein LOC110040364 [Orbicella faveolata].	XP_020601248	35.5
augustus.g70046.t1	n/a	n/a	n/a	n/a	n/a	n/a	3.018067128	7.89E-14	PREDICTED: uncharacterized protein LOC107342779 [Acropora digitifera].	XP_015763773	46.4
augustus.g70209.t1	n/a	n/a	n/a	n/a	n/a	n/a	-2.623081744	0.004207819	n/a	n/a	n/a
augustus.g70589.t1	n/a	n/a	n/a	n/a	n/a	n/a	1.298295765	0.026157956	uncharacterized protein LOC110044400 isoform X1 [Orbicella faveolata].	XP_020605601	85.3
augustus.g70620.t1	n/a	n/a	n/a	n/a	n/a	n/a	-1.012451673	0.017312999	PREDICTED: uncharacterized protein LOC107354862 [Acropora digitifera].	XP_015776841	59.4
augustus.g70746.t1	n/a	n/a	n/a	n/a	1.193280793	0.021227623	n/a	n/a	LOW QUALITY PROTEIN: uncharacterized protein LOC110055107 [Orbicella faveolata].	XP_020617148	39.9
augustus.g71175.t1	n/a	n/a	n/a	n/a	-25.30744412	5.76E-07	n/a	n/a	PREDICTED: uncharacterized protein LOC107339511 [Acropora digitifera].	XP_015760302	60.1
augustus.g71273.t1	n/a	n/a	n/a	n/a	n/a	n/a	11.12715832	0.013003663	PREDICTED: uncharacterized protein LOC107348470 [Acropora digitifera].	XP_015769996	90.7
augustus.g71453.t1	n/a	n/a	n/a	n/a	n/a	n/a	4.359149022	0.001071719	uncharacterized protein LOC110059905 [Orbicella faveolata].	XP_020622278	47.7
augustus.g7876.t1	n/a	n/a	1.381524629	0.037614585	n/a	n/a	n/a	n/a	PREDICTED: uncharacterized protein LOC107342710 isoform X1 [Acropora digitifera].	XP_015763691	80.2
augustus.g7912.t1	n/a	n/a	n/a	n/a	n/a	n/a	-1.228546115	0.049833184	n/a	n/a	n/a
augustus.g7925.t1	n/a	n/a	1.589052116	0.020478647	n/a	n/a	n/a	n/a	PREDICTED: uncharacterized protein LOC107346118 [Acropora digitifera].	XP_015767376	94.1
augustus.g8017.t1	n/a	n/a	n/a	n/a	n/a	n/a	2.175846411	0.0230895	uncharacterized protein LOC110039631 [Orbicella faveolata].	XP_020600419	60
augustus.g802.t1	n/a	n/a	n/a	n/a	n/a	n/a	7.15916302	0.027077481	PREDICTED: uncharacterized protein LOC107356355 [Acropora digitifera].	XP_015778462	77.3
augustus.g831.t1	n/a	n/a	n/a	n/a	n/a	n/a	1.203440486	0.000750463	PREDICTED: uncharacterized protein LOC107341038 isoform X1 [Acropora digitifera].	XP_015761939	72.3
augustus.g8391.t1	n/a	0.01388984	n/a	n/a	n/a	n/a	n/a	n/a	uncharacterized protein LOC110059021 isoform X2 [Orbicella faveolata].	XP_020621348	53.4
augustus.g8584.t1	n/a	n/a	n/a	n/a	-9.38538974	3.80E-05	n/a	n/a	PREDICTED: uncharacterized protein LOC107336613 [Acropora digitifera].	XP_015757162	48.5
augustus.g8907.t1	n/a	n/a	n/a	n/a	n/a	n/a	1.322200481	0.024640174	uncharacterized protein LOC110069738 [Orbicella faveolata].	XP_020632939	74.2
augustus.g9087.t1	n/a	n/a	4.255659688	0.038828213	n/a	n/a	n/a	n/a	PREDICTED: uncharacterized protein LOC107344933 [Acropora digitifera].	XP_015766105	86.9
augustus.g9143.t1	n/a	1.14E-08	n/a	n/a	n/a	n/a	n/a	n/a	uncharacterized protein LOC110061494, partial [Orbicella faveolata].	XP_020623987	41.3
augustus.g9150.t1	n/a	0.00911408	n/a	n/a	n/a	n/a	n/a	n/a	PREDICTED: uncharacterized protein LOC107358860 [Acropora digitifera].	XP_015780918	41.9
augustus.g9204.t1	n/a	0.044290334	n/a	n/a	n/a	n/a	n/a	n/a	uncharacterized protein LOC110048960 isoform X1 [Orbicella faveolata].	XP_020610379	71.8
augustus.g9284.t1	n/a	n/a	n/a	n/a	n/a	n/a	4.092388991	0.016099704	uncharacterized protein LOC110054167 [Orbicella faveolata].	XP_020616160	91.3
augustus.g9559.t1	n/a	n/a	n/a	n/a	n/a	n/a	-4.329726964	5.42E-05	hypothetical protein LOTGIDRAFT_174919 [Lottia gigantea].	XP_009053041	39.8
augustus.g9726.t1	n/a	n/a	n/a	n/a	-2.79181873	0.001468259	n/a	n/a	uncharacterized protein LOC110052694 [Orbicella faveolata].	XP_020614507	44.4
augustus.g9784.t1	n/a	n/a	n/a	n/a	n/a	n/a	1.154727435	0.000656634	PREDICTED: uncharacterized protein LOC107340806 [Acropora digitifera].	XP_015761662	61.2
augustus.g9785.t1	n/a	n/a	-6.165016762	0.001536566	n/a	n/a	n/a	n/a	PREDICTED: uncharacterized protein LOC107353091 [Acropora digitifera].	XP_015774893	58.2
augustus.g9905.t1	n/a	n/a	n/a	n/a	7.731151509	0.030867669	7.876189663	0.002526924	PREDICTED: uncharacterized protein K02A2.6-like [Austrofundulus limnaeus].	XP_013882267	38

Treatment	Temperature (˚C)	
	Time 1h	*Time 6h*
ATAC	26.87 ± 0.01	27.14 ± 0.06
ATHC	26.93 ± 0.01	27.33 ± 0.08
HTAC	28.67 ± 0.01	29.05 ± 0.08

Table S4. Test of diversifying selection in *M. capitata*.

(A) The top 1,220 (10%) proteins with the highest dN values that were selected and annotated prior to Gene Ontology (GO) assignment and placement in broader KEGG pathway maps;
(B) the largest number KO terms from the fast-evolving gene set (23.5% by number, excluding "Global and overview maps") that were assigned to pathways of the "Signal transduction" class;
(C) the ten over-represented GO terms in our test set that were identified using Fisher's exact test.

Acropora / *Montipora* Proteins in OG	KaKs	Ka	Top .10 Ka	Annotation	# GO terms	GO terms	GO Desciptions	Enzyme Codes
Acrdi.XM_015918993.1/1-825 | Monca.augustus.g47398.t1/1-825	0.567853	0.852175	Y	PREDICTED: uncharacterized protein LOC107352660				
Acrdi.XM_015903117.1/1-519 | Monca.augustus.g12313.t1/1-699	0.509755	0.823354	Y	PREDICTED: uncharacterized protein LOC107337883				
Acrdi.XM_015900042.1/1-729 | Monca.augustus.g43403.t1/1-324	0.471053	0.821616	Y	skeletal aspartic acid-rich 2-like	2	C:GO:0016020; C:GO:0016021	C:membrane; C:integral component of membrane	
Acrdi.XM_015914770.1/1-474 | Monca.augustus.g47153.t1/1-777	0.477467	0.798535	Y	Actin-depolymerizing factor 1	4	F:GO:0003779; P:GO:0030042; C:GO:0015629; C:GO:0005622	F:actin binding; P:actin filament depolymerization; C:actin cytoskeleton; C:intracellular	
Acrdi.XM_015899959.1/1-2385 | Monca.augustus.g44153.t1/1-3921	1.13575	0.787055	Y	PREDICTED: trichohyalin-like				
Acrdi.XM_015901572.1/1-810 | Monca.augustus.g13880.t1/1-720	0.435851	0.782083	Y	RNA-directed DNA polymerase from transposon BS				
Acrdi.XM_015896270.1/1-672 | Monca.augustus.g6795.t1/1-699	0.445838	0.767701	Y	PREDICTED: uncharacterized protein LOC107331660	2	C:GO:0016020; C:GO:0016021	C:membrane; C:integral component of membrane	
Acrdi.XM_015907334.1/1-1227 | Monca.augustus.g57763.t1/1-1524	0.40593	0.762328	Y	cytochrome P450 monooxygenase	9	F:GO:0005506; F:GO:0046872; F:GO:0016705; F:GO:0016491;	F:iron ion binding; F:metal ion binding; F:oxidoreductase activity, acting on paired donors, with incorporation or reduction of molecular oxygen; F:oxidoreductase activity; C:membrane; C:integral component of membrane; P:oxidation-reduction process; F:monooxygenase activity; F:heme binding	
Acrdi.XM_015897099.1/1-1164 | Monca.augustus.g48375.t1/1-1032	0.424918	0.74706	Y	S-antigen -like isoform X5				
Acrdi.XM_015904234.1/1-1224 | Monca.augustus.g62128.t1/1-840	0.392863	0.741345	Y	PREDICTED: nucleolin-like				
Acrdi.XM_015902302.1/1-1977 | Monca.augustus.g35083.t1/1-12756	0.449352	0.7398	Y	Hep_Hag family				
Acrdi.XM_015917340.1/1-459 | Monca.augustus.g67456.t1/1-480	0.399698	0.736565	Y	Caveolin-1	9	C:GO:0005901; P:GO:0070836; C:GO:0005768; F:GO:0005515;	C:caveola; P:caveola assembly; C:endosome; F:protein binding; P:T cell costimulation; C:integral component of membrane; C:Golgi membrane; P:receptor internalization; P:negative regulation of transforming growth factor beta receptor signaling pathway	
Acrdi.XM_015910839.1/1-993 | Monca.augustus.g57203.t1/1-1455	0.375927	0.730553	Y	Hsp70-binding 1	2	P:GO:0043086; F:GO:0004857	P:negative regulation of catalytic activity; F:enzyme inhibitor activity	
Acrdi.XM_015905205.1/1-627 | Monca.augustus.g67741.t1/1-552	0.370477	0.717778	Y	cyclin-dependent kinase inhibitor 1B-like	1	P:GO:0050794	P:regulation of cellular process	
Acrdi.XM_015919227.1/1-1530 | Monca.augustus.g52631.t1/1-1284	0.391271	0.71687	Y	aspartic and glutamic acid-rich				
Acrdi.XM_015918528.1/1-1212 | Monca.augustus.g36290.t1/1-1350	0.525139	0.715162	Y	PREDICTED: uncharacterized protein LOC107352192				
Acrdi.XM_015906736.1/1-444 | Monca.adi2mcaRNA16516_R0/1-351	0.356653	0.71351	Y	pre-rRNA processing FTSJ3-like	7	P:GO:0000463; F:GO:0008650; C:GO:0005730; P:GO:0000466;	[illegible]	
Acrdi.XM_015919326.1/1-768 | Monca.augustus.g15442.t1/1-777	0.523926	0.709268	Y	PREDICTED: uncharacterized protein LOC107353002				
Acrdi.XM_015892461.1/1-1845 | Monca.augustus.g61307.t1/1-2079	0.359728	0.708354	Y	starmaker-like isoform X2				
Acrdi.XM_015913848.1/1-579 | Monca.adi2mcaRNA24330_R2/1-615	0.352341	0.705117	Y	galectin-3-binding -like	3	C:GO:0016020; F:GO:0005044; P:GO:0006898	C:membrane; F:scavenger receptor activity; P:receptor-mediated endocytosis	
Acrdi.XM_015910394.1/1-3054 | Monca.augustus.g45160.t1/1-2799	0.322448	0.686657	Y	ankyrin repeat	3	F:GO:0008270; P:GO:0007165; F:GO:0046872	F:zinc ion binding; P:signal transduction; F:metal ion binding	
Acrdi.XM_015906021.1/1-915 | Monca.augustus.g41391.t1/1-1749	0.711393	0.68561	Y	PREDICTED: uncharacterized protein LOC107340654				
Acrdi.XM_015899790.1/1-852 | Monca.augustus.g43136.t1/1-1008	0.622767	0.681211	Y	PREDICTED: uncharacterized protein LOC107334834				
Acrdi.XM_015899727.1/1-1602 | Monca.augustus.g3036.t1/1-1293	0.307357	0.676902	Y	Zinc finger SWIM domain-containing				
Acrdi.XM_015908940.1/1-3894 | Monca.augustus.g31881.t1/1-4875	0.419569	0.675906	Y	PREDICTED: uncharacterized protein LOC107343376				
Acrdi.XM_015906780.1/1-417 | Monca.augustus.g5573.t1/1-378	0.311536	0.675165	Y	PREDICTED: uncharacterized protein LOC107341378				
Acrdi.XM_015919058.1/1-822 | Monca.augustus.g26921.t1/1-2199	0.384081	0.667165	Y	PREDICTED: uncharacterized protein LOC107352745	3	F:GO:0008270; P:GO:0007275; P:GO:0015031	F:zinc ion binding; P:multicellular organism development; P:protein transport	
Acrdi.XM_015903438.1/1-351 | Monca.augustus.g39089.t1/1-375	0.338967	0.666284	Y	PREDICTED: uncharacterized protein LOC107338205				
Acrdi.XM_015914969.1/1-1542 | Monca.adi2mcaRNA25617_R3/1-1479	0.302579	0.661515	Y	cnidarian egg lectin isoform d				
Acrdi.XM_015909507.1/1-327 | Monca.augustus.g55401.t1/1-1674	0.471156	0.660155	Y	PREDICTED: uncharacterized protein LOC107343917				
Acrdi.XM_015893343.1/1-2694 | Monca.adi2mcaRNA3995_R0/1-2769	0.59278	0.65998	Y	PREDICTED: uncharacterized protein LOC107328618				
Acrdi.XM_015898488.1/1-3339 | Monca.adi2mcaRNA7477_R0/1-2868	0.553039	0.659324	Y	PREDICTED: uncharacterized protein LOC107333657 isoform X2				
Acrdi.XM_015897760.1/1-561 | Monca.augustus.g32108.t1/1-543	0.29415	0.657108	Y	max isoform X2				
Acrdi.XM_015924265.1/1-507 | Monca.adi2mcaRNA36017_R0/1-495	0.293793	0.649976	Y	PREDICTED: uncharacterized protein LOC107357624				
Acrdi.XM_015911377.1/1-867 | Monca.augustus.g35255.t1/1-1242	0.382218	0.649371	Y	PREDICTED: uncharacterized protein C7orf50 homolog				
Acrdi.XM_015923646.1/1-642 | Monca.adi2mcaRNA35324_R0/1-654	0.352033	0.648081	Y	PREDICTED: uncharacterized protein LOC107357004				
Acrdi.XM_015923755.1/1-849 | Monca.augustus.g34634.t1/1-1110	0.301549	0.646092	Y	PREDICTED: uncharacterized protein LOC107357100				
Acrdi.XM_015897989.1/1-618 | Monca.augustus.g41789.t1/1-888	0.44552	0.639881	Y	PREDICTED: uncharacterized protein LOC107333187				
Acrdi.XM_015893491.1/1-561 | Monca.augustus.g59379.t1/1-393	0.299031	0.637727	Y	Mitochondrial inner membrane protease subunit 1				
Acrdi.XM_015921832.1/1-1665 | Monca.augustus.g62828.t1/1-504	0.300869	0.636204	Y	Toxin -A				
Acrdi.XM_015916367.1/1-636 | Monca.adi2mcaRNA27187_R1/1-594	0.257779	0.633698	Y	amnionless	4	[illegible]	[illegible]	
Acrdi.XM_015918130.1/1-363 | Monca.adi2mcaRNA29164_R0/1-387	0.649179	0.632641	Y	PREDICTED: uncharacterized protein LOC107351824				
Acrdi.XM_015921858.1/1-333 | Monca.adi2mcaRNA33254_R2/1-540	0.252184	0.630853	Y	PREDICTED: uncharacterized protein LOC107355317				
Acrdi.XM_015903327.1/1-1773 | Monca.augustus.g17132.t1/1-1200	0.27103	0.629602	Y	mab-21-like 3				
Acrdi.XM_015919384.1/1-603 | Monca.augustus.g10237.t1/1-558	0.267738	0.627885	Y	PREDICTED: uncharacterized protein LOC107353065				
Acrdi.XM_015920615.1/1-1500 | Monca.augustus.g34704.t1/1-1698	0.37132	0.626686	Y	TNF receptor-associated factor 4-like	7	[illegible]	[illegible]	
Acrdi.XM_015916530.1/1-1323 | Monca.augustus.g38985.t1/1-1437	0.406807	0.621478	Y	Matrix metallo ase-14	12	[illegible]	[illegible]	
Acrdi.XM_015915710.1/1-1161 | Monca.augustus.g28537.t1/1-978	0.316925	0.617078	Y	serum response factor-binding 1-like	5	[illegible]	[illegible]	
Acrdi.XM_015892300.1/1-1998 | Monca.augustus.g59696.t1/1-2544	0.272713	0.610037	Y	hemicentin-1 isoform X2				
Acrdi.XM_015924267.1/1-864 | Monca.adi2mcaRNA36039_R0/1-882	0.242098	0.609671	Y	RNA-binding squid isoform X2				
Acrdi.XM_015915971.1/1-1107 | Monca.augustus.g10727.t1/1-1095	0.290582	0.607207	Y	neuropeptide FF receptor 2-like	4	[illegible]	[illegible]	
Acrdi.XM_015902440.1/1-609 | Monca.adi2mcaRNA11783_R0/1-495	0.63151	0.602243	Y	starmaker-like isoform X2				
Acrdi.XM_015921446.1/1-321 | Monca.augustus.g54293.t1/1-306	0.317641	0.600574	Y	PREDICTED: uncharacterized protein LOC107354951				
Acrdi.XM_015898945.1/1-606 | Monca.augustus.g7893.t1/1-621	0.644336	0.599763	Y	PREDICTED: uncharacterized protein LOC107334064				
Acrdi.XM_015905299.1/1-726 | Monca.augustus.g23131.t1/1-876	0.382824	0.597946	Y	vascular endothelial growth factor A-like				
Acrdi.XM_015909575.1/1-2664 | Monca.augustus.g18775.t1/1-2577	0.543739	0.595984	Y	PREDICTED: uncharacterized protein LOC107343974 isoform X1				
Acrdi.XM_015899394.1/1-3360 | Monca.augustus.g46761.t1/1-4104	0.30817	0.595378	Y	glutamic acid-rich -like				
Acrdi.XM_015894127.1/1-1077 | Monca.adi2mcaRNA40351_R1/1-1071	0.255539	0.594111	Y	unc-45 homolog A isoform X1				
Acrdi.XM_015902702.1/1-2763 | Monca.augustus.g43238.t1/1-3465	0.474757	0.590607	Y	PREDICTED: uncharacterized protein LOC107337515				
Acrdi.XM_015897530.1/1-3723 | Monca.augustus.g22815.t1/1-4839	0.437562	0.589893	Y	PREDICTED: uncharacterized protein LOC107332783				
Acrdi.XM_015902217.1/1-738 | Monca.augustus.g34043.t1/1-777	0.22986	0.589489	Y	Onco -induced transcript 3				
Acrdi.XM_015909574.1/1-2802 | Monca.augustus.g33517.t1/1-2829	0.424514	0.586869	Y	PREDICTED: uncharacterized protein LOC107343973				
Acrdi.XM_015925000.1/1-762 | Monca.augustus.g39449.t1/1-1659	0.230111	0.586815	Y	splicing factor U2AF 35 kDa subunit isoform X2	10	[illegible]	[illegible]	
Acrdi.XM_015924509.1/1-1878 | Monca.augustus.g60345.t1/1-1863	0.65664	0.585962	Y	delta A	6	[illegible]	[illegible]	
Acrdi.XM_015913425.1/1-1119 | Monca.adi2mcaRNA23874_R0/1-1296	0.426816	0.5852	Y	PREDICTED: putative protein TPRXL				
Acrdi.XM_015915789.1/1-1353 | Monca.augustus.g34182.t1/1-1302	0.223152	0.584058	Y	ATP-binding cassette sub-family D member 3	3	[illegible]	[illegible]	
Acrdi.XM_015915761.1/1-1056 | Monca.augustus.g15326.t1/1-963	0.320848	0.582897	Y	adrenocorticotropic hormone receptor-like	1	F:GO:0004871	[illegible]	
Acrdi.XM_015916086.1/1-399 | Monca.adi2mcaRNA26844_R1/1-423	0.402775	0.582806	Y	poly [ADP-ribose] polymerase 14-like				
Acrdi.XM_015911059.1/1-1524 | Monca.augustus.g30523.t1/1-1365	0.236268	0.579835	Y	L-amino-acid oxidase-like	4	[illegible]	[illegible]	
Acrdi.XM_015922239.1/1-453 | Monca.augustus.g59144.t1/1-519	0.335882	0.577732	Y	PREDICTED: uncharacterized protein LOC107355658				
Acrdi.XM_015902323.1/1-693 | Monca.adi2mcaRNA11673_R0/1-573	0.240115	0.577175	Y	zinc finger 862-like	6	[illegible]	[illegible]	
Acrdi.XM_015902128.1/1-939 | Monca.augustus.g15068.t1/1-1071	0.556887	0.577059	Y	PREDICTED: uncharacterized protein LOC107337018				
Acrdi.XM_015905893.1/1-1350 | Monca.adi2mcaRNA15624_R0/1-1341	0.255286	0.576703	Y	collagen alpha-2(IV) chain-like				
Acrdi.XM_015896073.1/1-1089 | Monca.adi2mcaRNA4927_R0/1-1002	0.245668	0.574095	Y	structural maintenance of chromosomes 1A-like	3	[illegible]	[illegible]	
Acrdi.XM_015911875.1/1-732 | Monca.augustus.g17907.t1/1-762	0.241187	0.568585	Y	PREDICTED: uncharacterized protein LOC107346103				
Acrdi.XM_015923833.1/1-585 | Monca.augustus.g8183.t1/1-441	0.240047	0.567919	Y	PREDICTED: uncharacterized protein LOC107357193				
Acrdi.XM_015892425.1/1-687 | Monca.augustus.g33778.t1/1-729	0.221276	0.565477	Y	V(D)J recombination activating				
Acrdi.XM_015915895.1/1-1203 | Monca.augustus.g61000.t1/1-1521	0.459974	0.563994	Y	PREDICTED: uncharacterized protein LOC107349695 isoform X3				
Acrdi.XM_015918782.1/1-636 | Monca.augustus.g41903.t1/1-729	0.221246	0.562637	Y	eukaryotic translation initiation factor 3 subunit C-like				
Acrdi.XM_015924896.1/1-591 | Monca.augustus.g34997.t1/1-1074	0.370341	0.561741	Y	PREDICTED: uncharacterized protein LOC107358277				
Acrdi.XM_015917171.1/1-717 | Monca.augustus.g42537.t1/1-831	0.215537	0.557575	Y	endonuclease exonuclease phosphatase family	7	[illegible]	[illegible]	
Acrdi.XM_015908228.1/1-462 | Monca.adi2mcaRNA18203_R3/1-339	0.553483	0.557431	Y	PREDICTED: uncharacterized protein LOC107342736				
Acrdi.XM_015912372.1/1-867 | Monca.augustus.g50435.t1/1-768	0.462804	0.557353	Y	Discoidin domain-containing receptor 2	1	F:GO:0070492	[illegible]	
Acrdi.XM_015923312.1/1-768 | Monca.adi2mcaRNA34963_R0/1-762	0.216402	0.555817	Y	PREDICTED: uncharacterized protein LOC107356689	2	[illegible]	[illegible]	
Acrdi.XM_015895512.1/1-525 | Monca.adi2mcaRNA4240_R0/1-546	0.254915	0.554775	Y	PREDICTED: uncharacterized protein LOC107330978				
Acrdi.XM_015892080.1/1-1128 | Monca.augustus.g60522.t1/1-1128	0.224504	0.55469	Y	endonuclease domain-containing 1 -like				
Acrdi.XM_015902620.1/1-10908 | Monca.adi2mcaRNA12031_R0/1-1006	0.442018	0.553376	Y	serine-rich adhesin for platelets-like				
Acrdi.XM_015914540.1/1-579 | Monca.augustus.g29166.t1/1-1101	0.558581	0.552611	Y	PREDICTED: uncharacterized protein LOC107348499				
Acrdi.XM_015910764.1/1-837 | Monca.augustus.g29671.t1/1-1209	0.492405	0.551778	Y	PREDICTED: uncharacterized protein LOC107345070				
Acrdi.XM_015894629.1/1-927 | Monca.augustus.g53538.t1/1-705	0.366314	0.551534	Y	PREDICTED: uncharacterized protein LOC107329972				
Acrdi.XM_015903258.1/1-414 | Monca.augustus.g51687.t1/1-645	0.584331	0.551127	Y	PREDICTED: uncharacterized protein LOC107338010				
Acrdi.XM_015905252.1/1-1125 | Monca.augustus.g1756.t1/1-1107	0.467495	0.550933	Y	PREDICTED: uncharacterized protein LOC107339906				
Acrdi.XM_015916319.1/1-1311 | Monca.augustus.g49887.t1/1-1236	0.323579	0.550591	Y	PREDICTED: uncharacterized protein LOC107350097				
Acrdi.XM_015902796.1/1-633 | Monca.augustus.g10683.t1/1-933	0.317496	0.549596	Y	PREDICTED: uncharacterized protein LOC107337591				
Acrdi.XM_015895343.1/1-801 | Monca.augustus.g8873.t1/1-780	0.223927	0.548478	Y	G2 M phase-specific E3 ubiquitin- ligase-like	2	[illegible]	[illegible]	
Acrdi.XM_015893910.1/1-1221 | Monca.adi2mcaRNA3988_R0/1-1227	0.376112	0.547134	Y	low-density lipo receptor-related 4-like	2	[illegible]	[illegible]	
Acrdi.XM_015913418.1/1-447 | Monca.augustus.g32130.t1/1-441	0.216992	0.546475	Y	ephrin domain	4	[illegible]	[illegible]	
Acrdi.XM_015901541.1/1-4257 | Monca.augustus.g65901.t1/1-4404	0.494645	0.545526	Y	U7 snRNA-associated Sm LSm11	3	[illegible]	[illegible]	
Acrdi.XM_015910954.1/1-531 | Monca.augustus.g68190.t1/1-528	0.32614	0.545306	Y	Epididymal secretory E1	2	[illegible]	[illegible]	
Acrdi.XM_015895564.1/1-465 | Monca.augustus.g32974.t1/1-450	0.718473	0.544289	Y	PREDICTED: uncharacterized protein LOC107331028				
Acrdi.XM_015911212.1/1-1737 | Monca.augustus.g35949.t1/1-1680	0.195598	0.543389	Y	lectin C-type domain	2	[illegible]	[illegible]	
Acrdi.XM_015919164.1/1-318 | Monca.augustus.g50966.t1/1-327	0.21584	0.541132	Y	PREDICTED: uncharacterized protein LOC107334261				
Acrdi.XM_015920958.1/1-612 | Monca.adi2mcaRNA32267_R7/1-384	0.671911	0.539734	Y	signal transducer and activator of transcription 2-like	2	[illegible]	[illegible]	
Acrdi.XM_015895283.1/1-519 | Monca.augustus.g54365.t1/1-564	0.217302	0.539647	Y	lecithin retinol acyltransferase family	4	[illegible]	[illegible]	
Acrdi.XM_015911763.1/1-1113 | Monca.augustus.g26645.t1/1-957	0.202037	0.538537	Y	Werner syndrome ATP-dependent helicase-like	5	[illegible]	[illegible]	
Acrdi.XM_015897460.1/1-2019 | Monca.augustus.g22826.t1/1-2052	0.404485	0.537856	Y	transcriptional regulator ATRX homolog				
Acrdi.XM_015891999.1/1-876 | Monca.augustus.g32099.t1/1-894	0.231183	0.53713	Y	histamine H2 receptor-like	6	[illegible]	[illegible]	
Acrdi.XM_015905527.1/1-1059 | Monca.augustus.g28252.t1/1-1320	0.552527	0.536379	Y	PREDICTED: uncharacterized protein LOC107340162 isoform X4				
Acrdi.XM_015909431.1/1-933 | Monca.augustus.g55055.t1/1-1488	0.539705	0.536176	Y	PREDICTED: uncharacterized protein LOC107343843				
Acrdi.XM_015898037.1/1-981 | Monca.augustus.g39788.t1/1-909	0.199688	0.534518	Y	neuronal pentraxin-2-like isoform X1	3	[illegible]	[illegible]	
Acrdi.XM_015904716.1/1-1254 | Monca.augustus.g36188.t1/1-1797	0.421699	0.534456	Y	PREDICTED: uncharacterized protein LOC107339431	2	[illegible]	[illegible]	
Acrdi.XM_015913456.1/1-468 | Monca.augustus.g58016.t1/1-696	0.200077	0.533113	Y	nucleolar transcription factor 1-A-like				
Acrdi.XM_015905626.1/1-318 | Monca.augustus.g24051.t1/1-318	0.371656	0.532476	Y	PREDICTED: uncharacterized protein LOC107340279				
Acrdi.XM_015901656.1/1-1086 | Monca.adi2mcaRNA10947_R0/1-1089	0.412756	0.532292	Y	PREDICTED: uncharacterized protein LOC107336587				
Acrdi.XM_015904486.1/1-1959 | Monca.augustus.g33606.t1/1-2109	0.541122	0.530833	Y	PREDICTED: uncharacterized protein LOC107339225				
Acrdi.XM_015900483.1/1-708 | Monca.augustus.g49624.t1/1-1083	0.254764	0.530794	Y	Pre-mRNA-splicing factor 38B	1	C:GO:0071011	[illegible]	
Acrdi.XM_015924715.1/1-519 | Monca.augustus.g53787.t1/1-519	0.685704	0.530537	Y	PREDICTED: uncharacterized protein LOC107358081				
Acrdi.XM_015911329.1/1-1002 | Monca.augustus.g35283.t1/1-1116	0.413335	0.530002	Y	PREDICTED: uncharacterized protein LOC107345598				
Acrdi.XM_015892760.1/1-525 | Monca.adi2mcaRNA38776_R4/1-468	0.186885	0.526335	Y	RNA-directed DNA polymerase from mobile element jockey				
Acrdi.XM_015893473.1/1-1431 | Monca.augustus.g18953.t1/1-1335	0.183296	0.525122	Y	branchpoint-bridging -like				
Acrdi.XM_015904448.1/1-462 | Monca.adi2mcaRNA13962_R0/1-501	0.194688	0.524761	Y	activin receptor type-2B-like isoform X1				
Acrdi.XM_015903624.1/1-621 | Monca.augustus.g59871.t1/1-3876	0.412191	0.523581	Y	translation initiation factor eIF-2B subunit delta-like				
Acrdi.XM_015924418.1/1-696 | Monca.augustus.g39523.t1/1-1149	0.226795	0.522687	Y	gliding motility regulatory -like	5	[illegible]	[illegible]	
Acrdi.XM_015894071.1/1-399 | Monca.adi2mcaRNA40272_R4/1-354	0.582221	0.518234	Y	PREDICTED: uncharacterized protein LOC107329379				
Acrdi.XM_015923353.1/1-450 | Monca.augustus.g29093.t1/1-558	0.268287	0.517584	Y	PREDICTED: uncharacterized protein LOC107356729				
Acrdi.XM_015924775.1/1-462 | Monca.adi2mcaRNA36631_R3/1-414	0.205273	0.517506	Y	RNA-directed DNA polymerase from mobile element jockey				
Acrdi.XM_015893367.1/1-1167 | Monca.augustus.g50884.t1/1-3189	0.391726	0.517295	Y	PREDICTED: uncharacterized protein LOC107328634				
Acrdi.XM_015902191.1/1-492 | Monca.augustus.g69079.t1/1-468	0.20242	0.516398	Y	urokinase plasminogen activator surface receptor-like isoform X2				
Acrdi.XM_015912816.1/1-732 | Monca.adi2mcaRNA23180_R0/1-702	0.266556	0.514502	Y	Phosphatidylinositol-glycan biosynthesis class X	4	[illegible]	[illegible]	
Acrdi.XM_015921611.1/1-1881 | Monca.adi2mcaRNA32973_R0/1-2262	0.327028	0.511881	Y	latrophilin Cirl isoform X9				
Acrdi.XM_015918777.1/1-504 | Monca.adi2mcaRNA29855_R3/1-558	0.189624	0.510683	Y	threonine dehydratase chloroplastic-like	4	[illegible]	[illegible]	
Acrdi.XM_015905203.1/1-813 | Monca.augustus.g52822.t1/1-741	0.434628	0.510676	Y	PREDICTED: uncharacterized protein LOC107339870				
Acrdi.XM_015912279.1/1-873 | Monca.adi2mcaRNA22587_R0/1-1002	0.227646	0.509577	Y	secreted acidic 1A-like				
Acrdi.XM_015908804.1/1-474 | Monca.augustus.g69461.t1/1-1071	0.225178	0.50912	Y	arginase- mitochondrial-like	6	[illegible]	[illegible]	
Acrdi.XM_015908282.1/1-1188 | Monca.augustus.g70050.t1/1-1203	0.408332	0.508157	Y	PREDICTED: uncharacterized protein LOC107342776				
Acrdi.XM_015900575.1/1-735 | Monca.augustus.g33645.t1/1-1899	0.34252	0.505341	Y	probable serine threonine- kinase fhkE				
Acrdi.XM_015891976.1/1-1128 | Monca.augustus.g25165.t1/1-1152	0.371815	0.504434	Y	EGF and laminin G domain-containing -like isoform X5	2	[illegible]	[illegible]	
Acrdi.XM_015898419.1/1-801 | Monca.augustus.g60829.t1/1-1269	0.437467	0.503998	Y	telomere repeats-binding bouquet formation 2-like isoform X3				
Acrdi.XM_015920714.1/1-1587 | Monca.augustus.g53726.t1/1-2430	0.186187	0.501756	Y	E3 ubiquitin- ligase TOM1-				
Acrdi.XM_015911775.1/1-831 | Monca.adi2mcaRNA22032_R0/1-717	0.42588	0.501275	Y	vesicle-associated membrane synaptobrevin-binding -like isoform X1	7	[illegible]	[illegible]	
Acrdi.XM_015922868.1/1-1491 | Monca.augustus.g16208.t1/1-1476	0.251618	0.501212	Y	Ribosomal RNA-processing 8	8	[illegible]	[illegible]	
Acrdi.XM_015904052.1/1-540 | Monca.augustus.g27779.t1/1-624	0.330023	0.499474	Y	PREDICTED: uncharacterized protein LOC107338802				
Acrdi.XM_015906098.1/1-1260 | Monca.augustus.g34428.t1/1-2769	0.453466	0.49945	Y	PREDICTED: uncharacterized protein LOC107340717				
Acrdi.XM_015923936.1/1-1461 | Monca.augustus.g1642.t1/1-1728	0.369444	0.498409	Y	von Willebrand factor A domain-containing 7-like	1	F:GO:0005509	[illegible]	
Acrdi.XM_015895357.1/1-528 | Monca.adi2mcaRNA41798_R2/1-390	0.269968	0.498001	Y	Nucleo TPR				
Acrdi.XM_015900150.1/1-612 | Monca.augustus.g65817.t1/1-630	0.184347	0.496511	Y	PREDICTED: uncharacterized protein LOC107335161				
Acrdi.XM_015896873.1/1-420 | Monca.adi2mcaRNA5730_R0/1-444	0.190148	0.495898	Y	PREDICTED: uncharacterized protein LOC107332144				
Acrdi.XM_015909486.1/1-399 | Monca.adi2mcaRNA19547_R4/1-342	0.177055	0.494888	Y	Pogo transposable element with	2	[illegible]	[illegible]	
Acrdi.XM_015918227.1/1-432 | Monca.augustus.g53124.t1/1-663	0.180972	0.494796	Y	PREDICTED: uncharacterized protein LOC107351923				
Acrdi.XM_015910616.1/1-1095 | Monca.augustus.g45392.t1/1-1089	0.394278	0.492052	Y	PREDICTED: uncharacterized protein LOC107344929				
Acrdi.XM_015895842.1/1-7242 | Monca.adi2mcaRNA434_R0/1-6264	0.347529	0.491801	Y	PREDICTED: uncharacterized protein LOC107331268				
Acrdi.XM_015911129.1/1-627 | Monca.augustus.g7157.t1/1-846	0.281005	0.491201	Y	secreted frizzled-related 3	9	[illegible]	[illegible]	
Acrdi.XM_015893970.1/1-1281 | Monca.augustus.g50890.t1/1-1287	0.406369	0.491137	Y	PREDICTED: uncharacterized protein LOC107329237				
Acrdi.XM_015893337.1/1-741 | Monca.adi2mcaRNA39413_R2/1-615	0.19386	0.490823	Y	hemicentin-2-like isoform X1	1	F:GO:0070492	[illegible]	
Acrdi.XM_015901982.1/1-579 | Monca.augustus.g69328.t1/1-1239	0.27079	0.490434	Y	onco -induced transcript 3 -like				
Acrdi.XM_015921839.1/1-759 | Monca.augustus.g16480.t1/1-1035	0.367842	0.490263	Y	PREDICTED: uncharacterized protein LOC107355298				
Acrdi.XM_015919348.1/1-498 | Monca.adi2mcaRNA30522_R0/1-588	0.27629	0.490008	Y	PREDICTED: uncharacterized protein LOC107353030				
Acrdi.XM_015905112.1/1-885 | Monca.adi2mcaRNA14762_R5/1-696	0.174524	0.489728	Y	PREDICTED: uncharacterized protein LOC107339799				
Acrdi.XM_015900788.1/1-792 | Monca.augustus.g39839.t1/1-1320	0.192595	0.489492	Y	leukocyte tyrosine kinase receptor-like				
Acrdi.XM_015893948.1/1-3147 | Monca.adi2mcaRNA4005_R0/1-3294	0.472506	0.488944	Y	PREDICTED: uncharacterized protein LOC107329210 isoform X1				
Acrdi.XM_015913916.1/1-3105 | Monca.adi2mcaRNA24402_R0/1-3225	0.511133	0.488676	Y	PREDICTED: uncharacterized protein LOC107347925				
Acrdi.XM_015910922.1/1-2631 | Monca.adi2mcaRNA21127_R0/1-2928	0.152722	0.488337	Y	Retrovirus-related Pol poly from transposon	2	[illegible]	[illegible]	
Acrdi.XM_015917971.1/1-312 | Monca.adi2mcaRNA28971_R8/1-405	0.197049	0.488204	Y	PREDICTED: uncharacterized protein LOC107351679				
Acrdi.XM_015899984.1/1-1509 | Monca.augustus.g43420.t1/1-1524	0.510309	0.48808	Y	deleted in malignant brain tumors 1 -like				
Acrdi.XM_015896230.1/1-2604 | Monca.augustus.g19340.t1/1-2286	0.411224	0.488023	Y	PREDICTED: uncharacterized protein LOC107331620				
Acrdi.XM_015908795.1/1-1341 | Monca.adi2mcaRNA18775_R2/1-1425	0.177728	0.486189	Y	ATP-dependent DNA helicase PIF1				
Acrdi.XM_015905327.1/1-708 | Monca.augustus.g39293.t1/1-708	0.178339	0.485975	Y	RNA-directed DNA polymerase from mobile element jockey-like				
Acrdi.XM_015901660.1/1-675 | Monca.augustus.g55895.t1/1-486	0.320709	0.48521	Y	AN1-type zinc finger 1	3	[illegible]	[illegible]	
Acrdi.XM_015911105.1/1-1365 | Monca.augustus.g55141.t1/1-1611	0.455895	0.484518	Y	PREDICTED: uncharacterized protein LOC107345404	2	[illegible]	[illegible]	
Acrdi.XM_015915682.1/1-564 | Monca.adi2mcaRNA26419_R0/1-573	0.271533	0.483548	Y	PREDICTED: uncharacterized protein LOC107349504				
Acrdi.XM_015903900.1/1-1125 | Monca.augustus.g32298.t1/1-1494	0.463894	0.483492	Y	lysosome-associated membrane glyco 1-like isoform X2	2	[illegible]	[illegible]	
Acrdi.XM_015897534.1/1-777 | Monca.augustus.g22819.t1/1-2781	0.265255	0.483276	Y	PREDICTED: uncharacterized protein LOC107332787				
Acrdi.XM_015917918.1/1-810 | Monca.augustus.g25160.t1/1-858	0.179509	0.482131	Y	lysophosphatidic acid receptor 3-like	6	[illegible]	[illegible]	
Acrdi.XM_015908754.1/1-618 | Monca.augustus.g26276.t1/1-1863	0.368128	0.482117	Y	PREDICTED: uncharacterized protein LOC107343180				
Acrdi.XM_015906616.1/1-693 | Monca.augustus.g70212.t1/1-948	0.426649	0.481568	Y	PREDICTED: uncharacterized protein LOC107341208				
Acrdi.XM_015899168.1/1-921 | Monca.augustus.g34553.t1/1-831	0.185002	0.481353	Y	Secreted frizzled-related 1	1	P:GO:0007165	[illegible]	
Acrdi.XM_015923504.1/1-1224 | Monca.adi2mcaRNA35184_R0/1-1260	0.511427	0.480414	Y	PREDICTED: uncharacterized protein LOC107356875				
Acrdi.XM_015922924.1/1-423 | Monca.augustus.g2952.t1/1-513	0.170866	0.480356	Y	N-alpha-acetyltransferase 50 isoform X2	5	[illegible]	[illegible]	
Acrdi.XM_015918527.1/1-1419 | Monca.adi2mcaRNA29576_R0/1-1575	0.383563	0.479832	Y	eukaryotic translation initiation factor 5B-like				
Acrdi.XM_015892345.1/1-348 | Monca.augustus.g21710.t1/1-336	0.362502	0.478999	Y	PREDICTED: uncharacterized protein LOC107327603				
Acrdi.XM_015902132.1/1-1026 | Monca.augustus.g46374.t1/1-1020	0.433053	0.478941	Y	bone morphogenetic 2-like	2	[illegible]	[illegible]	
Acrdi.XM_015899561.1/1-774 | Monca.augustus.g46959.t1/1-828	0.20069	0.478762	Y	Nucleolar transcription factor 1				
Acrdi.XM_015922705.1/1-435 | Monca.augustus.g46494.t1/1-438	0.311751	0.478361	Y	PREDICTED: uncharacterized protein LOC107356068				
Acrdi.XM_015917979.1/1-738 | Monca.augustus.g45181.t1/1-1083	0.356784	0.477006	Y	PREDICTED: uncharacterized protein LOC107351692				
Acrdi.XM_015921162.1/1-1464 | Monca.augustus.g53832.t1/1-2184	0.437826	0.475054	Y	PREDICTED: uncharacterized protein LOC107354682				
Acrdi.XM_015921597.1/1-2118 | Monca.augustus.g41678.t1/1-2253	0.670352	0.475	Y	PREDICTED: uncharacterized protein LOC107355085				
Acrdi.XM_015892836.1/1-1287 | Monca.augustus.g25505.t1/1-1308	0.20973	0.47453	Y	hemicentin-1				
Acrdi.XM_015906018.1/1-1785 | Monca.augustus.g41396.t1/1-2025	0.444682	0.47419	Y	PREDICTED: uncharacterized protein LOC107340651				
Acrdi.XM_015903012.1/1-960 | Monca.augustus.g47585.t1/1-1092	0.321084	0.473912	Y	RNA RNP complex-1-interacting phosphatase	2	[illegible]	[illegible]	
Acrdi.XM_015898177.1/1-348 | Monca.augustus.g29253.t1/1-576	0.165627	0.47384	Y	PREDICTED: uncharacterized protein LOC107333354 isoform X1				
Acrdi.XM_015908733.1/1-1251 | Monca.augustus.g204.t1/1-1230	0.453914	0.473793	Y	PREDICTED: uncharacterized protein LOC107343166				
Acrdi.XM_015894846.1/1-1749 | Monca.augustus.g31002.t1/1-2934	0.295972	0.472712	Y	Pleckstrin-likey domain-containing family A member 7				
Acrdi.XM_015899259.1/1-1761 | Monca.augustus.g62002.t1/1-2577	0.44079	0.472457	Y	PREDICTED: uncharacterized protein LOC107334327 isoform X2				
Acrdi.XM_015912704.1/1-663 | Monca.adi2mcaRNA23087_R0/1-657	0.258594	0.471952	Y	PREDICTED: uncharacterized protein LOC107346854				
Acrdi.XM_015908001.1/1-1041 | Monca.augustus.g43121.t1/1-1122	0.408085	0.471923	Y	PREDICTED: uncharacterized protein LOC107342499				
Acrdi.XM_015891900.1/1-1311 | Monca.augustus.g25613.t1/1-1308	0.482321	0.471855	Y	CAP-Gly domain-containing linker 1-like isoform X1	1	P:GO:0031116	[illegible]	
Acrdi.XM_015897287.1/1-540 | Monca.augustus.g49861.t1/1-657	0.189074	0.471284	Y	PREDICTED: uncharacterized protein LOC107332548				
Acrdi.XM_015892580.1/1-477 | Monca.augustus.g20311.t1/1-639	0.226512	0.471166	Y	DNA ligase				
Acrdi.XM_015916118.1/1-2751 | Monca.augustus.g24448.t1/1-1875	0.277193	0.4709	Y	histidine-rich PFHRP-II-				
Acrdi.XM_015920307.1/1-1443 | Monca.adi2mcaRNA31566_R1/1-1695	0.295991	0.470855	Y	F-box DNA helicase 1 isoform X1	5	[illegible]	[illegible]	
Acrdi.XM_015907937.1/1-327 | Monca.augustus.g4333.t1/1-390	0.369308	0.470704	Y	PREDICTED: uncharacterized protein LOC107342440 isoform X1				
Acrdi.XM_015894986.1/1-2541 | Monca.adi2mcaRNA41351_R1/1-2064	0.151058	0.469294	Y	PREDICTED: uncharacterized protein LOC107330360				
Acrdi.XM_015894653.1/1-1602 | Monca.augustus.g46369.t1/1-2097	0.255203	0.469234	Y	ras and EF-hand domain-containing homolog	3	[illegible]	[illegible]	
Acrdi.XM_015918775.1/1-1050 | Monca.adi2mcaRNA3027_R0/1-1434	0.316158	0.46888	Y	B Chain Rhamnose-Binding Lectin Csl3	3	[illegible]	[illegible]	
Acrdi.XM_015891979.1/1-630 | Monca.augustus.g18682.t1/1-570	0.220476	0.468325	Y	PREDICTED: uncharacterized protein LOC107327229 isoform X1				
Acrdi.XM_015899270.1/1-1803 | Monca.augustus.g50906.t1/1-1893	0.397864	0.468066	Y	serine threonine- kinase pim-1-like				
Acrdi.XM_015908183.1/1-825 | Monca.augustus.g10443.t1/1-1560	0.538237	0.466888	Y	PREDICTED: uncharacterized protein LOC107342681				
Acrdi.XM_015922906.1/1-864 | Monca.augustus.g56841.t1/1-840	0.575504	0.466735	Y	PREDICTED: uncharacterized protein LOC107356272				
Acrdi.XM_015901587.1/1-1236 | Monca.augustus.g55979.t1/1-1206	0.380647	0.46605	Y	PREDICTED: uncharacterized protein LOC107336515				
Acrdi.XM_015922466.1/1-1149 | Monca.augustus.g40393.t1/1-3780	0.489814	0.465969	Y	PREDICTED: uncharacterized protein LOC107355842				
Acrdi.XM_015907553.1/1-366 | Monca.augustus.g19978.t1/1-414	0.172159	0.464488	Y	RNA-directed DNA polymerase from mobile element jockey-like				
Acrdi.XM_015914538.1/1-1767 | Monca.augustus.g29164.t1/1-1740	0.325763	0.464193	Y	PREDICTED: uncharacterized protein LOC107348496				
Acrdi.XM_015919082.1/1-735 | Monca.augustus.g43655.t1/1-990	0.296128	0.464054	Y	PREDICTED: uncharacterized protein LOC107352760 isoform X2	2	[illegible]	[illegible]	
Acrdi.XM_015906831.1/1-1026 | Monca.augustus.g29122.t1/1-1605	0.173214	0.463345	Y	uncharacterized skeletal organic matrix 3-like isoform X2				
Acrdi.XM_015901927.1/1-1071 | Monca.augustus.g27159.t1/1-1200	0.487829	0.46328	Y	zinc finger 346-like isoform X2	5	[illegible]	[illegible]	
Acrdi.XM_015913999.1/1-834 | Monca.augustus.g47883.t1/1-807	0.168818	0.462921	Y	PREDICTED: uncharacterized protein LOC107348010				
Acrdi.XM_015906673.1/1-558 | Monca.augustus.g60582.t1/1-537	0.341196	0.462819	Y	PREDICTED: uncharacterized protein LOC107341272				
Acrdi.XM_015908433.1/1-1194 | Monca.adi2mcaRNA18418_R5/1-621	0.313006	0.461359	Y	pelota homolog	3	[illegible]	[illegible]	
Acrdi.XM_015924095.1/1-447 | Monca.augustus.g7974.t1/1-519	0.156186	0.461113	Y	Frizzled-8	6	[illegible]	[illegible]	
Acrdi.XM_015897616.1/1-576 | Monca.augustus.g4549.t1/1-720	0.507471	0.460341	Y	PREDICTED: uncharacterized protein LOC107332862				
Acrdi.XM_015918824.1/1-1644 | Monca.adi2mcaRNA29905_R2/1-1374	0.143709	0.460106	Y	ATP-dependent DNA helicase -like	2	[illegible]	[illegible]	
Acrdi.XM_015917149.1/1-1116 | Monca.augustus.g67344.t1/1-1839	0.419626	0.460032	Y	PREDICTED: uncharacterized protein LOC107350900				
Acrdi.XM_015923775.1/1-1080 | Monca.augustus.g54395.t1/1-1086	0.258185	0.459984	Y	growth differentiation factor 8	11	[illegible]	[illegible]	
Acrdi.XM_015904469.1/1-1752 | Monca.augustus.g32062.t1/1-1701	0.146735	0.45958	Y	moesin isoform X1	66	[illegible]	[illegible]	
Acrdi.XM_015918727.1/1-483 | Monca.adi2mcaRNA29808_R3/1-378	0.164276	0.459418	Y	RNA-directed DNA polymerase from mobile element				
Acrdi.XM_015919012.1/1-1380 | Monca.augustus.g32012.t1/1-1113	0.236967	0.458928	Y	probable serine threonine- kinase irlF				
Acrdi.XM_015910772.1/1-510 | Monca.adi2mcaRNA2151_R4/1-459	0.449234	0.458528	Y	contactin-associated -like 2				
Acrdi.XM_015898606.1/1-465 | Monca.augustus.g34556.t1/1-447	0.285086	0.458406	Y	PREDICTED: uncharacterized protein LOC107333756				
Acrdi.XM_015893569.1/1-3933 | Monca.adi2mcaRNA4074_R1/1-3462	0.281905	0.45594	Y	collagen alpha-1(II) chain	3	[illegible]	[illegible]	
Acrdi.XM_015905806.1/1-600 | Monca.adi2mcaRNA15468_R1/1-519	0.153021	0.455906	Y	PREDICTED: uncharacterized protein LOC107340454				
Acrdi.XM_015909648.1/1-1965 | Monca.adi2mcaRNA19714_R0/1-1776	0.394822	0.454074	Y	piggyBac transposable element-derived 4-like				
Acrdi.XM_015920077.1/1-405 | Monca.augustus.g24398.t1/1-912	0.39878	0.45333	Y	ame: Full=Coadhesin				
Acrdi.XM_015904109.1/1-438 | Monca.augustus.g36723.t1/1-501	0.47448	0.453095	Y	PREDICTED: uncharacterized protein LOC107338857				
Acrdi.XM_015894694.1/1-1638 | Monca.augustus.g68831.t1/1-636	0.285729	0.451845	Y	L-rhamnose-binding lectin CSL3-like isoform X1	3	[illegible]	[illegible]	
Acrdi.XM_015899310.1/1-681 | Monca.augustus.g665.t1/1-690	0.457618	0.451492	Y	Homeobox not2	3	[illegible]	[illegible]	
Acrdi.XM_015917089.1/1-681 | Monca.adi2mcaRNA2773_R2/1-660	0.249692	0.451406	Y	PREDICTED: uncharacterized protein LOC107350837				
Acrdi.XM_015904407.1/1-375 | Monca.augustus.g48063.t1/1-567	0.439597	0.45124	Y	PREDICTED: uncharacterized protein LOC107339162				
Acrdi.XM_015898046.1/1-600 | Monca.adi2mcaRNA7023_R0/1-573	1.31417	0.450803	Y	PREDICTED: uncharacterized protein LOC107333261				
Acrdi.XM_015906115.1/1-1908 | Monca.augustus.g10611.t1/1-1899	0.190937	0.450793	Y	Coiled-coil domain-containing 9	3	[illegible]	[illegible]	
Acrdi.XM_015900207.1/1-1245 | Monca.augustus.g62850.t1/1-1353	0.341475	0.450636	Y	PREDICTED: uncharacterized protein LOC107335213 isoform X2				
Acrdi.XM_015914976.1/1-3975 | Monca.augustus.g42433.t1/1-6306	0.523058	0.450501	Y	PREDICTED: uncharacterized protein LOC107348892				
Acrdi.XM_015897896.1/1-1245 | Monca.augustus.g39400.t1/1-1788	0.160356	0.450236	Y	glutamic acid-rich -like isoform X2				
Acrdi.XM_015908147.1/1-942 | Monca.adi2mcaRNA18085_R0/1-537	0.290905	0.450129	Y	ATP-dependent DNA helicase PIF1				
Acrdi.XM_015917693.1/1-5778 | Monca.augustus.g42284.t1/1-7236	0.315924	0.44989	Y	leucine-rich repeat-containing DDB_G0290503				
Acrdi.XM_015908758.1/1-789 | Monca.augustus.g27997.t1/1-789	0.540442	0.44974	Y	PREDICTED: uncharacterized protein LOC107343181				
Acrdi.XM_015918410.1/1-411 | Monca.augustus.g22381.t1/1-861	0.159812	0.449616	Y	patatin-like phospholipase domain-containing 4 isoform X1	2	[illegible]	[illegible]	
Acrdi.XM_015892726.1/1-4017 | Monca.augustus.g33458.t1/1-16155	0.332799	0.449615	Y	PREDICTED: uncharacterized protein LOC107328003				
Acrdi.XM_015899903.1/1-1104 | Monca.augustus.g44158.t1/1-1992	0.426206	0.449079	Y	transmembrane 2-like	5	[illegible]	[illegible]	
Acrdi.XM_015918120.1/1-954 | Monca.augustus.g29955.t1/1-939	0.587749	0.448641	Y	type II cytoskeletal 7-like				
Acrdi.XM_015918737.1/1-624 | Monca.adi2mcaRNA3015_R8/1-642	0.230592	0.447565	Y	Short transient receptor potential channel 4	1	P:GO:0006810	[illegible]	
Acrdi.XM_015908038.1/1-858 | Monca.augustus.g20212.t1/1-843	0.335801	0.447224	Y	PREDICTED: uncharacterized protein LOC107342533				
Acrdi.XM_015917756.1/1-663 | Monca.augustus.g46225.t1/1-657	0.247145	0.4466	Y	-like 1 homeobox	4	[illegible]	[illegible]	
Acrdi.XM_015921615.1/1-528 | Monca.augustus.g67243.t1/1-552	0.240275	0.446012	Y	cell wall integrity and stress response component 4-like	2	[illegible]	[illegible]	
Acrdi.XM_015893362.1/1-1176 | Monca.augustus.g30627.t1/1-1167	0.531582	0.445916	Y	corepressor interacting with RBPJ 1-like				
Acrdi.XM_015918614.1/1-1794 | Monca.augustus.g7620.t1/1-2109	0.345905	0.445362	Y	carbohydrate sulfotransferase 3-like	2	[illegible]	[illegible]	
Acrdi.XM_015915249.1/1-1128 | Monca.augustus.g46198.t1/1-1446	0.357587	0.444899	Y	PREDICTED: uncharacterized protein LOC107349149				
Acrdi.XM_015913101.1/1-1683 | Monca.augustus.g25090.t1/1-1758	0.362274	0.444715	Y	N-myc proto-oncogene -like	4	[illegible]	[illegible]	
Acrdi.XM_015922944.1/1-1401 | Monca.adi2mcaRNA34529_R0/1-1335	0.266935	0.44463	Y	PREDICTED: uncharacterized protein LOC107356328				
Acrdi.XM_015915455.1/1-513 | Monca.augustus.g32784.t1/1-1773	0.184897	0.443674	Y	PREDICTED: uncharacterized protein LOC107349320				
Acrdi.XM_015922037.1/1-5376 | Monca.adi2mcaRNA3362_R0/1-5307	0.364386	0.443606	Y	PREDICTED: uncharacterized protein LOC107355456				
Acrdi.XM_015896812.1/1-426 | Monca.augustus.g17706.t1/1-465	0.151598	0.44287	Y	transcription initiation factor TFIID subunit 10	4	[illegible]	[illegible]	
Acrdi.XM_015905444.1/1-891 | Monca.augustus.g33957.t1/1-858	0.178667	0.442093	Y	calumenin isoform X1	9	[illegible]	[illegible]	
Acrdi.XM_015904835.1/1-369 | Monca.augustus.g15364.t1/1-708	0.347418	0.441876	Y	proline-rich 11-like				
Acrdi.XM_015912604.1/1-768 | Monca.adi2mcaRNA2350_R0/1-474	0.256749	0.440165	Y	tctex1 domain-containing 1				
Acrdi.XM_015909834.1/1-735 | Monca.augustus.g47103.t1/1-1173	0.559085	0.440047	Y	PREDICTED: uncharacterized protein LOC107344190				
Acrdi.XM_015911688.1/1-1449 | Monca.augustus.g38091.t1/1-1530	0.350044	0.439991	Y	PREDICTED: extensin-like				
Acrdi.XM_015897137.1/1-1134 | Monca.adi2mcaRNA6046_R0/1-1116	0.167318	0.439253	Y	PREDICTED: uncharacterized protein LOC107332404				
Acrdi.XM_015897600.1/1-1116 | Monca.adi2mcaRNA6524_R6/1-1257	0.126795	0.437509	Y	ATP-dependent DNA helicase PIF1				
Acrdi.XM_015924346.1/1-504 | Monca.augustus.g32388.t1/1-489	0.268506	0.436831	Y	PREDICTED: uncharacterized protein LOC107357708				
Acrdi.XM_015907318.1/1-786 | Monca.augustus.g63467.t1/1-666	0.410572	0.436651	Y	giant isoform X1	3	[illegible]	[illegible]	
Acrdi.XM_015910569.1/1-4083 | Monca.augustus.g47630.t1/1-3960	0.555166	0.4364	Y	PREDICTED: uncharacterized protein LOC107344847				
Acrdi.XM_015903344.1/1-723 | Monca.augustus.g36048.t1/1-522	0.167707	0.435822	Y	PREDICTED: uncharacterized protein LOC107338107				
Acrdi.XM_015918584.1/1-1008 | Monca.augustus.g23491.t1/1-1167	0.427703	0.435605	Y	LWamide neuropeptide				
Acrdi.XM_015895301.1/1-4527 | Monca.augustus.g25934.t1/1-6849	0.316928	0.435303	Y	microtubule-associated futsch-like				
Acrdi.XM_015905110.1/1-1038 | Monca.augustus.g34914.t1/1-1104	0.475921	0.435194	Y	PREDICTED: uncharacterized protein K02A2.6-like				
Acrdi.XM_015921105.1/1-2271 | Monca.augustus.g48109.t1/1-2559	0.456293	0.434168	Y	PREDICTED: uncharacterized protein LOC107354618				
Acrdi.XM_015905688.1/1-1305 | Monca.augustus.g26824.t1/1-2166	0.465249	0.433533	Y	PREDICTED: uncharacterized protein LOC107340334				
Acrdi.XM_015915260.1/1-1317 | Monca.augustus.g46199.t1/1-1494	0.343409	0.433486	Y	PREDICTED: uncharacterized protein LOC107349159				
Acrdi.XM_015917086.1/1-441 | Monca.adi2mcaRNA27980_R0/1-375	0.218925	0.433299	Y	Transmembrane 241	2	[illegible]	[illegible]	
Acrdi.XM_015914036.1/1-1449 | Monca.augustus.g18744.t1/1-1488	0.486167	0.432951	Y	Nucleoporin Nup43				
Acrdi.XM_015903757.1/1-1290 | Monca.augustus.g25064.t1/1-1350	0.143677	0.431761	Y	restriction endonuclease subunit R	6	[illegible]	[illegible]	
Acrdi.XM_015903529.1/1-2004 | Monca.augustus.g11969.t1/1-3024	0.375949	0.430987	Y	PREDICTED: uncharacterized protein LOC107338296				
Acrdi.XM_015904434.1/1-507 | Monca.augustus.g10190.t1/1-555	0.350154	0.430969	Y	PREDICTED: uncharacterized protein C3orf30-like				
Acrdi.XM_015918325.1/1-1521 | Monca.augustus.g29389.t1/1-2103	0.323292	0.430756	Y	PREDICTED: uncharacterized protein LOC107352021 isoform X2				
Acrdi.XM_015913815.1/1-777 | Monca.augustus.g57185.t1/1-1308	0.310689	0.430614	Y	PREDICTED: uncharacterized protein LOC107347840				
Acrdi.XM_015914986.1/1-2034 | Monca.adi2mcaRNA25610_R0/1-2217	0.352232	0.430483	Y	complement component C8 beta chain	5	[illegible]	[illegible]	
Acrdi.XM_015895070.1/1-669 | Monca.augustus.g6924.t1/1-1443	0.382096	0.430379	Y	ubiquitin receptor RAD23c-				
Acrdi.XM_015915443.1/1-19317 | Monca.adi2mcaRNA26096_R0/1-2120	0.317383	0.429699	Y	microtubule-associated futsch-like				
Acrdi.XM_015922926.1/1-330 | Monca.adi2mcaRNA34504_R0/1-348	0.241446	0.429518	Y	ribonuclease P subunit p14	3	[illegible]	[illegible]	
Acrdi.XM_015900158.1/1-1065 | Monca.augustus.g47744.t1/1-900	0.167822	0.429448	Y	trace amine-associated receptor 1	1	F:GO:0004871	[illegible]	
Acrdi.XM_015894984.1/1-2712 | Monca.adi2mcaRNA41353_R0/1-2199	0.126423	0.428967	Y	Glutathione mitochondrial				
Acrdi.XM_015908363.1/1-582 | Monca.adi2mcaRNA18336_R2/1-339	0.168258	0.428763	Y	venom C-type lectin mannose binding isoform 3	3	[illegible]	[illegible]	
Acrdi.XM_015907342.1/1-621 | Monca.augustus.g31808.t1/1-591	0.515951	0.428298	Y	PREDICTED: uncharacterized protein LOC107341866				
Acrdi.XM_015902267.1/1-1377 | Monca.augustus.g71160.t1/1-2007	0.608429	0.428127	Y	PREDICTED: uncharacterized protein LOC107337146				
Acrdi.XM_015901788.1/1-2070 | Monca.augustus.g58381.t1/1-2925	0.359371	0.426739	Y	PREDICTED: uncharacterized protein LOC107336719				
Acrdi.XM_015911792.1/1-597 | Monca.augustus.g4085.t1/1-639	0.343445	0.426288	Y	Tissue inhibitor of metalloproteases	6	[illegible]	[illegible]	
Acrdi.XM_015891856.1/1-1047 | Monca.adi2mcaRNA37729_R1/1-798	0.16828	0.42624	Y	PREDICTED: uncharacterized protein LOC107327104	2	[illegible]	[illegible]	
Acrdi.XM_015911423.1/1-318 | Monca.augustus.g71135.t1/1-345	0.549912	0.426205	Y	PREDICTED: uncharacterized protein LOC107345689				
Acrdi.XM_015909432.1/1-1683 | Monca.adi2mcaRNA19534_R0/1-1644	0.317895	0.426059	Y	PREDICTED: uncharacterized protein LOC107343845 isoform X1				
Acrdi.XM_015911370.1/1-1173 | Monca.augustus.g35263.t1/1-1887	0.332162	0.424152	Y	cell wall DAN4-like isoform X1				
Acrdi.XM_015914752.1/1-1377 | Monca.augustus.g22513.t1/1-1386	0.42275	0.423717	Y	transmembrane 145-like	4	[illegible]	[illegible]	
Acrdi.XM_015892322.1/1-1206 | Monca.adi2mcaRNA38246_R2/1-834	0.187024	0.423547	Y	EGF-like repeat and discoidin I-like domain-containing 3				
Acrdi.XM_015907936.1/1-1074 | Monca.adi2mcaRNA17870_R0/1-1257	0.573068	0.423534	Y	PREDICTED: uncharacterized protein LOC107342439	3	[illegible]	[illegible]	
Acrdi.XM_015922704.1/1-543 | Monca.augustus.g46493.t1/1-540	0.430079	0.423026	Y	PREDICTED: uncharacterized protein LOC107356067 isoform X2				
Acrdi.XM_015913230.1/1-642 | Monca.adi2mcaRNA23651_R8/1-666	0.139028	0.422407	Y	Transposable element P transposase				

Acrdi.XM_015911542.1/1-1686 | Monca.augustus.g45430.t1/1-2871	0.258057	0.422004	Y	nucleolar 8	9
Acrdi.XM_015892459.1/1-3690 | Monca.augustus.g61313.t1/1-3108	0.300052	0.421338	Y	macrophage mannose receptor 1-like	4
Acrdi.XM_015896271.1/1-444 | Monca.adi2mcaRNA5105_R0/1-426	0.453505	0.421032	Y	PREDICTED: uncharacterized protein LOC107331661	
Acrdi.XM_015904358.1/1-1479 | Monca.augustus.g59958.t1/1-1530	0.403669	0.421017	Y	coilin isoform X1	1
Acrdi.XM_015908493.1/1-783 | Monca.augustus.g15581.t1/1-855	0.42816	0.42057	Y	KxDL motif-containing 1	1
Acrdi.XM_015900303.1/1-936 | Monca.augustus.g23886.t1/1-1224	0.322522	0.418898	Y	PREDICTED: uncharacterized protein LOC107335298	
Acrdi.XM_015902688.1/1-984 | Monca.adi2mcaRNA12036_R0/1-942	0.376894	0.418607	Y	PREDICTED: uncharacterized protein LOC107337503	
Acrdi.XM_015900731.1/1-1683 | Monca.augustus.g34878.t1/1-1710	0.428451	0.418082	Y	PREDICTED: uncharacterized protein LOC107335698	
Acrdi.XM_015920980.1/1-588 | Monca.adi2mcaRNA32295_R0/1-378	0.141898	0.417979	Y	PREDICTED: uncharacterized protein LOC107354493	
Acrdi.XM_015900212.1/1-606 | Monca.augustus.g62855.t1/1-1278	0.76835	0.417835	Y	PREDICTED: uncharacterized protein LOC107335218	
Acrdi.XM_015896221.1/1-507 | Monca.augustus.g23050.t1/1-459	0.292363	0.417691	Y	GNAT family acetyltransferase	5
Acrdi.XM_015917963.1/1-480 | Monca.augustus.g51554.t1/1-459	0.150285	0.417086	Y	PREDICTED: uncharacterized protein LOC107351672	
Acrdi.XM_015908937.1/1-2244 | Monca.augustus.g29214.t1/1-2541	0.268527	0.416682	Y	GRIP and coiled-coil domain-containing 2-like	
Acrdi.XM_015898752.1/1-2592 | Monca.augustus.g2079.t1/1-2604	0.437165	0.416621	Y	PREDICTED: uncharacterized protein LOC107333887	
Acrdi.XM_015906017.1/1-1416 | Monca.augustus.g41397.t1/1-1401	0.32947	0.416327	Y	GRB2-associated and regulator of MAPK -like	
Acrdi.XM_015923047.1/1-3852 | Monca.augustus.g644.t1/1-4278	0.529764	0.41594	Y	PREDICTED: uncharacterized protein LOC107356432 isoform X2	
Acrdi.XM_015923368.1/1-381 | Monca.augustus.g24895.t1/1-567	0.506982	0.415398	Y	PREDICTED: uncharacterized protein LOC107356747	
Acrdi.XM_015893934.1/1-4536 | Monca.augustus.g50880.t1/1-4401	0.468314	0.415386	Y	PREDICTED: uncharacterized protein LOC107329195 isoform X1	
Acrdi.XM_015923498.1/1-7653 | Monca.augustus.g47457.t1/1-11910	0.335516	0.415171	Y	neuroblast differentiation-associated AHNAK-like	
Acrdi.XM_015897479.1/1-1026 | Monca.augustus.g54259.t1/1-1029	0.411167	0.414678	Y	PREDICTED: uncharacterized protein LOC107332735	
Acrdi.XM_015907941.1/1-402 | Monca.augustus.g62658.t1/1-1068	0.231941	0.414365	Y	PREDICTED: uncharacterized protein LOC107342446	
Acrdi.XM_015923807.1/1-2769 | Monca.augustus.g23691.t1/1-2643	0.309422	0.414021	Y	Golgi-associated plant pathogenesis-related 1	3
Acrdi.XM_015907620.1/1-1812 | Monca.adi2mcaRNA17525_R2/1-1632	0.266911	0.413765	Y	Down syndrome cell adhesion molecule 1 homolog isoform X5	
Acrdi.XM_015916576.1/1-429 | Monca.augustus.g71223.t1/1-483	0.344251	0.412681	Y	PREDICTED: uncharacterized protein LOC107350348	
Acrdi.XM_015906489.1/1-2625 | Monca.augustus.g13120.t1/1-3342	0.342074	0.412635	Y	PREDICTED: uncharacterized protein LOC107341069 isoform X1	
Acrdi.XM_015921865.1/1-1686 | Monca.augustus.g21255.t1/1-1704	0.339779	0.412399	Y	spore wall 2-like	
Acrdi.XM_015921545.1/1-879 | Monca.augustus.g23007.t1/1-957	0.535995	0.411891	Y	PREDICTED: uncharacterized protein LOC107355029	
Acrdi.XM_015898033.1/1-450 | Monca.augustus.g29894.t1/1-480	0.193457	0.411806	Y	PREDICTED: uncharacterized protein LOC107333255	
Acrdi.XM_015892400.1/1-648 | Monca.augustus.g42232.t1/1-1563	0.347235	0.411676	Y	PREDICTED: uncharacterized protein LOC107327654	
Acrdi.XM_015914716.1/1-513 | Monca.augustus.g57982.t1/1-471	0.154129	0.411574	Y	nuclease HARBI1	
Acrdi.XM_015896526.1/1-1437 | Monca.augustus.g59721.t1/1-1275	0.143074	0.411118	Y	DUF21 domain-containing At4g14230-like	7
Acrdi.XM_015925286.1/1-582 | Monca.augustus.g31541.t1/1-714	0.534123	0.410535	Y	PREDICTED: uncharacterized protein LOC107358697	
Acrdi.XM_015899719.1/1-417 | Monca.adi2mcaRNA823_R0/1-441	0.249999	0.409902	Y	PREDICTED: uncharacterized protein LOC107334744	
Acrdi.XM_015903609.1/1-948 | Monca.augustus.g24962.t1/1-1047	0.393625	0.409779	Y	PREDICTED: uncharacterized protein LOC107338375 isoform X2	
Acrdi.XM_015918825.1/1-1929 | Monca.adi2mcaRNA29906_R1/1-1797	0.129117	0.409634	Y	PREDICTED: uncharacterized protein LOC107352495	9
Acrdi.XM_015891830.1/1-2088 | Monca.augustus.g35736.t1/1-2328	0.346053	0.409472	Y	PREDICTED: uncharacterized protein LOC107327078	
Acrdi.XM_015910408.1/1-2772 | Monca.augustus.g70898.t1/1-2790	0.439401	0.409257	Y	PREDICTED: uncharacterized protein LOC107344715	
Acrdi.XM_015918482.1/1-657 | Monca.augustus.g55057.t1/1-708	0.378343	0.409183	Y	PREDICTED: uncharacterized protein LOC107352143	
Acrdi.XM_015908000.1/1-1044 | Monca.augustus.g43120.t1/1-1119	0.424586	0.409112	Y	PREDICTED: uncharacterized protein LOC107342498	
Acrdi.XM_015921706.1/1-681 | Monca.augustus.g11335.t1/1-663	0.250899	0.40897	Y	PREDICTED: uncharacterized protein LOC107355178	
Acrdi.XM_015911983.1/1-354 | Monca.adi2mcaRNA22277_R3/1-444	0.143895	0.40878	Y	Transposon Tf2-6 poly	
Acrdi.XM_015894387.1/1-483 | Monca.augustus.g53888.t1/1-1764	0.411875	0.408769	Y	zinc finger CCHC domain-containing 7-like	
Acrdi.XM_015895392.1/1-843 | Monca.augustus.g46197.t1/1-1113	0.236024	0.408671	Y	PREDICTED: uncharacterized protein LOC107330839 isoform X2	
Acrdi.XM_015895565.1/1-981 | Monca.augustus.g2038.t1/1-1503	0.402198	0.408628	Y	PREDICTED: uncharacterized protein LOC107331032	
Acrdi.XM_015898946.1/1-1575 | Monca.augustus.g37978.t1/1-1578	0.392919	0.408194	Y	PREDICTED: uncharacterized protein LOC107334066	
Acrdi.XM_015906359.1/1-861 | Monca.augustus.g45312.t1/1-861	0.744517	0.407273	Y	PREDICTED: uncharacterized protein LOC107340965	
Acrdi.XM_015917794.1/1-642 | Monca.adi2mcaRNA28757_R0/1-630	0.399326	0.40696	Y	epidermal growth factor 8	1
Acrdi.XM_015919102.1/1-822 | Monca.augustus.g12206.t1/1-849	0.325956	0.406392	Y	balbiani ring 3-like isoform X1	7
Acrdi.XM_015905552.1/1-1014 | Monca.augustus.g24110.t1/1-1050	0.541612	0.406077	Y	PREDICTED: uncharacterized protein LOC107340189	
Acrdi.XM_015892275.1/1-1185 | Monca.augustus.g38241.t1/1-1374	0.218358	0.405994	Y	Crt-like 1	5
Acrdi.XM_015903607.1/1-1362 | Monca.augustus.g24963.t1/1-1569	0.435169	0.405459	Y	PREDICTED: uncharacterized protein LOC107338374	
Acrdi.XM_015913163.1/1-960 | Monca.augustus.g70991.t1/1-1299	0.353403	0.405393	Y	E3 ubiquitin- ligase RNF180-like	2
Acrdi.XM_015912865.1/1-684 | Monca.augustus.g5143.t1/1-816	0.235298	0.404614	Y	PREDICTED: uncharacterized protein LOC107346997	
Acrdi.XM_015901759.1/1-801 | Monca.augustus.g58374.t1/1-2739	0.492746	0.404419	Y	PREDICTED: uncharacterized protein LOC107336688	
Acrdi.XM_015911186.1/1-1305 | Monca.augustus.g24012.t1/1-1278	0.313261	0.404063	Y	Beta-1,3-galactosyltransferase 5	8
Acrdi.XM_015900817.1/1-2928 | Monca.adi2mcaRNA10013_R0/1-2883	0.290265	0.40367	Y	serine threonine- kinase N2-like isoform X1	7
Acrdi.XM_015904226.1/1-414 | Monca.augustus.g11833.t1/1-351	0.628118	0.40301	Y	PREDICTED: uncharacterized protein LOC107338981	
Acrdi.XM_015921779.1/1-600 | Monca.adi2mcaRNA33159_R7/1-591	0.128677	0.402893	Y	PREDICTED: uncharacterized protein LOC107355240	
Acrdi.XM_015894662.1/1-882 | Monca.adi2mcaRNA40974_R4/1-744	0.255707	0.402766	Y	Transposon Tf2-9 poly	
Acrdi.XM_015918245.1/1-1488 | Monca.augustus.g11682.t1/1-1482	0.321586	0.402242	Y	neuronal migration doublecortin isoform X4	5
Acrdi.XM_015924964.1/1-651 | Monca.augustus.g41164.t1/1-606	0.427961	0.40187	Y	PREDICTED: uncharacterized protein LOC107358359	
Acrdi.XM_015905833.1/1-1098 | Monca.augustus.g31768.t1/1-1926	0.241695	0.401328	Y	PREDICTED: uncharacterized protein LOC107340479	1
Acrdi.XM_015907508.1/1-720 | Monca.augustus.g17399.t1/1-915	0.446072	0.400893	Y	PREDICTED: uncharacterized protein LOC107342048	
Acrdi.XM_015908362.1/1-846 | Monca.augustus.g35189.t1/1-816	0.377307	0.400778	Y	PREDICTED: uncharacterized protein LOC107342846	
Acrdi.XM_015910390.1/1-444 | Monca.adi2mcaRNA20565_R6/1-468	0.130006	0.399413	Y	PREDICTED: uncharacterized protein LOC107344697	5
Acrdi.XM_015906296.1/1-954 | Monca.augustus.g26544.t1/1-1026	0.483149	0.399392	Y	PREDICTED: syndecan-3-like	
Acrdi.XM_015903048.1/1-3345 | Monca.adi2mcaRNA12454_R0/1-3531	0.338521	0.399158	Y	Fez family zinc finger 2	2
Acrdi.XM_015921204.1/1-915 | Monca.augustus.g14879.t1/1-1155	0.285879	0.399038	Y	treacle -like isoform X1	
Acrdi.XM_015899654.1/1-567 | Monca.augustus.g54476.t1/1-567	0.282736	0.398626	Y	PREDICTED: uncharacterized protein LOC107334687	
Acrdi.XM_015912043.1/1-1275 | Monca.adi2mcaRNA22343_R0/1-1293	0.350242	0.398471	Y	collectin-12-like isoform X2	3
Acrdi.XM_015915692.1/1-627 | Monca.augustus.g37352.t1/1-600	0.16735	0.398094	Y	EGF-like domain-containing comC isoform X1	
Acrdi.XM_015903966.1/1-900 | Monca.adi2mcaRNA13430_R0/1-1194	0.386652	0.397318	Y	PREDICTED: uncharacterized protein LOC107338733	
Acrdi.XM_015913215.1/1-1047 | Monca.augustus.g32072.t1/1-1020	0.409445	0.397033	Y	PREDICTED: uncharacterized protein LOC107347320	
Acrdi.XM_015894031.1/1-1626 | Monca.augustus.g36767.t1/1-2019	0.244463	0.396789	Y	nucleolar and coiled-body phospho 1	3
Acrdi.XM_015896005.1/1-2712 | Monca.augustus.g32498.t1/1-2715	0.197124	0.396684	Y	JAB domain-containing	
Acrdi.XM_015921614.1/1-567 | Monca.augustus.g26625.t1/1-639	0.388807	0.39657	Y	transcription factor -like	8
Acrdi.XM_015922097.1/1-1011 | Monca.augustus.g39481.t1/1-2124	0.4253	0.396322	Y	PREDICTED: uncharacterized protein LOC107355524	
Acrdi.XM_015898893.1/1-357 | Monca.augustus.g35499.t1/1-375	0.249348	0.395963	Y	PREDICTED: uncharacterized protein LOC107334018	
[illegible]	[illegible]	[illegible]	[illegible]	[illegible]	[illegible]

Acrdi.XM_015922250.1/1-1017 | Monca.augustus.g50444.t1/1-972	0.406065	0.354954	Y	ephrin domain	1	C:GO:0016020	C:membrane
Acrdi.XM_015910615.1/1-1005 | Monca.augustus.g45393.t1/1-1185	0.29526	0.35483	Y	PREDICTED: uncharacterized protein LOC107344928			
Acrdi.XM_015923613.1/1-981 | Monca.augustus.g46832.t1/1-981	0.509112	0.354785	Y	PREDICTED: uncharacterized protein LOC107356969			
Acrdi.XM_015902224.1/1-411 | Monca.adi2mcaRNA11555_R0/1-417	0.408499	0.354682	Y	60S ribosomal L28	3	F:GO:0003735; C:GO:0022625; P:GO:0006412	F:structural constituent of ribosome; C:cytosolic large ribosomal subunit; P:translation
Acrdi.XM_015913856.1/1-612 | Monca.augustus.g2817.t1/1-585	0.500878	0.354482	Y	mammalian ependymin-related 1-like	3	P:GO:0007160; F:GO:0005509; C:GO:0005576	P:cell-matrix adhesion; F:calcium ion binding; C:extracellular region
Acrdi.XM_015897817.1/1-1020 | Monca.adi2mcaRNA642_R0/1-846	0.106485	0.354469	Y	ATP-dependent DNA helicase PIF1			
Acrdi.XM_015916427.1/1-528 | Monca.augustus.g64493.t1/1-1761	0.289022	0.354434	Y	PREDICTED: uncharacterized protein LOC107350201			
Acrdi.XM_015924857.1/1-993 | Monca.augustus.g14248.t1/1-1746	0.4021	0.354253	Y	basement membrane-specific heparan sulfate proteoglycan core -like			
Acrdi.XM_015895310.1/1-810 | Monca.augustus.g48504.t1/1-915	0.367045	0.354236	Y	CUE domain-containing 2			
Acrdi.XM_015903986.1/1-3141 | Monca.adi2mcaRNA13482_R1/1-3024	0.111873	0.354195	Y	CGG triplet repeat-binding 1			
Acrdi.XM_015908712.1/1-840 | Monca.augustus.g51797.t1/1-1329	0.398459	0.354011	Y	fibrillin-1			
Acrdi.XM_015895347.1/1-645 | Monca.adi2mcaRNA41783_R5/1-486	0.121081	0.353972	Y	Coiled-coil domain-containing 50			
Acrdi.XM_015898202.1/1-348 | Monca.augustus.g29231.t1/1-360	0.56213	0.353684	Y	PREDICTED: uncharacterized protein LOC107333382			
Acrdi.XM_015901826.1/1-1026 | Monca.adi2mcaRNA1254_R0/1-1092	0.105087	0.35361	Y	kelch domain-containing 3 isoform X1	4	C:GO:0000790; C:GO:0005737; P:GO:0000469; F:GO:0003682	C:nuclear chromatin; C:cytoplasm; P:cleavage involved in rRNA processing; F:chromatin binding
Acrdi.XM_015911379.1/1-1713 | Monca.augustus.g35297.t1/1-1914	0.302976	0.353266	Y	Decapping and exoribonuclease	3	C:GO:0005634; C:GO:0005737; C:GO:0005886	C:nucleus; C:cytoplasm; C:plasma membrane
Acrdi.XM_015905808.1/1-351 | Monca.adi2mcaRNA15479_R0/1-300	0.303823	0.353233	Y	PREDICTED: uncharacterized protein LOC107340457 isoform X2			
Acrdi.XM_015899090.1/1-2265 | Monca.augustus.g26228.t1/1-2277	0.322587	0.352933	Y	probable G- coupled receptor 21	10	F:GO:0004930; C:GO:0005887; P:GO:0007218; P:GO:0007186; [illegible]	F:G-protein coupled receptor activity; C:integral component of plasma membrane; P:neuropeptide signaling pathway; P:G-protein coupled receptor signaling pathway; P:signal [illegible]
Acrdi.XM_015903178.1/1-798 | Monca.augustus.g32284.t1/1-2439	0.402599	0.352859	Y	PREDICTED: uncharacterized protein LOC107337932			
Acrdi.XM_015912619.1/1-501 | Monca.adi2mcaRNA22990_R0/1-459	0.114177	0.352699	Y	piggyBac transposable element-derived 4-like			
Acrdi.XM_015912199.1/1-1083 | Monca.augustus.g22442.t1/1-1050	0.29741	0.352472	Y	phe13-bombesin receptor-like isoform X1	6	F:GO:0004930; P:GO:0007186; P:GO:0007165; C:GO:0016020; [illegible]	F:G-protein coupled receptor activity; P:G-protein coupled receptor signaling pathway; P:signal transduction; C:membrane; C:integral component of membrane; F:signal transduc[illegible]
Acrdi.XM_015917596.1/1-1179 | Monca.augustus.g14918.t1/1-2421	0.349917	0.352274	Y	Hemicentin-1			
Acrdi.XM_015915407.1/1-1941 | Monca.augustus.g32701.t1/1-1956	0.327085	0.352266	Y	PREDICTED: uncharacterized protein LOC107349288			
Acrdi.XM_015904476.1/1-3354 | Monca.augustus.g52737.t1/1-3744	0.423854	0.352084	Y	viral A-type inclusion			
Acrdi.XM_015902677.1/1-543 | Monca.adi2mcaRNA12026_R0/1-597	0.46799	0.351435	Y	PREDICTED: uncharacterized protein LOC107337496			
Acrdi.XM_015921208.1/1-366 | Monca.augustus.g14878.t1/1-399	0.46409	0.35133	Y	threonine synthase-like 1	2	C:GO:0005739; C:GO:0005654	C:mitochondrion; C:nucleoplasm
Acrdi.XM_015917436.1/1-459 | Monca.augustus.g29304.t1/1-462	0.329668	0.351243	Y	PREDICTED: uncharacterized protein LOC107351158			
Acrdi.XM_015914539.1/1-1293 | Monca.augustus.g29165.t1/1-1308	0.33852	0.351207	Y	PREDICTED: uncharacterized protein LOC107348497			
Acrdi.XM_015916558.1/1-849 | Monca.augustus.g42813.t1/1-888	0.333871	0.351095	Y	PREDICTED: uncharacterized protein LOC107350322			
Acrdi.XM_015924354.1/1-432 | Monca.adi2mcaRNA36122_R0/1-528	0.253212	0.350864	Y	PREDICTED: uncharacterized protein LOC107357717			
Acrdi.XM_015898683.1/1-777 | Monca.augustus.g43062.t1/1-1965	0.211825	0.350482	Y	Latent-transforming growth factor beta-binding 2			
Acrdi.XM_015892057.1/1-534 | Monca.adi2mcaRNA37952_R1/1-534	0.704288	0.350305	Y	high affinity copper uptake 1	4	C:GO:0016020; C:GO:0016021; F:GO:0005375; P:GO:0035434	C:membrane; C:integral component of membrane; F:copper ion transmembrane transporter activity; P:copper ion transmembrane transport
Acrdi.XM_015911304.1/1-537 | Monca.augustus.g67459.t1/1-519	0.238458	0.35028	Y	PREDICTED: uncharacterized protein LOC107345572			
Acrdi.XM_015898270.1/1-882 | Monca.augustus.g64610.t1/1-2367	0.399525	0.349993	Y	whirlin isoform X1			
Acrdi.XM_015899832.1/1-1497 | Monca.augustus.g43430.t1/1-2343	0.357886	0.349481	Y	PREDICTED: uncharacterized protein LOC107334854			
Acrdi.XM_015895286.1/1-570 | Monca.augustus.g27561.t1/1-1017	0.308896	0.349321	Y	RISC-loading complex subunit TARBP2			

[illegible]

Acrdi.XM_015901380.1/1-10332 | Monca.augustus.g28495.t1/1-26937	0.331752	0.311993	Y	Contactin-associated -like 2	2	C:GO:0016020; C:GO:0016021	C:membrane; C:integral component of membrane
Acrdi.XM_015923544.1/1-3084 | Monca.augustus.g809.t1/1-3099	0.27945	0.311783	Y	Proto-oncogene tyrosine- kinase receptor Ret	5	P:GO:0018108; F:GO:0005524; F:GO:0004713; F:GO:0004672; [illegible]	P:peptidyl-tyrosine phosphorylation; F:ATP binding; F:protein tyrosine kinase activity; [illegible]
Acrdi.XM_015912101.1/1-2511 | Monca.adi2mcaRNA22434_R1/1-2217	0.0799328	0.311766	Y	PREDICTED: uncharacterized protein LOC107346319	4	C:GO:0005634; F:GO:0046872; F:GO:0003682; P:GO:0006357	C:nucleus; F:metal ion binding; F:chromatin binding; P:regulation of transcription from RNA polymerase [illegible]
Acrdi.XM_015894917.1/1-441 | Monca.adi2mcaRNA41271_R5/1-390	0.475442	0.311699	Y	TNF receptor-associated factor 4	9	F:GO:0004842; F:GO:0008270; P:GO:0007165; F:GO:0046872; [illegible]	F:ubiquitin-protein transferase activity; F:zinc ion binding; P:signal transduction; F:metal ion binding; [illegible]
Acrdi.XM_015920953.1/1-2277 | Monca.augustus.g53588.t1/1-2295	0.296623	0.311402	Y	ankyrin repeat			
Acrdi.XM_015897750.1/1-807 | Monca.augustus.g46329.t1/1-897	0.262235	0.311366	Y	coadhesin-like isoform X2	3	C:GO:0016020; F:GO:0005044; P:GO:0006898	C:membrane; F:scavenger receptor activity; P:receptor-mediated endocytosis
Acrdi.XM_015906841.1/1-657 | Monca.augustus.g29172.t1/1-2070	0.261889	0.311358	Y	PREDICTED: uncharacterized protein LOC107341426			
Acrdi.XM_015911283.1/1-492 | Monca.augustus.g37485.t1/1-741	0.30397	0.311189	Y	baculoviral IAP repeat-containing 1f-like isoform X1	1	C:GO:0016021	C:integral component of membrane
Acrdi.XM_015915716.1/1-954 | Monca.adi2mcaRNA26405_R0/1-810	0.222604	0.311097	Y	poly(U)-specific endoribonuclease-like	7	F:GO:0004601; F:GO:0016788; P:GO:0006979; F:GO:0004602; [illegible]	F:peroxidase activity; F:hydrolase activity, acting on ester bonds; P:response to oxidative stress; [illegible]
Acrdi.XM_015915269.1/1-699 | Monca.augustus.g15250.t1/1-699	0.272328	0.310972	Y	GTPase IMAP family member 7-like			
Acrdi.XM_015921000.1/1-3663 | Monca.augustus.g41191.t1/1-3657	0.276414	0.310828	Y	PREDICTED: uncharacterized protein LOC107354515	4	P:GO:0042273; C:GO:0016020; C:GO:0016021; P:GO:0006397	P:ribosomal large subunit biogenesis; C:membrane; C:integral component of membrane; P:mRNA [illegible]
Acrdi.XM_015914714.1/1-4701 | Monca.augustus.g36645.t1/1-9516	0.283354	0.310818	Y	Breast cancer type 2 susceptibility			
Acrdi.XM_015906613.1/1-1755 | Monca.augustus.g70208.t1/1-1743	0.282312	0.310794	Y	hemicentin-1-like isoform X1	3	P:GO:0007165; C:GO:0016020; C:GO:0016021	P:signal transduction; C:membrane; C:integral component of membrane
Acrdi.XM_015897891.1/1-1275 | Monca.augustus.g23838.t1/1-1314	0.323847	0.310706	Y	G- coupled receptor 161-like	2	F:GO:0004930; P:GO:0007165	F:G-protein coupled receptor activity; P:signal transduction
Acrdi.XM_015900241.1/1-924 | Monca.augustus.g16492.t1/1-1188	0.291804	0.310396	Y	contactin-associated like 5-4-like isoform X2			
Acrdi.XM_015912682.1/1-474 | Monca.adi2mcaRNA23004_R0/1-393	0.337818	0.310386	Y	PREDICTED: uncharacterized protein LOC107346837			
Acrdi.XM_015892228.1/1-1293 | Monca.augustus.g2819.t1/1-1491	0.254832	0.310178	Y	otolin-1-like isoform X1	1	F:GO:0005488	F:binding
Acrdi.XM_015916981.1/1-4230 | Monca.augustus.g67198.t1/1-3549	0.247288	0.309949	Y	transport and Golgi organization 6 homolog			
Acrdi.XM_015899751.1/1-936 | Monca.augustus.g66184.t1/1-963	0.25699	0.309898	Y	TRAF-type zinc finger domain-containing 1	3	F:GO:0008270; C:GO:0016020; C:GO:0016021	F:zinc ion binding; C:membrane; C:integral component of membrane
Acrdi.XM_015913458.1/1-462 | Monca.augustus.g53398.t1/1-486	0.660393	0.309829	Y	Rhs family	1	P:GO:0097264	P:self proteolysis
Acrdi.XM_015918104.1/1-867 | Monca.augustus.g9312.t1/1-1545	0.364154	0.309727	Y	serine beta-lactamase mitochondrial			
Acrdi.XM_015915101.1/1-1653 | Monca.adi2mcaRNA25749_R7/1-1491	0.0911157	0.30971	Y	RNA-directed DNA polymerase from mobile element jockey	2	C:GO:0016020; C:GO:0016021	C:membrane; C:integral component of membrane
Acrdi.XM_015919678.1/1-1203 | Monca.augustus.g23530.t1/1-1269	0.22356	0.309526	Y	PREDICTED: uncharacterized protein LOC107353299			
Acrdi.XM_015905432.1/1-756 | Monca.adi2mcaRNA15065_R1/1-819	0.28622	0.309492	Y	titin isoform X2			
Acrdi.XM_015907931.1/1-648 | Monca.augustus.g35198.t1/1-672	0.252103	0.309489	Y	PREDICTED: uncharacterized protein LOC107342423 isoform X2			
Acrdi.XM_015900773.1/1-2046 | Monca.augustus.g53992.t1/1-1968	0.289596	0.309471	Y	transcription factor Sp5-like	2	F:GO:0003676; F:GO:0046872	F:nucleic acid binding; F:metal ion binding
Acrdi.XM_015910549.1/1-3153 | Monca.augustus.g10113.t1/1-3039	0.351475	0.309234	Y	flocculation FLO11-like			
Acrdi.XM_015900007.1/1-2304 | Monca.augustus.g51851.t1/1-2394	0.223564	0.30923	Y	RB1-inducible coiled-coil 1 isoform X1			
Acrdi.XM_015902933.1/1-570 | Monca.augustus.g41335.t1/1-585	0.239215	0.308987	Y	receptor-transporting 3-like	5	P:GO:0007606; F:GO:0031849; P:GO:0006612; C:GO:0005622; [illegible]	P:sensory perception of chemical stimulus; F:olfactory receptor binding; [illegible]
Acrdi.XM_015907095.1/1-414 | Monca.augustus.g2523.t1/1-1284	0.399255	0.30892	Y	Guanine nucleotide exchange factor for Rab-3A	1	F:GO:0005085	F:guanyl-nucleotide exchange factor activity
Acrdi.XM_015915094.1/1-453 | Monca.adi2mcaRNA25746_R2/1-453	0.501497	0.308818	Y	Chromodomain Y 2	1	C:GO:0005634	C:nucleus
Acrdi.XM_015894405.1/1-894 | Monca.augustus.g29854.t1/1-819	0.209219	0.308809	Y	metaxin-2 isoform X2	2	P:GO:0006626; C:GO:0005741	P:protein targeting to mitochondrion; C:mitochondrial outer membrane
Acrdi.XM_015922956.1/1-1941 | Monca.augustus.g8025.t1/1-2151	0.236736	0.308704	Y	probable serine threonine- kinase DDB_G0268642	2	C:GO:0016020; C:GO:0016021	C:membrane; C:integral component of membrane
Acrdi.XM_015894530.1/1-846 | Monca.augustus.g67922.t1/1-747	0.522791	0.308473	Y	PREDICTED: uncharacterized protein LOC107329876 isoform X1			
Acrdi.XM_015910059.1/1-1551 | Monca.augustus.g36892.t1/1-1155	0.362145	0.30838	Y	histamine H2 receptor-like	3	P:GO:0007165; C:GO:0016020; F:GO:0004871	P:signal transduction; C:membrane; F:signal transducer activity
Acrdi.XM_015899294.1/1-567 | Monca.augustus.g16833.t1/1-1812	0.247913	0.308024	Y	golgi-associated plant pathogenesis-related 1	1	C:GO:0005576	C:extracellular region
Acrdi.XM_015904404.1/1-720 | Monca.augustus.g48065.t1/1-786	0.295637	0.307978	Y	nidogen-2 isoform X1	8	P:GO:0007160; P:GO:0006955; F:GO:0005509; F:GO:0005044; [illegible]	P:cell-matrix adhesion; P:immune response; F:calcium ion binding; [illegible]
Acrdi.XM_015898656.1/1-306 | Monca.augustus.g34599.t1/1-582	0.193493	0.307902	Y	PREDICTED: uncharacterized protein LOC107333789 isoform X1			
Acrdi.XM_015915082.1/1-750 | Monca.augustus.g64368.t1/1-936	0.24764	0.307896	Y	5-hydroxytryptamine receptor 4-like	3	F:GO:0004930; P:GO:0007154; P:GO:0044700	F:G-protein coupled receptor activity; P:cell communication; [illegible]
Acrdi.XM_015907551.1/1-1353 | Monca.augustus.g24057.t1/1-1746	0.265344	0.30782	Y	transcription factor TFIIIB component B homolog			
Acrdi.XM_015910650.1/1-546 | Monca.augustus.g58080.t1/1-696	0.252098	0.307795	Y	#NAME?	1	F:GO:0003677	F:DNA binding
Acrdi.XM_015908512.1/1-546 | Monca.augustus.g46690.t1/1-522	0.338039	0.307603	Y	PREDICTED: uncharacterized protein LOC107342988 isoform X1			
Acrdi.XM_015898980.1/1-531 | Monca.augustus.g38012.t1/1-324	0.402226	0.307514	Y	fibroblast growth factor 2	7	P:GO:0008283; P:GO:0048522; P:GO:0048731; C:GO:0005737; [illegible]	P:cell proliferation; P:positive regulation of cellular process; [illegible]
Acrdi.XM_015917304.1/1-2847 | Monca.augustus.g48824.t1/1-3396	0.315724	0.307489	Y	ubiquitin carboxyl-terminal hydrolase 45 isoform X1	9	P:GO:0016579; F:GO:0008270; F:GO:0046872; P:GO:0006508; [illegible]	P:protein deubiquitination; F:zinc ion binding; F:metal ion binding; [illegible]
Acrdi.XM_015909612.1/1-399 | Monca.augustus.g61630.t1/1-1014	0.242457	0.30731	Y	PREDICTED: uncharacterized protein LOC107344014			
Acrdi.XM_015897990.1/1-1614 | Monca.augustus.g30592.t1/1-1842	0.262009	0.307304	Y	SUMO-interacting motif-containing 1	1	F:GO:0032184	F:SUMO polymer binding
Acrdi.XM_015920056.1/1-762 | Monca.augustus.g42007.t1/1-864	0.310195	0.30729	Y	transcription factor HES-1-B-like	5	F:GO:0003677; C:GO:0005634; P:GO:0006351; P:GO:0006355; [illegible]	F:DNA binding; C:nucleus; P:transcription, DNA-templated; [illegible]
Acrdi.XM_015921483.1/1-963 | Monca.augustus.g29336.t1/1-2211	0.281701	0.307173	Y	polycystin-1-like isoform X1	5	P:GO:0050982; F:GO:0005262; C:GO:0016020; C:GO:0016021; [illegible]	P:detection of mechanical stimulus; F:calcium channel activity; [illegible]
Acrdi.XM_015914484.1/1-336 | Monca.augustus.g11465.t1/1-348	0.300464	0.307119	Y	SOSS complex subunit C homolog			
Acrdi.XM_015898010.1/1-645 | Monca.adi2mcaRNA6974_R2/1-564	0.560069	0.307101	Y	suppressor SRP40-like			
Acrdi.XM_015897373.1/1-2553 | Monca.adi2mcaRNA786_R1/1-2016	0.0978812	0.307019	Y	Pro-Pol poly			
Acrdi.XM_015892141.1/1-1068 | Monca.augustus.g4682.t1/1-1104	0.295441	0.307017	Y	somatostatin receptor type 1-like	9	F:GO:0004930; F:GO:0001588; P:GO:0001963; P:GO:0007186; [illegible]	F:G-protein coupled receptor activity; F:dopamine neurotransmitter [illegible]
Acrdi.XM_015918212.1/1-954 | Monca.augustus.g51055.t1/1-4416	0.188545	0.307017	Y	EGF-like repeat and discoidin I-like domain-containing 3			
Acrdi.XM_015921767.1/1-303 | Monca.augustus.g70551.t1/1-798	0.439687	0.306896	Y	PREDICTED: uncharacterized protein LOC107355232			
Acrdi.XM_015896430.1/1-2820 | Monca.augustus.g47641.t1/1-4014	0.334112	0.306815	Y	Collagen alpha-1(XII) chain	2	P:GO:0015786; C:GO:0005793	P:UDP-glucose transport; C:endoplasmic reticulum-Golgi intermediate [illegible]
Acrdi.XM_015916155.1/1-489 | Monca.augustus.g25393.t1/1-486	0.256365	0.306795	Y	PREDICTED: uncharacterized protein LOC107349956			
Acrdi.XM_015924632.1/1-3513 | Monca.augustus.g35407.t1/1-3756	0.365933	0.306783	Y	SH3 domain-containing 19 isoform X7	7	F:GO:0070064; P:GO:0007010; P:GO:0051044; P:GO:0022604; [illegible]	F:proline-rich region binding; P:cytoskeleton organization; [illegible]
Acrdi.XM_015921853.1/1-789 | Monca.adi2mcaRNA33253_R3/1-579	0.431162	0.306681	Y	PREDICTED: uncharacterized protein LOC107355312			
Acrdi.XM_015925310.1/1-1566 | Monca.augustus.g10734.t1/1-1701	0.329002	0.3066	Y	PREDICTED: uncharacterized protein LOC107358721			
Acrdi.XM_015902648.1/1-690 | Monca.augustus.g22753.t1/1-771	0.286341	0.3065	Y	neural cell adhesion molecule 1-like isoform X1	2	C:GO:0016020; C:GO:0016021	C:membrane; C:integral component of membrane
Acrdi.XM_015903334.1/1-765 | Monca.augustus.g29888.t1/1-729	0.306694	0.306186	Y	PREDICTED: uncharacterized protein LOC107338096	2	C:GO:0016020; C:GO:0016021	C:membrane; C:integral component of membrane
Acrdi.XM_015922000.1/1-753 | Monca.augustus.g60065.t1/1-831	0.259203	0.306084	Y	Zinc finger MYND domain-containing 10	1	F:GO:0046872	F:metal ion binding
Acrdi.XM_015917028.1/1-1593 | Monca.adi2mcaRNA27912_R0/1-1167	0.239846	0.305865	Y	potassium channel	3	F:GO:0022836; C:GO:0016021; F:GO:0005267	F:gated channel activity; C:integral component of membrane; F:potassium [illegible]
Acrdi.XM_015902092.1/1-3468 | Monca.augustus.g17200.t1/1-3573	0.245938	0.305693	Y	F-box LRR-repeat 7			
Acrdi.XM_015924034.1/1-2523 | Monca.augustus.g65406.t1/1-2643	0.265366	0.305615	Y	probable ATP-dependent helicase PF08_0048	3	C:GO:0005634; F:GO:0003700; P:GO:0006355	C:nucleus; F:transcription factor activity, sequence-specific DNA binding; [illegible]
Acrdi.XM_015906409.1/1-522 | Monca.augustus.g56781.t1/1-639	0.374483	0.305607	Y	PREDICTED: uncharacterized protein LOC107341007			
Acrdi.XM_015916925.1/1-1119 | Monca.augustus.g38696.t1/1-1149	0.447022	0.305422	Y	A disintegrin and metallo ase with thrombospondin motifs 19	3	F:GO:0003700; P:GO:0006355; F:GO:0043565	F:transcription factor activity, sequence-specific DNA binding; [illegible]
Acrdi.XM_015910423.1/1-324 | Monca.adi2mcaRNA20569_R2/1-405	0.325029	0.305379	Y	P2X purinoceptor 7-like isoform X1			
Acrdi.XM_015912240.1/1-732 | Monca.augustus.g22432.t1/1-723	0.377788	0.305301	Y	hemicentin-2-like isoform X2	2	C:GO:0016020; C:GO:0016021	C:membrane; C:integral component of membrane
Acrdi.XM_015896174.1/1-1605 | Monca.adi2mcaRNA5042_R0/1-1353	0.167142	0.305102	Y	SUMO-activating enzyme subunit 2	3	C:GO:0044428; F:GO:0005515; F:GO:0008641	C:nuclear part; F:protein binding; F:small protein activating enzyme [illegible]
Acrdi.XM_015909452.1/1-588 | Monca.augustus.g21350.t1/1-405	0.0951885	0.305026	Y	threonine-rich GPI-anchored glyco			
Acrdi.XM_015899083.1/1-1623 | Monca.augustus.g26217.t1/1-1869	0.312953	0.304894	Y	Golgi integral membrane 4 isoform X1			
Acrdi.XM_015919780.1/1-3597 | Monca.augustus.g40319.t1/1-5802	0.356751	0.30475	Y	golgin subfamily B member 1-like			
Acrdi.XM_015919076.1/1-726 | Monca.augustus.g17785.t1/1-1566	0.348984	0.304648	Y	tweety isoform X2	11	C:GO:0034707; P:GO:0006811; P:GO:1902476; C:GO:0016020; [illegible]	C:chloride channel complex; P:ion transport; P:chloride transmembrane [illegible]
Acrdi.XM_015902628.1/1-1767 | Monca.augustus.g22800.t1/1-2946	0.209703	0.304541	Y	serine arginine repetitive matrix 2-like			
Acrdi.XM_015905583.1/1-990 | Monca.augustus.g24111.t1/1-957	0.318061	0.304512	Y	FAM181B			
Acrdi.XM_015897761.1/1-456 | Monca.augustus.g46339.t1/1-450	0.343612	0.304496	Y	high affinity copper uptake 1 isoform X2	4	C:GO:0016020; C:GO:0016021; F:GO:0005375; P:GO:0035434	C:membrane; C:integral component of membrane; F:copper ion [illegible]
Acrdi.XM_015913692.1/1-1068 | Monca.augustus.g26405.t1/1-1059	0.238354	0.304013	Y	galanin receptor type 1-like	3	P:GO:0007186; C:GO:0016020; F:GO:0004871	P:G-protein coupled receptor signaling pathway; C:membrane; F:signal [illegible]
Acrdi.XM_015902579.1/1-402 | Monca.augustus.g39913.t1/1-405	0.408998	0.303994	Y	PREDICTED: uncharacterized protein LOC107337428			
Acrdi.XM_015904275.1/1-1479 | Monca.augustus.g8623.t1/1-1431	0.342288	0.303819	Y	PREDICTED: uncharacterized protein LOC107339032			
Acrdi.XM_015924087.1/1-453 | Monca.augustus.g50409.t1/1-1314	0.179278	0.303673	Y	PREDICTED: uncharacterized protein LOC107357448			
Acrdi.XM_015908857.1/1-882 | Monca.adi2mcaRNA1880_R1/1-849	0.36767	0.303604	Y	FAM35A isoform X1			
Acrdi.XM_015900329.1/1-489 | Monca.adi2mcaRNA9576_R0/1-432	0.26485	0.303597	Y	60S ribosomal L6	4	F:GO:0003735; P:GO:0002181; C:GO:0022625; P:GO:0000027	F:structural constituent of ribosome; P:cytoplasmic translation; C:cytosolic [illegible]
Acrdi.XM_015923813.1/1-1554 | Monca.augustus.g61140.t1/1-1569	0.28271	0.303452	Y	nef-associated 1			
Acrdi.XM_015925022.1/1-684 | Monca.augustus.g13172.t1/1-705	0.177202	0.303367	Y	Transient receptor potential cation channel subfamily A member 1			
Acrdi.XM_015899497.1/1-2118 | Monca.augustus.g37206.t1/1-2220	0.374625	0.303299	Y	PREDICTED: uncharacterized protein LOC107334557	2	C:GO:0016020; C:GO:0016021	C:membrane; C:integral component of membrane
Acrdi.XM_015925337.1/1-1362 | Monca.augustus.g53780.t1/1-1326	0.31273	0.303221	Y	PREDICTED: uncharacterized protein LOC107358749			
Acrdi.XM_015899865.1/1-621 | Monca.augustus.g48634.t1/1-1011	0.268557	0.303147	Y	CD63 antigen	2	P:GO:0035188; C:GO:0016020	P:hatching; C:membrane
Acrdi.XM_015911056.1/1-681 | Monca.adi2mcaRNA21267_R0/1-654	0.554062	0.303085	Y	tetraspanin-3-like	1	C:GO:0016021	C:integral component of membrane
Acrdi.XM_015916409.1/1-1497 | Monca.augustus.g64487.t1/1-2874	0.146979	0.30296	Y	OS-9 isoform X1	3	C:GO:0005788; P:GO:0030970; P:GO:0030433	C:endoplasmic reticulum lumen; P:retrograde protein transport, ER to [illegible]
Acrdi.XM_015906246.1/1-537 | Monca.augustus.g21684.t1/1-480	0.316156	0.302888	Y	PREDICTED: uncharacterized protein LOC107340847			
Acrdi.XM_015917036.1/1-909 | Monca.augustus.g45828.t1/1-1017	0.406635	0.302827	Y	PREDICTED: uncharacterized protein LOC107350797			
Acrdi.XM_015917629.1/1-1092 | Monca.adi2mcaRNA28546_R0/1-993	0.260355	0.302392	Y	collagen alpha-1(I) chain-like			
Acrdi.XM_015913920.1/1-1572 | Monca.augustus.g59530.t1/1-1467	0.330244	0.302357	Y	PREDICTED: uncharacterized protein LOC107347928	2	C:GO:0016020; C:GO:0016021	C:membrane; C:integral component of membrane
Acrdi.XM_015895802.1/1-891 | Monca.adi2mcaRNA4581_R0/1-663	0.270331	0.302233	Y	Polycystic kidney disease 1-like 2	1	F:GO:0070492	F:oligosaccharide binding
Acrdi.XM_015924828.1/1-360 | Monca.adi2mcaRNA3779_R1/1-408	0.155069	0.302203	Y	39S ribosomal mitochondrial			
Acrdi.XM_015905750.1/1-1083 | Monca.augustus.g53317.t1/1-990	0.258101	0.301981	Y	two pore potassium channel sup-9-like	2	C:GO:0016020; P:GO:0006810	C:membrane; P:transport
Acrdi.XM_015920076.1/1-525 | Monca.augustus.g24408.t1/1-933	0.200804	0.301824	Y	centromere K-like	1	C:GO:0005634	C:nucleus
Acrdi.XM_015923892.1/1-822 | Monca.augustus.g33781.t1/1-852	0.328859	0.301748	Y	PREDICTED: uncharacterized protein LOC107357256 isoform X1	3	C:GO:0005802; C:GO:0016020; C:GO:0016021	C:trans-Golgi network; C:membrane; C:integral component of membrane
Acrdi.XM_015896817.1/1-756 | Monca.adi2mcaRNA5734_R1/1-555	0.196912	0.3017	Y	EGF-like repeat and discoidin I-like domain-containing 3 isoform X5			
Acrdi.XM_015922259.1/1-1416 | Monca.augustus.g50438.t1/1-1923	0.202324	0.301555	Y	tomoregulin-2-like isoform X2			
Acrdi.XM_015900227.1/1-429 | Monca.augustus.g65825.t1/1-1377	0.337125	0.301522	Y	Golgi membrane 1-like isoform X1	2	C:GO:0016020; C:GO:0016021	C:membrane; C:integral component of membrane
Acrdi.XM_015919189.1/1-4485 | Monca.augustus.g6786.t1/1-5142	0.393844	0.301491	Y	myb transcription factor	3	F:GO:0003677; C:GO:0016020; C:GO:0016021	F:DNA binding; C:membrane; C:integral component of membrane
Acrdi.XM_015895789.1/1-795 | Monca.augustus.g47016.t1/1-807	0.197067	0.301452	Y	rhamnospondin 1			
Acrdi.XM_015901295.1/1-999 | Monca.augustus.g21093.t1/1-1005	0.366449	0.301339	Y	PREDICTED: uncharacterized protein LOC107336231			
Acrdi.XM_015891694.1/1-1746 | Monca.adi2mcaRNA37546_R0/1-1791	0.264915	0.301212	Y	PREDICTED: uncharacterized protein LOC107326942			
Acrdi.XM_015913187.1/1-369 | Monca.augustus.g71002.t1/1-375	0.293879	0.301211	Y	lymphocyte antigen 6 complex locus G6d-like isoform X2			
Acrdi.XM_015908776.1/1-612 | Monca.augustus.g24451.t1/1-534	0.171979	0.301138	Y	MORN repeat-containing 5 isoform X1			
Acrdi.XM_015905123.1/1-1404 | Monca.adi2mcaRNA14977_R2/1-1350	0.0938643	0.301075	Y	PREDICTED: uncharacterized protein LOC107339965			
Acrdi.XM_015923181.1/1-2703 | Monca.augustus.g49876.t1/1-3105	0.316009	0.30078	Y	Tyrosine- phosphatase non-receptor type	2	F:GO:0004721; P:GO:0006470	F:phosphoprotein phosphatase activity; P:protein dephosphorylation
Acrdi.XM_015910412.1/1-1491 | Monca.adi2mcaRNA29636_R9/1-1242	0.100506	0.300681	Y	RNA-directed DNA polymerase from mobile element jockey-like			
Acrdi.XM_015915900.1/1-783 | Monca.adi2mcaRNA26632_R2/1-732	0.0898763	0.300466	Y	RNA-directed DNA polymerase from mobile element jockey-like			
Acrdi.XM_015903793.1/1-3597 | Monca.augustus.g66863.t1/1-4503	0.31991	0.300408	Y	Zinc finger SWIM domain-containing 8			
Acrdi.XM_015911486.1/1-1959 | Monca.augustus.g53640.t1/1-3429	0.204111	0.300278	Y	Golgi-associated plant pathogenesis-related 1	1	C:GO:0005576	C:extracellular region
Acrdi.XM_015915080.1/1-870 | Monca.augustus.g64367.t1/1-1014	0.283012	0.300197	Y	PREDICTED: uncharacterized protein LOC107348984 isoform X1	2	C:GO:0016020; C:GO:0016021	C:membrane; C:integral component of membrane
Acrdi.XM_015923254.1/1-1377 | Monca.augustus.g1828.t1/1-1347	0.252156	0.300109	Y	mitochondrial sodium hydrogen exchanger 9B2 isoform X1	3	F:GO:0015299; C:GO:0016021; P:GO:1902600	F:solute:proton antiporter activity; C:integral component of membrane; [illegible]
Acrdi.XM_015905735.1/1-1290 | Monca.augustus.g7690.t1/1-1350	0.270263	0.300101	Y	PREDICTED: uncharacterized protein LOC107340382			
Acrdi.XM_015898302.1/1-1368 | Monca.augustus.g61847.t1/1-1344	0.286193	0.29994	Y	transmembrane glyco NMB-like	3	C:GO:0005887; C:GO:0016020; C:GO:0016021	C:integral component of plasma membrane; C:membrane; C:integral [illegible]
Acrdi.XM_015899559.1/1-5493 | Monca.augustus.g9241.t1/1-5925	0.120788	0.299801	Y	von Willebrand factor type EGF and pentraxin domain-containing 1-like	1	F:GO:0005509	F:calcium ion binding
Acrdi.XM_015921060.1/1-1545 | Monca.augustus.g52037.t1/1-2196	0.258712	0.299607	Y	Zinc finger B-box domain-containing 1	2	F:GO:0008270; C:GO:0005622	F:zinc ion binding; C:intracellular
Acrdi.XM_015920238.1/1-909 | Monca.augustus.g54332.t1/1-1050	0.248994	0.299467	Y	Collagen alpha-6(VI) chain	4	F:GO:0004867; C:GO:0005581; C:GO:0005576; C:GO:0005578	F:serine-type endopeptidase inhibitor activity; C:collagen trimer; [illegible]
Acrdi.XM_015904748.1/1-996 | Monca.augustus.g4285.t1/1-1410	0.403355	0.299291	Y	PREDICTED: uncharacterized protein LOC107339460			
Acrdi.XM_015898468.1/1-351 | Monca.augustus.g33975.t1/1-345	0.242237	0.29925	Y	54S ribosomal mitochondrial	2	F:GO:0003735; C:GO:0005762	F:structural constituent of ribosome; C:mitochondrial large ribosomal [illegible]
Acrdi.XM_015893566.1/1-564 | Monca.adi2mcaRNA39661_R5/1-591	0.324332	0.299245	Y	hemicentin-1-like			
Acrdi.XM_015904726.1/1-1965 | Monca.augustus.g36184.t1/1-1785	0.34284	0.299155	Y	Bone morphogenetic 8A			
Acrdi.XM_015914559.1/1-2826 | Monca.augustus.g29131.t1/1-3084	0.274868	0.299142	Y	PREDICTED: uncharacterized protein LOC107348518			
Acrdi.XM_015917331.1/1-1461 | Monca.augustus.g25540.t1/1-3870	0.311917	0.299131	Y	Fibroblast growth factor receptor 3	2	F:GO:0004713; P:GO:0016310	F:protein tyrosine kinase activity; P:phosphorylation
Acrdi.XM_015898371.1/1-3900 | Monca.augustus.g60821.t1/1-3027	0.228258	0.299081	Y	integrator complex subunit 3-like	8	P:GO:0006281; P:GO:0016180; F:GO:0005515; P:GO:0007093; [illegible]	P:DNA repair; P:snRNA processing; F:protein binding; [illegible]
Acrdi.XM_015904700.1/1-498 | Monca.augustus.g36232.t1/1-930	0.256418	0.298997	Y	tRNA wybutosine-synthesizing 3 homolog			
Acrdi.XM_015898216.1/1-1392 | Monca.augustus.g8194.t1/1-2955	0.280197	0.298913	Y	fibronectin type III domain-containing -like	2	C:GO:0016020; C:GO:0016021	C:membrane; C:integral component of membrane
Acrdi.XM_015913037.1/1-780 | Monca.augustus.g15091.t1/1-798	0.337177	0.298802	Y	PREDICTED: uncharacterized protein LOC107347160			
Acrdi.XM_015907189.1/1-921 | Monca.adi2mcaRNA17009_R0/1-807	0.249525	0.298672	Y	beta-lactamase 2	2	F:GO:0046872; F:GO:0016787	F:metal ion binding; F:hydrolase activity
Acrdi.XM_015908496.1/1-1614 | Monca.augustus.g33949.t1/1-1707	0.286797	0.298477	Y	wiskott-Aldrich syndrome homolog	1	P:GO:0030036	P:actin cytoskeleton organization
Acrdi.XM_015916081.1/1-498 | Monca.augustus.g35048.t1/1-564	0.241423	0.298303	Y	APOPT family mitochondrial			
Acrdi.XM_015896805.1/1-1413 | Monca.augustus.g49292.t1/1-1692	0.124729	0.298115	Y	tumor p73 isoform X6	7	F:GO:0003677; C:GO:0005634; F:GO:0003700; P:GO:0051262; [illegible]	F:DNA binding; C:nucleus; F:transcription factor activity, sequence-specific [illegible]
Acrdi.XM_015905958.1/1-1068 | Monca.augustus.g69509.t1/1-2520	0.339503	0.29809	Y	PREDICTED: uncharacterized protein LOC107340594	3	F:GO:0000166; F:GO:0003676; F:GO:0016779	F:nucleotide binding; F:nucleic acid binding; F:nucleotidyltransferase [illegible]
Acrdi.XM_015925334.1/1-648 | Monca.augustus.g66554.t1/1-651	0.290287	0.29796	Y	Homeobox Hox-D3	6	C:GO:0005634; P:GO:0006355; F:GO:0043565; P:GO:0007420; [illegible]	C:nucleus; P:regulation of transcription, DNA-templated; [illegible]
Acrdi.XM_015891692.1/1-918 | Monca.augustus.g55368.t1/1-1332	0.285905	0.297847	Y	receptor for egg jelly precursor	2	C:GO:0016020; C:GO:0016021	C:membrane; C:integral component of membrane
Acrdi.XM_015923234.1/1-537 | Monca.augustus.g1834.t1/1-498	0.475047	0.297841	Y	NAD-dependent deacetylase sirtuin-1			
Acrdi.XM_015917298.1/1-690 | Monca.augustus.g9523.t1/1-537	0.246458	0.297837	Y	adhesion G- coupled receptor D1-like	4	F:GO:0004930; P:GO:0007186; C:GO:0016021; P:GO:0007166	F:G-protein coupled receptor activity; P:G-protein coupled receptor [illegible]
Acrdi.XM_015901763.1/1-2250 | Monca.augustus.g12070.t1/1-2307	0.273684	0.29781	Y	Ankyrin repeat domain-containing 34B			
Acrdi.XM_015894169.1/1-408 | Monca.adi2mcaRNA40398_R1/1-405	0.236936	0.297804	Y	Galactosamine (N-acetyl)-6-sulfate sulfatase	6	C:GO:0070062; F:GO:0003943; F:GO:0046872; F:GO:0043890; [illegible]	C:extracellular exosome; F:N-acetylgalactosamine-4-sulfatase activity; [illegible]
Acrdi.XM_015908604.1/1-945 | Monca.adi2mcaRNA18601_R0/1-603	0.208957	0.297784	Y	Short-chain collagen C4			
Acrdi.XM_015924781.1/1-453 | Monca.augustus.g5739.t1/1-453	0.0867166	0.297678	Y	GPI transamidase component PIG-S			
Acrdi.XM_015908204.1/1-3423 | Monca.augustus.g44727.t1/1-5619	0.314572	0.297527	Y	PREDICTED: uncharacterized protein LOC107342713			
Acrdi.XM_015902659.1/1-2880 | Monca.augustus.g65489.t1/1-3663	0.337052	0.297508	Y	Hermansky-Pudlak syndrome 4	16	P:GO:0007040; F:GO:0042803; C:GO:0042827; P:GO:1903232; [illegible]	P:lysosome organization; F:protein homodimerization activity; [illegible]
Acrdi.XM_015919114.1/1-1077 | Monca.augustus.g35177.t1/1-2523	0.21087	0.297432	Y	MCM10 homolog	4	F:GO:0003688; P:GO:0006270; C:GO:0031298; F:GO:0003697	F:DNA replication origin binding; P:DNA replication initiation; [illegible]
Acrdi.XM_015909490.1/1-2493 | Monca.adi2mcaRNA19558_R0/1-2067	0.168426	0.297346	Y	ATP-dependent RNA helicase DDX1			
Acrdi.XM_015918310.1/1-813 | Monca.augustus.g29435.t1/1-1911	0.171343	0.29725	Y	ecdysoneless homolog isoform X1			
Acrdi.XM_015891716.1/1-1707 | Monca.augustus.g52057.t1/1-2865	0.222524	0.297048	Y	G patch domain-containing 1	2	F:GO:0003676; P:GO:0006397	F:nucleic acid binding; P:mRNA processing
Acrdi.XM_015904521.1/1-462 | Monca.augustus.g33571.t1/1-603	0.4102	0.29686	Y	60S ribosomal L13a	4	F:GO:0003735; F:GO:0003729; C:GO:0022625; P:GO:0006412	F:structural constituent of ribosome; F:mRNA binding; [illegible]
Acrdi.XM_015913012.1/1-1551 | Monca.augustus.g45144.t1/1-1470	0.321572	0.296681	Y	flocculation FLO11-like	1	C:GO:0016021	C:integral component of membrane
Acrdi.XM_015916176.1/1-798 | Monca.augustus.g25388.t1/1-1413	0.285626	0.29665	Y	Glutaredoxin domain-containing cysteine-rich 1			
Acrdi.XM_015895827.1/1-1293 | Monca.augustus.g63629.t1/1-1296	0.29941	0.296634	Y	Glioma tumor suppressor candidate region gene 2			
Acrdi.XM_015910643.1/1-753 | Monca.augustus.g31892.t1/1-942	0.137073	0.29655	Y	schlafen 1	1	F:GO:0005524	F:ATP binding
Acrdi.XM_015904523.1/1-582 | Monca.augustus.g33572.t1/1-687	0.343964	0.296476	Y	PREDICTED: uncharacterized protein LOC107339256	2	P:GO:0048598; C:GO:0072669	P:embryonic morphogenesis; C:tRNA-splicing ligase complex
Acrdi.XM_015908673.1/1-1485 | Monca.augustus.g53870.t1/1-1782	0.377893	0.296469	Y	class III chitinase 1	5	F:GO:0003824; C:GO:0016020; C:GO:0044464; F:GO:0051536; [illegible]	F:catalytic activity; C:membrane; C:cell part; F:iron-sulfur cluster binding; [illegible]
Acrdi.XM_015904118.1/1-1416 | Monca.augustus.g36703.t1/1-2322	0.261279	0.296403	Y	Ankyrin repeat and zinc finger domain-containing 1	5	F:GO:0004843; P:GO:0072671; C:GO:0036266; P:GO:0071630; [illegible]	F:thiol-dependent ubiquitin-specific protease activity; [illegible]
Acrdi.XM_015924427.1/1-2481 | Monca.augustus.g27456.t1/1-3261	0.203714	0.296375	Y	RNA exonuclease			
Acrdi.XM_015905329.1/1-522 | Monca.augustus.g32915.t1/1-510	0.450223	0.296319	Y	PREDICTED: uncharacterized protein LOC107339973			
Acrdi.XM_015900204.1/1-642 | Monca.augustus.g45847.t1/1-672	0.324515	0.296319	Y	achacin-like isoform X3			
Acrdi.XM_015923594.1/1-2280 | Monca.augustus.g61157.t1/1-2766	0.204073	0.296076	Y	Low-density lipo receptor-related 4	1	F:GO:0005509	F:calcium ion binding
Acrdi.XM_015892403.1/1-1140 | Monca.augustus.g57168.t1/1-1134	0.345919	0.296025	Y	PREDICTED: uncharacterized protein LOC107327658			
Acrdi.XM_015908405.1/1-600 | Monca.augustus.g34815.t1/1-1500	0.275751	0.296005	Y	PREDICTED: uncharacterized protein LOC107342894			
Acrdi.XM_015891947.1/1-1497 | Monca.augustus.g33209.t1/1-2511	0.280815	0.295985	Y	collagen alpha-5(VI) chain-	1	F:GO:0005509	F:calcium ion binding
Acrdi.XM_015923325.1/1-3069 | Monca.augustus.g36084.t1/1-5040	0.390558	0.295939	Y	unhealthy ribosome biogenesis 2 homolog			
Acrdi.XM_015898502.1/1-738 | Monca.augustus.g3320.t1/1-738	0.121407	0.295877	Y	Histone	3	F:GO:0003677; C:GO:0000790; P:GO:0006323	F:DNA binding; C:nuclear chromatin; P:DNA packaging
Acrdi.XM_015894342.1/1-318 | Monca.augustus.g22264.t1/1-2880	0.204659	0.295862	Y	PREDICTED: uncharacterized protein LOC107329670			
Acrdi.XM_015896458.1/1-576 | Monca.adi2mcaRNA5307_R0/1-495	0.298604	0.295612	Y	PREDICTED: uncharacterized protein LOC107331810			
Acrdi.XM_015911074.1/1-1470 | Monca.adi2mcaRNA21295_R3/1-1356	0.069006	0.295358	Y	PREDICTED: uncharacterized protein LOC107345372	9	C:GO:0005634; F:GO:0030170; F:GO:0046872; F:GO:0003824; [illegible]	C:nucleus; F:pyridoxal phosphate binding; F:metal ion binding; [illegible]
Acrdi.XM_015923830.1/1-339 | Monca.augustus.g33433.t1/1-336	0.234349	0.295233	Y	thioredoxin-	9	F:GO:0015035; C:GO:0005739; P:GO:0006662; F:GO:0016671; [illegible]	F:protein disulfide oxidoreductase activity; C:mitochondrion; [illegible]
Acrdi.XM_015905501.1/1-603 | Monca.augustus.g55575.t1/1-933	0.0961612	0.295097	Y	PREDICTED: uncharacterized protein LOC107340141			
Acrdi.XM_015919256.1/1-1176 | Monca.augustus.g60220.t1/1-1182	0.254336	0.295085	Y	5-hydroxytryptamine receptor 7-like	1	P:GO:0009987	P:cellular process
Acrdi.XM_015907960.1/1-2205 | Monca.augustus.g31005.t1/1-2463	0.322578	0.294976	Y	proline-rich 36-like			
Acrdi.XM_015912737.1/1-750 | Monca.augustus.g43030.t1/1-1176	0.419154	0.294649	Y	PREDICTED: uncharacterized protein LOC107346881			
Acrdi.XM_015915297.1/1-519 | Monca.augustus.g25268.t1/1-426	0.249415	0.294607	Y	glia maturation factor beta	4	F:GO:0003779; P:GO:0034316; C:GO:0005622; F:GO:0071933	F:actin binding; P:negative regulation of Arp2/3 complex-mediated actin [illegible]
Acrdi.XM_015906537.1/1-501 | Monca.augustus.g21902.t1/1-960	0.355188	0.294459	Y	PREDICTED: uncharacterized protein LOC107341116			
Acrdi.XM_015919820.1/1-786 | Monca.augustus.g41935.t1/1-1920	0.23083	0.294424	Y	PREDICTED: uncharacterized protein LOC107353490	5	P:GO:0001189; C:GO:0005668; F:GO:0001187; F:GO:0001164; [illegible]	P:RNA polymerase I transcriptional preinitiation complex assembly [illegible]
Acrdi.XM_015902340.1/1-555 | Monca.adi2mcaRNA11670_R2/1-540	0.0869089	0.294416	Y	reverse	2	F:GO:0003964; P:GO:0006278	F:RNA-directed DNA polymerase activity; P:RNA-dependent DNA [illegible]
Acrdi.XM_015923709.1/1-474 | Monca.augustus.g34080.t1/1-441	0.0901182	0.294395	Y	PREDICTED: uncharacterized protein LOC107357065	6	F:GO:0003676; C:GO:0005813; C:GO:0016020; C:GO:0016021; [illegible]	F:nucleic acid binding; C:centrosome; C:membrane; [illegible]
Acrdi.XM_015900498.1/1-1017 | Monca.augustus.g26438.t1/1-2310	0.332144	0.294171	Y	DNA excision repair ERCC-6-like 2 isoform X1			
Acrdi.XM_015915248.1/1-723 | Monca.augustus.g15251.t1/1-735	0.27681	0.294153	Y	GTPase IMAP family member 7-like	3	F:GO:0005525; P:GO:0008150; C:GO:0005575	F:GTP binding; P:biological_process; C:cellular_component
Acrdi.XM_015913094.1/1-975 | Monca.augustus.g25095.t1/1-1110	0.262158	0.294002	Y	myc proto-oncogene	4	C:GO:0005634; F:GO:0003700; P:GO:0006355; F:GO:0046983	C:nucleus; F:transcription factor activity, sequence-specific DNA binding; [illegible]
Acrdi.XM_015902877.1/1-1161 | Monca.adi2mcaRNA12219_R0/1-1143	0.240004	0.293699	Y	neurogenic locus Notch -like isoform X4			
Acrdi.XM_015903399.1/1-990 | Monca.augustus.g36018.t1/1-984	0.319719	0.293692	Y	probable G- coupled receptor No9	3	F:GO:0004930; P:GO:0007154; P:GO:0044700	F:G-protein coupled receptor activity; P:cell communication; [illegible]
Acrdi.XM_015904250.1/1-3156 | Monca.adi2mcaRNA13789_R2/1-2373	0.448893	0.29363	Y	RNA-directed DNA polymerase from transposon X-element	2	P:GO:0006508; F:GO:0004252	P:proteolysis; F:serine-type endopeptidase activity
Acrdi.XM_015896559.1/1-3135 | Monca.augustus.g62601.t1/1-3078	0.325648	0.293572	Y	poly(ADP-ribose) glycohydrolase-like	3	P:GO:0005975; F:GO:0004649; F:GO:0016787	P:carbohydrate metabolic process; F:poly(ADP-ribose) glycohydrolase [illegible]
Acrdi.XM_015901879.1/1-2166 | Monca.augustus.g20165.t1/1-2403	0.24437	0.293429	Y	G2 M phase-specific E3 ubiquitin- ligase	4	F:GO:0004842; F:GO:0008270; F:GO:0046872; F:GO:0016874	F:ubiquitin-protein transferase activity; F:zinc ion binding; [illegible]
Acrdi.XM_015904033.1/1-1170 | Monca.augustus.g2932.t1/1-1167	0.250451	0.293417	Y	allatostatin-A receptor-like	3	P:GO:0007165; C:GO:0016020; F:GO:0004871	P:signal transduction; C:membrane; F:signal transducer activity
Acrdi.XM_015921344.1/1-774 | Monca.adi2mcaRNA32685_R1/1-738	0.0882788	0.2933	Y	myb SANT-like DNA-binding domain-containing 4	2	C:GO:0005634; P:GO:0006915	C:nucleus; P:apoptotic process
Acrdi.XM_015894551.1/1-354 | Monca.augustus.g35357.t1/1-477	0.219218	0.293281	Y	TNF receptor-associated factor 3-like	6	F:GO:0004842; F:GO:0008270; F:GO:0046872; P:GO:0007165; [illegible]	F:ubiquitin-protein transferase activity; F:zinc ion binding; [illegible]
Acrdi.XM_015920111.1/1-642 | Monca.augustus.g28554.t1/1-1344	0.275962	0.293257	Y	caprin-1-like isoform X1			
Acrdi.XM_015900308.1/1-972 | Monca.augustus.g23876.t1/1-978	0.20363	0.293006	Y	ribosome-binding 1-like			
Acrdi.XM_015912665.1/1-978 | Monca.augustus.g53949.t1/1-885	0.290333	0.292765	Y	type II cytoskeletal 1-like			
Acrdi.XM_015917502.1/1-642 | Monca.augustus.g26552.t1/1-645	0.284824	0.292571	Y	MICOS complex subunit Mic27	6	P:GO:0042407; C:GO:0005739; C:GO:0016020; C:GO:0016021; [illegible]	P:cristae formation; C:mitochondrion; C:membrane; [illegible]
Acrdi.XM_015924136.1/1-1287 | Monca.augustus.g62405.t1/1-2439	0.284896	0.292542	Y	Rho guanine nucleotide exchange factor 10			
Acrdi.XM_015904852.1/1-567 | Monca.adi2mcaRNA14391_R0/1-591	0.27649	0.292415	Y	PREDICTED: uncharacterized protein LOC107339542			
Acrdi.XM_015896697.1/1-648 | Monca.adi2mcaRNA5543_R0/1-513	0.209061	0.292174	Y	sentrin-specific protease 1-like	4	C:GO:0005634; P:GO:0016926; F:GO:0004175; F:GO:0016929	C:nucleus; P:protein desumoylation; F:endopeptidase activity; [illegible]
Acrdi.XM_015922490.1/1-1218 | Monca.augustus.g60690.t1/1-1203	0.259538	0.292057	Y	PREDICTED: uncharacterized protein LOC107355870			
Acrdi.XM_015896011.1/1-738 | Monca.augustus.g32492.t1/1-1974	0.278507	0.291974	Y	PREDICTED: uncharacterized protein LOC107331432	2	C:GO:0016020; C:GO:0016021	C:membrane; C:integral component of membrane
Acrdi.XM_015919201.1/1-660 | Monca.augustus.g54961.t1/1-660	0.302487	0.291869	Y	urokinase plasminogen activator surface receptor-like			
Acrdi.XM_015924740.1/1-915 | Monca.augustus.g4177.t1/1-909	0.196506	0.291842	Y	Golgi-associated plant pathogenesis-related 1	1	C:GO:0005576	C:extracellular region
Acrdi.XM_015909323.1/1-1980 | Monca.augustus.g27148.t1/1-1668	0.329176	0.291806	Y	D-inositol 3-phosphate glycosyltransferase-like isoform X2			
Acrdi.XM_015904154.1/1-846 | Monca.augustus.g8498.t1/1-834	0.275065	0.291743	Y	PREDICTED: uncharacterized protein LOC107338897			
Acrdi.XM_015913065.1/1-708 | Monca.augustus.g25066.t1/1-1722	0.267405	0.291679	Y	harmonin isoform X2	2	P:GO:0007605; F:GO:0051015	P:sensory perception of sound; F:actin filament binding
Acrdi.XM_015914523.1/1-678 | Monca.augustus.g27287.t1/1-693	0.348994	0.291529	Y	PREDICTED: uncharacterized protein LOC107348482			
Acrdi.XM_015910583.1/1-3408 | Monca.augustus.g45401.t1/1-5517	0.29115	0.291421	Y	DNA annealing helicase and endonuclease ZRANB3	6	F:GO:0003676; P:GO:0090305; F:GO:0005524; F:GO:0008270; [illegible]	F:nucleic acid binding; P:nucleic acid phosphodiester bond hydrolysis; [illegible]
Acrdi.XM_015904383.1/1-2625 | Monca.augustus.g48077.t1/1-2337	0.286854	0.291417	Y	endothelin-converting enzyme 2-like isoform X3	1	P:GO:0070285	P:pigment cell development
Acrdi.XM_015904936.1/1-1734 | Monca.augustus.g40461.t1/1-1749	0.311264	0.291416	Y	zinc C3HC4 type (RING finger) domain containing	2	F:GO:0008270; F:GO:0046872	F:zinc ion binding; F:metal ion binding
Acrdi.XM_015909757.1/1-708 | Monca.augustus.g46912.t1/1-693	0.284505	0.2912	Y	PREDICTED: uncharacterized protein LOC107344143			
Acrdi.XM_015910983.1/1-3192 | Monca.augustus.g63050.t1/1-3981	0.428002	0.291139	Y	centrosomal of 295 kDa-like isoform X15			
Acrdi.XM_015910674.1/1-1308 | Monca.augustus.g37483.t1/1-1527	0.277868	0.291093	Y	calponin homology domain-containing DDB_G0272472-like			
Acrdi.XM_015892462.1/1-1146 | Monca.augustus.g56728.t1/1-1098	0.385448	0.291036	Y	PREDICTED: uncharacterized protein LOC107327721 isoform X1			
Acrdi.XM_015920332.1/1-525 | Monca.augustus.g36357.t1/1-546	0.154443	0.290983	Y	tyrosine- kinase BTK-like	1	F:GO:0046872	F:metal ion binding
Acrdi.XM_015921104.1/1-927 | Monca.adi2mcaRNA32436_R0/1-1155	0.369404	0.290949	Y	PREDICTED: uncharacterized protein LOC107354617			
Acrdi.XM_015899620.1/1-1014 | Monca.augustus.g54469.t1/1-1314	0.300975	0.290944	Y	leukocyte tyrosine kinase receptor-like			
Acrdi.XM_015906525.1/1-951 | Monca.augustus.g38396.t1/1-1128	0.167162	0.290824	Y	26S proteasome non-ATPase regulatory subunit 13	2	C:GO:0022624; P:GO:0009987	C:proteasome accessory complex; P:cellular process
Acrdi.XM_015896639.1/1-6654 | Monca.augustus.g48536.t1/1-9402	0.326936	0.290678	Y	polycystic kidney disease 1-like 1	6	P:GO:0050982; F:GO:0005262; F:GO:0005509; C:GO:0016020; [illegible]	P:detection of mechanical stimulus; F:calcium channel activity; [illegible]
Acrdi.XM_015914753.1/1-507 | Monca.augustus.g22519.t1/1-492	0.248155	0.290625	Y	fibroblast growth factor 1 isoform X2	22	P:GO:0043406; P:GO:0010594; P:GO:0007165; P:GO:0009891; [illegible]	P:positive regulation of MAP kinase activity; P:regulation of endothelial [illegible]
Acrdi.XM_015891743.1/1-1293 | Monca.augustus.g49609.t1/1-1296	0.269034	0.290581	Y	Acyl- -binding domain-containing 5A			
Acrdi.XM_015916391.1/1-1956 | Monca.augustus.g52620.t1/1-1917	0.401053	0.290536	Y	trithorax group osa-like	1	F:GO:0051087	F:chaperone binding
Acrdi.XM_015900400.1/1-408 | Monca.augustus.g49645.t1/1-378	0.355148	0.290528	Y	collagen alpha-6(VI) chain-like			
Acrdi.XM_015902578.1/1-612 | Monca.augustus.g39911.t1/1-645	0.361664	0.290516	Y	PREDICTED: uncharacterized protein LOC107337427	2	C:GO:0016020; C:GO:0016021	C:membrane; C:integral component of membrane
Acrdi.XM_015905443.1/1-2436 | Monca.augustus.g33961.t1/1-2637	0.297393	0.290514	Y	PREDICTED: uncharacterized protein LOC107340091 isoform X2			
Acrdi.XM_015909438.1/1-2787 | Monca.augustus.g45724.t1/1-4131	0.31107	0.290506	Y	Telomerase reverse transcriptase	4	F:GO:0003677; F:GO:0003721; F:GO:0003964; P:GO:0006278	F:DNA binding; F:telomerase RNA reverse transcriptase activity; [illegible]
Acrdi.XM_015908930.1/1-588 | Monca.augustus.g31859.t1/1-3801	0.24965	0.290411	Y	PREDICTED: extensin-2-like			
Acrdi.XM_015920160.1/1-405 | Monca.augustus.g26436.t1/1-435	0.107765	0.290398	Y	cAMP-specific 3 ,5 -cyclic isoform F-like isoform X1	3	F:GO:0046872; P:GO:0007165; F:GO:0004114	F:metal ion binding; P:signal transduction; F:3',5'-cyclic-nucleotide [illegible]
Acrdi.XM_015908096.1/1-4680 | Monca.augustus.g33239.t1/1-5058	0.246476	0.290243	Y	Mucin	6	P:GO:0007160; F:GO:0005509; C:GO:0016020; C:GO:0016021; [illegible]	P:cell-matrix adhesion; F:calcium ion binding; C:membrane; [illegible]
Acrdi.XM_015910421.1/1-1188 | Monca.augustus.g26326.t1/1-1155	0.276979	0.290136	Y	prostacyclin receptor	8	F:GO:0004930; F:GO:0004955; F:GO:0004957; P:GO:0007186; [illegible]	F:G-protein coupled receptor activity; F:prostaglandin receptor activity; [illegible]
Acrdi.XM_015907277.1/1-414 | Monca.augustus.g15571.t1/1-1560	0.234365	0.290076	Y	PREDICTED: uncharacterized protein LOC107341808			
Acrdi.XM_015912774.1/1-303 | Monca.adi2mcaRNA23142_R0/1-321	0.454667	0.290072	Y	PREDICTED: uncharacterized protein LOC107346912			
Acrdi.XM_015916173.1/1-1407 | Monca.augustus.g43210.t1/1-1425	0.160975	0.290063	Y	PREDICTED: uncharacterized protein LOC107349977			
Acrdi.XM_015908983.1/1-939 | Monca.augustus.g61791.t1/1-975	0.233179	0.290019	Y	methyl- -binding domain 4	4	F:GO:0003677; P:GO:0006281; C:GO:0005634; F:GO:0003824	F:DNA binding; P:DNA repair; C:nucleus; F:catalytic activity
Acrdi.XM_015918446.1/1-1371 | Monca.augustus.g55077.t1/1-2010	0.311858	0.28993	Y	neuronal acetylcholine receptor subunit alpha-6-like	9	P:GO:0099565; C:GO:0030425; C:GO:0098794; P:GO:0006812; [illegible]	P:chemical synaptic transmission, postsynaptic; C:dendrite; [illegible]
Acrdi.XM_015917534.1/1-378 | Monca.augustus.g25122.t1/1-660	0.333463	0.289798	Y	Ras-related Rab-35	2	P:GO:0006904; P:GO:0009306	P:vesicle docking involved in exocytosis; P:protein secretion
Acrdi.XM_015920183.1/1-1266 | Monca.augustus.g52980.t1/1-330	0.0651679	0.289706	Y	RNA-directed DNA polymerase from mobile element jockey			
Acrdi.XM_015899831.1/1-1572 | Monca.augustus.g52526.t1/1-1626	0.487044	0.289651	Y	Gametocyte-specific factor 1			
Acrdi.XM_015897734.1/1-1506 | Monca.augustus.g50752.t1/1-1569	0.264263	0.289582	Y	PREDICTED: uncharacterized protein LOC107332971			
Acrdi.XM_015915558.1/1-1137 | Monca.augustus.g31915.t1/1-2130	0.343045	0.28951	Y	Mitochondrial dynamics MID51	4	F:GO:0000166; F:GO:0016779; C:GO:0005737; F:GO:0043167	F:nucleotide binding; F:nucleotidyltransferase activity; C:cytoplasm; [illegible]
Acrdi.XM_015897969.1/1-1365 | Monca.adi2mcaRNA6996_R0/1-1272	0.0829391	0.289493	Y	THAP domain-containing 10	7	P:GO:0055085; P:GO:0007154; C:GO:0016020; P:GO:0006816; [illegible]	P:transmembrane transport; P:cell communication; C:membrane; [illegible]
Acrdi.XM_015920556.1/1-3087 | Monca.adi2mcaRNA31835_R0/1-3159	0.372779	0.289491	Y	TATA element modulatory factor			
Acrdi.XM_015918801.1/1-468 | Monca.augustus.g43817.t1/1-465	0.292365	0.28948	Y	PREDICTED: uncharacterized protein LOC107352472			
Acrdi.XM_015900980.1/1-1800 | Monca.augustus.g39836.t1/1-2166	0.37576	0.289407	Y	N-acetyltransferase ESCO2			
Acrdi.XM_015917095.1/1-1056 | Monca.adi2mcaRNA27993_R7/1-1089	0.185529	0.288821	Y	PREDICTED: uncharacterized protein K02A2.6-like			
Acrdi.XM_015899895.1/1-1134 | Monca.augustus.g32804.t1/1-1194	0.299356	0.28878	Y	Secretion-regulating guanine nucleotide exchange factor			
Acrdi.XM_015912447.1/1-2757 | Monca.augustus.g24148.t1/1-2793	0.293406	0.288762	Y	PREDICTED: uncharacterized protein LOC107346644			
Acrdi.XM_015896828.1/1-1422 | Monca.augustus.g33190.t1/1-3876	0.366526	0.288759	Y	uncharacterized serine-rich -like			
Acrdi.XM_015919849.1/1-870 | Monca.augustus.g4018.t1/1-774	0.58563	0.288447	Y	uncharacterized transposon-derived			
Acrdi.XM_015902644.1/1-909 | Monca.augustus.g22760.t1/1-1818	0.260388	0.288395	Y	leucine-rich repeat-containing	11	P:GO:0008063; F:GO:0004888; P:GO:0007352; P:GO:0007165; [illegible]	P:Toll signaling pathway; F:transmembrane signaling receptor activity; [illegible]
Acrdi.XM_015899268.1/1-522 | Monca.augustus.g579.t1/1-453	0.344598	0.288119	Y	PREDICTED: uncharacterized protein LOC107334336			
Acrdi.XM_015911684.1/1-1206 | Monca.augustus.g24271.t1/1-969	0.21754	0.288056	Y	PIN2 TERF1-interacting telomerase inhibitor 1 isoform X1	4	F:GO:0003676; C:GO:0005730; P:GO:0051974; F:GO:0010521	F:nucleic acid binding; C:nucleolus; P:negative regulation of telomerase [illegible]
Acrdi.XM_015906792.1/1-10371 | Monca.augustus.g60647.t1/1-11196	0.328735	0.288035	Y	centromere-associated E-like			
Acrdi.XM_015906432.1/1-1002 | Monca.augustus.g51501.t1/1-999	0.230193	0.287908	Y	FRAS1-related extracellular matrix 1 isoform X1	5	P:GO:0007160; C:GO:0005604; P:GO:0007154; F:GO:0005509; [illegible]	P:cell-matrix adhesion; C:basement membrane; P:cell communication; [illegible]
Acrdi.XM_015913413.1/1-474 | Monca.augustus.g58017.t1/1-432	0.210452	0.287838	Y	PREDICTED: uncharacterized protein LOC107347472 isoform X1			
Acrdi.XM_015914525.1/1-1467 | Monca.augustus.g48152.t1/1-3609	0.290974	0.287697	Y	PREDICTED: uncharacterized protein LOC107348474			
Acrdi.XM_015914521.1/1-1425 | Monca.augustus.g27276.t1/1-1422	0.224738	0.287695	Y	lisH domain-containing -like			
Acrdi.XM_015898195.1/1-6993 | Monca.augustus.g29224.t1/1-6951	0.349187	0.287688	Y	zinc finger 106-like isoform X3			
Acrdi.XM_015896193.1/1-798 | Monca.augustus.g33687.t1/1-1299	0.243376	0.287643	Y	Suppressor of cytokine signaling 6	1	P:GO:0050794	P:regulation of cellular process
Acrdi.XM_015918188.1/1-1500 | Monca.augustus.g4379.t1/1-1893	0.341233	0.28751	Y	PREDICTED: uncharacterized protein LOC107351885	2	C:GO:0016020; C:GO:0016021	C:membrane; C:integral component of membrane
Acrdi.XM_015918354.1/1-3423 | Monca.augustus.g41642.t1/1-2529	0.33024	0.287376	Y	5 exonuclease Apollo	7	C:GO:0005815; P:GO:0031848; P:GO:0036297; F:GO:0035312; [illegible]	C:microtubule organizing center; P:protection from non-homologous end [illegible]
Acrdi.XM_015897657.1/1-1284 | Monca.augustus.g50753.t1/1-1353	0.331868	0.287297	Y	PREDICTED: uncharacterized protein LOC107332906			
Acrdi.XM_015894440.1/1-774 | Monca.adi2mcaRNA4102_R0/1-783	0.309142	0.28728	Y	doublesex isoform X1	4	C:GO:0005634; F:GO:0003700; P:GO:0006355; F:GO:0043565	C:nucleus; F:transcription factor activity, sequence-specific DNA binding; [illegible]
Acrdi.XM_015900270.1/1-1278 | Monca.augustus.g23899.t1/1-1422	0.298144	0.287238	Y	C-ets-2-like isoform X1	5	C:GO:0005634; F:GO:0003677; P:GO:0030154; F:GO:0000981; [illegible]	C:nucleus; F:DNA binding; P:cell differentiation; [illegible]
Acrdi.XM_015898823.1/1-717 | Monca.augustus.g14163.t1/1-771	0.454304	0.287158	Y	caspase activity and apoptosis inhibitor 1-like isoform X3			
Acrdi.XM_015907509.1/1-1914 | Monca.augustus.g45775.t1/1-1938	0.204495	0.287141	Y	basic salivary proline-rich 2-like			
Acrdi.XM_015920721.1/1-432 | Monca.adi2mcaRNA32019_R8/1-327	0.701181	0.287051	Y	PREDICTED: uncharacterized protein LOC107354269 isoform X1			
Acrdi.XM_015894715.1/1-1911 | Monca.adi2mcaRNA41042_R1/1-1923	0.209404	0.287042	Y	phosphatidylinositol phosphatase PTPRQ-like isoform X17			
Acrdi.XM_015921136.1/1-846 | Monca.augustus.g42387.t1/1-1719	0.24014	0.286895	Y	polyadenylate-binding -interacting 1 isoform X1	1	F:GO:0003723	F:RNA binding
Acrdi.XM_015907071.1/1-447 | Monca.augustus.g43270.t1/1-531	0.425967	0.286856	Y	PREDICTED: uncharacterized protein LOC107341628			
Acrdi.XM_015902793.1/1-1869 | Monca.augustus.g54266.t1/1-1827	0.299386	0.286848	Y	peroxisomal leader peptide-processing protease	4	C:GO:0005777; P:GO:0031998; F:GO:0004252; P:GO:0016485	C:peroxisome; P:regulation of fatty acid beta-oxidation; [illegible]
Acrdi.XM_015917983.1/1-1062 | Monca.augustus.g45186.t1/1-1851	0.281917	0.28684	Y	enolase ENO4	4	F:GO:0004634; F:GO:0000287; P:GO:0006096; C:GO:0000015	F:phosphopyruvate hydratase activity; F:magnesium ion binding; [illegible]
Acrdi.XM_015896787.1/1-825 | Monca.adi2mcaRNA564_R0/1-816	0.192755	0.286778	Y	Serine threonine- kinase			
Acrdi.XM_015893447.1/1-2370 | Monca.augustus.g36549.t1/1-2358	0.256583	0.286683	Y	nucleolar complex 3 homolog	3	P:GO:0007517; C:GO:0005634; P:GO:0019216	P:muscle organ development; C:nucleus; P:regulation of lipid metabolic [illegible]
Acrdi.XM_015896199.1/1-852 | Monca.augustus.g23017.t1/1-747	0.351855	0.286642	Y	probable serine threonine- kinase dyrk1 isoform X2	2	C:GO:0016020; C:GO:0016021	C:membrane; C:integral component of membrane
Acrdi.XM_015916121.1/1-669 | Monca.augustus.g8608.t1/1-933	0.251369	0.286602	Y	rolling stone-like	2	C:GO:0016020; C:GO:0016021	C:membrane; C:integral component of membrane
Acrdi.XM_015895937.1/1-2313 | Monca.augustus.g36088.t1/1-4374	0.213432	0.286276	Y	major yolk	9	P:GO:0055072; P:GO:0006811; P:GO:0006614; F:GO:0046872; [illegible]	P:iron ion homeostasis; P:ion transport; P:SRP-dependent cotranslational [illegible]
Acrdi.XM_015911066.1/1-2076 | Monca.augustus.g46489.t1/1-2151	0.169331	0.286183	Y	von Willebrand factor A domain-containing 7-like	2	C:GO:0016020; C:GO:0016021	C:membrane; C:integral component of membrane
Acrdi.XM_015915030.1/1-2757 | Monca.augustus.g39612.t1/1-3039	0.379394	0.286134	Y	rho GTPase-activating gacF-like			
Acrdi.XM_015916145.1/1-1137 | Monca.adi2mcaRNA26930_R0/1-1050	0.354703	0.286032	Y	PREDICTED: extensin-like			
Acrdi.XM_015897870.1/1-594 | Monca.augustus.g66880.t1/1-1575	0.186482	0.285969	Y	ribosomal biogenesis LAS1L-like	6	P:GO:0000460; P:GO:0000470; P:GO:0000478; P:GO:0006325; [illegible]	P:maturation of 5.8S rRNA; P:maturation of LSU-rRNA; [illegible]
Acrdi.XM_015903265.1/1-4248 | Monca.augustus.g55383.t1/1-5337	0.34113	0.285897	Y	ankyrin-2-like isoform X1	5	F:GO:0005524; F:GO:0004702; C:GO:0005737; P:GO:0023014; [illegible]	F:ATP binding; F:signal transducer, downstream of receptor, with serine/[illegible]
Acrdi.XM_015923524.1/1-1248 | Monca.augustus.g47472.t1/1-1323	0.366085	0.28589	Y	N-acetylated-alpha-linked acidic dipeptidase 2	2	C:GO:0016020; C:GO:0016021	C:membrane; C:integral component of membrane
Acrdi.XM_015900143.1/1-2988 | Monca.augustus.g59983.t1/1-3363	0.286452	0.285781	Y	PREDICTED: uncharacterized protein LOC107335157	1	P:GO:0032502	P:developmental process
Acrdi.XM_015919613.1/1-852 | Monca.augustus.g7742.t1/1-1212	0.396566	0.285767	Y	PREDICTED: uncharacterized protein LOC107353282			
Acrdi.XM_015912646.1/1-936 | Monca.augustus.g67316.t1/1-975	0.208405	0.285666	Y	PREDICTED: uncharacterized protein LOC107346810			
Acrdi.XM_015905722.1/1-771 | Monca.augustus.g53319.t1/1-1008	0.245966	0.285501	Y	two pore potassium channel sup-9-like	9	C:GO:0005887; P:GO:0006811; P:GO:0071805; C:GO:0016020; [illegible]	C:integral component of plasma membrane; P:ion transport; [illegible]
Acrdi.XM_015910609.1/1-1524 | Monca.augustus.g45405.t1/1-1746	0.338175	0.285297	Y	adhesion G- coupled receptor D1-like			
Acrdi.XM_015899358.1/1-1170 | Monca.augustus.g44823.t1/1-1263	0.243945	0.285261	Y	peptidase inhibitor 16-like isoform X1	3	C:GO:0005576; P:GO:0035556; C:GO:0005622	C:extracellular region; P:intracellular signal transduction; C:intracellular
Acrdi.XM_015911759.1/1-852 | Monca.augustus.g30206.t1/1-1674	0.336774	0.285251	Y	Proline-rich PRCC			
Acrdi.XM_015901917.1/1-996 | Monca.augustus.g27166.t1/1-993	0.314107	0.285218	Y	PREDICTED: uncharacterized protein LOC107336847			
Acrdi.XM_015902866.1/1-918 | Monca.augustus.g27522.t1/1-936	0.291975	0.285193	Y	PREDICTED: uncharacterized protein LOC107337657	2	C:GO:0016020; C:GO:0016021	C:membrane; C:integral component of membrane
Acrdi.XM_015894958.1/1-546 | Monca.augustus.g54281.t1/1-576	0.186526	0.284916	Y	FAM207A			
Acrdi.XM_015913859.1/1-669 | Monca.augustus.g69365.t1/1-678	0.185609	0.284873	Y	ADP-ribosylation factor 6-interacting 4	2	C:GO:0005634; P:GO:0008380	C:nucleus; P:RNA splicing
Acrdi.XM_015914809.1/1-3339 | Monca.adi2mcaRNA2699_R0/1-2406	0.219744	0.284848	Y	Kanadaptin	2	P:GO:0090502; F:GO:0016891	P:RNA phosphodiester bond hydrolysis, endonucleolytic; [illegible]
Acrdi.XM_015900160.1/1-699 | Monca.augustus.g47253.t1/1-723	0.260263	0.284734	Y	PREDICTED: uncharacterized protein LOC107335171	2	F:GO:0003824; P:GO:0009987	F:catalytic activity; P:cellular process
Acrdi.XM_015902315.1/1-732 | Monca.augustus.g50955.t1/1-1146	0.13443	0.284608		PREDICTED: uncharacterized protein LOC107337196			
Acrdi.XM_015891681.1/1-1353 | Monca.augustus.g28863.t1/1-1140	0.0892595	0.284584		PREDICTED: uncharacterized protein LOC107326929			
Acrdi.XM_015897232.1/1-609 | Monca.augustus.g59967.t1/1-1209	0.21531	0.284474		calmodulin-like isoform X2	1	F:GO:0046872	F:metal ion binding
Acrdi.XM_015915290.1/1-612 | Monca.augustus.g15242.t1/1-663	0.304504	0.284356		PREDICTED: uncharacterized protein LOC107349184			
Acrdi.XM_015899345.1/1-381 | Monca.augustus.g64428.t1/1-825	0.175066	0.284347		upstream activation factor subunit spp27	2	P:GO:0090304; F:GO:0005488	P:nucleic acid metabolic process; F:binding
Acrdi.XM_015898700.1/1-3432 | Monca.augustus.g55626.t1/1-3870	0.247713	0.284264		patched domain-containing 2	3	C:GO:0016020; C:GO:0016021; P:GO:0007224	C:membrane; C:integral component of membrane; P:smoothened [illegible]
Acrdi.XM_015924056.1/1-2403 | Monca.augustus.g13005.t1/1-2385	0.326429	0.284138		armadillo repeat-containing 5			
Acrdi.XM_015905962.1/1-864 | Monca.augustus.g69516.t1/1-849	0.408	0.284118		Transmembrane and ubiquitin-like domain-containing	2	C:GO:0016020; C:GO:0016021	C:membrane; C:integral component of membrane
Acrdi.XM_015918663.1/1-780 | Monca.augustus.g7584.t1/1-783	0.382218	0.28411		PREDICTED: uncharacterized protein LOC107352327			
Acrdi.XM_015892119.1/1-2859 | Monca.adi2mcaRNA3846_R0/1-2523	0.28313	0.284106		F-box only			
Acrdi.XM_015897249.1/1-2106 | Monca.adi2mcaRNA6157_R0/1-1791	0.237841	0.284101		Acyl- dehydrogenase family member	8	F:GO:0003995; P:GO:0033539; F:GO:0000062; F:GO:0050660; [illegible]	F:acyl-CoA dehydrogenase activity; P:fatty acid beta-oxidation using [illegible]
Acrdi.XM_015907639.1/1-1242 | Monca.adi2mcaRNA17564_R0/1-1131	0.247032	0.28387		semaphorin-6C-like isoform X1			
Acrdi.XM_015898273.1/1-2706 | Monca.augustus.g64613.t1/1-2649	0.350929	0.283862		Reversion-inducing cysteine-rich with Kazal motifs	5	C:GO:0016020; C:GO:0016021; P:GO:0030198; P:GO:0010951; [illegible]	C:membrane; C:integral component of membrane; P:extracellular matrix [illegible]
Acrdi.XM_015922200.1/1-1932 | Monca.adi2mcaRNA3380_R0/1-1515	0.317484	0.283849		centrosomal of 83 kDa isoform X2			
Acrdi.XM_015918080.1/1-618 | Monca.augustus.g2209.t1/1-591	0.182561	0.283771		transcription factor -like	4	C:GO:0005634; F:GO:0003677; F:GO:0001228; P:GO:0006357	C:nucleus; F:DNA binding; F:transcriptional activator activity, RNA [illegible]
Acrdi.XM_015892500.1/1-993 | Monca.augustus.g57753.t1/1-960	0.210925	0.283752		thyrotroph embryonic factor-like isoform X2	4	P:GO:0071482; F:GO:0003700; F:GO:0045893; F:GO:0043565	P:cellular response to light stimulus; F:transcription factor activity, [illegible]

Acrdi.XM_015896924.1/1-1236 | Monca.augustus.g28869.t1/1-1239	0.260391	0.283745	hydroxylysine kinase	1	F:GO:0016740	F:transferase activity		
Acrdi.XM_015914364.1/1-3690 | Monca.adi2mcaRNA31144_R6/1-3528	0.0855423	0.283709	PREDICTED: uncharacterized protein LOC107348336					
Acrdi.XM_015907902.1/1-753 | Monca.augustus.g46274.t1/1-732	0.307685	0.283661	PREDICTED: uncharacterized protein LOC107342405					
Acrdi.XM_015903531.1/1-1038 | Monca.augustus.g44080.t1/1-945	0.25184	0.283661	ankyrin repeat domain-containing 63-like					
Acrdi.XM_015923165.1/1-867 | Monca.augustus.g14242.t1/1-888	0.260332	0.283656	Glutamate-rich 2					
Acrdi.XM_015906235.1/1-903 | Monca.augustus.g21687.t1/1-855	0.277901	0.283592	PREDICTED: uncharacterized protein LOC107340839					
Acrdi.XM_015920978.1/1-2526 | Monca.adi2mcaRNA32291_R9/1-2391	0.081138	0.283557	PREDICTED: uncharacterized protein LOC107354491					
Acrdi.XM_015913212.1/1-1308 | Monca.augustus.g32060.t1/1-1338	0.23706	0.283529	EGF-like domain-containing	3	F:GO:0005509; C:GO:0016020; C:GO:0016021	F:calcium ion binding; C:membrane; C:integral component of membrane		
Acrdi.XM_015924951.1/1-1065 | Monca.augustus.g60396.t1/1-1335	0.262692	0.283149	axoneme-associated mst101(2)-like	1	F:GO:0046872	F:metal ion binding		
Acrdi.XM_015909209.1/1-1062 | Monca.adi2mcaRNA19262_R1/1-1008	0.195808	0.283132	CD97 antigen isoform X2	9	F:GO:0004930; F:GO:0004888; C:GO:0070062; C:GO:0005925;	F:G-protein coupled receptor activity; F:transmembrane signaling receptor activity; C:extracellular exosome; C:focal adhesion; P:G-protein coupled receptor signaling pathway; F:c[illegible]		
Acrdi.XM_015916228.1/1-3852 | Monca.augustus.g35034.t1/1-4125	0.337601	0.283123	PREDICTED: uncharacterized protein LOC107350012 isoform X2					
Acrdi.XM_015924955.1/1-1179 | Monca.augustus.g4524.t1/1-1188	0.204972	0.283117	PREDICTED: uncharacterized protein LOC107358350					
Acrdi.XM_015906353.1/1-1434 | Monca.augustus.g45320.t1/1-1452	0.229517	0.283056	Dol-P-Glc:Glc(2)Man(9) c(2)-PP-Dol alpha-1,2-glucosyltransferase	6	C:GO:0005789; C:GO:0016020; C:GO:0016021; F:GO:0004583;	C:endoplasmic reticulum membrane; C:membrane; C:integral component of membrane; F:dolichyl-phosphate-glucose-glycolipid alpha-glucosyltransferase activity; F:transferase activity; P:dolichol-linked oligosaccharide biosynthetic process		
Acrdi.XM_015896541.1/1-864 | Monca.augustus.g22623.t1/1-849	0.313194	0.28296	nucleolar transcription factor 1 isoform X1	5	C:GO:0005634; F:GO:0001165; F:GO:0001164; C:GO:0005730;	C:nucleus; F:RNA polymerase I upstream control element sequence-specific DNA binding; F:RNA polymerase I CORE element sequence-specific DNA binding; C:nucleolus; F:RNA binding		
Acrdi.XM_015896887.1/1-1380 | Monca.augustus.g33176.t1/1-1881	0.341997	0.282927	Multiple PDZ domain	3	F:GO:0008270; F:GO:0046872; F:GO:0016874	F:zinc ion binding; F:metal ion binding; F:ligase activity		
Acrdi.XM_015924851.1/1-975 | Monca.augustus.g30961.t1/1-981	0.0860543	0.282901	Mitochondrial carrier	5	P:GO:0055085; F:GO:0003735; C:GO:0016021; C:GO:0005743;	P:transmembrane transport; F:structural constituent of ribosome; C:integral component of membrane; C:mitochondrial inner membrane; P:translation		
Acrdi.XM_015896736.1/1-375 | Monca.augustus.g15646.t1/1-1053	0.431926	0.282804	shugoshin-like 2 isoform X1					
Acrdi.XM_015907635.1/1-3318 | Monca.augustus.g38563.t1/1-3354	0.310553	0.282718	von Willebrand factor type EGF and pentraxin domain-containing 1-like	2	F:GO:0070492; F:GO:0005509	F:oligosaccharide binding; F:calcium ion binding		
Acrdi.XM_015898731.1/1-1191 | Monca.augustus.g2544.t1/1-1179	0.22925	0.282706	adhesion G -coupled receptor E1 isoform X6	8	P:GO:0007160; F:GO:0005509; C:GO:0016020; C:GO:0016021;	P:cell-matrix adhesion; F:calcium ion binding; C:membrane; C:integral component of membrane; F:scavenger receptor activity; F:extracellular matrix structural constituent; P:receptor-mediated endocytosis; C:proteinaceous extracellular matrix		
Acrdi.XM_015913272.1/1-999 | Monca.adi2mcaRNA23696_R0/1-1026	0.440999	0.282667	PREDICTED: uncharacterized protein LOC107347384					
Acrdi.XM_015917426.1/1-1389 | Monca.adi2mcaRNA28350_R0/1-1413	0.352765	0.282639	RING finger 151-like	4	F:GO:0004842; F:GO:0008270; F:GO:0046872; P:GO:0016567	F:ubiquitin-protein transferase activity; F:zinc ion binding; F:metal ion binding; P:protein ubiquitination		
Acrdi.XM_015899608.1/1-1761 | Monca.augustus.g54484.t1/1-1545	0.338137	0.282385	ALK tyrosine kinase receptor-like					
Acrdi.XM_015915489.1/1-1074 | Monca.augustus.g38566.t1/1-987	0.160293	0.28238	substance-K receptor-like	3	P:GO:0007165; C:GO:0016020; F:GO:0004871	P:signal transduction; C:membrane; F:signal transducer activity		
Acrdi.XM_015901055.1/1-492 | Monca.augustus.g57611.t1/1-630	0.211082	0.282364	MKI67 FHA domain-interacting nucleolar phospho -like	4	F:GO:0000166; F:GO:0003676; C:GO:0005634; P:GO:0010923	F:nucleotide binding; F:nucleic acid binding; C:nucleus; P:negative regulation of phosphatase activity		
Acrdi.XM_015911408.1/1-1209 | Monca.augustus.g70332.t1/1-1260	0.361183	0.282336	Centromere	4	F:GO:0005515; P:GO:0051276; C:GO:0000776; P:GO:0000278	F:protein binding; P:chromosome organization; C:kinetochore; P:mitotic cell cycle		
Acrdi.XM_015907377.1/1-1095 | Monca.augustus.g7587.t1/1-1116	0.180013	0.282333	phosphatidylcholine-hydrolyzing phospholipase C	2	F:GO:0034480; F:GO:0016787	F:phosphatidylcholine phospholipase C activity; F:hydrolase activity		
Acrdi.XM_015906564.1/1-396 | Monca.adi2mcaRNA16316_R0/1-408	0.279379	0.282325	cation-transporting ATPase 13A3					
Acrdi.XM_015910648.1/1-528 | Monca.augustus.g58081.t1/1-690	0.244715	0.282145	#NAME?	5	C:GO:0005634; P:GO:0021515; P:GO:0002040; P:GO:0006355;	C:nucleus; P:cell differentiation in spinal cord; P:sprouting angiogenesis; P:regulation of transcription, DNA-templated; F:sequence-specific DNA binding		
Acrdi.XM_015902113.1/1-2004 | Monca.augustus.g46409.t1/1-2013	0.219794	0.28203	inactive serine threonine- kinase TEX14 isoform X1	5	F:GO:0005515; C:GO:0044421; P:GO:0016043; P:GO:0022402;	F:protein binding; C:extracellular region part; P:cellular component organization; P:cell cycle process; C:chromosome		
Acrdi.XM_015922994.1/1-507 | Monca.augustus.g67848.t1/1-804	0.324376	0.282015	Ribonuclease P subunit p25	1	F:GO:0003676	F:nucleic acid binding		
Acrdi.XM_015924528.1/1-1098 | Monca.adi2mcaRNA3652_R0/1-1032	0.24795	0.28201	contactin-associated -like 2					
Acrdi.XM_015919044.1/1-843 | Monca.augustus.g26925.t1/1-1266	0.299871	0.281997	RNA-binding NOB1	5	P:GO:0090502; P:GO:0042274; P:GO:0000469; P:GO:0007601;	P:RNA phosphodiester bond hydrolysis, endonucleolytic; P:ribosomal small subunit biogenesis; P:cleavage involved in rRNA processing; P:visual perception; F:endoribonuclease activity		
Acrdi.XM_015924642.1/1-480 | Monca.augustus.g7891.t1/1-393	0.682288	0.281794	PREDICTED: uncharacterized protein LOC107358010					
Acrdi.XM_015923432.1/1-3729 | Monca.augustus.g37674.t1/1-1695	0.0997133	0.281687	roundabout homolog 2					
Acrdi.XM_015905028.1/1-648 | Monca.adi2mcaRNA14602_R1/1-630	0.0872457	0.281667	cell wall integrity and stress response component 4-like					
Acrdi.XM_015894198.1/1-852 | Monca.augustus.g25684.t1/1-852	0.359398	0.281533	peroxisome assembly 26	3	C:GO:0005779; P:GO:0045046; F:GO:0032403	C:integral component of peroxisomal membrane; P:protein import into peroxisome membrane; F:protein complex binding		
Acrdi.XM_015893040.1/1-1041 | Monca.augustus.g25140.t1/1-1038	0.210788	0.281383	PREDICTED: uncharacterized protein LOC107328299	1	F:GO:0003676	F:nucleic acid binding		
Acrdi.XM_015893236.1/1-1416 | Monca.augustus.g33531.t1/1-2076	0.18636	0.281348	HAUS augmin-like complex subunit 5	2	P:GO:0051225; C:GO:0070652	P:spindle assembly; C:HAUS complex		
Acrdi.XM_015908931.1/1-498 | Monca.augustus.g31854.t1/1-576	0.222507	0.281336	PREDICTED: uncharacterized protein LOC107343366					
Acrdi.XM_015902801.1/1-522 | Monca.augustus.g27228.t1/1-675	0.228993	0.281175	PREDICTED: uncharacterized protein LOC107337597					
Acrdi.XM_015916823.1/1-714 | Monca.adi2mcaRNA27668_R0/1-711	0.372528	0.281021	PREDICTED: uncharacterized protein LOC107350580					
Acrdi.XM_015903039.1/1-2319 | Monca.adi2mcaRNA12420_R0/1-2193	0.129566	0.280962	dopamine receptor 2-like	3	F:GO:0004930; P:GO:0007165; C:GO:0016020	F:G-protein coupled receptor activity; P:signal transduction; C:membrane		
Acrdi.XM_015917368.1/1-1746 | Monca.augustus.g23813.t1/1-1677	0.270345	0.28095	ubiquitin carboxyl-terminal hydrolase 2-like	2	P:GO:0006508; F:GO:0008234	P:proteolysis; F:cysteine-type peptidase activity		
Acrdi.XM_015892103.1/1-534 | Monca.augustus.g12053.t1/1-576	0.23858	0.28078	peptidase S8	3	F:GO:0008061; P:GO:0006030; C:GO:0005576	F:chitin binding; P:chitin metabolic process; C:extracellular region		
Acrdi.XM_015921840.1/1-624 | Monca.augustus.g16481.t1/1-567	0.279576	0.280694	PREDICTED: uncharacterized protein LOC107355299					
Acrdi.XM_015918024.1/1-1236 | Monca.adi2mcaRNA29054_R0/1-1554	0.0844354	0.280694	branchpoint-bridging -like					
Acrdi.XM_015905187.1/1-3519 | Monca.augustus.g57161.t1/1-3828	0.243972	0.280665	lactose-binding lectin l-2-like					
Acrdi.XM_015898654.1/1-753 | Monca.augustus.g34598.t1/1-831	0.280166	0.280458	Na(+) H(+) exchange regulatory cofactor NHE-RF1	2	F:GO:0008022; C:GO:0044464	F:protein C-terminus binding; C:cell part		
Acrdi.XM_015899607.1/1-2568 | Monca.augustus.g54485.t1/1-2823	0.29946	0.280385	VPS9 domain-containing 1					
Acrdi.XM_015924640.1/1-939 | Monca.augustus.g20221.t1/1-927	0.319135	0.280301	PREDICTED: uncharacterized protein LOC107358007					
Acrdi.XM_015924344.1/1-750 | Monca.augustus.g32393.t1/1-1005	0.300605	0.280267	apextrin 1	3	F:GO:0005509; C:GO:0016020; C:GO:0016021	F:calcium ion binding; C:membrane; C:integral component of membrane		
Acrdi.XM_015894144.1/1-771 | Monca.augustus.g23885.t1/1-783	0.322457	0.2801	PREDICTED: uncharacterized protein LOC107329459					
Acrdi.XM_015921710.1/1-990 | Monca.augustus.g67308.t1/1-729	0.207238	0.279985	survival of motor neuron-related-splicing factor 30-like	1	C:GO:0044424	C:intracellular part		
Acrdi.XM_015911645.1/1-1134 | Monca.augustus.g66868.t1/1-1575	0.225366	0.279814	poly(A) RNA polymerase GLD2-like					
Acrdi.XM_015898294.1/1-834 | Monca.augustus.g61835.t1/1-810	0.268878	0.279699	Forkhead box L1	4	C:GO:0005634; F:GO:0043565; F:GO:0000981; P:GO:0006357	C:nucleus; F:sequence-specific DNA binding; F:RNA polymerase II transcription factor activity, sequence-specific DNA binding; P:regulation of transcription from RNA polymerase II promoter		
Acrdi.XM_015914382.1/1-990 | Monca.augustus.g67295.t1/1-915	0.19467	0.279531	Mitochondrial uncoupling 4	7	C:GO:0031966; F:GO:0003735; P:GO:0006839; C:GO:0016020;	C:mitochondrial membrane; F:structural constituent of ribosome; P:mitochondrial transport; C:membrane; C:integral component of membrane; P:transport; P:translation		
Acrdi.XM_015917729.1/1-1134 | Monca.augustus.g1561.t1/1-1059	0.170348	0.279485	allatostatin-A receptor-like	8	F:GO:0004930; C:GO:0005887; P:GO:0007186; P:GO:0007165;	F:G-protein coupled receptor activity; C:integral component of plasma membrane; P:G-protein coupled receptor signaling pathway; P:signal transduction; C:membrane; C:integral component of membrane; P:cell surface receptor signaling pathway; F:signal transducer activity		
Acrdi.XM_015915025.1/1-4788 | Monca.augustus.g39609.t1/1-4578	0.243653	0.279482	phosphatase Slingshot isoform X2	4	F:GO:0003677; F:GO:0004725; P:GO:0035335; F:GO:0008138	F:DNA binding; F:protein tyrosine phosphatase activity; P:peptidyl-tyrosine dephosphorylation; F:protein tyrosine/serine/threonine phosphatase activity	EC:3.1.3.16; EC:3.1.3.48; EC:3.1.3.41	Protein-serine/threonine phosphatase; Protein-tyrosine-phosphatase; 4-nitrophenylphosphatase
Acrdi.XM_015910512.1/1-2160 | Monca.augustus.g25435.t1/1-2541	0.199225	0.279369	dystroglycan-like isoform X4	5	P:GO:0050794; P:GO:0045995; P:GO:0022603; C:GO:0044425;	P:regulation of cellular process; P:regulation of embryonic development; P:regulation of anatomical structure morphogenesis; C:membrane part; C:plasma membrane		
Acrdi.XM_015903088.1/1-1143 | Monca.augustus.g18850.t1/1-1146	0.193475	0.279358	probable G- coupled receptor 135	8	F:GO:0004930; C:GO:0005887; P:GO:0007186; P:GO:0007165;	F:G-protein coupled receptor activity; C:integral component of plasma membrane; P:G-protein coupled receptor signaling pathway; P:signal transduction; C:membrane; C:integral component of membrane; P:cell surface receptor signaling pathway; F:signal transducer activity		
Acrdi.XM_015925261.1/1-1194 | Monca.adi2mcaRNA3710_R3/1-1632	0.0830799	0.279285	RNA-directed DNA polymerase from transposon BS					
Acrdi.XM_015903130.1/1-447 | Monca.augustus.g44582.t1/1-1869	0.0836148	0.279271	PREDICTED: uncharacterized protein LOC107337893					
Acrdi.XM_015897475.1/1-1266 | Monca.augustus.g2234.t1/1-1212	0.311218	0.27921	Fanconi anemia group F	1	C:GO:0043240	C:Fanconi anaemia nuclear complex		
Acrdi.XM_015897459.1/1-351 | Monca.augustus.g22824.t1/1-2925	0.360103	0.279153	PREDICTED: uncharacterized protein LOC107332713					
Acrdi.XM_015923842.1/1-1311 | Monca.augustus.g45344.t1/1-2136	0.233465	0.279065	PREDICTED: uncharacterized protein LOC107357198 isoform X2	2	C:GO:0016020; C:GO:0016021	C:membrane; C:integral component of membrane		
Acrdi.XM_015918504.1/1-1653 | Monca.augustus.g23501.t1/1-1680	0.245236	0.279036	PREDICTED: uncharacterized protein LOC107352158					
Acrdi.XM_015911970.1/1-2538 | Monca.augustus.g58625.t1/1-2424	0.271322	0.27901	potassium voltage-gated channel subfamily D member 1	7	P:GO:0006813; P:GO:0055085; F:GO:0005251; C:GO:0016021;	P:potassium ion transport; P:transmembrane transport; F:delayed rectifier potassium channel activity; C:integral component of membrane; C:plasma membrane; P:cellular process; P:cardiac conduction		
Acrdi.XM_015912023.1/1-435 | Monca.augustus.g67645.t1/1-477	0.181994	0.278984	AP-4 complex subunit sigma	1	P:GO:0006810	P:transport		
Acrdi.XM_015906605.1/1-444 | Monca.augustus.g63795.t1/1-426	0.284507	0.278944	RING finger 150-like	1	F:GO:0046872	F:metal ion binding		
Acrdi.XM_015915932.1/1-912 | Monca.augustus.g24376.t1/1-1383	0.208702	0.278723	coadhesin-like isoform X3					
Acrdi.XM_015897736.1/1-1011 | Monca.augustus.g50749.t1/1-990	0.261781	0.27872	deoxynucleotidyltransferase terminal-interacting 2-like					
Acrdi.XM_015922811.1/1-1170 | Monca.adi2mcaRNA34345_R0/1-1032	0.411101	0.278557	Spindle and centriole-associated 1	3	C:GO:0015630; C:GO:0044430; P:GO:0022402	C:microtubule cytoskeleton; C:cytoskeletal part; P:cell cycle process		
Acrdi.XM_015904143.1/1-804 | Monca.augustus.g36707.t1/1-828	0.446401	0.278465	cyclic pyranopterin monophosphate synthase accessory mitochondrial	6	F:GO:0003824; C:GO:0043231; C:GO:0005829; F:GO:0051539;	F:catalytic activity; C:intracellular membrane-bounded organelle; C:cytosol; F:4 iron, 4 sulfur cluster binding; P:Mo-molybdopterin cofactor biosynthetic process; P:molybdopterin cofactor biosynthetic process		
Acrdi.XM_015919679.1/1-1200 | Monca.augustus.g43846.t1/1-1479	0.299596	0.278105	PREDICTED: uncharacterized protein LOC107353331					
Acrdi.XM_015899765.1/1-1014 | Monca.adi2mcaRNA8882_R0/1-1044	0.812248	0.278035	volume-regulated anion channel subunit LRRC8A-like	2	C:GO:0016020; C:GO:0016021	C:membrane; C:integral component of membrane		
Acrdi.XM_015919040.1/1-501 | Monca.augustus.g26965.t1/1-597	0.282261	0.277968	coagulation factor VII-like					
Acrdi.XM_015924498.1/1-1128 | Monca.augustus.g13467.t1/1-1230	0.269461	0.27792	PREDICTED: uncharacterized protein LOC107357860	2	C:GO:0016020; C:GO:0016021	C:membrane; C:integral component of membrane		
Acrdi.XM_015909707.1/1-834 | Monca.augustus.g55264.t1/1-1083	0.271918	0.277836	PREDICTED: uncharacterized protein LOC107344099 isoform X1	2	C:GO:0016020; C:GO:0016021	C:membrane; C:integral component of membrane		
Acrdi.XM_015908259.1/1-786 | Monca.augustus.g20162.t1/1-825	0.464689	0.277765	tumor necrosis factor receptor superfamily member 16-like	2	C:GO:0016020; C:GO:0016021	C:membrane; C:integral component of membrane		
Acrdi.XM_015916587.1/1-840 | Monca.augustus.g65199.t1/1-1044	0.148996	0.27762	RNA-directed DNA polymerase from mobile element jockey					
Acrdi.XM_015916803.1/1-1617 | Monca.augustus.g46438.t1/1-1620	0.270037	0.277557	Ubiquitin-associated 1					
Acrdi.XM_015921733.1/1-2163 | Monca.adi2mcaRNA33121_R0/1-1998	0.283106	0.277415	von Willebrand factor type EGF and pentraxin domain-containing 1-like isoform X1					
Acrdi.XM_015905587.1/1-1059 | Monca.augustus.g24115.t1/1-2085	0.497087	0.277376	PREDICTED: uncharacterized protein LOC107340235	10	F:GO:0004930; C:GO:0005887; P:GO:0007186; P:GO:0007187;	F:G-protein coupled receptor activity; C:integral component of plasma membrane; P:G-protein coupled receptor signaling pathway; P:G-protein coupled receptor signaling pathway, coupled to cyclic nucleotide second messenger; P:signal transduction; C:membrane; C:integral component of membrane; F:peptide binding; F:signal transducer activity; C:neuron projection		
Acrdi.XM_015895615.1/1-1608 | Monca.augustus.g64084.t1/1-1524	0.181951	0.277359	Fibrous sheath-interacting 1					
Acrdi.XM_015901936.1/1-1731 | Monca.augustus.g4671.t1/1-1875	0.360728	0.277229	PREDICTED: uncharacterized protein LOC107336861					
Acrdi.XM_015913777.1/1-855 | Monca.augustus.g26414.t1/1-1056	0.29991	0.277223	gremlin-2	6	P:GO:0071310; P:GO:0007275; P:GO:0007166; C:GO:0005576;	P:cellular response to organic substance; P:multicellular organism development; P:cell surface receptor signaling pathway; C:extracellular region; F:binding; P:regulation of transcription from RNA polymerase II promoter		
Acrdi.XM_015905263.1/1-969 | Monca.augustus.g23149.t1/1-933	0.307914	0.277185	orexin receptor type 2-like	3	F:GO:0004930; P:GO:0007186; C:GO:0016021	F:G-protein coupled receptor activity; P:G-protein coupled receptor signaling pathway; C:integral component of membrane		
Acrdi.XM_015912923.1/1-2787 | Monca.adi2mcaRNA23328_R0/1-2709	0.316941	0.277153	F-box LRR-repeat 4	1	C:GO:0005634	C:nucleus		
Acrdi.XM_015920125.1/1-1497 | Monca.augustus.g28571.t1/1-1506	0.248961	0.276897	transcription initiation factor IIB	5	P:GO:0090304; P:GO:0044271; P:GO:0010467; F:GO:0005488;	P:nucleic acid metabolic process; P:cellular nitrogen compound biosynthetic process; P:gene expression; F:binding; P:cellular macromolecule biosynthetic process		
Acrdi.XM_015907868.1/1-1083 | Monca.augustus.g47392.t1/1-1104	0.296656	0.276895	neuropeptide FF receptor 1	1	P:GO:0007186	P:G-protein coupled receptor signaling pathway		
Acrdi.XM_015901134.1/1-1563 | Monca.augustus.g15294.t1/1-1632	0.284848	0.27689	beta-1,4-N-acetylgalactosaminyltransferase 3-like	5	C:GO:0032580; C:GO:0016020; C:GO:0016021; F:GO:0008376;	C:Golgi cisterna membrane; C:membrane; C:integral component of membrane; F:acetylgalactosaminyltransferase activity; F:transferase activity		
Acrdi.XM_015895831.1/1-1644 | Monca.adi2mcaRNA423_R0/1-1653	0.298276	0.276806	PREDICTED: uncharacterized protein LOC107331259					
Acrdi.XM_015908391.1/1-984 | Monca.augustus.g34798.t1/1-951	0.296106	0.276592	macrophage scavenger receptor types I and II-like isoform X2	2	C:GO:0016020; C:GO:0016021	C:membrane; C:integral component of membrane		
Acrdi.XM_015903878.1/1-1164 | Monca.augustus.g48097.t1/1-1197	0.308461	0.276468	PREDICTED: uncharacterized protein LOC107338644					
Acrdi.XM_015907814.1/1-1176 | Monca.adi2mcaRNA17747_R6/1-1194	0.296063	0.27638	Retrovirus-related Pol poly from transposon					
Acrdi.XM_015919405.1/1-1080 | Monca.augustus.g22488.t1/1-1053	0.159907	0.276295	PREDICTED: uncharacterized protein LOC107353086	2	P:GO:0035556; C:GO:0005622	P:intracellular signal transduction; C:intracellular		
Acrdi.XM_015924348.1/1-2787 | Monca.augustus.g32409.t1/1-2775	0.319184	0.276141	KAT8 regulatory NSL complex subunit 2 isoform X1	5	P:GO:0043967; F:GO:0046972; F:GO:0043996; C:GO:0044545;	P:histone H4 acetylation; F:histone acetyltransferase activity (H4-K16 specific); F:histone acetyltransferase activity (H4-K8 specific); C:NSL complex; F:histone acetyltransferas	EC:2.3.1.48	Histone acetyltransferase
Acrdi.XM_015922254.1/1-5529 | Monca.augustus.g4389.t1/1-4986	0.259367	0.276078	E3 ubiquitin- ligase RNF31	10	P:GO:0003677; F:GO:0004842; F:GO:0003700; F:GO:0008270;	F:DNA binding; F:ubiquitin-protein transferase activity; F:transcription factor activity, sequence-specific DNA binding; F:zinc ion binding; F:metal ion binding; P:regulation of transcription, DNA-templated; F:sequence-specific DNA binding; C:LUBAC complex; C:intracellular; P:protein ubiquitination		
Acrdi.XM_015902897.1/1-651 | Monca.augustus.g27531.t1/1-1395	0.248354	0.276046	XK-related 6-like isoform X1					
Acrdi.XM_015910624.1/1-3279 | Monca.augustus.g37496.t1/1-4671	0.269532	0.275774	tyrosine- kinase BAZ1B					
Acrdi.XM_015913724.1/1-1056 | Monca.augustus.g26406.t1/1-1038	0.221218	0.275703	neuromedin-U receptor 2-like	3	P:GO:0007186; C:GO:0016020; F:GO:0004871	P:G-protein coupled receptor signaling pathway; C:membrane; F:signal transducer activity		
Acrdi.XM_015902369.1/1-2790 | Monca.augustus.g37591.t1/1-3138	0.22014	0.27565	Golgi-associated plant pathogenesis-related 1-like isoform X3					
Acrdi.XM_015903600.1/1-1326 | Monca.augustus.g53510.t1/1-1899	0.224389	0.275649	PREDICTED: uncharacterized protein LOC107338368	2	C:GO:0016020; C:GO:0016021	C:membrane; C:integral component of membrane		
Acrdi.XM_015925232.1/1-876 | Monca.augustus.g53767.t1/1-999	0.406448	0.275644	EGF-like repeat and discoidin I-like domain-containing 3					
Acrdi.XM_015908380.1/1-1770 | Monca.augustus.g34816.t1/1-3024	0.394385	0.275594	PREDICTED: uncharacterized protein LOC107342864					
Acrdi.XM_015905804.1/1-2901 | Monca.adi2mcaRNA15470_R0/1-2601	0.0958012	0.275588	Retrovirus-related Pol poly from transposon TNT 1-					
Acrdi.XM_015911118.1/1-867 | Monca.augustus.g9904.t1/1-1011	0.361513	0.275547	Sodium channel modifier 1	5	C:GO:0005634; P:GO:0008380; F:GO:0046872; C:GO:0016607;	C:nucleus; P:RNA splicing; F:metal ion binding; C:nuclear speck; P:mRNA processing		
Acrdi.XM_015895081.1/1-468 | Monca.augustus.g25187.t1/1-1023	0.336612	0.275531	zinc transporter 2-like isoform X2	5	P:GO:0010043; C:GO:0016020; P:GO:0061088; P:GO:0098655;	P:response to zinc ion; C:membrane; P:regulation of sequestering of zinc ion; P:cation transmembrane transport; F:zinc ion transmembrane transporter activity		
Acrdi.XM_015921620.1/1-2199 | Monca.augustus.g26612.t1/1-2304	0.276966	0.275486	Chromosome 6 open reading frame 150	3	F:GO:0097159; F:GO:1901363; P:GO:0002376	F:organic cyclic compound binding; F:heterocyclic compound binding; P:immune system process		
Acrdi.XM_015920438.1/1-582 | Monca.augustus.g47239.t1/1-594	0.407383	0.275457	PREDICTED: uncharacterized protein LOC107354036					
Acrdi.XM_015903773.1/1-1743 | Monca.adi2mcaRNA13228_R0/1-1617	0.259761	0.275396	stimulated by retinoic acid gene 6 homolog	1	C:GO:0016020	C:membrane		
Acrdi.XM_015895575.1/1-3099 | Monca.augustus.g22276.t1/1-3000	0.26001	0.275371	PREDICTED: trichohyalin-like					
Acrdi.XM_015899386.1/1-555 | Monca.augustus.g46725.t1/1-843	0.29939	0.275325	3-oxo-5-alpha-steroid 4-dehydrogenase 1-like	6	C:GO:0016627; C:GO:0005737; C:GO:0016020; P:GO:0006629;	F:oxidoreductase activity, acting on the CH-CH group of donors; C:cytoplasm; C:membrane; P:lipid metabolic process; C:integral component of membrane; P:oxidation-reduction process		
Acrdi.XM_015899372.1/1-1050 | Monca.augustus.g44792.t1/1-1686	0.284518	0.275274	interferon regulatory factor 8-like	2	F:GO:0003700; P:GO:0006355	F:transcription factor activity, sequence-specific DNA binding; P:regulation of transcription, DNA-templated		
Acrdi.XM_015912011.1/1-651 | Monca.adi2mcaRNA22293_R0/1-708	1.12601	0.275166	Integrase recombinase xerD-like					
Acrdi.XM_015904529.1/1-567 | Monca.augustus.g6298.t1/1-570	0.274201	0.275091	Cartilage matrix	2	C:GO:0016020; C:GO:0016021	C:membrane; C:integral component of membrane		
Acrdi.XM_015900553.1/1-825 | Monca.augustus.g26469.t1/1-882	0.388768	0.275015	threonine ammonia-lyase	2	F:GO:0016829; P:GO:0006520	F:lyase activity; P:cellular amino acid metabolic process		
Acrdi.XM_015911914.1/1-663 | Monca.augustus.g30057.t1/1-723	0.170992	0.2746	methylthioribulose-1-phosphate dehydratase	1	P:GO:0009086	P:methionine biosynthetic process		
Acrdi.XM_015903526.1/1-2226 | Monca.augustus.g34543.t1/1-2271	0.270397	0.274543	DDB1- and CUL4-associated factor 15-like					
Acrdi.XM_015891630.1/1-528 | Monca.augustus.g4262.t1/1-525	0.182484	0.274528	CBFA2T3 isoform X6	5	F:GO:0003700; P:GO:0045892; F:GO:0003714; F:GO:0046872;	F:transcription factor activity, sequence-specific DNA binding; P:negative regulation of transcription, DNA-templated; F:transcription corepressor activity; F:metal ion binding; P:regulation of transcription, DNA-templated		
Acrdi.XM_015900613.1/1-1461 | Monca.augustus.g14896.t1/1-1578	0.203038	0.274469	collagen alpha-1(XXVIII) chain-like	1	P:GO:0034219	P:carbohydrate transmembrane transport		
Acrdi.XM_015915288.1/1-1764 | Monca.augustus.g21303.t1/1-1767	0.312278	0.274343	C-C chemokine receptor 1 1	3	F:GO:0004930; P:GO:0007165; C:GO:0016020	F:G-protein coupled receptor activity; P:signal transduction; C:membrane		
Acrdi.XM_015906598.1/1-1401 | Monca.adi2mcaRNA33252_R8/1-1677	0.0803029	0.274238	PREDICTED: uncharacterized protein LOC107341186					
Acrdi.XM_015920061.1/1-1107 | Monca.adi2mcaRNA31308_R0/1-1113	0.241063	0.274133	ame: Full=Coadhesin					
Acrdi.XM_015904853.1/1-468 | Monca.augustus.g49263.t1/1-582	0.351648	0.273926	PREDICTED: uncharacterized protein LOC107339543					
Acrdi.XM_015896616.1/1-1461 | Monca.adi2mcaRNA544_R0/1-1386	0.28489	0.273916	galactoside 3(4)-L-fucosyltransferase-like	2	C:GO:0016020; F:GO:0016740	C:membrane; F:transferase activity		
Acrdi.XM_015892020.1/1-1989 | Monca.augustus.g37394.t1/1-2040	0.225868	0.273875	PREDICTED: triadin-like					
Acrdi.XM_015905056.1/1-588 | Monca.augustus.g15476.t1/1-588	0.302496	0.273829	ubiA prenyltransferase domain-containing 1 homolog	4	F:GO:0004659; C:GO:0016020; C:GO:0016021; F:GO:0016740	F:prenyltransferase activity; C:membrane; C:integral component of membrane; F:transferase activity		
Acrdi.XM_015892340.1/1-1437 | Monca.adi2mcaRNA38266_R0/1-1359	0.388192	0.273826	zinc metallo ase nas-13-like	3	F:GO:0008270; P:GO:0006508; F:GO:0004222	F:zinc ion binding; P:proteolysis; F:metalloendopeptidase activity	EC:3.4.24	Acting on peptide bonds (peptidases)
Acrdi.XM_015916142.1/1-918 | Monca.adi2mcaRNA26916_R0/1-711	0.374433	0.273807	Coagulation factor V					
Acrdi.XM_015904670.1/1-900 | Monca.augustus.g12715.t1/1-1710	0.320601	0.273804	PREDICTED: uncharacterized protein LOC107339391					
Acrdi.XM_015922714.1/1-411 | Monca.augustus.g46485.t1/1-378	0.175955	0.273788	PREDICTED: uncharacterized protein LOC107356071 isoform X1					
Acrdi.XM_015899455.1/1-699 | Monca.augustus.g48272.t1/1-660	0.356972	0.273778	PREDICTED: uncharacterized protein LOC107334519					
Acrdi.XM_015906992.1/1-1224 | Monca.augustus.g33369.t1/1-1278	0.334395	0.273702	SH3 domain-binding 2-like					
Acrdi.XM_015912800.1/1-558 | Monca.augustus.g3598.t1/1-1110	0.248318	0.273685	Forkhead box K1	6	F:GO:0004930; P:GO:0007186; P:GO:0007165; C:GO:0016020;	F:G-protein coupled receptor activity; P:G-protein coupled receptor signaling pathway; P:signal transduction; C:membrane; C:integral component of membrane; F:signal transducer activity		
Acrdi.XM_015913827.1/1-657 | Monca.adi2mcaRNA24335_R1/1-621	0.249115	0.273631	MAM and LDL-receptor class A domain-containing 2-like isoform X6	1	C:GO:0016020	C:membrane		
Acrdi.XM_015897879.1/1-861 | Monca.augustus.g66883.t1/1-840	0.328423	0.273569	PREDICTED: uncharacterized protein LOC107333118					
Acrdi.XM_015919689.1/1-2856 | Monca.adi2mcaRNA30897_R0/1-2685	0.32603	0.273397	myb-binding 1A	4	F:GO:0003677; F:GO:0003887; P:GO:0006351; P:GO:0071897	F:DNA binding; F:DNA-directed DNA polymerase activity; P:transcription, DNA-templated; P:DNA biosynthetic process		
Acrdi.XM_015904336.1/1-966 | Monca.augustus.g61772.t1/1-939	0.265323	0.273345	PREDICTED: uncharacterized protein LOC107339097 isoform X1	2	C:GO:0016020; C:GO:0016021	C:membrane; C:integral component of membrane		
Acrdi.XM_015908381.1/1-903 | Monca.augustus.g34814.t1/1-1110	0.270846	0.273319	PREDICTED: uncharacterized protein LOC107342865					
Acrdi.XM_015924734.1/1-1767 | Monca.augustus.g4189.t1/1-2457	0.254353	0.273314	mdm2-binding isoform X1	6	P:GO:0045839; P:GO:0008285; P:GO:0034501; P:GO:0007089;	P:negative regulation of mitotic nuclear division; P:negative regulation of cell proliferation; P:protein localization to kinetochore; P:traversing start control point of mitotic cell cycle; C:kinetochore; C:chromatin		
Acrdi.XM_015904745.1/1-405 | Monca.augustus.g4279.t1/1-396	0.400291	0.273264	PREDICTED: uncharacterized protein LOC107339458					
Acrdi.XM_015899465.1/1-534 | Monca.augustus.g48260.t1/1-501	0.517662	0.273017	Ribonuclease P MRP subunit POP5	3	P:GO:0090501; F:GO:0004540; P:GO:0009249	P:RNA phosphodiester bond hydrolysis; F:ribonuclease activity; P:protein lipoylation		
Acrdi.XM_015910392.1/1-315 | Monca.augustus.g35315.t1/1-312	0.28792	0.272997	brain I3	2	C:GO:0016020; C:GO:0016021	C:membrane; C:integral component of membrane		
Acrdi.XM_015910507.1/1-3849 | Monca.augustus.g25428.t1/1-6216	0.333317	0.272888	Dystroglycan	6	P:GO:0048731; C:GO:0016020; C:GO:0044464; F:GO:0005488;	P:system development; C:membrane; C:cell part; F:binding; P:regulation of biological process; P:cellular process		
Acrdi.XM_015903049.1/1-2910 | Monca.augustus.g45188.t1/1-2847	0.277379	0.272645	fibroblast growth factor receptor 1 isoform X4	10	P:GO:0009790; P:GO:0060255; P:GO:0048522; P:GO:0007420;	P:embryo development; P:regulation of macromolecule metabolic process; P:positive regulation of cellular process; P:brain development; P:generation of neurons; F:binding; F:kinase activity; P:tissue development; P:animal organ morphogenesis; P:regulation of multicellular organismal development		
Acrdi.XM_015914921.1/1-1146 | Monca.augustus.g41788.t1/1-1137	0.239262	0.272634	PREDICTED: uncharacterized protein LOC107348816					
Acrdi.XM_015897898.1/1-1560 | Monca.augustus.g23836.t1/1-1695	0.223477	0.272634	glypican-4					
Acrdi.XM_015919832.1/1-447 | Monca.augustus.g69874.t1/1-1296	0.336589	0.272592	PREDICTED: uncharacterized protein LOC107353503					
Acrdi.XM_015902847.1/1-1902 | Monca.augustus.g27232.t1/1-4671	0.289029	0.272375	proline-rich 36-like					
Acrdi.XM_015915029.1/1-1062 | Monca.augustus.g41786.t1/1-1083	0.280363	0.272369	PREDICTED: uncharacterized protein LOC107348903					
Acrdi.XM_015911113.1/1-438 | Monca.augustus.g58863.t1/1-447	0.323107	0.272351	Interferon-induced transmembrane	3	P:GO:0009607; C:GO:0016020; C:GO:0016021	P:response to biotic stimulus; C:membrane; C:integral component of membrane		
Acrdi.XM_015907314.1/1-582 | Monca.augustus.g68538.t1/1-570	0.346489	0.272346	homeobox orthopedia-like					
Acrdi.XM_015923356.1/1-588 | Monca.augustus.g51720.t1/1-585	0.31983	0.272236	PREDICTED: uncharacterized protein LOC107356733					
Acrdi.XM_015898906.1/1-1239 | Monca.augustus.g37979.t1/1-1782	0.300163	0.272147	Kynurenine 3-monooxygenase and related flavo monooxygenases (ISS)	3	F:GO:0071949; F:GO:0016491; P:GO:0055114	F:FAD binding; F:oxidoreductase activity; P:oxidation-reduction process		
Acrdi.XM_015898676.1/1-843 | Monca.adi2mcaRNA7704_R0/1-783	0.372584	0.272121	PREDICTED: uncharacterized protein LOC107333805					
Acrdi.XM_015896768.1/1-1056 | Monca.adi2mcaRNA5639_R1/1-1236	0.263309	0.272048	N-acetylglucosamine-1-phosphotransferase subunit gamma precursor	1	F:GO:0016740	F:transferase activity		
Acrdi.XM_015905621.1/1-639 | Monca.augustus.g19786.t1/1-642	0.395817	0.272034	PREDICTED: uncharacterized protein LOC107340276					
Acrdi.XM_015902081.1/1-2112 | Monca.adi2mcaRNA11434_R0/1-1851	0.219734	0.271988	PAX-interacting 1	9	P:GO:0090304; C:GO:0005634; P:GO:0019219; P:GO:0044260;	P:nucleic acid metabolic process; C:nucleus; P:regulation of nucleobase-containing compound metabolic process; P:cellular macromolecule metabolic process; P:single-multicellular organism process; P:anatomical structure development; P:positive regulation of histone modification; P:cellular response to DNA damage stimulus; P:single-organism developmental process		
Acrdi.XM_015912254.1/1-705 | Monca.augustus.g28979.t1/1-636	0.31727	0.271982	PREDICTED: uncharacterized protein LOC107346463	2	C:GO:0016020; C:GO:0016021	C:membrane; C:integral component of membrane		
Acrdi.XM_015910243.1/1-510 | Monca.adi2mcaRNA20384_R0/1-525	0.25325	0.271946	tissue factor pathway inhibitor	2	F:GO:0004867; P:GO:0010951	F:serine-type endopeptidase inhibitor activity; P:negative regulation of endopeptidase activity		
Acrdi.XM_015909417.1/1-1131 | Monca.adi2mcaRNA2026_R0/1-1029	0.3008	0.271935	Neurogenic locus Notch					
Acrdi.XM_015904326.1/1-807 | Monca.augustus.g8030.t1/1-810	0.321107	0.271885	methyltransferase mitochondrial	3	F:GO:0008168; F:GO:0016740; P:GO:0032259	F:methyltransferase activity; F:transferase activity; P:methylation		
Acrdi.XM_015918714.1/1-954 | Monca.adi2mcaRNA29786_R1/1-966	0.0780334	0.271771	craniofacial development 2-like					
Acrdi.XM_015919821.1/1-2067 | Monca.adi2mcaRNA31047_R0/1-1707	0.266235	0.271654	PREDICTED: uncharacterized protein LOC107353491					
Acrdi.XM_015902654.1/1-1041 | Monca.augustus.g54288.t1/1-1074	0.171629	0.271486	Actin-related 3					
Acrdi.XM_015923991.1/1-2958 | Monca.augustus.g54034.t1/1-3846	0.275971	0.271483	E3 ubiquitin- ligase TTC3-like isoform X1	6	C:GO:0005634; F:GO:0004842; C:GO:0016020; C:GO:0016021;	C:nucleus; F:ubiquitin-protein transferase activity; C:membrane; C:integral component of membrane; P:ubiquitin-dependent protein catabolic process; P:protein ubiquitination		
Acrdi.XM_015897066.1/1-2265 | Monca.augustus.g51945.t1/1-2121	0.314386	0.271357	Neogenin	8	P:GO:0055072; P:GO:0030513; C:GO:0005887; C:GO:0016020;	P:iron ion homeostasis; P:positive regulation of BMP signaling pathway; C:integral component of plasma membrane; C:membrane; C:integral component of membrane; P:nervous system development; P:axon guidance; F:receptor activity		
Acrdi.XM_015899698.1/1-768 | Monca.augustus.g43436.t1/1-1023	0.373744	0.271342	CUB and sushi domain-containing 3-	3	F:GO:0004930; P:GO:0007186; C:GO:0016020	F:G-protein coupled receptor activity; P:G-protein coupled receptor signaling pathway; C:membrane		
Acrdi.XM_015894253.1/1-900 | Monca.augustus.g57601.t1/1-864	0.362592	0.271222	transmembrane FAM155A-like isoform X1					
Acrdi.XM_015901419.1/1-657 | Monca.augustus.g31701.t1/1-3804	0.148109	0.271205	collagen alpha-1(XII) chain-like	3	F:GO:0030414; C:GO:0005576; P:GO:0010466	F:peptidase inhibitor activity; C:extracellular region; P:negative regulation of peptidase activity		
Acrdi.XM_015900044.1/1-2487 | Monca.augustus.g6665.t1/1-3735	0.185909	0.271159	chorion peroxidase-like	8	F:GO:0004601; P:GO:0006979; F:GO:0010181; F:GO:0003824;	F:peroxidase activity; P:response to oxidative stress; F:FMN binding; F:catalytic activity; F:oxidoreductase activity; P:cellular oxidant detoxification; P:oxidation-reduction process; F:heme binding		
Acrdi.XM_015904300.1/1-549 | Monca.augustus.g8031.t1/1-546	0.247512	0.271114	von Willebrand factor D and EGF domain-containing	3	F:GO:0004867; F:GO:0005509; P:GO:0010951	F:serine-type endopeptidase inhibitor activity; F:calcium ion binding; P:negative regulation of endopeptidase activity		
Acrdi.XM_015911570.1/1-2442 | Monca.augustus.g44322.t1/1-2643	0.158256	0.271109	PREDICTED: uncharacterized protein LOC107345820					
Acrdi.XM_015897305.1/1-663 | Monca.augustus.g67497.t1/1-822	0.221878	0.27109	barH-like 1 homeobox	3	C:GO:0005634; P:GO:0006355; F:GO:0043565	C:nucleus; P:regulation of transcription, DNA-templated; F:sequence-specific DNA binding		
Acrdi.XM_015911321.1/1-879 | Monca.augustus.g67455.t1/1-879	0.151761	0.270964	JAB domain-containing					
Acrdi.XM_015891746.1/1-741 | Monca.augustus.g31552.t1/1-651	0.548187	0.270875	SURP and G-patch domain-containing 1					
Acrdi.XM_015906031.1/1-1443 | Monca.augustus.g68933.t1/1-1476	0.28983	0.270785	cyclic nucleotide-binding domain-containing 2-like isoform X1	2	P:GO:0016310; F:GO:0016301	P:phosphorylation; F:kinase activity		
Acrdi.XM_015899957.1/1-576 | Monca.augustus.g44173.t1/1-1551	0.313396	0.270745	PREDICTED: uncharacterized protein LOC107334983 isoform X2					
Acrdi.XM_015911813.1/1-630 | Monca.augustus.g29294.t1/1-543	0.471631	0.270666	homeobox ESX1	3	C:GO:0005634; P:GO:0006355; F:GO:0043565	C:nucleus; P:regulation of transcription, DNA-templated; F:sequence-specific DNA binding		
Acrdi.XM_015919333.1/1-336 | Monca.augustus.g5194.t1/1-330	0.25723	0.270621	mitochondrial	5	P:GO:0006506; C:GO:0000506; C:GO:0016020; C:GO:0016021;	P:GPI anchor biosynthetic process; C:glycosylphosphatidylinositol-N-acetylglucosaminyltransferase (GPI-GnT) complex; C:membrane; C:integral component of membrane; F:molecular_function		
Acrdi.XM_015905254.1/1-495 | Monca.augustus.g1754.t1/1-513	0.272235	0.270569	neutral phospholipase A2 agkistrodotoxin-like	5	F:GO:0047498; F:GO:0005509; P:GO:0035899; P:GO:0044398;	F:calcium-dependent phospholipase A2 activity; F:calcium ion binding; P:negative regulation of blood coagulation in other organism; P:envenomation resulting in induction of	EC:3.1.1.1; EC:3.1.1.4	Carboxylesterase; Phospholipase A(2)
Acrdi.XM_015897943.1/1-1557 | Monca.augustus.g23832.t1/1-2568	0.259088	0.270569	Proline- glutamic acid- and leucine-rich 1	8	C:GO:0005634; C:GO:0005737; P:GO:0006351; C:GO:0035327;	C:nucleus; C:cytoplasm; P:transcription, DNA-templated; C:transcriptionally active chromatin; F:chromatin binding; P:positive regulation of transcription from RNA polymerase II promoter; P:cellular response to estrogen stimulus; C:MLL1 complex		
Acrdi.XM_015912577.1/1-408 | Monca.augustus.g63188.t1/1-426	0.434684	0.270554	PREDICTED: uncharacterized protein LOC107346761					
Acrdi.XM_015921234.1/1-1137 | Monca.augustus.g67783.t1/1-1227	0.235649	0.270532	TPA: opsin	9	P:GO:0071482; F:GO:0004930; C:GO:0005887; P:GO:0007186;	P:cellular response to light stimulus; F:G-protein coupled receptor activity; C:integral component of plasma membrane; P:G-protein coupled receptor signaling pathway; P:detection of visible light; F:G-protein coupled photoreceptor activity; C:membrane; C:integral component of membrane; P:phototransduction		
Acrdi.XM_015910236.1/1-627 | Monca.augustus.g25476.t1/1-633	0.470787	0.270514	diencephalon mesencephalon homeobox 1	4	P:GO:0030901; P:GO:0060041; P:GO:0051726; P:GO:0050767	P:midbrain development; P:retina development in camera-type eye; P:regulation of cell cycle; P:regulation of neurogenesis		
Acrdi.XM_015905280.1/1-1170 | Monca.augustus.g23174.t1/1-1224	0.128774	0.270432	Wnt-5a-like isoform X1	87	P:GO:0040037; P:GO:0032092; P:GO:0043507; P:GO:0060762;	P:negative regulation of fibroblast growth factor receptor signaling pathway; P:positive regulation of protein binding; P:positive regulation of JUN kinase activity; P:regulation of branching involved in mammary gland duct morphogenesis; P:midgut development; P:male gonad development; P:planar cell polarity pathway involved in neural tube closure; P:positive regulation of endocytosis; P:positive regulation of interleukin-1 beta secretion; F:frizzled-2 binding; P:JNK cascade		
Acrdi.XM_015907023.1/1-2046 | Monca.augustus.g43285.t1/1-2127	0.242841	0.270424	coagulation factor V isoform X2	4	F:GO:0070492; C:GO:0016020; C:GO:0016021; C:GO:0030246	F:oligosaccharide binding; C:membrane; C:integral component of membrane; F:carbohydrate binding		
Acrdi.XM_015917251.1/1-3753 | Monca.augustus.g48810.t1/1-4140	0.323098	0.270411	flocculation FLO11-like isoform X3	4	C:GO:0005813; P:GO:0007052; C:GO:0097546; P:GO:0031122	C:centrosome; P:mitotic spindle organization; C:ciliary base; P:cytoplasmic microtubule organization		
Acrdi.XM_015896540.1/1-2100 | Monca.augustus.g17757.t1/1-2742	0.27019	0.270404	MAM and LDL-receptor class A domain-containing 1-like	2	C:GO:0016020; C:GO:0016021	C:membrane; C:integral component of membrane		
Acrdi.XM_015919204.1/1-402 | Monca.augustus.g54933.t1/1-318	0.246562	0.270357	PREDICTED: uncharacterized protein LOC107352883					
Acrdi.XM_015903602.1/1-1776 | Monca.augustus.g23065.t1/1-1806	0.249963	0.270182	PREDICTED: uncharacterized protein LOC107338370					
Acrdi.XM_015900085.1/1-567 | Monca.augustus.g43398.t1/1-573	0.270342	0.270161	neurogenic locus notch homolog 3-like	5	F:GO:0008061; P:GO:0006030; C:GO:0016020; C:GO:0016021;	F:chitin binding; P:chitin metabolic process; C:membrane; C:integral component of membrane; C:extracellular region		
Acrdi.XM_015918806.1/1-732 | Monca.adi2mcaRNA29886_R0/1-741	0.23248	0.27013	PREDICTED: uncharacterized protein LOC107352476					
Acrdi.XM_015906176.1/1-1491 | Monca.augustus.g9784.t1/1-1959	0.302347	0.270129	EF-hand domain-containing D1	2	C:GO:0016020; C:GO:0016021	C:membrane; C:integral component of membrane		
Acrdi.XM_015921868.1/1-564 | Monca.adi2mcaRNA33258_R0/1-534	0.131344	0.270113	PREDICTED: uncharacterized protein LOC107355324					
Acrdi.XM_015891855.1/1-810 | Monca.augustus.g17276.t1/1-1182	0.0757703	0.270094	PREDICTED: uncharacterized protein LOC107327103					
Acrdi.XM_015901405.1/1-996 | Monca.augustus.g31346.t1/1-1011	0.292801	0.269921	probable G- coupled receptor 157	7	F:GO:0004930; F:GO:0004888; P:GO:0007186; C:GO:0016020;	F:G-protein coupled receptor activity; F:transmembrane signaling receptor activity; P:G-protein coupled receptor signaling pathway; C:membrane; C:integral component of membrane; P:cell surface receptor signaling pathway; F:cAMP binding		
Acrdi.XM_015908420.1/1-786 | Monca.adi2mcaRNA27400_R0/1-1227	0.069199	0.269871	nuclease HARBI1					
Acrdi.XM_015896877.1/1-891 | Monca.augustus.g53973.t1/1-936	0.227704	0.26984	PREDICTED: uncharacterized protein LOC107332153					
Acrdi.XM_015910482.1/1-2958 | Monca.adi2mcaRNA2054_R0/1-2997	0.26454	0.269679	Extracellular calcium-sensing receptor	4	P:GO:0007186; C:GO:0044425; F:GO:0004871; C:GO:0005886	P:G-protein coupled receptor signaling pathway; C:membrane part; F:signal transducer activity; C:plasma membrane		
Acrdi.XM_015897054.1/1-1152 | Monca.augustus.g48649.t1/1-1527	0.225046	0.269518	SPT2 homolog					
Acrdi.XM_015899446.1/1-4575 | Monca.augustus.g46755.t1/1-4458	0.277432	0.269461	PREDICTED: uncharacterized protein LOC107334510					
Acrdi.XM_015913513.1/1-4107 | Monca.augustus.g25375.t1/1-4197	0.376969	0.269352	Retinoic acid-induced 1	5	C:GO:0005634; F:GO:0003700; F:GO:0008270; F:GO:0044212;	C:nucleus; F:transcription factor activity, sequence-specific DNA binding; F:zinc ion binding; F:transcription regulatory region DNA binding; P:positive regulation of transcription from RNA polymerase II promoter		
Acrdi.XM_015919026.1/1-819 | Monca.augustus.g56502.t1/1-828	0.31978	0.269245	polypeptide N-acetylgalactosaminyltransferase 2	8	F:GO:0016757; C:GO:0016020; C:GO:0016021; P:GO:0006486;	F:transferase activity, transferring glycosyl groups; C:membrane; C:integral component of membrane; P:protein glycosylation; C:Golgi membrane; C:Golgi apparatus; F:transferase activity; F:carbohydrate binding		
Acrdi.XM_015893103.1/1-2253 | Monca.augustus.g52209.t1/1-2241	0.32786	0.269225	TNFAIP3-interacting 1 isoform X1					
Acrdi.XM_015913982.1/1-2751 | Monca.augustus.g59716.t1/1-2787	0.298279	0.269196	baculoviral IAP repeat-containing 7	12	P:GO:1990001; F:GO:0004842; C:GO:0005876; C:GO:0005634;	P:inhibition of cysteine-type endopeptidase activity involved in apoptotic process; F:ubiquitin-protein transferase activity; C:spindle microtubule; C:nucleus; F:zinc ion binding; F:cysteine-type endopeptidase inhibitor activity; C:cytoplasm; F:metal ion binding; P:regulation of apoptotic process; P:regulation of signal transduction; P:mitotic spindle assembly; P:protein ubiquitination		
Acrdi.XM_015912983.1/1-612 | Monca.augustus.g64767.t1/1-597	0.305396	0.269181	homeobox not2-					
Acrdi.XM_015892832.1/1-1293 | Monca.augustus.g26652.t1/1-411	0.296129	0.269147	RNA-directed DNA polymerase from mobile element jockey					
Acrdi.XM_015919470.1/1-1740 | Monca.augustus.g25235.t1/1-1728	0.304084	0.269133	50S ribosomal L2					
Acrdi.XM_015901258.1/1-684 | Monca.augustus.g61810.t1/1-660	0.173838	0.269042	AKT-interacting isoform X3	6	F:GO:0061630; F:GO:0031625; C:GO:0016020; C:GO:0044444;	F:ubiquitin protein ligase activity; F:ubiquitin protein ligase binding; C:membrane; C:cytoplasmic part; P:transport; P:cellular process		
Acrdi.XM_015893501.1/1-1398 | Monca.augustus.g53469.t1/1-1668	0.253767	0.26898	Sugar phosphate exchanger					
Acrdi.XM_015920152.1/1-1068 | Monca.augustus.g27086.t1/1-1191	0.264388	0.268937	Zinc finger Gfi-1b	2	F:GO:0003676; F:GO:0046872	F:nucleic acid binding; F:metal ion binding		
Acrdi.XM_015903342.1/1-5622 | Monca.augustus.g36045.t1/1-5655	0.302629	0.268919	endoribonuclease Dicer	21	P:GO:0014013; P:GO:0045664; P:GO:0035195; P:GO:0035295;	P:regulation of gliogenesis; P:regulation of neuron differentiation; P:gene silencing by miRNA; P:tube development; P:cellular component assembly; P:production of small RNA involved in	EC:3.1.30	Acting on ester bonds
Acrdi.XM_015923896.1/1-1539 | Monca.adi2mcaRNA35619_R0/1-1569	0.272944	0.268802	coadhesin-like isoform X2	3	C:GO:0016020; F:GO:0005044; P:GO:0006898	C:membrane; F:scavenger receptor activity; P:receptor-mediated endocytosis		
Acrdi.XM_015910361.1/1-558 | Monca.adi2mcaRNA20508_R0/1-513	0.232618	0.268654	60S ribosomal L35	11	P:GO:0000463; P:GO:0019083; P:GO:0006614; F:GO:0003735;	P:maturation of LSU-rRNA from tricistronic rRNA transcript (SSU-rRNA, 5.8S rRNA, LSU-rRNA); P:viral transcription; P:SRP-dependent cotranslational protein targeting to membrane; F:structural constituent of ribosome; P:nuclear-transcribed mRNA catabolic process, nonsense-mediated decay; C:membrane; F:ribonucleoprotein complex binding; F:mRNA binding; C:cytosolic large ribosomal subunit; C:nucleolus; P:translational initiation		
Acrdi.XM_015907037.1/1-1467 | Monca.adi2mcaRNA16909_R0/1-1554	0.282677	0.2686	Actin-related 2 3 complex subunit 1A	2	C:GO:0016020; C:GO:0016021	C:membrane; C:integral component of membrane		
Acrdi.XM_015898845.1/1-2439 | Monca.augustus.g35506.t1/1-2820	0.300017	0.268557	Ribonucleases P MRP subunit POP1	1	P:GO:0006396	P:RNA processing		
Acrdi.XM_015919278.1/1-471 | Monca.augustus.g60225.t1/1-456	0.426633	0.268522	PREDICTED: uncharacterized protein LOC107352956					
Acrdi.XM_015914195.1/1-936 | Monca.augustus.g50916.t1/1-963	0.229302	0.268506	lipase -like	2	P:GO:0006629; F:GO:0016787	P:lipid metabolic process; F:hydrolase activity		
Acrdi.XM_015901653.1/1-5898 | Monca.augustus.g8590.t1/1-6015	0.0962522	0.268487	polycystic kidney disease 1-like 2					
Acrdi.XM_015911805.1/1-1656 | Monca.augustus.g24243.t1/1-1653	0.266248	0.268386	PREDICTED: uncharacterized protein LOC107346036					
Acrdi.XM_015900610.1/1-513 | Monca.augustus.g14893.t1/1-513	0.260429	0.268386	PREDICTED: uncharacterized protein LOC107335586					
Acrdi.XM_015893471.1/1-486 | Monca.augustus.g66996.t1/1-504	0.340881	0.268383	THAP domain-containing 4-like					
Acrdi.XM_015901712.1/1-354 | Monca.augustus.g12073.t1/1-564	0.243708	0.268332	Dihydrofolate reductase	1	P:GO:0044710	P:single-organism metabolic process		
Acrdi.XM_015917420.1/1-525 | Monca.adi2mcaRNA28341_R0/1-567	0.193955	0.268277	FAD-linked sulfhydryl oxidase ALR	2	F:GO:0016491; P:GO:0044699	F:oxidoreductase activity; P:single-organism process		
Acrdi.XM_015906298.1/1-552 | Monca.augustus.g21044.t1/1-516	0.300786	0.26827	fibroblast growth factor 2-like					
Acrdi.XM_015915074.1/1-1926 | Monca.augustus.g64353.t1/1-1944	0.285569	0.268243	PREDICTED: uncharacterized protein LOC107348980	2	C:GO:0016020; C:GO:0016021	C:membrane; C:integral component of membrane		
Acrdi.XM_015905875.1/1-960 | Monca.adi2mcaRNA1543_R1/1-1065	0.177055	0.268141	pyroglutamylated RFamide peptide receptor-like isoform X2					
Acrdi.XM_015896548.1/1-3036 | Monca.augustus.g22622.t1/1-3225	0.265201	0.268105	ATR-interacting	1	P:GO:0000077	P:DNA damage checkpoint		
Acrdi.XM_015908116.1/1-4578 | Monca.augustus.g33245.t1/1-4344	0.210353	0.267947	RNA polymerase II-associated 1	2	C:GO:0005634; P:GO:0006366	C:nucleus; P:transcription from RNA polymerase II promoter		
Acrdi.XM_015910373.1/1-1605 | Monca.augustus.g70893.t1/1-1632	0.28848	0.267927	Fibrinogen C domain-containing 1	2	C:GO:0016020; C:GO:0016021	C:membrane; C:integral component of membrane		
Acrdi.XM_015907080.1/1-534 | Monca.augustus.g40153.t1/1-774	0.216478	0.267891	Zinc finger CCCH domain-containing 14	3	F:GO:0003723; P:GO:0010468; C:GO:0044424	F:RNA binding; P:regulation of gene expression; C:intracellular part		
Acrdi.XM_015915422.1/1-564 | Monca.augustus.g32714.t1/1-957	0.372689	0.267814	leucine-rich repeats and immunoglobulin-like domains 3					
Acrdi.XM_015904725.1/1-1011 | Monca.augustus.g36185.t1/1-1026	0.333892	0.267701	PREDICTED: uncharacterized protein LOC107339440					
Acrdi.XM_015896182.1/1-405 | Monca.augustus.g33749.t1/1-393	0.256377	0.267632	p53 and DNA damage-regulated 1	7	P:GO:0008150; C:GO:0005737; C:GO:0016272; F:GO:0051082;	P:biological_process; C:cytoplasm; C:prefoldin complex; F:unfolded protein binding; P:protein folding; C:cellular_component; F:molecular_function		
Acrdi.XM_015917471.1/1-1338 | Monca.adi2mcaRNA28387_R0/1-1113	0.30071	0.267595	fibrinogen alpha chain-like					
Acrdi.XM_015922849.1/1-735 | Monca.augustus.g23926.t1/1-756	0.244769	0.26757	cysteine-rich motor neuron 1 -like isoform X1					
Acrdi.XM_015905487.1/1-801 | Monca.augustus.g28285.t1/1-690	0.250046	0.267401	ETS domain-containing Elk-3-like	5	C:GO:0005634; P:GO:0030154; F:GO:0043565; F:GO:0000981;	C:nucleus; P:cell differentiation; F:sequence-specific DNA binding; F:RNA polymerase II transcription factor activity, sequence-specific DNA binding; P:regulation of transcription from RNA polymerase II promoter		
Acrdi.XM_015906214.1/1-681 | Monca.augustus.g21698.t1/1-687	0.373204	0.267349	PREDICTED: uncharacterized protein LOC107340824					
Acrdi.XM_015910850.1/1-708 | Monca.augustus.g15801.t1/1-969	0.227426	0.267338	PREDICTED: uncharacterized protein LOC107345143 isoform X1					
Acrdi.XM_015909604.1/1-2295 | Monca.adi2mcaRNA19704_R0/1-2343	0.339805	0.267302	synergin gamma isoform X3					
Acrdi.XM_015891636.1/1-819 | Monca.adi2mcaRNA37490_R0/1-606	0.231585	0.267201	peptide methionine sulfoxide reductase-like	4	P:GO:0030091; P:GO:0006979; F:GO:0008113; P:GO:0055114	P:protein repair; P:response to oxidative stress; F:peptide-methionine (S)-S-oxide reductase activity; P:oxidation-reduction process	EC:1.8.4.11	Peptide-methionine (S)-S-oxide reductase
Acrdi.XM_015903403.1/1-717 | Monca.adi2mcaRNA12777_R0/1-732	0.295615	0.267118	Ribonuclease Oy	3	P:GO:0090502; F:GO:0003723; F:GO:0033897	P:RNA phosphodiester bond hydrolysis, endonucleolytic; F:RNA binding; F:ribonuclease T2 activity		
Acrdi.XM_015922292.1/1-1236 | Monca.adi2mcaRNA3932_R0/1-1173	0.36806	0.267103	limbic system-associated membrane -like isoform X2					
Acrdi.XM_015901668.1/1-2319 | Monca.augustus.g54282.t1/1-2427	0.252008	0.266935	MAGUK p55 subfamily member 7 isoform X2	3	C:GO:0071212; P:GO:0008582; P:GO:0048812	C:subsynaptic reticulum; P:regulation of synaptic growth at neuromuscular junction; P:neuron projection morphogenesis		
Acrdi.XM_015898067.1/1-1029 | Monca.augustus.g29241.t1/1-1197	0.298779	0.266855	CNPPD1	2	F:GO:0019901; P:GO:0000079	F:protein kinase binding; P:regulation of cyclin-dependent protein serine/threonine kinase activity		
Acrdi.XM_015900537.1/1-660 | Monca.augustus.g49619.t1/1-2091	0.356636	0.266746	PREDICTED: uncharacterized protein LOC107335514					
Acrdi.XM_015913861.1/1-1164 | Monca.augustus.g70084.t1/1-1083	0.265527	0.266588	UPF0764 C16orf89 homolog	2	C:GO:0016020; C:GO:0016021	C:membrane; C:integral component of membrane		
Acrdi.XM_015918682.1/1-2310 | Monca.augustus.g56501.t1/1-2811	0.250879	0.266581	ribosomal S6 kinase-like 1 isoform X1	7	F:GO:0005524; C:GO:0005737; P:GO:0007165; C:GO:0043231;	F:ATP binding; C:cytoplasm; P:signal transduction; C:intracellular membrane-bounded organelle; F:protein kinase activity; F:protein serine/threonine kinase activity; P:protein phosphorylation		
Acrdi.XM_015901678.1/1-1179 | Monca.augustus.g8583.t1/1-1215	0.237377	0.266573	PREDICTED: uncharacterized protein LOC107336614	2	C:GO:0016020; C:GO:0016021	C:membrane; C:integral component of membrane		
Acrdi.XM_015896075.1/1-2283 | Monca.augustus.g14824.t1/1-2268	0.233617	0.266481	DNA endonuclease RBBP8	4	P:GO:0000077; P:GO:0000724; F:GO:0000014; P:GO:0007049	P:DNA damage checkpoint; P:double-strand break repair via homologous recombination; F:single-stranded DNA endodeoxyribonuclease activity; P:cell cycle		
Acrdi.XM_015899807.1/1-1077 | Monca.adi2mcaRNA8970_R0/1-708	0.159009	0.266423	E3 ubiquitin- ligase	6	P:GO:0008150; F:GO:0008270; F:GO:0046872; F:GO:0016874;	P:biological_process; F:zinc ion binding; F:metal ion binding; F:ligase activity; F:molecular_function; P:protein ubiquitination		
Acrdi.XM_015898152.1/1-1839 | Monca.augustus.g29267.t1/1-1860	0.238843	0.266405	mitogen-activated kinase kinase kinase 2-like	4	F:GO:0000166; F:GO:0005524; F:GO:0004672; P:GO:0006468	F:nucleotide binding; F:ATP binding; F:protein kinase activity; P:protein phosphorylation		
Acrdi.XM_015907472.1/1-1611 | Monca.augustus.g67731.t1/1-2421	0.240506	0.266389	ATP-dependent RNA helicase DDX51	5	F:GO:0000166; F:GO:0003676; F:GO:0005524; F:GO:0004386;	F:nucleotide binding; F:nucleic acid binding; F:ATP binding; F:helicase activity; F:hydrolase activity		
Acrdi.XM_015920724.1/1-1221 | Monca.adi2mcaRNA32015_R0/1-1071	0.372133	0.266352	ATP-dependent DNA helicase Q1					
Acrdi.XM_015905951.1/1-1005 | Monca.augustus.g16086.t1/1-1014	0.25086	0.266318	PREDICTED: uncharacterized protein LOC107340587					
Acrdi.XM_015913921.1/1-2661 | Monca.augustus.g59525.t1/1-2070	0.285291	0.26625	poly [ADP-ribose] polymerase 14-like					
Acrdi.XM_015922774.1/1-1488 | Monca.augustus.g39160.t1/1-2913	0.440147	0.266249	DNA repair-scaffolding					
Acrdi.XM_015908057.1/1-3552 | Monca.augustus.g20213.t1/1-4497	0.315685	0.266233	flocculation FLO11-like					
Acrdi.XM_015900091.1/1-1050 | Monca.augustus.g47742.t1/1-1086	0.266002	0.266153	neuropeptide Y receptor-like	3	P:GO:0007186; C:GO:0016020; F:GO:0004871	P:G-protein coupled receptor signaling pathway; C:membrane; F:signal transducer activity		
Acrdi.XM_015916062.1/1-5298 | Monca.augustus.g5230.t1/1-5415	0.350879	0.266035	Centrosomal C10orf90-like					
Acrdi.XM_015908759.1/1-1017 | Monca.augustus.g24456.t1/1-1140	0.151958	0.26603	Fez family zinc finger 2	2	F:GO:0003676; F:GO:0046872	F:nucleic acid binding; F:metal ion binding		
Acrdi.XM_015921448.1/1-1017 | Monca.augustus.g24908.t1/1-993	0.275578	0.26599	heparan sulfate glucosamine 3-O-sulfotransferase 5	1	F:GO:0016740	F:transferase activity		
Acrdi.XM_015905669.1/1-459 | Monca.augustus.g26849.t1/1-426	0.347489	0.265944	PREDICTED: uncharacterized protein LOC107340323					
Acrdi.XM_015907528.1/1-918 | Monca.augustus.g23759.t1/1-1065	0.284337	0.265819	PREDICTED: uncharacterized protein LOC107342067					
Acrdi.XM_015921076.1/1-1074 | Monca.augustus.g34983.t1/1-1584	0.25989	0.265749	proline-rich transmembrane 4-like	2	C:GO:0016020; C:GO:0016021	C:membrane; C:integral component of membrane		
Acrdi.XM_015911568.1/1-1422 | Monca.augustus.g45454.t1/1-1452	0.220919	0.265648	28S ribosomal mitochondrial					
Acrdi.XM_015906038.1/1-1239 | Monca.augustus.g34437.t1/1-1383	0.265336	0.265586	WD repeat-containing 18	3	C:GO:0005737; P:GO:0007275; C:GO:0005654	C:cytoplasm; P:multicellular organism development; C:nucleoplasm		
Acrdi.XM_015908524.1/1-561 | Monca.augustus.g65970.t1/1-561	0.331119	0.265551	PREDICTED: uncharacterized protein LOC107342998	2	C:GO:0016020; C:GO:0016021	C:membrane; C:integral component of membrane		
Acrdi.XM_015907578.1/1-552 | Monca.adi2mcaRNA17486_R0/1-549	0.0772087	0.265516	serine arginine repetitive matrix 2-like	4	P:GO:0090305; F:GO:0003677; C:GO:0005634; F:GO:0004518	P:nucleic acid phosphodiester bond hydrolysis; F:DNA binding; C:nucleus; F:nuclease activity		
Acrdi.XM_015899481.1/1-693 | Monca.augustus.g37230.t1/1-1341	0.282056	0.265494	PREDICTED: nucleolin-like					
Acrdi.XM_015906963.1/1-1164 | Monca.adi2mcaRNA116_R0/1-1209	0.220773	0.265478	Caspase-3	4	P:GO:0006915; P:GO:0006508; F:GO:0004197; P:GO:0008234	P:apoptotic process; P:proteolysis; F:cysteine-type endopeptidase activity; F:cysteine-type peptidase activity		
Acrdi.XM_015906090.1/1-1158 | Monca.adi2mcaRNA15775_R0/1-1164	0.302373	0.265464	PREDICTED: uncharacterized protein LOC107340713	2	C:GO:0016020; C:GO:0016021	C:membrane; C:integral component of membrane		
Acrdi.XM_015923885.1/1-528 | Monca.augustus.g60451.t1/1-1029	0.284033	0.265408	PREDICTED: uncharacterized protein LOC107357244					
Acrdi.XM_015896717.1/1-789 | Monca.augustus.g11235.t1/1-960	0.292682	0.265315	melatonin receptor type 1B-like	8	F:GO:0004930; C:GO:0005887; P:GO:0007186; P:GO:0007165;	F:G-protein coupled receptor activity; C:integral component of plasma membrane; P:G-protein coupled receptor signaling pathway; P:signal transduction; C:membrane; C:integral component of membrane; P:cell surface receptor signaling pathway; F:signal transducer activity		
Acrdi.XM_015924620.1/1-303 | Monca.augustus.g15768.t1/1-480	0.221426	0.26521	PREDICTED: uncharacterized protein LOC107357986					
Acrdi.XM_015898424.1/1-657 | Monca.augustus.g54963.t1/1-663	0.0803684	0.264882	MAM and LDL-receptor class A domain-containing 1-like isoform X1	3	C:GO:0005615; P:GO:0006508; F:GO:0004252	C:extracellular space; P:proteolysis; F:serine-type endopeptidase activity	EC:3.4.21	Acting on peptide bonds (peptidases)
Acrdi.XM_015904553.1/1-846 | Monca.augustus.g68292.t1/1-1443	0.0806698	0.264872	ATP-dependent DNA helicase PIF1	1	P:GO:0009987	P:cellular process		
Acrdi.XM_015892015.1/1-513 | Monca.augustus.g10038.t1/1-468	0.536265	0.264806	Transposon Ty3-G Gag-Pol poly					
Acrdi.XM_015907002.1/1-690 | Monca.augustus.g33392.t1/1-693	0.318573	0.264772	homeobox not2-like	14	P:GO:0061371; P:GO:0006355; P:GO:0048646; P:GO:0043565;	P:determination of heart left/right asymmetry; P:regulation of transcription, DNA-templated; P:anatomical structure formation involved in morphogenesis; F:sequence-specific DNA binding; P:embryonic heart tube morphogenesis; P:endocrine system development; P:gland development; P:axial mesoderm morphogenesis; C:nucleus; P:central nervous system development; P:glomerulus development; P:notochord development; P:cell differentiation; P:floor plate development		
Acrdi.XM_015908302.1/1-984 | Monca.augustus.g34621.t1/1-1551	0.29797	0.264761	Potassium voltage-gated channel subfamily F member 1	2	F:GO:0005249; C:GO:0016021	F:voltage-gated potassium channel activity; C:integral component of membrane		
Acrdi.XM_015905210.1/1-501 | Monca.augustus.g52831.t1/1-558	0.309221	0.264719	UPF0547 C16orf87 homolog					
Acrdi.XM_015923222.1/1-498 | Monca.augustus.g21707.t1/1-510	1.00919	0.264704	PREDICTED: uncharacterized protein LOC107356619 isoform X1					
Acrdi.XM_015906165.1/1-804 | Monca.augustus.g44122.t1/1-1113	0.238879	0.264632	Ceramide synthase 1	5	C:GO:0016020; C:GO:0016021; F:GO:0050291; P:GO:0046513;	C:membrane; C:integral component of membrane; F:sphingosine N-acyltransferase activity; P:ceramide biosynthetic process; C:endoplasmic reticulum		
Acrdi.XM_015903295.1/1-5256 | Monca.adi2mcaRNA1373_R0/1-5142	0.318639	0.26462	dual serine threonine and tyrosine kinase-like isoform X1	1	F:GO:0003824	F:catalytic activity		
Acrdi.XM_015917094.1/1-1125 | Monca.augustus.g42868.t1/1-1455	0.734261	0.264475	PREDICTED: uncharacterized protein LOC107350845					
Acrdi.XM_015902859.1/1-2502 | Monca.adi2mcaRNA12272_R0/1-2313	0.241155	0.264445	proto-oncogene tyrosine- kinase receptor Ret-like	3	F:GO:0005524; P:GO:0018108; F:GO:0004713	F:ATP binding; P:peptidyl-tyrosine phosphorylation; F:protein tyrosine kinase activity	EC:2.7.10	Transferring phosphorus-containing groups
Acrdi.XM_015896627.1/1-408 | Monca.adi2mcaRNA5492_R3/1-516	0.305969	0.264365	PREDICTED: uncharacterized protein LOC107331931					
Acrdi.XM_015903871.1/1-723 | Monca.augustus.g48088.t1/1-723	0.246542	0.264339	CD63 antigen	2	C:GO:0005887; P:GO:0007166	C:integral component of plasma membrane; P:cell surface receptor signaling pathway		
Acrdi.XM_015903516.1/1-2103 | Monca.augustus.g34549.t1/1-3378	0.321722	0.264215	A disintegrin and metallo ase with thrombospondin motifs 18	8	F:GO:0008270; P:GO:0007229; P:GO:0006508; F:GO:0008233;	F:zinc ion binding; P:integrin-mediated signaling pathway; P:proteolysis; F:peptidase activity; C:extracellular matrix; F:metalloendopeptidase activity; C:proteinaceous extracellular matrix; F:metallopeptidase activity		
Acrdi.XM_015923081.1/1-5109 | Monca.augustus.g70886.t1/1-7650	0.346104	0.264164	nucleosome-remodeling factor subunit BPTF-like					
Acrdi.XM_015924132.1/1-1302 | Monca.augustus.g60421.t1/1-1440	0.197067	0.263822	OTU domain-containing 3					
Acrdi.XM_015920996.1/1-324 | Monca.adi2mcaRNA32304_R8/1-399	0.355671	0.263763	junction-mediating and -regulatory -like					
Acrdi.XM_015901997.1/1-819 | Monca.augustus.g2312.t1/1-930	0.278613	0.263741	PREDICTED: uncharacterized protein LOC107336917 isoform X1					
Acrdi.XM_015904964.1/1-903 | Monca.augustus.g46463.t1/1-900	0.139574	0.26361	galanin receptor type 1-like	2	P:GO:0007165; C:GO:0016020	P:signal transduction; C:membrane		
Acrdi.XM_015915399.1/1-1461 | Monca.augustus.g32689.t1/1-1182	0.255899	0.263555	PREDICTED: uncharacterized protein LOC107349280	3	C:GO:0016592; F:GO:0001105; P:GO:0045944	C:mediator complex; F:RNA polymerase II transcription coactivator activity; P:positive regulation of transcription from RNA polymerase II promoter		
Acrdi.XM_015902241.1/1-2190 | Monca.adi2mcaRNA1099_R0/1-2160	0.268916	0.263443	adhesion G -coupled receptor L4-like	6	F:GO:0004930; F:GO:0004888; P:GO:0007186; C:GO:0016020;	F:G-protein coupled receptor activity; F:transmembrane signaling receptor activity; P:G-protein coupled receptor signaling pathway; C:membrane; C:integral component of membrane; P:cell surface receptor signaling pathway		
Acrdi.XM_015913075.1/1-402 | Monca.augustus.g15094.t1/1-807	0.241483	0.263414	riken cdna family					
Acrdi.XM_015920430.1/1-495 | Monca.augustus.g13916.t1/1-489	0.252641	0.263384	PREDICTED: uncharacterized protein LOC107354031					
Acrdi.XM_015895726.1/1-447 | Monca.augustus.g35426.t1/1-345	0.289468	0.263349	PREDICTED: uncharacterized protein LOC107331172					
Acrdi.XM_015900092.1/1-1146 | Monca.adi2mcaRNA9212_R0/1-1110	0.250457	0.263231	neuropeptide Y receptor-like	2	P:GO:0007165; C:GO:0016020	P:signal transduction; C:membrane		
Acrdi.XM_015916485.1/1-555 | Monca.augustus.g9025.t1/1-882	0.208274	0.263223	PREDICTED: uncharacterized protein LOC107350264 isoform X1					
Acrdi.XM_015903877.1/1-1266 | Monca.adi2mcaRNA13338_R0/1-1239	0.22742	0.26322	neuropeptide Y receptor-like	2	P:GO:0007165; F:GO:0004871	P:signal transduction; F:signal transducer activity		
Acrdi.XM_015911070.1/1-606 | Monca.augustus.g46918.t1/1-600	0.336787	0.263205	PREDICTED: uncharacterized protein LOC107345365					
Acrdi.XM_015925415.1/1-348 | Monca.adi2mcaRNA37374_R0/1-342	0.132997	0.263152	strawberry notch-like isoform X1	1	P:GO:0006355	P:regulation of transcription, DNA-templated		
Acrdi.XM_015914128.1/1-441 | Monca.augustus.g24985.t1/1-438	0.145593	0.263134	radial spoke head 10 homolog B-like	3	P:GO:0019538; P:GO:0016310; F:GO:0016301	P:protein metabolic process; P:phosphorylation; F:kinase activity		
Acrdi.XM_015896667.1/1-636 | Monca.augustus.g36999.t1/1-654	0.324698	0.263083	PREDICTED: uncharacterized protein LOC107331973					
Acrdi.XM_015917681.1/1-351 | Monca.augustus.g11958.t1/1-357	0.407541	0.262983	PREDICTED: uncharacterized protein LOC107351393					
Acrdi.XM_015910230.1/1-825 | Monca.augustus.g25475.t1/1-822	0.423495	0.262955	diencephalon mesencephalon homeobox 1 isoform X2					
Acrdi.XM_015913824.1/1-1854 | Monca.augustus.g17352.t1/1-1374	0.27502	0.262825	MAM and LDL-receptor class A domain-containing 2-like isoform X6					

Acrdi.XM_015901109.1/1-801 | Monca.augustus.g1896.t1/1-1236	0.277982	0.262801	ectonucleoside triphosphate diphosphohydrolase 1-like isoform X1	6	F:GO:0017110; P:GO:1901135; P:GO:0044763; P:GO:0044238;	F:nucleoside-diphosphatase activity; P:carbohydrate derivative metabolic process; P:single-organism cellular process; P:primary metabolic process; P:cellular metabolic proce [illegible]
Acrdi.XM_015898824.1/1-10743 | Monca.augustus.g30623.t1/1-10644	0.153622	0.262797	type I polyketide synthase	1	F:GO:0003824	F:catalytic activity
Acrdi.XM_015911407.1/1-2250 | Monca.augustus.g53234.t1/1-2727	0.264745	0.262774	RNA-binding 28	3	F:GO:0000166; F:GO:0003676; F:GO:0003723	F:nucleotide binding; F:nucleic acid binding; F:RNA binding
Acrdi.XM_015891825.1/1-1200 | Monca.augustus.g43710.t1/1-1185	0.272045	0.262528	serine protease 23-like	2	C:GO:0005576; F:GO:0016787	C:extracellular region; F:hydrolase activity
Acrdi.XM_015914203.1/1-1251 | Monca.adi2mcaRNA24733_R0/1-1245	0.337266	0.262521	DNA replication factor Cdt1			
Acrdi.XM_015917982.1/1-666 | Monca.augustus.g45183.t1/1-2034	0.225963	0.262512	PREDICTED: uncharacterized protein LOC107351694			
Acrdi.XM_015913825.1/1-945 | Monca.augustus.g26430.t1/1-891	0.220497	0.262471	transcription factor Sox-14-like	1	F:GO:0043565	F:sequence-specific DNA binding
Acrdi.XM_015902435.1/1-1506 | Monca.augustus.g37592.t1/1-1578	0.250009	0.262424	PREDICTED: uncharacterized protein LOC107337302			
Acrdi.XM_015918405.1/1-489 | Monca.augustus.g22400.t1/1-498	0.405053	0.262288	PREDICTED: uncharacterized protein LOC107352087			
Acrdi.XM_015917999.1/1-324 | Monca.augustus.g15268.t1/1-336	0.461133	0.262061	PREDICTED: uncharacterized protein LOC107351699 isoform X2	1	C:GO:0005739	C:mitochondrion
Acrdi.XM_015907907.1/1-408 | Monca.augustus.g11080.t1/1-1233	0.420736	0.262005	PREDICTED: uncharacterized protein LOC107342408			
Acrdi.XM_015903735.1/1-1692 | Monca.augustus.g41345.t1/1-1683	0.456374	0.261941	transcription factor SPT20 homolog			
Acrdi.XM_015904480.1/1-666 | Monca.augustus.g52725.t1/1-678	0.312151	0.261839	PREDICTED: uncharacterized protein LOC107339220			
Acrdi.XM_015905715.1/1-450 | Monca.augustus.g1925.t1/1-951	0.269179	0.261427	PREDICTED: uncharacterized protein LOC107340359			
Acrdi.XM_015912525.1/1-1650 | Monca.adi2mcaRNA22887_R0/1-1590	0.275537	0.261414	von Willebrand factor D and EGF domain-containing -like	4	F:GO:0008061; F:GO:0005509; P:GO:0006030; C:GO:0005576	F:chitin binding; F:calcium ion binding; P:chitin metabolic process; C:extracellular region
Acrdi.XM_015898390.1/1-393 | Monca.augustus.g35068.t1/1-333	0.0807813	0.261378	neuronal stem cell leukemia 2	1	F:GO:0005488	F:binding
Acrdi.XM_015915236.1/1-1395 | Monca.augustus.g41245.t1/1-1404	0.237495	0.261301	neuronal acetylcholine receptor subunit alpha-5	2	C:GO:0016020; P:GO:0006810	C:membrane; P:transport
Acrdi.XM_015904040.1/1-1083 | Monca.augustus.g27763.t1/1-1062	0.240884	0.261285	D(2) dopamine receptor A-like	2	P:GO:0007186; C:GO:0016020	P:G-protein coupled receptor signaling pathway; C:membrane
Acrdi.XM_015912407.1/1-1881 | Monca.augustus.g40701.t1/1-1950	0.268818	0.261281	platelet glyco 4	12	P:GO:0048584; P:GO:0010033; P:GO:0048522; P:GO:0007165;	P:positive regulation of response to stimulus; P:response to organic substance; P:positive regulation of cellular process; P:signal transduction; C:membrane; C:cell part; P:lipid local [illegible]
Acrdi.XM_015893586.1/1-675 | Monca.augustus.g60501.t1/1-570	0.287908	0.261021	phosphatase 1 regulatory subunit 3D-like			
Acrdi.XM_015900355.1/1-1284 | Monca.augustus.g69135.t1/1-1299	0.275565	0.26096	PREDICTED: uncharacterized protein LOC107335341			
Acrdi.XM_015919576.1/1-414 | Monca.augustus.g52105.t1/1-786	0.277295	0.260931	collagen alpha-1(XII) chain	8	C:GO:0005615; C:GO:0070062; F:GO:0005509; C:GO:0016020;	C:extracellular space; C:extracellular exosome; F:calcium ion binding; C:membrane; P:endodermal cell differentiation; P:cell adhesion; C:extracellular matrix; P:homophilic cell adh [illegible]
Acrdi.XM_015893547.1/1-429 | Monca.augustus.g25409.t1/1-432	0.105378	0.260894	Zinc finger CCHC domain-containing 10	1	F:GO:0005515	F:protein binding
Acrdi.XM_015908808.1/1-2457 | Monca.augustus.g8666.t1/1-2463	0.437767	0.260864	PREDICTED: uncharacterized protein LOC107343236			
Acrdi.XM_015902299.1/1-405 | Monca.augustus.g35092.t1/1-405	0.308929	0.260846	PREDICTED: uncharacterized protein LOC107337180			
Acrdi.XM_015923661.1/1-939 | Monca.augustus.g40001.t1/1-900	0.250426	0.260794	proline-rich transmembrane 4-like	2	C:GO:0016020; C:GO:0016021	C:membrane; C:integral component of membrane
Acrdi.XM_015913008.1/1-1188 | Monca.augustus.g51629.t1/1-1260	0.250936	0.260793	PREDICTED: uncharacterized protein LOC107347131			
Acrdi.XM_015919685.1/1-363 | Monca.adi2mcaRNA3109_R0/1-375	0.147529	0.260701	PREDICTED: uncharacterized protein LOC107353334			
Acrdi.XM_015915272.1/1-1413 | Monca.augustus.g46179.t1/1-2181	0.274794	0.26057	Cell surface glyco 1	2	C:GO:0016020; C:GO:0016021	C:membrane; C:integral component of membrane
Acrdi.XM_015896076.1/1-1575 | Monca.augustus.g14823.t1/1-1614	0.216134	0.260551	PREDICTED: uncharacterized protein LOC107331487			
Acrdi.XM_015910766.1/1-735 | Monca.augustus.g29673.t1/1-723	0.329456	0.260548	PREDICTED: uncharacterized protein LOC107345071 isoform X2			
Acrdi.XM_015922056.1/1-1794 | Monca.adi2mcaRNA33468_R0/1-2085	0.0820154	0.260214	PREDICTED: uncharacterized protein LOC107355480			
Acrdi.XM_015921866.1/1-873 | Monca.augustus.g61102.t1/1-1026	0.323975	0.260212	uncharacterized skeletal organic matrix 8-like			
Acrdi.XM_015909023.1/1-1353 | Monca.augustus.g38377.t1/1-1356	0.328961	0.260182	2 ,3 -cyclic-nucleotide 3 -phosphodiesterase-like	4	C:GO:0005737; C:GO:0016020; P:GO:0009214; F:GO:0004113	C:cytoplasm; C:membrane; P:cyclic nucleotide catabolic process; F:2',3'-cyclic-nucleotide 3'-phosphodiesterase activity
Acrdi.XM_015925413.1/1-600 | Monca.augustus.g4252.t1/1-714	0.31774	0.259963	PREDICTED: uncharacterized protein LOC107358835 isoform X1			
Acrdi.XM_015912391.1/1-3300 | Monca.augustus.g45159.t1/1-3831	0.281087	0.259884	DNA mismatch repair Mlh3 isoform X1	6	C:GO:0005712; F:GO:0016887; C:GO:0032300; P:GO:0006298;	C:chiasma; F:ATPase activity; C:mismatch repair complex; P:mismatch rep [illegible]
Acrdi.XM_015913650.1/1-2331 | Monca.adi2mcaRNA2390_R0/1-2406	0.295227	0.259728	Asparagine synthetase domain-containing 1	1	P:GO:1901605	P:alpha-amino acid metabolic process
Acrdi.XM_015898292.1/1-2376 | Monca.augustus.g62897.t1/1-2415	0.361331	0.259704	centriolar coiled-coil of 110 kDa isoform X1	1	P:GO:0051298	P:centrosome duplication
Acrdi.XM_015922675.1/1-1809 | Monca.augustus.g69571.t1/1-2436	0.200836	0.25966	PDZ and LIM domain 5 isoform X9	1	F:GO:0008270	F:zinc ion binding
Acrdi.XM_015923754.1/1-654 | Monca.augustus.g10019.t1/1-612	0.11786	0.259658	PREDICTED: uncharacterized protein LOC107357103			
Acrdi.XM_015914803.1/1-882 | Monca.augustus.g47149.t1/1-867	0.223232	0.259612	PREDICTED: uncharacterized protein LOC107348737	2	C:GO:0016020; C:GO:0016021	C:membrane; C:integral component of membrane
Acrdi.XM_015893252.1/1-1233 | Monca.augustus.g48122.t1/1-1203	0.21948	0.259572	solute carrier family 35 member G1	2	C:GO:0016020; C:GO:0016021	C:membrane; C:integral component of membrane
Acrdi.XM_015892430.1/1-1068 | Monca.augustus.g32806.t1/1-1092	0.247553	0.259562	Cell growth-regulating nucleolar	1	C:GO:0005634	C:nucleus
Acrdi.XM_015921294.1/1-1014 | Monca.augustus.g20085.t1/1-1257	0.0986797	0.259488	nucleoplasmin ANO39	3	F:GO:0003676; C:GO:0031981; P:GO:0044763	F:nucleic acid binding; C:nuclear lumen; P:single-organism cellular process
Acrdi.XM_015897320.1/1-1905 | Monca.adi2mcaRNA6195_R0/1-1851	0.152552	0.259472	Cyclic GMP-AMP synthase			
Acrdi.XM_015903959.1/1-504 | Monca.augustus.g41776.t1/1-564	0.272304	0.259344	growth arrest-specific 1-like			
Acrdi.XM_015898406.1/1-630 | Monca.augustus.g58529.t1/1-1161	0.228792	0.259163	RAB6-interacting golgin			
Acrdi.XM_015903775.1/1-837 | Monca.adi2mcaRNA13238_R0/1-825	0.147424	0.259104	melanocyte-stimulating hormone receptor-like	7	F:GO:0004930; F:GO:0004977; P:GO:0007186; P:GO:0007165;	F:G-protein coupled receptor activity; F:melanocortin receptor activity; P:G [illegible]
Acrdi.XM_015902785.1/1-909 | Monca.augustus.g33301.t1/1-909	0.243909	0.259037	neurocan core	4	P:GO:0006790; P:GO:1903510; P:GO:0006029; P:GO:0043436	P:sulfur compound metabolic process; P:mucopolysaccharide metabolic pr [illegible]
Acrdi.XM_015907177.1/1-564 | Monca.augustus.g12551.t1/1-858	0.200483	0.259033	PREDICTED: uncharacterized protein LOC107341713	1	P:GO:0042981	P:regulation of apoptotic process
Acrdi.XM_015913666.1/1-669 | Monca.augustus.g30496.t1/1-660	0.210841	0.25896	transcription factor Maf-like	11	C:GO:0005634; P:GO:0021571; P:GO:0021572; P:GO:0009880;	C:nucleus; P:rhombomere 5 development; P:rhombomere 6 development; [illegible]
Acrdi.XM_015923051.1/1-651 | Monca.augustus.g20806.t1/1-717	0.293768	0.258804	PREDICTED: uncharacterized protein LOC107356436			
Acrdi.XM_015923361.1/1-663 | Monca.augustus.g51722.t1/1-2352	0.330345	0.258669	splicing suppressor of white-apricot homolog	1	P:GO:0006396	P:RNA processing
Acrdi.XM_015919210.1/1-849 | Monca.augustus.g54946.t1/1-768	0.258708	0.258618	Major facilitator superfamily domain-containing 6-A	1	F:GO:0004622	F:lysophospholipase activity
Acrdi.XM_015923335.1/1-1800 | Monca.augustus.g32975.t1/1-1884	0.273646	0.258474	PREDICTED: uncharacterized protein LOC107356682	2	C:GO:0016020; C:GO:0016021	C:membrane; C:integral component of membrane
Acrdi.XM_015901382.1/1-1590 | Monca.augustus.g17231.t1/1-1686	0.299701	0.258423	fibroblast growth factor receptor-like 1	2	C:GO:0016020; C:GO:0016021	C:membrane; C:integral component of membrane
Acrdi.XM_015913390.1/1-1260 | Monca.augustus.g58022.t1/1-1251	0.234665	0.258353	PREDICTED: uncharacterized protein LOC107347458			
Acrdi.XM_015896989.1/1-714 | Monca.augustus.g28879.t1/1-759	0.271645	0.258229	PREDICTED: uncharacterized protein LOC107332261			
Acrdi.XM_015902438.1/1-1371 | Monca.augustus.g37599.t1/1-1380	0.287999	0.25803	monocarboxylate transporter 10-like	1	P:GO:0006810	P:transport
Acrdi.XM_015918533.1/1-600 | Monca.augustus.g23499.t1/1-579	0.22335	0.258025	LWamide neuropeptide			
Acrdi.XM_015918333.1/1-873 | Monca.augustus.g41655.t1/1-2373	0.203778	0.258007	nucleoporin GLE1-like			
Acrdi.XM_015903115.1/1-1020 | Monca.augustus.g52460.t1/1-1029	0.245724	0.258002	beta-2 adrenergic receptor-like	16	P:GO:0065008; P:GO:0007165; P:GO:0044707; P:GO:0007267;	P:regulation of biological quality; P:signal transduction; P:single-multicellu [illegible]
Acrdi.XM_015915046.1/1-1053 | Monca.augustus.g49274.t1/1-1035	0.286818	0.257979	Glutamate receptor NMDA 1			
Acrdi.XM_015891639.1/1-1080 | Monca.augustus.g21638.t1/1-1125	0.323221	0.25779	GCN5 family acetyltransferase			
Acrdi.XM_015908923.1/1-1512 | Monca.adi2mcaRNA18945_R0/1-1575	0.194004	0.257621	kielin chordin	7	F:GO:0016491; F:GO:0046872; P:GO:0008152; C:GO:0016020;	F:oxidoreductase activity; F:metal ion binding; P:metabolic process; C:mem [illegible]
Acrdi.XM_015911928.1/1-3402 | Monca.augustus.g58645.t1/1-4674	0.238846	0.257602	Hermansky-Pudlak syndrome 5 isoform X2	6	F:GO:0005515; C:GO:0005737; C:GO:0031084; P:GO:0043473;	F:protein binding; C:cytoplasm; C:BLOC-2 complex; P:pigmentation; P:bloo [illegible]
Acrdi.XM_015892849.1/1-1287 | Monca.augustus.g13411.t1/1-1815	0.254133	0.257593	PREDICTED: uncharacterized protein LOC107328122			
Acrdi.XM_015916808.1/1-603 | Monca.augustus.g1824.t1/1-741	0.130558	0.257589	PREDICTED: uncharacterized protein LOC107350571	3	P:GO:0055085; F:GO:0022857; C:GO:0016021	P:transmembrane transport; F:transmembrane transporter activity; C:integ [illegible]
Acrdi.XM_015916375.1/1-1971 | Monca.augustus.g40951.t1/1-1935	0.225203	0.257588	SUMO-specific isopeptidase USPL1	6	P:GO:0008283; F:GO:0070140; F:GO:0032183; P:GO:0016926;	P:cell proliferation; F:SUMO-specific isopeptidase activity; F:SUMO binding [illegible]
Acrdi.XM_015920929.1/1-3882 | Monca.augustus.g53578.t1/1-3873	0.270155	0.257551	PREDICTED: uncharacterized protein LOC107354445 isoform X1	4	P:GO:0042273; C:GO:0016020; C:GO:0016021; P:GO:0006397	P:ribosomal large subunit biogenesis; C:membrane; C:integral component [illegible]
Acrdi.XM_015906116.1/1-1257 | Monca.augustus.g10607.t1/1-1350	0.365309	0.257478	lava lamp-like			
Acrdi.XM_015895741.1/1-1809 | Monca.augustus.g59287.t1/1-1782	0.28828	0.257375	Polycystic kidney disease 1-like 2	1	F:GO:0070492	F:oligosaccharide binding
Acrdi.XM_015919639.1/1-6936 | Monca.adi2mcaRNA30850_R0/1-7053	0.303346	0.257355	Fanconi anemia group M isoform X3	2	F:GO:0097159; F:GO:1901363	F:organic cyclic compound binding; F:heterocyclic compound binding
Acrdi.XM_015900402.1/1-786 | Monca.augustus.g46625.t1/1-744	0.278411	0.25732	14-3-3 family	1	F:GO:0019904	F:protein domain specific binding
Acrdi.XM_015909077.1/1-1065 | Monca.adi2mcaRNA19110_R0/1-1050	0.33781	0.257297	proline-rich transmembrane 4-like	2	C:GO:0016020; C:GO:0016021	C:membrane; C:integral component of membrane
Acrdi.XM_015925468.1/1-4149 | Monca.augustus.g58565.t1/1-4125	0.299737	0.257212	PREDICTED: uncharacterized protein LOC107358898			
Acrdi.XM_015900262.1/1-600 | Monca.augustus.g40543.t1/1-621	0.117025	0.257057	PREDICTED: uncharacterized protein LOC107335269			
Acrdi.XM_015901676.1/1-1209 | Monca.augustus.g56492.t1/1-1248	0.377433	0.256992	PREDICTED: uncharacterized protein LOC107336613			
Acrdi.XM_015917904.1/1-552 | Monca.augustus.g14693.t1/1-555	0.228108	0.256925	PREDICTED: uncharacterized protein LOC107351610			
Acrdi.XM_015906513.1/1-993 | Monca.augustus.g22566.t1/1-1056	0.368531	0.256923	inhibitor of activated STAT	13	F:GO:0008270; C:GO:0035861; P:GO:0007131; P:GO:0016925;	F:zinc ion binding; C:site of double-strand break; P:reciprocal meiotic recom [illegible]
Acrdi.XM_015896743.1/1-312 | Monca.augustus.g15639.t1/1-375	0.0754036	0.256871	Nuclear transport	2	P:GO:0006810; C:GO:0005622	P:transport; C:intracellular
Acrdi.XM_015897151.1/1-1431 | Monca.augustus.g63823.t1/1-1536	0.139056	0.256769	prolyl 3-hydroxylase OGFOD1	2	F:GO:0016706; C:GO:0044424	F:oxidoreductase activity, acting on paired donors, with incorporation or re [illegible]
Acrdi.XM_015898385.1/1-1701 | Monca.adi2mcaRNA7440_R3/1-2538	0.0773438	0.256754	Transposon Ty3-G Gag-Pol poly			
Acrdi.XM_015920162.1/1-387 | Monca.augustus.g27093.t1/1-1650	0.251655	0.256708	RNA polymerase II subunit B1 CTD phosphatase RPAP2 isoform X2	4	C:GO:0005634; C:GO:0005737; P:GO:0070940; F:GO:0008420	C:nucleus; C:cytoplasm; P:dephosphorylation of RNA polymerase II C-termi [illegible]
Acrdi.XM_015903164.1/1-933 | Monca.adi2mcaRNA12529_R0/1-933	0.2777	0.256661	melanocyte-stimulating hormone receptor-like			
Acrdi.XM_015898462.1/1-1038 | Monca.augustus.g33988.t1/1-1599	0.272484	0.256658	histone-lysine N-methyltransferase PRDM6	1	F:GO:0005488	F:binding
Acrdi.XM_015925461.1/1-2157 | Monca.adi2mcaRNA37448_R0/1-2031	0.18438	0.25655	Anaphase-promoting complex subunit 2	4	F:GO:0061630; F:GO:0031625; C:GO:0005680; P:GO:0042787	F:ubiquitin protein ligase activity; F:ubiquitin protein ligase binding; C:anap [illegible]
Acrdi.XM_015920972.1/1-624 | Monca.augustus.g30430.t1/1-1299	0.17209	0.256548	sodium metabolite cotransporter chloroplastic	2	C:GO:0016020; C:GO:0016021	C:membrane; C:integral component of membrane
Acrdi.XM_015912819.1/1-678 | Monca.augustus.g13250.t1/1-822	0.0793803	0.25651	formin 2-			
Acrdi.XM_015909508.1/1-1764 | Monca.augustus.g1392.t1/1-4584	0.255954	0.256404	coiled-coil domain-containing 18-like			
Acrdi.XM_015907328.1/1-936 | Monca.augustus.g20598.t1/1-945	0.263748	0.256166	phosphatase 1 regulatory subunit 27			
Acrdi.XM_015899709.1/1-615 | Monca.augustus.g66180.t1/1-624	0.289181	0.25602	outer membrane lipo Blc-like			
Acrdi.XM_015924967.1/1-3501 | Monca.augustus.g22978.t1/1-3381	0.286579	0.255986	Ankyrin repeat domain-containing	4	F:GO:0016614; F:GO:0050660; F:GO:0003824; P:GO:0055114	F:oxidoreductase activity, acting on CH-OH group of donors; F:flavin adenin [illegible]
Acrdi.XM_015906471.1/1-1416 | Monca.adi2mcaRNA1686_R3/1-1134	0.161001	0.255953	PREDICTED: uncharacterized protein LOC107341044			
Acrdi.XM_015909488.1/1-885 | Monca.augustus.g26311.t1/1-1176	0.243996	0.255936	Mitochondrial genome maintenance exonuclease 1	3	F:GO:0008297; C:GO:0005739; P:GO:0006264	F:single-stranded DNA exodeoxyribonuclease activity; C:mitochondrion; P:m [illegible]
Acrdi.XM_015896433.1/1-858 | Monca.augustus.g48021.t1/1-909	0.231532	0.255926	collagenase 3-like	1	F:GO:0016787	F:hydrolase activity
Acrdi.XM_015914564.1/1-999 | Monca.augustus.g13694.t1/1-864	0.312632	0.255764	somatostatin receptor type 1-like	9	F:GO:0004930; F:GO:0001588; P:GO:0001963; P:GO:0007186;	F:G-protein coupled receptor activity; F:dopamine neurotransmitter recept [illegible]
Acrdi.XM_015897962.1/1-1587 | Monca.adi2mcaRNA36952_R2/1-882	0.244815	0.255735	PREDICTED: uncharacterized protein LOC107333193			
Acrdi.XM_015920122.1/1-831 | Monca.adi2mcaRNA31354_R0/1-618	0.530075	0.255688	PREDICTED: uncharacterized protein LOC107353740			
Acrdi.XM_015893952.1/1-693 | Monca.augustus.g2205.t1/1-690	0.140917	0.255661	PREDICTED: uncharacterized protein LOC107329217	4	F:GO:0003676; P:GO:0006281; C:GO:0005634; F:GO:0008081	F:nucleic acid binding; P:DNA repair; C:nucleus; F:phosphoric diester hydrol [illegible]
Acrdi.XM_015906005.1/1-1707 | Monca.adi2mcaRNA15683_R0/1-1770	0.312473	0.255655	PREDICTED: uncharacterized protein LOC107340638	1	P:GO:0060828	P:regulation of canonical Wnt signaling pathway
Acrdi.XM_015897263.1/1-2043 | Monca.augustus.g41220.t1/1-3714	0.186103	0.255551	pleckstrin homology domain-containing family G member 1	6	P:GO:0035023; P:GO:0008150; F:GO:0005089; C:GO:0005575;	P:regulation of Rho protein signal transduction; P:biological_process; F:Rh [illegible]
Acrdi.XM_015912797.1/1-900 | Monca.augustus.g3595.t1/1-1698	0.242522	0.255499	Small RNA degrading nuclease 5	3	F:GO:0003676; F:GO:0003824; P:GO:0008152	F:nucleic acid binding; F:catalytic activity; P:metabolic process
Acrdi.XM_015911493.1/1-918 | Monca.augustus.g53653.t1/1-570	0.169149	0.255312	Golgi-associated plant pathogenesis-related 1	1	C:GO:0005576	C:extracellular region
Acrdi.XM_015898825.1/1-2856 | Monca.augustus.g30624.t1/1-3003	0.0781018	0.255273	patched domain-containing 3-like	1	C:GO:0016020	C:membrane
Acrdi.XM_015894939.1/1-786 | Monca.augustus.g35310.t1/1-1053	0.0777508	0.255249	pyroglutamylated RFamide peptide receptor-like isoform X2	2	P:GO:0007165; C:GO:0016020	P:signal transduction; C:membrane
Acrdi.XM_015918184.1/1-576 | Monca.augustus.g64473.t1/1-4500	0.278427	0.255094	brain-specific angiogenesis inhibitor 1-like			
Acrdi.XM_015893790.1/1-1632 | Monca.augustus.g32057.t1/1-2253	0.338803	0.255024	transmembrane 214	5	C:GO:0005789; P:GO:0006915; C:GO:0016020; C:GO:0016021;	C:endoplasmic reticulum membrane; P:apoptotic process; C:membrane; C: [illegible]
Acrdi.XM_015910963.1/1-3366 | Monca.adi2mcaRNA21168_R0/1-3441	0.301039	0.25501	PREDICTED: uncharacterized protein LOC107345261 isoform X2			
Acrdi.XM_015909720.1/1-558 | Monca.augustus.g41741.t1/1-1368	0.24437	0.254948	GON-4-			
Acrdi.XM_015897698.1/1-1632 | Monca.augustus.g56115.t1/1-1623	0.288097	0.254908	PREDICTED: uncharacterized protein LOC107332945			
Acrdi.XM_015918771.1/1-3612 | Monca.adi2mcaRNA29845_R0/1-3762	0.292529	0.254825	phosphatidylinositol phosphatase PTPRQ-like	2	C:GO:0016020; C:GO:0016021	C:membrane; C:integral component of membrane
Acrdi.XM_015908039.1/1-711 | Monca.augustus.g20211.t1/1-705	0.220753	0.25482	Hepatic leukemia factor	3	F:GO:0003700; P:GO:0006355; F:GO:0043565	F:transcription factor activity, sequence-specific DNA binding; P:regulation [illegible]
Acrdi.XM_015919088.1/1-546 | Monca.augustus.g12011.t1/1-543	0.362052	0.254799	PREDICTED: transmembrane inner ear expressed protein-like			
Acrdi.XM_015922732.1/1-438 | Monca.augustus.g51669.t1/1-345	0.277016	0.254755	B9 domain-containing			
Acrdi.XM_015902380.1/1-1779 | Monca.augustus.g61653.t1/1-1743	0.208578	0.254645	Cholinesterase	1	F:GO:0052689	F:carboxylic ester hydrolase activity
Acrdi.XM_015909180.1/1-894 | Monca.augustus.g42301.t1/1-3159	0.29677	0.254576	WSC domain-containing 1			
Acrdi.XM_015925328.1/1-3648 | Monca.augustus.g53778.t1/1-5604	0.356928	0.254536	Jumonji-like isoform X1	1	F:GO:0003677	F:DNA binding
Acrdi.XM_015910197.1/1-846 | Monca.augustus.g38619.t1/1-1056	0.296452	0.254525	probable G- coupled receptor 157	7	F:GO:0004930; F:GO:0004888; P:GO:0007186; C:GO:0016020;	F:G-protein coupled receptor activity; F:transmembrane signaling receptor [illegible]
Acrdi.XM_015904111.1/1-528 | Monca.augustus.g36725.t1/1-711	0.224972	0.254418	PREDICTED: uncharacterized protein LOC107338858			
Acrdi.XM_015918346.1/1-837 | Monca.augustus.g41650.t1/1-810	0.316928	0.254395	transmembrane 163-like	6	P:GO:0055085; P:GO:0006812; P:GO:0098655; C:GO:0016020;	P:transmembrane transport; P:cation transport; P:cation transmembrane t [illegible]
Acrdi.XM_015916705.1/1-1962 | Monca.adi2mcaRNA27552_R0/1-1821	0.263248	0.254194	arginyl-tRNA-- transferase 1 isoform X2	4	F:GO:0016746; F:GO:0016740; F:GO:0004057; P:GO:0016598	F:transferase activity, transferring acyl groups; F:transferase activity; F:arg [illegible]
Acrdi.XM_015901007.1/1-354 | Monca.augustus.g57618.t1/1-459	0.348212	0.254175	UPF0538 C2orf76 homolog			
Acrdi.XM_015897486.1/1-933 | Monca.augustus.g35245.t1/1-951	0.309379	0.253963	melanocyte-stimulating hormone receptor-like	7	F:GO:0004930; P:GO:0007186; P:GO:0007165; C:GO:0016020;	F:G-protein coupled receptor activity; P:G-protein coupled receptor signali [illegible]
Acrdi.XM_015909112.1/1-3405 | Monca.augustus.g67744.t1/1-2244	0.369264	0.253944	Proline and serine-rich 3			
Acrdi.XM_015894044.1/1-744 | Monca.augustus.g49892.t1/1-948	0.327572	0.253943	LEM 2-like isoform X2	2	C:GO:0016020; C:GO:0016021	C:membrane; C:integral component of membrane
Acrdi.XM_015917325.1/1-1983 | Monca.augustus.g25546.t1/1-2031	0.304928	0.253938	inner nuclear membrane Man1-like	1	C:GO:0016020	C:membrane
Acrdi.XM_015910997.1/1-1743 | Monca.augustus.g59075.t1/1-1698	0.311229	0.25392	major facilitator superfamily domain-containing 6 isoform X1	2	C:GO:0016020; C:GO:0016021	C:membrane; C:integral component of membrane
Acrdi.XM_015917833.1/1-684 | Monca.augustus.g23508.t1/1-2841	0.274473	0.253918	PREDICTED: uncharacterized protein LOC107351537			
Acrdi.XM_015907430.1/1-720 | Monca.augustus.g67993.t1/1-867	0.225032	0.253917	TNF receptor-associated factor 5-like	7	F:GO:0003677; F:GO:0004842; F:GO:0008270; F:GO:0046872;	F:DNA binding; F:ubiquitin-protein transferase activity; F:zinc ion binding; [illegible]
Acrdi.XM_015896713.1/1-669 | Monca.adi2mcaRNA557_R2/1-495	0.620106	0.253869	sterile alpha motif domain-containing 3			
Acrdi.XM_015897725.1/1-663 | Monca.augustus.g9973.t1/1-660	0.255205	0.253752	NK1 transcription factor-related 2	3	C:GO:0005634; P:GO:0006355; F:GO:0043565	C:nucleus; P:regulation of transcription, DNA-templated; F:sequence-speci [illegible]
Acrdi.XM_015917879.1/1-474 | Monca.augustus.g25189.t1/1-459	0.209873	0.253717	PREDICTED: uncharacterized protein LOC107351587			
Acrdi.XM_015901194.1/1-909 | Monca.augustus.g58578.t1/1-912	0.275932	0.253704	EGF-like repeat and discoidin I-like domain-containing 3	1	F:GO:0070492	F:oligosaccharide binding
Acrdi.XM_015901408.1/1-1914 | Monca.augustus.g9879.t1/1-2583	0.221606	0.253681	PREDICTED: uncharacterized protein LOC107336326	2	C:GO:0016020; C:GO:0016021	C:membrane; C:integral component of membrane
Acrdi.XM_015913694.1/1-2544 | Monca.augustus.g45549.t1/1-2667	0.317343	0.253635	Collagen alpha-1(XII) chain	2	C:GO:0016020; C:GO:0016021	C:membrane; C:integral component of membrane
Acrdi.XM_015907442.1/1-330 | Monca.augustus.g3962.t1/1-354	0.344712	0.253578	PREDICTED: uncharacterized protein LOC107341976			
Acrdi.XM_015915983.1/1-1383 | Monca.augustus.g60996.t1/1-1710	0.182436	0.253488	matrix metallo ase-25-like	9	F:GO:0008270; F:GO:0046872; F:GO:0005509; P:GO:0006508;	F:zinc ion binding; F:metal ion binding; F:calcium ion binding; P:proteolysis [illegible]
Acrdi.XM_015900615.1/1-471 | Monca.augustus.g71563.t1/1-768	0.236627	0.253484	Collagen alpha-3(VI) chain	2	C:GO:0016020; C:GO:0016021	C:membrane; C:integral component of membrane
Acrdi.XM_015920984.1/1-1239 | Monca.augustus.g51591.t1/1-1431	0.224449	0.253476	disrupted in renal carcinoma 2	1	P:GO:0006810	P:transport
Acrdi.XM_015896668.1/1-1377 | Monca.augustus.g37038.t1/1-2676	0.299542	0.253456	cyclic nucleotide-binding domain-containing 2-like			
Acrdi.XM_015917564.1/1-1149 | Monca.augustus.g44191.t1/1-1128	0.319001	0.25345	ankyrin repeat domain-containing 27-like			
Acrdi.XM_015904493.1/1-735 | Monca.augustus.g33598.t1/1-729	0.237901	0.253319	chloride intracellular channel 2	16	P:GO:0010880; P:GO:1902476; F:GO:0005244; F:GO:0005254;	P:regulation of release of sequestered calcium ion into cytosol by sarcopla [illegible]
Acrdi.XM_015916840.1/1-2928 | Monca.augustus.g55427.t1/1-3003	0.268718	0.253269	coiled-coil and C2 domain-containing 1-like	3	P:GO:0000122; C:GO:0005634; F:GO:0000981	P:negative regulation of transcription from RNA polymerase II promoter; C [illegible]
Acrdi.XM_015905098.1/1-687 | Monca.adi2mcaRNA14686_R0/1-717	0.19427	0.253197	latent-transforming growth factor beta-binding 1-like	3	F:GO:0005509; F:GO:0005201; C:GO:0005578	F:calcium ion binding; F:extracellular matrix structural constituent; C:prote [illegible]
Acrdi.XM_015920621.1/1-885 | Monca.augustus.g19865.t1/1-1083	0.310892	0.253105	adenosine receptor A3-like	8	F:GO:0004930; P:GO:0007186; P:GO:0007165; C:GO:0016020;	F:G-protein coupled receptor activity; P:G-protein coupled receptor signali [illegible]
Acrdi.XM_015907059.1/1-360 | Monca.augustus.g43296.t1/1-741	0.343309	0.253021	PREDICTED: uncharacterized protein LOC107341618			
Acrdi.XM_015919214.1/1-1113 | Monca.augustus.g43606.t1/1-1353	0.32911	0.252976	eva-1 homolog A isoform X1	3	C:GO:0016020; C:GO:0016021; F:GO:0030246	C:membrane; C:integral component of membrane; F:carbohydrate binding
Acrdi.XM_015923140.1/1-5622 | Monca.adi2mcaRNA373_R2/1-5496	0.284174	0.252949	polycystic kidney disease 1-like 2	6	P:GO:0050982; F:GO:0005509; F:GO:0005262; C:GO:0016020;	P:detection of mechanical stimulus; F:calcium ion binding; F:calcium chan [illegible]
Acrdi.XM_015893992.1/1-969 | Monca.augustus.g56585.t1/1-972	0.0644527	0.252868	ATP-dependent DNA helicase pfh1			
Acrdi.XM_015905604.1/1-1398 | Monca.augustus.g65061.t1/1-1932	0.261504	0.252861	onco -induced transcript 3 -like	2	C:GO:0016020; C:GO:0016021	C:membrane; C:integral component of membrane
Acrdi.XM_015896443.1/1-885 | Monca.augustus.g48056.t1/1-1119	0.3057	0.252749	matrix metallo ase-19-like	1	F:GO:0016787	F:hydrolase activity
Acrdi.XM_015918442.1/1-969 | Monca.adi2mcaRNA29504_R1/1-987	0.34464	0.252747	indian hedgehog -like			
Acrdi.XM_015919183.1/1-645 | Monca.augustus.g54950.t1/1-2970	0.236564	0.252641	selenocysteine insertion sequence-binding 2-like			
Acrdi.XM_015894752.1/1-468 | Monca.augustus.g4730.t1/1-468	0.323967	0.252536	#NAME?			
Acrdi.XM_015903368.1/1-1911 | Monca.augustus.g29881.t1/1-2328	0.335185	0.252489	SKI DACH domain-containing 1			
Acrdi.XM_015908495.1/1-2643 | Monca.adi2mcaRNA18420_R0/1-2709	0.263242	0.252476	helicase-like transcription factor			
Acrdi.XM_015910264.1/1-792 | Monca.adi2mcaRNA2018_R0/1-915	0.27838	0.25247	probable G- coupled receptor 157	7	F:GO:0004930; F:GO:0004888; P:GO:0007186; C:GO:0016020;	F:G-protein coupled receptor activity; F:transmembrane signaling receptor [illegible]
Acrdi.XM_015914419.1/1-912 | Monca.augustus.g4699.t1/1-903	0.131487	0.25247	epidermal retinol dehydrogenase 2-like	4	F:GO:0016491; C:GO:0016020; C:GO:0016021; P:GO:0055114	F:oxidoreductase activity; C:membrane; C:integral component of membran [illegible]
Acrdi.XM_015905853.1/1-828 | Monca.adi2mcaRNA15528_R0/1-693	0.166655	0.252464	cation-dependent mannose-6-phosphate receptor-like	2	C:GO:0016020; C:GO:0016021	C:membrane; C:integral component of membrane
Acrdi.XM_015909833.1/1-1134 | Monca.augustus.g47088.t1/1-1275	0.184086	0.252354	Arrestin domain-containing 3	1	P:GO:0006810	P:transport
Acrdi.XM_015915402.1/1-2550 | Monca.adi2mcaRNA26122_R0/1-2301	0.206515	0.25227	potassium sodium hyperpolarization-activated cyclic nucleotide-gated	5	P:GO:0006813; C:GO:0005887; F:GO:0005249; P:GO:0042391;	P:potassium ion transport; C:integral component of plasma membrane; F: [illegible]
Acrdi.XM_015909866.1/1-1338 | Monca.adi2mcaRNA19990_R1/1-1140	0.237159	0.252221	PREDICTED: uncharacterized protein LOC107344225			
Acrdi.XM_015905983.1/1-2616 | Monca.augustus.g41403.t1/1-3123	0.2222	0.252202	Actin-binding anillin			
Acrdi.XM_015899780.1/1-771 | Monca.augustus.g27103.t1/1-717	0.0633714	0.25204	Tetratricopeptide repeat 28			
Acrdi.XM_015897508.1/1-2091 | Monca.augustus.g32448.t1/1-2076	0.224603	0.251915	PREDICTED: uncharacterized protein LOC107332759			
Acrdi.XM_015891987.1/1-843 | Monca.augustus.g32104.t1/1-819	0.268905	0.251829	forkhead box L1-like	8	F:GO:0003677; C:GO:0005634; F:GO:0003700; P:GO:0006351;	F:DNA binding; C:nucleus; F:transcription factor activity, sequence-specific [illegible]
Acrdi.XM_015914497.1/1-744 | Monca.adi2mcaRNA25068_R0/1-750	0.330985	0.251771	PREDICTED: uncharacterized protein LOC107348457			
Acrdi.XM_015922884.1/1-552 | Monca.augustus.g46871.t1/1-588	0.281165	0.251767	Venom prothrombin activator pseutarin-C non-catalytic subunit			
Acrdi.XM_015901005.1/1-711 | Monca.augustus.g39841.t1/1-693	0.36202	0.251753	leukocyte tyrosine kinase receptor-like			
Acrdi.XM_015900576.1/1-1365 | Monca.augustus.g33646.t1/1-1362	0.290304	0.251673	cGMP-dependent isozyme 1			
Acrdi.XM_015909064.1/1-2421 | Monca.augustus.g13102.t1/1-3099	0.284581	0.251655	basic helix-loop-helix domain-containing KIAA2018-like			
Acrdi.XM_015919529.1/1-867 | Monca.augustus.g22480.t1/1-1842	0.111307	0.25163	PREDICTED: uncharacterized protein LOC107353203 isoform X1			
Acrdi.XM_015908067.1/1-1938 | Monca.augustus.g17403.t1/1-1965	0.257306	0.251618	CUB domain-containing 2			
Acrdi.XM_015897117.1/1-2088 | Monca.augustus.g55681.t1/1-2133	0.321096	0.251608	PREDICTED: uncharacterized protein LOC107332384			
Acrdi.XM_015903163.1/1-945 | Monca.augustus.g52459.t1/1-1023	0.265542	0.251535	dopamine receptor 2-like	3	F:GO:0004930; P:GO:0007154; P:GO:0044700	F:G-protein coupled receptor activity; P:cell communication; P:single organ [illegible]
Acrdi.XM_015909228.1/1-546 | Monca.augustus.g21581.t1/1-531	0.379983	0.251281	Low density lipo receptor adapter 1-A			
Acrdi.XM_015898619.1/1-1773 | Monca.augustus.g7783.t1/1-1767	0.196077	0.251266	FAM63B-like isoform X4			
Acrdi.XM_015904258.1/1-1614 | Monca.augustus.g62120.t1/1-1680	0.251626	0.251213	FAM189A1 isoform X2	1	C:GO:0016020	C:membrane
Acrdi.XM_015918638.1/1-939 | Monca.augustus.g33667.t1/1-945	0.319815	0.251138	beta-1,3-galactosyltransferase 5-like	2	C:GO:0016020; F:GO:0016740	C:membrane; F:transferase activity
Acrdi.XM_015909701.1/1-840 | Monca.augustus.g55273.t1/1-837	0.306908	0.251118	FAM26E-like isoform X1	1	C:GO:0016020	C:membrane
Acrdi.XM_015899471.1/1-600 | Monca.augustus.g21745.t1/1-1362	0.193408	0.251043	intraflagellar transport 52 homolog			
Acrdi.XM_015901318.1/1-477 | Monca.augustus.g67931.t1/1-486	0.288101	0.250988	group 10 secretory phospholipase A2	7	F:GO:0004623; F:GO:0046872; F:GO:0005509; P:GO:0006629;	F:phospholipase A2 activity; F:metal ion binding; F:calcium ion binding; P:li [illegible]
Acrdi.XM_015911525.1/1-372 | Monca.augustus.g10119.t1/1-816	0.225014	0.250972	PREDICTED: uncharacterized protein LOC107345778			
Acrdi.XM_015895544.1/1-1785 | Monca.adi2mcaRNA4312_R0/1-1851	0.251287	0.250959	fibrillin-1-like isoform X4	3	F:GO:0005509; F:GO:0005201; C:GO:0005578	F:calcium ion binding; F:extracellular matrix structural constituent; C:prote [illegible]
Acrdi.XM_015923780.1/1-2763 | Monca.adi2mcaRNA35487_R0/1-2868	0.282444	0.250952	Tudor domain-containing 1			
Acrdi.XM_015920813.1/1-810 | Monca.augustus.g11611.t1/1-807	0.24576	0.250946	PREDICTED: uncharacterized protein LOC107354346	2	C:GO:0016020; C:GO:0016021	C:membrane; C:integral component of membrane
Acrdi.XM_015904069.1/1-1467 | Monca.augustus.g57750.t1/1-1473	0.0833805	0.250899	mab-21-like 2			
Acrdi.XM_015895320.1/1-1089 | Monca.augustus.g25966.t1/1-1557	0.219707	0.25081	uncharacterized skeletal organic matrix 2-like			
Acrdi.XM_015907084.1/1-1161 | Monca.augustus.g54992.t1/1-2028	0.232101	0.250789	Actin-related 2 3 complex subunit 1A	2	C:GO:0016020; C:GO:0016021	C:membrane; C:integral component of membrane
Acrdi.XM_015892101.1/1-819 | Monca.adi2mcaRNA37989_R0/1-801	0.301508	0.250775	SH3 domain-containing YSC84 1			
Acrdi.XM_015903340.1/1-3093 | Monca.augustus.g32348.t1/1-3354	0.230798	0.250632	A disintegrin and metallo ase with thrombospondin motifs 6	7	F:GO:0008270; P:GO:0006508; F:GO:0008233; C:GO:0031012;	F:zinc ion binding; P:proteolysis; F:peptidase activity; C:extracellular matrix [illegible]
Acrdi.XM_015898661.1/1-2409 | Monca.adi2mcaRNA7685_R0/1-1875	0.250477	0.250529	aurora borealis			
Acrdi.XM_015903838.1/1-465 | Monca.adi2mcaRNA13304_R1/1-303	0.0783106	0.250469	G2 M phase-specific E3 ubiquitin- ligase-like			
Acrdi.XM_015895186.1/1-2436 | Monca.augustus.g13752.t1/1-1848	0.199745	0.250407	chorion peroxidase	8	F:GO:0004601; P:GO:0006979; F:GO:0010181; F:GO:0003824;	F:peroxidase activity; P:response to oxidative stress; F:FMN binding; F:cata [illegible]
Acrdi.XM_015896286.1/1-633 | Monca.augustus.g33694.t1/1-660	0.313804	0.250392	Rab 8 GTPase	1	C:GO:0005622	C:intracellular
Acrdi.XM_015899945.1/1-1008 | Monca.augustus.g32800.t1/1-999	0.234861	0.250278	PREDICTED: uncharacterized protein LOC107334973			
Acrdi.XM_015912799.1/1-633 | Monca.augustus.g3597.t1/1-627	0.280174	0.250247	A-kinase anchor 9-like			
Acrdi.XM_015905780.1/1-1296 | Monca.augustus.g32753.t1/1-1314	0.231942	0.25022	FAM69B-like isoform X1	3	F:GO:0005509; C:GO:0016020; C:GO:0016021	F:calcium ion binding; C:membrane; C:integral component of membrane
Acrdi.XM_015924966.1/1-585 | Monca.augustus.g41163.t1/1-594	0.3039	0.25001	PREDICTED: uncharacterized protein LOC107358361			
Acrdi.XM_015908336.1/1-894 | Monca.augustus.g36784.t1/1-897	0.287036	0.24997	Trace amine-associated receptor 5	6	F:GO:0004930; P:GO:0007186; C:GO:0016020; C:GO:0016021;	F:G-protein coupled receptor activity; P:G-protein coupled receptor signali [illegible]
Acrdi.XM_015893101.1/1-810 | Monca.augustus.g13165.t1/1-750	0.353611	0.249929	sidekick-1 isoform X1	2	C:GO:0016020; C:GO:0016021	C:membrane; C:integral component of membrane
Acrdi.XM_015920928.1/1-3699 | Monca.adi2mcaRNA32255_R0/1-3672	0.281895	0.249925	OTU domain-containing 4 isoform X6			
Acrdi.XM_015911332.1/1-1269 | Monca.augustus.g35280.t1/1-1218	0.206781	0.24981	Lysophospholipid acyltransferase 7	1	C:GO:0016020	C:membrane
Acrdi.XM_015892316.1/1-1470 | Monca.adi2mcaRNA3941_R9/1-1536	0.80471	0.249764	RNA-directed DNA polymerase from mobile element jockey-like			
Acrdi.XM_015912671.1/1-2763 | Monca.adi2mcaRNA2378_R0/1-2751	0.159554	0.24975	von Willebrand factor A domain-containing 7-like	2	C:GO:0016020; C:GO:0016021	C:membrane; C:integral component of membrane
Acrdi.XM_015915304.1/1-879 | Monca.adi2mcaRNA2579_R0/1-936	0.236267	0.249599	bone morphogenetic	1	F:GO:0016787	F:hydrolase activity
Acrdi.XM_015897680.1/1-654 | Monca.adi2mcaRNA611_R8/1-687	0.0673963	0.249514	PREDICTED: uncharacterized protein LOC107332926			
Acrdi.XM_015913223.1/1-690 | Monca.augustus.g32059.t1/1-678	0.235701	0.249475	PREDICTED: uncharacterized protein LOC107347326			
Acrdi.XM_015915672.1/1-1347 | Monca.augustus.g28531.t1/1-1659	0.244224	0.249425	hexosaminidase D-like	8	P:GO:0005975; P:GO:0008152; C:GO:0016020; C:GO:0016021;	P:carbohydrate metabolic process; P:metabolic process; C:membrane; C:in [illegible]
Acrdi.XM_015893249.1/1-567 | Monca.augustus.g48120.t1/1-1176	0.203171	0.249395	nucleolar and spindle-associated 1-A-like	5	P:GO:0040001; P:GO:0000281; C:GO:0005819; P:GO:0000226;	P:establishment of mitotic spindle localization; P:mitotic cytokinesis; C:spi [illegible]
Acrdi.XM_015900481.1/1-1137 | Monca.augustus.g1363.t1/1-975	0.219442	0.24939	myeloid differentiation primary response 88	7	P:GO:0006954; P:GO:0002755; C:GO:0005737; P:GO:0007165;	P:inflammatory response; P:MyD88-dependent toll-like receptor signaling [illegible]
Acrdi.XM_015916168.1/1-861 | Monca.augustus.g70519.t1/1-873	0.264513	0.249369	PREDICTED: uncharacterized protein LOC107349972			
Acrdi.XM_015907668.1/1-1542 | Monca.augustus.g29937.t1/1-1602	0.281674	0.249308	telomeric repeat-binding factor 2-interacting 1	12	C:GO:0005635; F:GO:0005515; C:GO:0005737; F:GO:0042162;	C:nuclear envelope; F:protein binding; C:cytoplasm; F:telomeric DNA bindin [illegible]
Acrdi.XM_015897745.1/1-1164 | Monca.augustus.g17408.t1/1-1518	0.120918	0.249297	zinc transporter Slc39a7-like	7	P:GO:0006829; C:GO:0043231; C:GO:0016020; C:GO:0012505;	P:zinc II ion transport; C:intracellular membrane-bounded organelle; C:mer [illegible]
Acrdi.XM_015901633.1/1-837 | Monca.augustus.g19769.t1/1-1122	0.18239	0.249275	PREDICTED: uncharacterized protein C7orf57-like			
Acrdi.XM_015911825.1/1-393 | Monca.augustus.g39601.t1/1-1044	0.256582	0.249228	histamine H2 receptor-like	3	P:GO:0007165; C:GO:0016020; F:GO:0004871	P:signal transduction; C:membrane; F:signal transducer activity
Acrdi.XM_015911642.1/1-1932 | Monca.augustus.g4722.t1/1-2184	0.180208	0.249142	extracellular matrix A-like	3	F:GO:0005509; F:GO:0046539; P:GO:0032259	F:calcium ion binding; F:histamine N-methyltransferase activity; P:methyla [illegible]
Acrdi.XM_015891945.1/1-6729 | Monca.augustus.g33211.t1/1-7575	0.237413	0.249105	agrin-like isoform X2	1	F:GO:0005509	F:calcium ion binding
Acrdi.XM_015892382.1/1-321 | Monca.augustus.g36499.t1/1-330	0.296045	0.249099	PREDICTED: uncharacterized protein LOC107327635			
Acrdi.XM_015921508.1/1-885 | Monca.augustus.g22991.t1/1-867	0.215182	0.249071	alpha- kinase vwkA-like	5	F:GO:0005524; P:GO:0016310; F:GO:0004674; P:GO:0006468;	F:ATP binding; P:phosphorylation; F:protein serine/threonine kinase activity [illegible]
Acrdi.XM_015923235.1/1-1281 | Monca.augustus.g69881.t1/1-1239	0.262188	0.249061	E3 ubiquitin- ligase E3D			
Acrdi.XM_015920105.1/1-1152 | Monca.augustus.g11419.t1/1-792	0.167345	0.248973	ras association domain-containing 8-like	5	P:GO:0040015; C:GO:0005912; P:GO:0007165; P:GO:0048749;	P:negative regulation of multicellular organism growth; C:adherens junctio [illegible]
Acrdi.XM_015897148.1/1-417 | Monca.augustus.g63840.t1/1-978	0.447076	0.24889	PREDICTED: uncharacterized protein LOC107332417			
Acrdi.XM_015900528.1/1-378 | Monca.augustus.g1359.t1/1-675	0.278806	0.248815	manganese superoxide dismutase	1	P:GO:0008152	P:metabolic process
Acrdi.XM_015899678.1/1-363 | Monca.augustus.g64261.t1/1-783	0.281771	0.248808	calbindin isoform X2	1	F:GO:0005509	F:calcium ion binding
Acrdi.XM_015892775.1/1-1602 | Monca.augustus.g50413.t1/1-1482	0.194724	0.248635	hemicentin-1-like isoform X1	3	P:GO:0007165; C:GO:0016020; C:GO:0016021	P:signal transduction; C:membrane; C:integral component of membrane
Acrdi.XM_015901433.1/1-483 | Monca.augustus.g31715.t1/1-759	0.195134	0.2484	Collagen alpha-5(VI) chain	10	F:GO:0030414; C:GO:0005634; F:GO:0008270; P:GO:0007218;	F:peptidase inhibitor activity; C:nucleus; F:zinc ion binding; P:neuropeptide [illegible]
Acrdi.XM_015905189.1/1-1173 | Monca.augustus.g57160.t1/1-1386	0.292205	0.248336	Collagen alpha-1(XII) chain			
Acrdi.XM_015902821.1/1-1584 | Monca.augustus.g24608.t1/1-3102	0.21264	0.248311	RNA-binding 25-like isoform X1			
Acrdi.XM_015910458.1/1-909 | Monca.augustus.g46395.t1/1-939	0.280324	0.248193	melanocyte-stimulating hormone receptor-like	6	F:GO:0004930; P:GO:0007186; P:GO:0007165; C:GO:0016020;	F:G-protein coupled receptor activity; P:G-protein coupled receptor signali [illegible]
Acrdi.XM_015920725.1/1-1182 | Monca.augustus.g63440.t1/1-948	0.107794	0.248144	Transposon TX1 uncharacterized 149 kDa			
Acrdi.XM_015906030.1/1-1878 | Monca.augustus.g27807.t1/1-1875	0.299584	0.248126	delta 4	2	C:GO:0016020; C:GO:0016021	C:membrane; C:integral component of membrane
Acrdi.XM_015892368.1/1-1362 | Monca.augustus.g38248.t1/1-1524	0.298499	0.247988	RNA-binding 34	2	F:GO:0000166; F:GO:0003676	F:nucleotide binding; F:nucleic acid binding
Acrdi.XM_015909549.1/1-735 | Monca.augustus.g16560.t1/1-825	0.315566	0.247921	PREDICTED: uncharacterized protein LOC107343949	1	F:GO:0043167	F:ion binding
Acrdi.XM_015900016.1/1-522 | Monca.augustus.g51848.t1/1-831	0.232092	0.247907	R3H domain-containing 4-like	3	F:GO:0003676; C:GO:0016020; C:GO:0016021	F:nucleic acid binding; C:membrane; C:integral component of membrane
Acrdi.XM_015900802.1/1-477 | Monca.augustus.g58760.t1/1-474	0.248905	0.247886	PREDICTED: prominin-2-like	2	C:GO:0016020; C:GO:0016021	C:membrane; C:integral component of membrane
Acrdi.XM_015903355.1/1-768 | Monca.augustus.g35992.t1/1-906	0.275677	0.247728	MAP3K12-binding inhibitory			
Acrdi.XM_015907889.1/1-1845 | Monca.augustus.g53179.t1/1-1851	0.272018	0.247723	kelch 12	7	F:GO:0004842; P:GO:0008584; C:GO:0005737; P:GO:0007286;	F:ubiquitin-protein transferase activity; P:male gonad development; C:cyto [illegible]
Acrdi.XM_015924611.1/1-492 | Monca.augustus.g15767.t1/1-546	0.271325	0.247705	PREDICTED: uncharacterized protein LOC107357977			
Acrdi.XM_015903066.1/1-1476 | Monca.augustus.g56756.t1/1-1473	0.20659	0.247694	PREDICTED: uncharacterized protein LOC107337831	2	C:GO:0016020; C:GO:0016021	C:membrane; C:integral component of membrane
Acrdi.XM_015908751.1/1-2763 | Monca.augustus.g26273.t1/1-2841	0.272275	0.2475	Fanconi anemia core complex-associated 100-like isoform X1	1	C:GO:0043229	C:intracellular organelle
Acrdi.XM_015900358.1/1-2388 | Monca.adi2mcaRNA9511_R6/1-1944	0.157261	0.247468	iron-regulated -like			
Acrdi.XM_015897782.1/1-1008 | Monca.augustus.g65409.t1/1-1515	0.3117	0.247394	F-box LRR-repeat 6			
Acrdi.XM_015916442.1/1-1134 | Monca.augustus.g61212.t1/1-396	0.0717318	0.247317	tachylectin-			
Acrdi.XM_015892449.1/1-342 | Monca.augustus.g69045.t1/1-585	0.273272	0.247242	chromodomain Y 2	3	C:GO:0005634; C:GO:0016020; C:GO:0016021	C:nucleus; C:membrane; C:integral component of membrane
Acrdi.XM_015894771.1/1-435 | Monca.augustus.g26539.t1/1-549	0.218175	0.24723	LKAAEAR1 isoform X2			
Acrdi.XM_015919447.1/1-621 | Monca.augustus.g52511.t1/1-795	0.247467	0.247213	carbonic anhydrase	6	P:GO:0042539; P:GO:0015670; P:GO:0097369; F:GO:0004089;	P:hypotonic salinity response; P:carbon dioxide transport; P:sodium ion imp [illegible]
Acrdi.XM_015915581.1/1-1608 | Monca.augustus.g5415.t1/1-2247	0.197822	0.2472	nucleolar complex 2 homolog	7	C:GO:0030689; F:GO:0005515; P:GO:0010605; C:GO:0031981;	C:Noc complex; F:protein binding; P:negative regulation of macromolecule [illegible]
Acrdi.XM_015893638.1/1-426 | Monca.augustus.g47258.t1/1-927	0.178191	0.24717	unconventional myosin-VIIb			
Acrdi.XM_015906040.1/1-10230 | Monca.augustus.g34432.t1/1-11130	0.318716	0.247122	PREDICTED: uncharacterized protein LOC107340675			
Acrdi.XM_015901622.1/1-4245 | Monca.adi2mcaRNA1174_R0/1-4014	0.284248	0.24711	myosin-M heavy chain-like	1	P:GO:0065007	P:biological regulation
Acrdi.XM_015916875.1/1-1170 | Monca.augustus.g71463.t1/1-1593	0.586046	0.247044	nucleotide-binding oligomerization domain-containing 2-like			
Acrdi.XM_015908650.1/1-1161 | Monca.augustus.g53874.t1/1-1200	0.244445	0.246915	FAM185A isoform X1	3	P:GO:0008150; C:GO:0005737; F:GO:0003674	P:biological_process; C:cytoplasm; F:molecular_function
Acrdi.XM_015915073.1/1-2322 | Monca.augustus.g64354.t1/1-2217	0.276457	0.246914	PREDICTED: uncharacterized protein LOC107348979	2	C:GO:0016020; C:GO:0016021	C:membrane; C:integral component of membrane
Acrdi.XM_015914689.1/1-450 | Monca.adi2mcaRNA25285_R3/1-423	0.596781	0.246831	P2X purinoceptor 7-like	7	F:GO:0004931; C:GO:0005887; C:GO:0005639; P:GO:0035590;	F:extracellular ATP-gated cation channel activity; C:integral component of [illegible]
Acrdi.XM_015917860.1/1-1566 | Monca.augustus.g31699.t1/1-1584	0.198188	0.246723	activin receptor type-2A-like isoform X2			
Acrdi.XM_015922735.1/1-2859 | Monca.augustus.g3797.t1/1-2862	0.271948	0.246717	BRCA1-associated ATM activator 1			
Acrdi.XM_015918956.1/1-1095 | Monca.augustus.g47739.t1/1-1113	0.274726	0.246706	neuropeptide Y receptor-like	3	P:GO:0007165; C:GO:0016021; F:GO:0004871	P:signal transduction; C:integral component of membrane; F:signal transdu [illegible]
Acrdi.XM_015902789.1/1-1788 | Monca.adi2mcaRNA37543_R3/1-1887	0.0674992	0.246694	PREDICTED: uncharacterized protein LOC107337583			
Acrdi.XM_015911119.1/1-1251 | Monca.augustus.g7986.t1/1-1236	0.194425	0.246562	RNA-binding squid isoform X1	2	F:GO:0000166; F:GO:0003676	F:nucleotide binding; F:nucleic acid binding
Acrdi.XM_015898944.1/1-765 | Monca.augustus.g37969.t1/1-768	0.142695	0.246542	PREDICTED: uncharacterized protein LOC107334063			
Acrdi.XM_015918448.1/1-612 | Monca.adi2mcaRNA29499_R2/1-546	0.215972	0.24651	histone-lysine N- H3 lysine-36 specific-like isoform X1	4	C:GO:0005634; F:GO:0008168; F:GO:0016740; P:GO:0032259	C:nucleus; F:methyltransferase activity; F:transferase activity; P:methylatio [illegible]
Acrdi.XM_015901098.1/1-804 | Monca.augustus.g15291.t1/1-807	0.206147	0.24646	Out at first	1	P:GO:0016321	P:female meiosis chromosome segregation
Acrdi.XM_015920811.1/1-900 | Monca.augustus.g11610.t1/1-879	0.246969	0.246454	PREDICTED: uncharacterized protein LOC107354345 isoform X1	2	C:GO:0016020; C:GO:0016021	C:membrane; C:integral component of membrane
Acrdi.XM_015906527.1/1-3561 | Monca.augustus.g28246.t1/1-4920	0.218403	0.246345	X-linked retinitis pigmentosa GTPase regulator			
Acrdi.XM_015902870.1/1-900 | Monca.augustus.g27608.t1/1-888	0.231887	0.246273	cilia and spindle-associated	2	C:GO:0005856; C:GO:0044422	C:cytoskeleton; C:organelle part
Acrdi.XM_015892759.1/1-1002 | Monca.adi2mcaRNA38777_R4/1-762	0.0681054	0.246264	PREDICTED: uncharacterized protein LOC107328046			
Acrdi.XM_015923290.1/1-1182 | Monca.augustus.g35012.t1/1-1218	0.325175	0.246251	sphingosine-1-phosphate phosphatase 1-like	1	C:GO:0016020	C:membrane
Acrdi.XM_015899839.1/1-393 | Monca.augustus.g52528.t1/1-417	0.310348	0.246178	PREDICTED: uncharacterized protein LOC107334875			
Acrdi.XM_015899616.1/1-465 | Monca.augustus.g4247.t1/1-960	0.183721	0.246162	adenosine receptor A1-like	2	P:GO:0007210; F:GO:0004993	P:serotonin receptor signaling pathway; F:G-protein coupled serotonin rec [illegible]
Acrdi.XM_015919136.1/1-687 | Monca.augustus.g35170.t1/1-657	0.21769	0.246129	PREDICTED: uncharacterized protein LOC107352823	2	C:GO:0016020; C:GO:0016021	C:membrane; C:integral component of membrane
Acrdi.XM_015903133.1/1-1239 | Monca.augustus.g12311.t1/1-1227	0.379783	0.246125	PREDICTED: uncharacterized protein LOC107337895			
Acrdi.XM_015923188.1/1-948 | Monca.augustus.g49869.t1/1-1401	0.128907	0.246106	Histone-lysine N-methyltransferase SUV39H2	2	C:GO:0043229; F:GO:0046872	C:intracellular organelle; F:metal ion binding
Acrdi.XM_015899066.1/1-2163 | Monca.augustus.g56391.t1/1-2139	0.239284	0.246071	zinc transporter ZIP10-like	9	C:GO:0005887; P:GO:0055085; C:GO:0016020; C:GO:0016021;	C:integral component of plasma membrane; P:transmembrane transport; [illegible]
Acrdi.XM_015899069.1/1-681 | Monca.augustus.g6445.t1/1-837	0.243384	0.246047	PREDICTED: uncharacterized protein LOC107334156 isoform X1			
Acrdi.XM_015904309.1/1-2553 | Monca.adi2mcaRNA13827_R0/1-2493	0.235547	0.246021	lutropin-choriogonadotropic hormone receptor	4	F:GO:0004930; P:GO:0007186; C:GO:0016021; C:GO:0005886	F:G-protein coupled receptor activity; P:G-protein coupled receptor signali [illegible]
Acrdi.XM_015917198.1/1-1290 | Monca.augustus.g67112.t1/1-2157	0.0881783	0.245933	ESF1 homolog	3	C:GO:0005634; C:GO:0005615; F:GO:0003723	C:nucleus; C:extracellular space; F:RNA binding
Acrdi.XM_015903365.1/1-909 | Monca.augustus.g29889.t1/1-909	0.182265	0.245923	Leucine-rich repeat-containing 3	2	C:GO:0016020; C:GO:0016021	C:membrane; C:integral component of membrane
Acrdi.XM_015917755.1/1-1323 | Monca.augustus.g1572.t1/1-1716	0.211554	0.245898	melanopsin-like isoform X1	1	C:GO:0016020	C:membrane
Acrdi.XM_015904591.1/1-765 | Monca.adi2mcaRNA14098_R3/1-717	0.0662616	0.245744	PREDICTED: uncharacterized protein LOC107339316			
Acrdi.XM_015902290.1/1-2229 | Monca.augustus.g35093.t1/1-4875	0.162241	0.245739	Sortilin-related receptor	4	C:GO:0016020; C:GO:0016021; C:GO:0005794; P:GO:0006810	C:membrane; C:integral component of membrane; C:Golgi apparatus; P:tra [illegible]
Acrdi.XM_015893322.1/1-2112 | Monca.augustus.g55122.t1/1-2112	0.253649	0.245724	Dorsal-ventral patterning tolloid 1	6	P:GO:0050982; F:GO:0005509; F:GO:0005262; C:GO:0016020;	P:detection of mechanical stimulus; F:calcium ion binding; F:calcium chan [illegible]
Acrdi.XM_015919380.1/1-531 | Monca.augustus.g62407.t1/1-687	0.101152	0.245723	synapsin-2 isoform X4			
Acrdi.XM_015923050.1/1-666 | Monca.augustus.g42211.t1/1-657	0.203073	0.24565	pre-rRNA-processing TSR2 homolog	2	P:GO:0000462; C:GO:0005634	P:maturation of SSU-rRNA from tricistronic rRNA transcript (SSU-rRNA, 5. [illegible]
Acrdi.XM_015921171.1/1-363 | Monca.augustus.g25203.t1/1-672	0.212501	0.245532	Sugar transporter SWEET1	3	C:GO:0016021; P:GO:0008643; F:GO:0051119	C:integral component of membrane; P:carbohydrate transport; F:sugar tra [illegible]
Acrdi.XM_015916383.1/1-555 | Monca.augustus.g40958.t1/1-564	0.281787	0.245464	RBPJ-interacting and tubulin-associated 1	4	F:GO:0015631; P:GO:0007219; P:GO:0051168; C:GO:0005622	F:tubulin binding; P:Notch signaling pathway; P:nuclear export; C:intracellu [illegible]
Acrdi.XM_015915277.1/1-1236 | Monca.augustus.g38343.t1/1-1233	0.396899	0.245453	keratin-associated 10-6-like			
Acrdi.XM_015921194.1/1-1560 | Monca.augustus.g51418.t1/1-1530	0.295118	0.245362	diacylglycerol O-acyltransferase MT1468	1	C:GO:0016020	C:membrane
Acrdi.XM_015898304.1/1-702 | Monca.augustus.g61848.t1/1-1257	0.238824	0.245226	PREDICTED: uncharacterized protein LOC107333482			
Acrdi.XM_015907654.1/1-2772 | Monca.augustus.g49952.t1/1-2805	0.270394	0.245213	GRB2-associated-binding 1 isoform X4			
Acrdi.XM_015911021.1/1-7971 | Monca.augustus.g3789.t1/1-9411	0.273075	0.245187	polycystic kidney disease 1-like 2	2	C:GO:0016020; C:GO:0016021	C:membrane; C:integral component of membrane
Acrdi.XM_015920965.1/1-600 | Monca.augustus.g30426.t1/1-597	0.236643	0.245161	PREDICTED: uncharacterized protein LOC107354479			
Acrdi.XM_015903814.1/1-480 | Monca.augustus.g61993.t1/1-495	0.239491	0.245149	disulfide-isomerase A3	6	F:GO:0003756; P:GO:0045454; C:GO:0005783; P:GO:0006457;	F:protein disulfide isomerase activity; P:cell redox homeostasis; C:endopla [illegible]
Acrdi.XM_015918016.1/1-825 | Monca.adi2mcaRNA29001_R0/1-792	0.226206	0.244956	PREDICTED: uncharacterized protein LOC107351718	2	C:GO:0016020; C:GO:0016021	C:membrane; C:integral component of membrane
Acrdi.XM_015920561.1/1-1122 | Monca.augustus.g38788.t1/1-1077	0.27407	0.244867	PREDICTED: uncharacterized protein LOC107354130			
Acrdi.XM_015893667.1/1-2607 | Monca.augustus.g30719.t1/1-3045	0.274726	0.244841	PREDICTED: uncharacterized protein LOC107328927			
Acrdi.XM_015903094.1/1-549 | Monca.augustus.g45205.t1/1-555	0.287098	0.244818	atonal homolog 1-like	4	P:GO:0030182; F:GO:0005488; P:GO:0007423; P:GO:0009888	P:neuron differentiation; F:binding; P:sensory organ development; P:tissue [illegible]
Acrdi.XM_015895580.1/1-3540 | Monca.augustus.g19936.t1/1-4212	0.188053	0.244797	eukaryotic translation initiation factor 5B isoform X2	4	F:GO:0005525; F:GO:0003924; C:GO:0005622; P:GO:0006412	F:GTP binding; F:GTPase activity; C:intracellular; P:translation
Acrdi.XM_015894853.1/1-2196 | Monca.augustus.g40311.t1/1-2253	0.283461	0.244787	DNA-directed primase polymerase isoform X1	3	F:GO:0003896; P:GO:0006260; P:GO:0006269	F:DNA primase activity; P:DNA replication; P:DNA replication, synthesis of [illegible]
Acrdi.XM_015914093.1/1-708 | Monca.augustus.g59124.t1/1-1260	0.272953	0.244729	collagen alpha-1(XI) chain-like isoform X2			
Acrdi.XM_015914573.1/1-2352 | Monca.adi2mcaRNA25193_R0/1-2265	0.228696	0.244715	5-hydroxytryptamine receptor 1-like	3	P:GO:0007165; C:GO:0016020; F:GO:0004871	P:signal transduction; C:membrane; F:signal transducer activity
Acrdi.XM_015903036.1/1-1767 | Monca.augustus.g56759.t1/1-1692	0.205058	0.244595	Shootin-1			
Acrdi.XM_015897965.1/1-723 | Monca.augustus.g69419.t1/1-591	0.860128	0.24451	THAP domain-containing 4			
Acrdi.XM_015913633.1/1-576 | Monca.augustus.g48686.t1/1-528	0.387672	0.244469	PREDICTED: uncharacterized protein LOC107347671 isoform X1			
Acrdi.XM_015892908.1/1-1464 | Monca.augustus.g7554.t1/1-1446	0.200633	0.244397	PREDICTED: uncharacterized protein LOC107328169			
Acrdi.XM_015904765.1/1-2346 | Monca.augustus.g31931.t1/1-2463	0.239288	0.244242	PREDICTED: uncharacterized protein LOC107339472			
Acrdi.XM_015912789.1/1-642 | Monca.augustus.g68777.t1/1-711	0.130165	0.244178	Nucleolar 12	5	P:GO:0008150; C:GO:0005634; F:GO:0003723; C:GO:0005730;	P:biological_process; C:nucleus; F:RNA binding; C:nucleolus; F:rRNA bindin [illegible]
Acrdi.XM_015897523.1/1-675 | Monca.augustus.g32450.t1/1-720	0.296106	0.24402	proline and serine-rich 1-like			
Acrdi.XM_015906793.1/1-2133 | Monca.augustus.g60648.t1/1-2151	0.286563	0.243941	beta-1,4-N-acetylgalactosaminyltransferase 3-like	4	C:GO:0032580; C:GO:0016020; C:GO:0016021; F:GO:0008376	C:Golgi cisterna membrane; C:membrane; C:integral component of memb [illegible]
Acrdi.XM_015919386.1/1-486 | Monca.augustus.g30256.t1/1-480	0.0860059	0.243914	101 kDa malaria antigen-like			

Acrdi.XM_015909059.1/1-846 | Monca.augustus.g11518.t1/1-969	0.253923	0.243848	tachykinin-like peptides receptor 86C	3	F:GO:0004930; P:GO:0007186; C:GO:0016020	F:G-protein coupled receptor activity; P:G-protein coupled receptor signaling pathway; C:membrane		
Acrdi.XM_015913129.1/1-819 | Monca.augustus.g25092.t1/1-813	0.330267	0.243826	PREDICTED: uncharacterized protein LOC107347232 isoform X1					
Acrdi.XM_015925120.1/1-1686 | Monca.augustus.g48765.t1/1-1455	0.242935	0.243645	zinc finger 768-like isoform X3	2	F:GO:0003676; F:GO:0046872	F:nucleic acid binding; F:metal ion binding		
Acrdi.XM_015903263.1/1-945 | Monca.augustus.g51694.t1/1-948	0.314747	0.243586	Aspartate beta-hydroxylase domain-containing 2	1	P:GO:0018193	P:peptidyl-amino acid modification		
Acrdi.XM_015922238.1/1-351 | Monca.adi2mcaRNA33672_R0/1-384	0.304851	0.24357	troponin skeletal muscle	1	F:GO:0005509	F:calcium ion binding		
Acrdi.XM_015913235.1/1-642 | Monca.adi2mcaRNA23664_R9/1-369	0.387672	0.243535	reverse	2	F:GO:0003964; P:GO:0006278	F:RNA-directed DNA polymerase activity; P:RNA-dependent DNA biosynthetic process		
Acrdi.XM_015919045.1/1-462 | Monca.augustus.g64652.t1/1-555	0.542064	0.24336	zinc finger MYM-type 1-like					
Acrdi.XM_015895702.1/1-708 | Monca.augustus.g47626.t1/1-684	0.257846	0.243344	BTB POZ domain-containing 6	2	C:GO:0005737; C:GO:0000932	C:cytoplasm; C:P-body		
Acrdi.XM_015919984.1/1-2166 | Monca.augustus.g60872.t1/1-2163	0.145835	0.243288	KRI1 homolog	2	C:GO:0005730; F:GO:0003723	C:nucleolus; F:RNA binding		
Acrdi.XM_015900304.1/1-756 | Monca.augustus.g23889.t1/1-774	0.279952	0.243269	PREDICTED: uncharacterized protein LOC107335300					
Acrdi.XM_015914088.1/1-2637 | Monca.adi2mcaRNA24607_R0/1-2331	0.253874	0.243236	sodium potassium calcium exchanger 5 isoform X3	4	F:GO:0046872; C:GO:0016020; P:GO:0044763; P:GO:0006810	F:metal ion binding; C:membrane; P:single-organism cellular process; P:transport		
Acrdi.XM_015919714.1/1-990 | Monca.augustus.g10300.t1/1-1497	0.29876	0.243202	galectin-3-binding isoform X2	7	C:GO:0005615; C:GO:0070062; C:GO:0072562; C:GO:0016020;	C:extracellular space; C:extracellular exosome; C:blood microparticle; C:membrane; F:scavenger receptor activity; C:extracellular matrix; P:receptor-mediated endocytosis		
Acrdi.XM_015903527.1/1-1503 | Monca.augustus.g34545.t1/1-1458	0.276609	0.243106	Proteophosphoglycan ppg4	4	C:GO:0016020; P:GO:0036066; C:GO:0016021; F:GO:0046922	C:membrane; P:protein O-linked fucosylation; C:integral component of membrane; F:peptide-O-fucosyltransferase activity		
Acrdi.XM_015920971.1/1-372 | Monca.augustus.g30428.t1/1-741	0.203075	0.243081	enoyl- delta isomerase peroxisomal-like	3	F:GO:0003824; P:GO:0008152; F:GO:0016853	F:catalytic activity; P:metabolic process; F:isomerase activity		
Acrdi.XM_015917800.1/1-2184 | Monca.augustus.g14925.t1/1-2235	0.165106	0.242934	solute carrier family 13 member 2	4	P:GO:0055085; C:GO:0016021; P:GO:0006820; F:GO:0005215	P:transmembrane transport; C:integral component of membrane; P:anion transport; F:transporter activity		
Acrdi.XM_015917457.1/1-1527 | Monca.augustus.g54586.t1/1-1500	0.256974	0.242876	serine arginine repetitive matrix 1-like					
Acrdi.XM_015923647.1/1-978 | Monca.augustus.g25166.t1/1-2799	0.332715	0.242846	Polycystic kidney disease 1-like 2	6	P:GO:0050982; F:GO:0005509; F:GO:0005262; C:GO:0016020;	P:detection of mechanical stimulus; F:calcium ion binding; F:calcium channel activity; C:membrane; C:integral component of membrane; P:calcium ion transmembrane transport		
Acrdi.XM_015895834.1/1-1113 | Monca.augustus.g63626.t1/1-2160	0.262221	0.242635	PREDICTED: uncharacterized protein LOC107331262 isoform X3					
Acrdi.XM_015920075.1/1-543 | Monca.augustus.g24405.t1/1-702	0.199874	0.242594	ame: Full=Coadhesin	4	F:GO:0070492; F:GO:0016758; C:GO:0016020; C:GO:0016021	F:oligosaccharide binding; F:transferase activity, transferring hexosyl groups; C:membrane; C:integral component of membrane		
Acrdi.XM_015892189.1/1-3363 | Monca.augustus.g33461.t1/1-4188	0.238312	0.242569	Epidermal growth factor receptor substrate 15	1	F:GO:0005509	F:calcium ion binding		
Acrdi.XM_015901336.1/1-636 | Monca.augustus.g48488.t1/1-642	0.284624	0.242503	PREDICTED: uncharacterized protein LOC107336266					
Acrdi.XM_015902379.1/1-1980 | Monca.augustus.g61655.t1/1-2022	0.230833	0.242447	G- coupled receptor 161-like isoform X1	6	F:GO:0001588; P:GO:0007218; P:GO:0001963; F:GO:0035240;	F:dopamine neurotransmitter receptor activity, coupled via Gs; P:neuropeptide signaling pathway; P:synaptic transmission, dopaminergic; F:dopamine binding; C:integral component of membrane; F:neuropeptide Y receptor activity		
Acrdi.XM_015910530.1/1-1134 | Monca.augustus.g36042.t1/1-1143	0.230204	0.2424	PREDICTED: uncharacterized protein LOC107344840					
Acrdi.XM_015896921.1/1-2862 | Monca.augustus.g7307.t1/1-3168	0.333189	0.242389	Toxin -2					
Acrdi.XM_015924636.1/1-1779 | Monca.augustus.g35399.t1/1-1767	0.28301	0.242376	probable tRNA methyltransferase 9 isoform X2	2	P:GO:0008152; F:GO:0016740	P:metabolic process; F:transferase activity		
Acrdi.XM_015908621.1/1-1299 | Monca.augustus.g22355.t1/1-1296	0.256218	0.242337	amiloride-sensitive sodium channel subunit alpha-like	7	P:GO:0006814; P:GO:0006811; F:GO:0005272; C:GO:0016020;	P:sodium ion transport; P:ion transport; F:sodium channel activity; C:membrane; C:integral component of membrane; P:sodium ion transmembrane transport; P:transport		
Acrdi.XM_015905008.1/1-1266 | Monca.adi2mcaRNA1468_R0/1-822	0.268227	0.242331	RNA pseudouridylate synthase domain-containing 2	4	P:GO:0031119; C:GO:0005739; F:GO:0009982; F:GO:0019239	P:tRNA pseudouridine synthesis; C:mitochondrion; F:pseudouridine synthase activity; F:deaminase activity	EC:5.4.99.12	tRNA pseudouridine(38-40) synthase
Acrdi.XM_015916162.1/1-609 | Monca.adi2mcaRNA252_R0/1-660	0.172513	0.242241	ketosamine-3-kinase-like isoform X2	2	P:GO:0016310; F:GO:0016301	P:phosphorylation; F:kinase activity		
[illegible]	[illegible]	[illegible]	[illegible]	[illegible]	[illegible]	[illegible]	[illegible]	[illegible]

Acrdi.XM_015902406.1/1-921 | Monca.augustus.g57285.t1/1-921	0.305343	0.228364	proline-rich transmembrane 3-	2	C:GO:0016020; C:GO:0016021	C:membrane; C:integral component of membrane
Acrdi.XM_015895351.1/1-2769 | Monca.adi2mcaRNA4252_R0/1-2796	0.215343	0.228279	PREDICTED: uncharacterized protein LOC107330787	6	C:GO:0005615; P:GO:0032504; F:GO:0003824; P:GO:0008152;	C:extracellular space; P:multicellular organism reproduction; F:catalytic activity; P:metabolic process; C:membrane; C:integral component of membrane
Acrdi.XM_015905037.1/1-975 | Monca.augustus.g60149.t1/1-939	0.303988	0.228264	chymotrypsin-like protease 1	1	F:GO:0008233	F:peptidase activity
Acrdi.XM_015899336.1/1-3639 | Monca.augustus.g64414.t1/1-3651	0.252052	0.228252	zinc finger 239-like	2	F:GO:0003676; F:GO:0046872	F:nucleic acid binding; F:metal ion binding
Acrdi.XM_015898003.1/1-537 | Monca.augustus.g40304.t1/1-537	0.713197	0.228173	PREDICTED: uncharacterized protein LOC107333227			
Acrdi.XM_015909956.1/1-3522 | Monca.adi2mcaRNA20075_R1/1-2838	0.102694	0.228156	PREDICTED: uncharacterized protein LOC107344303	2	F:GO:0003676; P:GO:0015074	F:nucleic acid binding; P:DNA integration
Acrdi.XM_015892370.1/1-402 | Monca.augustus.g58189.t1/1-1260	0.0602176	0.228073	probable prolyl 4-hydroxylase 10	5	F:GO:0031418; F:GO:0005506; F:GO:0016705; C:GO:0016021;	F:L-ascorbic acid binding; F:iron ion binding; F:oxidoreductase activity, acting on paired donors, with incorporation or reduction of molecular oxygen; C:integral component of mem[illegible]
Acrdi.XM_015918148.1/1-2256 | Monca.augustus.g30021.t1/1-3843	0.234188	0.22806	probable serine threonine- kinase pats1	1	P:GO:0009987	P:cellular process
Acrdi.XM_015913173.1/1-1074 | Monca.augustus.g39267.t1/1-1596	0.318575	0.228004	TELO2-interacting 2	2	C:GO:0016020; C:GO:0016021	C:membrane; C:integral component of membrane
Acrdi.XM_015895973.1/1-747 | Monca.augustus.g68277.t1/1-726	0.112795	0.228004	PREDICTED: uncharacterized protein LOC107331396			
Acrdi.XM_015920336.1/1-540 | Monca.augustus.g36360.t1/1-549	0.211594	0.227974	suppressor of cytokine signaling 3-like	2	P:GO:0007165; P:GO:0048523	P:signal transduction; P:negative regulation of cellular process
Acrdi.XM_015913699.1/1-834 | Monca.augustus.g21640.t1/1-663	0.312902	0.227938	CDP-diacylglycerol--inositol 3-phosphatidyltransferase	8	C:GO:0016020; C:GO:0016021; F:GO:0016780; P:GO:0008654;	C:membrane; C:integral component of membrane; F:phosphotransferase activity, for other substituted phosphate groups; P:phospholipid biosynthetic process; C:Golgi apparatus; [illegible]
Acrdi.XM_015901228.1/1-1104 | Monca.adi2mcaRNA10488_R0/1-1032	0.200502	0.227924	nudix hydrolase chloroplastic-like	1	F:GO:0044715	F:8-oxo-dGDP phosphatase activity
Acrdi.XM_015922991.1/1-540 | Monca.augustus.g67856.t1/1-540	0.245033	0.227907	PREDICTED: uncharacterized protein LOC107356374	2	C:GO:0016020; C:GO:0016021	C:membrane; C:integral component of membrane
Acrdi.XM_015922279.1/1-1626 | Monca.augustus.g58967.t1/1-1614	0.237396	0.22781	G- coupled receptor 64-like	2	P:GO:0007165; F:GO:0004871	P:signal transduction; F:signal transducer activity
Acrdi.XM_015895632.1/1-369 | Monca.augustus.g22269.t1/1-318	0.276119	0.227764	PREDICTED: uncharacterized protein LOC107331097 isoform X1			
Acrdi.XM_015914125.1/1-1293 | Monca.augustus.g25000.t1/1-1917	0.253043	0.227758	cyclic nucleotide-binding domain-containing 2-like			
Acrdi.XM_015896267.1/1-1320 | Monca.adi2mcaRNA5107_R0/1-3684	0.191746	0.227734	platelet endothelial aggregation receptor 1-	5	P:GO:0007219; P:GO:0007275; P:GO:0007154; C:GO:0016020;	P:Notch signaling pathway; P:multicellular organism development; P:cell communication; C:membrane; C:integral component of membrane
Acrdi.XM_015906137.1/1-1815 | Monca.augustus.g52593.t1/1-1839	0.243931	0.227673	GPI mannosyltransferase 4	7	P:GO:0006506; F:GO:0016757; F:GO:0000030; P:GO:0097502;	P:GPI anchor biosynthetic process; F:transferase activity, transferring glycosyl groups; F:mannosyltransferase activity; P:mannosylation; C:membrane; C:integral component of me[illegible]
Acrdi.XM_015923954.1/1-1593 | Monca.augustus.g62267.t1/1-1824	0.279127	0.227672	hepatocyte nuclear factor 1-beta isoform X2			
Acrdi.XM_015900500.1/1-1515 | Monca.augustus.g49622.t1/1-1503	0.250155	0.22767	proline-rich transmembrane 4-like	2	C:GO:0016020; C:GO:0016021	C:membrane; C:integral component of membrane
Acrdi.XM_015896613.1/1-2808 | Monca.augustus.g1583.t1/1-2775	0.295127	0.227579	Ecto-NOX disulfide-thiol exchanger	2	F:GO:0000166; F:GO:0003676	F:nucleotide binding; F:nucleic acid binding
Acrdi.XM_015899882.1/1-972 | Monca.augustus.g37402.t1/1-996	0.169663	0.227549	D(1A) dopamine receptor	9	F:GO:0004930; P:GO:0065008; P:GO:0007212; P:GO:0007399;	F:G-protein coupled receptor activity; P:regulation of biological quality; P:dopamine receptor signaling pathway; P:nervous system development; P:positive regulation of adenylate [illegible]
Acrdi.XM_015898111.1/1-7179 | Monca.adi2mcaRNA7087_R0/1-6387	0.21904	0.227465	neurogenic locus Notch -like isoform X3			
Acrdi.XM_015924078.1/1-924 | Monca.augustus.g22596.t1/1-1719	0.272678	0.227451	Ras-related Rab-38	4	F:GO:0005525; P:GO:0007264; P:GO:0034389; C:GO:0005622	F:GTP binding; P:small GTPase mediated signal transduction; P:lipid particle organization; C:intracellular
Acrdi.XM_015892126.1/1-3084 | Monca.adi2mcaRNA38028_R6/1-2778	0.0729583	0.227391	PREDICTED: uncharacterized protein LOC107327384	4	C:GO:0005634; F:GO:0046872; F:GO:0003682; P:GO:0006357	C:nucleus; F:metal ion binding; F:chromatin binding; P:regulation of transcription from RNA polymerase II promoter
Acrdi.XM_015911335.1/1-1554 | Monca.augustus.g35272.t1/1-1590	0.241627	0.227378	sphingosine kinase 1-like	2	P:GO:0016310; F:GO:0016301	P:phosphorylation; F:kinase activity
Acrdi.XM_015899264.1/1-1821 | Monca.augustus.g582.t1/1-1788	0.193329	0.227351	bromodomain-containing DDB_G0280777-like	1	P:GO:0009987	P:cellular process
Acrdi.XM_015898941.1/1-1293 | Monca.augustus.g7892.t1/1-1260	0.280152	0.227338	PREDICTED: uncharacterized protein LOC107334061			
Acrdi.XM_015913581.1/1-2562 | Monca.augustus.g14288.t1/1-2466	0.245612	0.227308	A disintegrin and metallo ase with thrombospondin motifs 6	11	P:GO:0007507; F:GO:0008270; P:GO:0035904; P:GO:0006508;	P:heart development; F:zinc ion binding; P:aorta development; P:proteolysis; P:coronary vasculature development; F:peptidase activity; C:extracellular matrix; F:metalloendopeptid[illegible]
Acrdi.XM_015897448.1/1-774 | Monca.augustus.g32446.t1/1-1014	0.236604	0.227301	Mitochondria-eating	1	P:GO:0007005	P:mitochondrion organization
Acrdi.XM_015906552.1/1-927 | Monca.adi2mcaRNA16288_R1/1-813	0.16528	0.227298	licD family	3	C:GO:0016020; C:GO:0016021; F:GO:0016740	C:membrane; C:integral component of membrane; F:transferase activity
Acrdi.XM_015921049.1/1-309 | Monca.augustus.g43452.t1/1-303	0.137956	0.227195	G2 M phase-specific E3 ubiquitin- ligase			
Acrdi.XM_015905796.1/1-3249 | Monca.adi2mcaRNA15493_R0/1-3201	0.269532	0.227172	follistatin-related 1	5	F:GO:0004867; F:GO:0005509; C:GO:0016020; C:GO:0016021;	F:serine-type endopeptidase inhibitor activity; F:calcium ion binding; C:membrane; C:integral component of membrane; P:negative regulation of serine-type endopeptidase activit[illegible]
Acrdi.XM_015917633.1/1-1128 | Monca.augustus.g44146.t1/1-1089	0.261785	0.227126	substance-K receptor-like	3	F:GO:0004930; P:GO:0007186; C:GO:0016020	F:G-protein coupled receptor activity; P:G-protein coupled receptor signaling pathway; C:membrane
Acrdi.XM_015901631.1/1-741 | Monca.augustus.g62437.t1/1-651	0.246261	0.227083	PREDICTED: uncharacterized protein LOC107336561			
Acrdi.XM_015897672.1/1-5130 | Monca.augustus.g46330.t1/1-5451	0.24563	0.227044	complement component C3 precursor	4	C:GO:0005615; F:GO:0004866; P:GO:0010951; C:GO:0005576	C:extracellular space; F:endopeptidase inhibitor activity; P:negative regula[illegible]
Acrdi.XM_015923982.1/1-930 | Monca.augustus.g54040.t1/1-4461	0.274861	0.227007	roundabout homolog 1-like isoform X2			
Acrdi.XM_015909870.1/1-3228 | Monca.augustus.g64594.t1/1-3288	0.288466	0.226901	Tumor necrosis factor alpha-induced 3	6	P:GO:0016579; F:GO:0005515; P:GO:0009968; P:GO:0045088;	P:protein deubiquitination; F:protein binding; P:negative regulation of sig[illegible]
Acrdi.XM_015910820.1/1-2133 | Monca.augustus.g8134.t1/1-2337	0.226374	0.22683	endothelin-converting enzyme 2-like isoform X2			
Acrdi.XM_015921075.1/1-1368 | Monca.augustus.g34984.t1/1-1404	0.23088	0.226694	proline-rich transmembrane 4-like	2	C:GO:0016020; C:GO:0016021	C:membrane; C:integral component of membrane
Acrdi.XM_015915204.1/1-720 | Monca.augustus.g56229.t1/1-1497	0.160871	0.226679	BTB POZ domain-containing 2-like			
Acrdi.XM_015910364.1/1-1119 | Monca.adi2mcaRNA20515_R0/1-888	0.210491	0.226674	PREDICTED: uncharacterized protein LOC107344674			
Acrdi.XM_015921025.1/1-759 | Monca.augustus.g65310.t1/1-1056	0.246482	0.226657	Tumor p63-regulated gene 1			
Acrdi.XM_015900059.1/1-702 | Monca.augustus.g32000.t1/1-2670	0.151543	0.226623	PREDICTED: uncharacterized protein LOC107335086			
Acrdi.XM_015923787.1/1-1671 | Monca.augustus.g54324.t1/1-1638	0.227192	0.226569	isoform A	10	P:GO:0007605; C:GO:0005634; F:GO:0003677; P:GO:0001752;	P:sensory perception of sound; C:nucleus; F:DNA binding; P:compound eye [illegible]
Acrdi.XM_015911426.1/1-1581 | Monca.augustus.g7440.t1/1-1542	0.259164	0.22653	mitochondrial ribonuclease P 3 isoform X1	2	C:GO:0005739; C:GO:0042645	C:mitochondrion; C:mitochondrial nucleoid
Acrdi.XM_015899486.1/1-510 | Monca.augustus.g37224.t1/1-1983	0.407937	0.226488	N-lysine methyltransferase SETD6	3	F:GO:0008168; P:GO:0050789; P:GO:0032259	F:methyltransferase activity; P:regulation of biological process; P:methylat[illegible]
Acrdi.XM_015911126.1/1-798 | Monca.adi2mcaRNA21392_R0/1-825	0.237117	0.226466	Alpha-2-macroglobulin receptor-associated	3	F:GO:0050750; C:GO:0005783; F:GO:0008201	F:low-density lipoprotein particle receptor binding; C:endoplasmic reticulu[illegible]
Acrdi.XM_015909755.1/1-444 | Monca.augustus.g2260.t1/1-441	0.361625	0.226399	PREDICTED: uncharacterized protein LOC107344141			
Acrdi.XM_015904712.1/1-2799 | Monca.augustus.g36193.t1/1-3096	0.205196	0.226398	N-acetyl-beta-glucosaminyl-glyco 4-beta-N-acetylgalactosaminyltrans	2	P:GO:0046373; F:GO:0046556	P:L-arabinose metabolic process; F:alpha-L-arabinofuranosidase activity
Acrdi.XM_015918746.1/1-1062 | Monca.augustus.g43631.t1/1-1071	0.259045	0.226397	neuromedin-U receptor 2-like	1	P:GO:0007165	P:signal transduction
Acrdi.XM_015903027.1/1-1062 | Monca.augustus.g47566.t1/1-1125	0.208509	0.226379	Kazal-like serine protease inhibitor domain-containing	2	P:GO:0006508; F:GO:0008233	P:proteolysis; F:peptidase activity
Acrdi.XM_015895507.1/1-729 | Monca.augustus.g25940.t1/1-699	0.196611	0.226352	methyltransferase type 11	1	F:GO:0016740	F:transferase activity
Acrdi.XM_015906308.1/1-441 | Monca.augustus.g66694.t1/1-444	0.178465	0.226325	PREDICTED: uncharacterized protein LOC107340910			
Acrdi.XM_015902499.1/1-1398 | Monca.augustus.g41964.t1/1-1665	0.23454	0.226294	titin-like isoform X5	7	F:GO:0000166; F:GO:0005524; F:GO:0004672; P:GO:0016310;	F:nucleotide binding; F:ATP binding; F:protein kinase activity; P:phosphoryl[illegible]
Acrdi.XM_015905502.1/1-1680 | Monca.augustus.g28273.t1/1-1881	0.2503	0.226197	E3 ubiquitin- ligase RAD18			
Acrdi.XM_015899610.1/1-1245 | Monca.augustus.g8470.t1/1-1968	0.187772	0.226189	inter-alpha-trypsin inhibitor heavy chain H4 isoform X2			
Acrdi.XM_015912395.1/1-1572 | Monca.adi2mcaRNA22752_R0/1-1596	0.216438	0.226117	nuclear receptor subfamily 2 group E member 1	37	P:GO:0021819; P:GO:0060041; P:GO:0060164; P:GO:0045665;	P:layer formation in cerebral cortex; P:retina development in camera-type [illegible]
Acrdi.XM_015920494.1/1-2394 | Monca.augustus.g52660.t1/1-2544	0.205117	0.226084	UV radiation resistance-associated gene	15	P:GO:0046718; P:GO:0006281; P:GO:0007051; P:GO:0035493;	P:viral entry into host cell; P:DNA repair; P:spindle organization; P:SNARE c[illegible]
Acrdi.XM_015921536.1/1-1356 | Monca.adi2mcaRNA32897_R0/1-1356	0.0618476	0.226035	Delta(5) fatty acid desaturase	2	P:GO:0006629; C:GO:0016021	P:lipid metabolic process; C:integral component of membrane
Acrdi.XM_015901198.1/1-444 | Monca.augustus.g58589.t1/1-975	0.166574	0.22597	PREDICTED: uncharacterized protein LOC107336135			
Acrdi.XM_015908707.1/1-582 | Monca.adi2mcaRNA18710_R0/1-633	0.230992	0.225913	peptidyl-prolyl cis-trans isomerase B-like	14	P:GO:0044829; C:GO:0005790; P:GO:0009987; P:GO:0040018;	P:positive regulation by host of viral genome replication; C:smooth endopl[illegible]
Acrdi.XM_015900354.1/1-906 | Monca.augustus.g69137.t1/1-873	0.319141	0.225853	CREG2	4	F:GO:0010181; F:GO:0016491; P:GO:0055114; C:GO:0005575	F:FMN binding; F:oxidoreductase activity; P:oxidation-reduction process; C:[illegible]
Acrdi.XM_015893146.1/1-1032 | Monca.augustus.g28694.t1/1-1509	0.299658	0.225853	B-cell receptor CD22-like			
Acrdi.XM_015907726.1/1-1500 | Monca.augustus.g10298.t1/1-1524	0.307965	0.225839	UPF0489 C5orf22 homolog isoform X1			
Acrdi.XM_015896300.1/1-1140 | Monca.augustus.g28092.t1/1-1506	0.243752	0.22579	relA-associated inhibitor-like			
Acrdi.XM_015918655.1/1-3228 | Monca.adi2mcaRNA29720_R0/1-3129	0.229693	0.225731	FERM and PDZ domain-containing 2			
Acrdi.XM_015902102.1/1-3525 | Monca.augustus.g16610.t1/1-3588	0.239702	0.225709	double-stranded RNA-specific editase Adar-like isoform X3	3	F:GO:0003723; P:GO:0006396; F:GO:0004000	F:RNA binding; P:RNA processing; F:adenosine deaminase activity
Acrdi.XM_015913567.1/1-2805 | Monca.augustus.g22347.t1/1-2880	0.240668	0.225691	dual serine threonine and tyrosine kinase-like	3	F:GO:0005524; F:GO:0004672; P:GO:0006468	F:ATP binding; F:protein kinase activity; P:protein phosphorylation
Acrdi.XM_015901313.1/1-1509 | Monca.augustus.g67932.t1/1-1425	0.18674	0.225668	hairy enhancer-of-split related with YRPW motif	1	P:GO:0009987	P:cellular process
Acrdi.XM_015913967.1/1-975 | Monca.augustus.g16600.t1/1-987	0.116628	0.225638	ribosomal RNA-processing 14-like	3	P:GO:0042273; P:GO:0042274; C:GO:0005730	P:ribosomal large subunit biogenesis; P:ribosomal small subunit biogenesi[illegible]
Acrdi.XM_015911348.1/1-1554 | Monca.augustus.g35286.t1/1-1749	0.196137	0.225582	Acid-sensing ion channel 4	7	P:GO:0006814; P:GO:0006811; F:GO:0005272; C:GO:0016020;	P:sodium ion transport; P:ion transport; F:sodium channel activity; C:memb[illegible]
Acrdi.XM_015903804.1/1-1017 | Monca.augustus.g61999.t1/1-921	0.155949	0.225519	melanocyte-stimulating hormone receptor-like	7	F:GO:0004930; F:GO:0004977; P:GO:0007186; P:GO:0007165;	F:G-protein coupled receptor activity; F:melanocortin receptor activity; P:G[illegible]
Acrdi.XM_015900225.1/1-1395 | Monca.adi2mcaRNA9380_R0/1-1419	0.216114	0.225512	Target of EGR1 1	3	F:GO:0003676; C:GO:0005634; F:GO:0046872	F:nucleic acid binding; C:nucleus; F:metal ion binding
Acrdi.XM_015901434.1/1-2562 | Monca.augustus.g6336.t1/1-2595	0.249627	0.225483	metabotropic glutamate receptor 4 isoform X1	4	F:GO:0004888; P:GO:0007186; C:GO:0016021; C:GO:0005886	F:transmembrane signaling receptor activity; P:G-protein coupled receptor [illegible]
Acrdi.XM_015912171.1/1-606 | Monca.augustus.g6357.t1/1-753	0.26335	0.225436	ribosomal RNA processing 36 homolog	3	P:GO:0000462; C:GO:0005730; C:GO:0030686	P:maturation of SSU-rRNA from tricistronic rRNA transcript (SSU-rRNA, 5.[illegible]
Acrdi.XM_015904752.1/1-753 | Monca.augustus.g51676.t1/1-801	0.30202	0.225395	PREDICTED: uncharacterized protein LOC107339465 isoform X1			
Acrdi.XM_015899677.1/1-1071 | Monca.augustus.g43434.t1/1-1074	0.418574	0.225377	bone morphogenetic 2-like	8	C:GO:0005615; P:GO:0043408; F:GO:0005160; P:GO:0060395;	C:extracellular space; P:regulation of MAPK cascade; F:transforming growt[illegible]
Acrdi.XM_015914370.1/1-795 | Monca.augustus.g50295.t1/1-939	0.768117	0.225333	glutamic acid-rich -like	2	C:GO:0016020; C:GO:0016021	C:membrane; C:integral component of membrane
Acrdi.XM_015908087.1/1-798 | Monca.augustus.g33217.t1/1-786	0.372092	0.225279	Translin-associated X	1	C:GO:0005634	C:nucleus
Acrdi.XM_015902082.1/1-4623 | Monca.adi2mcaRNA11437_R1/1-4068	0.174013	0.22527	lipoxygenase homology domain-containing 1-like	6	P:GO:0007605; P:GO:0050982; F:GO:0005262; C:GO:0016020;	P:sensory perception of sound; P:detection of mechanical stimulus; F:calciu[illegible]
Acrdi.XM_015921259.1/1-369 | Monca.augustus.g42831.t1/1-504	0.211749	0.225276	PREDICTED: uncharacterized protein LOC107354767			
Acrdi.XM_015903924.1/1-648 | Monca.adi2mcaRNA6260_R8/1-357	0.0626138	0.225265	Zinc finger MYM-type 3	4	P:GO:0007165; C:GO:0005739; P:GO:0051607; F:GO:0004871	P:signal transduction; C:mitochondrion; P:defense response to virus; F:sign[illegible]
Acrdi.XM_015911791.1/1-1080 | Monca.augustus.g14130.t1/1-1035	0.185851	0.225264	somatostatin receptor type 1-like	9	F:GO:0004930; F:GO:0001588; P:GO:0001963; P:GO:0007186;	F:G-protein coupled receptor activity; F:dopamine neurotransmitter recept[illegible]
Acrdi.XM_015897045.1/1-378 | Monca.augustus.g48376.t1/1-495	0.209598	0.225217	translocator -like isoform X2	6	F:GO:0051033; C:GO:0043231; C:GO:0016020; C:GO:0016021;	F:RNA transmembrane transporter activity; C:intracellular membrane-bou[illegible]
Acrdi.XM_015915293.1/1-1329 | Monca.augustus.g46143.t1/1-1569	0.281585	0.225138	DEP domain-containing 7-like	2	P:GO:0035556; C:GO:0005622	P:intracellular signal transduction; C:intracellular
Acrdi.XM_015892752.1/1-981 | Monca.augustus.g33466.t1/1-984	0.291868	0.225103	Centromere P	4	C:GO:0005634; C:GO:0005730; C:GO:0000775; P:GO:0034080	C:nucleus; C:nucleolus; C:chromosome, centromeric region; P:CENP-A conta[illegible]
Acrdi.XM_015915493.1/1-1098 | Monca.augustus.g32777.t1/1-1119	0.221412	0.225066	Neuropeptide FF receptor 2	5	P:GO:0010033; P:GO:0007165; P:GO:0009719; C:GO:0016021;	P:response to organic substance; P:signal transduction; P:response to endo[illegible]
Acrdi.XM_015916972.1/1-1482 | Monca.augustus.g35984.t1/1-1866	0.224008	0.225052	cleft lip and palate transmembrane 1	3	C:GO:0005887; F:GO:0005515; P:GO:0007275	C:integral component of plasma membrane; F:protein binding; P:multicellu[illegible]
Acrdi.XM_015913256.1/1-3111 | Monca.augustus.g67370.t1/1-2334	0.252453	0.22505	endothelin-converting enzyme 2-like isoform X3	4	P:GO:0006508; C:GO:0016020; C:GO:0016021; F:GO:0004222	P:proteolysis; C:membrane; C:integral component of membrane; F:metallo[illegible]
Acrdi.XM_015892274.1/1-2781 | Monca.augustus.g385.t1/1-2853	0.235673	0.225002	histone H2A deubiquitinase MYSM1 isoform X4	9	F:GO:0004843; C:GO:0005634; F:GO:0006338; F:GO:0003713;	F:thiol-dependent ubiquitin-specific protease activity; C:nucleus; P:chroma[illegible]
Acrdi.XM_015898757.1/1-1524 | Monca.augustus.g55637.t1/1-1503	0.295038	0.224948	major facilitator superfamily domain-containing 6-like	2	C:GO:0016020; C:GO:0016021	C:membrane; C:integral component of membrane
Acrdi.XM_015915601.1/1-477 | Monca.augustus.g5422.t1/1-1968	0.252028	0.224938	Ankyrin repeat and SOCS box 7			
Acrdi.XM_015900051.1/1-1044 | Monca.augustus.g43402.t1/1-999	0.221803	0.224879	skeletal aspartic acid-rich 2-like	2	C:GO:0016020; C:GO:0016021	C:membrane; C:integral component of membrane
Acrdi.XM_015898253.1/1-1023 | Monca.augustus.g45299.t1/1-1017	0.272325	0.224865	histamine H2 receptor-like	3	F:GO:0004930; P:GO:0007154; P:GO:0044700	F:G-protein coupled receptor activity; P:cell communication; P:single orga[illegible]
Acrdi.XM_015903623.1/1-2094 | Monca.adi2mcaRNA13043_R0/1-1749	0.178389	0.224808	fidgetin 1	2	F:GO:0000166; F:GO:0005524	F:nucleotide binding; F:ATP binding
Acrdi.XM_015917422.1/1-441 | Monca.augustus.g2710.t1/1-345	0.189449	0.224681	Retrovirus-related Pol poly from transposon			
Acrdi.XM_015917828.1/1-1104 | Monca.augustus.g7373.t1/1-1329	0.17351	0.224655	BTN1-like	6	P:GO:0051453; P:GO:0015809; P:GO:0007033; P:GO:0007034;	P:regulation of intracellular pH; P:arginine transport; P:vacuole organizatio[illegible]
Acrdi.XM_015904808.1/1-834 | Monca.augustus.g42622.t1/1-300	0.180254	0.224654	RPA-interacting A-like			
Acrdi.XM_015909801.1/1-1866 | Monca.augustus.g46893.t1/1-5781	0.227271	0.224587	deleted in malignant brain tumors 1 -	3	C:GO:0016020; F:GO:0005044; P:GO:0006898	C:membrane; F:scavenger receptor activity; P:receptor-mediated endocyto[illegible]
Acrdi.XM_015903989.1/1-837 | Monca.augustus.g27768.t1/1-885	0.146593	0.224578	D(2) dopamine receptor A-like	2	P:GO:0007186; C:GO:0016020	P:G-protein coupled receptor signaling pathway; C:membrane
Acrdi.XM_015916050.1/1-5235 | Monca.adi2mcaRNA26825_R0/1-4674	0.271589	0.224491	RRP5 homolog	1	C:GO:0044424	C:intracellular part
Acrdi.XM_015921776.1/1-1053 | Monca.augustus.g70569.t1/1-918	0.201522	0.224488	carbohydrate sulfotransferase 3-like	5	P:GO:0006790; C:GO:0016020; C:GO:0016021; F:GO:0001517;	P:sulfur compound metabolic process; C:membrane; C:integral component [illegible]
Acrdi.XM_015919133.1/1-3090 | Monca.augustus.g35165.t1/1-3111	0.196507	0.224475	Centromere F	7	C:GO:0044428; C:GO:0015630; F:GO:0005515; C:GO:0044430;	C:nuclear part; C:microtubule cytoskeleton; F:protein binding; C:cytoskeleta[illegible]
Acrdi.XM_015907293.1/1-9054 | Monca.adi2mcaRNA1712_R1/1-7035	0.179491	0.22447	cadherin EGF LAG seven-pass G-type receptor 1-like	12	F:GO:0004930; F:GO:0004888; P:GO:0007186; P:GO:0007165;	F:G-protein coupled receptor activity; F:transmembrane signaling receptor [illegible]
Acrdi.XM_015923849.1/1-2445 | Monca.augustus.g60452.t1/1-3897	0.287196	0.224452	AT-rich interactive domain-containing 5B isoform X2	5	C:GO:0005634; F:GO:0044212; P:GO:0048513; P:GO:0009987;	C:nucleus; F:transcription regulatory region DNA binding; P:animal organ d[illegible]
Acrdi.XM_015898904.1/1-3993 | Monca.augustus.g7895.t1/1-3852	0.243447	0.224437	Lysosomal-trafficking regulator			
Acrdi.XM_015905065.1/1-2280 | Monca.augustus.g24497.t1/1-3309	0.185374	0.224376	EGF-like repeat and discoidin I-like domain-containing 3	1	F:GO:0070492	F:oligosaccharide binding
Acrdi.XM_015907988.1/1-2328 | Monca.augustus.g13015.t1/1-2187	0.252436	0.22419	neurogenic locus notch homolog 2-like	3	F:GO:0005509; C:GO:0016020; C:GO:0016021	F:calcium ion binding; C:membrane; C:integral component of membrane
Acrdi.XM_015899894.1/1-1809 | Monca.augustus.g32798.t1/1-1683	0.250591	0.224107	Rho GTPase-activating 8			
Acrdi.XM_015897401.1/1-2112 | Monca.augustus.g51663.t1/1-2058	0.249535	0.224021	ankyrin repeat			
Acrdi.XM_015909372.1/1-534 | Monca.augustus.g27154.t1/1-1107	0.220496	0.224018	NF-kappa-B inhibitor 1			
Acrdi.XM_015920990.1/1-1827 | Monca.augustus.g42001.t1/1-2139	0.236531	0.224007	PREDICTED: uncharacterized protein LOC107354506			
Acrdi.XM_015900004.1/1-1320 | Monca.augustus.g16047.t1/1-1164	0.164034	0.223993	carbohydrate sulfotransferase 3-like	3	C:GO:0016020; P:GO:0008152; F:GO:0008146	C:membrane; P:metabolic process; F:sulfotransferase activity
Acrdi.XM_015905450.1/1-1128 | Monca.augustus.g68940.t1/1-1251	0.203916	0.22396	Hyaluronidase-1	6	P:GO:0050794; F:GO:0003824; P:GO:0051716; P:GO:0042221;	P:regulation of cellular process; F:catalytic activity; P:cellular response to s[illegible]
Acrdi.XM_015910840.1/1-708 | Monca.augustus.g57297.t1/1-1260	0.27072	0.22394	serine arginine repetitive matrix 1-like			
Acrdi.XM_015923010.1/1-9345 | Monca.augustus.g46070.t1/1-9480	0.286151	0.223931	polycystic kidney disease 1-like 2	1	C:GO:0016020	C:membrane
Acrdi.XM_015897127.1/1-906 | Monca.augustus.g55692.t1/1-897	0.198295	0.223829	keratin-associated 10-4-like			
Acrdi.XM_015916652.1/1-1293 | Monca.augustus.g67116.t1/1-1665	0.225657	0.223828	peptide-N(4)-(N-acetyl-beta-glucosaminyl)asparagine amidase isoforr	2	P:GO:0006516; C:GO:0005737	P:glycoprotein catabolic process; C:cytoplasm
Acrdi.XM_015899699.1/1-1071 | Monca.augustus.g33790.t1/1-1074	0.227148	0.223824	beta-1,3-galactosyltransferase 2	2	C:GO:0016020; F:GO:0016740	C:membrane; F:transferase activity
Acrdi.XM_015898453.1/1-5406 | Monca.adi2mcaRNA7512_R0/1-3717	0.194346	0.223817	sodium channel type 2 subunit alpha-like isoform X2	2	F:GO:0005216; P:GO:0006811	F:ion channel activity; P:ion transport
Acrdi.XM_015920367.1/1-4287 | Monca.adi2mcaRNA31660_R0/1-4155	0.237709	0.223777	deleted in malignant brain tumors 1	3	C:GO:0016020; F:GO:0005044; P:GO:0006898	C:membrane; F:scavenger receptor activity; P:receptor-mediated endocyto[illegible]
Acrdi.XM_015897222.1/1-1707 | Monca.augustus.g63821.t1/1-1593	0.16997	0.223694	Transmembrane prolyl 4-hydroxylase			
Acrdi.XM_015897170.1/1-1578 | Monca.augustus.g31732.t1/1-2094	0.212527	0.223617	diphthine--ammonia ligase	3	F:GO:0005524; P:GO:0017183; F:GO:0017178	F:ATP binding; P:peptidyl-diphthamide biosynthetic process from peptidyl-[illegible]
Acrdi.XM_015907659.1/1-1314 | Monca.augustus.g49944.t1/1-1320	0.236609	0.223592	monocarboxylate transporter 10-like	7	P:GO:0055085; P:GO:0034220; C:GO:0016020; P:GO:0015718;	P:transmembrane transport; P:ion transmembrane transport; C:membrane[illegible]
Acrdi.XM_015898687.1/1-840 | Monca.augustus.g2549.t1/1-765	0.27526	0.223541	OTU domain-containing 3-like			
Acrdi.XM_015910310.1/1-8952 | Monca.adi2mcaRNA20473_R0/1-4614	0.251267	0.223416	probable E3 ubiquitin- ligase HERC6	7	F:GO:0005525; F:GO:0042803; P:GO:0007165; P:GO:0007264;	F:GTP binding; F:protein homodimerization activity; P:signal transduction; [illegible]
Acrdi.XM_015924630.1/1-1911 | Monca.augustus.g28493.t1/1-1377	0.134921	0.223371	chromosome-associated kinesin KIF4A	6	F:GO:0005524; C:GO:0005871; F:GO:0008574; P:GO:0007018;	F:ATP binding; C:kinesin complex; F:ATP-dependent microtubule motor act[illegible]
Acrdi.XM_015913541.1/1-2649 | Monca.augustus.g22346.t1/1-2778	0.265025	0.223339	dual serine threonine and tyrosine kinase-like	1	F:GO:0003924	F:GTPase activity
Acrdi.XM_015899053.1/1-4575 | Monca.augustus.g43405.t1/1-5508	0.28251	0.223281	roundabout homolog 2-like	2	C:GO:0016020; C:GO:0016021	C:membrane; C:integral component of membrane
Acrdi.XM_015900803.1/1-402 | Monca.augustus.g58764.t1/1-396	0.262638	0.223275	prominin-1-A-like isoform X1	2	C:GO:0016020; C:GO:0016021	C:membrane; C:integral component of membrane
Acrdi.XM_015910632.1/1-4923 | Monca.augustus.g31888.t1/1-6705	0.182198	0.223269	Sterile alpha motif domain-containing 9-like	1	F:GO:0005524	F:ATP binding
Acrdi.XM_015892077.1/1-1008 | Monca.augustus.g68877.t1/1-1038	0.220605	0.22322	probable G- coupled receptor 19	2	P:GO:0051716; F:GO:0004871	P:cellular response to stimulus; F:signal transducer activity
Acrdi.XM_015898758.1/1-894 | Monca.augustus.g18748.t1/1-2013	0.215042	0.223213	thyrotropin-releasing hormone receptor-like	3	P:GO:0007154; P:GO:0044700; F:GO:0004871	P:cell communication; P:single organism signaling; F:signal transducer acti[illegible]
Acrdi.XM_015919772.1/1-1797 | Monca.augustus.g23535.t1/1-2109	0.314501	0.223183	eukaryotic translation initiation factor 3 subunit A-like			
Acrdi.XM_015905370.1/1-516 | Monca.augustus.g41367.t1/1-459	0.533628	0.223089	PREDICTED: uncharacterized protein LOC107340020			
Acrdi.XM_015892600.1/1-498 | Monca.adi2mcaRNA38567_R3/1-369	1.10701	0.223082	PREDICTED: uncharacterized protein LOC107327873			
Acrdi.XM_015891799.1/1-915 | Monca.augustus.g21478.t1/1-921	0.175243	0.223005	exonuclease 3 -5 domain-containing 1-like	4	F:GO:0003676; P:GO:0090305; F:GO:0008408; P:GO:0006139	F:nucleic acid binding; P:nucleic acid phosphodiester bond hydrolysis; F:3'-5[illegible]
Acrdi.XM_015892076.1/1-1101 | Monca.augustus.g38767.t1/1-1002	0.220452	0.222992	neuropeptide FF receptor 2	1	P:GO:0051716	P:cellular response to stimulus
Acrdi.XM_015899874.1/1-810 | Monca.augustus.g48618.t1/1-891	0.344949	0.222982	cyclic AMP-dependent transcription factor ATF-4-like	4	C:GO:0005634; F:GO:0000977; F:GO:0000981; P:GO:0006357	C:nucleus; F:RNA polymerase II regulatory region sequence-specific DNA b[illegible]
Acrdi.XM_015908315.1/1-2106 | Monca.augustus.g36823.t1/1-2130	0.219754	0.222978	PC-esterase domain-containing 1A-like	1	F:GO:0016788	F:hydrolase activity, acting on ester bonds
Acrdi.XM_015897131.1/1-846 | Monca.augustus.g55699.t1/1-828	0.335021	0.222965	collagen alpha-1(XII) chain-like			
Acrdi.XM_015909202.1/1-582 | Monca.adi2mcaRNA19252_R2/1-366	0.537562	0.222959	calmodulin isoform X1	70	F:GO:0043548; F:GO:0015276; P:GO:0009792; P:GO:0043388;	F:phosphatidylinositol 3-kinase binding; F:ligand-gated ion channel activity[illegible]
Acrdi.XM_015916390.1/1-441 | Monca.augustus.g52608.t1/1-1299	0.208671	0.222855	amnionless	5	C:GO:0045177; P:GO:0008104; C:GO:0044425; P:GO:0006810;	C:apical part of cell; P:protein localization; C:membrane part; P:transport; [illegible]
Acrdi.XM_015920918.1/1-1047 | Monca.augustus.g46853.t1/1-975	0.177854	0.222841	melatonin receptor type 1B-like	3	P:GO:0007165; C:GO:0016020; F:GO:0004871	P:signal transduction; C:membrane; F:signal transducer activity
Acrdi.XM_015916051.1/1-984 | Monca.augustus.g65538.t1/1-984	0.163502	0.222783	trace amine-associated receptor 5-like	3	P:GO:0007154; P:GO:0044700; F:GO:0004871	P:cell communication; P:single organism signaling; F:signal transducer acti[illegible]
Acrdi.XM_015897115.1/1-1896 | Monca.augustus.g55678.t1/1-1746	0.279826	0.222754	PREDICTED: uncharacterized protein LOC107332382			
Acrdi.XM_015912662.1/1-3543 | Monca.adi2mcaRNA23039_R0/1-4290	0.180384	0.222752	cysteine-rich secretory LCCL domain-containing 2-like	1	C:GO:0005576	C:extracellular region
Acrdi.XM_015908747.1/1-366 | Monca.augustus.g55163.t1/1-744	0.0531335	0.222742	homolog subfamily C member 8			
Acrdi.XM_015915867.1/1-1488 | Monca.augustus.g61002.t1/1-1491	0.29547	0.222681	trefoil factor	7	P:GO:0016539; P:GO:0007275; C:GO:0016020; P:GO:0006508;	P:intein-mediated protein splicing; P:multicellular organism development; [illegible]
Acrdi.XM_015908915.1/1-732 | Monca.augustus.g31882.t1/1-2559	0.214373	0.222658	calcitonin gene-related peptide type 1 receptor-like	9	F:GO:0004930; F:GO:0004888; P:GO:0007186; P:GO:0007165;	F:G-protein coupled receptor activity; F:transmembrane signaling receptor [illegible]
Acrdi.XM_015912723.1/1-1374 | Monca.augustus.g43039.t1/1-1446	0.182896	0.222547	caspase-9 isoform X1	1	F:GO:0008234	F:cysteine-type peptidase activity
Acrdi.XM_015920731.1/1-6090 | Monca.augustus.g7344.t1/1-7827	0.200944	0.222534	macrophage mannose receptor 1-like	3	C:GO:0016020; C:GO:0016021; F:GO:0030246	C:membrane; C:integral component of membrane; F:carbohydrate binding
Acrdi.XM_015923055.1/1-432 | Monca.augustus.g42209.t1/1-552	0.230424	0.222466	Transcription factor IIIA	1	F:GO:0003676	F:nucleic acid binding
Acrdi.XM_015895515.1/1-1014 | Monca.augustus.g25958.t1/1-1008	0.20271	0.222463	ame: Full=Galaxin Flags: Precursor	1	C:GO:0005576	C:extracellular region
Acrdi.XM_015893280.1/1-1422 | Monca.augustus.g67438.t1/1-1581	0.178317	0.222414	lysosome membrane 2-like	4	C:GO:0016020; C:GO:0016021; F:GO:0004872; C:GO:0005764	C:membrane; C:integral component of membrane; F:receptor activity; C:ly[illegible]
Acrdi.XM_015922831.1/1-843 | Monca.augustus.g31210.t1/1-852	0.455976	0.222388	PREDICTED: uncharacterized protein C8orf74-like			
Acrdi.XM_015913780.1/1-711 | Monca.augustus.g21666.t1/1-696	0.298601	0.222372	TSSC4			
Acrdi.XM_015916510.1/1-873 | Monca.augustus.g29760.t1/1-705	0.189834	0.222351	zinc finger NFX1-type containing	4	C:GO:0005634; F:GO:0003700; F:GO:0008270; P:GO:0006355	C:nucleus; F:transcription factor activity, sequence-specific DNA binding; F:[illegible]
Acrdi.XM_015915382.1/1-3528 | Monca.adi2mcaRNA26067_R0/1-3414	0.238144	0.22234	tyrosine- phosphatase non-receptor	2	F:GO:0004721; P:GO:0006470	F:phosphoprotein phosphatase activity; P:protein dephosphorylation
Acrdi.XM_015910914.1/1-1251 | Monca.augustus.g41342.t1/1-1251	0.228482	0.22282	caspase 3	2	F:GO:0097153; P:GO:0009987	F:cysteine-type endopeptidase activity involved in apoptotic process; P:cell[illegible]
Acrdi.XM_015897366.1/1-1614 | Monca.augustus.g29312.t1/1-1662	0.175949	0.222272	probable E3 ubiquitin- ligase MGRN1 isoform X3	2	F:GO:0008270; F:GO:0046872	F:zinc ion binding; F:metal ion binding
Acrdi.XM_015921700.1/1-987 | Monca.augustus.g43108.t1/1-972	0.272306	0.22214	prolactin-releasing peptide receptor-like	3	P:GO:0007165; C:GO:0016020; F:GO:0004871	P:signal transduction; C:membrane; F:signal transducer activity
Acrdi.XM_015925090.1/1-2166 | Monca.augustus.g4272.t1/1-2361	0.248924	0.222107	PREDICTED: uncharacterized protein LOC107358490	1	P:GO:0060828	P:regulation of canonical Wnt signaling pathway
Acrdi.XM_015910566.1/1-489 | Monca.augustus.g31952.t1/1-429	0.275663	0.222106	PREDICTED: uncharacterized protein LOC107344877			
Acrdi.XM_015914503.1/1-315 | Monca.augustus.g60712.t1/1-564	0.150829	0.22202	calcyphosin	2	F:GO:0016491; F:GO:0005509	F:oxidoreductase activity; F:calcium ion binding
Acrdi.XM_015907682.1/1-360 | Monca.augustus.g3489.t1/1-1023	0.337291	0.221981	Electron transfer flavo subunit mitochondrial	11	F:GO:0003995; P:GO:0031998; P:GO:0033539; P:GO:0070584;	F:acyl-CoA dehydrogenase activity; P:regulation of fatty acid beta-oxidation[illegible]
Acrdi.XM_015895959.1/1-2067 | Monca.augustus.g22582.t1/1-2121	0.241178	0.221972	neurexin-3-like isoform X2			
Acrdi.XM_015922189.1/1-1077 | Monca.augustus.g43195.t1/1-1104	0.231778	0.221883	PREDICTED: uncharacterized protein LOC107355600			
Acrdi.XM_015895471.1/1-942 | Monca.augustus.g18322.t1/1-477	0.353551	0.221765	ZP domain-containing -like	6	F:GO:0030414; F:GO:0005509; C:GO:0016020; C:GO:0016021;	F:peptidase inhibitor activity; F:calcium ion binding; C:membrane; C:integr[illegible]
Acrdi.XM_015914842.1/1-2436 | Monca.adi2mcaRNA25459_R0/1-2238	0.1883	0.221742	Golgi apparatus 1	3	C:GO:0016020; C:GO:0016021; C:GO:0000139	C:membrane; C:integral component of membrane; C:Golgi membrane
Acrdi.XM_015911039.1/1-1659 | Monca.augustus.g3794.t1/1-1764	0.243438	0.221731	PREDICTED: uncharacterized protein LOC107345336			
Acrdi.XM_015891618.1/1-552 | Monca.augustus.g34416.t1/1-552	0.185018	0.221729	PREDICTED: uncharacterized protein LOC107326866			
Acrdi.XM_015916712.1/1-1155 | Monca.augustus.g37097.t1/1-2772	0.28262	0.2217	Smith-Magenis syndrome chromosomal region candidate gene 8 -like isoform X1			
Acrdi.XM_015906475.1/1-1554 | Monca.augustus.g22538.t1/1-1683	0.255498	0.221672	Steryl-sulfatase	3	F:GO:0003824; F:GO:0008484; P:GO:0008152	F:catalytic activity; F:sulfuric ester hydrolase activity; P:metabolic process
Acrdi.XM_015900476.1/1-1209 | Monca.augustus.g68175.t1/1-1347	0.146728	0.221663	stAR-related lipid transfer 3	8	P:GO:0006701; C:GO:0005768; F:GO:0015485; C:GO:0005739;	P:progesterone biosynthetic process; C:endosome; F:cholesterol binding; C:[illegible]
Acrdi.XM_015908980.1/1-900 | Monca.augustus.g47247.t1/1-894	0.233605	0.221536	X prolyl aminopeptidase	4	F:GO:0046872; P:GO:0006508; F:GO:0004177; F:GO:0016787	F:metal ion binding; P:proteolysis; F:aminopeptidase activity; F:hydrolase a[illegible]
Acrdi.XM_015904099.1/1-1071 | Monca.augustus.g44553.t1/1-1878	0.263246	0.221456	PREDICTED: uncharacterized protein LOC107338846			
Acrdi.XM_015919608.1/1-891 | Monca.augustus.g65333.t1/1-1125	0.281861	0.22142	Neural proliferation differentiation and control 1	1	C:GO:0016021	C:integral component of membrane
Acrdi.XM_015901165.1/1-1533 | Monca.adi2mcaRNA10415_R0/1-1455	0.22508	0.221329	RRNAD1 isoform X1	1	P:GO:0044699	P:single-organism process
Acrdi.XM_015897652.1/1-1242 | Monca.augustus.g56105.t1/1-2889	0.171189	0.221271	Transcriptional SWT1			
Acrdi.XM_015897026.1/1-696 | Monca.augustus.g48644.t1/1-714	0.168228	0.221214	G patch domain-containing 4			
Acrdi.XM_015909401.1/1-984 | Monca.augustus.g45721.t1/1-981	0.206014	0.221194	carbohydrate sulfotransferase 11-like	3	P:GO:0016051; C:GO:0016021; F:GO:0008146	P:carbohydrate biosynthetic process; C:integral component of membrane; [illegible]
Acrdi.XM_015897202.1/1-456 | Monca.adi2mcaRNA6060_R0/1-420	0.361017	0.221171	transmembrane 56-like	2	C:GO:0016020; C:GO:0016021	C:membrane; C:integral component of membrane
Acrdi.XM_015895527.1/1-723 | Monca.augustus.g25982.t1/1-1173	0.245158	0.221141	Interferon-induced helicase C domain-containing 1	6	F:GO:0003677; F:GO:0005524; P:GO:0007165; F:GO:0016817;	F:DNA binding; F:ATP binding; P:signal transduction; F:hydrolase activity, a[illegible]
Acrdi.XM_015913603.1/1-1971 | Monca.augustus.g26387.t1/1-5124	0.260154	0.221075	vitellogenin-like isoform X1	2	P:GO:0006869; F:GO:0005319	P:lipid transport; F:lipid transporter activity
Acrdi.XM_015902540.1/1-5742 | Monca.adi2mcaRNA11941_R0/1-5574	0.255103	0.221037	LIM domain only 7 isoform X9	4	F:GO:0008270; P:GO:0023051; F:GO:0046872; P:GO:0030155	F:zinc ion binding; P:regulation of signaling; F:metal ion binding; P:regulati[illegible]
Acrdi.XM_015900141.1/1-1629 | Monca.augustus.g23824.t1/1-1374	0.237617	0.221031	DDB1- and CUL4-associated factor 8 isoform X1			
Acrdi.XM_015905183.1/1-468 | Monca.adi2mcaRNA14852_R0/1-564	0.230361	0.220983	lysosomal acid phosphatase	4	C:GO:0016020; C:GO:0016021; P:GO:0016311; F:GO:0003993	C:membrane; C:integral component of membrane; P:dephosphorylation; F:[illegible]
Acrdi.XM_015901585.1/1-2445 | Monca.augustus.g55975.t1/1-2397	0.223728	0.220976	apextrin 1	2	C:GO:0016020; C:GO:0016021	C:membrane; C:integral component of membrane
Acrdi.XM_015902252.1/1-1050 | Monca.augustus.g35078.t1/1-1242	0.290014	0.22093	Golgi-associated plant pathogenesis-related 1			
Acrdi.XM_015901830.1/1-4020 | Monca.augustus.g56977.t1/1-5190	0.222735	0.220911	E3 ubiquitin- ligase SHPRH	2	F:GO:0005524; F:GO:0008270	F:ATP binding; F:zinc ion binding
Acrdi.XM_015918550.1/1-4653 | Monca.augustus.g2344.t1/1-4650	0.22527	0.220899	golgin subfamily B member 1-like			
Acrdi.XM_015892176.1/1-6390 | Monca.adi2mcaRNA3875_R0/1-6549	0.219743	0.220897	PREDICTED: trichohyalin-like			
Acrdi.XM_015922528.1/1-339 | Monca.augustus.g5719.t1/1-432	0.23659	0.220826	membrane FAM174-like	2	C:GO:0016020; C:GO:0016021	C:membrane; C:integral component of membrane
Acrdi.XM_015901850.1/1-5811 | Monca.adi2mcaRNA11233_R0/1-5874	0.287819	0.220803	Calmodulin-regulated spectrin-associated 1	1	F:GO:0005515	F:protein binding
Acrdi.XM_015920746.1/1-942 | Monca.adi2mcaRNA32069_R0/1-894	0.254504	0.2208	Gamma-tubulin complex component 5	3	C:GO:0005815; P:GO:0022402; P:GO:0000226	C:microtubule organizing center; P:cell cycle process; P:microtubule cytosk[illegible]
Acrdi.XM_015907065.1/1-2403 | Monca.augustus.g43276.t1/1-2430	0.245488	0.220788	Disintegrin and metallo ase domain-containing 12			
Acrdi.XM_015897973.1/1-1869 | Monca.augustus.g28131.t1/1-1776	0.280632	0.220758	adhesive plaque matrix 2-like	1	F:GO:0005509	F:calcium ion binding
Acrdi.XM_015899509.1/1-846 | Monca.augustus.g17227.t1/1-819	0.322304	0.220713	FAM92A1 isoform X2			
Acrdi.XM_015892479.1/1-699 | Monca.adi2mcaRNA38416_R3/1-303	0.294173	0.220685	PREDICTED: uncharacterized protein LOC107327734			
Acrdi.XM_015903974.1/1-831 | Monca.augustus.g27726.t1/1-858	0.147537	0.220679	MAK16 homolog			
Acrdi.XM_015912214.1/1-1632 | Monca.augustus.g22426.t1/1-1602	0.275184	0.220669	diacylglycerol O-acyltransferase MT1468	4	F:GO:0047196; P:GO:0019432; F:GO:0004144; C:GO:0005886	F:long-chain-alcohol O-fatty-acyltransferase activity; P:triglyceride biosynt[illegible]
Acrdi.XM_015919276.1/1-597 | Monca.augustus.g60230.t1/1-567	0.265783	0.220645	PREDICTED: uncharacterized protein LOC107352955 isoform X1			
Acrdi.XM_015912757.1/1-1647 | Monca.augustus.g9223.t1/1-1770	0.272972	0.220642	ankyrin repeat and SAM domain-containing 3			
Acrdi.XM_015916101.1/1-1041 | Monca.adi2mcaRNA26857_R0/1-1176	0.0666138	0.220639	nuclease HARBI1			
Acrdi.XM_015913498.1/1-579 | Monca.augustus.g20506.t1/1-651	0.192273	0.220598	ADP-ribosylation factor 6-interacting 4			
Acrdi.XM_015915128.1/1-1140 | Monca.augustus.g26559.t1/1-1143	0.143334	0.220579	myosin heavy striated muscle-like			
Acrdi.XM_015924068.1/1-519 | Monca.augustus.g46919.t1/1-513	0.277682	0.220569	PREDICTED: uncharacterized protein LOC107357427			
Acrdi.XM_015914621.1/1-894 | Monca.adi2mcaRNA25199_R0/1-879	0.273586	0.220531	adhesion G -coupled receptor L4-like			
Acrdi.XM_015902031.1/1-1041 | Monca.augustus.g69324.t1/1-1068	0.194105	0.2205	PREDICTED: uncharacterized protein LOC107336936			
Acrdi.XM_015920140.1/1-1503 | Monca.augustus.g27080.t1/1-1521	0.190526	0.22045	PREDICTED: uncharacterized protein LOC107353759	5	F:GO:0031418; F:GO:0005506; F:GO:0016491; F:GO:0016705;	F:L-ascorbic acid binding; F:iron ion binding; F:oxidoreductase activity; F:o[illegible]
Acrdi.XM_015924749.1/1-909 | Monca.augustus.g41968.t1/1-891	0.207096	0.220422	melanocyte-stimulating hormone receptor-like	8	F:GO:0004930; F:GO:0004977; P:GO:0007186; P:GO:0007165;	F:G-protein coupled receptor activity; F:melanocortin receptor activity; P:G[illegible]
Acrdi.XM_015898239.1/1-648 | Monca.augustus.g61840.t1/1-807	0.301386	0.220421	polyadenylate-binding 3-like isoform X2			
Acrdi.XM_015923455.1/1-27996 | Monca.augustus.g31109.t1/1-18288	0.20163	0.220409	Collagen alpha-3(VI) chain			
Acrdi.XM_015911777.1/1-747 | Monca.augustus.g5174.t1/1-1641	0.0858461	0.220408	major facilitator superfamily domain-containing 12-like	2	C:GO:0016020; C:GO:0016021	C:membrane; C:integral component of membrane
Acrdi.XM_015907223.1/1-1611 | Monca.augustus.g33144.t1/1-1578	0.249328	0.220389	Sialin	4	P:GO:0055085; F:GO:0022857; C:GO:0016021; P:GO:0006820	P:transmembrane transport; F:transmembrane transporter activity; C:integ[illegible]
Acrdi.XM_015904709.1/1-3840 | Monca.adi2mcaRNA14264_R0/1-3840	0.239241	0.220387	proline-rich 36-like	2	P:GO:0046373; F:GO:0046556	P:L-arabinose metabolic process; F:alpha-L-arabinofuranosidase activity
Acrdi.XM_015916606.1/1-2220 | Monca.augustus.g22960.t1/1-2244	0.259451	0.220323	non-canonical poly(A) RNA polymerase PAPD7 isoform X2			
Acrdi.XM_015903451.1/1-1596 | Monca.augustus.g49063.t1/1-1533	0.202986	0.220308	TPA: opsin	5	P:GO:0071482; C:GO:0005887; P:GO:0007186; F:GO:0008020;	P:cellular response to light stimulus; C:integral component of plasma mem[illegible]
Acrdi.XM_015902543.1/1-1323 | Monca.augustus.g39906.t1/1-1533	0.275184	0.220283	U3 small nucleolar RNA-associated 18 homolog	3	C:GO:0032040; P:GO:0000462; C:GO:0034388	C:small-subunit processome; P:maturation of SSU-rRNA from tricistronic r[illegible]
Acrdi.XM_015905578.1/1-1065 | Monca.augustus.g24106.t1/1-1068	0.219249	0.220276	PREDICTED: uncharacterized protein LOC107340224	2	C:GO:0016020; C:GO:0016021	C:membrane; C:integral component of membrane
Acrdi.XM_015914613.1/1-771 | Monca.augustus.g57116.t1/1-771	0.13267	0.220245	tropomyosin- isoforms 9A A B-like			
Acrdi.XM_015900037.1/1-771 | Monca.augustus.g6673.t1/1-870	0.186364	0.22016	tetraspanin-9 isoform X2	2	C:GO:0005887; P:GO:0007166	C:integral component of plasma membrane; P:cell surface receptor signali[illegible]
Acrdi.XM_015896792.1/1-1071 | Monca.augustus.g66627.t1/1-1053	0.229475	0.22015	BTB POZ domain-containing KCTD21	3	C:GO:0016020; C:GO:0016021; P:GO:0051260	C:membrane; C:integral component of membrane; P:protein homooligome[illegible]
Acrdi.XM_015914458.1/1-1419 | Monca.augustus.g71051.t1/1-1389	0.259348	0.220111	DBF4 homolog B isoform X3	2	F:GO:0003676; F:GO:0008270	F:nucleic acid binding; F:zinc ion binding
Acrdi.XM_015905932.1/1-1941 | Monca.augustus.g60549.t1/1-1938	0.172253	0.220047	kelch 12			
Acrdi.XM_015901725.1/1-666 | Monca.adi2mcaRNA11011_R0/1-675	0.236042	0.220045	Transmembrane 69	1	C:GO:0016020	C:membrane
Acrdi.XM_015922572.1/1-1290 | Monca.augustus.g54974.t1/1-1362	0.149623	0.220013	zinc metallo ase nas-13-like	7	F:GO:0008270; F:GO:0046872; P:GO:0006508; F:GO:0008233;	F:zinc ion binding; F:metal ion binding; P:proteolysis; F:peptidase activity; [illegible]
Acrdi.XM_015900179.1/1-687 | Monca.augustus.g59995.t1/1-690	0.264991	0.219947	Tctex1 domain-containing 1-B			
Acrdi.XM_015894744.1/1-777 | Monca.augustus.g22788.t1/1-696	0.498299	0.219923	PREDICTED: uncharacterized protein LOC107330097			
Acrdi.XM_015919524.1/1-504 | Monca.augustus.g36900.t1/1-510	0.205035	0.219751	nuclear nucleic acid-binding C1D			
Acrdi.XM_015904093.1/1-1407 | Monca.augustus.g44542.t1/1-1410	0.255966	0.219679	kinase D-interacting substrate of 220 kDa-			
Acrdi.XM_015924809.1/1-2811 | Monca.adi2mcaRNA36665_R0/1-2766	0.201946	0.219635	sperm-associated antigen 1			
Acrdi.XM_015905648.1/1-939 | Monca.augustus.g65075.t1/1-1713	0.318054	0.219629	chordin 1	5	P:GO:0009790; P:GO:0007389; P:GO:0009653; P:GO:0048513;	P:embryo development; P:pattern specification process; P:anatomical stru[illegible]
Acrdi.XM_015903202.1/1-831 | Monca.augustus.g44710.t1/1-3177	0.195582	0.219608	Coiled-coil domain-containing 17			
Acrdi.XM_015904876.1/1-756 | Monca.augustus.g29903.t1/1-756	0.307258	0.219561	Cation-dependent mannose-6-phosphate receptor	3	C:GO:0005802; C:GO:0016020; C:GO:0016021	C:trans-Golgi network; C:membrane; C:integral component of membrane
Acrdi.XM_015897868.1/1-1908 | Monca.augustus.g50625.t1/1-1872	0.237946	0.219554	rho-related racA-like	3	F:GO:0005525; P:GO:0007264; C:GO:0005622	F:GTP binding; P:small GTPase mediated signal transduction; C:intracellula[illegible]
Acrdi.XM_015902314.1/1-993 | Monca.augustus.g1765.t1/1-990	0.41376	0.219481	cathepsin L1-like	4	C:GO:0005615; F:GO:0004197; P:GO:0051603; C:GO:0005764	C:extracellular space; F:cysteine-type endopeptidase activity; P:proteolysis [illegible]
Acrdi.XM_015924970.1/1-609 | Monca.augustus.g40883.t1/1-702	0.185364	0.219473	disintegrin and metallo ase domain-containing 15-like			
Acrdi.XM_015907516.1/1-2106 | Monca.adi2mcaRNA17426_R0/1-2067	0.258529	0.219411	transcriptional regulator	2	F:GO:0005524; F:GO:0004386	F:ATP binding; F:helicase activity
Acrdi.XM_015905180.1/1-2298 | Monca.augustus.g52811.t1/1-4371	0.203197	0.219363	Werner syndrome ATP-dependent			
Acrdi.XM_015918790.1/1-2361 | Monca.augustus.g54020.t1/1-2418	0.277205	0.219305	Hormone-sensitive lipase	2	P:GO:0006629; F:GO:0016787	P:lipid metabolic process; F:hydrolase activity
Acrdi.XM_015906926.1/1-1911 | Monca.augustus.g33365.t1/1-1326	0.248926	0.219221	macrophage mannose receptor 1-like	3	C:GO:0016020; C:GO:0016021; F:GO:0030246	C:membrane; C:integral component of membrane; F:carbohydrate binding
Acrdi.XM_015903785.1/1-5625 | Monca.augustus.g66867.t1/1-8748	0.20613	0.219215	PREDICTED: uncharacterized protein LOC107338547 isoform X1			
Acrdi.XM_015891929.1/1-1890 | Monca.augustus.g25636.t1/1-2715	0.197693	0.219135	smoothelin isoform X2			
Acrdi.XM_015901345.1/1-1002 | Monca.augustus.g61868.t1/1-5766	0.326762	0.219123	PREDICTED: uncharacterized protein LOC107336274			
Acrdi.XM_015897126.1/1-1002 | Monca.augustus.g55696.t1/1-987	0.165731	0.219072	PREDICTED: uncharacterized protein LOC107332393			
Acrdi.XM_015910340.1/1-1224 | Monca.augustus.g36477.t1/1-1230	0.283229	0.219044	DNA polymerase subunit gamma- mitochondrial	3	F:GO:0004820; P:GO:0006426; C:GO:0005737	F:glycine-tRNA ligase activity; P:glycyl-tRNA aminoacylation; C:cytoplasm
Acrdi.XM_015911509.1/1-411 | Monca.augustus.g45458.t1/1-1869	0.236707	0.219022	Pre-mRNA 3 -end-processing factor			
Acrdi.XM_015909454.1/1-1245 | Monca.augustus.g35590.t1/1-2019	0.287082	0.219013	Double-stranded RNA-binding 1	1	F:GO:0016787	F:hydrolase activity
Acrdi.XM_015915921.1/1-888 | Monca.augustus.g24375.t1/1-888	0.239671	0.218924	octopamine receptor-like isoform X1	4	F:GO:0004930; P:GO:0007154; P:GO:0044700; P:GO:0050789	F:G-protein coupled receptor activity; P:cell communication; P:single orga[illegible]
Acrdi.XM_015897241.1/1-621 | Monca.augustus.g31734.t1/1-996	0.242764	0.218915	probable RNA polymerase II nuclear localization SLC7A6OS	7	C:GO:0005634; P:GO:0007417; C:GO:0005737; P:GO:0061053;	C:nucleus; P:central nervous system development; C:cytoplasm; P:somite d[illegible]
Acrdi.XM_015907724.1/1-975 | Monca.augustus.g68016.t1/1-993	0.191704	0.218914	CREB ATF bZIP transcription factor	5	C:GO:0005634; F:GO:0003700; P:GO:0006355; F:GO:0043565;	C:nucleus; F:transcription factor activity, sequence-specific DNA binding; P:[illegible]
Acrdi.XM_015902481.1/1-513 | Monca.augustus.g42489.t1/1-528	0.183527	0.218896	Nucleolar 16	2	P:GO:0042273; C:GO:0005730	P:ribosomal large subunit biogenesis; C:nucleolus
Acrdi.XM_015921719.1/1-840 | Monca.augustus.g12116.t1/1-717	0.125803	0.218887	probable N-acetyltransferase CML1	4	C:GO:0016020; C:GO:0016021; F:GO:0016740; F:GO:0008080	C:membrane; C:integral component of membrane; F:transferase activity; F[illegible]
Acrdi.XM_015897955.1/1-801 | Monca.adi2mcaRNA6952_R0/1-807	0.215625	0.218849	pleckstrin homology domain-containing family A member 1 isoform X1	4	C:GO:0005737; C:GO:0016020; F:GO:0030165; F:GO:0035091	C:cytoplasm; C:membrane; F:PDZ domain binding; F:phosphatidylinositol b[illegible]
Acrdi.XM_015915383.1/1-678 | Monca.augustus.g30172.t1/1-666	0.267338	0.218789	coiled-coil domain-containing 115	2	C:GO:0016020; C:GO:0005764	C:membrane; C:lysosome
Acrdi.XM_015893293.1/1-582 | Monca.augustus.g14106.t1/1-525	0.202797	0.21874	V-type proton ATPase 21 kDa proteolipid subunit	5	C:GO:0000220; P:GO:0015991; C:GO:0016021; F:GO:0046961;	C:vacuolar proton-transporting V-type ATPase, V0 domain; P:ATP hydrolysi[illegible]
Acrdi.XM_015922578.1/1-990 | Monca.augustus.g25376.t1/1-978	0.241069	0.218713	neuropeptide FF receptor 1-like	8	C:GO:0005887; F:GO:0004930; P:GO:0007186; P:GO:0007165;	C:integral component of plasma membrane; F:G-protein coupled receptor [illegible]
Acrdi.XM_015909101.1/1-618 | Monca.augustus.g67739.t1/1-597	0.129567	0.218708	homeobox -like 1	3	P:GO:0048522; P:GO:0048513; P:GO:0009888	P:positive regulation of cellular process; P:animal organ development; P:tis[illegible]
Acrdi.XM_015923098.1/1-1281 | Monca.augustus.g71467.t1/1-1440	0.0584308	0.218665	PREDICTED: uncharacterized protein LOC107356490	5	F:GO:0003677; P:GO:0090305; F:GO:0008270; F:GO:0004527;	F:DNA binding; P:nucleic acid phosphodiester bond hydrolysis; F:zinc ion bi[illegible]
Acrdi.XM_015907608.1/1-3090 | Monca.augustus.g50780.t1/1-3462	0.218404	0.218539	ubiquitin carboxyl-terminal hydrolase 19-like	4	P:GO:0016579; F:GO:0046872; F:GO:0036459; P:GO:0006511	P:protein deubiquitination; F:metal ion binding; F:thiol-dependent ubiquiti[illegible]
Acrdi.XM_015901264.1/1-1686 | Monca.augustus.g6189.t1/1-1692	0.248599	0.218526	diacylglycerol O-acyltransferase MT1809	7	F:GO:0047196; C:GO:0016020; C:GO:0016021; P:GO:0019432;	F:long-chain-alcohol O-fatty-acyltransferase activity; C:membrane; C:integ[illegible]
Acrdi.XM_015903505.1/1-1440 | Monca.adi2mcaRNA12975_R0/1-1455	0.165698	0.218522	PREDICTED: uncharacterized protein LOC107338274	3	C:GO:0005737; F:GO:0003824; P:GO:0006259	C:cytoplasm; F:catalytic activity; P:DNA metabolic process
Acrdi.XM_015898344.1/1-1392 | Monca.augustus.g44531.t1/1-1413	0.218807	0.218417	ATP-dependent (S)-NAD(P)H-hydrate dehydratase isoform X1			
Acrdi.XM_015924052.1/1-354 | Monca.augustus.g33007.t1/1-453	0.254123	0.218412	Zinc finger SWIM domain-containing 7	1	F:GO:0008270	F:zinc ion binding
Acrdi.XM_015911925.1/1-819 | Monca.adi2mcaRNA22237_R0/1-819	0.138164	0.218411	RAD51-associated 1-like			
Acrdi.XM_015920383.1/1-963 | Monca.augustus.g47489.t1/1-927	0.329701	0.218301	CREB ATF bZIP transcription factor isoform X1	5	C:GO:0005634; F:GO:0003700; P:GO:0006355; F:GO:0043565;	C:nucleus; F:transcription factor activity, sequence-specific DNA binding; P:[illegible]
Acrdi.XM_015923552.1/1-426 | Monca.augustus.g15160.t1/1-621	0.233664	0.218271	PREDICTED: uncharacterized protein LOC107356920			
Acrdi.XM_015918659.1/1-2415 | Monca.adi2mcaRNA29739_R0/1-2004	0.127389	0.218239	gamma-aminobutyric acid receptor subunit beta-2-like	5	P:GO:1902476; C:GO:0016021; F:GO:0005230; F:GO:0005254;	P:chloride transmembrane transport; C:integral component of membrane; [illegible]
Acrdi.XM_015921927.1/1-807 | Monca.adi2mcaRNA33333_R1/1-1056	0.434843	0.218223	PREDICTED: uncharacterized protein LOC107355353			
Acrdi.XM_015897756.1/1-492 | Monca.augustus.g46333.t1/1-957	0.179367	0.218217	Casein kinase II subunit beta			
Acrdi.XM_015920967.1/1-1404 | Monca.adi2mcaRNA3247_R0/1-1401	0.320011	0.21818	glutamic acid-rich -like isoform X1			
Acrdi.XM_015920297.1/1-5259 | Monca.augustus.g39622.t1/1-5727	0.268979	0.218179	regulator of G- signaling 22	4	C:GO:0005737; F:GO:0005096; P:GO:0043547; C:GO:0005886	C:cytoplasm; F:GTPase activator activity; P:positive regulation of GTPase a[illegible]
Acrdi.XM_015913697.1/1-2031 | Monca.adi2mcaRNA24218_R0/1-1980	0.238076	0.218165	adrenocorticotropic hormone receptor-like	6	F:GO:0004930; F:GO:0008270; P:GO:0007186; F:GO:0046872;	F:G-protein coupled receptor activity; F:zinc ion binding; P:G-protein couple[illegible]
Acrdi.XM_015920904.1/1-1323 | Monca.augustus.g2646.t1/1-1299	0.204184	0.218143	PREDICTED: uncharacterized protein LOC107354422			
Acrdi.XM_015906039.1/1-1371 | Monca.augustus.g34435.t1/1-1386	0.249723	0.218065	Major facilitator superfamily domain-containing 6	2	C:GO:0016020; C:GO:0016021	C:membrane; C:integral component of membrane
Acrdi.XM_015923216.1/1-1035 | Monca.augustus.g60461.t1/1-1032	0.273048	0.218039	Cytidine and dCMP deaminase domain-containing 1	1	F:GO:0016788	F:hydrolase activity, acting on ester bonds
Acrdi.XM_015895930.1/1-1260 | Monca.augustus.g58272.t1/1-1650	0.196696	0.218038	AF-9 isoform X1	7	P:GO:0090090; C:GO:0070062; C:GO:0008023; P:GO:2000096;	P:negative regulation of canonical Wnt signaling pathway; C:extracellular e[illegible]
Acrdi.XM_015901142.1/1-1257 | Monca.augustus.g1895.t1/1-1263	0.175133	0.21799	Ectonucleoside triphosphate diphosphohydrolase 1	1	F:GO:0016787	F:hydrolase activity
Acrdi.XM_015919823.1/1-3414 | Monca.augustus.g69877.t1/1-7698	0.236578	0.217965	nuclear receptor corepressor 1-like			
Acrdi.XM_015903156.1/1-771 | Monca.augustus.g52451.t1/1-702	0.285408	0.217935	transmembrane 198-like	2	C:GO:0016020; C:GO:0016021	C:membrane; C:integral component of membrane
Acrdi.XM_015905699.1/1-1809 | Monca.augustus.g18866.t1/1-2886	0.0580451	0.217925	PREDICTED: uncharacterized protein LOC107340343	1	F:GO:0046872	F:metal ion binding
Acrdi.XM_015893436.1/1-2190 | Monca.augustus.g50900.t1/1-2127	0.303251	0.217916	monocarboxylate transporter 10-like	3	P:GO:0034220; C:GO:0016021; F:GO:0005215	P:ion transmembrane transport; C:integral component of membrane; F:tra[illegible]
Acrdi.XM_015915602.1/1-996 | Monca.augustus.g12154.t1/1-1179	0.191722	0.217868	Sideroflexin-2	4	C:GO:0031966; P:GO:0034220; F:GO:0015075; C:GO:0016021	C:mitochondrial membrane; P:ion transmembrane transport; F:ion transm[illegible]
Acrdi.XM_015921748.1/1-2343 | Monca.adi2mcaRNA33119_R0/1-2505	0.203582	0.217863	complement factor B-1	1	F:GO:0008233	F:peptidase activity
Acrdi.XM_015896637.1/1-1584 | Monca.augustus.g21909.t1/1-1386	0.241312	0.217806	Peroxisomal carnitine O-octanoyltransferase	2	F:GO:0016746; F:GO:0016740	F:transferase activity, transferring acyl groups; F:transferase activity
Acrdi.XM_015902862.1/1-801 | Monca.adi2mcaRNA12291_R6/1-735	0.259623	0.217798	BTB POZ domain-containing 6-like			
Acrdi.XM_015916457.1/1-2007 | Monca.augustus.g2849.t1/1-942	0.168064	0.217675	monocarboxylate transporter 10-like	7	P:GO:0055085; P:GO:0034220; C:GO:0016020; P:GO:0015718;	P:transmembrane transport; P:ion transmembrane transport; C:membrane[illegible]
Acrdi.XM_015892892.1/1-879 | Monca.augustus.g7550.t1/1-1983	0.292988	0.217632	PREDICTED: uncharacterized protein LOC107328166			
Acrdi.XM_015902203.1/1-1338 | Monca.augustus.g58161.t1/1-1185	0.130376	0.217578	adenosine receptor A1-like	11	F:GO:0004930; F:GO:0001588; F:GO:0008270; P:GO:0001963;	F:G-protein coupled receptor activity; F:dopamine neurotransmitter recept[illegible]
Acrdi.XM_015899729.1/1-594 | Monca.augustus.g66179.t1/1-573	0.165963	0.217518	peptidase inhibitor 16-like isoform X1	1	C:GO:0005576	C:extracellular region
Acrdi.XM_015904764.1/1-1260 | Monca.augustus.g31932.t1/1-1290	0.251459	0.217517	PREDICTED: uncharacterized protein LOC107339471			
Acrdi.XM_015921515.1/1-1428 | Monca.augustus.g45822.t1/1-1398	0.273373	0.217477	FAM134C	3	C:GO:0016020; C:GO:0016021; P:GO:0010976	C:membrane; C:integral component of membrane; P:positive regulation of [illegible]
Acrdi.XM_015906036.1/1-3012 | Monca.augustus.g7161.t1/1-3330	0.294659	0.217451	Peptide toxins Am-1			
Acrdi.XM_015897116.1/1-450 | Monca.augustus.g55679.t1/1-324	0.280177	0.217437	PREDICTED: uncharacterized protein LOC107332383			
Acrdi.XM_015891699.1/1-1470 | Monca.adi2mcaRNA37566_R1/1-1470	0.180488	0.21743	proto-oncogene tyrosine- kinase receptor Ret-like			
Acrdi.XM_015924651.1/1-1305 | Monca.adi2mcaRNA36505_R0/1-1131	0.132301	0.217376	C-factor-like	1	C:GO:0005575	C:cellular_component
Acrdi.XM_015896993.1/1-1026 | Monca.augustus.g28883.t1/1-1029	0.282466	0.217369	dopamine receptor 2-like	3	F:GO:0004930; P:GO:0007154; P:GO:0044700	F:G-protein coupled receptor activity; P:cell communication; P:single orga[illegible]
Acrdi.XM_015891897.1/1-1518 | Monca.augustus.g25626.t1/1-1305	0.241556	0.217341	restin homolog isoform X1			
Acrdi.XM_015902242.1/1-486 | Monca.augustus.g35117.t1/1-984	0.236125	0.217334	t-SNARE domain-containing 1	5	F:GO:0005515; P:GO:0016192; C:GO:0044464; P:GO:0044763;	F:protein binding; P:vesicle-mediated transport; C:cell part; P:single-organi[illegible]
Acrdi.XM_015895590.1/1-3495 | Monca.augustus.g63252.t1/1-2787	0.223894	0.21733	LINE-1 retrotransposable element ORF2	1	P:GO:0007165	P:signal transduction
Acrdi.XM_015899804.1/1-1983 | Monca.augustus.g52529.t1/1-2499	0.214133	0.217202	deleted in malignant brain tumors 1 -like			
Acrdi.XM_015896411.1/1-957 | Monca.adi2mcaRNA5243_R0/1-1059	0.469642	0.217099	pollen-specific leucine-rich repeat extensin 2 isoform X2			
Acrdi.XM_015919271.1/1-1116 | Monca.augustus.g60224.t1/1-1044	0.213841	0.217092	substance-P receptor-like isoform X1	2	P:GO:0007165; C:GO:0016020	P:signal transduction; C:membrane
Acrdi.XM_015901864.1/1-579 | Monca.augustus.g56985.t1/1-612	0.234916	0.216998	Glucosidase 2 subunit beta	1	P:GO:0006491	P:N-glycan processing
Acrdi.XM_015903376.1/1-669 | Monca.augustus.g29879.t1/1-2661	0.246701	0.216898	UBX domain-containing 4-like isoform X1			
Acrdi.XM_015909758.1/1-672 | Monca.augustus.g46915.t1/1-1029	0.187153	0.216875	RNA-binding 24-like	10	F:GO:0000166; F:GO:0097157; F:GO:0005515; P:GO:0032956;	F:nucleotide binding; F:pre-mRNA intronic binding; F:protein binding; P:reg[illegible]
Acrdi.XM_015920933.1/1-1581 | Monca.augustus.g16465.t1/1-1551	0.20472	0.216802	PREDICTED: uncharacterized protein LOC107354448			

Acrdi.XM_015909374.1/1-975 | Monca.augustus.g50719.t1/1-960	0.209513	0.216799	zinc finger Gfi-1b-like	2	F:GO:0003676; F:GO:0046872	F:nucleic acid binding; F:metal ion binding
Acrdi.XM_015907273.1/1-1515 | Monca.adi2mcaRNA17117_R0/1-1506	0.19568	0.216788	deleted in malignant brain tumors 1 -like			
Acrdi.XM_015906917.1/1-4383 | Monca.augustus.g11639.t1/1-4290	0.253644	0.216784	Fanconi anemia group J isoform X2			
Acrdi.XM_015924639.1/1-1086 | Monca.augustus.g35404.t1/1-1092	0.25409	0.216727	melatonin receptor type 1C-like	2	P:GO:0007154; P:GO:0044700	P:cell communication; P:single organism signaling
Acrdi.XM_015903037.1/1-1116 | Monca.augustus.g56754.t1/1-1155	0.219101	0.216679	serine threonine- kinase pim-1-like			
Acrdi.XM_015896615.1/1-756 | Monca.augustus.g1582.t1/1-819	0.27361	0.216608	tRNA-specific adenosine deaminase 2	6	F:GO:0008270; F:GO:0005515; F:GO:0052717; C:GO:0052718; [illegible]	F:zinc ion binding; F:protein binding; F:tRNA-specific adenosine-34 deaminase activity; C:tRNA-specific [illegible]
Acrdi.XM_015924117.1/1-654 | Monca.augustus.g66958.t1/1-543	0.219045	0.216555	mediator of RNA polymerase II transcription subunit 30-like			
Acrdi.XM_015910547.1/1-876 | Monca.adi2mcaRNA2065_R0/1-999	0.307351	0.21652	RING finger 151-like	4	F:GO:0004842; F:GO:0008270; F:GO:0046872; P:GO:0016567	F:ubiquitin-protein transferase activity; F:zinc ion binding; F:metal ion binding; P:protein ubiquitination
Acrdi.XM_015908329.1/1-999 | Monca.augustus.g36788.t1/1-999	0.23614	0.216441	adenosine receptor A3-like	2	F:GO:0004930; P:GO:0007165	F:G-protein coupled receptor activity; P:signal transduction
Acrdi.XM_015924504.1/1-1290 | Monca.augustus.g28545.t1/1-1275	0.211752	0.21644	transcription factor AP-1	7	C:GO:0005634; F:GO:0031490; P:GO:0071310; P:GO:0045893; [illegible]	C:nucleus; F:chromatin DNA binding; P:cellular response to organic substance; P:positive regulation of [illegible]
Acrdi.XM_015902492.1/1-1056 | Monca.augustus.g42494.t1/1-1044	0.186252	0.216408	dopamine receptor 2-like	3	F:GO:0004930; P:GO:0007154; P:GO:0044700	F:G-protein coupled receptor activity; P:cell communication; P:single organism signaling
Acrdi.XM_015893606.1/1-1011 | Monca.augustus.g23916.t1/1-1446	0.217526	0.216368	Nuclear fragile X mental retardation-interacting 1	2	C:GO:0005634; F:GO:0005488	C:nucleus; F:binding
Acrdi.XM_015906143.1/1-774 | Monca.adi2mcaRNA28807_R4/1-885	0.661164	0.216352	follicle-stimulating hormone receptor-like	5	C:GO:0005887; F:GO:0008528; P:GO:0007189; P:GO:0007190; [illegible]	C:integral component of plasma membrane; F:G-protein coupled peptide receptor activity; P:adenylate [illegible]
Acrdi.XM_015892055.1/1-666 | Monca.augustus.g8373.t1/1-645	0.257032	0.21635	high affinity copper uptake 1	1	P:GO:0042221	P:response to chemical
Acrdi.XM_015907268.1/1-951 | Monca.augustus.g17638.t1/1-993	0.228475	0.216307	Wnt-1-like isoform X1	3	P:GO:0044763; C:GO:0005576; P:GO:0044767	P:single-organism cellular process; C:extracellular region; P:single-organism developmental process
Acrdi.XM_015905054.1/1-1407 | Monca.augustus.g60170.t1/1-501	0.255535	0.216208	F-box LRR-repeat 20			
Acrdi.XM_015895920.1/1-1224 | Monca.augustus.g58283.t1/1-1212	0.23845	0.2162	hydroxylysine kinase isoform X1	1	F:GO:0016740	F:transferase activity
Acrdi.XM_015912463.1/1-600 | Monca.augustus.g11247.t1/1-1458	0.106202	0.216143	Serine threonine- kinase pim-3			
Acrdi.XM_015922022.1/1-1320 | Monca.augustus.g46435.t1/1-1236	0.303523	0.216131	family with sequence similarity member B	3	F:GO:0005509; C:GO:0016020; C:GO:0016021	F:calcium ion binding; C:membrane; C:integral component of membrane
Acrdi.XM_015917874.1/1-636 | Monca.augustus.g31696.t1/1-519	0.148853	0.216127	PREDICTED: uncharacterized protein LOC107351580			
Acrdi.XM_015911956.1/1-1098 | Monca.augustus.g58629.t1/1-1101	0.165662	0.216116	octopamine receptor-like	3	F:GO:0004930; P:GO:0007154; P:GO:0044700	F:G-protein coupled receptor activity; P:cell communication; P:single organism signaling
Acrdi.XM_015894380.1/1-1710 | Monca.adi2mcaRNA4092_R0/1-1617	0.198489	0.216037	Telomerase Cajal body 1			
Acrdi.XM_015901163.1/1-2181 | Monca.adi2mcaRNA10397_R0/1-1992	0.305914	0.215928	Retinoblastoma-associated			
Acrdi.XM_015917295.1/1-453 | Monca.augustus.g40282.t1/1-807	0.442673	0.215913	alpha-1,3-mannosyl-glyco 2-beta-N-acetylglucosaminyltransferase	4	F:GO:0008375; C:GO:0016020; C:GO:0016021; P:GO:0006486	F:acetylglucosaminyltransferase activity; C:membrane; C:integral component of membrane; P:protein gl[illegible]
Acrdi.XM_015908464.1/1-429 | Monca.augustus.g11527.t1/1-432	0.358177	0.215885	transmembrane 35	2	C:GO:0016020; C:GO:0016021	C:membrane; C:integral component of membrane
Acrdi.XM_015915377.1/1-4068 | Monca.augustus.g30180.t1/1-5475	0.232798	0.215863	tyrosine- phosphatase non-receptor	2	F:GO:0004721; P:GO:0006470	F:phosphoprotein phosphatase activity; P:protein dephosphorylation
Acrdi.XM_015912099.1/1-1653 | Monca.augustus.g24169.t1/1-2184	0.173613	0.21578	piRNA biogenesis EXD1 isoform X1	1	P:GO:0090304	P:nucleic acid metabolic process
Acrdi.XM_015921749.1/1-2505 | Monca.adi2mcaRNA33118_R0/1-2481	0.279663	0.215717	von Willebrand factor type EGF and pentraxin domain-containing 1	2	F:GO:0004867; P:GO:0010951	F:serine-type endopeptidase inhibitor activity; P:negative regulation of endopeptidase activity
Acrdi.XM_015914163.1/1-678 | Monca.augustus.g28622.t1/1-1374	0.188726	0.21568	hemicentin-1-like isoform X1			
Acrdi.XM_015920787.1/1-567 | Monca.adi2mcaRNA32094_R0/1-552	0.332628	0.215575	slit homolog 1 -like isoform X1	2	C:GO:0016020; C:GO:0016021	C:membrane; C:integral component of membrane
Acrdi.XM_015921015.1/1-843 | Monca.adi2mcaRNA32320_R4/1-849	0.0643721	0.215464	jerky homolog-like			
Acrdi.XM_015921286.1/1-3129 | Monca.augustus.g21739.t1/1-3144	0.239992	0.215397	zinc finger 271-like	3	F:GO:0003700; P:GO:0006355; F:GO:0005488	F:transcription factor activity, sequence-specific DNA binding; P:regulation of transcription, DNA-templat[illegible]
Acrdi.XM_015899319.1/1-1155 | Monca.augustus.g64397.t1/1-1230	0.181973	0.215378	substance-K receptor-like	3	F:GO:0008528; P:GO:0007186; C:GO:0016021	F:G-protein coupled peptide receptor activity; P:G-protein coupled receptor signaling pathway; C:integral [illegible]
Acrdi.XM_015919087.1/1-384 | Monca.augustus.g17787.t1/1-999	0.302451	0.215241	tRNA-splicing endonuclease subunit Sen54	1	P:GO:0016070	P:RNA metabolic process
Acrdi.XM_015911946.1/1-1680 | Monca.augustus.g24225.t1/1-1605	0.162289	0.215226	potassium channel subfamily K member 4-like	4	C:GO:0005887; P:GO:0071805; P:GO:0030322; F:GO:0022841	C:integral component of plasma membrane; P:potassium ion transmembrane transport; P:stabilization [illegible]
Acrdi.XM_015897575.1/1-3429 | Monca.augustus.g31032.t1/1-3606	0.170957	0.215145	PREDICTED: uncharacterized protein LOC107332838			
Acrdi.XM_015898430.1/1-777 | Monca.augustus.g35067.t1/1-780	0.252807	0.215136	PREDICTED: uncharacterized protein LOC107333601	3	C:GO:0016020; C:GO:0016021; C:GO:0030133	C:membrane; C:integral component of membrane; C:transport vesicle
Acrdi.XM_015912049.1/1-636 | Monca.augustus.g67636.t1/1-1512	0.22205	0.215103	PREDICTED: uncharacterized protein LOC107346275 isoform X3	2	C:GO:0016020; C:GO:0016021	C:membrane; C:integral component of membrane
Acrdi.XM_015921036.1/1-1101 | Monca.augustus.g10690.t1/1-1113	0.180486	0.215101	E3 ubiquitin- ligase MARCH8-like	2	F:GO:0046872; C:GO:0016020	F:metal ion binding; C:membrane
Acrdi.XM_015912191.1/1-1059 | Monca.augustus.g34498.t1/1-1254	0.202959	0.215063	MAM and LDL-receptor class A domain-containing 2-like			
Acrdi.XM_015903572.1/1-546 | Monca.adi2mcaRNA13039_R0/1-372	0.25782	0.215021	probable 28S ribosomal mitochondrial	1	C:GO:0005739	C:mitochondrion
Acrdi.XM_015894227.1/1-1386 | Monca.adi2mcaRNA40465_R0/1-1446	0.162988	0.215016	canopy homolog 4			
Acrdi.XM_015919453.1/1-1695 | Monca.augustus.g25236.t1/1-1905	0.231595	0.214997	seleno O-like			
Acrdi.XM_015908460.1/1-597 | Monca.augustus.g33930.t1/1-1281	0.293713	0.214979	DNA-binding transcriptional activator of the SARP family	4	P:GO:0006629; C:GO:0016020; C:GO:0016021; F:GO:0008081	P:lipid metabolic process; C:membrane; C:integral component of membra[illegible]
Acrdi.XM_015904329.1/1-600 | Monca.augustus.g31913.t1/1-1308	0.173219	0.214969	Coiled-coil domain-containing 51			
Acrdi.XM_015923007.1/1-1284 | Monca.augustus.g46067.t1/1-1470	0.124332	0.214912	singed	1	F:GO:0005515	F:protein binding
Acrdi.XM_015924303.1/1-351 | Monca.augustus.g39155.t1/1-3129	0.22176	0.214861	zinc finger CCCH domain-containing 18-like			
Acrdi.XM_015899818.1/1-2685 | Monca.augustus.g6185.t1/1-3297	0.251844	0.214859	sorting nexin-29-like	6	F:GO:0016887; F:GO:0003777; P:GO:0000070; C:GO:0005871; [illegible]	F:ATPase activity; F:microtubule motor activity; P:mitotic sister chromatid [illegible]
Acrdi.XM_015911365.1/1-906 | Monca.augustus.g35276.t1/1-882	0.153461	0.214823	melanocyte-stimulating hormone receptor-like	7	F:GO:0004930; P:GO:0007186; P:GO:0007165; C:GO:0016020; [illegible]	F:G-protein coupled receptor activity; P:G-protein coupled receptor signali[illegible]
Acrdi.XM_015913945.1/1-3486 | Monca.augustus.g59708.t1/1-5541	0.291514	0.214804	E3 ubiquitin- ligase listerin			
Acrdi.XM_015922004.1/1-3531 | Monca.augustus.g60061.t1/1-3606	0.26325	0.214799	acyl- synthetase family member 4 isoform X1	8	F:GO:0000166; F:GO:0005524; F:GO:0016878; F:GO:0003824; [illegible]	F:nucleotide binding; F:ATP binding; F:acid-thiol ligase activity; F:catalytic [illegible]
Acrdi.XM_015921980.1/1-1734 | Monca.augustus.g8349.t1/1-1596	0.211798	0.214742	PREDICTED: uncharacterized protein LOC107355398	3	C:GO:0005739; F:GO:0003690; P:GO:0006355	C:mitochondrion; F:double-stranded DNA binding; P:regulation of transcrip[illegible]
Acrdi.XM_015913651.1/1-2736 | Monca.adi2mcaRNA24145_R0/1-2961	0.248621	0.214734	WSC domain-containing 2-like	5	F:GO:0030414; F:GO:0004867; P:GO:0010951; C:GO:0005576; [illegible]	F:peptidase inhibitor activity; F:serine-type endopeptidase inhibitor activity[illegible]
Acrdi.XM_015900918.1/1-3543 | Monca.augustus.g27882.t1/1-3498	0.24072	0.214732	contactin-associated -like 2	2	C:GO:0016020; C:GO:0016021	C:membrane; C:integral component of membrane
Acrdi.XM_015917207.1/1-618 | Monca.augustus.g54725.t1/1-666	0.282097	0.214726	PREDICTED: uncharacterized protein LOC107350941			
Acrdi.XM_015916044.1/1-2679 | Monca.augustus.g67868.t1/1-3474	0.229055	0.214707	VPS10 domain-containing receptor 1-like	2	C:GO:0016020; C:GO:0016021	C:membrane; C:integral component of membrane
Acrdi.XM_015925133.1/1-1635 | Monca.augustus.g37268.t1/1-2325	0.182023	0.214681	GRAM domain-containing 4	2	C:GO:0016020; C:GO:0016021	C:membrane; C:integral component of membrane
Acrdi.XM_015923155.1/1-1125 | Monca.augustus.g70147.t1/1-1137	0.237123	0.214644	CUB domain-containing 2	8	F:GO:0008270; F:GO:0046872; F:GO:0005509; P:GO:0006508; [illegible]	F:zinc ion binding; F:metal ion binding; F:calcium ion binding; P:proteolysis[illegible]
Acrdi.XM_015898272.1/1-681 | Monca.adi2mcaRNA7268_R0/1-726	0.235961	0.214551	PREDICTED: uncharacterized protein LOC107333455	1	C:GO:0016020	C:membrane
Acrdi.XM_015900548.1/1-732 | Monca.augustus.g26472.t1/1-723	0.221803	0.214547	PREDICTED: uncharacterized protein LOC107335527			
Acrdi.XM_015918465.1/1-432 | Monca.augustus.g55070.t1/1-1638	0.274504	0.214533	PREDICTED: protein ALEX-like			
Acrdi.XM_015919445.1/1-1137 | Monca.augustus.g31487.t1/1-1116	0.287059	0.214529	PREDICTED: uncharacterized protein LOC107353124			
Acrdi.XM_015901794.1/1-2439 | Monca.augustus.g34954.t1/1-3216	0.229196	0.214513	-related lipid transfer 9	3	C:GO:0016020; C:GO:0016021; F:GO:0008289	C:membrane; C:integral component of membrane; F:lipid binding
Acrdi.XM_015908590.1/1-1728 | Monca.augustus.g28020.t1/1-1692	0.233169	0.214459	Tripartite motif-containing 2	2	F:GO:0008270; F:GO:0046872	F:zinc ion binding; F:metal ion binding
Acrdi.XM_015893990.1/1-1077 | Monca.augustus.g11691.t1/1-630	0.376266	0.214422	probable serine threonine- kinase DDB_G0271682			
Acrdi.XM_015903476.1/1-471 | Monca.augustus.g1048.t1/1-522	0.306802	0.214398	PREDICTED: uncharacterized protein LOC107338238			
Acrdi.XM_015905727.1/1-693 | Monca.augustus.g9358.t1/1-771	0.21066	0.214382	nap homolog 3A-like isoform X2			
Acrdi.XM_015923739.1/1-525 | Monca.augustus.g32968.t1/1-717	0.205819	0.214366	syntaxin-8	4	F:GO:0008270; C:GO:0012505; C:GO:0044444; C:GO:0043227	F:zinc ion binding; C:endomembrane system; C:cytoplasmic part; C:membr[illegible]
Acrdi.XM_015896893.1/1-633 | Monca.augustus.g33183.t1/1-573	0.201608	0.214355	riboflavin-binding -like			
Acrdi.XM_015899714.1/1-4407 | Monca.augustus.g66182.t1/1-4080	0.248839	0.214347	Codanin-			
Acrdi.XM_015915334.1/1-1323 | Monca.adi2mcaRNA26004_R0/1-1308	0.26533	0.214318	monocarboxylate transporter 10-like	5	P:GO:0055085; P:GO:0034220; C:GO:0016020; C:GO:0016021; [illegible]	P:transmembrane transport; P:ion transmembrane transport; C:membrane[illegible]
Acrdi.XM_015912421.1/1-1611 | Monca.augustus.g40749.t1/1-2832	0.157043	0.214308	PREDICTED: uncharacterized protein LOC107346612			
Acrdi.XM_015898345.1/1-1287 | Monca.augustus.g44530.t1/1-1278	0.258906	0.214254	Ankyrin repeat domain-containing 33B			
Acrdi.XM_015913630.1/1-840 | Monca.augustus.g48688.t1/1-834	0.400883	0.214247	UPF0692 C19orf54 homolog			
Acrdi.XM_015900377.1/1-444 | Monca.augustus.g54194.t1/1-465	0.186343	0.214111	PREDICTED: uncharacterized protein LOC107335365	2	P:GO:0045892; P:GO:0042754	P:negative regulation of transcription, DNA-templated; P:negative regulati[illegible]
Acrdi.XM_015907841.1/1-609 | Monca.augustus.g47386.t1/1-600	0.213304	0.214104	F-box only 36	3	P:GO:0031146; C:GO:0005575; F:GO:0003674	P:SCF-dependent proteasomal ubiquitin-dependent protein catabolic proce[illegible]
Acrdi.XM_015893209.1/1-4167 | Monca.augustus.g69405.t1/1-3915	0.229848	0.214083	MICAL 2	2	F:GO:0008270; F:GO:0046872	F:zinc ion binding; F:metal ion binding
Acrdi.XM_015904722.1/1-1491 | Monca.adi2mcaRNA14268_R0/1-1518	0.276684	0.214031	fibronectin isoform X5	25	F:GO:0002020; F:GO:0005178; C:GO:0005793; P:GO:1904237; [illegible]	F:protease binding; F:integrin binding; C:endoplasmic reticulum-Golgi inte[illegible]
Acrdi.XM_015900708.1/1-2478 | Monca.adi2mcaRNA9970_R3/1-1902	0.699568	0.214004	RNA-directed DNA polymerase from mobile element jockey-like			
Acrdi.XM_015917770.1/1-2094 | Monca.augustus.g61062.t1/1-2313	0.228402	0.213982	rho GTPase-activating 9 isoform X3	2	P:GO:0007165; F:GO:0005543	P:signal transduction; F:phospholipid binding
Acrdi.XM_015896981.1/1-750 | Monca.augustus.g50249.t1/1-738	0.256	0.21394	piggyBac transposable element-derived 4-like			
Acrdi.XM_015923369.1/1-1878 | Monca.augustus.g24894.t1/1-1932	0.249926	0.21394	Nucleolar MIF4G domain-containing 1			
Acrdi.XM_015916732.1/1-1017 | Monca.augustus.g37101.t1/1-1056	0.151061	0.213915	TGF-beta-activated kinase 1 and MAP3K7-binding 3 isoform X1	7	F:GO:0008270; C:GO:0070062; C:GO:0005737; F:GO:0046872; [illegible]	F:zinc ion binding; C:extracellular exosome; C:cytoplasm; F:metal ion bindi[illegible]
Acrdi.XM_015903330.1/1-4686 | Monca.adi2mcaRNA1380_R0/1-4260	0.246737	0.213806	A disintegrin and metallo ase with thrombospondin motifs 6	6	F:GO:0008270; P:GO:0006508; C:GO:0031012; F:GO:0004222; [illegible]	F:zinc ion binding; P:proteolysis; C:extracellular matrix; F:metalloendopept[illegible]
Acrdi.XM_015919936.1/1-567 | Monca.augustus.g38718.t1/1-633	0.319809	0.213789	ubiquitin-like-conjugating enzyme	4	P:GO:0006914; P:GO:0006497; F:GO:0019777; P:GO:0032446	P:autophagy; P:protein lipidation; F:Atg12 transferase activity; P:protein m[illegible]
Acrdi.XM_015924705.1/1-969 | Monca.augustus.g55029.t1/1-975	0.201563	0.213754	somatostatin receptor type 4-like	4	F:GO:0004930; P:GO:0007186; C:GO:0016020; C:GO:0016021	F:G-protein coupled receptor activity; P:G-protein coupled receptor signali[illegible]
Acrdi.XM_015916404.1/1-1176 | Monca.augustus.g40951.t1/1-1245	0.247671	0.213703	starmaker-like isoform X2			
Acrdi.XM_015905762.1/1-1113 | Monca.augustus.g32749.t1/1-1191	0.205107	0.213646	neuropeptide Y receptor type 6	3	P:GO:0007165; C:GO:0016020; F:GO:0004871	P:signal transduction; C:membrane; F:signal transducer activity
Acrdi.XM_015921953.1/1-5127 | Monca.augustus.g23749.t1/1-5349	0.300195	0.213576	Adhesion G -coupled receptor A3	8	F:GO:0004930; F:GO:0004888; P:GO:0007186; P:GO:0007165; [illegible]	F:G-protein coupled receptor activity; F:transmembrane signaling receptor[illegible]
Acrdi.XM_015907502.1/1-1641 | Monca.augustus.g55648.t1/1-1713	0.298095	0.213506	major facilitator superfamily domain-containing 12-like	2	C:GO:0016020; C:GO:0016021	C:membrane; C:integral component of membrane
Acrdi.XM_015904897.1/1-3777 | Monca.augustus.g61115.t1/1-3735	0.265351	0.213471	CST complex subunit	1	F:GO:0003697	F:single-stranded DNA binding
Acrdi.XM_015905690.1/1-1131 | Monca.augustus.g26825.t1/1-1116	0.244173	0.213406	probable glycerophosphoryl diester phosphodiesterase 3	3	P:GO:0006629; F:GO:0008889; F:GO:0008081	P:lipid metabolic process; F:glycerophosphodiester phosphodiesterase acti[illegible]
Acrdi.XM_015897447.1/1-384 | Monca.augustus.g35236.t1/1-486	0.261922	0.213379	probable D-tyrosyl-tRNA(Tyr) deacylase 2	7	F:GO:0016788; F:GO:0051500; C:GO:0005737; P:GO:0006450; [illegible]	F:hydrolase activity, acting on ester bonds; F:D-tyrosyl-tRNA(Tyr) deacylase [illegible]
Acrdi.XM_015908608.1/1-2115 | Monca.adi2mcaRNA1849_R0/1-2091	0.11023	0.213364	Long-chain-fatty-acid-- ligase ACSBG2	3	F:GO:0000166; F:GO:0016874; P:GO:0006631	F:nucleotide binding; F:ligase activity; P:fatty acid metabolic process
Acrdi.XM_015898666.1/1-957 | Monca.augustus.g34612.t1/1-966	0.233437	0.213334	PREDICTED: uncharacterized protein LOC107333796	2	C:GO:0016020; C:GO:0016021	C:membrane; C:integral component of membrane
Acrdi.XM_015900367.1/1-2157 | Monca.augustus.g9983.t1/1-675	0.170543	0.213304	nucleo TPR-like isoform X2			
Acrdi.XM_015891858.1/1-810 | Monca.augustus.g23262.t1/1-1728	0.341667	0.213302	Cubilin	1	F:GO:0005509	F:calcium ion binding
Acrdi.XM_015919143.1/1-420 | Monca.augustus.g35181.t1/1-411	0.310082	0.213297	Ubiquitin 4A	2	P:GO:0051234; C:GO:0005829	P:establishment of localization; C:cytosol
Acrdi.XM_015898665.1/1-1122 | Monca.augustus.g34608.t1/1-1104	0.224601	0.213285	leucine-rich repeat-containing 74B-like	1	F:GO:0005509	F:calcium ion binding
Acrdi.XM_015897032.1/1-2667 | Monca.augustus.g64672.t1/1-1878	0.230676	0.213279	ubiquitin carboxyl-terminal hydrolase 4 isoform X2	11	F:GO:0004843; P:GO:0016579; P:GO:0034394; C:GO:0005634; [illegible]	F:thiol-dependent ubiquitin-specific protease activity; P:protein deubiquitin[illegible]
Acrdi.XM_015910944.1/1-1989 | Monca.augustus.g3083.t1/1-1296	0.168135	0.213207	Interferon-induced 44	7	F:GO:0005525; F:GO:0008270; F:GO:0003735; P:GO:0007165; [illegible]	F:GTP binding; F:zinc ion binding; F:structural constituent of ribosome; P:si[illegible]
Acrdi.XM_015914059.1/1-2568 | Monca.augustus.g24061.t1/1-3108	0.166063	0.213187	RNA-binding 27 isoform X2	2	F:GO:0097159; F:GO:1901363	F:organic cyclic compound binding; F:heterocyclic compound binding
Acrdi.XM_015905289.1/1-2580 | Monca.adi2mcaRNA1533_R3/1-1890	0.214833	0.213098	macrophage mannose receptor 1-like	3	C:GO:0016020; C:GO:0016021; F:GO:0030246	C:membrane; C:integral component of membrane; F:carbohydrate binding
Acrdi.XM_015905402.1/1-543 | Monca.augustus.g33958.t1/1-897	0.172278	0.21306	calumenin	1	F:GO:0005509	F:calcium ion binding
Acrdi.XM_015898267.1/1-1173 | Monca.augustus.g64608.t1/1-1134	0.224596	0.213035	centromere L-like			
Acrdi.XM_015915235.1/1-822 | Monca.augustus.g41796.t1/1-822	0.265406	0.213001	leucine-rich repeat-containing DDB_G0290503			
Acrdi.XM_015918642.1/1-2124 | Monca.augustus.g33671.t1/1-2040	0.262124	0.212998	beta-1,4-N-acetylgalactosaminyltransferase 3-like	3	C:GO:0032580; F:GO:0008376; F:GO:0016740	C:Golgi cisterna membrane; F:acetylgalactosaminyltransferase activity; F:[illegible]
Acrdi.XM_015901840.1/1-2250 | Monca.augustus.g7720.t1/1-1581	0.173368	0.212996	aspartyl asparaginyl beta-hydroxylase-like	2	P:GO:0018193; C:GO:0016020	P:peptidyl-amino acid modification; C:membrane
Acrdi.XM_015901904.1/1-1221 | Monca.augustus.g58302.t1/1-1272	0.151106	0.212914	ZP domain-containing	1	C:GO:0016021	C:integral component of membrane
Acrdi.XM_015896881.1/1-573 | Monca.adi2mcaRNA5743_R1/1-525	0.20465	0.2129	PREDICTED: uncharacterized protein LOC107332156			
Acrdi.XM_015904160.1/1-1209 | Monca.augustus.g30768.t1/1-1647	0.197682	0.212849	PREDICTED: uncharacterized protein LOC107338906			
Acrdi.XM_015923231.1/1-1530 | Monca.augustus.g69880.t1/1-2757	0.212934	0.21282	PREDICTED: uncharacterized protein LOC107356627			
Acrdi.XM_015919153.1/1-792 | Monca.augustus.g35151.t1/1-1824	0.264815	0.212786	NCK-interacting with SH3			
Acrdi.XM_015921846.1/1-471 | Monca.augustus.g50984.t1/1-405	0.339434	0.212756	PREDICTED: uncharacterized protein LOC107355305			
Acrdi.XM_015896171.1/1-1716 | Monca.augustus.g23038.t1/1-1761	0.237312	0.212663	DEAD-box ATP-dependent RNA helicase 22	1	F:GO:0005488	F:binding
Acrdi.XM_015909993.1/1-1062 | Monca.augustus.g44405.t1/1-2019	0.217123	0.212652	pseudouridylate synthase 7 homolog	2	P:GO:0009451; F:GO:0016853	P:RNA modification; F:isomerase activity
Acrdi.XM_015910454.1/1-1407 | Monca.augustus.g41058.t1/1-1341	0.225528	0.212631	steroid 17-alpha-hydroxylase 17,20 lyase-like isoform X2	5	P:GO:0042448; F:GO:0004508; F:GO:0047442; F:GO:0047006; [illegible]	P:progesterone metabolic process; F:steroid 17-alpha-monooxygenase acti[illegible]
Acrdi.XM_015896791.1/1-1194 | Monca.augustus.g66638.t1/1-1203	0.180539	0.212612	skeletal aspartic acid-rich 2-like	2	C:GO:0016020; C:GO:0016021	C:membrane; C:integral component of membrane
Acrdi.XM_015899693.1/1-1035 | Monca.augustus.g35413.t1/1-1149	0.810426	0.212588	2 -5 -oligoadenylate synthase 1	1	F:GO:0005488	F:binding
Acrdi.XM_015923487.1/1-978 | Monca.augustus.g42217.t1/1-999	0.128073	0.212583	tartrate-resistant acid phosphatase type 5-like	1	F:GO:0016787	F:hydrolase activity
Acrdi.XM_015922147.1/1-1719 | Monca.adi2mcaRNA33574_R1/1-1722	0.211338	0.212582	probable ATP-dependent RNA helicase DDX10	4	F:GO:0005524; F:GO:0003723; P:GO:0010501; F:GO:0004004	F:ATP binding; F:RNA binding; P:RNA secondary structure unwinding; F:AT[illegible]
Acrdi.XM_015898743.1/1-513 | Monca.augustus.g14701.t1/1-810	0.182271	0.212578	Geminin	1	P:GO:0006275	P:regulation of DNA replication
Acrdi.XM_015897670.1/1-1359 | Monca.augustus.g17412.t1/1-2205	0.177103	0.212558	ral guanine nucleotide dissociation stimulator-like 1	7	P:GO:0007165; P:GO:0007264; F:GO:0005085; C:GO:0016020; [illegible]	P:signal transduction; P:small GTPase mediated signal transduction; F:gua[illegible]
Acrdi.XM_015902919.1/1-858 | Monca.augustus.g54842.t1/1-1704	0.171625	0.212553	Kynurenine 3-monooxygenase and related flavo monooxygenases (ISS)			
Acrdi.XM_015905240.1/1-726 | Monca.adi2mcaRNA14851_R1/1-681	0.151289	0.212505	Swarming motility ybiA			
Acrdi.XM_015924451.1/1-519 | Monca.adi2mcaRNA36231_R1/1-723	0.142951	0.212497	myb SANT-like DNA-binding domain-containing 4	2	C:GO:0005634; P:GO:0006915	C:nucleus; P:apoptotic process
Acrdi.XM_015899269.1/1-1092 | Monca.augustus.g13003.t1/1-1392	0.196247	0.212484	LTV1 homolog	1	C:GO:0044424	C:intracellular part
Acrdi.XM_015909268.1/1-1287 | Monca.augustus.g35333.t1/1-1275	0.229983	0.212458	chromosome 6 open reading frame 136			
Acrdi.XM_015892730.1/1-420 | Monca.augustus.g54278.t1/1-648	0.132263	0.212437	eukaryotic translation initiation factor 3 subunit K	4	C:GO:0032991; C:GO:0044444; F:GO:0005488; P:GO:0006412	C:macromolecular complex; C:cytoplasmic part; F:binding; P:translation
Acrdi.XM_015904094.1/1-594 | Monca.augustus.g44544.t1/1-441	0.224995	0.212395	leukocyte receptor cluster member 8-like			
Acrdi.XM_015916449.1/1-1296 | Monca.augustus.g58756.t1/1-1335	0.178977	0.212374	mab-21	5	P:GO:0008150; C:GO:0016020; C:GO:0016021; C:GO:0005575; [illegible]	P:biological_process; C:membrane; C:integral component of membrane; C:[illegible]
Acrdi.XM_015908519.1/1-684 | Monca.augustus.g65962.t1/1-687	0.396416	0.21231	Ribonuclease P subunit p29	7	F:GO:0004526; P:GO:0090501; C:GO:0000172; F:GO:0000171; [illegible]	F:ribonuclease P activity; P:RNA phosphodiester bond hydrolysis; C:ribonuc[illegible]
Acrdi.XM_015919648.1/1-1068 | Monca.augustus.g32518.t1/1-1110	0.272333	0.212306	histamine H2 receptor-like	3	P:GO:0007154; P:GO:0044700; F:GO:0004871	P:cell communication; P:single organism signaling; F:signal transducer act[illegible]
Acrdi.XM_015918615.1/1-2331 | Monca.augustus.g7622.t1/1-2079	0.152957	0.212238	pumilio domain-containing KIAA0020 homolog	3	P:GO:0006417; F:GO:0003729; C:GO:0005730	P:regulation of translation; F:mRNA binding; C:nucleolus
Acrdi.XM_015892663.1/1-1992 | Monca.adi2mcaRNA38640_R0/1-1638	0.0808637	0.212189	dnaJ homolog subfamily C member 3-like			
Acrdi.XM_015924893.1/1-1659 | Monca.augustus.g35002.t1/1-1470	0.231407	0.212095	Inactive rhomboid 1	10	C:GO:0005789; P:GO:0050708; C:GO:0016020; P:GO:0006508; [illegible]	C:endoplasmic reticulum membrane; P:regulation of protein secretion; C:m[illegible]
Acrdi.XM_015912085.1/1-738 | Monca.augustus.g49198.t1/1-741	0.250076	0.212053	NEDD8-conjugating enzyme UBC12-like	5	F:GO:0061630; F:GO:0031625; C:GO:0005737; P:GO:0045116; [illegible]	F:ubiquitin protein ligase activity; F:ubiquitin protein ligase binding; C:cyto[illegible]
Acrdi.XM_015904959.1/1-1524 | Monca.augustus.g46466.t1/1-1509	0.267424	0.21199	galanin receptor type 1-like	3	P:GO:0007165; C:GO:0016020; F:GO:0004871	P:signal transduction; C:membrane; F:signal transducer activity
Acrdi.XM_015911476.1/1-1302 | Monca.augustus.g53620.t1/1-1164	0.144773	0.211958	galactose-3-O-sulfotransferase 2-like	4	F:GO:0001733; C:GO:0016021; P:GO:0009247; C:GO:0005794	F:galactosylceramide sulfotransferase activity; C:integral component of m[illegible]
Acrdi.XM_015899462.1/1-1068 | Monca.augustus.g37263.t1/1-1080	0.30232	0.211948	metallophosphoesterase 1 homolog isoform X1	5	P:GO:0006888; C:GO:0005737; C:GO:0016020; C:GO:0016021; [illegible]	P:ER to Golgi vesicle-mediated transport; C:cytoplasm; C:membrane; C:inte[illegible]
Acrdi.XM_015896459.1/1-1107 | Monca.augustus.g54916.t1/1-1101	0.242093	0.211943	kelch domain-containing 8B			
Acrdi.XM_015897122.1/1-2748 | Monca.adi2mcaRNA6019_R0/1-2787	0.271067	0.211766	PREDICTED: uncharacterized protein LOC107332388			
Acrdi.XM_015907977.1/1-4293 | Monca.adi2mcaRNA17871_R0/1-3831	0.128002	0.211723	cullin-associated NEDD8-dissociated 1	3	C:GO:0005634; P:GO:0010265; P:GO:0016567	C:nucleus; P:SCF complex assembly; P:protein ubiquitination
Acrdi.XM_015918754.1/1-1821 | Monca.augustus.g43629.t1/1-2058	0.222876	0.211668	WD G-beta repeat-containing			
Acrdi.XM_015922353.1/1-1158 | Monca.adi2mcaRNA3425_R0/1-1101	0.207253	0.211641	lysophosphatidylcholine acyltransferase 2	5	F:GO:0016746; F:GO:0005509; P:GO:0008152; C:GO:0016020; [illegible]	F:transferase activity, transferring acyl groups; F:calcium ion binding; P:me[illegible]
Acrdi.XM_015907952.1/1-2349 | Monca.augustus.g525.t1/1-2319	0.215091	0.211623	insulin receptor substrate 1	8	F:GO:0043548; P:GO:0008286; C:GO:0043231; C:GO:0005829; [illegible]	F:phosphatidylinositol 3-kinase binding; P:insulin receptor signaling pathw[illegible]
Acrdi.XM_015898368.1/1-2058 | Monca.augustus.g68152.t1/1-3210	0.222062	0.211606	Inactive tyrosine- kinase 7	13	P:GO:0007169; P:GO:0038083; P:GO:0009653; F:GO:0005102; [illegible]	P:transmembrane receptor protein tyrosine kinase signaling pathway; P:pe[illegible]
Acrdi.XM_015906952.1/1-1065 | Monca.augustus.g49141.t1/1-1215	0.253406	0.211596	PREDICTED: uncharacterized protein LOC107341524 isoform X2			
Acrdi.XM_015909098.1/1-831 | Monca.augustus.g67733.t1/1-963	0.30405	0.211561	neural cell adhesion molecule 1-like isoform X1	2	C:GO:0016020; C:GO:0016021	C:membrane; C:integral component of membrane
Acrdi.XM_015921659.1/1-945 | Monca.augustus.g55546.t1/1-900	0.591449	0.21154	PREDICTED: uncharacterized protein LOC107355139			
Acrdi.XM_015906374.1/1-2406 | Monca.augustus.g31955.t1/1-2415	0.195913	0.21151	serine threonine- kinase Nek3-like	4	F:GO:0005524; F:GO:0004702; C:GO:0005737; P:GO:0023014	F:ATP binding; F:signal transducer, downstream of receptor, with serine/th[illegible]
Acrdi.XM_015908450.1/1-1278 | Monca.augustus.g33945.t1/1-1263	0.273446	0.211489	MATH and LRR domain-containing PFE0570w-like			
Acrdi.XM_015918380.1/1-2046 | Monca.augustus.g22404.t1/1-1977	0.23398	0.211461	angiotensin-converting enzyme-like	1	F:GO:0070011	F:peptidase activity, acting on L-amino acid peptides
Acrdi.XM_015916466.1/1-660 | Monca.augustus.g31297.t1/1-999	0.194416	0.211443	hemK methyltransferase family member 1	3	F:GO:0005515; F:GO:0008168; P:GO:0032259	F:protein binding; F:methyltransferase activity; P:methylation
Acrdi.XM_015919482.1/1-1017 | Monca.augustus.g59639.t1/1-1020	0.238202	0.211421	adrenocorticotropic hormone receptor-like	9	F:GO:0004930; F:GO:0001588; P:GO:0001963; P:GO:0007186; [illegible]	F:G-protein coupled receptor activity; F:dopamine neurotransmitter recept[illegible]
Acrdi.XM_015906987.1/1-4581 | Monca.adi2mcaRNA16810_R0/1-5868	0.243488	0.21138	leucine-rich repeat-containing DDB_G0290503 isoform X4			
Acrdi.XM_015896542.1/1-669 | Monca.augustus.g65913.t1/1-672	0.308017	0.211348	transmembrane cell adhesion receptor mua-3-like	5	F:GO:0015165; F:GO:0005509; C:GO:0016020; P:GO:0090481; [illegible]	F:pyrimidine nucleotide-sugar transmembrane transporter activity; F:calciu[illegible]
Acrdi.XM_015914380.1/1-1059 | Monca.augustus.g67303.t1/1-1155	0.174303	0.21123	palmitoyltransferase ZDHHC6-like	7	F:GO:0016746; F:GO:0019706; F:GO:0008270; F:GO:0046872; [illegible]	F:transferase activity, transferring acyl groups; F:protein-cysteine S-palmit[illegible]
Acrdi.XM_015916104.1/1-822 | Monca.adi2mcaRNA26847_R8/1-810	0.0606335	0.21121	RNA-directed DNA polymerase from transposon BS			
Acrdi.XM_015903760.1/1-1296 | Monca.adi2mcaRNA13200_R1/1-1302	0.0540902	0.211082	nuclease HARBI1			
Acrdi.XM_015918795.1/1-2202 | Monca.augustus.g43808.t1/1-2829	0.268951	0.211056	remodeling and spacing factor 1	8	P:GO:1902680; C:GO:0044428; P:GO:0006338; F:GO:0005515; [illegible]	P:positive regulation of RNA biosynthetic process; C:nuclear part; P:chroma[illegible]
Acrdi.XM_015913487.1/1-1164 | Monca.augustus.g60180.t1/1-1161	0.302842	0.211025	G patch domain and ankyrin repeat-containing 1	1	F:GO:0003676	F:nucleic acid binding
Acrdi.XM_015911257.1/1-657 | Monca.augustus.g48131.t1/1-894	0.25348	0.210991	intestinal mucin			
Acrdi.XM_015911764.1/1-492 | Monca.augustus.g7410.t1/1-828	0.0757304	0.210948	PREDICTED: uncharacterized protein K02A2.6-like			
Acrdi.XM_015910393.1/1-1350 | Monca.augustus.g10415.t1/1-1377	0.226063	0.210888	brain-specific angiogenesis inhibitor 1-associated 2-like isoform X2	1	P:GO:0007009	P:plasma membrane organization
Acrdi.XM_015896198.1/1-1134 | Monca.augustus.g23020.t1/1-2328	0.216736	0.210886	proclotting enzyme	1	F:GO:0008233	F:peptidase activity
Acrdi.XM_015907405.1/1-5367 | Monca.augustus.g64312.t1/1-2871	0.25419	0.210843	tudor domain-containing 1 isoform X1	3	P:GO:0007276; P:GO:0044763; P:GO:0044767	P:gamete generation; P:single-organism cellular process; P:single-organis[illegible]
Acrdi.XM_015901888.1/1-609 | Monca.augustus.g7724.t1/1-735	0.167516	0.210785	fibroblast growth factor	5	P:GO:0048522; P:GO:0048731; C:GO:0005737; P:GO:0070121; [illegible]	P:positive regulation of cellular process; P:system development; C:cytoplas[illegible]
Acrdi.XM_015900423.1/1-1782 | Monca.augustus.g49251.t1/1-1917	0.229416	0.210768	E3 ubiquitin- ligase RNF14-like	1	P:GO:0009987	P:cellular process
Acrdi.XM_015906677.1/1-3054 | Monca.augustus.g60587.t1/1-3345	0.24673	0.210732	DNA repair REV1 isoform X1	3	F:GO:0016779; P:GO:0006259; P:GO:0006974	F:nucleotidyltransferase activity; P:DNA metabolic process; P:cellular respo[illegible]
Acrdi.XM_015895942.1/1-1458 | Monca.augustus.g36101.t1/1-1458	0.291744	0.210724	RING finger 26	4	F:GO:0008270; F:GO:0046872; C:GO:0016020; C:GO:0016021	F:zinc ion binding; F:metal ion binding; C:membrane; C:integral component[illegible]
Acrdi.XM_015911325.1/1-1995 | Monca.adi2mcaRNA21567_R0/1-2217	0.228994	0.210701	Src kinase-associated phospho 2	7	F:GO:0005070; C:GO:0005737; P:GO:0002757; P:GO:0016310; [illegible]	F:SH3/SH2 adaptor activity; C:cytoplasm; P:immune response-activating si[illegible]
Acrdi.XM_015913946.1/1-3978 | Monca.augustus.g59707.t1/1-3891	0.191521	0.210697	WD repeat-containing 17-like	1	F:GO:0005509	F:calcium ion binding
Acrdi.XM_015915678.1/1-972 | Monca.augustus.g37364.t1/1-1677	0.176866	0.210654	Elongator complex 4	1	C:GO:0033588	C:Elongator holoenzyme complex
Acrdi.XM_015915051.1/1-1155 | Monca.augustus.g58141.t1/1-1152	0.15165	0.210576	Sesquipedalian-1	2	F:GO:0003676; F:GO:0046983	F:nucleic acid binding; F:protein dimerization activity
Acrdi.XM_015908656.1/1-762 | Monca.augustus.g53869.t1/1-753	0.288183	0.210568	Adhesion G- coupled receptor D1	6	F:GO:0004930; F:GO:0004888; P:GO:0007186; C:GO:0016020; [illegible]	F:G-protein coupled receptor activity; F:transmembrane signaling receptor[illegible]
Acrdi.XM_015912273.1/1-738 | Monca.augustus.g28373.t1/1-1296	0.0757475	0.210566	late secretory pathway AVL9-like			
Acrdi.XM_015905682.1/1-606 | Monca.augustus.g26828.t1/1-858	0.244152	0.210535	PREDICTED: uncharacterized protein LOC107340331 isoform X1			
Acrdi.XM_015898194.1/1-630 | Monca.augustus.g29223.t1/1-612	0.276334	0.21053	goosecoid	3	C:GO:0005634; P:GO:0006355; F:GO:0043565	C:nucleus; P:regulation of transcription, DNA-templated; F:sequence-speci[illegible]
Acrdi.XM_015901276.1/1-960 | Monca.augustus.g61814.t1/1-951	0.20094	0.210492	trace amine-associated receptor 9-like	3	F:GO:0004930; P:GO:0007154; P:GO:0044700	F:G-protein coupled receptor activity; P:cell communication; P:single organ[illegible]
Acrdi.XM_015917649.1/1-1203 | Monca.augustus.g44141.t1/1-1182	0.223231	0.210491	substance-K receptor-like	4	F:GO:0004930; P:GO:0007186; C:GO:0016021; C:GO:0005886	F:G-protein coupled receptor activity; P:G-protein coupled receptor signali[illegible]
Acrdi.XM_015904048.1/1-7380 | Monca.augustus.g32314.t1/1-7500	0.222907	0.21048	golgin subfamily B member 1-like isoform X1			
Acrdi.XM_015897686.1/1-699 | Monca.augustus.g46350.t1/1-933	0.236837	0.21047	HMG domain-containing 3			
Acrdi.XM_015892626.1/1-354 | Monca.augustus.g16419.t1/1-687	0.24371	0.210441	PREDICTED: uncharacterized protein LOC107327902			
Acrdi.XM_015898894.1/1-5442 | Monca.augustus.g35504.t1/1-5487	0.230891	0.2104	PREDICTED: trichohyalin-like			
Acrdi.XM_015915715.1/1-1161 | Monca.augustus.g69607.t1/1-1095	0.196874	0.210378	uncharacterized skeletal organic matrix 5-like			
Acrdi.XM_015917912.1/1-948 | Monca.augustus.g39693.t1/1-972	0.137197	0.210349	proline-rich transmembrane 4-like	2	C:GO:0016020; C:GO:0016021	C:membrane; C:integral component of membrane
Acrdi.XM_015900577.1/1-711 | Monca.adi2mcaRNA9790_R0/1-612	0.197271	0.210315	Catechol O-methyltransferase domain-containing	1	F:GO:0016740	F:transferase activity
Acrdi.XM_015917218.1/1-1806 | Monca.adi2mcaRNA28149_R0/1-1440	0.185977	0.210312	serine--pyruvate aminotransferase isoform X1	4	C:GO:0005777; P:GO:0019265; F:GO:0004760; F:GO:0008453	C:peroxisome; P:glycine biosynthetic process, by transamination of glyoxyl[illegible]
Acrdi.XM_015914496.1/1-780 | Monca.augustus.g27256.t1/1-771	0.231731	0.21027	PREDICTED: uncharacterized protein LOC107348456			
Acrdi.XM_015907727.1/1-732 | Monca.augustus.g10297.t1/1-654	0.375672	0.210263	ribosome-binding factor mitochondrial	1	P:GO:0006364	P:rRNA processing
Acrdi.XM_015894035.1/1-441 | Monca.augustus.g54155.t1/1-411	0.485159	0.210231	Sentrin-specific protease 3			
Acrdi.XM_015912134.1/1-528 | Monca.augustus.g9729.t1/1-531	0.182497	0.210169	PREDICTED: uncharacterized protein LOC107346341			
Acrdi.XM_015891759.1/1-1233 | Monca.adi2mcaRNA37618_R0/1-1299	0.277346	0.210153	calpain-D-like isoform X1	3	C:GO:0005737; P:GO:0006508; F:GO:0004198	C:cytoplasm; P:proteolysis; F:calcium-dependent cysteine-type endopeptid[illegible]
Acrdi.XM_015900593.1/1-8346 | Monca.augustus.g28807.t1/1-9048	0.265636	0.210147	DNA polymerase zeta catalytic subunit	8	F:GO:0003676; F:GO:0003887; F:GO:0005515; P:GO:0006261; [illegible]	F:nucleic acid binding; F:DNA-directed DNA polymerase activity; F:protein [illegible]
Acrdi.XM_015897444.1/1-1497 | Monca.augustus.g35247.t1/1-1698	0.165521	0.210113	rab11 family-interacting 4	1	F:GO:0005509	F:calcium ion binding
Acrdi.XM_015915770.1/1-735 | Monca.augustus.g43232.t1/1-747	0.125898	0.210042	low choriolytic enzyme	1	F:GO:0016787	F:hydrolase activity
Acrdi.XM_015924854.1/1-309 | Monca.augustus.g40424.t1/1-549	0.226572	0.210039	UPF0176 stu0548-like	3	C:GO:0016021; P:GO:0006810; F:GO:0005215	C:integral component of membrane; P:transport; F:transporter activity
Acrdi.XM_015892295.1/1-1173 | Monca.augustus.g57119.t1/1-1164	0.253932	0.210023	contactin-associated like 5-1-like isoform X1			
Acrdi.XM_015897281.1/1-435 | Monca.augustus.g51746.t1/1-3399	0.172825	0.209846	PREDICTED: uncharacterized protein LOC107332541			
Acrdi.XM_015903042.1/1-1821 | Monca.adi2mcaRNA12432_R0/1-1608	0.238686	0.209771	alpha-adducin-like isoform X1			
Acrdi.XM_015898414.1/1-1812 | Monca.augustus.g68149.t1/1-1929	0.279384	0.209753	matrix-remodeling-associated 5-like			
Acrdi.XM_015907987.1/1-3819 | Monca.augustus.g31011.t1/1-5865	0.217964	0.209679	adhesive plaque matrix 2-	1	F:GO:0005509	F:calcium ion binding
Acrdi.XM_015896964.1/1-1059 | Monca.adi2mcaRNA5833_R0/1-1017	0.282409	0.209561	Cat eye syndrome critical region 6-like	2	C:GO:0016020; C:GO:0016021	C:membrane; C:integral component of membrane
Acrdi.XM_015911822.1/1-1320 | Monca.augustus.g39598.t1/1-1305	0.239739	0.209526	bromodomain-containing 4-like isoform X1	2	C:GO:0016020; C:GO:0016021	C:membrane; C:integral component of membrane
Acrdi.XM_015902997.1/1-2010 | Monca.adi2mcaRNA12356_R2/1-2034	0.138046	0.20942	TPA: transposase domain-containing			
Acrdi.XM_015916775.1/1-780 | Monca.augustus.g62795.t1/1-783	0.191406	0.209398	PREDICTED: uncharacterized protein LOC107350543			
Acrdi.XM_015901496.1/1-726 | Monca.augustus.g4071.t1/1-798	0.623219	0.209316	PREDICTED: uncharacterized protein LOC107336420			
Acrdi.XM_015917518.1/1-1062 | Monca.augustus.g22424.t1/1-1065	0.121572	0.209276	NADPH-dependent aldehyde reductase ARI1-like isoform X1	2	F:GO:0016616; P:GO:0044710	F:oxidoreductase activity, acting on the CH-OH group of donors, NAD or NA[illegible]
Acrdi.XM_015898404.1/1-717 | Monca.augustus.g58539.t1/1-717	0.273413	0.20922	UDP-glucuronosyltransferase 2B13-like	5	F:GO:0008194; P:GO:0052696; F:GO:0016758; C:GO:0043231; [illegible]	F:UDP-glycosyltransferase activity; P:flavonoid glucuronidation; F:transfera[illegible]
Acrdi.XM_015924138.1/1-1086 | Monca.augustus.g39219.t1/1-1110	0.162387	0.209179	PREDICTED: protein FAM26E-like	6	C:GO:0005887; P:GO:0034220; F:GO:0005261; C:GO:0016020; [illegible]	C:integral component of plasma membrane; P:ion transmembrane transpo[illegible]
Acrdi.XM_015901266.1/1-1209 | Monca.augustus.g67930.t1/1-2871	0.207358	0.209173	probable G- coupled receptor	6	F:GO:0004930; F:GO:0004888; P:GO:0007186; C:GO:0016020; [illegible]	F:G-protein coupled receptor activity; F:transmembrane signaling receptor[illegible]
Acrdi.XM_015902140.1/1-651 | Monca.augustus.g45391.t1/1-651	0.179388	0.20913	hematopoietic prostaglandin D synthase-like	3	F:GO:0004667; F:GO:0016740; F:GO:0016853	F:prostaglandin-D synthase activity; F:transferase activity; F:isomerase act[illegible]
Acrdi.XM_015903383.1/1-957 | Monca.augustus.g36038.t1/1-981	0.131826	0.209084	NF-kappa-B-repressing factor-like	1	F:GO:0003676	F:nucleic acid binding
Acrdi.XM_015923947.1/1-1155 | Monca.augustus.g34516.t1/1-1164	0.206487	0.208961	neuropeptide Y receptor type 2-like	1	P:GO:0050896	P:response to stimulus
Acrdi.XM_015905225.1/1-654 | Monca.adi2mcaRNA14833_R0/1-543	0.26545	0.20891	insulin receptor substrate 1-B-like	1	F:GO:0005158	F:insulin receptor binding
Acrdi.XM_015904526.1/1-1290 | Monca.augustus.g6293.t1/1-1296	0.220984	0.20891	splicing factor u2af 65 kda	2	F:GO:0008270; F:GO:0016814	F:zinc ion binding; F:hydrolase activity, acting on carbon-nitrogen (but not [illegible]
Acrdi.XM_015922280.1/1-3291 | Monca.augustus.g27616.t1/1-3285	0.210848	0.208896	metabotropic glutamate receptor 3-like	4	F:GO:0004888; P:GO:0007186; C:GO:0016021; C:GO:0005886	F:transmembrane signaling receptor activity; P:G-protein coupled receptor[illegible]
Acrdi.XM_015900905.1/1-432 | Monca.augustus.g27851.t1/1-582	0.164384	0.20889	coactosin	2	F:GO:0003779; C:GO:0005622	F:actin binding; C:intracellular
Acrdi.XM_015915762.1/1-1662 | Monca.augustus.g43231.t1/1-1740	0.199025	0.20885	PREDICTED: uncharacterized protein LOC107349582			
Acrdi.XM_015923372.1/1-1827 | Monca.augustus.g31219.t1/1-2139	0.281879	0.208846	nibrin isoform X1	6	C:GO:0044428; P:GO:0050794; P:GO:0051276; P:GO:0006259; [illegible]	C:nuclear part; P:regulation of cellular process; P:chromosome organizatio[illegible]
Acrdi.XM_015923810.1/1-870 | Monca.augustus.g23685.t1/1-2310	0.395959	0.208826	deoxyribonuclease TATDN2	1	F:GO:0008270	F:zinc ion binding
Acrdi.XM_015907686.1/1-1188 | Monca.augustus.g29928.t1/1-1482	0.232153	0.208782	Shroom2	5	F:GO:0051015; C:GO:0005912; P:GO:0090251; P:GO:0030950; [illegible]	F:actin filament binding; C:adherens junction; P:protein localization involve[illegible]
Acrdi.XM_015919722.1/1-1248 | Monca.augustus.g8116.t1/1-1803	0.233457	0.208767	dorsal-ventral patterning tolloid	1	P:GO:0048731	P:system development
Acrdi.XM_015895009.1/1-1272 | Monca.adi2mcaRNA41381_R0/1-1119	0.194253	0.208767	soma ferritin-like	6	P:GO:0006879; F:GO:0008199; C:GO:0005737; P:GO:0006826; [illegible]	P:cellular iron ion homeostasis; F:ferric iron binding; C:cytoplasm; P:iron io[illegible]
Acrdi.XM_015914704.1/1-1773 | Monca.adi2mcaRNA2591_R0/1-1734	0.186633	0.208747	fibulin-2 isoform X1	1	F:GO:0005509	F:calcium ion binding
Acrdi.XM_015919556.1/1-396 | Monca.augustus.g29625.t1/1-807	0.230011	0.20871	WW domain-binding 2-like	5	C:GO:0005634; C:GO:0005737; P:GO:0045893; P:GO:0045572; [illegible]	C:nucleus; C:cytoplasm; P:positive regulation of transcription, DNA-templa[illegible]
Acrdi.XM_015893192.1/1-564 | Monca.augustus.g42758.t1/1-447	0.451111	0.208683	multidrug resistance-associated 4-like	2	F:GO:0019992; P:GO:0007268	F:diacylglycerol binding; P:chemical synaptic transmission
Acrdi.XM_015925035.1/1-2460 | Monca.augustus.g61141.t1/1-2733	0.131436	0.208643	low-density lipo receptor-related 4	1	F:GO:0005509	F:calcium ion binding
Acrdi.XM_015917986.1/1-5001 | Monca.augustus.g45179.t1/1-4836	0.241266	0.208557	zinc finger 665-like			
Acrdi.XM_015921998.1/1-1569 | Monca.augustus.g60066.t1/1-1395	0.170797	0.208554	Charged multivesicular body 7	3	C:GO:0043229; P:GO:0006810; C:GO:0043227	C:intracellular organelle; P:transport; C:membrane-bounded organelle
Acrdi.XM_015903508.1/1-1362 | Monca.augustus.g45512.t1/1-1461	0.139693	0.208545	Phospholipase D3	1	F:GO:0003824	F:catalytic activity
Acrdi.XM_015899244.1/1-735 | Monca.augustus.g47507.t1/1-873	0.252305	0.208539	Centromere	2	P:GO:0034508; C:GO:0000776	P:centromere complex assembly; C:kinetochore
Acrdi.XM_015917098.1/1-2439 | Monca.augustus.g71378.t1/1-2454	0.225991	0.208478	skeletal receptor tyrosine- kinase isoform X2	3	F:GO:0005524; F:GO:0004672; P:GO:0006468	F:ATP binding; F:protein kinase activity; P:protein phosphorylation
Acrdi.XM_015893189.1/1-444 | Monca.adi2mcaRNA39243_R1/1-426	0.63017	0.208404	PREDICTED: uncharacterized protein LOC107328458			
Acrdi.XM_015908223.1/1-1299 | Monca.augustus.g10927.t1/1-1293	0.663718	0.20836	type III restriction res subunit	7	F:GO:0000166; F:GO:0003677; P:GO:0090305; F:GO:0005524; [illegible]	F:nucleotide binding; F:DNA binding; P:nucleic acid phosphodiester bond h[illegible]
Acrdi.XM_015905377.1/1-1032 | Monca.augustus.g61121.t1/1-1014	0.193994	0.208329	bone morphogenetic 2-like	2	P:GO:0048856; P:GO:0044767	P:anatomical structure development; P:single-organism developmental pr[illegible]
Acrdi.XM_015901878.1/1-897 | Monca.augustus.g36354.t1/1-885	0.153874	0.208317	E3 ubiquitin- ligase NRDP1-like	7	F:GO:0003676; F:GO:0004842; F:GO:0008270; F:GO:0046872; [illegible]	F:nucleic acid binding; F:ubiquitin-protein transferase activity; F:zinc ion bi[illegible]
Acrdi.XM_015919013.1/1-1353 | Monca.augustus.g32013.t1/1-1674	0.181752	0.208313	Meckel syndrome type 1			
Acrdi.XM_015901556.1/1-1878 | Monca.augustus.g55987.t1/1-1893	0.191332	0.208287	chromosome 1 open reading frame isoform CRA_a	1	F:GO:0005509	F:calcium ion binding
Acrdi.XM_015904814.1/1-804 | Monca.augustus.g29902.t1/1-1434	0.282545	0.208233	probable flavin-containing monoamine oxidase A	4	F:GO:0016491; C:GO:0016020; C:GO:0016021; P:GO:0055114	F:oxidoreductase activity; C:membrane; C:integral component of membra[illegible]
Acrdi.XM_015919478.1/1-705 | Monca.augustus.g61029.t1/1-690	0.161661	0.208198	glutamyl aminopeptidase	1	C:GO:0016020	C:membrane
Acrdi.XM_015921110.1/1-408 | Monca.augustus.g48105.t1/1-393	0.308798	0.208186	PREDICTED: uncharacterized protein LOC107354625			
Acrdi.XM_015905885.1/1-2004 | Monca.augustus.g58494.t1/1-2346	0.251989	0.208177	eukaryotic translation initiation factor 4B isoform X1	2	F:GO:0003723; P:GO:0009987	F:RNA binding; P:cellular process
Acrdi.XM_015900875.1/1-3297 | Monca.adi2mcaRNA10172_R0/1-3216	0.0561685	0.208145	Retrovirus-related Pol poly from transposon			
Acrdi.XM_015899327.1/1-1551 | Monca.augustus.g64424.t1/1-1545	0.197456	0.208047	Proton-coupled folate transporter	3	P:GO:0055085; C:GO:0016020; C:GO:0016021	P:transmembrane transport; C:membrane; C:integral component of memb[illegible]
Acrdi.XM_015912275.1/1-855 | Monca.augustus.g28343.t1/1-846	0.21393	0.208025	PREDICTED: uncharacterized protein LOC107346479 isoform X1			
Acrdi.XM_015910969.1/1-465 | Monca.augustus.g3080.t1/1-468	0.201649	0.208025	PREDICTED: uncharacterized protein LOC107345266			
Acrdi.XM_015891719.1/1-732 | Monca.augustus.g52056.t1/1-1818	0.220759	0.207995	alpha carbonic anhydrase 1			
Acrdi.XM_015909836.1/1-1596 | Monca.augustus.g40673.t1/1-1596	0.231181	0.207957	Xaa-Pro aminopeptidase 1	5	F:GO:0004314; F:GO:0016746; C:GO:0016020; C:GO:0016021; [illegible]	F:[acyl-carrier-protein] S-malonyltransferase activity; F:transferase activity[illegible]
Acrdi.XM_015901772.1/1-1152 | Monca.augustus.g61692.t1/1-1152	0.227401	0.207909	prosaposin isoform X1	1	C:GO:0005737	C:cytoplasm
Acrdi.XM_015898542.1/1-1734 | Monca.augustus.g33983.t1/1-1956	0.227351	0.207841	histone-lysine N-methyltransferase PRDM6	7	P:GO:0043170; C:GO:0005634; F:GO:0042803; P:GO:0045892; [illegible]	P:macromolecule metabolic process; C:nucleus; F:protein homodimerizatio[illegible]
Acrdi.XM_015910791.1/1-2052 | Monca.augustus.g48002.t1/1-2076	0.209998	0.207834	E3 ubiquitin- ligase RNF216	3	F:GO:0046872; P:GO:0021549; P:GO:0043010	F:metal ion binding; P:cerebellum development; P:camera-type eye develo[illegible]
Acrdi.XM_015917336.1/1-1407 | Monca.augustus.g25543.t1/1-2142	0.226089	0.207819	Transcription initiation factor TFIID subunit 3	5	F:GO:0005515; P:GO:0006355; P:GO:0006366; P:GO:0048519; [illegible]	F:protein binding; P:regulation of transcription, DNA-templated; P:transcrip[illegible]
Acrdi.XM_015905658.1/1-1101 | Monca.adi2mcaRNA15356_R0/1-1815	0.0662343	0.207736	PREDICTED: uncharacterized protein LOC107340308			
Acrdi.XM_015920302.1/1-540 | Monca.augustus.g7949.t1/1-558	0.168548	0.207691	macrophage mannose receptor 1-like			
Acrdi.XM_015901189.1/1-1722 | Monca.augustus.g58579.t1/1-1707	0.237955	0.207675	vacuolar segregation PEP7-like	4	P:GO:0016192; C:GO:0005737; F:GO:0005488; P:GO:0009987	P:vesicle-mediated transport; C:cytoplasm; F:binding; P:cellular process
Acrdi.XM_015911095.1/1-303 | Monca.adi2mcaRNA21296_R6/1-333	0.714252	0.207659	PREDICTED: uncharacterized protein LOC107345394			
Acrdi.XM_015923932.1/1-1044 | Monca.augustus.g18227.t1/1-1041	0.230039	0.207651	Cytidine and dCMP deaminase domain-containing 1	1	F:GO:0016788	F:hydrolase activity, acting on ester bonds
Acrdi.XM_015902925.1/1-1275 | Monca.augustus.g41323.t1/1-1473	0.238662	0.207637	Xyloside xylosyltransferase 1	4	F:GO:0016757; C:GO:0016020; C:GO:0016021; F:GO:0016740	F:transferase activity, transferring glycosyl groups; C:membrane; C:integra[illegible]
Acrdi.XM_015914831.1/1-624 | Monca.augustus.g33304.t1/1-627	0.193454	0.207629	calcium-activated potassium channel subunit beta-4	12	F:GO:0015269; P:GO:0006813; F:GO:0005513; C:GO:0005887; [illegible]	F:calcium-activated potassium channel activity; P:potassium ion transport;[illegible]
Acrdi.XM_015920518.1/1-2895 | Monca.augustus.g25710.t1/1-2880	0.211267	0.207585	centromere-associated E-like			
Acrdi.XM_015893905.1/1-579 | Monca.adi2mcaRNA40052_R5/1-780	0.0658958	0.207553	RNA-directed DNA polymerase from mobile element jockey-like			
Acrdi.XM_015907067.1/1-636 | Monca.adi2mcaRNA16885_R0/1-639	0.246934	0.207513	ammonium transporter	1	P:GO:0006810	P:transport
Acrdi.XM_015892329.1/1-1236 | Monca.augustus.g5372.t1/1-1239	0.232537	0.207503	transcription factor 19-like			
Acrdi.XM_015910950.1/1-2340 | Monca.augustus.g63046.t1/1-2613	0.229001	0.20746	glutamate-rich 6-like			
Acrdi.XM_015921699.1/1-1131 | Monca.augustus.g43103.t1/1-1128	0.182926	0.207429	prolactin-releasing peptide receptor-like	3	F:GO:0004930; P:GO:0007165; C:GO:0016020	F:G-protein coupled receptor activity; P:signal transduction; C:membrane
Acrdi.XM_015908543.1/1-837 | Monca.augustus.g65971.t1/1-801	0.283267	0.207348	Leukocyte receptor cluster member 1			
Acrdi.XM_015908669.1/1-789 | Monca.augustus.g53877.t1/1-1272	0.244294	0.207328	PREDICTED: uncharacterized protein LOC107343117			
Acrdi.XM_015896183.1/1-1212 | Monca.augustus.g33744.t1/1-1221	0.229942	0.207289	F-box only 31	3	P:GO:0050794; C:GO:0044464; P:GO:0044763	P:regulation of cellular process; C:cell part; P:single-organism cellular proc[illegible]
Acrdi.XM_015913557.1/1-3456 | Monca.augustus.g22317.t1/1-4914	0.218972	0.207207	capicua homolog isoform X1			
Acrdi.XM_015901843.1/1-1350 | Monca.augustus.g19636.t1/1-1440	0.186223	0.207138	TATA box-binding -associated factor RNA polymerase I subunit A isofo	1	C:GO:0043231	C:intracellular membrane-bounded organelle
Acrdi.XM_015914321.1/1-1821 | Monca.adi2mcaRNA24858_R2/1-1812	0.181474	0.207092	PREDICTED: uncharacterized protein LOC107348297 isoform X2			
Acrdi.XM_015914530.1/1-2064 | Monca.adi2mcaRNA25130_R0/1-1731	0.153611	0.207089	receptor-type tyrosine- phosphatase delta-	3	P:GO:0006464; P:GO:0016311; F:GO:0016787	P:cellular protein modification process; P:dephosphorylation; F:hydrolase a[illegible]
Acrdi.XM_015896337.1/1-750 | Monca.augustus.g28085.t1/1-750	0.252449	0.207082	coiled-coil domain-containing 112-like			
Acrdi.XM_015909544.1/1-615 | Monca.augustus.g41989.t1/1-588	0.351881	0.207056	Sodium-dependent glucose transporter 1	3	P:GO:0055085; C:GO:0016020; C:GO:0016021	P:transmembrane transport; C:membrane; C:integral component of memb[illegible]
Acrdi.XM_015909847.1/1-816 | Monca.augustus.g47091.t1/1-1104	0.152941	0.206988	NADP-dependent oxidoreductase domain-containing 1-like	3	F:GO:0004735; P:GO:0006561; P:GO:0055114	F:pyrroline-5-carboxylate reductase activity; P:proline biosynthetic process;[illegible]
Acrdi.XM_015898929.1/1-2508 | Monca.augustus.g37953.t1/1-2589	0.223481	0.206917	SID1 transmembrane family member	4	F:GO:0051033; C:GO:0016020; C:GO:0016021; P:GO:0033227	F:RNA transmembrane transporter activity; C:membrane; C:integral comp[illegible]
Acrdi.XM_015909675.1/1-1356 | Monca.augustus.g44683.t1/1-2268	0.123732	0.206903	oxidation resistance 1-like isoform X1			
Acrdi.XM_015904515.1/1-1578 | Monca.adi2mcaRNA14031_R0/1-1545	0.171969	0.206863	PREDICTED: uncharacterized protein LOC107339252 isoform X1			
Acrdi.XM_015908467.1/1-897 | Monca.augustus.g33927.t1/1-1107	0.260996	0.206811	Survival motor neuron			
Acrdi.XM_015904475.1/1-1242 | Monca.augustus.g16845.t1/1-1359	0.227304	0.206743	major facilitator superfamily domain-containing 9-like	4	P:GO:0055085; C:GO:0016020; C:GO:0016021; F:GO:0005215	P:transmembrane transport; C:membrane; C:integral component of memb[illegible]
Acrdi.XM_015919383.1/1-1383 | Monca.adi2mcaRNA30550_R0/1-1374	0.258606	0.206693	V-type proton ATPase subunit S1-like	6	P:GO:0015991; C:GO:0016020; C:GO:0016021; F:GO:0046961; [illegible]	P:ATP hydrolysis coupled proton transport; C:membrane; C:integral compo[illegible]
Acrdi.XM_015914572.1/1-735 | Monca.augustus.g57109.t1/1-654	0.141349	0.206689	Very-long-chain (3R)-3-hydroxyacyl- dehydratase 2	2	C:GO:0016020; C:GO:0016021	C:membrane; C:integral component of membrane
Acrdi.XM_015918725.1/1-1641 | Monca.augustus.g53967.t1/1-1647	0.21943	0.206674	Serine threonine- kinase mitochondrial	5	F:GO:0005524; F:GO:0004672; P:GO:0016310; P:GO:0006468; [illegible]	F:ATP binding; F:protein kinase activity; P:phosphorylation; P:protein phosp[illegible]
Acrdi.XM_015918563.1/1-1167 | Monca.augustus.g20715.t1/1-1206	0.266327	0.206651	leukocyte tyrosine kinase receptor-like			
Acrdi.XM_015915165.1/1-657 | Monca.augustus.g39756.t1/1-621	0.133646	0.206564	metallo-beta-lactamase domain-containing 1			
Acrdi.XM_015905446.1/1-858 | Monca.augustus.g33963.t1/1-1095	0.292391	0.206545	Lipid storage droplets surface-binding 2			
Acrdi.XM_015904522.1/1-639 | Monca.augustus.g33567.t1/1-1374	0.20666	0.206529	PR domain zinc finger 13	2	F:GO:0003676; F:GO:0046872	F:nucleic acid binding; F:metal ion binding
Acrdi.XM_015892011.1/1-618 | Monca.adi2mcaRNA37898_R0/1-411	0.343515	0.206523	transcription factor Adf-1-like			
Acrdi.XM_015910177.1/1-1149 | Monca.adi2mcaRNA20313_R5/1-1434	0.0907522	0.206371	tRNA (adenine(58)-N(1))-methyltransferase non-catalytic subunit TRM6-like			
Acrdi.XM_015902574.1/1-864 | Monca.augustus.g39920.t1/1-846	0.255601	0.206345	PREDICTED: uncharacterized protein LOC107337422	2	C:GO:0016020; C:GO:0016021	C:membrane; C:integral component of membrane
Acrdi.XM_015895889.1/1-1923 | Monca.adi2mcaRNA4724_R1/1-1284	0.0912628	0.206342	Alpha-N-acetylgalactosaminidase	2	F:GO:0004553; P:GO:0071704	F:hydrolase activity, hydrolyzing O-glycosyl compounds; P:organic substan[illegible]
Acrdi.XM_015901627.1/1-2370 | Monca.adi2mcaRNA10905_R4/1-2406	0.0489452	0.206327	PREDICTED: uncharacterized protein LOC107336557			
Acrdi.XM_015909753.1/1-2070 | Monca.augustus.g65482.t1/1-2061	0.275629	0.206314	PREDICTED: uncharacterized protein LOC107343818			
Acrdi.XM_015903387.1/1-966 | Monca.augustus.g36031.t1/1-960	0.17843	0.206268	probable G- coupled receptor No9	2	P:GO:0007154; P:GO:0044700	P:cell communication; P:single organism signaling
Acrdi.XM_015915886.1/1-1272 | Monca.augustus.g39009.t1/1-1281	0.154095	0.206213	DDI-3-like			
Acrdi.XM_015912633.1/1-2718 | Monca.augustus.g53954.t1/1-2982	0.243097	0.206159	Discoidin domain-containing receptor	2	F:GO:0004672; P:GO:0009987	F:protein kinase activity; P:cellular process
Acrdi.XM_015925263.1/1-576 | Monca.augustus.g12519.t1/1-471	0.181714	0.206151	PREDICTED: uncharacterized protein LOC107358669			
Acrdi.XM_015895502.1/1-1263 | Monca.augustus.g25937.t1/1-1323	0.197429	0.2061	Polycomb complex BMI-1	6	C:GO:0005634; P:GO:0021549; P:GO:0010468; P:GO:0035701; [illegible]	C:nucleus; P:cerebellum development; P:regulation of gene expression; P:h[illegible]
Acrdi.XM_015905535.1/1-933 | Monca.augustus.g28244.t1/1-930	0.181074	0.20609	octopamine receptor-like	3	F:GO:0004930; P:GO:0007154; P:GO:0044700	F:G-protein coupled receptor activity; P:cell communication; P:single organ[illegible]
Acrdi.XM_015917482.1/1-1326 | Monca.augustus.g13816.t1/1-1449	0.127501	0.206	PREDICTED: uncharacterized protein LOC107351186	2	C:GO:0016020; C:GO:0016021	C:membrane; C:integral component of membrane
Acrdi.XM_015917638.1/1-2610 | Monca.augustus.g44147.t1/1-6636	0.18962	0.205955	Zinc finger C3H1 domain-containing	1	P:GO:0006396	P:RNA processing
Acrdi.XM_015895112.1/1-1017 | Monca.augustus.g13695.t1/1-990	0.153421	0.205946	somatostatin receptor type 1-like	3	P:GO:0007154; P:GO:0044700; F:GO:0004871	P:cell communication; P:single organism signaling; F:signal transducer acti[illegible]
Acrdi.XM_015924547.1/1-1341 | Monca.augustus.g26875.t1/1-1587	0.250877	0.205944	Lamin-B receptor	4	C:GO:0005634; C:GO:0016021; C:GO:0012505; C:GO:0044446	C:nucleus; C:integral component of membrane; C:endomembrane system;[illegible]
Acrdi.XM_015914966.1/1-558 | Monca.augustus.g42429.t1/1-612	0.208923	0.205873	transcription factor 15-like	1	F:GO:0046983	F:protein dimerization activity
Acrdi.XM_015897119.1/1-10143 | Monca.augustus.g55685.t1/1-11910	0.254713	0.20586	aminomethyltransferase domain			
Acrdi.XM_015893811.1/1-2220 | Monca.augustus.g50546.t1/1-2493	0.232942	0.20584	X-ray radiation resistance-associated 1			
Acrdi.XM_015896055.1/1-1005 | Monca.augustus.g70750.t1/1-912	0.140596	0.20582	deoxyhypusine hydroxylase	4	P:GO:0008152; C:GO:0044424; F:GO:0004497; P:GO:0009987	P:metabolic process; C:intracellular part; F:monooxygenase activity; P:cellu[illegible]
Acrdi.XM_015921976.1/1-1146 | Monca.augustus.g60064.t1/1-1110	0.175168	0.205805	ubiquitin carboxyl-terminal hydrolase 12	3	P:GO:0006508; P:GO:0044267; F:GO:0036459	P:proteolysis; P:cellular protein metabolic process; F:thiol-dependent ubiqu[illegible]
Acrdi.XM_015897440.1/1-4899 | Monca.augustus.g2238.t1/1-6405	0.195398	0.205792	Transcriptional regulator ATRX	1	F:GO:0005524	F:ATP binding

Acrdi.XM_015894620.1/1-1011	Monca.augustus.g17481.t1/1-1047	0.185986	0.20578	somatostatin receptor type 1-like	3	P:GO:0007154; P:GO:0044700; F:GO:0004871	P:cell communication; P:single organism signaling; F:signal transducer activity		
Acrdi.XM_015899626.1/1-11670	Monca.augustus.g50130.t1/1-14778	0.293459	0.205721	E3 ubiquitin- ligase RNF213	3	F:GO:0000166; F:GO:0016887; F:GO:0005524	F:nucleotide binding; F:ATPase activity; F:ATP binding		
Acrdi.XM_015896930.1/1-1143	Monca.augustus.g28891.t1/1-1038	0.125198	0.205647	G- coupled receptor 161-like isoform X1	5	F:GO:0001588; P:GO:0001963; P:GO:0007186; F:GO:0035240;	F:dopamine neurotransmitter receptor activity, coupled via Gs; P:synaptic transmission, dopaminergic; P:G-protein coupled receptor signaling pathway; F:dopamine binding; C:membrane		
Acrdi.XM_015907737.1/1-1524	Monca.augustus.g62581.t1/1-1323	0.181372	0.205644	RILP 1					
Acrdi.XM_015892650.1/1-3624	Monca.augustus.g51179.t1/1-5802	0.206451	0.205578	WD repeat-containing 87	3	F:GO:0000386; P:GO:0000350; C:GO:0071013	F:second spliceosomal transesterification activity; P:generation of catalytic spliceosome for second transesterification step; C:catalytic step 2 spliceosome		
Acrdi.XM_015915685.1/1-1032	Monca.augustus.g37349.t1/1-1023	0.15698	0.205565	retrograde of 51 kDa-like					
Acrdi.XM_015925002.1/1-1386	Monca.augustus.g53480.t1/1-2391	0.214613	0.205549	GRAM domain-containing 1B-like	1	C:GO:0016020	C:membrane		
Acrdi.XM_015925020.1/1-1134	Monca.adi2mcaRNA36911_R0/1-1179	0.0570691	0.205516	transient receptor potential cation channel subfamily A member 1-like					
Acrdi.XM_015900489.1/1-1215	Monca.augustus.g37043.t1/1-1512	0.266679	0.205515	Coiled-coil domain-containing 12					
Acrdi.XM_015908166.1/1-546	Monca.augustus.g36918.t1/1-642	0.226325	0.20551	membrane-anchored junction -like isoform X2					
Acrdi.XM_015904517.1/1-1716	Monca.augustus.g68203.t1/1-1734	0.272283	0.205396	adhesion G- coupled receptor D1 isoform X1	6	F:GO:0004930; F:GO:0004888; P:GO:0007186; C:GO:0016020;	F:G-protein coupled receptor activity; F:transmembrane signaling receptor activity; P:G-protein coupled receptor signaling pathway; C:membrane; C:integral component of membrane; P:cell surface receptor signaling pathway		
Acrdi.XM_015920310.1/1-2862	Monca.augustus.g24354.t1/1-9744	0.233145	0.205388	PREDICTED: uncharacterized protein LOC107353919					
Acrdi.XM_015910971.1/1-1014	Monca.augustus.g3087.t1/1-1017	0.237631	0.20538	8-oxo-dGDP phosphatase NUDT18-like	1	F:GO:0016787	F:hydrolase activity		
Acrdi.XM_015920856.1/1-1476	Monca.augustus.g30738.t1/1-3018	0.311978	0.205347	Vacuolar sorting-associated 13D	4	P:GO:0045053; C:GO:0019898; P:GO:0006623; C:GO:0005622	P:protein retention in Golgi apparatus; C:extrinsic component of membrane; P:protein targeting to vacuole; C:intracellular		
Acrdi.XM_015892455.1/1-2700	Monca.augustus.g61311.t1/1-3645	0.162827	0.205337	phosphatidylinositol phosphatase PTPRQ-like	8	P:GO:0006813; F:GO:0005249; P:GO:0071805; C:GO:0008076;	P:potassium ion transport; F:voltage-gated potassium channel activity; P:potassium ion transmembrane transport; C:voltage-gated potassium channel complex; C:membrane; C:integral component of membrane; P:ionotropic glutamate receptor signaling pathway; F:ionotropic glutamate receptor activity		
Acrdi.XM_015892174.1/1-693	Monca.adi2mcaRNA38085_R4/1-723	0.172628	0.205264	adhesion G -coupled receptor E1-like					
Acrdi.XM_015916160.1/1-2091	Monca.augustus.g12401.t1/1-3006	0.195475	0.205248	Amyloid beta A4 precursor -binding family B member 2					
Acrdi.XM_015892801.1/1-2748	Monca.augustus.g59502.t1/1-1452	0.28552	0.205208	C-type mannose receptor 2					
Acrdi.XM_015918905.1/1-11250	Monca.augustus.g48516.t1/1-12081	0.276933	0.205184	Polycystic kidney disease 1-like 2	3	P:GO:0050982; F:GO:0005262; C:GO:0016020	P:detection of mechanical stimulus; F:calcium channel activity; C:membrane		
Acrdi.XM_015904806.1/1-711	Monca.augustus.g49267.t1/1-852	0.230968	0.205084	probable 18S rRNA (guanine-N(7))-methyltransferase	4	P:GO:0008152; F:GO:0008168; F:GO:0016740; P:GO:0032259	P:metabolic process; F:methyltransferase activity; F:transferase activity; P:methylation		
Acrdi.XM_015892880.1/1-375	Monca.augustus.g71152.t1/1-393	0.278743	0.20505	Meiotic recombination SPO11	6	F:GO:0003677; F:GO:0005524; F:GO:0003824; P:GO:0000737;	F:DNA binding; F:ATP binding; F:catalytic activity; P:DNA catabolic process, endonucleolytic; C:chromosome; P:DNA metabolic process		
Acrdi.XM_015918169.1/1-3228	Monca.adi2mcaRNA29184_R0/1-3255	0.170072	0.20502	collagen alpha-1(II) chain isoform X1	7	P:GO:0065007; P:GO:0048568; P:GO:0044763; F:GO:0005488;	P:biological regulation; P:embryonic organ development; P:single-organism cellular process; F:binding; P:skeletal system morphogenesis; C:proteinaceous extracellular matrix; P:cartilage development		
Acrdi.XM_015922623.1/1-705	Monca.augustus.g32435.t1/1-720	0.235363	0.205012	pyridoxal phosphate phosphatase PHOSPHO2	1	F:GO:0016791	F:phosphatase activity	EC:3.1.3.41	4-nitrophenylphosphatase
Acrdi.XM_015905894.1/1-3120	Monca.adi2mcaRNA15625_R0/1-3528	0.205453	0.205012	multidrug resistance 1	8	F:GO:0000166; F:GO:0016887; F:GO:0005524; F:GO:0042626;	F:nucleotide binding; F:ATPase activity; F:ATP binding; F:ATPase activity, coupled to transmembrane movement of substances; P:transmembrane transport; C:membrane; C:integral component of membrane; P:transport		
Acrdi.XM_015892113.1/1-468	Monca.adi2mcaRNA38019_R0/1-489	0.26748	0.204986	epithelial discoidin domain-containing receptor 1-like					
Acrdi.XM_015915353.1/1-2871	Monca.augustus.g53736.t1/1-2889	0.247069	0.204962	PREDICTED: uncharacterized protein LOC107349235	3	F:GO:0005509; C:GO:0016020; P:GO:0007156	F:calcium ion binding; C:membrane; P:homophilic cell adhesion via plasma membrane adhesion molecules		
Acrdi.XM_015897892.1/1-1011	Monca.augustus.g23837.t1/1-1005	0.215196	0.204937	presenilins-associated rhomboid mitochondrial	1	P:GO:0050794	P:regulation of cellular process		
Acrdi.XM_015903454.1/1-567	Monca.augustus.g49065.t1/1-567	0.130065	0.204917	kinetochore Spc24-like					
Acrdi.XM_015912621.1/1-2289	Monca.augustus.g9556.t1/1-3870	0.231115	0.204886	YTH domain family 2	2	F:GO:0003723; F:GO:0046872	F:RNA binding; F:metal ion binding		
Acrdi.XM_015916202.1/1-1086	Monca.augustus.g5397.t1/1-1188	0.269142	0.204878	DNA-directed RNA polymerase III subunit RPC4	13	P:GO:0006383; F:GO:0003682; P:GO:0002376; C:GO:0005666;	P:transcription from RNA polymerase III promoter; F:chromatin binding; P:immune system process; C:DNA-directed RNA polymerase III complex; F:DNA binding; F:RNA polymerase III activity; C:nucleus; F:DNA-directed 5'-3' RNA polymerase activity; C:nuclear chromatin; P:positive regulation of innate immune response; P:positive regulation of interferon-beta production; P:innate immune response; P:defense response to virus		
Acrdi.XM_015903665.1/1-1023	Monca.augustus.g17911.t1/1-1347	0.25157	0.204761	spindle and kinetochore-associated 3					
Acrdi.XM_015905387.1/1-714	Monca.augustus.g43321.t1/1-720	0.269605	0.204748	PREDICTED: uncharacterized protein LOC107340044					
Acrdi.XM_015893158.1/1-333	Monca.augustus.g37066.t1/1-450	0.239805	0.204733	myophilin isoform X2	2	F:GO:0003779; P:GO:0031032	F:actin binding; P:actomyosin structure organization		
Acrdi.XM_015925095.1/1-2127	Monca.augustus.g4273.t1/1-2049	0.265355	0.204721	PREDICTED: uncharacterized protein LOC107358496	1	P:GO:0060828	P:regulation of canonical Wnt signaling pathway		
Acrdi.XM_015913837.1/1-984	Monca.augustus.g26432.t1/1-981	0.312783	0.204657	PREDICTED: uncharacterized protein LOC107347860					
Acrdi.XM_015917941.1/1-873	Monca.augustus.g62261.t1/1-2166	0.18621	0.204605	TRAF-type zinc finger domain-containing 1	3	F:GO:0008270; P:GO:0006915; P:GO:0035456	F:zinc ion binding; P:apoptotic process; P:response to interferon-beta		
Acrdi.XM_015912423.1/1-1200	Monca.adi2mcaRNA22735_R1/1-1122	0.272012	0.204596	lethal(3)malignant brain tumor 3 isoform X1	2	C:GO:0005634; P:GO:0006355	C:nucleus; P:regulation of transcription, DNA-templated		
Acrdi.XM_015911823.1/1-957	Monca.augustus.g39595.t1/1-954	0.161358	0.204527	histamine H2 receptor-like	2	F:GO:0004930; P:GO:0007165	F:G-protein coupled receptor activity; P:signal transduction		
Acrdi.XM_015913019.1/1-2085	Monca.augustus.g9115.t1/1-2091	0.183775	0.204462	trichohyalin-like isoform X1	4	F:GO:0000166; F:GO:0005524; F:GO:0004672; P:GO:0006468	F:nucleotide binding; F:ATP binding; F:protein kinase activity; P:protein phosphorylation		
Acrdi.XM_015897081.1/1-552	Monca.augustus.g4158.t1/1-549	0.174256	0.20444	Wnt-7b	1	P:GO:0044699	P:single-organism process		
Acrdi.XM_015905952.1/1-453	Monca.augustus.g58523.t1/1-675	0.207979	0.204419	ribonuclease	3	P:GO:0006508; P:GO:0006364; F:GO:0004222	P:proteolysis; P:rRNA processing; F:metalloendopeptidase activity		
Acrdi.XM_015922859.1/1-3708	Monca.adi2mcaRNA34399_R1/1-3225	0.202769	0.204416	Aminopeptidase O	8	F:GO:0008270; P:GO:0043171; C:GO:0005737; P:GO:0006508;	F:zinc ion binding; P:peptide catabolic process; C:cytoplasm; P:proteolysis; F:metalloaminopeptidase activity; F:peptide binding; C:nucleolus; C:plasma membrane	EC:3.4.11	Acting on peptide bonds (peptidases)
Acrdi.XM_015910943.1/1-4557	Monca.augustus.g3078.t1/1-5136	0.293108	0.204401	Nucleoside diphosphate kinase 7	1	P:GO:0006796	P:phosphate-containing compound metabolic process		
Acrdi.XM_015891803.1/1-993	Monca.augustus.g43713.t1/1-978	0.15981	0.204311	orexin receptor type 2-like	4	C:GO:0005887; F:GO:0004930; P:GO:0007186; P:GO:0007166	C:integral component of plasma membrane; F:G-protein coupled receptor activity; P:G-protein coupled receptor signaling pathway; P:cell surface receptor signaling pathway		
Acrdi.XM_015910339.1/1-1521	Monca.augustus.g36481.t1/1-1512	0.272271	0.204201	26S proteasome non-ATPase regulatory subunit 5	2	C:GO:0008540; P:GO:0070682	C:proteasome regulatory particle, base subcomplex; P:proteasome regulatory particle assembly		
Acrdi.XM_015916003.1/1-2052	Monca.augustus.g37741.t1/1-2421	0.198362	0.20417	E3 UFM1- ligase 1	1	F:GO:0016874	F:ligase activity		
Acrdi.XM_015897563.1/1-2658	Monca.augustus.g88.t1/1-3261	0.212058	0.204134	telomerase-binding EST1A isoform X2					
Acrdi.XM_015905479.1/1-585	Monca.adi2mcaRNA1588_R0/1-624	0.280823	0.204082	PREDICTED: uncharacterized protein LOC107340127					
Acrdi.XM_015924782.1/1-8277	Monca.adi2mcaRNA3762_R0/1-7269	0.230932	0.204009	Polycystic kidney disease 1-like 2	1	C:GO:0016020	C:membrane		
Acrdi.XM_015903490.1/1-2400	Monca.augustus.g49074.t1/1-2415	0.205764	0.204008	uncharacterized skeletal organic matrix 1-like					
Acrdi.XM_015895248.1/1-1149	Monca.augustus.g69375.t1/1-2115	0.189841	0.203876	transmembrane prolyl 4-hydroxylase-like	1	F:GO:0043167	F:ion binding		
Acrdi.XM_015924366.1/1-1044	Monca.augustus.g30192.t1/1-1218	0.130595	0.203843	secretory carrier-associated membrane 1 isoform X1	4	C:GO:0016020; P:GO:0015031; C:GO:0016021; F:GO:0003674	C:membrane; P:protein transport; C:integral component of membrane; F:molecular_function		
Acrdi.XM_015901462.1/1-1482	Monca.adi2mcaRNA10725_R0/1-1494	0.190728	0.203793	collagen alpha-2(I) chain isoform X1	6	F:GO:0030414; C:GO:0005581; C:GO:0005576; F:GO:0005201;	F:peptidase inhibitor activity; C:collagen trimer; C:extracellular region; F:extracellular matrix structural constituent; C:proteinaceous extracellular matrix; P:negative regulation of peptidase activity		
Acrdi.XM_015903408.1/1-966	Monca.augustus.g35985.t1/1-972	0.260884	0.203741	retinol retinaldehyde reductase	4	F:GO:0016491; C:GO:0016020; C:GO:0016021; P:GO:0055114	F:oxidoreductase activity; C:membrane; C:integral component of membrane; P:oxidation-reduction process		
Acrdi.XM_015898066.1/1-993	Monca.augustus.g29243.t1/1-969	0.174721	0.203736	acropin 1					
Acrdi.XM_015892237.1/1-459	Monca.adi2mcaRNA38152_R0/1-459	0.139455	0.203605	AIG2 family	2	P:GO:0006750; F:GO:0003839	P:glutathione biosynthetic process; F:gamma-glutamylcyclotransferase activity		
Acrdi.XM_015920595.1/1-738	Monca.augustus.g2770.t1/1-1083	0.10157	0.203579	nuclease HARBI1					
Acrdi.XM_015916499.1/1-618	Monca.augustus.g9033.t1/1-633	0.141643	0.203536	differentially expressed in FDCP 6 homolog					
Acrdi.XM_015905917.1/1-582	Monca.augustus.g58489.t1/1-3969	0.118977	0.203526	FYVE and coiled-coil domain-containing 1					
Acrdi.XM_015893143.1/1-1446	Monca.augustus.g15704.t1/1-1341	0.24535	0.203404	PREDICTED: uncharacterized protein LOC107328407	4	F:GO:0004888; C:GO:0016020; C:GO:0016021; P:GO:0007166	F:transmembrane signaling receptor activity; C:membrane; C:integral component of membrane; P:cell surface receptor signaling pathway		
Acrdi.XM_015897128.1/1-852	Monca.augustus.g55691.t1/1-840	0.184438	0.203395	keratin-associated 4-3-like					
Acrdi.XM_015916735.1/1-663	Monca.augustus.g37079.t1/1-663	0.299031	0.203385	PREDICTED: uncharacterized protein LOC107350510					
Acrdi.XM_015909968.1/1-1941	Monca.augustus.g6683.t1/1-1920	0.238948	0.203273	follicle-stimulating hormone receptor-like isoform X1	2	P:GO:0007165; F:GO:0004871	P:signal transduction; F:signal transducer activity		
Acrdi.XM_015904965.1/1-1164	Monca.augustus.g46461.t1/1-1146	0.188666	0.203261	galanin receptor type 1-like	6	C:GO:0005887; F:GO:0004930; P:GO:0010033; P:GO:0007186;	C:integral component of plasma membrane; F:G-protein coupled receptor activity; P:response to organic substance; P:G-protein coupled receptor signaling pathway; P:response to endogenous stimulus; P:cell surface receptor signaling pathway		
Acrdi.XM_015903995.1/1-906	Monca.augustus.g27762.t1/1-918	0.173899	0.203223	D(2) dopamine receptor A-like	2	P:GO:0007186; C:GO:0016020	P:G-protein coupled receptor signaling pathway; C:membrane		
Acrdi.XM_015923950.1/1-4137	Monca.augustus.g62268.t1/1-4863	0.232054	0.203193	DEP domain-containing 5 isoform X4					
Acrdi.XM_015911658.1/1-765	Monca.augustus.g24279.t1/1-762	0.254455	0.203181	PREDICTED: uncharacterized protein LOC107345913 isoform X1					
Acrdi.XM_015897123.1/1-4062	Monca.augustus.g55687.t1/1-3885	0.178429	0.203129	PREDICTED: elastin-like					
Acrdi.XM_015913382.1/1-1275	Monca.augustus.g15039.t1/1-1308	0.330514	0.203069	F-box WD repeat-containing 7 isoform X1					
Acrdi.XM_015909610.1/1-948	Monca.augustus.g61631.t1/1-882	0.222873	0.203068	PREDICTED: uncharacterized protein LOC107344012					
Acrdi.XM_015919889.1/1-1659	Monca.augustus.g33625.t1/1-1641	0.269155	0.203054	metabotropic glutamate receptor 3-like	8	P:GO:0050794; P:GO:0044708; P:GO:0007154; P:GO:0044707;	P:regulation of cellular process; P:single-organism behavior; P:cell communication; P:single-multicellular organism process; C:membrane part; P:single organism signaling; P:neur[illegible]		
Acrdi.XM_015902238.1/1-1116	Monca.augustus.g71158.t1/1-3075	0.253908	0.202984	coiled-coil domain-containing 178-like					
Acrdi.XM_015902240.1/1-2520	Monca.adi2mcaRNA11591_R0/1-2469	0.233474	0.20297	neurexin-3-like isoform X2	8	C:GO:0042734; C:GO:0005887; F:GO:0046872; F:GO:0005509;	C:presynaptic membrane; C:integral component of plasma membrane; F:metal ion binding; F:calcium ion binding; C:membrane; C:integral component of membrane; P:chemical s[illegible]		
Acrdi.XM_015907246.1/1-642	Monca.augustus.g7066.t1/1-1581	0.274905	0.202941	PREDICTED: uncharacterized protein C8orf34-like					
Acrdi.XM_015900977.1/1-582	Monca.adi2mcaRNA10204_R0/1-486	0.619207	0.202919	zinc finger 397-like					
Acrdi.XM_015918341.1/1-2373	Monca.augustus.g41661.t1/1-2436	0.264654	0.202887	YEATS domain-containing 2	1	P:GO:0006355	P:regulation of transcription, DNA-templated		
Acrdi.XM_015917025.1/1-621	Monca.augustus.g32844.t1/1-720	0.233562	0.202872	potassium channel	1	C:GO:0016020	C:membrane		
Acrdi.XM_015901905.1/1-1173	Monca.augustus.g58301.t1/1-1056	0.230521	0.202829	ZP domain-containing	2	C:GO:0016020; C:GO:0016021	C:membrane; C:integral component of membrane		
Acrdi.XM_015921774.1/1-1308	Monca.augustus.g41856.t1/1-1314	0.292666	0.202783	leucine-rich repeat-containing 42					
Acrdi.XM_015921368.1/1-504	Monca.augustus.g11100.t1/1-1101	0.363926	0.202772	cell growth regulator with RING finger domain 1-like isoform X2	1	F:GO:0008270	F:zinc ion binding		
Acrdi.XM_015917893.1/1-2268	Monca.augustus.g14689.t1/1-1368	0.100961	0.202582	diacylglycerol O-acyltransferase 1	1	F:GO:0016740	F:transferase activity		
Acrdi.XM_015894623.1/1-540	Monca.augustus.g10514.t1/1-441	0.0538839	0.202497	PREDICTED: uncharacterized protein LOC107329968 isoform X1	4	C:GO:0005634; F:GO:0046872; F:GO:0003682; P:GO:0006357	C:nucleus; F:metal ion binding; F:chromatin binding; P:regulation of transcription from RNA polymerase II promoter		
Acrdi.XM_015918331.1/1-2853	Monca.augustus.g41664.t1/1-2283	0.182026	0.202393	conserved oligomeric Golgi complex subunit 7-like	7	C:GO:0017119; P:GO:0098656; P:GO:0006890; P:GO:0007030;	C:Golgi transport complex; P:anion transmembrane transport; P:retrograde vesicle-mediated transport, Golgi to ER; P:Golgi organization; F:anion transmembrane transporter acti[illegible]		
Acrdi.XM_015915907.1/1-1209	Monca.augustus.g24369.t1/1-1092	0.133211	0.202381	potassium channel subfamily K member 15-like isoform X2	2	C:GO:0016020; P:GO:0006810	C:membrane; P:transport		
Acrdi.XM_015907006.1/1-636	Monca.augustus.g33397.t1/1-1917	0.235722	0.202366	Discoidin domain-containing receptor 2					
Acrdi.XM_015901421.1/1-3597	Monca.adi2mcaRNA10726_R0/1-3327	0.212922	0.20236	coadhesin-like isoform X2					
Acrdi.XM_015918345.1/1-1404	Monca.augustus.g41641.t1/1-2541	0.3141	0.202355	cap-specific mRNA (nucleoside-2 -O-)-methyltransferase 1	7	F:GO:0003676; P:GO:0006370; F:GO:0004483; F:GO:0008168;	F:nucleic acid binding; P:7-methylguanosine mRNA capping; F:mRNA (nucleoside-2'-O-)-methyltransferase activity; F:methyltransferase activity; F:transferase activity; P:methylat[illegible]		
Acrdi.XM_015898218.1/1-987	Monca.augustus.g64609.t1/1-975	0.285221	0.202315	L-threonine dehydratase catabolic -like	6	F:GO:0003941; F:GO:0030170; C:GO:0005737; P:GO:0006567;	F:L-serine ammonia-lyase activity; F:pyridoxal phosphate binding; C:cytoplasm; P:threonine catabolic process; F:L-threonine ammonia-lyase activity; P:L-serine catabolic proc	EC:4.[illegible]	[illegible]
Acrdi.XM_015903015.1/1-1212	Monca.adi2mcaRNA12379_R0/1-1092	0.166504	0.202315	collagen alpha-1(XXVII) chain-like isoform X1	1	P:GO:0009987	P:cellular process		
Acrdi.XM_015897385.1/1-1284	Monca.augustus.g29324.t1/1-1281	0.284801	0.202305	chromobox homolog 2	12	P:GO:0000122; C:GO:0000792; F:GO:0003677; C:GO:0000791;	P:negative regulation of transcription from RNA polymerase II promoter; C:heterochromatin; F:DNA binding; C:euchromatin; C:PRC1 complex; P:transcription, DNA-templated; P:pr[illegible]		
Acrdi.XM_015901504.1/1-4755	Monca.augustus.g36343.t1/1-4734	0.261771	0.202257	Brain-specific angiogenesis inhibitor 3	4	F:GO:0004930; P:GO:0007186; C:GO:0016020; C:GO:0016021	F:G-protein coupled receptor activity; P:G-protein coupled receptor signaling pathway; C:membrane; C:integral component of membrane		
Acrdi.XM_015892323.1/1-2592	Monca.adi2mcaRNA38248_R0/1-2298	0.16378	0.202188	1-phosphatidylinositol 4,5-bisphosphate phosphodiesterase eta-2 isofc	3	F:GO:0004629; P:GO:0046488; C:GO:0005886	F:phospholipase C activity; P:phosphatidylinositol metabolic process; C:plasma membrane		
Acrdi.XM_015918515.1/1-1803	Monca.adi2mcaRNA29557_R0/1-927	0.231677	0.202128	ferric-chelate reductase 1	3	C:GO:0016020; C:GO:0016021; P:GO:0055114	C:membrane; C:integral component of membrane; P:oxidation-reduction process		
Acrdi.XM_015896691.1/1-1041	Monca.augustus.g37002.t1/1-2943	0.238922	0.202075	PREDICTED: uncharacterized protein LOC107331990					
Acrdi.XM_015920625.1/1-3069	Monca.adi2mcaRNA31951_R0/1-2901	0.195014	0.202062	PREDICTED: uncharacterized protein LOC107354189	4	P:GO:0042273; C:GO:0016020; C:GO:0016021; P:GO:0006397	P:ribosomal large subunit biogenesis; C:membrane; C:integral component of membrane; P:mRNA processing		
Acrdi.XM_015904471.1/1-1602	Monca.augustus.g52726.t1/1-1881	0.311576	0.202048	Biotin-- ligase					
Acrdi.XM_015900717.1/1-1515	Monca.adi2mcaRNA31306_R5/1-1299	0.175772	0.201921	coadhesin-like isoform X2	3	C:GO:0016020; F:GO:0005044; P:GO:0006898	C:membrane; F:scavenger receptor activity; P:receptor-mediated endocytosis		
Acrdi.XM_015908661.1/1-1041	Monca.augustus.g32496.t1/1-1674	0.238908	0.20192	BDNF NT-3 growth factors receptor-like	12	F:GO:0000166; F:GO:0005524; C:GO:0016020; C:GO:0016021;	F:nucleotide binding; F:ATP binding; C:membrane; C:integral component of membrane; F:protein kinase activity; P:phosphorylation; F:transferase activity; F:protein serine/threoni[illegible]		
Acrdi.XM_015906208.1/1-3366	Monca.augustus.g21700.t1/1-3495	0.243693	0.201907	flocculation FLO11-like isoform X2					
Acrdi.XM_015925352.1/1-840	Monca.augustus.g6774.t1/1-1227	0.205991	0.201843	monocarboxylate transporter 10-like	4	P:GO:0006811; P:GO:0055085; C:GO:0016020; F:GO:0005215	P:ion transport; P:transmembrane transport; C:membrane; F:transporter activity		
Acrdi.XM_015896153.1/1-3420	Monca.augustus.g16992.t1/1-6618	0.230603	0.20184	leucine-rich repeat-containing DDB_G0281931	2	C:GO:0016020; C:GO:0016021	C:membrane; C:integral component of membrane		
Acrdi.XM_015911389.1/1-999	Monca.augustus.g9156.t1/1-774	0.269505	0.20183	N-terminal EF-hand calcium-binding 3					
Acrdi.XM_015900145.1/1-2340	Monca.augustus.g59988.t1/1-2856	0.200437	0.201823	phosphatidylinositol 3-kinase regulatory subunit alpha-like isoform X2	3	P:GO:0050794; P:GO:0044699; P:GO:0009987	P:regulation of cellular process; P:single-organism process; P:cellular process		
Acrdi.XM_015909732.1/1-564	Monca.augustus.g2270.t1/1-564	0.359814	0.201819	mitochondrial isoform X3	1	P:GO:0008152	P:metabolic process		
Acrdi.XM_015916054.1/1-1620	Monca.augustus.g60988.t1/1-1491	0.219576	0.201722	diacylglycerol O-acyltransferase MT1468	3	F:GO:0008374; C:GO:0016020; P:GO:0045017	F:O-acyltransferase activity; C:membrane; P:glycerolipid biosynthetic process		
Acrdi.XM_015920177.1/1-360	Monca.adi2mcaRNA31418_R0/1-366	0.210983	0.201681	complement C1q tumor necrosis factor-related 3-like	11	F:GO:0008270; P:GO:0006811; P:GO:0034220; P:GO:0006508;	F:zinc ion binding; P:ion transport; P:ion transmembrane transport; P:proteolysis; C:membrane; C:integral component of membrane; F:extracellular ligand-gated ion channel activi[illegible]		
Acrdi.XM_015901075.1/1-1347	Monca.augustus.g57647.t1/1-1815	0.173778	0.201666	DNA polymerase iota	2	P:GO:0006281; F:GO:0003684	P:DNA repair; F:damaged DNA binding		
Acrdi.XM_015904540.1/1-432	Monca.augustus.g52747.t1/1-447	0.331158	0.201646	transmembrane 242	2	C:GO:0016020; C:GO:0016021	C:membrane; C:integral component of membrane		
Acrdi.XM_015924948.1/1-1737	Monca.augustus.g60390.t1/1-936	0.173479	0.201633	Sorting nexin-27	2	P:GO:0007165; F:GO:0035091	P:signal transduction; F:phosphatidylinositol binding		
Acrdi.XM_015905674.1/1-1194	Monca.augustus.g26833.t1/1-1122	0.181629	0.20163	Abscission ut checkpoint regulator	12	P:GO:0051301; P:GO:0009838; C:GO:0005813; C:GO:0005856;	P:cell division; P:abscission; C:centrosome; C:cytoskeleton; C:cytoplasm; F:metal ion binding; C:cleavage furrow; P:negative regulation of cytokinesis; F:lipid binding; C:midbody; F:p[illegible]		
Acrdi.XM_015917867.1/1-1497	Monca.augustus.g25191.t1/1-1686	0.187465	0.201609	Activin receptor type-2B	3	F:GO:0004672; P:GO:0048513; P:GO:0009987	F:protein kinase activity; P:animal organ development; P:cellular process		
Acrdi.XM_015925006.1/1-771	Monca.augustus.g53482.t1/1-765	0.244596	0.201564	TM2 domain-containing almondex	1	C:GO:0016020	C:membrane		
Acrdi.XM_015897061.1/1-774	Monca.augustus.g16447.t1/1-780	0.260866	0.201555	Alpha beta hydrolase domain-containing 14A	1	F:GO:0016787	F:hydrolase activity		
Acrdi.XM_015911534.1/1-459	Monca.augustus.g45445.t1/1-462	0.223952	0.201502	PREDICTED: uncharacterized protein LOC107345786					
Acrdi.XM_015914188.1/1-2271	Monca.augustus.g50918.t1/1-2352	0.252661	0.201423	monocarboxylate transporter 10-like	4	P:GO:0006811; P:GO:0055085; C:GO:0016021; F:GO:0008028	P:ion transport; P:transmembrane transport; C:integral component of membrane; F:monocarboxylic acid transmembrane transporter activity		
Acrdi.XM_015917617.1/1-1689	Monca.augustus.g9234.t1/1-1737	0.25337	0.201381	Tripartite motif-containing 2	2	F:GO:0008270; F:GO:0046872	F:zinc ion binding; F:metal ion binding		
Acrdi.XM_015920651.1/1-2310	Monca.augustus.g44638.t1/1-1818	0.133952	0.201319	endothelin-converting enzyme 2-like isoform X1	7	P:GO:0006508; C:GO:0016020; C:GO:0016021; P:GO:0016485;	P:proteolysis; C:membrane; C:integral component of membrane; P:protein processing; P:olfactory learning; F:metalloendopeptidase activity; F:metallopeptidase activity		
Acrdi.XM_015903260.1/1-1422	Monca.augustus.g51693.t1/1-1422	0.30283	0.201302	probable E3 ubiquitin- ligase DTX2	2	F:GO:0008270; F:GO:0046872	F:zinc ion binding; F:metal ion binding		
Acrdi.XM_015907324.1/1-1281	Monca.augustus.g31775.t1/1-1266	0.270812	0.201268	phosphatase 1 regulatory subunit 27-like	5	F:GO:0019901; F:GO:0030165; F:GO:0019887; C:GO:0005575;	F:protein kinase binding; F:PDZ domain binding; F:protein kinase regulator activity; C:cellular_component; P:nerve growth factor signaling pathway		
Acrdi.XM_015902592.1/1-1428	Monca.augustus.g21275.t1/1-1410	0.272225	0.201267	F-box LRR-repeat					
Acrdi.XM_015919771.1/1-2148	Monca.augustus.g48084.t1/1-2184	0.220467	0.201258	phosphatase 2C-like domain-containing 1	7	F:GO:0004722; F:GO:0004721; F:GO:0003824; F:GO:0046872;	F:protein serine/threonine phosphatase activity; F:phosphoprotein phosphatase activity; F:catalytic activity; F:metal ion binding; P:protein dephosphorylation; F:cation binding; F:h[illegible]		
Acrdi.XM_015908775.1/1-393	Monca.augustus.g24458.t1/1-900	0.174614	0.20125	PREDICTED: uncharacterized protein LOC107343203					
Acrdi.XM_015922527.1/1-3546	Monca.augustus.g12155.t1/1-6354	0.22121	0.201243	hemicentin-1 isoform X1	1	F:GO:0005509	F:calcium ion binding		
Acrdi.XM_015921364.1/1-759	Monca.augustus.g70621.t1/1-759	0.226705	0.201232	TLC domain-containing 2	4	P:GO:0008150; C:GO:0016020; C:GO:0016021; F:GO:0003674	P:biological_process; C:membrane; C:integral component of membrane; F:molecular_function		
Acrdi.XM_015903619.1/1-1605	Monca.augustus.g66471.t1/1-2283	0.251028	0.201228	LETM1 and EF-hand domain-containing	3	F:GO:0005509; F:GO:0043022; C:GO:0016021	F:calcium ion binding; F:ribosome binding; C:integral component of membrane		
Acrdi.XM_015900379.1/1-1026	Monca.augustus.g63387.t1/1-1053	0.259967	0.201094	histamine H2 receptor-like	9	F:GO:0004930; F:GO:0001588; P:GO:0001963; P:GO:0007186;	F:G-protein coupled receptor activity; F:dopamine neurotransmitter receptor activity, coupled via Gs; P:synaptic transmission, dopaminergic; P:G-protein coupled receptor signali[illegible]		
Acrdi.XM_015892558.1/1-3186	Monca.augustus.g36531.t1/1-3291	0.230858	0.201074	transcriptional repressor NF-X1 isoform X1					
Acrdi.XM_015913013.1/1-1107	Monca.augustus.g45143.t1/1-1251	0.255944	0.201067	probable inactive tRNA-specific adenosine deaminase 3	6	F:GO:0008270; F:GO:0003824; F:GO:0008033; P:GO:0009451;	F:zinc ion binding; F:catalytic activity; P:tRNA processing; P:RNA modification; C:cytosol; F:deaminase activity		
Acrdi.XM_015893387.1/1-540	Monca.adi2mcaRNA39462_R1/1-510	0.246016	0.201024	tRNA-dihydrouridine(16 17) synthase [NAD(P)(+)]-like isoform X1	1	F:GO:0003824	F:catalytic activity		
Acrdi.XM_015892486.1/1-789	Monca.augustus.g41314.t1/1-681	0.0571384	0.20098	PREDICTED: uncharacterized protein LOC107327743					
Acrdi.XM_015892548.1/1-777	Monca.augustus.g36541.t1/1-1170	0.182178	0.200971	alpha-(1,3)-fucosyltransferase C-like	2	C:GO:0016020; F:GO:0016740	C:membrane; F:transferase activity		
Acrdi.XM_015895966.1/1-2196	Monca.augustus.g22586.t1/1-2208	0.171863	0.200963	Nucleoporin GLE1	8	P:GO:0006406; P:GO:0006449; C:GO:0005737; F:GO:0000822;	P:mRNA export from nucleus; P:regulation of translational termination; C:cytoplasm; F:inositol hexakisphosphate binding; F:phospholipid binding; F:translation initiation factor bin[illegible]		
Acrdi.XM_015914534.1/1-990	Monca.augustus.g65444.t1/1-990	0.24572	0.200959	major facilitator superfamily domain-containing 3-like	1	C:GO:0016020	C:membrane		
Acrdi.XM_015913477.1/1-1164	Monca.augustus.g9919.t1/1-1557	0.222305	0.20087	transmembrane 26	2	C:GO:0016020; C:GO:0016021	C:membrane; C:integral component of membrane		
Acrdi.XM_015917808.1/1-657	Monca.augustus.g2155.t1/1-657	0.204562	0.200837	PREDICTED: uncharacterized protein LOC107351477					
Acrdi.XM_015903291.1/1-987	Monca.adi2mcaRNA12696_R7/1-795	0.0517387	0.200792	ATP-dependent DNA helicase -like	5	F:GO:0097159; F:GO:1901363; F:GO:0004386; P:GO:0006259;	F:organic cyclic compound binding; F:heterocyclic compound binding; F:helicase activity; P:DNA metabolic process; C:intracellular part	EC:3.[illegible]	[illegible]
Acrdi.XM_015903431.1/1-4521	Monca.adi2mcaRNA1407_R0/1-4308	0.265746	0.200767	WASH complex subunit FAM21-like	1	C:GO:0071203	C:WASH complex		
Acrdi.XM_015903205.1/1-768	Monca.augustus.g61024.t1/1-813	0.258234	0.200761	keratin-associated 10-6-like					
Acrdi.XM_015903005.1/1-453	Monca.augustus.g23675.t1/1-507	0.185751	0.200759	PREDICTED: uncharacterized protein LOC107337772					
Acrdi.XM_015893201.1/1-738	Monca.adi2mcaRNA39264_R8/1-888	0.292478	0.200746	PREDICTED: uncharacterized protein LOC107328473					
Acrdi.XM_015901315.1/1-516	Monca.adi2mcaRNA10569_R0/1-585	0.121198	0.20072	acidic phospholipase A2 homolog	5	F:GO:0047498; F:GO:0005509; P:GO:0035899; P:GO:0044398;	F:calcium-dependent phospholipase A2 activity; F:calcium ion binding; P:negative regulation of blood coagulation in other organism; P:envenomation resulting in induction of	EC:3.[illegible]	[illegible]
Acrdi.XM_015905204.1/1-636	Monca.augustus.g57138.t1/1-630	0.277225	0.200717	Synaptonemal complex 1					
Acrdi.XM_015892938.1/1-864	Monca.augustus.g19275.t1/1-882	0.237484	0.200717	PREDICTED: uncharacterized protein LOC107328200					
Acrdi.XM_015901404.1/1-882	Monca.adi2mcaRNA10664_R0/1-846	0.169044	0.200704	Tetratricopeptide repeat 1					
Acrdi.XM_015906756.1/1-522	Monca.adi2mcaRNA16554_R0/1-534	1.05496	0.200693	nuclease HARBI1	1	F:GO:0003676	F:nucleic acid binding		
Acrdi.XM_015892757.1/1-1191	Monca.augustus.g22456.t1/1-1182	0.310416	0.200662	abnormal spindle-like microcephaly-associated homolog isoform X8	2	P:GO:0007339; C:GO:0016020	P:binding of sperm to zona pellucida; C:membrane		
Acrdi.XM_015891832.1/1-984	Monca.adi2mcaRNA37699_R0/1-963	0.247113	0.200535	adenosine receptor A3-like					
Acrdi.XM_015920203.1/1-1683	Monca.augustus.g66737.t1/1-1623	0.198307	0.200522	phosphatidylinositol phosphatase PTPRQ-like	8	P:GO:0006813; F:GO:0005249; P:GO:0071805; C:GO:0008076;	P:potassium ion transport; F:voltage-gated potassium channel activity; P:potassium ion transmembrane transport; C:voltage-gated potassium channel complex; C:membrane; C:in[illegible]		
Acrdi.XM_015916646.1/1-1029	Monca.augustus.g51058.t1/1-1005	0.144041	0.200509	Neuropeptide FF receptor 2	1	P:GO:0007165	P:signal transduction		
Acrdi.XM_015912620.1/1-954	Monca.augustus.g5180.t1/1-960	0.192857	0.200492	proline-rich transmembrane 4-like	2	C:GO:0016020; C:GO:0016021	C:membrane; C:integral component of membrane		
Acrdi.XM_015898771.1/1-1272	Monca.augustus.g55610.t1/1-1260	0.151766	0.200488	Transmembrane prolyl 4-hydroxylase	10	P:GO:0032836; F:GO:0031418; F:GO:0005506; F:GO:0016705;	P:glomerular basement membrane development; F:L-ascorbic acid binding; F:iron ion binding; F:oxidoreductase activity, acting on paired donors, with incorporation or reduction o[illegible]		
Acrdi.XM_015910126.1/1-2706	Monca.augustus.g38323.t1/1-4329	0.214064	0.200474	Cadherin EGF LAG seven-pass G-type receptor 1	3	F:GO:0004888; P:GO:0007165; C:GO:0016020	F:transmembrane signaling receptor activity; P:signal transduction; C:membrane		
Acrdi.XM_015897049.1/1-879	Monca.augustus.g55688.t1/1-471	0.199343	0.200405	PREDICTED: uncharacterized protein LOC107332322					
Acrdi.XM_015909601.1/1-1176	Monca.augustus.g7755.t1/1-1116	0.20956	0.200361	tyramine receptor Ser-2-like	2	P:GO:0007165; F:GO:0004871	P:signal transduction; F:signal transducer activity		
Acrdi.XM_015910970.1/1-2127	Monca.adi2mcaRNA21177_R0/1-1902	0.200307	0.200359	nicastrin-like isoform X1	3	C:GO:0016020; C:GO:0016021; P:GO:0016485	C:membrane; C:integral component of membrane; P:protein processing		
Acrdi.XM_015900349.1/1-2091	Monca.augustus.g63386.t1/1-5307	0.270211	0.200342	zinc finger FYVE domain-containing 16	9	F:GO:0005547; C:GO:0005769; C:GO:0005737; F:GO:0046872;	F:phosphatidylinositol-3,4,5-trisphosphate binding; C:early endosome; C:cytoplasm; F:metal ion binding; C:intracellular membrane-bounded organelle; P:endosomal transport; F:p[illegible]		
Acrdi.XM_015924751.1/1-636	Monca.adi2mcaRNA16125_R0/1-591	0.231976	0.200337	chymotrypsin-like elastase family member 2A	2	P:GO:0006508; F:GO:0004252	P:proteolysis; F:serine-type endopeptidase activity	EC:3.[illegible]	[illegible]
Acrdi.XM_015923348.1/1-1143	Monca.augustus.g51723.t1/1-2706	0.275127	0.200336	ankyrin repeat domain-containing					
Acrdi.XM_015921939.1/1-570	Monca.adi2mcaRNA33343_R1/1-525	0.329258	0.200297	PREDICTED: uncharacterized protein LOC107355361					
Acrdi.XM_015907880.1/1-975	Monca.augustus.g53159.t1/1-1188	0.225458	0.200279	GTPase mitochondrial	2	F:GO:0097159; F:GO:1901363	F:organic cyclic compound binding; F:heterocyclic compound binding		
Acrdi.XM_015921576.1/1-1764	Monca.augustus.g2829.t1/1-2565	0.240398	0.200262	Phosphatidylinositol-glycan-specific phospholipase D	1	C:GO:0005576	C:extracellular region		
Acrdi.XM_015919739.1/1-714	Monca.augustus.g40902.t1/1-948	0.198744	0.200186	UPF0600 C5orf51 homolog					
Acrdi.XM_015895723.1/1-948	Monca.augustus.g64081.t1/1-1524	0.288143	0.200172	F-box only 7 isoform X1	4	F:GO:0005515; C:GO:0005737; P:GO:0048523; P:GO:0009987	F:protein binding; C:cytoplasm; P:negative regulation of cellular process; P:cellular process		
Acrdi.XM_015900579.1/1-1239	Monca.augustus.g33653.t1/1-1713	0.249071	0.200163	NADPH oxidase 4 isoform X1	4	F:GO:0016491; C:GO:0016020; C:GO:0016021; P:GO:0055114	F:oxidoreductase activity; C:membrane; C:integral component of membrane; P:oxidation-reduction process		
Acrdi.XM_015923545.1/1-3621	Monca.augustus.g58091.t1/1-4401	0.217569	0.200113	Serine threonine- kinase 11-interacting					
Acrdi.XM_015915645.1/1-1671	Monca.augustus.g55115.t1/1-1656	0.271949	0.200085	Cortactin-binding 2					
Acrdi.XM_015909995.1/1-1059	Monca.augustus.g6687.t1/1-990	0.21587	0.199996	poly(A) RNA polymerase GLD2 isoform X1	3	F:GO:0005488; C:GO:0044424; P:GO:0009987	F:binding; C:intracellular part; P:cellular process		
Acrdi.XM_015914205.1/1-732	Monca.augustus.g58341.t1/1-1239	0.308159	0.199937	FAM69C-like	2	C:GO:0016020; C:GO:0016021	C:membrane; C:integral component of membrane		
Acrdi.XM_015898328.1/1-714	Monca.augustus.g66594.t1/1-720	0.239744	0.199921	Ras-related Rab-26					
Acrdi.XM_015921994.1/1-927	Monca.augustus.g23720.t1/1-1005	0.216888	0.199898	trapped in endoderm-1-like	3	P:GO:0007165; C:GO:0016020; F:GO:0004871	P:signal transduction; C:membrane; F:signal transducer activity		
Acrdi.XM_015898763.1/1-1212	Monca.augustus.g55630.t1/1-1371	0.188875	0.199884	L-amino-acid oxidase	4	F:GO:0016491; C:GO:0016020; C:GO:0016021; P:GO:0055114	F:oxidoreductase activity; C:membrane; C:integral component of membrane; P:oxidation-reduction process		
Acrdi.XM_015895520.1/1-2637	Monca.adi2mcaRNA4256_R0/1-2544	0.196531	0.199882	mitogen-activated kinase kinase kinase MLT-like	8	F:GO:0000166; P:GO:0000165; F:GO:0005524; P:GO:0000186;	F:nucleotide binding; P:MAPK cascade; F:ATP binding; P:activation of MAPKK activity; F:protein kinase activity; F:MAP kinase kinase kinase activity; P:protein phosphorylation; C:int[illegible]		
Acrdi.XM_015907881.1/1-600	Monca.augustus.g53158.t1/1-1137	0.288562	0.199879	arrestin domain-containing A-like	2	P:GO:0007165; F:GO:0046872	P:signal transduction; F:metal ion binding		
Acrdi.XM_015900940.1/1-1653	Monca.augustus.g27904.t1/1-1656	0.244759	0.199876	Bardet-Biedl syndrome 10 isoform X1	6	F:GO:0000166; F:GO:0005524; C:GO:0005737; P:GO:0060026;	F:nucleotide binding; F:ATP binding; C:cytoplasm; P:convergent extension; P:convergent extension involved in gastrulation; P:gastrulation		
Acrdi.XM_015893543.1/1-900	Monca.augustus.g25401.t1/1-900	0.244232	0.199874	neuropeptide Y receptor type 2-like	3	F:GO:0004930; P:GO:0007186; C:GO:0016021	F:G-protein coupled receptor activity; P:G-protein coupled receptor signaling pathway; C:integral component of membrane		
Acrdi.XM_015892324.1/1-1893	Monca.augustus.g5375.t1/1-2010	0.199187	0.199873	leucine- glutamate- and lysine-rich 1 isoform X2					
Acrdi.XM_015893123.1/1-912	Monca.augustus.g26279.t1/1-1686	0.202097	0.199819	Tyrosine--tRNA cytoplasmic	4	F:GO:0097159; F:GO:1901363; F:GO:0016874; P:GO:0006412	F:organic cyclic compound binding; F:heterocyclic compound binding; F:ligase activity; P:translation		
Acrdi.XM_015906970.1/1-1146	Monca.augustus.g16780.t1/1-1143	0.127648	0.19975	glycosyl transferase	2	C:GO:0016020; C:GO:0016021	C:membrane; C:integral component of membrane		
Acrdi.XM_015900147.1/1-504	Monca.augustus.g59994.t1/1-501	0.245582	0.199738	tctex1 domain-containing 1-B-like					
Acrdi.XM_015911161.1/1-834	Monca.adi2mcaRNA21380_R0/1-795	0.160634	0.199729	PREDICTED: uncharacterized protein LOC107345445					
Acrdi.XM_015923865.1/1-1083	Monca.augustus.g50398.t1/1-1932	0.20553	0.199681	calponin homology domain-containing DDB_G0272472-like					
Acrdi.XM_015904183.1/1-1788	Monca.augustus.g18703.t1/1-2973	0.202692	0.199648	eukaryotic translation initiation factor 2D isoform X1	3	F:GO:0003743; F:GO:0003723; P:GO:0006413	F:translation initiation factor activity; F:RNA binding; P:translational initiation		
Acrdi.XM_015910444.1/1-2283	Monca.augustus.g60935.t1/1-2061	0.245931	0.199639	xenotropic and polytropic retrovirus receptor 1 homolog	2	C:GO:0016020; C:GO:0016021	C:membrane; C:integral component of membrane		
Acrdi.XM_015919955.1/1-1023	Monca.adi2mcaRNA3178_R0/1-963	0.155863	0.199598	caspase 3	5	F:GO:0097200; C:GO:0005737; P:GO:0006508; P:GO:0042981;	F:cysteine-type endopeptidase activity involved in execution phase of apoptosis; C:cytoplasm; P:proteolysis; P:regulation of apoptotic process; P:execution phase of apoptosis	EC:3.[illegible]	[illegible]
Acrdi.XM_015916480.1/1-996	Monca.augustus.g37312.t1/1-1257	0.150677	0.199561	carboxypeptidase B	2	C:GO:0005615; F:GO:0004181	C:extracellular space; F:metallocarboxypeptidase activity	EC:3.[illegible]	[illegible]
Acrdi.XM_015897562.1/1-414	Monca.augustus.g87.t1/1-669	0.183838	0.199519	Telomerase-binding EST1A					
Acrdi.XM_015922678.1/1-537	Monca.augustus.g1943.t1/1-531	0.220899	0.199485	metallo ase inhibitor 3-like					
Acrdi.XM_015897485.1/1-2661	Monca.augustus.g38042.t1/1-1773	0.173983	0.199439	voltage-dependent calcium channel subunit alpha-2 delta-2-like	2	C:GO:0016020; C:GO:0016021	C:membrane; C:integral component of membrane		
Acrdi.XM_015912944.1/1-2130	Monca.augustus.g38072.t1/1-2484	0.224405	0.199404	Protection of telomeres 1	6	P:GO:0065008; F:GO:0005515; P:GO:2000278; F:GO:0043047;	P:regulation of biological quality; F:protein binding; P:regulation of DNA biosynthetic process; F:single-stranded telomeric DNA binding; P:telomere organization; C:nuclear telomer[illegible]		
Acrdi.XM_015900088.1/1-984	Monca.augustus.g24386.t1/1-513	0.13259	0.199349	PREDICTED: uncharacterized protein LOC107335122					
Acrdi.XM_015921567.1/1-1290	Monca.augustus.g3441.t1/1-1410	0.193509	0.199348	Phosphatidylinositol phosphatase PTPRQ	2	C:GO:0016020; C:GO:0016021	C:membrane; C:integral component of membrane		
Acrdi.XM_015920869.1/1-3003	Monca.adi2mcaRNA32171_R0/1-2700	0.243252	0.199344	Lipase maturation factor 2	2	C:GO:0005789; C:GO:0016021	C:endoplasmic reticulum membrane; C:integral component of membrane		
Acrdi.XM_015899649.1/1-597	Monca.augustus.g54482.t1/1-597	0.344011	0.199333	transmembrane 145					
Acrdi.XM_015893788.1/1-492	Monca.augustus.g31995.t1/1-516	0.208086	0.199328	PREDICTED: uncharacterized protein LOC107329057					
Acrdi.XM_015915513.1/1-654	Monca.augustus.g66104.t1/1-657	0.1948	0.199301	PREDICTED: uncharacterized protein LOC107349369 isoform X1					
Acrdi.XM_015911414.1/1-717	Monca.augustus.g70348.t1/1-750	0.21746	0.199255	Sorting nexin-21	1	F:GO:0035091	F:phosphatidylinositol binding		
Acrdi.XM_015893211.1/1-6726	Monca.augustus.g44523.t1/1-7239	0.244886	0.199247	polycystin-1-like isoform X2	4	P:GO:0050982; F:GO:0005262; C:GO:0016020; P:GO:0070588	P:detection of mechanical stimulus; F:calcium channel activity; C:membrane; P:calcium ion transmembrane transport		
Acrdi.XM_015903096.1/1-1317	Monca.augustus.g45190.t1/1-2958	0.172898	0.199231	SMC5-SMC6 complex localization factor 2-like isoform X1					
Acrdi.XM_015898018.1/1-1275	Monca.augustus.g39410.t1/1-1308	0.300408	0.199131	doublesex- and mab-3-related transcription factor A2-like	6	F:GO:0003677; C:GO:0005634; F:GO:0003700; F:GO:0046872;	F:DNA binding; C:nucleus; F:transcription factor activity, sequence-specific DNA binding; F:metal ion binding; P:regulation of transcription, DNA-templated; F:sequence-specific DN[illegible]		
Acrdi.XM_015904704.1/1-1107	Monca.augustus.g36199.t1/1-1380	0.251514	0.199092	ADP-ribosyl cyclase cyclic ADP-ribose hydrolase 1	5	P:GO:0048522; C:GO:0016020; C:GO:0044464; P:GO:0002684;	P:positive regulation of cellular process; C:membrane; C:cell part; P:positive regulation of immune system process; F:hydrolase activity, acting on glycosyl bonds		
Acrdi.XM_015919461.1/1-339	Monca.augustus.g22483.t1/1-348	0.293887	0.199083	PREDICTED: uncharacterized protein LOC107353140 isoform X1					
Acrdi.XM_015910438.1/1-576	Monca.augustus.g60929.t1/1-1596	0.184429	0.199075	Abhydrolase domain-containing 16A	2	C:GO:0016020; C:GO:0016021	C:membrane; C:integral component of membrane		
Acrdi.XM_015893588.1/1-441	Monca.augustus.g41449.t1/1-438	0.134828	0.198938	KIAA1143 like					
Acrdi.XM_015917299.1/1-1137	Monca.adi2mcaRNA28231_R3/1-1185	0.198187	0.198921	brain-specific angiogenesis inhibitor 1-like	4	F:GO:0004930; P:GO:0007186; C:GO:0016021; P:GO:0007166	F:G-protein coupled receptor activity; P:G-protein coupled receptor signaling pathway; C:integral component of membrane; P:cell surface receptor signaling pathway		
Acrdi.XM_015924735.1/1-2268	Monca.augustus.g4186.t1/1-2583	0.206147	0.198864	mismatch repair endonuclease PMS2	10	F:GO:0016887; F:GO:0032407; C:GO:0015630; C:GO:0005737;	F:ATPase activity; F:MutSalpha complex binding; C:microtubule cytoskeleton; C:cytoplasm; F:nuclease activity; C:MutLalpha complex; P:mismatch repair; C:nucleoplasm; F:sin[illegible]	EC:3.[illegible]	[illegible]
Acrdi.XM_015914878.1/1-537	Monca.adi2mcaRNA25499_R5/1-549	0.336634	0.198842	Transposon Tf2-6 poly					
Acrdi.XM_015909342.1/1-1026	Monca.augustus.g13084.t1/1-2802	0.218105	0.198765	PREDICTED: uncharacterized protein LOC107343752	2	C:GO:0016020; C:GO:0016021	C:membrane; C:integral component of membrane		
Acrdi.XM_015905206.1/1-1221	Monca.augustus.g52828.t1/1-1329	0.187811	0.198711	synaptotagmin 7	9	C:GO:0098793; P:GO:0048791; F:GO:0019905; P:GO:0006906;	C:presynapse; P:calcium ion-regulated exocytosis of neurotransmitter; F:syntaxin binding; P:vesicle fusion; F:calcium ion binding; F:clathrin binding; P:regulation of calcium ion-dep[illegible]		
Acrdi.XM_015906975.1/1-1038	Monca.adi2mcaRNA16801_R0/1-1092	0.235717	0.198698	PREDICTED: uncharacterized protein LOC107341543					
Acrdi.XM_015901079.1/1-873	Monca.augustus.g57653.t1/1-864	0.202129	0.198698	two pore potassium channel sup-9-like	2	C:GO:0016020; P:GO:0006810	C:membrane; P:transport		
Acrdi.XM_015920110.1/1-372	Monca.augustus.g28570.t1/1-639	0.197854	0.198685	cholestenol delta-isomerase	2	C:GO:0016020; F:GO:0016853	C:membrane; F:isomerase activity		
Acrdi.XM_015898300.1/1-603	Monca.augustus.g61844.t1/1-915	0.251603	0.198672	endonuclease V isoform X2	9	F:GO:0000287; F:GO:0003677; F:GO:0016888; P:GO:0006281;	F:magnesium ion binding; F:DNA binding; F:endodeoxyribonuclease activity, producing 5'-phosphomonoesters; P:DNA repair; P:nucleic acid phosphodiester bond hydrolysis; C:[illegible]	EC:3.[illegible]	[illegible]
Acrdi.XM_015906959.1/1-1017	Monca.augustus.g49150.t1/1-1026	0.370975	0.198637	E3 ubiquitin- ligase RNF34	3	P:GO:0050794; F:GO:0005488; P:GO:0009987	P:regulation of cellular process; F:binding; P:cellular process		
Acrdi.XM_015914031.1/1-957	Monca.augustus.g19519.t1/1-948	0.283227	0.198637	melatonin receptor type 1C-like	1	P:GO:0007165	P:signal transduction		
Acrdi.XM_015899332.1/1-582	Monca.augustus.g64407.t1/1-600	0.307372	0.198625	ras-related and estrogen-regulated growth inhibitor isoform X2	6	F:GO:0005525; F:GO:0000166; P:GO:0007264; P:GO:0007165;	F:GTP binding; F:nucleotide binding; P:small GTPase mediated signal transduction; P:signal transduction; C:membrane; C:intracellular		
Acrdi.XM_015913058.1/1-1665	Monca.augustus.g732.t1/1-1587	0.286641	0.198575	Low choriolytic enzyme	7	F:GO:0008270; F:GO:0046872; P:GO:0006508; F:GO:0008233;	F:zinc ion binding; F:metal ion binding; P:proteolysis; F:peptidase activity; F:metalloendopeptidase activity; F:metallopeptidase activity; F:hydrolase activity		
Acrdi.XM_015908978.1/1-4038	Monca.augustus.g47243.t1/1-5901	0.216549	0.198558	Voltage-dependent L-type calcium channel subunit alpha-1C	1	F:GO:0003824	F:catalytic activity		
Acrdi.XM_015898350.1/1-714	Monca.augustus.g6521.t1/1-669	0.346859	0.198541	PREDICTED: uncharacterized protein LOC107333522					
Acrdi.XM_015914102.1/1-855	Monca.augustus.g14933.t1/1-858	0.246314	0.19851	RWD domain-containing 3 isoform X1	5	C:GO:0005634; P:GO:1902073; C:GO:0005737; P:GO:0032088;	C:nucleus; P:positive regulation of hypoxia-inducible factor-1alpha signaling pathway; C:cytoplasm; P:negative regulation of NF-kappaB transcription factor activity; P:positive regu[illegible]		
Acrdi.XM_015917985.1/1-513	Monca.augustus.g56733.t1/1-522	0.206657	0.198448	U4 small nuclear ribonucleo 27 kDa -like					
Acrdi.XM_015917202.1/1-3129	Monca.augustus.g67107.t1/1-3138	0.229793	0.198425	Neuronal PAS domain-containing 3	3	F:GO:0003700; P:GO:0006355; C:GO:0044424	F:transcription factor activity, sequence-specific DNA binding; P:regulation of transcription, DNA-templated; C:intracellular part		
Acrdi.XM_015901171.1/1-570	Monca.augustus.g58614.t1/1-930	0.249656	0.198388	Coagulation factor VIII	1	F:GO:0070492	F:oligosaccharide binding		
Acrdi.XM_015900936.1/1-2106	Monca.augustus.g27900.t1/1-2937	0.270579	0.19833	nuclear receptor coactivator 5-like isoform X1	2	F:GO:0000166; F:GO:0003676	F:nucleotide binding; F:nucleic acid binding		
Acrdi.XM_015892798.1/1-897	Monca.augustus.g33468.t1/1-948	0.15162	0.198323	histamine H2 receptor-like	3	F:GO:0004930; P:GO:0007154; P:GO:0044700	F:G-protein coupled receptor activity; P:cell communication; P:single organism signaling		
Acrdi.XM_015899493.1/1-561	Monca.augustus.g37221.t1/1-567	0.353225	0.198266	Ankyrin repeat domain-containing 66					
Acrdi.XM_015913629.1/1-729	Monca.augustus.g48685.t1/1-1221	0.227413	0.19824	carboxypeptidase O-like	7	F:GO:0008270; C:GO:0005615; F:GO:0004180; F:GO:0004181;	F:zinc ion binding; C:extracellular space; F:carboxypeptidase activity; F:metallocarboxypeptidase activity; P:proteolysis; C:membrane; C:integral component of membrane		
Acrdi.XM_015922752.1/1-2472	Monca.adi2mcaRNA38074_R1/1-1290	0.640592	0.198222	NFX1-type zinc finger-containing 1-like	4	C:GO:0005634; F:GO:0003700; F:GO:0008270; P:GO:0006355	C:nucleus; F:transcription factor activity, sequence-specific DNA binding; F:zinc ion binding; P:regulation of transcription, DNA-templated		
Acrdi.XM_015895567.1/1-1209	Monca.augustus.g2045.t1/1-1200	0.20675	0.19819	PREDICTED: uncharacterized protein LOC107331034					
Acrdi.XM_015919996.1/1-3765	Monca.augustus.g49596.t1/1-2532	0.217574	0.198124	neurogenic locus notch homolog 1-like	3	F:GO:0005509; C:GO:0016020; C:GO:0016021	F:calcium ion binding; C:membrane; C:integral component of membrane		
Acrdi.XM_015898851.1/1-570	Monca.augustus.g39879.t1/1-1272	0.117164	0.198089	DEK					
Acrdi.XM_015916109.1/1-1440	Monca.augustus.g50714.t1/1-1281	0.154142	0.198087	carboxypeptidase B-like	5	F:GO:0004180; C:GO:0005615; F:GO:0008270; F:GO:0004181;	F:carboxypeptidase activity; C:extracellular space; F:zinc ion binding; F:metallocarboxypeptidase activity; P:proteolysis		
Acrdi.XM_015908569.1/1-924	Monca.augustus.g38908.t1/1-1701	0.427639	0.198059	PREDICTED: uncharacterized protein LOC107343048	1	F:GO:0046872	F:metal ion binding		
Acrdi.XM_015893552.1/1-1767	Monca.augustus.g58505.t1/1-1782	0.217659	0.198048	ZP domain-containing -like	3	F:GO:0005509; C:GO:0016020; C:GO:0016021	F:calcium ion binding; C:membrane; C:integral component of membrane		
Acrdi.XM_015903381.1/1-849	Monca.augustus.g36047.t1/1-849	0.178389	0.198041	probable E3 ubiquitin- ligase RNF217	12	F:GO:0004842; F:GO:0061630; F:GO:0031624; C:GO:0000151;	F:ubiquitin-protein transferase activity; F:ubiquitin protein ligase activity; F:ubiquitin conjugating enzyme binding; C:ubiquitin ligase complex; C:cytoplasm; F:metal ion binding; P:p[illegible]		
Acrdi.XM_015892313.1/1-1689	Monca.augustus.g54429.t1/1-1686	0.135923	0.198008	beta-lactamase-like 1					
Acrdi.XM_015912149.1/1-5631	Monca.adi2mcaRNA22454_R1/1-5433	0.27809	0.198005	Angiopoietin-related 7					
Acrdi.XM_015909741.1/1-969	Monca.augustus.g2254.t1/1-1464	0.366051	0.197997	slowpoke-binding -like					
Acrdi.XM_015911455.1/1-849	Monca.augustus.g33321.t1/1-756	0.208133	0.197959	nudix hydrolase 11	7	F:GO:0000210; P:GO:2001294; F:GO:0010945; P:GO:0007275;	F:NAD+ diphosphatase activity; P:malonyl-CoA catabolic process; F:CoA pyrophosphatase activity; P:multicellular organism development; P:coenzyme A biosynthetic process;	EC:3.[illegible]	[illegible]
Acrdi.XM_015902890.1/1-1950	Monca.augustus.g27538.t1/1-1911	0.168349	0.197881	XK-related 6	3	P:GO:0070782; C:GO:0016021; C:GO:0005886	P:phosphatidylserine exposure on apoptotic cell surface; C:integral component of membrane; C:plasma membrane		
Acrdi.XM_015898087.1/1-1038	Monca.augustus.g43542.t1/1-1062	0.171061	0.197829	DNA repair XRCC4-like	6	F:GO:0003677; C:GO:0005634; C:GO:0016020; C:GO:0016021;	F:DNA binding; C:nucleus; C:membrane; C:integral component of membrane; P:DNA recombination; P:double-strand break repair		
Acrdi.XM_015905433.1/1-489	Monca.augustus.g42739.t1/1-945	0.225199	0.19781	oxidoreductase NAD-binding domain-containing 1 isoform X1	1	F:GO:0016491	F:oxidoreductase activity		
Acrdi.XM_015898470.1/1-792	Monca.adi2mcaRNA7568_R0/1-855	0.244131	0.197773	disintegrin and metallo ase domain-containing 10 isoform X3	7	P:GO:0019538; F:GO:0005515; P:GO:0050794; C:GO:0005737;	P:protein metabolic process; F:protein binding; P:regulation of cellular process; C:cytoplasm; C:intracellular membrane-bounded organelle; F:peptidase activity; P:cellular process		
Acrdi.XM_015921304.1/1-1269	Monca.augustus.g40066.t1/1-1455	0.206185	0.197766	substance-K receptor-like	4	C:GO:0005887; F:GO:0008528; P:GO:0007186; P:GO:0007166	C:integral component of plasma membrane; F:G-protein coupled peptide receptor activity; P:G-protein coupled receptor signaling pathway; P:cell surface receptor signaling pathw[illegible]		
Acrdi.XM_015911931.1/1-1218	Monca.augustus.g30036.t1/1-1215	0.245916	0.197724	patatin-like phospholipase domain-containing 2	2	P:GO:0065007; P:GO:0006629	P:biological regulation; P:lipid metabolic process		
Acrdi.XM_015918350.1/1-342	Monca.augustus.g41646.t1/1-324	0.20839	0.197687	PREDICTED: uncharacterized protein LOC107352048 isoform X1					
Acrdi.XM_015916852.1/1-402	Monca.adi2mcaRNA27703_R0/1-537	0.240035	0.197682	Neuropeptide FF receptor	5	P:GO:0010033; F:GO:0008188; P:GO:0007165; P:GO:0009719;	P:response to organic substance; F:neuropeptide receptor activity; P:signal transduction; P:response to endogenous stimulus; C:integral component of membrane		
Acrdi.XM_015921938.1/1-1098	Monca.adi2mcaRNA3339_R0/1-870	0.218831	0.197576	Ankyrin repeat domain-containing 55					
Acrdi.XM_015900589.1/1-1461	Monca.augustus.g28779.t1/1-2466	0.212484	0.197576	mitogen-activated kinase kinase kinase 7 isoform X2					
Acrdi.XM_015912045.1/1-753	Monca.augustus.g67629.t1/1-2103	0.153934	0.19755	Serologically defined colon cancer antigen 8	2	C:GO:0005813; P:GO:0031023	C:centrosome; P:microtubule organizing center organization		
Acrdi.XM_015895965.1/1-1305	Monca.augustus.g22587.t1/1-2853	0.22219	0.197523	usherin isoform X2	5	C:GO:0005604; P:GO:0048731; C:GO:0016020; C:GO:0016021;	C:basement membrane; P:system development; C:membrane; C:integral component of membrane; P:basement membrane assembly		
Acrdi.XM_015907348.1/1-522	Monca.augustus.g31825.t1/1-528	0.205455	0.197502	PREDICTED: uncharacterized protein LOC107341870					
Acrdi.XM_015924910.1/1-963	Monca.augustus.g34995.t1/1-3948	0.236658	0.19746	Mis18-binding 1	1	F:GO:0003677	F:DNA binding		
Acrdi.XM_015921370.1/1-2046	Monca.augustus.g4639.t1/1-1995	0.199781	0.197457	Smaug homolog 2	2	F:GO:0003723; P:GO:0006355	F:RNA binding; P:regulation of transcription, DNA-templated		
Acrdi.XM_015909586.1/1-1452	Monca.augustus.g33544.t1/1-1710	0.108822	0.197386	amyloid beta A4 isoform X6	4	F:GO:0046914; C:GO:0016020; C:GO:0016021; F:GO:0008201	F:transition metal ion binding; C:membrane; C:integral component of membrane; F:heparin binding		
Acrdi.XM_015900419.1/1-1041	Monca.augustus.g49252.t1/1-1014	0.140131	0.197348	adenosine receptor A2a-like	7	F:GO:0004930; F:GO:0001588; P:GO:0001963; P:GO:0007186;	F:G-protein coupled receptor activity; F:dopamine neurotransmitter receptor activity, coupled via Gs; P:synaptic transmission, dopaminergic; P:G-protein coupled receptor signali[illegible]		
Acrdi.XM_015923595.1/1-801	Monca.augustus.g61158.t1/1-903	0.175801	0.197326	ribosome biogenesis BRX1 homolog	3	F:GO:0003723; C:GO:0005730; P:GO:0000027	F:RNA binding; C:nucleolus; P:ribosomal large subunit assembly		
Acrdi.XM_015921383.1/1-1134	Monca.augustus.g4652.t1/1-1140	0.174455	0.197301	Serine threonine- kinase Nek7	7	F:GO:0000166; F:GO:0005524; F:GO:0004672; P:GO:0016310;	F:nucleotide binding; F:ATP binding; F:protein kinase activity; P:phosphorylation; F:protein serine/threonine kinase activity; P:protein phosphorylation; F:kinase activity		
Acrdi.XM_015906276.1/1-348	Monca.augustus.g49073.t1/1-423	0.307136	0.197281	60S ribosomal L34-like	5	F:GO:0003723; F:GO:0003735; P:GO:0042254; C:GO:0022625;	F:RNA binding; F:structural constituent of ribosome; P:ribosome biogenesis; C:cytosolic large ribosomal subunit; P:translation		
Acrdi.XM_015913451.1/1-7752	Monca.augustus.g29847.t1/1-16779	0.211763	0.197232	Collagen alpha-6(VI) chain					
Acrdi.XM_015893257.1/1-726	Monca.augustus.g43805.t1/1-771	0.168097	0.19721	ankyrin repeat domain-containing 40-like					
Acrdi.XM_015918463.1/1-2205	Monca.augustus.g55071.t1/1-4284	0.243837	0.197198	VWFA and cache domain-containing 1-like	2	C:GO:0016020; C:GO:0016021	C:membrane; C:integral component of membrane		
Acrdi.XM_015921310.1/1-1500	Monca.adi2mcaRNA32647_R0/1-1428	0.647542	0.197171	exonuclease R569					
Acrdi.XM_015924824.1/1-471	Monca.augustus.g67359.t1/1-441	0.259973	0.197156	PREDICTED: uncharacterized protein LOC107358203					
Acrdi.XM_015897419.1/1-660	Monca.adi2mcaRNA6330_R3/1-429	0.457755	0.197116	PREDICTED: uncharacterized protein LOC107332679					
Acrdi.XM_015916046.1/1-1710	Monca.augustus.g5223.t1/1-2004	0.193164	0.197105	transmembrane anterior posterior transformation 1 homolog	1	C:GO:0016021	C:integral component of membrane		
Acrdi.XM_015899412.1/1-1008	Monca.augustus.g15181.t1/1-1080	0.322426	0.197023	exonuclease V	5	F:GO:0004518; F:GO:0005488; P:GO:0006259; P:GO:0006974;	F:nuclease activity; F:binding; P:DNA metabolic process; P:cellular response to DNA damage stimulus; C:intracellular part		
Acrdi.XM_015896761.1/1-1083	Monca.augustus.g38109.t1/1-1512	0.194574	0.19702	suppressor of tumorigenicity 14	2	P:GO:0006508; F:GO:0004252	P:proteolysis; F:serine-type endopeptidase activity		
Acrdi.XM_015910234.1/1-654	Monca.augustus.g25474.t1/1-720	0.201604	0.196947	Diencephalon mesencephalon homeobox 1-B	3	C:GO:0005634; P:GO:0006355; F:GO:0043565	C:nucleus; P:regulation of transcription, DNA-templated; F:sequence-specific DNA binding		
Acrdi.XM_015903417.1/1-1515	Monca.augustus.g18167.t1/1-2379	0.176216	0.196928	amine oxidase	2	F:GO:0016491; P:GO:0055114	F:oxidoreductase activity; P:oxidation-reduction process		
Acrdi.XM_015893784.1/1-768	Monca.augustus.g31996.t1/1-1137	0.17069	0.196925	amino acid oxidase	5	F:GO:0071949; F:GO:0016491; P:GO:0055114; P:GO:0046416;	F:FAD binding; F:oxidoreductase activity; P:oxidation-reduction process; P:D-amino acid metabolic process; F:D-amino-acid oxidase activity		
Acrdi.XM_015894217.1/1-795	Monca.augustus.g19170.t1/1-792	0.221995	0.196914	amiloride-sensitive sodium channel subunit alpha-like	1	P:GO:0006810	P:transport		
Acrdi.XM_015915418.1/1-912	Monca.augustus.g32678.t1/1-1686	0.113891	0.196906	PREDICTED: uncharacterized protein LOC107349294					
Acrdi.XM_015916900.1/1-1425	Monca.adi2mcaRNA27766_R0/1-1455	0.155683	0.196905	rho-associated kinase let-502-like	1	P:GO:0042981	P:regulation of apoptotic process		
Acrdi.XM_015898741.1/1-426	Monca.augustus.g2056.t1/1-600	0.244765	0.196887	UPF0488 C8orf33 homolog					
Acrdi.XM_015913713.1/1-2808	Monca.augustus.g21651.t1/1-3087	0.230638	0.196872	dispatched homolog 1 isoform X1	1	P:GO:0007275	P:multicellular organism development		
Acrdi.XM_015903038.1/1-1143	Monca.augustus.g48846.t1/1-3243	0.207837	0.196867	FAM193A isoform X1					
Acrdi.XM_015908262.1/1-1614	Monca.adi2mcaRNA18229_R0/1-1695	0.245098	0.196858	TNF receptor-associated factor 3-like	6	F:GO:0004842; F:GO:0008270; F:GO:0046872; P:GO:0007165;	F:ubiquitin-protein transferase activity; F:zinc ion binding; F:metal ion binding; P:signal transduction; P:regulation of apoptotic process; P:protein ubiquitination		
Acrdi.XM_015904098.1/1-1386	Monca.augustus.g44551.t1/1-1377	0.339321	0.196826	E3 ubiquitin- ligase TRIM71-like	3	F:GO:0008270; F:GO:0046872; C:GO:0005622	F:zinc ion binding; F:metal ion binding; C:intracellular		
Acrdi.XM_015911544.1/1-933	Monca.augustus.g45431.t1/1-951	0.183364	0.196807	adrenocorticotropic hormone receptor-like	11	F:GO:0004930; F:GO:0001588; F:GO:0008270; P:GO:0001963;	F:G-protein coupled receptor activity; F:dopamine neurotransmitter receptor activity, coupled via Gs; F:zinc ion binding; P:synaptic transmission, dopaminergic; P:G-protein couple[illegible]		
Acrdi.XM_015894122.1/1-8331	Monca.adi2mcaRNA40352_R0/1-8391	0.202335	0.196804	helicase with zinc finger domain 2-like	1	F:GO:0005488	F:binding		
Acrdi.XM_015895750.1/1-1197	Monca.augustus.g52642.t1/1-1845	0.387853	0.196746	Retrovirus-related Pol poly from transposon					
Acrdi.XM_015898255.1/1-1554	Monca.augustus.g45297.t1/1-1947	0.189803	0.196714	zinc finger 341-like isoform X1	1	F:GO:0005488	F:binding		
Acrdi.XM_015921338.1/1-858	Monca.adi2mcaRNA32681_R0/1-876	0.182867	0.196671	G2 M phase-specific E3 ubiquitin- ligase-like isoform X1					
Acrdi.XM_015897124.1/1-3507	Monca.augustus.g55697.t1/1-3882	0.276519	0.19665	PREDICTED: uncharacterized protein LOC107332390					
Acrdi.XM_015903719.1/1-1248	Monca.augustus.g16542.t1/1-849	0.193891	0.196544	Dynein assembly factor with WDR repeat domains 1					
Acrdi.XM_015924920.1/1-1392	Monca.augustus.g60900.t1/1-1707	0.212604	0.196393	methylcrotonoyl- carboxylase beta mitochondrial-like					
Acrdi.XM_015896124.1/1-864	Monca.augustus.g55598.t1/1-1263	0.227251	0.19637	U11 U12 small nuclear ribonucleo 48 kDa					
Acrdi.XM_015900578.1/1-660	Monca.augustus.g33651.t1/1-762	0.159102	0.196344	RWD domain-containing 1	3	C:GO:0005844; P:GO:0002181; F:GO:0003674	C:polysome; P:cytoplasmic translation; F:molecular_function		
Acrdi.XM_015899478.1/1-1167	Monca.augustus.g63374.t1/1-1155	0.203289	0.196314	retinal homeobox Rx	3	C:GO:0005634; P:GO:0006355; F:GO:0043565	C:nucleus; P:regulation of transcription, DNA-templated; F:sequence-specific DNA binding		
Acrdi.XM_015910315.1/1-4584	Monca.adi2mcaRNA20490_R0/1-3573	0.189493	0.196312	Lipoxygenase-like domain-containing 1	4	P:GO:0050982; F:GO:0005262; C:GO:0016020; P:GO:0070588	P:detection of mechanical stimulus; F:calcium channel activity; C:membrane; P:calcium ion transmembrane transport		
Acrdi.XM_015895152.1/1-1305	Monca.augustus.g65546.t1/1-1317	0.224381	0.196307	BTN1-like	2	C:GO:0016020; C:GO:0043227	C:membrane; C:membrane-bounded organelle		
Acrdi.XM_015917553.1/1-1002	Monca.augustus.g44189.t1/1-993	0.236098	0.196286	adrenocorticotropic hormone receptor-like	3	P:GO:0007154; P:GO:0044700; F:GO:0004871	P:cell communication; P:single organism signaling; F:signal transducer activity		
Acrdi.XM_015922809.1/1-1620	Monca.augustus.g374.t1/1-2088	0.180856	0.196245	potassium voltage-gated channel Shaw-like	3	P:GO:0071805; C:GO:0008076; F:GO:0005251	P:potassium ion transmembrane transport; C:voltage-gated potassium channel complex; F:delayed rectifier potassium channel activity		
Acrdi.XM_015923084.1/1-939	Monca.augustus.g70882.t1/1-948	0.202645	0.196222	histidine methyltransferase 1 homolog					
Acrdi.XM_015895251.1/1-615	Monca.adi2mcaRNA41672_R1/1-942	0.0984002	0.196222	Tetratricopeptide repeat 28					
Acrdi.XM_015908098.1/1-1173	Monca.augustus.g33236.t1/1-1173	0.236976	0.19622	doublesex- and mab-3-related transcription factor A2-like isoform X1	4	C:GO:0005634; F:GO:0003700; P:GO:0006355; F:GO:0043565	C:nucleus; F:transcription factor activity, sequence-specific DNA binding; P:regulation of transcription, DNA-templated; F:sequence-specific DNA binding		
Acrdi.XM_015909076.1/1-1113	Monca.augustus.g67747.t1/1-1101	0.236253	0.196162	proline-rich transmembrane 4-like	2	C:GO:0016020; C:GO:0016021	C:membrane; C:integral component of membrane		
Acrdi.XM_015906853.1/1-1089	Monca.augustus.g32955.t1/1-1455	0.256323	0.19616	NADPH:adrenodoxin mitochondrial	8	P:GO:0006091; F:GO:0005515; C:GO:0005759; P:GO:0055114;	P:generation of precursor metabolites and energy; F:protein binding; C:mitochondrial matrix; P:oxidation-reduction process; P:C21-steroid hormone biosynthetic process; P:ste[illegible]	EC:1.[illegible]	[illegible]
Acrdi.XM_015903589.1/1-555	Monca.augustus.g53519.t1/1-1623	0.241914	0.196146	Amyotrophic lateral sclerosis 2 chromosomal region candidate gene 1[illegible]	2	P:GO:0007155; F:GO:0005540	P:cell adhesion; F:hyaluronic acid binding		
Acrdi.XM_015891978.1/1-1815	Monca.augustus.g66333.t1/1-1911	0.221396	0.196135	Major facilitator superfamily domain-containing 12	1	C:GO:0016020	C:membrane		
Acrdi.XM_015910531.1/1-996	Monca.augustus.g36041.t1/1-984	0.291906	0.196096	chromobox homolog 2	1	C:GO:0005634	C:nucleus		
Acrdi.XM_015913083.1/1-1158	Monca.augustus.g25058.t1/1-1326	0.180527	0.195955	beta-1,4-N-acetylgalactosaminyltransferase 3-like	3	C:GO:0032580; F:GO:0008376; F:GO:0016740	C:Golgi cisterna membrane; F:acetylgalactosaminyltransferase activity; F:transferase activity		
Acrdi.XM_015904159.1/1-387	Monca.augustus.g32316.t1/1-651	0.276788	0.195939	Transmembrane protease serine 5					
Acrdi.XM_015896079.1/1-3030	Monca.augustus.g15120.t1/1-3588	0.161175	0.195932	argonaute-2-like isoform X2	6	F:GO:0035198; C:GO:0030529; P:GO:0031054; P:GO:0043363;	F:miRNA binding; C:intracellular ribonucleoprotein complex; P:pre-miRNA processing; P:nucleate erythrocyte differentiation; F:nuclease activity; F:pre-miRNA binding		
Acrdi.XM_015898627.1/1-1320	Monca.adi2mcaRNA7641_R3/1-1329	0.229284	0.195826	solute carrier family 15 member 4-like	5	P:GO:0006857; C:GO:0016020; C:GO:0016021; P:GO:0006810;	P:oligopeptide transport; C:membrane; C:integral component of membrane; P:transport; F:transporter activity		
Acrdi.XM_015895184.1/1-1779	Monca.adi2mcaRNA41597_R0/1-1884	0.191885	0.195806	PREDICTED: uncharacterized protein LOC107330606	2	C:GO:0016020; C:GO:0016021	C:membrane; C:integral component of membrane		
Acrdi.XM_015891702.1/1-1161	Monca.adi2mcaRNA37553_R1/1-1128	0.168811	0.195806	peritrophic membrane	3	F:GO:0008061; P:GO:0006030; C:GO:0005576	F:chitin binding; P:chitin metabolic process; C:extracellular region		
Acrdi.XM_015898712.1/1-816	Monca.augustus.g54237.t1/1-978	0.243139	0.195797	E3 ubiquitin- ligase RNF13	5	F:GO:0008270; F:GO:0046872; C:GO:0016020; C:GO:0016021;	F:zinc ion binding; F:metal ion binding; C:membrane; C:integral component of membrane; F:ligase activity		
Acrdi.XM_015916154.1/1-489	Monca.augustus.g25394.t1/1-486	0.151237	0.195748	PREDICTED: uncharacterized protein LOC107349955					
Acrdi.XM_015908721.1/1-654	Monca.augustus.g3907.t1/1-633	0.886552	0.195703	YTH domain-containing 1-like					
Acrdi.XM_015905982.1/1-1554	Monca.augustus.g41414.t1/1-2040	0.201719	0.195681	sodium-dependent neutral amino acid transporter B(0)AT3-like	3	F:GO:0015075; C:GO:0016021; P:GO:0006810	F:ion transmembrane transporter activity; C:integral component of membrane; P:transport		

Acrdi.XM_015913561.1/1-3939 | Monca.augustus.g22351.t1/1-3951	0.268021	0.195627	BCL-6 corepressor 1 isoform X3	
Acrdi.XM_015920427.1/1-714 | Monca.adi2mcaRNA31712_R1/1-642	0.179779	0.195617	PREDICTED: uncharacterized protein LOC107354026	5
Acrdi.XM_015917892.1/1-1203 | Monca.augustus.g14687.t1/1-1533	0.199854	0.195592	F-box only 15-like	
Acrdi.XM_015910509.1/1-498 | Monca.augustus.g25429.t1/1-4290	0.174465	0.195592	PREDICTED: uncharacterized protein LOC107344821	1
Acrdi.XM_015901807.1/1-1143 | Monca.augustus.g310.t1/1-1389	0.136312	0.195584	egl nine homolog 1	
Acrdi.XM_015900495.1/1-534 | Monca.augustus.g26464.t1/1-642	0.173739	0.195582	transmembrane 249-like	2
Acrdi.XM_015910592.1/1-918 | Monca.adi2mcaRNA20786_R0/1-921	0.192083	0.19558	G- coupled receptor 54-like isoform X1	2
Acrdi.XM_015917105.1/1-1566 | Monca.augustus.g1140.t1/1-1587	0.204605	0.195571	major facilitator superfamily domain-containing 6-like	2
Acrdi.XM_015921020.1/1-915 | Monca.augustus.g1380.t1/1-909	0.238479	0.19555	secreted frizzled-related 3	1
Acrdi.XM_015903674.1/1-585 | Monca.adi2mcaRNA13094_R1/1-579	0.163442	0.195501	biogenesis of lysosome-related organelles complex 1 subunit 3-like	2
Acrdi.XM_015899655.1/1-1332 | Monca.adi2mcaRNA809_R0/1-1332	0.272782	0.195481	mannose-binding lectin associated serine protease-1	1
Acrdi.XM_015911505.1/1-1707 | Monca.augustus.g49535.t1/1-1710	0.160369	0.195404	AP-1 complex subunit gamma-1	
Acrdi.XM_015911381.1/1-3147 | Monca.adi2mcaRNA21638_R0/1-2973	0.165418	0.195373	conserved oligomeric Golgi complex subunit 4 isoform X1	7
Acrdi.XM_015900009.1/1-2646 | Monca.augustus.g43407.t1/1-7968	0.243957	0.195344	PREDICTED: uncharacterized protein LOC107335035	
Acrdi.XM_015901329.1/1-774 | Monca.augustus.g48493.t1/1-810	0.208973	0.195329	fructose-2,6-bisphosphatase TIGAR	9
Acrdi.XM_015904352.1/1-612 | Monca.augustus.g68751.t1/1-588	0.158187	0.195306	riboflavin-binding -like	3
Acrdi.XM_015898369.1/1-1464 | Monca.augustus.g68151.t1/1-1728	0.262392	0.195293	RNA helicase	5
Acrdi.XM_015913544.1/1-471 | Monca.augustus.g22344.t1/1-420	0.232687	0.195251	profilin-like isoform X3	4
Acrdi.XM_015915341.1/1-546 | Monca.augustus.g25299.t1/1-552	0.237783	0.195228	thioredoxin AAED1	
Acrdi.XM_015893821.1/1-1707 | Monca.augustus.g29471.t1/1-2052	0.173109	0.195207	family C2 unassigned peptidase (C02 family)	2
Acrdi.XM_015892488.1/1-342 | Monca.adi2mcaRNA3829_R0/1-423	0.125219	0.195164	PREDICTED: uncharacterized protein LOC107327740	
Acrdi.XM_015895850.1/1-4065 | Monca.adi2mcaRNA4632_R1/1-5166	0.216345	0.195149	nidogen-1-like isoform X4	3
Acrdi.XM_015915871.1/1-1905 | Monca.augustus.g39031.t1/1-2250	0.208009	0.195123	Nitric oxide-associated 1	4
Acrdi.XM_015912070.1/1-1155 | Monca.augustus.g67650.t1/1-1155	0.2337	0.195105	Serine protease 23	1
Acrdi.XM_015910873.1/1-1896 | Monca.augustus.g3118.t1/1-1695	0.159364	0.195093	nicalin isoform X4	3
Acrdi.XM_015916977.1/1-765 | Monca.adi2mcaRNA27858_R0/1-549	0.248395	0.195069	Golgi integral membrane 4-like	
Acrdi.XM_015899892.1/1-3258 | Monca.adi2mcaRNA9033_R0/1-3138	0.157386	0.195038	glycerol-3-phosphate acyltransferase mitochondrial isoform X1	2
Acrdi.XM_015923555.1/1-876 | Monca.augustus.g15156.t1/1-891	0.272904	0.195006	phytanoyl- dioxygenase	2
Acrdi.XM_015892676.1/1-3384 | Monca.augustus.g19484.t1/1-4044	0.214039	0.194951	Hermansky-Pudlak syndrome 3	
Acrdi.XM_015898496.1/1-834 | Monca.augustus.g39446.t1/1-861	0.258898	0.194926	sodium potassium-transporting ATPase subunit beta-2	5
Acrdi.XM_015895582.1/1-7224 | Monca.adi2mcaRNA4393_R0/1-6429	0.202398	0.194913	lipopolysaccharide-responsive and beige-like anchor isoform X2	
Acrdi.XM_015923617.1/1-852 | Monca.augustus.g67299.t1/1-615	0.12598	0.194849	Transposon Ty3-G Gag-Pol poly	
Acrdi.XM_015909099.1/1-684 | Monca.adi2mcaRNA19121_R0/1-696	0.404785	0.194824	contactin-3-like isoform X1	2
Acrdi.XM_015903211.1/1-939 | Monca.augustus.g51696.t1/1-957	0.194516	0.194771	amino acid oxidase	5
Acrdi.XM_015908704.1/1-789 | Monca.augustus.g66252.t1/1-1494	0.142652	0.194768	NAD-dependent deacetylase sirtuin-6	20
[illegible]				

Acrdi.XM_015913845.1/1-1494 | Monca.adi2mcaRNA24323_R8/1-1293	0.175802	0.187746	mucin	3	P:GO:0007160; F:GO:0005509; C:GO:0016020	P:cell-matrix adhesion; F:calcium ion binding; C:membrane		
Acrdi.XM_015913729.1/1-2706 | Monca.augustus.g21659.t1/1-2790	0.259149	0.18774	ankyrin repeat domain-containing 55-like					
Acrdi.XM_015897086.1/1-837 | Monca.adi2mcaRNA640_R1/1-804	0.673078	0.187702	ATP-dependent DNA helicase -like	2	P:GO:0006259; F:GO:0016787	P:DNA metabolic process; F:hydrolase activity		
Acrdi.XM_015901089.1/1-633 | Monca.augustus.g25256.t1/1-639	0.127102	0.187676	fibroblast growth factor 2	2	P:GO:0048522; F:GO:0005488	P:positive regulation of cellular process; F:binding		
Acrdi.XM_015905216.1/1-1104 | Monca.augustus.g52838.t1/1-1128	0.217102	0.187643	myb SANT-like DNA-binding domain-containing 4	2	C:GO:0005634; P:GO:0006915	C:nucleus; P:apoptotic process		
Acrdi.XM_015898025.1/1-759 | Monca.augustus.g28147.t1/1-750	0.226863	0.187531	bcl-2-associated transcription factor 1-like					
Acrdi.XM_015901844.1/1-2193 | Monca.augustus.g58309.t1/1-2811	0.219781	0.187449	PREDICTED: uncharacterized protein LOC107336780	5	F:GO:0005524; C:GO:0005634; C:GO:0005737; C:GO:0016020;	F:ATP binding; C:nucleus; C:cytoplasm; C:membrane; C:integral component of membrane		
Acrdi.XM_015902071.1/1-966 | Monca.augustus.g56090.t1/1-1287	0.282427	0.187423	tRNA (cytosine(38)-C(5))-methyltransferase	2	F:GO:0008168; P:GO:0032259	F:methyltransferase activity; P:methylation		
Acrdi.XM_015923445.1/1-1107 | Monca.adi2mcaRNA35114_R0/1-1407	0.0970212	0.187372	mediator of RNA polymerase II transcription subunit 34-like	4	F:GO:0003676; C:GO:0043229; P:GO:0006281; F:GO:0003678	F:nucleic acid binding; C:intracellular organelle; P:DNA repair; F:DNA helicase activity	EC:3.[illegible]	[illegible]
Acrdi.XM_015907157.1/1-1110 | Monca.augustus.g2516.t1/1-1110	0.251188	0.187359	substance-K receptor-like	2	P:GO:0007165; C:GO:0016020	P:signal transduction; C:membrane		
Acrdi.XM_015910406.1/1-1254 | Monca.augustus.g26327.t1/1-1275	0.14473	0.187354	monocarboxylate transporter 10-like	1	P:GO:0006810	P:transport		
Acrdi.XM_015897779.1/1-1011 | Monca.augustus.g65421.t1/1-996	0.217885	0.187299	melanocyte-stimulating hormone receptor-like	3	F:GO:0004930; P:GO:0007154; P:GO:0044700	F:G-protein coupled receptor activity; P:cell communication; P:single organism signaling		
Acrdi.XM_015907280.1/1-1050 | Monca.augustus.g28935.t1/1-1125	0.134926	0.187257	phosphatidate phosphatase LPIN1 isoform X1	9	F:GO:0008195; C:GO:0005634; F:GO:0003755; P:GO:0009062;	F:phosphatidate phosphatase activity; C:nucleus; F:peptidyl-prolyl cis-trans isomerase activity; P:fatty acid catabolic process; F:transcription coactivator activity; P:triglyceride [illegible]	EC:5.[illegible]	[illegible]
Acrdi.XM_015902840.1/1-618 | Monca.augustus.g27204.t1/1-2511	0.188756	0.187252	Bromodomain testis-specific					
Acrdi.XM_015917831.1/1-1422 | Monca.augustus.g54900.t1/1-1422	0.150023	0.187237	galanin receptor type 1-like	6	C:GO:0005887; F:GO:0004930; P:GO:0010033; P:GO:0007186;	C:integral component of plasma membrane; F:G-protein coupled receptor activity; P:response to organic substance; P:G-protein coupled receptor signaling pathway; P:response to [illegible]		
Acrdi.XM_015914263.1/1-2181 | Monca.adi2mcaRNA24804_R2/1-2124	0.1963	0.187233	endothelin-converting enzyme 1 isoform X2	6	P:GO:0006508; C:GO:0016020; C:GO:0016021; P:GO:0008355;	P:proteolysis; C:membrane; C:integral component of membrane; P:olfactory learning; F:metalloendopeptidase activity; F:metallopeptidase activity		
Acrdi.XM_015923014.1/1-540 | Monca.augustus.g46076.t1/1-537	0.173754	0.187189	histone H1-delta-like	1	C:GO:0005694	C:chromosome		
Acrdi.XM_015904101.1/1-582 | Monca.augustus.g44554.t1/1-570	0.237802	0.187094	peptidyl-tRNA hydrolase mitochondrial-like	7	F:GO:0016150; P:GO:0072344; F:GO:0043022; P:GO:0070126;	F:translation release factor activity, codon nonspecific; P:rescue of stalled ribosome; F:ribosome binding; P:mitochondrial translational termination; F:aminoacyl-tRNA hydrol[illegible]	EC:3.[illegible]	[illegible]
Acrdi.XM_015911403.1/1-4833 | Monca.augustus.g44961.t1/1-5859	0.227044	0.187048	dynamin-binding isoform X1	4	P:GO:0035023; C:GO:0005737; F:GO:0005089; P:GO:0043547	P:regulation of Rho protein signal transduction; C:cytoplasm; F:Rho guanyl-nucleotide exchange factor activity; P:positive regulation of GTPase activity		
Acrdi.XM_015924852.1/1-1149 | Monca.augustus.g37686.t1/1-1920	0.18633	0.186978	eyes					
Acrdi.XM_015925448.1/1-2379 | Monca.adi2mcaRNA3746_R0/1-2376	0.211747	0.186968	ranBP-type and C3HC4-type zinc finger-containing 1-like	1	F:GO:0008270	F:zinc ion binding		
Acrdi.XM_015901909.1/1-1188 | Monca.augustus.g58295.t1/1-1527	0.215546	0.186948	jmjC domain-containing 7-like					
Acrdi.XM_015901010.1/1-588 | Monca.augustus.g57622.t1/1-768	0.252166	0.186918	leucine-rich repeat-containing 46					
Acrdi.XM_015900010.1/1-1494 | Monca.augustus.g51838.t1/1-1500	0.16991	0.18686	PREDICTED: uncharacterized protein LOC107335039					
Acrdi.XM_015911043.1/1-1173 | Monca.adi2mcaRNA21262_R2/1-1170	0.186287	0.186846	arachidonate 5-lipoxygenase-like	4	F:GO:0016702; F:GO:0016491; F:GO:0046872; P:GO:0055114	F:oxidoreductase activity, acting on single donors with incorporation of molecular oxygen, incorporation of two atoms of oxygen; F:oxidoreductase activity; F:metal ion binding; [illegible]		
Acrdi.XM_015905671.1/1-1692 | Monca.augustus.g26831.t1/1-1692	0.173588	0.186843	Kelch diablo	7	F:GO:0004842; P:GO:0008584; C:GO:0005737; P:GO:0007286;	F:ubiquitin-protein transferase activity; P:male gonad development; C:cytoplasm; P:spermatid development; P:fertilization; C:Cul3-RING ubiquitin ligase complex; P:protein ubiqui[illegible]		
Acrdi.XM_015899485.1/1-633 | Monca.augustus.g37228.t1/1-621	0.176211	0.186832	PREDICTED: uncharacterized protein LOC107334544	1	F:GO:0046872	F:metal ion binding		
Acrdi.XM_015908280.1/1-4476 | Monca.adi2mcaRNA18251_R0/1-3957	0.189329	0.186822	PREDICTED: uncharacterized protein LOC107342772 isoform X17					
Acrdi.XM_015903114.1/1-1944 | Monca.augustus.g52458.t1/1-4842	0.256284	0.186798	kinesin KIF14	1	F:GO:0017111	F:nucleoside-triphosphatase activity	EC:3.[illegible]	[illegible]
Acrdi.XM_015916415.1/1-1929 | Monca.augustus.g64483.t1/1-1683	0.17758	0.186776	PREDICTED: uncharacterized protein LOC107350192	5	C:GO:0016020; C:GO:0016021; P:GO:0036071; P:GO:0006487;	C:membrane; C:integral component of membrane; P:N-glycan fucosylation; P:protein N-linked glycosylation; F:alpha-(1->6)-fucosyltransferase activity		
Acrdi.XM_015917395.1/1-390 | Monca.augustus.g23805.t1/1-519	0.170047	0.186766	diamine acetyltransferase 2	7	P:GO:0032917; P:GO:0009445; F:GO:0005488; F:GO:0008080;	P:polyamine acetylation; P:putrescine metabolic process; F:binding; F:N-acetyltransferase activity; P:polyamine catabolic process; P:spermidine metabolic process; P:spermine me[illegible]		
Acrdi.XM_015914492.1/1-360 | Monca.adi2mcaRNA25054_R0/1-351	0.217114	0.186764	acetylcholine receptor subunit alpha	2	P:GO:0006811; C:GO:0016020	P:ion transport; C:membrane		
Acrdi.XM_015907914.1/1-459 | Monca.augustus.g35196.t1/1-984	0.122969	0.186764	transmembrane adipocyte-associated 1 homolog	2	C:GO:0016020; C:GO:0016021	C:membrane; C:integral component of membrane		
Acrdi.XM_015911860.1/1-1935 | Monca.augustus.g17909.t1/1-1923	0.216654	0.18667	E3 ubiquitin- ligase TRIM71	5	F:GO:0008270; F:GO:0046872; P:GO:0042981; F:GO:0016874;	F:zinc ion binding; F:metal ion binding; P:regulation of apoptotic process; F:ligase activity; C:intracellular		
Acrdi.XM_015901321.1/1-537 | Monca.adi2mcaRNA10572_R0/1-483	0.25059	0.186663	phospholipase A2	7	F:GO:0004623; F:GO:0046872; F:GO:0005509; P:GO:0006629;	F:phospholipase A2 activity; F:metal ion binding; F:calcium ion binding; P:lipid metabolic process; P:lipid catabolic process; C:extracellular region; F:hydrolase activity		
Acrdi.XM_015919454.1/1-2442 | Monca.augustus.g25234.t1/1-2748	0.181527	0.186561	glutamate receptor NMDA 2B-like	16	P:GO:0007165; F:GO:0005261; F:GO:0043167; F:GO:0005230;	P:signal transduction; F:cation channel activity; F:ion binding; F:extracellular ligand-gated ion channel activity; P:response to chemical; P:single-organism cellular process; C:plasm[illegible]		
Acrdi.XM_015903388.1/1-1215 | Monca.augustus.g18806.t1/1-1035	0.206108	0.186537	probable G- coupled receptor No9	3	P:GO:0007165; C:GO:0016020; F:GO:0004871	P:signal transduction; C:membrane; F:signal transducer activity		
Acrdi.XM_015921140.1/1-1005 | Monca.augustus.g29196.t1/1-1014	0.134033	0.186531	Alpha-1A adrenergic receptor	7	F:GO:0004930; P:GO:0007186; P:GO:0051240; C:GO:0016020;	F:G-protein coupled receptor activity; P:G-protein coupled receptor signaling pathway; P:positive regulation of multicellular organismal process; C:membrane; P:regulation of syst[illegible]		
Acrdi.XM_015905687.1/1-438 | Monca.adi2mcaRNA15333_R0/1-432	0.302308	0.186505	centrosomal of 170 kDa B-like	3	C:GO:0031965; F:GO:0005515; C:GO:0005814	C:nuclear membrane; F:protein binding; C:centriole		
Acrdi.XM_015893803.1/1-1203 | Monca.augustus.g13568.t1/1-1185	0.216722	0.186486	Proliferation-associated 2G4	1	F:GO:0016787	F:hydrolase activity		
Acrdi.XM_015914167.1/1-378 | Monca.augustus.g37415.t1/1-801	0.262056	0.186483	PREDICTED: uncharacterized protein LOC107348150	2	C:GO:0016020; C:GO:0016021	C:membrane; C:integral component of membrane		
Acrdi.XM_015921395.1/1-753 | Monca.augustus.g11104.t1/1-906	0.158744	0.186458	MAP7 domain-containing 2-like					
Acrdi.XM_015899859.1/1-2124 | Monca.augustus.g60331.t1/1-2178	0.218962	0.186403	DDB1- and CUL4-associated factor 5	2	P:GO:0008150; F:GO:0003674	P:biological_process; F:molecular_function		
Acrdi.XM_015924287.1/1-1431 | Monca.augustus.g27641.t1/1-1467	0.191362	0.186386	potassium voltage-gated channel subfamily A member 2-like	3	F:GO:0005249; C:GO:0016021; P:GO:0006810	F:voltage-gated potassium channel activity; C:integral component of membrane; P:transport		
Acrdi.XM_015897330.1/1-672 | Monca.augustus.g54239.t1/1-906	0.183192	0.186384	homocysteine S-methyltransferase -like	3	F:GO:0008168; F:GO:0016740; P:GO:0032259	F:methyltransferase activity; F:transferase activity; P:methylation		
Acrdi.XM_015908494.1/1-2898 | Monca.augustus.g15580.t1/1-2916	0.186715	0.186349	fanconi-associated nuclease 1					
Acrdi.XM_015908753.1/1-1197 | Monca.augustus.g26275.t1/1-1248	0.262747	0.186281	TFG isoform X1					
Acrdi.XM_015894838.1/1-1116 | Monca.adi2mcaRNA41172_R0/1-1056	0.183982	0.186279	artemis isoform X1	2	P:GO:0006259; P:GO:0006974	P:DNA metabolic process; P:cellular response to DNA damage stimulus		
Acrdi.XM_015904135.1/1-966 | Monca.augustus.g36722.t1/1-960	0.222353	0.186265	Deoxyguanosine kinase	3	F:GO:0005524; P:GO:0009165; F:GO:0019206	F:ATP binding; P:nucleotide biosynthetic process; F:nucleoside kinase activity		
Acrdi.XM_015916239.1/1-915 | Monca.augustus.g40333.t1/1-924	0.228101	0.186244	octopamine receptor-like	3	F:GO:0001588; P:GO:0001963; F:GO:0035240	F:dopamine neurotransmitter receptor activity, coupled via Gs; P:synaptic transmission, dopaminergic; F:dopamine binding		
Acrdi.XM_015900570.1/1-564 | Monca.augustus.g14891.t1/1-483	0.225117	0.186169	PREDICTED: uncharacterized protein LOC107335553					
Acrdi.XM_015910388.1/1-4032 | Monca.augustus.g10944.t1/1-4038	0.223562	0.186158	CASK interacting isoform CRA_b					
[illegible]	[illegible]	[illegible]	[illegible]	[illegible]	[illegible]	[illegible]	[illegible]	[illegible]

Acrdi.XM_015923497.1/1-1446 | Monca.adi2mcaRNA35167_R0/1-1278	0.223252	0.179755	L-asparaginase-like isoform X2	2	P:GO:0006520; F:GO:0004067	P:cellular amino acid metabolic process; F:asparaginase activity
Acrdi.XM_015897070.1/1-864 | Monca.augustus.g51934.t1/1-879	0.217862	0.179754	probable G- coupled receptor No9	9	F:GO:0004930; F:GO:0001588; P:GO:0001963; P:GO:0007186; [illegible]	F:G-protein coupled receptor activity; F:dopamine neurotransmitter receptor activity, coupled via Gs; P:synaptic transmission, dopaminergic; P:G-protein coupled receptor signalin[illegible]
Acrdi.XM_015923960.1/1-1329 | Monca.augustus.g62274.t1/1-1341	0.200274	0.179744	serine incorporator 1	9	C:GO:0005789; P:GO:0006658; P:GO:1904222; C:GO:0016021; [illegible]	C:endoplasmic reticulum membrane; P:phosphatidylserine metabolic process; P:positive regulation of serine C-palmitoyltransferase activity; C:integral component of membrane; [illegible]
Acrdi.XM_015916929.1/1-870 | Monca.augustus.g38700.t1/1-870	0.231157	0.179737	alpha-1A adrenergic receptor-like	5	F:GO:0004935; P:GO:0007165; C:GO:0016021; F:GO:0043169; [illegible]	F:adrenergic receptor activity; P:signal transduction; C:integral component of membrane; F:cation binding; C:plasma membrane
Acrdi.XM_015906555.1/1-1113 | Monca.augustus.g31414.t1/1-1092	0.251799	0.179657	PREDICTED: uncharacterized protein LOC107341137			
Acrdi.XM_015901979.1/1-552 | Monca.augustus.g69323.t1/1-549	0.103347	0.179635	1,2-dihydroxy-3-keto-5-methylthiopentene dioxygenase	6	F:GO:0051213; C:GO:0005634; C:GO:0005737; F:GO:0046872; [illegible]	F:dioxygenase activity; C:nucleus; C:cytoplasm; F:metal ion binding; P:L-methionine salvage from methylthioadenosine; C:plasma membrane
Acrdi.XM_015918904.1/1-9267 | Monca.augustus.g48519.t1/1-6639	0.205884	0.179606	mucin-4-like isoform X2	8	P:GO:0007160; F:GO:0005509; C:GO:0016020; C:GO:0016021; [illegible]	P:cell-matrix adhesion; F:calcium ion binding; C:membrane; C:integral component of membrane; F:scavenger receptor activity; F:extracellular matrix structural constituent; [illegible]
Acrdi.XM_015907298.1/1-657 | Monca.augustus.g20599.t1/1-708	0.227023	0.179587	Coiled-coil domain-containing 103	1	P:GO:0007275	P:multicellular organism development
Acrdi.XM_015907680.1/1-2361 | Monca.adi2mcaRNA17557_R0/1-2070	0.193976	0.17957	Coiled-coil domain-containing 158			
Acrdi.XM_015902882.1/1-2205 | Monca.augustus.g27556.t1/1-2196	0.12357	0.179559	UV-stimulated scaffold A			
Acrdi.XM_015896898.1/1-1029 | Monca.augustus.g33197.t1/1-978	0.145875	0.17955	PREDICTED: uncharacterized protein LOC107332178 isoform X1	2	C:GO:0016020; C:GO:0016021	C:membrane; C:integral component of membrane
Acrdi.XM_015900336.1/1-534 | Monca.adi2mcaRNA9608_R9/1-492	0.0469933	0.179532	PREDICTED: uncharacterized protein LOC107335333			
Acrdi.XM_015922585.1/1-3591 | Monca.augustus.g62036.t1/1-5169	0.141714	0.179484	nitric oxide brain	18	C:GO:0043229; P:GO:0065008; P:GO:0045935; F:GO:1901363; [illegible]	C:intracellular organelle; P:regulation of biological quality; P:positive regulation of nucleobase-containing compound metabolic process; F:heterocyclic compound binding; [illegible]
Acrdi.XM_015906920.1/1-1149 | Monca.augustus.g49151.t1/1-1146	0.213313	0.17946	Nuclear cap-binding subunit 3	1	F:GO:0003723	F:RNA binding
Acrdi.XM_015924452.1/1-978 | Monca.augustus.g11013.t1/1-1206	0.175298	0.17936	cubilin			
Acrdi.XM_015898533.1/1-639 | Monca.augustus.g34001.t1/1-2538	0.170476	0.179321	chromatin assembly factor 1 subunit A	3	C:GO:0005634; C:GO:0033186; P:GO:0006334	C:nucleus; C:CAF-1 complex; P:nucleosome assembly
Acrdi.XM_015901385.1/1-999 | Monca.augustus.g17230.t1/1-996	0.222469	0.179315	fibroblast growth factor receptor-like 1	8	F:GO:0000166; F:GO:0004713; F:GO:0097367; C:GO:0016020; [illegible]	F:nucleotide binding; F:protein tyrosine kinase activity; F:carbohydrate derivative binding; C:membrane; P:anatomical structure development; F:anion binding; P:protein phosp[illegible]
Acrdi.XM_015898931.1/1-1191 | Monca.augustus.g7900.t1/1-1092	0.177104	0.179265	Carbohydrate sulfotransferase 1	2	P:GO:0008152; F:GO:0016740	P:metabolic process; F:transferase activity
Acrdi.XM_015896176.1/1-2424 | Monca.adi2mcaRNA5050_R0/1-1932	0.199456	0.179264	Regulator of G- signaling -like	1	C:GO:0044464	C:cell part
Acrdi.XM_015917279.1/1-3261 | Monca.augustus.g45705.t1/1-6522	0.226944	0.179252	Dedicator of cytokinesis			
Acrdi.XM_015914343.1/1-2898 | Monca.adi2mcaRNA2507_R0/1-2973	0.258253	0.1792	subfamily C member 14			
Acrdi.XM_015897350.1/1-333 | Monca.augustus.g51731.t1/1-339	0.210804	0.179172	PREDICTED: uncharacterized protein LOC107332617			
Acrdi.XM_015915459.1/1-6327 | Monca.augustus.g17210.t1/1-4422	0.23557	0.179148	PREDICTED: uncharacterized protein LOC107349325			
Acrdi.XM_015918047.1/1-621 | Monca.augustus.g34655.t1/1-651	0.271478	0.179142	RING finger 166	1	F:GO:0005488	F:binding
Acrdi.XM_015904834.1/1-639 | Monca.augustus.g16528.t1/1-552	0.0973282	0.179138	proline-rich 11			
Acrdi.XM_015914098.1/1-1566 | Monca.augustus.g8024.t1/1-1194	0.174947	0.17913	UDP-sugar transporter DDB_G0278631 isoform X5	4	F:GO:0005338; P:GO:1901264; C:GO:0016020; P:GO:0015931	F:nucleotide-sugar transmembrane transporter activity; P:carbohydrate derivative transport; C:membrane; P:nucleobase-containing compound transport
Acrdi.XM_015910246.1/1-450 | Monca.augustus.g25484.t1/1-732	0.226794	0.179126	Testican-3	3	P:GO:0007165; F:GO:0005509; C:GO:0005578	P:signal transduction; F:calcium ion binding; C:proteinaceous extracellular matrix
Acrdi.XM_015923652.1/1-963 | Monca.augustus.g39999.t1/1-963	0.155089	0.179092	proline-rich transmembrane 4-like	2	C:GO:0016020; C:GO:0016021	C:membrane; C:integral component of membrane
Acrdi.XM_015910237.1/1-1956 | Monca.augustus.g25482.t1/1-1938	0.243851	0.179048	peroxidasin homolog			
Acrdi.XM_015901729.1/1-2877 | Monca.augustus.g34403.t1/1-2898	0.221216	0.178953	PREDICTED: uncharacterized protein LOC107336659			
Acrdi.XM_015915404.1/1-1965 | Monca.augustus.g56853.t1/1-2100	0.185829	0.178944	complement factor B precursor	1	F:GO:0008233	F:peptidase activity
Acrdi.XM_015917529.1/1-1851 | Monca.adi2mcaRNA28469_R0/1-1728	0.178068	0.178935	pseudouridine-metabolizing bifunctional -like isoform X2	3	F:GO:0016773; C:GO:0005575; F:GO:0016798	F:phosphotransferase activity, alcohol group as acceptor; C:cellular_component; F:hydrolase activity, acting on glycosyl bonds
Acrdi.XM_015924554.1/1-1053 | Monca.augustus.g26865.t1/1-1023	0.204613	0.17889	adrenocorticotropic hormone receptor-like	9	F:GO:0004930; F:GO:0001588; F:GO:0008270; P:GO:0001963; [illegible]	F:G-protein coupled receptor activity; F:dopamine neurotransmitter receptor activity, coupled via Gs; F:zinc ion binding; P:synaptic transmission, dopaminergic; P:G-protein couple[illegible]
Acrdi.XM_015906243.1/1-756 | Monca.augustus.g21672.t1/1-753	0.252366	0.178799	N-acetylglucosaminyl-phosphatidylinositol de-N-acetylase	2	C:GO:0016020; C:GO:0016021	C:membrane; C:integral component of membrane
Acrdi.XM_015899393.1/1-723 | Monca.augustus.g35509.t1/1-885	0.271423	0.178792	alpha-N-acetyl-neuraminyl-2,3-beta-galactosyl-1,3-N-acetyl-galactosa	1	P:GO:0071704	P:organic substance metabolic process
[illegible]	[illegible]	[illegible]	[illegible]	[illegible]	[illegible]	[illegible]

Acrdi.XM_015897166.1/1-1962 | Monca.augustus.g31743.t1/1-2355	0.238642	0.172427	phosphatase 1 regulatory subunit 21	2	F:GO:0019902; C:GO:0016020	F:phosphatase binding; C:membrane		
Acrdi.XM_015899470.1/1-1215 | Monca.augustus.g37241.t1/1-1212	0.243108	0.172413	glucoside xylosyltransferase 1	2	F:GO:0016757; F:GO:0016740	F:transferase activity, transferring glycosyl groups; F:transferase activity		
Acrdi.XM_015902916.1/1-1050 | Monca.augustus.g54834.t1/1-1041	0.185809	0.172391	synaptotagmin-16-like isoform X1	1	F:GO:0005488	F:binding		
Acrdi.XM_015897714.1/1-762 | Monca.augustus.g56103.t1/1-2328	0.228705	0.172347	kelch 12	5	C:GO:0043229; P:GO:0016192; C:GO:0044444; P:GO:0009987;	C:intracellular organelle; P:vesicle-mediated transport; C:cytoplasmic part; P:cellular process; C:membrane-bounded organelle		
Acrdi.XM_015916363.1/1-1188 | Monca.augustus.g40941.t1/1-1041	0.197464	0.172335	TBC1 domain family member 2B	2	P:GO:0065007; C:GO:0044464	P:biological regulation; C:cell part		
Acrdi.XM_015914825.1/1-399 | Monca.augustus.g33248.t1/1-564	0.29085	0.172317	Phosphatidylinositol N-acetylglucosaminyltransferase subunit H	5	F:GO:0016757; C:GO:0016020; C:GO:0016021; F:GO:0017176;	F:transferase activity, transferring glycosyl groups; C:membrane; C:integral component of membrane; F:phosphatidylinositol N-acetylglucosaminyltransferase activity; F:transferase activity		
Acrdi.XM_015908320.1/1-7035 | Monca.adi2mcaRNA18307_R0/1-7329	0.173422	0.172314	Collagen alpha-6(VI) chain					
Acrdi.XM_015911038.1/1-1863 | Monca.augustus.g3793.t1/1-1872	0.166959	0.172308	PREDICTED: uncharacterized protein LOC107345335					
Acrdi.XM_015902262.1/1-2850 | Monca.augustus.g63803.t1/1-2937	0.216252	0.172293	unconventional myosin-XIX	5	F:GO:0000166; C:GO:0016459; F:GO:0005524; F:GO:0003779;	F:nucleotide binding; C:myosin complex; F:ATP binding; F:actin binding; F:motor activity		
Acrdi.XM_015897805.1/1-852 | Monca.adi2mcaRNA6831_R0/1-999	0.483847	0.172249	Retrovirus-related Pol poly from transposon					
Acrdi.XM_015908101.1/1-921 | Monca.augustus.g33241.t1/1-915	0.207357	0.172239	FAM26F-like isoform X1	2	P:GO:0034220; C:GO:0016021	P:ion transmembrane transport; C:integral component of membrane		
Acrdi.XM_015898899.1/1-1086 | Monca.augustus.g7902.t1/1-1134	0.192109	0.172208	BRCA1-A complex subunit BRE					
Acrdi.XM_015906288.1/1-792 | Monca.augustus.g26534.t1/1-792	0.0849348	0.172121	14-3-3 zeta	1	F:GO:0019904	F:protein domain specific binding		
Acrdi.XM_015905931.1/1-1455 | Monca.augustus.g58509.t1/1-1446	0.178055	0.172088	ZP domain-containing -like	5	F:GO:0005509; C:GO:0016020; C:GO:0016021; F:GO:0005044;	F:calcium ion binding; C:membrane; C:integral component of membrane; F:scavenger receptor activity; P:receptor-mediated endocytosis		
Acrdi.XM_015924369.1/1-330 | Monca.augustus.g30190.t1/1-333	0.0356995	0.17208	Tubulin-specific chaperone A	8	C:GO:0070062; P:GO:0007021; C:GO:0005737; F:GO:0048487;	C:extracellular exosome; P:tubulin complex assembly; C:cytoplasm; F:beta-tubulin binding; F:chaperone binding; P:post-chaperonin tubulin folding pathway; C:nucleolus; C:microtubule		
Acrdi.XM_015895988.1/1-1206 | Monca.augustus.g51355.t1/1-1392	0.167208	0.172057	PR domain zinc finger 14-like	2	F:GO:0003676; F:GO:0046872	F:nucleic acid binding; F:metal ion binding		
Acrdi.XM_015920331.1/1-1905 | Monca.adi2mcaRNA31587_R0/1-1917	0.146018	0.17205	O-linked-mannose beta-1,2-N-acetylglucosaminyltransferase 1	2	F:GO:0008375; P:GO:0006486	F:acetylglucosaminyltransferase activity; P:protein glycosylation		
Acrdi.XM_015907052.1/1-657 | Monca.augustus.g43299.t1/1-657	0.166099	0.171944	GTP-binding Di-Ras2	8	F:GO:0005525; F:GO:0000166; P:GO:0008150; P:GO:0007264;	F:GTP binding; F:nucleotide binding; P:biological_process; P:small GTPase mediated signal transduction; P:signal transduction; C:membrane; C:intracellular; C:plasma membrane		
Acrdi.XM_015893532.1/1-1086 | Monca.augustus.g24909.t1/1-942	0.189303	0.171911	heparan sulfate glucosamine 3-O-sulfotransferase 3B1-like	1	F:GO:0008146	F:sulfotransferase activity		
Acrdi.XM_015915745.1/1-1419 | Monca.augustus.g54907.t1/1-2508	0.143168	0.171888	sortilin isoform X2	6	C:GO:0016020; C:GO:0016021; C:GO:0005794; C:GO:0005576;	C:membrane; C:integral component of membrane; C:Golgi apparatus; C:extracellular region; P:transport; F:carboxylic ester hydrolase activity		
Acrdi.XM_015904474.1/1-1170 | Monca.augustus.g63036.t1/1-1164	0.198913	0.171879	dopamine receptor 2-like	3	F:GO:0001588; P:GO:0001963; F:GO:0035240	F:dopamine neurotransmitter receptor activity, coupled via Gs; P:synaptic transmission, dopaminergic; F:dopamine binding		
Acrdi.XM_015894630.1/1-690 | Monca.adi2mcaRNA40938_R4/1-684	0.0956064	0.17185	Retrovirus-related Pol poly from transposon					
Acrdi.XM_015914166.1/1-3405 | Monca.augustus.g24989.t1/1-3438	0.234986	0.171836	Ras guanine nucleotide exchange factor Y	6	P:GO:0007264; F:GO:0005085; C:GO:0016020; C:GO:0016021;	P:small GTPase mediated signal transduction; F:guanyl-nucleotide exchange factor activity; C:membrane; C:integral component of membrane; P:positive regulation of GTPase activity; C:intracellular		
Acrdi.XM_015897503.1/1-1914 | Monca.augustus.g18032.t1/1-2040	0.17102	0.171784	PREDICTED: uncharacterized protein LOC107332752	1	P:GO:0007165	P:signal transduction		
Acrdi.XM_015921491.1/1-780 | Monca.adi2mcaRNA32848_R0/1-684	0.174866	0.171765	Coiled-coil domain-containing 33					
Acrdi.XM_015907869.1/1-1560 | Monca.augustus.g47389.t1/1-1482	0.208685	0.171759	tRNA:m(4)X modification enzyme TRM13 homolog	4	P:GO:0008033; F:GO:0008175; F:GO:0008168; P:GO:0030488	P:tRNA processing; F:tRNA methyltransferase activity; F:methyltransferase activity; P:tRNA methylation		
Acrdi.XM_015900943.1/1-501 | Monca.augustus.g27910.t1/1-501	0.141988	0.171743	M-phase phospho 6	2	C:GO:0000176; P:GO:0000460	C:nuclear exosome (RNase complex); P:maturation of 5.8S rRNA		
Acrdi.XM_015920641.1/1-1506 | Monca.augustus.g36311.t1/1-2613	0.215558	0.171714	Cyclin-dependent kinase-like 5					
Acrdi.XM_015912161.1/1-675 | Monca.augustus.g55535.t1/1-2682	0.158002	0.171626	collagen alpha-1(XII) chain-like					
Acrdi.XM_015903744.1/1-2703 | Monca.augustus.g48643.t1/1-2724	0.184253	0.171585	ankyrin repeat	3	F:GO:0032440; F:GO:0016491; P:GO:0055114	F:2-alkenal reductase [NAD(P)] activity; F:oxidoreductase activity; P:oxidation-reduction process		
Acrdi.XM_015925340.1/1-1032 | Monca.augustus.g6772.t1/1-1023	0.157382	0.171556	G- coupled receptor 161-like	4	C:GO:0005887; F:GO:0004930; P:GO:0007186; P:GO:0007166	C:integral component of plasma membrane; F:G-protein coupled receptor activity; P:G-protein coupled receptor signaling pathway; P:cell surface receptor signaling pathway		
Acrdi.XM_015903596.1/1-2436 | Monca.augustus.g53512.t1/1-2385	0.203033	0.171488	PREDICTED: uncharacterized protein LOC107338363 isoform X1					
Acrdi.XM_015902508.1/1-405 | Monca.augustus.g41959.t1/1-1071	0.181124	0.171482	PREDICTED: uncharacterized protein LOC107337372 isoform X2					
Acrdi.XM_015913499.1/1-2169 | Monca.augustus.g60194.t1/1-2277	0.174775	0.17148	Zinc phosphodiesterase ELAC 2					
Acrdi.XM_015905758.1/1-501 | Monca.augustus.g9356.t1/1-537	0.21731	0.171476	PREDICTED: uncharacterized protein C5orf49 homolog isoform X1					
Acrdi.XM_015914547.1/1-1200 | Monca.augustus.g29128.t1/1-1218	0.244497	0.171455	PREDICTED: uncharacterized protein LOC107348504 isoform X2					
Acrdi.XM_015908814.1/1-909 | Monca.augustus.g4768.t1/1-915	0.134106	0.171444	Homeobox ARX	5	F:GO:0003677; C:GO:0005634; P:GO:0007275; P:GO:0006355;	F:DNA binding; C:nucleus; P:multicellular organism development; P:regulation of transcription, DNA-templated; F:sequence-specific DNA binding		
Acrdi.XM_015906216.1/1-345 | Monca.augustus.g21699.t1/1-348	0.17862	0.171384	PREDICTED: uncharacterized protein LOC107340825					
Acrdi.XM_015910816.1/1-1119 | Monca.augustus.g70014.t1/1-1515	0.17227	0.171342	monocarboxylate transporter 10-like	4	P:GO:0006811; P:GO:0055085; C:GO:0016020; F:GO:0005215	P:ion transport; P:transmembrane transport; C:membrane; F:transporter activity		
Acrdi.XM_015908989.1/1-864 | Monca.augustus.g59503.t1/1-3339	0.18784	0.17134	PREDICTED: uncharacterized protein LOC107343422					
Acrdi.XM_015895904.1/1-627 | Monca.augustus.g30157.t1/1-1422	0.186274	0.171326	indian hedgehog	5	P:GO:0016539; P:GO:0007275; P:GO:0006508; P:GO:0007267;	P:intein-mediated protein splicing; P:multicellular organism development; P:proteolysis; P:cell-cell signaling; F:peptidase activity		
Acrdi.XM_015897972.1/1-2190 | Monca.augustus.g28133.t1/1-2499	0.169442	0.171264	adhesive plaque matrix 2-like	1	F:GO:0005509	F:calcium ion binding		
Acrdi.XM_015895089.1/1-336 | Monca.augustus.g4728.t1/1-597	0.240918	0.171243	TIGR00730 family Rossman fold	3	F:GO:0016799; P:GO:0009691; C:GO:0005829	F:hydrolase activity, hydrolyzing N-glycosyl compounds; P:cytokinin biosynthetic process; C:cytosol		
Acrdi.XM_015917692.1/1-681 | Monca.adi2mcaRNA28667_R0/1-702	0.0882638	0.171215	glycine receptor subunit alpha-4	5	P:GO:1902476; C:GO:0016021; F:GO:0005230; F:GO:0005254;	P:chloride transmembrane transport; C:integral component of membrane; F:extracellular ligand-gated ion channel activity; F:chloride channel activity; C:plasma membrane		
Acrdi.XM_015909422.1/1-2055 | Monca.adi2mcaRNA19518_R1/1-2025	0.229544	0.171213	small conductance calcium-activated potassium channel 3 isoform X1	3	F:GO:0022836; C:GO:0016020; F:GO:0005267	F:gated channel activity; C:membrane; F:potassium channel activity		
Acrdi.XM_015898009.1/1-1437 | Monca.augustus.g39412.t1/1-1635	0.259401	0.171155	regulator of nonsense transcripts 3B	1	F:GO:0000166	F:nucleotide binding		
Acrdi.XM_015900648.1/1-852 | Monca.augustus.g28806.t1/1-897	0.151839	0.171142	PREDICTED: uncharacterized protein LOC107335619					
Acrdi.XM_015900173.1/1-1164 | Monca.augustus.g59982.t1/1-1152	0.327979	0.17114	Mediator of RNA polymerase II transcription subunit 26	3	F:GO:0003677; C:GO:0005634; P:GO:0006351	F:DNA binding; C:nucleus; P:transcription, DNA-templated		
Acrdi.XM_015911082.1/1-1623 | Monca.augustus.g55145.t1/1-1542	0.127522	0.171134	WD40 repeat-containing SMU1	1	C:GO:0044424	C:intracellular part		
Acrdi.XM_015924907.1/1-594 | Monca.augustus.g35003.t1/1-3810	0.239119	0.171119	PREDICTED: uncharacterized protein LOC107358288	3	F:GO:0005509; C:GO:0016020; C:GO:0016021	F:calcium ion binding; C:membrane; C:integral component of membrane		
Acrdi.XM_015924626.1/1-2376 | Monca.augustus.g22979.t1/1-2604	0.212946	0.17109	pyruvate dehydrogenase phosphatase regulatory mitochondrial-like	1	F:GO:0003824	F:catalytic activity		
Acrdi.XM_015922456.1/1-1266 | Monca.augustus.g40384.t1/1-1287	0.111935	0.171082	coiled-coil domain-containing 181 isoform X1					
Acrdi.XM_015903993.1/1-996 | Monca.augustus.g27785.t1/1-3450	0.173195	0.171049	attractin 1	4	P:GO:0007186; C:GO:0016020; C:GO:0016021; F:GO:0030246	P:G-protein coupled receptor signaling pathway; C:membrane; C:integral component of membrane; F:carbohydrate binding		
Acrdi.XM_015897737.1/1-474 | Monca.augustus.g50740.t1/1-852	0.216963	0.170982	ubiquinone biosynthesis COQ4 mitochondrial	2	P:GO:0006744; C:GO:0005743	P:ubiquinone biosynthetic process; C:mitochondrial inner membrane		
Acrdi.XM_015903078.1/1-1104 | Monca.augustus.g45229.t1/1-1119	0.166337	0.170956	dopamine receptor 2-like	3	F:GO:0004930; P:GO:0007154; P:GO:0044700	F:G-protein coupled receptor activity; P:cell communication; P:single organism signaling		
Acrdi.XM_015918259.1/1-1677 | Monca.augustus.g48582.t1/1-1689	0.221996	0.17092	PREDICTED: uncharacterized protein LOC107351956	2	C:GO:0016020; C:GO:0016021	C:membrane; C:integral component of membrane		
Acrdi.XM_015917812.1/1-1563 | Monca.augustus.g62347.t1/1-1566	0.15686	0.170912	Lysosome membrane 2	4	C:GO:0016020; C:GO:0016021; F:GO:0004872; C:GO:0005764	C:membrane; C:integral component of membrane; F:receptor activity; C:lysosome		
Acrdi.XM_015909451.1/1-2160 | Monca.adi2mcaRNA19549_R9/1-1488	0.403658	0.170897	PREDICTED: uncharacterized protein LOC107343863					
Acrdi.XM_015905107.1/1-612 | Monca.augustus.g31958.t1/1-585	0.229576	0.170896	Cancer-related nucleoside-triphosphatase	2	F:GO:0097159; F:GO:1901363	F:organic cyclic compound binding; F:heterocyclic compound binding		
Acrdi.XM_015901677.1/1-2544 | Monca.augustus.g54279.t1/1-5526	0.193868	0.170895	YLP motif-containing 1					
Acrdi.XM_015909457.1/1-1395 | Monca.adi2mcaRNA2034_R0/1-1809	0.218045	0.170851	ralBP1-associated Eps domain-containing 1 isoform X1					
Acrdi.XM_015893878.1/1-411 | Monca.augustus.g6176.t1/1-567	0.192172	0.17085	PREDICTED: uncharacterized protein LOC107334914	3	P:GO:0000077; C:GO:0005634; P:GO:0071479	P:DNA damage checkpoint; C:nucleus; P:cellular response to ionizing radiation		
Acrdi.XM_015912143.1/1-486 | Monca.augustus.g67775.t1/1-510	0.235749	0.170794	probable peptide chain release factor C12orf65 mitochondrial	7	F:GO:0016150; P:GO:0072344; F:GO:0043022; P:GO:0070126;	F:translation release factor activity, codon nonspecific; P:rescue of stalled ribosome; F:ribosome binding; P:mitochondrial translational termination; F:aminoacyl-tRNA hydrol EC:3.1.1.29; EC:3.1.1.1		Aminoacyl-tRNA hydrolase; Carboxylesterase

[illegible]

Acrdi.XM_015918353.1/1-633 | Monca.augustus.g41647.t1/1-4947	0.152218	0.165594	spindle assembly checkpoint component MAD1-like			
Acrdi.XM_015918430.1/1-2463 | Monca.augustus.g66936.t1/1-3177	0.195333	0.165587	Stonin-2	6	C:GO:0098793; P:GO:0036465; C:GO:0005829; C:GO:0030131;	C:presynapse; P:synaptic vesicle recycling; C:cytosol; C:clathrin adaptor complex; P:intracellular protein tr [illegible]
Acrdi.XM_015892451.1/1-1731 | Monca.augustus.g69046.t1/1-4383	0.214476	0.165584	Fanconi anemia group A	7	C:GO:0005634; P:GO:0008584; C:GO:0043240; P:GO:0007140;	C:nucleus; P:male gonad development; C:Fanconi anaemia nuclear complex; P:male meiosis; P:female g [illegible]
Acrdi.XM_015900651.1/1-681 | Monca.augustus.g28782.t1/1-669	0.16643	0.165548	fukutin-related -like	2	C:GO:0016020; C:GO:0016021	C:membrane; C:integral component of membrane
Acrdi.XM_015896256.1/1-4461 | Monca.augustus.g33728.t1/1-4440	0.218161	0.165534	hornerin-like isoform X1	2	C:GO:0042151; F:GO:0030246	C:nematocyst; F:carbohydrate binding
Acrdi.XM_015921243.1/1-699 | Monca.augustus.g67784.t1/1-714	0.170469	0.165523	TPA: opsin	5	F:GO:0004930; F:GO:0009881; P:GO:0007165; P:GO:0009583;	F:G-protein coupled receptor activity; F:photoreceptor activity; P:signal transduction; P:detection of light [illegible]
Acrdi.XM_015903715.1/1-696 | Monca.augustus.g31173.t1/1-1590	0.184846	0.16552	Prolyl 4-hydroxylase subunit alpha-1	1	F:GO:0016491	F:oxidoreductase activity
Acrdi.XM_015906720.1/1-6669 | Monca.adi2mcaRNA16536_R0/1-5430	0.166441	0.165496	Polycystic kidney disease and receptor for egg jelly-related	6	P:GO:0050982; F:GO:0005509; F:GO:0005262; C:GO:0016020;	P:detection of mechanical stimulus; F:calcium ion binding; F:calcium channel activity; C:membrane; C:in [illegible]
Acrdi.XM_015907739.1/1-1152 | Monca.augustus.g62583.t1/1-1152	0.207131	0.165473	SH3 and PX domain-containing 2B			
Acrdi.XM_015912847.1/1-837 | Monca.augustus.g19112.t1/1-1017	0.158823	0.165465	epoxide hydrolase 4			
Acrdi.XM_015924210.1/1-831 | Monca.augustus.g18219.t1/1-1125	0.143293	0.165458	carboxypeptidase B	2	C:GO:0005615; F:GO:0004181	C:extracellular space; F:metallocarboxypeptidase activity
Acrdi.XM_015896288.1/1-3417 | Monca.adi2mcaRNA5133_R0/1-3366	0.232105	0.165434	skeletal organic matrix 5	10	P:GO:0008063; F:GO:0004888; P:GO:0007352; C:GO:0016020;	P:Toll signaling pathway; F:transmembrane signaling receptor activity; P:zygotic specification of dorsal/v [illegible]
Acrdi.XM_015908103.1/1-396 | Monca.augustus.g33238.t1/1-828	0.161114	0.165423	E3 ubiquitin- ligase HECTD1	3	F:GO:0008061; P:GO:0006030; C:GO:0005576	F:chitin binding; P:chitin metabolic process; C:extracellular region
Acrdi.XM_015896404.1/1-960 | Monca.augustus.g54919.t1/1-933	0.164264	0.165405	methylglutaconyl- mitochondrial	2	F:GO:0003824; P:GO:0008152	F:catalytic activity; P:metabolic process
Acrdi.XM_015895698.1/1-1131 | Monca.augustus.g2246.t1/1-1197	0.143296	0.165371	mitogen-activated kinase	1	F:GO:0016301	F:kinase activity
Acrdi.XM_015924397.1/1-1476 | Monca.augustus.g39978.t1/1-1455	0.187989	0.165351	coagulation factor V-like	3	F:GO:0070492; C:GO:0016020; C:GO:0016021	F:oligosaccharide binding; C:membrane; C:integral component of membrane
Acrdi.XM_015909201.1/1-720 | Monca.augustus.g44604.t1/1-948	0.14931	0.165351	peroxisome biogenesis factor 10-like	6	C:GO:0005778; P:GO:0016558; F:GO:0008270; C:GO:0005779;	C:peroxisomal membrane; P:protein import into peroxisome matrix; F:zinc ion binding; C:integral compo [illegible]
Acrdi.XM_015905355.1/1-765 | Monca.augustus.g12753.t1/1-834	0.234647	0.165348	ankyrin repeat domain-containing 49-like			
Acrdi.XM_015924065.1/1-420 | Monca.augustus.g58429.t1/1-1092	0.156544	0.165333	Transmembrane 116	4	F:GO:0004930; P:GO:0007186; C:GO:0016020; C:GO:0016021	F:G-protein coupled receptor activity; P:G-protein coupled receptor signaling pathway; C:membrane; C:in [illegible]
Acrdi.XM_015914696.1/1-2322 | Monca.augustus.g53742.t1/1-2574	0.181602	0.165305	IQ and ubiquitin-like domain-containing isoform X2	4	C:GO:0031514; P:GO:0060271; C:GO:0001669; P:GO:0007224	C:motile cilium; P:cilium assembly; C:acrosomal vesicle; P:smoothened signaling pathway
Acrdi.XM_015907058.1/1-360 | Monca.augustus.g43297.t1/1-372	0.255847	0.165304	oxidoreductase-like domain-containing 1			
Acrdi.XM_015901082.1/1-723 | Monca.augustus.g57646.t1/1-744	0.142246	0.165296	14-3-3 theta	16	F:GO:0019904; F:GO:0008022; P:GO:0007264; C:GO:0043234;	F:protein domain specific binding; F:protein C-terminus binding; P:small GTPase mediated signal transdu [illegible]
Acrdi.XM_015916317.1/1-1362 | Monca.augustus.g64077.t1/1-1521	0.161318	0.165274	nuclear-interacting partner of ALK-like	2	C:GO:0005634; F:GO:0008270	C:nucleus; F:zinc ion binding
Acrdi.XM_015919325.1/1-780 | Monca.augustus.g10289.t1/1-804	0.174288	0.165272	FIT family	4	C:GO:0005789; P:GO:0065007; C:GO:0016021; P:GO:0006996	C:endoplasmic reticulum membrane; P:biological regulation; C:integral component of membrane; P:orga [illegible]
Acrdi.XM_015902615.1/1-1077 | Monca.augustus.g22741.t1/1-1314	0.0882016	0.165254	IQ domain-containing D isoform X2			
Acrdi.XM_015906147.1/1-1452 | Monca.augustus.g29204.t1/1-1452	0.147356	0.16523	ectonucleotide pyrophosphatase phosphodiesterase family member 5-	7	P:GO:0030194; F:GO:0003824; P:GO:0008152; C:GO:0016020;	P:positive regulation of blood coagulation; F:catalytic activity; P:metabolic process; C:membrane; C:integ [illegible]
Acrdi.XM_015892312.1/1-2514 | Monca.adi2mcaRNA38239_R0/1-2577	0.105368	0.165221	cytochrome P450 3A24-like	2	F:GO:0016491; F:GO:0046872	F:oxidoreductase activity; F:metal ion binding
Acrdi.XM_015910044.1/1-5880 | Monca.augustus.g36886.t1/1-6660	0.227914	0.165217	Thyroid receptor-interacting 11			
Acrdi.XM_015896227.1/1-1071 | Monca.augustus.g14712.t1/1-1122	0.247566	0.165212	ribonuclease P subunit p40 isoform X1	6	C:GO:0005634; F:GO:0004526; P:GO:0008033; P:GO:0001682;	C:nucleus; F:ribonuclease P activity; P:tRNA processing; P:tRNA 5'-leader removal; C:nucleolar ribonuclea [illegible]
Acrdi.XM_015902493.1/1-666 | Monca.augustus.g41966.t1/1-675	0.164018	0.165177	Homeobox Nkx-	4	P:GO:0003309; P:GO:0045165; P:GO:0021522; P:GO:0003310	P:type B pancreatic cell differentiation; P:cell fate commitment; P:spinal cord motor neuron differentiati [illegible]
Acrdi.XM_015917602.1/1-1239 | Monca.augustus.g9240.t1/1-951	0.133287	0.165154	TRAF3-interacting 1	4	C:GO:0005813; P:GO:0042073; C:GO:0005929; C:GO:0030992	C:centrosome; P:intraciliary transport; C:cilium; C:intraciliary transport particle B
Acrdi.XM_015912200.1/1-1074 | Monca.adi2mcaRNA22520_R0/1-1044	0.357247	0.165144	PREDICTED: uncharacterized protein LOC107346420	1	C:GO:0016020	C:membrane
Acrdi.XM_015905154.1/1-1377 | Monca.augustus.g16165.t1/1-1959	0.239497	0.165136	arginine-glutamic acid dipeptide repeats -like			
Acrdi.XM_015900411.1/1-1242 | Monca.augustus.g42581.t1/1-1230	0.140313	0.165116	nucleoredoxin-like			
Acrdi.XM_015911513.1/1-2445 | Monca.augustus.g45428.t1/1-3165	0.140386	0.165081	latent-transforming growth factor beta-binding 4	1	F:GO:0005509	F:calcium ion binding
Acrdi.XM_015915670.1/1-5421 | Monca.adi2mcaRNA26373_R0/1-4038	0.127419	0.16507	EGF-like repeat and discoidin I-like domain-containing 3	3	P:GO:0006836; F:GO:0005328; C:GO:0016021	P:neurotransmitter transport; F:neurotransmitter:sodium symporter activity; C:integral component of me [illegible]
Acrdi.XM_015911855.1/1-1497 | Monca.augustus.g43165.t1/1-1734	0.197093	0.165063	Motile sperm domain-containing 2	2	C:GO:0016020; C:GO:0016021	C:membrane; C:integral component of membrane
Acrdi.XM_015896750.1/1-2172 | Monca.augustus.g42415.t1/1-2061	0.197319	0.165059	tRNA wybutosine-synthesizing 4	2	F:GO:0008168; P:GO:0032259	F:methyltransferase activity; P:methylation
Acrdi.XM_015923336.1/1-678 | Monca.augustus.g39154.t1/1-648	0.327258	0.164999	Transmembrane 70- mitochondrial	1	C:GO:0016020	C:membrane
Acrdi.XM_015916146.1/1-3552 | Monca.adi2mcaRNA26938_R0/1-2370	0.184687	0.164998	SH3 domain-containing RING finger 3	2	C:GO:0044464; F:GO:0005488	C:cell part; F:binding
Acrdi.XM_015908456.1/1-657 | Monca.augustus.g33929.t1/1-654	0.149927	0.164958	DNA-directed RNA polymerase III subunit RPC7-like isoform X1	4	F:GO:0001056; C:GO:0000790; P:GO:0006384; C:GO:0005666	F:RNA polymerase III activity; C:nuclear chromatin; P:transcription initiation from RNA polymerase III pro [illegible]
Acrdi.XM_015917231.1/1-1008 | Monca.augustus.g55543.t1/1-1401	0.183411	0.164951	L-cysteine desulfhydrase-like	1	F:GO:0003824	F:catalytic activity
Acrdi.XM_015918788.1/1-1152 | Monca.adi2mcaRNA29866_R0/1-1140	0.141787	0.164941	glycerol kinase 5	6	F:GO:0000166; P:GO:0006071; C:GO:0005737; P:GO:0016310;	F:nucleotide binding; P:glycerol metabolic process; C:cytoplasm; P:phosphorylation; F:phosphotransfera [illegible]
Acrdi.XM_015919121.1/1-744 | Monca.augustus.g35184.t1/1-987	0.18384	0.164905	FAM50 homolog	1	C:GO:0005634	C:nucleus
Acrdi.XM_015925179.1/1-321 | Monca.augustus.g35960.t1/1-648	0.151093	0.164898	Tumor D54			
Acrdi.XM_015916031.1/1-807 | Monca.augustus.g70003.t1/1-819	0.115218	0.164881	folate receptor alpha-like			
Acrdi.XM_015902121.1/1-1104 | Monca.augustus.g64287.t1/1-1068	0.174225	0.164877	adenosine receptor A1-like	2	F:GO:0004930; P:GO:0007165	F:G-protein coupled receptor activity; P:signal transduction
Acrdi.XM_015904851.1/1-1896 | Monca.augustus.g59050.t1/1-1905	0.150521	0.164866	N-acetyl-beta-glucosaminyl-glyco 4-beta-N-acetylgalactosaminyltrans	3	C:GO:0032580; F:GO:0008376; F:GO:0016740	C:Golgi cisterna membrane; F:acetylgalactosaminyltransferase activity; F:transferase activity
Acrdi.XM_015907356.1/1-1356 | Monca.augustus.g63468.t1/1-1347	0.177742	0.164845	suppressor of cytokine signaling 5	12	P:GO:0045629; P:GO:0045627; C:GO:0005737; P:GO:0007173;	P:negative regulation of T-helper 2 cell differentiation; P:positive regulation of T-helper 1 cell differentia [illegible]
Acrdi.XM_015902130.1/1-1035 | Monca.augustus.g46396.t1/1-1032	0.153205	0.164704	melanocyte-stimulating hormone receptor-like	7	F:GO:0004930; F:GO:0004977; P:GO:0007186; P:GO:0007165;	F:G-protein coupled receptor activity; F:melanocortin receptor activity; P:G-protein coupled receptor sign [illegible]
Acrdi.XM_015919185.1/1-2208 | Monca.augustus.g54965.t1/1-2220	0.152594	0.1647	endothelin-converting enzyme 2-like isoform X3	2	P:GO:0006508; F:GO:0004222	P:proteolysis; F:metalloendopeptidase activity
Acrdi.XM_015913818.1/1-4134 | Monca.augustus.g47500.t1/1-5157	0.205477	0.164682	Tectonin beta-propeller repeat-containing 2			
Acrdi.XM_015897944.1/1-996 | Monca.augustus.g23833.t1/1-1089	0.184111	0.164672	E3 ubiquitin- ligase RNF168	3	F:GO:0046872; P:GO:0033522; C:GO:0044424	F:metal ion binding; P:histone H2A ubiquitination; C:intracellular part
Acrdi.XM_015924337.1/1-3606 | Monca.augustus.g32418.t1/1-6021	0.178422	0.164634	E3 ubiquitin- ligase UBR3	2	F:GO:0008270; F:GO:0016874	F:zinc ion binding; F:ligase activity
Acrdi.XM_015892063.1/1-1362 | Monca.adi2mcaRNA37938_R1/1-1437	0.18814	0.164633	molybdate-anion transporter-like isoform X2	1	C:GO:0016020	C:membrane
Acrdi.XM_015896249.1/1-552 | Monca.augustus.g33746.t1/1-549	0.208875	0.16463	probable RNA-binding 18	4	F:GO:0000166; F:GO:0003676; P:GO:0002230; P:GO:0098792	F:nucleotide binding; F:nucleic acid binding; P:positive regulation of defense response to virus by host; P: [illegible]
Acrdi.XM_015895405.1/1-2499 | Monca.adi2mcaRNA4286_R0/1-3264	0.182406	0.164626	A-kinase anchor 9			
Acrdi.XM_015898456.1/1-5754 | Monca.augustus.g39447.t1/1-7413	0.203946	0.164625	Cilia- and flagella-associated 44			
Acrdi.XM_015921655.1/1-996 | Monca.adi2mcaRNA33021_R0/1-990	0.183869	0.164617	NDRG3-like isoform X2	2	C:GO:0005737; P:GO:0007165	C:cytoplasm; P:signal transduction
Acrdi.XM_015909748.1/1-2091 | Monca.augustus.g41738.t1/1-3111	0.141969	0.164601	GON-4			
Acrdi.XM_015903171.1/1-3876 | Monca.augustus.g52465.t1/1-3927	0.219803	0.1646	GTPase-activating and VPS9 domain-containing 1-like	4	P:GO:0051223; F:GO:0005085; C:GO:0005829; F:GO:0032794	P:regulation of protein transport; F:guanyl-nucleotide exchange factor activity; C:cytosol; F:GTPase activa [illegible]
Acrdi.XM_015892360.1/1-1107 | Monca.augustus.g3564.t1/1-1110	0.16364	0.16459	high choriolytic enzyme 1-like	3	F:GO:0008270; P:GO:0006508; F:GO:0004222	F:zinc ion binding; P:proteolysis; F:metalloendopeptidase activity
Acrdi.XM_015919798.1/1-408 | Monca.augustus.g40326.t1/1-408	0.145793	0.164581	Mediator of RNA polymerase II transcription subunit 28	2	C:GO:0005634; C:GO:0044446	C:nucleus; C:intracellular organelle part
Acrdi.XM_015899627.1/1-1218 | Monca.augustus.g33795.t1/1-1314	0.121951	0.16453	FAM172A isoform X7	1	C:GO:0005783	C:endoplasmic reticulum
Acrdi.XM_015900013.1/1-1200 | Monca.augustus.g51834.t1/1-1194	0.114169	0.16451	carbohydrate sulfotransferase 3-like	1	F:GO:0016740	F:transferase activity
Acrdi.XM_015914791.1/1-1722 | Monca.adi2mcaRNA25385_R0/1-1671	0.166904	0.164474	Potassium voltage-gated channel subfamily H member 6	5	C:GO:0005887; F:GO:0005249; P:GO:0006811; P:GO:0042391;	C:integral component of plasma membrane; F:voltage-gated potassium channel activity; P:ion transport; [illegible]
Acrdi.XM_015896393.1/1-807 | Monca.augustus.g48034.t1/1-1041	0.150475	0.164466	matrix metallo ase-19-like	2	F:GO:0046872; F:GO:0008237	F:metal ion binding; F:metallopeptidase activity
Acrdi.XM_015900539.1/1-1059 | Monca.augustus.g37042.t1/1-987	0.128571	0.164411	phosphatidate mitochondrial	3	F:GO:0016779; P:GO:0046474; C:GO:0005743	F:nucleotidyltransferase activity; P:glycerophospholipid biosynthetic process; C:mitochondrial inner mem [illegible]
Acrdi.XM_015920241.1/1-1536 | Monca.augustus.g54341.t1/1-1533	0.196751	0.164392	Telomeric repeat-binding factor 2	4	F:GO:0003677; P:GO:0031627; F:GO:0003691; C:GO:0000783	F:DNA binding; P:telomeric loop formation; F:double-stranded telomeric DNA binding; C:nuclear telomer [illegible]
Acrdi.XM_015901294.1/1-1014 | Monca.augustus.g61811.t1/1-1023	0.158631	0.164388	trace amine-associated receptor 9-like	3	F:GO:0004930; P:GO:0007154; P:GO:0044700	F:G-protein coupled receptor activity; P:cell communication; P:single organism signaling
Acrdi.XM_015897770.1/1-495 | Monca.augustus.g46351.t1/1-1140	0.167696	0.164382	BRO1 domain-containing BROX			
Acrdi.XM_015909047.1/1-1353 | Monca.adi2mcaRNA19056_R0/1-1374	0.245823	0.164353	amino acid transporter ANTL1-like isoform X2	4	C:GO:0016020; C:GO:0016021; F:GO:0015171; P:GO:0003333	C:membrane; C:integral component of membrane; F:amino acid transmembrane transporter activity; P:a [illegible]
Acrdi.XM_015903207.1/1-1719 | Monca.augustus.g51160.t1/1-2739	0.176775	0.164266	serine arginine repetitive matrix 1-like	1	F:GO:0005509	F:calcium ion binding
Acrdi.XM_015912693.1/1-2319 | Monca.augustus.g43020.t1/1-3561	0.189717	0.16425	echinoderm microtubule-associated -like 2			
Acrdi.XM_015912352.1/1-3792 | Monca.adi2mcaRNA22656_R0/1-3249	0.658036	0.164234	NLRC3-like			
Acrdi.XM_015924701.1/1-1404 | Monca.augustus.g58164.t1/1-1659	0.157088	0.164197	uncharacterized sodium-dependent transporter HI_0736-like	3	C:GO:0016020; F:GO:0015293; P:GO:0044765	C:membrane; F:symporter activity; P:single-organism transport
Acrdi.XM_015915067.1/1-996 | Monca.adi2mcaRNA2552_R4/1-1038	0.143659	0.164188	receptor-type tyrosine- phosphatase delta-like	2	P:GO:0006470; F:GO:0016791	P:protein dephosphorylation; F:phosphatase activity
Acrdi.XM_015904216.1/1-2442 | Monca.augustus.g11836.t1/1-2475	0.203148	0.16416	potassium voltage-gated channel Shaw-like	5	P:GO:0071805; C:GO:0008076; F:GO:0005251; P:GO:0034765;	P:potassium ion transmembrane transport; C:voltage-gated potassium channel complex; F:delayed recti [illegible]
Acrdi.XM_015902645.1/1-2157 | Monca.augustus.g22755.t1/1-5487	0.182695	0.164105	histone-lysine N-methyltransferase SETDB1-like	6	C:GO:0005634; F:GO:0003677; F:GO:0008270; P:GO:0034968;	C:nucleus; F:DNA binding; F:zinc ion binding; P:histone lysine methylation; F:histone-lysine N-methyltran [illegible]
Acrdi.XM_015905625.1/1-2373 | Monca.augustus.g24053.t1/1-2376	0.195056	0.164051	leucine rich repeat	5	F:GO:0005525; P:GO:0007264; C:GO:0016020; C:GO:0016021;	F:GTP binding; P:small GTPase mediated signal transduction; C:membrane; C:integral component of me [illegible]
Acrdi.XM_015895893.1/1-2853 | Monca.adi2mcaRNA4740_R0/1-2589	0.235947	0.164031	nucleolar 6	5	C:GO:0032040; P:GO:0006409; C:GO:0034456; P:GO:0006364;	C:small-subunit processome; P:tRNA export from nucleus; C:UTP-C complex; P:rRNA processing; C:CURI [illegible]
Acrdi.XM_015904095.1/1-1296 | Monca.augustus.g44550.t1/1-1308	0.213788	0.164026	F-box LRR-repeat 20-like isoform X2			
Acrdi.XM_015901201.1/1-1665 | Monca.augustus.g58574.t1/1-1662	0.148203	0.163983	dnaJ homolog subfamily C member 21-like	3	F:GO:0003676; F:GO:0046872; C:GO:0005829	F:nucleic acid binding; F:metal ion binding; C:cytosol
Acrdi.XM_015908358.1/1-3984 | Monca.augustus.g9815.t1/1-6693	0.198684	0.163953	WD repeat-containing 81			
Acrdi.XM_015895330.1/1-1794 | Monca.augustus.g25969.t1/1-7686	0.142888	0.16393	receptor-type tyrosine- phosphatase	2	P:GO:0006470; F:GO:0016791	P:protein dephosphorylation; F:phosphatase activity
Acrdi.XM_015915251.1/1-753 | Monca.augustus.g46184.t1/1-777	0.165509	0.163922	PREDICTED: uncharacterized protein LOC107349150			
Acrdi.XM_015900856.1/1-414 | Monca.augustus.g42352.t1/1-417	0.162307	0.163896	probable 28S ribosomal mitochondrial	3	F:GO:0003735; P:GO:0032543; C:GO:0005763	F:structural constituent of ribosome; P:mitochondrial translation; C:mitochondrial small ribosomal subu [illegible]
Acrdi.XM_015899871.1/1-2433 | Monca.augustus.g48623.t1/1-2439	0.162877	0.163888	kyphoscoliosis peptidase-like	2	C:GO:0016020; C:GO:0016021	C:membrane; C:integral component of membrane
Acrdi.XM_015897658.1/1-3405 | Monca.augustus.g50751.t1/1-7710	0.18851	0.16386	#NAME?	3	F:GO:0071949; F:GO:0016491; P:GO:0055114	F:FAD binding; F:oxidoreductase activity; P:oxidation-reduction process
Acrdi.XM_015917118.1/1-924 | Monca.adi2mcaRNA28020_R0/1-1770	0.5049	0.163855	PREDICTED: uncharacterized protein LOC107350877			
Acrdi.XM_015911249.1/1-1179 | Monca.adi2mcaRNA2105_R0/1-1227	0.264838	0.163844	tRNA (guanine-N(7)-)-methyltransferase non-catalytic subunit WDR4	1	P:GO:0036265	P:RNA (guanine-N7)-methylation
Acrdi.XM_015918304.1/1-456 | Monca.augustus.g20419.t1/1-1260	0.958753	0.163835	PREDICTED: uncharacterized protein LOC107352002			
Acrdi.XM_015901926.1/1-1215 | Monca.augustus.g7651.t1/1-1281	0.204998	0.163828	zinc finger C2HC domain-containing 1A			
Acrdi.XM_015920878.1/1-1134 | Monca.augustus.g42018.t1/1-1233	0.16524	0.163822	CUB domain-containing -like isoform X5	2	C:GO:0016020; C:GO:0016021	C:membrane; C:integral component of membrane
Acrdi.XM_015919500.1/1-414 | Monca.augustus.g16849.t1/1-891	0.183622	0.163818	nodal modulator 1-like	3	C:GO:0005789; C:GO:0016021; F:GO:0030246	C:endoplasmic reticulum membrane; C:integral component of membrane; F:carbohydrate binding
Acrdi.XM_015914905.1/1-1842 | Monca.augustus.g62444.t1/1-1782	0.204029	0.163797	Potassium voltage-gated channel subfamily H member 6	5	P:GO:0006813; C:GO:0005887; F:GO:0005249; P:GO:0042391;	P:potassium ion transport; C:integral component of plasma membrane; F:voltage-gated potassium chan [illegible]
Acrdi.XM_015924637.1/1-4095 | Monca.augustus.g22975.t1/1-4422	0.197869	0.163781	ATP-dependent DNA helicase Q4	11	F:GO:0003676; C:GO:0005634; P:GO:0006270; P:GO:0006281;	F:nucleic acid binding; C:nucleus; P:DNA replication initiation; P:DNA repair; F:DNA helicase activity; P:po [illegible]
Acrdi.XM_015899194.1/1-1440 | Monca.adi2mcaRNA8259_R0/1-1068	0.103446	0.163777	FAD-linked oxidase	5	F:GO:0016614; F:GO:0050660; F:GO:0003824; F:GO:0016491;	F:oxidoreductase activity, acting on CH-OH group of donors; F:flavin adenine dinucleotide binding; F:cata [illegible]
Acrdi.XM_015901825.1/1-711 | Monca.augustus.g52525.t1/1-711	0.173064	0.163727	thiopurine S-methyltransferase	1	F:GO:0008168	F:methyltransferase activity
Acrdi.XM_015915679.1/1-2583 | Monca.augustus.g37388.t1/1-2673	0.224136	0.163719	ankyrin repeat and BTB POZ domain-containing 2	4	F:GO:0003677; C:GO:0005634; F:GO:0046982; C:GO:0000786	F:DNA binding; C:nucleus; F:protein heterodimerization activity; C:nucleosome
Acrdi.XM_015923450.1/1-3960 | Monca.augustus.g31127.t1/1-3963	0.130194	0.163716	pleckstrin homology domain-containing family G member 5-like	4	P:GO:0050794; C:GO:0044464; P:GO:0044699; P:GO:0009987	P:regulation of cellular process; C:cell part; P:single-organism process; P:cellular process
Acrdi.XM_015904734.1/1-939 | Monca.augustus.g31924.t1/1-1074	0.171111	0.163705	Death-associated kinase	3	F:GO:0005524; F:GO:0004672; P:GO:0006468	F:ATP binding; F:protein kinase activity; P:protein phosphorylation
Acrdi.XM_015896926.1/1-2457 | Monca.augustus.g28880.t1/1-3180	0.197551	0.16369	epithelial cell-transforming sequence 2 oncogene-like isoform X2	3	P:GO:0035023; F:GO:0005089; P:GO:0043547	P:regulation of Rho protein signal transduction; F:Rho guanyl-nucleotide exchange factor activity; P:posit [illegible]
Acrdi.XM_015910857.1/1-3045 | Monca.augustus.g24266.t1/1-3087	0.189528	0.163646	DNA-binding SMUBP-2	13	P:GO:0050905; F:GO:0043167; P:GO:0021522; F:GO:0008026;	P:neuromuscular process; F:ion binding; P:spinal cord motor neuron differentiation; F:ATP-dependent hel [illegible]
Acrdi.XM_015915854.1/1-2370 | Monca.adi2mcaRNA26592_R0/1-2082	0.206845	0.16364	kazrin isoform X1	7	C:GO:0005634; C:GO:0001533; C:GO:0005856; C:GO:0005737;	C:nucleus; C:cornified envelope; C:cytoskeleton; C:cytoplasm; C:cell junction; C:desmosome; P:keratinizati [illegible]
Acrdi.XM_015925247.1/1-1854 | Monca.adi2mcaRNA37176_R1/1-1890	0.15695	0.163623	Prostaglandin F synthase	2	F:GO:0016491; P:GO:0055114	F:oxidoreductase activity; P:oxidation-reduction process
Acrdi.XM_015905091.1/1-453 | Monca.augustus.g29788.t1/1-807	0.745213	0.163589	cnidCAP isoform			
Acrdi.XM_015909394.1/1-582 | Monca.augustus.g32239.t1/1-597	0.102892	0.163588	PRELI domain-containing mitochondrial	2	C:GO:0005758; F:GO:1990050	C:mitochondrial intermembrane space; F:phosphatidic acid transporter activity
Acrdi.XM_015905736.1/1-420 | Monca.augustus.g9357.t1/1-318	0.164162	0.163563	60S ribosomal L44	3	F:GO:0003735; C:GO:0022625; P:GO:0006412	F:structural constituent of ribosome; C:cytosolic large ribosomal subunit; P:translation
Acrdi.XM_015909830.1/1-1419 | Monca.augustus.g40678.t1/1-1506	0.127709	0.163525	serine threonine- kinase Nek6-like	5	F:GO:0000166; F:GO:0005524; F:GO:0004672; F:GO:0004674;	F:nucleotide binding; F:ATP binding; F:protein kinase activity; F:protein serine/threonine kinase activity; [illegible]
Acrdi.XM_015902318.1/1-669 | Monca.adi2mcaRNA11658_R0/1-390	0.131226	0.163434	PREDICTED: uncharacterized protein LOC107337201			
Acrdi.XM_015915777.1/1-471 | Monca.augustus.g61013.t1/1-459	0.215861	0.163419	bifunctional -like			
Acrdi.XM_015919308.1/1-2763 | Monca.augustus.g60677.t1/1-2562	0.191713	0.163419	tetratricopeptide repeat 27			
Acrdi.XM_015904472.1/1-1515 | Monca.augustus.g16843.t1/1-1122	0.166595	0.163402	HIV Tat-specific factor 1	5	F:GO:0000166; F:GO:0003723; C:GO:0005684; C:GO:0005686;	F:nucleotide binding; F:RNA binding; C:U2-type spliceosomal complex; C:U2 snRNP; P:mRNA splicing, via [illegible]
Acrdi.XM_015908160.1/1-666 | Monca.augustus.g6695.t1/1-654	0.135995	0.163336	calcium-dependent kinase-like	1	F:GO:0005488	F:binding
Acrdi.XM_015921123.1/1-831 | Monca.augustus.g29197.t1/1-996	0.123817	0.163311	cyclin-Y 1	2	F:GO:0019901; P:GO:0000079	F:protein kinase binding; P:regulation of cyclin-dependent protein serine/threonine kinase activity
Acrdi.XM_015915049.1/1-561 | Monca.augustus.g49272.t1/1-492	0.188214	0.163289	glutamate receptor kainate 2-like	14	P:GO:0034220; C:GO:0045202; F:GO:0004872; F:GO:0005234;	P:ion transmembrane transport; C:synapse; F:receptor activity; F:extracellular-glutamate-gated ion chan [illegible]
Acrdi.XM_015913768.1/1-1185 | Monca.augustus.g45552.t1/1-1881	0.0988708	0.163278	thrombospondin-1-like isoform X1			
Acrdi.XM_015906953.1/1-2085 | Monca.adi2mcaRNA16775_R0/1-2049	0.253474	0.163265	rasGAP-activating 1			
Acrdi.XM_015903350.1/1-1953 | Monca.augustus.g32350.t1/1-3294	0.19661	0.163254	A disintegrin and metallo ase with thrombospondin motifs 6	6	F:GO:0008270; P:GO:0006508; C:GO:0031012; F:GO:0004222;	F:zinc ion binding; P:proteolysis; C:extracellular matrix; F:metalloendopeptidase activity; C:proteinaceous [illegible]
Acrdi.XM_015894656.1/1-2352 | Monca.augustus.g48617.t1/1-3015	0.136694	0.163234	Ubiquitin carboxyl-terminal hydrolase 36	3	P:GO:0016579; F:GO:0036459; P:GO:0006511	P:protein deubiquitination; F:thiol-dependent ubiquitinyl hydrolase activity; P:ubiquitin-dependent protei [illegible]
Acrdi.XM_015912156.1/1-1170 | Monca.augustus.g57800.t1/1-1284	0.17358	0.163232	lysine-specific demethylase JMJD5 isoform X2	1	F:GO:0005509	F:calcium ion binding
Acrdi.XM_015893834.1/1-474 | Monca.augustus.g537.t1/1-624	0.151115	0.163224	zinc finger HIT domain-containing 1			
Acrdi.XM_015892879.1/1-594 | Monca.adi2mcaRNA38900_R0/1-630	0.169971	0.163189	UPF0573 C2orf70 homolog A-like			
Acrdi.XM_015918343.1/1-1035 | Monca.augustus.g41657.t1/1-1023	0.170545	0.163165	PREDICTED: uncharacterized protein LOC107352040			
Acrdi.XM_015891761.1/1-1746 | Monca.augustus.g43722.t1/1-2304	0.156547	0.163165	sodium-dependent neutral amino acid transporter B(0)AT3 isoform X2	3	C:GO:0016020; F:GO:0015293; P:GO:0006810	C:membrane; F:symporter activity; P:transport
Acrdi.XM_015917408.1/1-1278 | Monca.augustus.g66919.t1/1-1350	0.135717	0.163154	Casein kinase I isoform	8	P:GO:0008360; C:GO:0005634; F:GO:0005524; C:GO:0005737;	P:regulation of cell shape; C:nucleus; F:ATP binding; C:cytoplasm; P:Wnt signaling pathway; F:protein ser [illegible]
Acrdi.XM_015909609.1/1-882 | Monca.augustus.g61634.t1/1-1092	0.19657	0.163151	PREDICTED: uncharacterized protein LOC107344008			
Acrdi.XM_015898584.1/1-4869 | Monca.adi2mcaRNA7669_R0/1-4887	0.192606	0.163148	leucine-rich repeat-containing G- coupled receptor 6-like	9	F:GO:0005524; P:GO:0018108; F:GO:0004713; C:GO:0016020;	F:ATP binding; P:peptidyl-tyrosine phosphorylation; F:protein tyrosine kinase activity; C:membrane; C:inte [illegible]
Acrdi.XM_015902954.1/1-543 | Monca.augustus.g68837.t1/1-702	0.2611	0.163138	KTI12 homolog	4	F:GO:0000166; P:GO:0008150; F:GO:0005524; C:GO:0005575	F:nucleotide binding; P:biological_process; F:ATP binding; C:cellular_component
Acrdi.XM_015916151.1/1-4218 | Monca.augustus.g25392.t1/1-4197	0.175827	0.163123	circadian locomoter output cycles kaput	8	F:GO:0003677; C:GO:0005667; C:GO:0005634; F:GO:0003700;	F:DNA binding; C:transcription factor complex; C:nucleus; F:transcription factor activity, sequence-specifi [illegible]
Acrdi.XM_015909711.1/1-3897 | Monca.augustus.g41730.t1/1-3915	0.198981	0.163115	regulation of nuclear pre-mRNA domain-containing 2 isoform X1			
Acrdi.XM_015900518.1/1-1212 | Monca.augustus.g1376.t1/1-1209	0.177159	0.163107	tiggy-winkle hedgehog -like	7	P:GO:0030900; P:GO:0042063; P:GO:0048663; P:GO:0048702;	P:forebrain development; P:gliogenesis; P:neuron fate commitment; P:embryonic neurocranium morpho [illegible]
Acrdi.XM_015899086.1/1-1680 | Monca.augustus.g43392.t1/1-1770	0.187999	0.163106	degenerin deg-1-like	1	P:GO:0006811	P:ion transport
Acrdi.XM_015899342.1/1-1017 | Monca.augustus.g40289.t1/1-1317	0.135307	0.163052	PREDICTED: uncharacterized protein LOC107334410			
Acrdi.XM_015915978.1/1-2526 | Monca.augustus.g51185.t1/1-4161	0.258971	0.162985	lymphoid-restricted membrane -like			
Acrdi.XM_015920044.1/1-1713 | Monca.adi2mcaRNA31309_R0/1-1713	0.160672	0.162951	alpha collagen			
Acrdi.XM_015913372.1/1-3087 | Monca.augustus.g32121.t1/1-2106	0.159224	0.162925	histone-lysine N-methyltransferase SETD2-like	8	F:GO:0003723; P:GO:0034968; F:GO:0018024; P:GO:0006355;	F:RNA binding; P:histone lysine methylation; F:histone-lysine N-methyltransferase activity; P:regulation [illegible]
Acrdi.XM_015899613.1/1-1668 | Monca.augustus.g4244.t1/1-2184	0.187849	0.162913	Bromodomain-containing 7			
Acrdi.XM_015925324.1/1-1725 | Monca.augustus.g63031.t1/1-3723	0.236822	0.162911	Epithelial-stromal interaction 1			
Acrdi.XM_015907734.1/1-7509 | Monca.augustus.g62582.t1/1-11004	0.188446	0.162863	plectin-like isoform X1			
Acrdi.XM_015892216.1/1-537 | Monca.augustus.g7545.t1/1-963	0.268581	0.162849	delta(3,5)-Delta(2,4)-dienoyl- mitochondrial	6	P:GO:0009062; C:GO:0070062; F:GO:0003824; C:GO:0005739;	P:fatty acid catabolic process; C:extracellular exosome; F:catalytic activity; C:mitochondrion; C:membrane [illegible]
Acrdi.XM_015924353.1/1-1371 | Monca.augustus.g39141.t1/1-1371	0.175527	0.162847	naked cuticle homolog 2 isoform X2	2	P:GO:0050794; P:GO:0044699	P:regulation of cellular process; P:single-organism process
Acrdi.XM_015904798.1/1-723 | Monca.augustus.g15367.t1/1-948	0.112647	0.162818	melatonin receptor type 1A-like	2	P:GO:0007165; C:GO:0016021	P:signal transduction; C:integral component of membrane
Acrdi.XM_015913761.1/1-1161 | Monca.augustus.g12530.t1/1-1155	0.220588	0.162779	NIF3 1	1	C:GO:0005739	C:mitochondrion
Acrdi.XM_015900333.1/1-1035 | Monca.augustus.g49259.t1/1-1107	0.201557	0.162636	tyrosinase tyr-3	7	F:GO:0008270; F:GO:0046872; P:GO:0006508; F:GO:0008233;	F:zinc ion binding; F:metal ion binding; P:proteolysis; F:peptidase activity; F:metalloendopeptidase activi [illegible]
Acrdi.XM_015918294.1/1-1704 | Monca.adi2mcaRNA29329_R0/1-1698	0.188039	0.162618	kinetochore-associated 1	1	C:GO:0044424	C:intracellular part
Acrdi.XM_015898548.1/1-1452 | Monca.augustus.g33984.t1/1-1452	0.209672	0.162583	histone-lysine N-methyltransferase PRDM6	2	F:GO:0003676; F:GO:0046872	F:nucleic acid binding; F:metal ion binding
Acrdi.XM_015913506.1/1-5070 | Monca.augustus.g16109.t1/1-5094	0.20027	0.162522	ATP-binding cassette sub-family A member 5-like isoform X2	1	P:GO:0006810	P:transport
Acrdi.XM_015923275.1/1-1146 | Monca.augustus.g55714.t1/1-687	0.226232	0.162502	lysosomal acid lipase cholesteryl ester hydrolase	4	C:GO:0070062; P:GO:0044707; P:GO:0006629; F:GO:0004771	C:extracellular exosome; P:single-multicellular organism process; P:lipid metabolic process; F:sterol este [illegible]
Acrdi.XM_015924930.1/1-1020 | Monca.augustus.g5012.t1/1-1023	0.166979	0.162498	type-1 angiotensin II receptor B-like	1	P:GO:0007165	P:signal transduction
Acrdi.XM_015907136.1/1-822 | Monca.augustus.g36957.t1/1-771	0.137397	0.162476	Brain-specific angiogenesis inhibitor 3	2	C:GO:0016020; C:GO:0016021	C:membrane; C:integral component of membrane
Acrdi.XM_015906170.1/1-2085 | Monca.adi2mcaRNA15941_R0/1-1905	0.161615	0.162469	two pore calcium channel 1 isoform X4	7	P:GO:0065007; F:GO:0005216; P:GO:0006811; P:GO:0055085;	P:biological regulation; F:ion channel activity; P:ion transport; P:transmembrane transport; F:gated chan [illegible]
Acrdi.XM_015923564.1/1-543 | Monca.augustus.g58093.t1/1-966	0.148051	0.162466	aquaporin-4 isoform X3	5	F:GO:0015267; F:GO:0022891; C:GO:0044425; P:GO:0006810;	F:channel activity; F:substrate-specific transmembrane transporter activity; C:membrane part; P:transpo [illegible]
Acrdi.XM_015915892.1/1-708 | Monca.augustus.g39028.t1/1-2955	0.124714	0.162405	SEC23-interacting	1	F:GO:0046872	F:metal ion binding
Acrdi.XM_015911770.1/1-2514 | Monca.adi2mcaRNA22058_R1/1-2394	0.0591601	0.162374	52 kDa repressor of the inhibitor of the kinase			
Acrdi.XM_015902856.1/1-1425 | Monca.adi2mcaRNA12268_R0/1-1338	0.170177	0.162351	RNA-directed DNA polymerase from mobile element jockey	1	P:GO:0006810	P:transport
Acrdi.XM_015892949.1/1-570 | Monca.augustus.g24463.t1/1-1416	0.167033	0.162323	Neutrophil cytosol factor 2			
Acrdi.XM_015914817.1/1-2400 | Monca.adi2mcaRNA25445_R0/1-2931	0.178315	0.162319	and pleckstrin domain-containing 2 isoform X1	7	P:GO:0035023; C:GO:0005856; C:GO:0005737; F:GO:0005089;	P:regulation of Rho protein signal transduction; C:cytoskeleton; C:cytoplasm; F:Rho guanyl-nucleotide ex [illegible]
Acrdi.XM_015900364.1/1-1200 | Monca.augustus.g11748.t1/1-1230	0.184804	0.162289	PREDICTED: uncharacterized protein LOC107335347			
Acrdi.XM_015907875.1/1-13368 | Monca.adi2mcaRNA17804_R1/1-1239	0.207322	0.162275	G- coupled receptor 98	7	F:GO:0004930; F:GO:0004888; P:GO:0007186; P:GO:0007154;	F:G-protein coupled receptor activity; F:transmembrane signaling receptor activity; P:G-protein coupled [illegible]
Acrdi.XM_015922606.1/1-639 | Monca.augustus.g32436.t1/1-1023	0.173315	0.162255	transmembrane 198-like	1	C:GO:0016020	C:membrane
Acrdi.XM_015899519.1/1-762 | Monca.augustus.g20822.t1/1-546	0.529652	0.16225	KRAB-A domain-containing 2-like	2	F:GO:0003676; P:GO:0015074	F:nucleic acid binding; P:DNA integration
Acrdi.XM_015922260.1/1-1506 | Monca.augustus.g4391.t1/1-1467	0.233379	0.162239	PREDICTED: uncharacterized protein LOC107355682			
Acrdi.XM_015921372.1/1-729 | Monca.augustus.g4645.t1/1-1578	0.140885	0.162211	PREDICTED: uncharacterized protein LOC107354880	2	C:GO:0016020; C:GO:0016021	C:membrane; C:integral component of membrane
Acrdi.XM_015911036.1/1-2763 | Monca.augustus.g17756.t1/1-2787	0.199203	0.162186	MAM and LDL-receptor class A domain-containing 1-like	2	C:GO:0016020; C:GO:0016021	C:membrane; C:integral component of membrane
Acrdi.XM_015903337.1/1-1131 | Monca.augustus.g29885.t1/1-2232	0.133993	0.162156	receptor-type tyrosine- phosphatase N2-like	2	P:GO:0006470; F:GO:0016791	P:protein dephosphorylation; F:phosphatase activity
Acrdi.XM_015904205.1/1-471 | Monca.augustus.g12945.t1/1-453	0.226102	0.16212	PREDICTED: uncharacterized protein C19orf60 homolog			
Acrdi.XM_015901282.1/1-735 | Monca.augustus.g61796.t1/1-987	0.126244	0.162119	adrenocorticotropic hormone receptor-like	3	F:GO:0004930; P:GO:0007165; C:GO:0016020	F:G-protein coupled receptor activity; P:signal transduction; C:membrane
Acrdi.XM_015902858.1/1-6450 | Monca.adi2mcaRNA12271_R0/1-5787	0.149169	0.162111	Adenylate cyclase type 10	4	F:GO:0016849; C:GO:0005622; P:GO:0009190; P:GO:0035556	F:phosphorus-oxygen lyase activity; C:intracellular; P:cyclic nucleotide biosynthetic process; P:intracellula [illegible]
Acrdi.XM_015915028.1/1-3843 | Monca.augustus.g39610.t1/1-3915	0.196845	0.162096	E3 ubiquitin- ligase	5	C:GO:0005634; F:GO:0004842; C:GO:0005737; F:GO:0016874;	C:nucleus; F:ubiquitin-protein transferase activity; C:cytoplasm; F:ligase activity; P:protein ubiquitination [illegible]
Acrdi.XM_015913177.1/1-312 | Monca.augustus.g39266.t1/1-312	0.280611	0.162088	PREDICTED: uncharacterized protein LOC107347277			
Acrdi.XM_015913370.1/1-2013 | Monca.adi2mcaRNA23838_R0/1-1668	0.218939	0.16206	tensin-3-			
Acrdi.XM_015909405.1/1-1536 | Monca.augustus.g26353.t1/1-1881	0.186732	0.161933	Neuronal acetylcholine receptor subunit alpha-7	9	P:GO:0007271; F:GO:0015464; C:GO:0045211; P:GO:0007165;	P:synaptic transmission, cholinergic; F:acetylcholine receptor activity; C:postsynaptic membrane; P:signal [illegible]
Acrdi.XM_015893005.1/1-2073 | Monca.adi2mcaRNA39053_R0/1-1182	0.202431	0.161932	transmembrane and TPR repeat-containing CG4050	2	C:GO:0016020; C:GO:0016021	C:membrane; C:integral component of membrane
Acrdi.XM_015899994.1/1-1533 | Monca.augustus.g31999.t1/1-4785	0.173813	0.161929	XK-related 4-like	2	P:GO:0070782; C:GO:0005886	P:phosphatidylserine exposure on apoptotic cell surface; C:plasma membrane
Acrdi.XM_015921490.1/1-4701 | Monca.augustus.g58041.t1/1-5832	0.130975	0.161904	myosin heavy skeletal muscle-like isoform X2			
Acrdi.XM_015899224.1/1-1920 | Monca.adi2mcaRNA8363_R0/1-2055	0.18131	0.161883	transient receptor potential cation channel subfamily V member 5-like	1	P:GO:0006810	P:transport
Acrdi.XM_015919975.1/1-981 | Monca.augustus.g60883.t1/1-984	0.096781	0.161851	PREDICTED: uncharacterized protein LOC107353638	1	F:GO:0046872	F:metal ion binding
Acrdi.XM_015909513.1/1-795 | Monca.augustus.g1391.t1/1-1770	0.216235	0.161837	tectonic-1-like isoform X5			
Acrdi.XM_015918660.1/1-3831 | Monca.augustus.g64238.t1/1-5109	0.167393	0.161778	SH3 and multiple ankyrin repeat domains 3-like			
Acrdi.XM_015894401.1/1-1263 | Monca.adi2mcaRNA40671_R1/1-1455	0.120493	0.161772	PREDICTED: uncharacterized protein LOC107329734			
Acrdi.XM_015903539.1/1-864 | Monca.augustus.g44100.t1/1-843	0.117824	0.161709	Fork head domain-containing FD3	4	C:GO:0005634; F:GO:0043565; F:GO:0000981; P:GO:0006357	C:nucleus; F:sequence-specific DNA binding; F:RNA polymerase II transcription factor activity, sequence- [illegible]
Acrdi.XM_015898313.1/1-702 | Monca.augustus.g37934.t1/1-723	0.209495	0.16168	AP-4 complex accessory subunit tepsin-like			
Acrdi.XM_015912003.1/1-915 | Monca.augustus.g35304.t1/1-918	0.137004	0.161655	melanocyte-stimulating hormone receptor-like	7	F:GO:0004930; P:GO:0007186; P:GO:0007165; C:GO:0016020;	F:G-protein coupled receptor activity; P:G-protein coupled receptor signaling pathway; P:signal transduct [illegible]
Acrdi.XM_015916389.1/1-372 | Monca.augustus.g52607.t1/1-369	0.171815	0.16162	ATPase inhibitor mai- mitochondrial-like	8	P:GO:0032780; F:GO:0042030; P:GO:0009396; C:GO:0005739;	P:negative regulation of ATPase activity; F:ATPase inhibitor activity; P:folic acid-containing compound bi [illegible]
Acrdi.XM_015925339.1/1-1644 | Monca.augustus.g6777.t1/1-2133	0.135417	0.16162	peroxidase	2	F:GO:0016491; P:GO:0050896	F:oxidoreductase activity; P:response to stimulus
Acrdi.XM_015921314.1/1-819 | Monca.augustus.g16011.t1/1-870	0.12194	0.161556	zinc finger Ran-binding domain-containing 2 isoform X1			
Acrdi.XM_015901283.1/1-1128 | Monca.augustus.g61800.t1/1-1140	0.226676	0.161555	Transcription factor SOX-18	48	P:GO:2000043; P:GO:0061031; P:GO:0061010; P:GO:0007493;	P:regulation of cardiac cell fate specification; P:endodermal digestive tract morphogenesis; P:gall bladde [illegible]
Acrdi.XM_015900763.1/1-2661 | Monca.augustus.g53995.t1/1-3597	0.197935	0.161548	cartilage oligomeric matrix -like	3	F:GO:0005509; P:GO:0007155; C:GO:0005576	F:calcium ion binding; P:cell adhesion; C:extracellular region
Acrdi.XM_015897463.1/1-1680 | Monca.augustus.g22833.t1/1-1674	0.203672	0.161541	SAAL1	1	C:GO:0005615	C:extracellular space
Acrdi.XM_015908767.1/1-5493 | Monca.augustus.g55156.t1/1-4017	0.192609	0.161492	Sperm-associated antigen 17	2	P:GO:0007165; P:GO:0042981	P:signal transduction; P:regulation of apoptotic process
Acrdi.XM_015920750.1/1-1317 | Monca.augustus.g35924.t1/1-1332	0.121751	0.16148	BTB POZ domain-containing 6-like			
Acrdi.XM_015897648.1/1-3021 | Monca.augustus.g56114.t1/1-3330	0.181173	0.161429	hephaestin isoform X2			
Acrdi.XM_015913613.1/1-2616 | Monca.augustus.g48684.t1/1-4026	0.167026	0.161382	200 kDa antigen			
Acrdi.XM_015898469.1/1-1518 | Monca.augustus.g33974.t1/1-2391	0.239188	0.161371	spermatogenesis-associated 5	1	F:GO:0000166	F:nucleotide binding
Acrdi.XM_015912227.1/1-915 | Monca.augustus.g34502.t1/1-1560	0.132922	0.161333	diacylglycerol O-acyltransferase MT1468	4	F:GO:0047196; P:GO:0019432; F:GO:0004144; C:GO:0005886	F:long-chain-alcohol O-fatty-acyltransferase activity; P:triglyceride biosynthetic process; F:diacylglycerol [illegible]
Acrdi.XM_015895542.1/1-1026 | Monca.augustus.g61603.t1/1-2370	0.178299	0.161316	gamma-aminobutyric acid type B receptor subunit 2			
Acrdi.XM_015898238.1/1-1812 | Monca.augustus.g1157.t1/1-1866	0.183667	0.161248	FAM227B-like isoform X2			
Acrdi.XM_015906908.1/1-933 | Monca.augustus.g8908.t1/1-2346	0.16636	0.161224	bifunctional apoptosis regulator	4	F:GO:0008270; F:GO:0046872; C:GO:0016020; C:GO:0016021	F:zinc ion binding; F:metal ion binding; C:membrane; C:integral component of membrane
Acrdi.XM_015925479.1/1-1437 | Monca.augustus.g34417.t1/1-1458	0.179142	0.161156	pancreatic triacylglycerol lipase-like	3	P:GO:0016042; F:GO:0016298; F:GO:0052689	P:lipid catabolic process; F:lipase activity; F:carboxylic ester hydrolase activity
Acrdi.XM_015924666.1/1-1350 | Monca.augustus.g62624.t1/1-1467	0.182862	0.161154	copine-3	16	F:GO:0048306; F:GO:0004674; C:GO:0005886; F:GO:0005215;	F:calcium-dependent protein binding; F:protein serine/threonine kinase activity; C:plasma membrane; F: [illegible]
Acrdi.XM_015923741.1/1-435 | Monca.augustus.g68718.t1/1-861	0.150828	0.161123	peptidyl-prolyl cis-trans isomerase CWC27 homolog	3	F:GO:0003755; P:GO:0000413; P:GO:0006457	F:peptidyl-prolyl cis-trans isomerase activity; P:protein peptidyl-prolyl isomerization; P:protein folding
Acrdi.XM_015900216.1/1-972 | Monca.augustus.g62863.t1/1-1629	0.12111	0.161105	follicle-stimulating hormone receptor-like	3	F:GO:0004930; P:GO:0007186; C:GO:0016021	F:G-protein coupled receptor activity; P:G-protein coupled receptor signaling pathway; C:integral compo [illegible]
Acrdi.XM_015914265.1/1-3147 | Monca.adi2mcaRNA24815_R0/1-3111	0.221892	0.161088	A disintegrin and metallo ase with thrombospondin motifs 18-like	6	F:GO:0008270; P:GO:0006508; C:GO:0031012; F:GO:0004222;	F:zinc ion binding; P:proteolysis; C:extracellular matrix; F:metalloendopeptidase activity; C:proteinaceous [illegible]
Acrdi.XM_015892445.1/1-660 | Monca.adi2mcaRNA38379_R0/1-555	0.172213	0.161088	FAM210B	1	C:GO:0016020	C:membrane
Acrdi.XM_015907403.1/1-645 | Monca.augustus.g64313.t1/1-615	0.178832	0.161046	homeobox not2-like	3	C:GO:0005634; P:GO:0006355; F:GO:0043565	C:nucleus; P:regulation of transcription, DNA-templated; F:sequence-specific DNA binding
Acrdi.XM_015902254.1/1-1170 | Monca.adi2mcaRNA11642_R0/1-1179	0.174961	0.161033	cysteine-rich secretory LCCL domain-containing 2-like	1	C:GO:0005576	C:extracellular region
Acrdi.XM_015916125.1/1-810 | Monca.augustus.g8609.t1/1-843	0.0973391	0.161017	rolling stone-like	2	C:GO:0016020; C:GO:0016021	C:membrane; C:integral component of membrane
Acrdi.XM_015916717.1/1-855 | Monca.augustus.g37083.t1/1-840	0.235469	0.160987	Proteasome assembly chaperone 1	2	C:GO:0005737; C:GO:0043231	C:cytoplasm; C:intracellular membrane-bounded organelle
Acrdi.XM_015899557.1/1-1617 | Monca.adi2mcaRNA8638_R0/1-1587	0.15468	0.160916	Bifunctional coenzyme A synthase	3	F:GO:0016772; P:GO:0009058; P:GO:0006796	F:transferase activity, transferring phosphorus-containing groups; P:biosynthetic process; P:phosphate-co [illegible]
Acrdi.XM_015908854.1/1-1239 | Monca.adi2mcaRNA18813_R4/1-1350	0.116481	0.160911	piggyBac transposable element-derived 4-like			
Acrdi.XM_015901052.1/1-669 | Monca.augustus.g57613.t1/1-669	0.226999	0.160877	two pore potassium channel sup-9-like	2	C:GO:0016020; P:GO:0006810	C:membrane; P:transport
Acrdi.XM_015911326.1/1-1128 | Monca.augustus.g35295.t1/1-2010	0.183759	0.160773	Pentatricopeptide repeat-containing mitochondrial			
Acrdi.XM_015922792.1/1-3192 | Monca.augustus.g34392.t1/1-1188	0.184814	0.160727	gamma-aminobutyric acid type B receptor subunit 1	8	F:GO:0004930; F:GO:0004965; P:GO:0007186; P:GO:0007165;	F:G-protein coupled receptor activity; F:G-protein coupled GABA receptor activity; P:G-protein coupled re [illegible]
Acrdi.XM_015906284.1/1-1938 | Monca.augustus.g49078.t1/1-1956	0.152546	0.160711	double-stranded RNA-binding Staufen homolog 1 isoform X4			
Acrdi.XM_015924387.1/1-2586 | Monca.augustus.g67820.t1/1-3153	0.213329	0.160658	TOX high mobility group box family member 3	3	P:GO:0007165; C:GO:0016020; C:GO:0016021	P:signal transduction; C:membrane; C:integral component of membrane
Acrdi.XM_015895749.1/1-867 | Monca.augustus.g23211.t1/1-1005	0.189193	0.160647	WD repeat-containing 55-like	4	P:GO:0042273; F:GO:0016905; F:GO:0016740; P:GO:0006468	P:ribosomal large subunit biogenesis; F:myosin heavy chain kinase activity; F:transferase activity; P:prote [illegible]
Acrdi.XM_015917012.1/1-657 | Monca.adi2mcaRNA27902_R0/1-579	0.149689	0.16063	Bifunctional heparan sulfate N-deacetylase N-sulfotransferase 4	4	C:GO:0016020; P:GO:0008152; F:GO:0008146; F:GO:0016787	C:membrane; P:metabolic process; F:sulfotransferase activity; F:hydrolase activity
Acrdi.XM_015903914.1/1-1947 | Monca.augustus.g71428.t1/1-2067	0.665251	0.160587	tyrosinase	4	F:GO:0016491; F:GO:0046872; P:GO:0008152; P:GO:0055114	F:oxidoreductase activity; F:metal ion binding; P:metabolic process; P:oxidation-reduction process
Acrdi.XM_015914129.1/1-1593 | Monca.augustus.g24981.t1/1-2217	0.108736	0.160561	cysteine--tRNA cytoplasmic isoform X5	5	F:GO:0000166; F:GO:0043167; C:GO:0005829; F:GO:0004817;	F:nucleotide binding; F:ion binding; C:cytosol; F:cysteine-tRNA ligase activity; P:cysteinyl-tRNA aminoacy [illegible]
Acrdi.XM_015910949.1/1-1170 | Monca.augustus.g63047.t1/1-1470	0.188622	0.160526	glycogenin-2 isoform X1	1	F:GO:0016757	F:transferase activity, transferring glycosyl groups
Acrdi.XM_015911969.1/1-912 | Monca.augustus.g30065.t1/1-2184	0.164406	0.160502	signal CUB and EGF-like domain-containing			
Acrdi.XM_015917405.1/1-597 | Monca.augustus.g23799.t1/1-600	0.152322	0.160501	homeobox zampogna-like	3	C:GO:0005634; P:GO:0006355; F:GO:0043565	C:nucleus; P:regulation of transcription, DNA-templated; F:sequence-specific DNA binding
Acrdi.XM_015906229.1/1-2811 | Monca.augustus.g21686.t1/1-4191	0.217045	0.16049	moonraker-like isoform X1	1	P:GO:0007099	P:centriole replication
Acrdi.XM_015914442.1/1-927 | Monca.augustus.g56338.t1/1-915	0.167027	0.160461	glyoxalase	2	F:GO:0051213; P:GO:0055114	F:dioxygenase activity; P:oxidation-reduction process
Acrdi.XM_015923863.1/1-1752 | Monca.augustus.g21534.t1/1-1734	0.21254	0.160455	von Willebrand factor type EGF and pentraxin domain-containing 1-like isoform X4			
Acrdi.XM_015901245.1/1-1737 | Monca.adi2mcaRNA10492_R0/1-1635	0.164579	0.160438	tyrosine phosphatase domain-containing 1 isoform X2	3	F:GO:0004721; P:GO:0006470; P:GO:0044699	F:phosphoprotein phosphatase activity; P:protein dephosphorylation; P:single-organism process
Acrdi.XM_015921285.1/1-534 | Monca.augustus.g40355.t1/1-717	0.24566	0.160436	PREDICTED: uncharacterized protein LOC107354792			
Acrdi.XM_015899142.1/1-1272 | Monca.adi2mcaRNA8268_R8/1-1281	0.0863384	0.160427	Forkhead box N4			
Acrdi.XM_015921297.1/1-1596 | Monca.augustus.g40037.t1/1-1620	0.162848	0.160419	periodic tryptophan 1 homolog			
Acrdi.XM_015917871.1/1-414 | Monca.adi2mcaRNA2982_R4/1-357	0.680733	0.160412	PREDICTED: uncharacterized protein LOC107351568			
Acrdi.XM_015910784.1/1-504 | Monca.augustus.g16154.t1/1-486	0.189411	0.160412	probable steroid-binding 3	2	C:GO:0016020; C:GO:0012505	C:membrane; C:endomembrane system
Acrdi.XM_015902527.1/1-8970 | Monca.adi2mcaRNA11890_R0/1-8841	0.1805	0.160409	protocadherin Fat 4	1	P:GO:0048513	P:animal organ development
Acrdi.XM_015894923.1/1-1239 | Monca.augustus.g6768.t1/1-1470	0.134526	0.160395	Potassium voltage-gated channel subfamily G member 1	5	P:GO:0006813; C:GO:0005887; F:GO:0005251; P:GO:0044699;	P:potassium ion transport; C:integral component of plasma membrane; F:delayed rectifier potassium ch [illegible]
Acrdi.XM_015923136.1/1-906 | Monca.augustus.g23387.t1/1-813	0.599116	0.160371	Mediator of RNA polymerase II transcription subunit 34			
Acrdi.XM_015908097.1/1-3162 | Monca.augustus.g33240.t1/1-3804	0.184379	0.16037	EGF-like domain-containing	3	F:GO:0005509; C:GO:0016020; C:GO:0016021	F:calcium ion binding; C:membrane; C:integral component of membrane
Acrdi.XM_015910232.1/1-615 | Monca.augustus.g25479.t1/1-612	0.159891	0.160362	Diencephalon mesencephalon homeobox 1-B	5	F:GO:0003677; C:GO:0005634; P:GO:0007275; P:GO:0006355;	F:DNA binding; C:nucleus; P:multicellular organism development; P:regulation of transcription, DNA-tem [illegible]
Acrdi.XM_015918134.1/1-474 | Monca.adi2mcaRNA29161_R1/1-492	0.513803	0.16033	dipeptidyl-peptidase 5	2	C:GO:0016020; C:GO:0016021	C:membrane; C:integral component of membrane
Acrdi.XM_015920153.1/1-921 | Monca.augustus.g27087.t1/1-906	0.159951	0.160306	zinc finger Gfi-1b	5	F:GO:0003676; F:GO:0046872; P:GO:0030097; P:GO:0006355;	F:nucleic acid binding; F:metal ion binding; P:hemopoiesis; P:regulation of transcription, DNA-templated [illegible]
Acrdi.XM_015914178.1/1-3210 | Monca.augustus.g50937.t1/1-4041	0.206727	0.160271	PREDICTED: uncharacterized protein LOC107348164	2	C:GO:0016020; C:GO:0016021	C:membrane; C:integral component of membrane
Acrdi.XM_015910981.1/1-606 | Monca.augustus.g63056.t1/1-609	0.228966	0.160253	lipoma HMGIC fusion partner-like 1	5	P:GO:0008150; C:GO:0016020; C:GO:0016021; C:GO:0005575;	P:biological_process; C:membrane; C:integral component of membrane; C:cellular_component; F:molecu [illegible]
Acrdi.XM_015898172.1/1-765 | Monca.adi2mcaRNA7157_R0/1-783	0.169082	0.160249	dual adapter for phosphotyrosine and 3-phosphotyrosine and 3-phosphoinositide			
Acrdi.XM_015913332.1/1-1065 | Monca.augustus.g61318.t1/1-969	0.166119	0.160177	NADH-cytochrome b5 reductase-like	1	F:GO:0016491	F:oxidoreductase activity
Acrdi.XM_015903943.1/1-1017 | Monca.augustus.g40632.t1/1-816	0.0416908	0.160164	Transposable element Tcb2 transposase			
Acrdi.XM_015896699.1/1-1044 | Monca.augustus.g21881.t1/1-1038	0.141302	0.160138	FAM124A-like			
Acrdi.XM_015921119.1/1-381 | Monca.augustus.g48115.t1/1-390	0.140451	0.160116	PREDICTED: uncharacterized protein LOC107354638 isoform X1			
Acrdi.XM_015899379.1/1-1113 | Monca.augustus.g44779.t1/1-1089	0.138333	0.160094	Alpha-1A adrenergic receptor	3	F:GO:0004930; P:GO:0007186; C:GO:0016020	F:G-protein coupled receptor activity; P:G-protein coupled receptor signaling pathway; C:membrane
Acrdi.XM_015921373.1/1-3018 | Monca.augustus.g4648.t1/1-4014	0.176099	0.160064	ATP-dependent RNA helicase DHX57	3	F:GO:0000166; F:GO:0043167; F:GO:0004386	F:nucleotide binding; F:ion binding; F:helicase activity
Acrdi.XM_015908294.1/1-783 | Monca.augustus.g31834.t1/1-930	0.229396	0.160056	Mediator of RNA polymerase II transcription subunit 27	1	P:GO:0007275	P:multicellular organism development
Acrdi.XM_015922003.1/1-1407 | Monca.augustus.g23712.t1/1-1479	0.265486	0.160032	Bactericidal permeability-increasing	6	C:GO:0005615; P:GO:0050829; P:GO:0045087; F:GO:0008289;	C:extracellular space; P:defense response to Gram-negative bacterium; P:innate immune response; F:lip [illegible]
Acrdi.XM_015917310.1/1-1593 | Monca.augustus.g23270.t1/1-1590	0.200641	0.159995	dimethylaniline monooxygenase [N-oxide-forming] 5-like	4	C:GO:0043231; C:GO:0016020; P:GO:0008152; F:GO:0004497	C:intracellular membrane-bounded organelle; C:membrane; P:metabolic process; F:monooxygenase acti [illegible]
Acrdi.XM_015912367.1/1-798 | Monca.augustus.g51034.t1/1-1965	0.198045	0.159945	inhibitor of nuclear factor kappa-B kinase subunit alpha-like	5	F:GO:0005524; F:GO:0004672; P:GO:0016310; P:GO:0006468;	F:ATP binding; F:protein kinase activity; P:phosphorylation; P:protein phosphorylation; F:kinase activity
Acrdi.XM_015921305.1/1-1065 | Monca.augustus.g40065.t1/1-1062	0.128949	0.159945	substance-K receptor-like	3	P:GO:0007165; C:GO:0016020; F:GO:0004871	P:signal transduction; C:membrane; F:signal transducer activity
Acrdi.XM_015920648.1/1-1011 | Monca.augustus.g7039.t1/1-1014	0.121304	0.159944	histamine H2 receptor-like	3	P:GO:0007154; P:GO:0044700; F:GO:0004871	P:cell communication; P:single organism signaling; F:signal transducer activity
Acrdi.XM_015903392.1/1-993 | Monca.augustus.g36026.t1/1-1092	0.191567	0.159938	forkhead box G1	7	C:GO:0005634; P:GO:0030182; P:GO:0006355; P:GO:0043584;	C:nucleus; P:neuron differentiation; P:regulation of transcription, DNA-templated; P:nose development; [illegible]
Acrdi.XM_015910985.1/1-1518 | Monca.augustus.g63048.t1/1-1521	0.177732	0.159926	Ectonucleotide pyrophosphatase phosphodiesterase family member 5	1	C:GO:0016020	C:membrane
Acrdi.XM_015912900.1/1-1524 | Monca.adi2mcaRNA23307_R1/1-1698	0.235154	0.159864	Transposon Tf2-6 poly	1	F:GO:0016854	F:racemase and epimerase activity
Acrdi.XM_015893398.1/1-555 | Monca.augustus.g50893.t1/1-1527	0.129986	0.159829	PREDICTED: uncharacterized protein LOC107328667	6	P:GO:0007264; P:GO:0007165; P:GO:0043087; F:GO:0005096;	P:small GTPase mediated signal transduction; P:signal transduction; P:regulation of GTPase activity; F:G [illegible]
Acrdi.XM_015924499.1/1-1224 | Monca.augustus.g28540.t1/1-1392	0.143053	0.159765	Zinc finger ZIC	2	F:GO:0003676; F:GO:0046872	F:nucleic acid binding; F:metal ion binding
Acrdi.XM_015907033.1/1-876 | Monca.augustus.g19212.t1/1-1341	0.149823	0.159745	probable ATP-dependent RNA helicase DDX47 isoform X2	4	F:GO:0000166; P:GO:0010501; P:GO:0006364; F:GO:0004004	F:nucleotide binding; P:RNA secondary structure unwinding; P:rRNA processing; F:ATP-dependent RNA h [illegible]
Acrdi.XM_015898228.1/1-2040 | Monca.augustus.g62896.t1/1-4713	0.17829	0.159714	unconventional myosin-IXb isoform X2	1	F:GO:0005488	F:binding
Acrdi.XM_015916013.1/1-1620 | Monca.augustus.g37742.t1/1-1740	0.151927	0.159703	BUD13 homolog			
Acrdi.XM_015918388.1/1-750 | Monca.augustus.g22421.t1/1-744	0.163877	0.159655	short stature homeobox 2-like	3	C:GO:0005634; P:GO:0006355; F:GO:0043565	C:nucleus; P:regulation of transcription, DNA-templated; F:sequence-specific DNA binding
Acrdi.XM_015921695.1/1-1083 | Monca.augustus.g43111.t1/1-1071	0.133442	0.159633	prolactin-releasing peptide receptor-like	8	F:GO:0004930; C:GO:0005887; P:GO:0007186; P:GO:0007165;	F:G-protein coupled receptor activity; C:integral component of plasma membrane; P:G-protein coupled r [illegible]
Acrdi.XM_015920900.1/1-1194 | Monca.adi2mcaRNA32214_R0/1-1089	0.17283	0.159614	aquaporin-4-like isoform X2	6	C:GO:0005887; F:GO:0015254; P:GO:0009992; P:GO:0034220;	C:integral component of plasma membrane; F:glycerol channel activity; P:cellular water homeostasis; P: [illegible]
Acrdi.XM_015891998.1/1-711 | Monca.augustus.g32101.t1/1-906	0.138771	0.159585	histamine H2 receptor-like	6	F:GO:0004930; P:GO:0007186; P:GO:0007165; C:GO:0016020;	F:G-protein coupled receptor activity; P:G-protein coupled receptor signaling pathway; P:signal transduct [illegible]
Acrdi.XM_015918412.1/1-894 | Monca.adi2mcaRNA29433_R0/1-888	0.123298	0.159577	histamine H2 receptor-like	2	F:GO:0004930; P:GO:0007165	F:G-protein coupled receptor activity; P:signal transduction
Acrdi.XM_015905172.1/1-492 | Monca.augustus.g52830.t1/1-513	0.232064	0.159563	UPF0547 C16orf87 homolog			
Acrdi.XM_015917600.1/1-1386 | Monca.augustus.g10251.t1/1-1458	0.183325	0.159547	FAM188A isoform X1	1	F:GO:0005509	F:calcium ion binding
Acrdi.XM_015924556.1/1-855 | Monca.augustus.g26870.t1/1-1293	0.126597	0.159534	vasodilator-stimulated phospho -like			
Acrdi.XM_015915054.1/1-1392 | Monca.augustus.g33663.t1/1-1575	0.208555	0.159438	RING finger and SPRY domain-containing 1	2	F:GO:0008270; F:GO:0046872	F:zinc ion binding; F:metal ion binding
Acrdi.XM_015916467.1/1-4032 | Monca.adi2mcaRNA27288_R1/1-4104	0.168779	0.159416	nischarin isoform X3	1	F:GO:0035091	F:phosphatidylinositol binding
Acrdi.XM_015908251.1/1-5661 | Monca.adi2mcaRNA18217_R0/1-5640	0.187814	0.159414	histone-lysine N-methyltransferase SETD1B-A-like isoform X1			
Acrdi.XM_015907590.1/1-618 | Monca.augustus.g42237.t1/1-585	0.150862	0.159398	fibroblast growth factor 2	5	F:GO:0008083; P:GO:0008543; C:GO:0005576; F:GO:0005102;	F:growth factor activity; P:fibroblast growth factor receptor signaling pathway; C:extracellular region; F:r [illegible]
Acrdi.XM_015910239.1/1-870 | Monca.adi2mcaRNA2015_R0/1-864	0.196165	0.159376	retinol dehydrogenase 8-like	4	F:GO:0016491; C:GO:0016020; C:GO:0016021; P:GO:0055114	F:oxidoreductase activity; C:membrane; C:integral component of membrane; P:oxidation-reduction proce [illegible]
Acrdi.XM_015914909.1/1-606 | Monca.augustus.g67904.t1/1-453	0.100464	0.159348	cytidine deaminase-like	4	F:GO:0008270; C:GO:0005829; P:GO:0009972; F:GO:0004126	F:zinc ion binding; C:cytosol; P:cytidine deamination; F:cytidine deaminase activity
Acrdi.XM_015906856.1/1-2502 | Monca.augustus.g32943.t1/1-3261	0.220525	0.1593	coiled-coil domain-containing 57-like			
Acrdi.XM_015911702.1/1-2010 | Monca.adi2mcaRNA21980_R0/1-1848	0.150845	0.159294	PREDICTED: uncharacterized protein LOC107345955	2	C:GO:0016020; C:GO:0016021	C:membrane; C:integral component of membrane
Acrdi.XM_015921070.1/1-1425 | Monca.augustus.g34987.t1/1-1422	0.175813	0.159216	multiple inositol polyphosphate phosphatase 1-like	3	F:GO:0016791; P:GO:0016311; F:GO:0003993	F:phosphatase activity; P:dephosphorylation; F:acid phosphatase activity
Acrdi.XM_015915187.1/1-615 | Monca.augustus.g693.t1/1-615	0.155534	0.159206	protease-associated domain-containing 1			
Acrdi.XM_015916656.1/1-1218 | Monca.augustus.g5871.t1/1-1503	0.117661	0.159194	N-acetylglutamate mitochondrial	1	F:GO:0016740	F:transferase activity
Acrdi.XM_015917216.1/1-3132 | Monca.augustus.g55544.t1/1-3441	0.187202	0.159174	transient receptor potential cation channel subfamily A member 1-like	1	P:GO:0050896	P:response to stimulus
Acrdi.XM_015917764.1/1-906 | Monca.augustus.g53138.t1/1-918	0.229259	0.159169	paired box Pax-3 isoform X2			
Acrdi.XM_015924898.1/1-1599 | Monca.augustus.g41431.t1/1-1665	0.195399	0.159166	N-acetylglucosamine-1-phosphodiester alpha-N-acetylglucosaminidase			
Acrdi.XM_015908889.1/1-801 | Monca.augustus.g8651.t1/1-810	0.169462	0.159152	PREDICTED: uncharacterized protein LOC107343327			
Acrdi.XM_015918917.1/1-1863 | Monca.augustus.g48515.t1/1-2373	0.141012	0.159141	serine threonine- kinase	12	C:GO:0005634; F:GO:0005524; C:GO:0015630; P:GO:0030182;	C:nucleus; F:ATP binding; C:microtubule cytoskeleton; P:neuron differentiation; C:synaptic vesicle; P:prote [illegible]
Acrdi.XM_015897319.1/1-852 | Monca.augustus.g54243.t1/1-996	0.198379	0.159109	calcium-binding 39	1	C:GO:0070062	C:extracellular exosome
Acrdi.XM_015913497.1/1-726 | Monca.augustus.g20508.t1/1-726	0.124634	0.1591	tropomyosin	5	C:GO:0044430; C:GO:0099512; C:GO:0015629; C:GO:0043234;	C:cytoskeletal part; C:supramolecular fiber; C:actin cytoskeleton; C:protein complex; C:cytoplasmic part
Acrdi.XM_015905802.1/1-2157 | Monca.augustus.g31769.t1/1-1956	0.190485	0.159095	WD repeat-containing			
Acrdi.XM_015915014.1/1-978 | Monca.augustus.g49279.t1/1-2286	0.191499	0.159088	glutamate receptor NMDA 2A-like			
Acrdi.XM_015923496.1/1-1677 | Monca.augustus.g47451.t1/1-2199	0.166936	0.159007	60 kDa	2	P:GO:0006520; F:GO:0004067	P:cellular amino acid metabolic process; F:asparaginase activity
Acrdi.XM_015910697.1/1-1590 | Monca.augustus.g35874.t1/1-1593	0.165835	0.158961	pollen-specific leucine-rich repeat extensin 2			
Acrdi.XM_015904178.1/1-5175 | Monca.adi2mcaRNA13665_R0/1-5538	0.188617	0.158957	receptor-type tyrosine- phosphatase delta-like isoform X2	2	P:GO:0016311; F:GO:0016787	P:dephosphorylation; F:hydrolase activity
Acrdi.XM_015915052.1/1-888 | Monca.augustus.g64704.t1/1-891	0.250603	0.158943	transmembrane 45A	2	C:GO:0016020; C:GO:0016021	C:membrane; C:integral component of membrane
Acrdi.XM_015898611.1/1-1179 | Monca.augustus.g34557.t1/1-1179	0.148349	0.158902	hepatocyte nuclear factor 6	8	C:GO:0005634; F:GO:0003677; F:GO:0001077; P:GO:0030154;	C:nucleus; F:DNA binding; F:transcriptional activator activity, RNA polymerase II core promoter proximal [illegible]
Acrdi.XM_015910940.1/1-828 | Monca.augustus.g59048.t1/1-957	0.211894	0.158877	etoposide-induced homolog	2	C:GO:0016020; C:GO:0016021	C:membrane; C:integral component of membrane
Acrdi.XM_015896349.1/1-2136 | Monca.augustus.g54412.t1/1-2262	0.197776	0.158873	Heme oxygenase 2	8	P:GO:0006979; P:GO:0055072; F:GO:0005515; F:GO:0004392;	P:response to oxidative stress; P:iron ion homeostasis; F:protein binding; F:heme oxygenase (decyclizing [illegible]
Acrdi.XM_015900444.1/1-591 | Monca.adi2mcaRNA9663_R0/1-771	0.373262	0.15879	ATP-dependent DNA helicase Q1	4	P:GO:0007165; C:GO:0005739; P:GO:0051607; F:GO:0004871	P:signal transduction; C:mitochondrion; P:defense response to virus; F:signal transducer activity
Acrdi.XM_015898644.1/1-936 | Monca.augustus.g7803.t1/1-969	0.247793	0.158784	Fez family zinc finger 2	2	F:GO:0003676; F:GO:0046872	F:nucleic acid binding; F:metal ion binding
Acrdi.XM_015919467.1/1-711 | Monca.augustus.g25230.t1/1-672	0.269166	0.158759	Riboflavin-binding			
Acrdi.XM_015908169.1/1-453 | Monca.augustus.g7871.t1/1-456	0.195901	0.158689	28S ribosomal mitochondrial	1	C:GO:0005739	C:mitochondrion
Acrdi.XM_015898289.1/1-1161 | Monca.augustus.g61834.t1/1-1143	0.155613	0.158687	Forkhead box L1	5	C:GO:0005634; P:GO:0006351; F:GO:0043565; F:GO:0000981;	C:nucleus; P:transcription, DNA-templated; F:sequence-specific DNA binding; F:RNA polymerase II transc [illegible]
Acrdi.XM_015922449.1/1-1281 | Monca.augustus.g40383.t1/1-1977	0.168497	0.158666	chromatin assembly factor 1 subunit B	4	C:GO:0005634; F:GO:0042393; C:GO:0033186; P:GO:0006334	C:nucleus; F:histone binding; C:CAF-1 complex; P:nucleosome assembly
Acrdi.XM_015894833.1/1-300 | Monca.augustus.g57972.t1/1-321	0.100051	0.158654	PREDICTED: uncharacterized protein LOC107330192			
Acrdi.XM_015895991.1/1-630 | Monca.augustus.g51364.t1/1-642	0.266985	0.158643	Segmentation polarity homeobox engrailed	23	P:GO:0035277; P:GO:0035288; P:GO:0035289; P:GO:0007388;	P:spiracle morphogenesis, open tracheal system; P:anterior head segmentation; P:posterior head segme [illegible]
Acrdi.XM_015914326.1/1-351 | Monca.augustus.g9582.t1/1-351	0.149814	0.158642	tctex1 domain-containing 1-B-like			
Acrdi.XM_015922817.1/1-2061 | Monca.augustus.g34396.t1/1-3153	0.222941	0.158625	gamma-aminobutyric acid type B receptor subunit 2	5	F:GO:0004930; F:GO:0004965; P:GO:0007186; C:GO:0016020;	F:G-protein coupled receptor activity; F:G-protein coupled GABA receptor activity; P:G-protein coupled re [illegible]
Acrdi.XM_015895872.1/1-777 | Monca.augustus.g23226.t1/1-777	0.142909	0.158606	post-GPI attachment to s factor 2 isoform X1	2	C:GO:0016020; C:GO:0016021	C:membrane; C:integral component of membrane
Acrdi.XM_015923984.1/1-951 | Monca.augustus.g54029.t1/1-1056	0.134826	0.158597	39S ribosomal mitochondrial	4	F:GO:0003735; C:GO:0005739; C:GO:0005840; P:GO:0006412	F:structural constituent of ribosome; C:mitochondrion; C:ribosome; P:translation
Acrdi.XM_015907689.1/1-561 | Monca.augustus.g29935.t1/1-576	0.132557	0.158583	EF-hand calcium-binding domain-containing 11-like	1	F:GO:0046872	F:metal ion binding

Acrdi.XM_015923152.1/1-1854 | Monca.augustus.g70153.t1/1-2295	0.157518	0.158552	disintegrin and metallo ase domain-containing 10	8	F:GO:0008270; P:GO:0007219; P:GO:0006509; C:GO:0016020;
Acrdi.XM_015922653.1/1-2520 | Monca.adi2mcaRNA34159_R0/1-2325	0.128223	0.15853	Zinc carboxypeptidase A 1	1	F:GO:0004180
Acrdi.XM_015919682.1/1-2826 | Monca.augustus.g67068.t1/1-4725	0.176068	0.158524	hemicentin-1-like isoform X17		
Acrdi.XM_015921856.1/1-2244 | Monca.augustus.g61100.t1/1-2223	0.238348	0.158468	transmembrane channel 7	2	C:GO:0016020; C:GO:0016021
Acrdi.XM_015922075.1/1-1212 | Monca.adi2mcaRNA3367_R8/1-579	0.27244	0.158463	vesicular glutamate transporter 1-like	4	P:GO:0055085; F:GO:0022857; C:GO:0016021; P:GO:0006820
Acrdi.XM_015916698.1/1-2421 | Monca.augustus.g17440.t1/1-2547	0.175711	0.158463	synaptotagmin 5	7	P:GO:0065007; F:GO:0005515; P:GO:0016192; C:GO:0016020;
Acrdi.XM_015923004.1/1-1647 | Monca.augustus.g46083.t1/1-3429	0.186658	0.158454	centrosomal of 131 kDa isoform X1	3	P:GO:0060271; C:GO:0044464; C:GO:0043226
Acrdi.XM_015913381.1/1-1530 | Monca.augustus.g32150.t1/1-1530	0.149324	0.158421	UDP-glucuronosyltransferase 1-2	2	F:GO:0016758; P:GO:0009812
Acrdi.XM_015899737.1/1-759 | Monca.augustus.g66176.t1/1-747	0.192148	0.158399	HEXIM1	7	P:GO:0000122; C:GO:0005634; F:GO:0097322; C:GO:0005737;
Acrdi.XM_015901601.1/1-693 | Monca.augustus.g51829.t1/1-696	0.143127	0.158396	PREDICTED: uncharacterized protein LOC107336528		
Acrdi.XM_015905333.1/1-2220 | Monca.augustus.g32889.t1/1-2922	0.193017	0.158395	E3 ubiquitin- ligase RBBP6 isoform X2		
Acrdi.XM_015915280.1/1-1122 | Monca.augustus.g46189.t1/1-1119	0.165943	0.158389	neuropeptide FF receptor 2-like	2	F:GO:0004930; P:GO:0007165
Acrdi.XM_015925406.1/1-2145 | Monca.augustus.g28105.t1/1-2382	0.119836	0.158342	ADAM 17-like protease	7	F:GO:0008270; C:GO:0016020; P:GO:0006508; C:GO:0016021;
Acrdi.XM_015906776.1/1-3903 | Monca.augustus.g60646.t1/1-3567	0.148875	0.158323	sodium-driven chloride bicarbonate exchanger isoform X3	5	C:GO:0030425; P:GO:0007275; C:GO:0016020; F:GO:0008509;
Acrdi.XM_015924328.1/1-642 | Monca.augustus.g16949.t1/1-480	0.0590385	0.158308	PREDICTED: uncharacterized protein LOC107357693	1	F:GO:0005488
Acrdi.XM_015899944.1/1-426 | Monca.augustus.g32799.t1/1-411	0.172497	0.1583	Zinc finger 593	2	F:GO:0003676; F:GO:0008270
Acrdi.XM_015903679.1/1-4776 | Monca.augustus.g32282.t1/1-4905	0.126825	0.158279	kinesin K39		
Acrdi.XM_015920768.1/1-483 | Monca.augustus.g35938.t1/1-3906	0.218261	0.158252	Tudor domain-containing 7	3	P:GO:0007281; P:GO:0007275; F:GO:0005488
Acrdi.XM_015906509.1/1-3138 | Monca.augustus.g22551.t1/1-3156	0.200183	0.158245	DC-STAMP domain-containing 1	3	F:GO:0008270; C:GO:0016020; C:GO:0016021
Acrdi.XM_015915709.1/1-651 | Monca.augustus.g28530.t1/1-1383	0.133274	0.158224	ELMO domain-containing 3	2	F:GO:0005096; P:GO:0043547
Acrdi.XM_015891845.1/1-978 | Monca.augustus.g67155.t1/1-1008	0.136502	0.158206	Enkurin domain-containing 1		
Acrdi.XM_015918562.1/1-969 | Monca.augustus.g41483.t1/1-1839	0.165719	0.15816	mRNA cap guanine-N7 methyltransferase	5	P:GO:0036265; C:GO:0005845; P:GO:0006370; C:GO:0031981;
Acrdi.XM_015901593.1/1-2937 | Monca.augustus.g70628.t1/1-5706	0.165375	0.158155	kinesin KIF26B		
Acrdi.XM_015909032.1/1-903 | Monca.augustus.g38392.t1/1-894	0.157787	0.158154	apextrin 1	2	C:GO:0016020; C:GO:0016021
Acrdi.XM_015920311.1/1-1899 | Monca.augustus.g24351.t1/1-1890	0.0966399	0.158154	kelch 12	7	F:GO:0004842; P:GO:0008584; C:GO:0005737; P:GO:0007286;
Acrdi.XM_015920058.1/1-915 | Monca.augustus.g24393.t1/1-972	0.174789	0.158152	thioredoxin domain-containing 3 homolog	1	F:GO:0070492
Acrdi.XM_015919450.1/1-6027 | Monca.augustus.g25245.t1/1-6105	0.179904	0.158136	E3 ubiquitin- ligase HECW2 isoform X1	2	F:GO:0016740; C:GO:0044424
Acrdi.XM_015924745.1/1-912 | Monca.augustus.g12806.t1/1-912	0.168787	0.158136	Cytidine and dCMP deaminase domain-containing 1	3	F:GO:0016788; C:GO:0016020; C:GO:0016021
Acrdi.XM_015908248.1/1-540 | Monca.augustus.g40418.t1/1-2712	0.211462	0.158124	T-box transcription factor	20	P:GO:0060044; P:GO:0060980; P:GO:0007267; F:GO:0043565;
Acrdi.XM_015900743.1/1-1866 | Monca.augustus.g34950.t1/1-1860	0.189812	0.158098	cationic amino acid transporter 3	4	C:GO:0016020; C:GO:0016021; F:GO:0015171; P:GO:0003333
Acrdi.XM_015895345.1/1-804 | Monca.augustus.g7656.t1/1-726	0.0358362	0.158098	PREDICTED: uncharacterized protein LOC107330783		
Acrdi.XM_015924903.1/1-1980 | Monca.augustus.g34959.t1/1-1968	0.171647	0.158069	HHIP 2	5	P:GO:0005975; F:GO:0016901; F:GO:0048038; F:GO:0003824;
Acrdi.XM_015902760.1/1-894 | Monca.augustus.g5675.t1/1-897	0.225369	0.15803	probable palmitoyltransferase ZDHHC24	7	F:GO:0016746; F:GO:0019706; F:GO:0008270; F:GO:0046872;
Acrdi.XM_015918543.1/1-336 | Monca.augustus.g15357.t1/1-1194	0.142841	0.158008	myosin heavy clone 203-like		
Acrdi.XM_015898266.1/1-1995 | Monca.augustus.g8198.t1/1-2448	0.14882	0.158006	PREDICTED: uncharacterized protein LOC107333448	2	C:GO:0016020; C:GO:0016021
Acrdi.XM_015898463.1/1-3096 | Monca.adi2mcaRNA7550_R0/1-3069	0.184788	0.158	histone-lysine N-methyltransferase PRDM6	2	F:GO:0003676; F:GO:0046872
Acrdi.XM_015893414.1/1-573 | Monca.augustus.g34509.t1/1-546	0.118677	0.157981	cupin		
Acrdi.XM_015902976.1/1-933 | Monca.augustus.g606.t1/1-807	0.187666	0.15797	lysM and peptidoglycan-binding domain-containing 1		
Acrdi.XM_015911169.1/1-2148 | Monca.augustus.g24035.t1/1-2151	0.197146	0.157959	follistatin-related 5 isoform X2	4	P:GO:0008150; F:GO:0046872; F:GO:0005509; C:GO:0005576
Acrdi.XM_015901846.1/1-741 | Monca.augustus.g48595.t1/1-735	0.130977	0.157955	PREDICTED: uncharacterized protein LOC107336778	7	P:GO:0033619; C:GO:0030660; C:GO:0071556; C:GO:0071458;
Acrdi.XM_015897924.1/1-1032 | Monca.augustus.g23840.t1/1-1008	0.212658	0.157922	angiomotin isoform X2		
Acrdi.XM_015916372.1/1-1827 | Monca.adi2mcaRNA27158_R0/1-1896	0.142545	0.157916	skeletal receptor tyrosine- kinase isoform X4	3	F:GO:0005524; P:GO:0018108; F:GO:0004713
Acrdi.XM_015917896.1/1-540 | Monca.augustus.g39695.t1/1-540	0.171239	0.157904	ankyrin repeat-containing	1	F:GO:0003824
Acrdi.XM_015906587.1/1-9450 | Monca.augustus.g70203.t1/1-8457	0.208325	0.157899	BTB POZ domain-containing 6-like		
Acrdi.XM_015919649.1/1-969 | Monca.augustus.g39905.t1/1-900	0.230986	0.157888	WW domain-binding 4-like	4	F:GO:0003676; C:GO:0005634; F:GO:0008270; F:GO:0046872
Acrdi.XM_015922262.1/1-1416 | Monca.augustus.g12783.t1/1-1413	0.169422	0.157875	Lysine-specific demethylase 8	1	F:GO:0005509
Acrdi.XM_015903824.1/1-675 | Monca.augustus.g32294.t1/1-675	0.285408	0.157873	MIS12 homolog	5	C:GO:0005634; P:GO:0051276; P:GO:0007059; P:GO:0007067;
Acrdi.XM_015903378.1/1-537 | Monca.augustus.g29878.t1/1-531	0.128538	0.157864	LSM12 homolog		
Acrdi.XM_015902131.1/1-1035 | Monca.augustus.g46376.t1/1-1101	0.121577	0.157829	histamine H2 receptor-like	9	F:GO:0004930; F:GO:0001588; P:GO:0001963; P:GO:0007186;
Acrdi.XM_015899779.1/1-819 | Monca.augustus.g43161.t1/1-954	0.168417	0.157803	JNK1 MAPK8-associated membrane	4	P:GO:0008150; C:GO:0016020; C:GO:0016021; F:GO:0003674
Acrdi.XM_015924140.1/1-990 | Monca.augustus.g39218.t1/1-882	0.134667	0.157765	Adipolin		
Acrdi.XM_015921398.1/1-846 | Monca.augustus.g4644.t1/1-840	0.183664	0.157743	PREDICTED: uncharacterized protein LOC107354907		
Acrdi.XM_015903524.1/1-726 | Monca.augustus.g34550.t1/1-648	0.156414	0.157715	tctex1 domain-containing 1-B-like		
Acrdi.XM_015924510.1/1-1230 | Monca.augustus.g31186.t1/1-1326	0.232616	0.157705	Cation efflux family	5	P:GO:0010043; P:GO:0061088; C:GO:0016021; F:GO:0005385;
Acrdi.XM_015923677.1/1-738 | Monca.augustus.g34965.t1/1-1221	0.12682	0.157684	NAD-dependent deacetylase sirtuin-2	4	F:GO:0016811; F:GO:0008270; P:GO:0006476; F:GO:0070403
Acrdi.XM_015920961.1/1-1578 | Monca.augustus.g30434.t1/1-1569	0.203851	0.157678	mitochondrial inner membrane OXA1L-like	1	C:GO:0016020
Acrdi.XM_015916978.1/1-2370 | Monca.augustus.g26565.t1/1-2526	0.140739	0.15764	N,N -diacetylchitobiase-like isoform X2	1	F:GO:0005488
Acrdi.XM_015916665.1/1-1572 | Monca.augustus.g636.t1/1-1647	0.168725	0.157621	alkaline phosphatase	1	F:GO:0003824
Acrdi.XM_015925212.1/1-3366 | Monca.augustus.g53765.t1/1-3396	0.210784	0.157518	dermatan-sulfate epimerase		
Acrdi.XM_015903346.1/1-633 | Monca.augustus.g36029.t1/1-1560	0.23835	0.157513	Gelation factor	2	C:GO:0016020; C:GO:0016021
Acrdi.XM_015902894.1/1-1425 | Monca.augustus.g27533.t1/1-1458	0.17197	0.157484	XK-related 6	1	C:GO:0016020
Acrdi.XM_015922698.1/1-741 | Monca.augustus.g46488.t1/1-762	0.159419	0.157467	PREDICTED: uncharacterized protein LOC107356061		
Acrdi.XM_015891837.1/1-393 | Monca.augustus.g40652.t1/1-657	0.0997873	0.157445	PREDICTED: uncharacterized protein LOC107327084		
Acrdi.XM_015894811.1/1-735 | Monca.augustus.g62470.t1/1-1854	0.161054	0.157371	oxysterol-binding -related 2 isoform X1	1	P:GO:0006869
Acrdi.XM_015906342.1/1-1371 | Monca.augustus.g45326.t1/1-1374	0.206679	0.157328	endonuclease exonuclease phosphatase family	5	P:GO:0090305; F:GO:0004527; F:GO:0004519; C:GO:0016020;
Acrdi.XM_015905609.1/1-990 | Monca.augustus.g65072.t1/1-1140	0.140182	0.157306	ZP domain-containing	2	C:GO:0016020; C:GO:0016021
Acrdi.XM_015904411.1/1-594 | Monca.augustus.g48073.t1/1-594	0.16251	0.157274	ras-related Rab-13	4	F:GO:0005525; P:GO:0001525; P:GO:0007264; C:GO:0005622
Acrdi.XM_015903910.1/1-1812 | Monca.augustus.g30299.t1/1-1815	0.195774	0.157246	F-box LRR-repeat 5	8	P:GO:0055072; C:GO:0048471; C:GO:0005737; F:GO:0005506;
Acrdi.XM_015911436.1/1-1464 | Monca.augustus.g7441.t1/1-1506	0.198367	0.157224	Mitochondrial ribonuclease P 3		
Acrdi.XM_015895907.1/1-981 | Monca.augustus.g30150.t1/1-996	0.198704	0.1572	forkhead box B2-like isoform X1	5	C:GO:0005634; P:GO:0006351; F:GO:0043565; F:GO:0000981;
Acrdi.XM_015910178.1/1-1395 | Monca.augustus.g38616.t1/1-1395	0.206436	0.15717	F-box WD repeat-containing 12		
Acrdi.XM_015913068.1/1-4203 | Monca.adi2mcaRNA23539_R0/1-3981	0.168418	0.157166	low-density lipo receptor-related 6 isoform X2	2	C:GO:0016020; C:GO:0016021
Acrdi.XM_015920831.1/1-1518 | Monca.augustus.g42016.t1/1-1269	0.170995	0.157159	CUB domain-containing -like isoform X5	8	F:GO:0008270; F:GO:0046872; F:GO:0005509; P:GO:0006508;
Acrdi.XM_015901277.1/1-942 | Monca.augustus.g48474.t1/1-924	0.129154	0.157159	histamine H2 receptor-like	2	F:GO:0004930; P:GO:0007165
Acrdi.XM_015896442.1/1-921 | Monca.adi2mcaRNA5280_R0/1-870	0.105092	0.157107	Chymotrypsinogen B	2	P:GO:0006508; F:GO:0004252
Acrdi.XM_015895599.1/1-2493 | Monca.augustus.g68549.t1/1-2085	0.17081	0.157082	ATP-dependent RNA helicase	4	F:GO:0003676; F:GO:0005524; P:GO:0010501; F:GO:0004004
Acrdi.XM_015911655.1/1-1413 | Monca.augustus.g27812.t1/1-1302	0.151522	0.157039	Transmembrane prolyl 4-hydroxylase	6	F:GO:0031418; F:GO:0005506; F:GO:0016491; F:GO:0016705;
Acrdi.XM_015895057.1/1-1545 | Monca.augustus.g16248.t1/1-1710	0.174178	0.157019	cytochrome P450 4V2	4	F:GO:0016491; F:GO:0046872; C:GO:0016020; P:GO:0055114
Acrdi.XM_015899929.1/1-435 | Monca.augustus.g57207.t1/1-432	0.158105	0.156957	PREDICTED: complexin-2-like		
Acrdi.XM_015902560.1/1-1074 | Monca.augustus.g10229.t1/1-1017	0.174901	0.156945	adenosine receptor A1-like	2	P:GO:0007165; F:GO:0004871
Acrdi.XM_015915874.1/1-5091 | Monca.augustus.g39008.t1/1-6339	0.170449	0.156938	NFX1-type zinc finger-containing 1-like	4	C:GO:0005634; F:GO:0003700; F:GO:0008270; P:GO:0006355
Acrdi.XM_015921114.1/1-5769 | Monca.augustus.g48116.t1/1-7110	0.174732	0.156928	phosphatidylinositol phosphatase PTPRQ-like	2	C:GO:0016020; C:GO:0016021
Acrdi.XM_015896745.1/1-606 | Monca.augustus.g59176.t1/1-1077	0.46397	0.156894	PREDICTED: uncharacterized protein LOC107332023		
Acrdi.XM_015909912.1/1-669 | Monca.augustus.g28076.t1/1-678	0.179016	0.156891	PREDICTED: uncharacterized protein LOC107344262		
Acrdi.XM_015900862.1/1-1833 | Monca.adi2mcaRNA10081_R0/1-1842	0.197664	0.156888	phosphatase 1 regulatory subunit 12B	2	C:GO:0016020; C:GO:0016021
Acrdi.XM_015912748.1/1-2304 | Monca.augustus.g26411.t1/1-2406	0.167714	0.156872	meiotically up-regulated gene 158 -like		
Acrdi.XM_015911621.1/1-1335 | Monca.augustus.g24289.t1/1-1428	0.16561	0.15685	zinc transporter ZIP14	7	P:GO:0055085; C:GO:0005737; P:GO:0006829; P:GO:0006826;
Acrdi.XM_015911832.1/1-534 | Monca.augustus.g6325.t1/1-528	0.211662	0.15679	partner of Y14 and mago-like	1	C:GO:0044428
Acrdi.XM_015903384.1/1-942 | Monca.augustus.g36039.t1/1-939	0.182906	0.156783	PREDICTED: uncharacterized protein LOC107338150	4	P:GO:0006869; F:GO:0005319; C:GO:0016020; C:GO:0016021
Acrdi.XM_015909056.1/1-843 | Monca.adi2mcaRNA19080_R0/1-849	0.165783	0.156783	phosphatase 1 regulatory subunit 12B-like	1	F:GO:0019901
Acrdi.XM_015906850.1/1-2877 | Monca.augustus.g32961.t1/1-3513	0.139336	0.156765	speract receptor-like	10	C:GO:0008074; F:GO:0005524; P:GO:0006182; C:GO:0016021;
Acrdi.XM_015908465.1/1-1509 | Monca.augustus.g33926.t1/1-1425	0.17565	0.156749	Coiled-coil domain-containing 15	1	C:GO:0005813
Acrdi.XM_015901102.1/1-861 | Monca.augustus.g1879.t1/1-837	0.114511	0.156737	omega-amidase NIT2	2	F:GO:0016810; P:GO:0006807
Acrdi.XM_015899206.1/1-1206 | Monca.augustus.g1501.t1/1-1287	0.165098	0.15673	homer homolog 2 isoform X2	1	F:GO:0005488
Acrdi.XM_015901324.1/1-408 | Monca.augustus.g58316.t1/1-1491	0.619584	0.156705	PREDICTED: uncharacterized protein LOC107336256		
Acrdi.XM_015911078.1/1-2877 | Monca.augustus.g55128.t1/1-4587	0.172424	0.156694	Adenomatous polyposis coli	47	P:GO:0007050; P:GO:0007094; P:GO:0008283; C:GO:1990909;
Acrdi.XM_015899420.1/1-3561 | Monca.augustus.g46723.t1/1-3522	0.220657	0.156662	tetratricopeptide repeat GNN-like	1	F:GO:0005524
Acrdi.XM_015913002.1/1-1548 | Monca.augustus.g56207.t1/1-1554	0.136139	0.156623	dopamine receptor 2-like	5	F:GO:0001588; P:GO:0001963; P:GO:0007165; F:GO:0035240;
Acrdi.XM_015909013.1/1-963 | Monca.augustus.g12998.t1/1-1932	0.129813	0.156594	transcription factor E2F8	12	F:GO:0003700; F:GO:0042802; P:GO:0045935; P:GO:0031328;
Acrdi.XM_015904379.1/1-2964 | Monca.augustus.g48061.t1/1-2940	0.208669	0.156589	ephrin type-B receptor 1	7	P:GO:0050794; P:GO:0031175; C:GO:0016020; C:GO:0044464;
Acrdi.XM_015894232.1/1-1932 | Monca.augustus.g22487.t1/1-2937	0.177067	0.156528	apoptosis-resistant E3 ubiquitin ligase 1-like	3	F:GO:0003824; C:GO:0044424; P:GO:0016567
Acrdi.XM_015923092.1/1-801 | Monca.augustus.g70889.t1/1-969	0.220784	0.156484	Poly [ADP-ribose] polymerase 3		
Acrdi.XM_015902878.1/1-1668 | Monca.augustus.g27566.t1/1-1668	0.169591	0.156446	TNF receptor-associated factor 3 isoform X2	3	P:GO:0050794; P:GO:0009987; P:GO:0050896
Acrdi.XM_015906837.1/1-549 | Monca.augustus.g23851.t1/1-567	0.0923136	0.156419	PREDICTED: uncharacterized protein LOC107341419		
Acrdi.XM_015909484.1/1-651 | Monca.augustus.g14873.t1/1-1437	0.136402	0.156396	probable carboxypeptidase PM20D1	11	P:GO:0065007; P:GO:0043603; F:GO:0046872; P:GO:0044707;
Acrdi.XM_015915031.1/1-2586 | Monca.adi2mcaRNA25667_R0/1-2520	0.147717	0.156381	cyclic nucleotide-gated cation channel alpha-3-like	4	C:GO:0005887; F:GO:0005249; P:GO:0042391; P:GO:0071805
Acrdi.XM_015906221.1/1-903 | Monca.augustus.g21692.t1/1-1146	0.141727	0.156364	DEP domain-containing mTOR-interacting -like	1	P:GO:0050794
Acrdi.XM_015923248.1/1-1290 | Monca.adi2mcaRNA34880_R1/1-987	0.415691	0.156359	integrase core domain	2	F:GO:0003676; P:GO:0015074
Acrdi.XM_015924753.1/1-4314 | Monca.adi2mcaRNA3743_R0/1-4203	0.20725	0.156355	CD109 antigen		
Acrdi.XM_015898908.1/1-2670 | Monca.adi2mcaRNA8004_R0/1-2235	0.224668	0.156349	F-box WD repeat-containing 1A-like		
Acrdi.XM_015917866.1/1-741 | Monca.augustus.g25192.t1/1-888	0.115403	0.156338	Cyclin-dependent kinase 2	15	P:GO:1904146; C:GO:0000307; F:GO:0043167; C:GO:0005694;
Acrdi.XM_015911567.1/1-858 | Monca.augustus.g57761.t1/1-846	0.200697	0.156327	Cilia- and flagella-associated 97		
Acrdi.XM_015912779.1/1-1119 | Monca.augustus.g9226.t1/1-1233	0.187633	0.156321	Indoleamine 2,3-dioxygenase 2	3	F:GO:0051213; P:GO:0055114; F:GO:0020037
Acrdi.XM_015917438.1/1-1623 | Monca.augustus.g29297.t1/1-2271	0.169376	0.156302	probable ATP-dependent RNA helicase DHX40	6	F:GO:0097159; F:GO:1901363; C:GO:0005737; P:GO:0006396;
Acrdi.XM_015901187.1/1-669 | Monca.augustus.g58598.t1/1-549	0.166661	0.156301	sorting nexin-24-like	1	F:GO:0035091
Acrdi.XM_015909293.1/1-1236 | Monca.adi2mcaRNA19337_R0/1-741	0.204944	0.156236	dual specificity phosphatase 12-like	1	F:GO:0016787
Acrdi.XM_015899116.1/1-1818 | Monca.augustus.g15870.t1/1-1902	0.207048	0.156234	ammonium transporter 3	5	F:GO:0008519; C:GO:0005887; P:GO:0015695; P:GO:0072488;
Acrdi.XM_015896580.1/1-1116 | Monca.augustus.g62599.t1/1-1137	0.175518	0.15621	allatostatin-A receptor-like	4	F:GO:0004930; P:GO:0007186; C:GO:0016021; C:GO:0005886
Acrdi.XM_015913997.1/1-1413 | Monca.augustus.g60907.t1/1-1566	0.175018	0.156206	F-box WD repeat-containing 9		
Acrdi.XM_015919691.1/1-627 | Monca.augustus.g40780.t1/1-636	0.183334	0.156199	ADP-ribosylation factor 15 isoform X1	4	F:GO:0005525; F:GO:0000166; P:GO:0007264; C:GO:0005622
Acrdi.XM_015895034.1/1-1239 | Monca.adi2mcaRNA41404_R0/1-1167	0.184926	0.156145	transcriptional regulator	2	F:GO:0005524; F:GO:0004386
Acrdi.XM_015893832.1/1-1164 | Monca.augustus.g53593.t1/1-2250	0.142278	0.156107	CSC1 ERD4	3	C:GO:0043231; C:GO:0016020; C:GO:0044444
Acrdi.XM_015910376.1/1-1287 | Monca.augustus.g35314.t1/1-1362	0.186246	0.156085	UNC93 MFSD11	5	P:GO:0008150; C:GO:0016020; C:GO:0016021; C:GO:0005575;
Acrdi.XM_015917671.1/1-1917 | Monca.augustus.g26792.t1/1-1848	0.171874	0.156037	seipin-like	1	C:GO:0016020
Acrdi.XM_015910789.1/1-2781 | Monca.augustus.g47995.t1/1-4269	0.178218	0.15602	Peroxinectin A		
Acrdi.XM_015917226.1/1-3315 | Monca.augustus.g55558.t1/1-3363	0.20778	0.155983	transient receptor potential cation channel subfamily A member 1-like		
Acrdi.XM_015900486.1/1-2178 | Monca.augustus.g37059.t1/1-2841	0.159203	0.155972	integrin beta-1 isoform X2	4	P:GO:0007275; P:GO:0055002; P:GO:0009653; P:GO:0016477
Acrdi.XM_015915222.1/1-669 | Monca.augustus.g30658.t1/1-669	0.190242	0.155968	nk homeobox 7	3	C:GO:0005634; P:GO:0006355; F:GO:0043565
Acrdi.XM_015920751.1/1-1152 | Monca.augustus.g35925.t1/1-2160	0.246154	0.155883	gamma-tubulin complex component		
Acrdi.XM_015908761.1/1-1020 | Monca.adi2mcaRNA18764_R0/1-1026	0.182211	0.155881	UDP-galactose translocator-like isoform X3	2	C:GO:0016020; P:GO:0044765
Acrdi.XM_015909443.1/1-756 | Monca.augustus.g13851.t1/1-1305	0.237543	0.155868	zinc finger 277 isoform X2	4	F:GO:0046872; F:GO:0000979; P:GO:0070301; P:GO:2000772
Acrdi.XM_015909714.1/1-1554 | Monca.augustus.g41734.t1/1-1560	0.245977	0.155827	steroid 17-alpha-hydroxylase 17,20 lyase-like isoform X2	1	F:GO:0016491
Acrdi.XM_015924810.1/1-354 | Monca.augustus.g41063.t1/1-381	0.192786	0.155817	elongation factor 1-	3	F:GO:0003746; P:GO:0006414; C:GO:0005853
Acrdi.XM_015899016.1/1-891 | Monca.augustus.g3429.t1/1-885	0.143228	0.155813	hydroxyethylthiazole kinase	2	F:GO:0016740; P:GO:0044237
Acrdi.XM_015920820.1/1-1635 | Monca.adi2mcaRNA32131_R0/1-1566	0.165867	0.155807	ankyrin repeat domain-containing 11-like	2	P:GO:0010972; P:GO:0030336
Acrdi.XM_015905190.1/1-2388 | Monca.augustus.g57158.t1/1-2973	0.167068	0.155792	proteoglycan 4-like		
Acrdi.XM_015892468.1/1-960 | Monca.augustus.g61309.t1/1-1173	0.121035	0.155789	Ubiquitin-conjugating enzyme E2 U	10	F:GO:0000166; P:GO:0006281; F:GO:0061630; P:GO:0043161;
Acrdi.XM_015902304.1/1-978 | Monca.augustus.g35082.t1/1-1758	0.148295	0.15576	E3 ubiquitin- ligase CHFR isoform X4		
Acrdi.XM_015913696.1/1-3603 | Monca.augustus.g45556.t1/1-4722	0.232554	0.155706	ribosome-binding 1-like isoform X2		
Acrdi.XM_015911246.1/1-1539 | Monca.adi2mcaRNA3177_R3/1-1629	0.0826561	0.155702	TGF-beta receptor type-1	7	F:GO:0000166; P:GO:0007165; C:GO:0016020; F:GO:0043167;
Acrdi.XM_015919730.1/1-2001 | Monca.augustus.g68187.t1/1-1848	0.432054	0.15569	collagen alpha-6(VI) chain-like		
Acrdi.XM_015913064.1/1-1257 | Monca.augustus.g25069.t1/1-1428	0.16618	0.155607	neuronal acetylcholine receptor subunit alpha-10-like isoform X2	8	F:GO:0005216; P:GO:0007271; C:GO:0098794; F:GO:0015464;
Acrdi.XM_015914977.1/1-1257 | Monca.augustus.g42435.t1/1-1182	0.194927	0.155523	inositol polyphosphate 1-phosphatase	5	P:GO:0019637; P:GO:0044763; P:GO:0044238; P:GO:0044710;
Acrdi.XM_015918679.1/1-1203 | Monca.adi2mcaRNA29729_R1/1-1227	0.139211	0.155498	prolactin-releasing peptide receptor-like	5	F:GO:0004930; P:GO:0010033; P:GO:0007165; P:GO:0009719;
Acrdi.XM_015910855.1/1-564 | Monca.augustus.g70016.t1/1-564	0.145036	0.155484	PREDICTED: uncharacterized protein LOC107345149		
Acrdi.XM_015913416.1/1-1836 | Monca.augustus.g32132.t1/1-1851	0.123148	0.155464	PREDICTED: trichohyalin-like		
Acrdi.XM_015917490.1/1-657 | Monca.augustus.g32482.t1/1-786	0.237443	0.155455	GTP-binding 8	2	F:GO:0043167; P:GO:0044763
Acrdi.XM_015895176.1/1-537 | Monca.augustus.g2547.t1/1-1800	0.110942	0.15542	nidogen-2 isoform X3		
Acrdi.XM_015910611.1/1-1050 | Monca.augustus.g36893.t1/1-942	0.158031	0.155411	dopamine receptor 2-like	3	F:GO:0004930; P:GO:0007186; C:GO:0016021
Acrdi.XM_015904742.1/1-1092 | Monca.augustus.g4704.t1/1-1134	0.191609	0.155393	HGH1 homolog		
Acrdi.XM_015903560.1/1-393 | Monca.augustus.g45510.t1/1-456	0.160018	0.155382	growth arrest and DNA damage-inducible GADD45 alpha-like	5	C:GO:0005634; P:GO:0000185; C:GO:0005737; P:GO:0051726;
Acrdi.XM_015899262.1/1-3504 | Monca.augustus.g58245.t1/1-3468	0.203661	0.155355	DNA mismatch repair Msh3	6	P:GO:0007131; C:GO:0032300; C:GO:0000228; P:GO:0000710;
Acrdi.XM_015910849.1/1-930 | Monca.augustus.g15803.t1/1-1107	0.236848	0.155347	transmembrane 183A isoform X1		
Acrdi.XM_015892956.1/1-786 | Monca.augustus.g31923.t1/1-1347	0.151802	0.155341	phosphatidylinositol N-acetylglucosaminyltransferase subunit A-like	4	P:GO:0006506; C:GO:0016021; F:GO:0017176; C:GO:0005783
Acrdi.XM_015906691.1/1-6054 | Monca.augustus.g65886.t1/1-6078	0.197761	0.155303	basement membrane-specific heparan sulfate proteoglycan core -like isoform X1		
Acrdi.XM_015921195.1/1-741 | Monca.augustus.g51416.t1/1-723	0.132187	0.155295	MAD2L1-binding	2	C:GO:0005634; P:GO:0007096
Acrdi.XM_015909453.1/1-1590 | Monca.augustus.g69866.t1/1-1557	0.222584	0.155272	RNA-binding 12B	2	F:GO:0000166; F:GO:0003676
Acrdi.XM_015892214.1/1-954 | Monca.augustus.g27420.t1/1-1200	0.14319	0.155236	cystathionine gamma-lyase	3	F:GO:0030170; F:GO:0003824; F:GO:0016829
Acrdi.XM_015901523.1/1-585 | Monca.augustus.g25822.t1/1-561	0.263916	0.155224	sclerostin domain-containing	1	C:GO:0005615
Acrdi.XM_015905181.1/1-543 | Monca.augustus.g1763.t1/1-930	0.165182	0.155212	mitochondrial carrier homolog 2	14	P:GO:1902231; P:GO:0061484; P:GO:0045820; P:GO:0006089;
Acrdi.XM_015909699.1/1-1104 | Monca.augustus.g24121.t1/1-1284	0.210009	0.155211	adenosine receptor A2b	2	F:GO:0004930; P:GO:0007165
Acrdi.XM_015899409.1/1-837 | Monca.augustus.g37208.t1/1-1155	0.241825	0.155191	beta-ureidopropionase	4	F:GO:0016810; C:GO:0070062; P:GO:0006807; F:GO:0003837
Acrdi.XM_015905817.1/1-750 | Monca.augustus.g31756.t1/1-756	0.223164	0.155155	homeobox XENK-2-like	3	C:GO:0005634; P:GO:0006355; F:GO:0043565
Acrdi.XM_015925343.1/1-1293 | Monca.augustus.g6781.t1/1-1395	0.180786	0.155151	UBX domain-containing 6	1	C:GO:0070062
Acrdi.XM_015921220.1/1-1476 | Monca.augustus.g67778.t1/1-2610	0.188675	0.155137	SH3 and PX domain-containing 2B		
Acrdi.XM_015914837.1/1-690 | Monca.adi2mcaRNA25453_R0/1-870	0.151921	0.155093	PREDICTED: uncharacterized protein LOC107348775	2	C:GO:0016020; C:GO:0016021
Acrdi.XM_015923951.1/1-1245 | Monca.adi2mcaRNA35695_R0/1-1251	0.171578	0.155078	zinc metallo ase		
Acrdi.XM_015924071.1/1-16527 | Monca.augustus.g22588.t1/1-12774	0.225227	0.15506	Usherin	2	C:GO:0016020; C:GO:0016021
Acrdi.XM_015911225.1/1-2067 | Monca.augustus.g31450.t1/1-2550	0.165463	0.155059	CAS1 domain-containing 1-like	1	C:GO:0016021
Acrdi.XM_015907245.1/1-708 | Monca.augustus.g11181.t1/1-1080	0.14999	0.155049	Adenosine deaminase	8	F:GO:0046872; C:GO:0005829; P:GO:0006154; P:GO:0043103;
Acrdi.XM_015905017.1/1-3114 | Monca.adi2mcaRNA14621_R1/1-2391	0.531663	0.155042	THAP domain-containing 2		
Acrdi.XM_015919817.1/1-1014 | Monca.augustus.g40321.t1/1-1002	0.171238	0.155034	histamine H2 receptor isoform X1	5	P:GO:0003008; P:GO:0044707; F:GO:0004871; P:GO:0050789;
Acrdi.XM_015909224.1/1-501 | Monca.augustus.g36737.t1/1-507	0.153957	0.155004	universal stress A	1	P:GO:0006950
Acrdi.XM_015912608.1/1-3213 | Monca.augustus.g41502.t1/1-3372	0.183952	0.154956	allene oxide synthase-lipoxygenase -like	3	F:GO:0051213; F:GO:0005488; P:GO:0006631
Acrdi.XM_015918574.1/1-1242 | Monca.augustus.g23483.t1/1-1209	0.191907	0.154948	probable G- coupled receptor 63	4	C:GO:0005887; F:GO:0004930; P:GO:0007186; P:GO:0007166
Acrdi.XM_015916394.1/1-669 | Monca.adi2mcaRNA27180_R0/1-675	0.137274	0.154945	Coiled-coil domain-containing 43	3	P:GO:0008150; C:GO:0005575; F:GO:0003674
Acrdi.XM_015891664.1/1-10902 | Monca.augustus.g34616.t1/1-11526	0.176725	0.154866	golgin subfamily A member 4-like		
Acrdi.XM_015908924.1/1-699 | Monca.augustus.g31865.t1/1-702	0.145843	0.154861	leucine-rich repeat extensin 3		
Acrdi.XM_015923820.1/1-1245 | Monca.augustus.g33428.t1/1-3678	0.240876	0.15482	patched homolog 1	4	P:GO:0007165; C:GO:0016020; C:GO:0016021; F:GO:0008158
Acrdi.XM_015917829.1/1-1398 | Monca.augustus.g7374.t1/1-1620	0.175295	0.154807	ceramide kinase-like isoform X2	2	P:GO:0016310; F:GO:0016301
Acrdi.XM_015909024.1/1-765 | Monca.augustus.g38378.t1/1-768	0.174728	0.154788	dickkopf-related 3-like	3	P:GO:0007275; P:GO:0030178; C:GO:0005576
Acrdi.XM_015913180.1/1-996 | Monca.augustus.g2108.t1/1-1023	0.173242	0.154698	cyclin-H	6	C:GO:0005634; P:GO:0045737; F:GO:0016538; P:GO:1901409;
Acrdi.XM_015921390.1/1-4083 | Monca.augustus.g61089.t1/1-3690	0.135157	0.154628	eukaryotic translation initiation factor 3 subunit A	12	F:GO:0003743; P:GO:0075522; P:GO:0001731; F:GO:0005515;
Acrdi.XM_015898245.1/1-1089 | Monca.adi2mcaRNA7230_R0/1-1077	0.181132	0.154627	histamine H2 receptor-like	3	F:GO:0004930; P:GO:0007154; P:GO:0044700
Acrdi.XM_015905965.1/1-426 | Monca.augustus.g69493.t1/1-426	0.207062	0.154607	39S ribosomal mitochondrial	2	C:GO:0044429; C:GO:0015934
Acrdi.XM_015905851.1/1-2031 | Monca.augustus.g61400.t1/1-2055	0.178724	0.154566	white	6	F:GO:0000166; F:GO:0016887; F:GO:0005524; C:GO:0016020;
Acrdi.XM_015914865.1/1-4515 | Monca.augustus.g48214.t1/1-4383	0.214565	0.154552	breakpoint cluster region isoform X2	4	P:GO:0050794; C:GO:0016020; F:GO:0005096; P:GO:0043547
Acrdi.XM_015916991.1/1-4104 | Monca.adi2mcaRNA27872_R0/1-4113	0.212227	0.154517	Methylcytosine dioxygenase	11	P:GO:0080182; F:GO:0008270; F:GO:0005515; P:GO:0006493;
Acrdi.XM_015902174.1/1-1983 | Monca.augustus.g34042.t1/1-4443	0.182294	0.154498	DENN domain-containing 3-like		
Acrdi.XM_015898248.1/1-1026 | Monca.augustus.g45301.t1/1-996	0.172539	0.154497	histamine H2 receptor-like	3	F:GO:0004930; P:GO:0007154; P:GO:0044700
Acrdi.XM_015918591.1/1-585 | Monca.augustus.g35360.t1/1-1734	0.154928	0.154488	follicle-stimulating hormone receptor-like isoform X1	3	F:GO:0004930; P:GO:0007186; C:GO:0016021
Acrdi.XM_015913743.1/1-354 | Monca.augustus.g21657.t1/1-819	0.18804	0.154472	Complement component 1 Q subcomponent-binding mitochondrial	1	C:GO:0005759
Acrdi.XM_015911915.1/1-1611 | Monca.augustus.g30051.t1/1-2727	0.152922	0.154451	double-stranded RNA-specific adenosine deaminase isoform X2	4	F:GO:0003723; F:GO:0003726; P:GO:0006396; F:GO:0004000
Acrdi.XM_015899260.1/1-1230 | Monca.augustus.g62001.t1/1-1350	0.179419	0.154434	repulsive guidance molecule A-like	3	F:GO:0015026; P:GO:0030509; C:GO:0005886
Acrdi.XM_015899391.1/1-6228 | Monca.adi2mcaRNA8521_R0/1-6222	0.213484	0.154418	Collagen alpha-1(VII) chain		
Acrdi.XM_015922723.1/1-1440 | Monca.augustus.g6733.t1/1-1443	0.159407	0.154382	PREDICTED: uncharacterized protein LOC107356075 isoform X1	5	C:GO:0016020; C:GO:0016021; P:GO:0036071; P:GO:0006487;
Acrdi.XM_015916405.1/1-450 | Monca.augustus.g40950.t1/1-450	0.175308	0.154365	E3 ubiquitin- ligase RNF181	3	F:GO:0003824; P:GO:0006511; P:GO:0016567
Acrdi.XM_015903416.1/1-1539 | Monca.augustus.g18163.t1/1-1542	0.161058	0.154364	MFS transporter	1	C:GO:0016020
Acrdi.XM_015903580.1/1-1119 | Monca.augustus.g19373.t1/1-1116	0.193257	0.154358	palmitoyltransferase ZDHHC23 isoform X1	1	F:GO:0016740
Acrdi.XM_015916424.1/1-528 | Monca.augustus.g64492.t1/1-561	0.213285	0.154343	mini-	1	C:GO:0005581
Acrdi.XM_015906676.1/1-900 | Monca.augustus.g38580.t1/1-948	0.20363	0.154334	sulfatase-modifying factor 2-like	1	C:GO:0005783
Acrdi.XM_015904687.1/1-2604 | Monca.augustus.g22469.t1/1-2796	0.243802	0.15433	transmembrane 232-like	5	F:GO:0061630; F:GO:0031624; P:GO:0000209; P:GO:0032436;
Acrdi.XM_015924658.1/1-2469 | Monca.augustus.g28215.t1/1-2478	0.184074	0.154318	Kinesin light chain 3	5	P:GO:0007165; C:GO:0016020; C:GO:0016021; P:GO:0042981;
Acrdi.XM_015912611.1/1-729 | Monca.augustus.g12811.t1/1-720	0.211345	0.1543	low-density lipo receptor-related 8-like	5	F:GO:0005509; C:GO:0016020; C:GO:0016021; C:GO:0005794;
Acrdi.XM_015921514.1/1-798 | Monca.augustus.g42444.t1/1-789	0.146214	0.154294	Single-strand selective monofunctional uracil DNA glycosylase	3	F:GO:0004844; F:GO:0000703; C:GO:0031981
Acrdi.XM_015911674.1/1-1041 | Monca.augustus.g27817.t1/1-1050	0.189095	0.154289	trace amine-associated receptor 5-like	3	F:GO:0001588; P:GO:0001963; F:GO:0035240
Acrdi.XM_015920142.1/1-1185 | Monca.augustus.g27085.t1/1-1092	0.105188	0.154233	Zinc finger Gfi-1b	8	F:GO:0003676; C:GO:0005667; F:GO:0001085; F:GO:0046872;
Acrdi.XM_015902222.1/1-1098 | Monca.augustus.g34159.t1/1-1332	0.238357	0.15423	odr-4 homolog	2	C:GO:0016020; C:GO:0016021
Acrdi.XM_015904342.1/1-336 | Monca.augustus.g61766.t1/1-357	0.163754	0.154168	PREDICTED: uncharacterized protein LOC107339100	2	C:GO:0016020; C:GO:0016021
Acrdi.XM_015918491.1/1-1017 | Monca.augustus.g55069.t1/1-1038	0.127517	0.154166	somatostatin receptor type 1-like	7	F:GO:0004930; F:GO:0001588; P:GO:0001963; P:GO:0007186;
Acrdi.XM_015902425.1/1-1080 | Monca.augustus.g37588.t1/1-1062	0.172561	0.154161	methyltransferase C9orf114 homolog	3	P:GO:0008150; C:GO:0005575; F:GO:0003674
Acrdi.XM_015924899.1/1-2859 | Monca.augustus.g35005.t1/1-3747	0.215497	0.154104	pecanex 4	2	C:GO:0016020; C:GO:0016021
Acrdi.XM_015907022.1/1-1560 | Monca.augustus.g43291.t1/1-1419	0.17956	0.154081	transmembrane 104 isoform X1	2	C:GO:0016020; C:GO:0016021
Acrdi.XM_015902537.1/1-3795 | Monca.augustus.g39926.t1/1-3540	0.159501	0.154052	transport Sec24B	2	P:GO:0046907; P:GO:0015031
Acrdi.XM_015912809.1/1-489 | Monca.augustus.g9383.t1/1-1224	0.222401	0.154041	phosphatase 1 regulatory subunit 7-like	3	C:GO:0005634; F:GO:0031491; P:GO:0006336
Acrdi.XM_015915328.1/1-816 | Monca.augustus.g25260.t1/1-1056	0.153594	0.154029	THUMP domain-containing	1	F:GO:0003723
Acrdi.XM_015904811.1/1-1974 | Monca.augustus.g29900.t1/1-2049	0.169663	0.154011	von Willebrand factor D and EGF domain-containing -like		
Acrdi.XM_015901528.1/1-933 | Monca.augustus.g14966.t1/1-933	0.207233	0.154001	PREDICTED: uncharacterized protein LOC107336450		
Acrdi.XM_015916324.1/1-501 | Monca.augustus.g49888.t1/1-798	0.230535	0.153989	rolling stone-like	2	C:GO:0016020; C:GO:0016021
Acrdi.XM_015910477.1/1-1536 | Monca.augustus.g41054.t1/1-1581	0.139418	0.153986	U3 snoRNP-associated Utp7	5	C:GO:0032040; P:GO:0000462; P:GO:0000479; C:GO:0005730;
Acrdi.XM_015898022.1/1-1272 | Monca.augustus.g28148.t1/1-1257	0.205364	0.153984	HCLS1-binding 3-like isoform X1	1	F:GO:0035091
Acrdi.XM_015905999.1/1-1011 | Monca.adi2mcaRNA15678_R2/1-1047	0.306183	0.153978	PREDICTED: uncharacterized protein LOC107340633	1	F:GO:0046872
Acrdi.XM_015897790.1/1-3183 | Monca.adi2mcaRNA6767_R0/1-3210	0.175919	0.153971	DNA topoisomerase 2-binding 1		
Acrdi.XM_015906151.1/1-909 | Monca.augustus.g29203.t1/1-909	0.167464	0.153962	Hydroxysteroid 11-beta-dehydrogenase 1	2	F:GO:0016491; P:GO:0055114
Acrdi.XM_015915066.1/1-1146 | Monca.augustus.g27924.t1/1-1152	0.173367	0.15396	myosin-J heavy chain		
Acrdi.XM_015903856.1/1-2052 | Monca.augustus.g32295.t1/1-3285	0.235167	0.153959	Centrobin	7	F:GO:0019904; C:GO:0005813; P:GO:0051299; C:GO:0005737;
Acrdi.XM_015903343.1/1-897 | Monca.augustus.g36046.t1/1-1296	0.180219	0.153948	kaptin isoform X2	8	P:GO:0008150; F:GO:0003779; C:GO:0005737; C:GO:0015629;
Acrdi.XM_015922230.1/1-576 | Monca.adi2mcaRNA33682_R7/1-615	0.633446	0.153924	zinc finger 862-like	4	F:GO:0003677; C:GO:0005737; C:GO:0005654; F:GO:0000981
Acrdi.XM_015896416.1/1-1140 | Monca.augustus.g22663.t1/1-1140	0.164894	0.153882	doublesex- and mab-3-related transcription factor A2-like isoform X1		
Acrdi.XM_015898692.1/1-615 | Monca.augustus.g2075.t1/1-972	0.159813	0.153881	Elongator complex 5	1	C:GO:0033588
Acrdi.XM_015923292.1/1-972 | Monca.augustus.g55709.t1/1-1062	0.158753	0.153875	adenosine kinase isoform X1	8	C:GO:0005634; C:GO:0005737; P:GO:0006167; P:GO:0006166;
Acrdi.XM_015915599.1/1-387 | Monca.augustus.g5416.t1/1-1236	0.201958	0.153873	Peroxisomal membrane PEX14	2	C:GO:0005778; P:GO:0016560
Acrdi.XM_015923088.1/1-858 | Monca.augustus.g70880.t1/1-858	0.154261	0.153838	meteorin	2	P:GO:0010951; F:GO:0008191
Acrdi.XM_015909644.1/1-1281 | Monca.augustus.g6198.t1/1-1320	0.183104	0.153833	monocarboxylate transporter 10-like	5	P:GO:0055085; P:GO:0034220; C:GO:0016020; C:GO:0016021;
Acrdi.XM_015892875.1/1-465 | Monca.adi2mcaRNA38902_R0/1-453	0.226916	0.153818	Counting factor associated D	4	P:GO:0006508; P:GO:0043065; P:GO:0010952; F:GO:0008234
Acrdi.XM_015892381.1/1-2157 | Monca.augustus.g26882.t1/1-1254	0.125974	0.153784	delta(14)-sterol reductase isoform X2	6	F:GO:0016627; C:GO:0043231; C:GO:0016021; C:GO:0012505;
Acrdi.XM_015910401.1/1-1593 | Monca.augustus.g70899.t1/1-1599	0.15235	0.153684	potassium channel	2	F:GO:0005216; C:GO:0016020
Acrdi.XM_015922992.1/1-2859 | Monca.augustus.g21628.t1/1-5940	0.17075	0.153679	rho GTPase-activating 21-like		
Acrdi.XM_015892075.1/1-801 | Monca.augustus.g38771.t1/1-987	0.207093	0.153657	low density lipo receptor adapter 1		
Acrdi.XM_015898852.1/1-891 | Monca.augustus.g39884.t1/1-891	0.21272	0.153589	E3 ubiquitin- ligase RNF144A isoform X1	4	F:GO:0004842; C:GO:0005737; F:GO:0005488; P:GO:0016567
Acrdi.XM_015895883.1/1-2265 | Monca.adi2mcaRNA4698_R0/1-2091	0.14887	0.153553	synaptotagmin-7-like isoform X1	10	C:GO:0070382; C:GO:0098793; F:GO:0048791; F:GO:0019905;
Acrdi.XM_015903453.1/1-2730 | Monca.augustus.g12663.t1/1-2529	0.216009	0.153552	SID1 transmembrane family member 1 isoform X2		
Acrdi.XM_015907459.1/1-300 | Monca.augustus.g43831.t1/1-426	0.17696	0.153533	transmembrane 254 isoform X2	2	C:GO:0016020; C:GO:0016021
Acrdi.XM_015904307.1/1-4011 | Monca.augustus.g5864.t1/1-4443	0.167223	0.153505	potassium voltage-gated channel subfamily H member 8	8	F:GO:0000155; C:GO:0005887; F:GO:0005249; P:GO:0042391;
Acrdi.XM_015917253.1/1-876 | Monca.adi2mcaRNA28169_R8/1-855	0.297186	0.153492	Fibrinogen C domain-containing 1		
Acrdi.XM_015903047.1/1-3600 | Monca.augustus.g45208.t1/1-3678	0.146076	0.153488	Erythroid differentiation-related factor 1		
Acrdi.XM_015921745.1/1-642 | Monca.augustus.g49908.t1/1-690	0.185642	0.153474	probable peptidyl-tRNA hydrolase	3	C:GO:0005739; P:GO:0032543; F:GO:0004045
Acrdi.XM_015903620.1/1-2619 | Monca.augustus.g66464.t1/1-2628	0.159524	0.153473	metabotropic glutamate receptor 3	6	C:GO:0005887; F:GO:0008066; C:GO:0097458; C:GO:0044456;
Acrdi.XM_015891714.1/1-1929 | Monca.augustus.g52053.t1/1-2367	0.146452	0.153442	pantothenate kinase 4-like	2	F:GO:0016301; P:GO:0006796
Acrdi.XM_015924627.1/1-468 | Monca.augustus.g37222.t1/1-537	0.198246	0.153417	All-trans retinoic acid-induced differentiation factor	2	C:GO:0016020; C:GO:0016021
Acrdi.XM_015923126.1/1-2001 | Monca.augustus.g32470.t1/1-1983	0.203344	0.153377	stromal interaction molecule 1 isoform X5	1	C:GO:0016021
Acrdi.XM_015907060.1/1-2520 | Monca.augustus.g43279.t1/1-4176	0.204271	0.153375	fas-binding factor 1 isoform X1	2	C:GO:0044430; P:GO:0044763
Acrdi.XM_015893602.1/1-567 | Monca.augustus.g25386.t1/1-672	0.635173	0.153368	TNF receptor-associated factor 5-like	4	F:GO:0004842; F:GO:0008270; F:GO:0046872; P:GO:0016567
Acrdi.XM_015924051.1/1-1806 | Monca.augustus.g33008.t1/1-1602	0.12931	0.153366	neuronal acetylcholine receptor subunit alpha-7-like	5	F:GO:0005216; F:GO:0099600; P:GO:0034220; C:GO:0016021;
Acrdi.XM_015911352.1/1-891 | Monca.adi2mcaRNA21582_R0/1-849	0.1706	0.153341	pyroglutamylated RFamide peptide receptor-like isoform X1	2	P:GO:0007165; C:GO:0016020
Acrdi.XM_015904850.1/1-1677 | Monca.adi2mcaRNA14388_R1/1-1818	0.149897	0.153335	neuronal acetylcholine receptor subunit alpha-7-like	3	F:GO:0005216; P:GO:0034220; C:GO:0044425
Acrdi.XM_015914154.1/1-654 | Monca.augustus.g24973.t1/1-2823	0.177115	0.153319	formin-binding 4		
Acrdi.XM_015897825.1/1-2274 | Monca.augustus.g13587.t1/1-2658	0.189002	0.153315	PREDICTED: uncharacterized protein LOC107333066 isoform X2		
Acrdi.XM_015903044.1/1-828 | Monca.augustus.g45220.t1/1-1149	0.169305	0.15331	STAGA complex 65 subunit gamma	6	P:GO:0043966; F:GO:0004402; F:GO:0003713; C:GO:0030914;
Acrdi.XM_015910754.1/1-3570 | Monca.augustus.g29672.t1/1-3198	0.157523	0.153237	serine threonine- kinase HT1	6	F:GO:0005524; F:GO:0004672; P:GO:0016310; F:GO:0004674;
Acrdi.XM_015897364.1/1-2739 | Monca.augustus.g29315.t1/1-3633	0.17456	0.153171	Neurogenic locus notch 3	1	F:GO:0070492
Acrdi.XM_015903985.1/1-867 | Monca.augustus.g2918.t1/1-969	0.0721112	0.153158	ubiquitin-like domain-containing CTD phosphatase 1		
Acrdi.XM_015900175.1/1-675 | Monca.augustus.g59989.t1/1-663	0.161165	0.153156	Cytochrome c oxidase assembly factor 7	4	P:GO:0008150; C:GO:0005739; C:GO:0005575; F:GO:0003674
Acrdi.XM_015896815.1/1-684 | Monca.augustus.g49310.t1/1-1305	0.158421	0.153156	CD209 antigen E		
Acrdi.XM_015923378.1/1-2754 | Monca.adi2mcaRNA35037_R0/1-2664	0.193247	0.15311	GPI inositol-deacylase	3	F:GO:0016788; C:GO:0016020; C:GO:0016021
Acrdi.XM_015905380.1/1-417 | Monca.augustus.g61126.t1/1-420	0.199993	0.153072	complement component C6	6	C:GO:0005579; P:GO:0006955; C:GO:0016020; C:GO:0016021;
Acrdi.XM_015917372.1/1-660 | Monca.adi2mcaRNA28333_R0/1-660	0.177138	0.153039	multiple epidermal growth factor-like domains 6		
Acrdi.XM_015905282.1/1-756 | Monca.adi2mcaRNA14882_R3/1-678	0.237423	0.153034	multifunctional hydroxymethylpyrimidine phosphokinase 4-amino-5-aminomethyl-2-methylpyrimidine hydrolase		
Acrdi.XM_015902846.1/1-897 | Monca.augustus.g27229.t1/1-1116	0.191079	0.153032	calumenin-like isoform X1	1	F:GO:0005509
Acrdi.XM_015902689.1/1-933 | Monca.augustus.g22681.t1/1-870	0.146948	0.153023	cyclase	3	F:GO:0004061; P:GO:0019441; C:GO:0016020
Acrdi.XM_015910829.1/1-1611 | Monca.augustus.g57313.t1/1-1614	0.170498	0.153014	Circadian locomoter output cycles	8	F:GO:0003677; C:GO:0005634; C:GO:0005667; F:GO:0003700;
Acrdi.XM_015913829.1/1-2040 | Monca.adi2mcaRNA24340_R1/1-2049	0.15781	0.152978	transmembrane 62	3	C:GO:0016020; C:GO:0016021; F:GO:0016787
Acrdi.XM_015902114.1/1-1281 | Monca.augustus.g55027.t1/1-1296	0.135166	0.152934	PARP domain-containing	4	C:GO:0005737; F:GO:0016740; P:GO:0006259; P:GO:0034645
Acrdi.XM_015914383.1/1-1014 | Monca.augustus.g61747.t1/1-1749	0.138682	0.152917	Translation initiation factor eIF-2B subunit delta	7	P:GO:0045947; P:GO:0019509; F:GO:0005085; F:GO:0046523;
Acrdi.XM_015912911.1/1-1137 | Monca.augustus.g63789.t1/1-2016	0.137822	0.15285	RNA-directed RNA polymerase	2	F:GO:0003968; P:GO:0001172
Acrdi.XM_015918855.1/1-10470 | Monca.adi2mcaRNA29946_R0/1-9408	0.152938	0.152775	Transformation transcription domain-associated	3	P:GO:0016573; C:GO:0043234; C:GO:0000123
Acrdi.XM_015911312.1/1-1920 | Monca.augustus.g67447.t1/1-1878	0.154601	0.152721	Solute carrier family 15 member 4	4	C:GO:0016020; C:GO:0016021; P:GO:0006810; F:GO:0005215
Acrdi.XM_015902137.1/1-1008 | Monca.augustus.g46377.t1/1-972	0.138609	0.15272	histamine H2 receptor-like	9	F:GO:0004930; F:GO:0001588; P:GO:0001963; P:GO:0007186;
Acrdi.XM_015917415.1/1-1026 | Monca.augustus.g66921.t1/1-1047	0.172515	0.152702	adenosine receptor A3-like	1	P:GO:0007165
Acrdi.XM_015924963.1/1-882 | Monca.augustus.g40887.t1/1-885	0.160105	0.152699	retinol dehydrogenase 8-like	4	F:GO:0016491; C:GO:0016020; C:GO:0016021; P:GO:0055114
Acrdi.XM_015908506.1/1-585 | Monca.augustus.g46700.t1/1-588	0.0789262	0.152669	PREDICTED: uncharacterized protein LOC107342983		
Acrdi.XM_015918867.1/1-363 | Monca.augustus.g25201.t1/1-360	0.210235	0.152668	PREDICTED: uncharacterized protein LOC107352543		
Acrdi.XM_015907544.1/1-1302 | Monca.augustus.g28070.t1/1-1989	0.112981	0.152653	E3 ubiquitin- ligase RFWD2-like		
Acrdi.XM_015924555.1/1-645 | Monca.augustus.g26852.t1/1-1314	0.159566	0.152622	eukaryotic translation initiation factor 2 alpha kinase	2	F:GO:0003824; P:GO:0006810
Acrdi.XM_015901605.1/1-390 | Monca.adi2mcaRNA10907_R0/1-405	0.55271	0.152595	PREDICTED: uncharacterized protein LOC107336533		
Acrdi.XM_015907313.1/1-4125 | Monca.adi2mcaRNA17199_R0/1-3681	0.149689	0.152569	1-phosphatidylinositol 4,5-bisphosphate phosphodiesterase beta-4 isoform	7	C:GO:0005634; C:GO:0014069; C:GO:0030425; F:GO:0005515;
Acrdi.XM_015898339.1/1-1416 | Monca.augustus.g66916.t1/1-1689	0.197973	0.152538	Histone-lysine N-methyltransferase SMYD3	1	F:GO:0046872
Acrdi.XM_015911921.1/1-1269 | Monca.augustus.g58623.t1/1-1656	0.153551	0.152535	Transmembrane 7 superfamily member 3	2	C:GO:0016020; C:GO:0016021
Acrdi.XM_015922570.1/1-1407 | Monca.augustus.g54975.t1/1-1392	0.13014	0.152512	Neuropeptide FF receptor 2	6	C:GO:0005887; F:GO:0004930; P:GO:0010033; P:GO:0007186;
Acrdi.XM_015917469.1/1-783 | Monca.augustus.g29303.t1/1-2247	0.216783	0.15249	PREDICTED: uncharacterized protein LOC107351178		
Acrdi.XM_015900510.1/1-1143 | Monca.augustus.g68178.t1/1-1152	0.0961369	0.152488	Zinc finger 622	4	F:GO:0046872; P:GO:0042273; C:GO:0022625; C:GO:0030687
Acrdi.XM_015911415.1/1-1029 | Monca.augustus.g70351.t1/1-999	0.178287	0.152486	Sorting nexin-21	1	F:GO:0035091
Acrdi.XM_015899329.1/1-459 | Monca.augustus.g64404.t1/1-672	0.125904	0.152456	Ras-related and estrogen-regulated growth inhibitor	6	F:GO:0000166; F:GO:0005525; P:GO:0007264; P:GO:0007165;
Acrdi.XM_015900772.1/1-303 | Monca.augustus.g41123.t1/1-1452	0.268394	0.152438	beta-1,3-glucosyltransferase-like	2	F:GO:0016757; C:GO:0016020
Acrdi.XM_015911578.1/1-11622 | Monca.augustus.g54368.t1/1-10551	0.164939	0.152435	Protocadherin Fat 4	1	P:GO:0048513
Acrdi.XM_015912097.1/1-2835 | Monca.augustus.g24181.t1/1-3378	0.147213	0.152435	hephaestin 1		
Acrdi.XM_015906496.1/1-9207 | Monca.augustus.g22533.t1/1-13470	0.200168	0.152433	sacsin isoform X2		
Acrdi.XM_015906517.1/1-930 | Monca.augustus.g22555.t1/1-915	0.209097	0.152389	5-hydroxytryptamine receptor 1A-alpha-like	3	F:GO:0004930; P:GO:0007154; P:GO:0044700
Acrdi.XM_015918477.1/1-1335 | Monca.augustus.g55060.t1/1-1320	0.182674	0.152371	Vesicular inhibitory amino acid transporter	4	C:GO:0016020; F:GO:0015171; C:GO:0043005; P:GO:0003333
Acrdi.XM_015913759.1/1-1464 | Monca.augustus.g12529.t1/1-1398	0.174121	0.15236	RUS1 family C16orf58 homolog	1	C:GO:0016021
Acrdi.XM_015900798.1/1-1074 | Monca.augustus.g58755.t1/1-1080	0.15737	0.152304	tyramine receptor Ser-2-like	2	P:GO:0007210; F:GO:0004993
Acrdi.XM_015910727.1/1-1350 | Monca.adi2mcaRNA20940_R0/1-1134	0.165646	0.15229	acid sphingomyelinase-like phosphodiesterase 3b	5	C:GO:0005615; F:GO:0004767; C:GO:0070062; P:GO:0006685;
Acrdi.XM_015923971.1/1-483 | Monca.augustus.g18225.t1/1-492	0.204133	0.152205	28S ribosomal mitochondrial	4	F:GO:0003735; C:GO:0022627; P:GO:0032543; C:GO:0005763
Acrdi.XM_015909980.1/1-468 | Monca.augustus.g30682.t1/1-2145	0.165742	0.152204	PREDICTED: uncharacterized protein LOC107344326		
Acrdi.XM_015900768.1/1-513 | Monca.augustus.g11794.t1/1-786	0.11569	0.152183	Negative elongation factor E	4	F:GO:0000166; F:GO:0003676; C:GO:0032021; P:GO:0006355
Acrdi.XM_015901746.1/1-885 | Monca.augustus.g14597.t1/1-921	0.217749	0.152164	Carboxypeptidase Q	4	C:GO:0005615; P:GO:0043171; F:GO:0070573; P:GO:0006508
Acrdi.XM_015924900.1/1-1287 | Monca.adi2mcaRNA36766_R0/1-1296	0.155259	0.152147	TNF receptor-associated factor 4-like	4	F:GO:0004842; F:GO:0008270; F:GO:0046872; P:GO:0016567
Acrdi.XM_015919334.1/1-1620 | Monca.augustus.g71328.t1/1-1647	0.18282	0.152141	Sialate O-acetylesterase		
Acrdi.XM_015908005.1/1-603 | Monca.augustus.g43115.t1/1-798	0.132127	0.152097	bombesin receptor subtype-3	11	P:GO:0031989; F:GO:0004930; C:GO:0005887; F:GO:0008528;
Acrdi.XM_015911637.1/1-564 | Monca.augustus.g24287.t1/1-1203	0.131971	0.152064	heterogeneous nuclear ribonucleo 27C	15	P:GO:0045727; P:GO:0007411; P:GO:0007298; C:GO:0043234;
Acrdi.XM_015911657.1/1-801 | Monca.augustus.g27814.t1/1-1335	0.115409	0.152063	transmembrane prolyl 4-hydroxylase-like	2	F:GO:0016491; F:GO:0046872

Acrdi.XM_015893057.1/1-807	Monca.augustus.g58646.t1/1-4659	0.177365	0.15205	leucine-rich repeat and IQ domain-containing 1-like	
Acrdi.XM_015896753.1/1-717	Monca.augustus.g42421.t1/1-657	0.178788	0.152036	Transcription factor 21	1
Acrdi.XM_015907440.1/1-1170	Monca.augustus.g7875.t1/1-1485	0.173879	0.152032	rabenosyn-5-like	7
Acrdi.XM_015894940.1/1-4461	Monca.augustus.g30245.t1/1-528	0.713265	0.152031	rotatin isoform X1	3
Acrdi.XM_015909198.1/1-1347	Monca.augustus.g44597.t1/1-1584	0.158224	0.15202	steroid 17-alpha-hydroxylase 17,20 lyase-like isoform X2	4
Acrdi.XM_015901537.1/1-2280	Monca.augustus.g65888.t1/1-2271	0.213602	0.152001	RAD50-interacting 1	2
Acrdi.XM_015906297.1/1-423	Monca.augustus.g26546.t1/1-426	0.129401	0.151999	Fibroblast growth factor 1	5
Acrdi.XM_015910018.1/1-2277	Monca.augustus.g49041.t1/1-2337	0.21447	0.151997	mastermind 3 isoform X2	5
Acrdi.XM_015891992.1/1-1929	Monca.augustus.g36363.t1/1-1905	0.153985	0.151952	Leishmanolysin-like peptidase	4
Acrdi.XM_015923898.1/1-2424	Monca.augustus.g40431.t1/1-3357	0.190226	0.151949	GAS2 2	1
Acrdi.XM_015895912.1/1-540	Monca.augustus.g30148.t1/1-537	0.254595	0.151938	histidine-rich glyco -like	
Acrdi.XM_015909108.1/1-3702	Monca.augustus.g23912.t1/1-4044	0.181037	0.151906	Canalicular multispecific organic anion transporter	9
Acrdi.XM_015918001.1/1-3273	Monca.augustus.g16990.t1/1-3339	0.168901	0.151905	HEAT repeat-containing 4-like	
Acrdi.XM_015903394.1/1-693	Monca.augustus.g36023.t1/1-669	0.176838	0.151892	tRNA 2 -phosphotransferase 1	2
Acrdi.XM_015915182.1/1-1212	Monca.augustus.g29750.t1/1-1245	0.14097	0.15189	band 1	1
Acrdi.XM_015913216.1/1-888	Monca.augustus.g32071.t1/1-879	0.162397	0.151865	Failed axon connections-like	2
Acrdi.XM_015912015.1/1-903	Monca.augustus.g35305.t1/1-825	0.145377	0.151852	melanocyte-stimulating hormone receptor-like	6
Acrdi.XM_015904719.1/1-1953	Monca.augustus.g36186.t1/1-3759	0.18141	0.151851	mediator of RNA polymerase II transcription subunit 12-like	
Acrdi.XM_015914998.1/1-1044	Monca.augustus.g21910.t1/1-1071	0.299985	0.151841	Low density lipo receptor adapter 1-A	
Acrdi.XM_015923265.1/1-669	Monca.augustus.g41205.t1/1-672	0.194398	0.151841	homeobox OTX-like isoform X1	5
Acrdi.XM_015922894.1/1-909	Monca.adi2mcaRNA3453_R0/1-756	0.165996	0.151815	Serologically defined colon cancer antigen 3	
Acrdi.XM_015892288.1/1-1713	Monca.augustus.g57164.t1/1-5016	0.182434	0.151807	C-type lectin domain family member A	
Acrdi.XM_015903500.1/1-675	Monca.adi2mcaRNA12942_R0/1-582	0.0860833	0.151796	ribosome production factor 1-like	6
Acrdi.XM_015908349.1/1-12693	Monca.adi2mcaRNA18304_R0/1-1236	0.196147	0.151764	nidogen-2 isoform X1	
Acrdi.XM_015899484.1/1-2415	Monca.augustus.g37232.t1/1-2409	0.209972	0.151738	SET and MYND domain-containing 4 isoform X1	
Acrdi.XM_015909704.1/1-723	Monca.augustus.g55271.t1/1-729	0.157552	0.151731	PREDICTED: uncharacterized protein LOC107344095	5
Acrdi.XM_015923696.1/1-372	Monca.augustus.g30538.t1/1-369	0.244125	0.151711	NADH dehydrogenase [ubiquinone] iron-sulfur mitochondrial	2
Acrdi.XM_015919246.1/1-1032	Monca.adi2mcaRNA30386_R1/1-1035	0.420691	0.151696	Arachidonate 5-lipoxygenase	5
Acrdi.XM_015923682.1/1-747	Monca.augustus.g34979.t1/1-924	0.225571	0.151687	PREDICTED: uncharacterized protein LOC107357046, partial	2
Acrdi.XM_015891641.1/1-429	Monca.adi2mcaRNA37489_R1/1-498	0.195589	0.151687	Glutathione mitochondrial	
Acrdi.XM_015897692.1/1-1668	Monca.augustus.g13629.t1/1-1680	0.134144	0.151633	mitoguardin-like isoform X2	1
Acrdi.XM_015919573.1/1-1026	Monca.adi2mcaRNA30781_R1/1-1023	0.151125	0.15162	gamma-glutamyl hydrolase-like	3
Acrdi.XM_015923779.1/1-864	Monca.augustus.g54323.t1/1-1587	0.195446	0.15156	Ectonucleoside triphosphate diphosphohydrolase 2	3
Acrdi.XM_015913144.1/1-4179	Monca.augustus.g39276.t1/1-5775	0.189251	0.151551	probable RNA-dependent RNA polymerase 1	2
Acrdi.XM_015911795.1/1-1065	Monca.augustus.g38093.t1/1-1062	0.208522	0.151543	inositol monophosphatase 3-like	10
Acrdi.XM_015920121.1/1-1026	Monca.adi2mcaRNA31355_R0/1-705	0.517941	0.151526	guanylate-binding 1-like	
Acrdi.XM_015894702.1/1-1581	Monca.augustus.g27845.t1/1-1734	0.195285	0.151526	methyltransferase mitochondrial	
Acrdi.XM_015908878.1/1-720	Monca.augustus.g8653.t1/1-723	0.169682	0.151516	PREDICTED: uncharacterized protein LOC107343316	
Acrdi.XM_015897235.1/1-2904	Monca.augustus.g31744.t1/1-3861	0.172406	0.151506	DIS3-like exonuclease 2	13
Acrdi.XM_015906667.1/1-1539	Monca.augustus.g15322.t1/1-1527	0.168416	0.151483	galactose-3-O-sulfotransferase 2-like	4
Acrdi.XM_015913160.1/1-372	Monca.adi2mcaRNA23547_R1/1-321	0.20547	0.151477	PREDICTED: uncharacterized protein C3orf14-like	
Acrdi.XM_015895583.1/1-1503	Monca.augustus.g35449.t1/1-1647	0.22514	0.151459	nuclear MDM1-like isoform X3	2
Acrdi.XM_015896125.1/1-1578	Monca.augustus.g38249.t1/1-3276	0.166036	0.151447	AT-rich interactive domain-containing 4B-like isoform X1	
Acrdi.XM_015896632.1/1-2928	Monca.adi2mcaRNA5502_R0/1-2598	0.145995	0.151409	timeless homolog	7
Acrdi.XM_015905512.1/1-1032	Monca.augustus.g28264.t1/1-1038	0.215812	0.151408	G- coupled receptor 161	4
Acrdi.XM_015906796.1/1-573	Monca.augustus.g5566.t1/1-486	0.186882	0.151403	PREDICTED: uncharacterized protein LOC107341398	
Acrdi.XM_015903245.1/1-1335	Monca.augustus.g48456.t1/1-1368	0.234559	0.15139	keratin-associated 10-6-like	1
Acrdi.XM_015902992.1/1-357	Monca.augustus.g593.t1/1-342	0.16551	0.151385	heat shock factor 2-binding	
Acrdi.XM_015903611.1/1-1806	Monca.augustus.g24956.t1/1-1743	0.168464	0.151378	major facilitator superfamily domain-containing 6 isoform X2	4
Acrdi.XM_015906785.1/1-1026	Monca.augustus.g4905.t1/1-1038	0.178683	0.151377	N-acyl-phosphatidylethanolamine-hydrolyzing phospholipase D-like	
Acrdi.XM_015892307.1/1-4065	Monca.adi2mcaRNA3939_R0/1-4044	0.160736	0.151363	deleted in malignant brain tumors 1 -like	3
Acrdi.XM_015903586.1/1-369	Monca.augustus.g48212.t1/1-366	0.281421	0.151355	coiled-coil domain-containing 86-like	2
Acrdi.XM_015908781.1/1-1194	Monca.adi2mcaRNA27138_R6/1-2838	0.0570443	0.151338	PREDICTED: uncharacterized protein LOC107343213	
Acrdi.XM_015916052.1/1-1248	Monca.augustus.g64750.t1/1-1215	0.159445	0.151336	failed axon connections homolog	2
Acrdi.XM_015919870.1/1-1494	Monca.augustus.g33614.t1/1-3957	0.218225	0.151306	SCAF8 isoform X1	2
Acrdi.XM_015913656.1/1-1236	Monca.augustus.g30471.t1/1-1887	0.155269	0.151268	glycoside hydrolase family 9	2
Acrdi.XM_015921202.1/1-1191	Monca.adi2mcaRNA32544_R0/1-1596	0.154099	0.151265	ADP-ribosyl-[dinitrogen reductase] glycohydrolase-like	
Acrdi.XM_015918243.1/1-1953	Monca.augustus.g48581.t1/1-1992	0.21259	0.151237	PEP-CTERM domain	1
Acrdi.XM_015900479.1/1-1002	Monca.augustus.g1377.t1/1-1050	0.112269	0.151232	non-homologous end-joining factor 1-like	5
Acrdi.XM_015891715.1/1-1806	Monca.augustus.g52052.t1/1-2142	0.194898	0.151225	Fibrinogen C domain-containing 1	
Acrdi.XM_015919446.1/1-480	Monca.adi2mcaRNA30611_R3/1-327	0.19003	0.151213	PREDICTED: uncharacterized protein LOC107353127	
Acrdi.XM_015914033.1/1-1209	Monca.augustus.g44890.t1/1-1209	0.185013	0.151202	decaprenyl-diphosphate synthase subunit 2-like	2
Acrdi.XM_015908241.1/1-1476	Monca.augustus.g7880.t1/1-1464	0.186025	0.151196	E3 ubiquitin- ligase TRIM71-like	
Acrdi.XM_015895064.1/1-783	Monca.adi2mcaRNA41444_R0/1-771	0.109422	0.151188	Calmodulin	1
Acrdi.XM_015906441.1/1-1926	Monca.augustus.g51446.t1/1-1929	0.159365	0.151184	Conserved oligomeric Golgi complex subunit 8	2
Acrdi.XM_015897256.1/1-1860	Monca.adi2mcaRNA6174_R5/1-1785	0.175519	0.15106	ATP-binding cassette sub-family G member 2	3
Acrdi.XM_015908449.1/1-1590	Monca.augustus.g33946.t1/1-1602	0.200768	0.151044	synaptotagmin-4-like isoform X1	14
Acrdi.XM_015904152.1/1-2262	Monca.augustus.g8497.t1/1-2259	0.153324	0.151022	N-acetylated-alpha-linked acidic dipeptidase	2
Acrdi.XM_015893768.1/1-900	Monca.augustus.g32522.t1/1-1305	0.181314	0.151009	Histone deacetylase 6	1
Acrdi.XM_015891948.1/1-381	Monca.augustus.g33208.t1/1-372	0.0966059	0.150986	60S ribosomal L22	5
Acrdi.XM_015923341.1/1-762	Monca.augustus.g85.t1/1-1311	0.162478	0.150984	tumor p53-inducible 13	2
Acrdi.XM_015903879.1/1-1308	Monca.augustus.g48098.t1/1-1305	0.150509	0.150977	beta-1 adrenergic receptor	4
Acrdi.XM_015910670.1/1-1182	Monca.augustus.g47631.t1/1-1143	0.191952	0.150932	Collagen alpha-1(XII) chain	6
Acrdi.XM_015900535.1/1-1440	Monca.augustus.g37058.t1/1-1455	0.143467	0.150924	neuropeptide Y receptor-like	3
Acrdi.XM_015912627.1/1-1689	Monca.augustus.g53951.t1/1-2778	0.213015	0.150919	PREDICTED: uncharacterized protein C1orf112 homolog	1
Acrdi.XM_015908643.1/1-1263	Monca.augustus.g53879.t1/1-1263	0.14696	0.150912	carbohydrate sulfotransferase 4-like isoform X1	5
Acrdi.XM_015898193.1/1-978	Monca.augustus.g39418.t1/1-1101	0.225695	0.150885	2-acylglycerol O-acyltransferase 2	5
Acrdi.XM_015913179.1/1-1071	Monca.adi2mcaRNA23543_R0/1-996	0.226108	0.150877	methionyl-tRNA mitochondrial-like	6
Acrdi.XM_015902062.1/1-444	Monca.augustus.g19906.t1/1-1101	0.28377	0.150868	myb X	
Acrdi.XM_015903882.1/1-528	Monca.augustus.g48101.t1/1-3768	0.148876	0.150826	PREDICTED: uncharacterized protein LOC107338647	
Acrdi.XM_015910057.1/1-1095	Monca.adi2mcaRNA20178_R0/1-951	0.193788	0.150824	deoxyribonuclease TATDN3	2
Acrdi.XM_015896225.1/1-1830	Monca.augustus.g14711.t1/1-1695	0.190328	0.150806	ATM interactor-like	2
Acrdi.XM_015921686.1/1-711	Monca.adi2mcaRNA3304_R0/1-804	0.545414	0.150782	PREDICTED: uncharacterized protein LOC107355161	
Acrdi.XM_015895470.1/1-663	Monca.augustus.g6370.t1/1-720	0.1359	0.150754	saposin-like type region 1	1
Acrdi.XM_015910355.1/1-1920	Monca.augustus.g36441.t1/1-1929	0.185445	0.150748	Long-chain fatty acid transport 4	4
Acrdi.XM_015916059.1/1-432	Monca.augustus.g67869.t1/1-402	0.156578	0.150748	60S ribosomal L26	4
Acrdi.XM_015903713.1/1-2364	Monca.augustus.g41351.t1/1-3822	0.158848	0.150738	msx2-interacting -like	3
Acrdi.XM_015902397.1/1-3954	Monca.augustus.g37555.t1/1-4680	0.158003	0.150728	DNA repair RAD50	5
Acrdi.XM_015925170.1/1-984	Monca.augustus.g51643.t1/1-957	0.118088	0.150712	BTB POZ domain-containing 6-like	
Acrdi.XM_015899373.1/1-1029	Monca.augustus.g44787.t1/1-1080	0.110045	0.150692	trace amine-associated receptor 9	7
Acrdi.XM_015919266.1/1-2370	Monca.augustus.g60219.t1/1-2355	0.144666	0.150686	amiloride-sensitive amine oxidase [copper-containing]-like	1
Acrdi.XM_015899440.1/1-552	Monca.augustus.g46732.t1/1-483	0.226692	0.150677	PREDICTED: uncharacterized protein LOC107334505 isoform X1	1
Acrdi.XM_015918189.1/1-558	Monca.augustus.g20698.t1/1-546	0.14667	0.150635	motor neuron and pancreas homeobox 1-like	6
Acrdi.XM_015901124.1/1-3489	Monca.adi2mcaRNA10451_R0/1-3378	0.198223	0.15056	BRCA2-interacting transcriptional repressor EMSY isoform X1	3
Acrdi.XM_015893010.1/1-1311	Monca.augustus.g23868.t1/1-1857	0.178117	0.150556	glycine receptor subunit alpha-3 isoform X1	9
Acrdi.XM_015908894.1/1-645	Monca.augustus.g29216.t1/1-1416	0.16359	0.150551	vacuolar 8-like	1
Acrdi.XM_015919829.1/1-2490	Monca.adi2mcaRNA31065_R0/1-2349	0.147453	0.150542	von Willebrand factor D and EGF domain-containing -like	1
Acrdi.XM_015899388.1/1-858	Monca.augustus.g46756.t1/1-771	0.133569	0.150542	rhamnose-binding lectin-like	1
Acrdi.XM_015904938.1/1-1107	Monca.augustus.g46469.t1/1-2910	0.0682196	0.150501	GRIP1-associated 1-like	
Acrdi.XM_015912848.1/1-13218	Monca.augustus.g11206.t1/1-13974	0.16377	0.150472	protocadherin Fat 1 isoform X1	1
Acrdi.XM_015915037.1/1-1059	Monca.augustus.g63347.t1/1-1074	0.133995	0.150465	neuropeptide FF receptor 2-like	4
Acrdi.XM_015900757.1/1-1749	Monca.augustus.g57305.t1/1-2838	0.160437	0.150448	ankyrin repeat domain-containing 24-like	
Acrdi.XM_015908835.1/1-762	Monca.adi2mcaRNA18845_R0/1-717	0.191829	0.150444	glutaminase kidney mitochondrial-like	
Acrdi.XM_015910625.1/1-663	Monca.augustus.g58090.t1/1-996	0.171122	0.150444	universal stress Slr1101-like	1
Acrdi.XM_015904600.1/1-693	Monca.augustus.g25488.t1/1-675	0.183178	0.150441	PREDICTED: uncharacterized protein LOC107339330	2
Acrdi.XM_015924443.1/1-2154	Monca.adi2mcaRNA36234_R1/1-1638	0.376082	0.15043	Retrovirus-related Pol poly from transposon	
Acrdi.XM_015907932.1/1-801	Monca.augustus.g53181.t1/1-1014	0.144312	0.150426	glycogenin-1-like	1
Acrdi.XM_015893212.1/1-810	Monca.augustus.g32204.t1/1-813	0.180231	0.150424	SAM-dependent methyltransferase	2
Acrdi.XM_015893386.1/1-774	Monca.augustus.g68179.t1/1-2205	0.204188	0.150406	SWI SNF-related matrix-associated actin-dependent regulator of chror	10
Acrdi.XM_015919559.1/1-675	Monca.augustus.g29630.t1/1-684	0.13592	0.150345	membrane-associated progesterone receptor component 2	3
Acrdi.XM_015919954.1/1-732	Monca.augustus.g67045.t1/1-735	0.223858	0.15032	PREDICTED: uncharacterized protein LOC107353619	
Acrdi.XM_015892210.1/1-1191	Monca.augustus.g13464.t1/1-1242	0.118741	0.150316	hydroxysteroid dehydrogenase 2	1
Acrdi.XM_015899953.1/1-1386	Monca.augustus.g10903.t1/1-1386	0.154495	0.150256	thymidine phosphorylase-like	4
Acrdi.XM_015922265.1/1-900	Monca.adi2mcaRNA33703_R1/1-900	0.225431	0.150251	forkhead box Q1	4
Acrdi.XM_015914498.1/1-717	Monca.augustus.g27252.t1/1-879	0.151863	0.150205	PIH1 domain-containing 1 isoform X1	2
Acrdi.XM_015917530.1/1-2442	Monca.adi2mcaRNA28487_R0/1-2361	0.162099	0.150181	Glutamine-rich 2	
Acrdi.XM_015922671.1/1-477	Monca.augustus.g1936.t1/1-486	0.162165	0.15018	40S ribosomal S24 isoform X1	5
Acrdi.XM_015920394.1/1-1038	Monca.augustus.g46009.t1/1-1290	0.199721	0.150155	EGF-like repeat and discoidin I-like domain-containing 3	7
Acrdi.XM_015922837.1/1-1017	Monca.augustus.g1404.t1/1-1026	0.181382	0.150154	Nucleoporin NUP53	8
Acrdi.XM_015892084.1/1-1920	Monca.augustus.g36061.t1/1-5061	0.170026	0.150136	PREDICTED: uncharacterized protein LOC107327340	2
Acrdi.XM_015919233.1/1-681	Monca.augustus.g40933.t1/1-681	0.220678	0.150071	PREDICTED: uncharacterized protein LOC107352913	2
Acrdi.XM_015906299.1/1-405	Monca.augustus.g21045.t1/1-402	0.102058	0.150023	fibroblast growth factor 2	5
Acrdi.XM_015895577.1/1-2835	Monca.augustus.g22271.t1/1-3084	0.195402	0.15	PREDICTED: uncharacterized protein LOC107331047	2
Acrdi.XM_015893809.1/1-858	Monca.augustus.g61113.t1/1-858	0.223996	0.149985	ATP-dependent DNA helicase PIF1	
Acrdi.XM_015898759.1/1-618	Monca.augustus.g18749.t1/1-633	0.156316	0.149972	MKRN2 opposite strand -like	
Acrdi.XM_015911434.1/1-1563	Monca.augustus.g53229.t1/1-1554	0.174533	0.14995	activating signal cointegrator 1	1
Acrdi.XM_015910317.1/1-3504	Monca.augustus.g36439.t1/1-3573	0.155575	0.149939	WD repeat-containing 47	
Acrdi.XM_015899035.1/1-1500	Monca.augustus.g26226.t1/1-1503	0.175103	0.149924	Acid-sensing ion channel 4	7
Acrdi.XM_015910491.1/1-1056	Monca.augustus.g65046.t1/1-1269	0.105935	0.149901	proto-oncogene Wnt-3	
Acrdi.XM_015900771.1/1-4299	Monca.adi2mcaRNA10022_R0/1-4083	0.164954	0.149891	Complement C3	4
Acrdi.XM_015903014.1/1-2190	Monca.augustus.g47577.t1/1-2172	0.212626	0.149885	Cation channel sperm-associated subunit beta	2
Acrdi.XM_015901326.1/1-1647	Monca.augustus.g48490.t1/1-1644	0.101934	0.14988	Tripartite motif containing 13	3
Acrdi.XM_015900824.1/1-474	Monca.augustus.g21076.t1/1-303	0.0733023	0.149864	chondroitin proteoglycan-2-like	3
Acrdi.XM_015921133.1/1-429	Monca.augustus.g7909.t1/1-765	0.164132	0.149793	N-terminal Xaa-Pro-Lys N-methyltransferase 1	4
Acrdi.XM_015899857.1/1-1104	Monca.augustus.g51434.t1/1-1110	0.161394	0.149793	histamine H2 receptor-like	3
Acrdi.XM_015892683.1/1-2694	Monca.adi2mcaRNA3874_R0/1-2733	0.178965	0.149777	Extracellular calcium-sensing receptor	4
Acrdi.XM_015905007.1/1-636	Monca.adi2mcaRNA14584_R0/1-525	0.617241	0.149775	PREDICTED: uncharacterized protein LOC107339704	
Acrdi.XM_015918464.1/1-738	Monca.adi2mcaRNA29491_R0/1-768	0.259757	0.149767	probable G- coupled receptor 133	9
Acrdi.XM_015918305.1/1-363	Monca.augustus.g29411.t1/1-696	0.130568	0.149752	PREDICTED: uncharacterized protein LOC107352003	2
Acrdi.XM_015911934.1/1-561	Monca.augustus.g24238.t1/1-1743	0.140994	0.149751	leucine-rich repeat soc-2 homolog	
Acrdi.XM_015922478.1/1-3066	Monca.adi2mcaRNA33962_R0/1-2802	0.603445	0.14974	RNA-directed DNA polymerase from mobile element jockey-like	
Acrdi.XM_015903075.1/1-453	Monca.augustus.g48859.t1/1-735	0.0389506	0.149726	prostatic spermine-binding -	
Acrdi.XM_015903462.1/1-1023	Monca.augustus.g22470.t1/1-1080	0.196137	0.149686	Activating signal cointegrator 1 complex subunit 1	1
Acrdi.XM_015907771.1/1-1104	Monca.augustus.g4921.t1/1-1293	0.179902	0.14967	gamma-aminobutyric acid receptor subunit alpha-5-like isoform X2	6
Acrdi.XM_015923701.1/1-837	Monca.augustus.g34963.t1/1-831	0.133413	0.149647	integrin alpha-D-like isoform X2	
Acrdi.XM_015917644.1/1-483	Monca.augustus.g44133.t1/1-480	0.142865	0.149642	ketohexokinase isoform X2	12
Acrdi.XM_015916412.1/1-1827	Monca.augustus.g64481.t1/1-3114	0.141855	0.149626	disulfide-isomerase A5	3
Acrdi.XM_015898746.1/1-2958	Monca.augustus.g54234.t1/1-3165	0.180047	0.149592	zinc finger X-linked ZXDB-like	2
Acrdi.XM_015914289.1/1-3321	Monca.augustus.g40759.t1/1-3351	0.209159	0.149587	GPI ethanolamine phosphate transferase 3-like	1
Acrdi.XM_015922500.1/1-1413	Monca.augustus.g33877.t1/1-1308	0.152823	0.149561	actin-binding LIM 2 isoform X30	2
Acrdi.XM_015913778.1/1-870	Monca.augustus.g21664.t1/1-873	0.140045	0.149555	3-mercaptopyruvate sulfurtransferase	3
Acrdi.XM_015905570.1/1-804	Monca.adi2mcaRNA15209_R4/1-753	0.449315	0.149553	RNA-directed DNA polymerase from mobile element jockey	
Acrdi.XM_015905375.1/1-1524	Monca.adi2mcaRNA1560_R1/1-2325	0.191837	0.149552	RNA-directed DNA polymerase from transposon BS	
Acrdi.XM_015903627.1/1-1170	Monca.augustus.g52894.t1/1-1320	0.155926	0.149525	allantoicase-like	2
Acrdi.XM_015922814.1/1-2463	Monca.augustus.g366.t1/1-2472	0.147593	0.14952	potassium voltage-gated channel subfamily C member 1-like	5
Acrdi.XM_015905723.1/1-3543	Monca.augustus.g53314.t1/1-6315	0.173361	0.149492	ankyrin repeat and KH domain-containing 1 isoform X9	
Acrdi.XM_015916364.1/1-861	Monca.augustus.g52622.t1/1-963	0.139521	0.149491	IMPACT isoform X1	1
Acrdi.XM_015919607.1/1-447	Monca.augustus.g48682.t1/1-612	0.607829	0.149489	PREDICTED: uncharacterized protein LOC107353277	
Acrdi.XM_015913479.1/1-981	Monca.augustus.g20509.t1/1-1308	0.185568	0.149388	RUN and FYVE domain-containing 4	
Acrdi.XM_015919814.1/1-483	Monca.augustus.g40317.t1/1-483	0.118097	0.149361	HIG1 domain family member mitochondrial	1
Acrdi.XM_015924522.1/1-723	Monca.augustus.g40899.t1/1-1506	0.134247	0.149359	Ran-binding 3	
Acrdi.XM_015907505.1/1-1077	Monca.augustus.g55650.t1/1-1068	0.180103	0.149329	phosphatase 1 regulatory subunit 27	
Acrdi.XM_015901436.1/1-2706	Monca.augustus.g6346.t1/1-2676	0.149453	0.149316	Kinesin KIFC3	4
Acrdi.XM_015911851.1/1-4944	Monca.augustus.g43173.t1/1-5637	0.200407	0.149296	transmembrane 131-like	1
Acrdi.XM_015893587.1/1-1200	Monca.augustus.g41448.t1/1-1185	0.159909	0.149267	tyramine octopamine receptor-like	5
Acrdi.XM_015907494.1/1-1068	Monca.augustus.g23760.t1/1-1056	0.199155	0.149265	stAR-related lipid transfer mitochondrial	1
Acrdi.XM_015906622.1/1-456	Monca.augustus.g70207.t1/1-687	0.1797	0.149238	CDP-diacylglycerol--inositol 3-phosphatidyltransferase	8
Acrdi.XM_015896650.1/1-1119	Monca.augustus.g42405.t1/1-1281	0.190214	0.14922	growth factor receptor-bound 14	
Acrdi.XM_015900937.1/1-1341	Monca.augustus.g27903.t1/1-1560	0.148274	0.149183	pre-mRNA-splicing factor	6
Acrdi.XM_015908333.1/1-2154	Monca.augustus.g35185.t1/1-2136	0.162302	0.149181	PREDICTED: uncharacterized protein C12orf56-like	
Acrdi.XM_015917753.1/1-2136	Monca.adi2mcaRNA2908_R0/1-1938	0.181245	0.149177	Paired box Pax-3	6
Acrdi.XM_015913432.1/1-651	Monca.augustus.g32113.t1/1-642	0.182712	0.149105	dnaJ homolog subfamily C member 4	1
Acrdi.XM_015917810.1/1-1677	Monca.adi2mcaRNA28781_R0/1-1788	0.185953	0.149068	rabphilin-3A isoform X1	12
Acrdi.XM_015919113.1/1-7752	Monca.adi2mcaRNA30276_R0/1-7761	0.213969	0.149067	A disintegrin and metallo ase with thrombospondin motifs 20	6
Acrdi.XM_015901047.1/1-960	Monca.augustus.g57605.t1/1-1320	0.224298	0.149054	abl interactor 1 isoform X9	11
Acrdi.XM_015903660.1/1-606	Monca.augustus.g32280.t1/1-642	0.127501	0.149006	Thymidylate kinase	10
Acrdi.XM_015908146.1/1-1803	Monca.adi2mcaRNA18011_R6/1-867	0.529034	0.148954	PREDICTED: uncharacterized protein LOC107342644, partial	
Acrdi.XM_015911350.1/1-1185	Monca.augustus.g35289.t1/1-1182	0.150935	0.148941	DNA-(apurinic or apyrimidinic site) lyase-like isoform X5	7
Acrdi.XM_015917493.1/1-537	Monca.augustus.g1825.t1/1-1188	0.193037	0.148936	G- coupled receptor 143-like	4
Acrdi.XM_015906930.1/1-690	Monca.augustus.g33393.t1/1-1071	0.271625	0.14892	probable palmitoyltransferase ZDHHC16 isoform X1	5
Acrdi.XM_015917915.1/1-708	Monca.augustus.g62260.t1/1-1056	0.169434	0.14889	TBC1 domain family member 10B	7
Acrdi.XM_015899395.1/1-9696	Monca.augustus.g48280.t1/1-10530	0.203315	0.148871	Laminin subunit alpha	1
Acrdi.XM_015895389.1/1-2526	Monca.augustus.g25995.t1/1-6672	0.145367	0.148863	myosin heavy striated muscle-like	
Acrdi.XM_015898991.1/1-1251	Monca.adi2mcaRNA8045_R9/1-1245	0.0333639	0.148834	Transposable element P transposase	
Acrdi.XM_015924011.1/1-792	Monca.augustus.g51895.t1/1-516	0.754513	0.148813	nuclease HARBI1	
Acrdi.XM_015923230.1/1-1077	Monca.adi2mcaRNA34864_R0/1-993	0.2347	0.148812	histone deacetylase 8 isoform X2	2
Acrdi.XM_015906068.1/1-993	Monca.adi2mcaRNA15724_R1/1-1002	0.17566	0.148757	solute carrier family 35 member E1	2
Acrdi.XM_015901400.1/1-687	Monca.adi2mcaRNA10655_R0/1-492	0.270111	0.148727	fibroblast growth factor 18-like	2
Acrdi.XM_015900148.1/1-666	Monca.augustus.g62841.t1/1-1032	0.135141	0.148671	mitochondrial ribosome-associated GTPase 1	4
Acrdi.XM_015904044.1/1-2796	Monca.adi2mcaRNA13512_R0/1-2493	0.127025	0.148663	zinc metallo ase nas-13-like	2
Acrdi.XM_015899933.1/1-714	Monca.augustus.g32788.t1/1-2337	0.197114	0.148606	two pore calcium channel	
Acrdi.XM_015914642.1/1-828	Monca.adi2mcaRNA25229_R6/1-795	0.463398	0.148593	Carboxypeptidase D	
Acrdi.XM_015900834.1/1-795	Monca.augustus.g42332.t1/1-792	0.236242	0.148573	proline and serine-rich 1 isoform X1	
Acrdi.XM_015915011.1/1-3363	Monca.augustus.g66539.t1/1-2730	0.144311	0.14857	Squamous cell carcinoma antigen recognized by T-cells 3	2
Acrdi.XM_015893854.1/1-1845	Monca.adi2mcaRNA39999_R0/1-1680	0.181622	0.148512	PREDICTED: uncharacterized protein LOC107329124	
Acrdi.XM_015900546.1/1-912	Monca.augustus.g26474.t1/1-873	0.128361	0.148512	calmodulin-lysine N-methyltransferase	7
Acrdi.XM_015899803.1/1-1551	Monca.adi2mcaRNA8940_R3/1-1020	0.503254	0.148486	PREDICTED: uncharacterized protein LOC107334842	2
Acrdi.XM_015907028.1/1-1959	Monca.adi2mcaRNA16886_R0/1-1041	0.193402	0.148477	serine threonine- phosphatase 6 regulatory ankyrin repeat subunit B-like	
Acrdi.XM_015919773.1/1-1266	Monca.augustus.g48083.t1/1-1695	0.132278	0.148468	tubulin delta chain-like	1
Acrdi.XM_015922509.1/1-1077	Monca.augustus.g33867.t1/1-1080	0.158109	0.148448	dopamine receptor 2-like	3
Acrdi.XM_015922088.1/1-1854	Monca.augustus.g24782.t1/1-1866	0.202233	0.14843	Formin-2	
Acrdi.XM_015923315.1/1-408	Monca.adi2mcaRNA34967_R6/1-297	0.497869	0.148414	PREDICTED: uncharacterized protein LOC107356692	
Acrdi.XM_015906118.1/1-300	Monca.augustus.g10606.t1/1-684	0.123868	0.148414	brain-specific angiogenesis inhibitor 1-like	
Acrdi.XM_015901941.1/1-630	Monca.augustus.g4669.t1/1-600	0.104637	0.148403	PREDICTED: uncharacterized protein C19orf52-like	
Acrdi.XM_015912014.1/1-933	Monca.augustus.g35308.t1/1-924	0.166785	0.148386	melanocyte-stimulating hormone receptor-like	6
Acrdi.XM_015913148.1/1-2277	Monca.augustus.g39275.t1/1-2757	0.164167	0.148298	Aminopeptidase N	6
Acrdi.XM_015899210.1/1-1446	Monca.augustus.g62004.t1/1-1476	0.1234	0.148298	disulfide-isomerase A3	2
Acrdi.XM_015910378.1/1-5985	Monca.augustus.g15614.t1/1-5607	0.150559	0.148274	voltage-dependent T-type calcium channel subunit alpha-1H	5
Acrdi.XM_015909292.1/1-792	Monca.augustus.g35324.t1/1-2259	0.181355	0.148263	zer-1 homolog	4
Acrdi.XM_015921867.1/1-1056	Monca.augustus.g63512.t1/1-1056	0.19216	0.148251	PREDICTED: uncharacterized protein LOC107355323	
Acrdi.XM_015899858.1/1-1701	Monca.augustus.g51422.t1/1-1695	0.146659	0.148242	coiled-coil domain-containing 146-like	
Acrdi.XM_015898288.1/1-693	Monca.augustus.g64625.t1/1-639	0.199711	0.148179	sentrin-specific protease 8	1
Acrdi.XM_015920434.1/1-621	Monca.augustus.g23825.t1/1-603	0.157196	0.148132	splicing regulator RBM11	
Acrdi.XM_015900040.1/1-570	Monca.augustus.g6672.t1/1-546	0.14865	0.14812	Eukaryotic translation elongation factor 1 epsilon-1	5
Acrdi.XM_015920000.1/1-900	Monca.augustus.g49594.t1/1-1635	0.116212	0.148066	28S ribosomal mitochondrial	4
Acrdi.XM_015899487.1/1-378	Monca.augustus.g49635.t1/1-807	0.131313	0.148065	-tyrosine sulfotransferase 2	5
Acrdi.XM_015896931.1/1-420	Monca.augustus.g28903.t1/1-492	0.124984	0.148061	chromatin modification-related MEAF6 isoform X2	2
Acrdi.XM_015898319.1/1-1287	Monca.adi2mcaRNA7331_R0/1-1179	0.215262	0.148029	Transcription elongation mitochondrial	1
Acrdi.XM_015906574.1/1-999	Monca.augustus.g21900.t1/1-1134	0.222989	0.148023	Serine protease 23	4
Acrdi.XM_015898403.1/1-1437	Monca.augustus.g58530.t1/1-1371	0.162443	0.148019	UPF0759 -like isoform X2	
Acrdi.XM_015910553.1/1-1287	Monca.augustus.g70623.t1/1-2055	0.172674	0.147993	ATP-binding cassette sub-family F member 1	1
Acrdi.XM_015914097.1/1-843	Monca.augustus.g8022.t1/1-1689	0.185929	0.147971	EF-hand domain-containing family member B	1
Acrdi.XM_015920752.1/1-966	Monca.augustus.g35922.t1/1-951	0.171547	0.14796	melatonin receptor type 1A-like	4
Acrdi.XM_015904308.1/1-855	Monca.augustus.g5863.t1/1-2079	0.155494	0.14795	tRNA wybutosine-synthesizing 5 isoform X2	2
Acrdi.XM_015901610.1/1-7464	Monca.adi2mcaRNA11_R1/1-6543	0.158338	0.147887	EGF-like repeat and discoidin I-like domain-containing 3	1
Acrdi.XM_015911852.1/1-1461	Monca.augustus.g43172.t1/1-1110	0.162926	0.147878	palmitoyltransferase ZDHHC2 isoform X1	3
Acrdi.XM_015902088.1/1-1338	Monca.augustus.g46391.t1/1-1746	0.0905592	0.147865	ras-GEF domain-containing family member 1B isoform X2	3
Acrdi.XM_015919809.1/1-654	Monca.augustus.g41940.t1/1-885	0.173783	0.14786	SH3 and PX domain-containing 2A isoform X2	
Acrdi.XM_015917563.1/1-567	Monca.augustus.g30303.t1/1-621	0.13804	0.147858	outer membrane lipo Blc-like	
Acrdi.XM_015902265.1/1-1836	Monca.augustus.g71156.t1/1-1824	0.222819	0.147852	cationic amino acid transporter 4	3
Acrdi.XM_015924126.1/1-792	Monca.augustus.g55602.t1/1-792	0.12303	0.147799	sepiapterin reductase	3
Acrdi.XM_015924124.1/1-1356	Monca.augustus.g66957.t1/1-1344	0.111049	0.147679	Bone morphogenetic 7	43
Acrdi.XM_015906077.1/1-1416	Monca.augustus.g34443.t1/1-1221	0.192557	0.147673	sidekick-1-like isoform X1	2
Acrdi.XM_015909039.1/1-2004	Monca.augustus.g38356.t1/1-2313	0.144674	0.147668	#NAME?	3
Acrdi.XM_015898205.1/1-1791	Monca.augustus.g32107.t1/1-1764	0.131271	0.147653	alpha-(1,6)-fucosyltransferase isoform X1	4
Acrdi.XM_015896994.1/1-1635	Monca.augustus.g28884.t1/1-1638	0.143163	0.147593	alkaline phosphatase	1
Acrdi.XM_015920011.1/1-990	Monca.augustus.g4866.t1/1-1053	0.150912	0.147589	PREDICTED: uncharacterized protein LOC107353665	
Acrdi.XM_015898769.1/1-777	Monca.augustus.g55625.t1/1-3852	0.138298	0.14756	PERQ amino acid-rich with GYF domain-containing 2-like	
Acrdi.XM_015900729.1/1-1218	Monca.augustus.g34872.t1/1-1227	0.146117	0.147548	Beta-1,3-galactosyltransferase 6	3
Acrdi.XM_015911693.1/1-531	Monca.adi2mcaRNA21963_R0/1-534	0.253028	0.147536	Tether containing UBX domain for GLUT4	
Acrdi.XM_015893765.1/1-1512	Monca.augustus.g20225.t1/1-1524	0.146391	0.147525	EGF domain-specific O-linked N-acetylglucosamine transferase	2
Acrdi.XM_015896169.1/1-5262	Monca.adi2mcaRNA5019_R0/1-5331	0.216372	0.147488	rotatin-like	3
Acrdi.XM_015915946.1/1-5472	Monca.adi2mcaRNA26694_R0/1-5346	0.193525	0.14747	Serine threonine- kinase tousled-like	3
Acrdi.XM_015922173.1/1-642	Monca.adi2mcaRNA33597_R1/1-603	0.876559	0.14746	PREDICTED: uncharacterized protein LOC107355587	
Acrdi.XM_015903279.1/1-564	Monca.augustus.g36970.t1/1-1212	0.352073	0.14744	Transposon Ty3-I Gag-Pol poly	
Acrdi.XM_015913280.1/1-4566	Monca.augustus.g67361.t1/1-5673	0.179144	0.147431	Centrosomal of 152 kDa	1
Acrdi.XM_015911982.1/1-771	Monca.adi2mcaRNA22275_R2/1-1221	0.592514	0.147397	PREDICTED: uncharacterized protein LOC107346203	
Acrdi.XM_015898466.1/1-2895	Monca.augustus.g33977.t1/1-3024	0.213385	0.147358	SMG5	
Acrdi.XM_015900188.1/1-2871	Monca.augustus.g65826.t1/1-4221	0.201894	0.147329	amyloid beta A4 precursor -binding family A member 2 isoform X2	3
Acrdi.XM_015892734.1/1-1605	Monca.augustus.g33460.t1/1-1611	0.162725	0.147326	nucleolar complex 4 homolog	3
Acrdi.XM_015899414.1/1-915	Monca.augustus.g26785.t1/1-1011	0.125398	0.147312	Gamma-soluble NSF attachment	1
Acrdi.XM_015901255.1/1-2418	Monca.augustus.g61794.t1/1-2571	0.181762	0.147256	transmembrane and TPR repeat-containing 2-like	2
Acrdi.XM_015896619.1/1-618	Monca.augustus.g8402.t1/1-612	0.198178	0.147226	PREDICTED: uncharacterized protein LOC107331926	
Acrdi.XM_015919635.1/1-1110	Monca.augustus.g32523.t1/1-1218	0.199887	0.147223	aminoacylase-1	3
Acrdi.XM_015918064.1/1-1278	Monca.augustus.g39974.t1/1-1275	0.176239	0.147182	flavin-containing monooxygenase FMO GS-OX-like 2	2
Acrdi.XM_015911223.1/1-813	Monca.augustus.g41211.t1/1-1263	0.111022	0.147178	domain-containing 4	
Acrdi.XM_015913959.1/1-1176	Monca.augustus.g20013.t1/1-1179	0.179097	0.14712	kinase C and casein kinase substrate in neurons 1	10
Acrdi.XM_015905164.1/1-1788	Monca.augustus.g57143.t1/1-1536	0.172876	0.147112	ADP-ribosylation factor GTPase-activating 2-like	2
Acrdi.XM_015900208.1/1-393	Monca.augustus.g62851.t1/1-444	0.16057	0.147091	39S ribosomal mitochondrial	
Acrdi.XM_015910893.1/1-708	Monca.augustus.g39557.t1/1-1644	0.127602	0.147076	PREDICTED: uncharacterized protein LOC107345185 isoform X1	2
Acrdi.XM_015903641.1/1-3861	Monca.augustus.g37316.t1/1-3336	0.170322	0.147061	syntaxin-binding 5-like	
Acrdi.XM_015901414.1/1-1854	Monca.augustus.g46702.t1/1-2115	0.18295	0.147043	probable C-mannosyltransferase DPY19L3 isoform X1	1
Acrdi.XM_015912759.1/1-1548	Monca.augustus.g9224.t1/1-1593	0.138016	0.147023	proton-coupled amino acid transporter 1-like	3
Acrdi.XM_015904606.1/1-885	Monca.augustus.g31424.t1/1-1581	0.190472	0.147021	BMP-binding endothelial regulator	2
Acrdi.XM_015909547.1/1-1794	Monca.adi2mcaRNA155_R0/1-1773	0.200049	0.147014	rho-related racA-like	3
Acrdi.XM_015907557.1/1-3501	Monca.augustus.g5850.t1/1-3522	0.233561	0.147013	zinc finger 431 isoform X1	2
Acrdi.XM_015891939.1/1-546	Monca.augustus.g54130.t1/1-741	0.770232	0.147006	PREDICTED: uncharacterized protein LOC107327185	
Acrdi.XM_015917222.1/1-3591	Monca.augustus.g55547.t1/1-3561	0.191315	0.146996	transient receptor potential cation channel subfamily A member 1-like	
Acrdi.XM_015911948.1/1-603	Monca.augustus.g58634.t1/1-834	0.175627	0.146879	apoptosis regulator BAX-like	6
Acrdi.XM_015906430.1/1-1617	Monca.augustus.g52872.t1/1-1677	0.197756	0.146842	cysteine-rich motor neuron 1	9
Acrdi.XM_015905980.1/1-777	Monca.augustus.g41415.t1/1-1104	0.153876	0.146838	PREDICTED: uncharacterized protein LOC107340616	
Acrdi.XM_015903068.1/1-1749	Monca.augustus.g56753.t1/1-1755	0.148775	0.146831	PREDICTED: uncharacterized protein LOC107337834	
Acrdi.XM_015895843.1/1-852	Monca.adi2mcaRNA4628_R0/1-816	0.175963	0.146822	orexin receptor type 2	7
Acrdi.XM_015913784.1/1-1191	Monca.augustus.g57172.t1/1-2241	0.163631	0.146822	DNA mismatch repair Mlh1	6
Acrdi.XM_015896598.1/1-2085	Monca.augustus.g1588.t1/1-2079	0.269309	0.146821	centromere I	7
Acrdi.XM_015898103.1/1-2019	Monca.augustus.g50136.t1/1-2022	0.152702	0.146807	kinesin KIF17	8
Acrdi.XM_015892885.1/1-1239	Monca.augustus.g16745.t1/1-1203	0.144299	0.146806	gastric triacylglycerol lipase-like	2
Acrdi.XM_015909706.1/1-1875	Monca.augustus.g55268.t1/1-1854	0.16748	0.146772	E3 ubiquitin- ligase DZIP3	5
Acrdi.XM_015912403.1/1-549	Monca.adi2mcaRNA22730_R0/1-813	0.15442	0.146741	PREDICTED: consortin-like	2
Acrdi.XM_015901230.1/1-1944	Monca.augustus.g45967.t1/1-1632	0.149615	0.146705	zinc transporter 9	1
Acrdi.XM_015910159.1/1-1089	Monca.augustus.g4415.t1/1-1086	0.176131	0.146679	Forkhead box B1	5
Acrdi.XM_015910096.1/1-2538	Monca.augustus.g38604.t1/1-4002	0.181001	0.146658	PREDICTED: uncharacterized protein LOC107344446	
Acrdi.XM_015902888.1/1-975	Monca.augustus.g27544.t1/1-975	0.146704	0.146657	annexin A13	1
Acrdi.XM_015901106.1/1-1833	Monca.augustus.g1890.t1/1-2730	0.177837	0.146655	coiled-coil domain-containing KIAA1407 homolog	3
Acrdi.XM_015917004.1/1-606	Monca.augustus.g48285.t1/1-1623	0.169709	0.146558	ralA-binding 1 isoform X2	1
Acrdi.XM_015913562.1/1-1488	Monca.augustus.g22350.t1/1-1527	0.187635	0.146555	renin receptor isoform X1	3
Acrdi.XM_015906925.1/1-1500	Monca.adi2mcaRNA16805_R0/1-1239	0.187864	0.146553	gamma-aminobutyric acid receptor subunit beta-2-like	9
Acrdi.XM_015917069.1/1-2706	Monca.augustus.g49951.t1/1-2685	0.152076	0.146513	patched domain-containing 3-like	1
Acrdi.XM_015903617.1/1-669	Monca.augustus.g66476.t1/1-669	0.214252	0.146511	F-box LRR-repeat 2-like	
Acrdi.XM_015911187.1/1-1134	Monca.augustus.g37503.t1/1-1566	0.19743	0.146482	Diphthamide biosynthesis 2	1
Acrdi.XM_015904473.1/1-567	Monca.augustus.g16844.t1/1-1032	0.216443	0.146436	peroxisomal biogenesis factor 7	6
Acrdi.XM_015924796.1/1-966	Monca.augustus.g68158.t1/1-921	0.146817	0.146397	CDK-activating kinase assembly factor MAT1	6
Acrdi.XM_015910646.1/1-513	Monca.augustus.g19027.t1/1-492	0.144569	0.146378	PREDICTED: uncharacterized protein LOC107344963	2
Acrdi.XM_015906937.1/1-363	Monca.augustus.g11631.t1/1-675	0.100371	0.146284	Ran-specific GTPase-activating	11
Acrdi.XM_015904083.1/1-1557	Monca.augustus.g23830.t1/1-1698	0.165989	0.146272	PREDICTED: uncharacterized protein LOC107338834	2
Acrdi.XM_015915584.1/1-2835	Monca.augustus.g5423.t1/1-3261	0.183659	0.14625	G- coupled receptor GRL101	1
Acrdi.XM_015900797.1/1-1092	Monca.augustus.g58754.t1/1-1152	0.160398	0.146246	tyramine receptor Ser-2-like	2
Acrdi.XM_015916902.1/1-420	Monca.adi2mcaRNA27758_R1/1-306	0.421603	0.146239	glutamic acid-rich -like	
Acrdi.XM_015913199.1/1-2013	Monca.adi2mcaRNA23633_R0/1-1926	0.151257	0.146226	cytosolic phospholipase A2-like isoform X5	2
Acrdi.XM_015898359.1/1-1725	Monca.augustus.g1159.t1/1-1791	0.232752	0.146216	Major facilitator superfamily domain-containing	2
Acrdi.XM_015917883.1/1-744	Monca.augustus.g25184.t1/1-783	0.136702	0.146152	synaptotagmin-10 isoform X3	9
Acrdi.XM_015918689.1/1-1245	Monca.augustus.g47191.t1/1-1230	0.134888	0.14615	PREDICTED: uncharacterized protein LOC107352350	2
Acrdi.XM_015910622.1/1-3750	Monca.adi2mcaRNA20833_R0/1-3690	0.168379	0.146143	partitioning defective 3 homolog isoform X2	
Acrdi.XM_015914386.1/1-939	Monca.augustus.g37650.t1/1-2772	0.180418	0.146138	general transcription factor 3C polypeptide 1	7
Acrdi.XM_015911755.1/1-594	Monca.augustus.g61982.t1/1-603	0.159641	0.146117	PREDICTED: uncharacterized protein LOC107345990	
Acrdi.XM_015910767.1/1-1143	Monca.augustus.g65486.t1/1-1281	0.158671	0.146104	phosphorylase b kinase gamma catalytic skeletal muscle heart isoforr	3
Acrdi.XM_015910043.1/1-372	Monca.adi2mcaRNA20173_R0/1-381	0.155395	0.146103	39S ribosomal mitochondrial	4
Acrdi.XM_015918732.1/1-1266	Monca.adi2mcaRNA295_R1/1-1284	0.218796	0.146092	probable proline dehydrogenase 2	1
Acrdi.XM_015923694.1/1-423	Monca.augustus.g34971.t1/1-411	0.284511	0.14608	mediator of RNA polymerase II transcription subunit 29	4
Acrdi.XM_015913490.1/1-726	Monca.augustus.g60185.t1/1-3102	0.119129	0.146072	CMT1A duplicated region transcript 1 -like	
Acrdi.XM_015897796.1/1-657	Monca.adi2mcaRNA639_R2/1-573	0.0603561	0.146036	PREDICTED: uncharacterized protein LOC107333035	9

Acrdi.XM_015914432.1/1-1560 | Monca.augustus.g71045.t1/1-1671	0.142141	0.146029	coiled-coil domain-containing 149 isoform X1					
Acrdi.XM_015915118.1/1-1218 | Monca.adi2mcaRNA25780_R8/1-984	0.598771	0.145999	PREDICTED: uncharacterized protein LOC107349021					
Acrdi.XM_015904528.1/1-1422 | Monca.augustus.g6296.t1/1-1419	0.167388	0.145989	isoform CRA_a					
Acrdi.XM_015920914.1/1-384 | Monca.augustus.g34473.t1/1-390	0.158713	0.145966	DNA-binding inhibitor ID-2	31	P:GO:0061030; P:GO:0061031; P:GO:0090398; P:GO:0045665;	P:epithelial cell differentiation involved in mammary gland alveolus development; P:endodermal digestive tract morphogenesis; P:cellular senescence; P:negative regulation of neuron differentiation; P:regulation of glial cell differentiation; P:embryonic digestive tract morphogenesis; C:nucleoplasm; P:positive regulation of sm		
Acrdi.XM_015898290.1/1-684 | Monca.adi2mcaRNA703_R0/1-591	0.164203	0.145944	Integrator complex subunit 4					
Acrdi.XM_015906240.1/1-1038 | Monca.adi2mcaRNA15962_R0/1-1023	0.218789	0.145942	2-oxoglutarate and iron-dependent oxygenase domain-containing 2 isc	2	F:GO:0016491; F:GO:0043167	F:oxidoreductase activity; F:ion binding		
Acrdi.XM_015892030.1/1-972 | Monca.augustus.g37390.t1/1-1725	0.194488	0.145884	Sodium-coupled monocarboxylate transporter 1	7	C:GO:0005887; P:GO:0055085; P:GO:0015718; F:GO:0015293;	C:integral component of plasma membrane; P:transmembrane transport; P:monocarboxylic acid transport; F:symporter activity; P:cofactor transport; P:vitamin transport; P:amide transport		
Acrdi.XM_015905918.1/1-2949 | Monca.adi2mcaRNA15602_R0/1-2964	0.158345	0.14582	CD209 antigen E					
Acrdi.XM_015902221.1/1-1989 | Monca.adi2mcaRNA11548_R0/1-2100	0.105727	0.145805	fibropellin-1-like isoform X1	1	F:GO:0005509	F:calcium ion binding		
Acrdi.XM_015918293.1/1-4788 | Monca.augustus.g29434.t1/1-10926	0.194749	0.14576	kinetochore-associated 1	1	C:GO:0044464	C:cell part		
Acrdi.XM_015899349.1/1-1182 | Monca.augustus.g40307.t1/1-1119	0.145002	0.145733	histamine H2 receptor isoform X1	2	P:GO:0050794; P:GO:0044699	P:regulation of cellular process; P:single-organism process		
Acrdi.XM_015910272.1/1-1488 | Monca.augustus.g64207.t1/1-1557	0.193819	0.14573	zinc finger 474-like					
Acrdi.XM_015921434.1/1-975 | Monca.augustus.g61110.t1/1-1434	0.2026	0.145663	ankyrin repeat domain-containing 55-like	3	P:GO:0008150; C:GO:0005575; F:GO:0003674	P:biological_process; C:cellular_component; F:molecular_function		
Acrdi.XM_015925409.1/1-441 | Monca.augustus.g28103.t1/1-903	0.126468	0.145642	inositol polyphosphate multikinase	3	F:GO:0008440; P:GO:0016310; F:GO:0016301	F:inositol-1,4,5-trisphosphate 3-kinase activity; P:phosphorylation; F:kinase activity		
Acrdi.XM_015913122.1/1-759 | Monca.adi2mcaRNA23500_R0/1-762	0.172667	0.145616	endoplasmic reticulum-Golgi intermediate compartment 2-like	4	C:GO:0016021; C:GO:0005783; C:GO:0044422; P:GO:0006810	C:integral component of membrane; C:endoplasmic reticulum; C:organelle part; P:transport		
Acrdi.XM_015904466.1/1-1029 | Monca.augustus.g33582.t1/1-1035	0.177716	0.145571	5-hydroxytryptamine receptor 7-like	3	F:GO:0004930; P:GO:0007165; C:GO:0016020	F:G-protein coupled receptor activity; P:signal transduction; C:membrane		
Acrdi.XM_015901629.1/1-804 | Monca.adi2mcaRNA1175_R0/1-672	0.156916	0.145551	serine threonine- kinase 1 6-like	1	F:GO:0016740	F:transferase activity		
Acrdi.XM_015899740.1/1-333 | Monca.augustus.g68166.t1/1-1311	0.153394	0.14552	regulator of chromosome condensation-like					
Acrdi.XM_015913643.1/1-1068 | Monca.augustus.g48694.t1/1-1068	0.20426	0.145514	histamine H2 receptor-like	3	F:GO:0004930; P:GO:0007165; C:GO:0016021	F:G-protein coupled receptor activity; P:signal transduction; C:integral component of membrane		
Acrdi.XM_015898777.1/1-915 | Monca.augustus.g58961.t1/1-951	0.152176	0.14551	Transmembrane 177	7	C:GO:0016020; P:GO:0006508; C:GO:0016021; F:GO:0008233;	C:membrane; P:proteolysis; C:integral component of membrane; F:peptidase activity; F:metalloendopeptidase activity; F:metallopeptidase activity; F:hydrolase activity		
Acrdi.XM_015895322.1/1-1458 | Monca.augustus.g9758.t1/1-1743	0.121529	0.145498	misato homolog 1-like	1	F:GO:0003924	F:GTPase activity		
Acrdi.XM_015896971.1/1-963 | Monca.augustus.g62732.t1/1-903	0.13414	0.145496	Cell growth regulator with EF hand domain 1	1	F:GO:0005509	F:calcium ion binding		
Acrdi.XM_015923989.1/1-2916 | Monca.augustus.g54038.t1/1-6045	0.210496	0.145481	flocculation FLO11-like					
Acrdi.XM_015898054.1/1-1242 | Monca.adi2mcaRNA7057_R0/1-1302	0.230034	0.145471	tetrameric potassium-selective cyclic nucleotide gated channel	5	P:GO:0006812; F:GO:0022836; P:GO:0034220; C:GO:0016021;	P:cation transport; F:gated channel activity; P:ion transmembrane transport; C:integral component of membrane; F:potassium channel activity		
Acrdi.XM_015900487.1/1-1482 | Monca.adi2mcaRNA9723_R0/1-852	0.178664	0.145459	Integral membrane GPR155	5	P:GO:0055085; C:GO:0016020; C:GO:0016021; P:GO:0035556;	P:transmembrane transport; C:membrane; C:integral component of membrane; P:intracellular signal transduction; C:intracellular		
Acrdi.XM_015922499.1/1-810 | Monca.augustus.g60696.t1/1-5133	0.168537	0.145445	Sodium potassium calcium exchanger mitochondrial	4	P:GO:0065008; P:GO:0055085; C:GO:0044444; C:GO:0044425	P:regulation of biological quality; P:transmembrane transport; C:cytoplasmic part; C:membrane part		
Acrdi.XM_015913757.1/1-1056 | Monca.augustus.g12526.t1/1-1014	0.206205	0.14541	galanin receptor type 1-like	2	P:GO:0007165; C:GO:0016020	P:signal transduction; C:membrane		
Acrdi.XM_015920764.1/1-909 | Monca.augustus.g35955.t1/1-1395	0.146437	0.145392	transcription elongation factor B polypeptide 3-like	2	C:GO:0005634; P:GO:0006351	C:nucleus; P:transcription, DNA-templated		
Acrdi.XM_015893827.1/1-513 | Monca.augustus.g536.t1/1-531	0.20027	0.14539	tRNA-splicing endonuclease subunit Sen15	1	P:GO:0016070	P:RNA metabolic process		
Acrdi.XM_015921495.1/1-1209 | Monca.augustus.g58047.t1/1-1221	0.128633	0.145361	cerebellar degeneration-related 2-like					
Acrdi.XM_015917367.1/1-603 | Monca.augustus.g23811.t1/1-555	0.169586	0.145348	seleno S	25	F:GO:0016209; P:GO:1902236; P:GO:0006983; P:GO:0009749;	F:antioxidant activity; P:negative regulation of endoplasmic reticulum stress-induced intrinsic apoptotic signaling pathway; P:ER overload response; P:response to glucose; P:endoplasmic reticulum unfolded protein response; P:negative regulation of macrophage apoptotic process; C:Derlin-1-VIMP complex; C:very-low-density lip		
Acrdi.XM_015922253.1/1-621 | Monca.augustus.g4383.t1/1-606	0.236774	0.145342	forkhead box Q1-like	4	C:GO:0005634; F:GO:0043565; F:GO:0000981; P:GO:0006357	C:nucleus; F:sequence-specific DNA binding; F:RNA polymerase II transcription factor activity, sequence-specific DNA binding; P:regulation of transcription from RNA polymerase II promoter		
Acrdi.XM_015896587.1/1-1116 | Monca.augustus.g62600.t1/1-1122	0.198348	0.145305	allatostatin-A receptor-like	4	F:GO:0004930; P:GO:0007186; C:GO:0016021; C:GO:0005886	F:G-protein coupled receptor activity; P:G-protein coupled receptor signaling pathway; C:integral component of membrane; C:plasma membrane		
Acrdi.XM_015913652.1/1-5283 | Monca.augustus.g30490.t1/1-15246	0.143556	0.145286	coadhesin-like isoform X2					
Acrdi.XM_015899237.1/1-1074 | Monca.adi2mcaRNA951_R0/1-1131	0.137116	0.14528	60 kDa SS-A Ro ribonucleo	3	F:GO:0003723; C:GO:0030529; C:GO:0019013	F:RNA binding; C:intracellular ribonucleoprotein complex; C:viral nucleocapsid		
Acrdi.XM_015924304.1/1-549 | Monca.augustus.g16023.t1/1-711	0.529292	0.145258	PREDICTED: uncharacterized protein LOC107357664					
Acrdi.XM_015907192.1/1-660 | Monca.augustus.g28176.t1/1-831	0.194128	0.145257	exosome complex component MTR3-like	9	C:GO:0000177; C:GO:0000176; P:GO:0071051; P:GO:0034475;	C:cytoplasmic exosome (RNase complex); C:nuclear exosome (RNase complex); P:polyadenylation-dependent snoRNA 3'-end processing; P:U4 snRNA 3'-end processing; P:rRNA catabolic process; P:nuclear mRNA surveillance; C:nucleolus; P:nuclear-transcribed mRNA catabolic process, exonucleolytic, 3'-5'; P:rRNA 3'-end proces		
Acrdi.XM_015894460.1/1-1230 | Monca.augustus.g64052.t1/1-1239	0.164428	0.145256	Transcription factor Ovo-like 2	6	F:GO:0003676; C:GO:0005634; F:GO:0046872; P:GO:0006366;	F:nucleic acid binding; C:nucleus; F:metal ion binding; P:transcription from RNA polymerase II promoter; F:RNA polymerase II transcription factor activity, sequence-specific DNA binding; P:regulation of transcription from RNA polymerase II promoter		
Acrdi.XM_015913984.1/1-1842 | Monca.augustus.g59714.t1/1-2358	0.122939	0.145211	ribonuclease					
Acrdi.XM_015900056.1/1-1764 | Monca.augustus.g31990.t1/1-2607	0.151451	0.145195	basement membrane-specific heparan sulfate proteoglycan core -like	2	C:GO:0016020; C:GO:0016021	C:membrane; C:integral component of membrane		
Acrdi.XM_015912804.1/1-624 | Monca.augustus.g3592.t1/1-675	0.130454	0.145191	Poly [ADP-ribose] polymerase 3					
Acrdi.XM_015916174.1/1-471 | Monca.augustus.g35041.t1/1-471	0.136751	0.14512	iron-sulfur cluster assembly 2 mitochondrial	3	F:GO:0005198; F:GO:0051536; P:GO:0016226	F:structural molecule activity; F:iron-sulfur cluster binding; P:iron-sulfur cluster assembly		
Acrdi.XM_015899782.1/1-678 | Monca.adi2mcaRNA8903_R0/1-651	0.304002	0.145091	Tetratricopeptide repeat 28					
Acrdi.XM_015919115.1/1-2751 | Monca.augustus.g35178.t1/1-3639	0.17953	0.145075	PREDICTED: uncharacterized protein LOC107352803					
Acrdi.XM_015897674.1/1-912 | Monca.augustus.g46335.t1/1-4119	0.124934	0.145069	collagen alpha-1(XXIV) chain					
Acrdi.XM_015905902.1/1-474 | Monca.augustus.g69499.t1/1-600	0.119709	0.145065	prostaglandin E synthase 3					
Acrdi.XM_015912464.1/1-483 | Monca.augustus.g11246.t1/1-483	0.224236	0.145049	PREDICTED: uncharacterized protein LOC107346667	2	C:GO:0016020; C:GO:0016021	C:membrane; C:integral component of membrane		
Acrdi.XM_015916478.1/1-792 | Monca.augustus.g6243.t1/1-780	0.149461	0.145028	zinc finger SNAI2	2	P:GO:0014036; P:GO:0001755	P:neural crest cell fate specification; P:neural crest cell migration		
Acrdi.XM_015909811.1/1-888 | Monca.augustus.g47070.t1/1-2022	0.115712	0.145012	transcriptional activator Myb isoform X1	3	F:GO:0003677; F:GO:0016491; P:GO:0055114	F:DNA binding; F:oxidoreductase activity; P:oxidation-reduction process		
Acrdi.XM_015905800.1/1-504 | Monca.augustus.g31754.t1/1-663	0.163838	0.145	nicolin-1	2	F:GO:0016301; P:GO:0009987	F:kinase activity; P:cellular process		
Acrdi.XM_015912798.1/1-759 | Monca.adi2mcaRNA23187_R0/1-807	0.138622	0.144993	phosphomannomutase A1	3	C:GO:0005737; F:GO:0004615; P:GO:0009298	C:cytoplasm; F:phosphomannomutase activity; P:GDP-mannose biosynthetic process	EC:5.4.2.8	Phosphomannomutase
Acrdi.XM_015904837.1/1-2052 | Monca.augustus.g40463.t1/1-2160	0.208886	0.144923	RNA-binding	2	F:GO:0000166; F:GO:0003676	F:nucleotide binding; F:nucleic acid binding		
Acrdi.XM_015913753.1/1-999 | Monca.augustus.g45563.t1/1-996	0.177177	0.144915	guanine nucleotide-binding subunit beta 1	2	P:GO:0035176; C:GO:0005737	P:social behavior; C:cytoplasm		
Acrdi.XM_015913170.1/1-1485 | Monca.augustus.g39265.t1/1-1491	0.215469	0.144901	tetratricopeptide repeat 23-like					
Acrdi.XM_015918180.1/1-348 | Monca.augustus.g66559.t1/1-765	0.25937	0.144868	homeobox -like	5	C:GO:0005634; F:GO:0003700; P:GO:0007275; P:GO:0006355;	C:nucleus; F:transcription factor activity, sequence-specific DNA binding; P:multicellular organism development; P:regulation of transcription, DNA-templated; F:sequence-specific DNA binding		
Acrdi.XM_015908447.1/1-1293 | Monca.augustus.g33947.t1/1-1305	0.188764	0.144862	synaptotagmin-7 isoform X2					
Acrdi.XM_015899128.1/1-1887 | Monca.augustus.g69583.t1/1-1983	0.183267	0.14485	E3 ubiquitin- ligase RFWD3	1	F:GO:0046872	F:metal ion binding		
Acrdi.XM_015904739.1/1-1929 | Monca.adi2mcaRNA14313_R4/1-1872	0.188785	0.144804	zinc finger 862-like					
Acrdi.XM_015899954.1/1-1359 | Monca.augustus.g10901.t1/1-1347	0.151082	0.144781	BTN1-like	6	C:GO:0016021; C:GO:0005773; C:GO:0031090; P:GO:0006810;	C:integral component of membrane; C:vacuole; C:organelle membrane; P:transport; P:single-organism process; P:cellular process		
Acrdi.XM_015907004.1/1-1356 | Monca.augustus.g33396.t1/1-1356	0.189783	0.14477	retinoid-inducible serine carboxypeptidase	1	P:GO:0006508	P:proteolysis		
Acrdi.XM_015909734.1/1-837 | Monca.augustus.g24128.t1/1-810	0.171766	0.144767	DNA damage-regulated autophagy modulator 1-like	3	C:GO:0016020; C:GO:0016021; C:GO:0005764	C:membrane; C:integral component of membrane; C:lysosome		
Acrdi.XM_015898320.1/1-657 | Monca.adi2mcaRNA7330_R1/1-660	0.210355	0.144764	collagen triple helix repeat-containing 1-like isoform X1					
Acrdi.XM_015900485.1/1-3339 | Monca.augustus.g37060.t1/1-15228	0.155311	0.144756	histone-lysine N-methyltransferase 2C-like isoform X1					
Acrdi.XM_015913540.1/1-2898 | Monca.augustus.g22345.t1/1-3015	0.15263	0.144739	dual serine threonine and tyrosine kinase-like	7	F:GO:0000166; F:GO:0005524; C:GO:0005829; F:GO:0004672;	F:nucleotide binding; F:ATP binding; C:cytosol; F:protein kinase activity; F:protein serine/threonine kinase activity; P:protein phosphorylation; P:intracellular signal transduction		
Acrdi.XM_015909025.1/1-1845 | Monca.augustus.g38379.t1/1-1854	0.161763	0.144687	Gamma-glutamyltranspeptidase 1	1	P:GO:0044237	P:cellular metabolic process		
Acrdi.XM_015899403.1/1-2184 | Monca.augustus.g37234.t1/1-2349	0.228651	0.144674	Sphingomyelin phosphodiesterase	3	C:GO:0016020; C:GO:0016021; F:GO:0050290	C:membrane; C:integral component of membrane; F:sphingomyelin phosphodiesterase D activity		
Acrdi.XM_015910161.1/1-486 | Monca.augustus.g14109.t1/1-486	0.189004	0.14467	PFAM domain(EF-Hand)-containing	2	P:GO:0007165; F:GO:0005509	P:signal transduction; F:calcium ion binding		
Acrdi.XM_015903456.1/1-411 | Monca.augustus.g49064.t1/1-411	0.112338	0.144659	tetratricopeptide repeat 32					
Acrdi.XM_015898960.1/1-1134 | Monca.augustus.g37998.t1/1-1131	0.172207	0.144645	bone morphogenetic 2-A-like	21	P:GO:0060485; F:GO:0008083; P:GO:0008152; P:GO:0042981;	P:mesenchyme development; F:growth factor activity; P:metabolic process; P:regulation of apoptotic process; P:positive regulation of cell differentiation; P:positive regulation of MAPK cascade; P:epithelium development; C:extracellular region; P:growth; P:animal organ morphogenesis; P:heart development; P:negative regulati		
Acrdi.XM_015896159.1/1-441 | Monca.augustus.g55044.t1/1-714	0.153753	0.144638	elongation of very long chain fatty acids 5-like	2	F:GO:0016740; P:GO:0006633	F:transferase activity; P:fatty acid biosynthetic process		
Acrdi.XM_015913684.1/1-996 | Monca.augustus.g30447.t1/1-1002	0.100928	0.144626	D(1A) dopamine receptor	8	F:GO:0004952; P:GO:0065008; P:GO:0007186; P:GO:0048522;	F:dopamine neurotransmitter receptor activity; P:regulation of biological quality; P:G-protein coupled receptor signaling pathway; P:positive regulation of cellular process; C:membrane; C:cell part; P:single-organism cellular process; P:learning		
Acrdi.XM_015899371.1/1-1071 | Monca.augustus.g44791.t1/1-1065	0.134737	0.144614	tyramine receptor tyra-2-like	3	F:GO:0004930; P:GO:0007186; C:GO:0016020	F:G-protein coupled receptor activity; P:G-protein coupled receptor signaling pathway; C:membrane		
Acrdi.XM_015899363.1/1-951 | Monca.augustus.g44797.t1/1-1017	0.175404	0.144611	transmembrane 198-B-like	1	C:GO:0016020	C:membrane		
Acrdi.XM_015898643.1/1-540 | Monca.augustus.g54246.t1/1-918	0.175499	0.1446	GPALPP motifs-containing					
Acrdi.XM_015922332.1/1-1491 | Monca.augustus.g60147.t1/1-1500	0.18505	0.144594	potassium voltage-gated channel subfamily A member 7-like	5	P:GO:0071805; C:GO:0008076; F:GO:0005251; P:GO:0034765;	P:potassium ion transmembrane transport; C:voltage-gated potassium channel complex; F:delayed rectifier potassium channel activity; P:regulation of ion transmembrane transport; P:protein homooligomerization		
Acrdi.XM_015921521.1/1-672 | Monca.augustus.g22992.t1/1-660	0.19809	0.144565	phosphatase 1 regulatory subunit 16A isoform X1					
Acrdi.XM_015922832.1/1-807 | Monca.augustus.g70821.t1/1-1134	0.173196	0.144549	Pyroglutamylated RFamide peptide receptor	3	F:GO:0008528; P:GO:0007186; C:GO:0016021	F:G-protein coupled peptide receptor activity; P:G-protein coupled receptor signaling pathway; C:integral component of membrane		
Acrdi.XM_015921534.1/1-1395 | Monca.augustus.g42445.t1/1-1488	0.120646	0.144504	heterogeneous nuclear ribonucleo A3 isoform X1	2	F:GO:0000166; F:GO:0003676	F:nucleotide binding; F:nucleic acid binding		
Acrdi.XM_015903977.1/1-4314 | Monca.augustus.g27731.t1/1-4701	0.145899	0.144475	dysferlin isoform X3	2	C:GO:0016020; C:GO:0016021	C:membrane; C:integral component of membrane		
Acrdi.XM_015905757.1/1-771 | Monca.augustus.g9359.t1/1-1020	0.192503	0.144452	PREDICTED: uncharacterized protein LOC107340407	2	F:GO:0008270; F:GO:0046872	F:zinc ion binding; F:metal ion binding		
Acrdi.XM_015923858.1/1-2913 | Monca.augustus.g21536.t1/1-2973	0.157146	0.144435	AF-10 isoform X2	1	F:GO:0008270	F:zinc ion binding		
Acrdi.XM_015897374.1/1-6051 | Monca.adi2mcaRNA6321_R0/1-5697	0.194748	0.144433	DNA ligase 1-like	7	F:GO:0000166; F:GO:0003676; P:GO:0051103; P:GO:1903461;	F:nucleotide binding; F:nucleic acid binding; P:DNA ligation involved in DNA repair; P:Okazaki fragment processing involved in mitotic DNA replication; C:nucleus; F:DNA ligas	EC:6.5.1.1	DNA ligase (ATP)
Acrdi.XM_015905724.1/1-669 | Monca.augustus.g9369.t1/1-2952	0.189693	0.144429	PREDICTED: uncharacterized protein LOC107340371	1	C:GO:0016020	C:membrane		
Acrdi.XM_015893611.1/1-1743 | Monca.augustus.g64061.t1/1-1713	0.160921	0.144427	eukaryotic translation initiation factor 2A	8	C:GO:0005615; P:GO:0032933; F:GO:0003723; C:GO:0032991;	C:extracellular space; P:SREBP signaling pathway; F:RNA binding; C:macromolecular complex; C:cytoplasmic part; P:protein phosphorylation; P:positive regulation of signal transduction; P:translation		
Acrdi.XM_015908992.1/1-2460 | Monca.adi2mcaRNA19002_R0/1-2445	0.133243	0.144407	Short transient receptor potential channel 2	1	P:GO:0006810	P:transport		
Acrdi.XM_015924020.1/1-1560 | Monca.augustus.g32438.t1/1-1614	0.177571	0.144405	spartin isoform X1					
Acrdi.XM_015903290.1/1-528 | Monca.augustus.g60211.t1/1-534	0.288162	0.144385	Heart and neural crest derivatives expressed transcript 2	15	P:GO:0060047; P:GO:0071908; F:GO:0046983; P:GO:0003142;	P:heart contraction; P:determination of intestine left/right asymmetry; F:protein dimerization activity; P:cardiogenic plate morphogenesis; P:embryonic heart tube formation; P:proepicardium development; P:embryonic viscerocranium morphogenesis; P:noradrenergic neuron differentiation; P:sympathetic nervous system devel		
Acrdi.XM_015900592.1/1-4413 | Monca.adi2mcaRNA9843_R0/1-3588	0.208927	0.144341	DNA polymerase theta	2	F:GO:0003676; P:GO:0006260	F:nucleic acid binding; P:DNA replication		
Acrdi.XM_015913485.1/1-3108 | Monca.augustus.g25374.t1/1-4494	0.192575	0.144309	ras-associating and dilute domain-containing -like	1	C:GO:0016020	C:membrane		
Acrdi.XM_015917906.1/1-492 | Monca.augustus.g39678.t1/1-489	0.178077	0.1443	transmembrane 229B	1	C:GO:0016020	C:membrane		
Acrdi.XM_015900634.1/1-729 | Monca.augustus.g28765.t1/1-720	0.0847523	0.144299	PREDICTED: uncharacterized protein LOC107335607	1	F:GO:0051087	F:chaperone binding		
Acrdi.XM_015911849.1/1-492 | Monca.augustus.g17902.t1/1-774	0.168544	0.144297	transmembrane 187	1	C:GO:0016021	C:integral component of membrane		
Acrdi.XM_015915481.1/1-1116 | Monca.augustus.g32768.t1/1-1092	0.120342	0.144243	Neuropeptide FF receptor 2	6	C:GO:0005887; F:GO:0004930; P:GO:0007186; P:GO:1901652;	C:integral component of plasma membrane; F:G-protein coupled receptor activity; P:G-protein coupled receptor signaling pathway; P:response to peptide; F:peptide binding; P:cellular response to hormone stimulus		
Acrdi.XM_015901889.1/1-453 | Monca.augustus.g7733.t1/1-453	0.138432	0.144235	microsomal glutathione S-transferase 1-like	2	C:GO:0016020; C:GO:0016021	C:membrane; C:integral component of membrane		
Acrdi.XM_015919122.1/1-1299 | Monca.augustus.g35163.t1/1-1302	0.156633	0.144212	zinc transporter 1-like	1	P:GO:0006810	P:transport		
Acrdi.XM_015908778.1/1-1953 | Monca.augustus.g18983.t1/1-4137	0.18213	0.144207	Serine threonine- kinase 36	1	P:GO:0044699	P:single-organism process		
Acrdi.XM_015917329.1/1-864 | Monca.augustus.g25542.t1/1-3084	0.206103	0.144206	voltage-dependent calcium channel subunit alpha-2 delta-1-like					
Acrdi.XM_015917608.1/1-954 | Monca.augustus.g7707.t1/1-1419	0.215927	0.14415	Basic leucine zipper and W2 domain-containing 1	2	C:GO:0005737; C:GO:0016020	C:cytoplasm; C:membrane		
Acrdi.XM_015908401.1/1-5019 | Monca.augustus.g9822.t1/1-5793	0.158988	0.144105	bromodomain adjacent to zinc finger domain 1A-like	2	F:GO:0008270; F:GO:0046872	F:zinc ion binding; F:metal ion binding		
Acrdi.XM_015913786.1/1-2952 | Monca.augustus.g57184.t1/1-5220	0.183759	0.144104	regulator of G- signaling 12-	8	C:GO:0005737; P:GO:0007165; F:GO:0005096; C:GO:0016020;	C:cytoplasm; P:signal transduction; F:GTPase activator activity; C:membrane; C:integral component of membrane; F:signal transducer activity, downstream of receptor; P:positive regulation of GTPase activity; C:plasma membrane		
Acrdi.XM_015914776.1/1-537 | Monca.augustus.g285.t1/1-546	0.194175	0.144098	PREDICTED: uncharacterized protein LOC107348715					
Acrdi.XM_015906088.1/1-1098 | Monca.augustus.g34431.t1/1-1560	0.188328	0.144088	cytochrome P450 4B1-like	2	F:GO:0016491; F:GO:0005488	F:oxidoreductase activity; F:binding		
Acrdi.XM_015905755.1/1-1206 | Monca.augustus.g9361.t1/1-1212	0.160187	0.144051	C-Jun-amino-terminal kinase-interacting 1-like					
Acrdi.XM_015916691.1/1-1959 | Monca.adi2mcaRNA27527_R2/1-1965	0.190918	0.144025	Pao retrotransposon peptidase superfamily	2	F:GO:0003676; P:GO:0015074	F:nucleic acid binding; P:DNA integration		
Acrdi.XM_015897751.1/1-4053 | Monca.augustus.g46334.t1/1-4158	0.168464	0.144008	DNA-directed RNA mitochondrial					
Acrdi.XM_015906604.1/1-573 | Monca.augustus.g63793.t1/1-741	0.154659	0.143955	RING finger 215-like	4	F:GO:0008270; F:GO:0046872; C:GO:0016020; C:GO:0016021	F:zinc ion binding; F:metal ion binding; C:membrane; C:integral component of membrane		
Acrdi.XM_015918486.1/1-1572 | Monca.adi2mcaRNA29512_R0/1-1584	0.115108	0.143941	neuronal acetylcholine receptor subunit alpha-10-like isoform X2	3	F:GO:0005216; P:GO:0034220; C:GO:0044425	F:ion channel activity; P:ion transmembrane transport; C:membrane part		
Acrdi.XM_015904399.1/1-6201 | Monca.augustus.g27198.t1/1-6447	0.164829	0.143884	Tetratricopeptide repeat 6	1	C:GO:0034464	C:BBSome		
Acrdi.XM_015903160.1/1-3243 | Monca.augustus.g32351.t1/1-3414	0.211589	0.143852	fibronectin type-III domain-containing 3A isoform X2	2	C:GO:0016020; C:GO:0016021	C:membrane; C:integral component of membrane		
Acrdi.XM_015923168.1/1-1347 | Monca.augustus.g51597.t1/1-1209	0.161861	0.143813	histamine H2 receptor-like isoform X1	4	C:GO:0005887; F:GO:0004930; P:GO:0007186; P:GO:0007166	C:integral component of plasma membrane; F:G-protein coupled receptor activity; P:G-protein coupled receptor signaling pathway; P:cell surface receptor signaling pathway		
Acrdi.XM_015904187.1/1-2118 | Monca.augustus.g17138.t1/1-1587	0.179509	0.143802	ATP-binding cassette sub-family D member 3	5	F:GO:0000166; F:GO:0016887; C:GO:0016020; P:GO:0006810;	F:nucleotide binding; F:ATPase activity; C:membrane; P:transport; F:transporter activity	EC:3.6.1.3; EC:3.6.1.15	Adenosinetriphosphatase; Nucleoside-triphosphate phosphatase
Acrdi.XM_015899105.1/1-1719 | Monca.augustus.g26265.t1/1-1644	0.207582	0.143795	fibrillin-2-like isoform X1	3	F:GO:0005509; C:GO:0016020; P:GO:0007156	F:calcium ion binding; C:membrane; P:homophilic cell adhesion via plasma membrane adhesion molecules		
Acrdi.XM_015904225.1/1-468 | Monca.augustus.g11838.t1/1-480	0.197652	0.143781	universal stress Slr1101-like	1	P:GO:0006950	P:response to stress		
Acrdi.XM_015902483.1/1-1050 | Monca.augustus.g42491.t1/1-1071	0.165534	0.143779	dopamine receptor 2-like	5	F:GO:0001588; P:GO:0001963; P:GO:0007165; F:GO:0035240;	F:dopamine neurotransmitter receptor activity, coupled via Gs; P:synaptic transmission, dopaminergic; P:signal transduction; F:dopamine binding; C:membrane		
Acrdi.XM_015909941.1/1-555 | Monca.adi2mcaRNA20077_R0/1-345	0.568447	0.143749	transposase					
Acrdi.XM_015920118.1/1-852 | Monca.augustus.g28553.t1/1-852	0.152178	0.143744	transmembrane 53-B-like					
Acrdi.XM_015918919.1/1-1062 | Monca.augustus.g48512.t1/1-636	0.157248	0.143737	melanocyte-stimulating hormone receptor-like	7	F:GO:0004930; F:GO:0004977; P:GO:0007186; P:GO:0007165;	F:G-protein coupled receptor activity; F:melanocortin receptor activity; P:G-protein coupled receptor signaling pathway; P:signal transduction; C:membrane; C:integral component of membrane; F:signal transducer activity		
Acrdi.XM_015895556.1/1-900 | Monca.augustus.g14882.t1/1-372	0.196374	0.143722	F-box LRR-repeat 20					
Acrdi.XM_015921068.1/1-1335 | Monca.augustus.g34988.t1/1-1374	0.14236	0.14372	WD repeat and SOCS box-containing 1-like	2	P:GO:0035556; C:GO:0005622	P:intracellular signal transduction; C:intracellular		
Acrdi.XM_015916706.1/1-369 | Monca.augustus.g37096.t1/1-378	0.0869102	0.143712	CASP8-associated 2					
Acrdi.XM_015896319.1/1-468 | Monca.adi2mcaRNA5205_R0/1-489	0.16939	0.143692	MOB kinase activator-like 4	2	P:GO:0016310; F:GO:0016301	P:phosphorylation; F:kinase activity		
Acrdi.XM_015911667.1/1-1752 | Monca.adi2mcaRNA21959_R0/1-1533	0.139169	0.143687	Nuclear pore complex Nup85	5	P:GO:0006406; C:GO:0031080; P:GO:0045893; P:GO:0006606;	P:mRNA export from nucleus; C:nuclear pore outer ring; P:positive regulation of transcription, DNA-templated; P:protein import into nucleus; F:structural constituent of nuclear pore		
Acrdi.XM_015907677.1/1-6021 | Monca.adi2mcaRNA17549_R0/1-5847	0.159284	0.143676	von Willebrand factor type EGF and pentraxin domain-containing 1	3	F:GO:0005509; C:GO:0016020; C:GO:0016021	F:calcium ion binding; C:membrane; C:integral component of membrane		
Acrdi.XM_015921152.1/1-948 | Monca.augustus.g12310.t1/1-1002	0.163648	0.143662	iron-sulfur NUBPL-like	1	F:GO:0005524	F:ATP binding		
Acrdi.XM_015896863.1/1-990 | Monca.augustus.g17707.t1/1-1206	0.130776	0.143638	DDB1- and CUL4-associated factor 10					
Acrdi.XM_015908052.1/1-2574 | Monca.augustus.g5044.t1/1-3039	0.16964	0.143619	Ubiquitin carboxyl-terminal hydrolase 38	3	P:GO:0006508; P:GO:0044267; F:GO:0036459	P:proteolysis; P:cellular protein metabolic process; F:thiol-dependent ubiquitinyl hydrolase activity	EC:3.4.19.12	Ubiquitinyl hydrolase 1
Acrdi.XM_015897610.1/1-528 | Monca.adi2mcaRNA592_R6/1-453	1.35585	0.143611	glutamic acid-rich -like					
Acrdi.XM_015910996.1/1-426 | Monca.augustus.g59079.t1/1-558	0.186679	0.143605	40S ribosomal S19 isoform 1	22	F:GO:0019901; P:GO:0060265; P:GO:0060266; F:GO:0042803;	F:protein kinase binding; P:positive regulation of respiratory burst involved in inflammatory response; P:negative regulation of respiratory burst involved in inflammatory response; F:protein homodimerization activity; C:cytosolic small ribosomal subunit; P:nucleolus organization; P:ribosomal small subunit assembly; C:nucleolu		
Acrdi.XM_015913725.1/1-429 | Monca.augustus.g21634.t1/1-618	0.166234	0.143579	keratin-associated 5-9-like	2	C:GO:0016020; C:GO:0016021	C:membrane; C:integral component of membrane		
Acrdi.XM_015915976.1/1-1494 | Monca.augustus.g60993.t1/1-1578	0.122798	0.143575	matrix metallo ase-24-like	1	F:GO:0016787	F:hydrolase activity		
Acrdi.XM_015900906.1/1-1413 | Monca.augustus.g27862.t1/1-1524	0.214172	0.143566	Multidrug and toxin extrusion	6	P:GO:0006855; P:GO:0055085; F:GO:0015297; C:GO:0016020;	P:drug transmembrane transport; P:transmembrane transport; F:antiporter activity; C:membrane; C:integral component of membrane; F:drug transmembrane transporter activity		
Acrdi.XM_015913773.1/1-939 | Monca.augustus.g45558.t1/1-957	0.2149	0.143536	Alpha beta hydrolase domain-containing 11	1	F:GO:0003824	F:catalytic activity		
Acrdi.XM_015898529.1/1-1509 | Monca.augustus.g2151.t1/1-1479	0.184121	0.143529	synaptic vesicle 2-related -like isoform X2					
Acrdi.XM_015918165.1/1-447 | Monca.augustus.g66568.t1/1-453	0.243388	0.143512	40S ribosomal S13	17	C:GO:0022627; P:GO:0006364; C:GO:0005730; P:GO:0006413;	C:cytosolic small ribosomal subunit; P:rRNA processing; C:nucleolus; P:translational initiation; F:small ribosomal subunit rRNA binding; C:nucleoplasm; P:viral transcription; C:extracellular exosome; F:protein binding; P:SRP-dependent cotranslational protein targeting to membrane; C:focal adhesion; F:structural constituent of ri		
Acrdi.XM_015908410.1/1-1668 | Monca.augustus.g34801.t1/1-2988	0.224251	0.143493	myotubularin-related 1 isoform X1					
Acrdi.XM_015912806.1/1-573 | Monca.augustus.g26429.t1/1-696	0.165921	0.143481	reticulon-1-A isoform X3	2	C:GO:0016020; C:GO:0012505	C:membrane; C:endomembrane system		
Acrdi.XM_015910913.1/1-1764 | Monca.augustus.g41341.t1/1-1734	0.181835	0.143473	Cubilin	6	F:GO:0005509; C:GO:0016020; C:GO:0016021; F:GO:0004872;	F:calcium ion binding; C:membrane; C:integral component of membrane; F:receptor activity; P:receptor-mediated endocytosis; F:hydrolase activity		
Acrdi.XM_015891850.1/1-702 | Monca.augustus.g55356.t1/1-708	0.160278	0.143466	neurogenic differentiation factor 1	1	F:GO:0046983	F:protein dimerization activity		
Acrdi.XM_015917100.1/1-1563 | Monca.adi2mcaRNA27991_R0/1-1554	0.185466	0.143427	cell cycle checkpoint RAD17 isoform X2	9	C:GO:0000781; P:GO:0000077; F:GO:0005515; P:GO:0007093;	C:chromosome, telomeric region; P:DNA damage checkpoint; F:protein binding; P:mitotic cell cycle checkpoint; P:negative regulation of DNA replication; C:nucleolus; P:DNA metabolic process; P:regulation of phosphorylation; C:nucleoplasm		
Acrdi.XM_015917458.1/1-651 | Monca.augustus.g29298.t1/1-651	0.214083	0.143426	ADP-ribosylation factor 4A	3	F:GO:0005525; C:GO:0005634; P:GO:0007264	F:GTP binding; C:nucleus; P:small GTPase mediated signal transduction		
Acrdi.XM_015911999.1/1-741 | Monca.augustus.g35302.t1/1-1497	0.253315	0.143406	delphilin-like isoform X1	5	F:GO:0003779; C:GO:0016020; C:GO:0016021; P:GO:0007015;	F:actin binding; C:membrane; C:integral component of membrane; P:actin filament organization; P:cell cycle		
Acrdi.XM_015916058.1/1-1338 | Monca.augustus.g27786.t1/1-1380	0.256144	0.143389	CREB3 regulatory factor isoform X1	3	F:GO:0003700; P:GO:0006355; F:GO:0043565	F:transcription factor activity, sequence-specific DNA binding; P:regulation of transcription, DNA-templated; F:sequence-specific DNA binding		
Acrdi.XM_015900164.1/1-624 | Monca.augustus.g59999.t1/1-624	0.155521	0.143365	tctex1 domain-containing 1-B-like					
Acrdi.XM_015896879.1/1-795 | Monca.augustus.g60749.t1/1-795	0.118543	0.143322	PREDICTED: uncharacterized protein LOC107332155					
Acrdi.XM_015914039.1/1-756 | Monca.augustus.g18743.t1/1-753	0.181006	0.143296	probable U3 small nucleolar RNA-associated 11	4	C:GO:0032040; P:GO:0000462; P:GO:0000479; C:GO:0005730	C:small-subunit processome; P:maturation of SSU-rRNA from tricistronic rRNA transcript (SSU-rRNA, 5.8S rRNA, LSU-rRNA); P:endonucleolytic cleavage of tricistronic rRNA transcript (SSU-rRNA, 5.8S rRNA, LSU-rRNA); C:nucleolus		
Acrdi.XM_015914838.1/1-3663 | Monca.adi2mcaRNA2729_R0/1-3693	0.493544	0.143258	Short transient receptor potential channel 2	1	P:GO:0006810	P:transport		
Acrdi.XM_015904699.1/1-834 | Monca.adi2mcaRNA14244_R1/1-834	0.160948	0.143253	1-acyl-sn-glycerol-3-phosphate acyltransferase alpha	1	F:GO:0016746	F:transferase activity, transferring acyl groups		
Acrdi.XM_015895897.1/1-2883 | Monca.augustus.g36104.t1/1-3825	0.159615	0.143243	Axonemal dynein light chain domain-containing 1					
Acrdi.XM_015895993.1/1-1872 | Monca.augustus.g2190.t1/1-1863	0.238588	0.143219	PREDICTED: uncharacterized protein LOC107331423 isoform X1					
Acrdi.XM_015908453.1/1-2112 | Monca.augustus.g33941.t1/1-2103	0.20137	0.143179	WD repeat-containing 93-like	2	F:GO:0016651; P:GO:0022900	F:oxidoreductase activity, acting on NAD(P)H; P:electron transport chain		
Acrdi.XM_015900606.1/1-717 | Monca.augustus.g7877.t1/1-762	0.132028	0.143158	PREDICTED: uncharacterized protein LOC107335583	2	C:GO:0016020; C:GO:0016021	C:membrane; C:integral component of membrane		
Acrdi.XM_015904730.1/1-633 | Monca.augustus.g22467.t1/1-1416	0.179217	0.143132	glycine receptor subunit alpha-2-like	5	F:GO:0099600; P:GO:0034220; C:GO:0016020; F:GO:0005254;	F:transmembrane receptor activity; P:ion transmembrane transport; C:membrane; F:chloride channel activity; P:chloride transport		
Acrdi.XM_015914007.1/1-492 | Monca.augustus.g44889.t1/1-567	0.121715	0.143115	PREDICTED: uncharacterized protein LOC107348017					
Acrdi.XM_015904865.1/1-1854 | Monca.augustus.g40340.t1/1-1836	0.143197	0.143104	gamma-glutamyltranspeptidase 1-like	1	F:GO:0016740	F:transferase activity		
Acrdi.XM_015918182.1/1-336 | Monca.augustus.g40354.t1/1-609	0.244106	0.143088	tctex1 domain-containing 1-B-like					
Acrdi.XM_015900401.1/1-738 | Monca.augustus.g45079.t1/1-753	0.0727323	0.143051	14-3-3 zeta delta-like	1	F:GO:0019904	F:protein domain specific binding		
Acrdi.XM_015918687.1/1-471 | Monca.augustus.g47177.t1/1-465	0.208195	0.143027	PREDICTED: uncharacterized protein LOC107352348	2	C:GO:0016020; C:GO:0016021	C:membrane; C:integral component of membrane		
Acrdi.XM_015917256.1/1-2673 | Monca.augustus.g55540.t1/1-2442	0.171047	0.143025	arginine N-methyltransferase 9	4	F:GO:0008168; F:GO:0016740; P:GO:0006479; P:GO:0032259	F:methyltransferase activity; F:transferase activity; P:protein methylation; P:methylation		
Acrdi.XM_015904193.1/1-669 | Monca.augustus.g61674.t1/1-669	0.108886	0.143021	radial spoke head 1 homolog	1	C:GO:0044424	C:intracellular part		
Acrdi.XM_015899443.1/1-351 | Monca.augustus.g17677.t1/1-351	0.183071	0.143014	DNA polymerase delta subunit 4	2	C:GO:0005634; P:GO:0006260	C:nucleus; P:DNA replication		
Acrdi.XM_015897393.1/1-1434 | Monca.augustus.g29307.t1/1-1452	0.204193	0.143	PREDICTED: uncharacterized protein LOC107332658 isoform X1					
Acrdi.XM_015893169.1/1-900 | Monca.adi2mcaRNA39447_R0/1-876	0.782791	0.142986	Integrase recombinase xerD-like	5	F:GO:0003677; C:GO:0016020; C:GO:0016021; P:GO:0015074;	F:DNA binding; C:membrane; C:integral component of membrane; P:DNA integration; P:DNA recombination		
Acrdi.XM_015901877.1/1-2553 | Monca.adi2mcaRNA11174_R0/1-2586	0.154214	0.14296	metabotropic glutamate receptor 2	5	C:GO:0042734; C:GO:0005887; F:GO:0008066; P:GO:0007196;	C:presynaptic membrane; C:integral component of plasma membrane; F:glutamate receptor activity; P:adenylate cyclase-inhibiting G-protein coupled glutamate receptor signaling pathway; P:regulation of synaptic transmission, glutamatergic		
Acrdi.XM_015909738.1/1-1002 | Monca.augustus.g2263.t1/1-933	0.254022	0.142948	Metaxin-1	1	C:GO:0016020	C:membrane		
Acrdi.XM_015911342.1/1-1434 | Monca.augustus.g35294.t1/1-2871	0.149093	0.142937	anoctamin-5-like isoform X1	1	C:GO:0016020	C:membrane		
Acrdi.XM_015911159.1/1-699 | Monca.augustus.g24019.t1/1-702	0.217518	0.142925	ras-related Rab-20-like	7	C:GO:0005789; C:GO:0030139; C:GO:0005769; P:GO:0006888;	C:endoplasmic reticulum membrane; C:endocytic vesicle; C:early endosome; P:ER to Golgi vesicle-mediated transport; C:Golgi membrane; P:regulation of endocytosis; C:plasma membrane		
Acrdi.XM_015901301.1/1-4827 | Monca.augustus.g43367.t1/1-4974	0.169109	0.142925	NACHT domain- and WD repeat-containing 1-like					
Acrdi.XM_015922806.1/1-597 | Monca.augustus.g34383.t1/1-501	0.200595	0.142917	nucleoredoxin 2	2	C:GO:0005737; F:GO:0004791	C:cytoplasm; F:thioredoxin-disulfide reductase activity	EC:1.8.1.9	Thioredoxin-disulfide reductase
Acrdi.XM_015908106.1/1-1590 | Monca.augustus.g33242.t1/1-3030	0.186968	0.142909	Poly [ADP-ribose] polymerase 1	7	P:GO:0051103; F:GO:0016757; F:GO:0003910; F:GO:0097159;	P:DNA ligation involved in DNA repair; F:transferase activity, transferring glycosyl groups; F:DNA ligase (ATP) activity; F:organic cyclic compound binding; F:heterocyclic comp	EC:6.5.1.1	DNA ligase (ATP)
Acrdi.XM_015895541.1/1-2727 | Monca.adi2mcaRNA4299_R0/1-2544	0.19889	0.1429	DNA helicase MCM9	7	C:GO:0005634; F:GO:0003677; F:GO:0005524; P:GO:0006260;	C:nucleus; F:DNA binding; F:ATP binding; P:DNA replication; P:double-strand break repair via homologous recombination; C:MCM8-MCM9 complex; F:hydrolase activity		
Acrdi.XM_015920654.1/1-1455 | Monca.augustus.g4868.t1/1-1719	0.186017	0.142897	Beta-1,4 N-acetylgalactosaminyltransferase 1	1	C:GO:0016020	C:membrane		
Acrdi.XM_015899423.1/1-1146 | Monca.augustus.g35514.t1/1-2556	0.130953	0.142885	Trafficking particle complex subunit 12	1	F:GO:0004175	F:endopeptidase activity		
Acrdi.XM_015906632.1/1-1245 | Monca.adi2mcaRNA16409_R4/1-1014	0.458168	0.142882	Retrovirus-related Pol poly from transposon 297					
Acrdi.XM_015903992.1/1-1275 | Monca.augustus.g27772.t1/1-2622	0.117894	0.142877	Golgi-associated plant pathogenesis-related 1	1	C:GO:0005576	C:extracellular region		
Acrdi.XM_015919456.1/1-663 | Monca.augustus.g25232.t1/1-1647	0.175985	0.142876	Polypeptide N-acetylgalactosaminyltransferase 11	6	P:GO:0050794; P:GO:0018243; C:GO:0000139; F:GO:0005112;	P:regulation of cellular process; P:protein O-linked glycosylation via threonine; C:Golgi membrane; F:Notch binding; P:O-glycan processing; F:polypeptide N-acetylgalactosami	EC:2.4.1.41	Polypeptide N-acetylgalactosaminyltransferase
Acrdi.XM_015905955.1/1-2025 | Monca.augustus.g60546.t1/1-2043	0.165361	0.142872	GTP-binding 10	3	F:GO:0005525; F:GO:0000287; F:GO:0003924	F:GTP binding; F:magnesium ion binding; F:GTPase activity		
Acrdi.XM_015911134.1/1-486 | Monca.augustus.g24011.t1/1-2322	0.165242	0.142849	ubiquitin thioesterase otulin-like					
Acrdi.XM_015906237.1/1-2007 | Monca.augustus.g21677.t1/1-2487	0.162835	0.142823	coiled-coil domain-containing 157 isoform X1					
Acrdi.XM_015909746.1/1-2298 | Monca.augustus.g17531.t1/1-2154	0.132091	0.142823	threonine--tRNA cytoplasmic	4	F:GO:0003723; C:GO:0005737; F:GO:0004829; P:GO:0006435	F:RNA binding; C:cytoplasm; F:threonine-tRNA ligase activity; P:threonyl-tRNA aminoacylation	EC:6.1.1.3	Threonine--tRNA ligase
Acrdi.XM_015904593.1/1-1896 | Monca.augustus.g63204.t1/1-1509	0.452979	0.142788	mediator of RNA polymerase II transcription subunit 34-like	11	C:GO:0005634; F:GO:0003677; P:GO:0006284; C:GO:0005737;	C:nucleus; F:DNA binding; P:base-excision repair; C:cytoplasm; F:ATP-dependent 3'-5' DNA helicase activity; P:DNA replication; P:DNA duplex unwinding; P:DNA recombination	EC:3.6.1.3; EC:3.6.1.15	Adenosinetriphosphatase; Nucleoside-triphosphate phosphatase
Acrdi.XM_015891624.1/1-1629 | Monca.augustus.g49977.t1/1-1938	0.127146	0.142783	probable 28S rRNA (cytosine(4447)-C(5))-methyltransferase	4	F:GO:0003723; P:GO:0006364; F:GO:0008757; P:GO:0032259	F:RNA binding; P:rRNA processing; F:S-adenosylmethionine-dependent methyltransferase activity; P:methylation		
Acrdi.XM_015916144.1/1-2640 | Monca.adi2mcaRNA26927_R0/1-2775	0.114773	0.142783	tyrosine decarboxylase Tdc	1	F:GO:0016829	F:lyase activity		
Acrdi.XM_015916053.1/1-1116 | Monca.augustus.g5222.t1/1-933	0.173754	0.14278	RNA-binding	2	C:GO:0044428; F:GO:0003723	C:nuclear part; F:RNA binding		
Acrdi.XM_015898890.1/1-2298 | Monca.augustus.g35490.t1/1-2313	0.137062	0.142773	E3 ubiquitin- ligase TRIM71-like	3	F:GO:0008270; F:GO:0046872; C:GO:0005622	F:zinc ion binding; F:metal ion binding; C:intracellular		
Acrdi.XM_015923521.1/1-852 | Monca.augustus.g47448.t1/1-861	0.136172	0.142718	2-epi-5-epi-valiolone synthase					
Acrdi.XM_015898839.1/1-1194 | Monca.augustus.g5110.t1/1-1503	0.170315	0.142713	ADP-ribosylation factor GTPase-activating 1-like	2	F:GO:0005096; P:GO:0043547	F:GTPase activator activity; P:positive regulation of GTPase activity		
Acrdi.XM_015901867.1/1-750 | Monca.augustus.g28480.t1/1-723	0.18916	0.142651	Meiotic recombination REC114-					
Acrdi.XM_015915295.1/1-2781 | Monca.augustus.g25265.t1/1-3597	0.142982	0.142625	Polycystic kidney disease 1-like 2	6	P:GO:0050982; F:GO:0005509; F:GO:0005262; C:GO:0016020;	P:detection of mechanical stimulus; F:calcium ion binding; F:calcium channel activity; C:membrane; C:integral component of membrane; P:calcium ion transmembrane transport		
Acrdi.XM_015923628.1/1-543 | Monca.adi2mcaRNA35310_R2/1-627	0.563232	0.14262	PREDICTED: uncharacterized protein LOC107356992					
Acrdi.XM_015905378.1/1-381 | Monca.augustus.g61120.t1/1-4446	0.130615	0.142613	C-myc promoter-binding -like					
Acrdi.XM_015905969.1/1-1230 | Monca.augustus.g69492.t1/1-1242	0.167377	0.142612	Zinc transporter	8	P:GO:0010043; P:GO:0061088; C:GO:0016021; F:GO:0005385;	P:response to zinc ion; P:regulation of sequestering of zinc ion; C:integral component of membrane; F:zinc ion transmembrane transporter activity; C:cytoplasmic vesicle; P:zinc II ion transmembrane transport; C:vacuole; C:plasma membrane		
Acrdi.XM_015922534.1/1-1185 | Monca.augustus.g23470.t1/1-1146	0.200645	0.142574	laminin subunit alpha-1-like	7	P:GO:0050794; P:GO:0016043; P:GO:0007155; P:GO:0009653;	P:regulation of cellular process; P:cellular component organization; P:cell adhesion; P:anatomical structure morphogenesis; P:animal organ development; P:single-organism cellular process; P:response to stimulus		
Acrdi.XM_015910807.1/1-1854 | Monca.augustus.g57309.t1/1-2220	0.163179	0.142527	glycosyltransferase LARGE1	4	F:GO:0016757; C:GO:0016020; C:GO:0016021; F:GO:0016740	F:transferase activity, transferring glycosyl groups; C:membrane; C:integral component of membrane; F:transferase activity		
Acrdi.XM_015905693.1/1-1011 | Monca.adi2mcaRNA31_R0/1-927	0.172941	0.142494	spinster homolog 1	7	P:GO:0040011; C:GO:0005739; C:GO:0031982; P:GO:0003376;	P:locomotion; C:mitochondrion; C:vesicle; P:sphingosine-1-phosphate signaling pathway; P:transport; C:lysosomal membrane; F:sphingolipid transporter activity		
Acrdi.XM_015901247.1/1-408 | Monca.augustus.g45969.t1/1-969	0.204939	0.142466	transcription initiation factor TFIID subunit 7-like					
Acrdi.XM_015903328.1/1-435 | Monca.augustus.g28780.t1/1-1089	0.115592	0.142458	tryptophanyl-tRNA synthetase	2	F:GO:0016874; P:GO:0006412	F:ligase activity; P:translation		
Acrdi.XM_015913135.1/1-1839 | Monca.adi2mcaRNA23574_R0/1-1593	0.173982	0.142417	Centrosomal POC5					
Acrdi.XM_015898776.1/1-1401 | Monca.augustus.g58960.t1/1-1401	0.094529	0.142397	mab-21-like 2 isoform X1					
Acrdi.XM_015894063.1/1-321 | Monca.augustus.g34656.t1/1-822	0.182384	0.142395	Centrosomal of 290 kDa	1	C:GO:0043229	C:intracellular organelle		
Acrdi.XM_015893553.1/1-711 | Monca.augustus.g13228.t1/1-708	0.141154	0.142392	PREDICTED: uncharacterized protein LOC107328797					
Acrdi.XM_015906108.1/1-519 | Monca.augustus.g34407.t1/1-699	0.211645	0.142382	PREDICTED: uncharacterized protein LOC107340731					
Acrdi.XM_015901959.1/1-1653 | Monca.augustus.g45531.t1/1-1683	0.200871	0.142369	GPI transamidase component PIG-S-like	4	C:GO:0016020; C:GO:0016021; P:GO:0016255; C:GO:0042765	C:membrane; C:integral component of membrane; P:attachment of GPI anchor to protein; C:GPI-anchor transamidase complex		
Acrdi.XM_015894427.1/1-1317 | Monca.augustus.g67151.t1/1-1551	0.128897	0.142349	dnaJ homolog subfamily C member 7					
Acrdi.XM_015916990.1/1-2544 | Monca.augustus.g26586.t1/1-2616	0.131467	0.142329	bifunctional heparan sulfate N-deacetylase N-sulfotransferase 2 isofor	4	F:GO:0015016; F:GO:0008146; F:GO:0016740; F:GO:0016787	F:[heparan sulfate]-glucosamine N-sulfotransferase activity; F:sulfotransferase activity; F:transferase activity; F:hydrolase activity		
Acrdi.XM_015903353.1/1-1377 | Monca.augustus.g36011.t1/1-1791	0.168473	0.142323	bifunctional ATP-dependent dihydroxyacetone kinase FAD-AMP lyase ((	5	P:GO:0006071; F:GO:0005524; F:GO:0004371; F:GO:0016829;	P:glycerol metabolic process; F:ATP binding; F:glycerone kinase activity; F:lyase activity; P:phosphorylation	EC:2.7.1.29	Glycerone kinase
Acrdi.XM_015908216.1/1-1953 | Monca.adi2mcaRNA18175_R1/1-1968	0.124245	0.142303	EGF-like repeat and discoidin I-like domain-containing 3	1	F:GO:0070492	F:oligosaccharide binding		
Acrdi.XM_015901778.1/1-561 | Monca.augustus.g58378.t1/1-546	0.169674	0.142282	Dual specificity phosphatase 10	8	P:GO:0000188; F:GO:0033549; P:GO:0006470; P:GO:0032873;	P:inactivation of MAPK activity; F:MAP kinase phosphatase activity; P:protein dephosphorylation; P:negative regulation of stress-activated MAPK cascade; C:Golgi apparatus; F	EC:3.1.3.16; EC:3.1.3.41	Protein-serine/threonine phosphatase; 4-nitrophenylphosphatase
Acrdi.XM_015923511.1/1-1908 | Monca.augustus.g47459.t1/1-2325	0.134099	0.142271	N-terminal kinase	3	F:GO:0005524; F:GO:0004672; P:GO:0006468	F:ATP binding; F:protein kinase activity; P:protein phosphorylation		
Acrdi.XM_015900403.1/1-696 | Monca.augustus.g46627.t1/1-747	0.1234	0.142259	14-3-3	1	F:GO:0019904	F:protein domain specific binding		
Acrdi.XM_015891798.1/1-483 | Monca.augustus.g39201.t1/1-936	0.130041	0.142219	DNA-binding SATB1	1	C:GO:0016021	C:integral component of membrane		
Acrdi.XM_015916923.1/1-7167 | Monca.augustus.g38699.t1/1-7086	0.173636	0.142196	leucine-rich repeat serine threonine- kinase 1-like isoform X1	3	F:GO:0005525; P:GO:0007264; C:GO:0005622	F:GTP binding; P:small GTPase mediated signal transduction; C:intracellular		
Acrdi.XM_015920902.1/1-1938 | Monca.augustus.g46854.t1/1-2787	0.137112	0.142153	glutamate receptor 2-like isoform X1	12	C:GO:0005887; P:GO:0006811; C:GO:0036477; C:GO:0098794;	C:integral component of plasma membrane; P:ion transport; C:somatodendritic compartment; C:postsynapse; F:protein binding; P:regulation of cellular process; P:regulation of signaling; C:macromolecular complex; F:ionotropic glutamate receptor activity; P:single-organism process; P:cellular process; P:response to stimulus		
Acrdi.XM_015893489.1/1-1023 | Monca.augustus.g53470.t1/1-1188	0.156297	0.142129	Ankyrin PH and SEC7 domain containing secG	2	F:GO:0004842; P:GO:0016567	F:ubiquitin-protein transferase activity; P:protein ubiquitination		
Acrdi.XM_015911331.1/1-5223 | Monca.adi2mcaRNA21597_R0/1-5139	0.172221	0.142126	voltage-dependent T-type calcium channel subunit alpha-1H-like	5	P:GO:0034220; F:GO:0005261; P:GO:0006816; P:GO:0086010;	P:ion transmembrane transport; F:cation channel activity; P:calcium ion transport; P:membrane depolarization during action potential; C:plasma membrane		
Acrdi.XM_015895891.1/1-2367 | Monca.augustus.g4113.t1/1-2346	0.16513	0.142119	PREDICTED: uncharacterized protein LOC107331317	2	C:GO:0016020; C:GO:0016021	C:membrane; C:integral component of membrane		
Acrdi.XM_015913658.1/1-1311 | Monca.adi2mcaRNA24176_R0/1-1335	0.130964	0.14211	kinesin KIFC3 isoform X3	5	F:GO:0000166; F:GO:0016887; F:GO:0003777; C:GO:0005871;	F:nucleotide binding; F:ATPase activity; F:microtubule motor activity; C:kinesin complex; P:microtubule-based movement	EC:3.6.1.3; EC:3.6.1.15	Adenosinetriphosphatase; Nucleoside-triphosphate phosphatase
Acrdi.XM_015913121.1/1-3873 | Monca.augustus.g68058.t1/1-3885	0.128328	0.142098	EGF-like repeat and discoidin I-like domain-containing 3	1	F:GO:0070492	F:oligosaccharide binding		
Acrdi.XM_015906729.1/1-3876 | Monca.augustus.g12125.t1/1-3852	0.262068	0.142092	Stonustoxin subunit alpha					
Acrdi.XM_015919215.1/1-492 | Monca.augustus.g54964.t1/1-492	0.173799	0.142078	filamin-A-like isoform X1	1	F:GO:0051015	F:actin filament binding		
Acrdi.XM_015911239.1/1-1002 | Monca.augustus.g15414.t1/1-1047	0.107156	0.142058	N-glycosylase DNA lyase	8	P:GO:0003313; C:GO:0005634; P:GO:0006284; P:GO:0055013;	P:heart rudiment development; C:nucleus; P:base-excision repair; P:cardiac muscle cell development; F:DNA N-glycosylase activity; P:brain development; P:regulation of neuron projection development; P:negative regulation of neuron apoptotic process		
Acrdi.XM_015893160.1/1-1326 | Monca.adi2mcaRNA39216_R7/1-1272	0.53529	0.142056	Retrovirus-related Pol poly from transposon					
Acrdi.XM_015907741.1/1-1146 | Monca.augustus.g62575.t1/1-1446	0.174127	0.142013	pyruvate dehydrogenase X mitochondrial	3	F:GO:0016746; P:GO:0008152; F:GO:0016740	F:transferase activity, transferring acyl groups; P:metabolic process; F:transferase activity		
Acrdi.XM_015907026.1/1-312 | Monca.augustus.g43281.t1/1-459	0.216495	0.14198	ribonuclease H2 subunit C-like	3	F:GO:0004523; C:GO:0032299; P:GO:0006401	F:RNA-DNA hybrid ribonuclease activity; C:ribonuclease H2 complex; P:RNA catabolic process	EC:3.1.30; EC:3.1.26; EC:3.1.26.4	Acting on ester bonds; Acting on ester bonds; Ribonuclease H
Acrdi.XM_015894593.1/1-1056 | Monca.augustus.g70676.t1/1-1188	0.186482	0.141967	F-box WD repeat-containing 4					
Acrdi.XM_015919787.1/1-585 | Monca.adi2mcaRNA31045_R0/1-573	0.134571	0.141948	vesicular integral-membrane VIP36	1	C:GO:0016020	C:membrane		
Acrdi.XM_015904495.1/1-456 | Monca.augustus.g33588.t1/1-825	0.20924	0.141943	CMSS1					
Acrdi.XM_015898401.1/1-1653 | Monca.augustus.g58532.t1/1-1659	0.179584	0.141923	acyl-coenzyme A thioesterase 11 isoform X2	1	F:GO:0008289	F:lipid binding		
Acrdi.XM_015904674.1/1-10176 | Monca.augustus.g36182.t1/1-11922	0.203934	0.141914	vacuolar sorting-associated 13B isoform X1	4	P:GO:0045053; C:GO:0019898; P:GO:0006623; C:GO:0005622	P:protein retention in Golgi apparatus; C:extrinsic component of membrane; P:protein targeting to vacuole; C:intracellular		
Acrdi.XM_015915254.1/1-3243 | Monca.augustus.g46144.t1/1-3513	0.149183	0.141899	ubiquitin carboxyl-terminal hydrolase 47 isoform X1	4	P:GO:0044260; P:GO:0050794; P:GO:0044238; C:GO:0044424	P:cellular macromolecule metabolic process; P:regulation of cellular process; P:primary metabolic process; C:intracellular part		
Acrdi.XM_015898331.1/1-999 | Monca.augustus.g70900.t1/1-1038	0.148541	0.141896	ribosome biogenesis regulatory homolog	5	P:GO:0000055; P:GO:0042273; P:GO:0000447; C:GO:0005730;	P:ribosomal large subunit export from nucleus; P:ribosomal large subunit biogenesis; P:endonucleolytic cleavage in ITS1 to separate SSU-rRNA from 5.8S rRNA and LSU-rRNA from tricistronic rRNA transcript (SSU-rRNA, 5.8S rRNA, LSU-rRNA); C:nucleolus; C:preribosome, large subunit precursor		
Acrdi.XM_015915071.1/1-603 | Monca.augustus.g27922.t1/1-963	0.238283	0.141886	methyltransferase 9	1	C:GO:0005575	C:cellular_component		
Acrdi.XM_015905596.1/1-1887 | Monca.augustus.g24054.t1/1-1788	0.0767094	0.141875	plastin-3 isoform X1	2	F:GO:0003779; F:GO:0005509	F:actin binding; F:calcium ion binding		
Acrdi.XM_015909171.1/1-1044 | Monca.augustus.g57513.t1/1-1047	0.125732	0.14187	TPA: opsin	5	F:GO:0004930; F:GO:0009881; P:GO:0007165; P:GO:0009583;	F:G-protein coupled receptor activity; F:photoreceptor activity; P:signal transduction; P:detection of light stimulus; C:integral component of membrane		
Acrdi.XM_015912986.1/1-876 | Monca.augustus.g64754.t1/1-861	0.113117	0.141868	potassium channel subfamily K member 5	4	C:GO:0005887; P:GO:0071805; P:GO:0030322; F:GO:0022841	C:integral component of plasma membrane; P:potassium ion transmembrane transport; P:stabilization of membrane potential; F:potassium ion leak channel activity		
Acrdi.XM_015918075.1/1-726 | Monca.augustus.g34632.t1/1-669	0.143822	0.141861	ras-related Rab-38-like	4	F:GO:0005525; P:GO:0007264; C:GO:0005776; C:GO:0005622	F:GTP binding; P:small GTPase mediated signal transduction; C:autophagosome; C:intracellular		
Acrdi.XM_015915035.1/1-804 | Monca.adi2mcaRNA25654_R0/1-855	0.149042	0.141847	Serine threonine- kinase					
Acrdi.XM_015896219.1/1-537 | Monca.augustus.g23048.t1/1-546	0.0742148	0.141815	PREDICTED: uncharacterized protein LOC107331612					
Acrdi.XM_015916504.1/1-1206 | Monca.adi2mcaRNA27327_R1/1-1363	0.151568	0.141802	lysyl oxidase homolog 3	7	P:GO:0050794; F:GO:0016491; P:GO:0044707; P:GO:0008152;	P:regulation of cellular process; F:oxidoreductase activity; P:single-multicellular organism process; P:metabolic process; C:extracellular region part; P:single-organism cellular process; F:binding		
Acrdi.XM_015902288.1/1-1374 | Monca.augustus.g35102.t1/1-1656	0.108827	0.141802	phosphatase 1G isoform X2	4	F:GO:0004722; P:GO:0007417; F:GO:0046872; P:GO:0006470	F:protein serine/threonine phosphatase activity; P:central nervous system development; F:metal ion binding; P:protein dephosphorylation	EC:3.1.3.16; EC:3.1.3.41	Protein-serine/threonine phosphatase; 4-nitrophenylphosphatase
Acrdi.XM_015910599.1/1-480 | Monca.augustus.g45385.t1/1-660	0.216149	0.141761	40S ribosomal S8	1	C:GO:0044424	C:intracellular part		
Acrdi.XM_015898593.1/1-879 | Monca.augustus.g4403.t1/1-1953	0.136826	0.141758	methyltransferase 25 isoform X1					
Acrdi.XM_015909946.1/1-825 | Monca.augustus.g24151.t1/1-825	0.197415	0.141755	DNA damage-regulated autophagy modulator 1	3	C:GO:0016020; C:GO:0016021; C:GO:0005764	C:membrane; C:integral component of membrane; C:lysosome		
Acrdi.XM_015905347.1/1-3441 | Monca.augustus.g12756.t1/1-3711	0.166052	0.141753	condensin-2 complex subunit G2 isoform X1	5	P:GO:0051301; C:GO:0005634; P:GO:0030261; P:GO:0007067;	P:cell division; C:nucleus; P:chromosome condensation; P:mitotic nuclear division; P:cell cycle		
Acrdi.XM_015918489.1/1-1605 | Monca.adi2mcaRNA29507_R0/1-1449	0.146029	0.14175	neuronal acetylcholine receptor subunit alpha-3-like isoform X1	2	P:GO:0006811; C:GO:0016020	P:ion transport; C:membrane		
Acrdi.XM_015900670.1/1-735 | Monca.augustus.g55755.t1/1-921	0.13548	0.141749	Ubiquinone biosynthesis O- mitochondrial	3	C:GO:0005737; F:GO:0008757; P:GO:0032259	C:cytoplasm; F:S-adenosylmethionine-dependent methyltransferase activity; P:methylation		
Acrdi.XM_015914720.1/1-5145 | Monca.adi2mcaRNA25351_R0/1-5094	0.158344	0.141721	voltage-gated sodium channel	7	P:GO:0006814; P:GO:0034220; C:GO:0016021; P:GO:0086010;	P:sodium ion transport; P:ion transmembrane transport; C:integral component of membrane; P:membrane depolarization during action potential; C:plasma membrane; F:voltage-gated sodium channel activity; P:neuronal action potential		
Acrdi.XM_015922293.1/1-1128 | Monca.augustus.g22296.t1/1-3282	0.167686	0.141704	phosphatidylinositol 3,4,5-trisphosphate 5-phosphatase					
Acrdi.XM_015903631.1/1-2451 | Monca.augustus.g15430.t1/1-2799	0.176872	0.141701	WD repeat-containing 3	3	F:GO:0003723; C:GO:0030684; C:GO:0005730	F:RNA binding; C:preribosome; C:nucleolus		
Acrdi.XM_015918234.1/1-804 | Monca.augustus.g48579.t1/1-873	0.166825	0.141699	coiled-coil domain-containing 169-like	6	C:GO:0005667; C:GO:0005634; F:GO:0003700; C:GO:0005737;	C:transcription factor complex; C:nucleus; F:transcription factor activity, sequence-specific DNA binding; C:cytoplasm; P:regulation of transcription, DNA-templated; F:protein dimerization activity		
Acrdi.XM_015911096.1/1-633 | Monca.augustus.g55126.t1/1-648	0.159891	0.141698	LDLR chaperone MESD	4	P:GO:0034394; P:GO:0050750; P:GO:0006457; C:GO:0005886	P:protein localization to cell surface; P:low-density lipoprotein particle receptor binding; P:protein folding; C:plasma membrane		
Acrdi.XM_015908845.1/1-579 | Monca.augustus.g26490.t1/1-564	0.203259	0.141681	FK506-binding 2	5	C:GO:0005789; F:GO:0003755; F:GO:0005528; P:GO:0061077;	C:endoplasmic reticulum membrane; F:peptidyl-prolyl cis-trans isomerase activity; F:FK506 binding; P:chaperone-mediated protein folding; P:protein peptidyl-prolyl isomeriz	EC:5.2.1.8	Peptidylprolyl isomerase
Acrdi.XM_015913776.1/1-324 | Monca.augustus.g45559.t1/1-342	0.270448	0.141657	glutamic acid-rich -like					
Acrdi.XM_015911234.1/1-669 | Monca.augustus.g13706.t1/1-789	0.1044	0.141654	N-lysine methyltransferase METTL21A	7	P:GO:0018022; C:GO:0016020; C:GO:0016021; P:GO:0006479;	P:peptidyl-lysine methylation; C:membrane; C:integral component of membrane; P:protein methylation; F:protein-lysine N-methyltransferase activity; F:Hsp70 protein binding; F:ATPase binding		
Acrdi.XM_015911613.1/1-348 | Monca.augustus.g54361.t1/1-348	0.241709	0.14163	histone deacetylase 6 isoform X1	4	F:GO:0008270; C:GO:0016020; C:GO:0016021; F:GO:0016787	F:zinc ion binding; C:membrane; C:integral component of membrane; F:hydrolase activity		
Acrdi.XM_015921571.1/1-318 | Monca.augustus.g3448.t1/1-435	0.139651	0.141598	Disco-interacting 2	2	F:GO:0003824; P:GO:0008152	F:catalytic activity; P:metabolic process		
Acrdi.XM_015899265.1/1-1230 | Monca.adi2mcaRNA8323_R0/1-1131	0.142009	0.14156	alpha-N-acetylgalactosaminidase-like	3	F:GO:0004557; P:GO:0016139; C:GO:0005737	F:alpha-galactosidase activity; P:glycoside catabolic process; C:cytoplasm	EC:3.2.1.22	Alpha-galactosidase
Acrdi.XM_015896377.1/1-1242 | Monca.augustus.g54400.t1/1-2658	0.16851	0.14155	PREDICTED: uncharacterized protein LOC107331750					
Acrdi.XM_015911947.1/1-462 | Monca.augustus.g30046.t1/1-459	0.13698	0.14155	succinate dehydrogenase assembly factor mitochondrial-like	3	P:GO:0018293; C:GO:0005759; P:GO:0006121	P:protein-FAD linkage; C:mitochondrial matrix; P:mitochondrial electron transport, succinate to ubiquinone		
Acrdi.XM_015922106.1/1-1191 | Monca.augustus.g21730.t1/1-1179	0.16005	0.141548	CUE domain-containing 1					
Acrdi.XM_015909427.1/1-1119 | Monca.augustus.g26347.t1/1-1821	0.170569	0.141542	solute carrier family 35 member F1	1	C:GO:0016020	C:membrane		
Acrdi.XM_015908483.1/1-537 | Monca.augustus.g19172.t1/1-534	0.126691	0.14154	JTB isoform X1					
Acrdi.XM_015915889.1/1-546 | Monca.augustus.g39005.t1/1-546	0.120457	0.141508	Testis-expressed sequence 36					
Acrdi.XM_015911794.1/1-1248 | Monca.adi2mcaRNA54_R0/1-1143	0.189896	0.14148	beta-glucuronidase-like	2	C:GO:0043227; F:GO:0016798	C:membrane-bounded organelle; F:hydrolase activity, acting on glycosyl bonds		
Acrdi.XM_015924822.1/1-441 | Monca.augustus.g67357.t1/1-477	0.130287	0.14144	PREDICTED: uncharacterized protein LOC107358200					
Acrdi.XM_015901615.1/1-960 | Monca.adi2mcaRNA10923_R0/1-966	0.220237	0.141436	pleckstrin-2	2	P:GO:0035556; C:GO:0005622	P:intracellular signal transduction; C:intracellular		
Acrdi.XM_015915741.1/1-2187 | Monca.adi2mcaRNA26447_R0/1-2205	0.151064	0.141388	nose resistant to fluoxetine 6-like	3	F:GO:0016747; C:GO:0016020; C:GO:0016021	F:transferase activity, transferring acyl groups other than amino-acyl groups; C:membrane; C:integral component of membrane		
Acrdi.XM_015912828.1/1-2640 | Monca.augustus.g60904.t1/1-2847	0.157883	0.141384	patched domain-containing 3-like isoform X1	1	C:GO:0016020	C:membrane		
Acrdi.XM_015905871.1/1-1293 | Monca.adi2mcaRNA15560_R0/1-1296	0.13719	0.141375	Neuropeptide FF receptor 2	3	P:GO:0007165; C:GO:0016020; F:GO:0004871	P:signal transduction; C:membrane; F:signal transducer activity		
Acrdi.XM_015920283.1/1-543 | Monca.augustus.g39628.t1/1-750	0.147776	0.141314	integrin alpha-D-like isoform X2					
Acrdi.XM_015894979.1/1-468 | Monca.augustus.g11961.t1/1-1098	0.153125	0.141305	PREDICTED: uncharacterized protein LOC107330355					
Acrdi.XM_015914231.1/1-1425 | Monca.adi2mcaRNA24763_R0/1-1368	0.192941	0.141299	dnaJ homolog subfamily A member mitochondrial isoform X2	5	F:GO:0005515; C:GO:0005739; F:GO:0043167; P:GO:0042981;	F:protein binding; C:mitochondrion; F:ion binding; P:regulation of apoptotic process; P:mitochondrion organization		
Acrdi.XM_015916979.1/1-2349 | Monca.augustus.g26570.t1/1-5178	0.137403	0.141295	beta-hexosaminidase-like isoform X3	3	F:GO:0004553; F:GO:0030246; P:GO:0071704	F:hydrolase activity, hydrolyzing O-glycosyl compounds; F:carbohydrate binding; P:organic substance metabolic process		
Acrdi.XM_015908352.1/1-2307 | Monca.augustus.g36810.t1/1-4743	0.176084	0.141279	nidogen-2 isoform X2					
Acrdi.XM_015909468.1/1-1251 | Monca.augustus.g35553.t1/1-1254	0.15435	0.141223	sorting nexin-16 isoform X1	7	C:GO:0005769; P:GO:0045022; C:GO:0005770; F:GO:0035091;	C:early endosome; P:early endosome to late endosome transport; C:late endosome; F:phosphatidylinositol binding; P:endosome to lysosome transport; P:protein targeting to lysosome; C:extrinsic component of endosome membrane		
Acrdi.XM_015914763.1/1-2712 | Monca.adi2mcaRNA25374_R1/1-2736	0.199311	0.141141	Spermatogenesis-associated 5	1	F:GO:0005524	F:ATP binding		
Acrdi.XM_015902126.1/1-789 | Monca.adi2mcaRNA11436_R1/1-789	0.162005	0.141122	Lipoxygenase-likey domain-containing 1	2	F:GO:0070492; F:GO:0008289	F:oligosaccharide binding; F:lipid binding		
Acrdi.XM_015923374.1/1-948 | Monca.augustus.g24907.t1/1-927	0.214042	0.141089	heparan sulfate glucosamine 3-O-sulfotransferase 1-like	1	F:GO:0008146	F:sulfotransferase activity		
Acrdi.XM_015899567.1/1-2298 | Monca.adi2mcaRNA8658_R0/1-2166	0.152533	0.140965	probable G- coupled receptor CG31760	4	F:GO:0004930; P:GO:0007186; C:GO:0016020; C:GO:0016021	F:G-protein coupled receptor activity; P:G-protein coupled receptor signaling pathway; C:membrane; C:integral component of membrane		
Acrdi.XM_015919946.1/1-9750 | Monca.augustus.g354.t1/1-10263	0.180883	0.140954	E3 ubiquitin- ligase RNF213	5	F:GO:0016887; F:GO:0004842; F:GO:0008270; F:GO:0046872;	F:ATPase activity; F:ubiquitin-protein transferase activity; F:zinc ion binding; F:metal ion binding; P:protein ubiquitination		
Acrdi.XM_015920458.1/1-1971 | Monca.augustus.g23827.t1/1-3930	0.158505	0.140954	myb V					
Acrdi.XM_015895511.1/1-771 | Monca.augustus.g32981.t1/1-765	0.180571	0.140877	noggin-2-like	8	P:GO:0030514; C:GO:0005615; P:GO:0001501; P:GO:0045596;	P:negative regulation of BMP signaling pathway; C:extracellular space; P:skeletal system development; P:negative regulation of cell differentiation; C:membrane; P:osteoblast differentiation; C:integral component of membrane; P:nervous system development		
Acrdi.XM_015908492.1/1-1149 | Monca.augustus.g15579.t1/1-1077	0.118245	0.140871	beta-1,3-galactosyltransferase 5-like	2	C:GO:0016020; F:GO:0016740	C:membrane; F:transferase activity		
Acrdi.XM_015919687.1/1-1656 | Monca.adi2mcaRNA30890_R0/1-1755	0.151793	0.140868	Angiogenic factor with G patch and FHA domains 1	1	F:GO:0003676	F:nucleic acid binding		
Acrdi.XM_015899962.1/1-1641 | Monca.augustus.g44149.t1/1-1785	0.163197	0.140841	histone-lysine N-methyltransferase setd3	4	C:GO:0005634; P:GO:0034968; P:GO:0045893; F:GO:0018024	C:nucleus; P:histone lysine methylation; P:positive regulation of transcription, DNA-templated; F:histone-lysine N-methyltransferase activity	EC:2.1.1.43	Histone-lysine N-methyltransferase
Acrdi.XM_015906882.1/1-1191 | Monca.augustus.g32950.t1/1-1176	0.243505	0.140781	G -activated inward rectifier potassium channel 4	3	C:GO:0005887; F:GO:0005242; P:GO:0010107	C:integral component of plasma membrane; F:inward rectifier potassium channel activity; P:potassium ion import		
Acrdi.XM_015892654.1/1-3480 | Monca.adi2mcaRNA38625_R0/1-3468	0.179406	0.140781	CD209 antigen E					
Acrdi.XM_015922981.1/1-660 | Monca.augustus.g67841.t1/1-1029	0.139319	0.140751	Bardet-Biedl syndrome 5 isoform X2	4	C:GO:0034464; C:GO:0036064; P:GO:0046907; F:GO:0032266	C:BBSome; C:ciliary basal body; P:intracellular transport; F:phosphatidylinositol-3-phosphate binding		
Acrdi.XM_015921219.1/1-1929 | Monca.augustus.g67777.t1/1-2598	0.0944687	0.140709	piwi 1	13	F:GO:0019901; C:GO:0033391; F:GO:0034584; P:GO:0007286;	F:protein kinase binding; C:chromatoid body; F:piRNA binding; P:spermatid development; P:pachytene; C:polysome; P:regulation of translation; C:nucleus; C:mRNA cap binding complex; C:dense body; F:single-stranded RNA binding; F:mRNA binding; P:piRNA metabolic process		
Acrdi.XM_015907005.1/1-1263 | Monca.augustus.g33398.t1/1-1263	0.126867	0.140674	Transcription factor SOX-30					
Acrdi.XM_015893378.1/1-1005 | Monca.augustus.g50891.t1/1-888	0.157129	0.140662	S-formylglutathione hydrolase isoform X1	1	F:GO:0016788	F:hydrolase activity, acting on ester bonds		
Acrdi.XM_015898443.1/1-1920 | Monca.adi2mcaRNA7472_R0/1-1803	0.139654	0.140647	neuroblastoma-amplified sequence					
Acrdi.XM_015899102.1/1-2070 | Monca.augustus.g52435.t1/1-2073	0.161911	0.140623	Mannan-binding lectin serine protease	1	F:GO:0008233	F:peptidase activity		
Acrdi.XM_015904219.1/1-1023 | Monca.augustus.g48220.t1/1-1032	0.190216	0.140622	carbonic anhydrase 2	4	F:GO:0008270; F:GO:0046872; F:GO:0004089; P:GO:0006730	F:zinc ion binding; F:metal ion binding; F:carbonate dehydratase activity; P:one-carbon metabolic process		
Acrdi.XM_015924668.1/1-1248 | Monca.augustus.g28220.t1/1-1257	0.180145	0.14061	sonic hedgehog	34	P:GO:0061075; P:GO:0009952; P:GO:0048877; P:GO:0021513;	P:positive regulation of neural retina development; P:anterior/posterior pattern specification; P:homeostasis of number of retina cells; P:spinal cord dorsal/ventral patterning; P:growth plate cartilage chondrocyte growth; P:positive regulation of osteoblast differentiation; P:regulation of apoptosis involved in tissue homeostasis		
Acrdi.XM_015902204.1/1-1086 | Monca.augustus.g25702.t1/1-1095	0.137922	0.140601	5-hydroxytryptamine receptor 4-like	4	C:GO:0005887; F:GO:0008528; P:GO:0007218; P:GO:0007200	C:integral component of plasma membrane; F:G-protein coupled peptide receptor activity; P:neuropeptide signaling pathway; P:phospholipase C-activating G-protein coupled receptor signaling pathway		
Acrdi.XM_015915171.1/1-1041 | Monca.adi2mcaRNA25849_R0/1-1029	0.122026	0.140598	band 2 isoform X1	4	C:GO:0005856; C:GO:0005737; C:GO:0019898; F:GO:0008092	C:cytoskeleton; C:cytoplasm; C:extrinsic component of membrane; F:cytoskeletal protein binding		
Acrdi.XM_015919577.1/1-1911 | Monca.augustus.g52106.t1/1-1920	0.180949	0.14058	Alpha-L-iduronidase	3	F:GO:0004553; P:GO:0044238; P:GO:0071704	F:hydrolase activity, hydrolyzing O-glycosyl compounds; P:primary metabolic process; P:organic substance metabolic process		
Acrdi.XM_015919560.1/1-1035 | Monca.augustus.g29520.t1/1-324	0.139374	0.14058	nudC domain-containing 3	4	C:GO:0005737; P:GO:0007275; P:GO:0051082; P:GO:0006457	C:cytoplasm; P:multicellular organism development; F:unfolded protein binding; P:protein folding		
Acrdi.XM_015903866.1/1-624 | Monca.augustus.g17716.t1/1-1413	0.242096	0.140543	nardilysin isoform X4	3	F:GO:0008270; P:GO:0016485; F:GO:0004222	F:zinc ion binding; P:protein processing; F:metalloendopeptidase activity	EC:3.4.24	Acting on peptide bonds (peptidases)
Acrdi.XM_015917332.1/1-2619 | Monca.augustus.g25539.t1/1-2430	0.166517	0.140533	Fibroblast growth factor receptor 3	3	F:GO:0005524; P:GO:0018108; F:GO:0004713	F:ATP binding; P:peptidyl-tyrosine phosphorylation; F:protein tyrosine kinase activity	EC:2.7.10	Transferring phosphorus-containing groups
Acrdi.XM_015920955.1/1-2436 | Monca.augustus.g30435.t1/1-2775	0.0561954	0.14053	kinesin KIFC3 isoform X3	7	F:GO:0016887; F:GO:0005524; F:GO:0003777; C:GO:0005871;	F:ATPase activity; F:ATP binding; F:microtubule motor activity; C:kinesin complex; P:microtubule-based movement; C:microtubule; F:microtubule binding	EC:3.6.1.3; EC:3.6.1.15	Adenosinetriphosphatase; Nucleoside-triphosphate phosphatase
Acrdi.XM_015916063.1/1-972 | Monca.augustus.g5225.t1/1-984	0.12863	0.140513	UPF0704 C6orf165 homolog					
Acrdi.XM_015898309.1/1-1038 | Monca.augustus.g37935.t1/1-1029	0.206986	0.140507	probable aminopyrimidine mitochondrial isoform X2	3	C:GO:0016020; C:GO:0016021; C:GO:0005829	C:membrane; C:integral component of membrane; C:cytosol		
Acrdi.XM_015910316.1/1-1719 | Monca.augustus.g36442.t1/1-1836	0.176265	0.140473	long-chain fatty acid transport 4-like	1	C:GO:0016020	C:membrane		
Acrdi.XM_015904955.1/1-3066 | Monca.augustus.g46470.t1/1-3057	0.107158	0.14046	helicase MOV-10	2	F:GO:0000166; F:GO:0005524	F:nucleotide binding; F:ATP binding		
Acrdi.XM_015909028.1/1-771 | Monca.augustus.g13635.t1/1-1116	0.149231	0.140457	substance-K receptor-like	3	F:GO:0004930; P:GO:0007186; C:GO:0016020	F:G-protein coupled receptor activity; P:G-protein coupled receptor signaling pathway; C:membrane		
Acrdi.XM_015898008.1/1-870 | Monca.adi2mcaRNA6978_R0/1-885	0.466078	0.140424	PREDICTED: uncharacterized protein LOC107333232					
Acrdi.XM_015892434.1/1-840 | Monca.augustus.g69054.t1/1-1062	0.151655	0.140372	nucleoporin seh1 isoform X2	7	C:GO:0005634; P:GO:0051234; P:GO:0051641; P:GO:0007059;	C:nucleus; P:establishment of localization; P:cellular localization; P:chromosome segregation; P:mitotic nuclear division; C:protein complex; C:chromosome, centromeric region		
Acrdi.XM_015893961.1/1-1470 | Monca.augustus.g41189.t1/1-3624	0.107166	0.140369	PREDICTED: uncharacterized protein LOC107329229	4	P:GO:0042273; C:GO:0016020; C:GO:0016021; P:GO:0006397	P:ribosomal large subunit biogenesis; C:membrane; C:integral component of membrane; P:mRNA processing		
Acrdi.XM_015913531.1/1-672 | Monca.augustus.g22327.t1/1-738	0.212781	0.140314	Zinc finger 511	1	F:GO:0046872	F:metal ion binding		
Acrdi.XM_015919867.1/1-1092 | Monca.augustus.g33624.t1/1-1056	0.134075	0.14029	dimethyladenosine transferase mitochondrial	4	P:GO:0000154; C:GO:0005759; F:GO:0000179; P:GO:0032259	P:rRNA modification; C:mitochondrial matrix; F:rRNA (adenine-N6,N6-)-dimethyltransferase activity; P:methylation		

Acrdi.XM_015893662.1/1-1464 | Monca.augustus.g43284.t1/1-2277	0.199254	0.140275	Pyridoxal-dependent decarboxylase domain-containing	1	F:GO:0003824	F:catalytic activity	
Acrdi.XM_015910836.1/1-732 | Monca.augustus.g57306.t1/1-1173	0.130185	0.140261	gelsolin 2	1	F:GO:0003779	F:actin binding	
Acrdi.XM_015906479.1/1-1971 | Monca.augustus.g22553.t1/1-1893	0.166428	0.140236	Mediator of RNA polymerase II transcription subunit 17	3	C:GO:0016592; F:GO:0001104; P:GO:0006357	C:mediator complex; F:RNA polymerase II transcription cofactor activity; P:regulation of transcription from RNA polymerase II promoter	
Acrdi.XM_015901743.1/1-981 | Monca.augustus.g49663.t1/1-990	0.166008	0.140221	histamine H2 receptor-like	3	P:GO:0007154; P:GO:0044700; F:GO:0004871	P:cell communication; P:single organism signaling; F:signal transducer activity	
Acrdi.XM_015899868.1/1-414 | Monca.augustus.g48630.t1/1-2538	0.186867	0.140218	BTB and MATH domain-containing 42-like	3	F:GO:0004842; C:GO:0031463; P:GO:0016567	F:ubiquitin-protein transferase activity; C:Cul3-RING ubiquitin ligase complex; P:protein ubiquitination	
Acrdi.XM_015905269.1/1-597 | Monca.augustus.g23130.t1/1-1959	0.183846	0.140217	Serine threonine- phosphatase	5	F:GO:0046914; C:GO:0016020; C:GO:0043025; C:GO:0042995;	F:transition metal ion binding; C:membrane; C:neuronal cell body; C:cell projection; F:hydrolase activity	
Acrdi.XM_015920019.1/1-1194 | Monca.adi2mcaRNA3211_R1/1-981	0.137205	0.140212	FAS-associated factor 2				
Acrdi.XM_015895559.1/1-594 | Monca.augustus.g14888.t1/1-1896	0.171503	0.140198	Switch-associated 70	5	P:GO:0007096; P:GO:0060026; P:GO:0060027; P:GO:0060028;	P:regulation of exit from mitosis; P:convergent extension; P:convergent extension involved in gastrulation; P:convergent extension involved in axis elongation; P:positive regulation of oligodendrocyte differentiation	
Acrdi.XM_015892742.1/1-1974 | Monca.augustus.g22625.t1/1-2175	0.178578	0.14018	Golgin subfamily A member 5	2	C:GO:0016020; C:GO:0005794	C:membrane; C:Golgi apparatus	
Acrdi.XM_015901444.1/1-636 | Monca.augustus.g46706.t1/1-669	0.174192	0.140171	regulation of enolase 1-like				
Acrdi.XM_015919372.1/1-4191 | Monca.adi2mcaRNA3062_R0/1-4374	0.170727	0.140165	ribosome-binding 1-like isoform X2	2	F:GO:0005509; F:GO:0005544	F:calcium ion binding; F:calcium-dependent phospholipid binding	
Acrdi.XM_015913591.1/1-1812 | Monca.adi2mcaRNA24060_R1/1-1932	0.161599	0.140163	A disintegrin and metallo ase with thrombospondin motifs 18				
Acrdi.XM_015892987.1/1-816 | Monca.augustus.g25660.t1/1-840	0.182976	0.140144	L-aspartate dehydrogenase	1	P:GO:0019362	P:pyridine nucleotide metabolic process	
Acrdi.XM_015909199.1/1-504 | Monca.augustus.g44601.t1/1-699	0.11215	0.140106	Ubiquitin-conjugating enzyme E2 J2	6	F:GO:0061630; F:GO:0031625; P:GO:0000209; C:GO:0016021;	F:ubiquitin protein ligase activity; F:ubiquitin protein ligase binding; P:protein polyubiquitination; C:integral component of membrane; C:endoplasmic reticulum; P:ER-associated ubiquitin-dependent protein catabolic [illegible]	
Acrdi.XM_015916484.1/1-732 | Monca.augustus.g9026.t1/1-732	0.174435	0.140097	Complex I assembly factor mitochondrial	3	C:GO:0005739; C:GO:0016020; C:GO:0044446	C:mitochondrion; C:membrane; C:intracellular organelle part	
Acrdi.XM_015904175.1/1-2781 | Monca.augustus.g61671.t1/1-4614	0.173957	0.14006	E3 ubiquitin- ligase RNF123				
Acrdi.XM_015900335.1/1-1956 | Monca.adi2mcaRNA9602_R1/1-1038	0.107403	0.140056	cardioacceleratory peptide receptor-like	3	F:GO:0004930; P:GO:0007165; C:GO:0016020	F:G-protein coupled receptor activity; P:signal transduction; C:membrane	
Acrdi.XM_015922458.1/1-846 | Monca.augustus.g40389.t1/1-849	0.217446	0.14005	PREDICTED: uncharacterized protein LOC107355837				
Acrdi.XM_015916694.1/1-1767 | Monca.augustus.g48819.t1/1-2427	0.10198	0.140036	Cell division cycle 5	26	P:GO:0006281; P:GO:0008380; C:GO:0016607; P:GO:1990090;	P:DNA repair; P:RNA splicing; C:nuclear speck; P:cellular response to nerve growth factor stimulus; F:transcription regulatory region DNA binding; F:sequence-specific DNA binding; C:Prp19 complex; C:nucleolus; [illegible]	
Acrdi.XM_015915174.1/1-897 | Monca.augustus.g48124.t1/1-2202	0.129573	0.140025	Potassium voltage-gated channel subfamily F member 1	2	F:GO:0005216; C:GO:0016021	F:ion channel activity; C:integral component of membrane	
Acrdi.XM_015898203.1/1-789 | Monca.augustus.g39420.t1/1-792	0.181526	0.140023	chromosome partition Smc-like				
Acrdi.XM_015909280.1/1-1428 | Monca.augustus.g61670.t1/1-1335	0.127151	0.140019	Coiled-coil domain-containing 130	1	P:GO:0009615	P:response to virus	
Acrdi.XM_015920082.1/1-1233 | Monca.augustus.g28556.t1/1-1464	0.148691	0.14001	histone-lysine N-methyltransferase SUV420H1	4	C:GO:0043229; P:GO:0040014; P:GO:0034773; F:GO:0042799	C:intracellular organelle; P:regulation of multicellular organism growth; P:histone H4-K20 trimethylation; F:histone methyltransferase activity (H4-K20 specific)	EC:2.1.1.43
Acrdi.XM_015924511.1/1-723 | Monca.augustus.g28538.t1/1-717	0.18267	0.140008	ER membrane complex subunit 10-like	1	C:GO:0016020	C:membrane	
Acrdi.XM_015910645.1/1-654 | Monca.augustus.g19025.t1/1-1227	0.129094	0.139984	TNF receptor-associated factor 4-like	3	F:GO:0004842; F:GO:0008270; P:GO:0016567	F:ubiquitin-protein transferase activity; F:zinc ion binding; P:protein ubiquitination	
Acrdi.XM_015914527.1/1-708 | Monca.augustus.g29141.t1/1-1782	0.124804	0.139961	PREDICTED: uncharacterized protein LOC107348486				
Acrdi.XM_015903386.1/1-1632 | Monca.adi2mcaRNA12756_R0/1-1587	0.159321	0.139938	Glutamine-rich 2	2	C:GO:0016020; C:GO:0016021	C:membrane; C:integral component of membrane	
Acrdi.XM_015908454.1/1-1554 | Monca.augustus.g33938.t1/1-1521	0.175225	0.139903	Lariat debranching enzyme	4	P:GO:0090502; C:GO:0005634; F:GO:0008419; P:GO:0000398	P:RNA phosphodiester bond hydrolysis, endonucleolytic; C:nucleus; F:RNA lariat debranching enzyme activity; P:mRNA splicing, via spliceosome	EC:3.1.30; EC:3.1.26
Acrdi.XM_015921657.1/1-939 | Monca.augustus.g47370.t1/1-870	0.160594	0.139879	neuferricin isoform X1	3	C:GO:0016020; C:GO:0012505; F:GO:0020037	C:membrane; C:endomembrane system; F:heme binding	
Acrdi.XM_015922553.1/1-1299 | Monca.augustus.g19801.t1/1-1992	0.147033	0.139876	Polycomb Suz12	1	F:GO:0046872	F:metal ion binding	
Acrdi.XM_015907327.1/1-1236 | Monca.augustus.g31777.t1/1-1116	0.156582	0.139865	egl nine homolog 1-like isoform X1	2	F:GO:0016491; F:GO:0046872	F:oxidoreductase activity; F:metal ion binding	
Acrdi.XM_015897978.1/1-873 | Monca.adi2mcaRNA6989_R0/1-780	0.781436	0.139856	ac transposable element-derived 4				
Acrdi.XM_015901065.1/1-1389 | Monca.augustus.g57631.t1/1-1356	0.105722	0.13985	chloroplast stem-loop binding of 41 kDa chloroplastic-like	4	F:GO:0050662; F:GO:0003824; C:GO:0016020; C:GO:0016021	F:coenzyme binding; F:catalytic activity; C:membrane; C:integral component of membrane	
Acrdi.XM_015904064.1/1-1662 | Monca.augustus.g44547.t1/1-1899	0.198938	0.139844	leukocyte receptor cluster member 8	1	F:GO:0005515	F:protein binding	
Acrdi.XM_015909152.1/1-2229 | Monca.augustus.g63671.t1/1-2961	0.126135	0.139837	type II inositol 3,4-bisphosphate 4-phosphatase	4	F:GO:0016316; C:GO:0005737; P:GO:0007165; P:GO:0036092	F:phosphatidylinositol-3,4-bisphosphate 4-phosphatase activity; C:cytoplasm; P:signal transduction; P:phosphatidylinositol-3-phosphate biosynthetic process	EC:3.1.3.66; EC:3.1.3.41
Acrdi.XM_015923262.1/1-1524 | Monca.augustus.g55723.t1/1-1899	0.224779	0.139818	copine-1 isoform X2				
Acrdi.XM_015922863.1/1-1212 | Monca.augustus.g53606.t1/1-1215	0.242317	0.139792	4-hydroxybenzoate mitochondrial	5	C:GO:0031305; F:GO:0002083; P:GO:0008299; F:GO:0047293;	C:integral component of mitochondrial inner membrane; F:4-hydroxybenzoate decaprenyltransferase activity; P:isoprenoid biosynthetic process; F:4-hydroxybenzoate nonapr	EC:2.5.1.39
Acrdi.XM_015924659.1/1-675 | Monca.augustus.g28211.t1/1-675	0.198055	0.139778	probable N-acetyltransferase camello	3	C:GO:0016020; C:GO:0016021; F:GO:0008080	C:membrane; C:integral component of membrane; F:N-acetyltransferase activity	
Acrdi.XM_015904257.1/1-522 | Monca.augustus.g62116.t1/1-516	0.114986	0.139755	PREDICTED: uncharacterized protein LOC107339015				
Acrdi.XM_015910510.1/1-615 | Monca.augustus.g25430.t1/1-6654	0.198224	0.139736	FAM208A isoform X1	1	F:GO:0003950	F:NAD+ ADP-ribosyltransferase activity	
Acrdi.XM_015901036.1/1-468 | Monca.augustus.g39844.t1/1-474	0.159856	0.139721	lipopolysaccharide-induced tumor necrosis factor-alpha factor				
Acrdi.XM_015918021.1/1-3297 | Monca.augustus.g57099.t1/1-2382	0.114695	0.139714	Rho GTPase-activating 18	1	P:GO:0050794	P:regulation of cellular process	
Acrdi.XM_015906171.1/1-963 | Monca.augustus.g21685.t1/1-1317	0.117506	0.139712	Transcriptional adapter 2-alpha	2	C:GO:0043229; P:GO:0031399	C:intracellular organelle; P:regulation of protein modification process	
Acrdi.XM_015897671.1/1-894 | Monca.adi2mcaRNA6696_R0/1-630	0.146476	0.139638	peroxiredoxin 6	4	F:GO:0004601; P:GO:0098869; P:GO:0055114; F:GO:0051920	F:peroxidase activity; P:cellular oxidant detoxification; P:oxidation-reduction process; F:peroxiredoxin activity	EC:1.11.1.15; EC:1.11.1.7
Acrdi.XM_015896245.1/1-2460 | Monca.augustus.g33756.t1/1-2457	0.190167	0.139633	radial spoke head 10 homolog B-like				
Acrdi.XM_015899889.1/1-3591 | Monca.augustus.g37401.t1/1-4215	0.152256	0.139627	cat eye syndrome critical region 2-like isoform X2				
Acrdi.XM_015923423.1/1-537 | Monca.augustus.g30579.t1/1-570	0.231553	0.139609	PREDICTED: uncharacterized protein LOC107356799				
Acrdi.XM_015913274.1/1-999 | Monca.augustus.g61316.t1/1-1062	0.180082	0.139609	neuropeptide FF receptor 2-like	2	P:GO:0007165; C:GO:0016020	P:signal transduction; C:membrane	
Acrdi.XM_015899041.1/1-699 | Monca.adi2mcaRNA8104_R1/1-600	0.550079	0.139608	PREDICTED: uncharacterized protein LOC107334138				
Acrdi.XM_015898751.1/1-675 | Monca.augustus.g2078.t1/1-678	0.162474	0.139597	Glutathione S-transferase kappa 1	2	F:GO:0015035; P:GO:0055114	F:protein disulfide oxidoreductase activity; P:oxidation-reduction process	
Acrdi.XM_015905663.1/1-972 | Monca.augustus.g1923.t1/1-1167	0.14048	0.139566	NSFL1 cofactor p47 isoform X2	7	C:GO:0005634; P:GO:0043161; P:GO:0061025; F:GO:0043130;	C:nucleus; P:proteasome-mediated ubiquitin-dependent protein catabolic process; P:membrane fusion; F:ubiquitin binding; P:Golgi organization; C:cytosol; P:nuclear envelope reassembly	
Acrdi.XM_015907760.1/1-5811 | Monca.augustus.g62585.t1/1-6489	0.165628	0.139552	peripheral-type benzodiazepine receptor-associated 1-like				
Acrdi.XM_015892401.1/1-975 | Monca.augustus.g67128.t1/1-1107	0.108241	0.139525	D-threo-3-hydroxyaspartate dehydratase-like				
Acrdi.XM_015905622.1/1-1023 | Monca.augustus.g40457.t1/1-1044	0.168826	0.13952	NFATC2-interacting -like	6	C:GO:0005634; C:GO:0005737; P:GO:0016925; P:GO:0001816;	C:nucleus; C:cytoplasm; P:protein sumoylation; P:cytokine production; F:protein tag; P:positive regulation of transcription from RNA polymerase II promoter	
Acrdi.XM_015905795.1/1-387 | Monca.augustus.g31748.t1/1-900	0.131219	0.139489	syntaxin-17-like	12	P:GO:0006906; P:GO:0007033; C:GO:0043231; P:GO:0048278;	P:vesicle fusion; P:vacuole organization; C:intracellular membrane-bounded organelle; P:vesicle docking; C:integral component of membrane; F:SNAP receptor activity; C:endomembrane system; C:SNARE complex; [illegible]	
Acrdi.XM_015915961.1/1-1578 | Monca.augustus.g60997.t1/1-1605	0.140569	0.139457	matrix metallo ase-24-like	1	F:GO:0016787	F:hydrolase activity	
Acrdi.XM_015893042.1/1-783 | Monca.augustus.g67613.t1/1-501	0.576272	0.139451	retrovirus-related Pol poly from transposon isoform X1				
Acrdi.XM_015907598.1/1-8979 | Monca.augustus.g50783.t1/1-10095	0.162514	0.139441	Polycystic kidney disease and receptor for egg jelly-related	6	P:GO:0050982; F:GO:0005509; F:GO:0005262; C:GO:0016020;	P:detection of mechanical stimulus; F:calcium ion binding; F:calcium channel activity; C:membrane; C:integral component of membrane; P:calcium ion transmembrane transport	
Acrdi.XM_015920950.1/1-906 | Monca.augustus.g53584.t1/1-843	0.124764	0.139421	high choriolytic enzyme 1-like	2	F:GO:0046872; F:GO:0008237	F:metal ion binding; F:metallopeptidase activity	
Acrdi.XM_015906429.1/1-1662 | Monca.augustus.g52873.t1/1-2088	0.142426	0.13941	histone acetyltransferase KAT7-like isoform X1	7	C:GO:0005634; F:GO:0004402; F:GO:0003700; F:GO:0008270;	C:nucleus; F:histone acetyltransferase activity; F:transcription factor activity, sequence-specific DNA binding; F:zinc ion binding; F:transferase activity, transferring acyl groups other than amino-acyl groups; [illegible]	
Acrdi.XM_015898291.1/1-405 | Monca.augustus.g41852.t1/1-405	0.281939	0.139401	PREDICTED: uncharacterized protein LOC107333471				
Acrdi.XM_015891846.1/1-1302 | Monca.augustus.g43709.t1/1-1230	0.158227	0.139389	serine protease 23-like	1	F:GO:0016787	F:hydrolase activity	
Acrdi.XM_015917225.1/1-3483 | Monca.augustus.g55548.t1/1-3603	0.158197	0.139384	transient receptor potential cation channel subfamily A member 1-like				
Acrdi.XM_015912972.1/1-585 | Monca.augustus.g51633.t1/1-933	0.206365	0.139376	WD repeat-containing 5				
Acrdi.XM_015920423.1/1-2178 | Monca.augustus.g46971.t1/1-2106	0.124624	0.139351	long-chain-fatty-acid-- ligase 1 isoform X1	3	F:GO:0003824; P:GO:0008152; P:GO:0044699	F:catalytic activity; P:metabolic process; P:single-organism process	
Acrdi.XM_015912190.1/1-1647 | Monca.adi2mcaRNA22536_R0/1-1635	0.191368	0.139288	MAM and LDL-receptor class A domain-containing 2				
Acrdi.XM_015913863.1/1-1209 | Monca.augustus.g70078.t1/1-1419	0.157586	0.139282	serine incorporator 1	2	C:GO:0016020; P:GO:0006629	C:membrane; P:lipid metabolic process	
Acrdi.XM_015904467.1/1-4614 | Monca.adi2mcaRNA14029_R0/1-3819	0.173353	0.139227	Rho GTPase-activating 20	5	P:GO:0035023; P:GO:0007165; F:GO:0005096; F:GO:0005089;	P:regulation of Rho protein signal transduction; P:signal transduction; F:GTPase activator activity; F:Rho guanyl-nucleotide exchange factor activity; P:positive regulation of GTPase activity	
Acrdi.XM_015915275.1/1-1755 | Monca.augustus.g38344.t1/1-1764	0.145395	0.139226	Acyloxyacyl hydrolase	1	F:GO:0016788	F:hydrolase activity, acting on ester bonds	
Acrdi.XM_015891778.1/1-1149 | Monca.augustus.g64463.t1/1-1431	0.164345	0.139221	Golgi-associated plant pathogenesis-related 1	1	C:GO:0005576	C:extracellular region	
Acrdi.XM_015916264.1/1-13578 | Monca.adi2mcaRNA27051_R0/1-10638	0.177012	0.139194	Fibrocystin-L				
Acrdi.XM_015918485.1/1-1509 | Monca.augustus.g20456.t1/1-1554	0.1423	0.139189	Neuronal acetylcholine receptor subunit alpha-7	3	P:GO:0034220; C:GO:0016021; F:GO:0005230	P:ion transmembrane transport; C:integral component of membrane; F:extracellular ligand-gated ion channel activity	
Acrdi.XM_015916700.1/1-1962 | Monca.augustus.g37104.t1/1-1374	0.105543	0.139172	glutaredoxin-1 isoform X2	4	C:GO:0005623; P:GO:0050794; P:GO:0044699; P:GO:0009987	C:cell; P:regulation of cellular process; P:single-organism process; P:cellular process	
Acrdi.XM_015920031.1/1-2529 | Monca.augustus.g11391.t1/1-2625	0.16527	0.139171	serine threonine- kinase greatwall	13	C:GO:0032154; P:GO:0007067; F:GO:0004674; P:GO:0006974;	C:cleavage furrow; P:mitotic nuclear division; F:protein serine/threonine kinase activity; P:cellular response to DNA damage stimulus; P:cell division; C:nucleus; F:ATP binding;	EC:2.7.11
Acrdi.XM_015904462.1/1-504 | Monca.augustus.g33602.t1/1-882	0.182662	0.139156	GTP-binding Rho1	4	P:GO:0050794; C:GO:0044464; P:GO:0044699; P:GO:0009987	P:regulation of cellular process; C:cell part; P:single-organism process; P:cellular process	
Acrdi.XM_015905384.1/1-498 | Monca.augustus.g20529.t1/1-498	0.798087	0.139154	mannose-binding	1	F:GO:0030246	F:carbohydrate binding	
Acrdi.XM_015903201.1/1-2169 | Monca.augustus.g32290.t1/1-2652	0.136197	0.139145	Disks large				
Acrdi.XM_015920411.1/1-4554 | Monca.augustus.g46007.t1/1-4548	0.150854	0.139085	Adhesion G- coupled receptor G4	6	F:GO:0004930; F:GO:0004888; P:GO:0007186; C:GO:0016020;	F:G-protein coupled receptor activity; F:transmembrane signaling receptor activity; P:G-protein coupled receptor signaling pathway; C:membrane; C:integral component of membrane; P:cell surface receptor signaling [illegible]	
Acrdi.XM_015897395.1/1-873 | Monca.augustus.g29306.t1/1-1122	0.231548	0.139081	G- coupled receptor moody				
Acrdi.XM_015901379.1/1-2577 | Monca.adi2mcaRNA10635_R0/1-2589	0.163567	0.139065	fibroblast growth factor receptor 1 isoform X1	4	F:GO:0000166; C:GO:0016020; F:GO:0004672; P:GO:0006468	F:nucleotide binding; C:membrane; F:protein kinase activity; P:protein phosphorylation	
Acrdi.XM_015907497.1/1-1356 | Monca.augustus.g55642.t1/1-1326	0.151984	0.139036	ankyrin repeat	1	P:GO:0009987	P:cellular process	
Acrdi.XM_015899875.1/1-1962 | Monca.augustus.g6183.t1/1-2955	0.160682	0.139029	G- coupled receptor GRL101-like	6	C:GO:0005887; F:GO:0008528; P:GO:0007189; P:GO:0007190;	C:integral component of plasma membrane; F:G-protein coupled peptide receptor activity; P:adenylate cyclase-activating G-protein coupled receptor signaling pathway; P:activation of adenylate cyclase activity; [illegible]	
Acrdi.XM_015905336.1/1-627 | Monca.augustus.g32876.t1/1-735	0.176102	0.139018	PREDICTED: uncharacterized protein C7orf62 homolog isoform X2	3	F:GO:0071949; F:GO:0009882; P:GO:0009785	F:FAD binding; F:blue light photoreceptor activity; P:blue light signaling pathway	
Acrdi.XM_015908813.1/1-747 | Monca.augustus.g4766.t1/1-744	0.172465	0.139003	tetraspanin-7	2	C:GO:0005887; P:GO:0007166	C:integral component of plasma membrane; P:cell surface receptor signaling pathway	
Acrdi.XM_015903043.1/1-1239 | Monca.adi2mcaRNA12434_R0/1-1167	0.169683	0.138986	ancient ubiquitous 1	1	C:GO:0016020	C:membrane	
Acrdi.XM_015901388.1/1-1023 | Monca.augustus.g70854.t1/1-1044	0.180302	0.138983	poly(ADP-ribose) glycohydrolase ARH3-like	1	F:GO:0016787	F:hydrolase activity	
Acrdi.XM_015922447.1/1-1725 | Monca.augustus.g40380.t1/1-2745	0.152801	0.138959	Inositol polyphosphate 5-phosphatase OCRL-1	3	P:GO:0007165; F:GO:0016787; P:GO:0046856	P:signal transduction; F:hydrolase activity; P:phosphatidylinositol dephosphorylation	
Acrdi.XM_015917865.1/1-912 | Monca.augustus.g25193.t1/1-1239	0.1701	0.138955	Origin recognition complex subunit 4	4	F:GO:0000166; F:GO:0003688; P:GO:0006270; C:GO:0005664	F:nucleotide binding; F:DNA replication origin binding; P:DNA replication initiation; C:nuclear origin of replication recognition complex	
Acrdi.XM_015901930.1/1-717 | Monca.augustus.g27158.t1/1-771	0.103636	0.138942	Interferon regulatory factor 2	1	F:GO:0003677	F:DNA binding	
Acrdi.XM_015900185.1/1-2427 | Monca.augustus.g62838.t1/1-3498	0.168706	0.13894	Transient receptor potential cation channel subfamily A member 1				
Acrdi.XM_015899695.1/1-1254 | Monca.augustus.g33788.t1/1-1407	0.165558	0.138903	SURP and G-patch domain-containing 1-like	1	F:GO:0003676	F:nucleic acid binding	
Acrdi.XM_015919992.1/1-510 | Monca.augustus.g14113.t1/1-501	0.122315	0.138888	probable calcium-binding CML15	2	P:GO:0007165; F:GO:0005509	P:signal transduction; F:calcium ion binding	
Acrdi.XM_015920623.1/1-966 | Monca.augustus.g36315.t1/1-1167	0.176482	0.138882	vacuolar -sorting-associated 36	1	C:GO:0005737	C:cytoplasm	
Acrdi.XM_015907843.1/1-1425 | Monca.augustus.g47378.t1/1-1440	0.122525	0.13886	neuronal acetylcholine receptor subunit alpha-10-like	5	P:GO:0006811; F:GO:0099600; C:GO:0016021; P:GO:0007268;	P:ion transport; F:transmembrane receptor activity; C:integral component of membrane; P:chemical synaptic transmission; C:plasma membrane part	
Acrdi.XM_015911799.1/1-2994 | Monca.adi2mcaRNA22076_R0/1-2970	0.169725	0.138848	golgin subfamily A member 2 isoform X2	1	C:GO:0005794	C:Golgi apparatus	
Acrdi.XM_015909745.1/1-1602 | Monca.augustus.g19977.t1/1-1242	0.172199	0.138818	PREDICTED: uncharacterized protein LOC107344131	5	F:GO:0003677; C:GO:0016020; C:GO:0016021; P:GO:0015074;	F:DNA binding; C:membrane; C:integral component of membrane; P:DNA integration; P:DNA recombination	
Acrdi.XM_015916832.1/1-2529 | Monca.adi2mcaRNA27676_R0/1-2484	0.156744	0.138816	PREDICTED: uncharacterized protein LOC107350589 isoform X2				
Acrdi.XM_015921108.1/1-483 | Monca.augustus.g48104.t1/1-768	0.230233	0.138773	Thioredoxin-related transmembrane 1	6	F:GO:0003756; C:GO:0016021; C:GO:0005783; P:GO:0045454;	F:protein disulfide isomerase activity; C:integral component of membrane; C:endoplasmic reticulum; P:cell redox homeostasis; P:protein folding; P:response to endoplasmic r	EC:5.3.4.1
Acrdi.XM_015900258.1/1-1827 | Monca.augustus.g65831.t1/1-1848	0.145966	0.138765	tetratricopeptide repeat 24-like				
Acrdi.XM_015916368.1/1-2514 | Monca.augustus.g52606.t1/1-1233	0.113506	0.138728	oxysterol-binding -related 1-like isoform X2	2	P:GO:0006869; P:GO:0006810	P:lipid transport; P:transport	
Acrdi.XM_015895229.1/1-1131 | Monca.augustus.g9733.t1/1-1152	0.715976	0.138694	glycerophosphoryl diester phosphodiesterase	5	P:GO:0006629; C:GO:0016020; C:GO:0016021; F:GO:0008081;	P:lipid metabolic process; C:membrane; C:integral component of membrane; F:phosphoric diester hydrolase activity; F:glycerophosphodiester phosphodiesterase activity	
Acrdi.XM_015904142.1/1-312 | Monca.augustus.g36710.t1/1-321	0.190225	0.13865	UPF0562 C7orf55 homolog				
Acrdi.XM_015904189.1/1-339 | Monca.augustus.g12081.t1/1-2889	0.118187	0.138635	General transcription factor 3C polypeptide 3	1	C:GO:0044428	C:nuclear part	
Acrdi.XM_015891847.1/1-1059 | Monca.augustus.g67156.t1/1-1173	0.163599	0.138591	prolyl 4-hydroxylase subunit alpha-1 isoform X2	9	F:GO:0031418; C:GO:0005788; F:GO:0016702; F:GO:0005506;	F:L-ascorbic acid binding; C:endoplasmic reticulum lumen; F:oxidoreductase activity, acting on single donors with incorporation of molecular oxygen, incorporation of two atoms of oxygen; F:iron ion binding; [illegible]	
Acrdi.XM_015913500.1/1-1284 | Monca.augustus.g19346.t1/1-1353	0.144433	0.138591	SUN domain-containing 2	3	C:GO:0005635; P:GO:0006998; C:GO:0016020	C:nuclear envelope; P:nuclear envelope organization; C:membrane	
Acrdi.XM_015901275.1/1-2307 | Monca.adi2mcaRNA10614_R0/1-1947	0.144301	0.138574	WD repeat-containing 91				
Acrdi.XM_015896636.1/1-2145 | Monca.augustus.g21906.t1/1-3027	0.174467	0.138538	Serine threonine- phosphatase 6 regulatory ankyrin repeat subunit A				
Acrdi.XM_015899935.1/1-834 | Monca.augustus.g32792.t1/1-840	0.135599	0.138498	cyclic AMP-responsive element-binding 3 2	11	F:GO:0001077; F:GO:0035497; P:GO:0006888; P:GO:0030968;	F:transcriptional activator activity, RNA polymerase II core promoter proximal region sequence-specific binding; F:cAMP response element binding; P:ER to Golgi vesicle-mediated transport; P:endoplasmic reticulum [illegible]	
Acrdi.XM_015905457.1/1-1095 | Monca.augustus.g28267.t1/1-1233	0.139229	0.138424	N-acyl-phosphatidylethanolamine-hydrolyzing phospholipase D isoform	4	F:GO:0008270; C:GO:0070062; C:GO:0005737; F:GO:0070290	F:zinc ion binding; C:extracellular exosome; C:cytoplasm; F:N-acylphosphatidylethanolamine-specific phospholipase D activity	
Acrdi.XM_015895612.1/1-1077 | Monca.augustus.g64087.t1/1-1473	0.180986	0.138422	Succinate-semialdehyde mitochondrial	5	F:GO:0004029; F:GO:0004777; F:GO:0009013; P:GO:0009450;	F:aldehyde dehydrogenase (NAD) activity; F:succinate-semialdehyde dehydrogenase (NAD+) activity; F:succinate-semialdehyde dehydrogenase [NAD(P)+] activity; P:gamma-[illegible]	EC:1.2.1.16; EC:1.2.1.24; EC:1.2.1.3
Acrdi.XM_015900792.1/1-18369 | Monca.adi2mcaRNA10073_R0/1-17583	0.157827	0.138414	protocadherin Fat 3	7	P:GO:0007275; F:GO:0005509; C:GO:0016020; C:GO:0016021;	P:multicellular organism development; F:calcium ion binding; C:membrane; C:integral component of membrane; P:cell adhesion; P:homophilic cell adhesion via plasma membrane adhesion molecules; C:plasma [illegible]	
Acrdi.XM_015918316.1/1-468 | Monca.augustus.g29392.t1/1-477	0.182835	0.138374	39S ribosomal mitochondrial	3	F:GO:0003735; C:GO:0005762; P:GO:0006412	F:structural constituent of ribosome; C:mitochondrial large ribosomal subunit; P:translation	
Acrdi.XM_015914440.1/1-6456 | Monca.augustus.g56326.t1/1-7899	0.202304	0.138331	leucine-rich repeat serine threonine- kinase 2-like	3	F:GO:0005524; F:GO:0004672; P:GO:0006468	F:ATP binding; F:protein kinase activity; P:protein phosphorylation	
Acrdi.XM_015924405.1/1-702 | Monca.augustus.g67821.t1/1-702	0.193797	0.138312	oxidoreductase	2	F:GO:0016491; P:GO:0055114	F:oxidoreductase activity; P:oxidation-reduction process	
Acrdi.XM_015893921.1/1-1038 | Monca.adi2mcaRNA40065_R1/1-1008	0.0912521	0.138245	neuronal acetylcholine receptor subunit alpha-7-like	2	C:GO:0016020; P:GO:0006810	C:membrane; P:transport	
Acrdi.XM_015899152.1/1-1260 | Monca.adi2mcaRNA8284_R1/1-1308	0.0568142	0.138235	piggyBac transposable element-derived 4-like				
Acrdi.XM_015896347.1/1-855 | Monca.augustus.g69705.t1/1-855	0.175172	0.138231	Heme oxygenase 2	5	F:GO:0004392; C:GO:0016020; C:GO:0016021; P:GO:0055114;	F:heme oxygenase (decyclizing) activity; C:membrane; C:integral component of membrane; P:oxidation-reduction process; P:heme oxidation	
Acrdi.XM_015913919.1/1-3324 | Monca.adi2mcaRNA24388_R0/1-3207	0.158037	0.138224	PREDICTED: uncharacterized protein LOC107347927				
Acrdi.XM_015904019.1/1-1440 | Monca.augustus.g2917.t1/1-1422	0.163039	0.138204	Alpha-1,6-mannosyl-glyco 2-beta-N-acetylglucosaminyltransferase				
Acrdi.XM_015896829.1/1-1617 | Monca.augustus.g33196.t1/1-1452	0.125527	0.13816	tumor p73-like isoform X1	7	F:GO:0003677; C:GO:0005634; F:GO:0003700; P:GO:0051262;	F:DNA binding; C:nucleus; F:transcription factor activity, sequence-specific DNA binding; P:protein tetramerization; P:apoptotic process; F:transcription regulatory region DNA binding; P:regulation of transcription, [illegible]	
Acrdi.XM_015904768.1/1-3036 | Monca.augustus.g31928.t1/1-3087	0.164845	0.13815	PREDICTED: uncharacterized protein LOC107339474	4	F:GO:0008270; P:GO:0006508; F:GO:0030246; F:GO:0004222	F:zinc ion binding; P:proteolysis; F:carbohydrate binding; F:metalloendopeptidase activity	
Acrdi.XM_015900450.1/1-600 | Monca.augustus.g36283.t1/1-1374	0.582737	0.138148	ATP-dependent DNA helicase PIF1	4	P:GO:0007165; C:GO:0005739; P:GO:0051607; F:GO:0004871	P:signal transduction; C:mitochondrion; P:defense response to virus; F:signal transducer activity	
Acrdi.XM_015917768.1/1-387 | Monca.adi2mcaRNA28746_R1/1-348	0.188597	0.138148	insulin-induced gene 2 -like	2	C:GO:0016020; P:GO:0006629	C:membrane; P:lipid metabolic process	
Acrdi.XM_015891742.1/1-2568 | Monca.augustus.g49608.t1/1-2568	0.204663	0.138115	Leucine-rich repeat and IQ domain-containing 3				
Acrdi.XM_015910782.1/1-708 | Monca.augustus.g16149.t1/1-699	0.147012	0.138018	haloacid dehalogenase-like hydrolase domain-containing 3	2	P:GO:0008152; F:GO:0016787	P:metabolic process; F:hydrolase activity	
Acrdi.XM_015918309.1/1-924 | Monca.adi2mcaRNA29322_R0/1-969	0.120741	0.138006	Glutaminyl-peptide cyclotransferase				
Acrdi.XM_015903401.1/1-966 | Monca.augustus.g36006.t1/1-978	0.169607	0.137978	hydrolethalus syndrome 1				
Acrdi.XM_015906363.1/1-423 | Monca.augustus.g45310.t1/1-657	0.13433	0.137974	MICOS complex subunit MIC19 isoform X2	2	C:GO:0016020; C:GO:0016021	C:membrane; C:integral component of membrane	
Acrdi.XM_015923516.1/1-2169 | Monca.augustus.g47462.t1/1-1914	0.167576	0.137924	nischarin isoform X2	1	F:GO:0005488	F:binding	
Acrdi.XM_015897250.1/1-1482 | Monca.augustus.g56891.t1/1-2229	0.136536	0.137916	ras guanyl-releasing 3-like isoform X1	23	P:GO:0043406; F:GO:0008270; F:GO:0042803; P:GO:0090630;	P:positive regulation of MAP kinase activity; F:zinc ion binding; F:protein homodimerization activity; P:activation of GTPase activity; P:regulation of phospholipase C activity; P:small GTPase mediated signal transductio[illegible]	
Acrdi.XM_015904868.1/1-708 | Monca.augustus.g42619.t1/1-711	0.19204	0.137912	Synaptosomal-associated 29	6	P:GO:0016082; F:GO:0019905; P:GO:0031629; F:GO:0005484;	P:synaptic vesicle priming; F:syntaxin binding; P:synaptic vesicle fusion to presynaptic active zone membrane; F:SNAP receptor activity; C:SNARE complex; C:plasma membrane	
Acrdi.XM_015920081.1/1-1842 | Monca.augustus.g28569.t1/1-1842	0.159006	0.13789	palmitoyltransferase ZDHHC17	1	F:GO:0016746	F:transferase activity, transferring acyl groups	
Acrdi.XM_015899983.1/1-882 | Monca.augustus.g6675.t1/1-5340	0.144038	0.137888	Monocarboxylate transporter 10	3	C:GO:0016020; P:GO:0006810; F:GO:0005215	C:membrane; P:transport; F:transporter activity	
Acrdi.XM_015923246.1/1-3966 | Monca.augustus.g1830.t1/1-4758	0.183342	0.137871	A disintegrin and metallo ase with thrombospondin motifs				
Acrdi.XM_015904053.1/1-3540 | Monca.augustus.g27782.t1/1-3366	0.142353	0.137867	hephaestin isoform X1	6	F:GO:0005507; F:GO:0016491; F:GO:0046872; C:GO:0016020;	F:copper ion binding; F:oxidoreductase activity; F:metal ion binding; C:membrane; C:integral component of membrane; P:oxidation-reduction process	
Acrdi.XM_015908338.1/1-3144 | Monca.augustus.g34620.t1/1-5133	0.182277	0.137838	Multidrug resistance-associated 4	8	F:GO:0000166; F:GO:0016887; F:GO:0005524; F:GO:0042626;	F:nucleotide binding; F:ATPase activity; F:ATP binding; F:ATPase activity, coupled to transmembrane movement of substances; P:transmembrane transport; C:membrane; C:integral component of membrane; [illegible]	
Acrdi.XM_015909282.1/1-1653 | Monca.augustus.g61668.t1/1-2634	0.167358	0.137826	Collagen alpha-1(VII) chain	3	F:GO:0003824; F:GO:0046872; P:GO:0008152	F:catalytic activity; F:metal ion binding; P:metabolic process	
Acrdi.XM_015906663.1/1-1380 | Monca.adi2mcaRNA16487_R0/1-1149	0.353262	0.137789	PREDICTED: uncharacterized protein LOC107341260	5	F:GO:0003677; C:GO:0016020; C:GO:0016021; P:GO:0015074;	F:DNA binding; C:membrane; C:integral component of membrane; P:DNA integration; P:DNA recombination	
Acrdi.XM_015909820.1/1-2472 | Monca.augustus.g47085.t1/1-2478	0.143896	0.137785	DNA-binding RFX6	4	C:GO:0005634; F:GO:0003700; F:GO:0000978; P:GO:0006357	C:nucleus; F:transcription factor activity, sequence-specific DNA binding; F:RNA polymerase II core promoter proximal region sequence-specific DNA binding; P:regulation of transcription from RNA polymerase II pro[illegible]	
Acrdi.XM_015910002.1/1-849 | Monca.augustus.g7104.t1/1-1224	0.132859	0.137779	zinc transporter ZIP13 isoform X2	3	P:GO:0055085; F:GO:0046873; P:GO:0030001	P:transmembrane transport; F:metal ion transmembrane transporter activity; P:metal ion transport	
Acrdi.XM_015896437.1/1-540 | Monca.augustus.g48026.t1/1-861	0.1934	0.137746	chymotrypsin-like elastase family member 2A	1	F:GO:0008236	F:serine-type peptidase activity	
Acrdi.XM_015901305.1/1-1254 | Monca.augustus.g43377.t1/1-1329	0.170417	0.137715	Tumor susceptibility gene 101	2	P:GO:0015031; P:GO:0006464	P:protein transport; P:cellular protein modification process	
Acrdi.XM_015914417.1/1-456 | Monca.augustus.g67296.t1/1-747	0.123387	0.137704	60S ribosomal L7-like	4	P:GO:0000463; F:GO:0003735; P:GO:0002181; C:GO:0022625	P:maturation of LSU-rRNA from tricistronic rRNA transcript (SSU-rRNA, 5.8S rRNA, LSU-rRNA); F:structural constituent of ribosome; P:cytoplasmic translation; C:cytosolic large ribosomal subunit	
Acrdi.XM_015903806.1/1-1068 | Monca.augustus.g6557.t1/1-1065	0.200773	0.137695	Wnt-7b isoform X2	42	P:GO:0060484; P:GO:0060482; P:GO:0008284; P:GO:0060560;	P:lung-associated mesenchyme development; P:lobar bronchus development; P:positive regulation of cell proliferation; P:developmental growth involved in morphogenesis; F:receptor agonist activity; P:odontogenesis [illegible]	
Acrdi.XM_015910994.1/1-2082 | Monca.augustus.g3408.t1/1-2115	0.138406	0.137694	calpain-9 isoform X1	2	F:GO:0008234; C:GO:0005622	F:cysteine-type peptidase activity; C:intracellular	
Acrdi.XM_015906327.1/1-516 | Monca.augustus.g23852.t1/1-531	0.204323	0.13769	low density lipo receptor adapter 1-like				
Acrdi.XM_015915586.1/1-1743 | Monca.augustus.g12504.t1/1-2664	0.144877	0.137688	centrosomal of 104 kDa isoform X1	1	C:GO:0005814	C:centriole	
Acrdi.XM_015901551.1/1-2112 | Monca.augustus.g55983.t1/1-2523	0.172994	0.137673	sodium bicarbonate transporter 11	3	C:GO:0016020; P:GO:0006820; F:GO:0005215	C:membrane; P:anion transport; F:transporter activity	
Acrdi.XM_015917804.1/1-1278 | Monca.augustus.g53112.t1/1-1785	0.197782	0.137648	ubiquilin-1 isoform X1	12	P:GO:0032270; F:GO:0005515; C:GO:0043231; P:GO:0033554;	P:positive regulation of cellular protein metabolic process; F:protein binding; C:intracellular membrane-bounded organelle; P:cellular response to stress; P:response to chemical; P:regulation of cellular response to st[illegible]	
Acrdi.XM_015897224.1/1-1038 | Monca.augustus.g63825.t1/1-993	0.139488	0.137637	melatonin receptor type 1A-like	3	P:GO:0007165; C:GO:0016020; F:GO:0004871	P:signal transduction; C:membrane; F:signal transducer activity	
Acrdi.XM_015908789.1/1-2022 | Monca.augustus.g4698.t1/1-2031	0.162143	0.13762	NEDD1 isoform X1	6	C:GO:0000242; C:GO:0005814; C:GO:0036064; C:GO:0000922;	C:pericentriolar material; C:centriole; C:ciliary basal body; C:spindle pole; P:single-organism cellular process; P:protein localization to centrosome	
Acrdi.XM_015896004.1/1-1068 | Monca.augustus.g47633.t1/1-891	0.149931	0.1376	Collagen alpha-1(XII) chain				
Acrdi.XM_015905114.1/1-744 | Monca.adi2mcaRNA14715_R2/1-597	0.606609	0.137547	Retrovirus-related Pol poly from transposon				
Acrdi.XM_015909290.1/1-2130 | Monca.augustus.g35319.t1/1-2304	0.212714	0.137537	anaphase-promoting complex subunit 5 isoform X1				
Acrdi.XM_015921379.1/1-1383 | Monca.augustus.g61083.t1/1-1488	0.183264	0.137535	PREDICTED: uncharacterized protein LOC107354885				
Acrdi.XM_015896955.1/1-660 | Monca.augustus.g29648.t1/1-657	0.128379	0.137492	proto-oncogene c-Fos-like isoform X1	2	F:GO:0003700; F:GO:0043565	F:transcription factor activity, sequence-specific DNA binding; F:sequence-specific DNA binding	
Acrdi.XM_015915321.1/1-1059 | Monca.augustus.g25288.t1/1-987	0.15996	0.137479	extradiol ring-cleavage dioxygenase	5	P:GO:0006725; F:GO:0051213; F:GO:0016491; F:GO:0008198;	P:cellular aromatic compound metabolic process; F:dioxygenase activity; F:oxidoreductase activity; F:ferrous iron binding; P:oxidation-reduction process	
Acrdi.XM_015893276.1/1-2172 | Monca.augustus.g534.t1/1-2193	0.144077	0.137475	centrosomal of 63 kDa-like				
Acrdi.XM_015915175.1/1-3345 | Monca.augustus.g48125.t1/1-6486	0.16299	0.137464	Phosphatidylinositol phosphatase PTPRQ	2	C:GO:0016020; C:GO:0016021	C:membrane; C:integral component of membrane	
Acrdi.XM_015905217.1/1-999 | Monca.augustus.g52837.t1/1-1380	0.132097	0.137454	synaptotagmin-7 isoform X4	6	P:GO:0065007; F:GO:0005515; C:GO:0016020; F:GO:0043167;	P:biological regulation; F:protein binding; C:membrane; F:ion binding; C:cell part; P:exocytosis	
Acrdi.XM_015911752.1/1-984 | Monca.augustus.g16306.t1/1-960	0.150018	0.137439	Calcium calmodulin-dependent kinase type IV	10	C:GO:0005634; F:GO:0005524; F:GO:0005516; C:GO:0005737;	C:nucleus; F:ATP binding; F:calmodulin binding; C:cytoplasm; P:positive regulation of transcription, DNA-templated; P:protein autophosphorylation; F:calmodulin-dependent p	EC:2.7.11; EC:2.7.11.17
Acrdi.XM_015919812.1/1-1650 | Monca.augustus.g41942.t1/1-3213	0.125066	0.137423	SH3 and PX domain-containing 2A-like isoform X4				
Acrdi.XM_015924633.1/1-1794 | Monca.augustus.g35417.t1/1-1824	0.164458	0.13742	cyclic nucleotide-binding domain-containing 2-like				
Acrdi.XM_015925459.1/1-783 | Monca.augustus.g58564.t1/1-954	0.202195	0.1374	U11 U12 small nuclear ribonucleo 35 kDa	2	F:GO:0003676; C:GO:0005681	F:nucleic acid binding; C:spliceosomal complex	
Acrdi.XM_015920626.1/1-3339 | Monca.augustus.g36305.t1/1-3783	0.144212	0.137399	Pre-mRNA cleavage complex 2 Pcf11				
Acrdi.XM_015922397.1/1-747 | Monca.augustus.g25487.t1/1-732	0.163334	0.13738	PREDICTED: uncharacterized protein LOC107355801 isoform X2				
Acrdi.XM_015925210.1/1-2298 | Monca.augustus.g23864.t1/1-3333	0.185245	0.137362	zinc finger DZIP1L-like	1	F:GO:0046872	F:metal ion binding	
Acrdi.XM_015908504.1/1-1824 | Monca.augustus.g28025.t1/1-1818	0.143497	0.137346	EH domain-binding 1 isoform X6	2	C:GO:0005737; C:GO:0005886	C:cytoplasm; C:plasma membrane	
Acrdi.XM_015900942.1/1-1086 | Monca.augustus.g27907.t1/1-1053	0.188668	0.137341	beta-1,3-galactosyltransferase 5-like	2	C:GO:0016020; F:GO:0016740	C:membrane; F:transferase activity	
Acrdi.XM_015913948.1/1-2154 | Monca.augustus.g20983.t1/1-2472	0.167111	0.137335	DDB1- and CUL4-associated factor 6 isoform X5	6	C:GO:0005634; C:GO:0005925; C:GO:0005737; F:GO:0030374;	C:nucleus; C:focal adhesion; C:cytoplasm; F:ligand-dependent nuclear receptor transcription coactivator activity; P:positive regulation of transcription from RNA polymerase II promoter; C:Cul4-RING E3 ubiquitin ligase [illegible]	
Acrdi.XM_015904128.1/1-516 | Monca.augustus.g32308.t1/1-519	0.182223	0.1373	J domain-containing				
Acrdi.XM_015905481.1/1-2415 | Monca.augustus.g28291.t1/1-2649	0.134711	0.137296	Short transient receptor potential channel 5	5	C:GO:0005887; F:GO:0015279; P:GO:0006828; P:GO:0051480;	C:integral component of plasma membrane; F:store-operated calcium channel activity; P:manganese ion transport; P:regulation of cytosolic calcium ion concentration; P:calcium ion transmembrane transport	
Acrdi.XM_015917725.1/1-1332 | Monca.augustus.g1562.t1/1-1620	0.146878	0.137257	Formimidoyltransferase-cyclodeaminase	2	F:GO:0003824; P:GO:0008152	F:catalytic activity; P:metabolic process	
Acrdi.XM_015906493.1/1-954 | Monca.augustus.g13119.t1/1-954	0.19103	0.137255	Nucleoside diphosphate-linked moiety X motif 22	2	C:GO:0005654; F:GO:0016787	C:nucleoplasm; F:hydrolase activity	
Acrdi.XM_015897830.1/1-1896 | Monca.adi2mcaRNA6779_R0/1-1881	0.185067	0.137238	E3 ubiquitin- ligase RNF103	5	F:GO:0008270; F:GO:0046872; C:GO:0016020; C:GO:0016021;	F:zinc ion binding; F:metal ion binding; C:membrane; C:integral component of membrane; F:ligase activity	
Acrdi.XM_015895829.1/1-3099 | Monca.augustus.g63627.t1/1-3132	0.15942	0.137215	dispatched homolog 1	2	C:GO:0016020; C:GO:0016021	C:membrane; C:integral component of membrane	
Acrdi.XM_015915124.1/1-333 | Monca.augustus.g26562.t1/1-345	0.174277	0.137138	hypothetical LOC496790	1	C:GO:0016020	C:membrane	
Acrdi.XM_015913937.1/1-1068 | Monca.augustus.g10758.t1/1-1038	0.167241	0.137129	MHCK EF2 kinase domain containing	1	P:GO:0009987	P:cellular process	
Acrdi.XM_015900221.1/1-468 | Monca.augustus.g62865.t1/1-465	0.133963	0.137109	calmodulin	1	F:GO:0005509	F:calcium ion binding	
Acrdi.XM_015915283.1/1-1164 | Monca.augustus.g15241.t1/1-1248	0.240709	0.1371	formin 14				
Acrdi.XM_015919518.1/1-888 | Monca.augustus.g54052.t1/1-903	0.202322	0.137073	mitochondrial basic amino acids transporter-like	6	P:GO:0055085; F:GO:0003735; C:GO:0016020; C:GO:0016021;	P:transmembrane transport; F:structural constituent of ribosome; C:membrane; C:integral component of membrane; P:transport; P:translation	
Acrdi.XM_015893801.1/1-354 | Monca.augustus.g51054.t1/1-540	0.126694	0.137065	Dr1	2	C:GO:0005634; F:GO:0046982	C:nucleus; F:protein heterodimerization activity	
Acrdi.XM_015908113.1/1-1797 | Monca.augustus.g33226.t1/1-1647	0.127968	0.137053	DNA helicase MCM8	5	F:GO:0097159; F:GO:1901363; P:GO:0000724; P:GO:0044699;	F:organic cyclic compound binding; F:heterocyclic compound binding; P:double-strand break repair via homologous recombination; P:single-organism process; C:MCM8-MCM9 complex	
Acrdi.XM_015898411.1/1-1305 | Monca.augustus.g58549.t1/1-1506	0.167921	0.137052	carboxyl-terminal PDZ ligand of neuronal nitric oxide synthase -like				
Acrdi.XM_015915139.1/1-1842 | Monca.adi2mcaRNA25790_R1/1-1638	0.161717	0.137044	FAM91A1 isoform X2				
Acrdi.XM_015900621.1/1-672 | Monca.augustus.g33658.t1/1-645	0.196953	0.137036	calcipressin-2-like isoform X1	2	P:GO:0050794; C:GO:0005622	P:regulation of cellular process; C:intracellular	
Acrdi.XM_015911179.1/1-618 | Monca.augustus.g37505.t1/1-621	0.178084	0.13703	SGNH hydrolase	1	F:GO:0016787	F:hydrolase activity	
Acrdi.XM_015904461.1/1-2241 | Monca.augustus.g33604.t1/1-2262	0.189294	0.137012	Solute carrier family 15 member 1	5	P:GO:0006857; C:GO:0016020; C:GO:0016021; P:GO:0006810;	P:oligopeptide transport; C:membrane; C:integral component of membrane; P:transport; F:transporter activity	
Acrdi.XM_015911543.1/1-1407 | Monca.augustus.g45429.t1/1-3039	0.119046	0.136973	matrilin-2-like isoform X1	3	F:GO:0005509; P:GO:0006508; F:GO:0004252	F:calcium ion binding; P:proteolysis; F:serine-type endopeptidase activity	
Acrdi.XM_015906929.1/1-2469 | Monca.augustus.g33391.t1/1-1584	0.192749	0.136957	Organic cation transporter	2	F:GO:0022891; C:GO:0016021	F:substrate-specific transmembrane transporter activity; C:integral component of membrane	
Acrdi.XM_015904921.1/1-777 | Monca.augustus.g8818.t1/1-786	0.131806	0.136951	Transmembrane 163	1	P:GO:0006810	P:transport	
Acrdi.XM_015912169.1/1-2706 | Monca.augustus.g57801.t1/1-2781	0.15945	0.136945	Hormonally up-regulated neu tumor-associated kinase	6	C:GO:0005634; F:GO:0005524; C:GO:0005737; F:GO:0004674;	C:nucleus; F:ATP binding; C:cytoplasm; F:protein serine/threonine kinase activity; P:protein phosphorylation; P:intracellular signal transduction	EC:2.7.11
Acrdi.XM_015897214.1/1-1167 | Monca.augustus.g7166.t1/1-1164	0.164675	0.136938	Chitin deacetylase	4	F:GO:0004568; F:GO:0008061; P:GO:0006032; C:GO:0005576	F:chitinase activity; F:chitin binding; P:chitin catabolic process; C:extracellular region	EC:3.2.1.14
Acrdi.XM_015919202.1/1-1260 | Monca.augustus.g54941.t1/1-1254	0.171805	0.136924	neutral sphingomyelinase	2	C:GO:0016020; C:GO:0016021	C:membrane; C:integral component of membrane	
Acrdi.XM_015898465.1/1-2034 | Monca.augustus.g33979.t1/1-2136	0.140428	0.136901	peptidyl-glycine alpha-amidating monooxygenase	1	F:GO:0003824	F:catalytic activity	
Acrdi.XM_015913619.1/1-981 | Monca.augustus.g48693.t1/1-1155	0.175363	0.136881	histamine H2 receptor-like	4	C:GO:0005887; F:GO:0004930; P:GO:0007186; P:GO:0007166	C:integral component of plasma membrane; F:G-protein coupled receptor activity; P:G-protein coupled receptor signaling pathway; P:cell surface receptor signaling pathway	
Acrdi.XM_015892081.1/1-606 | Monca.augustus.g32810.t1/1-903	0.181072	0.136864	non-structural maintenance of chromosomes element 4 homolog A	3	P:GO:0006281; C:GO:0005634; C:GO:0030915	P:DNA repair; C:nucleus; C:Smc5-Smc6 complex	
Acrdi.XM_015917825.1/1-1095 | Monca.augustus.g23511.t1/1-1533	0.163751	0.136851	U3 small nucleolar RNA-associated 15 homolog	3	P:GO:0006364; C:GO:0005730; P:GO:0045766	P:rRNA processing; C:nucleolus; P:positive regulation of angiogenesis	
Acrdi.XM_015913434.1/1-1437 | Monca.augustus.g58015.t1/1-3564	0.159503	0.136829	adhesion G -coupled receptor L2-like isoform X1	6	F:GO:0004930; F:GO:0004888; P:GO:0007186; C:GO:0016020;	F:G-protein coupled receptor activity; F:transmembrane signaling receptor activity; P:G-protein coupled receptor signaling pathway; C:membrane; C:integral component of membrane; P:cell surface receptor signaling [illegible]	
Acrdi.XM_015923681.1/1-2079 | Monca.augustus.g34973.t1/1-2940	0.16762	0.136824	PREDICTED: uncharacterized protein LOC107357045				
Acrdi.XM_015919203.1/1-1008 | Monca.adi2mcaRNA30330_R0/1-1005	0.188833	0.136798	octopamine receptor	1	F:GO:0004871	F:signal transducer activity	
Acrdi.XM_015904465.1/1-1044 | Monca.augustus.g33590.t1/1-1260	0.127241	0.136794	Polycomb EED	1	C:GO:0035098	C:ESC/E(Z) complex	
Acrdi.XM_015923091.1/1-894 | Monca.augustus.g70891.t1/1-765	0.218906	0.136782	thiopurine S-methyltransferase	3	F:GO:0008757; F:GO:0008119; P:GO:0032259	F:S-adenosylmethionine-dependent methyltransferase activity; F:thiopurine S-methyltransferase activity; P:methylation	
Acrdi.XM_015903086.1/1-714 | Monca.augustus.g48432.t1/1-747	0.18218	0.136742	carbonyl reductase family member 4 isoform X1	9	P:GO:0006725; P:GO:1901360; F:GO:0048037; P:GO:0030638;	P:cellular aromatic compound metabolic process; P:organic cyclic compound metabolic process; F:cofactor binding; P:polyketide metabolic process; C:mitochondrion; P:quinone metabolic process; P:glycoside metaboli[illegible]	
Acrdi.XM_015895888.1/1-519 | Monca.augustus.g16930.t1/1-516	0.142603	0.13674	phosphatase 1 regulatory subunit 27				
Acrdi.XM_015899195.1/1-1035 | Monca.augustus.g52004.t1/1-990	0.0772149	0.13673	family ATP-grasp ribosomal peptide maturase	4	F:GO:0005524; F:GO:0046872; F:GO:0016740; F:GO:0016874	F:ATP binding; F:metal ion binding; F:transferase activity; F:ligase activity	
Acrdi.XM_015892085.1/1-1323 | Monca.augustus.g36059.t1/1-1437	0.17264	0.136702	legumain	3	P:GO:0006624; F:GO:0004197; P:GO:0051603	P:vacuolar protein processing; F:cysteine-type endopeptidase activity; P:proteolysis involved in cellular protein catabolic process	EC:3.4.22
Acrdi.XM_015900886.1/1-2079 | Monca.augustus.g27843.t1/1-2139	0.160216	0.13669	targeting for Xklp2 isoform X2				
Acrdi.XM_015920159.1/1-1116 | Monca.augustus.g23539.t1/1-1116	0.175788	0.136658	nodulin 21 -like transporter family	3	F:GO:0022857; P:GO:0006810; C:GO:0005886	F:transmembrane transporter activity; P:transport; C:plasma membrane	
Acrdi.XM_015914423.1/1-1950 | Monca.augustus.g41425.t1/1-1896	0.178576	0.13664	ATPase WRNIP1	3	F:GO:0003677; F:GO:0005524; P:GO:0006260	F:DNA binding; F:ATP binding; P:DNA replication	
Acrdi.XM_015897165.1/1-534 | Monca.adi2mcaRNA6122_R0/1-651	0.147328	0.136639	intraflagellar transport 43 homolog	2	C:GO:0031514; C:GO:0030991	C:motile cilium; C:intraciliary transport particle A	
Acrdi.XM_015908484.1/1-999 | Monca.augustus.g19171.t1/1-996	0.139202	0.136625	probable G- coupled receptor No9	3	C:GO:0016020; P:GO:0007210; F:GO:0004993	C:membrane; P:serotonin receptor signaling pathway; F:G-protein coupled serotonin receptor activity	
Acrdi.XM_015909988.1/1-5214 | Monca.augustus.g44397.t1/1-5052	0.167033	0.136604	polycystic kidney disease 1-like 2				
Acrdi.XM_015912238.1/1-912 | Monca.augustus.g22447.t1/1-948	0.22493	0.136585	Peroxisomal biogenesis factor 19	5	C:GO:0005777; C:GO:0031526; F:GO:0005515; P:GO:0072663;	C:peroxisome; C:brush border membrane; F:protein binding; P:establishment of protein localization to peroxisome; P:protein transport	
Acrdi.XM_015897919.1/1-654 | Monca.augustus.g23845.t1/1-651	0.16458	0.136584	dynactin complex subunit	3	C:GO:0005869; P:GO:0010970; F:GO:0070840	C:dynactin complex; P:transport along microtubule; F:dynein complex binding	
Acrdi.XM_015920592.1/1-591 | Monca.adi2mcaRNA31889_R0/1-663	0.439196	0.136576	Retrovirus-related Pol poly from transposon				
Acrdi.XM_015895142.1/1-531 | Monca.augustus.g35256.t1/1-1407	0.192725	0.136574	ankyrin repeat				
Acrdi.XM_015915937.1/1-963 | Monca.augustus.g24363.t1/1-1572	0.105864	0.136556	transmembrane 209-like	2	C:GO:0016020; C:GO:0016021	C:membrane; C:integral component of membrane	
Acrdi.XM_015913257.1/1-840 | Monca.augustus.g67371.t1/1-999	0.191652	0.136548	prune homolog	2	C:GO:0005737; F:GO:0016462	C:cytoplasm; F:pyrophosphatase activity	
Acrdi.XM_015891763.1/1-987 | Monca.augustus.g64465.t1/1-1158	0.132686	0.136543	BTB POZ domain-containing KCTD9-like	1	P:GO:0051260	P:protein homooligomerization	
Acrdi.XM_015900190.1/1-816 | Monca.augustus.g62843.t1/1-804	0.173446	0.136525	tetratricopeptide repeat 28-like				
Acrdi.XM_015896907.1/1-732 | Monca.augustus.g10291.t1/1-1458	0.157835	0.136456	Thymidine kinase mitochondrial	7	F:GO:0000166; P:GO:0009262; P:GO:0034654; P:GO:0006264;	F:nucleotide binding; P:deoxyribonucleotide metabolic process; P:nucleobase-containing compound biosynthetic process; P:mitochondrial DNA replication; P:pyrimidine deoxy	EC:2.7.1.145
Acrdi.XM_015910386.1/1-546 | Monca.augustus.g51106.t1/1-570	0.190793	0.136447	josephin-2 isoform X2	2	F:GO:0004843; P:GO:0016579	F:thiol-dependent ubiquitin-specific protease activity; P:protein deubiquitination	EC:3.4.19.12
Acrdi.XM_015903980.1/1-1272 | Monca.augustus.g32302.t1/1-1284	0.182159	0.136433	tyramine receptor 1	3	F:GO:0004930; P:GO:0007154; P:GO:0044700	F:G-protein coupled receptor activity; P:cell communication; P:single organism signaling	
Acrdi.XM_015904668.1/1-3972 | Monca.augustus.g21034.t1/1-714	0.13039	0.136425	transport Sec16A isoform X3				
Acrdi.XM_015917177.1/1-2184 | Monca.augustus.g31454.t1/1-2478	0.154401	0.136419	PREDICTED: uncharacterized protein LOC107350921	2	C:GO:0016020; C:GO:0016021	C:membrane; C:integral component of membrane	
Acrdi.XM_015916502.1/1-2439 | Monca.adi2mcaRNA27311_R0/1-2499	0.216966	0.136375	KAT8 regulatory NSL complex subunit 3-like	3	P:GO:0043967; C:GO:0031981; F:GO:0010485	P:histone H4 acetylation; C:nuclear lumen; F:H4 histone acetyltransferase activity	EC:2.3.1.48
Acrdi.XM_015907455.1/1-723 | Monca.augustus.g3954.t1/1-1188	0.186976	0.136366	Peptidyl-prolyl cis-trans isomerase FKBP4	7	C:GO:0005789; C:GO:0031072; F:GO:0003755; C:GO:0070062;	C:endoplasmic reticulum membrane; F:heat shock protein binding; F:peptidyl-prolyl cis-trans isomerase activity; C:extracellular exosome; F:FK506 binding; P:chaperone-medi	EC:5.2.1.8
Acrdi.XM_015911917.1/1-540 | Monca.augustus.g30049.t1/1-1218	0.0973211	0.136341	ETS homologous factor isoform X2	5	C:GO:0005634; P:GO:0030154; F:GO:0043565; F:GO:0000981;	C:nucleus; P:cell differentiation; F:sequence-specific DNA binding; F:RNA polymerase II transcription factor activity, sequence-specific DNA binding; P:regulation of transcription from RNA polymerase II promoter	
Acrdi.XM_015909986.1/1-1431 | Monca.augustus.g7099.t1/1-4098	0.145742	0.13633	M-phase inducer phosphatase-like isoform X1	3	F:GO:0004725; P:GO:0035335; C:GO:0005622	F:protein tyrosine phosphatase activity; P:peptidyl-tyrosine dephosphorylation; C:intracellular	EC:3.1.3.16; EC:3.1.3.48; EC:3.1.3.41
Acrdi.XM_015919859.1/1-11865 | Monca.augustus.g40453.t1/1-12441	0.202831	0.136322	probable E3 ubiquitin- ligase HECTD4 isoform X3				
Acrdi.XM_015919977.1/1-3588 | Monca.augustus.g9628.t1/1-4572	0.164428	0.136251	Synaptojanin-1	4	F:GO:0000166; F:GO:0042578; F:GO:0016787; P:GO:0046856	F:nucleotide binding; F:phosphoric ester hydrolase activity; F:hydrolase activity; P:phosphatidylinositol dephosphorylation	
Acrdi.XM_015901841.1/1-528 | Monca.augustus.g35915.t1/1-627	0.164541	0.136235	40S ribosomal S8	4	P:GO:0000462; F:GO:0003735; C:GO:0022627; P:GO:0006412	P:maturation of SSU-rRNA from tricistronic rRNA transcript (SSU-rRNA, 5.8S rRNA, LSU-rRNA); F:structural constituent of ribosome; C:cytosolic small ribosomal subunit; P:translation	
Acrdi.XM_015921775.1/1-1053 | Monca.augustus.g47558.t1/1-1056	0.190084	0.136178	N-acylethanolamine-hydrolyzing acid amidase	2	C:GO:0005737; C:GO:0043227	C:cytoplasm; C:membrane-bounded organelle	
Acrdi.XM_015902287.1/1-969 | Monca.augustus.g35101.t1/1-1236	0.184247	0.136172	Ethanolaminephosphotransferase 1	5	C:GO:0016020; C:GO:0016021; F:GO:0016780; P:GO:0008654;	C:membrane; C:integral component of membrane; F:phosphotransferase activity, for other substituted phosphate groups; P:phospholipid biosynthetic process; F:transferase activity	
Acrdi.XM_015925192.1/1-1188 | Monca.adi2mcaRNA37094_R0/1-1047	0.555326	0.13615	espin isoform X2	8	F:GO:0000166; F:GO:0005525; F:GO:0005524; P:GO:0007165;	F:nucleotide binding; F:GTP binding; F:ATP binding; P:signal transduction; P:small GTPase mediated signal transduction; C:membrane; C:integral component of membrane; C:intracellular	
Acrdi.XM_015911611.1/1-1182 | Monca.augustus.g30380.t1/1-1188	0.0981631	0.136148	Neuropeptide FF receptor 2	6	C:GO:0005887; F:GO:0004930; P:GO:0010033; P:GO:0007186;	C:integral component of plasma membrane; F:G-protein coupled receptor activity; P:response to organic substance; P:G-protein coupled receptor signaling pathway; P:response to endogenous stimulus; P:cell surface [illegible]	
Acrdi.XM_015917224.1/1-3564 | Monca.augustus.g55545.t1/1-3600	0.149811	0.136141	transient receptor potential cation channel subfamily A member 1-like				
Acrdi.XM_015901833.1/1-1164 | Monca.adi2mcaRNA11167_R0/1-1230	0.138989	0.13612	dynein regulatory complex 1-like				
Acrdi.XM_015923795.1/1-798 | Monca.augustus.g58441.t1/1-807	0.16734	0.136049	alpha-ketoglutarate-dependent dioxygenase alkB homolog 2 isoform X	6	C:GO:0043229; P:GO:0006281; F:GO:0046872; F:GO:0016706;	C:intracellular organelle; P:DNA repair; F:metal ion binding; F:oxidoreductase activity, acting on paired donors, with incorporation or reduction of molecular oxygen, 2-oxoglut	EC:1.14.11
Acrdi.XM_015919430.1/1-639 | Monca.augustus.g45490.t1/1-639	0.194734	0.136026	PREDICTED: uncharacterized protein LOC107353107				
Acrdi.XM_015911687.1/1-2409 | Monca.augustus.g44571.t1/1-2502	0.22054	0.136024	axin-1 isoform X2	4	C:GO:0005737; F:GO:0005096; C:GO:0005886; P:GO:0043547	C:cytoplasm; F:GTPase activator activity; C:plasma membrane; P:positive regulation of GTPase activity	
Acrdi.XM_015891619.1/1-414 | Monca.augustus.g34413.t1/1-480	0.106884	0.136016	Transient receptor potential cation channel subfamily V member 4	4	C:GO:0005887; F:GO:0015279; P:GO:0006828; P:GO:0051480	C:integral component of plasma membrane; F:store-operated calcium channel activity; P:manganese ion transport; P:regulation of cytosolic calcium ion concentration	
Acrdi.XM_015922754.1/1-888 | Monca.augustus.g267.t1/1-882	0.409327	0.136009	5-hydroxytryptamine receptor 5A-like	8	F:GO:0004930; P:GO:0007186; P:GO:0007165; C:GO:0016020;	F:G-protein coupled receptor activity; P:G-protein coupled receptor signaling pathway; P:signal transduction; C:membrane; C:integral component of membrane; P:serotonin receptor signaling pathway; F:G-protein cou[illegible]	
Acrdi.XM_015924650.1/1-1311 | Monca.augustus.g28212.t1/1-2361	0.161844	0.135937	serine threonine- kinase TBK1	25	P:GO:0043123; P:GO:0045944; P:GO:1904417; F:GO:0005524;	P:positive regulation of I-kappaB kinase/NF-kappaB signaling; P:positive regulation of transcription from RNA polymerase II promoter; P:positive regulation of xenophagy; F:#	EC:2.7.11
Acrdi.XM_015892021.1/1-1329 | Monca.augustus.g37393.t1/1-1278	0.211447	0.135936	serine incorporator 1	3	C:GO:0016021; F:GO:0015194; P:GO:0044699	C:integral component of membrane; F:L-serine transmembrane transporter activity; P:single-organism process	
Acrdi.XM_015909520.1/1-4692 | Monca.adi2mcaRNA2055_R0/1-4728	0.14023	0.13593	Extracellular calcium-sensing receptor	4	P:GO:0007186; C:GO:0044425; F:GO:0004871; C:GO:0005886	P:G-protein coupled receptor signaling pathway; C:membrane part; F:signal transducer activity; C:plasma membrane	
Acrdi.XM_015908020.1/1-330 | Monca.augustus.g5056.t1/1-375	0.160244	0.135879	ubiquitin 3	2	C:GO:0070062; C:GO:0005622	C:extracellular exosome; C:intracellular	
Acrdi.XM_015913976.1/1-921 | Monca.augustus.g59709.t1/1-2706	0.204754	0.135853	Trafficking particle complex subunit 10	4	C:GO:0005829; C:GO:1990071; P:GO:0006891; P:GO:0034498	C:cytosol; C:TRAPPII protein complex; P:intra-Golgi vesicle-mediated transport; P:early endosome to Golgi transport	
Acrdi.XM_015920397.1/1-1929 | Monca.augustus.g15945.t1/1-3651	0.16245	0.135836	zinc finger FYVE domain-containing 9	2	F:GO:0005515; P:GO:0007178	F:protein binding; P:transmembrane receptor protein serine/threonine kinase signaling pathway	
Acrdi.XM_015908620.1/1-1065 | Monca.augustus.g22359.t1/1-1062	0.119935	0.135817	Neuropeptide FF receptor 2	4	C:GO:0005887; P:GO:0007218; P:GO:0007166; F:GO:0004983	C:integral component of plasma membrane; P:neuropeptide signaling pathway; P:cell surface receptor signaling pathway; F:neuropeptide Y receptor activity	
Acrdi.XM_015914010.1/1-2250 | Monca.augustus.g68892.t1/1-2355	0.138107	0.135811	cullin-5 isoform X1	4	F:GO:0061630; F:GO:0031625; C:GO:0031466; P:GO:0042787	F:ubiquitin protein ligase activity; F:ubiquitin protein ligase binding; C:Cul5-RING ubiquitin ligase complex; P:protein ubiquitination involved in ubiquitin-dependent protein catabolic process	
Acrdi.XM_015898909.1/1-1656 | Monca.augustus.g38000.t1/1-2136	0.13221	0.135811	ATP-binding cassette sub-family B member mitochondrial	4	F:GO:0000166; F:GO:0042626; P:GO:0055085; C:GO:0016021	F:nucleotide binding; F:ATPase activity, coupled to transmembrane movement of substances; P:transmembrane transport; C:integral component of membrane	EC:3.6.1.3; EC:3.6.1.15
Acrdi.XM_015905848.1/1-1317 | Monca.augustus.g32222.t1/1-1353	0.157727	0.135806	hyccin isoform X1				
Acrdi.XM_015914661.1/1-1188 | Monca.augustus.g39754.t1/1-1503	0.104485	0.135798	gamma-aminobutyric acid receptor subunit rho-1-like	5	P:GO:1902476; C:GO:0016021; F:GO:0005230; F:GO:0005254;	P:chloride transmembrane transport; C:integral component of membrane; F:extracellular ligand-gated ion channel activity; F:chloride channel activity; C:plasma membrane	
Acrdi.XM_015908811.1/1-1824 | Monca.augustus.g4764.t1/1-2541	0.101603	0.135798	fibropellin-1 isoform X2	9	C:GO:0005615; C:GO:0033166; F:GO:0005509; C:GO:0016020;	C:extracellular space; C:hyaline layer; F:calcium ion binding; C:membrane; C:integral component of membrane; C:cytoplasmic vesicle; C:extracellular region; C:apical lamina of hyaline layer; C:proteinaceous extracellu[illegible]	
Acrdi.XM_015905447.1/1-585 | Monca.augustus.g33959.t1/1-1368	0.189276	0.13577	leucine-rich repeat-containing 28				
Acrdi.XM_015898197.1/1-1110 | Monca.augustus.g29229.t1/1-1110	0.151359	0.135753	Wnt-6	4	P:GO:0044763; C:GO:0005576; P:GO:0050789; P:GO:0061303	P:single-organism cellular process; C:extracellular region; P:regulation of biological process; P:cornea development in camera-type eye	
Acrdi.XM_015898047.1/1-5223 | Monca.adi2mcaRNA7026_R0/1-5472	0.149036	0.135748	neurogenic locus notch homolog 1-like	1	F:GO:0005509	F:calcium ion binding	
Acrdi.XM_015902441.1/1-2013 | Monca.augustus.g36355.t1/1-2037	0.125284	0.135747	alpha-1,6-mannosylglyco 6-beta-N-acetylglucosaminyltransferase A	1	F:GO:0016740	F:transferase activity	
Acrdi.XM_015899424.1/1-5301 | Monca.augustus.g46753.t1/1-5310	0.166856	0.135723	Carboxypeptidase D	7	C:GO:0005634; C:GO:0005615; C:GO:0005802; F:GO:0004181;	C:nucleus; C:extracellular space; C:trans-Golgi network; F:metallocarboxypeptidase activity; P:peptide metabolic process; F:serine-type carboxypeptidase activity; P:protein pr	EC:3.4.21; EC:3.4.17; EC:3.4.16
Acrdi.XM_015895281.1/1-1152 | Monca.augustus.g57155.t1/1-1158	0.153473	0.135721	neuropeptide FF receptor 2-like	4	C:GO:0005887; F:GO:0004930; P:GO:0007186; P:GO:0007166	C:integral component of plasma membrane; F:G-protein coupled receptor activity; P:G-protein coupled receptor signaling pathway; P:cell surface receptor signaling pathway	
Acrdi.XM_015909406.1/1-741 | Monca.augustus.g50696.t1/1-783	0.588329	0.135719	leukocyte cell-derived chemotaxin 1-like	6	P:GO:0006508; F:GO:0004252; F:GO:0008236; P:GO:0016485;	P:proteolysis; F:serine-type endopeptidase activity; F:serine-type peptidase activity; P:protein processing; F:peptidase activity; F:hydrolase activity	
Acrdi.XM_015891775.1/1-654 | Monca.augustus.g41930.t1/1-2031	0.141907	0.135712	metal transporter CNNM4-like	1	C:GO:0016020	C:membrane	
Acrdi.XM_015896720.1/1-1392 | Monca.augustus.g11231.t1/1-1287	0.163025	0.135707	octopamine receptor 1-like	1	C:GO:0016020	C:membrane	
Acrdi.XM_015899267.1/1-1758 | Monca.augustus.g578.t1/1-1758	0.149409	0.135698	kelch 12 isoform X1				
Acrdi.XM_015897727.1/1-591 | Monca.augustus.g9970.t1/1-585	0.129263	0.135693	NK1 transcription factor-related 2-like	3	C:GO:0005634; P:GO:0006355; F:GO:0043565	C:nucleus; P:regulation of transcription, DNA-templated; F:sequence-specific DNA binding	
Acrdi.XM_015912394.1/1-1179 | Monca.augustus.g40688.t1/1-1116	0.149751	0.13563	WD repeat-containing 74	1	C:GO:0044424	C:intracellular part	
Acrdi.XM_015924853.1/1-1398 | Monca.augustus.g37684.t1/1-1398	0.223162	0.135605	adenylyl cyclase-associated 1	3	F:GO:0003779; P:GO:0007010; P:GO:0000902	F:actin binding; P:cytoskeleton organization; P:cell morphogenesis	
Acrdi.XM_015899922.1/1-1806 | Monca.augustus.g57216.t1/1-2433	0.155816	0.135601	MICOS complex subunit MIC60-like	2	C:GO:0016021; C:GO:0005743	C:integral component of membrane; C:mitochondrial inner membrane	
Acrdi.XM_015903179.1/1-1449 | Monca.adi2mcaRNA12554_R0/1-1263	0.181403	0.135583	Coiled-coil domain-containing 112				
Acrdi.XM_015912261.1/1-1245 | Monca.augustus.g28328.t1/1-1245	0.111451	0.13558	synaptotagmin-14 isoform X4	8	P:GO:0048791; F:GO:0019905; P:GO:0006906; F:GO:0005509;	P:calcium ion-regulated exocytosis of neurotransmitter; F:syntaxin binding; P:vesicle fusion; F:calcium ion binding; F:clathrin binding; P:regulation of calcium ion-dependent exocytosis; C:plasma membrane; F:calcium [illegible]	
Acrdi.XM_015923456.1/1-3573 | Monca.augustus.g31110.t1/1-3783	0.184582	0.13557	collagen alpha-6(VI) chain-like	5	F:GO:0008270; P:GO:0006508; P:GO:0007155; F:GO:0005540;	F:zinc ion binding; P:proteolysis; P:cell adhesion; F:hyaluronic acid binding; F:metalloendopeptidase activity	
Acrdi.XM_015903349.1/1-1818 | Monca.augustus.g36021.t1/1-1881	0.160445	0.135566	TAF6-like RNA polymerase II p300 CBP-associated factor-associated fa	4	C:GO:0005634; F:GO:0046982; P:GO:0006352; P:GO:0051090	C:nucleus; F:protein heterodimerization activity; P:DNA-templated transcription, initiation; P:regulation of sequence-specific DNA binding transcription factor activity	
Acrdi.XM_015904492.1/1-987 | Monca.augustus.g33591.t1/1-993	0.129467	0.135527	poly(A) RNA polymerase GLD2 isoform X2	3	F:GO:0005488; C:GO:0044424; P:GO:0009987	F:binding; C:intracellular part; P:cellular process	
Acrdi.XM_015917538.1/1-555 | Monca.augustus.g25118.t1/1-549	0.201939	0.135522	39S ribosomal mitochondrial isoform X1	4	F:GO:0003735; C:GO:0005739; C:GO:0005840; P:GO:0006412	F:structural constituent of ribosome; C:mitochondrion; C:ribosome; P:translation	
Acrdi.XM_015919553.1/1-696 | Monca.augustus.g3672.t1/1-1014	0.0888713	0.135522	integrin-linked kinase-associated serine threonine phosphatase 2C isof	3	F:GO:0004722; F:GO:0046872; P:GO:0006470	F:protein serine/threonine phosphatase activity; F:metal ion binding; P:protein dephosphorylation	EC:3.1.3.16; EC:3.1.3.41
Acrdi.XM_015896495.1/1-5445 | Monca.adi2mcaRNA5355_R0/1-5271	0.192713	0.135499	DNA-dependent kinase catalytic subunit	3	C:GO:0031981; F:GO:0016301; P:GO:0006302	C:nuclear lumen; F:kinase activity; P:double-strand break repair	
Acrdi.XM_015921160.1/1-522 | Monca.adi2mcaRNA3280_R1/1-510	0.124405	0.135484	PREDICTED: uncharacterized protein CXorf65 homolog				
Acrdi.XM_015911666.1/1-3063 | Monca.adi2mcaRNA21955_R0/1-2964	0.186678	0.135479	DNA excision repair ERCC-6				
Acrdi.XM_015905297.1/1-1695 | Monca.adi2mcaRNA14912_R0/1-1473	0.164594	0.135475	RING finger 219	1	P:GO:0048026	P:positive regulation of mRNA splicing, via spliceosome	
Acrdi.XM_015918057.1/1-921 | Monca.augustus.g4309.t1/1-1356	0.396469	0.135449	tolloid 1 isoform X2				
Acrdi.XM_015903123.1/1-1299 | Monca.augustus.g38330.t1/1-2025	0.177692	0.135438	PREDICTED: uncharacterized protein LOC107337887 isoform X1	1	C:GO:0016021	C:integral component of membrane	
Acrdi.XM_015922999.1/1-633 | Monca.augustus.g67847.t1/1-513	0.129738	0.135435	chromatin assembly factor 1 subunit A-B isoform X1	2	C:GO:0016020; C:GO:0016021	C:membrane; C:integral component of membrane	
Acrdi.XM_015892988.1/1-558 | Monca.augustus.g25677.t1/1-564	0.185623	0.135418	proline-rich 36-like				
Acrdi.XM_015899742.1/1-1314 | Monca.augustus.g66195.t1/1-1761	0.140144	0.135417	AMP-dependent synthetase	4	F:GO:0003824; P:GO:0008152; C:GO:0016020; C:GO:0016021	F:catalytic activity; P:metabolic process; C:membrane; C:integral component of membrane	
Acrdi.XM_015905987.1/1-2148 | Monca.augustus.g27808.t1/1-4119	0.174291	0.135394	fantom isoform X1	2	P:GO:0006935; C:GO:0035869	P:chemotaxis; C:ciliary transition zone	
Acrdi.XM_015899398.1/1-825 | Monca.augustus.g37244.t1/1-846	0.162438	0.135364	eukaryotic translation initiation factor 4H isoform X1				
Acrdi.XM_015891925.1/1-1038 | Monca.augustus.g25658.t1/1-987	0.151406	0.135348	citrate lyase subunit beta mitochondrial	1	F:GO:0003824	F:catalytic activity	
Acrdi.XM_015907126.1/1-936 | Monca.augustus.g36964.t1/1-993	0.18755	0.135342	5-hydroxytryptamine receptor 4-like	3	F:GO:0004930; P:GO:0007186; C:GO:0016021	F:G-protein coupled receptor activity; P:G-protein coupled receptor signaling pathway; C:integral component of membrane	
Acrdi.XM_015899546.1/1-861 | Monca.augustus.g50192.t1/1-975	0.190659	0.135294	Testis-specific serine threonine- kinase 3	9	F:GO:0000166; F:GO:0005524; C:GO:0005634; C:GO:0005737;	F:nucleotide binding; F:ATP binding; C:nucleus; C:cytoplasm; P:spermatogenesis; F:protein kinase activity; F:protein serine/threonine kinase activity; P:protein phosphorylation; P:intracellular signal transduction	
Acrdi.XM_015904454.1/1-930 | Monca.augustus.g10685.t1/1-2160	0.167584	0.135282	activating molecule in BECN1-regulated autophagy 1-like isoform X1	2	C:GO:0043231; P:GO:0006914	C:intracellular membrane-bounded organelle; P:autophagy	
Acrdi.XM_015897985.1/1-1236 | Monca.augustus.g33360.t1/1-1320	0.0843523	0.135269	gamma-aminobutyric acid receptor subunit beta-2-like	6	P:GO:0034220; C:GO:0016021; C:GO:0030054; F:GO:0005230;	P:ion transmembrane transport; C:integral component of membrane; C:cell junction; F:extracellular ligand-gated ion channel activity; C:synapse; C:plasma membrane	
Acrdi.XM_015905029.1/1-1818 | Monca.adi2mcaRNA1469_R0/1-1824	0.151909	0.135259	probable ATP-dependent RNA helicase DDX52	5	F:GO:0003676; F:GO:0005524; F:GO:0010501; P:GO:0006364;	F:nucleic acid binding; F:ATP binding; P:RNA secondary structure unwinding; P:rRNA processing; F:ATP-dependent RNA helicase activity	EC:3.6.1.3; EC:3.6.1.15
Acrdi.XM_015909656.1/1-1305 | Monca.adi2mcaRNA19756_R0/1-1182	0.13603	0.13525	transcription factor E2F3 isoform X1	6	F:GO:0003677; C:GO:0005634; C:GO:0005667; F:GO:0003700;	F:DNA binding; C:nucleus; C:transcription factor complex; F:transcription factor activity, sequence-specific DNA binding; P:transcription, DNA-templated; P:regulation of transcription, DNA-templated	
Acrdi.XM_015903971.1/1-1011 | Monca.augustus.g69789.t1/1-1281	0.106463	0.135223	nucleolysin TIA-1 isoform p40-like isoform X1				
Acrdi.XM_015902227.1/1-2712 | Monca.augustus.g34162.t1/1-3720	0.208376	0.135217	BUD13 homolog isoform X3				
Acrdi.XM_015915649.1/1-438 | Monca.augustus.g55118.t1/1-417	0.131854	0.135214	UPF0047				
Acrdi.XM_015900313.1/1-729 | Monca.augustus.g63377.t1/1-711	0.142185	0.135208	haloacid dehalogenase-like hydrolase domain-containing At2g33255	2	P:GO:0008152; F:GO:0016787	P:metabolic process; F:hydrolase activity	
Acrdi.XM_015923834.1/1-3129 | Monca.augustus.g32966.t1/1-3162	0.133108	0.135208	atrial natriuretic peptide receptor 2-like	13	P:GO:0006182; F:GO:0016849; F:GO:0016829; F:GO:0004383;	P:cGMP biosynthetic process; F:phosphorus-oxygen lyase activity; F:lyase activity; F:guanylate cyclase activity; F:protein kinase activity; P:protein phosphorylation; C:intracellular; F:nucleotide binding; F:ATP binding; [illegible]	
Acrdi.XM_015920232.1/1-495 | Monca.augustus.g62715.t1/1-945	0.142138	0.135171	Histone RNA hairpin-binding	1	F:GO:0003729	F:mRNA binding	
Acrdi.XM_015910238.1/1-1488 | Monca.augustus.g25486.t1/1-1482	0.137256	0.135171	Alpha-(1,3)-fucosyltransferase 10	4	P:GO:0070085; F:GO:0008417; C:GO:0000139; P:GO:0044763	P:glycosylation; F:fucosyltransferase activity; C:Golgi membrane; P:single-organism cellular process	
Acrdi.XM_015902460.1/1-1260 | Monca.augustus.g41952.t1/1-1371	0.161311	0.135142	Suppressor of	5	P:GO:0050794; P:GO:0048519; C:GO:0044424; P:GO:0044699;	P:regulation of cellular process; P:negative regulation of biological process; C:intracellular part; P:single-organism process; P:cellular process	
Acrdi.XM_015921504.1/1-1371 | Monca.augustus.g58052.t1/1-1377	0.170333	0.135124	Usher syndrome type-1G	2	C:GO:0044464; P:GO:0032501	C:cell part; P:multicellular organismal process	
Acrdi.XM_015896917.1/1-1671 | Monca.augustus.g62726.t1/1-2304	0.130443	0.135123	ribosomal S6 kinase alpha-3 isoform X2	4	P:GO:0007165; F:GO:0004672; F:GO:0005488; C:GO:0044424	P:signal transduction; F:protein kinase activity; F:binding; C:intracellular part	
Acrdi.XM_015897660.1/1-1035 | Monca.augustus.g50739.t1/1-819	0.123476	0.135104	T-box transcription factor TBX4	5	C:GO:0005634; F:GO:0003677; F:GO:0003700; P:GO:0006351;	C:nucleus; F:DNA binding; F:transcription factor activity, sequence-specific DNA binding; P:transcription, DNA-templated; P:regulation of transcription, DNA-templated	
Acrdi.XM_015904009.1/1-975 | Monca.augustus.g69804.t1/1-927	0.138765	0.135086	retinol dehydrogenase 3-like isoform X3	4	F:GO:0016491; C:GO:0016020; C:GO:0016021; P:GO:0055114	F:oxidoreductase activity; C:membrane; C:integral component of membrane; P:oxidation-reduction process	
Acrdi.XM_015925477.1/1-1263 | Monca.augustus.g4261.t1/1-1263	0.182212	0.135068	homocysteine-responsive endoplasmic reticulum-resident ubiquitin-lik	4	C:GO:0016020; C:GO:0016021; P:GO:0006986; F:GO:0003674	C:membrane; C:integral component of membrane; P:response to unfolded protein; F:molecular_function	
Acrdi.XM_015910360.1/1-1389 | Monca.augustus.g36419.t1/1-1392	0.227643	0.135043	PREDICTED: uncharacterized protein LOC107344670	2	P:GO:0018026; F:GO:0016279	P:peptidyl-lysine monomethylation; F:protein-lysine N-methyltransferase activity	
Acrdi.XM_015914765.1/1-1992 | Monca.augustus.g47155.t1/1-4041	0.183477	0.135028	Structure-specific endonuclease subunit SLX4				
Acrdi.XM_015905890.1/1-2868 | Monca.adi2mcaRNA15607_R0/1-2757	0.16816	0.134995	ZP domain-containing -like	3	F:GO:0005509; C:GO:0016020; C:GO:0016021	F:calcium ion binding; C:membrane; C:integral component of membrane	

Acrdi.XM_015913742.1/1-543	Monca.augustus.g21656.t1/1-540	0.134758	0.134981	PRKR-interacting 1			
Acrdi.XM_015901717.1/1-2103	Monca.augustus.g10808.t1/1-2814	0.177007	0.134958	maltase- intestinal-like			
Acrdi.XM_015917435.1/1-1272	Monca.adi2mcaRNA28396_R1/1-1029	0.109983	0.134953	nitrogen permease regulator 3 isoform X3			
Acrdi.XM_015899437.1/1-717	Monca.adi2mcaRNA8500_R0/1-750	0.244473	0.134952	Inositol oxygenase	5	F:GO:0050113; C:GO:0005737; F:GO:0005506; P:GO:0019310;	F:inositol oxygenase activity; C:cytoplasm; F:iron ion binding; P:inositol catabolic process; P:oxidation-reduction process
Acrdi.XM_015905482.1/1-684	Monca.augustus.g28292.t1/1-663	0.0832268	0.134915	ETS domain-containing Elk-1	13	C:GO:0036477; P:GO:0006351; F:GO:0000978; P:GO:0009314;	[illegible]
Acrdi.XM_015922210.1/1-1338	Monca.augustus.g9706.t1/1-1803	0.160906	0.134903	cholinesterase 1-like isoform X2	8	F:GO:0005515; P:GO:0044708; P:GO:0050808; C:GO:0016021;	[illegible]
Acrdi.XM_015905459.1/1-2781	Monca.augustus.g40452.t1/1-3153	0.156174	0.134894	MORC family CW-type zinc finger 2 isoform X1			
Acrdi.XM_015910768.1/1-8073	Monca.augustus.g47639.t1/1-12672	0.188333	0.134872	Collagen alpha-1(XII) chain			
Acrdi.XM_015903351.1/1-1041	Monca.augustus.g36014.t1/1-1050	0.142434	0.134867	histamine H2 receptor-like	3	P:GO:0007165; C:GO:0016020; F:GO:0004871	P:signal transduction; C:membrane; F:signal transducer activity
Acrdi.XM_015921278.1/1-1248	Monca.augustus.g40349.t1/1-1551	0.155744	0.134865	acid-sensing ion channel 1-like	2	P:GO:0006814; C:GO:0016020	P:sodium ion transport; C:membrane
Acrdi.XM_015922018.1/1-2763	Monca.augustus.g46429.t1/1-8088	0.161486	0.134861	spatacsin isoform X3			
Acrdi.XM_015899370.1/1-1674	Monca.augustus.g44796.t1/1-1680	0.180943	0.134859	kelch 20	3	F:GO:0004842; C:GO:0031463; P:GO:0016567	F:ubiquitin-protein transferase activity; C:Cul3-RING ubiquitin ligase complex; P:protein ubiquitination
Acrdi.XM_015911333.1/1-837	Monca.augustus.g35279.t1/1-1005	0.241328	0.13483	dihydroorotate dehydrogenase			
Acrdi.XM_015907534.1/1-3129	Monca.augustus.g45764.t1/1-1785	0.133902	0.134826	TELO2-interacting 1 homolog			
Acrdi.XM_015904615.1/1-1065	Monca.augustus.g7188.t1/1-1269	0.132781	0.134814	mitochondrial-like	3	F:GO:0004325; C:GO:0005739; P:GO:0006783	F:ferrochelatase activity; C:mitochondrion; P:heme biosynthetic process
Acrdi.XM_015899425.1/1-2640	Monca.augustus.g17682.t1/1-2754	0.193499	0.134803	rab GTPase-binding effector 1-like	1	F:GO:0046872	F:metal ion binding
Acrdi.XM_015895266.1/1-1095	Monca.augustus.g12981.t1/1-1095	0.658665	0.134788	enterotoxin	3	C:GO:0016020; C:GO:0016021; P:GO:0009405	C:membrane; C:integral component of membrane; P:pathogenesis
Acrdi.XM_015895699.1/1-573	Monca.augustus.g2244.t1/1-573	0.143415	0.134786	COMM domain-containing 8			
Acrdi.XM_015915751.1/1-1002	Monca.augustus.g43211.t1/1-1398	0.178125	0.134782	PREDICTED: uncharacterized protein LOC107349566	4	P:GO:0007160; C:GO:0016020; C:GO:0016021; C:GO:0009986	P:cell-matrix adhesion; C:membrane; C:integral component of membrane; C:cell surface
Acrdi.XM_015903981.1/1-2223	Monca.augustus.g27733.t1/1-2238	0.147157	0.134775	NHL repeat-containing 2			
Acrdi.XM_015914292.1/1-1431	Monca.adi2mcaRNA24820_R0/1-1440	0.199759	0.134762	m7 -mRNA hydrolase	4	P:GO:0000290; C:GO:0000932; F:GO:0050072; F:GO:0005488	P:deadenylation-dependent decapping of nuclear-transcribed mRNA; C:P-body; F:m7G(5')pppN diphosphatase activity; F:binding
Acrdi.XM_015897028.1/1-873	Monca.adi2mcaRNA5934_R0/1-1143	0.1955	0.134762	PREDICTED: uncharacterized protein LOC107332300	2	C:GO:0016020; F:GO:0016787	C:membrane; F:hydrolase activity
Acrdi.XM_015896208.1/1-2409	Monca.augustus.g11211.t1/1-2409	0.193853	0.134761	PREDICTED: uncharacterized protein LOC107331603 isoform X1			
Acrdi.XM_015922237.1/1-561	Monca.adi2mcaRNA33671_R1/1-750	0.637143	0.134754	PREDICTED: uncharacterized protein LOC107355654	4	F:GO:0004930; P:GO:0007186; C:GO:0016020; C:GO:0016021	[illegible]
Acrdi.XM_015919527.1/1-2730	Monca.augustus.g36912.t1/1-3417	0.190187	0.134739	Nuclear pore complex Nup133	1	C:GO:0044446	C:intracellular organelle part
Acrdi.XM_015898971.1/1-867	Monca.augustus.g38002.t1/1-867	0.185669	0.134691	uncharacterized LOC103187628	4	C:GO:0005737; C:GO:0016020; F:GO:0008757; P:GO:0032259	C:cytoplasm; C:membrane; F:S-adenosylmethionine-dependent methyltransferase activity; P:methylation
Acrdi.XM_015908942.1/1-3291	Monca.augustus.g31857.t1/1-3456	0.160619	0.134669	probable ATP-dependent RNA helicase DHX37	6	F:GO:0000166; F:GO:0003676; C:GO:0005737; P:GO:0006396;	F:nucleotide binding; F:nucleic acid binding; C:cytoplasm; P:RNA processing; C:nucleolus; F:ATP-dependent RNA helicase activity
Acrdi.XM_015911303.1/1-984	Monca.augustus.g18335.t1/1-984	0.132997	0.134658	TPA: opsin	4	F:GO:0004930; P:GO:0007165; P:GO:0009583; C:GO:0016021	F:G-protein coupled receptor activity; P:signal transduction; P:detection of light stimulus; C:integral component of membrane
Acrdi.XM_015918236.1/1-3843	Monca.augustus.g48574.t1/1-3879	0.179154	0.134646	linear gramicidin synthase subunit D-like	2	F:GO:0003824; P:GO:0008152	F:catalytic activity; P:metabolic process
Acrdi.XM_015902061.1/1-1290	Monca.augustus.g43511.t1/1-1272	0.10327	0.134639	potassium voltage-gated channel subfamily A member 2-like isoform :	5	P:GO:0006813; P:GO:0055085; F:GO:0005251; C:GO:0016021;	[illegible]
Acrdi.XM_015923903.1/1-771	Monca.augustus.g4417.t1/1-1137	0.146658	0.134621	PREDICTED: uncharacterized protein LOC107357265			
Acrdi.XM_015901838.1/1-3495	Monca.augustus.g56957.t1/1-4203	0.163721	0.134614	leucine-rich repeat-containing 9-like	1	F:GO:0003950	F:NAD+ ADP-ribosyltransferase activity
Acrdi.XM_015916935.1/1-1590	Monca.adi2mcaRNA27804_R0/1-1584	0.146125	0.134609	major facilitator superfamily domain-containing 12-like	1	C:GO:0016021	C:integral component of membrane
Acrdi.XM_015901894.1/1-1602	Monca.augustus.g17431.t1/1-1596	0.181976	0.134592	zinc finger 474-like			
Acrdi.XM_015908766.1/1-3969	Monca.adi2mcaRNA18727_R0/1-3846	0.172007	0.134566	EGF-like repeat and discoidin I-like domain-containing 3	1	F:GO:0070492	F:oligosaccharide binding
Acrdi.XM_015913601.1/1-972	Monca.augustus.g10362.t1/1-963	0.139945	0.134537	rolling stone-like	2	C:GO:0016020; C:GO:0016021	C:membrane; C:integral component of membrane
Acrdi.XM_015904247.1/1-783	Monca.augustus.g51900.t1/1-600	0.841743	0.134526	ARL14 effector -like			
Acrdi.XM_015918944.1/1-2973	Monca.augustus.g66309.t1/1-5763	0.192041	0.134513	PHD finger 3 isoform X5	6	P:GO:0008150; F:GO:0008270; P:GO:0006351; F:GO:0046872;	[illegible]
Acrdi.XM_015899063.1/1-2862	Monca.augustus.g8513.t1/1-2874	0.192572	0.134504	Aminopeptidase N	7	F:GO:0008270; P:GO:0043171; C:GO:0005737; P:GO:0006508;	[illegible]
Acrdi.XM_015904360.1/1-624	Monca.augustus.g59956.t1/1-1626	0.203175	0.134503	lysine-specific demethylase 8-like			
Acrdi.XM_015918546.1/1-966	Monca.augustus.g19963.t1/1-1017	0.198696	0.1345	2,4-dienoyl- mitochondrial			
Acrdi.XM_015923702.1/1-831	Monca.augustus.g34962.t1/1-825	0.0960774	0.134495	collagen alpha-1(XII) chain-like			
Acrdi.XM_015897645.1/1-1632	Monca.augustus.g13628.t1/1-3255	0.189793	0.134472	Integrator complex subunit 5			
Acrdi.XM_015894834.1/1-843	Monca.augustus.g58524.t1/1-1140	0.109961	0.134424	UPF0661 TPR repeat-containing -like			
Acrdi.XM_015924767.1/1-1929	Monca.augustus.g9569.t1/1-2625	0.14732	0.13442	vasa	3	F:GO:0000166; F:GO:0046872; F:GO:0016787	F:nucleotide binding; F:metal ion binding; F:hydrolase activity
Acrdi.XM_015899093.1/1-1599	Monca.augustus.g26242.t1/1-1572	0.153149	0.134419	CAS1 domain-containing 1-like	1	C:GO:0016021	C:integral component of membrane
Acrdi.XM_015906727.1/1-1188	Monca.augustus.g17883.t1/1-1176	0.169285	0.134393	clavaminate synthase At3g21360-like	2	F:GO:0016491; P:GO:0055114	F:oxidoreductase activity; P:oxidation-reduction process
Acrdi.XM_015897337.1/1-1149	Monca.augustus.g48434.t1/1-2253	0.202349	0.134336	zinc finger 468 isoform X3	1	F:GO:0005488	F:binding
Acrdi.XM_015912824.1/1-489	Monca.adi2mcaRNA23203_R0/1-525	0.74403	0.134246	PREDICTED: uncharacterized protein LOC107346954			
Acrdi.XM_015905813.1/1-1638	Monca.augustus.g31747.t1/1-1692	0.12004	0.134221	numb homolog isoform X2	10	P:GO:0007405; F:GO:0005515; P:GO:0030900; P:GO:0032879;	[illegible]
Acrdi.XM_015911448.1/1-1557	Monca.augustus.g53636.t1/1-1530	0.145255	0.134204	UDP- c:beta-1,3-N-acetylgalactosaminyltransferase	8	F:GO:0000030; C:GO:0043231; F:GO:0035251; P:GO:0006486;	[illegible]
Acrdi.XM_015919950.1/1-2802	Monca.adi2mcaRNA31180_R1/1-2916	0.16327	0.134202	Voltage-dependent L-type calcium channel subunit alpha-1C	2	F:GO:0003824; F:GO:0004553	F:catalytic activity; F:hydrolase activity, hydrolyzing O-glycosyl compounds
Acrdi.XM_015924079.1/1-1563	Monca.augustus.g55719.t1/1-1560	0.15032	0.134181	pantetheinase	15	P:GO:0016337; F:GO:0016811; P:GO:0043603; P:GO:0033089;	[illegible]
Acrdi.XM_015899283.1/1-903	Monca.augustus.g64426.t1/1-984	0.126761	0.134179	sideroflexin-5-like	2	C:GO:0016020; P:GO:0006810	C:membrane; P:transport
Acrdi.XM_015898556.1/1-624	Monca.augustus.g33987.t1/1-1179	0.222888	0.134177	histone-lysine N-methyltransferase PRDM6	1	F:GO:0005488	F:binding
Acrdi.XM_015909953.1/1-1020	Monca.adi2mcaRNA20069_R6/1-864	0.639519	0.134167	PREDICTED: uncharacterized protein LOC107344300			
Acrdi.XM_015922984.1/1-1113	Monca.augustus.g67840.t1/1-1107	0.167578	0.134167	ceramide synthase 6 isoform X2	3	F:GO:0050291; P:GO:0046513; C:GO:0005783	F:sphingosine N-acyltransferase activity; P:ceramide biosynthetic process; C:endoplasmic reticulum
Acrdi.XM_015891640.1/1-4008	Monca.augustus.g43737.t1/1-4032	0.179952	0.134152	Serine threonine- kinase ULK4	2	P:GO:1902531; P:GO:0009987	P:regulation of intracellular signal transduction; P:cellular process
Acrdi.XM_015903822.1/1-1962	Monca.augustus.g33838.t1/1-1923	0.134521	0.134129	Mucosa-associated lymphoid tissue lymphoma translocation 1	2	P:GO:0006508; F:GO:0004197	P:proteolysis; F:cysteine-type endopeptidase activity
Acrdi.XM_015913278.1/1-900	Monca.augustus.g7504.t1/1-1218	0.1464	0.134116	Coiled-coil domain-containing 17			
Acrdi.XM_015899124.1/1-645	Monca.augustus.g69572.t1/1-642	0.110521	0.134095	Zinc finger CCHC domain-containing 24			
Acrdi.XM_015923931.1/1-4482	Monca.augustus.g32965.t1/1-4875	0.14294	0.134087	speract receptor-like	7	C:GO:0008074; F:GO:0000166; P:GO:0006182; P:GO:0007165;	[illegible]
Acrdi.XM_015902724.1/1-498	Monca.adi2mcaRNA12093_R5/1-360	1.2876	0.134078	Transposon Tf2-9 poly			
Acrdi.XM_015902864.1/1-2724	Monca.adi2mcaRNA12307_R0/1-2562	0.197939	0.134047	Transposon Tf2-6 poly			
Acrdi.XM_015906251.1/1-1923	Monca.augustus.g21667.t1/1-1920	0.184857	0.134046	leucine-rich repeat-containing 43-like			
Acrdi.XM_015895516.1/1-993	Monca.augustus.g25968.t1/1-951	0.100142	0.134035	melatonin receptor type 1B-like	4	C:GO:0005887; F:GO:0004930; P:GO:0007186; P:GO:0007166	[illegible]
Acrdi.XM_015905922.1/1-1257	Monca.augustus.g58495.t1/1-1233	0.154832	0.134029	STE20-related kinase adapter alpha isoform X3	4	P:GO:0050794; F:GO:0004672; P:GO:0016310; C:GO:0044424	[illegible]
Acrdi.XM_015910709.1/1-1746	Monca.adi2mcaRNA2139_R0/1-1569	0.141736	0.134025	Alpha-1,6-mannosylglyco 6-beta-N-acetylglucosaminyltransferase A	2	P:GO:0006487; F:GO:0030144	[illegible]
Acrdi.XM_015910357.1/1-2154	Monca.augustus.g36433.t1/1-2853	0.179153	0.13399	inactive cadmium zinc-transporting ATPase HMA3	9	P:GO:0070838; C:GO:0005887; P:GO:0098662; F:GO:0019829;	[illegible]
Acrdi.XM_015918694.1/1-633	Monca.augustus.g11397.t1/1-810	0.673391	0.133983	tigger transposable element-derived 6-like			
Acrdi.XM_015901658.1/1-1716	Monca.augustus.g54283.t1/1-1980	0.0647907	0.133977	myotubularin-related 10-like			
Acrdi.XM_015913079.1/1-1677	Monca.augustus.g25062.t1/1-1668	0.157823	0.133965	PREDICTED: uncharacterized protein LOC107347197			
Acrdi.XM_015895932.1/1-849	Monca.augustus.g58273.t1/1-870	0.155126	0.133911	leucine-rich repeat-containing 57-like			
Acrdi.XM_015917978.1/1-2625	Monca.adi2mcaRNA29018_R0/1-2265	0.152628	0.133911	Condensin complex subunit 2	1	P:GO:0044763	P:single-organism cellular process
Acrdi.XM_015896284.1/1-606	Monca.augustus.g33693.t1/1-615	0.176236	0.133902	ras-related Rab-14	2	P:GO:0007264; C:GO:0043025	P:small GTPase mediated signal transduction; C:neuronal cell body
Acrdi.XM_015901619.1/1-435	Monca.augustus.g26165.t1/1-372	0.0282268	0.133889	DNA polymerase	3	P:GO:0090304; F:GO:0097159; F:GO:1901363	[illegible]
Acrdi.XM_015903765.1/1-876	Monca.adi2mcaRNA14717_R0/1-714	0.180842	0.133875	Leucine-rich repeat-containing 57	1	C:GO:0070062	C:extracellular exosome
Acrdi.XM_015899828.1/1-987	Monca.augustus.g53429.t1/1-981	0.132086	0.133873	kiSS-1 receptor-like isoform X2	3	F:GO:0004930; P:GO:0007186; C:GO:0016020	[illegible]
Acrdi.XM_015907312.1/1-2664	Monca.augustus.g31822.t1/1-2940	0.150917	0.133861	ankyrin repeat and IBR domain-containing 1 isoform X4	4	F:GO:0004842; F:GO:0005488; C:GO:0044424; P:GO:0016567	[illegible]
Acrdi.XM_015919320.1/1-678	Monca.augustus.g5210.t1/1-1947	0.148352	0.133861	PREDICTED: uncharacterized protein LOC107352995	3	F:GO:0003779; P:GO:0007010; P:GO:0000902	F:actin binding; P:cytoskeleton organization; P:cell morphogenesis
Acrdi.XM_015907301.1/1-1872	Monca.augustus.g12352.t1/1-2502	0.153371	0.133841	PREDICTED: uncharacterized protein LOC107341826	1	P:GO:0007165	P:signal transduction
Acrdi.XM_015922071.1/1-2886	Monca.augustus.g47168.t1/1-2892	0.163272	0.133831	calcium-responsive transcription factor isoform X1	5	C:GO:0005634; F:GO:0003677; F:GO:0003700; P:GO:0071277;	[illegible]
Acrdi.XM_015904898.1/1-549	Monca.augustus.g61117.t1/1-867	0.158029	0.133821	endonuclease exonuclease phosphatase family	6	P:GO:0090305; F:GO:0004527; F:GO:0005509; F:GO:0004519;	[illegible]
Acrdi.XM_015899897.1/1-840	Monca.augustus.g10904.t1/1-720	0.163359	0.13382	ATP synthase F(0) complex subunit mitochondrial	4	C:GO:0000276; F:GO:0015078; P:GO:0015986; F:GO:0046933	[illegible]
Acrdi.XM_015916181.1/1-3261	Monca.augustus.g5398.t1/1-3036	0.186636	0.133809	bicaudal C homolog 1	3	F:GO:0003676; F:GO:0003723; P:GO:0007275	[illegible]
Acrdi.XM_015892417.1/1-477	Monca.augustus.g9649.t1/1-1086	0.173585	0.133809	Transient receptor potential cation channel subfamily A member 1			
Acrdi.XM_015910584.1/1-951	Monca.augustus.g45402.t1/1-951	0.132626	0.133783	trace amine-associated receptor 13c-like	9	F:GO:0004930; F:GO:0004977; P:GO:0007186; P:GO:0007165;	[illegible]
Acrdi.XM_015917049.1/1-474	Monca.augustus.g52188.t1/1-735	0.0891268	0.133742	transposase Hi-	1	F:GO:0003676	F:nucleic acid binding
Acrdi.XM_015922256.1/1-1965	Monca.augustus.g4394.t1/1-2028	0.155525	0.133741	Transcription factor 25			
Acrdi.XM_015910356.1/1-1920	Monca.augustus.g36438.t1/1-1896	0.167184	0.133702	serine arginine repetitive matrix 2-like			
Acrdi.XM_015905270.1/1-1440	Monca.adi2mcaRNA14910_R0/1-987	0.170225	0.133696	probable dolichyl pyrophosphate Glc1Man9 c2 alpha-1,3-glucosyltransf	6	F:GO:0000033; P:GO:0006490; P:GO:0006487; C:GO:0005783;	[illegible]
Acrdi.XM_015916309.1/1-1740	Monca.augustus.g35029.t1/1-2319	0.13232	0.133691	FAM161A isoform X3			
Acrdi.XM_015910558.1/1-1290	Monca.augustus.g55374.t1/1-1428	0.127896	0.133674	F-box At1g78280-like	2	C:GO:0016020; C:GO:0016021	C:membrane; C:integral component of membrane
Acrdi.XM_015905429.1/1-2112	Monca.augustus.g36747.t1/1-2640	0.129405	0.133668	ras-associated and pleckstrin homology domains-containing 1-like	1	P:GO:0007165	P:signal transduction
Acrdi.XM_015899853.1/1-504	Monca.augustus.g52546.t1/1-504	0.175508	0.133654	pleckstrin homology domain-containing family A member 1-like			
Acrdi.XM_015911046.1/1-1674	Monca.adi2mcaRNA21272_R0/1-1620	0.163916	0.133619	SHC-transforming 1-like	5	P:GO:0007169; F:GO:0030971; P:GO:0035556; C:GO:0005622;	[illegible]
Acrdi.XM_015922504.1/1-3555	Monca.augustus.g33866.t1/1-3456	0.16107	0.133615	Phosphatidylinositide phosphatase SAC2	1	F:GO:0042578	F:phosphoric ester hydrolase activity
Acrdi.XM_015897340.1/1-471	Monca.adi2mcaRNA6235_R0/1-495	0.224908	0.133563	LITAF PIG7 possible membrane associated motif in LPS-induced tumor	3	C:GO:0016020; C:GO:0016021; C:GO:0033644	C:membrane; C:integral component of membrane; C:host cell membrane
Acrdi.XM_015912689.1/1-2064	Monca.augustus.g43032.t1/1-2238	0.140441	0.133558	procollagen-lysine,2-oxoglutarate 5-dioxygenase 2 isoform X2	3	F:GO:0016491; F:GO:0043167; C:GO:0043227	F:oxidoreductase activity; F:ion binding; C:membrane-bounded organelle
Acrdi.XM_015906531.1/1-3261	Monca.augustus.g31411.t1/1-3315	0.136344	0.133557	WD repeat and HMG-box DNA-binding 1	1	F:GO:0003677	F:DNA binding
Acrdi.XM_015895563.1/1-615	Monca.augustus.g14883.t1/1-1011	0.221285	0.133548	uridine phosphorylase 1-like	4	C:GO:0005737; P:GO:0009166; P:GO:0009116; F:GO:0004850	[illegible]
Acrdi.XM_015894121.1/1-654	Monca.augustus.g15947.t1/1-642	0.195149	0.133523	PREDICTED: uncharacterized protein LOC107329430			
Acrdi.XM_015907118.1/1-1449	Monca.augustus.g36976.t1/1-1452	0.147659	0.133523	serine threonine tyrosine-interacting 1	2	F:GO:0016791; P:GO:0016311	F:phosphatase activity; P:dephosphorylation
Acrdi.XM_015898688.1/1-1926	Monca.augustus.g2550.t1/1-2442	0.111771	0.133506	amiloride-sensitive amine oxidase [copper-containing]-like	2	F:GO:0005488; F:GO:0016641	[illegible]
Acrdi.XM_015895150.1/1-486	Monca.augustus.g54488.t1/1-666	0.131557	0.133503	ras GEF	4	P:GO:0007264; F:GO:0005085; C:GO:0005622; P:GO:0043547	[illegible]
Acrdi.XM_015898126.1/1-504	Monca.augustus.g10374.t1/1-504	0.17914	0.133488	PREDICTED: uncharacterized protein LOC107333317			
Acrdi.XM_015913442.1/1-1005	Monca.augustus.g32131.t1/1-963	0.0948354	0.133479	galanin receptor type 1-like	3	P:GO:0007165; C:GO:0016021; F:GO:0004871	[illegible]
Acrdi.XM_015908977.1/1-3555	Monca.augustus.g47240.t1/1-3939	0.170271	0.133477	Membrane progestin receptor gamma-B	2	F:GO:0003824; F:GO:0004553	F:catalytic activity; F:hydrolase activity, hydrolyzing O-glycosyl compounds
Acrdi.XM_015897650.1/1-1716	Monca.augustus.g56108.t1/1-2613	0.132749	0.133475	tetratricopeptide repeat 16-like			
Acrdi.XM_015914149.1/1-1695	Monca.augustus.g24974.t1/1-1680	0.143887	0.133474	NAD-dependent deacetylase sirtuin-3-like isoform X1	4	F:GO:0016811; F:GO:0008270; P:GO:0006476; F:GO:0070403	[illegible]
Acrdi.XM_015898145.1/1-1815	Monca.augustus.g29280.t1/1-1785	0.150485	0.133426	2 ,5 -phosphodiesterase 12	1	C:GO:0005739	C:mitochondrion
Acrdi.XM_015897721.1/1-903	Monca.augustus.g9972.t1/1-906	0.11181	0.133416	substance-K receptor-like	3	F:GO:0004930; P:GO:0007186; C:GO:0016021	[illegible]
Acrdi.XM_015902555.1/1-888	Monca.augustus.g36239.t1/1-903	0.23244	0.13341	PREDICTED: uncharacterized protein LOC107337410	3	F:GO:0030414; C:GO:0005576; P:GO:0010466	[illegible]
Acrdi.XM_015900360.1/1-1350	Monca.augustus.g63388.t1/1-1341	0.17595	0.133348	BTN1-like	6	P:GO:0051453; P:GO:0015809; P:GO:0007033; P:GO:0007034;	[illegible]
Acrdi.XM_015925099.1/1-888	Monca.augustus.g4270.t1/1-1377	0.179545	0.133339	P2X purinoceptor 4	3	F:GO:0004931; C:GO:0005639; F:GO:0001614	[illegible]
Acrdi.XM_015922655.1/1-399	Monca.augustus.g46837.t1/1-666	0.226665	0.133267	PREDICTED: uncharacterized protein LOC107356015			
Acrdi.XM_015918117.1/1-1107	Monca.augustus.g35394.t1/1-1734	0.155716	0.133189	signal CUB and EGF-like domain-containing 2	4	P:GO:0007160; F:GO:0005509; C:GO:0016020; C:GO:0016021	[illegible]
Acrdi.XM_015922693.1/1-1164	Monca.augustus.g1938.t1/1-1206	0.175025	0.133139	synaptotagmin-6 isoform X2	9	C:GO:0070382; P:GO:0048791; F:GO:0019905; P:GO:0006906;	[illegible]
Acrdi.XM_015907236.1/1-1248	Monca.augustus.g7058.t1/1-1254	0.157515	0.133134	BTN1-like	2	C:GO:0016020; C:GO:0043227	C:membrane; C:membrane-bounded organelle
Acrdi.XM_015896246.1/1-825	Monca.augustus.g33757.t1/1-792	0.169411	0.13313	Palmitoyltransferase ZDHHC3	3	F:GO:0008270; F:GO:0019706; C:GO:0016021	[illegible]
Acrdi.XM_015899816.1/1-1860	Monca.augustus.g48619.t1/1-2205	0.125979	0.133119	cyclin-F-like isoform X3	4	C:GO:0005634; C:GO:0016020; C:GO:0016021; P:GO:0051726	[illegible]
Acrdi.XM_015914471.1/1-1608	Monca.adi2mcaRNA25036_R0/1-1605	0.208053	0.133099	Hermansky-Pudlak syndrome 1			
Acrdi.XM_015904415.1/1-435	Monca.augustus.g48079.t1/1-579	0.0283492	0.133089	EF-hand domain-containing D1	1	C:GO:0005739	C:mitochondrion
Acrdi.XM_015903407.1/1-1101	Monca.augustus.g35983.t1/1-1281	0.0868995	0.133054	lysocardiolipin acyltransferase 1	1	C:GO:0016020	C:membrane
Acrdi.XM_015907785.1/1-1392	Monca.augustus.g55791.t1/1-1380	0.123919	0.133037	potassium voltage-gated channel subfamily D member 2-like	3	F:GO:0005249; P:GO:0006811; C:GO:0016021	[illegible]
Acrdi.XM_015923005.1/1-2970	Monca.adi2mcaRNA34600_R0/1-2736	0.171246	0.133032	E2 E3 hybrid ubiquitin- ligase UBE2O	4	F:GO:0061630; F:GO:0031625; C:GO:0005737; P:GO:0016567	[illegible]
Acrdi.XM_015920698.1/1-2094	Monca.augustus.g33768.t1/1-1131	0.167529	0.133027	Exocyst complex component 5	2	P:GO:0048731; P:GO:0044763	P:system development; P:single-organism cellular process
Acrdi.XM_015900562.1/1-1236	Monca.augustus.g10464.t1/1-1257	0.156336	0.133002	F-box only 22			
Acrdi.XM_015903396.1/1-1173	Monca.augustus.g36019.t1/1-1209	0.150394	0.132957	histamine H2 receptor-like	3	P:GO:0007165; C:GO:0016020; F:GO:0004871	P:signal transduction; C:membrane; F:signal transducer activity
Acrdi.XM_015907021.1/1-456	Monca.augustus.g43292.t1/1-624	0.119991	0.13295	N-acetyltransferase 9	2	P:GO:0006474; F:GO:0008080	P:N-terminal protein amino acid acetylation; F:N-acetyltransferase activity
Acrdi.XM_015924324.1/1-2532	Monca.augustus.g45880.t1/1-1083	0.0775052	0.13295	52 kDa repressor of the inhibitor of the kinase			
Acrdi.XM_015921430.1/1-1605	Monca.augustus.g23823.t1/1-2259	0.181284	0.132941	tetratricopeptide repeat 12			
Acrdi.XM_015912879.1/1-399	Monca.augustus.g14932.t1/1-1218	0.170423	0.132914	mitotic spindle assembly checkpoint MAD1-like			
Acrdi.XM_015905291.1/1-1479	Monca.augustus.g23138.t1/1-1656	0.206062	0.132855	phosphatidylinositol 3,4,5-trisphosphate 3-phosphatase TPTE2-like	8	F:GO:0005216; F:GO:0004725; C:GO:0016021; P:GO:0006810;	[illegible]
Acrdi.XM_015893361.1/1-834	Monca.augustus.g52742.t1/1-4464	0.173606	0.132843	crossover junction endonuclease MUS81-like	8	F:GO:0003677; P:GO:0090305; P:GO:0006281; C:GO:0005634;	[illegible]
Acrdi.XM_015912020.1/1-4833	Monca.augustus.g67638.t1/1-6366	0.169974	0.132762	autophagy-related 2 homolog B isoform X2			
Acrdi.XM_015918783.1/1-1638	Monca.augustus.g61754.t1/1-1653	0.144369	0.132757	activin receptor type-1C isoform X1	1	F:GO:0016301	F:kinase activity
Acrdi.XM_015901545.1/1-1089	Monca.augustus.g14607.t1/1-1572	0.177262	0.132718	Lipoamide acyltransferase component of branched-chain alpha-keto ac	3	F:GO:0016746; P:GO:0008152; F:GO:0016740	[illegible]
Acrdi.XM_015911254.1/1-2064	Monca.augustus.g59543.t1/1-2424	0.203114	0.132708	probable serine threonine- kinase pats1	14	P:GO:0055085; P:GO:0007165; P:GO:0007264; P:GO:0006355;	[illegible]
Acrdi.XM_015925342.1/1-984	Monca.augustus.g21151.t1/1-1002	0.136278	0.132688	PIH1 domain-containing 2			
Acrdi.XM_015920316.1/1-4620	Monca.augustus.g24360.t1/1-7227	0.13248	0.132678	trichohyalin-like isoform X1			
Acrdi.XM_015910046.1/1-969	Monca.augustus.g36887.t1/1-963	0.187317	0.132613	vasohibin-2 isoform X1	2	C:GO:0005737; P:GO:0045765	C:cytoplasm; P:regulation of angiogenesis
Acrdi.XM_015924164.1/1-831	Monca.adi2mcaRNA35908_R0/1-834	0.220714	0.132609	cyclin-I-like isoform X2	1	P:GO:0051726	P:regulation of cell cycle
Acrdi.XM_015915261.1/1-885	Monca.augustus.g41255.t1/1-984	0.18011	0.132606	Nucleoside diphosphate-linked moiety X motif 17	1	F:GO:0047429	F:nucleoside-triphosphate diphosphatase activity
Acrdi.XM_015913590.1/1-921	Monca.augustus.g26386.t1/1-921	0.150903	0.132565	Transmembrane protease serine 11D	2	P:GO:0006508; F:GO:0004252	P:proteolysis; F:serine-type endopeptidase activity
Acrdi.XM_015910522.1/1-1182	Monca.augustus.g65045.t1/1-1644	0.143218	0.13255	suppressor of tumorigenicity 7 isoform X2	1	C:GO:0016021	C:integral component of membrane
Acrdi.XM_015913146.1/1-3108	Monca.adi2mcaRNA23565_R0/1-3081	0.153561	0.132548	endoplasmic reticulum aminopeptidase 2 isoform X1	6	C:GO:0016020; C:GO:0044464; F:GO:0004177; F:GO:0005488;	[illegible]
Acrdi.XM_015916632.1/1-1758	Monca.augustus.g15413.t1/1-2133	0.0917389	0.132548	Phospholipase DDHD1	1	F:GO:0046872	F:metal ion binding
Acrdi.XM_015913691.1/1-435	Monca.augustus.g30473.t1/1-456	0.136755	0.132544	PREDICTED: uncharacterized protein LOC107347730			
Acrdi.XM_015924368.1/1-1086	Monca.augustus.g30191.t1/1-1416	0.125522	0.132529	Lysine-specific demethylase 8	1	F:GO:0005509	F:calcium ion binding
Acrdi.XM_015919481.1/1-2544	Monca.augustus.g59634.t1/1-2655	0.16954	0.132527	PAT1 homolog 1	1	P:GO:0009987	P:cellular process
Acrdi.XM_015904770.1/1-10935	Monca.augustus.g59067.t1/1-11733	0.194228	0.132515	golgin subfamily B member 1-like isoform X1			
Acrdi.XM_015917663.1/1-1536	Monca.adi2mcaRNA12754_R0/1-2514	0.176373	0.132506	beta,beta-carotene 15,15 -dioxygenase-like	2	F:GO:0016702; P:GO:0055114	[illegible]
Acrdi.XM_015915969.1/1-780	Monca.augustus.g51186.t1/1-795	0.160367	0.132501	carbonic anhydrase 2	13	F:GO:0008270; P:GO:0038166; F:GO:0004064; F:GO:0004089;	[illegible]
Acrdi.XM_015905896.1/1-1353	Monca.augustus.g58515.t1/1-5865	0.195916	0.13248	telomere-associated RIF1 isoform X2	1	C:GO:0043229	C:intracellular organelle
Acrdi.XM_015924169.1/1-2595	Monca.augustus.g5178.t1/1-4482	0.172024	0.132456	FK506-binding 15	6	C:GO:0030426; P:GO:0010923; C:GO:0016020; C:GO:0005884;	[illegible]
Acrdi.XM_015898454.1/1-1881	Monca.augustus.g34006.t1/1-5049	0.155259	0.132451	calmodulin-binding carboxy-terminal kinesin	4	F:GO:0005524; F:GO:0003777; P:GO:0007018; F:GO:0008017	[illegible]
Acrdi.XM_015919655.1/1-2226	Monca.augustus.g48585.t1/1-2238	0.146617	0.13242	prestin isoform X1	9	P:GO:0065008; P:GO:0098661; C:GO:0016021; P:GO:0015291;	[illegible]
Acrdi.XM_015897242.1/1-1050	Monca.augustus.g22258.t1/1-3591	0.136112	0.132374	Centrosomal of 128 kDa			
Acrdi.XM_015911425.1/1-1767	Monca.augustus.g7438.t1/1-1776	0.157575	0.132355	E3 ubiquitin- ligase TRIM71-like	3	F:GO:0008270; F:GO:0046872; C:GO:0005622	F:zinc ion binding; F:metal ion binding; C:intracellular
Acrdi.XM_015906453.1/1-1461	Monca.augustus.g831.t1/1-1530	0.146542	0.132317	Schwannomin-interacting 1			
Acrdi.XM_015914385.1/1-1344	Monca.augustus.g60734.t1/1-1365	0.178664	0.132312	Prostatic acid phosphatase	4	C:GO:0016020; C:GO:0016021; P:GO:0016311; F:GO:0003993	[illegible]
Acrdi.XM_015924894.1/1-4446	Monca.augustus.g35000.t1/1-4710	0.165395	0.132312	neurogenic locus notch homolog 1-	4	F:GO:0008270; F:GO:0005509; C:GO:0016020; C:GO:0016021	[illegible]
Acrdi.XM_015919014.1/1-852	Monca.augustus.g32017.t1/1-864	0.0903345	0.132308	substance-K receptor-like	4	C:GO:0005887; F:GO:0004930; P:GO:0007186; P:GO:0007166	[illegible]
Acrdi.XM_015907866.1/1-1134	Monca.adi2mcaRNA17773_R0/1-1134	0.13432	0.132296	Armadillo repeat-containing 6			
Acrdi.XM_015914725.1/1-450	Monca.augustus.g22507.t1/1-444	0.17002	0.132286	Fanconi anemia group M	1	F:GO:0043167	F:ion binding
Acrdi.XM_015910810.1/1-1842	Monca.augustus.g14054.t1/1-552	0.133944	0.132272	sorting and assembly machinery component 50 homolog	2	C:GO:0016020; C:GO:0043227	C:membrane; C:membrane-bounded organelle
Acrdi.XM_015898049.1/1-4734	Monca.augustus.g39419.t1/1-4704	0.211436	0.132264	rho guanine nucleotide exchange factor 4 isoform X6	5	P:GO:0035023; F:GO:0005089; C:GO:0005622; P:GO:0035556;	[illegible]
Acrdi.XM_015913454.1/1-4512	Monca.adi2mcaRNA23910_R1/1-3837	0.171296	0.132261	Collagen alpha-6(VI) chain			
Acrdi.XM_015912718.1/1-1350	Monca.augustus.g11957.t1/1-1341	0.124282	0.132256	odorant gustatory chemosensory receptor-like 122	5	P:GO:0050909; F:GO:0008527; C:GO:0016020; C:GO:0016021;	[illegible]
Acrdi.XM_015908742.1/1-1080	Monca.augustus.g41901.t1/1-1047	0.17274	0.132249	Coagulation factor VIII	1	F:GO:0070492	F:oligosaccharide binding
Acrdi.XM_015917550.1/1-786	Monca.augustus.g25138.t1/1-816	0.200813	0.132211	proteasome subunit beta type-4-like	5	C:GO:0005634; C:GO:0005737; C:GO:0005839; F:GO:0004298;	[illegible]
Acrdi.XM_015917886.1/1-1083	Monca.augustus.g39692.t1/1-1278	0.144134	0.132197	pachytene checkpoint 2 homolog			
Acrdi.XM_015898168.1/1-435	Monca.augustus.g29268.t1/1-432	0.133891	0.132197	lipopolysaccharide-induced tumor necrosis factor-alpha factor	2	C:GO:0043231; P:GO:0050789	[illegible]
Acrdi.XM_015914143.1/1-354	Monca.augustus.g25015.t1/1-354	0.145531	0.132186	myotrophin	1	C:GO:0005737	C:cytoplasm
Acrdi.XM_015904829.1/1-333	Monca.adi2mcaRNA14346_R1/1-339	0.133809	0.132153	UPF0545 C22orf39 homolog			
Acrdi.XM_015906401.1/1-495	Monca.augustus.g22817.t1/1-1539	0.218492	0.132143	C immunoglobulin-A-binding beta			
Acrdi.XM_015901446.1/1-504	Monca.augustus.g46703.t1/1-504	0.0792687	0.132137	2Fe-2S ferredoxin-like	7	F:GO:0046872; P:GO:0051188; F:GO:0009055; P:GO:1901576;	[illegible]
Acrdi.XM_015909840.1/1-1899	Monca.augustus.g46899.t1/1-2118	0.11931	0.132115	prolyl 3-hydroxylase 1-like	5	F:GO:0031418; F:GO:0005506; F:GO:0016491; F:GO:0016705;	[illegible]
Acrdi.XM_015901855.1/1-513	Monca.augustus.g4670.t1/1-552	0.195801	0.132104	Translation machinery-associated 16			
Acrdi.XM_015910469.1/1-1440	Monca.augustus.g45865.t1/1-1458	0.173373	0.132102	disrupted in renal carcinoma 2	1	C:GO:0016020	C:membrane
Acrdi.XM_015906512.1/1-999	Monca.augustus.g22559.t1/1-975	0.12183	0.132091	5-hydroxytryptamine receptor 1A-alpha-like	3	F:GO:0004930; P:GO:0007154; P:GO:0044700	[illegible]
Acrdi.XM_015922852.1/1-3150	Monca.augustus.g53607.t1/1-3267	0.159004	0.132062	ubinuclein-1 isoform X3	1	C:GO:0005634	C:nucleus
Acrdi.XM_015911758.1/1-2415	Monca.augustus.g30201.t1/1-2400	0.124379	0.132009	PREDICTED: uncharacterized protein LOC107345993	2	C:GO:0016020; C:GO:0016021	C:membrane; C:integral component of membrane
Acrdi.XM_015923687.1/1-2049	Monca.augustus.g34975.t1/1-2037	0.157801	0.132001	Dihydroxyacetone phosphate acyltransferase	2	F:GO:0008374; P:GO:0044255	F:O-acyltransferase activity; P:cellular lipid metabolic process
Acrdi.XM_015906410.1/1-1653	Monca.adi2mcaRNA16147_R0/1-1656	0.140791	0.131952	Unconventional myosin-	6	F:GO:0000166; C:GO:0016459; F:GO:0005524; C:GO:0005856;	[illegible]
Acrdi.XM_015899401.1/1-2919	Monca.augustus.g37238.t1/1-2817	0.197605	0.13195	DNA repair and recombination RAD54B	1	F:GO:0005524	F:ATP binding
Acrdi.XM_015915317.1/1-1782	Monca.augustus.g25284.t1/1-2085	0.160477	0.131925	transforming growth factor-beta-induced ig-h3-like	1	C:GO:0044421	C:extracellular region part
Acrdi.XM_015919186.1/1-1218	Monca.augustus.g54966.t1/1-1614	0.183032	0.131893	Leucine-rich repeat-containing 71			
Acrdi.XM_015919129.1/1-444	Monca.augustus.g35160.t1/1-453	0.167432	0.131876	CCAAT enhancer-binding beta-like	4	F:GO:0003700; P:GO:0006351; P:GO:0006355; F:GO:0043565	[illegible]
Acrdi.XM_015913211.1/1-1461	Monca.augustus.g32066.t1/1-1461	0.152409	0.131858	acid-sensing ion channel 3-like	1	P:GO:0006810	P:transport
Acrdi.XM_015905356.1/1-753	Monca.adi2mcaRNA14986_R0/1-696	0.112078	0.131821	PREDICTED: uncharacterized protein LOC107340003			
Acrdi.XM_015910702.1/1-1989	Monca.augustus.g35868.t1/1-1995	0.182919	0.131778	nuclear RNA export factor 1	4	P:GO:0006406; F:GO:0097159; F:GO:1901363; C:GO:0044424	[illegible]
Acrdi.XM_015906220.1/1-948	Monca.augustus.g21691.t1/1-1101	0.170774	0.13176	1-acyl-sn-glycerol-3-phosphate acyltransferase epsilon	9	F:GO:0003841; F:GO:0016746; P:GO:0006639; P:GO:0008152;	[illegible]
Acrdi.XM_015901214.1/1-336	Monca.augustus.g62606.t1/1-342	0.127965	0.131634	cytochrome b-c1 complex subunit mitochondrial	3	F:GO:0016491; C:GO:0016020; P:GO:0045333	F:oxidoreductase activity; C:membrane; P:cellular respiration
Acrdi.XM_015894106.1/1-1623	Monca.adi2mcaRNA40326_R0/1-1605	0.165838	0.131632	WD repeat-containing 7-			
Acrdi.XM_015897178.1/1-1143	Monca.adi2mcaRNA192_R1/1-1068	0.146066	0.131632	Golgi-associated plant pathogenesis-related 1			
Acrdi.XM_015919027.1/1-1395	Monca.augustus.g33664.t1/1-1395	0.162806	0.131616	Ornithine decarboxylase	8	C:GO:0048471; F:GO:0042803; P:GO:0001822; P:GO:0008284;	[illegible]
Acrdi.XM_015892652.1/1-759	Monca.augustus.g22627.t1/1-873	0.145487	0.1316	Soluble NSF attachment	8	P:GO:0061025; C:GO:0070062; F:GO:0019905; P:GO:0035494;	[illegible]
Acrdi.XM_015899389.1/1-2403	Monca.adi2mcaRNA8518_R0/1-2454	0.153246	0.131578	L-rhamnose-binding lectin CSL1-like	1	F:GO:0030246	F:carbohydrate binding
Acrdi.XM_015911199.1/1-1092	Monca.augustus.g62300.t1/1-522	0.143997	0.131573	N(4)-(Beta-N-acetylglucosaminyl)-L-asparaginase-like isoform X1	1	F:GO:0016787	F:hydrolase activity
Acrdi.XM_015902568.1/1-2226	Monca.augustus.g39922.t1/1-2874	0.144201	0.131534	gamma-secretase-activating -like			
Acrdi.XM_015906045.1/1-1596	Monca.augustus.g34420.t1/1-2811	0.156251	0.131508	importin-13 isoform X2	5	C:GO:0031965; C:GO:0005737; P:GO:0006606; F:GO:0008565;	[illegible]
Acrdi.XM_015921369.1/1-1248	Monca.augustus.g47562.t1/1-1203	0.221043	0.131507	Peptide chain release factor 1	4	F:GO:0043022; C:GO:0005739; P:GO:0070126; F:GO:0016149	[illegible]
Acrdi.XM_015911660.1/1-1203	Monca.augustus.g27819.t1/1-1230	0.126146	0.131504	transmembrane prolyl 4-hydroxylase-like	2	F:GO:0016491; F:GO:0046872	F:oxidoreductase activity; F:metal ion binding
Acrdi.XM_015899687.1/1-774	Monca.augustus.g37116.t1/1-1263	0.195599	0.131495	von Willebrand factor type EGF and pentraxin domain-containing 1	5	P:GO:0007339; F:GO:0005509; C:GO:0016020; C:GO:0016021;	[illegible]
Acrdi.XM_015919782.1/1-474	Monca.augustus.g40320.t1/1-474	0.102246	0.131483	6-pyruvoyl tetrahydrobiopterin synthase	3	F:GO:0003874; P:GO:0006729; F:GO:0046872	[illegible]
Acrdi.XM_015908201.1/1-735	Monca.augustus.g4572.t1/1-1044	0.186004	0.13146	avidin-related 4 5-			
Acrdi.XM_015914760.1/1-3822	Monca.adi2mcaRNA25403_R0/1-3594	0.143576	0.131421	phospholipid-transporting ATPase IA-like	9	F:GO:0000287; F:GO:0005524; C:GO:0005802; P:GO:0045332;	[illegible]
Acrdi.XM_015910621.1/1-1044	Monca.augustus.g58073.t1/1-978	0.114683	0.131415	snRNA-activating complex subunit 1	3	C:GO:0005634; F:GO:0003677; P:GO:0009301	C:nucleus; F:DNA binding; P:snRNA transcription
Acrdi.XM_015918673.1/1-732	Monca.augustus.g13645.t1/1-1197	0.110023	0.131379	Gamma-aminobutyric acid receptor subunit beta-like	4	P:GO:0034220; C:GO:0016021; F:GO:0005230; C:GO:0005886	[illegible]
Acrdi.XM_015900838.1/1-1173	Monca.augustus.g42338.t1/1-4956	0.225167	0.131375	coiled-coil domain-containing 18			
Acrdi.XM_015910447.1/1-4302	Monca.augustus.g60939.t1/1-4731	0.150297	0.131372	Pappalysin-1	3	F:GO:0008270; P:GO:0006508; F:GO:0008237	F:zinc ion binding; P:proteolysis; F:metallopeptidase activity
Acrdi.XM_015906420.1/1-861	Monca.augustus.g242.t1/1-840	0.159604	0.131368	retinol dehydrogenase 8-like	3	F:GO:0016491; C:GO:0016020; P:GO:0008152	F:oxidoreductase activity; C:membrane; P:metabolic process
Acrdi.XM_015922997.1/1-1503	Monca.augustus.g67836.t1/1-1497	0.179393	0.131313	vesicular inhibitory amino acid transporter-like	3	C:GO:0016021; F:GO:0015171; P:GO:0003333	[illegible]
Acrdi.XM_015895837.1/1-4956	Monca.adi2mcaRNA4619_R0/1-4788	0.161	0.131295	FAM179B isoform X4			
Acrdi.XM_015893034.1/1-480	Monca.adi2mcaRNA39076_R1/1-459	0.627197	0.131275	PREDICTED: uncharacterized protein LOC107328294			
Acrdi.XM_015916343.1/1-1617	Monca.adi2mcaRNA27095_R1/1-1650	0.147507	0.131268	PREDICTED: uncharacterized protein LOC107350112			
Acrdi.XM_015910088.1/1-1926	Monca.augustus.g21996.t1/1-4479	0.106324	0.131267	Pro-Pol poly	2	F:GO:0003676; P:GO:0015074	F:nucleic acid binding; P:DNA integration
Acrdi.XM_015899249.1/1-2058	Monca.augustus.g1502.t1/1-2028	0.164445	0.131246	sodium potassium calcium exchanger 2 isoform X2	3	C:GO:0016021; F:GO:0015085; F:GO:0031420	[illegible]
Acrdi.XM_015907003.1/1-1338	Monca.augustus.g33394.t1/1-1341	0.161003	0.131223	retinoid-inducible serine carboxypeptidase-like	2	F:GO:0004185; P:GO:0051603	[illegible]
Acrdi.XM_015918348.1/1-777	Monca.augustus.g41649.t1/1-1329	0.213004	0.131209	myomegalin isoform X7			
Acrdi.XM_015912567.1/1-1665	Monca.adi2mcaRNA22933_R0/1-1551	0.202806	0.131202	sentrin-specific protease 7-like isoform X1			
Acrdi.XM_015893181.1/1-672	Monca.augustus.g21550.t1/1-999	0.774847	0.131186	tripartite motif-containing 16-like			
Acrdi.XM_015919232.1/1-855	Monca.augustus.g40932.t1/1-867	0.0952601	0.131179	Metallo-beta-lactamase domain-containing 2			
Acrdi.XM_015919097.1/1-2175	Monca.augustus.g12202.t1/1-2463	0.131987	0.131164	ARF GTPase-activating GIT2 isoform X5	2	F:GO:0005096; P:GO:0043547	F:GTPase activator activity; P:positive regulation of GTPase activity
Acrdi.XM_015911707.1/1-567	Monca.augustus.g16302.t1/1-1248	0.185985	0.131162	cat eye syndrome critical region 5-like	2	C:GO:0005739; P:GO:0046474	C:mitochondrion; P:glycerophospholipid biosynthetic process
Acrdi.XM_015905193.1/1-870	Monca.augustus.g57150.t1/1-864	0.11388	0.131149	Zinc finger CCCH domain-containing 10	1	F:GO:0046872	F:metal ion binding
Acrdi.XM_015908378.1/1-465	Monca.augustus.g34819.t1/1-465	0.242411	0.131104	Trace amine-associated receptor 7d			
Acrdi.XM_015905051.1/1-1590	Monca.augustus.g31218.t1/1-2673	0.171931	0.131104	Cirhin			
Acrdi.XM_015911997.1/1-3084	Monca.augustus.g35303.t1/1-3594	0.160389	0.131084	tubulin-specific chaperone D	5	C:GO:0030054; C:GO:0044464; P:GO:0006457; P:GO:0034329;	[illegible]
Acrdi.XM_015911404.1/1-2832	Monca.augustus.g53228.t1/1-3789	0.1709	0.131047	PREDICTED: uncharacterized protein KIAA0355-like			
Acrdi.XM_015924648.1/1-501	Monca.augustus.g50798.t1/1-1914	0.112487	0.131009	Maternal embryonic leucine zipper kinase			
Acrdi.XM_015898217.1/1-9126	Monca.augustus.g8199.t1/1-9972	0.212101	0.130991	cilia- and flagella-associated 54-like			
Acrdi.XM_015922317.1/1-2868	Monca.adi2mcaRNA3408_R0/1-2862	0.179659	0.130986	Solute carrier family 12 member 4			
Acrdi.XM_015914963.1/1-855	Monca.augustus.g42535.t1/1-681	0.244916	0.130977	PREDICTED: uncharacterized protein LOC107348880			
Acrdi.XM_015904931.1/1-2028	Monca.augustus.g61119.t1/1-2034	0.116095	0.130966	fem-1 homolog B	5	C:GO:0005634; C:GO:0005737; P:GO:0006915; P:GO:2000001;	[illegible]
Acrdi.XM_015916976.1/1-600	Monca.augustus.g50849.t1/1-903	0.138003	0.130949	proteasome inhibitor PI31 subunit	2	C:GO:0000502; P:GO:0006511	C:proteasome complex; P:ubiquitin-dependent protein catabolic process
Acrdi.XM_015901299.1/1-666	Monca.augustus.g43354.t1/1-639	0.129148	0.130931	BTB POZ domain-containing KCTD6-like	1	P:GO:0051260	P:protein homooligomerization
Acrdi.XM_015904990.1/1-2631	Monca.adi2mcaRNA14579_R0/1-2421	0.318369	0.130925	WDFY family member 4			
Acrdi.XM_015896655.1/1-1764	Monca.augustus.g13441.t1/1-1683	0.174295	0.130923	hexosaminidase D-like	2	P:GO:0005975; F:GO:0004553	[illegible]
Acrdi.XM_015908951.1/1-363	Monca.augustus.g47241.t1/1-351	0.0929837	0.130923	tenascin-like isoform X1	2	C:GO:0016020; C:GO:0016021	C:membrane; C:integral component of membrane
Acrdi.XM_015908397.1/1-957	Monca.augustus.g34846.t1/1-2097	0.192077	0.130868	DEFECTIVE IN EXINE FORMATION 1-like	4	P:GO:0007229; F:GO:0005509; C:GO:0016020; C:GO:0016021	[illegible]
Acrdi.XM_015899397.1/1-1494	Monca.augustus.g48268.t1/1-1836	0.128557	0.130842	histone deacetylase 2	39	F:GO:0000978; P:GO:0008284; P:GO:0061198; P:GO:0060789;	[illegible]
Acrdi.XM_015904191.1/1-699	Monca.adi2mcaRNA13719_R1/1-621	0.138785	0.130792	BTB POZ domain-containing 19-like			
Acrdi.XM_015905877.1/1-954	Monca.augustus.g32230.t1/1-1038	0.170323	0.130775	cell division control 6	2	F:GO:0000166; P:GO:0009987	F:nucleotide binding; P:cellular process
Acrdi.XM_015895866.1/1-1131	Monca.augustus.g23198.t1/1-1134	0.199009	0.130752	Neuropeptide FF receptor 2	3	P:GO:0007165; C:GO:0016020; F:GO:0004871	P:signal transduction; C:membrane; F:signal transducer activity
Acrdi.XM_015898753.1/1-1929	Monca.augustus.g2081.t1/1-1926	0.138896	0.130739	ankycorbin-like isoform X1			
Acrdi.XM_015895927.1/1-867	Monca.adi2mcaRNA4727_R0/1-858	0.168694	0.130727	alcohol dehydrogenase	1	F:GO:0046872	F:metal ion binding
Acrdi.XM_015915457.1/1-3033	Monca.augustus.g53451.t1/1-3225	0.133592	0.130727	transient receptor potential channel pyrexia-like	1	P:GO:0006810	P:transport
Acrdi.XM_015912486.1/1-489	Monca.augustus.g8251.t1/1-648	0.0709337	0.130727	P2X purinoceptor 7-like	1	C:GO:0016020	C:membrane
Acrdi.XM_015897402.1/1-2892	Monca.augustus.g51664.t1/1-2991	0.139145	0.130673	MKL myocardin 1	4	P:GO:0072359; P:GO:0048513; P:GO:0044763; P:GO:0050789	[illegible]
Acrdi.XM_015904179.1/1-1746	Monca.augustus.g48548.t1/1-1764	0.165439	0.130648	gamma-glutamyltranspeptidase 1-like	2	P:GO:0006749; F:GO:0003840	P:glutathione metabolic process; F:gamma-glutamyltransferase activity
Acrdi.XM_015912044.1/1-1158	Monca.augustus.g67630.t1/1-1179	0.0964076	0.130634	nuclease mitochondrial-like	2	C:GO:0016020; F:GO:0005488	C:membrane; F:binding
Acrdi.XM_015897144.1/1-2250	Monca.augustus.g63834.t1/1-2439	0.150557	0.130626	ectonucleotide pyrophosphatase phosphodiesterase family member 3	3	P:GO:0008152; F:GO:0005488; F:GO:0016787	P:metabolic process; F:binding; F:hydrolase activity
Acrdi.XM_015914023.1/1-723	Monca.augustus.g68879.t1/1-735	0.186287	0.130623	gamma-aminobutyric acid receptor subunit beta-like	2	F:GO:0005216; P:GO:0006811	F:ion channel activity; P:ion transport
Acrdi.XM_015911641.1/1-2541	Monca.adi2mcaRNA21912_R1/1-2334	0.1557	0.130602	PREDICTED: uncharacterized protein LOC107345889	3	C:GO:0016020; F:GO:0046539; P:GO:0032259	C:membrane; F:histamine N-methyltransferase activity; P:methylation
Acrdi.XM_015905359.1/1-849	Monca.augustus.g61122.t1/1-936	0.107403	0.130585	Transcription initiation factor IIA subunit	4	F:GO:0003743; C:GO:0005672; P:GO:0006413; P:GO:0006367	[illegible]
Acrdi.XM_015905797.1/1-3444	Monca.augustus.g31752.t1/1-4809	0.120537	0.130582	myosin-IIIa isoform X2			
Acrdi.XM_015912378.1/1-1116	Monca.augustus.g51036.t1/1-1272	0.146926	0.130525	STAM-binding -like A			
Acrdi.XM_015893721.1/1-2724	Monca.adi2mcaRNA569_R2/1-2796	0.388308	0.130473	Gypsy retrotransposon integrase 1	1	F:GO:0003676	F:nucleic acid binding
Acrdi.XM_015907165.1/1-2241	Monca.augustus.g66881.t1/1-2211	0.155036	0.130462	X-ray repair cross-complementing 5-like	13	F:GO:0003690; P:GO:0006310; P:GO:0000723; C:GO:0043564;	[illegible]
Acrdi.XM_015907428.1/1-1446	Monca.augustus.g64326.t1/1-2616	0.147078	0.13045	cGMP-specific 3 ,5 -cyclic phosphodiesterase-like	4	C:GO:0016020; P:GO:0044699; P:GO:0009987; F:GO:0004114	[illegible]
Acrdi.XM_015902837.1/1-615	Monca.augustus.g27205.t1/1-822	0.145659	0.130423	tumor p53-inducible 11	2	C:GO:0016020; C:GO:0016021	C:membrane; C:integral component of membrane
Acrdi.XM_015922282.1/1-330	Monca.augustus.g27635.t1/1-657	0.115565	0.130392	Voltage-gated hydrogen channel 1	1	P:GO:0006810	P:transport
Acrdi.XM_015900629.1/1-1119	Monca.augustus.g3623.t1/1-1068	0.154412	0.130387	prostaglandin reductase 2	4	F:GO:0008270; F:GO:0016491; C:GO:0005739; P:GO:0055114	[illegible]
Acrdi.XM_015899189.1/1-441	Monca.augustus.g24884.t1/1-444	0.206063	0.130378	universal stress -like isoform X1	1	P:GO:0006950	P:response to stress
Acrdi.XM_015919158.1/1-789	Monca.adi2mcaRNA30312_R1/1-843	0.877421	0.130362	DUF1758 and DUF1759 domain containing			
Acrdi.XM_015913720.1/1-855	Monca.augustus.g21637.t1/1-852	0.162475	0.130322	bis(5 -nucleosyl)-tetraphosphatase [asymmetrical]-like	3	C:GO:0016020; C:GO:0016021; F:GO:0016787	C:membrane; C:integral component of membrane; F:hydrolase activity
Acrdi.XM_015914092.1/1-2514	Monca.augustus.g59125.t1/1-2499	0.145762	0.130302	PREDICTED: uncharacterized protein LOC107348081	6	C:GO:0005615; P:GO:0032504; F:GO:0003824; P:GO:0008152;	[illegible]
Acrdi.XM_015906857.1/1-1614	Monca.augustus.g8900.t1/1-1437	0.1707	0.130264	DNA polymerase delta subunit 2	4	F:GO:0003677; F:GO:0003887; P:GO:0006260; P:GO:0071897	[illegible]
Acrdi.XM_015904133.1/1-2895	Monca.augustus.g47259.t1/1-2112	0.130306	0.130241	Unconventional myosin-VIIa	2	F:GO:0000166; C:GO:0005856	F:nucleotide binding; C:cytoskeleton
Acrdi.XM_015905960.1/1-1008	Monca.augustus.g69498.t1/1-1863	0.176723	0.130236	Seleno O			
Acrdi.XM_015911610.1/1-1611	Monca.augustus.g54362.t1/1-1650	0.0946858	0.130225	DNA-(apurinic or apyrimidinic site) lyase 2	6	P:GO:0006281; F:GO:0004536; C:GO:0043231; F:GO:0004519;	[illegible]
Acrdi.XM_015908353.1/1-1842	Monca.adi2mcaRNA18316_R0/1-1803	0.136688	0.130217	adenosine receptor A3-like	2	F:GO:0004930; P:GO:0007165	F:G-protein coupled receptor activity; P:signal transduction
Acrdi.XM_015898174.1/1-1002	Monca.augustus.g39426.t1/1-1002	0.160906	0.13021	WD repeat-containing 53			
Acrdi.XM_015898534.1/1-1158	Monca.augustus.g33994.t1/1-1164	0.227724	0.130171	centromere F-like			
Acrdi.XM_015925345.1/1-1050	Monca.augustus.g6775.t1/1-1041	0.124435	0.130142	G- coupled receptor 161-like	3	F:GO:0004930; P:GO:0007186; C:GO:0016021	[illegible]
Acrdi.XM_015910781.1/1-855	Monca.augustus.g16153.t1/1-918	0.149863	0.130112	E2F-associated phospho	3	C:GO:0005634; P:GO:0034243; P:GO:0008284	[illegible]
Acrdi.XM_015893612.1/1-903	Monca.augustus.g23917.t1/1-1011	0.181691	0.130097	Beta-1,3-galactosyltransferase 6	3	F:GO:0016757; C:GO:0016020; C:GO:0005794	[illegible]
Acrdi.XM_015921106.1/1-1665	Monca.augustus.g48113.t1/1-903	0.164358	0.130079	ZDHHC-type palmitoyltransferase 6	7	F:GO:0016746; F:GO:0008270; F:GO:0019706; F:GO:0046872;	[illegible]
Acrdi.XM_015919531.1/1-468	Monca.augustus.g36911.t1/1-468	0.175424	0.130067	Sjoegren syndrome scleroderma autoantigen 1	1	F:GO:0042802	F:identical protein binding
Acrdi.XM_015897537.1/1-912	Monca.augustus.g22821.t1/1-819	0.12106	0.130064	PREDICTED: uncharacterized protein LOC107332792			
Acrdi.XM_015916507.1/1-4197	Monca.adi2mcaRNA27327_R0/1-3723	0.124331	0.130053	lysyl oxidase homolog 2	4	P:GO:0050794; P:GO:0002040; P:GO:0044763; C:GO:0005576	[illegible]
Acrdi.XM_015924161.1/1-1050	Monca.adi2mcaRNA35906_R0/1-846	0.129613	0.130044	Structural maintenance of chromosomes 1A	15	P:GO:0019827; P:GO:0007093; F:GO:0036033; P:GO:0007064;	[illegible]
Acrdi.XM_015915881.1/1-1929	Monca.augustus.g39019.t1/1-2250	0.129211	0.130042	DD3-3-like			
Acrdi.XM_015894029.1/1-2310	Monca.adi2mcaRNA40234_R0/1-2265	0.212901	0.130034	Protocadherin-	6	F:GO:0005509; C:GO:0016020; C:GO:0016021; P:GO:0007155;	[illegible]
Acrdi.XM_015917830.1/1-1578	Monca.adi2mcaRNA28813_R0/1-1164	0.137982	0.130029	cyclin-dependent kinase 17	3	F:GO:0005524; F:GO:0004674; P:GO:0006468	[illegible]
Acrdi.XM_015899926.1/1-2874	Monca.adi2mcaRNA9037_R0/1-2769	0.1561	0.130016	Condensin complex subunit 3	3	C:GO:0000793; P:GO:0007076; C:GO:0044446	[illegible]
Acrdi.XM_015920740.1/1-582	Monca.augustus.g35953.t1/1-1143	0.123413	0.129999	Translocating chain-associated membrane 1-like 1	3	P:GO:0006613; C:GO:0016021; C:GO:0005791	[illegible]
Acrdi.XM_015910324.1/1-1104	Monca.adi2mcaRNA20514_R0/1-1089	0.114677	0.129985	calumenin isoform X2	1	F:GO:0005509	F:calcium ion binding
Acrdi.XM_015899097.1/1-786	Monca.augustus.g26259.t1/1-990	0.166753	0.129976	DNA fragmentation factor subunit beta	3	C:GO:0000790; F:GO:0004536; P:GO:0006309	[illegible]
Acrdi.XM_015901969.1/1-1725	Monca.augustus.g45528.t1/1-1725	0.180114	0.129955	uncharacterized GTP-binding -like	6	F:GO:0005525; P:GO:0006813; P:GO:0071805; C:GO:0016020;	[illegible]
Acrdi.XM_015907895.1/1-1200	Monca.augustus.g40419.t1/1-1161	0.151959	0.129949	proline-rich transmembrane 4-like	2	C:GO:0016020; C:GO:0016021	C:membrane; C:integral component of membrane
Acrdi.XM_015900555.1/1-573	Monca.augustus.g26468.t1/1-573	0.235324	0.129918	Glutathione peroxidase 7	1	F:GO:0016491	F:oxidoreductase activity
Acrdi.XM_015904922.1/1-789	Monca.augustus.g8816.t1/1-786	0.153513	0.129916	transmembrane 163-like	3	P:GO:0006812; P:GO:0055085; C:GO:0016020	P:cation transport; P:transmembrane transport; C:membrane
Acrdi.XM_015910737.1/1-1248	Monca.augustus.g47990.t1/1-1374	0.142569	0.129893	cilia- and flagella-associated 36-like			
Acrdi.XM_015920288.1/1-672	Monca.augustus.g23537.t1/1-1083	0.120176	0.129825	cystinosin homolog	2	C:GO:0016020; C:GO:0016021	C:membrane; C:integral component of membrane
Acrdi.XM_015913939.1/1-1605	Monca.augustus.g59717.t1/1-2418	0.150117	0.129822	exportin-4 isoform X2	1	C:GO:0044428	C:nuclear part
Acrdi.XM_015898779.1/1-960	Monca.augustus.g11046.t1/1-966	0.48232	0.129812	Hemolytic lectin LSLb	1	F:GO:0030246	F:carbohydrate binding
Acrdi.XM_015895648.1/1-585	Monca.adi2mcaRNA4396_R0/1-573	0.179956	0.129811	PREDICTED: uncharacterized protein LOC107331112			
Acrdi.XM_015911520.1/1-1173	Monca.augustus.g45451.t1/1-1176	0.121653	0.129807	neuropeptide Y receptor type 2-like	3	P:GO:0007165; C:GO:0016021; F:GO:0004871	[illegible]
Acrdi.XM_015913865.1/1-1929	Monca.augustus.g70077.t1/1-2433	0.173367	0.129769	S1 RNA-binding domain-containing 1	2	F:GO:0003676; P:GO:0006139	[illegible]
Acrdi.XM_015920635.1/1-939	Monca.augustus.g36312.t1/1-933	0.128349	0.129747	Beta-sarcoglycan	2	C:GO:0016011; P:GO:0009987	C:dystroglycan complex; P:cellular process
Acrdi.XM_015913819.1/1-1119	Monca.augustus.g17355.t1/1-2493	0.115189	0.129697	Neuron navigator 3			
Acrdi.XM_015910005.1/1-2244	Monca.augustus.g7091.t1/1-3357	0.183914	0.129662	FAM184A isoform X2			
Acrdi.XM_015900925.1/1-351	Monca.augustus.g27887.t1/1-957	0.142409	0.129649	OTU domain-containing 6B			
Acrdi.XM_015921759.1/1-1239	Monca.adi2mcaRNA33134_R0/1-1338	0.164519	0.129639	Coagulation factor VIII	1	F:GO:0070492	F:oligosaccharide binding
Acrdi.XM_015919311.1/1-684	Monca.augustus.g10288.t1/1-306	0.137392	0.129622	Mitochondrial import inner membrane translocase subunit Tim21	4	C:GO:0005744; P:GO:0033617; P:GO:0030150; P:GO:0032981	[illegible]
Acrdi.XM_015906674.1/1-1035	Monca.augustus.g60583.t1/1-1014	0.171017	0.12961	membrane progestin receptor gamma-A-like	3	C:GO:0016020; C:GO:0016021; F:GO:0004872	C:membrane; C:integral component of membrane; F:receptor activity
Acrdi.XM_015922979.1/1-759	Monca.augustus.g67828.t1/1-1485	0.145235	0.129585	proline-serine-threonine phosphatase-interacting 1-	1	P:GO:0007165	P:signal transduction

Acrdi.XM_015908613.1/1-4110	Monca.augustus.g54439.t1/1-5535	0.102099	0.12956	supervillin isoform X11	3	F:GO:0051015; F:GO:0003779; P:GO:0007010	F:actin filament binding; F:actin binding; P:cytoskeleton organization
Acrdi.XM_015913626.1/1-477	Monca.adi2mcaRNA24089_R1/1-486	0.1153	0.12955	tetratricopeptide repeat 9C	1	P:GO:0009987	P:cellular process
Acrdi.XM_015921203.1/1-1914	Monca.adi2mcaRNA32551_R0/1-1926	0.118697	0.12954	threonine synthase-like 1	2	C:GO:0005737; P:GO:0006520	C:cytoplasm; P:cellular amino acid metabolic process
Acrdi.XM_015915196.1/1-660	Monca.augustus.g29739.t1/1-654	0.157361	0.129538	transcription factor LBX2	3	C:GO:0005634; P:GO:0006355; F:GO:0043565	C:nucleus; P:regulation of transcription, DNA-templated; F:sequence-specific DNA binding
Acrdi.XM_015914777.1/1-5454	Monca.augustus.g61014.t1/1-5847	0.161918	0.129537	Mitogen-activated kinase-binding			
Acrdi.XM_015904244.1/1-1152	Monca.augustus.g7193.t1/1-1278	0.152955	0.129535	proline-serine-threonine phosphatase-interacting 1 isoform X1	3	C:GO:0005737; C:GO:0016020; P:GO:0050896	C:cytoplasm; C:membrane; P:response to stimulus
Acrdi.XM_015922502.1/1-1878	Monca.augustus.g33869.t1/1-1947	0.160349	0.129523	Histidine ammonia-lyase	3	P:GO:0006536; P:GO:0006548; F:GO:0016841	P:glutamate metabolic process; P:histidine catabolic process; F:ammonia-lyase activity
Acrdi.XM_015920460.1/1-1383	Monca.augustus.g51639.t1/1-4206	0.133182	0.129505	transient receptor potential cation channel subfamily A member 1-like	7	F:GO:0005216; P:GO:0006811; P:GO:0055085; P:GO:0034220;	F:ion channel activity; P:ion transport; P:transmembrane transport; P:ion transmembrane transport; C:membrane; C:integral component of membrane; P:transport
Acrdi.XM_015910362.1/1-324	Monca.augustus.g36422.t1/1-303	0.135324	0.129455	Ubiquitin-related modifier 1	4	C:GO:0005829; P:GO:0002098; P:GO:0032447; P:GO:0034227	C:cytosol; P:tRNA wobble uridine modification; P:protein urmylation; P:tRNA thio-modification
Acrdi.XM_015917540.1/1-1137	Monca.augustus.g2156.t1/1-1140	0.136185	0.12945	tyramine receptor Ser-2-like	2	P:GO:0007210; F:GO:0004993	P:serotonin receptor signaling pathway; F:G-protein coupled serotonin receptor activity
Acrdi.XM_015901484.1/1-639	Monca.augustus.g31722.t1/1-642	0.22673	0.129446	homeobox MOX-2-like	3	C:GO:0005634; P:GO:0006355; F:GO:0043565	C:nucleus; P:regulation of transcription, DNA-templated; F:sequence-specific DNA binding
Acrdi.XM_015895896.1/1-1461	Monca.augustus.g36087.t1/1-2031	0.13103	0.129443	Cyclic AMP-dependent transcription factor ATF-6 alpha	3	P:GO:0050794; C:GO:0043231; F:GO:0005488	P:regulation of cellular process; C:intracellular membrane-bounded organelle; F:binding
Acrdi.XM_015897280.1/1-1179	Monca.augustus.g4201.t1/1-1872	0.207794	0.129411	mediator of RNA polymerase II transcription subunit 15	3	C:GO:0016592; F:GO:0001104; P:GO:0006357	C:mediator complex; F:RNA polymerase II transcription cofactor activity; P:regulation of transcription from RNA polymerase II promoter
Acrdi.XM_015923141.1/1-918	Monca.augustus.g30555.t1/1-1011	0.141984	0.129369	jmjC domain-containing 4-like	2	C:GO:0016020; C:GO:0016021	C:membrane; C:integral component of membrane
Acrdi.XM_015924013.1/1-4653	Monca.augustus.g46218.t1/1-15540	0.141777	0.129353	dynein heavy chain domain-containing 1-like	9	F:GO:0000166; F:GO:0016887; F:GO:0005524; F:GO:0003777;	F:nucleotide binding; F:ATPase activity; F:ATP binding; F:microtubule motor activity; C:axonemal dynein complex; C:dynein complex; P:cilium movement; P:microtubule-based mov[illegible]
Acrdi.XM_015906318.1/1-3444	Monca.augustus.g45311.t1/1-4044	0.189859	0.129313	probable ATP-dependent DNA helicase HFM1			
Acrdi.XM_015899170.1/1-1119	Monca.augustus.g14542.t1/1-1110	0.477235	0.129245	glutamic acid-rich -like	2	C:GO:0016020; C:GO:0016021	C:membrane; C:integral component of membrane
Acrdi.XM_015915485.1/1-798	Monca.augustus.g36665.t1/1-798	0.160869	0.12924	Delta(14)-sterol reductase	5	F:GO:0016627; C:GO:0005737; P:GO:0006629; C:GO:0016021;	F:oxidoreductase activity, acting on the CH-CH group of donors; C:cytoplasm; P:lipid metabolic process; C:integral component of membrane; P:oxidation-reduction process
Acrdi.XM_015902277.1/1-573	Monca.augustus.g35119.t1/1-621	0.165788	0.129207	NC domain-containing	3	C:GO:0005634; C:GO:0016020; C:GO:0016021	C:nucleus; C:membrane; C:integral component of membrane
Acrdi.XM_015898902.1/1-2004	Monca.augustus.g7897.t1/1-1518	0.164622	0.129191	Heterogeneous nuclear ribonucleo L	5	F:GO:0000166; F:GO:0003676; C:GO:0005634; F:GO:0003723;	F:nucleotide binding; F:nucleic acid binding; C:nucleus; F:RNA binding; P:mRNA processing
Acrdi.XM_015905678.1/1-1623	Monca.augustus.g26830.t1/1-3591	0.185004	0.129183	Centrosomal of 170 kDa B			
Acrdi.XM_015906166.1/1-1317	Monca.augustus.g44123.t1/1-1458	0.159495	0.129157	Sulfide:quinone mitochondrial	4	F:GO:0016491; C:GO:0005739; P:GO:0055114; C:GO:0005743	F:oxidoreductase activity; C:mitochondrion; P:oxidation-reduction process; C:mitochondrial inner membrane
Acrdi.XM_015915122.1/1-897	Monca.augustus.g26554.t1/1-924	0.12599	0.129149	probable T4-type lysozyme 2	1	F:GO:0016787	F:hydrolase activity
Acrdi.XM_015897555.1/1-2736	Monca.adi2mcaRNA6538_R0/1-2391	0.154568	0.129148	AP-4 complex subunit	2	C:GO:0016020; P:GO:0015031	C:membrane; P:protein transport
Acrdi.XM_015895476.1/1-708	Monca.augustus.g2040.t1/1-732	0.203249	0.129117	lifeguard 1-like	1	C:GO:0016021	C:integral component of membrane
Acrdi.XM_015922010.1/1-1659	Monca.augustus.g35325.t1/1-1629	0.187213	0.129101	mab-21-like 2			
Acrdi.XM_015918166.1/1-1140	Monca.augustus.g66567.t1/1-1275	0.201879	0.129096	sorting nexin-4-like	5	C:GO:0005768; P:GO:0016050; F:GO:0035091; C:GO:0019898;	C:endosome; P:vesicle organization; F:phosphatidylinositol binding; C:extrinsic component of membrane; P:endocytosis
Acrdi.XM_015903256.1/1-636	Monca.augustus.g51686.t1/1-513	0.137415	0.129083	Diphosphoinositol polyphosphate phosphohydrolase 1	9	F:GO:0000287; F:GO:0052841; F:GO:0052842; P:GO:0015961;	F:magnesium ion binding; F:inositol bisdiphosphate tetrakisphosphate diphosphatase activity; F:inositol diphosphate pentakisphosphate diphosphatase activity; P:diadenosine[illegible]
Acrdi.XM_015917793.1/1-840	Monca.augustus.g53118.t1/1-1011	0.203971	0.129048	arrestin domain-containing 3-like			
Acrdi.XM_015898405.1/1-708	Monca.augustus.g58531.t1/1-1011	0.18048	0.129035	carbohydrate sulfotransferase 11-like	3	P:GO:0016051; C:GO:0016021; F:GO:0008146	P:carbohydrate biosynthetic process; C:integral component of membrane; F:sulfotransferase activity
Acrdi.XM_015893818.1/1-969	Monca.augustus.g535.t1/1-963	0.111151	0.129018	alpha- kinase 1-like			
Acrdi.XM_015902259.1/1-312	Monca.augustus.g63809.t1/1-705	0.248065	0.128984	thiamine biosynthesis	2	F:GO:0019172; P:GO:0019243	F:glyoxalase III activity; P:methylglyoxal catabolic process to D-lactate via S-lactoyl-glutathione
Acrdi.XM_015915497.1/1-3000	Monca.adi2mcaRNA26194_R7/1-2721	0.362218	0.128982	Retrovirus-related Pol poly from transposon			
Acrdi.XM_015923299.1/1-366	Monca.augustus.g35011.t1/1-366	0.165804	0.128972	PREDICTED: uncharacterized protein LOC107356680 isoform X2	8	F:GO:0019825; C:GO:0005737; C:GO:0016020; P:GO:0006508;	F:oxygen binding; C:cytoplasm; C:membrane; P:proteolysis; C:integral component of membrane; F:calcium-dependent cysteine-type endopeptidase activity; C:intracellular; F:heme[illegible]
Acrdi.XM_015922040.1/1-2049	Monca.augustus.g31847.t1/1-5484	0.134517	0.128961	NACHT domain- and WD repeat-containing 1-like			
Acrdi.XM_015910735.1/1-1632	Monca.augustus.g47981.t1/1-2535	0.185458	0.128956	BOBBER 1-like	1	P:GO:0006464	P:cellular protein modification process
Acrdi.XM_015899870.1/1-2793	Monca.augustus.g48624.t1/1-2802	0.165498	0.128898	Kyphoscoliosis peptidase			
Acrdi.XM_015902525.1/1-1134	Monca.augustus.g64954.t1/1-1143	0.158237	0.128866	ATP-dependent (S)-NAD(P)H-hydrate dehydratase isoform X2	1	F:GO:0016829	F:lyase activity
Acrdi.XM_015911316.1/1-957	Monca.augustus.g67446.t1/1-975	0.151927	0.128858	Dehydrogenase reductase SDR family member on chromosome X	2	F:GO:0016491; P:GO:0055114	F:oxidoreductase activity; P:oxidation-reduction process
Acrdi.XM_015901328.1/1-873	Monca.augustus.g48491.t1/1-1035	0.169417	0.12885	PREDICTED: uncharacterized protein LOC107336261			
Acrdi.XM_015924738.1/1-768	Monca.augustus.g4187.t1/1-768	0.119572	0.128839	NADH dehydrogenase [ubiquinone] flavo mitochondrial	6	C:GO:0045272; C:GO:0005747; F:GO:0046872; P:GO:0006120;	C:plasma membrane respiratory chain complex I; C:mitochondrial respiratory chain complex I; F:metal ion binding; P:mitochondrial electron transport, NADH to ubiquinone; F:EC:1.[illegible]
Acrdi.XM_015895466.1/1-2595	Monca.adi2mcaRNA4305_R0/1-2388	0.158321	0.128807	Methionine--tRNA cytoplasmic	2	F:GO:0097159; F:GO:1901363	F:organic cyclic compound binding; F:heterocyclic compound binding
Acrdi.XM_015912891.1/1-489	Monca.augustus.g58406.t1/1-735	0.765554	0.128803	L-rhamnose-binding lectin CSL3-like isoform X1	5	C:GO:0016020; C:GO:0016021; F:GO:0005044; F:GO:0030246;	C:membrane; C:integral component of membrane; F:scavenger receptor activity; F:carbohydrate binding; P:receptor-mediated endocytosis
Acrdi.XM_015895545.1/1-849	Monca.augustus.g4319.t1/1-849	0.147144	0.128769	rolling stone-like	2	C:GO:0016020; C:GO:0016021	C:membrane; C:integral component of membrane
Acrdi.XM_015915257.1/1-1134	Monca.adi2mcaRNA25959_R0/1-1128	0.141566	0.128762	neuropeptide FF receptor 2-like	4	C:GO:0005887; F:GO:0008188; P:GO:0007186; P:GO:0007166	C:integral component of plasma membrane; F:neuropeptide receptor activity; P:G-protein coupled receptor signaling pathway; P:cell surface receptor signaling pathway
Acrdi.XM_015891993.1/1-1332	Monca.augustus.g36364.t1/1-2082	0.168302	0.12875	PREDICTED: uncharacterized protein LOC107327242			
Acrdi.XM_015900866.1/1-1746	Monca.augustus.g27864.t1/1-1665	0.150126	0.128745	probable D-lactate mitochondrial	3	F:GO:0016614; F:GO:0050660; P:GO:0055114	F:oxidoreductase activity, acting on CH-OH group of donors; F:flavin adenine dinucleotide binding; P:oxidation-reduction process
Acrdi.XM_015906334.1/1-3825	Monca.augustus.g45341.t1/1-3825	0.211442	0.128732	F-box LRR-repeat 2-like	4	F:GO:0005525; P:GO:0007165; P:GO:0007264; C:GO:0005622	F:GTP binding; P:signal transduction; P:small GTPase mediated signal transduction; C:intracellular
Acrdi.XM_015908175.1/1-900	Monca.adi2mcaRNA18100_R9/1-798	0.178486	0.128724	piggyBac transposable element-derived 4-like			
Acrdi.XM_015911258.1/1-609	Monca.augustus.g13707.t1/1-1572	0.211992	0.128696	nesprin-1-like isoform X2	1	F:GO:0003779	F:actin binding
Acrdi.XM_015920854.1/1-1077	Monca.adi2mcaRNA32151_R1/1-1041	0.161597	0.128688	transferase mitochondrial	2	C:GO:0005759; P:GO:0016226	C:mitochondrial matrix; P:iron-sulfur cluster assembly
Acrdi.XM_015899629.1/1-1539	Monca.augustus.g33797.t1/1-1839	0.114455	0.128667	Tyrosine- phosphatase non-receptor type 9	2	F:GO:0016791; P:GO:0016311	F:phosphatase activity; P:dephosphorylation
Acrdi.XM_015914298.1/1-2757	Monca.augustus.g40765.t1/1-2754	0.127695	0.128663	PREDICTED: uncharacterized protein LOC107348277			
Acrdi.XM_015903390.1/1-3081	Monca.augustus.g48202.t1/1-3111	0.132222	0.128653	Mannosyl oligosaccharide glucosidase	4	C:GO:0005789; P:GO:0006487; F:GO:0004573; P:GO:0009311	C:endoplasmic reticulum membrane; P:protein N-linked glycosylation; F:m[illegible]
Acrdi.XM_015915979.1/1-423	Monca.augustus.g51182.t1/1-435	0.206741	0.128647	isoform CRA_a	3	P:GO:0008150; C:GO:0005575; F:GO:0003674	P:biological_process; C:cellular_component; F:molecular_function
Acrdi.XM_015908517.1/1-1644	Monca.augustus.g46687.t1/1-1626	0.110116	0.128644	Frizzled homolog 4 (Drosophila)	23	P:GO:0061299; F:GO:0042813; F:GO:0042803; P:GO:0007186;	P:retina vasculature morphogenesis in camera-type eye; F:Wnt-activated r[illegible]
Acrdi.XM_015919554.1/1-1653	Monca.augustus.g65902.t1/1-1695	0.172014	0.128627	gamma-glutamyltransferase	1	F:GO:0016746	F:transferase activity, transferring acyl groups
Acrdi.XM_015902094.1/1-1449	Monca.augustus.g9254.t1/1-1638	0.144257	0.128589	WD repeat-containing 76	1	P:GO:0006974	P:cellular response to DNA damage stimulus
Acrdi.XM_015909698.1/1-693	Monca.augustus.g55277.t1/1-714	0.172331	0.128587	PREDICTED: uncharacterized protein LOC107344092	5	F:GO:0003677; C:GO:0016020; C:GO:0016021; P:GO:0015074;	F:DNA binding; C:membrane; C:integral component of membrane; P:DNA i[illegible]
Acrdi.XM_015907911.1/1-594	Monca.augustus.g46302.t1/1-594	0.125273	0.128587	histone	2	C:GO:0005694; P:GO:0006323	C:chromosome; P:DNA packaging
Acrdi.XM_015910255.1/1-2328	Monca.adi2mcaRNA20406_R0/1-2247	0.134784	0.128583	AP-3 complex subunit beta-1	6	P:GO:0065007; C:GO:0030119; F:GO:0005515; P:GO:0016192;	P:biological regulation; C:AP-type membrane coat adaptor complex; F:prot[illegible]
Acrdi.XM_015917882.1/1-1125	Monca.adi2mcaRNA28878_R0/1-1110	0.154398	0.128574	synaptotagmin-6 isoform X4	7	P:GO:0065007; F:GO:0005515; P:GO:0016192; C:GO:0016020;	P:biological regulation; F:protein binding; P:vesicle-mediated transport; C:m[illegible]
Acrdi.XM_015898091.1/1-1509	Monca.augustus.g5581.t1/1-1509	0.131632	0.128567	fatty aldehyde dehydrogenase isoform X1	10	F:GO:0004029; F:GO:0004028; C:GO:0043231; C:GO:0016020;	F:aldehyde dehydrogenase (NAD) activity; F:3-chloroallyl aldehyde dehydro[illegible]
Acrdi.XM_015922794.1/1-672	Monca.adi2mcaRNA34327_R0/1-705	0.0937222	0.128533	cysteine protease ATG4D-like	9	P:GO:0000045; P:GO:0051697; P:GO:0044804; F:GO:0004197;	P:autophagosome assembly; P:protein delipidation; P:nucleophagy; F:cyste[illegible]
Acrdi.XM_015904384.1/1-717	Monca.augustus.g48080.t1/1-714	0.118839	0.128532	HAD family hydrolase	3	F:GO:0016791; C:GO:0005829; P:GO:0016311	F:phosphatase activity; C:cytosol; P:dephosphorylation
Acrdi.XM_015901061.1/1-1200	Monca.adi2mcaRNA10296_R0/1-1242	0.217936	0.128498	WAS WASL-interacting family member 2			
Acrdi.XM_015901084.1/1-1722	Monca.augustus.g57661.t1/1-1692	0.137598	0.128479	zinc finger CW-type PWWP domain 2	1	F:GO:0008270	F:zinc ion binding
Acrdi.XM_015914968.1/1-2196	Monca.augustus.g42432.t1/1-2490	0.137689	0.128451	WD repeat-containing 24 isoform X1			
Acrdi.XM_015901350.1/1-813	Monca.augustus.g61865.t1/1-813	0.167381	0.128442	PREDICTED: uncharacterized protein LOC107336278			
Acrdi.XM_015909954.1/1-459	Monca.augustus.g41474.t1/1-438	0.503922	0.128291	THAP domain-containing 2			
Acrdi.XM_015917237.1/1-609	Monca.augustus.g48825.t1/1-645	0.169863	0.128263	F-box only 21	3	F:GO:0003677; C:GO:0016020; C:GO:0016021	F:DNA binding; C:membrane; C:integral component of membrane
Acrdi.XM_015905173.1/1-1176	Monca.augustus.g52833.t1/1-1176	0.107237	0.128234	vicilin-like seed storage At2g18540			
Acrdi.XM_015897037.1/1-378	Monca.augustus.g70184.t1/1-444	0.110533	0.128231	Transmembrane 72	2	C:GO:0016020; C:GO:0016021	C:membrane; C:integral component of membrane
Acrdi.XM_015909070.1/1-417	Monca.augustus.g13100.t1/1-1011	0.181853	0.128212	Coiled-coil domain-containing 84			
Acrdi.XM_015922286.1/1-351	Monca.augustus.g27633.t1/1-3546	0.158375	0.128186	PREDICTED: uncharacterized protein LOC107355709			
Acrdi.XM_015924996.1/1-1116	Monca.augustus.g22972.t1/1-1110	0.164895	0.128173	histamine H2 receptor	6	P:GO:0003008; P:GO:0044707; F:GO:0004871; P:GO:0050789;	P:system process; P:single-multicellular organism process; F:signal transdu[illegible]
Acrdi.XM_015903110.1/1-2115	Monca.augustus.g12312.t1/1-2211	0.155037	0.128131	72 kDa inositol polyphosphate 5-phosphatase	2	P:GO:0046856; F:GO:0016787	P:phosphatidylinositol dephosphorylation; F:hydrolase activity
Acrdi.XM_015897689.1/1-963	Monca.augustus.g65412.t1/1-1335	0.126255	0.128101	FAD-dependent oxidoreductase domain-containing 1-like	3	F:GO:0016491; F:GO:0008115; P:GO:0055114	F:oxidoreductase activity; F:sarcosine oxidase activity; P:oxidation-reductio[illegible]
Acrdi.XM_015910445.1/1-654	Monca.augustus.g60937.t1/1-1668	0.171409	0.128099	tRNA methyltransferase 10 homolog B	5	F:GO:0008171; F:GO:0008649; F:GO:0008170; F:GO:0008276;	F:O-methyltransferase activity; F:rRNA methyltransferase activity; F:N-me[illegible]
Acrdi.XM_015899841.1/1-1371	Monca.augustus.g52531.t1/1-1293	0.215315	0.128084	arginine N-methyltransferase 2 isoform X1	7	F:GO:0035242; P:GO:0016571; C:GO:0005829; P:GO:0006355;	F:protein-arginine omega-N asymmetric methyltransferase activity; P:hist[illegible]
Acrdi.XM_015898846.1/1-1767	Monca.augustus.g39876.t1/1-8793	0.159404	0.128077	laminin epi-1			
Acrdi.XM_015897088.1/1-1029	Monca.augustus.g48384.t1/1-1029	0.160239	0.128068	acropsin 1	1	P:GO:0051716	P:cellular response to stimulus
Acrdi.XM_015925248.1/1-1401	Monca.augustus.g1543.t1/1-1419	0.163605	0.128044	vicilin-like seed storage At2g18540			
Acrdi.XM_015904422.1/1-1230	Monca.augustus.g10184.t1/1-2388	0.191161	0.128041	methyltransferase NSUN6	1	F:GO:0016740	F:transferase activity
Acrdi.XM_015908163.1/1-837	Monca.augustus.g10444.t1/1-825	0.121952	0.128034	coiled-coil domain-containing 97	1	C:GO:0005686	C:U2 snRNP
Acrdi.XM_015918552.1/1-2367	Monca.augustus.g19964.t1/1-2355	0.168158	0.128021	nucleotide-binding oligomerization domain-containing 2-like isoform X	1	P:GO:0042981	P:regulation of apoptotic process
Acrdi.XM_015919257.1/1-3441	Monca.augustus.g22500.t1/1-3849	0.180085	0.128012	tubulin polyglutamylase TTLL5-like isoform X1	1	P:GO:0006464	P:cellular protein modification process
Acrdi.XM_015918045.1/1-3093	Monca.adi2mcaRNA29029_R0/1-3117	0.158685	0.128	myotubularin-related 3 isoform X1	2	F:GO:0016791; P:GO:0016311	F:phosphatase activity; P:dephosphorylation
Acrdi.XM_015899826.1/1-2760	Monca.augustus.g53428.t1/1-2751	0.124972	0.127986	transient receptor potential cation channel subfamily A member 1-like	1	P:GO:0006810	P:transport
Acrdi.XM_015920776.1/1-2490	Monca.augustus.g62144.t1/1-3720	0.12164	0.12798	Regulator of nonsense transcripts 2	1	F:GO:0003723	F:RNA binding
Acrdi.XM_015920909.1/1-2832	Monca.augustus.g54781.t1/1-2724	0.132891	0.127969	glutamate receptor 4 isoform X2	6	P:GO:0006811; F:GO:0004888; F:GO:0005230; C:GO:0045202;	P:ion transport; F:transmembrane signaling receptor activity; F:extracellula[illegible]
Acrdi.XM_015919031.1/1-462	Monca.augustus.g32031.t1/1-453	0.0715292	0.127903	Rieske domain-containing	3	F:GO:0016491; F:GO:0051536; P:GO:0044710	F:oxidoreductase activity; F:iron-sulfur cluster binding; P:single-organism m[illegible]
Acrdi.XM_015899162.1/1-1587	Monca.augustus.g15059.t1/1-1710	0.182679	0.127902	monocarboxylate transporter 10-like	4	P:GO:0006811; P:GO:0055085; C:GO:0016021; F:GO:0008028	P:ion transport; P:transmembrane transport; C:integral component of mem[illegible]
Acrdi.XM_015905401.1/1-3828	Monca.augustus.g33960.t1/1-3942	0.172765	0.12786	Elongator complex 1	1	C:GO:0044424	C:intracellular part
Acrdi.XM_015891769.1/1-792	Monca.adi2mcaRNA37634_R0/1-801	0.122061	0.127842	adrenocorticotropic hormone receptor-like	9	F:GO:0004930; F:GO:0001588; P:GO:0001963; P:GO:0007186;	F:G-protein coupled receptor activity; F:dopamine neurotransmitter recept[illegible]
Acrdi.XM_015925259.1/1-1611	Monca.adi2mcaRNA37184_R0/1-1626	0.157537	0.127768	Lysine-specific demethylase 8	1	F:GO:0005509	F:calcium ion binding
Acrdi.XM_015899101.1/1-4128	Monca.adi2mcaRNA8168_R0/1-3738	0.146006	0.127759	peroxidasin homolog	5	F:GO:0004601; P:GO:0006979; P:GO:0098869; P:GO:0055114;	F:peroxidase activity; P:response to oxidative stress; P:cellular oxidant deto[illegible]
Acrdi.XM_015892743.1/1-1425	Monca.augustus.g13420.t1/1-1419	0.184712	0.127749	Ectonucleotide pyrophosphatase phosphodiesterase family member 5	1	C:GO:0016020	C:membrane
Acrdi.XM_015904035.1/1-408	Monca.augustus.g12305.t1/1-726	0.143672	0.127748	PREDICTED: uncharacterized protein LOC107338788			
Acrdi.XM_015895374.1/1-405	Monca.augustus.g46141.t1/1-591	0.121512	0.12772	AP-3 complex subunit sigma-1	1	P:GO:0015031	P:protein transport
Acrdi.XM_015909111.1/1-2496	Monca.augustus.g13103.t1/1-3186	0.157043	0.127718	Hypoxia up-regulated 1	2	F:GO:0000166; F:GO:0005524	F:nucleotide binding; F:ATP binding
Acrdi.XM_015917516.1/1-1710	Monca.augustus.g56373.t1/1-1683	0.13668	0.127701	Tripartite motif-containing 2	2	F:GO:0008270; F:GO:0046872	F:zinc ion binding; F:metal ion binding
Acrdi.XM_015921061.1/1-1284	Monca.augustus.g27610.t1/1-1629	0.131264	0.127701	Atlastin-	2	F:GO:0000166; C:GO:0016020	F:nucleotide binding; C:membrane
Acrdi.XM_015913960.1/1-2286	Monca.augustus.g63306.t1/1-2268	0.135341	0.127603	gamma-aminobutyric acid type B receptor subunit 1-like	3	F:GO:0004930; P:GO:0007165; C:GO:0016020	F:G-protein coupled receptor activity; P:signal transduction; C:membrane
Acrdi.XM_015902931.1/1-912	Monca.augustus.g41322.t1/1-987	0.103656	0.12758	28S ribosomal mitochondrial			
Acrdi.XM_015892104.1/1-492	Monca.augustus.g12057.t1/1-1107	0.0866767	0.127576	dnaJ homolog subfamily B member 12-like	1	C:GO:0016020	C:membrane
Acrdi.XM_015918046.1/1-684	Monca.augustus.g60684.t1/1-669	0.18074	0.127528	intraflagellar transport 22 homolog	3	F:GO:0005525; P:GO:0007264; C:GO:0005622	F:GTP binding; P:small GTPase mediated signal transduction; C:intracellula[illegible]
Acrdi.XM_015902129.1/1-948	Monca.augustus.g46397.t1/1-942	0.148442	0.12751	forkhead box subgroup	4	C:GO:0005634; F:GO:0003677; P:GO:0006355; F:GO:0000981	C:nucleus; F:DNA binding; P:regulation of transcription, DNA-templated; F:[illegible]
Acrdi.XM_015922087.1/1-762	Monca.augustus.g24779.t1/1-576	0.177901	0.127497	probable prolyl 4-hydroxylase 3	1	F:GO:0016491	F:oxidoreductase activity
Acrdi.XM_015901099.1/1-2331	Monca.augustus.g15292.t1/1-2412	0.152075	0.127487	dixin isoform X1	3	P:GO:0050794; P:GO:0007399; P:GO:0044763	P:regulation of cellular process; P:nervous system development; P:single-o[illegible]
Acrdi.XM_015908907.1/1-657	Monca.augustus.g31887.t1/1-696	0.196325	0.12745	ribulose-phosphate 3-epimerase-like	6	P:GO:0009052; F:GO:0046872; P:GO:0044262; C:GO:0005829;	P:pentose-phosphate shunt, non-oxidative branch; F:metal ion binding; P:c[illegible]
Acrdi.XM_015913530.1/1-2190	Monca.augustus.g22315.t1/1-3000	0.166223	0.127424	Ubiquitin carboxyl-terminal hydrolase 25			
Acrdi.XM_015906043.1/1-1866	Monca.augustus.g34427.t1/1-1899	0.110914	0.127424	E3 ubiquitin- ligase RNF220 isoform X1	7	F:GO:0004842; F:GO:0061630; F:GO:0008270; C:GO:0005737;	F:ubiquitin-protein transferase activity; F:ubiquitin protein ligase activity; [illegible]
Acrdi.XM_015908162.1/1-309	Monca.augustus.g10446.t1/1-315	0.189122	0.127404	cytochrome c oxidase 20 homolog	4	P:GO:0033617; C:GO:0005739; C:GO:0016020; P:GO:0009060	P:mitochondrial respiratory chain complex IV assembly; C:mitochondrion; [illegible]
Acrdi.XM_015907540.1/1-621	Monca.augustus.g3197.t1/1-345	0.461075	0.127397	ATP-dependent DNA helicase PIF1			
Acrdi.XM_015899866.1/1-1524	Monca.augustus.g48631.t1/1-1524	0.17567	0.12738	CDP-diacylglycerol--glycerol-3-phosphate 3- mitochondrial	1	F:GO:0016740	F:transferase activity
Acrdi.XM_015916770.1/1-2856	Monca.augustus.g62794.t1/1-2895	0.170644	0.127332	helicase POLQ-like	3	F:GO:0097159; F:GO:1901363; F:GO:0016787	F:organic cyclic compound binding; F:heterocyclic compound binding; F:hyd[illegible]
Acrdi.XM_015899042.1/1-1455	Monca.augustus.g27056.t1/1-1458	0.12524	0.127285	carboxypeptidase D-like	5	C:GO:0005615; F:GO:0004181; P:GO:0006518; F:GO:0004185;	C:extracellular space; F:metallocarboxypeptidase activity; P:peptide metab[illegible]
Acrdi.XM_015903058.1/1-678	Monca.augustus.g10954.t1/1-684	0.193192	0.127253	sorting nexin-10B-like	4	C:GO:0005768; C:GO:0016020; F:GO:0008289; P:GO:0006810	C:endosome; C:membrane; F:lipid binding; P:transport
Acrdi.XM_015923674.1/1-684	Monca.augustus.g30539.t1/1-675	0.0660239	0.12724	retinitis pigmentosa 9			
Acrdi.XM_015898867.1/1-681	Monca.augustus.g30796.t1/1-684	0.155885	0.127219	P2X purinoceptor 7-like			
Acrdi.XM_015919182.1/1-2859	Monca.augustus.g54947.t1/1-2991	0.17243	0.127204	phospholipase D2 isoform X1	2	P:GO:0044699; P:GO:0009987	P:single-organism process; P:cellular process
Acrdi.XM_015903209.1/1-1107	Monca.augustus.g51688.t1/1-957	0.138276	0.127159	Serine dehydratase-like	3	F:GO:0030170; C:GO:0005737; P:GO:0006520	F:pyridoxal phosphate binding; C:cytoplasm; P:cellular amino acid metabol[illegible]
Acrdi.XM_015910405.1/1-924	Monca.augustus.g10946.t1/1-930	0.133775	0.127149	FAM181A			
Acrdi.XM_015897726.1/1-621	Monca.adi2mcaRNA6651_R0/1-591	0.13817	0.127138	barH-like 1 homeobox	3	C:GO:0005634; P:GO:0006355; F:GO:0043565	C:nucleus; P:regulation of transcription, DNA-templated; F:sequence-specif[illegible]
Acrdi.XM_015907355.1/1-1062	Monca.augustus.g35200.t1/1-1464	0.170891	0.127133	dol-P-Man:Man(7) c(2)-PP-Dol alpha-1,6-mannosyltransferase	3	F:GO:0000030; P:GO:0035251; F:GO:0008378	F:mannosyltransferase activity; F:UDP-glucosyltransferase activity; F:galac[illegible]
Acrdi.XM_015892587.1/1-483	Monca.augustus.g69601.t1/1-486	0.188083	0.127124	calcium channel flower homolog isoform X1	1	C:GO:0016020	C:membrane
Acrdi.XM_015902953.1/1-654	Monca.adi2mcaRNA12344_R0/1-651	0.159147	0.127124	Mitochondrial coenzyme A transporter SLC25A42	5	P:GO:0055085; F:GO:0003735; C:GO:0016021; C:GO:0005743;	P:transmembrane transport; F:structural constituent of ribosome; C:integr[illegible]
Acrdi.XM_015901649.1/1-1050	Monca.augustus.g54285.t1/1-1656	0.0895735	0.127119	MAGUK p55 subfamily member 2			
Acrdi.XM_015910806.1/1-549	Monca.adi2mcaRNA21014_R0/1-519	0.172282	0.127118	CD151 antigen-like	1	C:GO:0016020	C:membrane
Acrdi.XM_015895631.1/1-1932	Monca.augustus.g22272.t1/1-1920	0.168615	0.127018	filamin-A isoform X1	5	C:GO:0005623; P:GO:0065008; F:GO:0005515; P:GO:0050794;	C:cell; P:regulation of biological quality; F:protein binding; P:regulation of c[illegible]
Acrdi.XM_015901553.1/1-2832	Monca.augustus.g55986.t1/1-5448	0.145513	0.12701	Minor histocompatibility HA-1	4	P:GO:0007165; F:GO:0046872; P:GO:0035556; C:GO:0005622	P:signal transduction; F:metal ion binding; P:intracellular signal transducti[illegible]
Acrdi.XM_015897407.1/1-1608	Monca.augustus.g51666.t1/1-1611	0.122399	0.126998	TGF-beta-activated kinase 1 and MAP3K7-binding 1-like	2	F:GO:0004722; P:GO:0006470	F:protein serine/threonine phosphatase activity; P:protein dephosphorylati[illegible]
Acrdi.XM_015915465.1/1-1230	Monca.augustus.g17212.t1/1-1242	0.172972	0.126994	PREDICTED: uncharacterized protein LOC107349328			
Acrdi.XM_015919495.1/1-1686	Monca.augustus.g22482.t1/1-1671	0.167763	0.126953	Inositol-trisphosphate 3-kinase	2	F:GO:0008440; P:GO:0016310	F:inositol-1,4,5-trisphosphate 3-kinase activity; P:phosphorylation
Acrdi.XM_015910358.1/1-1683	Monca.augustus.g36431.t1/1-1665	0.146751	0.126939	retinoic acid receptor RXR-like isoform X8	3	P:GO:0050794; F:GO:0005488; P:GO:0009987	P:regulation of cellular process; F:binding; P:cellular process
Acrdi.XM_015916297.1/1-768	Monca.augustus.g70712.t1/1-1854	0.134195	0.126934	nuclear localization 4 homolog	5	C:GO:0005634; F:GO:0031625; F:GO:0043130; F:GO:0046872;	C:nucleus; F:ubiquitin protein ligase binding; F:ubiquitin binding; F:metal io[illegible]
Acrdi.XM_015900867.1/1-4917	Monca.augustus.g27866.t1/1-4962	0.159539	0.126898	Phosphatidylinositol 4-phosphate 3-kinase C2 domain-containing subu	8	C:GO:0005942; P:GO:0046488; P:GO:0016310; F:GO:0035005;	C:phosphatidylinositol 3-kinase complex; P:phosphatidylinositol metabolic [illegible]
Acrdi.XM_015917910.1/1-1311	Monca.augustus.g39685.t1/1-1338	0.117035	0.126895	iroquois A isoform 1	4	F:GO:0003677; C:GO:0005634; P:GO:0006355; F:GO:0043565	F:DNA binding; C:nucleus; P:regulation of transcription, DNA-templated; F:[illegible]
Acrdi.XM_015920655.1/1-1980	Monca.augustus.g32595.t1/1-981	0.132241	0.126881	D(2) dopamine receptor-like	3	F:GO:0004930; P:GO:0007154; P:GO:0044700	F:G-protein coupled receptor activity; P:cell communication; P:single organ[illegible]
Acrdi.XM_015910565.1/1-735	Monca.augustus.g23046.t1/1-2346	0.241978	0.126872	Zinc finger CCCH domain-containing 3	1	F:GO:0046872	F:metal ion binding
Acrdi.XM_015900172.1/1-1206	Monca.augustus.g59991.t1/1-1200	0.130772	0.12687	serine threonine- phosphatase 6 regulatory ankyrin repeat subunit B-li	3	F:GO:0004842; F:GO:0043531; P:GO:0016567	F:ubiquitin-protein transferase activity; F:ADP binding; P:protein ubiquitina[illegible]
Acrdi.XM_015905348.1/1-639	Monca.adi2mcaRNA14971_R6/1-801	0.108238	0.126854	PREDICTED: uncharacterized protein LOC107339993			
Acrdi.XM_015905213.1/1-1257	Monca.adi2mcaRNA14811_R0/1-1263	0.143913	0.126853	Synaptotagmin-9	9	C:GO:0070382; P:GO:0048791; F:GO:0019905; P:GO:0006906;	C:exocytic vesicle; P:calcium ion-regulated exocytosis of neurotransmitter; [illegible]
Acrdi.XM_015916501.1/1-525	Monca.augustus.g39000.t1/1-702	0.0905495	0.126824	tetratricopeptide repeat 33			
Acrdi.XM_015909845.1/1-1920	Monca.augustus.g47043.t1/1-2913	0.137018	0.12682	FAM65B-like isoform X5			
Acrdi.XM_015910452.1/1-1449	Monca.adi2mcaRNA20645_R0/1-1416	0.139601	0.126803	SCAI isoform X2	4	C:GO:0005634; F:GO:0003714; P:GO:0006351; P:GO:1903507	C:nucleus; F:transcription corepressor activity; P:transcription, DNA-templa[illegible]
Acrdi.XM_015911576.1/1-1800	Monca.augustus.g15386.t1/1-2055	0.126842	0.126768	zinc finger 846 isoform X1	1	F:GO:0005488	F:binding
Acrdi.XM_015912399.1/1-807	Monca.augustus.g45149.t1/1-801	0.159614	0.126765	PREDICTED: uncharacterized protein LOC107346592			
Acrdi.XM_015910135.1/1-1125	Monca.augustus.g18034.t1/1-1164	0.115044	0.126725	neuropeptide FF receptor 2-like	4	C:GO:0005887; F:GO:0008188; P:GO:0007186; P:GO:0007166	C:integral component of plasma membrane; F:neuropeptide receptor activ[illegible]
Acrdi.XM_015895666.1/1-507	Monca.augustus.g63267.t1/1-519	0.116351	0.126715	boule-like isoform X3	2	F:GO:0000166; F:GO:0003676	F:nucleotide binding; F:nucleic acid binding
Acrdi.XM_015899422.1/1-348	Monca.augustus.g46724.t1/1-747	0.16284	0.126704	2-aminoethanethiol dioxygenase-like	3	F:GO:0016491; C:GO:0005739; P:GO:0055114	F:oxidoreductase activity; C:mitochondrion; P:oxidation-reduction process
Acrdi.XM_015922557.1/1-1365	Monca.augustus.g54978.t1/1-1491	0.125242	0.126665	3-phosphoinositide-dependent kinase 1 isoform X2	43	P:GO:0007173; P:GO:0038095; P:GO:0097191; P:GO:0043122;	P:epidermal growth factor receptor signaling pathway; P:Fc-epsilon recept[illegible]
Acrdi.XM_015908102.1/1-618	Monca.adi2mcaRNA18059_R0/1-531	0.157859	0.126632	HEG-like 1			
Acrdi.XM_015907143.1/1-957	Monca.augustus.g2522.t1/1-966	0.150157	0.126609	probable G- coupled receptor 22	4	F:GO:0004930; P:GO:0007154; F:GO:0005488; P:GO:0044700	F:G-protein coupled receptor activity; P:cell communication; F:binding; P:si[illegible]
Acrdi.XM_015901764.1/1-1821	Monca.augustus.g12066.t1/1-1800	0.167745	0.126608	Major facilitator superfamily domain-containing 6	2	C:GO:0016020; C:GO:0016021	C:membrane; C:integral component of membrane
Acrdi.XM_015897712.1/1-522	Monca.adi2mcaRNA617_R0/1-480	0.161024	0.126585	PREDICTED: uncharacterized protein LOC107332949			
Acrdi.XM_015909371.1/1-654	Monca.augustus.g27150.t1/1-2505	0.142777	0.12658	dual specificity kinase shkD-like	4	F:GO:0003677; C:GO:0005634; F:GO:0046872; P:GO:0006351	F:DNA binding; C:nucleus; F:metal ion binding; P:transcription, DNA-templa[illegible]
Acrdi.XM_015902990.1/1-2025	Monca.augustus.g594.t1/1-2025	0.131163	0.126575	E3 ubiquitin- ligase BRE1-like 2-like			
Acrdi.XM_015903397.1/1-1578	Monca.augustus.g36017.t1/1-1578	0.133906	0.126551	Glutamine-rich 2	2	C:GO:0016020; C:GO:0016021	C:membrane; C:integral component of membrane
Acrdi.XM_015918857.1/1-1311	Monca.augustus.g2.t1/1-1968	0.142169	0.126548	E3 ubiquitin- ligase ZSWIM2-like	2	F:GO:0008270; F:GO:0046872	F:zinc ion binding; F:metal ion binding
Acrdi.XM_015892625.1/1-885	Monca.augustus.g16418.t1/1-906	0.140054	0.126547	PREDICTED: uncharacterized protein LOC107327901			
Acrdi.XM_015897299.1/1-441	Monca.augustus.g13108.t1/1-1797	0.134067	0.126536	PREDICTED: uncharacterized protein LOC107332559			
Acrdi.XM_015918440.1/1-9408	Monca.adi2mcaRNA29492_R0/1-9333	0.134474	0.126512	fibrillin-1-like isoform X4	3	F:GO:0005509; F:GO:0005201; C:GO:0005578	F:calcium ion binding; F:extracellular matrix structural constituent; C:prote[illegible]
Acrdi.XM_015896496.1/1-309	Monca.augustus.g52083.t1/1-396	0.122062	0.126498	von Willebrand factor type EGF and pentraxin domain-containing 1			
Acrdi.XM_015907731.1/1-2136	Monca.augustus.g62584.t1/1-2133	0.176714	0.126491	acid trehalase 1	2	C:GO:0005618; F:GO:0016787	C:cell wall; F:hydrolase activity
Acrdi.XM_015902459.1/1-921	Monca.augustus.g41954.t1/1-1026	0.145927	0.126455	Fibronectin type 3 and ankyrin repeat domains 1	3	C:GO:0005634; F:GO:0004842; P:GO:0016567	C:nucleus; F:ubiquitin-protein transferase activity; P:protein ubiquitination
Acrdi.XM_015903252.1/1-2598	Monca.augustus.g32317.t1/1-3054	0.168672	0.126451	integrator complex subunit 6 isoform X1	1	C:GO:0043229	C:intracellular organelle
Acrdi.XM_015921649.1/1-1122	Monca.augustus.g47596.t1/1-1110	0.189492	0.126419	FAM53A-like			
Acrdi.XM_015910763.1/1-957	Monca.augustus.g29674.t1/1-960	0.121998	0.126396	TIGR02452 family	2	C:GO:0016020; C:GO:0016021	C:membrane; C:integral component of membrane
Acrdi.XM_015919377.1/1-6123	Monca.adi2mcaRNA30548_R0/1-6276	0.18709	0.126375	Rho guanine nucleotide exchange factor	3	P:GO:0035023; F:GO:0005089; P:GO:0043547	P:regulation of Rho protein signal transduction; F:Rho guanyl-nucleotide e[illegible]
Acrdi.XM_015897878.1/1-1017	Monca.augustus.g66882.t1/1-1257	0.0885229	0.126372	myb A	2	P:GO:0016180; C:GO:0032039	P:snRNA processing; C:integrator complex
Acrdi.XM_015904235.1/1-354	Monca.augustus.g62129.t1/1-438	0.100044	0.126371	mago nashi homolog 2	13	C:GO:0035145; C:GO:0016607; P:GO:0006369; P:GO:0000398;	C:exon-exon junction complex; C:nuclear speck; P:termination of RNA poly[illegible]
Acrdi.XM_015894218.1/1-1662	Monca.augustus.g66221.t1/1-5448	0.196897	0.126369	rho guanine nucleotide exchange factor 11-like			
Acrdi.XM_015911885.1/1-843	Monca.augustus.g20947.t1/1-975	0.138473	0.126364	quinone oxidoreductase	11	F:GO:0008270; C:GO:0070062; P:GO:0051289; C:GO:0005737;	F:zinc ion binding; C:extracellular exosome; P:protein homotetramerization[illegible]
Acrdi.XM_015912370.1/1-6615	Monca.adi2mcaRNA22715_R0/1-6216	0.182508	0.126357	Protocadherin Fat 4			
Acrdi.XM_015912720.1/1-606	Monca.augustus.g11956.t1/1-1656	0.151999	0.126351	Caltractin ICL1f	2	F:GO:0046872; F:GO:0005509	F:metal ion binding; F:calcium ion binding
Acrdi.XM_015916135.1/1-1092	Monca.augustus.g492.t1/1-1053	0.130514	0.126346	UDP-galactose translocator-like isoform X3	5	P:GO:0015992; C:GO:0016021; P:GO:0008643; F:GO:0005351;	P:proton transport; C:integral component of membrane; P:carbohydrate tra[illegible]
Acrdi.XM_015922875.1/1-690	Monca.augustus.g16209.t1/1-780	0.138516	0.126343	40S ribosomal S3a	11	C:GO:0005634; P:GO:0045727; F:GO:0003735; C:GO:0022627;	C:nucleus; P:positive regulation of translation; F:structural constituent of ri[illegible]
Acrdi.XM_015911753.1/1-744	Monca.augustus.g16304.t1/1-729	0.168844	0.126325	Krueppel-like factor 13	1	F:GO:0005488	F:binding
Acrdi.XM_015898570.1/1-3108	Monca.augustus.g34558.t1/1-3171	0.122503	0.126324	atrial natriuretic peptide receptor 1-like	12	P:GO:0006725; P:GO:0065008; P:GO:1901360; P:GO:0006807;	P:cellular aromatic compound metabolic process; P:regulation of biologica[illegible]
Acrdi.XM_015913142.1/1-1005	Monca.adi2mcaRNA23601_R0/1-1014	0.163874	0.126323	guanine deaminase	2	F:GO:0016810; P:GO:0046098	F:hydrolase activity, acting on carbon-nitrogen (but not peptide) bonds; P:g[illegible]
Acrdi.XM_015923285.1/1-1341	Monca.augustus.g35013.t1/1-1434	0.148167	0.126252	ubiquinone biosynthesis monooxygenase mitochondrial isoform X2	4	C:GO:0005739; C:GO:0016020; F:GO:0016709; P:GO:0044711	C:mitochondrion; C:membrane; F:oxidoreductase activity, acting on paired [illegible]
Acrdi.XM_015924515.1/1-1221	Monca.augustus.g28536.t1/1-1182	0.152224	0.12623	melatonin receptor type 1B-like	3	P:GO:0007165; C:GO:0016020; F:GO:0004871	P:signal transduction; C:membrane; F:signal transducer activity
Acrdi.XM_015901700.1/1-2034	Monca.augustus.g34400.t1/1-2103	0.14884	0.126221	mutS homolog 5	4	F:GO:0003677; P:GO:0006298; C:GO:0044424; P:GO:0007127	F:DNA binding; P:mismatch repair; C:intracellular part; P:meiosis I
Acrdi.XM_015919324.1/1-3810	Monca.augustus.g22494.t1/1-3930	0.139611	0.126213	N-acetylglucosamine-1-phosphotransferase subunits alpha beta	4	F:GO:0005509; C:GO:0016020; C:GO:0016021; F:GO:0016772	F:calcium ion binding; C:membrane; C:integral component of membrane; [illegible]
Acrdi.XM_015896631.1/1-369	Monca.augustus.g22617.t1/1-375	0.164801	0.126203	PREDICTED: uncharacterized protein LOC107331933			
Acrdi.XM_015915050.1/1-696	Monca.augustus.g49269.t1/1-702	0.133895	0.126179	dnaJ homolog subfamily C member 30			
Acrdi.XM_015913389.1/1-993	Monca.augustus.g15036.t1/1-960	0.139377	0.126176	serine threonine- phosphatase CPPED1-like	3	C:GO:0016020; C:GO:0016021; F:GO:0016787	C:membrane; C:integral component of membrane; F:hydrolase activity
Acrdi.XM_015905237.1/1-2274	Monca.augustus.g1761.t1/1-2316	0.153371	0.126157	Aryl hydrocarbon receptor	5	P:GO:0006351; C:GO:0034753; F:GO:0046983; P:GO:0009410;	P:transcription, DNA-templated; C:nuclear aryl hydrocarbon receptor comp[illegible]
Acrdi.XM_015918368.1/1-504	Monca.augustus.g41671.t1/1-459	0.30459	0.126115	PREDICTED: uncharacterized protein LOC107352065			
Acrdi.XM_015898829.1/1-1434	Monca.adi2mcaRNA7890_R2/1-1416	0.428193	0.126102	PREDICTED: uncharacterized protein LOC107333964			
Acrdi.XM_015922694.1/1-327	Monca.augustus.g1931.t1/1-2079	0.11368	0.126098	Poly(ADP-ribose) glycohydrolase			
Acrdi.XM_015909034.1/1-1662	Monca.augustus.g38348.t1/1-2670	0.154967	0.126087	staphylococcal nuclease domain-containing 1-like	1	P:GO:0060255	P:regulation of macromolecule metabolic process
Acrdi.XM_015903061.1/1-978	Monca.augustus.g56767.t1/1-7536	0.149435	0.126083	very KIND-like	2	P:GO:0065007; F:GO:0005085	P:biological regulation; F:guanyl-nucleotide exchange factor activity
Acrdi.XM_015905214.1/1-1143	Monca.augustus.g52834.t1/1-1176	0.146045	0.126041	vicilin-like seed storage At2g18540			
Acrdi.XM_015899143.1/1-1107	Monca.adi2mcaRNA8269_R7/1-1011	0.489773	0.126039	C2H2 zinc finger	3	F:GO:0003676; F:GO:0003677; F:GO:0046872	F:nucleic acid binding; F:DNA binding; F:metal ion binding
Acrdi.XM_015904736.1/1-24048	Monca.augustus.g31927.t1/1-35208	0.157367	0.126022	titin isoform X2			
Acrdi.XM_015898696.1/1-1725	Monca.augustus.g2086.t1/1-3114	0.11912	0.126005	Prostaglandin F synthase	2	F:GO:0016491; P:GO:0055114	F:oxidoreductase activity; P:oxidation-reduction process
Acrdi.XM_015912955.1/1-972	Monca.augustus.g51636.t1/1-933	0.180763	0.126003	Ketimine reductase mu-crystallin	14	F:GO:0042803; F:GO:0016639; P:GO:0042403; P:GO:0000122;	F:protein homodimerization activity; F:oxidoreductase activity, acting on th[illegible]
Acrdi.XM_015901391.1/1-822	Monca.augustus.g70851.t1/1-822	0.173036	0.125993	Fibroblast growth factor 8			
Acrdi.XM_015920133.1/1-618	Monca.augustus.g27122.t1/1-570	0.180652	0.125981	Neuronal calcium sensor 1	17	F:GO:0019901; P:GO:0050806; F:GO:0008427; F:GO:0005245;	F:protein kinase binding; P:positive regulation of synaptic transmission; F:[illegible]
Acrdi.XM_015898029.1/1-1251	Monca.augustus.g39415.t1/1-1245	0.162911	0.125959	arylacetamide deacetylase-like	5	P:GO:0008152; C:GO:0016020; C:GO:0016021; F:GO:0052689;	P:metabolic process; C:membrane; C:integral component of membrane; F:[illegible]
Acrdi.XM_015906482.1/1-2493	Monca.augustus.g22573.t1/1-3318	0.12328	0.125949	Nuclear export mediator factor Nemf			
Acrdi.XM_015905050.1/1-4368	Monca.adi2mcaRNA14640_R0/1-4194	0.209037	0.125885	Forkhead-associated domain-containing 1			
Acrdi.XM_015911905.1/1-858	Monca.augustus.g24242.t1/1-831	0.180797	0.125808	PREDICTED: uncharacterized protein LOC107346125 isoform X2			
Acrdi.XM_015906610.1/1-1275	Monca.augustus.g4000.t1/1-1281	0.127449	0.125802	kelch 10			
Acrdi.XM_015899986.1/1-1911	Monca.augustus.g6670.t1/1-2034	0.122527	0.125801	Polycomb group ASXL2	1	P:GO:0048522	P:positive regulation of cellular process
Acrdi.XM_015910802.1/1-744	Monca.augustus.g48008.t1/1-1113	0.130481	0.125789	coiled-coil domain-containing 85C-like			
Acrdi.XM_015910682.1/1-4572	Monca.augustus.g35875.t1/1-3762	0.144228	0.125783	WD repeat-containing on Y chromosome-like	1	F:GO:0005509	F:calcium ion binding
Acrdi.XM_015892006.1/1-1308	Monca.augustus.g830.t1/1-1362	0.117279	0.125773	PREDICTED: uncharacterized protein LOC107327255			
Acrdi.XM_015909472.1/1-714	Monca.augustus.g35551.t1/1-1509	0.181314	0.125754	Sorting nexin-16	1	P:GO:0065007	P:biological regulation
Acrdi.XM_015902125.1/1-660	Monca.augustus.g55028.t1/1-660	0.167137	0.125738	hemK methyltransferase family member 2	2	F:GO:0008168; P:GO:0032259	F:methyltransferase activity; P:methylation
Acrdi.XM_015917204.1/1-1581	Monca.augustus.g59096.t1/1-324	0.109798	0.125733	prolyl	4	C:GO:0005737; P:GO:0006508; F:GO:0004252; F:GO:0070008	C:cytoplasm; P:proteolysis; F:serine-type endopeptidase activity; F:serine-t[illegible]
Acrdi.XM_015906055.1/1-1776	Monca.adi2mcaRNA15745_R0/1-1785	0.140811	0.125729	mucolipin-3 isoform X1	1	C:GO:0016020	C:membrane
Acrdi.XM_015901530.1/1-699	Monca.adi2mcaRNA10800_R0/1-636	0.229165	0.125726	CDV3 homolog isoform X1	1	C:GO:0005737	C:cytoplasm
Acrdi.XM_015906012.1/1-2247	Monca.augustus.g41402.t1/1-2247	0.169043	0.125705	ATP-binding cassette sub-family B member 9	9	P:GO:0035264; F:GO:0005524; P:GO:0055085; F:GO:0015440;	P:multicellular organism growth; F:ATP binding; P:transmembrane transpo[illegible]
Acrdi.XM_015903206.1/1-1233	Monca.adi2mcaRNA12611_R0/1-1278	0.21041	0.125697	Peroxisomal acyl-coenzyme A oxidase 3	9	F:GO:0003995; C:GO:0005777; P:GO:0033539; F:GO:0000062;	F:acyl-CoA dehydrogenase activity; C:peroxisome; P:fatty acid beta-oxidatio[illegible]
Acrdi.XM_015896658.1/1-1242	Monca.augustus.g12343.t1/1-2301	0.163573	0.125696	sodium-coupled neutral amino acid transporter 10 isoform X1	4	C:GO:0016020; F:GO:0015171; P:GO:0003333; P:GO:0060348	C:membrane; F:amino acid transmembrane transporter activity; P:amino a[illegible]
Acrdi.XM_015921755.1/1-1149	Monca.augustus.g33627.t1/1-2277	0.0791439	0.12569	dystrophin-like isoform X5	6	F:GO:0008270; F:GO:0003779; F:GO:0046872; F:GO:0002162;	F:zinc ion binding; F:actin binding; F:metal ion binding; F:dystroglycan bind[illegible]
Acrdi.XM_015925076.1/1-1023	Monca.augustus.g62627.t1/1-1083	0.211971	0.125685	probable E3 SUMO- ligase RNF212 isoform X1	1	F:GO:0046872	F:metal ion binding
Acrdi.XM_015904268.1/1-507	Monca.augustus.g8635.t1/1-519	0.144942	0.125648	E3 ubiquitin- ligase RNF34-like isoform X1			
Acrdi.XM_015898900.1/1-885	Monca.augustus.g7901.t1/1-1365	0.12862	0.125578	tRNA (guanine(37)-N1)-methyltransferase	4	P:GO:0008033; C:GO:0005739; F:GO:0009019; P:GO:0032259	P:tRNA processing; C:mitochondrion; F:tRNA (guanine-N1-)-methyltransfe[illegible]
Acrdi.XM_015916353.1/1-1794	Monca.adi2mcaRNA27141_R0/1-1782	0.12708	0.125577	lymphoid-specific helicase isoform X1	1	F:GO:0005524	F:ATP binding
Acrdi.XM_015896059.1/1-1371	Monca.adi2mcaRNA4939_R1/1-1338	0.115648	0.125529	coiled-coil domain-containing 60 isoform X2			
Acrdi.XM_015897155.1/1-3153	Monca.adi2mcaRNA6103_R0/1-3114	0.146086	0.125511	Transmembrane prolyl 4-hydroxylase	2	F:GO:0016491; F:GO:0046872	F:oxidoreductase activity; F:metal ion binding
Acrdi.XM_015899362.1/1-1005	Monca.augustus.g44803.t1/1-1611	0.12943	0.125494	Ran-binding 9	1	C:GO:0044424	C:intracellular part
Acrdi.XM_015920252.1/1-1125	Monca.augustus.g49054.t1/1-1110	0.1571	0.125482	F-box LRR-repeat 8			
Acrdi.XM_015892569.1/1-504	Monca.adi2mcaRNA32053_R0/1-426	0.126165	0.12547	Enoyl- hydratase domain-containing mitochondrial	3	F:GO:0003824; C:GO:0005739; P:GO:0008152	F:catalytic activity; C:mitochondrion; P:metabolic process
Acrdi.XM_015894876.1/1-1512	Monca.augustus.g70482.t1/1-1662	0.133932	0.125469	folylpolyglutamate mitochondrial isoform X1	2	P:GO:1901564; P:GO:0019752	P:organonitrogen compound metabolic process; P:carboxylic acid metaboli[illegible]
Acrdi.XM_015907532.1/1-561	Monca.augustus.g45772.t1/1-918	0.1577	0.125462	DDRGK domain-containing 1	3	C:GO:0016021; C:GO:0005783; P:GO:1990592	C:integral component of membrane; C:endoplasmic reticulum; P:protein K[illegible]
Acrdi.XM_015914229.1/1-768	Monca.augustus.g2171.t1/1-609	0.348039	0.125431	Integrase recombinase xerD-like	3	F:GO:0003677; P:GO:0015074; P:GO:0006310	F:DNA binding; P:DNA integration; P:DNA recombination
Acrdi.XM_015892678.1/1-930	Monca.augustus.g58947.t1/1-1020	0.155995	0.125427	urease accessory D-like	3	P:GO:0043085; P:GO:0006807; F:GO:0016151	P:positive regulation of catalytic activity; P:nitrogen compound metabolic [illegible]
Acrdi.XM_015923008.1/1-1116	Monca.augustus.g46066.t1/1-2763	0.188546	0.125403	metabotropic glutamate receptor-like	7	F:GO:0004930; P:GO:0007186; P:GO:0007165; C:GO:0016020;	F:G-protein coupled receptor activity; P:G-protein coupled receptor signali[illegible]
Acrdi.XM_015900582.1/1-1152	Monca.augustus.g33654.t1/1-1221	0.129039	0.125394	Proteasomal ATPase-associated factor 1	2	F:GO:0005515; C:GO:0000502	F:protein binding; C:proteasome complex
Acrdi.XM_015897828.1/1-2703	Monca.augustus.g11735.t1/1-2748	0.13498	0.125391	PREDICTED: uncharacterized protein LOC107333070			
Acrdi.XM_015915115.1/1-2256	Monca.augustus.g26563.t1/1-2364	0.158771	0.125366	Wings apart	13	P:GO:0035562; C:GO:0015630; P:GO:0022402; P:GO:0045875;	P:negative regulation of chromatin binding; C:microtubule cytoskeleton; P:[illegible]
Acrdi.XM_015897685.1/1-582	Monca.augustus.g46349.t1/1-717	0.0913143	0.125338	ras-related Rab-14	6	C:GO:0005769; P:GO:0090387; C:GO:0045335; C:GO:0005794;	C:early endosome; P:phagolysosome assembly involved in apoptotic cell cl[illegible]
Acrdi.XM_015923983.1/1-930	Monca.augustus.g54030.t1/1-1662	0.127452	0.125334	cilia- and flagella-associated 45-like	4	F:GO:0003677; P:GO:0050794; F:GO:0046872; P:GO:0009987	F:DNA binding; P:regulation of cellular process; F:metal ion binding; P:cellu[illegible]
Acrdi.XM_015899306.1/1-2124	Monca.augustus.g54010.t1/1-972	0.182532	0.125331	isoaspartyl peptidase L-asparaginase-like	1	F:GO:0016787	F:hydrolase activity
Acrdi.XM_015924876.1/1-1293	Monca.augustus.g41437.t1/1-1749	0.142304	0.12533	nudC domain-containing 1-like			
Acrdi.XM_015921066.1/1-4377	Monca.augustus.g34989.t1/1-8298	0.16046	0.125321	Zinc finger FYVE domain-containing 26	4	F:GO:0046872; P:GO:0000724; P:GO:0000910; F:GO:0032266	F:metal ion binding; P:double-strand break repair via homologous recombi[illegible]
Acrdi.XM_015908026.1/1-957	Monca.augustus.g5043.t1/1-2211	0.101517	0.125302	PC3-like endoprotease variant B	2	P:GO:0006508; F:GO:0008236	P:proteolysis; F:serine-type peptidase activity
Acrdi.XM_015903978.1/1-6228	Monca.augustus.g27732.t1/1-6306	0.107319	0.125293	Myoferlin	2	C:GO:0016020; C:GO:0016021	C:membrane; C:integral component of membrane
Acrdi.XM_015922853.1/1-1524	Monca.augustus.g19811.t1/1-1560	0.104517	0.125265	N-acetylglucosamine-6-sulfatase-like	2	F:GO:0008484; P:GO:0008152	F:sulfuric ester hydrolase activity; P:metabolic process
Acrdi.XM_015924812.1/1-1173	Monca.adi2mcaRNA36670_R0/1-1146	0.717211	0.125256	Gag-Pol poly	2	C:GO:0016020; C:GO:0016021	C:membrane; C:integral component of membrane
Acrdi.XM_015917366.1/1-537	Monca.augustus.g23807.t1/1-720	0.0931415	0.125255	39S ribosomal mitochondrial	1	C:GO:0044429	C:mitochondrial part
Acrdi.XM_015893154.1/1-1056	Monca.adi2mcaRNA39206_R0/1-993	0.147008	0.125236	myophilin-like isoform X2			
Acrdi.XM_015896168.1/1-1416	Monca.augustus.g23026.t1/1-2223	0.13916	0.125235	ribosome biogenesis BOP1	7	P:GO:0000463; F:GO:0043021; C:GO:0070545; P:GO:0051726;	P:maturation of LSU-rRNA from tricistronic rRNA transcript (SSU-rRNA, 5.[illegible]
Acrdi.XM_015912870.1/1-726	Monca.augustus.g14934.t1/1-1107	0.164729	0.125231	cytoplasmic dynein 2 light intermediate chain 1-like	3	C:GO:0036064; F:GO:0045504; C:GO:0005930	C:ciliary basal body; F:dynein heavy chain binding; C:axoneme
Acrdi.XM_015912264.1/1-2379	Monca.augustus.g28336.t1/1-7350	0.131621	0.125227	Actin filament-associated 1	5	F:GO:0005070; C:GO:0005737; P:GO:0002757; P:GO:0009967;	F:SH3/SH2 adaptor activity; C:cytoplasm; P:immune response-activating si[illegible]
Acrdi.XM_015904470.1/1-2646	Monca.adi2mcaRNA14041_R0/1-2547	0.196072	0.125222	Testis-expressed sequence 11	1	P:GO:0051321	P:meiotic cell cycle
Acrdi.XM_015920211.1/1-2274	Monca.augustus.g8890.t1/1-2109	0.14551	0.125188	TBC1 domain family member 14 isoform X1	7	P:GO:0031338; P:GO:0090630; F:GO:0005096; C:GO:0012505;	P:regulation of vesicle fusion; P:activation of GTPase activity; F:GTPase ac[illegible]
Acrdi.XM_015909539.1/1-816	Monca.augustus.g1397.t1/1-924	0.14441	0.12516	aquaporin 7	7	C:GO:0005887; F:GO:0015254; P:GO:0009992; P:GO:0034220;	C:integral component of plasma membrane; F:glycerol channel activity; P:[illegible]
Acrdi.XM_015912280.1/1-1080	Monca.augustus.g3410.t1/1-1086	0.102804	0.125108	zinc phosphodiesterase ELAC 1-like	3	P:GO:0090502; P:GO:0042779; F:GO:0042781	P:RNA phosphodiester bond hydrolysis, endonucleolytic; P:tRNA 3'-trailer c[illegible]
Acrdi.XM_015914459.1/1-960	Monca.augustus.g71049.t1/1-957	0.149289	0.125023	PREDICTED: uncharacterized protein LOC107348420	1	F:GO:0016787	F:hydrolase activity
Acrdi.XM_015892817.1/1-810	Monca.augustus.g11826.t1/1-789	0.22798	0.124984	trimeric intracellular cation channel type B-B	3	P:GO:0015672; C:GO:0016020; F:GO:0005261	P:monovalent inorganic cation transport; C:membrane; F:cation channel ac[illegible]
Acrdi.XM_015897318.1/1-612	Monca.augustus.g29208.t1/1-1107	0.125353	0.124958	betaine--homocysteine S-methyltransferase			
Acrdi.XM_015919046.1/1-1785	Monca.augustus.g26984.t1/1-1818	0.156974	0.124951	Synembryn-A	6	P:GO:0007186; C:GO:0005737; F:GO:0001965; F:GO:0005085;	P:G-protein coupled receptor signaling pathway; C:cytoplasm; F:G-protein [illegible]
Acrdi.XM_015893654.1/1-1644	Monca.augustus.g545.t1/1-1635	0.107563	0.124938	coiled-coil domain-containing 63-like	2	C:GO:0036157; P:GO:0036158	C:outer dynein arm; P:outer dynein arm assembly
Acrdi.XM_015923963.1/1-1050	Monca.augustus.g34524.t1/1-1053	0.12416	0.124929	neuropeptide Y receptor type 2-like	2	C:GO:0016020; P:GO:0051716	C:membrane; P:cellular response to stimulus
Acrdi.XM_015915787.1/1-2001	Monca.augustus.g1470.t1/1-1926	0.143457	0.12491	tubulin--tyrosine ligase 12	2	P:GO:0006464; F:GO:0016874	P:cellular protein modification process; F:ligase activity
Acrdi.XM_015910585.1/1-1524	Monca.adi2mcaRNA20799_R0/1-1359	0.156391	0.124907	DNA polymerase alpha subunit B	3	P:GO:0006270; C:GO:0005658; F:GO:0046982	P:DNA replication initiation; C:alpha DNA polymerase:primase complex; F:[illegible]
Acrdi.XM_015892640.1/1-1287	Monca.augustus.g63896.t1/1-1812	0.601433	0.124901	PREDICTED: uncharacterized protein LOC107327921			
Acrdi.XM_015912960.1/1-1911	Monca.adi2mcaRNA23362_R0/1-1887	0.165388	0.124878	prominin-1-A-like isoform X1	2	C:GO:0016020; C:GO:0016021	C:membrane; C:integral component of membrane
Acrdi.XM_015911174.1/1-1470	Monca.augustus.g24041.t1/1-1449	0.142753	0.124873	dynein assembly factor axonemal	1	P:GO:0030030	P:cell projection organization
Acrdi.XM_015922921.1/1-618	Monca.adi2mcaRNA34501_R1/1-624	0.550593	0.124872	Calcium-responsive transcription factor	6	F:GO:0003677; C:GO:0005634; F:GO:0003700; P:GO:0071277;	F:DNA binding; C:nucleus; F:transcription factor activity, sequence-specific [illegible]
Acrdi.XM_015916975.1/1-1263	Monca.augustus.g50847.t1/1-1464	0.168416	0.124836	aldehyde dehydrogenase family 8 member A1	5	F:GO:0004029; P:GO:0001889; F:GO:0001758; P:GO:0008152;	F:aldehyde dehydrogenase (NAD) activity; P:liver development; F:retinal de[illegible]
Acrdi.XM_015900796.1/1-1494	Monca.augustus.g58759.t1/1-1488	0.166293	0.124819	serine threonine- phosphatase 6 regulatory ankyrin repeat subunit A-like			
Acrdi.XM_015924497.1/1-519	Monca.augustus.g13473.t1/1-537	0.186726	0.124749	mitochondrial	4	F:GO:0009055; C:GO:0016020; F:GO:0008137; P:GO:0055114	F:electron carrier activity; C:membrane; F:NADH dehydrogenase (ubiquino[illegible]
Acrdi.XM_015910920.1/1-2505	Monca.augustus.g49180.t1/1-2778	0.12934	0.124701	nuclear factor NF-kappa-B p100 subunit	12	P:GO:0000122; P:GO:0034097; P:GO:0006954; P:GO:0045087;	P:negative regulation of transcription from RNA polymerase II promoter; P[illegible]
Acrdi.XM_015898085.1/1-1101	Monca.adi2mcaRNA7052_R1/1-1101	0.130732	0.12465	arrestin domain-containing 3-like	1	P:GO:0006810	P:transport
Acrdi.XM_015895562.1/1-720	Monca.augustus.g14885.t1/1-714	0.163594	0.124624	LIM and SH3 domain 1-like	2	F:GO:0008270; F:GO:0046872	F:zinc ion binding; F:metal ion binding
Acrdi.XM_015902085.1/1-747	Monca.augustus.g15069.t1/1-1944	0.146361	0.124623	RUN domain-containing 1	7	P:GO:0031338; P:GO:0090630; F:GO:0005096; C:GO:0012505;	P:regulation of vesicle fusion; P:activation of GTPase activity; F:GTPase ac[illegible]
Acrdi.XM_015905948.1/1-1206	Monca.augustus.g58516.t1/1-1179	0.18113	0.124586	E3 ubiquitin- ligase MARCH9-like	2	F:GO:0046872; C:GO:0016020	F:metal ion binding; C:membrane
Acrdi.XM_015924794.1/1-1911	Monca.augustus.g43723.t1/1-2418	0.134907	0.12455	Sodium-dependent neutral amino acid transporter B(0)AT3	5	C:GO:0005887; P:GO:0055085; F:GO:0015293; F:GO:0015171;	C:integral component of plasma membrane; P:transmembrane transport; [illegible]
Acrdi.XM_015910728.1/1-465	Monca.augustus.g29676.t1/1-1491	0.125025	0.124529	Leucine-rich repeat-containing 74A	1	F:GO:0005509	F:calcium ion binding
Acrdi.XM_015914604.1/1-1452	Monca.augustus.g57118.t1/1-1428	0.137946	0.124521	PREDICTED: uncharacterized protein LOC107348549 isoform X1			
Acrdi.XM_015916435.1/1-1794	Monca.augustus.g64490.t1/1-1758	0.13136	0.124496	receptor-type guanylate cyclase daf-11 isoform X2	5	F:GO:0016849; C:GO:0016020; C:GO:0044464; P:GO:0044699;	F:phosphorus-oxygen lyase activity; C:membrane; C:cell part; P:single-orga[illegible]
Acrdi.XM_015903517.1/1-1533	Monca.augustus.g34548.t1/1-1530	0.11438	0.124496	PREDICTED: uncharacterized protein LOC107338287	4	P:GO:0036066; C:GO:0016020; C:GO:0016021; F:GO:0046922	P:protein O-linked fucosylation; C:membrane; C:integral component of me[illegible]
Acrdi.XM_015905324.1/1-945	Monca.adi2mcaRNA14956_R0/1-948	0.165357	0.124495	melanocyte-stimulating hormone receptor-like	7	F:GO:0004930; F:GO:0004977; P:GO:0007186; P:GO:0007165;	F:G-protein coupled receptor activity; F:melanocortin receptor activity; P:G[illegible]
Acrdi.XM_015907281.1/1-2193	Monca.augustus.g8083.t1/1-2664	0.166507	0.124484	Exostosin-like 3	5	P:GO:0015012; F:GO:0008375; C:GO:0016020; C:GO:0016021;	P:heparan sulfate proteoglycan biosynthetic process; F:acetylglucosaminyl[illegible]
Acrdi.XM_015906833.1/1-1080	Monca.augustus.g4903.t1/1-1131	0.134251	0.124456	N-acyl-phosphatidylethanolamine-hydrolyzing phospholipase D-like	2	F:GO:0008270; F:GO:0070290	F:zinc ion binding; F:N-acylphosphatidylethanolamine-specific phospholipa[illegible]
Acrdi.XM_015920377.1/1-1173	Monca.augustus.g47497.t1/1-1170	0.100419	0.124435	Chitin deacetylase	2	P:GO:0071704; F:GO:0016787	P:organic substance metabolic process; F:hydrolase activity
Acrdi.XM_015918340.1/1-1998	Monca.augustus.g23514.t1/1-1968	0.162384	0.124421	probable ATP-dependent RNA helicase DDX59	2	P:GO:0010501; F:GO:0004004	P:RNA secondary structure unwinding; F:ATP-dependent RNA helicase acti[illegible]
Acrdi.XM_015918183.1/1-627	Monca.augustus.g20700.t1/1-885	0.158723	0.124419	Brain-specific angiogenesis inhibitor 3			
Acrdi.XM_015894021.1/1-648	Monca.augustus.g11234.t1/1-1929	0.179412	0.124402	Cytoplasmic dynein 1 intermediate chain	2	C:GO:0005868; P:GO:0007018	C:cytoplasmic dynein complex; P:microtubule-based movement
Acrdi.XM_015908072.1/1-762	Monca.augustus.g7864.t1/1-759	0.120547	0.124398	PREDICTED: uncharacterized protein LOC107342568			
Acrdi.XM_015893110.1/1-1191	Monca.augustus.g44192.t1/1-1674	0.174002	0.124387	HBS1 isoform X1	1	P:GO:0009987	P:cellular process
Acrdi.XM_015913049.1/1-1128	Monca.augustus.g38043.t1/1-1116	0.124182	0.124384	Prostaglandin E2 receptor EP4 subtype	6	F:GO:0004930; P:GO:0007186; P:GO:0007165; C:GO:0016020;	F:G-protein coupled receptor activity; P:G-protein coupled receptor signali[illegible]
Acrdi.XM_015901088.1/1-525	Monca.augustus.g57659.t1/1-726	0.168574	0.124373	Transmembrane 98	3	C:GO:0016020; C:GO:0016021; C:GO:0005783	C:membrane; C:integral component of membrane; C:endoplasmic reticulu[illegible]
Acrdi.XM_015906264.1/1-750	Monca.adi2mcaRNA16025_R0/1-897	0.565749	0.124351	mediator of RNA polymerase II transcription subunit 34-like	7	C:GO:0005634; F:GO:0003677; C:GO:0005737; F:GO:0043140;	C:nucleus; F:DNA binding; C:cytoplasm; F:ATP-dependent 3'-5' DNA helicas[illegible]
Acrdi.XM_015912222.1/1-1353	Monca.augustus.g22428.t1/1-1458	0.113751	0.124343	G2 mitotic-specific cyclin-B3	4	C:GO:0005634; F:GO:0019901; P:GO:0010389; P:GO:0000079	C:nucleus; F:protein kinase binding; P:regulation of G2/M transition of mit[illegible]
Acrdi.XM_015918681.1/1-624	Monca.augustus.g47192.t1/1-2223	0.184058	0.124328	FAM149B1-like isoform X2			
Acrdi.XM_015910925.1/1-1917	Monca.augustus.g1732.t1/1-1935	0.151391	0.124327	PREDICTED: uncharacterized protein LOC107345213			
Acrdi.XM_015911840.1/1-969	Monca.augustus.g21439.t1/1-972	0.121194	0.124326	wnt inhibitory factor 1	2	P:GO:0045600; C:GO:0005576	P:positive regulation of fat cell differentiation; C:extracellular region
Acrdi.XM_015899527.1/1-2436	Monca.adi2mcaRNA8635_R0/1-2538	0.111502	0.124284	neurocalcin homolog	1	F:GO:0005509	F:calcium ion binding
Acrdi.XM_015923685.1/1-3453	Monca.adi2mcaRNA35378_R0/1-3243	0.186739	0.124274	WD repeat-containing 27			
Acrdi.XM_015912716.1/1-2031	Monca.adi2mcaRNA2395_R0/1-1974	0.104159	0.124264	1-phosphatidylinositol 4,5-bisphosphate phosphodiesterase delta-	3	P:GO:0007165; P:GO:0006629; F:GO:0008081	P:signal transduction; P:lipid metabolic process; F:phosphoric diester hydro[illegible]
Acrdi.XM_015908668.1/1-810	Monca.augustus.g53880.t1/1-708	0.179185	0.124258	MIT domain-containing 1			
Acrdi.XM_015917481.1/1-5109	Monca.augustus.g32477.t1/1-5433	0.133923	0.124245	transcription initiation factor TFIID subunit 1 isoform X3	4	F:GO:0003677; C:GO:0005669; P:GO:0006355; P:GO:0006352	F:DNA binding; C:transcription factor TFIID complex; P:regulation of transc[illegible]
Acrdi.XM_015901550.1/1-2097	Monca.augustus.g55974.t1/1-3540	0.124885	0.124211	F-BAR domain only 2-like			
Acrdi.XM_015916397.1/1-840	Monca.augustus.g52612.t1/1-843	0.125078	0.124184	PREDICTED: uncharacterized protein LOC107350169	2	C:GO:0016020; C:GO:0016021	C:membrane; C:integral component of membrane
Acrdi.XM_015899942.1/1-1572	Monca.augustus.g32797.t1/1-1584	0.150398	0.124172	hairy enhancer-of-split related with YRPW motif	4	C:GO:0005634; P:GO:0050794; P:GO:0048664; F:GO:0005488	C:nucleus; P:regulation of cellular process; P:neuron fate determination; F:[illegible]
Acrdi.XM_015906927.1/1-741	Monca.augustus.g17318.t1/1-699	0.135852	0.124142	gamma-aminobutyric acid type B receptor subunit 1-like	5	F:GO:0004930; F:GO:0004965; P:GO:0007186; C:GO:0016020;	F:G-protein coupled receptor activity; F:G-protein coupled GABA receptor a[illegible]
Acrdi.XM_015897656.1/1-570	Monca.augustus.g9978.t1/1-627	0.145751	0.124137	40S ribosomal S5	17	C:GO:0022627; P:GO:0006450; P:GO:0000028; P:GO:0006364;	C:cytosolic small ribosomal subunit; P:regulation of translational fidelity; P[illegible]
Acrdi.XM_015904107.1/1-576	Monca.adi2mcaRNA13576_R6/1-573	0.0756496	0.124106	PREDICTED: uncharacterized protein LOC107338853	3	F:GO:0003676; C:GO:0016020; C:GO:0016021	F:nucleic acid binding; C:membrane; C:integral component of membrane
Acrdi.XM_015905116.1/1-447	Monca.augustus.g44852.t1/1-471	0.483201	0.124095	PREDICTED: uncharacterized protein LOC107339803			
Acrdi.XM_015910481.1/1-1152	Monca.augustus.g16588.t1/1-2160	0.0620484	0.124056	Disintegrin and metallo ase domain-containing 10	6	F:GO:0008270; P:GO:0006508; C:GO:0016020; C:GO:0016021;	F:zinc ion binding; P:proteolysis; C:membrane; C:integral component of me[illegible]
Acrdi.XM_015896350.1/1-1887	Monca.augustus.g54413.t1/1-1893	0.149979	0.124034	GH3 domain-containing			
Acrdi.XM_015895566.1/1-2292	Monca.augustus.g2044.t1/1-2331	0.121578	0.124007	proteoglycan 4-like			

Acrdi.XM_015918072.1/1-402 | Monca.augustus.g34643.t1/1-417	0.192276	0.123972	proteasome assembly chaperone 4	1	P:GO:0043248	P:proteasome assembly
Acrdi.XM_015905094.1/1-3009 | Monca.augustus.g24495.t1/1-3411	0.146787	0.123945	PREDICTED: uncharacterized protein LOC107339777			
Acrdi.XM_015905577.1/1-627 | Monca.augustus.g24097.t1/1-1614	0.208542	0.123941	iduronate 2-sulfatase-like	1	F:GO:0003824	F:catalytic activity
Acrdi.XM_015918856.1/1-546 | Monca.augustus.g1.t1/1-1023	0.174328	0.123941	FAM221A isoform X1			
Acrdi.XM_015918742.1/1-1440 | Monca.augustus.g10061.t1/1-1497	0.530029	0.12394	Retrovirus-related Pol poly from transposon			
Acrdi.XM_015909044.1/1-5547 | Monca.augustus.g38365.t1/1-6147	0.156271	0.123936	nuclear pore complex Nup205	4	P:GO:0006999; F:GO:0017056; C:GO:0044611; P:GO:0000059	[illegible]
Acrdi.XM_015902734.1/1-471 | Monca.adi2mcaRNA12114_R0/1-564	0.176385	0.123934	probable cardiolipin synthase (CMP-forming)	5	[illegible]	[illegible]
Acrdi.XM_015916679.1/1-1110 | Monca.augustus.g58392.t1/1-1113	0.112719	0.123917	proline-rich transmembrane 4-like	2	C:GO:0016020; C:GO:0016021	[illegible]
Acrdi.XM_015903168.1/1-681 | Monca.augustus.g52462.t1/1-672	0.154297	0.123909	grpE homolog mitochondrial	2	F:GO:0005515; C:GO:0005739	[illegible]
Acrdi.XM_015920817.1/1-552 | Monca.adi2mcaRNA32126_R1/1-678	0.621649	0.123903	PREDICTED: uncharacterized protein LOC107354350			
Acrdi.XM_015912758.1/1-1446 | Monca.augustus.g26421.t1/1-2142	0.168094	0.123896	DNA repair and recombination RAD54-like	4	F:GO:0097159; F:GO:1901363; P:GO:0006259; P:GO:0006974	[illegible]
Acrdi.XM_015907935.1/1-1362 | Monca.augustus.g62655.t1/1-1353	0.0993211	0.123896	Tetratricopeptide repeat 38			
Acrdi.XM_015905801.1/1-1044 | Monca.augustus.g31765.t1/1-1152	0.15611	0.123887	WD repeat-containing 86-like			
Acrdi.XM_015900929.1/1-1557 | Monca.augustus.g27895.t1/1-1557	0.154927	0.123875	steroid 17-alpha-hydroxylase 17,20 lyase-like isoform X2	4	C:GO:0005783; C:GO:0044425; C:GO:0044446; F:GO:0004497	[illegible]
Acrdi.XM_015924895.1/1-1374 | Monca.adi2mcaRNA36784_R0/1-1311	0.137771	0.123842	amine oxidase [flavin-containing] B	6	[illegible]	[illegible]
Acrdi.XM_015915298.1/1-420 | Monca.augustus.g25269.t1/1-423	0.0427202	0.123823	cornichon homolog 1-like	2	C:GO:0016021; P:GO:0006810	[illegible]
Acrdi.XM_015916594.1/1-1677 | Monca.adi2mcaRNA27415_R0/1-1320	0.531757	0.123803	RNA-directed DNA polymerase from mobile element jockey-like			
Acrdi.XM_015895908.1/1-2532 | Monca.adi2mcaRNA166_R0/1-2745	0.181228	0.123785	Collagen alpha-1(XII) chain			
Acrdi.XM_015900472.1/1-1254 | Monca.augustus.g49634.t1/1-1263	0.128456	0.123743	Suppressor APC domain-containing 2			
Acrdi.XM_015905790.1/1-4923 | Monca.adi2mcaRNA15480_R5/1-3153	0.308868	0.123682	Pao retrotransposon peptidase superfamily	2	F:GO:0003676; P:GO:0015074	[illegible]
Acrdi.XM_015917741.1/1-13776 | Monca.adi2mcaRNA2898_R0/1-13251	0.172229	0.123651	probable E3 ubiquitin- ligase HERC1	3	F:GO:0004842; F:GO:0016874; P:GO:0016567	[illegible]
Acrdi.XM_015912154.1/1-2358 | Monca.adi2mcaRNA22469_R0/1-2145	0.159783	0.123643	MMS22-like			
Acrdi.XM_015900910.1/1-1221 | Monca.augustus.g53998.t1/1-1293	0.167166	0.123631	peroxisome biogenesis factor 13	3	C:GO:0005777; C:GO:0016021; P:GO:0016560	[illegible]
Acrdi.XM_015909340.1/1-399 | Monca.augustus.g27155.t1/1-552	0.0856499	0.123624	Endothelial differentiation-related factor 1	8	[illegible]	[illegible]
Acrdi.XM_015900094.1/1-1209 | Monca.augustus.g47753.t1/1-1134	0.118167	0.123622	multidrug resistance-associated 4-	4	F:GO:0005524; F:GO:0042626; P:GO:0055085; C:GO:0016021	[illegible]
Acrdi.XM_015893901.1/1-1371 | Monca.augustus.g22376.t1/1-810	0.151859	0.123615	proto-oncogene tyrosine- kinase receptor Ret-like			
Acrdi.XM_015916150.1/1-1620 | Monca.augustus.g70520.t1/1-2106	0.148264	0.123597	PREDICTED: uncharacterized protein LOC107349951			
Acrdi.XM_015907690.1/1-2514 | Monca.augustus.g29946.t1/1-1944	0.178097	0.123594	gem-associated 5			
Acrdi.XM_015892231.1/1-822 | Monca.augustus.g2820.t1/1-831	0.107426	0.123564	Phospholipid scramblase 3			
Acrdi.XM_015898231.1/1-387 | Monca.augustus.g51096.t1/1-846	0.198706	0.123561	RNA binding fox-1 homolog 2-like	2	F:GO:0000166; F:GO:0003676	[illegible]
Acrdi.XM_015920229.1/1-555 | Monca.adi2mcaRNA31485_R0/1-654	0.132232	0.123552	serine arginine-rich splicing factor 10 isoform X2	1	F:GO:0003676	F:nucleic acid binding
Acrdi.XM_015896170.1/1-1806 | Monca.adi2mcaRNA5020_R0/1-1500	0.11409	0.12355	PREDICTED: uncharacterized protein LOC107331563	1	C:GO:0016020	C:membrane
Acrdi.XM_015915948.1/1-2829 | Monca.augustus.g819.t1/1-3564	0.117389	0.123548	DNA-directed RNA polymerase I subunit RPA2	5	[illegible]	[illegible]
Acrdi.XM_015898346.1/1-1278 | Monca.augustus.g44532.t1/1-1263	0.163284	0.123539	nuclease HARBI1			
Acrdi.XM_015922153.1/1-822 | Monca.augustus.g65528.t1/1-915	0.157488	0.123534	ubiquitin-conjugating enzyme E2 Z-like	5	[illegible]	[illegible]
Acrdi.XM_015898094.1/1-1080 | Monca.augustus.g9828.t1/1-1176	0.152095	0.123517	arrestin domain-containing 3-like	3	[illegible]	[illegible]
Acrdi.XM_015902354.1/1-1653 | Monca.augustus.g31470.t1/1-1326	0.121786	0.123492	signal recognition particle receptor beta subunit	3	[illegible]	[illegible]
Acrdi.XM_015892442.1/1-1674 | Monca.augustus.g69047.t1/1-1689	0.16848	0.123491	ataxin-1-like	1	F:GO:0003723	[illegible]
Acrdi.XM_015914228.1/1-636 | Monca.augustus.g58322.t1/1-639	0.253071	0.123479	twisted gastrulation homolog 1	6	[illegible]	[illegible]
Acrdi.XM_015895522.1/1-2601 | Monca.augustus.g25975.t1/1-4638	0.134556	0.123454	NACHT domain- and WD repeat-containing 1-like	2	F:GO:0000166; F:GO:0005524	[illegible]
Acrdi.XM_015901349.1/1-846 | Monca.augustus.g61876.t1/1-846	0.137643	0.12345	Krueppel-like factor 16	2	F:GO:0003676; F:GO:0046872	[illegible]
Acrdi.XM_015909373.1/1-1395 | Monca.augustus.g13081.t1/1-1191	0.175139	0.123447	spinster homolog 1-like	1	C:GO:0016020	[illegible]
Acrdi.XM_015913770.1/1-1095 | Monca.augustus.g45555.t1/1-1296	0.120503	0.123424	PREDICTED: uncharacterized protein LOC107347795			
Acrdi.XM_015923829.1/1-597 | Monca.augustus.g33432.t1/1-1842	0.156571	0.123412	zinc finger 883	2	F:GO:0003676; F:GO:0046872	[illegible]
Acrdi.XM_015915365.1/1-1770 | Monca.augustus.g30170.t1/1-3549	0.107637	0.123403	Elongation factor Tu GTP-binding domain-containing 1	5	[illegible]	[illegible]
Acrdi.XM_015919563.1/1-3513 | Monca.augustus.g52100.t1/1-3510	0.15258	0.123392	BAI1-associated 3-like			
Acrdi.XM_015909178.1/1-1158 | Monca.augustus.g6502.t1/1-1026	0.0980943	0.123382	45 kDa calcium-binding	2	F:GO:0005509; P:GO:0002040	[illegible]
Acrdi.XM_015921796.1/1-1749 | Monca.augustus.g15676.t1/1-1746	0.212878	0.123381	RING finger 145-like	2	P:GO:0006511; P:GO:0016567	[illegible]
Acrdi.XM_015899864.1/1-726 | Monca.augustus.g48636.t1/1-726	0.127455	0.123348	CD151 antigen-like	3	[illegible]	[illegible]
Acrdi.XM_015915939.1/1-765 | Monca.augustus.g24372.t1/1-762	0.17961	0.123323	transcription factor HES-1	5	[illegible]	[illegible]
Acrdi.XM_015898503.1/1-645 | Monca.augustus.g3316.t1/1-738	0.151139	0.12332	40S ribosomal S6	15	[illegible]	[illegible]
Acrdi.XM_015902855.1/1-2490 | Monca.augustus.g27501.t1/1-2202	0.118116	0.123316	dipeptidyl peptidase 3	4	[illegible]	[illegible]
Acrdi.XM_015892208.1/1-402 | Monca.adi2mcaRNA3258_R0/1-417	0.159156	0.123269	40S ribosomal S11	3	[illegible]	[illegible]
Acrdi.XM_015905464.1/1-2268 | Monca.adi2mcaRNA15145_R1/1-2259	0.118795	0.123265	Helicase ARIP4			
Acrdi.XM_015919888.1/1-531 | Monca.augustus.g33619.t1/1-462	0.321642	0.123264	superoxide dismutase [Cu-Zn]	57	[illegible]	[illegible]
Acrdi.XM_015917281.1/1-2283 | Monca.augustus.g40305.t1/1-1257	0.461425	0.123263	receptor-interacting serine threonine- kinase 1-like isoform X3			
Acrdi.XM_015925242.1/1-834 | Monca.augustus.g56948.t1/1-2847	0.137738	0.123252	run domain Beclin-1 interacting and cysteine-rich containing -like			
Acrdi.XM_015921129.1/1-5214 | Monca.augustus.g36734.t1/1-5172	0.173454	0.123248	RNA-directed RNA	7	[illegible]	[illegible]
Acrdi.XM_015906670.1/1-546 | Monca.augustus.g15404.t1/1-546	0.131989	0.123234	tetratricopeptide repeat 36			
Acrdi.XM_015912419.1/1-978 | Monca.augustus.g40687.t1/1-990	0.149117	0.123233	Multiple inositol polyphosphate phosphatase 1	5	[illegible]	[illegible]
Acrdi.XM_015915090.1/1-1233 | Monca.augustus.g27920.t1/1-1173	0.0889237	0.12321	interferon-inducible GTPase 5-like			
Acrdi.XM_015901934.1/1-1068 | Monca.augustus.g4665.t1/1-1068	0.171065	0.123189	renalase isoform X2	2	F:GO:0016491; P:GO:0055114	[illegible]
Acrdi.XM_015925300.1/1-801 | Monca.augustus.g54927.t1/1-1158	0.125868	0.123188	Methylosome 50	1	C:GO:0035097	[illegible]
Acrdi.XM_015905709.1/1-846 | Monca.augustus.g1922.t1/1-837	0.195971	0.123182	crk	1	P:GO:0007520	[illegible]
Acrdi.XM_015916738.1/1-2037 | Monca.augustus.g62771.t1/1-2106	0.173706	0.123109	double-stranded RNA-specific editase 1 isoform X3	15	[illegible]	[illegible]
Acrdi.XM_015913668.1/1-441 | Monca.augustus.g30492.t1/1-459	0.210073	0.123095	WSC domain-containing 2-like	2	P:GO:0006508; F:GO:0004197	[illegible]
Acrdi.XM_015911919.1/1-4128 | Monca.adi2mcaRNA22212_R0/1-3609	0.10335	0.123018	DNA polymerase delta catalytic subunit	10	[illegible]	[illegible]
Acrdi.XM_015910254.1/1-1554 | Monca.augustus.g26670.t1/1-2502	0.0936706	0.123013	THAP domain-containing 2			
Acrdi.XM_015924396.1/1-390 | Monca.augustus.g45400.t1/1-390	0.175572	0.12301	PREDICTED: uncharacterized protein LOC107357768			
Acrdi.XM_015917857.1/1-1251 | Monca.augustus.g54899.t1/1-1251	0.115482	0.123004	Abhydrolase domain-containing 2	2	P:GO:0009611; P:GO:0030336	[illegible]
Acrdi.XM_015913005.1/1-498 | Monca.augustus.g56203.t1/1-1098	0.179384	0.122993	guanine nucleotide-binding G(t) subunit alpha-3	7	[illegible]	[illegible]
Acrdi.XM_015907350.1/1-1023 | Monca.augustus.g5328.t1/1-1008	0.143939	0.12298	trans-1,2-dihydrobenzene-1,2-diol dehydrogenase-like	2	F:GO:0016491; P:GO:0055114	[illegible]
Acrdi.XM_015900431.1/1-1041 | Monca.adi2mcaRNA9616_R2/1-1077	0.393926	0.12297	Transposon Ty3-G Gag-Pol poly			
Acrdi.XM_015908217.1/1-1995 | Monca.augustus.g44730.t1/1-3009	0.12551	0.122964	ubiquitin- ligase	4	[illegible]	[illegible]
Acrdi.XM_015918761.1/1-2982 | Monca.augustus.g61933.t1/1-1860	0.145498	0.122954	IQ domain-containing H-like isoform X1			
Acrdi.XM_015900380.1/1-3009 | Monca.augustus.g54212.t1/1-3201	0.148574	0.12293	Serine threonine- kinase ULK2			
Acrdi.XM_015916061.1/1-720 | Monca.augustus.g65539.t1/1-720	0.129098	0.122919	ras-related Rab-11B-like	2	C:GO:0055037; P:GO:0006887	[illegible]
Acrdi.XM_015907098.1/1-540 | Monca.augustus.g59033.t1/1-516	0.214781	0.122908	adenylate cyclase -like	1	C:GO:0016020	[illegible]
Acrdi.XM_015924859.1/1-642 | Monca.augustus.g37690.t1/1-645	0.119415	0.122895	Receptor-binding cancer antigen expressed on o cells			
Acrdi.XM_015899891.1/1-816 | Monca.augustus.g41221.t1/1-11184	0.0497161	0.122885	Centromere F			
Acrdi.XM_015915660.1/1-714 | Monca.augustus.g13474.t1/1-843	0.133142	0.122851	collagen alpha-1(XII) chain-like			
Acrdi.XM_015903649.1/1-1068 | Monca.augustus.g8095.t1/1-3387	0.135126	0.12283	Sterile alpha motif domain-containing 5			
Acrdi.XM_015901638.1/1-4986 | Monca.augustus.g1324.t1/1-5025	0.140842	0.122822	Eukaryotic translation initiation factor 2-alpha kinase 4			
Acrdi.XM_015904447.1/1-1677 | Monca.augustus.g27197.t1/1-2247	0.171145	0.122807	DNA-binding D-ETS-4-like isoform X3	5	[illegible]	[illegible]
Acrdi.XM_015905925.1/1-3528 | Monca.augustus.g58502.t1/1-3498	0.165293	0.122788	zinc finger GLI2-like			
Acrdi.XM_015895807.1/1-4572 | Monca.augustus.g59276.t1/1-4542	0.131604	0.122784	polycystic kidney disease 1-like 2			
Acrdi.XM_015910502.1/1-837 | Monca.augustus.g25425.t1/1-903	0.118895	0.122783	tRNA methyltransferase 10 homolog A	6	[illegible]	[illegible]
Acrdi.XM_015893957.1/1-1776 | Monca.adi2mcaRNA40105_R0/1-1749	0.0986368	0.122776	Afadin- and alpha-actinin-binding A	3	[illegible]	[illegible]
Acrdi.XM_015924847.1/1-1107 | Monca.augustus.g30967.t1/1-1959	0.146313	0.122764	Cytochrome b-245 heavy chain	4	[illegible]	[illegible]
Acrdi.XM_015897152.1/1-573 | Monca.adi2mcaRNA6094_R0/1-609	0.130105	0.122723	transmembrane prolyl 4-hydroxylase-like	4	[illegible]	[illegible]
Acrdi.XM_015916636.1/1-1944 | Monca.augustus.g1139.t1/1-2499	0.13742	0.122705	3 ,5 -cyclic phosphodiesterase pde-5	1	F:GO:0008081	[illegible]
Acrdi.XM_015906360.1/1-1275 | Monca.augustus.g45307.t1/1-1236	0.177995	0.122587	Transmembrane 43	1	C:GO:0016020	[illegible]
Acrdi.XM_015921676.1/1-3885 | Monca.augustus.g43098.t1/1-4062	0.155719	0.122586	host cell factor 2-like			
Acrdi.XM_015892071.1/1-774 | Monca.augustus.g13160.t1/1-798	0.0786551	0.122567	carbohydrate sulfotransferase 11-like	4	[illegible]	[illegible]
Acrdi.XM_015913872.1/1-540 | Monca.augustus.g5035.t1/1-576	0.109499	0.122553	Retrovirus-related Pol poly from transposon 412			
Acrdi.XM_015919238.1/1-5340 | Monca.augustus.g64135.t1/1-5610	0.138122	0.122536	polybromo-1 isoform X1	1	F:GO:0003682	[illegible]
Acrdi.XM_015907219.1/1-2370 | Monca.augustus.g7065.t1/1-1494	0.214086	0.122534	Dihydrolipoyllysine-residue acetyltransferase component of pyruvate d	12	[illegible]	[illegible]
Acrdi.XM_015902536.1/1-1662 | Monca.augustus.g39928.t1/1-2538	0.151697	0.122511	FERM domain-containing 4A	4	[illegible]	[illegible]
Acrdi.XM_015899065.1/1-2295 | Monca.adi2mcaRNA8124_R1/1-1821	0.183689	0.122474	glutamyl aminopeptidase	5	[illegible]	[illegible]
Acrdi.XM_015900392.1/1-1254 | Monca.augustus.g46624.t1/1-1779	0.154621	0.122461	ATP-dependent RNA helicase DDX55	5	[illegible]	[illegible]
Acrdi.XM_015912220.1/1-1863 | Monca.augustus.g22431.t1/1-1605	0.150862	0.122455	diacyglycerol O-acyltransferase MT1468	4	[illegible]	[illegible]
Acrdi.XM_015898747.1/1-792 | Monca.augustus.g2059.t1/1-792	0.143659	0.122451	E3 ubiquitin- ligase MARCH8	2	F:GO:0046872; C:GO:0016020	[illegible]
Acrdi.XM_015915519.1/1-1245 | Monca.augustus.g30911.t1/1-1671	0.203249	0.122396	PREDICTED: uncharacterized protein LOC107349372 isoform X2	1	F:GO:0070492	[illegible]
Acrdi.XM_015915330.1/1-1182 | Monca.adi2mcaRNA26002_R0/1-1158	0.102458	0.122394	monocarboxylate transporter 10-like	5	[illegible]	[illegible]
Acrdi.XM_015898170.1/1-1494 | Monca.augustus.g29262.t1/1-1512	0.143306	0.122378	Innexin unc-9	1	F:GO:0003824	[illegible]
Acrdi.XM_015916366.1/1-1029 | Monca.augustus.g35027.t1/1-954	0.153698	0.122365	ROK family	2	C:GO:0016020; F:GO:0016740	[illegible]
Acrdi.XM_015892354.1/1-717 | Monca.augustus.g69815.t1/1-708	0.0931083	0.122351	PREDICTED: uncharacterized protein LOC107327610	4	[illegible]	[illegible]
Acrdi.XM_015891712.1/1-339 | Monca.augustus.g43734.t1/1-3096	0.129678	0.122345	kinesin KIF13B isoform X1	1	F:GO:0005509	[illegible]
Acrdi.XM_015904218.1/1-2268 | Monca.augustus.g11835.t1/1-2256	0.162458	0.122342	potassium voltage-gated channel Shaw-like	5	[illegible]	[illegible]
Acrdi.XM_015919197.1/1-1023 | Monca.augustus.g54952.t1/1-996	0.184804	0.122331	beta-1,3-galactosyltransferase 5-like	3	[illegible]	[illegible]
Acrdi.XM_015898460.1/1-3414 | Monca.augustus.g33998.t1/1-4896	0.15607	0.122325	unc-13 homolog A-like	5	[illegible]	[illegible]
Acrdi.XM_015915687.1/1-3141 | Monca.augustus.g27933.t1/1-3246	0.135314	0.122277	allene oxide synthase-lipoxygenase -like	3	[illegible]	[illegible]
Acrdi.XM_015906825.1/1-672 | Monca.augustus.g29126.t1/1-663	0.140979	0.122258	Forkhead box P1-B			
Acrdi.XM_015896160.1/1-402 | Monca.augustus.g55042.t1/1-402	0.163162	0.122252	bolA DDB_G0274169			
Acrdi.XM_015893168.1/1-1062 | Monca.adi2mcaRNA39221_R0/1-906	0.241076	0.122242	Transposon Ty3-I Gag-Pol poly	5	[illegible]	[illegible]
Acrdi.XM_015919535.1/1-1209 | Monca.adi2mcaRNA30735_R0/1-1602	0.100413	0.122242	PREDICTED: uncharacterized protein LOC107353213			
Acrdi.XM_015911714.1/1-2067 | Monca.augustus.g24264.t1/1-2061	0.0891486	0.122222	mucosa-associated lymphoid tissue lymphoma translocation 1-like	2	P:GO:0006508; F:GO:0004197	[illegible]
Acrdi.XM_015898461.1/1-2214 | Monca.adi2mcaRNA7545_R0/1-2169	0.167417	0.12222	centrosomal of 120 kDa isoform X1	1	P:GO:0044699	[illegible]
Acrdi.XM_015902669.1/1-981 | Monca.augustus.g54280.t1/1-999	0.113045	0.122204	Tubulin-specific chaperone C	2	P:GO:0016043; P:GO:0006457	[illegible]
Acrdi.XM_015906742.1/1-708 | Monca.augustus.g3896.t1/1-930	0.221629	0.12216	serine threonine tyrosine-interacting 1	2	F:GO:0016791; P:GO:0016311	[illegible]
Acrdi.XM_015896115.1/1-1566 | Monca.augustus.g47634.t1/1-1632	0.2047	0.122148	Collagen alpha-1(XII) chain			
Acrdi.XM_015891859.1/1-1128 | Monca.adi2mcaRNA10446_R5/1-1422	0.141216	0.122134	arylsulfatase B-like	3	[illegible]	[illegible]
Acrdi.XM_015894286.1/1-1311 | Monca.adi2mcaRNA40530_R0/1-1287	0.485588	0.122095	PREDICTED: uncharacterized protein LOC107329608			
Acrdi.XM_015916795.1/1-1989 | Monca.augustus.g46544.t1/1-2337	0.142224	0.122062	Leucine-rich repeat-containing			
Acrdi.XM_015904494.1/1-705 | Monca.augustus.g33599.t1/1-693	0.157745	0.122051	chloride intracellular channel 4	14	[illegible]	[illegible]
Acrdi.XM_015901769.1/1-795 | Monca.augustus.g58377.t1/1-1086	0.0863184	0.122001	Spermatogenesis-associated 17			
Acrdi.XM_015911590.1/1-1752 | Monca.augustus.g24292.t1/1-1764	0.170656	0.121998	F-box only 30	5	[illegible]	[illegible]
Acrdi.XM_015925231.1/1-1122 | Monca.augustus.g22740.t1/1-1290	0.0712505	0.121972	probable imidazolonepropionase	2	F:GO:0016810; P:GO:0006547	[illegible]
Acrdi.XM_015905296.1/1-2436 | Monca.augustus.g23133.t1/1-2562	0.184885	0.12197	F-BAR and double SH3 domains 2			
Acrdi.XM_015922359.1/1-660 | Monca.augustus.g66200.t1/1-1233	0.110446	0.121942	PREDICTED: trichohyalin-like			
Acrdi.XM_015900737.1/1-756 | Monca.augustus.g34906.t1/1-858	0.160767	0.121919	epidermal retinol dehydrogenase 2-like	4	[illegible]	[illegible]
Acrdi.XM_015916183.1/1-25758 | Monca.adi2mcaRNA26989_R0/1-2532[illegible]	0.135564	0.121907	MAM and LDL-receptor class A domain-containing 2-like	1	C:GO:0016020	[illegible]
Acrdi.XM_015911440.1/1-2124 | Monca.adi2mcaRNA21700_R0/1-1887	0.153826	0.121901	arginine N-methyltransferase 7	2	F:GO:0016274; P:GO:0018216	[illegible]
Acrdi.XM_015901895.1/1-1302 | Monca.augustus.g17430.t1/1-1368	0.18957	0.121873	solute carrier family 22 member 18-like	1	C:GO:0016020	[illegible]
Acrdi.XM_015924066.1/1-1599 | Monca.augustus.g32998.t1/1-1599	0.120343	0.121845	dimethylaniline monooxygenase [N-oxide-forming] 5-like	8	[illegible]	[illegible]
Acrdi.XM_015906638.1/1-390 | Monca.adi2mcaRNA16418_R0/1-429	0.434577	0.121838	toll-like receptor 6	2	F:GO:0003676; C:GO:0044424	[illegible]
Acrdi.XM_015902419.1/1-903 | Monca.augustus.g58387.t1/1-819	0.640092	0.121787	PREDICTED: uncharacterized protein LOC107337292			
Acrdi.XM_015910741.1/1-1485 | Monca.augustus.g48010.t1/1-1275	0.121127	0.12178	elongation factor 1-gamma	13	[illegible]	[illegible]
Acrdi.XM_015914780.1/1-597 | Monca.augustus.g291.t1/1-594	0.14256	0.121764	high mobility group B2-like	6	[illegible]	[illegible]
Acrdi.XM_015922160.1/1-300 | Monca.augustus.g9769.t1/1-300	0.138349	0.121649	cystatin-B-like	3	[illegible]	[illegible]
Acrdi.XM_015902561.1/1-1497 | Monca.augustus.g39935.t1/1-1494	0.127121	0.121615	Death ligand signal enhancer			
Acrdi.XM_015899603.1/1-522 | Monca.augustus.g7523.t1/1-951	0.0943319	0.121593	Mitochondrial folate transporter carrier	4	[illegible]	[illegible]
Acrdi.XM_015921088.1/1-1023 | Monca.adi2mcaRNA32382_R0/1-933	0.173958	0.121563	28S ribosomal mitochondrial	5	[illegible]	[illegible]
Acrdi.XM_015898507.1/1-1563 | Monca.augustus.g3328.t1/1-1596	0.147671	0.121538	DNA-directed RNA polymerase III subunit RPC3	4	[illegible]	[illegible]
Acrdi.XM_015901767.1/1-2034 | Monca.augustus.g61686.t1/1-2049	0.15026	0.121519	major facilitator superfamily domain-containing 6 isoform X2	2	C:GO:0016020; C:GO:0016021	[illegible]
Acrdi.XM_015897216.1/1-2385 | Monca.augustus.g54256.t1/1-2364	0.197652	0.121505	Transmembrane and TPR repeat-containing 4	2	C:GO:0016020; C:GO:0016021	[illegible]
Acrdi.XM_015912877.1/1-1110 | Monca.augustus.g3667.t1/1-1092	0.194652	0.121396	Transcription factor Sox-7	48	[illegible]	[illegible]
Acrdi.XM_015892555.1/1-816 | Monca.augustus.g44011.t1/1-3396	0.085599	0.121392	transient receptor potential cation channel subfamily A member 1-like	7	[illegible]	[illegible]
Acrdi.XM_015899854.1/1-1086 | Monca.augustus.g52547.t1/1-1239	0.13677	0.121389	adenosine receptor A3-like	3	[illegible]	[illegible]
Acrdi.XM_015904535.1/1-1107 | Monca.adi2mcaRNA14051_R0/1-1095	0.0928073	0.121387	dnaJ homolog subfamily C member 28	6	[illegible]	[illegible]
Acrdi.XM_015916361.1/1-876 | Monca.augustus.g40955.t1/1-1047	0.182105	0.121335	Cathepsin B	5	[illegible]	[illegible]
Acrdi.XM_015904895.1/1-1089 | Monca.augustus.g8819.t1/1-1623	0.144935	0.121326	Dolichol kinase	2	C:GO:0016020; C:GO:0016021	[illegible]
Acrdi.XM_015895459.1/1-330 | Monca.augustus.g28774.t1/1-540	0.103266	0.121322	trafficking particle complex subunit 3	2	P:GO:0006888; C:GO:0005737	[illegible]
Acrdi.XM_015892883.1/1-666 | Monca.augustus.g71527.t1/1-657	0.082465	0.121317	transmembrane 65-like	1	C:GO:0016021	[illegible]
Acrdi.XM_015925335.1/1-603 | Monca.augustus.g66556.t1/1-606	0.123553	0.121296	Homeobox Hox-D3	3	[illegible]	[illegible]
Acrdi.XM_015898236.1/1-1749 | Monca.augustus.g44529.t1/1-5529	0.173266	0.121268	PREDICTED: uncharacterized protein KIAA0556-like			
Acrdi.XM_015919756.1/1-534 | Monca.augustus.g67163.t1/1-546	0.109149	0.121239	atonal homolog 8-like	4	[illegible]	[illegible]
Acrdi.XM_015899243.1/1-972 | Monca.augustus.g1493.t1/1-1602	0.175394	0.121185	Histone H5			
Acrdi.XM_015910580.1/1-621 | Monca.augustus.g70624.t1/1-624	0.147493	0.121173	signal peptidase complex subunit 2	5	[illegible]	[illegible]
Acrdi.XM_015923408.1/1-693 | Monca.augustus.g22242.t1/1-789	0.524207	0.121138	PREDICTED: uncharacterized protein LOC107356782			
Acrdi.XM_015900864.1/1-1503 | Monca.adi2mcaRNA10085_R0/1-1491	0.200219	0.121125	Calcium-dependent kinase 26	1	F:GO:0046872	[illegible]
Acrdi.XM_015903496.1/1-4551 | Monca.augustus.g34538.t1/1-5019	0.157355	0.121125	Multiple PDZ domain	1	C:GO:0044464	[illegible]
Acrdi.XM_015895474.1/1-555 | Monca.augustus.g14887.t1/1-1206	0.151989	0.121122	Histone acetyltransferase type B catalytic subunit	4	[illegible]	[illegible]
Acrdi.XM_015891737.1/1-831 | Monca.augustus.g49604.t1/1-798	0.132971	0.121121	acyl- -binding domain-containing 6	3	[illegible]	[illegible]
Acrdi.XM_015924192.1/1-777 | Monca.augustus.g477.t1/1-1008	0.163597	0.121071	dolichyl-phosphate beta-glucosyltransferase isoform X2	6	[illegible]	[illegible]
Acrdi.XM_015919477.1/1-4386 | Monca.augustus.g61038.t1/1-4518	0.10869	0.121064	inositol hexakisphosphate and diphosphoinositol-pentakisphosphate ki	2	P:GO:0016311; F:GO:0003993	[illegible]
Acrdi.XM_015898380.1/1-1413 | Monca.adi2mcaRNA7433_R0/1-1131	0.335835	0.121049	PREDICTED: uncharacterized protein LOC107333549			
Acrdi.XM_015909725.1/1-2712 | Monca.augustus.g3314.t1/1-2712	0.169502	0.121037	3-hydroxy-3-methylglutaryl-Coenzyme A reductase	4	[illegible]	[illegible]
Acrdi.XM_015910058.1/1-696 | Monca.augustus.g36890.t1/1-810	0.1579	0.121012	N(G),N(G)-dimethylarginine dimethylaminohydrolase 1	4	[illegible]	[illegible]
Acrdi.XM_015904304.1/1-9492 | Monca.augustus.g32334.t1/1-9444	0.147085	0.121009	golgin subfamily B member 1-like isoform X1			
Acrdi.XM_015908229.1/1-816 | Monca.adi2mcaRNA18205_R0/1-759	0.29101	0.121005	X-box-binding 1	74	[illegible]	[illegible]
Acrdi.XM_015904807.1/1-1785 | Monca.augustus.g42620.t1/1-2076	0.147627	0.120999	SH2B adapter 1-like	6	[illegible]	[illegible]
Acrdi.XM_015909080.1/1-1011 | Monca.augustus.g67748.t1/1-1014	0.150475	0.120956	proline-rich transmembrane 4-like	2	C:GO:0016020; C:GO:0016021	[illegible]
Acrdi.XM_015895779.1/1-1422 | Monca.augustus.g47028.t1/1-1368	0.135516	0.120934	F-box LRR-repeat	2	F:GO:0005515; P:GO:0044267	[illegible]
Acrdi.XM_015896342.1/1-660 | Monca.adi2mcaRNA5165_R0/1-456	0.129489	0.120934	cereblon homolog	2	C:GO:0016020; C:GO:0016021	[illegible]
Acrdi.XM_015904799.1/1-2751 | Monca.augustus.g12733.t1/1-1665	0.112264	0.12093	kinase suppressor of Ras 2 isoform X2	4	[illegible]	[illegible]
Acrdi.XM_015919337.1/1-3150 | Monca.augustus.g5828.t1/1-3174	0.151822	0.120926	Serine threonine- phosphatase 4 regulatory subunit 1	3	[illegible]	[illegible]
Acrdi.XM_015913614.1/1-873 | Monca.augustus.g48689.t1/1-1113	0.236909	0.120916	Pinin			
Acrdi.XM_015925299.1/1-1206 | Monca.augustus.g54929.t1/1-1215	0.133324	0.120896	lipase member K-like	4	[illegible]	[illegible]
Acrdi.XM_015918115.1/1-1008 | Monca.augustus.g38584.t1/1-1482	0.160647	0.120891	tRNA pseudouridine(38 39)	2	P:GO:0009451; F:GO:0016853	[illegible]
Acrdi.XM_015903116.1/1-4641 | Monca.augustus.g52470.t1/1-4557	0.144345	0.120837	A-kinase anchor 13-like isoform X1	3	[illegible]	[illegible]
Acrdi.XM_015920149.1/1-1059 | Monca.augustus.g27083.t1/1-1056	0.173335	0.120832	PREDICTED: uncharacterized protein LOC107353762			
Acrdi.XM_015914230.1/1-1068 | Monca.augustus.g24191.t1/1-1365	0.109585	0.120769	ruvB-like 1	24	[illegible]	[illegible]
Acrdi.XM_015914258.1/1-1005 | Monca.augustus.g57446.t1/1-1566	0.112335	0.120755	Lysosomal Pro-X carboxypeptidase	7	[illegible]	[illegible]
Acrdi.XM_015902239.1/1-951 | Monca.augustus.g71159.t1/1-1674	0.147784	0.120752	digestive cysteine ase 1-like	4	[illegible]	[illegible]
Acrdi.XM_015919585.1/1-552 | Monca.augustus.g13434.t1/1-723	0.223792	0.120717	PREDICTED: uncharacterized protein LOC107353258	2	P:GO:0006281; F:GO:0003824	[illegible]
Acrdi.XM_015917894.1/1-741 | Monca.augustus.g18123.t1/1-738	0.162888	0.120709	15-hydroxyprostaglandin dehydrogenase [NAD(+)]	2	F:GO:0016491; P:GO:0055114	[illegible]
Acrdi.XM_015905579.1/1-513 | Monca.augustus.g24099.t1/1-516	0.155152	0.120705	PREDICTED: uncharacterized protein LOC107340225			
Acrdi.XM_015921616.1/1-828 | Monca.augustus.g67242.t1/1-819	0.14247	0.120661	PREDICTED: uncharacterized protein LOC107355100			
Acrdi.XM_015910375.1/1-4230 | Monca.augustus.g47624.t1/1-7881	0.174509	0.120657	protocadherin Fat 4-like	12	[illegible]	[illegible]
Acrdi.XM_015917902.1/1-570 | Monca.augustus.g39679.t1/1-1182	0.141883	0.120644	WD repeat-containing 89			
Acrdi.XM_015912739.1/1-1617 | Monca.augustus.g2529.t1/1-1638	0.104953	0.120637	Prolyl 4-hydroxylase subunit alpha-1	1	F:GO:0016491	[illegible]
Acrdi.XM_015921376.1/1-801 | Monca.augustus.g4651.t1/1-717	0.132114	0.120609	RNA-binding pno1	2	F:GO:0003676; C:GO:0005730	[illegible]
Acrdi.XM_015915561.1/1-1665 | Monca.augustus.g25115.t1/1-4722	0.162016	0.120608	centrosome-associated CEP250-like isoform X1			
Acrdi.XM_015920281.1/1-1743 | Monca.adi2mcaRNA31534_R2/1-1404	0.423088	0.120602	SURP and G-patch domain-containing 1	1	F:GO:0046872	[illegible]
Acrdi.XM_015899896.1/1-1305 | Monca.augustus.g10908.t1/1-1650	0.13759	0.120564	vacuolar fusion MON1 homolog A-like	2	P:GO:0016192; C:GO:0012505	[illegible]
Acrdi.XM_015892276.1/1-4980 | Monca.augustus.g3500.t1/1-5757	0.116994	0.120556	sperm flagellar 2 isoform X3			
Acrdi.XM_015915803.1/1-537 | Monca.adi2mcaRNA26509_R4/1-486	0.135825	0.120527	PREDICTED: uncharacterized protein LOC107349627 isoform X1			
Acrdi.XM_015925153.1/1-588 | Monca.augustus.g37269.t1/1-1623	0.124901	0.120525	GRAM domain-containing 4-like	2	C:GO:0016020; C:GO:0016021	[illegible]
Acrdi.XM_015898833.1/1-1752 | Monca.adi2mcaRNA7895_R7/1-1488	0.771939	0.12051	RNA-directed DNA polymerase from mobile element jockey-like			
Acrdi.XM_015904901.1/1-1743 | Monca.augustus.g17420.t1/1-1746	0.144534	0.12049	Serine threonine- kinase Nek11	3	[illegible]	[illegible]
Acrdi.XM_015904702.1/1-588 | Monca.augustus.g18818.t1/1-588	0.144349	0.120487	E3 ubiquitin- ligase MARCH3	5	[illegible]	[illegible]
Acrdi.XM_015893807.1/1-909 | Monca.adi2mcaRNA39954_R3/1-1059	0.101144	0.120484	ATP-dependent DNA helicase PIF1			
Acrdi.XM_015897050.1/1-795 | Monca.augustus.g55698.t1/1-837	0.143615	0.120483	Tetraspanin-33	4	[illegible]	[illegible]
Acrdi.XM_015916378.1/1-1548 | Monca.augustus.g40954.t1/1-1551	0.0964495	0.120467	Serine threonine- kinase RIO3	3	[illegible]	[illegible]
Acrdi.XM_015892238.1/1-873 | Monca.augustus.g7556.t1/1-852	0.159368	0.120447	UDP-N-acetylglucosamine UDP-glucose GDP-mannose transporter-like	1	F:GO:0015165	[illegible]
Acrdi.XM_015919965.1/1-1413 | Monca.augustus.g47222.t1/1-1401	0.117549	0.120427	Pre-mRNA-splicing factor RBM22	6	[illegible]	[illegible]
Acrdi.XM_015915299.1/1-1482 | Monca.augustus.g25278.t1/1-1116	0.136672	0.120424	carbohydrate-binding family 9	3	[illegible]	[illegible]
Acrdi.XM_015894157.1/1-585 | Monca.adi2mcaRNA40386_R5/1-564	0.661174	0.120403	zinc finger MYM-type 1-like			
Acrdi.XM_015895413.1/1-5586 | Monca.adi2mcaRNA4287_R0/1-5484	0.139213	0.120399	A-kinase anchor 9			
Acrdi.XM_015906440.1/1-2766 | Monca.augustus.g51450.t1/1-2790	0.111459	0.120392	Anion exchange 3	5	[illegible]	[illegible]
Acrdi.XM_015897885.1/1-945 | Monca.augustus.g3104.t1/1-1665	0.124566	0.120368	glutamic acid-rich isoform X2	1	F:GO:0005509	[illegible]
Acrdi.XM_015909511.1/1-1116 | Monca.adi2mcaRNA19615_R0/1-876	0.243516	0.12035	histamine N-methyltransferase-like	2	F:GO:0046539; P:GO:0032259	[illegible]
Acrdi.XM_015898157.1/1-1074 | Monca.augustus.g29273.t1/1-1074	0.151172	0.12034	Peroxisome assembly 12	6	[illegible]	[illegible]
Acrdi.XM_015918723.1/1-3768 | Monca.augustus.g53969.t1/1-5481	0.129943	0.120329	voltage-dependent N-type calcium channel subunit alpha-1B-			
Acrdi.XM_015913380.1/1-1335 | Monca.augustus.g15043.t1/1-2649	0.107041	0.120326	alpha-1,6-mannosylglyco 6-beta-N-acetylglucosaminyltransferase	4	[illegible]	[illegible]
Acrdi.XM_015896633.1/1-2652 | Monca.augustus.g37028.t1/1-3690	0.125849	0.12032	Serine threonine- kinase Nek10	5	[illegible]	[illegible]
Acrdi.XM_015893462.1/1-774 | Monca.adi2mcaRNA4030_R0/1-780	0.161023	0.12031	activating signal cointegrator 1 complex subunit 2-like			
Acrdi.XM_015909864.1/1-411 | Monca.augustus.g30581.t1/1-408	0.194413	0.120304	60S ribosomal L14	4	[illegible]	[illegible]
Acrdi.XM_015923515.1/1-2193 | Monca.augustus.g47468.t1/1-2325	0.147777	0.120304	acid trehalase 1	2	C:GO:0005618; F:GO:0016787	[illegible]
Acrdi.XM_015891983.1/1-858 | Monca.augustus.g66331.t1/1-2181	0.132867	0.120275	beta-1,4-N-acetylgalactosaminyltransferase 3-like	3	[illegible]	[illegible]
Acrdi.XM_015921544.1/1-1854 | Monca.augustus.g23006.t1/1-3777	0.163105	0.12026	TPA_inf: venus kinase receptor	2	F:GO:0016740; P:GO:0006796	[illegible]
Acrdi.XM_015893372.1/1-1056 | Monca.augustus.g41427.t1/1-2709	0.184829	0.120254	trifunctional nucleotide phosphoesterase isoform X2	1	F:GO:0005488	[illegible]
Acrdi.XM_015901389.1/1-900 | Monca.augustus.g70853.t1/1-903	0.269818	0.120242	sprouty homolog 2	3	[illegible]	[illegible]
Acrdi.XM_015900749.1/1-4359 | Monca.augustus.g49528.t1/1-4359	0.165695	0.120235	PREDICTED: uncharacterized protein LOC107335716			
Acrdi.XM_015908972.1/1-1923 | Monca.augustus.g11911.t1/1-2343	0.14373	0.120229	High-affinity choline transport	5	[illegible]	[illegible]
Acrdi.XM_015920589.1/1-924 | Monca.augustus.g47221.t1/1-1467	0.161369	0.120208	calcium uptake mitochondrial-like isoform X3	1	F:GO:0005509	[illegible]
Acrdi.XM_015898550.1/1-2652 | Monca.augustus.g54251.t1/1-2670	0.151619	0.120186	WD repeat-containing 44			
Acrdi.XM_015903173.1/1-1398 | Monca.augustus.g52467.t1/1-1398	0.169608	0.120179	ankyrin repeat domain-containing 34A-like			
Acrdi.XM_015896521.1/1-1953 | Monca.augustus.g1587.t1/1-2121	0.170226	0.120177	Transmembrane and coiled-coil domain-containing 3	7	[illegible]	[illegible]
Acrdi.XM_015906011.1/1-891 | Monca.augustus.g41407.t1/1-891	0.136467	0.120151	PREDICTED: uncharacterized protein LOC107340645	2	C:GO:0016020; C:GO:0016021	[illegible]
Acrdi.XM_015914972.1/1-429 | Monca.augustus.g42428.t1/1-441	0.173096	0.120141	transcription factor 15-like	1	F:GO:0046983	[illegible]
Acrdi.XM_015895630.1/1-1167 | Monca.augustus.g22275.t1/1-1173	0.12319	0.120139	glycosyltransferase family 8	1	F:GO:0016740	[illegible]
Acrdi.XM_015921532.1/1-354 | Monca.augustus.g22995.t1/1-354	0.275897	0.120105	chromosome transmission fidelity 8 homolog	3	[illegible]	[illegible]
Acrdi.XM_015899286.1/1-1035 | Monca.augustus.g64432.t1/1-318	0.125736	0.120092	component of the counting factor complex	6	[illegible]	[illegible]
Acrdi.XM_015909806.1/1-771 | Monca.augustus.g47048.t1/1-1509	0.132968	0.120089	solute carrier family 22 member 15-like isoform X2	2	F:GO:0022857; C:GO:0016020	[illegible]
Acrdi.XM_015900157.1/1-2055 | Monca.augustus.g10475.t1/1-4773	0.136207	0.120066	receptor-type tyrosine- phosphatase	2	F:GO:0016791; P:GO:0016311	[illegible]
Acrdi.XM_015908748.1/1-1476 | Monca.augustus.g27998.t1/1-1455	0.111514	0.120023	galactose-3-O-sulfotransferase 2-like	4	[illegible]	[illegible]
Acrdi.XM_015905652.1/1-1311 | Monca.augustus.g65079.t1/1-3393	0.129944	0.119984	unc-80 homolog			
Acrdi.XM_015919318.1/1-1299 | Monca.adi2mcaRNA30495_R0/1-1305	0.171211	0.119982	Transmembrane with metallophosphoesterase domain	2	F:GO:0016788; C:GO:0016020	[illegible]
Acrdi.XM_015916771.1/1-1305 | Monca.augustus.g62791.t1/1-1296	0.170836	0.119982	male-specific lethal 1 homolog	3	[illegible]	[illegible]
Acrdi.XM_015891665.1/1-339 | Monca.augustus.g43736.t1/1-1266	0.17087	0.11997	unconventional prefoldin RPB5 interactor-like	6	[illegible]	[illegible]
Acrdi.XM_015903991.1/1-711 | Monca.augustus.g32303.t1/1-756	0.184402	0.119955	dopamine receptor 2-like	3	[illegible]	[illegible]
Acrdi.XM_015894193.1/1-303 | Monca.augustus.g53465.t1/1-306	0.122254	0.119946	gamma-secretase subunit PEN-2	5	[illegible]	[illegible]
Acrdi.XM_015914472.1/1-894 | Monca.augustus.g56341.t1/1-900	0.129164	0.119915	Cathepsin Z	4	[illegible]	[illegible]
Acrdi.XM_015904885.1/1-654 | Monca.augustus.g29905.t1/1-666	0.135041	0.119906	tetraspanin-13 isoform X2	1	C:GO:0016021	[illegible]
Acrdi.XM_015901487.1/1-648 | Monca.augustus.g31726.t1/1-681	0.0925432	0.119886	chromobox homolog 1-like isoform X1	1	C:GO:0005634	[illegible]
Acrdi.XM_015907191.1/1-837 | Monca.augustus.g28172.t1/1-942	0.13442	0.119872	Failed axon connections-like			
Acrdi.XM_015913377.1/1-1407 | Monca.augustus.g61325.t1/1-1434	0.172791	0.119869	dipeptidyl peptidase 2	4	[illegible]	[illegible]
Acrdi.XM_015919264.1/1-2706 | Monca.augustus.g65055.t1/1-3135	0.115288	0.119841	La-related 1			
Acrdi.XM_015906932.1/1-2577 | Monca.augustus.g33399.t1/1-4551	0.141245	0.11984	kinesin KIF15 isoform X3	7	[illegible]	[illegible]
Acrdi.XM_015896860.1/1-1287 | Monca.adi2mcaRNA586_R6/1-1080	0.236817	0.119831	PREDICTED: uncharacterized protein LOC107332131			
Acrdi.XM_015900687.1/1-654 | Monca.augustus.g15968.t1/1-831	0.115391	0.119799	Spermidine synthase	3	[illegible]	[illegible]
Acrdi.XM_015898481.1/1-333 | Monca.augustus.g43320.t1/1-1509	0.198465	0.119798	neuroblastoma-amplified sequence-like			
Acrdi.XM_015912714.1/1-2592 | Monca.augustus.g43036.t1/1-2568	0.152231	0.119775	chordin	5	[illegible]	[illegible]
Acrdi.XM_015905868.1/1-609 | Monca.augustus.g32202.t1/1-606	0.0948504	0.119737	cysteine serine-rich nuclear 1-like	4	[illegible]	[illegible]
Acrdi.XM_015892498.1/1-2253 | Monca.adi2mcaRNA38449_R0/1-2259	0.108474	0.119726	CWF19 2	1	F:GO:0003824	[illegible]
Acrdi.XM_015892169.1/1-591 | Monca.adi2mcaRNA38076_R1/1-561	0.689344	0.119725	NFX1-type zinc finger-containing 1-like	4	[illegible]	[illegible]
Acrdi.XM_015905175.1/1-1437 | Monca.augustus.g52841.t1/1-2175	0.163938	0.119714	sperm-tail PG-rich repeat-containing 2-like			
Acrdi.XM_015909819.1/1-1062 | Monca.augustus.g47077.t1/1-1062	0.113089	0.119698	PREDICTED: uncharacterized protein LOC107344183	2	C:GO:0016020; C:GO:0016021	[illegible]
Acrdi.XM_015905192.1/1-1071 | Monca.augustus.g57151.t1/1-1068	0.114375	0.119682	zinc finger AEBP2-like			
Acrdi.XM_015909692.1/1-360 | Monca.augustus.g44684.t1/1-300	0.0977876	0.119679	PREDICTED: uncharacterized protein LOC107344086			
Acrdi.XM_015903588.1/1-1695 | Monca.augustus.g53522.t1/1-1707	0.138607	0.119669	major facilitator superfamily domain-containing 6-like	2	C:GO:0016020; C:GO:0016021	[illegible]
Acrdi.XM_015902979.1/1-756 | Monca.augustus.g65498.t1/1-678	0.140815	0.119662	brain-specific homeobox homolog	4	[illegible]	[illegible]
Acrdi.XM_015898733.1/1-1179 | Monca.augustus.g2551.t1/1-1038	0.144268	0.119631	membrane progestin receptor gamma-A-like	3	[illegible]	[illegible]
Acrdi.XM_015910993.1/1-459 | Monca.augustus.g63041.t1/1-459	0.170669	0.119582	calmodulin 3	3	[illegible]	[illegible]
Acrdi.XM_015906991.1/1-1329 | Monca.adi2mcaRNA16816_R1/1-1311	0.112107	0.11958	Gamma-aminobutyric acid type B receptor subunit 2	8	[illegible]	[illegible]
Acrdi.XM_015911579.1/1-1788 | Monca.adi2mcaRNA21862_R0/1-1650	0.143356	0.119574	ubiquitin carboxyl-terminal hydrolase BAP1 isoform X3	8	[illegible]	[illegible]
Acrdi.XM_015913213.1/1-1005 | Monca.augustus.g32064.t1/1-1014	0.131737	0.11957	Mitochondrial ubiquitin ligase activator of NFKB 1	11	[illegible]	[illegible]
Acrdi.XM_015895884.1/1-1665 | Monca.adi2mcaRNA4700_R0/1-1560	0.1346	0.119562	synaptotagmin-11 isoform X1	8	[illegible]	[illegible]
Acrdi.XM_015910496.1/1-705 | Monca.augustus.g36044.t1/1-702	0.118327	0.119547	alpha-ketoglutarate-dependent dioxygenase alkB homolog 6	2	F:GO:0016491; P:GO:0055114	[illegible]
Acrdi.XM_015905025.1/1-375 | Monca.augustus.g68043.t1/1-2121	0.698796	0.119533	PREDICTED: uncharacterized protein LOC107339727			
Acrdi.XM_015903262.1/1-1206 | Monca.augustus.g51692.t1/1-1209	0.161921	0.119532	Coiled-coil domain-containing 92			
Acrdi.XM_015912688.1/1-711 | Monca.augustus.g43033.t1/1-606	0.135445	0.119515	Mediator of RNA polymerase II transcription subunit 18	6	[illegible]	[illegible]
Acrdi.XM_015911255.1/1-1110 | Monca.augustus.g48130.t1/1-2550	0.139978	0.119467	Nesprin-1			
Acrdi.XM_015903686.1/1-1449 | Monca.augustus.g37318.t1/1-2232	0.123244	0.119436	MAP7 domain-containing 2-like	3	[illegible]	[illegible]
Acrdi.XM_015897541.1/1-1044 | Monca.augustus.g22828.t1/1-1050	0.137322	0.119401	beta-2 adrenergic receptor-like			
Acrdi.XM_015902360.1/1-1038 | Monca.augustus.g57289.t1/1-594	0.11134	0.119367	DNA repair RAD50	5	[illegible]	[illegible]
Acrdi.XM_015907664.1/1-642 | Monca.augustus.g29932.t1/1-642	0.102702	0.119359	Deoxycytidylate deaminase	5	[illegible]	[illegible]
Acrdi.XM_015900414.1/1-909 | Monca.adi2mcaRNA9586_R0/1-906	0.16235	0.119306	zinc finger 429-like	2	F:GO:0003676; F:GO:0046872	[illegible]
Acrdi.XM_015907613.1/1-1269 | Monca.augustus.g12445.t1/1-1578	0.159485	0.119296	glucosylceramidase isoform X1	2	P:GO:0006629; F:GO:0016798	[illegible]
Acrdi.XM_015903754.1/1-555 | Monca.augustus.g32289.t1/1-921	0.131226	0.119285	Mediator of RNA polymerase II transcription subunit 4	4	[illegible]	[illegible]
Acrdi.XM_015915910.1/1-672 | Monca.augustus.g24364.t1/1-678	0.146255	0.119274	transcription factor HES-1	5	[illegible]	[illegible]
Acrdi.XM_015911392.1/1-1071 | Monca.augustus.g69038.t1/1-1548	0.123752	0.119263	phosphoethanolamine N-methyltransferase 3-like isoform X1			
Acrdi.XM_015897449.1/1-1881 | Monca.augustus.g32447.t1/1-2115	0.168724	0.119244	apoptosis-inducing factor mitochondrial-like	6	[illegible]	[illegible]
Acrdi.XM_015917164.1/1-1572 | Monca.augustus.g6783.t1/1-2589	0.117432	0.119238	tyrosine- kinase Fer	7	[illegible]	[illegible]
Acrdi.XM_015915458.1/1-570 | Monca.augustus.g53448.t1/1-531	0.160531	0.119232	F-box only 22			
Acrdi.XM_015892233.1/1-882 | Monca.augustus.g2821.t1/1-885	0.12948	0.119224	BTB POZ domain-containing	1	P:GO:0051260	[illegible]
Acrdi.XM_015912851.1/1-3186 | Monca.adi2mcaRNA23260_R0/1-2955	0.102875	0.119203	probable 3 ,5 -cyclic phosphodiesterase pde-5	2	P:GO:0007165; F:GO:0004114	[illegible]
Acrdi.XM_015903312.1/1-615 | Monca.augustus.g18350.t1/1-612	0.127749	0.119193	UBX domain-containing 1-like			
Acrdi.XM_015907315.1/1-2205 | Monca.augustus.g5325.t1/1-3726	0.127758	0.119166	1-phosphatidylinositol 4,5-bisphosphate phosphodiesterase beta-1	3	[illegible]	[illegible]
Acrdi.XM_015899299.1/1-831 | Monca.augustus.g44800.t1/1-1698	0.10844	0.119164	Organic cation transporter	2	F:GO:0022857; C:GO:0016020	[illegible]
Acrdi.XM_015922530.1/1-1872 | Monca.augustus.g18220.t1/1-1872	0.163453	0.119136	Tubulin polyglutamylase TTLL11	1	P:GO:0006464	[illegible]
Acrdi.XM_015917038.1/1-768 | Monca.adi2mcaRNA27930_R0/1-645	0.178267	0.119135	RNA-directed DNA polymerase from mobile element jockey	4	[illegible]	[illegible]
Acrdi.XM_015893230.1/1-3069 | Monca.augustus.g2998.t1/1-3117	0.145993	0.119128	solute carrier family 12 member 2 isoform X2	4	[illegible]	[illegible]
Acrdi.XM_015897623.1/1-1152 | Monca.augustus.g51735.t1/1-1119	0.158821	0.119105	PREDICTED: uncharacterized protein LOC107332865	2	C:GO:0016020; C:GO:0016021	[illegible]
Acrdi.XM_015893013.1/1-1560 | Monca.adi2mcaRNA3916_R0/1-1173	0.186817	0.119095	CDK5 regulatory subunit-associated 3-like	3	[illegible]	[illegible]
Acrdi.XM_015904503.1/1-1125 | Monca.augustus.g33581.t1/1-1122	0.18589	0.119077	SH3 and PX domain-containing 2B isoform X1			
Acrdi.XM_015906071.1/1-951 | Monca.augustus.g40449.t1/1-966	0.193698	0.119062	max-binding MNT-like	1	F:GO:0046983	[illegible]
Acrdi.XM_015892294.1/1-801 | Monca.adi2mcaRNA38208_R1/1-684	0.294282	0.119061	PREDICTED: uncharacterized protein LOC107327544			
Acrdi.XM_015909829.1/1-1800 | Monca.augustus.g47086.t1/1-1890	0.131486	0.119035	soluble guanylate cyclase 88E-like isoform X1	5	[illegible]	[illegible]
Acrdi.XM_015903109.1/1-777 | Monca.augustus.g18849.t1/1-792	0.160803	0.119027	methenyltetrahydrofolate synthase domain-containing isoform X2			
Acrdi.XM_015901603.1/1-1131 | Monca.augustus.g36335.t1/1-2457	0.128773	0.11902	calcium permeable stress-gated cation channel 1	3	[illegible]	[illegible]
Acrdi.XM_015913709.1/1-1071 | Monca.augustus.g12536.t1/1-1089	0.243736	0.118987	Glycosyltransferase 1 domain-containing 1	1	C:GO:0005737	[illegible]
Acrdi.XM_015903653.1/1-1137 | Monca.augustus.g10931.t1/1-1134	0.151456	0.118945	Ubiquitin-conjugating enzyme E2 J1			
Acrdi.XM_015903889.1/1-1068 | Monca.adi2mcaRNA13364_R6/1-885	0.43349	0.118938	Transposon Ty3-I Gag-Pol poly			
Acrdi.XM_015910550.1/1-1371 | Monca.augustus.g10111.t1/1-1914	0.114463	0.118927	serine arginine repetitive matrix 2-like	1	F:GO:0005158	[illegible]
Acrdi.XM_015895099.1/1-948 | Monca.augustus.g7698.t1/1-1692	0.147189	0.118896	dedicator of cytokinesis 9-			
Acrdi.XM_015904182.1/1-462 | Monca.augustus.g12947.t1/1-501	0.134708	0.118895	Thioredoxin domain-containing 12	2	C:GO:0005623; P:GO:0045454	[illegible]
Acrdi.XM_015903833.1/1-441 | Monca.augustus.g42708.t1/1-423	0.568276	0.118882	PHD finger ING1 isoform X1	2	C:GO:0005634; F:GO:0046872	[illegible]
Acrdi.XM_015900760.1/1-546 | Monca.augustus.g5457.t1/1-546	0.178724	0.118828	translocon-associated subunit gamma-like	3	[illegible]	[illegible]

Acrdi.XM_015895783.1/1-1746 | Monca.augustus.g47025.t1/1-1758	0.122024	0.118312	salvador homolog 1 isoform X3	3	P:GO:0050794; P:GO:0044699; P:GO:0009987	P:regulation of cellular process; P:single-organism process; P:cellular process		
Acrdi.XM_015895553.1/1-720 | Monca.augustus.g32976.t1/1-720	0.137339	0.118302	noggin	6	P:GO:0030514; P:GO:0048793; P:GO:0035844; P:GO:0007368;	P:negative regulation of BMP signaling pathway; P:pronephros development; P:cloaca development; P:determination of left/right symmetry; P:dorsal/ventral pattern formation; P:embryonic viscerocranium morphogenesis		
Acrdi.XM_015908466.1/1-363 | Monca.adi2mcaRNA18450_R0/1-447	0.0900711	0.118286	activated RNA polymerase II transcriptional coactivator p15-like	3	F:GO:0003677; F:GO:0003713; P:GO:0006355	F:DNA binding; F:transcription coactivator activity; P:regulation of transcription, DNA-templated		
Acrdi.XM_015898280.1/1-915 | Monca.augustus.g64617.t1/1-1068	0.0959967	0.118277	UPF0553 C9orf64 homolog	1	P:GO:0006400	P:tRNA modification		
Acrdi.XM_015898118.1/1-1293 | Monca.augustus.g50109.t1/1-1308	0.140318	0.118266	Bystin	2	P:GO:0042254; C:GO:0005730	P:ribosome biogenesis; C:nucleolus		
Acrdi.XM_015916602.1/1-684 | Monca.adi2mcaRNA2766_R1/1-621	0.443528	0.118244	G2 M phase-specific E3 ubiquitin- ligase-like					
Acrdi.XM_015912392.1/1-573 | Monca.augustus.g45164.t1/1-1029	0.168785	0.118241	NDRG3 isoform X5	2	C:GO:0005737; P:GO:0007165	C:cytoplasm; P:signal transduction		
Acrdi.XM_015908938.1/1-2283 | Monca.augustus.g31886.t1/1-1998	0.124433	0.11824	Extended synaptotagmin-3	1	C:GO:0005783	C:endoplasmic reticulum		
Acrdi.XM_015902473.1/1-1413 | Monca.augustus.g8731.t1/1-2184	0.10429	0.11823	Disintegrin and metallo ase domain-containing 9					
Acrdi.XM_015914811.1/1-1569 | Monca.augustus.g33303.t1/1-1800	0.142614	0.118213	ATP-binding cassette sub-family D member 4	8	F:GO:0000166; F:GO:0016887; F:GO:0005524; F:GO:0042626;	F:nucleotide binding; F:ATPase activity; F:ATP binding; F:ATPase activity, coupled to transmembrane movement of substances; P:transmembrane transport; C:membrane; C:integral component of membrane; P:transport		
Acrdi.XM_015912245.1/1-3951 | Monca.augustus.g28318.t1/1-4539	0.166842	0.118209	condensin complex subunit 1-like	7	C:GO:0000793; C:GO:0005634; P:GO:0030261; P:GO:0007059;	C:condensed chromosome; C:nucleus; P:chromosome condensation; P:chromosome segregation; P:mitotic nuclear division; F:binding; C:chromosomal part		
Acrdi.XM_015908994.1/1-1950 | Monca.adi2mcaRNA19014_R0/1-1923	0.117891	0.118188	cytochrome P450 3A24-like	1	F:GO:0016491	F:oxidoreductase activity		
Acrdi.XM_015920385.1/1-477 | Monca.augustus.g47488.t1/1-477	0.171147	0.118176	PREDICTED: uncharacterized protein LOC107353986					
Acrdi.XM_015905531.1/1-1179 | Monca.augustus.g28238.t1/1-1185	0.138101	0.118163	calcium-activated potassium channel subunit	1	C:GO:0016020	C:membrane		
Acrdi.XM_015896145.1/1-876 | Monca.augustus.g38251.t1/1-960	0.156683	0.118149	DUF445 domain-containing	2	C:GO:0016020; C:GO:0016021	C:membrane; C:integral component of membrane		
Acrdi.XM_015896858.1/1-600 | Monca.augustus.g49296.t1/1-600	0.161819	0.118125	50S ribosomal L9	2	C:GO:0043229; C:GO:0044444	C:intracellular organelle; C:cytoplasmic part		
Acrdi.XM_015911721.1/1-786 | Monca.augustus.g30203.t1/1-798	0.132216	0.118106	retinal homeobox Rx1-like	4	C:GO:0005634; P:GO:0007275; P:GO:0006355; F:GO:0043565	C:nucleus; P:multicellular organism development; P:regulation of transcription, DNA-templated; F:sequence-specific DNA binding		
Acrdi.XM_015915302.1/1-1641 | Monca.augustus.g25303.t1/1-1407	0.148316	0.118105	E3 ubiquitin- ligase RNF8-like	5	F:GO:0046872; F:GO:0016881; P:GO:0006325; P:GO:0050896;	F:metal ion binding; F:acid-amino acid ligase activity; P:chromatin organization; P:response to stimulus; P:protein ubiquitination		
Acrdi.XM_015918008.1/1-1029 | Monca.augustus.g18737.t1/1-1023	0.104942	0.118093	dopamine receptor 2-like	5	F:GO:0001588; P:GO:0001963; P:GO:0007186; F:GO:0035240;	F:dopamine neurotransmitter receptor activity, coupled via Gs; P:synaptic transmission, dopaminergic; P:G-protein coupled receptor signaling pathway; F:dopamine binding; C:integral component of membrane		
Acrdi.XM_015911227.1/1-1386 | Monca.augustus.g59546.t1/1-4860	0.117411	0.118083	hemocytin isoform X2	7	F:GO:0030414; F:GO:0004867; F:GO:0008061; P:GO:0006030;	F:peptidase inhibitor activity; F:serine-type endopeptidase inhibitor activity; F:chitin binding; P:chitin metabolic process; P:negative regulation of endopeptidase activity; C:extracellular region; P:negative regulation of peptidase activity		
Acrdi.XM_015912625.1/1-876 | Monca.augustus.g18544.t1/1-1119	0.113887	0.118075	kelch domain-containing 10					
Acrdi.XM_015906421.1/1-1008 | Monca.augustus.g243.t1/1-1008	0.138238	0.11807	Retinol dehydrogenase 8	2	F:GO:0016491; P:GO:0055114	F:oxidoreductase activity; P:oxidation-reduction process		
Acrdi.XM_015923406.1/1-3189 | Monca.augustus.g41307.t1/1-3351	0.129938	0.118061	ubiquitin carboxyl-terminal hydrolase 48 isoform X1					
Acrdi.XM_015894235.1/1-3180 | Monca.augustus.g33359.t1/1-3336	0.132903	0.118007	kinesin KIF11	4	F:GO:0003777; P:GO:0044763; F:GO:0005488; P:GO:0007017	F:microtubule motor activity; P:single-organism cellular process; F:binding; P:microtubule-based process	EC:3.6.1.15	Nucleoside-triphosphate phosphatase
Acrdi.XM_015896798.1/1-936 | Monca.augustus.g66636.t1/1-2433	0.122127	0.117982	twinkle mitochondrial	5	F:GO:0005524; P:GO:0006260; P:GO:0032508; F:GO:0003697;	F:ATP binding; P:DNA replication; P:DNA duplex unwinding; F:single-stranded DNA binding; F:5'-3' DNA helicase activity	EC:3.6.1.15	Nucleoside-triphosphate phosphatase
Acrdi.XM_015900544.1/1-2649 | Monca.augustus.g37040.t1/1-2664	0.143256	0.117961	transmembrane 245 isoform X2	2	C:GO:0016020; C:GO:0016021	C:membrane; C:integral component of membrane		
Acrdi.XM_015915949.1/1-495 | Monca.augustus.g61003.t1/1-492	0.201957	0.117947	CB1 cannabinoid receptor-interacting 1-like					
Acrdi.XM_015916032.1/1-1194 | Monca.augustus.g60991.t1/1-1233	0.163244	0.117934	PREDICTED: uncharacterized protein C1orf198 homolog					
Acrdi.XM_015913627.1/1-1020 | Monca.augustus.g47443.t1/1-1047	0.201424	0.117923	activator of 90 kDa heat shock ATPase homolog 1	4	P:GO:0032781; F:GO:0051087; F:GO:0001671; P:GO:0006950	P:positive regulation of ATPase activity; F:chaperone binding; F:ATPase activator activity; P:response to stress		
Acrdi.XM_015913678.1/1-1170 | Monca.augustus.g30462.t1/1-1170	0.11301	0.117921	chloroplast stem-loop binding of 41 kDa chloroplastic-like	4	F:GO:0050662; F:GO:0003824; C:GO:0016020; C:GO:0016021	F:coenzyme binding; F:catalytic activity; C:membrane; C:integral component of membrane		
Acrdi.XM_015893537.1/1-2493 | Monca.augustus.g25405.t1/1-2658	0.121304	0.117872	FAM160A1					
Acrdi.XM_015897173.1/1-2913 | Monca.augustus.g22252.t1/1-3114	0.145878	0.117858	Ubiquitin conjugation factor E4 A	5	F:GO:0004842; C:GO:0000151; F:GO:0034450; P:GO:0016567;	F:ubiquitin-protein transferase activity; C:ubiquitin ligase complex; F:ubiquitin-ubiquitin ligase activity; P:protein ubiquitination; P:ubiquitin-dependent protein catabolic process		
Acrdi.XM_015920675.1/1-1224 | Monca.adi2mcaRNA31985_R2/1-1194	0.369744	0.117844	Ephrin type-B receptor 1	15	P:GO:0018108; P:GO:0007411; P:GO:0046777; F:GO:0005005;	P:peptidyl-tyrosine phosphorylation; P:axon guidance; P:protein autophosphorylation; F:transmembrane-ephrin receptor activity; P:regulation of JNK cascade; P:cell chemotax	EC:2.7.10; EC:2.7.10.1; EC:2.7.11	Transferring phosphorus-containing groups; Receptor protein-tyrosine kinase [illegible]
Acrdi.XM_015915199.1/1-2847 | Monca.augustus.g29754.t1/1-2820	0.148287	0.117831	Extracellular calcium-sensing receptor	4	F:GO:0004888; C:GO:0016021; P:GO:0007216; C:GO:0044459	F:transmembrane signaling receptor activity; C:integral component of membrane; P:G-protein coupled glutamate receptor signaling pathway; C:plasma membrane part		
Acrdi.XM_015916147.1/1-1680 | Monca.augustus.g12400.t1/1-1944	0.142688	0.117753	methyltransferase NSUN7 isoform X1	2	F:GO:0008168; P:GO:0032259	F:methyltransferase activity; P:methylation		
Acrdi.XM_015925180.1/1-633 | Monca.augustus.g53763.t1/1-681	0.140875	0.117748	60S ribosomal L13	5	F:GO:0003723; F:GO:0003735; C:GO:0022625; P:GO:0051726;	F:RNA binding; F:structural constituent of ribosome; C:cytosolic large ribosomal subunit; P:regulation of cell cycle; P:translation		
Acrdi.XM_015896123.1/1-663 | Monca.augustus.g55597.t1/1-837	0.128053	0.117745	Lens fiber major intrinsic	5	C:GO:0005887; F:GO:0015254; P:GO:0009992; P:GO:0034220;	C:integral component of plasma membrane; F:glycerol channel activity; P:cellular water homeostasis; P:ion transmembrane transport; F:water channel activity		
Acrdi.XM_015906877.1/1-948 | Monca.augustus.g32951.t1/1-1212	0.176256	0.117738	ATP-sensitive inward rectifier potassium channel 12-like	3	C:GO:0005887; F:GO:0005242; P:GO:0010107	C:integral component of plasma membrane; F:inward rectifier potassium channel activity; P:potassium ion import		
Acrdi.XM_015894132.1/1-1548 | Monca.augustus.g53471.t1/1-1542	0.138536	0.117737	cell division cycle 20 homolog	4	P:GO:0051301; F:GO:0097027; P:GO:0050790; F:GO:0010997	P:cell division; F:ubiquitin-protein transferase activator activity; P:regulation of catalytic activity; F:anaphase-promoting complex binding		
Acrdi.XM_015892056.1/1-540 | Monca.augustus.g8376.t1/1-582	0.124928	0.117729	high affinity copper uptake 1-like	6	P:GO:0042539; C:GO:0016021; P:GO:0046688; F:GO:0005375;	P:hypotonic salinity response; C:integral component of membrane; P:response to copper ion; F:copper ion transmembrane transporter activity; P:copper ion transmembrane transport; P:cellular response to estrogen stimulus		
Acrdi.XM_015902462.1/1-1050 | Monca.augustus.g41947.t1/1-948	0.110453	0.117712	melatonin receptor type 1A-like	3	F:GO:0004930; P:GO:0007186; C:GO:0016021	F:G-protein coupled receptor activity; P:G-protein coupled receptor signaling pathway; C:integral component of membrane		
Acrdi.XM_015903576.1/1-924 | Monca.augustus.g12040.t1/1-1053	0.643975	0.117711	Retrovirus-related Pol poly from transposon					
Acrdi.XM_015906008.1/1-1089 | Monca.augustus.g41410.t1/1-1587	0.148775	0.117683	dTDP-glucose 4,6-dehydratase-like	5	P:GO:0070085; F:GO:0016757; F:GO:0050662; C:GO:0016020;	P:glycosylation; F:transferase activity, transferring glycosyl groups; F:coenzyme binding; C:membrane; C:Golgi apparatus		
Acrdi.XM_015903633.1/1-792 | Monca.augustus.g16343.t1/1-960	0.188138	0.117681	histidine N-acetyltransferase-like	3	P:GO:0006474; C:GO:0031248; F:GO:0004596	P:N-terminal protein amino acid acetylation; C:protein acetyltransferase complex; F:peptide alpha-N-acetyltransferase activity	EC:2.3.1.88	Peptide alpha-N-acetyltransferase
Acrdi.XM_015924544.1/1-573 | Monca.augustus.g17642.t1/1-648	0.143681	0.117667	glutathione S-transferase Mu 3-like	2	P:GO:0008152; F:GO:0004364	P:metabolic process; F:glutathione transferase activity		
Acrdi.XM_015915209.1/1-1626 | Monca.augustus.g30640.t1/1-969	0.186128	0.117652	WD repeat-containing 54 isoform X1					
Acrdi.XM_015917977.1/1-1323 | Monca.augustus.g45177.t1/1-1563	0.111799	0.117643	DDB1- and CUL4-associated factor 17	1	P:GO:0016567	P:protein ubiquitination		
Acrdi.XM_015918666.1/1-597 | Monca.augustus.g7588.t1/1-633	0.154814	0.1176	dickkopf-related 3-like	3	P:GO:0007275; P:GO:0030178; C:GO:0005576	P:multicellular organism development; P:negative regulation of Wnt signaling pathway; C:extracellular region		
Acrdi.XM_015908400.1/1-618 | Monca.augustus.g9821.t1/1-711	0.134478	0.117597	rRNA methyltransferase mitochondrial	3	F:GO:0008650; C:GO:0005739; P:GO:0000453	F:rRNA (uridine-2'-O-)-methyltransferase activity; C:mitochondrion; P:enzyme-directed rRNA 2'-O-methylation		
Acrdi.XM_015908540.1/1-936 | Monca.augustus.g65965.t1/1-963	0.167472	0.117564	translocon-associated subunit alpha isoform X2	2	C:GO:0005783; C:GO:0044425	C:endoplasmic reticulum; C:membrane part		
Acrdi.XM_015909750.1/1-2175 | Monca.adi2mcaRNA19855_R0/1-2055	0.138505	0.117559	neuropeptide FF receptor 2-like isoform X1	4	C:GO:0005887; F:GO:0004930; P:GO:0007186; P:GO:0007166	C:integral component of plasma membrane; F:G-protein coupled receptor activity; P:G-protein coupled receptor signaling pathway; P:cell surface receptor signaling pathway		
Acrdi.XM_015897823.1/1-609 | Monca.augustus.g65535.t1/1-615	0.161871	0.11755	DPCD isoform X2					
Acrdi.XM_015908114.1/1-3489 | Monca.augustus.g33227.t1/1-4650	0.158898	0.117538	ral GTPase-activating subunit beta isoform X3	3	P:GO:0051056; F:GO:0005096; P:GO:0043547	P:regulation of small GTPase mediated signal transduction; F:GTPase activator activity; P:positive regulation of GTPase activity		
Acrdi.XM_015912453.1/1-11145 | Monca.adi2mcaRNA22827_R0/1-8958	0.1266	0.117522	E3 ubiquitin- ligase HUWE1-like					
Acrdi.XM_015896413.1/1-954 | Monca.adi2mcaRNA5249_R0/1-900	0.38443	0.11752	PREDICTED: uncharacterized protein LOC107331788	2	C:GO:0016020; P:GO:0006259	C:membrane; P:DNA metabolic process		
Acrdi.XM_015913258.1/1-3105 | Monca.augustus.g46139.t1/1-3441	0.14567	0.117466	alpha-mannosidase 2x isoform X1	7	P:GO:0006517; F:GO:0004559; F:GO:0048731; P:GO:0006491;	P:protein deglycosylation; F:alpha-mannosidase activity; P:system development; P:N-glycan processing; P:mannose metabolic process; C:Golgi membrane; F:binding	EC:3.2.1.24	Alpha-mannosidase
Acrdi.XM_015918860.1/1-2733 | Monca.augustus.g66326.t1/1-3921	0.150482	0.117455	CLIP-associating 1 isoform X1					
Acrdi.XM_015899932.1/1-633 | Monca.adi2mcaRNA862_R0/1-684	0.127539	0.117454	palmitoleoyl- carboxylesterase notum1a-like	5	C:GO:0016020; P:GO:0006508; C:GO:0016021; F:GO:0008236;	C:membrane; P:proteolysis; C:integral component of membrane; F:serine-type peptidase activity; F:hydrolase activity		
Acrdi.XM_015893812.1/1-975 | Monca.augustus.g50545.t1/1-1026	0.149261	0.117439	neuropeptide FF receptor 2-like isoform X1	3	F:GO:0004930; P:GO:0007186; C:GO:0016021	F:G-protein coupled receptor activity; P:G-protein coupled receptor signaling pathway; C:integral component of membrane		
Acrdi.XM_015909997.1/1-825 | Monca.augustus.g6685.t1/1-1263	0.102845	0.117439	splicing factor 3B subunit 4	2	F:GO:0097159; F:GO:1901363	F:organic cyclic compound binding; F:heterocyclic compound binding		
Acrdi.XM_015904224.1/1-489 | Monca.augustus.g11837.t1/1-486	0.134432	0.117434	Universal stress	1	P:GO:0006950	P:response to stress		
Acrdi.XM_015909051.1/1-1392 | Monca.augustus.g38380.t1/1-1455	0.115151	0.117427	calcium calmodulin-dependent kinase type II alpha chain isoform X13	4	F:GO:0005524; F:GO:0005516; F:GO:0004683; P:GO:0006468	F:ATP binding; F:calmodulin binding; F:calmodulin-dependent protein kinase activity; P:protein phosphorylation	EC:2.7.11; EC:2.7.11.17	Transferring phosphorus-containing groups; Calcium/calmodulin-dependent [illegible]
Acrdi.XM_015899120.1/1-312 | Monca.augustus.g65832.t1/1-567	0.12785	0.117424	Translocon-associated subunit beta	2	C:GO:0005789; C:GO:0016021	C:endoplasmic reticulum membrane; C:integral component of membrane		
Acrdi.XM_015921378.1/1-723 | Monca.augustus.g4654.t1/1-984	0.169798	0.117417	PREDICTED: uncharacterized protein LOC107354887	4	C:GO:0005737; F:GO:0000062; F:GO:0008757; P:GO:0032259	C:cytoplasm; F:fatty-acyl-CoA binding; F:S-adenosylmethionine-dependent methyltransferase activity; P:methylation		
Acrdi.XM_015911428.1/1-798 | Monca.augustus.g69036.t1/1-1314	0.183531	0.117373	Phosphatidylinositol glycan anchor biosynthesis class U	1	C:GO:0016020	C:membrane		
Acrdi.XM_015901193.1/1-942 | Monca.adi2mcaRNA10443_R0/1-912	0.137224	0.117371	phospholipid scramblase 1	2	F:GO:0003676; P:GO:0006353	F:nucleic acid binding; P:DNA-templated transcription, termination		
Acrdi.XM_015897715.1/1-621 | Monca.augustus.g9967.t1/1-684	0.185043	0.117365	Ribosomal RNA small subunit methyltransferase NEP1	8	C:GO:0032040; P:GO:0000462; P:GO:0070475; P:GO:0001824;	C:small-subunit processome; P:maturation of SSU-rRNA from tricistronic rRNA transcript (SSU-rRNA, 5.8S rRNA, LSU-rRNA); P:rRNA base methylation; P:blastocyst development; F:rRNA (pseudouridine) methyltransferase activity; C:nucleolus; F:rRNA binding; P:nucleologenesis		
Acrdi.XM_015918976.1/1-2151 | Monca.augustus.g57244.t1/1-2178	0.139828	0.117341	pleckstrin domain-containing					
Acrdi.XM_015898382.1/1-1224 | Monca.adi2mcaRNA7436_R1/1-840	0.291588	0.117328	nuclease HARBI1	2	C:GO:0005813; C:GO:0005737	C:centrosome; C:cytoplasm		
Acrdi.XM_015914180.1/1-2004 | Monca.augustus.g50929.t1/1-1962	0.150169	0.117308	neuroendocrine convertase 1-like isoform X1	2	P:GO:0006508; F:GO:0004252	P:proteolysis; F:serine-type endopeptidase activity	EC:3.4.21	Acting on peptide bonds (peptidases)
Acrdi.XM_015903389.1/1-1245 | Monca.augustus.g16024.t1/1-1194	0.159009	0.117273	Programmed cell death 2	5	P:GO:0030218; C:GO:0005737; P:GO:0007088; F:GO:0046872;	P:erythrocyte differentiation; C:cytoplasm; P:regulation of mitotic nuclear division; F:metal ion binding; P:hematopoietic stem cell differentiation		
Acrdi.XM_015907670.1/1-798 | Monca.augustus.g29952.t1/1-786	0.188995	0.117249	transcription factor AP-4-like isoform X2	3	C:GO:0005634; F:GO:0003700; P:GO:0006357	C:nucleus; F:transcription factor activity, sequence-specific DNA binding; P:regulation of transcription from RNA polymerase II promoter		
Acrdi.XM_015912388.1/1-579 | Monca.augustus.g45151.t1/1-687	0.139318	0.117217	Cyclin-dependent kinase 2-interacting					
Acrdi.XM_015900159.1/1-3741 | Monca.augustus.g12825.t1/1-2457	0.113004	0.117207	DNA repair endonuclease XPF	5	C:GO:0044428; F:GO:0003677; P:GO:0033683; P:GO:0006310;	C:nuclear part; F:DNA binding; P:nucleotide-excision repair, DNA incision; P:DNA recombination; F:endodeoxyribonuclease activity		
[illegible]								

Acrdi.XM_015904079.1/1-1071 | Monca.augustus.g32306.t1/1-1071	0.189644	0.113286	PREDICTED: uncharacterized protein LOC107338826	2	C:GO:0016020; C:GO:0016021	C:membrane; C:integral component of membrane
Acrdi.XM_015912721.1/1-357 | Monca.augustus.g43031.t1/1-1569	0.102666	0.113286	PREDICTED: uncharacterized protein LOC107346866	5	F:GO:0031418; F:GO:0005506; F:GO:0016491; F:GO:0016705;	F:L-ascorbic acid binding; F:iron ion binding; F:oxidoreductase activity; F:oxidoreductase activity, acting on paired donors, with incorporation or reduction of molecular oxygen; [illegible]
Acrdi.XM_015898200.1/1-1083 | Monca.augustus.g29230.t1/1-1125	0.130375	0.113285	Wnt-10a-like isoform X1	5	C:GO:0005615; F:GO:0005109; P:GO:0030182; P:GO:0045165;	C:extracellular space; F:frizzled binding; P:neuron differentiation; P:cell fate commitment; P:Wnt signaling pathway
Acrdi.XM_015915736.1/1-993 | Monca.augustus.g55311.t1/1-987	0.100742	0.113271	adenosine receptor A2a-like	5	F:GO:0001588; P:GO:0001963; P:GO:0007165; F:GO:0035240;	F:dopamine neurotransmitter receptor activity, coupled via Gs; P:synaptic transmission, dopaminergic; P:signal transduction; F:dopamine binding; C:membrane
Acrdi.XM_015903087.1/1-639 | Monca.augustus.g65497.t1/1-651	0.161	0.113269	Homeobox -like 2	3	C:GO:0005634; P:GO:0006355; F:GO:0043565	C:nucleus; P:regulation of transcription, DNA-templated; F:sequence-specific DNA binding
Acrdi.XM_015922091.1/1-3264 | Monca.augustus.g39478.t1/1-3120	0.16896	0.113266	Plexin-A4	2	C:GO:0016021; P:GO:0071526	C:integral component of membrane; P:semaphorin-plexin signaling pathway
Acrdi.XM_015900372.1/1-501 | Monca.augustus.g54183.t1/1-513	0.104665	0.113261	ankyrin repeat and SOCS box 13-like			
Acrdi.XM_015921732.1/1-1314 | Monca.augustus.g49906.t1/1-1584	0.170529	0.113248	Folliculin	2	F:GO:0005085; P:GO:0043547	F:guanyl-nucleotide exchange factor activity; P:positive regulation of GTPase activity
Acrdi.XM_015903296.1/1-840 | Monca.augustus.g21555.t1/1-1077	0.110116	0.11322	RING finger 32 isoform X1	1	F:GO:0046872	F:metal ion binding
Acrdi.XM_015900705.1/1-4824 | Monca.adi2mcaRNA9965_R0/1-4797	0.158583	0.113189	chromodomain-helicase-DNA-binding 4-like			
Acrdi.XM_015913424.1/1-2616 | Monca.augustus.g32123.t1/1-3318	0.149119	0.113177	Trafficking kinesin-binding 1			
Acrdi.XM_015912425.1/1-1116 | Monca.augustus.g40747.t1/1-2403	0.105169	0.113172	exonuclease 1-like	4	F:GO:0003677; P:GO:0006281; P:GO:0090305; F:GO:0035312	F:DNA binding; P:DNA repair; P:nucleic acid phosphodiester bond hydrolysis; F:5'-3' exodeoxyribonuclease activity [illegible]
Acrdi.XM_015900171.1/1-861 | Monca.augustus.g65819.t1/1-855	0.169099	0.113163	lysM and peptidoglycan-binding domain-containing 3	2	C:GO:0016020; C:GO:0016021	C:membrane; C:integral component of membrane
Acrdi.XM_015917724.1/1-1776 | Monca.adi2mcaRNA28709_R0/1-1335	0.109058	0.113162	Polypeptide N-acetylgalactosaminyltransferase	5	F:GO:0016757; C:GO:0016021; P:GO:0006486; C:GO:0005794;	F:transferase activity, transferring glycosyl groups; C:integral component of membrane; P:protein glycosylation; C:Golgi apparatus; F:carbohydrate binding
Acrdi.XM_015907406.1/1-1119 | Monca.adi2mcaRNA17284_R0/1-1098	0.16211	0.113135	F-box LRR-repeat 2-like			
Acrdi.XM_015907801.1/1-813 | Monca.augustus.g9242.t1/1-372	1.26571	0.113105	nucleoporin GLE1-like			
Acrdi.XM_015924445.1/1-1023 | Monca.adi2mcaRNA36238_R0/1-1047	0.674699	0.1131	PREDICTED: uncharacterized protein LOC107357807			
Acrdi.XM_015914105.1/1-1323 | Monca.augustus.g59141.t1/1-1743	0.149943	0.113098	Transferrin receptor 1			
Acrdi.XM_015912485.1/1-492 | Monca.augustus.g40505.t1/1-813	0.362628	0.113068	PREDICTED: uncharacterized protein LOC107346681			
Acrdi.XM_015901897.1/1-1098 | Monca.augustus.g58313.t1/1-1107	0.119455	0.113056	MAGUK p55 subfamily member 5-A-like	1	F:GO:0016740	F:transferase activity
Acrdi.XM_015909195.1/1-1092 | Monca.augustus.g6499.t1/1-1224	0.106088	0.11305	gamma-aminobutyric acid receptor subunit beta-1 isoform X2	3	P:GO:0006811; F:GO:0099600; C:GO:0016020	P:ion transport; F:transmembrane receptor activity; C:membrane
Acrdi.XM_015912623.1/1-2703 | Monca.augustus.g53946.t1/1-2421	0.137171	0.113041	dynein assembly factor axonemal-like			
Acrdi.XM_015911367.1/1-780 | Monca.augustus.g35271.t1/1-1047	0.128776	0.113022	Serine arginine-rich splicing factor 2	8	F:GO:0000166; F:GO:0036002; P:GO:0008380; C:GO:0070062;	F:nucleotide binding; F:pre-mRNA binding; P:RNA splicing; C:extracellular exosome; P:regulation of alternative mRNA splicing, via spliceosome; C:nuclear speck; [illegible]
Acrdi.XM_015901286.1/1-900 | Monca.adi2mcaRNA10524_R0/1-840	0.120435	0.112972	cystinosin homolog	3	F:GO:0015184; C:GO:0005774; P:GO:0015811	F:L-cystine transmembrane transporter activity; C:vacuolar membrane; P:L-cystine transport
Acrdi.XM_015908564.1/1-2256 | Monca.augustus.g13043.t1/1-2283	0.152192	0.112941	E3 ubiquitin- ligase TRIM71	4	F:GO:0008270; F:GO:0046872; F:GO:0016874; C:GO:0005622	F:zinc ion binding; F:metal ion binding; F:ligase activity; C:intracellular
Acrdi.XM_015920688.1/1-1005 | Monca.augustus.g44632.t1/1-1011	0.109949	0.112915	melatonin receptor type 1B-like	4	C:GO:0005887; F:GO:0004930; P:GO:0007186; P:GO:0007166	C:integral component of plasma membrane; F:G-protein coupled receptor activity; P:G-protein coupled receptor signaling pathway; P:cell surface receptor signaling pathway
Acrdi.XM_015911009.1/1-1002 | Monca.augustus.g59078.t1/1-981	0.142439	0.112897	trace amine-associated receptor 9	3	F:GO:0004930; P:GO:0007154; P:GO:0044700	F:G-protein coupled receptor activity; P:cell communication; P:single organism signaling
Acrdi.XM_015918267.1/1-1182 | Monca.augustus.g18789.t1/1-1215	0.159406	0.112876	alpha-1A adrenergic receptor-like	7	F:GO:0004930; P:GO:0007186; C:GO:0016020; P:GO:0044057;	F:G-protein coupled receptor activity; P:G-protein coupled receptor signaling pathway; C:membrane; P:regulation of system process; P:single-organism cellular process; F:binding; [illegible]
Acrdi.XM_015909227.1/1-471 | Monca.augustus.g36735.t1/1-471	0.101728	0.112868	Universal stress	1	P:GO:0006950	P:response to stress
Acrdi.XM_015918215.1/1-1221 | Monca.augustus.g13567.t1/1-1257	0.118183	0.112833	Mannose-1-phosphate guanyltransferase alpha-A	1	F:GO:0016740	F:transferase activity
Acrdi.XM_015924145.1/1-624 | Monca.augustus.g62404.t1/1-627	0.120846	0.112817	eukaryotic translation initiation factor 5B-like			
Acrdi.XM_015923127.1/1-3636 | Monca.augustus.g32451.t1/1-5697	0.14571	0.112775	nuclear pore complex Nup98-Nup96 isoform X1	2	P:GO:0051641; P:GO:0006810	P:cellular localization; P:transport
Acrdi.XM_015900860.1/1-609 | Monca.augustus.g27841.t1/1-1968	0.124148	0.112763	phosphatase 1 regulatory inhibitor subunit 16B-like	9	P:GO:0007391; P:GO:0030725; P:GO:0048477; P:GO:0048675;	P:dorsal closure; P:germline ring canal formation; P:oogenesis; P:axon extension; P:regulation of locomotor rhythm; C:apical junction complex; P:imaginal disc-derived wing hair [illegible]
Acrdi.XM_015895237.1/1-3090 | Monca.augustus.g6366.t1/1-3102	0.155135	0.112757	tetratricopeptide repeat 34			
Acrdi.XM_015907685.1/1-969 | Monca.augustus.g29927.t1/1-1266	0.13532	0.112732	tryptophan 2,3-dioxygenase	7	F:GO:0004833; P:GO:0006727; P:GO:0019441; P:GO:0019442;	F:tryptophan 2,3-dioxygenase activity; P:ommochrome biosynthetic process; P:tryptophan catabolic process to kynurenine; P:tryptophan catabolic process to acetyl-CoA; P:pos [illegible]
Acrdi.XM_015896673.1/1-357 | Monca.augustus.g22611.t1/1-528	0.185273	0.112717	28S ribosomal mitochondrial-like	4	F:GO:0003735; C:GO:0022627; P:GO:0032543; C:GO:0005763	F:structural constituent of ribosome; C:cytosolic small ribosomal subunit; P:mitochondrial translation; C:mitochondrial small ribosomal subunit
Acrdi.XM_015913209.1/1-837 | Monca.augustus.g32052.t1/1-849	0.141028	0.112716	valacyclovir hydrolase	2	P:GO:0006520; F:GO:0016787	P:cellular amino acid metabolic process; F:hydrolase activity
Acrdi.XM_015909445.1/1-1902 | Monca.augustus.g1273.t1/1-2190	0.120671	0.112683	serine threonine- kinase B-	47	P:GO:0018108; P:GO:0051591; P:GO:0008542; P:GO:0043367;	P:peptidyl-tyrosine phosphorylation; P:response to cAMP; P:visual learning; P:CD4-positive, alpha-beta T cell differentiation; P:protein heterooligomerization; P:CD4-positive [illegible]
Acrdi.XM_015925103.1/1-849 | Monca.augustus.g51154.t1/1-855	0.151213	0.112674	PREDICTED: uncharacterized protein LOC107358503			
Acrdi.XM_015904195.1/1-720 | Monca.augustus.g61673.t1/1-726	0.214721	0.112658	exosome complex component rrp40	12	C:GO:0000177; C:GO:0000176; P:GO:0071051; F:GO:0003723;	C:cytoplasmic exosome (RNase complex); C:nuclear exosome (RNase complex); P:polyadenylation-dependent snoRNA 3'-end processing; F:RNA binding; P:U4 snRNA 3'-end [illegible]
Acrdi.XM_015908665.1/1-3327 | Monca.augustus.g53885.t1/1-3885	0.139427	0.112606	TBC1 domain family member	7	P:GO:0031338; P:GO:0090630; F:GO:0005096; C:GO:0012505;	P:regulation of vesicle fusion; P:activation of GTPase activity; F:GTPase activator activity; C:endomembrane system; P:intracellular protein transport; F:Rab GTPase binding; [illegible]
Acrdi.XM_015920093.1/1-1572 | Monca.augustus.g55385.t1/1-1620	0.141255	0.112601	CWF19 1	1	F:GO:0003824	F:catalytic activity
Acrdi.XM_015907655.1/1-2769 | Monca.augustus.g49947.t1/1-2772	0.117479	0.112547	tolloid 1	2	F:GO:0046872; F:GO:0008237	F:metal ion binding; F:metallopeptidase activity
Acrdi.XM_015902623.1/1-1434 | Monca.augustus.g22674.t1/1-1626	0.133615	0.112544	General transcription factor IIH subunit 1	3	P:GO:0006351; C:GO:0000439; P:GO:0006289	P:transcription, DNA-templated; C:core TFIIH complex; P:nucleotide-excision repair
Acrdi.XM_015907228.1/1-1323 | Monca.augustus.g8085.t1/1-1557	0.123836	0.11254	zinc finger 704-like	1	F:GO:0046872	F:metal ion binding
Acrdi.XM_015905985.1/1-1851 | Monca.augustus.g41390.t1/1-2469	0.118217	0.112538	nuclear pore complex Nup93-like	5	C:GO:0005635; P:GO:0006913; F:GO:0005198; P:GO:0006886;	C:nuclear envelope; P:nucleocytoplasmic transport; F:structural molecule activity; P:intracellular protein transport; P:mRNA transport
Acrdi.XM_015906934.1/1-2376 | Monca.augustus.g667.t1/1-2526	0.159186	0.112522	Elongator complex 2			
Acrdi.XM_015901890.1/1-453 | Monca.augustus.g7731.t1/1-513	0.10341	0.112514	Microsomal glutathione S-transferase 1	3	C:GO:0016020; C:GO:0016021; F:GO:0016740	C:membrane; C:integral component of membrane; F:transferase activity
Acrdi.XM_015908076.1/1-750 | Monca.adi2mcaRNA18026_R0/1-750	0.524256	0.112505	RNA-directed DNA polymerase from mobile element jockey-like	4	F:GO:0008270; F:GO:0046872; C:GO:0016020; C:GO:0016021	F:zinc ion binding; F:metal ion binding; C:membrane; C:integral component of membrane
Acrdi.XM_015909643.1/1-4116 | Monca.augustus.g53336.t1/1-4107	0.127619	0.112488	kinase domain containing	2	P:GO:0065007; P:GO:0009987	P:biological regulation; P:cellular process
Acrdi.XM_015920516.1/1-402 | Monca.augustus.g25714.t1/1-825	0.114871	0.112488	E3 ubiquitin- ligase RNF170	1	C:GO:0016020	C:membrane
Acrdi.XM_015921833.1/1-948 | Monca.augustus.g66169.t1/1-936	0.14144	0.11248	dnaJ homolog subfamily C member 17	2	F:GO:0097159; F:GO:1901363	F:organic cyclic compound binding; F:heterocyclic compound binding
Acrdi.XM_015893636.1/1-993 | Monca.augustus.g64054.t1/1-1341	0.118448	0.112469	Tat-linked quality control	1	F:GO:0016788	F:hydrolase activity, acting on ester bonds
Acrdi.XM_015906818.1/1-825 | Monca.adi2mcaRNA16590_R0/1-825	0.164408	0.112449	elongation of very long chain fatty acids 4-like isoform X1	3	C:GO:0016021; F:GO:0016740; P:GO:0006633	C:integral component of membrane; F:transferase activity; P:fatty acid biosynthetic process
Acrdi.XM_015903504.1/1-4074 | Monca.adi2mcaRNA12974_R0/1-4005	0.103255	0.112417	cyclin-G-associated kinase isoform X1	3	F:GO:0005524; F:GO:0004672; P:GO:0006468	F:ATP binding; F:protein kinase activity; P:protein phosphorylation
Acrdi.XM_015918177.1/1-762 | Monca.augustus.g66563.t1/1-777	0.133905	0.112414	thiamin pyrophosphokinase 1	1	P:GO:0044237	P:cellular metabolic process
Acrdi.XM_015895892.1/1-765 | Monca.augustus.g4112.t1/1-2250	0.0831454	0.112413	solute carrier family 15 member 2 isoform X2	6	P:GO:0006857; F:GO:0015198; C:GO:0016020; C:GO:0016021;	P:oligopeptide transport; F:oligopeptide transporter activity; C:membrane; C:integral component of membrane; P:transport; F:transporter activity
Acrdi.XM_015909657.1/1-1233 | Monca.augustus.g44689.t1/1-5463	0.104914	0.112412	germinal-center associated nuclear	2	C:GO:0005634; C:GO:0005737	C:nucleus; C:cytoplasm
Acrdi.XM_015918452.1/1-4074 | Monca.augustus.g55064.t1/1-5436	0.138025	0.112383	Multidrug resistance 1	8	F:GO:0000166; F:GO:0016887; F:GO:0005524; F:GO:0042626;	F:nucleotide binding; F:ATPase activity; F:ATP binding; F:ATPase activity, coupled to transmembrane movement of substances; P:transmembrane transport; C:membrane; [illegible]
Acrdi.XM_015908212.1/1-1356 | Monca.augustus.g7876.t1/1-1341	0.149325	0.112373	PREDICTED: uncharacterized protein LOC107342710 isoform X2	2	C:GO:0016020; C:GO:0016021	C:membrane; C:integral component of membrane
Acrdi.XM_015910644.1/1-582 | Monca.augustus.g14645.t1/1-1839	0.0756892	0.11237	Coiled-coil domain-containing 148			
Acrdi.XM_015913057.1/1-846 | Monca.augustus.g15092.t1/1-867	0.188726	0.112349	ATP synthase mitochondrial F1 complex assembly factor 2	1	P:GO:0043461	P:proton-transporting ATP synthase complex assembly
Acrdi.XM_015914999.1/1-1521 | Monca.augustus.g42515.t1/1-1770	0.101402	0.112338	cyclic nucleotide-binding domain-containing 2-like			
Acrdi.XM_015921765.1/1-5148 | Monca.adi2mcaRNA33139_R1/1-4626	0.144733	0.112326	EGF-like repeat and discoidin I-like domain-containing 3	1	F:GO:0070492	F:oligosaccharide binding
Acrdi.XM_015918415.1/1-963 | Monca.augustus.g61763.t1/1-816	0.121455	0.112305	prostaglandin E2 receptor EP4 subtype-like	3	F:GO:0004930; P:GO:0007186; C:GO:0016021	F:G-protein coupled receptor activity; P:G-protein coupled receptor signaling pathway; C:integral component of membrane

Acrdi.XM_015896766.1/1-489 | Monca.augustus.g14388.t1/1-1320	0.137299	0.108528	fuzzy homolog	7	P:GO:0001843; P:GO:0010172; P:GO:0001736; C:GO:0044424;	P:neural tube closure; P:embryonic body morphogenesis; P:establishment of planar polarity; C:intracellular part; P:transport; P:embryonic skeletal system morphogenesis; P:regulation of smoothened signaling pathway	
Acrdi.XM_015921690.1/1-1038 | Monca.augustus.g43099.t1/1-1041	0.080217	0.108526	cell division cycle 123 homolog	5	P:GO:0006417; P:GO:0007050; P:GO:0001732; C:GO:0005737;	P:regulation of translation; P:cell cycle arrest; P:formation of cytoplasmic translation initiation complex; C:cytoplasm; P:regulation of mitotic cell cycle	
Acrdi.XM_015913138.1/1-807 | Monca.augustus.g70993.t1/1-1182	0.14254	0.108512	carnosine N-methyltransferase-like	4	C:GO:0005634; F:GO:0030735; P:GO:0035498; C:GO:0005829	C:nucleus; F:carnosine N-methyltransferase activity; P:carnosine metabolic process; C:cytosol	EC:2.1.1.22
Acrdi.XM_015898448.1/1-3000 | Monca.augustus.g41768.t1/1-3393	0.137186	0.108501	cytosolic carboxypeptidase 1 isoform X1	11	P:GO:0043009; F:GO:0004180; P:GO:0030182; P:GO:0033077;	P:chordate embryonic development; F:carboxypeptidase activity; P:neuron differentiation; P:T cell differentiation in thymus; C:intracellular membrane-bounded organelle; P:protein deglutamylation; P:anatomical structure morphogenesis; C:cytoplasmic [illegible]	
Acrdi.XM_015912783.1/1-789 | Monca.augustus.g38073.t1/1-849	0.16657	0.108496	Syntenin-1	3	P:GO:0007154; P:GO:0044763; P:GO:0044700	P:cell communication; P:single-organism cellular process; P:single organism signaling	
Acrdi.XM_015913067.1/1-2391 | Monca.augustus.g25043.t1/1-3339	0.111453	0.108494	DNA damage-binding 1	10	P:GO:0043161; C:GO:0005737; C:GO:0031464; C:GO:0031981;	P:proteasome-mediated ubiquitin-dependent protein catabolic process; C:cytoplasm; C:Cul4A-RING E3 ubiquitin ligase complex; C:nuclear lumen; C:extracellular region part; C:Cul4B-RING E3 ubiquitin ligase complex; P:nucleotide-excision repair; P:posit[illegible]	
Acrdi.XM_015906520.1/1-2634 | Monca.augustus.g22572.t1/1-2748	0.106044	0.108487	cell fusion related	1	C:GO:0016021	C:integral component of membrane	
Acrdi.XM_015894878.1/1-417 | Monca.augustus.g18776.t1/1-1146	0.087333	0.108477	vacuolar sorting-associated 37A isoform X2	10	C:GO:0005813; F:GO:0005515; P:GO:0075733; P:GO:0043162;	C:centrosome; F:protein binding; P:intracellular transport of virus; P:ubiquitin-dependent protein catabolic process via the multivesicular body sorting pathway; P:autophagy; C:ESCRT I complex; P:endosomal transport; P:multivesicular body assembly; P:v[illegible]	
Acrdi.XM_015905248.1/1-387 | Monca.adi2mcaRNA14862_R2/1-378	0.484472	0.108474	Transposon Tf2-6 poly				
Acrdi.XM_015895718.1/1-1422 | Monca.augustus.g64089.t1/1-1425	0.125	0.108467	Uridine 5 -monophosphate synthase	4	P:GO:0006207; F:GO:0004590; P:GO:0044205; F:GO:0004588	P:'de novo' pyrimidine nucleobase biosynthetic process; F:orotidine-5'-phosphate decarboxylase activity; P:'de novo' UMP biosynthetic process; F:orotate phosphoribosyltransf[illegible]	EC:4.1.1.23; EC:2.4.2.10
Acrdi.XM_015916508.1/1-3051 | Monca.adi2mcaRNA27333_R0/1-3015	0.127197	0.108458	probable ATP-dependent RNA helicase DHX34	4	F:GO:0003676; P:GO:0016070; C:GO:0043231; F:GO:0004386	F:nucleic acid binding; P:RNA metabolic process; C:intracellular membrane-bounded organelle; F:helicase activity	EC:3.6.1.15
Acrdi.XM_015911965.1/1-504 | Monca.augustus.g30042.t1/1-501	0.124419	0.108457	Patatin-like phospholipase domain-containing 2	1	P:GO:0006629	P:lipid metabolic process	
Acrdi.XM_015903693.1/1-3282 | Monca.augustus.g28916.t1/1-5769	0.153407	0.108456	AT-rich interactive domain-containing	2	C:GO:0090544; P:GO:0006338	C:BAF-type complex; P:chromatin remodeling	
Acrdi.XM_015909455.1/1-912 | Monca.augustus.g35588.t1/1-909	0.114142	0.108444	NAD-dependent deacylase sirtuin- mitochondrial	17	F:GO:0008270; P:GO:0036047; F:GO:0061697; F:GO:0036054;	F:zinc ion binding; P:peptidyl-lysine demalonylation; F:protein-glutaryllysine deglutarylase activity; F:protein-malonyllysine demalonylase activity; F:protein-succinyllysine desuccinylase activity; P:protein deacetylation; P:peptidyl-lysine deglutarylation; [illegible]	
Acrdi.XM_015904926.1/1-1431 | Monca.augustus.g55923.t1/1-1608	0.135793	0.108429	SHC SH2 domain-binding 1				
Acrdi.XM_015916465.1/1-1563 | Monca.adi2mcaRNA2735_R0/1-1461	0.123578	0.108407	neuronal acetylcholine receptor subunit alpha-10-like	5	F:GO:0005216; P:GO:0034220; C:GO:0045202; C:GO:0044425;	F:ion channel activity; P:ion transmembrane transport; C:synapse; C:membrane part; C:plasma membrane	
Acrdi.XM_015910210.1/1-3591 | Monca.augustus.g25501.t1/1-4176	0.117033	0.108369	copper-transporting ATPase 1 isoform X2				
Acrdi.XM_015897297.1/1-423 | Monca.augustus.g56886.t1/1-960	0.184613	0.108368	la-related CG11505 isoform X1	1	F:GO:0000166	F:nucleotide binding	
Acrdi.XM_015923339.1/1-1722 | Monca.augustus.g36085.t1/1-1734	0.0856163	0.108366	serine threonine- kinase RIO1-like	3	F:GO:0000166; P:GO:0016310; F:GO:0016301	F:nucleotide binding; P:phosphorylation; F:kinase activity	
Acrdi.XM_015896185.1/1-1314 | Monca.augustus.g33736.t1/1-1413	0.164751	0.108363	cytoplasmic dynein 1 light intermediate chain 2 isoform X1	3	F:GO:0003777; C:GO:0005868; P:GO:0007018	F:microtubule motor activity; C:cytoplasmic dynein complex; P:microtubule-based movement	
Acrdi.XM_015904608.1/1-1836 | Monca.augustus.g31419.t1/1-2202	0.0919403	0.108354	BTB POZ domain-containing 7-like				
Acrdi.XM_015899852.1/1-933 | Monca.augustus.g43428.t1/1-933	0.162518	0.108353	PREDICTED: uncharacterized protein LOC107334876				
Acrdi.XM_015893449.1/1-2853 | Monca.augustus.g36524.t1/1-2850	0.145274	0.108348	GPI ethanolamine phosphate transferase 1	3	C:GO:0005789; F:GO:0051377; P:GO:0016254	C:endoplasmic reticulum membrane; F:mannose-ethanolamine phosphotransferase activity; P:preassembly of GPI anchor in ER membrane	
Acrdi.XM_015911310.1/1-1806 | Monca.augustus.g67450.t1/1-1734	0.141363	0.108327	beta-lactamase-like 1	6	F:GO:0004930; P:GO:0007186; P:GO:0007165; C:GO:0016020;	F:G-protein coupled receptor activity; P:G-protein coupled receptor signaling pathway; P:signal transduction; C:membrane; C:integral component of membrane; F:signal transducer activity	
Acrdi.XM_015908009.1/1-1773 | Monca.augustus.g6857.t1/1-1773	0.154486	0.10832	sodium-dependent multivitamin transporter-like	7	C:GO:0005887; P:GO:0055085; P:GO:0015718; F:GO:0015293;	C:integral component of plasma membrane; P:transmembrane transport; P:monocarboxylic acid transport; F:symporter activity; P:cofactor transport; P:vitamin transport; P:amide transport	
Acrdi.XM_015902841.1/1-1710 | Monca.augustus.g27208.t1/1-1800	0.0972797	0.108319	rho guanine nucleotide exchange factor 39-like	6	P:GO:0035023; F:GO:0005085; F:GO:0005089; P:GO:0035556;	P:regulation of Rho protein signal transduction; F:guanyl-nucleotide exchange factor activity; F:Rho guanyl-nucleotide exchange factor activity; P:intracellular signal transduction; P:positive regulation of GTPase activity; C:intracellular	
Acrdi.XM_015924189.1/1-435 | Monca.augustus.g475.t1/1-1116	0.152816	0.108295	fatty acid hydroxylase domain-containing 2-like	2	C:GO:0016020; P:GO:0044710	C:membrane; P:single-organism metabolic process	
Acrdi.XM_015902479.1/1-903 | Monca.augustus.g36349.t1/1-1047	0.128971	0.108293	IST1 homolog isoform X2	2	C:GO:0044464; P:GO:0009987	C:cell part; P:cellular process	
Acrdi.XM_015911508.1/1-624 | Monca.augustus.g45457.t1/1-1272	0.110034	0.108274	probable sphingosine-1-phosphate phosphatase	1	C:GO:0016021	C:integral component of membrane	
Acrdi.XM_015898372.1/1-1830 | Monca.adi2mcaRNA7416_R2/1-1860	0.431886	0.108244	RNA-directed DNA polymerase from mobile element jockey-like				
Acrdi.XM_015898446.1/1-1314 | Monca.augustus.g41763.t1/1-1617	0.103154	0.108234	5 -nucleotidase domain-containing 3-like	3	F:GO:0008253; F:GO:0046872; P:GO:0016311	F:5'-nucleotidase activity; F:metal ion binding; P:dephosphorylation	EC:3.1.3.41; EC:3.1.3.5; EC:3.1.3.31
Acrdi.XM_015914787.1/1-1986 | Monca.adi2mcaRNA25388_R0/1-2004	0.116151	0.108233	Potassium voltage-gated channel subfamily H member	4	C:GO:0005887; F:GO:0005249; P:GO:0042391; P:GO:0071805	C:integral component of plasma membrane; F:voltage-gated potassium channel activity; P:regulation of membrane potential; P:potassium ion transmembrane transport	
Acrdi.XM_015897791.1/1-1827 | Monca.augustus.g13586.t1/1-945	0.144095	0.108228	Syntaxin-18	1	C:GO:0016020	C:membrane	
Acrdi.XM_015893703.1/1-933 | Monca.adi2mcaRNA39821_R0/1-930	0.0926189	0.108216	estrogen sulfotransferase-like	1	F:GO:0008146	F:sulfotransferase activity	
Acrdi.XM_015902249.1/1-1212 | Monca.augustus.g35085.t1/1-4068	0.114199	0.108201	clustered mitochondria homolog	2	C:GO:0005737; P:GO:0048312	C:cytoplasm; P:intracellular distribution of mitochondria	
Acrdi.XM_015901086.1/1-1107 | Monca.augustus.g57657.t1/1-1113	0.119068	0.108195	histamine H2 receptor-like isoform X1	4	C:GO:0005887; F:GO:0004930; P:GO:0007186; P:GO:0007166	C:integral component of plasma membrane; F:G-protein coupled receptor activity; P:G-protein coupled receptor signaling pathway; P:cell surface receptor signaling pathway	
Acrdi.XM_015904316.1/1-1035 | Monca.augustus.g32333.t1/1-1029	0.161745	0.108172	octopamine receptor 1-like	5	F:GO:0004930; P:GO:0007186; P:GO:0009881; P:GO:0009583;	F:G-protein coupled receptor activity; P:G-protein coupled receptor signaling pathway; F:photoreceptor activity; P:detection of light stimulus; C:integral component of membrane	
Acrdi.XM_015901939.1/1-759 | Monca.augustus.g4668.t1/1-756	0.163864	0.108162	Tctex1 domain-containing 1-B				
Acrdi.XM_015899590.1/1-1581 | Monca.augustus.g21596.t1/1-1569	0.317892	0.108159	Tyrosinase	3	C:GO:0005737; F:GO:0005488; P:GO:0044699	C:cytoplasm; F:binding; P:single-organism process	
Acrdi.XM_015914826.1/1-438 | Monca.augustus.g33249.t1/1-912	0.184164	0.108159	actin depolymerizing factor 3	4	F:GO:0005515; C:GO:0043231; C:GO:0044444; P:GO:0050896	F:protein binding; C:intracellular membrane-bounded organelle; C:cytoplasmic part; P:response to stimulus	
Acrdi.XM_015915287.1/1-1092 | Monca.augustus.g46185.t1/1-1074	0.120894	0.10814	neuropeptide FF receptor 2-like	2	F:GO:0004930; P:GO:0007165	F:G-protein coupled receptor activity; P:signal transduction	
Acrdi.XM_015920696.1/1-408 | Monca.adi2mcaRNA32001_R2/1-369	0.0336437	0.108126	P2X purinoceptor 7-like	3	F:GO:0004888; C:GO:0016021; C:GO:0044464	F:transmembrane signaling receptor activity; C:integral component of membrane; C:cell part	
Acrdi.XM_015909419.1/1-1179 | Monca.augustus.g15950.t1/1-1179	0.385193	0.108102	PREDICTED: uncharacterized protein LOC107343828				
Acrdi.XM_015905019.1/1-693 | Monca.adi2mcaRNA14647_R2/1-840	0.365349	0.108088	PREDICTED: uncharacterized protein LOC107339722				
Acrdi.XM_015910396.1/1-429 | Monca.augustus.g10405.t1/1-2160	0.121871	0.108058	crossover junction endonuclease EME1	6	P:GO:0090305; F:GO:0003677; P:GO:0006281; C:GO:0005634;	P:nucleic acid phosphodiester bond hydrolysis; F:DNA binding; P:DNA repair; C:nucleus; C:Holliday junction resolvase complex; F:nuclease activity	
Acrdi.XM_015892837.1/1-690 | Monca.augustus.g13418.t1/1-1185	0.154765	0.108053	store-operated calcium entry-associated regulatory factor-like	1	C:GO:0016020	C:membrane	
Acrdi.XM_015913703.1/1-7809 | Monca.augustus.g21645.t1/1-8079	0.16034	0.108022	translational activator GCN1				
Acrdi.XM_015902237.1/1-390 | Monca.augustus.g71157.t1/1-468	0.156347	0.107955	calcium and integrin-binding 1-like	1	F:GO:0005509	F:calcium ion binding	
Acrdi.XM_015916362.1/1-1083 | Monca.adi2mcaRNA27174_R0/1-1047	0.165842	0.107936	nucleolar RNA helicase 2-like isoform X2				
Acrdi.XM_015897027.1/1-2202 | Monca.augustus.g48645.t1/1-2673	0.168628	0.107911	eukaryotic translation initiation factor 4 gamma	1	F:GO:0003723	F:RNA binding	
Acrdi.XM_015899507.1/1-1551 | Monca.augustus.g17220.t1/1-1551	0.123128	0.107903	F-box LRR-repeat 2-like				
Acrdi.XM_015916082.1/1-2406 | Monca.augustus.g60497.t1/1-2529	0.115368	0.107891	DNA topoisomerase				
Acrdi.XM_015894790.1/1-762 | Monca.augustus.g67867.t1/1-1041	0.118799	0.10783	POC1 centriolar homolog B-like				
Acrdi.XM_015900330.1/1-903 | Monca.adi2mcaRNA9577_R0/1-873	0.150369	0.107786	oxygen-dependent coproporphyrinogen-III oxidase	3	P:GO:0006779; F:GO:0004109; P:GO:0055114	P:porphyrin-containing compound biosynthetic process; F:coproporphyrinogen oxidase activity; P:oxidation-reduction process	EC:1.3.3.3
Acrdi.XM_015894203.1/1-849 | Monca.augustus.g49636.t1/1-744	0.113642	0.107744	probable palmitoyltransferase ZDHHC24	3	F:GO:0016746; F:GO:0046872; C:GO:0016020	F:transferase activity, transferring acyl groups; F:metal ion binding; C:membrane	
Acrdi.XM_015899995.1/1-2004 | Monca.augustus.g43415.t1/1-2022	0.133741	0.107732	endoplasmic reticulum mannosyl-oligosaccharide 1,2-alpha-mannosid	2	C:GO:0016020; F:GO:0016798	C:membrane; F:hydrolase activity, acting on glycosyl bonds	
Acrdi.XM_015922338.1/1-1524 | Monca.augustus.g13835.t1/1-1482	0.124326	0.107719	transmembrane 161B	2	C:GO:0016020; C:GO:0016021	C:membrane; C:integral component of membrane	
Acrdi.XM_015904605.1/1-2157 | Monca.augustus.g31426.t1/1-10326	0.133873	0.107707	cadherin EGF LAG seven-pass G-type receptor	1	C:GO:0016020	C:membrane	
Acrdi.XM_015908260.1/1-1164 | Monca.augustus.g31833.t1/1-1437	0.138699	0.10769	Glycosaminoglycan xylosylkinase	8	F:GO:0000166; C:GO:0005615; C:GO:0016020; F:GO:0043167;	F:nucleotide binding; C:extracellular space; C:membrane; F:ion binding; C:Golgi apparatus; F:phosphotransferase activity, alcohol group as acceptor; P:positive regulation of protein targeting to mitochondrion; C:nucleoplasm	
Acrdi.XM_015918540.1/1-1275 | Monca.adi2mcaRNA29587_R0/1-1248	0.156712	0.107648	calcium-transporting ATPase type 2C member 1-like	9	F:GO:0000166; C:GO:0005887; F:GO:0015410; P:GO:0098662;	F:nucleotide binding; C:integral component of plasma membrane; F:manganese-transporting ATPase activity; P:inorganic cation transmembrane transport; P:calcium ion tran[illegible]	EC:3.6.3.35; EC:3.6.1.3; EC:3.6.3.8; EC:3.6.1.15
Acrdi.XM_015896752.1/1-471 | Monca.augustus.g42422.t1/1-504	0.133351	0.107638	transcription factor 15-like				
Acrdi.XM_015921694.1/1-1521 | Monca.augustus.g6852.t1/1-2535	0.135907	0.107637	Fibroblast growth factor receptor 3	2	F:GO:0004713; P:GO:0006468	F:protein tyrosine kinase activity; P:protein phosphorylation	EC:2.7.10
Acrdi.XM_015910695.1/1-1032 | Monca.augustus.g35879.t1/1-1038	0.133395	0.107632	Cyclopropane-fatty-acyl-phospholipid synthase	6	C:GO:0043231; C:GO:0012505; C:GO:0044444; F:GO:0008757;	C:intracellular membrane-bounded organelle; C:endomembrane system; C:cytoplasmic part; F:S-adenosylmethionine-dependent methyltransferase activity; C:intracellular organelle part; P:methylation	
Acrdi.XM_015924649.1/1-2403 | Monca.augustus.g50797.t1/1-2436	0.123618	0.107623	von Willebrand factor type EGF and pentraxin domain-containing 1	3	F:GO:0005509; C:GO:0016020; C:GO:0016021	F:calcium ion binding; C:membrane; C:integral component of membrane	
Acrdi.XM_015907323.1/1-801 | Monca.augustus.g32293.t1/1-2163	0.205473	0.107607	Origin recognition complex subunit 3	3	F:GO:0003677; P:GO:0006260; C:GO:0005664	F:DNA binding; P:DNA replication; C:nuclear origin of replication recognition complex	
Acrdi.XM_015897266.1/1-1926 | Monca.adi2mcaRNA6200_R0/1-1809	0.162498	0.107606	huntingtin-interacting 1-like	3	F:GO:0003779; F:GO:0030276; P:GO:0006897	F:actin binding; F:clathrin binding; P:endocytosis	
Acrdi.XM_015907338.1/1-1530 | Monca.augustus.g31791.t1/1-1773	0.0963777	0.107597	PREDICTED: uncharacterized protein LOC107341862				
Acrdi.XM_015909835.1/1-951 | Monca.augustus.g47615.t1/1-948	0.139152	0.107596	inosine-uridine preferring nucleoside hydrolase-like	3	P:GO:0006152; C:GO:0005829; F:GO:0008477	P:purine nucleoside catabolic process; C:cytosol; F:purine nucleosidase activity	
Acrdi.XM_015924679.1/1-1104 | Monca.augustus.g2599.t1/1-1500	0.142309	0.107581	nuclear autoantigenic sperm isoform X3	7	C:GO:0000790; C:GO:0043234; P:GO:0006336; F:GO:0032403;	C:nuclear chromatin; C:protein complex; P:DNA replication-independent nucleosome assembly; F:protein complex binding; P:nucleosome assembly; C:nucleoplasm; P:DNA replication-dependent nucleosome assembly	

Acrdi.XM_015900156.1/1-1935	Monca.augustus.g10474.t1/1-1881	0.149675	0.103428	ATP-dependent DNA helicase PIF1	7	[illegible]
Acrdi.XM_015916045.1/1-474	Monca.augustus.g5233.t1/1-747	0.17991	0.103425	CDK2-associated and cullin domain-containing	10	[illegible]
Acrdi.XM_015914823.1/1-300	Monca.augustus.g44208.t1/1-363	0.447808	0.103399	PREDICTED: uncharacterized protein LOC107348758		
Acrdi.XM_015906346.1/1-846	Monca.augustus.g45338.t1/1-4041	0.155403	0.103395	Early endosome antigen 1		
Acrdi.XM_015921429.1/1-585	Monca.augustus.g23822.t1/1-606	0.117545	0.103365	26S proteasome non-ATPase regulatory subunit 9	4	[illegible]
Acrdi.XM_015913615.1/1-954	Monca.augustus.g48696.t1/1-1704	0.132746	0.103356	Glutamine-rich 2	2	[illegible]
Acrdi.XM_015914040.1/1-3156	Monca.augustus.g68898.t1/1-3222	0.125026	0.103334	serine threonine- kinase LATS1 isoform X1	21	[illegible]
Acrdi.XM_015918879.1/1-1686	Monca.adi2mcaRNA5_R0/1-1614	0.0959433	0.103331	EGF-like repeat and discoidin I-like domain-containing 3	1	[illegible]
Acrdi.XM_015899676.1/1-879	Monca.augustus.g4246.t1/1-1212	0.109838	0.103314	Transmembrane prolyl 4-hydroxylase	5	[illegible]
Acrdi.XM_015913431.1/1-723	Monca.augustus.g32116.t1/1-717	0.107236	0.103302	Hepatic leukemia factor	3	[illegible]
Acrdi.XM_015916729.1/1-1038	Monca.augustus.g37081.t1/1-1032	0.133423	0.103291	serine threonine- kinase mos-like	6	[illegible]
Acrdi.XM_015923680.1/1-2169	Monca.augustus.g34972.t1/1-2379	0.107477	0.103253	RNA-binding 25		
Acrdi.XM_015897654.1/1-1908	Monca.augustus.g56095.t1/1-1656	0.117456	0.103234	far upstream element-binding 1-like	2	[illegible]
Acrdi.XM_015899348.1/1-2448	Monca.augustus.g39838.t1/1-4860	0.140991	0.103225	DNA replication ATP-dependent helicase nuclease DNA2 isoform X2	6	[illegible]
Acrdi.XM_015919248.1/1-555	Monca.augustus.g61202.t1/1-717	0.273781	0.103216	arachidonate 5-lipoxygenase-like	4	[illegible]
Acrdi.XM_015899877.1/1-1149	Monca.adi2mcaRNA9007_R0/1-1005	0.135593	0.103213	P2X receptor	6	[illegible]
Acrdi.XM_015911424.1/1-2379	Monca.augustus.g69032.t1/1-2388	0.150049	0.103177	E3 ubiquitin- ligase TRIM71-like	3	[illegible]
Acrdi.XM_015902889.1/1-2355	Monca.augustus.g27534.t1/1-2355	0.126281	0.103172	Amiloride-sensitive amine oxidase [copper-containing]	1	[illegible]
Acrdi.XM_015915170.1/1-1461	Monca.augustus.g29740.t1/1-2073	0.122121	0.103171	BTB POZ domain-containing 16-like		
Acrdi.XM_015908230.1/1-858	Monca.augustus.g25683.t1/1-858	0.111389	0.103166	somatomedin-B and thrombospondin type-1 domain-containing -like i	4	[illegible]
Acrdi.XM_015913267.1/1-687	Monca.augustus.g18798.t1/1-678	0.189036	0.103152	calcyclin-binding		
Acrdi.XM_015911195.1/1-678	Monca.augustus.g11062.t1/1-702	0.125991	0.103138	UPF0547 C16orf87 homolog	3	[illegible]
Acrdi.XM_015922231.1/1-2868	Monca.augustus.g65974.t1/1-2850	0.152065	0.103137	ankyrin repeat	2	[illegible]
Acrdi.XM_015897968.1/1-702	Monca.adi2mcaRNA6994_R2/1-642	0.936261	0.10313	PREDICTED: uncharacterized protein LOC107333200		
Acrdi.XM_015893246.1/1-333	Monca.augustus.g48117.t1/1-1641	0.0989142	0.103119	prolyl 4-hydroxylase subunit alpha-2 isoform X1	8	[illegible]
Acrdi.XM_015911432.1/1-612	Monca.augustus.g13220.t1/1-627	0.18488	0.103111	tumor p53-inducible nuclear 2 isoform X3	9	[illegible]
Acrdi.XM_015916370.1/1-1758	Monca.augustus.g10884.t1/1-3087	0.07233	0.103107	Inactive ubiquitin carboxyl-terminal hydrolase 54	2	[illegible]
Acrdi.XM_015895641.1/1-777	Monca.augustus.g35435.t1/1-774	0.107289	0.1031	type I iodothyronine deiodinase-like	1	[illegible]
Acrdi.XM_015915872.1/1-2040	Monca.augustus.g39022.t1/1-1998	0.103865	0.10308	ATP-binding cassette sub-family D member 2	4	[illegible]
Acrdi.XM_015917460.1/1-930	Monca.augustus.g51654.t1/1-1467	0.192265	0.103066	BUB3-interacting and GLEBS motif-containing ZNF207 isoform X2		
Acrdi.XM_015908849.1/1-1059	Monca.augustus.g8657.t1/1-1050	0.161478	0.103044	PRRC1	1	[illegible]
Acrdi.XM_015896519.1/1-2589	Monca.augustus.g62596.t1/1-3387	0.140552	0.10304	Ankyrin repeat and MYND domain-containing 1		
Acrdi.XM_015909824.1/1-1989	Monca.augustus.g40679.t1/1-2070	0.116202	0.10303	soluble guanylate cyclase 88E-like		
Acrdi.XM_015905654.1/1-936	Monca.augustus.g71388.t1/1-1245	0.137307	0.10301	alpha-L-fucosidase 1	3	[illegible]
Acrdi.XM_015903347.1/1-513	Monca.augustus.g36025.t1/1-537	0.0989411	0.103007	spore coat -like		
Acrdi.XM_015897454.1/1-3768	Monca.adi2mcaRNA6444_R0/1-3315	0.474631	0.102992	Calcium-responsive transcription factor		
Acrdi.XM_015906615.1/1-987	Monca.augustus.g70216.t1/1-981	0.124351	0.102916	serine threonine- kinase mos-like	6	[illegible]
Acrdi.XM_015912628.1/1-4194	Monca.augustus.g53952.t1/1-4752	0.117618	0.102869	DNA (cytosine-5)-methyltransferase 1	8	[illegible]
Acrdi.XM_015925297.1/1-1167	Monca.adi2mcaRNA37242_R0/1-1224	0.125692	0.102853	Heat shock SSA3	2	[illegible]
Acrdi.XM_015924865.1/1-1509	Monca.adi2mcaRNA3793_R0/1-1404	0.137167	0.102834	phosphatidylinositide phosphatase SAC1	3	[illegible]
Acrdi.XM_015920703.1/1-987	Monca.augustus.g47408.t1/1-990	0.144972	0.102824	mono [ADP-ribose] polymerase PARP16	2	[illegible]
Acrdi.XM_015919125.1/1-984	Monca.adi2mcaRNA30269_R0/1-783	0.152666	0.102724	glycerol-3-phosphate transporter 5	3	[illegible]
Acrdi.XM_015899702.1/1-1503	Monca.augustus.g33796.t1/1-1497	0.110309	0.102696	mitogen-activated kinase 6-like	3	[illegible]
Acrdi.XM_015907086.1/1-4122	Monca.adi2mcaRNA16920_R0/1-4125	0.119542	0.102687	EF-hand calcium-binding domain-containing 6-like	1	[illegible]
Acrdi.XM_015924127.1/1-870	Monca.augustus.g55600.t1/1-861	0.0574628	0.102663	stress response NST1-like		
Acrdi.XM_015899473.1/1-1917	Monca.augustus.g48243.t1/1-2982	0.143387	0.102636	macrophage mannose receptor 1-	3	[illegible]
Acrdi.XM_015919676.1/1-807	Monca.augustus.g67066.t1/1-1677	0.115767	0.102636	proto-oncogene tyrosine- kinase receptor Ret-like		
Acrdi.XM_015913475.1/1-423	Monca.augustus.g2303.t1/1-537	0.100116	0.102632	28 kDa heat- and acid-stable phospho -like		
Acrdi.XM_015921013.1/1-1023	Monca.adi2mcaRNA32322_R0/1-918	0.20345	0.102561	Transposon Ty3-I Gag-Pol poly		
Acrdi.XM_015911558.1/1-750	Monca.augustus.g45427.t1/1-744	0.109129	0.102553	HMG box	1	[illegible]
Acrdi.XM_015913719.1/1-975	Monca.augustus.g21641.t1/1-1545	0.209124	0.102509	lin-7 homolog C		
Acrdi.XM_015895596.1/1-1320	Monca.augustus.g32967.t1/1-1761	0.107116	0.102496	netrin-1	8	[illegible]
Acrdi.XM_015896211.1/1-1005	Monca.augustus.g23045.t1/1-1005	0.135504	0.102479	tartrate-resistant acid phosphatase type 5-like isoform X1	1	[illegible]
Acrdi.XM_015923321.1/1-1323	Monca.augustus.g36086.t1/1-1659	0.0991097	0.102476	ras-specific guanine nucleotide-releasing factor 2 isoform X2	1	[illegible]
Acrdi.XM_015897988.1/1-2556	Monca.augustus.g30588.t1/1-2772	0.119767	0.102474	PDZ domain-containing 7		
Acrdi.XM_015897138.1/1-2730	Monca.adi2mcaRNA652_R0/1-2721	0.13217	0.102471	strawberry notch homolog 1-like	1	[illegible]
Acrdi.XM_015907188.1/1-1272	Monca.augustus.g28174.t1/1-1362	0.164558	0.102451	tRNA (guanine(10)-N2)-methyltransferase homolog	3	[illegible]
Acrdi.XM_015910989.1/1-1557	Monca.augustus.g63043.t1/1-1551	0.134228	0.10245	dimethylaniline monooxygenase [N-oxide-forming] 5-like	7	[illegible]
Acrdi.XM_015900761.1/1-471	Monca.augustus.g5462.t1/1-486	0.0949522	0.102447	PREDICTED: uncharacterized protein LOC107335726		
Acrdi.XM_015900931.1/1-1293	Monca.augustus.g27891.t1/1-1266	0.123636	0.102441	TNF receptor-associated factor 4-like	2	[illegible]
Acrdi.XM_015910079.1/1-7215	Monca.adi2mcaRNA1995_R0/1-7029	0.435066	0.102439	sterile alpha motif domain-containing 9-like		
Acrdi.XM_015923102.1/1-1116	Monca.augustus.g53928.t1/1-531	0.162777	0.102437	Transposon Tf2-6 poly		
Acrdi.XM_015905454.1/1-552	Monca.augustus.g4899.t1/1-1464	0.143635	0.102418	sodium-dependent glucose transporter 1	3	[illegible]
Acrdi.XM_015895585.1/1-5250	Monca.augustus.g35452.t1/1-5880	0.139064	0.102418	von Willebrand factor A domain-containing 3B		
Acrdi.XM_015893231.1/1-3828	Monca.augustus.g9960.t1/1-5301	0.0796168	0.102398	kinesin KIF13A	6	[illegible]
Acrdi.XM_015894691.1/1-1311	Monca.augustus.g23461.t1/1-1908	0.162462	0.102396	HAUS augmin-like complex subunit 6	2	[illegible]
Acrdi.XM_015907362.1/1-2661	Monca.augustus.g35202.t1/1-5361	0.122656	0.10239	laminin subunit beta-2	3	[illegible]
Acrdi.XM_015898207.1/1-438	Monca.augustus.g32111.t1/1-435	0.0864021	0.102383	phosphatase 1 regulatory subunit 27	4	[illegible]
Acrdi.XM_015922808.1/1-2085	Monca.augustus.g367.t1/1-2118	0.102361	0.102372	potassium voltage-gated channel Shaw-like	3	[illegible]
Acrdi.XM_015902959.1/1-1395	Monca.augustus.g47587.t1/1-1545	0.203121	0.102348	programmed cell death 4-like		
Acrdi.XM_015893254.1/1-1161	Monca.augustus.g13506.t1/1-1122	0.112924	0.102336	bifunctional D-cysteine desulfhydrase 1-aminocyclopropane-1-carboxyl	2	[illegible]
Acrdi.XM_015903718.1/1-468	Monca.augustus.g41333.t1/1-393	0.173609	0.102306	Proteasome subunit beta type-6	5	[illegible]
Acrdi.XM_015905170.1/1-831	Monca.augustus.g52823.t1/1-1032	0.134764	0.102266	WD repeat domain phosphoinositide-interacting 4	9	[illegible]
Acrdi.XM_015922405.1/1-726	Monca.augustus.g31905.t1/1-1500	0.126854	0.102221	Organic cation transporter	2	[illegible]
Acrdi.XM_015915816.1/1-801	Monca.augustus.g41824.t1/1-4329	0.1426	0.102193	centromere J-like isoform X1	13	[illegible]
Acrdi.XM_015916331.1/1-1611	Monca.augustus.g64074.t1/1-1605	0.101765	0.102173	Tripartite motif-containing 2	2	[illegible]
Acrdi.XM_015907469.1/1-1530	Monca.augustus.g17450.t1/1-1806	0.115047	0.102132	myotubularin-related 14 isoform X2	4	[illegible]
Acrdi.XM_015902961.1/1-2766	Monca.augustus.g47567.t1/1-3984	0.126918	0.10213	Fanconi anemia group I	3	[illegible]
Acrdi.XM_015914565.1/1-969	Monca.augustus.g29133.t1/1-969	0.118113	0.102119	Forkhead box	5	[illegible]
Acrdi.XM_015901699.1/1-1647	Monca.augustus.g54268.t1/1-2361	0.115254	0.102112	triple functional domain -like isoform X3	3	[illegible]
Acrdi.XM_015909999.1/1-945	Monca.augustus.g56280.t1/1-942	0.135921	0.102104	inosine-uridine preferring nucleoside hydrolase-like	3	[illegible]
Acrdi.XM_015898097.1/1-2526	Monca.augustus.g9830.t1/1-2262	0.129233	0.102093	rho GTPase-activating 39 isoform X2		
Acrdi.XM_015906988.1/1-498	Monca.augustus.g33368.t1/1-1461	0.0959331	0.102086	SH3 domain-binding 2 isoform X1		
Acrdi.XM_015925301.1/1-732	Monca.augustus.g54928.t1/1-732	0.120069	0.102067	39S ribosomal mitochondrial	2	[illegible]
Acrdi.XM_015909733.1/1-495	Monca.augustus.g2269.t1/1-495	0.164217	0.102051	probable 8-oxo-dGTP diphosphatase NUDT15	3	[illegible]
Acrdi.XM_015914640.1/1-939	Monca.adi2mcaRNA25230_R0/1-1083	0.320329	0.102035	Carboxypeptidase D		
Acrdi.XM_015922406.1/1-978	Monca.augustus.g15718.t1/1-1242	0.103339	0.102034	group XV phospholipase A2-like	2	[illegible]
Acrdi.XM_015914003.1/1-3624	Monca.adi2mcaRNA24510_R0/1-3426	0.107004	0.102026	lipoxygenase homology domain-containing 1-like	1	[illegible]
Acrdi.XM_015911599.1/1-519	Monca.augustus.g24291.t1/1-732	0.130833	0.10202	gamma-secretase subunit Aph-1	3	[illegible]
Acrdi.XM_015917813.1/1-1203	Monca.augustus.g69004.t1/1-1221	0.148191	0.102007	DNA repair RAD51 homolog 3 isoform X1	17	[illegible]
Acrdi.XM_015897135.1/1-1326	Monca.adi2mcaRNA6035_R1/1-1164	0.278387	0.102001	zinc finger 862-like		
Acrdi.XM_015897283.1/1-1032	Monca.adi2mcaRNA6253_R0/1-948	0.16665	0.101998	synaptonemal complex 2-like		
Acrdi.XM_015906534.1/1-2688	Monca.adi2mcaRNA16314_R0/1-2328	0.121151	0.101977	probable cation-transporting ATPase 13A3 isoform X1	4	[illegible]
Acrdi.XM_015904967.1/1-516	Monca.adi2mcaRNA14527_R0/1-525	0.119774	0.101963	Translocon-associated subunit delta	2	[illegible]
Acrdi.XM_015896764.1/1-2046	Monca.augustus.g14392.t1/1-1995	0.123754	0.101961	probable cysteine desulfurase	1	[illegible]
Acrdi.XM_015904639.1/1-1374	Monca.adi2mcaRNA14160_R0/1-1377	0.0728605	0.101952	macrophage mannose receptor 1-like	3	[illegible]
Acrdi.XM_015925096.1/1-1506	Monca.adi2mcaRNA36994_R0/1-1491	0.155475	0.101938	Synaptic vesicle 2-related	2	[illegible]
Acrdi.XM_015921050.1/1-1194	Monca.adi2mcaRNA32366_R0/1-1212	0.432086	0.101934	ATP-dependent DNA helicase Q1		
Acrdi.XM_015910828.1/1-1782	Monca.augustus.g57314.t1/1-1851	0.156322	0.101934	neuronal PAS domain-containing 2 isoform X1	8	[illegible]
Acrdi.XM_015912560.1/1-1008	Monca.adi2mcaRNA22934_R1/1-861	0.176288	0.101924	Polycystic kidney disease 1-like 3		
Acrdi.XM_015908341.1/1-708	Monca.augustus.g36826.t1/1-993	0.124679	0.101901	dnaJ homolog subfamily C member 25 homolog	1	[illegible]
Acrdi.XM_015902731.1/1-408	Monca.augustus.g33264.t1/1-408	0.0985578	0.101881	SAY domain-containing 1		
Acrdi.XM_015906918.1/1-3099	Monca.augustus.g49135.t1/1-3984	0.10478	0.101878	membrane-associated phosphatidylinositol transfer 2-like isoform X1	3	[illegible]
Acrdi.XM_015919836.1/1-1191	Monca.adi2mcaRNA31057_R1/1-1125	0.263503	0.101856	RNA-directed DNA polymerase from mobile element jockey-like		
Acrdi.XM_015912963.1/1-1857	Monca.augustus.g64763.t1/1-1989	0.101013	0.10185	cilia- and flagella-associated 99-like		
Acrdi.XM_015908068.1/1-1632	Monca.augustus.g17404.t1/1-1557	0.135375	0.101845	Apocarotenoid-15,15 -oxygenase	2	[illegible]
Acrdi.XM_015901100.1/1-1641	Monca.augustus.g35508.t1/1-1632	0.112345	0.101818	Band 5	4	[illegible]
Acrdi.XM_015898286.1/1-2856	Monca.augustus.g28197.t1/1-2844	0.163805	0.101787	ataxin-7 1 isoform X7		
Acrdi.XM_015916396.1/1-1122	Monca.augustus.g40963.t1/1-2007	0.0891584	0.101777	tyrosine- kinase transmembrane receptor Ror-like	3	[illegible]
Acrdi.XM_015897545.1/1-555	Monca.augustus.g22834.t1/1-558	0.09281	0.101744	transcription initiation factor TFIID subunit	6	[illegible]
Acrdi.XM_015892073.1/1-402	Monca.augustus.g1133.t1/1-1236	0.110389	0.101712	lanC 3		
Acrdi.XM_015893922.1/1-1317	Monca.augustus.g62473.t1/1-1317	0.117785	0.101669	neuronal acetylcholine receptor subunit alpha-7-like	2	[illegible]
Acrdi.XM_015908314.1/1-555	Monca.augustus.g34630.t1/1-507	0.109446	0.101667	17-beta-hydroxysteroid dehydrogenase 14	5	[illegible]
Acrdi.XM_015914043.1/1-534	Monca.augustus.g768.t1/1-609	0.15239	0.101661	NADH dehydrogenase [ubiquinone] 1 alpha subcomplex assembly fact	6	[illegible]
Acrdi.XM_015919507.1/1-675	Monca.augustus.g3344.t1/1-639	0.124605	0.101658	maleylacetoacetate isomerase isoform X1	7	[illegible]
Acrdi.XM_015897193.1/1-3096	Monca.augustus.g39424.t1/1-3783	0.137705	0.10164	tripeptidyl-peptidase 2 isoform X2		
Acrdi.XM_015918438.1/1-1218	Monca.augustus.g55063.t1/1-1377	0.108268	0.101619	rho GTPase-activating 24 isoform X1	1	[illegible]
Acrdi.XM_015909000.1/1-573	Monca.augustus.g3494.t1/1-849	0.160701	0.101612	Glutamate--cysteine ligase regulatory subunit	6	[illegible]
Acrdi.XM_015897542.1/1-420	Monca.augustus.g22827.t1/1-420	0.129803	0.101608	PREDICTED: uncharacterized protein LOC107332797		
Acrdi.XM_015907714.1/1-1674	Monca.augustus.g55840.t1/1-1215	0.123376	0.101595	E3 ubiquitin- ligase TRIM71-like	1	[illegible]
Acrdi.XM_015904746.1/1-1188	Monca.augustus.g15780.t1/1-1626	0.170596	0.101573	poly(A)-specific ribonuclease PARN-like domain-containing 1	2	[illegible]
Acrdi.XM_015904310.1/1-1182	Monca.augustus.g61779.t1/1-1278	0.106444	0.101561	AP-4 complex subunit mu-like	1	[illegible]
Acrdi.XM_015893097.1/1-1344	Monca.adi2mcaRNA39141_R1/1-1269	0.704524	0.101559	PREDICTED: uncharacterized protein LOC107328355		
Acrdi.XM_015901090.1/1-627	Monca.augustus.g25251.t1/1-657	0.117762	0.101541	Lysosomal alpha-mannosidase	6	[illegible]
Acrdi.XM_015906745.1/1-966	Monca.augustus.g3895.t1/1-966	0.127891	0.10153	WD40 repeat	6	[illegible]
Acrdi.XM_015897272.1/1-1077	Monca.augustus.g48406.t1/1-1038	0.148354	0.101524	transmembrane 120A-like	2	[illegible]
Acrdi.XM_015906356.1/1-2853	Monca.augustus.g45319.t1/1-9309	0.127024	0.101524	lysine-specific demethylase 7A-like	2	[illegible]
Acrdi.XM_015918821.1/1-1164	Monca.augustus.g22486.t1/1-1488	0.161104	0.10148	uncharacterized aarF domain-containing kinase 1 isoform X1	4	[illegible]
Acrdi.XM_015912131.1/1-1320	Monca.augustus.g60700.t1/1-1317	0.0882707	0.101467	serine threonine- kinase S6KL-like	2	[illegible]
Acrdi.XM_015920395.1/1-1947	Monca.augustus.g46006.t1/1-2043	0.11087	0.101466	Carboxypeptidase D	7	[illegible]
Acrdi.XM_015896269.1/1-1203	Monca.augustus.g33713.t1/1-1182	0.128704	0.101453	Peptidase M20 domain-containing 2	2	[illegible]
Acrdi.XM_015908774.1/1-1047	Monca.augustus.g1464.t1/1-1065	0.101524	0.101413	Neuropeptide FF receptor 1	4	[illegible]
Acrdi.XM_015895667.1/1-1338	Monca.augustus.g60355.t1/1-1338	0.141581	0.101363	Gamma-butyrobetaine dioxygenase	6	[illegible]
Acrdi.XM_015915475.1/1-762	Monca.augustus.g60465.t1/1-774	0.155136	0.101343	ES1 mitochondrial	1	[illegible]
Acrdi.XM_015914021.1/1-792	Monca.augustus.g18745.t1/1-795	0.10585	0.101318	40S ribosomal X isoform	13	[illegible]
Acrdi.XM_015901296.1/1-996	Monca.augustus.g43362.t1/1-1017	0.116827	0.101256	histamine H2 receptor-like	5	[illegible]
Acrdi.XM_015920563.1/1-759	Monca.augustus.g35472.t1/1-858	0.0941963	0.101245	Ribonuclease H1	2	[illegible]
Acrdi.XM_015895315.1/1-921	Monca.augustus.g56110.t1/1-1503	0.0760073	0.101239	general transcription factor 3C polypeptide 5	2	[illegible]
Acrdi.XM_015911797.1/1-6789	Monca.augustus.g29296.t1/1-6912	0.139603	0.101226	integrator complex subunit 1 isoform X1		
Acrdi.XM_015914903.1/1-1155	Monca.augustus.g39542.t1/1-1119	0.147066	0.101212	bone morphogenetic 2-like	11	[illegible]
Acrdi.XM_015901727.1/1-951	Monca.augustus.g34401.t1/1-954	0.165122	0.101208	transcription factor AP-1-like	10	[illegible]
Acrdi.XM_015914035.1/1-1233	Monca.augustus.g60918.t1/1-2361	0.176928	0.101205	NADH-cytochrome b5 reductase 1-like	9	[illegible]
Acrdi.XM_015892876.1/1-1017	Monca.adi2mcaRNA38903_R0/1-1005	0.120903	0.101189	cathepsin L1-like	1	[illegible]
Acrdi.XM_015914126.1/1-633	Monca.augustus.g60916.t1/1-621	0.0589917	0.101185	Calcyphosin	1	[illegible]
Acrdi.XM_015909481.1/1-441	Monca.augustus.g2687.t1/1-441	0.120836	0.101174	transmembrane 42-like		
Acrdi.XM_015910573.1/1-1032	Monca.augustus.g10112.t1/1-1029	0.111368	0.101156	orexin receptor type 1-like isoform X1	3	[illegible]
Acrdi.XM_015923175.1/1-4032	Monca.augustus.g30542.t1/1-4518	0.137428	0.101118	trafficking particle complex subunit 8 isoform X2	8	[illegible]
Acrdi.XM_015901067.1/1-1323	Monca.augustus.g57634.t1/1-1329	0.0854164	0.101107	GDP-fucose O-fucosyltransferase 2	2	[illegible]
Acrdi.XM_015900214.1/1-984	Monca.augustus.g62861.t1/1-984	0.139412	0.101025	UDP-xylose and UDP-N-acetylglucosamine transporter	8	[illegible]
Acrdi.XM_015923533.1/1-369	Monca.augustus.g30556.t1/1-1563	0.115004	0.101024	Something about silencing 10	1	[illegible]
Acrdi.XM_015895294.1/1-1047	Monca.augustus.g6868.t1/1-1599	0.125645	0.100976	arf-GAP with Rho-GAP ANK repeat and PH domain-containing 1-like	3	[illegible]
Acrdi.XM_015893050.1/1-531	Monca.augustus.g35718.t1/1-372	0.371491	0.100975	THAP domain-containing 10-like	1	[illegible]
Acrdi.XM_015893282.1/1-2664	Monca.augustus.g67445.t1/1-2676	0.125983	0.10095	inturned	11	[illegible]
Acrdi.XM_015918502.1/1-1116	Monca.augustus.g48103.t1/1-1275	0.0926432	0.100939	DNA primase small subunit-like	3	[illegible]
Acrdi.XM_015903184.1/1-1188	Monca.augustus.g52474.t1/1-1092	0.17543	0.10093	adenosine deaminase isoform X1	6	[illegible]
Acrdi.XM_015898151.1/1-1266	Monca.augustus.g39428.t1/1-1236	0.19445	0.100916	Integral membrane GPR180	4	[illegible]
Acrdi.XM_015902529.1/1-897	Monca.augustus.g64945.t1/1-1077	0.102415	0.100867	L-lactate dehydrogenase-like	2	[illegible]
Acrdi.XM_015907496.1/1-1146	Monca.augustus.g35187.t1/1-1149	0.125196	0.100849	cAMP-dependent kinase type II-alpha regulatory subunit	4	[illegible]
Acrdi.XM_015901845.1/1-2586	Monca.adi2mcaRNA11212_R0/1-2574	0.10355	0.100843	adenylate cyclase type 3 isoform X2		
Acrdi.XM_015919408.1/1-942	Monca.augustus.g27301.t1/1-942	0.101168	0.100842	BRISC and BRCA1-A complex member 1-like	3	[illegible]
Acrdi.XM_015923282.1/1-522	Monca.augustus.g55711.t1/1-1545	0.113945	0.10082	AN1-type zinc finger 4-like	1	[illegible]
Acrdi.XM_015908109.1/1-1290	Monca.adi2mcaRNA18011_R1/1-1152	0.255931	0.100788	PREDICTED: uncharacterized protein LOC107342607		
Acrdi.XM_015897129.1/1-537	Monca.augustus.g21047.t1/1-657	0.121831	0.100772	strawberry notch homolog 1-like	1	[illegible]
Acrdi.XM_015911019.1/1-6168	Monca.augustus.g3795.t1/1-8082	0.130302	0.10076	unconventional myosin-XV isoform X2		
Acrdi.XM_015902758.1/1-1122	Monca.augustus.g5672.t1/1-1128	0.0955801	0.100741	Alpha-1A adrenergic receptor		
Acrdi.XM_015909348.1/1-5334	Monca.adi2mcaRNA1989_R1/1-5502	0.090436	0.100729	adenylate kinase 9		
Acrdi.XM_015910040.1/1-1266	Monca.augustus.g38590.t1/1-1287	0.0932009	0.100716	TNF receptor-associated factor 5	6	[illegible]
Acrdi.XM_015921192.1/1-1323	Monca.augustus.g51414.t1/1-1428	0.171626	0.100715	Zinc transporter 6	6	[illegible]
Acrdi.XM_015921322.1/1-549	Monca.augustus.g16009.t1/1-555	0.0724466	0.100713	histone -B	4	[illegible]
Acrdi.XM_015902773.1/1-1776	Monca.augustus.g65485.t1/1-1722	0.140411	0.100657	Lactation elevated	1	[illegible]
Acrdi.XM_015921190.1/1-1869	Monca.adi2mcaRNA32532_R0/1-1572	0.0954013	0.100657	Integrator complex subunit 11	3	[illegible]
Acrdi.XM_015915477.1/1-1287	Monca.augustus.g32785.t1/1-1449	0.126662	0.100624	cyclic AMP-responsive element-binding 3 2 isoform X2	4	[illegible]
Acrdi.XM_015903652.1/1-1026	Monca.augustus.g5007.t1/1-1029	0.12872	0.100592	octopamine receptor 1-like	6	[illegible]
Acrdi.XM_015899835.1/1-1314	Monca.augustus.g52524.t1/1-1311	0.0952506	0.100584	synaptotagmin-16-like isoform X2	8	[illegible]
Acrdi.XM_015918625.1/1-825	Monca.augustus.g7621.t1/1-948	0.0914028	0.100565	GTP:AMP phosphotransferase mitochondrial	11	[illegible]
Acrdi.XM_015905653.1/1-774	Monca.adi2mcaRNA15315_R1/1-801	0.169424	0.10052	unc-80 homolog	2	[illegible]
Acrdi.XM_015899360.1/1-1245	Monca.adi2mcaRNA1019_R0/1-1074	0.144693	0.100507	Laccase domain-containing 1		
Acrdi.XM_015914439.1/1-564	Monca.augustus.g56325.t1/1-924	0.12841	0.100507	RTF2 homolog		
Acrdi.XM_015892386.1/1-4743	Monca.adi2mcaRNA38316_R0/1-4692	0.109981	0.100504	voltage-gated sodium channel	6	[illegible]
Acrdi.XM_015919112.1/1-1422	Monca.augustus.g35161.t1/1-1803	0.0971434	0.100503	SRSF kinase	16	[illegible]
Acrdi.XM_015909343.1/1-375	Monca.augustus.g13083.t1/1-375	0.117925	0.10049	14 kDa phosphohistidine phosphatase-like	3	[illegible]
Acrdi.XM_015925370.1/1-636	Monca.augustus.g41124.t1/1-645	0.134088	0.100488	Heme-binding 2	2	[illegible]
Acrdi.XM_015922958.1/1-8832	Monca.adi2mcaRNA34558_R0/1-8196	0.116079	0.100449	dynein heavy chain axonemal-like	8	[illegible]
Acrdi.XM_015918298.1/1-813	Monca.augustus.g29409.t1/1-942	0.101541	0.100446	Glycine N-methyltransferase	2	[illegible]
Acrdi.XM_015918351.1/1-1137	Monca.augustus.g23509.t1/1-1143	0.119977	0.100438	synaptotagmin-3 isoform X1	12	[illegible]
Acrdi.XM_015902810.1/1-1200	Monca.augustus.g58372.t1/1-1254	0.136023	0.100425	Golgi reassembly-stacking 2-like		
Acrdi.XM_015921847.1/1-1089	Monca.augustus.g61095.t1/1-2961	0.16452	0.100392	AP-5 complex subunit beta-1	3	[illegible]
Acrdi.XM_015918381.1/1-1893	Monca.adi2mcaRNA29409_R0/1-1893	0.0967591	0.100386	angiotensin-converting enzyme-like	6	[illegible]
Acrdi.XM_015918330.1/1-564	Monca.adi2mcaRNA29367_R0/1-585	0.137178	0.100385	Kinesin-associated 3	3	[illegible]
Acrdi.XM_015900693.1/1-534	Monca.augustus.g34853.t1/1-648	0.156139	0.100374	Rab	2	[illegible]
Acrdi.XM_015913512.1/1-633	Monca.augustus.g60203.t1/1-1146	0.112836	0.100357	DENN (AEX3) domain containing	3	[illegible]
Acrdi.XM_015924962.1/1-990	Monca.augustus.g40882.t1/1-990	0.0958623	0.100337	adiponectin receptor	3	[illegible]
Acrdi.XM_015893529.1/1-957	Monca.augustus.g46089.t1/1-1026	0.0975693	0.100324	cyclic AMP receptor A	3	[illegible]
Acrdi.XM_015902779.1/1-1260	Monca.augustus.g33292.t1/1-1260	0.116955	0.100236	leucine-rich repeat 1		
Acrdi.XM_015900520.1/1-1188	Monca.augustus.g1369.t1/1-1251	0.127502	0.100235	Chromosome 7 open reading frame 31	1	[illegible]
Acrdi.XM_015910770.1/1-588	Monca.augustus.g29675.t1/1-645	0.181265	0.100228	rRNA-processing FCF1 homolog	1	[illegible]
Acrdi.XM_015921147.1/1-375	Monca.augustus.g14160.t1/1-1185	0.104444	0.100208	PHD finger 24 isoform X2	4	[illegible]
Acrdi.XM_015903720.1/1-2529	Monca.augustus.g32287.t1/1-2538	0.169281	0.100198	PREDICTED: uncharacterized protein LOC107338482		
Acrdi.XM_015921849.1/1-1566	Monca.augustus.g63511.t1/1-1716	0.0994846	0.100174	TBC1 domain family member 25	1	[illegible]
Acrdi.XM_015921951.1/1-1320	Monca.augustus.g40444.t1/1-2955	0.11605	0.100165	LIM domain kinase 1 isoform X2	1	[illegible]
Acrdi.XM_015906979.1/1-603	Monca.adi2mcaRNA16799_R0/1-498	0.11969	0.100158	Transmembrane 17	5	[illegible]
Acrdi.XM_015911651.1/1-753	Monca.augustus.g4713.t1/1-744	0.13622	0.10015	Leucine-rich repeat-containing 61		
Acrdi.XM_015911482.1/1-1017	Monca.augustus.g53635.t1/1-2295	0.131767	0.100136	probable G- coupled receptor CG31760		
Acrdi.XM_015919735.1/1-654	Monca.augustus.g40903.t1/1-663	0.0871863	0.100134	ankyrin repeat domain-containing 49-like		
Acrdi.XM_015901396.1/1-741	Monca.augustus.g31329.t1/1-744	0.278481	0.100129	sprouty homolog 3	1	[illegible]
Acrdi.XM_015921841.1/1-621	Monca.augustus.g16482.t1/1-540	0.0963551	0.100128	PREDICTED: uncharacterized protein LOC107355300		
Acrdi.XM_015911125.1/1-474	Monca.adi2mcaRNA21388_R0/1-594	0.161158	0.100088	tRNA (guanine-N(7)-)-methyltransferase isoform X2	7	[illegible]
Acrdi.XM_015891919.1/1-546	Monca.augustus.g9574.t1/1-546	0.0604559	0.100088	U3 small nucleolar ribonucleo IMP3	9	[illegible]
Acrdi.XM_015913991.1/1-1515	Monca.adi2mcaRNA24504_R0/1-1362	0.708655	0.100065	Mediator of RNA polymerase II transcription subunit 15		
Acrdi.XM_015911564.1/1-933	Monca.augustus.g57760.t1/1-3015	0.170869	0.100052	tudor domain-containing 3 isoform X2	7	[illegible]
Acrdi.XM_015909272.1/1-636	Monca.adi2mcaRNA19332_R0/1-645	0.14933	0.100046	NMDA receptor synaptonuclear signaling and neuronal migration factor-like isoform X2		
Acrdi.XM_015896681.1/1-555	Monca.adi2mcaRNA552_R0/1-900	0.663578	0.10004	PREDICTED: uncharacterized protein LOC107331982		
Acrdi.XM_015904892.1/1-4920	Monca.adi2mcaRNA14457_R1/1-4665	0.2308	0.100036	PREDICTED: uncharacterized protein LOC107339571		
Acrdi.XM_015908678.1/1-1257	Monca.augustus.g53862.t1/1-1494	0.104279	0.100017	U4 tri-snRNP-associated 2	3	[illegible]
Acrdi.XM_015905258.1/1-2637	Monca.augustus.g23173.t1/1-2856	0.127115	0.0999918	ELKS Rab6-interacting CAST family member 1		
Acrdi.XM_015900637.1/1-891	Monca.augustus.g28769.t1/1-864	0.175454	0.0999869	PREDICTED: uncharacterized protein LOC107335610		
Acrdi.XM_015916113.1/1-1005	Monca.augustus.g505.t1/1-1221	0.0769644	0.099986	inhibitor of growth 3 isoform X2	11	[illegible]
Acrdi.XM_015918070.1/1-1860	Monca.augustus.g34642.t1/1-1860	0.140407	0.0999824	F-box LRR-repeat 14	3	[illegible]
Acrdi.XM_015892389.1/1-1755	Monca.augustus.g42233.t1/1-1875	0.123687	0.0999798	tyrosine- kinase Btk29A isoform X3	12	[illegible]
Acrdi.XM_015911117.1/1-1644	Monca.augustus.g7981.t1/1-1644	0.10534	0.0999529	frizzled-1-like	8	[illegible]
Acrdi.XM_015914276.1/1-2736	Monca.augustus.g40752.t1/1-1410	0.131839	0.0999483	myosin-IIIb-like isoform X1	5	[illegible]
Acrdi.XM_015914671.1/1-426	Monca.adi2mcaRNA25270_R0/1-366	0.505055	0.0999422	PREDICTED: uncharacterized protein LOC107348613		
Acrdi.XM_015895177.1/1-726	Monca.augustus.g6314.t1/1-594	0.151364	0.0999113	MEF2BNB isoform X1	1	[illegible]
Acrdi.XM_015900907.1/1-1014	Monca.augustus.g27865.t1/1-1014	0.108155	0.0999102	PREDICTED: uncharacterized protein LOC107335860		
Acrdi.XM_015910726.1/1-492	Monca.augustus.g24314.t1/1-1044	0.088463	0.0998903	XRP2 isoform X1	17	[illegible]
Acrdi.XM_015921397.1/1-1563	Monca.augustus.g31284.t1/1-930	0.25455	0.0998643	PREDICTED: uncharacterized protein LOC107354905		
Acrdi.XM_015902772.1/1-1587	Monca.adi2mcaRNA12131_R0/1-1587	0.132823	0.0998627	tyrosyl-DNA phosphodiesterase 1 isoform X1	3	[illegible]
Acrdi.XM_015903983.1/1-4110	Monca.augustus.g27734.t1/1-6072	0.0929191	0.0998554	myoferlin-like isoform X14	2	[illegible]
Acrdi.XM_015925467.1/1-525	Monca.adi2mcaRNA37444_R0/1-531	0.133938	0.0998551	Tudor domain-containing 5		
Acrdi.XM_015904331.1/1-492	Monca.augustus.g5869.t1/1-1653	0.102757	0.0998299	CLK4-associating serine arginine rich		
Acrdi.XM_015922117.1/1-4650	Monca.augustus.g59540.t1/1-5514	0.129374	0.0998223	proteasome activator complex subunit 4		
Acrdi.XM_015897233.1/1-1332	Monca.augustus.g59971.t1/1-1332	0.135499	0.0998045	gamma-aminobutyric acid receptor alpha-like	4	[illegible]
Acrdi.XM_015909470.1/1-981	Monca.augustus.g5282.t1/1-990	0.12533	0.099782	cathepsin L1-like	4	[illegible]
Acrdi.XM_015917981.1/1-1338	Monca.adi2mcaRNA29020_R0/1-1299	0.0872508	0.0997732	F-box WD repeat-containing 1A isoform X6	2	[illegible]
Acrdi.XM_015894841.1/1-726	Monca.adi2mcaRNA41178_R0/1-801	0.0395458	0.0997237	Glucosidase 2 subunit beta	4	[illegible]
Acrdi.XM_015896264.1/1-1341	Monca.augustus.g33723.t1/1-1350	0.128081	0.0997014	WD repeat and SOCS box-containing 1-like	3	[illegible]
Acrdi.XM_015915814.1/1-474	Monca.adi2mcaRNA26548_R5/1-597	0.389733	0.0996692	nucleoporin GLE1-like		
Acrdi.XM_015901409.1/1-1053	Monca.adi2mcaRNA10676_R0/1-1044	0.121103	0.0996263	CAAX prenyl protease 1 homolog	3	[illegible]
Acrdi.XM_015920416.1/1-990	Monca.augustus.g46004.t1/1-984	0.136829	0.0996244	Krueppel-like factor 7	2	[illegible]
Acrdi.XM_015903538.1/1-1041	Monca.augustus.g44082.t1/1-966	0.0794177	0.0996141	Alcohol dehydrogenase [NADP(+)]	2	[illegible]
Acrdi.XM_015898830.1/1-1398	Monca.adi2mcaRNA782_R1/1-1524	0.256772	0.0996017	PREDICTED: uncharacterized protein LOC107333961		
Acrdi.XM_015911562.1/1-852	Monca.augustus.g24294.t1/1-1041	0.0960992	0.0995964	melanopsin isoform X2	3	[illegible]
Acrdi.XM_015924033.1/1-558	Monca.augustus.g5725.t1/1-567	0.134514	0.0995916	HD domain-containing 2		
Acrdi.XM_015897748.1/1-1314	Monca.augustus.g17411.t1/1-1290	0.13602	0.0995801	T-box-containing TBX6L	5	[illegible]
Acrdi.XM_015891917.1/1-738	Monca.augustus.g6751.t1/1-744	0.132297	0.0995611	Type I iodothyronine deiodinase	2	[illegible]
Acrdi.XM_015903632.1/1-1995	Monca.adi2mcaRNA13066_R0/1-1599	0.114764	0.0995542	Coiled-coil domain-containing 81		
Acrdi.XM_015902602.1/1-2433	Monca.augustus.g48594.t1/1-2433	0.144157	0.0995307	85 88 kDa calcium-independent phospholipase A2 isoform X1	7	[illegible]
Acrdi.XM_015896747.1/1-561	Monca.augustus.g42411.t1/1-492	0.120928	0.0995297	CCA tRNA nucleotidyltransferase mitochondrial-like	4	[illegible]
Acrdi.XM_015904140.1/1-387	Monca.augustus.g32309.t1/1-378	0.146363	0.0995191	phosphatase 1 regulatory subunit 11	4	[illegible]
Acrdi.XM_015920877.1/1-576	Monca.augustus.g30091.t1/1-573	0.0844867	0.0995056	ARL14 effector	1	[illegible]
Acrdi.XM_015901081.1/1-726	Monca.augustus.g57645.t1/1-735	0.139307	0.0994985	14-3-3 beta alpha-like	7	[illegible]
Acrdi.XM_015894467.1/1-672	Monca.augustus.g58753.t1/1-672	0.128483	0.09948	Transmembrane 223	3	[illegible]
Acrdi.XM_015913810.1/1-330	Monca.augustus.g57176.t1/1-540	0.104763	0.0994568	ADP-ribosylation factor 3	21	[illegible]
Acrdi.XM_015907529.1/1-1098	Monca.augustus.g55652.t1/1-1239	0.113058	0.0994515	aspartate cytoplasmic-like	6	[illegible]
Acrdi.XM_015925338.1/1-693	Monca.augustus.g66557.t1/1-693	0.14101	0.0994471	PREDICTED: uncharacterized protein LOC107358753		
Acrdi.XM_015907130.1/1-1413	Monca.augustus.g36962.t1/1-1410	0.098205	0.0994419	E3 ubiquitin- ligase MYLIP	5	[illegible]
Acrdi.XM_015905939.1/1-1251	Monca.augustus.g58513.t1/1-1269	0.135107	0.0994406	multidrug resistance		
Acrdi.XM_015909291.1/1-477	Monca.augustus.g35322.t1/1-432	0.121323	0.0994306	15 kDa seleno		
Acrdi.XM_015903186.1/1-1176	Monca.augustus.g71356.t1/1-1182	0.126691	0.0993999	cyclin B	1	[illegible]
Acrdi.XM_015893782.1/1-507	Monca.augustus.g27909.t1/1-513	0.147238	0.0993844	PREDICTED: uncharacterized protein C5orf49 homolog		
Acrdi.XM_015910732.1/1-927	Monca.augustus.g20680.t1/1-1206	0.0990278	0.0993811	Sorting nexin-6	3	[illegible]
Acrdi.XM_015919962.1/1-639	Monca.augustus.g69053.t1/1-708	0.112518	0.0993699	neuralized 2	1	[illegible]
Acrdi.XM_015892991.1/1-918	Monca.augustus.g40909.t1/1-1002	0.0818416	0.0993399	DNA polymerase beta	5	[illegible]
Acrdi.XM_015904542.1/1-1125	Monca.augustus.g52752.t1/1-1107	0.131579	0.0993011	ornithine mitochondrial	5	[illegible]
Acrdi.XM_015903422.1/1-1368	Monca.augustus.g18171.t1/1-1362	0.104009	0.0992757	hect e3 ubiquitin	2	[illegible]
Acrdi.XM_015893475.1/1-1485	Monca.adi2mcaRNA39562_R4/1-1905	0.14814	0.099253	RNA-directed DNA polymerase from mobile element jockey-like		
Acrdi.XM_015898128.1/1-1281	Monca.augustus.g39421.t1/1-1245	0.101553	0.0992001	potassium voltage-gated channel subfamily A member 1	5	[illegible]
Acrdi.XM_015909478.1/1-936	Monca.adi2mcaRNA2042_R0/1-939	0.147895	0.0991982	S-adenosylmethionine decarboxylase proenzyme	3	[illegible]
Acrdi.XM_015904322.1/1-2403	Monca.augustus.g59952.t1/1-2493	0.117308	0.0991971	inactive rhomboid 1 isoform X1	5	[illegible]
Acrdi.XM_015901014.1/1-1137	Monca.augustus.g57656.t1/1-1473	0.131861	0.0991804	sodium-coupled neutral amino acid transporter 5-like	3	[illegible]
Acrdi.XM_015910500.1/1-567	Monca.augustus.g25411.t1/1-564	0.116571	0.0991657	60S ribosomal L9	4	[illegible]
Acrdi.XM_015919594.1/1-1914	Monca.adi2mcaRNA30793_R0/1-1710	0.161855	0.0991648	GTPase-activating Rap Ran-GAP domain 3	1	[illegible]
Acrdi.XM_015924716.1/1-2349	Monca.augustus.g46970.t1/1-2064	0.0890138	0.0991635	long-chain-fatty-acid-- ligase 1 isoform X1	6	[illegible]
Acrdi.XM_015920758.1/1-300	Monca.augustus.g35927.t1/1-519	0.138046	0.0991572	PREDICTED: uncharacterized protein LOC107354307		
Acrdi.XM_015917535.1/1-324	Monca.augustus.g25121.t1/1-324	0.124887	0.0991516	NATD1		
Acrdi.XM_015904432.1/1-705	Monca.augustus.g10189.t1/1-762	0.123699	0.0991423	signal recognition particle receptor subunit beta	4	[illegible]
Acrdi.XM_015905198.1/1-2235	Monca.adi2mcaRNA14791_R0/1-2217	0.135056	0.0991306	N-acetylated-alpha-linked acidic dipeptidase 2	1	[illegible]
Acrdi.XM_015897003.1/1-1932	Monca.adi2mcaRNA627_R0/1-1314	0.126472	0.0991206	cytoplasmic tyrosine- kinase BMX	3	[illegible]
Acrdi.XM_015897044.1/1-1143	Monca.augustus.g48377.t1/1-1332	0.114567	0.099118	Dual specificity mitogen-activated kinase kinase 5	4	[illegible]
Acrdi.XM_015896913.1/1-741	Monca.adi2mcaRNA5825_R0/1-756	0.0849961	0.0991038	proto-oncogene c-Fos	4	[illegible]
Acrdi.XM_015905581.1/1-444	Monca.augustus.g24098.t1/1-951	0.13412	0.0990978	ankyrin repeat domain-containing 10-like isoform X2		
Acrdi.XM_015908233.1/1-348	Monca.augustus.g25680.t1/1-405	0.190828	0.099092	60S acidic ribosomal P2-like	6	[illegible]
Acrdi.XM_015894794.1/1-621	Monca.augustus.g48529.t1/1-771	0.172767	0.0990668	ELL-associated factor 1	2	[illegible]
Acrdi.XM_015907445.1/1-804	Monca.augustus.g3956.t1/1-1158	0.121258	0.0990626	haloacid dehalogenase-like hydrolase	2	[illegible]
Acrdi.XM_015893043.1/1-2550	Monca.adi2mcaRNA39082_R3/1-2184	0.425357	0.0990577	Transposon Tf2-6 poly		
Acrdi.XM_015905199.1/1-2049	Monca.augustus.g52820.t1/1-2463	0.119202	0.0990346	kelch 12	7	[illegible]
Acrdi.XM_015905376.1/1-2253	Monca.augustus.g61128.t1/1-2250	0.117949	0.0990301	transcriptional repressor p66-beta isoform X1	4	[illegible]
Acrdi.XM_015910731.1/1-3162	Monca.augustus.g29725.t1/1-3573	0.102395	0.0990292	Symplekin		
Acrdi.XM_015923438.1/1-849	Monca.augustus.g26637.t1/1-1443	0.0987815	0.0990157	PREDICTED: uncharacterized protein LOC107356816		
Acrdi.XM_015896692.1/1-960	Monca.augustus.g37001.t1/1-945	0.114889	0.0990086	high-affinity lysophosphatidic acid receptor-like	4	[illegible]
Acrdi.XM_015912000.1/1-1494	Monca.augustus.g35300.t1/1-1533	0.108547	0.098982	Alanine--glyoxylate aminotransferase mitochondrial	4	[illegible]
Acrdi.XM_015925428.1/1-2100	Monca.augustus.g4254.t1/1-3243	0.124107	0.098951	ankyrin repeat domain-containing 27		
Acrdi.XM_015918367.1/1-1800	Monca.augustus.g41670.t1/1-1791	0.116806	0.0989268	Spermatogenesis- and oogenesis-specific basic helix-loop-helix-contai	7	[illegible]
Acrdi.XM_015919656.1/1-1041	Monca.adi2mcaRNA30403_R0/1-1017	0.0936301	0.0988587	UTP--glucose-1-phosphate uridylyltransferase isoform X1	2	[illegible]
Acrdi.XM_015924226.1/1-1338	Monca.augustus.g60347.t1/1-1746	0.0687999	0.0988492	serine threonine- phosphatase 2A 56 kDa regulatory subunit delta isof	1	[illegible]
Acrdi.XM_015896008.1/1-2160	Monca.augustus.g38239.t1/1-2190	0.153804	0.0988485	zinc finger FYVE domain-containing 1-like	15	[illegible]
Acrdi.XM_015895634.1/1-339	Monca.augustus.g22270.t1/1-339	0.127224	0.0988321	death-associated 1	3	[illegible]
Acrdi.XM_015898473.1/1-954	Monca.adi2mcaRNA7578_R1/1-750	0.472957	0.0988169	RNA-directed DNA polymerase from transposon X-element		
Acrdi.XM_015894290.1/1-1143	Monca.augustus.g7530.t1/1-2196	0.075121	0.0988098	prolyl endopeptidase-like	4	[illegible]
Acrdi.XM_015895226.1/1-663	Monca.augustus.g42808.t1/1-1500	0.0872972	0.0987534	vesicle-fusing ATPase-like	6	[illegible]
Acrdi.XM_015899966.1/1-732	Monca.augustus.g71258.t1/1-897	0.638695	0.0987346	PREDICTED: uncharacterized protein C14orf93 homolog		
Acrdi.XM_015893830.1/1-789	Monca.augustus.g23797.t1/1-1095	0.208319	0.0987316	ubiquitin-associated domain-containing	4	[illegible]
Acrdi.XM_015896788.1/1-1416	Monca.adi2mcaRNA5658_R0/1-1350	0.165347	0.0987288	ADP-dependent glucokinase-like	2	[illegible]
Acrdi.XM_015899706.1/1-1323	Monca.augustus.g66171.t1/1-1899	0.149197	0.0986798	peptidoglycan binding domain		
Acrdi.XM_015905642.1/1-1479	Monca.augustus.g13496.t1/1-1485	0.104473	0.0986754	alpha-(1,3)-fucosyltransferase 11-like	5	[illegible]
Acrdi.XM_015899383.1/1-1092	Monca.adi2mcaRNA1022_R0/1-951	0.127991	0.098659	acetylcholine receptor subunit beta isoform X1	1	[illegible]
Acrdi.XM_015917637.1/1-1773	Monca.augustus.g48584.t1/1-2160	0.147568	0.0986513	solute carrier family 26 member 6-like	7	[illegible]
Acrdi.XM_015891926.1/1-1248	Monca.augustus.g25633.t1/1-1464	0.133859	0.0986489	Diphthamide biosynthesis 1	1	[illegible]
Acrdi.XM_015901978.1/1-2310	Monca.augustus.g2311.t1/1-930	0.0903541	0.0985771	ATP-dependent 6- muscle type-like	5	[illegible]
Acrdi.XM_015909261.1/1-2157	Monca.augustus.g35326.t1/1-3291	0.123794	0.0985689	roquin-1 isoform X1	1	[illegible]
Acrdi.XM_015911725.1/1-918	Monca.adi2mcaRNA2211_R0/1-891	0.133094	0.0985636	TBC1 domain family member 7	2	[illegible]

Acrdi.XM_015919612.1/1-1173 | Monca.augustus.g37836.t1/1-1245	0.123035	0.0985498	TBC1 domain family member 13	7	[illegible]	[illegible]
Acrdi.XM_015921857.1/1-744 | Monca.adi2mcaRNA33255_R4/1-792	0.421701	0.0985357	PREDICTED: uncharacterized protein LOC107355316			
Acrdi.XM_015892723.1/1-870 | Monca.augustus.g28705.t1/1-1461	0.95565	0.0985096	cell wall integrity and stress response component 4-like			
Acrdi.XM_015895931.1/1-915 | Monca.augustus.g58268.t1/1-1491	0.120235	0.0984958	RNA polymerase II elongation factor ELL2 isoform X1	2	P:GO:0006366; C:GO:0044424	P:transcription from RNA polymerase II promoter; C:intracellular part
Acrdi.XM_015907207.1/1-798 | Monca.augustus.g27289.t1/1-810	0.300385	0.0984467	PREDICTED: uncharacterized protein LOC107341742			
Acrdi.XM_015907482.1/1-1011 | Monca.augustus.g3964.t1/1-1038	0.0931104	0.0984381	Neuropeptide FF receptor 2	3	P:GO:0007165; C:GO:0016021; F:GO:0004871	P:signal transduction; C:integral component of membrane; F:signal transducer activity
Acrdi.XM_015906851.1/1-2994 | Monca.augustus.g32960.t1/1-4206	0.102447	0.0984135	speract receptor-like	13	[illegible]	[illegible]
Acrdi.XM_015907838.1/1-1473 | Monca.augustus.g47382.t1/1-1479	0.130038	0.0983661	neuronal acetylcholine receptor subunit alpha-10-like	5	[illegible]	[illegible]
Acrdi.XM_015903923.1/1-2109 | Monca.adi2mcaRNA1314_R0/1-2136	0.176311	0.0983605	sodium potassium calcium exchanger 4-like isoform X1	9	[illegible]	[illegible]
Acrdi.XM_015912970.1/1-912 | Monca.augustus.g56200.t1/1-1629	0.148526	0.0983498	ectonucleoside triphosphate diphosphohydrolase 7-like			
Acrdi.XM_015920590.1/1-870 | Monca.augustus.g35470.t1/1-1035	0.117701	0.0983273	O(6)-methylguanine-induced apoptosis 2-like			
Acrdi.XM_015911267.1/1-1746 | Monca.augustus.g37488.t1/1-3033	0.124665	0.0983059	Cat eye syndrome critical region 2			
Acrdi.XM_015896200.1/1-687 | Monca.augustus.g3517.t1/1-693	0.147508	0.0982998	phosphatidylinositol 3-kinase 1-like	1	C:GO:0016020	C:membrane
Acrdi.XM_015917269.1/1-987 | Monca.augustus.g45712.t1/1-1116	0.0911653	0.0982718	Beta-2 adrenergic receptor	5	[illegible]	[illegible]
Acrdi.XM_015900417.1/1-1482 | Monca.adi2mcaRNA9592_R0/1-1410	0.124386	0.098239	A-kinase anchor mitochondrial	2	F:GO:0003676; F:GO:0003723	F:nucleic acid binding; F:RNA binding
Acrdi.XM_015924655.1/1-1005 | Monca.adi2mcaRNA36509_R0/1-1005	0.128513	0.0982275	rab GDP dissociation inhibitor alpha	8	[illegible]	[illegible]
Acrdi.XM_015922864.1/1-801 | Monca.adi2mcaRNA34401_R1/1-624	0.115681	0.098216	High mobility group B3			
Acrdi.XM_015915247.1/1-984 | Monca.augustus.g15246.t1/1-1434	0.112652	0.0981675	phosphatase 1 regulatory subunit 32-like			
Acrdi.XM_015915808.1/1-1026 | Monca.adi2mcaRNA26537_R0/1-855	0.54846	0.098155	PREDICTED: uncharacterized protein LOC107349633			
Acrdi.XM_015893920.1/1-2667 | Monca.augustus.g62478.t1/1-2781	0.117693	0.0981065	DNA mismatch repair	2	F:GO:0097159; F:GO:1901363	F:organic cyclic compound binding; F:heterocyclic compound binding
Acrdi.XM_015924807.1/1-864 | Monca.augustus.g66188.t1/1-885	0.0963635	0.0981034	transcription initiation factor IIE subunit beta	8	[illegible]	[illegible]
Acrdi.XM_015915655.1/1-402 | Monca.augustus.g15826.t1/1-387	0.140934	0.0980999	polyadenylate-binding -interacting 2B isoform X2			
Acrdi.XM_015920413.1/1-1641 | Monca.augustus.g46005.t1/1-1641	0.101235	0.0980823	Carboxypeptidase D	7	[illegible]	[illegible]
Acrdi.XM_015910257.1/1-981 | Monca.augustus.g66387.t1/1-1071	0.096905	0.0980487	Geranylgeranyl transferase type-1 subunit beta	3	[illegible]	[illegible]
Acrdi.XM_015896462.1/1-603 | Monca.augustus.g54918.t1/1-1533	0.104969	0.0980143	target of rapamycin complex 2 subunit MAPKAP1 isoform X1			
Acrdi.XM_015909442.1/1-2277 | Monca.adi2mcaRNA19502_R0/1-2148	0.10357	0.0980114	E3 ubiquitin- ligase UHRF1	7	[illegible]	[illegible]
Acrdi.XM_015895475.1/1-576 | Monca.augustus.g14884.t1/1-714	0.0806042	0.0979732	max X isoform X2	3	[illegible]	[illegible]
Acrdi.XM_015895977.1/1-1662 | Monca.adi2mcaRNA4804_R1/1-1509	0.270286	0.0979613	PREDICTED: uncharacterized protein LOC107331400			
Acrdi.XM_015911465.1/1-690 | Monca.augustus.g63600.t1/1-702	0.101843	0.0979609	TP53-regulating kinase	8	[illegible]	[illegible]
Acrdi.XM_015924508.1/1-993 | Monca.augustus.g28539.t1/1-1059	0.0934366	0.0979537	ethanolamine kinase 1-like	3	F:GO:0000166; P:GO:0008654; F:GO:0016301	F:nucleotide binding; P:phospholipid biosynthetic process; F:kinase activity
Acrdi.XM_015897412.1/1-801 | Monca.augustus.g3805.t1/1-804	0.131786	0.0979103	Tob1	11	[illegible]	[illegible]
Acrdi.XM_015898163.1/1-507 | Monca.augustus.g29275.t1/1-513	0.0897177	0.0979053	FAM133B isoform X2	1	F:GO:0003723	F:RNA binding
Acrdi.XM_015912852.1/1-981 | Monca.augustus.g44025.t1/1-1143	0.12225	0.0979042	minor histocompatibility antigen H13 isoform X2	14	[illegible]	[illegible]
Acrdi.XM_015908516.1/1-1953 | Monca.augustus.g46689.t1/1-1959	0.120896	0.0978543	frizzled-10	6	[illegible]	[illegible]
Acrdi.XM_015916500.1/1-438 | Monca.augustus.g39006.t1/1-765	0.0844215	0.0978524	proline synthase co-transcribed bacterial homolog	2	F:GO:0030170; C:GO:0005622	F:pyridoxal phosphate binding; C:intracellular
Acrdi.XM_015921971.1/1-747 | Monca.augustus.g23750.t1/1-747	0.176584	0.0978489	polycomb group RING finger 3	4	[illegible]	[illegible]
Acrdi.XM_015905819.1/1-675 | Monca.augustus.g31755.t1/1-678	0.150281	0.0978449	homeobox XENK-2-like	2	[illegible]	[illegible]
Acrdi.XM_015924926.1/1-1113 | Monca.adi2mcaRNA36802_R0/1-1062	0.117727	0.0978349	low choriolytic enzyme-like	1	F:GO:0016787	[illegible]
Acrdi.XM_015918123.1/1-1263 | Monca.adi2mcaRNA29151_R1/1-1227	0.154477	0.0978295	endothelin-converting enzyme 2-like	1	C:GO:0016020	[illegible]
Acrdi.XM_015909749.1/1-1032 | Monca.augustus.g2267.t1/1-1029	0.105271	0.0978082	cyclin-dependent kinase 7	3	[illegible]	[illegible]
Acrdi.XM_015896272.1/1-1224 | Monca.augustus.g33712.t1/1-1194	0.127507	0.0977544	peptidase M20 domain-containing 2-like	2	[illegible]	[illegible]
Acrdi.XM_015921371.1/1-1311 | Monca.augustus.g4643.t1/1-1452	0.116907	0.0977447	RNA polymerase II-associated factor 1 homolog	10	[illegible]	[illegible]
Acrdi.XM_015903247.1/1-2235 | Monca.augustus.g51158.t1/1-2232	0.138264	0.0977198	Peroxisomal multifunctional enzyme type 2	10	[illegible]	[illegible]
Acrdi.XM_015918979.1/1-1833 | Monca.augustus.g66507.t1/1-1818	0.111929	0.0976934	zinc metallo ase nas-13-like	3	[illegible]	[illegible]
Acrdi.XM_015923166.1/1-864 | Monca.adi2mcaRNA34799_R1/1-753	0.467529	0.0976745	Retrovirus-related Pol poly from transposon			
Acrdi.XM_015901837.1/1-771 | Monca.augustus.g20167.t1/1-888	0.114402	0.0976674	probable enoyl- mitochondrial	2	[illegible]	[illegible]
Acrdi.XM_015923113.1/1-471 | Monca.augustus.g30578.t1/1-723	0.0743473	0.0976338	dolichol-phosphate mannosyltransferase subunit 1 isoform X2	7	[illegible]	[illegible]
Acrdi.XM_015911124.1/1-1872 | Monca.augustus.g24023.t1/1-2001	0.104053	0.0976151	PREDICTED: protein BZZ1-like	3	[illegible]	[illegible]
Acrdi.XM_015892338.1/1-4422 | Monca.augustus.g21727.t1/1-4980	0.106888	0.0976122	Ubiquitin carboxyl-terminal hydrolase 32	1	F:GO:0016787	[illegible]
Acrdi.XM_015908554.1/1-609 | Monca.adi2mcaRNA18544_R0/1-711	0.16011	0.0975936	PREDICTED: uncharacterized protein LOC107343033 isoform X2			
Acrdi.XM_015898842.1/1-1671 | Monca.augustus.g5286.t1/1-1905	0.116902	0.0975686	spire homolog 1 isoform X4	3	[illegible]	[illegible]
Acrdi.XM_015916289.1/1-1380 | Monca.adi2mcaRNA27057_R0/1-1416	0.141292	0.0975347	Meiosis-specific with OB domain-containing	4	[illegible]	[illegible]
Acrdi.XM_015914037.1/1-1347 | Monca.adi2mcaRNA24544_R0/1-1239	0.101014	0.0975303	PREDICTED: uncharacterized protein KIAA0895-like			
Acrdi.XM_015892435.1/1-927 | Monca.augustus.g71122.t1/1-1041	0.129844	0.0975227	GMP reductase 2 isoform X2	2	[illegible]	[illegible]
Acrdi.XM_015915047.1/1-582 | Monca.augustus.g49275.t1/1-582	0.118417	0.097513	vacuolar sorting-associated 37B-like			
Acrdi.XM_015923006.1/1-1053 | Monca.augustus.g46068.t1/1-1404	0.14532	0.0975017	chitobiosyldiphosphodolichol beta-mannosyltransferase			
Acrdi.XM_015907103.1/1-657 | Monca.augustus.g36991.t1/1-651	0.177905	0.0974853	achaete-scute homolog 1	6	[illegible]	[illegible]
Acrdi.XM_015892641.1/1-1116 | Monca.augustus.g63894.t1/1-1119	0.652824	0.0974755	PREDICTED: uncharacterized protein LOC107327922			
Acrdi.XM_015909645.1/1-2127 | Monca.adi2mcaRNA19693_R1/1-2160	0.115152	0.0974108	phospholipase A-2-activating isoform X1	4	[illegible]	[illegible]
Acrdi.XM_015923835.1/1-450 | Monca.augustus.g8179.t1/1-450	0.130296	0.0974021	myosin-2 essential light chain-like	1	F:GO:0005509	[illegible]
Acrdi.XM_015898571.1/1-1857 | Monca.adi2mcaRNA752_R0/1-1857	0.17191	0.0973799	sodium-coupled monocarboxylate transporter 1-like	7	[illegible]	[illegible]
Acrdi.XM_015922451.1/1-363 | Monca.augustus.g40385.t1/1-363	0.158796	0.0973757	PREDICTED: uncharacterized protein LOC107355829			
Acrdi.XM_015899350.1/1-489 | Monca.augustus.g40308.t1/1-729	0.147824	0.0973477	Ras-related and estrogen-regulated growth inhibitor	2	[illegible]	[illegible]
Acrdi.XM_015901192.1/1-1503 | Monca.adi2mcaRNA10446_R0/1-1509	0.118849	0.0973467	Arylsulfatase B	7	[illegible]	[illegible]
Acrdi.XM_015900497.1/1-1428 | Monca.adi2mcaRNA9764_R0/1-1212	0.114585	0.0973077	DNA repair and recombination RAD26-	1	F:GO:0005524	[illegible]
Acrdi.XM_015891673.1/1-636 | Monca.adi2mcaRNA37526_R4/1-456	0.434399	0.0972745	Transposon Tf2-6 poly			
Acrdi.XM_015915746.1/1-2604 | Monca.adi2mcaRNA26457_R0/1-2562	0.122754	0.0972568	Gamma-tubulin complex component 6	12	[illegible]	[illegible]
Acrdi.XM_015904347.1/1-705 | Monca.augustus.g68752.t1/1-1893	0.130329	0.0972447	ecdysoneless homolog	3	[illegible]	[illegible]
Acrdi.XM_015904265.1/1-1131 | Monca.augustus.g62126.t1/1-1134	0.15583	0.0972437	tropomodulin-3	6	[illegible]	[illegible]
Acrdi.XM_015917267.1/1-2181 | Monca.adi2mcaRNA28192_R0/1-2505	0.129668	0.0972263	probable phospholipid-transporting ATPase	6	[illegible]	[illegible]
Acrdi.XM_015909893.1/1-1377 | Monca.adi2mcaRNA19996_R0/1-1320	0.1328	0.0972164	cytochrome b5 reductase 4	4	[illegible]	[illegible]
Acrdi.XM_015916513.1/1-1257 | Monca.augustus.g38971.t1/1-1257	0.0893643	0.0972151	cytochrome P450 120	3	[illegible]	[illegible]
Acrdi.XM_015901748.1/1-570 | Monca.augustus.g49651.t1/1-561	0.0808279	0.0972067	Bcl-2 2	6	[illegible]	[illegible]
Acrdi.XM_015924373.1/1-1023 | Monca.augustus.g17970.t1/1-510	0.114364	0.0971911	PREDICTED: uncharacterized protein LOC107357744	1	C:GO:0016021	[illegible]
Acrdi.XM_015914009.1/1-657 | Monca.augustus.g68896.t1/1-765	0.109771	0.0971908	pitchfork-like			
Acrdi.XM_015908548.1/1-1716 | Monca.augustus.g13046.t1/1-2676	0.0968869	0.0971746	3-dehydroquinate synthase	4	[illegible]	[illegible]
Acrdi.XM_015908301.1/1-1008 | Monca.augustus.g17447.t1/1-999	0.0852827	0.0971394	mesoderm-specific transcript homolog	3	[illegible]	[illegible]
Acrdi.XM_015909992.1/1-1131 | Monca.augustus.g44403.t1/1-1656	0.119092	0.0971359	UBX domain-containing 11	2	[illegible]	[illegible]
Acrdi.XM_015897254.1/1-2319 | Monca.adi2mcaRNA6169_R0/1-1887	0.123959	0.0971233	thioredoxin domain-containing 3 homolog	1	P:GO:0009117	[illegible]
Acrdi.XM_015909109.1/1-1227 | Monca.adi2mcaRNA19095_R0/1-1269	0.101738	0.0971219	histone deacetylase complex subunit SAP130-like			
Acrdi.XM_015910029.1/1-2655 | Monca.augustus.g38592.t1/1-2760	0.113663	0.0971195	PAX3- and PAX7-binding	3	[illegible]	[illegible]
Acrdi.XM_015903818.1/1-582 | Monca.augustus.g33837.t1/1-609	0.113894	0.0971093	gluconate kinase	8	[illegible]	[illegible]
Acrdi.XM_015923149.1/1-1503 | Monca.adi2mcaRNA34781_R0/1-1296	0.123366	0.0971034	ATP-dependent DNA helicase Q1			
Acrdi.XM_015906198.1/1-2088 | Monca.augustus.g9780.t1/1-2085	0.127499	0.0970976	serine incorporator 3	4	[illegible]	[illegible]
Acrdi.XM_015913491.1/1-675 | Monca.augustus.g60184.t1/1-678	0.111548	0.0970969	methyltransferase 23			
Acrdi.XM_015897570.1/1-633 | Monca.augustus.g66249.t1/1-471	0.441198	0.0970033	PREDICTED: uncharacterized protein LOC107332831			
Acrdi.XM_015924146.1/1-492 | Monca.augustus.g66161.t1/1-489	0.161394	0.0969648	Centrosomal of 19 kDa	1	C:GO:0005815	[illegible]
Acrdi.XM_015901961.1/1-735 | Monca.augustus.g45539.t1/1-768	0.113177	0.0969342	GUCD1	3	[illegible]	[illegible]
Acrdi.XM_015910572.1/1-2199 | Monca.augustus.g10116.t1/1-2112	0.110512	0.0969235	PREDICTED: uncharacterized protein LOC107344886			
Acrdi.XM_015907721.1/1-1074 | Monca.augustus.g68022.t1/1-1083	0.119955	0.0969122	RING finger 151-like	3	[illegible]	[illegible]
Acrdi.XM_015921763.1/1-9618 | Monca.adi2mcaRNA33138_R0/1-9690	0.107986	0.0969068	EGF-like repeat and discoidin I-like domain-containing 3	1	F:GO:0070492	[illegible]
Acrdi.XM_015903063.1/1-861 | Monca.augustus.g56765.t1/1-1383	0.129523	0.0968972	synaptic vesicular amine transporter-like	1	P:GO:0006810	[illegible]
Acrdi.XM_015899595.1/1-582 | Monca.augustus.g46937.t1/1-600	0.16401	0.0968859	AP-5 complex subunit sigma-1	3	[illegible]	[illegible]
Acrdi.XM_015904294.1/1-324 | Monca.augustus.g51411.t1/1-321	0.112675	0.0968784	ubiquinol-cytochrome-c reductase complex assembly factor 3-like			
Acrdi.XM_015920212.1/1-2577 | Monca.augustus.g62692.t1/1-2544	0.126486	0.0968696	heat shock 70 kDa 12A-like			
Acrdi.XM_015923985.1/1-1038 | Monca.augustus.g54042.t1/1-3462	0.0979307	0.0968546	sel-1 homolog 3-like	2	[illegible]	[illegible]
Acrdi.XM_015896966.1/1-507 | Monca.augustus.g38099.t1/1-507	0.0532492	0.0968493	Ankyrin repeat domain-containing 66			
Acrdi.XM_015919967.1/1-1443 | Monca.augustus.g16007.t1/1-1455	0.104686	0.0968394	MAP kinase-interacting serine threonine- kinase 2	13	[illegible]	[illegible]
Acrdi.XM_015916432.1/1-513 | Monca.augustus.g64476.t1/1-513	0.0978553	0.0968193	50S ribosomal L16	4	[illegible]	[illegible]
Acrdi.XM_015917441.1/1-1317 | Monca.augustus.g69701.t1/1-1449	0.132184	0.0968153	Coiled-coil domain-containing 47	2	[illegible]	[illegible]
Acrdi.XM_015921693.1/1-1116 | Monca.augustus.g43097.t1/1-1116	0.117664	0.0968126	ALK tyrosine kinase receptor	2	[illegible]	[illegible]
Acrdi.XM_015911112.1/1-507 | Monca.augustus.g58864.t1/1-510	0.134771	0.0967688	PREDICTED: uncharacterized protein LOC107345410	1	C:GO:0016020	[illegible]
Acrdi.XM_015916593.1/1-2022 | Monca.adi2mcaRNA25239_R7/1-1344	0.27405	0.0967609	PREDICTED: uncharacterized protein LOC107350371	2	[illegible]	[illegible]
Acrdi.XM_015895638.1/1-2400 | Monca.adi2mcaRNA4380_R0/1-2553	0.108689	0.0967468	aryl hydrocarbon receptor	5	[illegible]	[illegible]
Acrdi.XM_015898059.1/1-621 | Monca.augustus.g29269.t1/1-639	0.0968913	0.0967408	Golgi SNAP receptor complex member 2	15	[illegible]	[illegible]
Acrdi.XM_015924236.1/1-519 | Monca.augustus.g30266.t1/1-459	0.693424	0.0967271	UPF0746 DDB_G0281095-like			
Acrdi.XM_015920824.1/1-516 | Monca.augustus.g49854.t1/1-516	0.097633	0.096726	mesencephalic astrocyte-derived neurotrophic factor homolog			
Acrdi.XM_015905473.1/1-2271 | Monca.augustus.g28237.t1/1-2757	0.125398	0.0966947	sarcolemmal membrane-associated isoform X11			
Acrdi.XM_015917653.1/1-441 | Monca.adi2mcaRNA28611_R0/1-297	0.0894355	0.0966858	Phosphatidylinositol-glycan biosynthesis class F	1	C:GO:0016020	[illegible]
Acrdi.XM_015892648.1/1-1089 | Monca.augustus.g51170.t1/1-1194	0.107518	0.0966725	Actin-related 6	1	C:GO:0005634	[illegible]
Acrdi.XM_015907132.1/1-696 | Monca.augustus.g36958.t1/1-1449	0.0789479	0.0966544	BTB POZ domain-containing 6-like			
Acrdi.XM_015923303.1/1-1851 | Monca.adi2mcaRNA3595_R0/1-1701	0.156653	0.096611	Sec1 family domain-containing 2	2	[illegible]	[illegible]
Acrdi.XM_015894040.1/1-1779 | Monca.adi2mcaRNA4024_R0/1-1794	0.141776	0.096596	monocarboxylate transporter 10-like	3	[illegible]	[illegible]
Acrdi.XM_015912478.1/1-588 | Monca.augustus.g54444.t1/1-1143	0.103922	0.0965809	histidine N-acetyltransferase-	4	[illegible]	[illegible]
Acrdi.XM_015898326.1/1-1257 | Monca.augustus.g66595.t1/1-1740	0.12829	0.0965746	basic proline-rich -like			
Acrdi.XM_015896751.1/1-393 | Monca.augustus.g42414.t1/1-399	0.112362	0.096558	PREDICTED: uncharacterized protein LOC107332031			
Acrdi.XM_015898604.1/1-1041 | Monca.augustus.g54248.t1/1-1038	0.106941	0.0965387	histamine H2 receptor-like	3	[illegible]	[illegible]
Acrdi.XM_015908502.1/1-1452 | Monca.augustus.g15582.t1/1-2652	0.114668	0.0965282	Dimethylglycine mitochondrial	2	[illegible]	[illegible]
Acrdi.XM_015912068.1/1-1341 | Monca.augustus.g67648.t1/1-1338	0.118042	0.0965217	PREDICTED: uncharacterized protein LOC107346291	4	[illegible]	[illegible]
Acrdi.XM_015899456.1/1-507 | Monca.augustus.g48275.t1/1-516	0.127316	0.096507	phosphatase 1 regulatory subunit 27			
Acrdi.XM_015907243.1/1-918 | Monca.augustus.g7059.t1/1-897	0.100881	0.0964546	Solute carrier family 25 member 51	4	[illegible]	[illegible]
Acrdi.XM_015893142.1/1-447 | Monca.augustus.g15708.t1/1-729	0.0877038	0.0964432	ATP synthase subunit mitochondrial	3	[illegible]	[illegible]
Acrdi.XM_015893294.1/1-444 | Monca.augustus.g540.t1/1-651	0.07603	0.0964424	helix-loop-helix delilah-like	3	[illegible]	[illegible]
Acrdi.XM_015908659.1/1-1113 | Monca.augustus.g53861.t1/1-915	0.104929	0.0964344	Fibronectin type 3 and ankyrin repeat domains 1	1	C:GO:0005654	[illegible]
Acrdi.XM_015921367.1/1-711 | Monca.augustus.g11103.t1/1-1146	0.122834	0.0963995	E3 ubiquitin- ligase FANCL isoform X1	6	[illegible]	[illegible]
Acrdi.XM_015909287.1/1-1767 | Monca.augustus.g18287.t1/1-2601	0.156084	0.0963948	ubiquitin carboxyl-terminal hydrolase 33 isoform X1			
Acrdi.XM_015901244.1/1-732 | Monca.augustus.g67903.t1/1-735	0.102389	0.0963878	glutaredoxin domain-containing cysteine-rich CG31559-like	7	[illegible]	[illegible]
Acrdi.XM_015895288.1/1-615 | Monca.augustus.g8029.t1/1-678	0.0875533	0.0963582	#NAME?	2	[illegible]	[illegible]
Acrdi.XM_015905365.1/1-2874 | Monca.augustus.g60547.t1/1-2937	0.0928808	0.0963571	kinesin KIF28P	6	[illegible]	[illegible]
Acrdi.XM_015914064.1/1-663 | Monca.augustus.g24063.t1/1-735	0.201346	0.0963449	gamma-sarcoglycan isoform X2	5	[illegible]	[illegible]
Acrdi.XM_015900702.1/1-747 | Monca.augustus.g5460.t1/1-864	0.147968	0.096344	lipoma-preferred partner homolog isoform X2	1	F:GO:0046872	[illegible]
Acrdi.XM_015908299.1/1-1008 | Monca.augustus.g40908.t1/1-2049	0.105816	0.0963287	dual specificity tyrosine-phosphorylation-regulated kinase 4-like isofon	3	[illegible]	[illegible]
Acrdi.XM_015911344.1/1-2721 | Monca.augustus.g35290.t1/1-2670	0.097733	0.0963119	anoctamin-4-like isoform X1	1	C:GO:0016020	[illegible]
Acrdi.XM_015910229.1/1-1671 | Monca.augustus.g38621.t1/1-1830	0.0857561	0.0962672	prickle 3 isoform X1	2	[illegible]	[illegible]
Acrdi.XM_015908048.1/1-588 | Monca.augustus.g6867.t1/1-792	0.113177	0.0962652	spermatogenesis-associated 4-like	7	[illegible]	[illegible]
Acrdi.XM_015897844.1/1-1263 | Monca.augustus.g21048.t1/1-1263	0.162334	0.0962354	G- coupled receptor 161-like	2	[illegible]	[illegible]
Acrdi.XM_015903028.1/1-879 | Monca.augustus.g47572.t1/1-882	0.14915	0.0962284	lysosomal thioesterase PPT2	3	[illegible]	[illegible]
Acrdi.XM_015903266.1/1-852 | Monca.augustus.g55378.t1/1-834	0.146691	0.0962243	ras and EF-hand domain-containing homolog			
Acrdi.XM_015896218.1/1-681 | Monca.augustus.g23051.t1/1-2268	0.13139	0.0962169	short transient receptor potential channel 4-associated -like isoform X1			
Acrdi.XM_015921677.1/1-1953 | Monca.augustus.g43096.t1/1-2553	0.110395	0.096214	rho GTPase-activating 26 isoform X1	3	[illegible]	[illegible]
Acrdi.XM_015908765.1/1-6324 | Monca.adi2mcaRNA18723_R0/1-6300	0.129538	0.0962007	Leucine-rich repeat serine threonine- kinase			
Acrdi.XM_015918695.1/1-681 | Monca.augustus.g41753.t1/1-825	0.929241	0.0961734	Tetratricopeptide repeat 28			
Acrdi.XM_015900149.1/1-1005 | Monca.adi2mcaRNA9326_R0/1-933	0.13461	0.0961646	isoform B	1	F:GO:0046872	[illegible]
Acrdi.XM_015917212.1/1-792 | Monca.adi2mcaRNA28123_R1/1-702	0.758252	0.0961361	gliding motility regulatory -like	2	[illegible]	[illegible]
Acrdi.XM_015922368.1/1-450 | Monca.augustus.g66204.t1/1-2337	0.0822023	0.0961177	YTH domain-containing 1-like	5	[illegible]	[illegible]
Acrdi.XM_015921387.1/1-1914 | Monca.augustus.g11105.t1/1-1944	0.121652	0.0961115	E3 ubiquitin- ligase synoviolin isoform X2	18	[illegible]	[illegible]
Acrdi.XM_015896383.1/1-3165 | Monca.augustus.g49973.t1/1-5022	0.108815	0.0961107	5 -3 exoribonuclease 1 isoform X1	10	[illegible]	[illegible]
Acrdi.XM_015909504.1/1-1548 | Monca.augustus.g1394.t1/1-2466	0.110112	0.0961044	Glycine-rich domain-containing 2			
Acrdi.XM_015922208.1/1-2241 | Monca.adi2mcaRNA33643_R1/1-2043	0.0794787	0.0960972	tyrosine- phosphatase non-receptor type 4-like isoform X1	2	[illegible]	[illegible]
Acrdi.XM_015892822.1/1-1761 | Monca.adi2mcaRNA38839_R0/1-1722	0.106166	0.0960963	coiled-coil domain-containing 60 isoform X1	9	[illegible]	[illegible]
Acrdi.XM_015916359.1/1-513 | Monca.adi2mcaRNA27153_R0/1-459	0.091301	0.0960955	Marginal zone B- and B1-cell-specific	4	[illegible]	[illegible]
Acrdi.XM_015900557.1/1-843 | Monca.augustus.g24431.t1/1-975	0.354201	0.0960934	RNA-directed DNA polymerase from mobile element jockey			
Acrdi.XM_015900775.1/1-1089 | Monca.augustus.g67220.t1/1-2934	0.10403	0.0960562	CUB domain-containing 2			
Acrdi.XM_015895766.1/1-1356 | Monca.adi2mcaRNA4535_R0/1-1359	0.0934353	0.0960263	L-2-hydroxyglutarate mitochondrial	3	[illegible]	[illegible]
Acrdi.XM_015908770.1/1-7428 | Monca.augustus.g26270.t1/1-9348	0.135962	0.0960221	cilia- and flagella-associated 47			
Acrdi.XM_015891896.1/1-546 | Monca.augustus.g39640.t1/1-624	0.122091	0.0960219	coiled-coil domain-containing 25	2	[illegible]	[illegible]
Acrdi.XM_015923030.1/1-960 | Monca.augustus.g10942.t1/1-954	0.136571	0.0959962	Target of rapamycin complex subunit lst8	1	C:GO:0005737	[illegible]
Acrdi.XM_015893428.1/1-1716 | Monca.adi2mcaRNA39506_R0/1-1407	0.304309	0.0959418	ATP-dependent DNA helicase -like			
Acrdi.XM_015906634.1/1-615 | Monca.adi2mcaRNA106_R0/1-573	0.125158	0.0959405	PREDICTED: uncharacterized protein LOC107341192	2	[illegible]	[illegible]
Acrdi.XM_015905649.1/1-1407 | Monca.augustus.g65077.t1/1-1392	0.116415	0.0959189	leucine-rich repeat-containing 74B-like	1	F:GO:0005509	[illegible]
Acrdi.XM_015918181.1/1-642 | Monca.augustus.g66558.t1/1-642	0.184036	0.0959119	homeobox Hox-A5	3	[illegible]	[illegible]
Acrdi.XM_015922001.1/1-333 | Monca.augustus.g60063.t1/1-360	0.100123	0.09591	signal peptidase complex subunit 3	4	[illegible]	[illegible]
Acrdi.XM_015898349.1/1-1068 | Monca.augustus.g1156.t1/1-1026	0.116563	0.0958989	kelch domain-containing 9			
Acrdi.XM_015901747.1/1-633 | Monca.augustus.g14600.t1/1-639	0.17902	0.0958801	WASH complex subunit CCDC53	3	[illegible]	[illegible]
Acrdi.XM_015916779.1/1-1620 | Monca.augustus.g17204.t1/1-1626	0.111488	0.0958708	synaptotagmin-2 isoform X1	10	[illegible]	[illegible]
Acrdi.XM_015895844.1/1-1344 | Monca.augustus.g8579.t1/1-1335	0.139348	0.09587	integral membrane GPR137B	1	C:GO:0016020	[illegible]
Acrdi.XM_015899475.1/1-1092 | Monca.augustus.g37231.t1/1-1065	0.098481	0.0958658	aflatoxin B1 aldehyde reductase member 4-like	1	C:GO:0005575	[illegible]
Acrdi.XM_015896804.1/1-933 | Monca.augustus.g49286.t1/1-792	0.080547	0.0958599	PREDICTED: uncharacterized protein LOC107332076			
Acrdi.XM_015902281.1/1-903 | Monca.augustus.g35111.t1/1-774	0.0948063	0.0958443	non-structural maintenance of chromosomes element 1 homolog	2	[illegible]	[illegible]
Acrdi.XM_015915125.1/1-1374 | Monca.augustus.g26564.t1/1-1371	0.14894	0.0958397	Zinc finger 503	2	[illegible]	[illegible]
Acrdi.XM_015916392.1/1-702 | Monca.augustus.g52625.t1/1-774	0.111988	0.095809	stromal cell-derived factor 2 1	7	[illegible]	[illegible]
Acrdi.XM_015907248.1/1-534 | Monca.augustus.g12388.t1/1-555	0.113296	0.0958012	Mpv17 isoform X3	2	[illegible]	[illegible]
Acrdi.XM_015902334.1/1-447 | Monca.augustus.g18868.t1/1-582	0.76339	0.0957878	PREDICTED: uncharacterized protein LOC107337220			
Acrdi.XM_015893299.1/1-1323 | Monca.augustus.g21540.t1/1-1341	0.1467	0.0957701	BTN1-like	2	[illegible]	[illegible]
Acrdi.XM_015894012.1/1-420 | Monca.augustus.g16872.t1/1-366	0.118965	0.0957142	seleno T	1	C:GO:0016020	[illegible]
Acrdi.XM_015915294.1/1-2523 | Monca.augustus.g25263.t1/1-5445	0.128099	0.0957049	anaphase-promoting complex subunit 1-like	1	C:GO:0005680	[illegible]
Acrdi.XM_015907090.1/1-678 | Monca.augustus.g36994.t1/1-2028	0.148172	0.0956881	CCR4-NOT transcription complex subunit 2			
Acrdi.XM_015900291.1/1-882 | Monca.augustus.g63376.t1/1-891	0.152629	0.0956702	solute carrier family 25 member 38-like isoform X1	5	[illegible]	[illegible]
Acrdi.XM_015894982.1/1-420 | Monca.augustus.g11963.t1/1-1686	0.107891	0.0956691	inositol-3-phosphate synthase 1	3	[illegible]	[illegible]
Acrdi.XM_015892375.1/1-1281 | Monca.augustus.g58188.t1/1-1302	0.13222	0.0956386	glucocorticoid-induced transcript 1 -like isoform X1			
Acrdi.XM_015898322.1/1-5148 | Monca.augustus.g66591.t1/1-5145	0.131509	0.0956285	ATP-binding cassette sub-family A member 3	6	[illegible]	[illegible]
Acrdi.XM_015912858.1/1-1347 | Monca.augustus.g19113.t1/1-2385	0.108711	0.0955944	general transcription factor IIF subunit 1	3	[illegible]	[illegible]
Acrdi.XM_015898060.1/1-2838 | Monca.augustus.g39430.t1/1-2943	0.129171	0.0955803	cyclic nucleotide-gated olfactory channel isoform X1	5	[illegible]	[illegible]
Acrdi.XM_015892436.1/1-1203 | Monca.augustus.g69052.t1/1-1212	0.105728	0.0955669	Peroxisomal 3,2-trans-enoyl- isomerase	1	F:GO:0003824	[illegible]
Acrdi.XM_015924363.1/1-1530 | Monca.adi2mcaRNA3629_R0/1-1359	0.0957378	0.0955597	D-2-hydroxyglutarate mitochondrial			
Acrdi.XM_015904058.1/1-3354 | Monca.augustus.g32313.t1/1-5640	0.144458	0.0955431	von Willebrand factor A domain-containing 8	2	[illegible]	[illegible]
Acrdi.XM_015896649.1/1-1344 | Monca.adi2mcaRNA5604_R0/1-1353	0.13996	0.0955386	PREDICTED: uncharacterized protein LOC107331956	3	[illegible]	[illegible]
Acrdi.XM_015902755.1/1-3375 | Monca.adi2mcaRNA12103_R0/1-3339	0.110565	0.0955279	Xanthine dehydrogenase oxidase	6	[illegible]	[illegible]
Acrdi.XM_015892864.1/1-729 | Monca.augustus.g7547.t1/1-1230	0.14321	0.095519	F-box only 4-like	3	[illegible]	[illegible]
Acrdi.XM_015916755.1/1-2121 | Monca.augustus.g9019.t1/1-2397	0.129902	0.0954893	Integrin beta-PS	4	[illegible]	[illegible]
Acrdi.XM_015917120.1/1-828 | Monca.adi2mcaRNA28022_R9/1-1395	0.416741	0.0954793	PREDICTED: uncharacterized protein K02A2.6-like			
Acrdi.XM_015895734.1/1-1452 | Monca.augustus.g47026.t1/1-1608	0.0719091	0.09546	radial spoke head 4 homolog A isoform X1			
Acrdi.XM_015923654.1/1-381 | Monca.augustus.g67759.t1/1-432	0.823648	0.0954468	PREDICTED: uncharacterized protein LOC107357011			
Acrdi.XM_015908004.1/1-960 | Monca.augustus.g43113.t1/1-960	0.116293	0.0954351	neuropeptide FF receptor 2-like	8	[illegible]	[illegible]
Acrdi.XM_015916985.1/1-1578 | Monca.adi2mcaRNA27887_R0/1-1533	0.142892	0.0954321	WD repeat-containing and planar cell polarity effector fritz homolog isoform X1			
Acrdi.XM_015898134.1/1-3582 | Monca.augustus.g66915.t1/1-3510	0.129462	0.0954144	WD repeat-containing on Y chromosome-like isoform X2	1	F:GO:0005509	[illegible]
Acrdi.XM_015917750.1/1-813 | Monca.augustus.g46226.t1/1-792	0.116442	0.0954136	homeobox Dlx4a-like	4	[illegible]	[illegible]
Acrdi.XM_015925346.1/1-1707 | Monca.augustus.g22624.t1/1-2730	0.113275	0.095413	polyribonucleotide nucleotidyltransferase mitochondrial	1	P:GO:0016070	[illegible]
Acrdi.XM_015897916.1/1-1209 | Monca.augustus.g39402.t1/1-1245	0.127389	0.0953722	eukaryotic initiation factor 4A-II isoform X1	13	[illegible]	[illegible]
Acrdi.XM_015901442.1/1-1203 | Monca.augustus.g6348.t1/1-1200	0.105919	0.0953692	DNA excision repair ERCC-8			
Acrdi.XM_015900494.1/1-4845 | Monca.augustus.g26470.t1/1-5841	0.11794	0.0953456	rapamycin-insensitive companion of mTOR isoform X1	12	[illegible]	[illegible]
Acrdi.XM_015907713.1/1-1548 | Monca.augustus.g55822.t1/1-2262	0.154322	0.0953444	adhesion G- coupled receptor D1-like isoform X2	3	[illegible]	[illegible]
Acrdi.XM_015917197.1/1-1209 | Monca.augustus.g23592.t1/1-1005	0.503452	0.0953071	PREDICTED: uncharacterized protein LOC107350934			
Acrdi.XM_015903710.1/1-4071 | Monca.adi2mcaRNA13151_R0/1-4137	0.122996	0.0952889	neuralized 4 isoform X3			
Acrdi.XM_015899845.1/1-810 | Monca.augustus.g52538.t1/1-993	0.125175	0.095166	PREDICTED: uncharacterized protein LOC107334882 isoform X1			
Acrdi.XM_015913632.1/1-1212 | Monca.adi2mcaRNA2388_R0/1-1227	0.0901345	0.0951597	histone-lysine N-methyltransferase PRDM6	2	[illegible]	[illegible]
Acrdi.XM_015905310.1/1-3192 | Monca.augustus.g42656.t1/1-3276	0.129509	0.0951512	plasma membrane calcium-transporting ATPase 2 isoform X1	6	[illegible]	[illegible]
Acrdi.XM_015920992.1/1-2037 | Monca.adi2mcaRNA32303_R3/1-1887	0.0943195	0.095134	WD repeat-containing 87			
Acrdi.XM_015898422.1/1-549 | Monca.augustus.g43980.t1/1-573	0.201684	0.095126	28S ribosomal mitochondrial			
Acrdi.XM_015909113.1/1-753 | Monca.augustus.g67742.t1/1-1020	0.158822	0.09511	sepiapterin reductase	4	[illegible]	[illegible]
Acrdi.XM_015901083.1/1-408 | Monca.adi2mcaRNA10314_R0/1-408	0.0926269	0.0950922	60S ribosomal L27	13	[illegible]	[illegible]
Acrdi.XM_015905611.1/1-5928 | Monca.adi2mcaRNA15305_R0/1-5652	0.123924	0.095079	mediator of RNA polymerase II transcription subunit 12	6	[illegible]	[illegible]
Acrdi.XM_015918544.1/1-456 | Monca.augustus.g15353.t1/1-525	0.0847649	0.0950763	ADP-ribosylation factor 2-binding	7	[illegible]	[illegible]
Acrdi.XM_015917599.1/1-1488 | Monca.augustus.g9235.t1/1-1548	0.102928	0.095071	disrupted in renal carcinoma 2 homolog	2	[illegible]	[illegible]
Acrdi.XM_015919834.1/1-930 | Monca.adi2mcaRNA31050_R0/1-528	0.118606	0.0950649	malectin-A	1	C:GO:0016020	[illegible]
Acrdi.XM_015896605.1/1-705 | Monca.augustus.g1591.t1/1-414	0.0665649	0.0950117	U6 snRNA-associated Sm LSm1	4	[illegible]	[illegible]
Acrdi.XM_015918632.1/1-1128 | Monca.augustus.g33665.t1/1-1677	0.108536	0.0950055	Xaa-Pro dipeptidase	3	[illegible]	[illegible]
Acrdi.XM_015899410.1/1-1839 | Monca.augustus.g37207.t1/1-1995	0.14821	0.0950023	Acetoacetyl- synthetase	4	[illegible]	[illegible]
Acrdi.XM_015924861.1/1-438 | Monca.augustus.g37687.t1/1-447	0.137735	0.0950019	Transmembrane 18	2	[illegible]	[illegible]
Acrdi.XM_015909418.1/1-1353 | Monca.adi2mcaRNA19517_R0/1-1320	0.110487	0.0950007	cereblon isoform X1	2	[illegible]	[illegible]
Acrdi.XM_015899291.1/1-1485 | Monca.augustus.g40310.t1/1-2970	0.0886511	0.0949924	Thioredoxin domain-containing 12	2	[illegible]	[illegible]
Acrdi.XM_015922558.1/1-3489 | Monca.augustus.g54973.t1/1-3363	0.114049	0.0949663	CLEC16A isoform X1			
Acrdi.XM_015910856.1/1-2775 | Monca.augustus.g70017.t1/1-2778	0.13781	0.0949621	transcription intermediary factor 1-alpha-like	2	[illegible]	[illegible]
Acrdi.XM_015906341.1/1-1509 | Monca.augustus.g45342.t1/1-1506	0.106909	0.0949371	cholesterol 24-hydroxylase-like	1	F:GO:0005488	[illegible]
Acrdi.XM_015909963.1/1-756 | Monca.augustus.g44404.t1/1-1011	0.137212	0.094931	Quinone oxidoreductase PIG3	3	[illegible]	[illegible]
Acrdi.XM_015916410.1/1-2829 | Monca.adi2mcaRNA27218_R0/1-2724	0.102212	0.0949181	solute carrier family 12 member 2-like	3	[illegible]	[illegible]
Acrdi.XM_015893309.1/1-3684 | Monca.augustus.g41931.t1/1-3837	0.118326	0.0949127	Catenin alpha-2	3	[illegible]	[illegible]
Acrdi.XM_015915036.1/1-1050 | Monca.augustus.g63346.t1/1-1068	0.123738	0.0948209	dopamine receptor 2-like	3	[illegible]	[illegible]
Acrdi.XM_015912566.1/1-549 | Monca.adi2mcaRNA22940_R2/1-513	0.394943	0.0948207	enzymatic poly	3	[illegible]	[illegible]
Acrdi.XM_015909380.1/1-741 | Monca.augustus.g38615.t1/1-990	0.0923185	0.0947786	Nuclear migration nudC	2	[illegible]	[illegible]
Acrdi.XM_015902996.1/1-705 | Monca.augustus.g68838.t1/1-705	0.122405	0.094778	Thioredoxin domain-containing	2	[illegible]	[illegible]
Acrdi.XM_015913050.1/1-957 | Monca.augustus.g45142.t1/1-951	0.119822	0.0947564	mitochondrial amidoxime-reducing component 1-like	5	[illegible]	[illegible]
Acrdi.XM_015910812.1/1-1734 | Monca.augustus.g15805.t1/1-2307	0.0798159	0.0947543	Soluble guanylate cyclase 88E	6	[illegible]	[illegible]
Acrdi.XM_015911431.1/1-807 | Monca.augustus.g69042.t1/1-1308	0.117835	0.0947501	G1 S-specific cyclin-E1	1	C:GO:0005634	[illegible]
Acrdi.XM_015924258.1/1-2190 | Monca.augustus.g39156.t1/1-2190	0.102928	0.0947214	Chondroitin sulfate synthase 1	2	[illegible]	[illegible]
Acrdi.XM_015899618.1/1-1248 | Monca.adi2mcaRNA8778_R0/1-1293	0.110245	0.094702	F-box WD repeat-containing 7-like	2	[illegible]	[illegible]
Acrdi.XM_015900675.1/1-1065 | Monca.augustus.g68350.t1/1-1065	0.104108	0.0946826	PREDICTED: uncharacterized protein LOC107335641			
Acrdi.XM_015895863.1/1-1071 | Monca.augustus.g23191.t1/1-1074	0.105994	0.0946815	Wnt-7b isoform X1	41	[illegible]	[illegible]
Acrdi.XM_015906669.1/1-858 | Monca.adi2mcaRNA16431_R0/1-774	0.0926316	0.0946692	DNA-(apurinic or apyrimidinic site) lyase	10	[illegible]	[illegible]
Acrdi.XM_015923020.1/1-555 | Monca.augustus.g46062.t1/1-1419	0.120964	0.0946683	cytochrome b-c1 complex subunit mitochondrial-like	4	[illegible]	[illegible]
Acrdi.XM_015903848.1/1-768 | Monca.augustus.g31274.t1/1-927	1.47082	0.0946644	PREDICTED: uncharacterized protein LOC107338610			
Acrdi.XM_015908534.1/1-783 | Monca.augustus.g46686.t1/1-1776	0.0910973	0.0946619	bifunctional lysine-specific demethylase and histidyl-hydroxylase MINA	1	C:GO:0044424	[illegible]
Acrdi.XM_015911484.1/1-333 | Monca.augustus.g53637.t1/1-327	0.161001	0.0946443	bolA 3	2	[illegible]	[illegible]
Acrdi.XM_015914425.1/1-1023 | Monca.augustus.g9580.t1/1-1026	0.092671	0.0946329	ELAV 3 isoform X5	3	[illegible]	[illegible]
Acrdi.XM_015921970.1/1-345 | Monca.augustus.g34117.t1/1-537	0.744149	0.0945973	G2 M phase-specific E3 ubiquitin- ligase-like	2	[illegible]	[illegible]
Acrdi.XM_015897019.1/1-381 | Monca.augustus.g30100.t1/1-363	0.110907	0.0945931	PREDICTED: uncharacterized protein LOC107332290			
Acrdi.XM_015915740.1/1-417 | Monca.augustus.g55310.t1/1-417	0.153175	0.0945773	ubiquitin-conjugating enzyme E2 variant 2			
Acrdi.XM_015912218.1/1-900 | Monca.augustus.g22427.t1/1-900	0.156982	0.0945712	kidney mitochondrial carrier 1-like	5	[illegible]	[illegible]
Acrdi.XM_015911016.1/1-705 | Monca.adi2mcaRNA21242_R4/1-630	0.479411	0.0945643	Pro-Pol poly			
Acrdi.XM_015902235.1/1-861 | Monca.adi2mcaRNA11574_R1/1-894	0.164981	0.0945623	meiotic recombination SPO11	5	[illegible]	[illegible]
Acrdi.XM_015914379.1/1-1416 | Monca.augustus.g67304.t1/1-2757	0.136536	0.094554	DNA ligase 4-like	9	[illegible]	[illegible]
Acrdi.XM_015899343.1/1-690 | Monca.augustus.g64429.t1/1-690	0.096598	0.0945162	Homeobox EMX1	6	[illegible]	[illegible]
Acrdi.XM_015917179.1/1-1983 | Monca.augustus.g31456.t1/1-1980	0.107223	0.0945098	islet cell autoantigen 1 isoform X1	3	[illegible]	[illegible]
Acrdi.XM_015906254.1/1-342 | Monca.augustus.g26509.t1/1-654	0.0670001	0.0945042	vesicle transport through interaction with t-SNAREs homolog 1A isofor	13	[illegible]	[illegible]
Acrdi.XM_015903139.1/1-936 | Monca.augustus.g70484.t1/1-1476	0.112397	0.0945032	uncharacterized aminotransferase -like	3	[illegible]	[illegible]
Acrdi.XM_015910020.1/1-726 | Monca.augustus.g58888.t1/1-906	0.0929486	0.0944905	tRNA (cytidine(32) guanosine(34)-2 -O)-methyltransferase	4	[illegible]	[illegible]
Acrdi.XM_015903841.1/1-630 | Monca.augustus.g57993.t1/1-420	0.742555	0.0944873	RNA-directed DNA polymerase			
Acrdi.XM_015898586.1/1-2079 | Monca.augustus.g34600.t1/1-1866	0.113834	0.0944618	acetolactate synthase	5	[illegible]	[illegible]
Acrdi.XM_015899421.1/1-1356 | Monca.augustus.g46722.t1/1-1356	0.150452	0.0944457	origin recognition complex subunit 5	3	[illegible]	[illegible]
Acrdi.XM_015909462.1/1-1239 | Monca.augustus.g47024.t1/1-2616	0.440772	0.0944359	serine threonine- phosphatase 6 regulatory ankyrin repeat subunit C-like			
Acrdi.XM_015894500.1/1-1158 | Monca.adi2mcaRNA4109_R0/1-1044	0.139082	0.0944323	splicing factor U2AF 65 kDa subunit-like	8	[illegible]	[illegible]
Acrdi.XM_015902367.1/1-861 | Monca.augustus.g37589.t1/1-918	0.127261	0.0944262	WD repeat domain-containing 83	1	C:GO:0044424	[illegible]
Acrdi.XM_015895607.1/1-2787 | Monca.adi2mcaRNA4492_R0/1-2886	0.13214	0.0944247	membrane-associated guanylate WW and PDZ domain-containing 3-like isoform X2			
Acrdi.XM_015918073.1/1-309 | Monca.augustus.g34639.t1/1-615	0.10452	0.094418	biogenesis of lysosome-related organelles complex 1 subunit 4-like	2	[illegible]	[illegible]
Acrdi.XM_015899923.1/1-834 | Monca.augustus.g57215.t1/1-933	0.115516	0.0944093	uracil-DNA glycosylase-like	4	[illegible]	[illegible]
Acrdi.XM_015919321.1/1-1077 | Monca.adi2mcaRNA30500_R0/1-1083	0.138243	0.0943868	xylulose kinase isoform X3	4	[illegible]	[illegible]
Acrdi.XM_015906876.1/1-1197 | Monca.augustus.g32953.t1/1-1398	0.135161	0.0943575	IQ domain-containing D			
Acrdi.XM_015893019.1/1-693 | Monca.adi2mcaRNA39060_R1/1-657	0.33626	0.0943347	RNA-directed DNA polymerase from transposon BS	5	[illegible]	[illegible]
Acrdi.XM_015912746.1/1-2319 | Monca.adi2mcaRNA23140_R0/1-2316	0.122926	0.0943325	actin actin	1	F:GO:0005524	[illegible]
Acrdi.XM_015925115.1/1-684 | Monca.augustus.g53758.t1/1-1071	0.132081	0.094318	1-acylglycerol-3-phosphate O-acyltransferase ABHD5	1	F:GO:0052689	[illegible]
Acrdi.XM_015913063.1/1-786 | Monca.augustus.g25071.t1/1-981	0.122956	0.0943129	PREDICTED: uncharacterized protein LOC107347183	5	[illegible]	[illegible]
Acrdi.XM_015906958.1/1-2070 | Monca.augustus.g49148.t1/1-2067	0.134517	0.0942709	bicaudal D-related homolog			
Acrdi.XM_015893778.1/1-1653 | Monca.augustus.g32011.t1/1-1713	0.46303	0.0942678	PREDICTED: uncharacterized protein LOC107329045			
Acrdi.XM_015917590.1/1-633 | Monca.augustus.g64957.t1/1-1032	0.407088	0.0942593	Transposon Ty3-G Gag-Pol poly			
Acrdi.XM_015921827.1/1-768 | Monca.augustus.g16478.t1/1-2772	0.093049	0.0942442	probable 2-oxoglutarate dehydrogenase E1 component mitochondrial	2	[illegible]	[illegible]
Acrdi.XM_015911077.1/1-6828 | Monca.augustus.g55127.t1/1-8493	0.119377	0.0942202	ectopic P granules 5 homolog	4	[illegible]	[illegible]
Acrdi.XM_015897279.1/1-4995 | Monca.augustus.g4217.t1/1-3036	0.106843	0.0941606	mediator of RNA polymerase II transcription subunit 23 isoform X2			
Acrdi.XM_015907672.1/1-672 | Monca.augustus.g29954.t1/1-1062	0.116135	0.094159	zinc finger GLIS2-like	6	[illegible]	[illegible]
Acrdi.XM_015909019.1/1-2091 | Monca.augustus.g38376.t1/1-2952	0.126595	0.0941488	122 kDa TMEM16	3	[illegible]	[illegible]
Acrdi.XM_015917294.1/1-672 | Monca.augustus.g48822.t1/1-960	0.0972841	0.094148	flavin reductase (NADPH)-like			
Acrdi.XM_015895968.1/1-2112 | Monca.augustus.g22585.t1/1-2133	0.09883	0.0941454	Mitochondrial intermediate peptidase	5	[illegible]	[illegible]
Acrdi.XM_015915183.1/1-897 | Monca.augustus.g29749.t1/1-894	0.132954	0.0941426	serine threonine- phosphatase CPPED1 isoform X1	1	F:GO:0016787	[illegible]
Acrdi.XM_015911891.1/1-1107 | Monca.augustus.g7933.t1/1-1104	0.122984	0.0941149	prostaglandin E synthase 2-like	1	P:GO:0044699	[illegible]
Acrdi.XM_015903646.1/1-1002 | Monca.augustus.g37322.t1/1-1002	0.123348	0.0941085	Hydroxylysine kinase			
Acrdi.XM_015921520.1/1-714 | Monca.augustus.g42438.t1/1-636	0.157922	0.0940924	homologous-pairing 2 homolog			
Acrdi.XM_015903776.1/1-3216 | Monca.augustus.g61998.t1/1-4314	0.122826	0.0940829	Coiled-coil domain-containing 171			
Acrdi.XM_015896202.1/1-768 | Monca.augustus.g23021.t1/1-768	0.125257	0.0940779	Origin recognition complex subunit 6	3	[illegible]	[illegible]
Acrdi.XM_015903210.1/1-1377 | Monca.augustus.g51695.t1/1-1377	0.108646	0.0940463	F-box LRR-repeat 7			
Acrdi.XM_015913563.1/1-999 | Monca.augustus.g22326.t1/1-1290	0.0949064	0.0940426	tubulin-specific chaperone cofactor E	2	[illegible]	[illegible]
Acrdi.XM_015913534.1/1-480 | Monca.augustus.g22335.t1/1-687	0.105151	0.0940246	transmembrane 147 isoform X2	1	C:GO:0016020	[illegible]
Acrdi.XM_015905536.1/1-336 | Monca.augustus.g28247.t1/1-492	0.118289	0.0940147	PREDICTED: uncharacterized protein C1orf194 homolog	3	[illegible]	[illegible]
Acrdi.XM_015906167.1/1-6150 | Monca.augustus.g21704.t1/1-7065	0.126621	0.0939861	Tetratricopeptide repeat 28	3	[illegible]	[illegible]
Acrdi.XM_015923819.1/1-3822 | Monca.adi2mcaRNA35522_R0/1-3447	0.109608	0.0939811	N-acetylated-alpha-linked acidic dipeptidase			
Acrdi.XM_015904614.1/1-1245 | Monca.augustus.g7187.t1/1-2010	0.101116	0.0939795	transient receptor potential cation channel subfamily V member 5-like	5	[illegible]	[illegible]
Acrdi.XM_015921249.1/1-1401 | Monca.augustus.g42835.t1/1-1563	0.072397	0.0939758	DENND6A-like isoform X1	3	[illegible]	[illegible]
Acrdi.XM_015905264.1/1-948 | Monca.adi2mcaRNA14894_R0/1-903	0.142616	0.0939675	mitochondrial ornithine transporter 1-like	3	[illegible]	[illegible]
Acrdi.XM_015921488.1/1-906 | Monca.augustus.g29328.t1/1-1017	0.105443	0.0939672	THO complex subunit 6 homolog	2	[illegible]	[illegible]
Acrdi.XM_015907259.1/1-450 | Monca.augustus.g33142.t1/1-915	0.128776	0.0939562	neuron navigator 3-like	1	F:GO:0003779	[illegible]
Acrdi.XM_015900374.1/1-1842 | Monca.augustus.g54191.t1/1-1842	0.109028	0.0939347	proline dehydrogenase mitochondrial-like	3	[illegible]	[illegible]
Acrdi.XM_015904499.1/1-1119 | Monca.augustus.g33594.t1/1-1125	0.134025	0.0938905	FAM228B-like isoform X1			
Acrdi.XM_015918109.1/1-1026 | Monca.augustus.g43951.t1/1-1044	0.116666	0.0938867	paired box Pax-3 isoform X3	5	[illegible]	[illegible]
Acrdi.XM_015922167.1/1-2268 | Monca.augustus.g63279.t1/1-3129	0.11078	0.0938808	polyphosphoinositide phosphatase isoform X2	1	F:GO:0042578	[illegible]
Acrdi.XM_015920366.1/1-1485 | Monca.adi2mcaRNA31656_R0/1-1236	0.140798	0.0938459	tafazzin isoform X2	10	[illegible]	[illegible]
Acrdi.XM_015900549.1/1-708 | Monca.augustus.g26473.t1/1-1095	0.138468	0.0938323	RNA 3 -terminal phosphate cyclase	5	[illegible]	[illegible]
Acrdi.XM_015900692.1/1-1188 | Monca.augustus.g34852.t1/1-1620	0.114394	0.0938285	Acetyl-coenzyme A transporter 1	6	[illegible]	[illegible]
Acrdi.XM_015896544.1/1-765 | Monca.augustus.g29587.t1/1-765	0.147048	0.0938251	Krueppel-like factor 5	2	[illegible]	[illegible]
Acrdi.XM_015898728.1/1-381 | Monca.augustus.g2537.t1/1-384	0.188207	0.0938087	Phosphatidylinositol N-acetylglucosaminyltransferase subunit P	4	[illegible]	[illegible]
Acrdi.XM_015910693.1/1-2091 | Monca.augustus.g35882.t1/1-2058	0.126842	0.0938071	Ubiquitin-like modifier-activating enzyme ATG7	12	[illegible]	[illegible]
Acrdi.XM_015906311.1/1-1281 | Monca.augustus.g23858.t1/1-1491	0.110385	0.0938064	F-box-like WD repeat-containing TBL1X	4	[illegible]	[illegible]
Acrdi.XM_015901868.1/1-1248 | Monca.augustus.g21289.t1/1-1251	0.0662589	0.0937939	Ras-related and estrogen-regulated growth inhibitor	6	[illegible]	[illegible]
Acrdi.XM_015917817.1/1-1416 | Monca.augustus.g11189.t1/1-1419	0.151736	0.0937132	transmembrane 180	2	[illegible]	[illegible]

Acrdi.XM_015898718.1/1-762 | Monca.augustus.g54235.t1/1-828	0.132616	0.0937047	General transcription factor IIF subunit 2	8	[illegible]
Acrdi.XM_015903319.1/1-1083 | Monca.augustus.g60210.t1/1-1086	0.103855	0.0937006	T-box transcription factor TBX20	5	[illegible]
Acrdi.XM_015908003.1/1-1017 | Monca.augustus.g43118.t1/1-1014	0.0839636	0.0936682	neuropeptide FF receptor 2-like	3	[illegible]
Acrdi.XM_015900473.1/1-2094 | Monca.augustus.g68181.t1/1-2100	0.1044	0.0936414	calcium-binding mitochondrial carrier Aralar1 isoform X2		
Acrdi.XM_015911990.1/1-1494 | Monca.adi2mcaRNA22261_R0/1-1488	0.121118	0.0936351	THAP domain-containing 3		
Acrdi.XM_015920693.1/1-738 | Monca.augustus.g50019.t1/1-1176	0.582029	0.0936211	PREDICTED: uncharacterized protein LOC107354237		
Acrdi.XM_015893170.1/1-465 | Monca.augustus.g61988.t1/1-1179	0.0867282	0.0936123	DDB1- and CUL4-associated factor		
Acrdi.XM_015903158.1/1-960 | Monca.adi2mcaRNA12524_R0/1-1008	0.0944029	0.0935902	mitochondrial thiamine pyrophosphate carrier	6	[illegible]
Acrdi.XM_015902507.1/1-1131 | Monca.augustus.g3506.t1/1-798	0.815968	0.0935567	glycosyl hydrolase family 5	1	[illegible]
Acrdi.XM_015915007.1/1-5328 | Monca.augustus.g63358.t1/1-5985	0.107897	0.0935496	citron Rho-interacting kinase isoform X2	8	[illegible]
Acrdi.XM_015897295.1/1-594 | Monca.augustus.g56885.t1/1-864	0.105407	0.0935392	GATA zinc finger domain-containing 15-like isoform X1		
Acrdi.XM_015919074.1/1-1524 | Monca.augustus.g58064.t1/1-1518	0.0827661	0.0935086	neuronal acetylcholine receptor subunit alpha-10-like	5	[illegible]
Acrdi.XM_015918409.1/1-471 | Monca.augustus.g23510.t1/1-1260	0.115139	0.093508	RNA-binding Musashi homolog 2-like isoform X2	2	[illegible]
Acrdi.XM_015911773.1/1-603 | Monca.augustus.g60096.t1/1-609	0.421922	0.0934808	PREDICTED: uncharacterized protein LOC107346009		
Acrdi.XM_015908451.1/1-693 | Monca.augustus.g33942.t1/1-693	0.13824	0.0934766	peroxisomal membrane 11B-like	3	[illegible]
Acrdi.XM_015913255.1/1-525 | Monca.augustus.g67362.t1/1-627	0.123279	0.0934674	transmembrane emp24 domain-containing 7-like	1	[illegible]
Acrdi.XM_015920884.1/1-1320 | Monca.augustus.g30095.t1/1-1323	0.0836304	0.0934421	kinase C and casein kinase substrate in neurons 2	4	[illegible]
Acrdi.XM_015914144.1/1-1482 | Monca.adi2mcaRNA24656_R0/1-1539	0.0893911	0.0934413	cleavage and polyadenylation specificity factor subunit 6 isoform X2	2	[illegible]
Acrdi.XM_015897012.1/1-525 | Monca.adi2mcaRNA5899_R7/1-495	0.442023	0.0934381	P2X purinoceptor 7-like		
Acrdi.XM_015925223.1/1-552 | Monca.adi2mcaRNA3704_R0/1-528	0.162132	0.0934169	E3 ubiquitin- ligase ZNRF2	3	[illegible]
Acrdi.XM_015897546.1/1-1302 | Monca.augustus.g7251.t1/1-1338	0.176911	0.0933692	F-box only 47-like		
Acrdi.XM_015894142.1/1-1380 | Monca.adi2mcaRNA4060_R1/1-1383	0.130257	0.0933403	thiamine transporter 2 isoform X1	4	[illegible]
Acrdi.XM_015914979.1/1-801 | Monca.augustus.g42434.t1/1-762	0.092289	0.0933324	dnaJ homolog subfamily C member 9	6	[illegible]
Acrdi.XM_015919605.1/1-657 | Monca.augustus.g7141.t1/1-678	0.120096	0.0933309	PREDICTED: uncharacterized protein LOC107353275	2	[illegible]
Acrdi.XM_015898362.1/1-2418 | Monca.augustus.g58536.t1/1-3951	0.13637	0.0932871	helicase SKI2W-like	2	[illegible]
Acrdi.XM_015911395.1/1-783 | Monca.augustus.g13221.t1/1-825	0.135477	0.0932506	PREDICTED: uncharacterized protein F13E9.13, mitochondrial-like	2	[illegible]
Acrdi.XM_015904481.1/1-912 | Monca.adi2mcaRNA13978_R1/1-948	0.599621	0.0932295	PREDICTED: uncharacterized protein LOC107339221 isoform X1		
Acrdi.XM_015915023.1/1-609 | Monca.adi2mcaRNA25682_R1/1-609	0.134316	0.0932289	Glycolipid transfer	8	[illegible]
Acrdi.XM_015896522.1/1-1386 | Monca.augustus.g1581.t1/1-1410	0.124876	0.0932185	argininosuccinate lyase	4	[illegible]
Acrdi.XM_015921997.1/1-1824 | Monca.adi2mcaRNA33384_R0/1-1824	0.116976	0.0932056	Sodium channel and clathrin linker 1		
Acrdi.XM_015900664.1/1-1428 | Monca.adi2mcaRNA9855_R0/1-1437	0.13684	0.0931629	sestrin-1 isoform X3	2	[illegible]
Acrdi.XM_015901043.1/1-1410 | Monca.augustus.g57612.t1/1-1410	0.151163	0.0931596	malate dehydrogenase 1B	4	[illegible]
Acrdi.XM_015915620.1/1-1533 | Monca.augustus.g36642.t1/1-1521	0.108044	0.0931469	GDP-Man:Man(3) c(2)-PP-Dol alpha-1,2-mannosyltransferase	1	[illegible]
Acrdi.XM_015916306.1/1-954 | Monca.augustus.g68769.t1/1-939	0.131915	0.0931167	Ankyrin repeat and SOCS box 13		
Acrdi.XM_015910446.1/1-1842 | Monca.augustus.g60941.t1/1-2190	0.137659	0.0931103	SMG7 isoform X2	8	[illegible]
Acrdi.XM_015913737.1/1-750 | Monca.augustus.g21660.t1/1-804	0.100753	0.0930641	2-methoxy-6-polyprenyl-1,4-benzoquinol mitochondrial	4	[illegible]
Acrdi.XM_015913554.1/1-378 | Monca.augustus.g22336.t1/1-1017	0.128846	0.0930601	borealin-like	4	[illegible]
Acrdi.XM_015915919.1/1-1008 | Monca.augustus.g60998.t1/1-1026	0.118319	0.0930533	zinc finger -like 1	4	[illegible]
Acrdi.XM_015917691.1/1-6300 | Monca.augustus.g42288.t1/1-6714	0.122552	0.0930103	afadin-like isoform X2	1	[illegible]
Acrdi.XM_015894443.1/1-2586 | Monca.augustus.g15546.t1/1-7566	0.114005	0.0930068	poly(ADP-ribose) polymerase pme-5-like	1	[illegible]
Acrdi.XM_015913086.1/1-552 | Monca.augustus.g25049.t1/1-519	0.085832	0.0930025	myosin regulatory light polypeptide 9-like	1	[illegible]
Acrdi.XM_015893923.1/1-957 | Monca.augustus.g50873.t1/1-957	0.106738	0.0929998	GTP-binding Rit1	8	[illegible]
Acrdi.XM_015894178.1/1-654 | Monca.augustus.g56707.t1/1-1464	0.0972837	0.0929991	histone acetyltransferase KAT6B isoform X3	5	[illegible]
Acrdi.XM_015907767.1/1-807 | Monca.augustus.g16272.t1/1-2553	0.120948	0.0929918	beta actin	1	[illegible]
Acrdi.XM_015919600.1/1-1779 | Monca.augustus.g37835.t1/1-792	0.0737331	0.09296	heterogeneous nuclear ribonucleo A1-like isoform X1	2	[illegible]
Acrdi.XM_015900153.1/1-2565 | Monca.augustus.g62856.t1/1-2703	0.112176	0.0929387	WD repeat-containing 36	6	[illegible]
Acrdi.XM_015920520.1/1-1707 | Monca.augustus.g25709.t1/1-1707	0.140046	0.0929373	sodium-coupled neutral amino acid transporter 9 homolog	4	[illegible]
Acrdi.XM_015917821.1/1-1041 | Monca.augustus.g11193.t1/1-1041	0.104448	0.0928425	CD2 antigen cytoplasmic tail-binding 2-like	1	[illegible]
Acrdi.XM_015904937.1/1-1500 | Monca.augustus.g55922.t1/1-1485	0.104986	0.0928269	KDEL motif-containing 1	4	[illegible]
Acrdi.XM_015901127.1/1-2226 | Monca.augustus.g62604.t1/1-3324	0.0909622	0.0928226	guanine nucleotide exchange factor DBS-like	6	[illegible]
Acrdi.XM_015902286.1/1-981 | Monca.augustus.g35099.t1/1-843	0.0804805	0.0927886	poly(U)-specific endoribonuclease-B-like	2	[illegible]
Acrdi.XM_015908777.1/1-882 | Monca.augustus.g24447.t1/1-1428	0.0801402	0.0927704	Vesicular GABA transporter	4	[illegible]
Acrdi.XM_015921146.1/1-5151 | Monca.adi2mcaRNA32476_R0/1-4584	0.109955	0.0927689	dedicator of cytokinesis 7 isoform X7		
Acrdi.XM_015896831.1/1-2610 | Monca.adi2mcaRNA5785_R0/1-2442	0.104269	0.0927645	arf-GAP with coiled- ANK repeat and PH domain-containing 3 isoform	2	[illegible]
Acrdi.XM_015898068.1/1-1092 | Monca.augustus.g41210.t1/1-1671	0.0940948	0.0927153	PHD finger 10 isoform X1	2	[illegible]
Acrdi.XM_015913782.1/1-1218 | Monca.augustus.g45550.t1/1-2286	0.105783	0.0927026	Collagen alpha-1(XII) chain	5	[illegible]
Acrdi.XM_015900530.1/1-1911 | Monca.augustus.g37061.t1/1-2895	0.130335	0.0926682	mothers against decapentaplegic homolog 4-like	6	[illegible]
Acrdi.XM_015891809.1/1-1086 | Monca.augustus.g38660.t1/1-1092	0.120642	0.0926505	cytosolic acyl coenzyme A thioester hydrolase-like isoform X1	14	[illegible]
Acrdi.XM_015910833.1/1-729 | Monca.adi2mcaRNA21019_R0/1-732	0.156843	0.0926152	tetraspanin-4 isoform X2	2	[illegible]
Acrdi.XM_015904070.1/1-438 | Monca.augustus.g53771.t1/1-630	0.836829	0.0926136	PREDICTED: uncharacterized protein LOC107338819		
Acrdi.XM_015898140.1/1-1272 | Monca.augustus.g39429.t1/1-1254	0.117775	0.0926023	potassium voltage-gated channel subfamily A member 1	10	[illegible]
Acrdi.XM_015891865.1/1-813 | Monca.augustus.g16752.t1/1-999	0.358031	0.0926019	PREDICTED: uncharacterized protein LOC107327115		
Acrdi.XM_015912263.1/1-753 | Monca.augustus.g28330.t1/1-1146	0.0971069	0.0925895	Translocation SEC62	1	[illegible]
Acrdi.XM_015892544.1/1-1839 | Monca.augustus.g48666.t1/1-4203	0.126169	0.0925784	Rab3 GTPase-activating non-catalytic subunit	1	[illegible]
Acrdi.XM_015920249.1/1-2199 | Monca.adi2mcaRNA31501_R0/1-1983	0.110157	0.0925646	endothelin-converting enzyme 1-like isoform X1	2	[illegible]
Acrdi.XM_015925160.1/1-1818 | Monca.augustus.g51644.t1/1-2133	0.115395	0.0925452	ADP-ribosylation factor-binding GGA1 isoform X1		
Acrdi.XM_015903214.1/1-2502 | Monca.augustus.g51699.t1/1-4287	0.106233	0.0924947	nitric oxide synthase	8	[illegible]
Acrdi.XM_015903888.1/1-1620 | Monca.augustus.g30302.t1/1-1611	0.0863162	0.0924941	glucose-6-phosphate 1-dehydrogenase	4	[illegible]
Acrdi.XM_015911337.1/1-9690 | Monca.augustus.g35261.t1/1-11157	0.13026	0.0924873	serine threonine- kinase SMG1-like	5	[illegible]
Acrdi.XM_015925130.1/1-591 | Monca.augustus.g25767.t1/1-555	0.320814	0.092452	probable ATP-dependent DNA helicase	6	[illegible]
Acrdi.XM_015892281.1/1-1248 | Monca.augustus.g13815.t1/1-1386	0.1111	0.0924425	Translation initiation factor eIF-2B subunit gamma	2	[illegible]
Acrdi.XM_015899096.1/1-3510 | Monca.adi2mcaRNA894_R0/1-3495	0.12899	0.0924398	phospholipid-transporting ATPase ID-like	9	[illegible]
Acrdi.XM_015913471.1/1-996 | Monca.adi2mcaRNA23911_R1/1-945	0.102968	0.0924389	ZP domain-containing -like	6	[illegible]
Acrdi.XM_015919152.1/1-1008 | Monca.augustus.g35144.t1/1-1260	0.0876178	0.0924236	Serine threonine- kinase Nek2	3	[illegible]
Acrdi.XM_015905592.1/1-678 | Monca.augustus.g15266.t1/1-1932	0.101057	0.0924093	nuclear pore glyco p62	6	[illegible]
Acrdi.XM_015907319.1/1-1137 | Monca.augustus.g63472.t1/1-1599	0.10793	0.0924066	methylmalonate-semialdehyde dehydrogenase [acylating] mitochondr	11	[illegible]
Acrdi.XM_015899402.1/1-8427 | Monca.augustus.g37237.t1/1-8961	0.122656	0.0924022	helicase domino isoform X2	1	[illegible]
Acrdi.XM_015914799.1/1-2412 | Monca.augustus.g35042.t1/1-2523	0.119319	0.0923971	two pore calcium channel 1-like	6	[illegible]
Acrdi.XM_015919327.1/1-711 | Monca.augustus.g10290.t1/1-804	0.091913	0.0923501	Thymidine kinase mitochondrial	3	[illegible]
Acrdi.XM_015925326.1/1-1179 | Monca.adi2mcaRNA37267_R4/1-933	0.577491	0.0923444	Zinc finger MYM-type 3		
Acrdi.XM_015905475.1/1-3417 | Monca.augustus.g28241.t1/1-3756	0.0909377	0.0923371	calcium-dependent secretion activator 1 isoform X7		
Acrdi.XM_015896759.1/1-1128 | Monca.augustus.g13439.t1/1-1125	0.1039	0.0923317	soluble calcium-activated nucleotidase 1 isoform X2	4	[illegible]
Acrdi.XM_015910859.1/1-1125 | Monca.augustus.g70021.t1/1-1191	0.0972262	0.0923119	Choline kinase alpha	9	[illegible]
Acrdi.XM_015902557.1/1-1197 | Monca.adi2mcaRNA11892_R0/1-1209	0.141622	0.0923117	dachshund homolog 1-like		
Acrdi.XM_015908099.1/1-1107 | Monca.augustus.g33235.t1/1-1068	0.118959	0.0923063	AAR2 homolog	1	[illegible]
Acrdi.XM_015899467.1/1-921 | Monca.augustus.g48263.t1/1-918	0.128785	0.0922795	microtubule-associated RP EB family member 3 isoform X1	1	[illegible]
Acrdi.XM_015907666.1/1-3402 | Monca.augustus.g29936.t1/1-3540	0.110092	0.0922629	KIBRA isoform X2		
Acrdi.XM_015903161.1/1-360 | Monca.augustus.g19995.t1/1-360	0.153464	0.0922519	C10	4	[illegible]
Acrdi.XM_015921037.1/1-696 | Monca.augustus.g10689.t1/1-885	0.131977	0.092248	peroxisome biogenesis factor 2	2	[illegible]
Acrdi.XM_015893810.1/1-1860 | Monca.adi2mcaRNA39956_R0/1-1548	0.106358	0.0921791	UDP-glucose:glyco glucosyltransferase	3	[illegible]
Acrdi.XM_015923691.1/1-687 | Monca.augustus.g34967.t1/1-684	0.164038	0.092179	PREDICTED: uncharacterized protein LOC107357052		
Acrdi.XM_015923433.1/1-1761 | Monca.augustus.g32973.t1/1-1749	0.126052	0.0921774	rho GTPase-activating 44-like isoform X3	2	[illegible]
Acrdi.XM_015914788.1/1-1458 | Monca.adi2mcaRNA2679_R0/1-1368	0.147854	0.0921621	AP-5 complex subunit mu-1-like		
Acrdi.XM_015900332.1/1-1512 | Monca.augustus.g42582.t1/1-1509	0.112922	0.092154	Vacuole membrane 1	7	[illegible]
Acrdi.XM_015907934.1/1-1797 | Monca.augustus.g11085.t1/1-2025	0.0992886	0.0921525	exocyst complex component 7 isoform X6		
Acrdi.XM_015912167.1/1-1014 | Monca.augustus.g55530.t1/1-1014	0.138829	0.0921297	probable palmitoyltransferase ZDHHC16 isoform X1	1	[illegible]
Acrdi.XM_015914209.1/1-525 | Monca.augustus.g58350.t1/1-534	0.102306	0.0920813	Cytochrome b-245 light chain	26	[illegible]
Acrdi.XM_015908152.1/1-1191 | Monca.adi2mcaRNA18107_R0/1-924	0.274102	0.0920792	PREDICTED: uncharacterized protein LOC107342650		
Acrdi.XM_015895047.1/1-516 | Monca.augustus.g56218.t1/1-1059	0.0825458	0.0920671	LRP2-binding	1	[illegible]
Acrdi.XM_015925262.1/1-1233 | Monca.augustus.g1546.t1/1-1620	0.108864	0.0920615	glycine mitochondrial	3	[illegible]
Acrdi.XM_015914548.1/1-873 | Monca.augustus.g29129.t1/1-858	0.134481	0.0920395	Krueppel-like factor 15	2	[illegible]
Acrdi.XM_015909329.1/1-5040 | Monca.adi2mcaRNA1984_R1/1-4770	0.128465	0.0920356	unconventional myosin-IXa-like isoform X1	1	[illegible]
Acrdi.XM_015900373.1/1-1116 | Monca.adi2mcaRNA9535_R0/1-1083	0.122099	0.0920149	GDP-fucose O-fucosyltransferase 1	2	[illegible]
Acrdi.XM_015892404.1/1-465 | Monca.augustus.g67149.t1/1-633	0.0849258	0.0919963	synaptosomal-associated 25-like	12	[illegible]
Acrdi.XM_015919764.1/1-930 | Monca.augustus.g37136.t1/1-1146	1.2768	0.0919955	zinc finger 862-like		
Acrdi.XM_015920694.1/1-339 | Monca.augustus.g50020.t1/1-309	0.772384	0.0919922	PREDICTED: uncharacterized protein LOC107354238		
Acrdi.XM_015919052.1/1-837 | Monca.augustus.g56505.t1/1-831	0.110801	0.0919887	Leucine-rich repeat-containing 73		
Acrdi.XM_015909030.1/1-1692 | Monca.augustus.g38390.t1/1-1752	0.096305	0.0919519	Kelch 2	1	[illegible]
Acrdi.XM_015901303.1/1-555 | Monca.augustus.g43370.t1/1-948	0.0809756	0.0919509	PREDICTED: uncharacterized protein LOC107336239		
Acrdi.XM_015897169.1/1-639 | Monca.augustus.g31735.t1/1-1206	0.133893	0.0919406	ATP-sensitive inward rectifier potassium channel 12-like	4	[illegible]
Acrdi.XM_015901137.1/1-1281 | Monca.augustus.g1875.t1/1-1284	0.128161	0.0919148	Zinc finger 333	2	[illegible]
Acrdi.XM_015921506.1/1-1782 | Monca.adi2mcaRNA32866_R0/1-1785	0.116459	0.0918766	Sorting nexin-30	5	[illegible]
Acrdi.XM_015897406.1/1-903 | Monca.augustus.g51665.t1/1-903	0.135276	0.0918675	DCN1 3	1	[illegible]
Acrdi.XM_015907565.1/1-1362 | Monca.adi2mcaRNA17448_R1/1-1323	0.300863	0.0918568	PREDICTED: uncharacterized protein LOC107342111		
Acrdi.XM_015896674.1/1-927 | Monca.augustus.g37032.t1/1-1647	0.128473	0.091854	RNA-binding single-stranded-interacting 3-like		
Acrdi.XM_015894491.1/1-486 | Monca.augustus.g9945.t1/1-486	0.114004	0.0918476	Vesicle transport SFT2B	2	[illegible]
Acrdi.XM_015894001.1/1-2136 | Monca.augustus.g25180.t1/1-2493	0.0959902	0.0918247	chondroitin sulfate synthase 2-like	1	[illegible]
Acrdi.XM_015910769.1/1-1464 | Monca.augustus.g29678.t1/1-1821	0.0769439	0.0918117	keratin-associated 10-4-like isoform X2		
Acrdi.XM_015922952.1/1-1095 | Monca.adi2mcaRNA368_R0/1-798	0.0744856	0.0918051	tRNA-dihydrouridine(16 17) synthase [NAD(P)(+)]-like	2	[illegible]
Acrdi.XM_015907198.1/1-897 | Monca.augustus.g57676.t1/1-858	0.147349	0.0918039	probable E3 ubiquitin- ligase RNF217	7	[illegible]
Acrdi.XM_015922252.1/1-2817 | Monca.augustus.g8791.t1/1-3231	0.130077	0.0917754	importin-4	9	[illegible]
Acrdi.XM_015917695.1/1-3516 | Monca.augustus.g42282.t1/1-2787	0.114762	0.0917245	DNA mismatch repair Msh2	17	[illegible]
Acrdi.XM_015911241.1/1-1539 | Monca.augustus.g38101.t1/1-1539	0.125724	0.0917225	cytochrome p450 1A	4	[illegible]
Acrdi.XM_015912094.1/1-3045 | Monca.augustus.g60702.t1/1-3171	0.102692	0.0917014	Ubiquitin- ligase E3B	4	[illegible]
Acrdi.XM_015918048.1/1-513 | Monca.augustus.g34640.t1/1-786	0.102324	0.0917006	Small nuclear ribonucleo -associated B	11	[illegible]
Acrdi.XM_015921990.1/1-747 | Monca.augustus.g8351.t1/1-741	0.12758	0.0916927	mitochondrial import Pam17	3	[illegible]
Acrdi.XM_015898933.1/1-714 | Monca.augustus.g7898.t1/1-714	0.127209	0.0916637	Mitotic checkpoint serine threonine- kinase BUB1 beta	1	[illegible]
Acrdi.XM_015921524.1/1-2898 | Monca.augustus.g22998.t1/1-2904	0.120335	0.0916525	WD repeat-containing 59 isoform X1		
Acrdi.XM_015897172.1/1-357 | Monca.augustus.g18830.t1/1-564	0.141155	0.0916445	NEDD8-conjugating enzyme UBE2F	9	[illegible]
Acrdi.XM_015896213.1/1-495 | Monca.augustus.g40621.t1/1-639	0.154822	0.0916442	P2X purinoceptor 7-like	7	[illegible]
Acrdi.XM_015895786.1/1-1593 | Monca.augustus.g47021.t1/1-1584	0.0990808	0.0916192	glycosyl group 1	3	[illegible]
Acrdi.XM_015902210.1/1-630 | Monca.augustus.g34038.t1/1-1335	0.11826	0.0915928	WD repeat-containing 41-like		
Acrdi.XM_015909772.1/1-963 | Monca.augustus.g46900.t1/1-1203	0.0743865	0.091588	F-box LRR-repeat 2-like		
Acrdi.XM_015904186.1/1-957 | Monca.augustus.g17134.t1/1-1065	0.110028	0.091575	histamine H2 receptor-like	3	[illegible]
Acrdi.XM_015904417.1/1-624 | Monca.augustus.g48072.t1/1-618	0.0823839	0.0915718	ras-related Rab-8A	13	[illegible]
Acrdi.XM_015896498.1/1-1326 | Monca.augustus.g52081.t1/1-1272	0.121417	0.0915609	NAD-dependent deacetylase sirtuin-7	6	[illegible]
Acrdi.XM_015925132.1/1-1443 | Monca.augustus.g37267.t1/1-1578	0.0971063	0.0915426	Ceramide kinase	2	[illegible]
Acrdi.XM_015895820.1/1-591 | Monca.adi2mcaRNA4597_R0/1-588	0.111577	0.0915405	serine protease	3	[illegible]
Acrdi.XM_015920079.1/1-1305 | Monca.augustus.g28574.t1/1-1356	0.0940353	0.0914843	Phosphatidylserine synthase 2	2	[illegible]
Acrdi.XM_015924054.1/1-1257 | Monca.augustus.g19602.t1/1-1959	0.0815859	0.0914837	Leucine-rich repeat and WD repeat-containing 1	2	[illegible]
Acrdi.XM_015894787.1/1-684 | Monca.augustus.g56262.t1/1-579	0.128951	0.0914823	39S ribosomal mitochondrial	3	[illegible]
Acrdi.XM_015901848.1/1-960 | Monca.augustus.g58299.t1/1-1392	0.110591	0.0914795	V-type proton ATPase subunit H isoform X2	3	[illegible]
Acrdi.XM_015910619.1/1-2496 | Monca.augustus.g9087.t1/1-2634	0.249907	0.0914648	reverse transcriptase	2	[illegible]
Acrdi.XM_015904947.1/1-636 | Monca.adi2mcaRNA14556_R0/1-864	0.354821	0.0914373	transcription factor Adf-1-like		
Acrdi.XM_015904105.1/1-708 | Monca.augustus.g22982.t1/1-708	0.101409	0.0914313	Ribosome-recycling mitochondrial	3	[illegible]
Acrdi.XM_015893045.1/1-1410 | Monca.augustus.g16572.t1/1-1449	0.0916558	0.0914292	UDP-glucose 6-dehydrogenase	8	[illegible]
Acrdi.XM_015892112.1/1-1776 | Monca.adi2mcaRNA38017_R0/1-1689	0.146038	0.0913909	coiled-coil domain-containing 78-like isoform X1	6	[illegible]
Acrdi.XM_015909712.1/1-1395 | Monca.augustus.g17532.t1/1-1836	0.102639	0.0913894	PREDICTED: uncharacterized protein LOC107344102	5	[illegible]
Acrdi.XM_015917362.1/1-660 | Monca.adi2mcaRNA28291_R4/1-525	0.418151	0.0913759	vasopressin V1a receptor	3	[illegible]
Acrdi.XM_015897102.1/1-357 | Monca.augustus.g6967.t1/1-369	0.185593	0.0913633	eukaryotic translation initiation factor 4E-binding 2	3	[illegible]
Acrdi.XM_015925466.1/1-1605 | Monca.augustus.g58553.t1/1-1614	0.10722	0.0913193	high-affinity choline transporter 1-like	6	[illegible]
Acrdi.XM_015912771.1/1-324 | Monca.augustus.g26422.t1/1-738	0.109371	0.0912518	alpha-ketoglutarate-dependent dioxygenase alkB homolog mitochondr	1	[illegible]
Acrdi.XM_015907512.1/1-1812 | Monca.augustus.g45769.t1/1-1746	0.136376	0.0912445	smoothelin 1 isoform X10		
Acrdi.XM_015900851.1/1-1191 | Monca.augustus.g11846.t1/1-1197	0.107425	0.091229	probable G- coupled receptor 19	6	[illegible]
Acrdi.XM_015912889.1/1-2505 | Monca.augustus.g60725.t1/1-3414	0.113349	0.0912215	glycine dehydrogenase (decarboxylating) mitochondrial	7	[illegible]
Acrdi.XM_015922872.1/1-1446 | Monca.augustus.g5585.t1/1-1446	0.109666	0.0911829	ammonium transporter 3	5	[illegible]
Acrdi.XM_015910489.1/1-1197 | Monca.augustus.g25432.t1/1-1554	0.0884179	0.0911499	neuroepithelial cell-transforming gene 1 isoform X2	5	[illegible]
Acrdi.XM_015903033.1/1-564 | Monca.augustus.g39033.t1/1-855	0.128597	0.091144	FAM166B-like		
Acrdi.XM_015899297.1/1-1269 | Monca.augustus.g44804.t1/1-1590	0.109089	0.0911154	cleft lip and palate transmembrane 1	2	[illegible]
Acrdi.XM_015918237.1/1-1716 | Monca.augustus.g48556.t1/1-2451	0.123978	0.091098	coiled-coil domain-containing 22 homolog		
Acrdi.XM_015924414.1/1-384 | Monca.augustus.g27956.t1/1-687	0.104324	0.0910897	NAD(P)H-hydrate epimerase	5	[illegible]
Acrdi.XM_015922816.1/1-1650 | Monca.augustus.g365.t1/1-1665	0.0916108	0.0910401	potassium voltage-gated channel subfamily C member 1-like isoform	5	[illegible]
Acrdi.XM_015899569.1/1-885 | Monca.augustus.g46954.t1/1-1509	0.099613	0.091033	F-box WD repeat-containing 2		
Acrdi.XM_015901285.1/1-765 | Monca.augustus.g61801.t1/1-765	0.123639	0.0909994	AMN1 homolog		
Acrdi.XM_015924688.1/1-981 | Monca.augustus.g58996.t1/1-1437	0.364461	0.0909971	OTU domain-containing 6B isoform X1		
Acrdi.XM_015898964.1/1-1704 | Monca.augustus.g37109.t1/1-1803	0.126896	0.0909948	heparan-alpha-glucosaminide N-acetyltransferase-like	2	[illegible]
Acrdi.XM_015895660.1/1-441 | Monca.augustus.g35454.t1/1-1956	0.123105	0.0909908	LON peptidase N-terminal domain and RING finger 1		
Acrdi.XM_015901039.1/1-2466 | Monca.augustus.g12288.t1/1-2529	0.116764	0.0909888	INO80 complex subunit D	1	[illegible]
Acrdi.XM_015909071.1/1-699 | Monca.augustus.g56252.t1/1-1410	0.149753	0.0909802	notchless homolog	1	[illegible]
Acrdi.XM_015909639.1/1-456 | Monca.augustus.g53331.t1/1-540	0.0917976	0.0909795	60S ribosome subunit biogenesis NIP7 homolog	3	[illegible]
Acrdi.XM_015900405.1/1-1533 | Monca.augustus.g46635.t1/1-1536	0.0952486	0.0909654	Potassium voltage-gated channel subfamily A member 1	11	[illegible]
Acrdi.XM_015905610.1/1-1086 | Monca.augustus.g65073.t1/1-1533	0.0783887	0.0909161	Succinyl- ligase [ADP-forming] subunit mitochondrial	5	[illegible]
Acrdi.XM_015914446.1/1-750 | Monca.augustus.g9576.t1/1-747	0.135485	0.0909156	glutamine amidotransferase	2	[illegible]
Acrdi.XM_015898472.1/1-651 | Monca.adi2mcaRNA7577_R9/1-597	0.583705	0.0908991	RNA-directed DNA polymerase from mobile element jockey	3	[illegible]
Acrdi.XM_015907053.1/1-1158 | Monca.augustus.g43298.t1/1-1065	0.120758	0.0908822	[ADP-ribosylarginine]	10	[illegible]
Acrdi.XM_015906283.1/1-1968 | Monca.augustus.g49077.t1/1-2067	0.0729784	0.0908646	Solute carrier organic anion transporter family member 4A1	3	[illegible]
Acrdi.XM_015901856.1/1-474 | Monca.augustus.g15178.t1/1-1074	0.118664	0.0908361	DHHC zinc finger domain	3	[illegible]
Acrdi.XM_015921478.1/1-519 | Monca.adi2mcaRNA32843_R0/1-432	0.108766	0.0908297	E3 ubiquitin- ligase CHIP-like	2	[illegible]
Acrdi.XM_015907688.1/1-1461 | Monca.augustus.g29934.t1/1-1776	0.0962797	0.0908269	Red	4	[illegible]
Acrdi.XM_015924196.1/1-1320 | Monca.augustus.g474.t1/1-1779	0.0862119	0.0908018	ran GTPase-activating 1 isoform X1	2	[illegible]
Acrdi.XM_015920381.1/1-1164 | Monca.augustus.g47492.t1/1-1347	0.0941899	0.0907886	serine threonine- phosphatase 2A regulatory subunit B subunit gamm	11	[illegible]
Acrdi.XM_015906718.1/1-2115 | Monca.augustus.g3900.t1/1-2859	0.0998381	0.0907845	Eukaryotic translation initiation factor 3 subunit C	4	[illegible]
Acrdi.XM_015921601.1/1-1827 | Monca.augustus.g67239.t1/1-2025	0.108184	0.0907805	PREDICTED: uncharacterized protein LOC107355091		
Acrdi.XM_015913210.1/1-834 | Monca.augustus.g2661.t1/1-834	0.388118	0.0907635	dimethylsulfonioproprionate lyase 7-like	1	[illegible]
Acrdi.XM_015913533.1/1-927 | Monca.augustus.g22333.t1/1-1050	0.137275	0.0907408	porphobilinogen deaminase isoform X1	3	[illegible]
Acrdi.XM_015894070.1/1-597 | Monca.augustus.g34665.t1/1-852	0.106311	0.0907349	Inositol monophosphatase 1	6	[illegible]
Acrdi.XM_015907872.1/1-2940 | Monca.augustus.g47385.t1/1-2859	0.104134	0.0907251	neuronal acetylcholine receptor subunit alpha-10-like	5	[illegible]
Acrdi.XM_015896392.1/1-1494 | Monca.augustus.g49969.t1/1-1728	0.127328	0.0907101	Xaa-Pro aminopeptidase 1	1	[illegible]
Acrdi.XM_015897466.1/1-1149 | Monca.augustus.g17607.t1/1-1188	0.11865	0.0906976	PREDICTED: uncharacterized protein KIAA0930-like		
Acrdi.XM_015898634.1/1-324 | Monca.augustus.g7797.t1/1-666	0.129194	0.0906798	Eukaryotic translation initiation factor 4E	3	[illegible]
Acrdi.XM_015916843.1/1-1011 | Monca.adi2mcaRNA27705_R0/1-1002	0.0932332	0.0906773	KH domain- RNA- signal transduction-associated 3	1	[illegible]
Acrdi.XM_015892953.1/1-1548 | Monca.augustus.g22612.t1/1-1518	0.106151	0.0906695	kelch 20	3	[illegible]
Acrdi.XM_015921189.1/1-2049 | Monca.adi2mcaRNA32528_R1/1-2001	0.133979	0.0906689	conserved oligomeric Golgi complex subunit 2 isoform X2	5	[illegible]
Acrdi.XM_015901711.1/1-2355 | Monca.adi2mcaRNA11065_R0/1-2346	0.13004	0.090661	carnitine O-palmitoyltransferase liver isoform	2	[illegible]
Acrdi.XM_015909726.1/1-1998 | Monca.augustus.g3315.t1/1-1995	0.134214	0.0906435	gamma-tubulin complex component 4 isoform X1	12	[illegible]
Acrdi.XM_015893413.1/1-1122 | Monca.adi2mcaRNA39490_R0/1-1125	0.148751	0.0905987	required for meiotic nuclear division 1 homolog	2	[illegible]
Acrdi.XM_015894490.1/1-543 | Monca.augustus.g9944.t1/1-1095	0.148138	0.0905501	quinone oxidoreductase 1	1	[illegible]
Acrdi.XM_015907378.1/1-1383 | Monca.augustus.g35214.t1/1-2016	0.0885494	0.0905471	Carnitine O-palmitoyltransferase mitochondrial	6	[illegible]
Acrdi.XM_015894676.1/1-675 | Monca.augustus.g49919.t1/1-1374	0.118255	0.0905324	DENN domain-containing 1A-		
Acrdi.XM_015903272.1/1-522 | Monca.augustus.g32330.t1/1-714	0.129013	0.0905236	Transmembrane 164	1	[illegible]
Acrdi.XM_015913869.1/1-591 | Monca.augustus.g36678.t1/1-636	0.551576	0.0904965	THAP domain-containing 2		
Acrdi.XM_015899444.1/1-1821 | Monca.augustus.g35522.t1/1-2082	0.0929055	0.0904908	trichohyalin-like isoform X1		
Acrdi.XM_015900618.1/1-807 | Monca.augustus.g33652.t1/1-1359	0.0879475	0.090477	Pre-mRNA-splicing regulator WTAP	8	[illegible]
Acrdi.XM_015914759.1/1-1041 | Monca.adi2mcaRNA25397_R0/1-1008	0.150121	0.0904696	Peptidyl-prolyl cis-trans isomerase D	10	[illegible]
Acrdi.XM_015908860.1/1-2376 | Monca.adi2mcaRNA18850_R0/1-2046	0.0947596	0.0904598	Diacylglycerol kinase theta	3	[illegible]
Acrdi.XM_015896965.1/1-342 | Monca.augustus.g7318.t1/1-339	0.226106	0.090427	Cytochrome c oxidase subunit 7A-related mitochondrial	2	[illegible]
Acrdi.XM_015912924.1/1-4878 | Monca.augustus.g68828.t1/1-5454	0.114827	0.0904254	unconventional myosin-Vb isoform X2	2	[illegible]
Acrdi.XM_015919190.1/1-537 | Monca.adi2mcaRNA30352_R0/1-483	0.10943	0.0904238	high mobility group 20A isoform X1		
Acrdi.XM_015914319.1/1-651 | Monca.augustus.g9585.t1/1-654	0.0639698	0.0904211	Tctex1 domain-containing 1	3	[illegible]
Acrdi.XM_015923377.1/1-618 | Monca.augustus.g14410.t1/1-591	0.675588	0.0904008	PREDICTED: uncharacterized protein LOC107356755		
Acrdi.XM_015899312.1/1-678 | Monca.augustus.g668.t1/1-759	0.109757	0.0903954	Calcium-dependent kinase 5	3	[illegible]
Acrdi.XM_015904365.1/1-966 | Monca.augustus.g59951.t1/1-1926	0.111528	0.0903661	Golgi to ER traffic 4 homolog	5	[illegible]
Acrdi.XM_015924769.1/1-501 | Monca.adi2mcaRNA36613_R0/1-525	0.104915	0.090363	UMP-CMP kinase	8	[illegible]
Acrdi.XM_015913728.1/1-1314 | Monca.augustus.g21653.t1/1-5055	0.0936656	0.0903532	2 -5 -oligoadenylate synthase 1	3	[illegible]
Acrdi.XM_015914460.1/1-651 | Monca.augustus.g71050.t1/1-639	0.115737	0.0903459	Homeobox not2	4	[illegible]
Acrdi.XM_015923075.1/1-1341 | Monca.augustus.g70885.t1/1-1284	0.173027	0.0903356	nucleosome-remodeling factor subunit NURF301 isoform X1	1	[illegible]
Acrdi.XM_015908531.1/1-1332 | Monca.augustus.g46694.t1/1-1269	0.108884	0.0903123	acetyl- mitochondrial	2	[illegible]
Acrdi.XM_015903041.1/1-3879 | Monca.adi2mcaRNA12429_R1/1-3795	0.104813	0.0903197	dedicator of cytokinesis 1 isoform X2		
Acrdi.XM_015897713.1/1-828 | Monca.adi2mcaRNA6638_R0/1-645	0.101951	0.0902974	Autophagy 5	8	[illegible]
Acrdi.XM_015899640.1/1-993 | Monca.augustus.g54490.t1/1-1092	0.122618	0.0902824	Solute carrier family 35 member C2	2	[illegible]
Acrdi.XM_015922511.1/1-792 | Monca.augustus.g33870.t1/1-1365	0.115571	0.0902428	dipeptidyl peptidase 1	23	[illegible]
Acrdi.XM_015906511.1/1-1002 | Monca.augustus.g22563.t1/1-987	0.0712633	0.0902407	5-hydroxytryptamine receptor 1A-alpha-like	3	[illegible]
Acrdi.XM_015893435.1/1-714 | Monca.augustus.g49603.t1/1-1035	0.0979288	0.0902363	WD repeat-containing 31-like	2	[illegible]
Acrdi.XM_015924018.1/1-1326 | Monca.augustus.g55720.t1/1-2529	0.133741	0.0902279	N-acetylated-alpha-linked acidic dipeptidase 2-like		
Acrdi.XM_015903797.1/1-309 | Monca.augustus.g66860.t1/1-1359	0.0615951	0.0901927	ADAMTS 5	7	[illegible]
Acrdi.XM_015918537.1/1-1113 | Monca.augustus.g41458.t1/1-1323	0.116594	0.0901854	vacuolar sorting-associated 4B	35	[illegible]
Acrdi.XM_015912803.1/1-2286 | Monca.augustus.g3594.t1/1-2802	0.110532	0.0901835	sorting nexin-14-like isoform X2	1	[illegible]
Acrdi.XM_015908382.1/1-2118 | Monca.augustus.g34809.t1/1-2307	0.11193	0.0901637	arf-GAP with coiled- ANK repeat and PH domain-containing 2-like	2	[illegible]
Acrdi.XM_015914922.1/1-714 | Monca.augustus.g6247.t1/1-687	0.361853	0.0901582	RNA-directed DNA polymerase from mobile element jockey-like		
Acrdi.XM_015904973.1/1-357 | Monca.adi2mcaRNA14542_R1/1-354	1.04715	0.0901573	PREDICTED: uncharacterized protein LOC107339672		
Acrdi.XM_015907199.1/1-1074 | Monca.augustus.g57672.t1/1-1074	0.0896725	0.0901518	PREDICTED: uncharacterized protein LOC107341734	2	[illegible]
Acrdi.XM_015898396.1/1-1854 | Monca.augustus.g65350.t1/1-837	0.0618895	0.090135	KIF1-binding homolog	3	[illegible]
Acrdi.XM_015896897.1/1-1038 | Monca.augustus.g33191.t1/1-1032	0.084771	0.0901232	forkhead domain-containing	5	[illegible]
Acrdi.XM_015904560.1/1-2889 | Monca.adi2mcaRNA14109_R0/1-2721	0.532518	0.0900924	RNA-directed DNA polymerase from transposon BS		
Acrdi.XM_015913917.1/1-816 | Monca.augustus.g31441.t1/1-702	0.122369	0.0900822	Vesicle transport USE1	2	[illegible]
Acrdi.XM_015908982.1/1-1236 | Monca.augustus.g18152.t1/1-1323	0.0793352	0.0900592	PREDICTED: uncharacterized protein LOC107343412		
Acrdi.XM_015892701.1/1-1101 | Monca.augustus.g27333.t1/1-1446	0.12029	0.0900457	metalloprotease TIKI1	4	[illegible]
Acrdi.XM_015918643.1/1-1017 | Monca.augustus.g33670.t1/1-921	0.111293	0.0900192	kelch domain-containing 8B-like isoform X3	7	[illegible]
Acrdi.XM_015916765.1/1-1695 | Monca.augustus.g3865.t1/1-1668	0.118776	0.0899973	segmentation cap n collar-like	6	[illegible]
Acrdi.XM_015911804.1/1-5205 | Monca.augustus.g39597.t1/1-4965	0.100973	0.0899751	coiled-coil and C2 domain-containing 2A isoform X2	1	[illegible]
Acrdi.XM_015907357.1/1-1206 | Monca.augustus.g63469.t1/1-1218	0.123647	0.0899715	Angio-associated migratory cell		
Acrdi.XM_015916533.1/1-1017 | Monca.augustus.g29781.t1/1-1026	0.116557	0.0899608	octopamine receptor 1-like	3	[illegible]
Acrdi.XM_015925094.1/1-2694 | Monca.augustus.g51121.t1/1-2511	0.128564	0.0899477	breast carcinoma-amplified sequence 3 isoform X1	4	[illegible]
Acrdi.XM_015903511.1/1-867 | Monca.augustus.g1813.t1/1-867	0.0763476	0.0899269	angiopoietin-1 receptor-like isoform X3	34	[illegible]
Acrdi.XM_015919818.1/1-1683 | Monca.augustus.g41938.t1/1-1683	0.103064	0.089908	RING finger 145	1	[illegible]
Acrdi.XM_015901525.1/1-1980 | Monca.augustus.g14965.t1/1-1980	0.11601	0.0898506	Leucine-rich repeat-containing 45		
Acrdi.XM_015902743.1/1-1062 | Monca.augustus.g33293.t1/1-1062	0.100394	0.0898287	serine threonine- kinase Nek6 isoform X2	4	[illegible]
Acrdi.XM_015914004.1/1-2175 | Monca.adi2mcaRNA24514_R0/1-1785	0.162504	0.0898177	phosphatidylinositol-binding clathrin assembly LAP isoform X14		
Acrdi.XM_015905730.1/1-1455 | Monca.augustus.g32751.t1/1-3324	0.100377	0.0897662	sn1-specific diacylglycerol lipase alpha isoform X1	4	[illegible]
Acrdi.XM_015891886.1/1-1227 | Monca.augustus.g6746.t1/1-1218	0.133793	0.0897495	dopamine D2-like receptor	5	[illegible]
Acrdi.XM_015908768.1/1-681 | Monca.adi2mcaRNA18743_R0/1-726	0.0711932	0.0897493	2-oxoisovalerate dehydrogenase subunit mitochondrial	8	[illegible]
Acrdi.XM_015900152.1/1-7446 | Monca.augustus.g62847.t1/1-7797	0.122426	0.0897446	Ubiquitin carboxyl-terminal hydrolase 24	3	[illegible]
Acrdi.XM_015901087.1/1-732 | Monca.augustus.g57658.t1/1-732	0.0944448	0.0896965	cytochrome b561-like	6	[illegible]
Acrdi.XM_015902608.1/1-426 | Monca.augustus.g22802.t1/1-429	0.11119	0.089685	2 -deoxynucleoside 5 -phosphate N-hydrolase 1	4	[illegible]
Acrdi.XM_015896282.1/1-804 | Monca.augustus.g33696.t1/1-804	0.124429	0.089676	NADH dehydrogenase [ubiquinone] iron-sulfur mitochondrial	3	[illegible]
Acrdi.XM_015922799.1/1-3282 | Monca.augustus.g362.t1/1-3285	0.117004	0.0896759	Zinc finger SWIM domain-containing 5	1	[illegible]
Acrdi.XM_015907510.1/1-684 | Monca.augustus.g45771.t1/1-684	0.107275	0.0896594	haloacid dehalogenase	2	[illegible]
Acrdi.XM_015895757.1/1-699 | Monca.augustus.g2121.t1/1-699	0.135497	0.0896504	Exosome complex component RRP46	9	[illegible]
Acrdi.XM_015910493.1/1-3039 | Monca.adi2mcaRNA20709_R0/1-2973	0.0882314	0.0896423	kinesin KIF6	7	[illegible]
Acrdi.XM_015896641.1/1-762 | Monca.augustus.g11238.t1/1-891	0.106386	0.0896199	cyclin-dependent-like kinase 5	3	[illegible]
Acrdi.XM_015895756.1/1-1134 | Monca.adi2mcaRNA4511_R0/1-1131	0.126981	0.0895885	Adipocyte plasma membrane-associated	3	[illegible]
Acrdi.XM_015895282.1/1-615 | Monca.augustus.g4303.t1/1-678	0.318468	0.0895846	paired box Pax-5-like		
Acrdi.XM_015897587.1/1-747 | Monca.adi2mcaRNA588_R3/1-753	0.213338	0.0895275	myb X		
Acrdi.XM_015897396.1/1-651 | Monca.augustus.g51657.t1/1-660	0.050933	0.0895155	growth factor receptor-bound 2	32	[illegible]
Acrdi.XM_015925475.1/1-1863 | Monca.augustus.g34418.t1/1-1959	0.081848	0.0895102	transient receptor potential cation channel subfamily V member 5-like	3	[illegible]
Acrdi.XM_015921985.1/1-1974 | Monca.augustus.g8353.t1/1-2184	0.0896863	0.0894667	myotubularin-related 6-like	2	[illegible]
Acrdi.XM_015905794.1/1-2115 | Monca.augustus.g31746.t1/1-2520	0.115071	0.0894579	Tetratricopeptide repeat 13		
Acrdi.XM_015922867.1/1-1710 | Monca.augustus.g5584.t1/1-1710	0.0922736	0.0894515	Tripeptidyl-peptidase 1	4	[illegible]
Acrdi.XM_015903430.1/1-885 | Monca.adi2mcaRNA12830_R0/1-861	0.112062	0.0894491	hydroxyacyl-coenzyme A mitochondrial	5	[illegible]
Acrdi.XM_015923824.1/1-615 | Monca.augustus.g8186.t1/1-912	0.145928	0.0894448	very-long-chain enoyl- reductase-like	2	[illegible]
Acrdi.XM_015923832.1/1-1083 | Monca.augustus.g33430.t1/1-1122	0.112203	0.0894208	RNA 3 -terminal phosphate cyclase	5	[illegible]
Acrdi.XM_015922327.1/1-1701 | Monca.augustus.g1263.t1/1-1668	0.0970018	0.0894204	Glucose-6-phosphate isomerase	12	[illegible]
Acrdi.XM_015896118.1/1-942 | Monca.augustus.g55594.t1/1-945	0.122571	0.089387	hydroxymethylglutaryl- mitochondrial	10	[illegible]
Acrdi.XM_015913889.1/1-1197 | Monca.augustus.g66926.t1/1-1194	0.11602	0.0893673	doublesex- and mab-3-related transcription factor A2-like isoform X1	2	[illegible]
Acrdi.XM_015918523.1/1-3375 | Monca.augustus.g36303.t1/1-3924	0.107933	0.0893326	multidrug resistance-associated 4-like	8	[illegible]
Acrdi.XM_015904886.1/1-636 | Monca.augustus.g66114.t1/1-684	0.0870572	0.0893178	tetraspanin-13 isoform X2	2	[illegible]
Acrdi.XM_015896412.1/1-459 | Monca.adi2mcaRNA5242_R0/1-540	0.104082	0.0893023	ELYS	2	[illegible]
Acrdi.XM_015909017.1/1-465 | Monca.augustus.g38368.t1/1-465	0.11764	0.0892786	PREDICTED: uncharacterized protein LOC107343450		
Acrdi.XM_015908675.1/1-2610 | Monca.augustus.g16052.t1/1-2697	0.0777748	0.0892661	EF-hand calcium-binding domain-containing 6-like	1	[illegible]
Acrdi.XM_015914898.1/1-930 | Monca.adi2mcaRNA25538_R0/1-918	0.613304	0.0892434	PREDICTED: uncharacterized protein LOC107348825		
Acrdi.XM_015907311.1/1-1482 | Monca.augustus.g31806.t1/1-1845	0.107976	0.0892208	rap1 GTPase-GDP dissociation stimulator 1 isoform X1	3	[illegible]
Acrdi.XM_015895996.1/1-1521 | Monca.augustus.g2191.t1/1-1518	0.10651	0.0891693	pyruvate dehydrogenase [acetyl-transferring]-phosphatase mitochondr	1	[illegible]
Acrdi.XM_015909036.1/1-552 | Monca.adi2mcaRNA19026_R0/1-501	0.11183	0.0891534	phosphodiesterase alkaline phosphatase D	2	[illegible]
Acrdi.XM_015914201.1/1-687 | Monca.augustus.g58338.t1/1-693	0.0810041	0.0891448	UPF0598 C8orf82 homolog	1	[illegible]
Acrdi.XM_015907221.1/1-315 | Monca.augustus.g11180.t1/1-1323	0.0842091	0.0891413	PREDICTED: uncharacterized protein LOC107341760		
Acrdi.XM_015924815.1/1-1671 | Monca.augustus.g67346.t1/1-1722	0.0914509	0.0891062	coronin-2B-like isoform X1	3	[illegible]
Acrdi.XM_015892383.1/1-4365 | Monca.adi2mcaRNA38317_R0/1-4284	0.0969853	0.0891007	voltage-gated sodium channel	7	[illegible]
Acrdi.XM_015909392.1/1-3024 | Monca.augustus.g19730.t1/1-7167	0.103392	0.0890887	neurobeachin 1 isoform X2	3	[illegible]
Acrdi.XM_015895051.1/1-1179 | Monca.adi2mcaRNA41423_R2/1-1017	0.297915	0.0890759	SURP and G-patch domain-containing 1	2	[illegible]
Acrdi.XM_015914451.1/1-468 | Monca.augustus.g56327.t1/1-660	0.0743342	0.0890759	polycomb group RING finger 1 isoform X1	1	[illegible]
Acrdi.XM_015904672.1/1-804 | Monca.augustus.g22471.t1/1-804	0.097202	0.0890749	branched-chain-amino-acid aminotransferase 2	2	[illegible]
Acrdi.XM_015902824.1/1-1113 | Monca.augustus.g2188.t1/1-1041	0.113618	0.0890659	PREDICTED: uncharacterized protein LOC107337618	2	[illegible]
Acrdi.XM_015917109.1/1-2241 | Monca.adi2mcaRNA28015_R0/1-2058	0.4158	0.0890642	FAM171A1 isoform X1		
Acrdi.XM_015916037.1/1-894 | Monca.augustus.g16417.t1/1-894	0.139268	0.0890576	Nucleoside diphosphate-linked moiety X motif 6	1	[illegible]
Acrdi.XM_015911940.1/1-411 | Monca.augustus.g58636.t1/1-498	0.102214	0.0890084	50S ribosomal L24	2	[illegible]
Acrdi.XM_015897229.1/1-399 | Monca.adi2mcaRNA6092_R0/1-441	0.115259	0.0890075	U6 snRNA-associated Sm LSm4	11	[illegible]
Acrdi.XM_015901203.1/1-1149 | Monca.augustus.g58577.t1/1-1293	0.151172	0.0889884	Apoptosis-inducing factor 2	1	[illegible]
Acrdi.XM_015922156.1/1-1905 | Monca.augustus.g19697.t1/1-1905	0.110864	0.0889277	mannosyl-oligosaccharide 1,2-alpha-mannosidase IB	6	[illegible]
Acrdi.XM_015916757.1/1-3216 | Monca.augustus.g62772.t1/1-4791	0.109253	0.0889135	transient receptor potential cation channel subfamily M member 2-lik	3	[illegible]
Acrdi.XM_015907807.1/1-663 | Monca.augustus.g55797.t1/1-627	0.100846	0.0889002	Ras-related Rab-43	2	[illegible]
Acrdi.XM_015903404.1/1-1656 | Monca.augustus.g35987.t1/1-1671	0.11262	0.0888951	Menin	11	[illegible]
Acrdi.XM_015913115.1/1-1584 | Monca.adi2mcaRNA23535_R0/1-1569	0.10566	0.0888904	arylsulfatase J-like	1	[illegible]
Acrdi.XM_015911116.1/1-303 | Monca.augustus.g58859.t1/1-471	0.124249	0.0888831	PREDICTED: uncharacterized protein LOC107345414		
Acrdi.XM_015899939.1/1-843 | Monca.augustus.g32794.t1/1-843	0.131512	0.0888341	G1 S-specific cyclin-D2	7	[illegible]
Acrdi.XM_015915706.1/1-1476 | Monca.augustus.g37389.t1/1-1914	0.0887082	0.0888166	sodium-coupled monocarboxylate transporter 1-like	7	[illegible]
Acrdi.XM_015920264.1/1-1440 | Monca.adi2mcaRNA31526_R0/1-1128	0.367587	0.0887559	cell wall integrity and stress response component 4-like		
Acrdi.XM_015899453.1/1-897 | Monca.augustus.g48277.t1/1-897	0.144866	0.0887491	trichohyalin-like isoform X1	5	[illegible]
Acrdi.XM_015910838.1/1-1434 | Monca.augustus.g57299.t1/1-1401	0.109783	0.0887447	potassium voltage-gated channel subfamily A member 3	5	[illegible]
Acrdi.XM_015923360.1/1-543 | Monca.augustus.g9302.t1/1-543	0.105702	0.0887006	RWD domain-containing 4 isoform X1		
Acrdi.XM_015902742.1/1-2667 | Monca.augustus.g33295.t1/1-2613	0.0886675	0.0886869	serine threonine- kinase D3	4	[illegible]
Acrdi.XM_015892877.1/1-3114 | Monca.adi2mcaRNA38904_R0/1-3132	0.0823528	0.0886825	digestive cysteine ase 1-like	5	[illegible]

Acrdi.XM_015896265.1/1-402 | Monca.augustus.g33722.t1/1-951	0.18605	0.0886103	39S ribosomal mitochondrial			
Acrdi.XM_015905318.1/1-2040 | Monca.adi2mcaRNA14954_R0/1-1854	0.123068	0.0885952	cilia- and flagella-associated 52			
Acrdi.XM_015901584.1/1-915 | Monca.augustus.g55973.t1/1-918	0.0966439	0.0885703	Zinc finger 367	3	F:GO:0003676; C:GO:0005634; F:GO:0046872	[illegible]
Acrdi.XM_015906442.1/1-435 | Monca.augustus.g12470.t1/1-435	0.0883731	0.0885608	Mitochondrial fission 1	2	C:GO:0016021; P:GO:0000266	[illegible]
Acrdi.XM_015922137.1/1-1734 | Monca.adi2mcaRNA3377_R0/1-1224	0.0974242	0.08856	delta-1-pyrroline-5-carboxylate synthase isoform X1	6	F:GO:0004349; C:GO:0005739; F:GO:0004350; P:GO:0006561;	[illegible]
Acrdi.XM_015906981.1/1-1563 | Monca.augustus.g23302.t1/1-1557	0.10551	0.0885561	serine threonine- kinase Chk2-like	3	F:GO:0000166; F:GO:0004672; P:GO:0016310	[illegible]
Acrdi.XM_015910813.1/1-6132 | Monca.adi2mcaRNA2169_R0/1-6012	0.101879	0.0885316	neurogenic locus notch homolog 1-like	1	F:GO:0005509	[illegible]
Acrdi.XM_015900774.1/1-1932 | Monca.adi2mcaRNA10025_R0/1-1836	0.0947261	0.0885297	A disintegrin and metallo ase with thrombospondin motifs 16	1	F:GO:0016787	[illegible]
Acrdi.XM_015905614.1/1-1272 | Monca.augustus.g65080.t1/1-3729	0.0847775	0.0885286	unc-80 homolog			
Acrdi.XM_015913368.1/1-2160 | Monca.augustus.g32157.t1/1-2577	0.0998912	0.0885207	cap-specific mRNA (nucleoside-2 -O-)-methyltransferase 1	3	P:GO:0016071; F:GO:0016740; P:GO:0032259	[illegible]
Acrdi.XM_015901222.1/1-1749 | Monca.adi2mcaRNA10479_R0/1-1767	0.416471	0.0885075	Retrovirus-related Pol poly from transposon			
Acrdi.XM_015903916.1/1-1242 | Monca.augustus.g47360.t1/1-1311	0.204382	0.0884631	nuclease HARBI1			
Acrdi.XM_015904555.1/1-822 | Monca.adi2mcaRNA14105_R7/1-606	0.573948	0.0884585	PREDICTED: uncharacterized protein LOC107339291			
Acrdi.XM_015924877.1/1-633 | Monca.augustus.g24362.t1/1-621	0.176719	0.0884552	Bcl-2 1	6	F:GO:0042803; F:GO:0046982; P:GO:0008630; P:GO:0042981;	[illegible]
Acrdi.XM_015907755.1/1-3303 | Monca.augustus.g68021.t1/1-5637	0.119816	0.0884372	virilizer homolog	3	C:GO:0016607; C:GO:0036396; P:GO:0080009	[illegible]
Acrdi.XM_015905847.1/1-2571 | Monca.adi2mcaRNA15557_R0/1-2547	0.429183	0.0883949	pelota homolog			
Acrdi.XM_015898535.1/1-777 | Monca.augustus.g34000.t1/1-780	0.145557	0.0883376	E3 ubiquitin- ligase RNF115	7	C:GO:0005634; F:GO:0061630; F:GO:0008270; P:GO:0043161;	[illegible]
Acrdi.XM_015918099.1/1-858 | Monca.augustus.g43955.t1/1-846	0.127848	0.0883259	forkhead box D3-like	3	F:GO:0003700; P:GO:0006355; F:GO:0043565	[illegible]
Acrdi.XM_015907391.1/1-1062 | Monca.augustus.g64322.t1/1-1029	0.0900194	0.0883202	dopamine receptor 2-like	5	F:GO:0001588; P:GO:0001963; P:GO:0007165; F:GO:0035240;	[illegible]
Acrdi.XM_015913799.1/1-1494 | Monca.augustus.g26425.t1/1-1611	0.11635	0.0883135	Ammonium transporter Rh type C	5	F:GO:0008519; C:GO:0005887; P:GO:0015695; P:GO:0072488;	[illegible]
Acrdi.XM_015912066.1/1-423 | Monca.adi2mcaRNA22377_R0/1-384	0.148731	0.08831	PREDICTED: uncharacterized protein LOC107346288			
Acrdi.XM_015898486.1/1-378 | Monca.augustus.g45600.t1/1-432	0.103688	0.0883081	MIP18 family FAM96A isoform X1	6	F:GO:0005515; C:GO:0005737; F:GO:0046872; P:GO:0007059;	[illegible]
Acrdi.XM_015903712.1/1-993 | Monca.adi2mcaRNA13157_R0/1-900	0.136481	0.0882953	gamma-enolase isoform X2	4	F:GO:0004634; F:GO:0000287; P:GO:0006096; C:GO:0000015	[illegible]
Acrdi.XM_015901339.1/1-1116 | Monca.augustus.g48460.t1/1-1149	0.082159	0.0882912	hsp90 co-chaperone Cdc37	6	F:GO:0031072; C:GO:0005737; F:GO:0051087; F:GO:0051082;	[illegible]
Acrdi.XM_015916760.1/1-1398 | Monca.augustus.g62773.t1/1-1395	0.0961002	0.0882601	synaptotagmin 1 isoform X1	10	C:GO:0070382; C:GO:0098793; P:GO:0048791; F:GO:0019905;	[illegible]
Acrdi.XM_015909846.1/1-720 | Monca.augustus.g47089.t1/1-696	0.135724	0.0882451	transcription factor SOX-14	5	C:GO:0005634; F:GO:0003677; P:GO:0003006; P:GO:0009987;	[illegible]
Acrdi.XM_015912247.1/1-1002 | Monca.augustus.g28332.t1/1-3141	0.093779	0.088236	Arginine-glutamic acid dipeptide repeats			
Acrdi.XM_015922651.1/1-459 | Monca.augustus.g38228.t1/1-534	0.0982693	0.0882233	Kelch 3	1	C:GO:0016020	[illegible]
Acrdi.XM_015900583.1/1-3651 | Monca.adi2mcaRNA9798_R0/1-3474	0.112628	0.0882042	still isoform SIF type 1-like isoform X1	8	P:GO:0035023; P:GO:0090630; P:GO:0007165; F:GO:0005085;	[illegible]
Acrdi.XM_015905543.1/1-3354 | Monca.augustus.g24087.t1/1-3609	0.0890301	0.0881798	diaphanous homolog 2 isoform X1	4	F:GO:0003779; P:GO:0007015; F:GO:0017048; P:GO:0007049	[illegible]
Acrdi.XM_015910285.1/1-726 | Monca.augustus.g65957.t1/1-1542	0.0944782	0.0881397	Peptidyl-prolyl cis-trans isomerase	2	C:GO:0044425; P:GO:0006457	[illegible]
Acrdi.XM_015907166.1/1-993 | Monca.augustus.g28160.t1/1-1995	0.119556	0.0881188	peroxisomal acyl-coenzyme A oxidase 1 isoform X2	8	F:GO:0003995; C:GO:0005777; P:GO:0033539; F:GO:0000062;	[illegible]
Acrdi.XM_015901358.1/1-4227 | Monca.adi2mcaRNA1110_R0/1-3849	0.081751	0.0881182	Contactin-associated -like 2	1	C:GO:0016020	[illegible]
Acrdi.XM_015913190.1/1-720 | Monca.augustus.g39289.t1/1-720	0.0963378	0.0881084	arachidonate 5-lipoxygenase-like	2	F:GO:0016491; F:GO:0005488	[illegible]
Acrdi.XM_015911962.1/1-1149 | Monca.augustus.g58633.t1/1-1149	0.0949892	0.0881078	Peroxisomal biogenesis factor 3	3	C:GO:0016021; F:GO:0015171; P:GO:0003333	[illegible]
Acrdi.XM_015910277.1/1-849 | Monca.augustus.g64208.t1/1-801	0.153061	0.0880935	HAUS augmin-like complex subunit 1	2	P:GO:0051225; C:GO:0070652	[illegible]
Acrdi.XM_015892251.1/1-954 | Monca.augustus.g44168.t1/1-954	0.0986418	0.0880714	dehydrogenase reductase SDR family member 12-like	1	C:GO:0005575	[illegible]
Acrdi.XM_015896192.1/1-828 | Monca.adi2mcaRNA5128_R0/1-789	0.107227	0.0880663	Ubiquinone biosynthesis mitochondrial	3	F:GO:0008289; P:GO:0006744; C:GO:0005743	[illegible]
Acrdi.XM_015921548.1/1-699 | Monca.augustus.g40273.t1/1-1029	0.458126	0.0880486	E3 ubiquitin- ligase TRIM71-like			
Acrdi.XM_015909587.1/1-2379 | Monca.augustus.g33545.t1/1-1698	0.113089	0.0880465	Mitochondrial import receptor subunit	2	C:GO:0016020; C:GO:0016021	[illegible]
Acrdi.XM_015904805.1/1-6234 | Monca.augustus.g49265.t1/1-6480	0.098136	0.0880398	Unconventional myosin-XVIIIa			
Acrdi.XM_015903074.1/1-483 | Monca.augustus.g48860.t1/1-483	0.0888511	0.0880391	H ACA ribonucleo complex subunit 2	9	C:GO:0031429; P:GO:0031118; P:GO:0000470; P:GO:0031120;	[illegible]
Acrdi.XM_015910537.1/1-582 | Monca.adi2mcaRNA20725_R0/1-522	0.106709	0.0880271	ubiquitin-conjugating enzyme E2 T	16	P:GO:0006281; F:GO:0061630; P:GO:0070979; F:GO:0061631;	[illegible]
Acrdi.XM_015891928.1/1-1941 | Monca.augustus.g25632.t1/1-2019	0.110704	0.0880193	NADPH--cytochrome P450 reductase isoform X1	6	C:GO:0005789; F:GO:0010181; F:GO:0003958; C:GO:0016021;	[illegible]
Acrdi.XM_015901953.1/1-1470 | Monca.augustus.g45543.t1/1-1479	0.119221	0.0880163	unc-93 homolog A	1	C:GO:0016021	[illegible]
Acrdi.XM_015905159.1/1-948 | Monca.augustus.g57156.t1/1-1056	0.0902687	0.0880143	nematoblast-specific nb012a			
Acrdi.XM_015907848.1/1-1125 | Monca.augustus.g70309.t1/1-1242	0.0745287	0.0880059	28S ribosomal mitochondrial	2	F:GO:0003735; C:GO:0005763	[illegible]
Acrdi.XM_015914448.1/1-1644 | Monca.adi2mcaRNA25020_R0/1-1491	0.172616	0.0879727	RMD5 homolog A isoform X2	1	F:GO:0046872	[illegible]
Acrdi.XM_015909567.1/1-3843 | Monca.adi2mcaRNA19670_R0/1-4050	0.0880634	0.0879622	Contactin-associated -like 2	7	F:GO:0008270; F:GO:0008061; P:GO:0006508; C:GO:0016020;	[illegible]
Acrdi.XM_015919341.1/1-1278 | Monca.augustus.g71334.t1/1-1275	0.106937	0.0879062	E3 ubiquitin- ligase MYLIP	7	P:GO:0065007; F:GO:0004842; F:GO:0046872; C:GO:0016020;	[illegible]
Acrdi.XM_015901733.1/1-1629 | Monca.augustus.g34406.t1/1-1632	0.110928	0.0879061	wntless homolog	16	P:GO:0015031; P:GO:0090263; C:GO:0044431; C:GO:0005783;	[illegible]
Acrdi.XM_015903638.1/1-5616 | Monca.augustus.g49463.t1/1-6606	0.123646	0.0878968	activating signal cointegrator 1 complex subunit 3 isoform X1	8	F:GO:0003676; P:GO:0006307; P:GO:0008283; F:GO:0005524;	[illegible]
Acrdi.XM_015900735.1/1-1422 | Monca.augustus.g34886.t1/1-1425	0.125246	0.0878908	lysophospholipid acyltransferase 5	6	F:GO:0042171; F:GO:0047184; P:GO:0046470; P:GO:0097006;	[illegible]
Acrdi.XM_015895162.1/1-405 | Monca.adi2mcaRNA41564_R0/1-399	0.16876	0.0878878	Dna-			
Acrdi.XM_015911224.1/1-1188 | Monca.augustus.g67127.t1/1-1257	0.0830775	0.0878761	Fumarylacetoacetase	2	P:GO:0009072; F:GO:0004334	[illegible]
Acrdi.XM_015919094.1/1-1002 | Monca.augustus.g67790.t1/1-999	0.128979	0.087805	Serine racemase	11	F:GO:0000287; F:GO:0003941; F:GO:0005524; F:GO:0030170;	[illegible]
Acrdi.XM_015911079.1/1-1227 | Monca.augustus.g55129.t1/1-4512	0.0768157	0.0877965	Serine threonine- kinase Nek8	4	F:GO:0000166; C:GO:0016020; P:GO:0006468; P:GO:0009887	[illegible]
Acrdi.XM_015922495.1/1-489 | Monca.augustus.g60691.t1/1-2106	0.147808	0.0877819	Phosphoserine phosphatase	4	F:GO:0000287; F:GO:0004647; C:GO:0005737; P:GO:0006564	[illegible]
Acrdi.XM_015919835.1/1-621 | Monca.augustus.g12037.t1/1-564	0.134989	0.0877773	FAM57B isoform X1	7	C:GO:0016020; C:GO:0016021; F:GO:0050291; P:GO:0045599;	[illegible]
Acrdi.XM_015907944.1/1-1521 | Monca.augustus.g62662.t1/1-2331	0.0578717	0.0877739	extended synaptotagmin-3-like	2	C:GO:0016020; C:GO:0005783	[illegible]
Acrdi.XM_015899110.1/1-1056 | Monca.augustus.g52434.t1/1-1056	0.102558	0.0877525	Wnt-4 isoform X1	52	P:GO:0008584; P:GO:0008585; P:GO:0038030; P:GO:0048754;	[illegible]
Acrdi.XM_015901331.1/1-726 | Monca.augustus.g48492.t1/1-726	0.120335	0.0877357	PREDICTED: uncharacterized protein LOC107336263			
Acrdi.XM_015901133.1/1-1956 | Monca.adi2mcaRNA10344_R0/1-2013	0.085749	0.0877289	AMP deaminase 2 isoform X1	2	F:GO:0003876; P:GO:0032264	[illegible]
Acrdi.XM_015914130.1/1-1092 | Monca.augustus.g24979.t1/1-1086	0.0893579	0.0876981	U1 small nuclear ribonucleo 70 kDa-like	11	C:GO:0000243; C:GO:0019012; C:GO:0071011; F:GO:0030619;	[illegible]
Acrdi.XM_015910269.1/1-1071 | Monca.augustus.g7388.t1/1-1083	0.116141	0.0876872	FAM81A isoform X2			
Acrdi.XM_015921760.1/1-2196 | Monca.adi2mcaRNA33135_R0/1-2178	0.076792	0.087623	EGF-like repeat and discoidin I-like domain-containing 3	1	F:GO:0070492	[illegible]
Acrdi.XM_015909313.1/1-1113 | Monca.augustus.g27144.t1/1-1452	0.131902	0.0876114	rab5 GDP GTP exchange factor-like	2	F:GO:0003677; F:GO:0008270	[illegible]
Acrdi.XM_015895640.1/1-2301 | Monca.adi2mcaRNA4385_R0/1-2196	0.111147	0.08757	ubiquitin- ligase E3A isoform X1	10	C:GO:0005634; F:GO:0004842; P:GO:0060255; P:GO:0048522;	[illegible]
Acrdi.XM_015924762.1/1-981 | Monca.augustus.g43731.t1/1-1227	0.134624	0.0875102	calcium calmodulin-dependent kinase type II delta 1 chain isoform X9	3	F:GO:0005524; F:GO:0004674; P:GO:0006468	[illegible]
Acrdi.XM_015894786.1/1-2982 | Monca.augustus.g41892.t1/1-2940	0.111778	0.0875063	EGF-like repeat and discoidin I-like domain-containing 3	1	F:GO:0070492	[illegible]
Acrdi.XM_015906141.1/1-432 | Monca.adi2mcaRNA1587_R0/1-432	0.0836386	0.0874471	PREDICTED: uncharacterized protein C7orf72 homolog	3	P:GO:0008150; C:GO:0005575; F:GO:0003674	[illegible]
Acrdi.XM_015900022.1/1-2001 | Monca.augustus.g70558.t1/1-2427	0.0954557	0.0874454	Calcium homeostasis endoplasmic reticulum	5	F:GO:0003676; F:GO:0003723; C:GO:0016020; C:GO:0016021;	[illegible]
Acrdi.XM_015894352.1/1-2685 | Monca.augustus.g17624.t1/1-2520	0.100135	0.0874424	serine threonine- kinase endoribonuclease IRE1-like	2	P:GO:0044260; P:GO:0044238	[illegible]
Acrdi.XM_015922998.1/1-1443 | Monca.augustus.g67835.t1/1-1452	0.0823592	0.087436	vesicular inhibitory amino acid transporter-like	3	C:GO:0016021; F:GO:0015171; P:GO:0003333	[illegible]
Acrdi.XM_015899645.1/1-486 | Monca.augustus.g54489.t1/1-873	0.0841084	0.0874183	purine nucleoside phosphorylase-like isoform X2	4	C:GO:0005737; P:GO:0009116; F:GO:0043101; F:GO:0004731	[illegible]
Acrdi.XM_015914800.1/1-915 | Monca.adi2mcaRNA25415_R0/1-918	0.110061	0.087412	Non-histone 10			
Acrdi.XM_015918083.1/1-873 | Monca.augustus.g34654.t1/1-870	0.135613	0.0874078	exosome complex component RRP42	13	P:GO:0034475; P:GO:0034473; P:GO:0043928; P:GO:0000467;	[illegible]
Acrdi.XM_015922990.1/1-801 | Monca.augustus.g67855.t1/1-798	0.0899167	0.0874034	Enkurin			
Acrdi.XM_015899261.1/1-1113 | Monca.augustus.g21753.t1/1-3306	0.126721	0.0873946	FAM135A isoform X2	2	P:GO:0044255; F:GO:0052689	[illegible]
Acrdi.XM_015906722.1/1-2409 | Monca.adi2mcaRNA16543_R1/1-2361	0.268598	0.0873652	Retrovirus-related Pol poly from transposon 412			
Acrdi.XM_015918329.1/1-675 | Monca.augustus.g29413.t1/1-672	0.0984213	0.0873522	PREDICTED: uncharacterized protein LOC107352025			
Acrdi.XM_015902253.1/1-1536 | Monca.augustus.g35076.t1/1-1674	0.108448	0.0873391	probable lysosomal cobalamin transporter	2	C:GO:0016020; C:GO:0005764	[illegible]
Acrdi.XM_015916232.1/1-1098 | Monca.augustus.g18677.t1/1-1095	0.10897	0.0873293	octopamine receptor-like	5	F:GO:0001588; P:GO:0001963; P:GO:0007165; F:GO:0035240;	[illegible]
Acrdi.XM_015892296.1/1-1821 | Monca.adi2mcaRNA38209_R1/1-1701	0.0987385	0.0873169	pre-mRNA-splicing factor CWC22 homolog	4	F:GO:0003723; C:GO:0071013; C:GO:0071006; P:GO:0000398	[illegible]
Acrdi.XM_015898724.1/1-681 | Monca.augustus.g2540.t1/1-696	0.0866792	0.0872667	sperm-associated antigen 7	1	F:GO:0003676	[illegible]
Acrdi.XM_015904190.1/1-765 | Monca.augustus.g11839.t1/1-1569	0.12647	0.0872586	dual specificity phosphatase CDC14A isoform X3	12	F:GO:0004722; C:GO:0005813; P:GO:0051256; C:GO:0072686;	[illegible]
Acrdi.XM_015907030.1/1-2292 | Monca.augustus.g43269.t1/1-2490	0.101007	0.0872574	ubiquitin carboxyl-terminal hydrolase 31-like	2	P:GO:0006508; P:GO:0044267	[illegible]
Acrdi.XM_015905864.1/1-780 | Monca.augustus.g32198.t1/1-819	0.10439	0.0872376	SOSS complex subunit B2			
Acrdi.XM_015897157.1/1-1395 | Monca.augustus.g59970.t1/1-561	0.134775	0.0872301	HAUS augmin-like complex subunit 3	2	C:GO:0015630; C:GO:0044446	[illegible]
Acrdi.XM_015903673.1/1-1110 | Monca.augustus.g20095.t1/1-1110	0.134559	0.0872027	dehydrodolichyl diphosphate syntase complex subunit DHDDS-like	6	C:GO:0005789; P:GO:0050908; F:GO:0005515; F:GO:0002094;	[illegible]
Acrdi.XM_015912978.1/1-828 | Monca.augustus.g56214.t1/1-795	0.143761	0.0871503	NK1 transcription factor-related 1	4	F:GO:0003677; C:GO:0005634; P:GO:0006355; F:GO:0043565	[illegible]
Acrdi.XM_015919120.1/1-1263 | Monca.augustus.g35183.t1/1-1344	0.117519	0.0871476	PREDICTED: uncharacterized protein LOC107352807	2	C:GO:0016020; C:GO:0016021	[illegible]
Acrdi.XM_015892819.1/1-552 | Monca.augustus.g5332.t1/1-576	0.549926	0.0871307	gibberellin-regulated 14-like			
Acrdi.XM_015895464.1/1-693 | Monca.augustus.g61598.t1/1-981	0.0717795	0.0871293	small glutamine-rich tetratricopeptide repeat-containing alpha-like			
Acrdi.XM_015916119.1/1-1422 | Monca.augustus.g8603.t1/1-1425	0.0887652	0.0871149	DGCR14	5	F:GO:0003677; C:GO:0005634; F:GO:0003700; P:GO:0006351;	[illegible]
Acrdi.XM_015904088.1/1-936 | Monca.augustus.g32311.t1/1-936	0.11398	0.087111	phosphoglycolate phosphatase	2	P:GO:0008152; F:GO:0016791	[illegible]
Acrdi.XM_015919146.1/1-828 | Monca.augustus.g35149.t1/1-849	0.111693	0.0870777	S-adenosylmethionine mitochondrial carrier	3	F:GO:0000095; P:GO:0015805; C:GO:0005743	[illegible]
Acrdi.XM_015898504.1/1-402 | Monca.augustus.g3318.t1/1-396	0.121604	0.0870525	KRTCAP2 homolog	1	C:GO:0016021	[illegible]
Acrdi.XM_015922041.1/1-1263 | Monca.augustus.g31851.t1/1-2814	0.0900616	0.087031	TBC1 domain family member 5	9	P:GO:0031338; P:GO:0090630; P:GO:0006914; F:GO:0005096;	[illegible]
Acrdi.XM_015917908.1/1-1209 | Monca.augustus.g39689.t1/1-1209	0.0983933	0.0870171	Cystathionine gamma-synthase	1	F:GO:0016846	[illegible]
Acrdi.XM_015907117.1/1-1008 | Monca.augustus.g36978.t1/1-1020	0.125314	0.0870151	ELAV 2	2	F:GO:0097159; F:GO:1901363	[illegible]
Acrdi.XM_015919983.1/1-2499 | Monca.augustus.g16012.t1/1-2481	0.0886055	0.0869694	PREDICTED: uncharacterized protein LOC107353645	1	C:GO:0016020	[illegible]
Acrdi.XM_015907457.1/1-1947 | Monca.adi2mcaRNA17343_R0/1-1950	0.125163	0.086966	sodium hydrogen exchanger 6	5	F:GO:0015385; C:GO:0016021; P:GO:1902600; P:GO:0035725;	[illegible]
Acrdi.XM_015905328.1/1-1749 | Monca.augustus.g31849.t1/1-1197	0.444671	0.0869365	transmembrane 246-like	2	C:GO:0016020; C:GO:0016021	[illegible]
Acrdi.XM_015910320.1/1-1110 | Monca.augustus.g36428.t1/1-1500	0.120479	0.0869172	calcium-binding mitochondrial carrier S -2-like	8	P:GO:0055085; F:GO:0003735; F:GO:0005509; C:GO:0016020;	[illegible]
Acrdi.XM_015903077.1/1-909 | Monca.augustus.g48861.t1/1-909	0.0936373	0.0869028	PREDICTED: uncharacterized protein LOC107337842	2	C:GO:0016020; C:GO:0016021	[illegible]
Acrdi.XM_015907032.1/1-504 | Monca.augustus.g43262.t1/1-546	0.0878691	0.0869028	Armadillo repeat-containing 7	2	C:GO:0005634; C:GO:0005737	[illegible]
Acrdi.XM_015922241.1/1-1044 | Monca.augustus.g50449.t1/1-1044	0.111045	0.0868752	D-arabinitol dehydrogenase 1	3	F:GO:0008270; F:GO:0016491; P:GO:0055114	[illegible]
Acrdi.XM_015906091.1/1-891 | Monca.augustus.g34429.t1/1-2016	0.267249	0.0868609	nuclear factor 1 X-type			
Acrdi.XM_015922869.1/1-540 | Monca.augustus.g6372.t1/1-792	0.119092	0.0868546	THO complex subunit 4	10	F:GO:0000166; F:GO:0003676; C:GO:0005634; P:GO:0008380;	[illegible]
Acrdi.XM_015913618.1/1-1716 | Monca.augustus.g48691.t1/1-1716	0.105735	0.0868357	fucose-1-phosphate guanylyltransferase	1	F:GO:0016772	[illegible]
Acrdi.XM_015914457.1/1-1671 | Monca.augustus.g71052.t1/1-2001	0.112762	0.0868219	Signal recognition particle subunit SRP72	4	P:GO:0006614; F:GO:0008312; F:GO:0005047; C:GO:0005786	[illegible]
Acrdi.XM_015903815.1/1-1029 | Monca.augustus.g33836.t1/1-1491	0.0771819	0.0867884	FERM domain-containing	4	C:GO:0005856; C:GO:0005737; C:GO:0019898; F:GO:0008092	[illegible]
Acrdi.XM_015901190.1/1-1509 | Monca.augustus.g58580.t1/1-1506	0.119437	0.0867819	Arylsulfatase B	11	P:GO:0050794; C:GO:0043231; P:GO:0008152; F:GO:0004065;	[illegible]
Acrdi.XM_015924116.1/1-1518 | Monca.adi2mcaRNA35859_R0/1-1533	0.0944934	0.0867743	myelin expression factor 2 isoform X4	1	F:GO:0003676	[illegible]
Acrdi.XM_015900635.1/1-1023 | Monca.augustus.g28767.t1/1-1026	0.0720782	0.086768	dnaJ homolog 1	2	F:GO:0051082; P:GO:0006457	[illegible]
Acrdi.XM_015896653.1/1-1827 | Monca.adi2mcaRNA5620_R0/1-1869	0.0941849	0.0867666	Autophagy-related 9A	8	P:GO:0034727; P:GO:0044805; C:GO:0005768; P:GO:0034497;	[illegible]
Acrdi.XM_015903525.1/1-1950 | Monca.augustus.g34547.t1/1-1950	0.0898113	0.0867651	leishmanolysin-like peptidase	4	C:GO:0016020; P:GO:0006508; P:GO:0007155; F:GO:0004222	[illegible]
Acrdi.XM_015918404.1/1-603 | Monca.augustus.g22395.t1/1-918	0.127571	0.0867369	N(4)-(Beta-N-acetylglucosaminyl)-L-asparaginase-like isoform X2	1	F:GO:0016787	[illegible]
Acrdi.XM_015909776.1/1-1176 | Monca.augustus.g46895.t1/1-1176	0.0974518	0.0867086	omega-6 fatty acid endoplasmic reticulum isozyme 1-like	3	F:GO:0016491; C:GO:0016021; P:GO:0006631	[illegible]
Acrdi.XM_015920883.1/1-2811 | Monca.augustus.g30094.t1/1-2811	0.196075	0.0867073	PREDICTED: uncharacterized protein LOC107354407			
Acrdi.XM_015924628.1/1-1011 | Monca.augustus.g35401.t1/1-1119	0.0724949	0.0865783	Lipoyl mitochondrial	5	C:GO:0005739; F:GO:0016992; F:GO:0051536; P:GO:0009107;	[illegible]
Acrdi.XM_015903336.1/1-3708 | Monca.augustus.g29887.t1/1-3555	0.0949271	0.0865734	histone deacetylase 7 isoform X3			
Acrdi.XM_015896662.1/1-1998 | Monca.augustus.g66634.t1/1-2574	0.133973	0.0865616	glutamine-rich 2-like			
Acrdi.XM_015922616.1/1-1878 | Monca.augustus.g12421.t1/1-2493	0.0933765	0.0865436	arf-GAP with ANK repeat and PH domain-containing 3 isoform X2	1	P:GO:0065007	[illegible]
Acrdi.XM_015918141.1/1-858 | Monca.augustus.g2153.t1/1-855	0.126615	0.0865324	serine threonine- phosphatase mitochondrial isoform X1			
Acrdi.XM_015917901.1/1-783 | Monca.augustus.g39673.t1/1-774	0.0828032	0.0865228	Ankyrin PH and SEC7 domain containing secG			
Acrdi.XM_015918337.1/1-1200 | Monca.augustus.g35707.t1/1-1197	0.550187	0.0865178	stress response NST1-like			
Acrdi.XM_015906473.1/1-2826 | Monca.augustus.g22530.t1/1-3102	0.102754	0.0865053	MAGUK p55 subfamily member 5	3	C:GO:0043209; P:GO:0016043; P:GO:0044763	[illegible]
Acrdi.XM_015920970.1/1-1182 | Monca.augustus.g30429.t1/1-1353	0.107222	0.0864843	transmembrane 104	1	C:GO:0016020	[illegible]
Acrdi.XM_015914131.1/1-1203 | Monca.augustus.g24976.t1/1-2229	0.102348	0.08648	carboxy-terminal kinesin 2-like	5	F:GO:0016887; F:GO:0003777; C:GO:0005871; F:GO:0005488;	[illegible]
Acrdi.XM_015925228.1/1-2151 | Monca.augustus.g498.t1/1-2253	0.0837489	0.0864285	dynamin-1 isoform X1	1	F:GO:0000166	[illegible]
Acrdi.XM_015898840.1/1-2820 | Monca.augustus.g5119.t1/1-3438	0.0960325	0.0863835	focal adhesion kinase 1 isoform X5			
Acrdi.XM_015901188.1/1-1431 | Monca.augustus.g58584.t1/1-1434	0.0976517	0.0863644	arylsulfatase B-like	1	F:GO:0003824	[illegible]
Acrdi.XM_015892496.1/1-2112 | Monca.augustus.g36521.t1/1-2427	0.110797	0.0863582	mannosyl-oligosaccharide glucosidase-like	4	C:GO:0005789; P:GO:0006487; F:GO:0004573; P:GO:0009311	[illegible]
Acrdi.XM_015905829.1/1-621 | Monca.augustus.g31757.t1/1-624	0.175588	0.0863362	Homeobox Nkx-	3	C:GO:0005634; P:GO:0006355; F:GO:0043565	[illegible]
Acrdi.XM_015901706.1/1-1773 | Monca.augustus.g49656.t1/1-1824	0.107514	0.0863332	sodium- and chloride-dependent betaine transporter	4	C:GO:0005887; P:GO:0006836; F:GO:0005328; P:GO:0055085	[illegible]
Acrdi.XM_015899459.1/1-411 | Monca.augustus.g37245.t1/1-417	0.164761	0.0863232	Mediator of RNA polymerase II transcription subunit 31	7	C:GO:0070847; F:GO:0003713; P:GO:0048147; P:GO:0060173;	[illegible]
Acrdi.XM_015904596.1/1-2355 | Monca.augustus.g12662.t1/1-2352	0.10258	0.0862865	Splicing factor 45	2	C:GO:0016020; C:GO:0016021	[illegible]
Acrdi.XM_015917418.1/1-1017 | Monca.augustus.g66922.t1/1-951	0.100108	0.0862621	neuropeptide FF receptor 1-like	3	P:GO:0007165; C:GO:0016021; F:GO:0004871	[illegible]
Acrdi.XM_015891776.1/1-1356 | Monca.augustus.g64464.t1/1-2142	0.0424928	0.0862367	lactose-binding lectin l-2-like			
Acrdi.XM_015902526.1/1-3045 | Monca.adi2mcaRNA11889_R1/1-3195	0.107636	0.0862275	WD repeat-containing 17	2	C:GO:0016020; C:GO:0016021	[illegible]
Acrdi.XM_015894051.1/1-2052 | Monca.augustus.g14570.t1/1-1680	0.0978578	0.0861486	lipoxygenase homology domain-containing 1-like	6	P:GO:0007605; P:GO:0050982; F:GO:0005262; C:GO:0016020;	[illegible]
Acrdi.XM_015902296.1/1-1404 | Monca.augustus.g28484.t1/1-1545	0.0973316	0.0861233	Contactin-associated -like 2	2	C:GO:0016020; C:GO:0016021	[illegible]
Acrdi.XM_015899190.1/1-8841 | Monca.augustus.g49630.t1/1-7152	0.113889	0.0861053	ATP-binding cassette sub-family A member 2 isoform X3	6	F:GO:0005524; P:GO:0006869; F:GO:0042626; P:GO:0055085;	[illegible]
Acrdi.XM_015921198.1/1-819 | Monca.augustus.g35934.t1/1-798	0.103628	0.0860891	50S ribosomal L1	2	F:GO:0003723; C:GO:0015934	[illegible]
Acrdi.XM_015906960.1/1-861 | Monca.augustus.g49149.t1/1-1413	0.110989	0.0860779	RNA-binding cabeza-like isoform X1	3	F:GO:0000166; F:GO:0003676; F:GO:0008270	[illegible]
Acrdi.XM_015895969.1/1-975 | Monca.augustus.g22584.t1/1-984	0.074312	0.0860703	dehydrogenase reductase SDR family member 7-like isoform X1	1	C:GO:0016020	[illegible]
Acrdi.XM_015910319.1/1-2175 | Monca.augustus.g36429.t1/1-2784	0.0815264	0.0860614	Sarcosine mitochondrial	2	F:GO:0016491; P:GO:0055114	[illegible]
Acrdi.XM_015897960.1/1-1158 | Monca.augustus.g39408.t1/1-1155	0.10878	0.0860505	shaker-related potassium channel tsha2-like	5	P:GO:0006813; P:GO:0055085; F:GO:0005251; C:GO:0016021;	[illegible]
Acrdi.XM_015913612.1/1-780 | Monca.augustus.g48683.t1/1-1299	0.135519	0.0860476	phosphatase 1 regulatory subunit 36 isoform X2	2	F:GO:0019902; P:GO:0010923	[illegible]
Acrdi.XM_015919117.1/1-3114 | Monca.augustus.g35180.t1/1-2994	0.0949158	0.0860468	SLIT-ROBO Rho GTPase-activating 1 isoform X1	4	C:GO:0005737; F:GO:0005096; F:GO:0048365; P:GO:0030336	[illegible]
Acrdi.XM_015920221.1/1-333 | Monca.augustus.g62693.t1/1-333	0.1569	0.0860395	transmembrane 230-like	1	C:GO:0016021	[illegible]
Acrdi.XM_015906155.1/1-1002 | Monca.augustus.g9787.t1/1-1107	0.0942861	0.0860318	NAD-dependent epimerase dehydratase	2	F:GO:0050662; F:GO:0003824	[illegible]
Acrdi.XM_015915314.1/1-702 | Monca.augustus.g41263.t1/1-702	0.108318	0.0860271	lipoma HMGIC fusion partner-like 3	2	C:GO:0016020; C:GO:0016021	[illegible]
Acrdi.XM_015905816.1/1-1128 | Monca.augustus.g31749.t1/1-1296	0.135136	0.0860236	Endoplasmic reticulum resident 44	4	F:GO:0003756; C:GO:0005783; P:GO:0006457; P:GO:0034976	[illegible]
Acrdi.XM_015904346.1/1-1041 | Monca.augustus.g67666.t1/1-1056	0.0816415	0.0859942	neuropeptide FF receptor 1-like	2	P:GO:0007165; C:GO:0016020	[illegible]
Acrdi.XM_015907612.1/1-390 | Monca.augustus.g40423.t1/1-849	0.11623	0.0859935	#NAME?	11	C:GO:0005615; C:GO:0031526; P:GO:0046498; F:GO:0004719;	[illegible]
Acrdi.XM_015919067.1/1-1290 | Monca.adi2mcaRNA30220_R1/1-1338	0.101222	0.0859872	neuronal acetylcholine receptor subunit alpha-3-like isoform X4	5	F:GO:0005216; P:GO:0034220; C:GO:0045202; C:GO:0044425;	[illegible]
Acrdi.XM_015914266.1/1-1299 | Monca.augustus.g5908.t1/1-1518	0.103976	0.0859683	von Willebrand factor A domain-containing 9			
Acrdi.XM_015913123.1/1-519 | Monca.augustus.g25076.t1/1-510	0.0874246	0.0859458	SUMO-conjugating enzyme UBC9-B	7	F:GO:0000166; C:GO:0005634; F:GO:0061630; F:GO:0031625;	[illegible]
Acrdi.XM_015915168.1/1-2469 | Monca.augustus.g55097.t1/1-2703	0.103259	0.0859411	PAB-dependent poly(A)-specific ribonuclease subunit PAN2-like isofor	5	C:GO:0043229; F:GO:0004527; C:GO:0032991; C:GO:0044444;	[illegible]
Acrdi.XM_015897474.1/1-651 | Monca.augustus.g2239.t1/1-684	0.115775	0.085931	YIPF6	1	C:GO:0016021	[illegible]
Acrdi.XM_015920166.1/1-657 | Monca.augustus.g17373.t1/1-498	0.514079	0.085916	RNA-directed DNA polymerase from transposon X-element			
Acrdi.XM_015922898.1/1-1533 | Monca.adi2mcaRNA34453_R5/1-1080	0.445993	0.0858824	PREDICTED: uncharacterized protein LOC107356260			
Acrdi.XM_015915250.1/1-576 | Monca.augustus.g41244.t1/1-543	0.0861804	0.0858788	DNA damage-induced apoptosis suppressor isoform X4			
Acrdi.XM_015893048.1/1-5094 | Monca.augustus.g22603.t1/1-6900	0.100789	0.0858765	unc-79 homolog			
Acrdi.XM_015899528.1/1-729 | Monca.augustus.g46958.t1/1-876	0.0977402	0.0858575	Inorganic pyrophosphatase	4	F:GO:0000287; F:GO:0004427; C:GO:0005737; P:GO:0006796	[illegible]
Acrdi.XM_015905271.1/1-9606 | Monca.augustus.g23101.t1/1-8802	0.11122	0.0858489	furry homolog-like	3	F:GO:0005515; P:GO:0016043; P:GO:0044763	[illegible]
Acrdi.XM_015899469.1/1-3849 | Monca.augustus.g37242.t1/1-3846	0.119431	0.0858286	PREDICTED: uncharacterized protein LOC107334529			
Acrdi.XM_015901964.1/1-360 | Monca.augustus.g45529.t1/1-366	0.111157	0.0858251	Cytochrome c oxidase subunit mitochondrial	2	C:GO:0044425; C:GO:0005743	[illegible]
Acrdi.XM_015901012.1/1-1074 | Monca.adi2mcaRNA10310_R0/1-1041	0.0847117	0.0858012	dynactin subunit 2 isoform X2	4	C:GO:0015630; C:GO:0044430; P:GO:0007067; P:GO:0007017	[illegible]
Acrdi.XM_015909908.1/1-1113 | Monca.augustus.g28079.t1/1-1113	0.0810476	0.0857968	F-box only 16 isoform X1			
Acrdi.XM_015897040.1/1-2343 | Monca.augustus.g48388.t1/1-2316	0.0996104	0.0857905	Transportin-3	6	C:GO:0031965; C:GO:0005737; P:GO:0006606; F:GO:0008565;	[illegible]
Acrdi.XM_015918554.1/1-516 | Monca.augustus.g41494.t1/1-516	0.108069	0.0857869	transmembrane 208-like	1	P:GO:0006624	[illegible]
Acrdi.XM_015895645.1/1-1044 | Monca.adi2mcaRNA4390_R0/1-1053	0.0972413	0.0857752	calcium uptake mitochondrial isoform X2	1	F:GO:0005509	[illegible]
Acrdi.XM_015906514.1/1-993 | Monca.augustus.g22564.t1/1-990	0.0612465	0.0857607	5-hydroxytryptamine receptor 1A-alpha-like	5	F:GO:0001588; P:GO:0001963; P:GO:0007165; F:GO:0035240;	[illegible]
Acrdi.XM_015905725.1/1-627 | Monca.augustus.g40454.t1/1-933	0.134055	0.085732	general transcription factor IIH subunit 3 isoform X1			
Acrdi.XM_015915309.1/1-3168 | Monca.augustus.g25264.t1/1-3162	0.0786086	0.0857307	Polycystic kidney disease 1-like 2	3	P:GO:0050982; F:GO:0005262; C:GO:0016020	[illegible]
Acrdi.XM_015923574.1/1-1155 | Monca.augustus.g10817.t1/1-1155	0.108126	0.0857198	dopamine D2-like receptor	5	F:GO:0001588; P:GO:0001963; P:GO:0007186; F:GO:0035240;	[illegible]
Acrdi.XM_015915503.1/1-2700 | Monca.augustus.g11263.t1/1-2706	0.10703	0.0857136	Cilia- and flagella-associated 221	4	F:GO:0005516; P:GO:0060271; C:GO:0005929; P:GO:0003341	[illegible]
Acrdi.XM_015899095.1/1-1227 | Monca.augustus.g26246.t1/1-1224	0.123051	0.0856733	NAD kinase-like isoform X6	4	F:GO:0003951; P:GO:0019674; P:GO:0016310; P:GO:0006741	[illegible]
Acrdi.XM_015914235.1/1-2178 | Monca.augustus.g57433.t1/1-2346	0.120302	0.085644	progesterone-induced-blocking factor 1	3	P:GO:0048519; P:GO:0080090; P:GO:0031323	[illegible]
Acrdi.XM_015892328.1/1-534 | Monca.augustus.g5376.t1/1-534	0.137936	0.085643	Transmembrane 222	1	C:GO:0016021	[illegible]
Acrdi.XM_015915747.1/1-489 | Monca.augustus.g18823.t1/1-489	0.106447	0.0856253	Ragulator complex LAMTOR1	21	P:GO:0007040; P:GO:0007032; F:GO:0005085; C:GO:0005794;	[illegible]
Acrdi.XM_015906650.1/1-702 | Monca.augustus.g30808.t1/1-699	0.600149	0.0856242	PREDICTED: uncharacterized protein LOC107341247			
Acrdi.XM_015910433.1/1-1917 | Monca.augustus.g26309.t1/1-1944	0.0922585	0.0856185	zinc finger MYND domain-containing 11 isoform X1	7	C:GO:0005634; F:GO:0008270; F:GO:0003712; F:GO:0042393;	[illegible]
Acrdi.XM_015919863.1/1-819 | Monca.adi2mcaRNA31096_R6/1-993	0.135614	0.0856002	methionine-R-sulfoxide reductase B3			
Acrdi.XM_015901976.1/1-2412 | Monca.adi2mcaRNA11304_R0/1-2028	0.354632	0.0855796	RNA-directed DNA polymerase from mobile element jockey-like			
Acrdi.XM_015908515.1/1-462 | Monca.augustus.g46695.t1/1-585	0.0777156	0.0855625	Appr-1-p processing domain			
Acrdi.XM_015900322.1/1-870 | Monca.augustus.g11749.t1/1-1284	0.101838	0.0855609	4-hydroxyphenylpyruvate dioxygenase	3	F:GO:0051213; F:GO:0016701; P:GO:0044710	[illegible]
Acrdi.XM_015921579.1/1-2916 | Monca.augustus.g41673.t1/1-2880	0.112132	0.0855539	ER degradation-enhancing alpha-mannosidase 3 isoform X1	4	F:GO:0046872; P:GO:0008152; C:GO:0005783; F:GO:0016798	[illegible]
Acrdi.XM_015908307.1/1-441 | Monca.augustus.g34628.t1/1-441	0.180349	0.0854813	AN1-type zinc finger 2B isoform X2	2	F:GO:0008270; F:GO:0046872	[illegible]
Acrdi.XM_015916618.1/1-546 | Monca.adi2mcaRNA27425_R0/1-549	0.133835	0.0854765	Fanconi anemia-associated of 24 kDa	2	P:GO:0006281; C:GO:0043240	[illegible]
Acrdi.XM_015891666.1/1-1062 | Monca.augustus.g34618.t1/1-1062	0.100034	0.0854572	PREDICTED: uncharacterized protein LOC107326913	2	C:GO:0016020; C:GO:0016021	[illegible]
Acrdi.XM_015903001.1/1-1161 | Monca.augustus.g23676.t1/1-1161	0.0866226	0.0854427	Dual specificity phosphatase 1	5	P:GO:0000188; F:GO:0004725; P:GO:0001714; P:GO:0035335;	[illegible]
Acrdi.XM_015893278.1/1-1017 | Monca.adi2mcaRNA39346_R4/1-1047	0.402958	0.0854266	PREDICTED: uncharacterized protein LOC107328555			
Acrdi.XM_015907873.1/1-1716 | Monca.augustus.g70308.t1/1-1992	0.098866	0.0854012	Cactin			
Acrdi.XM_015911767.1/1-1182 | Monca.augustus.g4088.t1/1-1563	0.105515	0.0853618	major facilitator superfamily domain-containing 12-like	2	C:GO:0016020; C:GO:0016021	[illegible]
Acrdi.XM_015909548.1/1-840 | Monca.augustus.g16561.t1/1-933	0.134423	0.0853494	Very-long-chain 3-oxoacyl- reductase	2	F:GO:0016491; P:GO:0055114	[illegible]
Acrdi.XM_015921277.1/1-1572 | Monca.adi2mcaRNA32623_R0/1-1641	0.0976811	0.0853403	acid-sensing ion channel 1-like	1	P:GO:0006811	[illegible]
Acrdi.XM_015897156.1/1-510 | Monca.augustus.g59968.t1/1-1143	0.0891652	0.0853382	tetratricopeptide repeat 4			
Acrdi.XM_015900181.1/1-645 | Monca.augustus.g59998.t1/1-639	0.0917081	0.0853309	tctex1 domain-containing 1-B-like			
Acrdi.XM_015907900.1/1-2448 | Monca.augustus.g46293.t1/1-2475	0.145683	0.085324	CCR4-NOT transcription complex subunit 3	11	C:GO:0005634; F:GO:0005515; P:GO:0006351; P:GO:2000036;	[illegible]
Acrdi.XM_015921541.1/1-1053 | Monca.augustus.g42446.t1/1-1503	0.124151	0.0853214	Organic cation transporter	2	F:GO:0022857; C:GO:0016020	[illegible]
Acrdi.XM_015897103.1/1-2208 | Monca.augustus.g48358.t1/1-2220	0.087184	0.0852964	cGMP-dependent kinase 1-like	3	F:GO:0005524; F:GO:0004692; P:GO:0006468	[illegible]
Acrdi.XM_015916255.1/1-930 | Monca.adi2mcaRNA27030_R0/1-933	0.138881	0.0852945	Methionine adenosyltransferase 2 subunit	1	F:GO:0016740	[illegible]
Acrdi.XM_015917318.1/1-1956 | Monca.adi2mcaRNA28247_R0/1-1971	0.115994	0.0852911	Beta-glucuronidase	6	P:GO:0005975; C:GO:0005615; C:GO:0044194; F:GO:0005515;	[illegible]
Acrdi.XM_015900055.1/1-1839 | Monca.augustus.g31994.t1/1-2040	0.079988	0.0852843	Nucleolar 10	1	C:GO:0005634	[illegible]
Acrdi.XM_015896936.1/1-1557 | Monca.augustus.g56201.t1/1-1626	0.0789789	0.0852807	T-complex 1 subunit eta	12	C:GO:0005832; F:GO:0005524; F:GO:0042802; P:GO:0007339;	[illegible]
Acrdi.XM_015924453.1/1-1593 | Monca.augustus.g5072.t1/1-1548	0.10048	0.0852621	mothers against decapentaplegic homolog 4 isoform X2			
Acrdi.XM_015895029.1/1-1050 | Monca.adi2mcaRNA41398_R0/1-1041	0.0985711	0.0852295	UDP-glucuronic acid decarboxylase 1	4	P:GO:0033320; C:GO:0032580; C:GO:0016021; F:GO:0048040	[illegible]
Acrdi.XM_015913564.1/1-558 | Monca.augustus.g22325.t1/1-558	0.106069	0.0851881	Phosphatidylglycerophosphatase and -tyrosine phosphatase 1	4	F:GO:0004439; F:GO:0004725; P:GO:0035335; F:GO:0008138	[illegible]
Acrdi.XM_015917496.1/1-1014 | Monca.augustus.g32476.t1/1-1017	0.115809	0.085177	Solute carrier family 25 member 36	4	F:GO:0003735; C:GO:0016020; P:GO:0006810; P:GO:0006412	[illegible]
Acrdi.XM_015907279.1/1-2304 | Monca.adi2mcaRNA1711_R0/1-2346	0.135443	0.0851723	E3 ubiquitin- ligase PDZRN3-like	1	F:GO:0046872	[illegible]
Acrdi.XM_015895680.1/1-4311 | Monca.adi2mcaRNA405_R0/1-4350	0.125209	0.0851639	serine threonine- phosphatase 2A 56 kDa regulatory subunit epsilon is	2	P:GO:0007165; C:GO:0000159	[illegible]
Acrdi.XM_015917119.1/1-909 | Monca.adi2mcaRNA28021_R0/1-993	0.371583	0.0851312	PREDICTED: uncharacterized protein LOC107350879			
Acrdi.XM_015901468.1/1-1617 | Monca.augustus.g31710.t1/1-1602	0.0950389	0.0851165	NGFI-A-binding 1-like	4	C:GO:0005634; P:GO:0045892; F:GO:0000989; P:GO:0007399	[illegible]
Acrdi.XM_015909973.1/1-2544 | Monca.augustus.g10577.t1/1-2787	0.57598	0.0851044	PREDICTED: uncharacterized protein LOC107344317	1	F:GO:0003824	[illegible]
Acrdi.XM_015899985.1/1-555 | Monca.augustus.g6674.t1/1-1353	0.0851876	0.0850945	Histone acetyltransferase KAT8	18	P:GO:0006351; F:GO:0035064; P:GO:0043981; F:GO:0046972;	[illegible]
Acrdi.XM_015899547.1/1-2595 | Monca.augustus.g50191.t1/1-2658	0.0924814	0.0850808	non-lysosomal glucosylceramidase	3	F:GO:0004348; P:GO:0006680; C:GO:0016020	[illegible]
Acrdi.XM_015913125.1/1-888 | Monca.augustus.g25065.t1/1-954	0.0946149	0.0850744	PREDICTED: uncharacterized protein C6orf106 homolog	4	F:GO:0043130; C:GO:0000407; C:GO:0005776; P:GO:0016236	[illegible]
Acrdi.XM_015899505.1/1-441 | Monca.augustus.g37210.t1/1-456	0.115939	0.0850734	Sterile alpha motif domain-containing 15			
Acrdi.XM_015908507.1/1-942 | Monca.augustus.g46698.t1/1-939	0.135768	0.085039	pirin			
Acrdi.XM_015899928.1/1-879 | Monca.augustus.g57206.t1/1-1089	0.119422	0.0850328	O-mannose kinase	7	C:GO:0005789; F:GO:0005524; P:GO:0006493; F:GO:0019200;	[illegible]
Acrdi.XM_015912002.1/1-474 | Monca.adi2mcaRNA22313_R0/1-402	0.466324	0.0850056	PREDICTED: uncharacterized protein LOC107346232			
Acrdi.XM_015916921.1/1-3171 | Monca.adi2mcaRNA27795_R0/1-3099	0.0984757	0.0849346	structural maintenance of chromosomes 5 isoform X2			
Acrdi.XM_015915072.1/1-603 | Monca.augustus.g27921.t1/1-1002	0.0922296	0.0849132	Transmembrane 64	1	C:GO:0016021	[illegible]
Acrdi.XM_015911089.1/1-891 | Monca.adi2mcaRNA21338_R1/1-909	0.112499	0.0849037	mirror-image polydactyly gene 1 isoform X2			
Acrdi.XM_015917822.1/1-1005 | Monca.augustus.g42810.t1/1-996	0.412886	0.0848964	glyoxalase	2	F:GO:0051213; P:GO:0055114	[illegible]
Acrdi.XM_015921393.1/1-399 | Monca.augustus.g11101.t1/1-399	0.0919189	0.0848889	L-rhamnose 1-epimerase	2	F:GO:0016857; C:GO:0005737	[illegible]
Acrdi.XM_015921848.1/1-330 | Monca.augustus.g63509.t1/1-360	0.11532	0.0848877	UPF0691 C9orf116 homolog			
Acrdi.XM_015902349.1/1-3423 | Monca.adi2mcaRNA11688_R1/1-2940	0.235181	0.0848178	Pro-Pol poly			
Acrdi.XM_015921126.1/1-4494 | Monca.augustus.g12105.t1/1-2601	0.0994519	0.0847777	puromycin-sensitive aminopeptidase	7	F:GO:0008270; P:GO:0043171; C:GO:0005737; P:GO:0006508;	[illegible]
Acrdi.XM_015896533.1/1-1545 | Monca.augustus.g60644.t1/1-1887	0.140982	0.0847443	adiponectin receptor isoform X1	4	C:GO:0016021; F:GO:0042562; P:GO:0033211; C:GO:0005886	[illegible]
Acrdi.XM_015903614.1/1-2061 | Monca.augustus.g32276.t1/1-2070	0.108339	0.0846945	Double-strand break repair MRE11A	12	F:GO:0008408; P:GO:0090305; P:GO:0051321; P:GO:0051276;	[illegible]
Acrdi.XM_015918107.1/1-1998 | Monca.augustus.g30313.t1/1-1845	0.458945	0.0846906	Prolyl endopeptidase			
Acrdi.XM_015902086.1/1-2127 | Monca.augustus.g46398.t1/1-3063	0.0797121	0.0846798	Deubiquitinating VCIP135			
Acrdi.XM_015922328.1/1-714 | Monca.adi2mcaRNA33794_R8/1-1284	0.939098	0.0846709	RNA-directed DNA polymerase from mobile element jockey			
Acrdi.XM_015923588.1/1-1221 | Monca.augustus.g33327.t1/1-1173	0.128732	0.0846177	diphosphomevalonate decarboxylase	6	F:GO:0000166; P:GO:0019287; P:GO:0008203; C:GO:0005829;	[illegible]
Acrdi.XM_015904634.1/1-1296 | Monca.augustus.g12661.t1/1-1326	0.0987315	0.0846118	F-box only 3			
Acrdi.XM_015919043.1/1-987 | Monca.adi2mcaRNA30199_R0/1-627	0.101025	0.0846089	flap endonuclease 1	17	F:GO:0017108; P:GO:0090305; P:GO:0006284; P:GO:0006310;	[illegible]
Acrdi.XM_015917035.1/1-2970 | Monca.adi2mcaRNA27915_R0/1-2877	0.117199	0.0846035	centrosomal of 112 kDa isoform X1	1	C:GO:0005813	[illegible]
Acrdi.XM_015900758.1/1-1437 | Monca.augustus.g5463.t1/1-3018	0.120868	0.0845981	myosin-10-like isoform X16			
Acrdi.XM_015922503.1/1-1737 | Monca.augustus.g33868.t1/1-2028	0.118362	0.0845908	urocanate hydratase	1	F:GO:0016153	[illegible]
Acrdi.XM_015917512.1/1-1329 | Monca.augustus.g52769.t1/1-1518	0.3525	0.084565	PREDICTED: uncharacterized protein K02A2.6-like			
Acrdi.XM_015912329.1/1-10992 | Monca.augustus.g47194.t1/1-11778	0.439782	0.0845649	sacsin-like			
Acrdi.XM_015902414.1/1-1599 | Monca.augustus.g37580.t1/1-1599	0.120436	0.0845587	E3 ubiquitin- ligase MARCH5	2	F:GO:0046872; C:GO:0016020	[illegible]
Acrdi.XM_015923791.1/1-570 | Monca.augustus.g58442.t1/1-1113	0.0947364	0.0845576	Deoxynucleotidyltransferase terminal-interacting 1	3	C:GO:0005634; F:GO:0003677; F:GO:0031491	[illegible]
Acrdi.XM_015911457.1/1-1884 | Monca.adi2mcaRNA21702_R0/1-1764	0.0978006	0.0845214	E3 ubiquitin- ligase AMFR	15	P:GO:0065007; F:GO:0008270; F:GO:0061630; P:GO:0010033;	[illegible]
Acrdi.XM_015909362.1/1-390 | Monca.augustus.g50717.t1/1-372	0.104258	0.0844899	SNARE-associated Snapin-like	10	C:GO:0031083; C:GO:0048471; P:GO:0043393; C:GO:0008021;	[illegible]
Acrdi.XM_015903307.1/1-1767 | Monca.augustus.g60037.t1/1-1830	0.106834	0.0844828	AMP-binding	2	F:GO:0003824; P:GO:0008152	[illegible]
Acrdi.XM_015909578.1/1-915 | Monca.augustus.g18777.t1/1-795	0.0854867	0.0844701	Transcription factor Sox-14	1	P:GO:0006355	[illegible]
Acrdi.XM_015895148.1/1-858 | Monca.adi2mcaRNA8323_R1/1-1251	0.114481	0.0844584	alpha-N-acetylgalactosaminidase isoform X1	3	F:GO:0004557; P:GO:0016139; C:GO:0005737	[illegible]
Acrdi.XM_015902332.1/1-1170 | Monca.augustus.g28500.t1/1-3957	0.0490935	0.0844035	Niemann-Pick C1	29	P:GO:0009790; P:GO:0046718; P:GO:0006486; P:GO:0008202;	[illegible]
Acrdi.XM_015905507.1/1-1101 | Monca.augustus.g28275.t1/1-1101	0.108949	0.0843399	quinone oxidoreductase 2	3	F:GO:0008270; F:GO:0016491; P:GO:0055114	[illegible]
Acrdi.XM_015915593.1/1-813 | Monca.augustus.g5424.t1/1-849	0.0866977	0.0843287	REM2- and Rab-like small GTPase 1	3	F:GO:0005525; P:GO:0007264; C:GO:0005622	[illegible]
Acrdi.XM_015906538.1/1-1338 | Monca.augustus.g21903.t1/1-1230	0.115398	0.0843097	Polyribonucleotide 5 -hydroxyl-kinase Clp1	12	F:GO:0005524; C:GO:0005849; P:GO:0035087; P:GO:0006388;	[illegible]
Acrdi.XM_015913137.1/1-2631 | Monca.adi2mcaRNA23576_R0/1-2694	0.106558	0.0843052	WSC domain-containing 2-like	2	P:GO:0006508; F:GO:0004197	[illegible]
Acrdi.XM_015900666.1/1-876 | Monca.augustus.g55761.t1/1-1374	0.117757	0.084281	nacht and ankyrin domain			
Acrdi.XM_015896138.1/1-609 | Monca.augustus.g55599.t1/1-609	0.094447	0.0842539	Sodium potassium-transporting ATPase subunit beta-1-interacting 1	2	P:GO:0002028; C:GO:0016020	[illegible]
Acrdi.XM_015895579.1/1-11340 | Monca.augustus.g22266.t1/1-12024	0.10315	0.0841964	spectrin beta non-erythrocytic 5 isoform X4	2	F:GO:0003779; F:GO:0005543	[illegible]
Acrdi.XM_015899415.1/1-4608 | Monca.augustus.g35512.t1/1-4173	0.0838582	0.0841498	rho-associated kinase 2 isoform X1			
Acrdi.XM_015903083.1/1-930 | Monca.augustus.g45218.t1/1-933	0.0975394	0.0841383	fumarylacetoacetate hydrolase domain-containing 2-like	3	F:GO:0046872; P:GO:0008152; F:GO:0016787	[illegible]
Acrdi.XM_015906485.1/1-1647 | Monca.adi2mcaRNA1688_R0/1-1716	0.413561	0.0841382	RNA-directed DNA polymerase from mobile element jockey			
Acrdi.XM_015911656.1/1-597 | Monca.augustus.g27813.t1/1-1167	0.105249	0.0841244	transmembrane prolyl 4-hydroxylase-like	1	F:GO:0043167	[illegible]
Acrdi.XM_015899203.1/1-810 | Monca.adi2mcaRNA8293_R1/1-828	0.126624	0.0841008	Dual specificity mitogen-activated kinase kinase	6	F:GO:0005524; P:GO:0018108; F:GO:0004702; F:GO:0004713;	[illegible]
Acrdi.XM_015919665.1/1-3180 | Monca.augustus.g43845.t1/1-3879	0.104161	0.0841001	histone demethylase UTY-like			
Acrdi.XM_015921915.1/1-648 | Monca.augustus.g61104.t1/1-648	0.161727	0.0840804	ras-related Rab-1A	18	P:GO:0008360; P:GO:0008089; P:GO:0032482; P:GO:0007015;	[illegible]
Acrdi.XM_015924100.1/1-1629 | Monca.augustus.g7973.t1/1-1632	0.090092	0.084066	frizzled-5-like	11	P:GO:0090090; F:GO:0004930; F:GO:0042813; P:GO:0007186;	[illegible]
Acrdi.XM_015912967.1/1-729 | Monca.adi2mcaRNA23392_R0/1-702	0.0923072	0.0840597	Phosphoethanolamine N-methyltransferase	2	P:GO:0008152; F:GO:0008168	[illegible]
Acrdi.XM_015898540.1/1-312 | Monca.augustus.g33999.t1/1-522	0.101169	0.0840426	DET1- and DDB1-associated 1			
Acrdi.XM_015906852.1/1-2655 | Monca.adi2mcaRNA16880_R0/1-2502	0.0999413	0.0840201	atrial natriuretic peptide receptor 1	10	C:GO:0008074; F:GO:0005524; P:GO:0006182; C:GO:0016021;	[illegible]
Acrdi.XM_015911818.1/1-1233 | Monca.augustus.g7593.t1/1-1233	0.117745	0.0840092	PREDICTED: ninein-like			
Acrdi.XM_015917913.1/1-624 | Monca.augustus.g39694.t1/1-624	0.0797075	0.0840079	ankyrin SAM and basic leucine zipper domain-containing 1-like			
Acrdi.XM_015900389.1/1-1293 | Monca.augustus.g54211.t1/1-1293	0.0842666	0.0839953	probable G- coupled receptor 19	5	P:GO:0010033; P:GO:0009719; C:GO:0016021; P:GO:0051716;	[illegible]
Acrdi.XM_015896528.1/1-900 | Monca.augustus.g42961.t1/1-2022	0.112802	0.0839947	PREDICTED: uncharacterized protein LOC107331872			
Acrdi.XM_015918874.1/1-783 | Monca.adi2mcaRNA29962_R0/1-744	0.126368	0.0839921	gamma-butyrobetaine dioxygenase-like isoform X1	2	F:GO:0016491; P:GO:0055114	[illegible]
Acrdi.XM_015911427.1/1-846 | Monca.augustus.g7437.t1/1-846	0.0724393	0.0839692	FAM92A1 isoform X2	3	P:GO:0008150; C:GO:0005575; F:GO:0003674	[illegible]
Acrdi.XM_015924216.1/1-633 | Monca.augustus.g38916.t1/1-609	0.291908	0.0839515	THAP domain-containing 10-like			
Acrdi.XM_015919567.1/1-543 | Monca.adi2mcaRNA307_R0/1-708	0.123979	0.0839473	urease accessory F-like isoform X2	3	C:GO:0005737; P:GO:0006807; F:GO:0016151	[illegible]
Acrdi.XM_015916340.1/1-768 | Monca.augustus.g35033.t1/1-771	0.125351	0.0839384	G patch domain-containing 11	2	F:GO:0003676; C:GO:0000776	[illegible]
Acrdi.XM_015911966.1/1-1413 | Monca.augustus.g24224.t1/1-3954	0.0954276	0.0839252	Rap guanine nucleotide exchange factor	4	P:GO:0007264; F:GO:0005085; C:GO:0005622; P:GO:0043547	[illegible]
Acrdi.XM_015896104.1/1-465 | Monca.augustus.g63100.t1/1-573	0.0843591	0.0839058	Biogenesis of lysosome-related organelles complex 1 subunit 5	2	C:GO:0031083; P:GO:0016197	[illegible]
Acrdi.XM_015920664.1/1-1977 | Monca.adi2mcaRNA31978_R0/1-1632	0.549308	0.0838612	ephrin type-A receptor 3-like isoform X5	2	F:GO:0016740; P:GO:0009987	[illegible]
Acrdi.XM_015907531.1/1-2733 | Monca.augustus.g45778.t1/1-2700	0.113007	0.0838529	probable E3 ubiquitin- ligase HECTD2	5	C:GO:0005634; F:GO:0004842; C:GO:0005737; F:GO:0016874;	[illegible]
Acrdi.XM_015917085.1/1-450 | Monca.adi2mcaRNA27972_R5/1-660	0.369988	0.0838304	P2X purinoceptor 7-like	3	F:GO:0004888; C:GO:0016021; C:GO:0044464	[illegible]
Acrdi.XM_015916612.1/1-744 | Monca.augustus.g22961.t1/1-768	0.141848	0.0838279	transcriptional repressor scratch 1-like	2	F:GO:0003676; F:GO:0046872	[illegible]
Acrdi.XM_015904812.1/1-1479 | Monca.augustus.g59065.t1/1-1572	0.144089	0.0838159	Leucine-rich repeat-containing 74A	1	F:GO:0005509	[illegible]
Acrdi.XM_015896602.1/1-1065 | Monca.augustus.g1586.t1/1-1065	0.141153	0.0838086	class 2 F G isoform isoform X1	2	F:GO:0003700; P:GO:0006355	[illegible]
Acrdi.XM_015918657.1/1-1464 | Monca.adi2mcaRNA29732_R0/1-1494	0.0872893	0.0837996	gamma-aminobutyric acid receptor subunit beta-like	4	P:GO:0034220; C:GO:0016021; F:GO:0005230; C:GO:0005886	[illegible]
Acrdi.XM_015903023.1/1-516 | Monca.augustus.g47575.t1/1-663	0.103574	0.0837774	Exosome complex component CSL4	3	C:GO:0000176; F:GO:0003723; C:GO:0005737	[illegible]
Acrdi.XM_015899664.1/1-696 | Monca.augustus.g8468.t1/1-669	0.080041	0.0837658	peptide mitochondrial	4	F:GO:0005506; P:GO:0006464; F:GO:0042586; P:GO:0006412	[illegible]
Acrdi.XM_015917003.1/1-702 | Monca.augustus.g26591.t1/1-702	0.106784	0.0837423	AP-1 complex-associated regulatory -like	6	F:GO:0035650; P:GO:0048203; P:GO:2000146; P:GO:1900025;	[illegible]
Acrdi.XM_015921273.1/1-1029 | Monca.adi2mcaRNA32618_R1/1-951	0.137749	0.0837379	vesicular glutamate transporter 3 isoform X2			
Acrdi.XM_015920934.1/1-1473 | Monca.augustus.g16464.t1/1-1473	0.0908199	0.0837032	A-kinase anchor 17A			
Acrdi.XM_015902177.1/1-3462 | Monca.augustus.g34155.t1/1-3543	0.110173	0.0836933	disabled homolog 2-interacting isoform X2	1	P:GO:0065007	[illegible]
Acrdi.XM_015908120.1/1-1530 | Monca.augustus.g48085.t1/1-1533	0.114143	0.0836749	deoxyribodipyrimidine photo-lyase-like	2	P:GO:0006281; F:GO:0003904	[illegible]
Acrdi.XM_015910864.1/1-831 | Monca.augustus.g70037.t1/1-837	0.135391	0.0836625	PREDICTED: uncharacterized protein LOC107345155			
Acrdi.XM_015912165.1/1-930 | Monca.augustus.g55529.t1/1-930	0.137019	0.0836583	ZDHHC-type palmitoyltransferase 8	2	F:GO:0008270; F:GO:0019706	[illegible]
Acrdi.XM_015898417.1/1-2121 | Monca.augustus.g43981.t1/1-2418	0.0987049	0.0836489	Nuclear cap-binding subunit 1	4	C:GO:0005846; P:GO:0045292; F:GO:0000339; P:GO:0051028	[illegible]
Acrdi.XM_015919591.1/1-1959 | Monca.augustus.g7144.t1/1-2124	0.0954968	0.0836488	WD repeat-containing 48	6	C:GO:0043229; C:GO:0005737; P:GO:0048513; P:GO:0009987;	[illegible]
Acrdi.XM_015923797.1/1-543 | Monca.adi2mcaRNA21286_R0/1-309	0.293518	0.0836437	PREDICTED: uncharacterized protein LOC107357153			
Acrdi.XM_015894627.1/1-1236 | Monca.adi2mcaRNA40936_R0/1-1140	0.509274	0.0836171	craniofacial development 2-like			
Acrdi.XM_015903222.1/1-1356 | Monca.adi2mcaRNA1326_R0/1-1395	0.149471	0.083601	autophagy-related 16-1 isoform X2	3	P:GO:0000045; F:GO:0016740; C:GO:0000421	[illegible]
Acrdi.XM_015893365.1/1-4089 | Monca.augustus.g30625.t1/1-4983	0.12681	0.0835781	catenin alpha-1-like isoform X2	4	F:GO:0051015; F:GO:0005198; C:GO:0015629; P:GO:0007155	[illegible]
Acrdi.XM_015921301.1/1-2745 | Monca.augustus.g40039.t1/1-2649	0.104241	0.0835764	glutamate receptor NMDA 1-like	7	C:GO:0045211; P:GO:0034220; C:GO:0030054; C:GO:0017146;	[illegible]
Acrdi.XM_015914470.1/1-516 | Monca.augustus.g57201.t1/1-516	0.130907	0.0835596	transmembrane 128	1	C:GO:0016021	[illegible]
Acrdi.XM_015895753.1/1-903 | Monca.augustus.g60349.t1/1-1344	0.0842277	0.0835563	delta(8)-fatty-acid desaturase-like	4	F:GO:0016491; P:GO:0006629; C:GO:0016021; P:GO:0055114	[illegible]
Acrdi.XM_015907418.1/1-2769 | Monca.augustus.g41497.t1/1-4584	0.106992	0.0835542	ubiquitin-conjugating enzyme E2	4	F:GO:0004842; C:GO:0005737; P:GO:0009966; P:GO:0016567	[illegible]
Acrdi.XM_015919304.1/1-1203 | Monca.adi2mcaRNA3051_R0/1-1212	0.0934555	0.083528	Transmembrane 26	2	C:GO:0016020; C:GO:0016021	[illegible]

Acrdi.XM_015897311.1/1-333 | Monca.augustus.g67498.t1/1-333	0.0700484	0.0835118	eukaryotic translation initiation factor 1B	2	F:GO:0003743; P:GO:0006413	F:translation initiation factor activity; P:translational initiation	
Acrdi.XM_015904485.1/1-1347 | Monca.augustus.g68195.t1/1-1290	0.130614	0.0834772	disulfide with oxidoreductase activity	3	F:GO:0003824; P:GO:0008152; F:GO:0005488	F:catalytic activity; P:metabolic process; F:binding	
Acrdi.XM_015899840.1/1-1617 | Monca.adi2mcaRNA8957_R0/1-1614	0.124184	0.0834649	coiled-coil domain-containing 61				
Acrdi.XM_015924634.1/1-1233 | Monca.augustus.g35416.t1/1-1233	0.101023	0.0834611	hsc70-interacting	1	C:GO:0005737	C:cytoplasm	
Acrdi.XM_015907794.1/1-1338 | Monca.augustus.g55793.t1/1-1347	0.0655137	0.0834493	potassium voltage-gated channel Shal-like	3	F:GO:0005249; C:GO:0016021; P:GO:0006810	F:voltage-gated potassium channel activity; C:integral component of membrane; P:transport	
Acrdi.XM_015906942.1/1-1542 | Monca.augustus.g49134.t1/1-1563	0.138258	0.0834358	T-box transcription factor TBX3	10	C:GO:0005634; F:GO:0003677; P:GO:0030326; F:GO:0003700; [illegible]	C:nucleus; F:DNA binding; P:embryonic limb morphogenesis; F:transcription factor activity, sequence-specific DNA binding; P:genitalia development; P:gonadotropin secretion; [illegible]	
Acrdi.XM_015893210.1/1-4329 | Monca.augustus.g69407.t1/1-5820	0.0924452	0.0834158	WD repeat-containing 90				
Acrdi.XM_015898102.1/1-2808 | Monca.adi2mcaRNA241_R0/1-2835	0.12068	0.0834104	acyl- dehydrogenase family member 10	8	F:GO:0003995; P:GO:0033539; F:GO:0000062; F:GO:0050660; [illegible]	F:acyl-CoA dehydrogenase activity; P:fatty acid beta-oxidation using acyl-CoA dehydrogenase; F:fatty-acyl-CoA binding; F:flavin adenine dinucleotide binding; F:electron carrie[illegible]	EC:1.[illegible]
Acrdi.XM_015922270.1/1-7494 | Monca.adi2mcaRNA33714_R0/1-7860	0.434448	0.0833415	NFX1-type zinc finger-containing 1	4	C:GO:0005634; F:GO:0003700; F:GO:0008270; P:GO:0006355	C:nucleus; F:transcription factor activity, sequence-specific DNA binding; F:zinc ion binding; P:regulation of transcription, DNA-templated	
Acrdi.XM_015913134.1/1-1269 | Monca.augustus.g10874.t1/1-1422	0.119762	0.083335	cytoplasmic polyadenylation element-binding 1 isoform X2	15	F:GO:0000900; F:GO:0008135; C:GO:0045202; C:GO:0043005; [illegible]	F:translation repressor activity, nucleic acid binding; F:translation factor activity, RNA binding; C:synapse; C:neuron projection; P:positive regulation of biological process; C:nucleop[illegible]	
Acrdi.XM_015915246.1/1-438 | Monca.augustus.g30608.t1/1-438	0.0902375	0.0833163	FAM136A-like				
Acrdi.XM_015923151.1/1-3000 | Monca.augustus.g39997.t1/1-3126	0.096616	0.0833155	FACT complex subunit SPT16	8	P:GO:0032968; C:GO:0035101; F:GO:0042393; F:GO:0031491; [illegible]	P:positive regulation of transcription elongation from RNA polymerase II promoter; C:FACT complex; F:histone binding; F:nucleosome binding; P:DNA metabolic process; P:cellular [illegible]	
Acrdi.XM_015898105.1/1-930 | Monca.augustus.g50133.t1/1-1308	0.126379	0.0832997	Phosphonopyruvate decarboxylase	4	F:GO:0000287; F:GO:0030976; P:GO:0032923; F:GO:0033980	F:magnesium ion binding; F:thiamine pyrophosphate binding; P:organic phosphonate biosynthetic process; F:phosphonopyruvate decarboxylase activity	EC:4.[illegible]
Acrdi.XM_015910826.1/1-612 | Monca.augustus.g57326.t1/1-609	0.111002	0.0832788	FGFR1 oncogene partner 2 isoform X2	3	P:GO:0042060; F:GO:0042803; C:GO:0005737	P:wound healing; F:protein homodimerization activity; C:cytoplasm	
Acrdi.XM_015920068.1/1-2076 | Monca.augustus.g41999.t1/1-2154	0.104657	0.0832509	ATP-binding cassette sub-family B member mitochondrial	4	F:GO:0000166; F:GO:0016887; C:GO:0016020; P:GO:0006810	F:nucleotide binding; F:ATPase activity; C:membrane; P:transport	EC:3.[illegible]
Acrdi.XM_015908943.1/1-666 | Monca.augustus.g49874.t1/1-813	0.0933242	0.0832356	sorting nexin-5-like	5	C:GO:0005768; P:GO:0016050; F:GO:0035091; C:GO:0019898; [illegible]	C:endosome; P:vesicle organization; F:phosphatidylinositol binding; C:extrinsic component of membrane; P:endocytosis	
Acrdi.XM_015899044.1/1-771 | Monca.augustus.g8388.t1/1-759	0.452331	0.0832007	myb SANT-like DNA-binding domain-containing 4	2	C:GO:0005634; P:GO:0006915	C:nucleus; P:apoptotic process	
Acrdi.XM_015913423.1/1-1059 | Monca.augustus.g32119.t1/1-1197	0.0994727	0.0831956	Sec14 cytosolic factor	1	C:GO:0044464	C:cell part	
Acrdi.XM_015911912.1/1-3246 | Monca.adi2mcaRNA22197_R0/1-3225	0.127188	0.083186	Arylsulfatase J	2	F:GO:0008484; P:GO:0008152	F:sulfuric ester hydrolase activity; P:metabolic process	
Acrdi.XM_015908882.1/1-363 | Monca.adi2mcaRNA18888_R2/1-585	0.657533	0.0831785	Transposon TX1 uncharacterized 149 kDa				
Acrdi.XM_015904658.1/1-615 | Monca.augustus.g7180.t1/1-615	0.146596	0.0831768	ras-related ARA-3	5	F:GO:0005525; P:GO:0007264; P:GO:0006904; C:GO:0044424; [illegible]	F:GTP binding; P:small GTPase mediated signal transduction; P:vesicle docking involved in exocytosis; C:intracellular part; P:protein secretion	
Acrdi.XM_015903085.1/1-951 | Monca.augustus.g45210.t1/1-957	0.123403	0.0831758	proline-rich 5-like	1	P:GO:0065007	P:biological regulation	
Acrdi.XM_015907576.1/1-546 | Monca.adi2mcaRNA17442_R0/1-615	0.139078	0.0831757	Transposable element P transposase				
Acrdi.XM_015901011.1/1-1671 | Monca.augustus.g57639.t1/1-2313	0.0927735	0.0831487	tripartite motif-containing 2-like isoform X1	2	F:GO:0008270; C:GO:0005622	F:zinc ion binding; C:intracellular	
Acrdi.XM_015900630.1/1-993 | Monca.augustus.g28766.t1/1-999	0.107067	0.0831461	dnaJ homolog subfamily B member 5 isoform X2	2	F:GO:0051082; P:GO:0006457	F:unfolded protein binding; P:protein folding	
Acrdi.XM_015911185.1/1-900 | Monca.augustus.g71194.t1/1-900	0.122872	0.0831289	Vesicle-trafficking SEC22a	6	P:GO:0006888; F:GO:0005484; C:GO:0031201; F:GO:0000149; [illegible]	P:ER to Golgi vesicle-mediated transport; F:SNAP receptor activity; C:SNARE complex; F:SNARE binding; P:exocytosis; P:vesicle fusion with Golgi apparatus	
Acrdi.XM_015903057.1/1-681 | Monca.augustus.g10955.t1/1-675	0.113902	0.0831055	Nicotinamide nicotinic acid mononucleotide adenylyltransferase 1	5	F:GO:0000166; F:GO:0004515; P:GO:0034628; F:GO:0000309; [illegible]	F:nucleotide binding; F:nicotinate-nucleotide adenylyltransferase activity; P:'de novo' NAD biosynthetic process from aspartate; F:nicotinamide-nucleotide adenylyltransfera[illegible]	EC:2.[illegible]
Acrdi.XM_015906146.1/1-1578 | Monca.augustus.g29205.t1/1-1602	0.0784592	0.0831017	nucleolar 58	4	C:GO:0031428; C:GO:0032040; P:GO:0000154; F:GO:0030515	C:box C/D snoRNP complex; C:small-subunit processome; P:rRNA modification; F:snoRNA binding	
Acrdi.XM_015915909.1/1-744 | Monca.augustus.g24365.t1/1-744	0.0847339	0.0830989	3-hydroxybutyrate dehydrogenase type 2	8	P:GO:0055072; C:GO:0005737; F:GO:0016628; F:GO:0003858; [illegible]	P:iron ion homeostasis; C:cytoplasm; F:oxidoreductase activity, acting on the CH-CH group of donors, NAD or NADP as acceptor; F:3-hydroxybutyrate dehydrogenase activity; P[illegible]	EC:1.[illegible]
Acrdi.XM_015919330.1/1-1425 | Monca.augustus.g71335.t1/1-1422	0.119834	0.0830644	potassium voltage-gated channel subfamily A member 2-like	5	P:GO:0071805; C:GO:0008076; F:GO:0005251; P:GO:0034765; [illegible]	P:potassium ion transmembrane transport; C:voltage-gated potassium channel complex; F:delayed rectifier potassium channel activity; P:regulation of ion transmembrane transp[illegible]	
Acrdi.XM_015908323.1/1-666 | Monca.augustus.g17448.t1/1-1098	0.0985658	0.0830459	centrosomal of 41 kDa isoform X1	6	P:GO:0018095; C:GO:0005815; C:GO:0005737; C:GO:0005929; [illegible]	P:protein polyglutamylation; C:microtubule organizing center; C:cytoplasm; C:cilium; F:tubulin-glutamic acid ligase activity; P:axoneme assembly	
Acrdi.XM_015898882.1/1-804 | Monca.augustus.g8982.t1/1-807	0.0973567	0.0830439	armadillo repeat-containing 1-like	2	C:GO:0005634; C:GO:0005737	C:nucleus; C:cytoplasm	
Acrdi.XM_015904339.1/1-561 | Monca.augustus.g61770.t1/1-561	0.0978211	0.0830363	COP9 signalosome complex subunit 8	2	C:GO:0044424; P:GO:0009987	C:intracellular part; P:cellular process	
Acrdi.XM_015899324.1/1-834 | Monca.augustus.g64402.t1/1-894	0.079089	0.0829987	Ras-related and estrogen-regulated growth inhibitor	6	F:GO:0000166; F:GO:0005525; P:GO:0007264; P:GO:0007165; [illegible]	F:nucleotide binding; F:GTP binding; P:small GTPase mediated signal transduction; P:signal transduction; C:membrane; C:intracellular	
Acrdi.XM_015904758.1/1-651 | Monca.augustus.g31925.t1/1-648	0.142535	0.0829973	POU class transcription factor	5	C:GO:0005634; F:GO:0003700; P:GO:0006351; P:GO:0006355; [illegible]	C:nucleus; F:transcription factor activity, sequence-specific DNA binding; P:transcription, DNA-templated; P:regulation of transcription, DNA-templated; F:sequence-specific DNA b[illegible]	
[illegible]	[illegible]	[illegible]	[illegible]	[illegible]	[illegible]	[illegible]	[illegible]

Acrdi.XM_015899593.1/1-1389	Monca.adi2mcaRNA8685_R0/1-1392	0.0809134	0.07786	elongation factor 1-alpha	14	[illegible]
Acrdi.XM_015892497.1/1-990	Monca.augustus.g57754.t1/1-1206	0.113414	0.0778523	transmembrane 184C	1	[illegible]
Acrdi.XM_015918696.1/1-4071	Monca.adi2mcaRNA29766_R1/1-3828	0.363249	0.0778329	PREDICTED: uncharacterized protein K02A2.6-like		
Acrdi.XM_015906417.1/1-3372	Monca.adi2mcaRNA16154_R0/1-3327	0.0826292	0.0778012	probable phospholipid-transporting ATPase IF isoform X3	5	[illegible]
Acrdi.XM_015902730.1/1-993	Monca.adi2mcaRNA12104_R0/1-876	0.163657	0.0777733	CCAAT enhancer-binding gamma	4	[illegible]
Acrdi.XM_015910381.1/1-1188	Monca.augustus.g10408.t1/1-1536	0.0683502	0.077766	COP9 signalosome complex subunit 1	2	[illegible]
Acrdi.XM_015917694.1/1-489	Monca.augustus.g42283.t1/1-609	0.117723	0.0777561	28S ribosomal mitochondrial	1	[illegible]
Acrdi.XM_015902234.1/1-1362	Monca.augustus.g58546.t1/1-1389	0.0926834	0.0777327	probable serine-O-acetyltransferase cys2	6	[illegible]
Acrdi.XM_015895830.1/1-1710	Monca.augustus.g63625.t1/1-1851	0.109568	0.0777037	kelch 28	3	[illegible]
Acrdi.XM_015892247.1/1-825	Monca.augustus.g44167.t1/1-861	0.130804	0.0776881	toll-interacting isoform X2	9	[illegible]
Acrdi.XM_015898057.1/1-1104	Monca.augustus.g18522.t1/1-3135	0.0777774	0.0776682	mitogen-activated kinase kinase kinase kinase	4	[illegible]
Acrdi.XM_015901861.1/1-654	Monca.augustus.g56987.t1/1-654	0.0753689	0.077654	mRNA turnover 4 homolog	12	[illegible]
Acrdi.XM_015919198.1/1-885	Monca.augustus.g54951.t1/1-885	0.108897	0.0776285	Solute carrier family 25 member 44	4	[illegible]
Acrdi.XM_015911034.1/1-963	Monca.augustus.g19167.t1/1-999	0.108764	0.0776223	RNA demethylase ALKBH5	8	[illegible]
Acrdi.XM_015897158.1/1-1269	Monca.adi2mcaRNA6110_R0/1-1101	0.093458	0.0776156	intraflagellar transport 74 homolog isoform X1	7	[illegible]
Acrdi.XM_015921835.1/1-540	Monca.augustus.g66170.t1/1-1011	0.0718546	0.077584	synaptojanin-2-binding -like	13	[illegible]
Acrdi.XM_015896757.1/1-1977	Monca.adi2mcaRNA567_R1/1-1569	0.518288	0.0775817	Serine threonine- kinase		
Acrdi.XM_015916325.1/1-1500	Monca.augustus.g64079.t1/1-1500	0.10397	0.0775781	ankyrin repeat and BTB POZ domain-containing 1		
Acrdi.XM_015916699.1/1-1188	Monca.adi2mcaRNA27540_R0/1-1140	0.0791556	0.0775623	Lactation elevated 1	1	[illegible]
Acrdi.XM_015922967.1/1-636	Monca.augustus.g4481.t1/1-633	0.908137	0.0775615	perlucin isoform X1		
Acrdi.XM_015902538.1/1-2823	Monca.augustus.g39923.t1/1-2883	0.0735042	0.077523	Coiled-coil domain-containing 146	1	[illegible]
Acrdi.XM_015901103.1/1-1341	Monca.augustus.g1880.t1/1-1410	0.119	0.077521	serine threonine- kinase Chk1 isoform X2	16	[illegible]
Acrdi.XM_015893076.1/1-894	Monca.adi2mcaRNA39114_R2/1-1023	0.704117	0.0775149	RNA-directed DNA polymerase from mobile element jockey-like		
Acrdi.XM_015914896.1/1-1077	Monca.adi2mcaRNA25530_R1/1-1062	0.0780302	0.0774811	nucleoporin p54 isoform X2	6	[illegible]
Acrdi.XM_015919745.1/1-1338	Monca.augustus.g67172.t1/1-1452	0.0909571	0.0774762	serine threonine- phosphatase 2B catalytic subunit gamma isoform isc	2	[illegible]
Acrdi.XM_015894072.1/1-1596	Monca.adi2mcaRNA40279_R0/1-1530	0.072671	0.0774742	phosphorylase kinase alpha beta	5	[illegible]
Acrdi.XM_015921380.1/1-1917	Monca.augustus.g35971.t1/1-2118	0.0910492	0.0774621	Adenylate kinase 7	4	[illegible]
Acrdi.XM_015901728.1/1-615	Monca.augustus.g34399.t1/1-615	0.0893511	0.077448	GTP-binding Di-Ras3	6	[illegible]
Acrdi.XM_015924128.1/1-1806	Monca.augustus.g66164.t1/1-3282	0.108159	0.0774334	rho guanine nucleotide exchange factor 10 isoform X4	10	[illegible]
Acrdi.XM_015923413.1/1-2151	Monca.augustus.g30577.t1/1-2145	0.107671	0.077413	engulfment and cell motility 1 isoform X2	3	[illegible]
Acrdi.XM_015906481.1/1-1386	Monca.augustus.g22571.t1/1-1308	0.0770919	0.0774018	transcriptional adapter 2-beta	9	[illegible]
Acrdi.XM_015904527.1/1-966	Monca.augustus.g68200.t1/1-969	0.0814106	0.0774007	RNA-binding 42	4	[illegible]
Acrdi.XM_015905843.1/1-3369	Monca.augustus.g32197.t1/1-4350	0.111596	0.0774005	nuclear pore complex Nup160	2	[illegible]
Acrdi.XM_015917533.1/1-1800	Monca.augustus.g25120.t1/1-2505	0.0978936	0.0773845	Pro convertase subtilisin kexin type 7	2	[illegible]
Acrdi.XM_015914038.1/1-1143	Monca.augustus.g18742.t1/1-1143	0.118491	0.0773653	nucleoporin Nup43	1	[illegible]
Acrdi.XM_015912128.1/1-1122	Monca.augustus.g60701.t1/1-3090	0.0814984	0.0773519	Lethal(3)malignant brain tumor 1	4	[illegible]
Acrdi.XM_015911015.1/1-1086	Monca.adi2mcaRNA21241_R1/1-1629	0.579428	0.0773274	Pro-Pol poly		
Acrdi.XM_015900512.1/1-1065	Monca.augustus.g49620.t1/1-1215	0.110207	0.0773226	TBC1 domain family member 2B-like	7	[illegible]
Acrdi.XM_015904944.1/1-1086	Monca.augustus.g62753.t1/1-849	0.52155	0.0772925	ATP-dependent DNA helicase		
Acrdi.XM_015897979.1/1-1359	Monca.augustus.g33361.t1/1-1350	0.0808537	0.0772873	gamma-aminobutyric acid receptor subunit beta-2-like	4	[illegible]
Acrdi.XM_015899426.1/1-2427	Monca.adi2mcaRNA8499_R0/1-2436	0.130838	0.0772747	ABC transporter B family member 27-like isoform X1	4	[illegible]
Acrdi.XM_015897196.1/1-1893	Monca.augustus.g63832.t1/1-1887	0.082601	0.0772658	Kelch 20	3	[illegible]
Acrdi.XM_015907364.1/1-2505	Monca.augustus.g48612.t1/1-2553	0.349912	0.0772632	PREDICTED: uncharacterized protein LOC107341895	5	[illegible]
Acrdi.XM_015909104.1/1-3279	Monca.augustus.g23907.t1/1-4464	0.105176	0.0772613	canalicular multispecific organic anion transporter 2	4	[illegible]
Acrdi.XM_015895697.1/1-2328	Monca.augustus.g2245.t1/1-2325	0.0938163	0.0772506	kinesin KIF9	7	[illegible]
Acrdi.XM_015918577.1/1-738	Monca.augustus.g15745.t1/1-1419	0.575278	0.0772326	PREDICTED: uncharacterized protein LOC107352247		
Acrdi.XM_015901046.1/1-1002	Monca.augustus.g12293.t1/1-999	0.106711	0.0772011	solute carrier family 35 member B1	4	[illegible]
Acrdi.XM_015921178.1/1-420	Monca.augustus.g21743.t1/1-900	0.0704889	0.0771897	palmitoyl- thioesterase 1-like	4	[illegible]
Acrdi.XM_015912751.1/1-744	Monca.augustus.g58412.t1/1-921	0.147142	0.0771564	ubiquitin domain-containing UBFD1 isoform X2		
Acrdi.XM_015900391.1/1-1278	Monca.augustus.g63390.t1/1-1320	0.0987136	0.0771513	BTN1-like	6	[illegible]
Acrdi.XM_015920235.1/1-1200	Monca.augustus.g62717.t1/1-1200	0.0760241	0.0771497	E3 ubiquitin- ligase RNF38 isoform X1		
Acrdi.XM_015910111.1/1-789	Monca.adi2mcaRNA20238_R0/1-789	0.130439	0.0771411	dolichyldiphosphatase 1 isoform X1	4	[illegible]
Acrdi.XM_015898985.1/1-633	Monca.augustus.g59496.t1/1-1383	0.127483	0.0771234	tubby homolog isoform X3	4	[illegible]
Acrdi.XM_015897742.1/1-1023	Monca.augustus.g49011.t1/1-1074	0.132212	0.0771169	arfaptin-2-like isoform X4	4	[illegible]
Acrdi.XM_015897145.1/1-2004	Monca.augustus.g63837.t1/1-1971	0.108145	0.0771099	FAS-associated factor 1	16	[illegible]
Acrdi.XM_015908859.1/1-1485	Monca.augustus.g45277.t1/1-1674	0.0914395	0.0770952	Tetratricopeptide repeat 39C	3	[illegible]
Acrdi.XM_015919314.1/1-645	Monca.augustus.g5827.t1/1-717	0.156956	0.0770481	Cysteine serine-rich nuclear 2	8	[illegible]
Acrdi.XM_015901892.1/1-1440	Monca.augustus.g17429.t1/1-1725	0.0938427	0.0770398	dynactin subunit 4 isoform X2	1	[illegible]
Acrdi.XM_015906157.1/1-783	Monca.augustus.g9786.t1/1-2703	0.0684442	0.0770292	GA-binding subunit beta-	6	[illegible]
Acrdi.XM_015917721.1/1-1698	Monca.augustus.g16591.t1/1-1848	0.0438404	0.0770217	plastin-1 isoform X2	2	[illegible]
Acrdi.XM_015911166.1/1-822	Monca.augustus.g24027.t1/1-1047	0.0790067	0.0770212	YIPF3	1	[illegible]
Acrdi.XM_015900003.1/1-5268	Monca.augustus.g70550.t1/1-5220	0.0793039	0.0769164	Sodium leak channel non-selective	5	[illegible]
Acrdi.XM_015898324.1/1-2979	Monca.adi2mcaRNA7339_R0/1-2979	0.101273	0.0768974	1-phosphatidylinositol 4,5-bisphosphate phosphodiesterase delta-3	2	[illegible]
Acrdi.XM_015897039.1/1-651	Monca.augustus.g70192.t1/1-1395	0.103731	0.0768739	annexin A7-like	2	[illegible]
Acrdi.XM_015912098.1/1-432	Monca.augustus.g9730.t1/1-450	0.121652	0.0768705	transmembrane 216-like	1	[illegible]
Acrdi.XM_015917205.1/1-9135	Monca.augustus.g59089.t1/1-8118	0.107045	0.0768627	E3 ubiquitin- ligase UBR5	6	[illegible]
Acrdi.XM_015895990.1/1-648	Monca.augustus.g51360.t1/1-645	0.122729	0.0768245	Homeobox GBX-1	4	[illegible]
Acrdi.XM_015904943.1/1-948	Monca.augustus.g64929.t1/1-1038	1.06679	0.0768174	ATP-dependent DNA helicase -like		
Acrdi.XM_015896534.1/1-1413	Monca.augustus.g60642.t1/1-1440	0.112402	0.0768083	N-acetylgalactosamine kinase isoform X1	5	[illegible]
Acrdi.XM_015922934.1/1-801	Monca.adi2mcaRNA34517_R0/1-771	0.0542614	0.0768058	OTU domain-containing 5 isoform X5	2	[illegible]
Acrdi.XM_015894718.1/1-2091	Monca.augustus.g51675.t1/1-2802	0.102566	0.0767777	WD repeat-containing 66		
Acrdi.XM_015914775.1/1-828	Monca.augustus.g286.t1/1-822	0.135639	0.076741	AN1-type zinc finger 2A-like	2	[illegible]
Acrdi.XM_015895746.1/1-2985	Monca.adi2mcaRNA4649_R0/1-3240	0.455533	0.076736	PREDICTED: uncharacterized protein LOC107331194		
Acrdi.XM_015923705.1/1-342	Monca.augustus.g34978.t1/1-342	0.133004	0.0767058	churchill	3	[illegible]
Acrdi.XM_015893897.1/1-1434	Monca.adi2mcaRNA40042_R2/1-1128	0.452988	0.0766968	reverse transcriptase		
Acrdi.XM_015901526.1/1-3828	Monca.augustus.g52497.t1/1-4140	0.0721303	0.0766929	Bifunctional glutamate proline--tRNA	5	[illegible]
Acrdi.XM_015920946.1/1-525	Monca.augustus.g42003.t1/1-498	0.0337427	0.0766858	RNA-binding domain-containing	2	[illegible]
Acrdi.XM_015916360.1/1-2331	Monca.adi2mcaRNA27154_R0/1-2316	0.100997	0.0766603	general vesicular transport factor p115 isoform X3	10	[illegible]
Acrdi.XM_015916625.1/1-756	Monca.adi2mcaRNA2772_R1/1-639	0.144763	0.0766475	GPN-loop GTPase 2		
Acrdi.XM_015914056.1/1-765	Monca.augustus.g13783.t1/1-762	0.965424	0.0766465	THAP domain-containing 11		
Acrdi.XM_015912358.1/1-840	Monca.augustus.g3323.t1/1-2319	0.279468	0.0766421	PREDICTED: uncharacterized protein LOC107346551		
Acrdi.XM_015917697.1/1-1086	Monca.augustus.g42281.t1/1-1293	0.104144	0.0766223	FGFR1 oncogene partner	2	[illegible]
Acrdi.XM_015897168.1/1-1140	Monca.adi2mcaRNA6126_R0/1-990	0.0601601	0.076616	obg-like ATPase 1	6	[illegible]
Acrdi.XM_015922012.1/1-1401	Monca.augustus.g38457.t1/1-1380	0.100759	0.0765478	N-chimaerin isoform X4	4	[illegible]
Acrdi.XM_015917517.1/1-741	Monca.augustus.g56372.t1/1-705	0.122716	0.0765149	Triosephosphate isomerase	7	[illegible]
Acrdi.XM_015924641.1/1-627	Monca.augustus.g26476.t1/1-621	0.0748486	0.0765075	NADPH-dependent FMN reductase	2	[illegible]
Acrdi.XM_015906973.1/1-462	Monca.augustus.g16778.t1/1-450	0.0773415	0.0764324	dCTP pyrophosphatase 1	1	[illegible]
Acrdi.XM_015914375.1/1-1440	Monca.augustus.g60723.t1/1-1428	0.106582	0.0764243	C9orf72 homolog		
Acrdi.XM_015921222.1/1-2655	Monca.augustus.g67792.t1/1-2715	0.0727464	0.0764165	E3 ubiquitin- ligase Bre1		
Acrdi.XM_015906910.1/1-708	Monca.augustus.g8906.t1/1-708	0.07894	0.0764034	glutathione S-transferase U20-like	3	[illegible]
Acrdi.XM_015913955.1/1-912	Monca.augustus.g20014.t1/1-912	0.0776267	0.0764023	serine threonine- kinase mos-like	6	[illegible]
Acrdi.XM_015900885.1/1-1329	Monca.augustus.g27839.t1/1-1359	0.0840044	0.0763948	transmembrane prolyl 4-hydroxylase-like	2	[illegible]
Acrdi.XM_015918641.1/1-1821	Monca.augustus.g33669.t1/1-1827	0.0774606	0.0763569	kelch 12	3	[illegible]
Acrdi.XM_015906335.1/1-2778	Monca.augustus.g45333.t1/1-2778	0.0946356	0.0763456	rap guanine nucleotide exchange factor 1-like	4	[illegible]
Acrdi.XM_015898987.1/1-378	Monca.adi2mcaRNA825_R0/1-366	0.130873	0.0763379	vesicle-associated membrane 4 isoform X2	5	[illegible]
Acrdi.XM_015912617.1/1-456	Monca.augustus.g12812.t1/1-453	0.098449	0.076336	PREDICTED: uncharacterized protein LOC107346789		
Acrdi.XM_015924395.1/1-762	Monca.augustus.g3046.t1/1-792	0.0660746	0.0763	adenylate kinase mitochondrial isoform X2	8	[illegible]
Acrdi.XM_015914611.1/1-786	Monca.augustus.g57117.t1/1-783	0.0920402	0.076239	33 kDa inner dynein arm light axonemal	2	[illegible]
Acrdi.XM_015905505.1/1-2436	Monca.augustus.g28276.t1/1-2367	0.0858689	0.0761684	creatine flagellar-like	5	[illegible]
Acrdi.XM_015908344.1/1-1275	Monca.adi2mcaRNA1815_R0/1-1257	0.0819771	0.0761518	thioredoxin reductase 3	8	[illegible]
Acrdi.XM_015899199.1/1-1410	Monca.augustus.g49628.t1/1-1527	0.123999	0.0761314	Fatty-acid amide hydrolase 2	1	[illegible]
Acrdi.XM_015896849.1/1-1515	Monca.adi2mcaRNA578_R0/1-1503	0.121021	0.0760979	Transmembrane 9 superfamily member 2	1	[illegible]
Acrdi.XM_015909337.1/1-348	Monca.augustus.g40404.t1/1-576	0.127933	0.0760964	Histone deacetylase complex subunit		
Acrdi.XM_015921425.1/1-1320	Monca.augustus.g61101.t1/1-1374	0.0999807	0.0760325	phosphatidylinositol 4-kinase type 2-beta	2	[illegible]
Acrdi.XM_015924133.1/1-2145	Monca.augustus.g66162.t1/1-2163	0.0890472	0.0760301	Ski oncogene	3	[illegible]
Acrdi.XM_015900698.1/1-1023	Monca.adi2mcaRNA9946_R0/1-852	0.118278	0.0760278	cyclin-L1-like	6	[illegible]
Acrdi.XM_015920924.1/1-1089	Monca.augustus.g42006.t1/1-1101	0.103494	0.0760223	RNA-binding motif X chromosome	4	[illegible]
Acrdi.XM_015909728.1/1-1695	Monca.augustus.g2266.t1/1-1944	0.122741	0.0759992	nuclear intermediate filament	2	[illegible]
Acrdi.XM_015908729.1/1-417	Monca.augustus.g201.t1/1-423	0.0933661	0.0759978	Microsomal glutathione S-transferase 3	5	[illegible]
Acrdi.XM_015900484.1/1-696	Monca.augustus.g37063.t1/1-609	0.123577	0.0759645	phytanoyl- dioxygenase domain-containing 1		
Acrdi.XM_015907653.1/1-420	Monca.augustus.g49948.t1/1-522	0.094381	0.0759525	PREDICTED: uncharacterized protein LOC107342179		
Acrdi.XM_015901734.1/1-1065	Monca.augustus.g34405.t1/1-1020	0.0877268	0.0759363	doublesex- and mab-3-related transcription factor A2	6	[illegible]
Acrdi.XM_015911802.1/1-795	Monca.augustus.g71433.t1/1-1206	0.0881531	0.075923	coiled-coil domain-containing 105-like		
Acrdi.XM_015898658.1/1-2715	Monca.adi2mcaRNA7677_R0/1-2691	0.103755	0.0757972	Transforming growth factor-beta receptor-associated 1	5	[illegible]
Acrdi.XM_015905477.1/1-3258	Monca.augustus.g28248.t1/1-3294	0.0820971	0.0757744	constitutive coactivator of PPAR-gamma 1 homolog isoform X1	1	[illegible]
Acrdi.XM_015915726.1/1-1194	Monca.adi2mcaRNA26443_R0/1-1239	0.0700857	0.0757687	tRNA-dihydrouridine(20) synthase [NAD(P)+]-like	3	[illegible]
Acrdi.XM_015917880.1/1-447	Monca.augustus.g25188.t1/1-1365	0.0754113	0.0757016	synaptotagmin-2-like isoform X1		
Acrdi.XM_015893575.1/1-1101	Monca.adi2mcaRNA39678_R0/1-1230	0.0863028	0.0756977	U4 U6 small nuclear ribonucleo Prp4	6	[illegible]
Acrdi.XM_015916780.1/1-1881	Monca.augustus.g17206.t1/1-1875	0.126252	0.0756976	zinc finger 2-like	2	[illegible]
Acrdi.XM_015921309.1/1-957	Monca.augustus.g40036.t1/1-954	0.10301	0.075684	UPF0676 -like	1	[illegible]
Acrdi.XM_015923018.1/1-963	Monca.augustus.g17982.t1/1-1074	0.120198	0.075648	40S ribosomal SA	9	[illegible]
Acrdi.XM_015912298.1/1-1668	Monca.adi2mcaRNA22627_R0/1-1692	0.088276	0.0756377	UDP-glucose:glyco glucosyltransferase 1 isoform X1		
Acrdi.XM_015916201.1/1-1182	Monca.augustus.g43601.t1/1-1332	0.135712	0.0755623	phosphatidate cytidylyltransferase 2	4	[illegible]
Acrdi.XM_015910090.1/1-495	Monca.augustus.g38322.t1/1-1095	0.119636	0.0755356	heparan sulfate 2-O-sulfotransferase 1-like	2	[illegible]
Acrdi.XM_015914906.1/1-543	Monca.augustus.g62438.t1/1-924	0.094093	0.0754799	PREDICTED: uncharacterized protein LOC107348833	1	[illegible]
Acrdi.XM_015894488.1/1-2130	Monca.augustus.g33661.t1/1-2502	0.109585	0.0754518	Transcription initiation factor TFIID subunit 4	5	[illegible]
Acrdi.XM_015921575.1/1-513	Monca.augustus.g41679.t1/1-4860	0.0605059	0.0754493	collagen alpha-1(XI) chain-like	2	[illegible]
Acrdi.XM_015925174.1/1-5682	Monca.augustus.g35962.t1/1-6096	0.0933618	0.0754359	structural maintenance of chromosomes flexible hinge domain-contai	3	[illegible]
Acrdi.XM_015897100.1/1-1050	Monca.augustus.g6965.t1/1-1038	0.101291	0.0754281	Hypoxia-inducible factor 1-alpha inhibitor	3	[illegible]
Acrdi.XM_015908769.1/1-792	Monca.adi2mcaRNA18745_R0/1-654	0.0867839	0.0753995	ganglioside-induced differentiation-associated 2		
Acrdi.XM_015897141.1/1-2481	Monca.augustus.g10375.t1/1-2493	0.0992005	0.0753905	E3 ubiquitin- ligase HECTD3	5	[illegible]
Acrdi.XM_015908783.1/1-1107	Monca.adi2mcaRNA18782_R2/1-1032	0.277933	0.0753106	PREDICTED: uncharacterized protein LOC107343216	4	[illegible]
Acrdi.XM_015900782.1/1-1452	Monca.augustus.g17946.t1/1-1755	0.0937904	0.0753062	syntaxin-binding 1		
Acrdi.XM_015909947.1/1-1386	Monca.adi2mcaRNA20062_R0/1-1317	0.368528	0.0753005	THAP domain-containing 11		
Acrdi.XM_015898070.1/1-2283	Monca.augustus.g39432.t1/1-2316	0.104095	0.0752896	transcription factor RFX4 isoform X1	7	[illegible]
Acrdi.XM_015896562.1/1-1179	Monca.augustus.g62598.t1/1-1194	0.0647525	0.0752583	zinc finger OZF-like isoform X1	2	[illegible]
Acrdi.XM_015905899.1/1-1572	Monca.augustus.g58526.t1/1-1827	0.0914342	0.075256	polypeptide N-acetylgalactosaminyltransferase 13	5	[illegible]
Acrdi.XM_015895737.1/1-450	Monca.augustus.g60351.t1/1-450	0.105445	0.0752529	mitochondrial import receptor subunit TOM20 homolog	8	[illegible]
Acrdi.XM_015912717.1/1-582	Monca.augustus.g43037.t1/1-582	0.106513	0.0752413	RER1	8	[illegible]
Acrdi.XM_015921308.1/1-795	Monca.augustus.g40038.t1/1-786	0.0584057	0.075216	outer dense fiber 3B	3	[illegible]
Acrdi.XM_015895647.1/1-768	Monca.augustus.g35436.t1/1-765	0.117602	0.075205	Type III iodothyronine deiodinase	6	[illegible]
Acrdi.XM_015915658.1/1-519	Monca.augustus.g12153.t1/1-519	0.110914	0.0752045	BCL2 adenovirus E1B 19 kDa -interacting 3	1	[illegible]
Acrdi.XM_015923688.1/1-879	Monca.augustus.g34974.t1/1-867	0.121176	0.0751947	CAAX prenyl protease 2	4	[illegible]
Acrdi.XM_015900388.1/1-1293	Monca.augustus.g54209.t1/1-1293	0.086528	0.0751914	gamma-glutamyltranspeptidase 1-like	5	[illegible]
Acrdi.XM_015900780.1/1-2259	Monca.augustus.g11849.t1/1-2352	0.072824	0.0751888	ankyrin repeat and EF-hand domain-containing 1 isoform X1	1	[illegible]
Acrdi.XM_015920562.1/1-4911	Monca.augustus.g35482.t1/1-4968	0.10008	0.0751633	serine threonine- phosphatase 6 regulatory ankyrin repeat subunit B-li	1	[illegible]
Acrdi.XM_015914114.1/1-1092	Monca.augustus.g8026.t1/1-1083	0.0989248	0.0751467	Transcription factor AP-1	19	[illegible]
Acrdi.XM_015922366.1/1-1167	Monca.augustus.g51254.t1/1-1110	0.100451	0.0751426	PDZ domain-containing GIPC2	2	[illegible]
Acrdi.XM_015902622.1/1-2373	Monca.augustus.g54287.t1/1-2373	0.0772271	0.0751395	Double zinc ribbon and ankyrin repeat-containing 1	1	[illegible]
Acrdi.XM_015916417.1/1-669	Monca.augustus.g64486.t1/1-687	0.0915395	0.0751309	nascent polypeptide-associated complex subunit alpha	4	[illegible]
Acrdi.XM_015901503.1/1-1146	Monca.adi2mcaRNA10817_R2/1-1074	0.351225	0.0751236	G2 M phase-specific E3 ubiquitin- ligase-like		
Acrdi.XM_015910631.1/1-2739	Monca.augustus.g37495.t1/1-2898	0.0824243	0.0750771	vinculin isoform X5	4	[illegible]
Acrdi.XM_015910830.1/1-1395	Monca.augustus.g57307.t1/1-1218	0.066407	0.0750728	Nucleobindin-2	1	[illegible]
Acrdi.XM_015894059.1/1-1275	Monca.adi2mcaRNA40266_R0/1-1185	0.086623	0.07501	gamma-aminobutyric acid receptor subunit beta-like	4	[illegible]
Acrdi.XM_015921386.1/1-693	Monca.augustus.g4640.t1/1-702	0.0998873	0.0750079	GTP cyclohydrolase 1	29	[illegible]
Acrdi.XM_015901701.1/1-5118	Monca.augustus.g34404.t1/1-6108	0.107071	0.0750074	Phosphatidylinositol 4-kinase alpha	5	[illegible]
Acrdi.XM_015914524.1/1-2076	Monca.augustus.g29135.t1/1-2823	0.0702602	0.0749955	Alpha-aminoadipic semialdehyde mitochondrial	1	[illegible]
Acrdi.XM_015895881.1/1-1272	Monca.adi2mcaRNA4695_R0/1-1257	0.125399	0.0749917	AT-rich interactive domain-containing 3C	1	[illegible]
Acrdi.XM_015900378.1/1-624	Monca.augustus.g54199.t1/1-621	0.0284123	0.0749822	Heme-binding 2	6	[illegible]
Acrdi.XM_015915675.1/1-1083	Monca.adi2mcaRNA26401_R0/1-1026	0.0753533	0.0749745	choline ethanolaminephosphotransferase 1 isoform X1	5	[illegible]
Acrdi.XM_015901935.1/1-435	Monca.augustus.g41295.t1/1-528	0.580027	0.074966	PREDICTED: uncharacterized protein LOC107336860	2	[illegible]
Acrdi.XM_015915648.1/1-765	Monca.augustus.g12774.t1/1-765	0.113878	0.0749579	Ribose-5-phosphate isomerase	5	[illegible]
Acrdi.XM_015917252.1/1-1050	Monca.augustus.g45133.t1/1-2226	0.0742283	0.0749547	centrosomal of 85 kDa	1	[illegible]
Acrdi.XM_015923367.1/1-1038	Monca.augustus.g24903.t1/1-1293	0.110774	0.0749539	probable acyl- dehydrogenase 6	7	[illegible]
Acrdi.XM_015916603.1/1-1068	Monca.adi2mcaRNA27434_R0/1-1092	0.134769	0.0749368	LCHN		
Acrdi.XM_015898681.1/1-597	Monca.augustus.g54241.t1/1-624	0.0935001	0.0749251	ras-related Rab6	22	[illegible]
Acrdi.XM_015916149.1/1-906	Monca.augustus.g12399.t1/1-3639	0.0917961	0.0749118	AF4 FMR2 family member 3 isoform X6	10	[illegible]
Acrdi.XM_015895265.1/1-516	Monca.adi2mcaRNA41695_R6/1-408	0.517076	0.0748977	E3 ubiquitin- ligase TOM1-		
Acrdi.XM_015895586.1/1-432	Monca.adi2mcaRNA4413_R0/1-429	0.126001	0.07485	COMM domain-containing 5-like		
Acrdi.XM_015912164.1/1-1911	Monca.augustus.g55528.t1/1-2001	0.0868379	0.0748454	heterogeneous nuclear ribonucleo Q isoform X1	2	[illegible]
Acrdi.XM_015901869.1/1-828	Monca.augustus.g56981.t1/1-1125	0.0772975	0.0748375	alpha beta hydrolase domain-containing 13-like	1	[illegible]
Acrdi.XM_015903810.1/1-2520	Monca.augustus.g33835.t1/1-2646	0.0687199	0.074828	elongation factor 2	5	[illegible]
Acrdi.XM_015918355.1/1-5190	Monca.augustus.g41672.t1/1-5232	0.123631	0.0748257	voltage-dependent calcium channel type A subunit alpha-1 isoform X7		
Acrdi.XM_015924588.1/1-1209	Monca.augustus.g31437.t1/1-1206	0.105895	0.0748011	Photoreceptor-specific nuclear receptor	5	[illegible]
Acrdi.XM_015896914.1/1-8652	Monca.augustus.g29641.t1/1-7380	0.0838092	0.0747988	E3 ubiquitin- ligase HECTD1 isoform X4		
Acrdi.XM_015899346.1/1-543	Monca.augustus.g40300.t1/1-1539	0.0957508	0.0747888	Proton-coupled folate transporter	5	[illegible]
Acrdi.XM_015918373.1/1-762	Monca.adi2mcaRNA29427_R0/1-696	0.0770733	0.0747052	mitochondrial import receptor subunit TOM40 homolog	4	[illegible]
Acrdi.XM_015914454.1/1-1959	Monca.augustus.g56337.t1/1-1959	0.102543	0.0747038	rho-related BTB domain-containing 2-like isoform X1	3	[illegible]
Acrdi.XM_015915216.1/1-2385	Monca.augustus.g30652.t1/1-2400	0.0820775	0.0746917	vacuolar sorting-associated 51 homolog	8	[illegible]
Acrdi.XM_015909339.1/1-1680	Monca.augustus.g56273.t1/1-1692	0.10862	0.0746763	BRCA1-associated	12	[illegible]
Acrdi.XM_015916741.1/1-2313	Monca.augustus.g3867.t1/1-2439	0.107609	0.0746751	DNA replication licensing factor MCM6	7	[illegible]
Acrdi.XM_015918665.1/1-678	Monca.augustus.g7586.t1/1-678	0.154536	0.0746674	dickkopf-related 3-like	3	[illegible]
Acrdi.XM_015907461.1/1-759	Monca.augustus.g43833.t1/1-942	0.102897	0.0746663	NF-kappa-B-activating		
Acrdi.XM_015892602.1/1-1734	Monca.augustus.g36525.t1/1-2025	0.108515	0.0746113	heat shock 70	1	[illegible]
Acrdi.XM_015896400.1/1-1584	Monca.augustus.g1945.t1/1-2223	0.125588	0.0745934	Exocyst complex component 8	3	[illegible]
Acrdi.XM_015910313.1/1-876	Monca.augustus.g36452.t1/1-891	0.0900093	0.0745912	uricase-like	4	[illegible]
Acrdi.XM_015903393.1/1-918	Monca.augustus.g36028.t1/1-918	0.102394	0.0745608	Histamine N-methyltransferase	6	[illegible]
Acrdi.XM_015903637.1/1-4428	Monca.augustus.g70598.t1/1-5343	0.100724	0.0745386	bromodomain and WD repeat-containing 3-like	4	[illegible]
Acrdi.XM_015894706.1/1-597	Monca.augustus.g17178.t1/1-534	0.0500009	0.0745374	charged multivesicular body 2a	6	[illegible]
Acrdi.XM_015919442.1/1-960	Monca.augustus.g22485.t1/1-984	0.0861048	0.0745211	sphingolipid delta(4)-desaturase DES1-like	4	[illegible]
Acrdi.XM_015925190.1/1-1902	Monca.augustus.g62140.t1/1-1647	0.0711615	0.0745084	CXXC-type zinc finger 1-like	1	[illegible]
Acrdi.XM_015901957.1/1-1713	Monca.augustus.g45538.t1/1-1689	0.102417	0.0745048	malate synthase A	4	[illegible]
Acrdi.XM_015923171.1/1-1725	Monca.adi2mcaRNA3526_R0/1-1644	0.107222	0.0744804	VAC14 homolog	2	[illegible]
Acrdi.XM_015904500.1/1-360	Monca.augustus.g33593.t1/1-360	0.107855	0.0744584	U7 snRNA-associated Sm LSm10		
Acrdi.XM_015902454.1/1-2331	Monca.augustus.g42490.t1/1-2895	0.102084	0.0744493	nuclear pore complex Nup107 isoform X3	4	[illegible]
Acrdi.XM_015898651.1/1-1623	Monca.adi2mcaRNA7671_R0/1-1611	0.103523	0.0744446	membrane-associated tyrosine- and threonine-specific cdc2-inhibitory	3	[illegible]
Acrdi.XM_015896315.1/1-975	Monca.adi2mcaRNA5185_R0/1-930	0.602872	0.0744333	zinc finger MYM-type 2-like	4	[illegible]
Acrdi.XM_015908750.1/1-1560	Monca.augustus.g26271.t1/1-1620	0.0788634	0.0744307	synaptonemal complex 1-like	2	[illegible]
Acrdi.XM_015897659.1/1-1575	Monca.augustus.g50750.t1/1-1671	0.103918	0.0744057	PREDICTED: uncharacterized protein C9orf117 homolog	1	[illegible]
Acrdi.XM_015910321.1/1-681	Monca.augustus.g36425.t1/1-987	0.0905114	0.0743905	transcriptional adapter	1	[illegible]
Acrdi.XM_015914494.1/1-315	Monca.augustus.g60724.t1/1-315	0.0772695	0.0743879	DNA polymerase epsilon subunit 4	2	[illegible]
Acrdi.XM_015898970.1/1-882	Monca.adi2mcaRNA8001_R1/1-885	0.0674689	0.0743623	axin dorsalization-associated isoform X1		
Acrdi.XM_015896560.1/1-1305	Monca.augustus.g62602.t1/1-1299	0.0853002	0.0743502	Calcium-binding NCSA	2	[illegible]
Acrdi.XM_015906169.1/1-870	Monca.augustus.g21703.t1/1-993	0.134805	0.0743311	glycerophosphodiester phosphodiesterase domain-containing 1-like	2	[illegible]
Acrdi.XM_015924691.1/1-996	Monca.augustus.g70373.t1/1-993	0.0580158	0.0742939	melatonin receptor type 1B-like	4	[illegible]
Acrdi.XM_015902915.1/1-1164	Monca.augustus.g54833.t1/1-1479	0.0818523	0.0742916	4-hydroxybutyrate coenzyme A transferase-like	4	[illegible]
Acrdi.XM_015921573.1/1-741	Monca.augustus.g7120.t1/1-741	0.0844955	0.0742748	Carbonyl reductase [NADPH] 1		
Acrdi.XM_015899304.1/1-1011	Monca.augustus.g44777.t1/1-1248	0.0689727	0.0742581	UPF0183 C16orf70 homolog		
Acrdi.XM_015921182.1/1-1551	Monca.augustus.g25209.t1/1-1551	0.100689	0.0742221	D-3-phosphoglycerate dehydrogenase	4	[illegible]
Acrdi.XM_015895929.1/1-1374	Monca.augustus.g16936.t1/1-1377	0.107265	0.0742215	la-related 6-like	4	[illegible]
Acrdi.XM_015912029.1/1-414	Monca.augustus.g38034.t1/1-639	0.120098	0.0741934	Serine threonine tyrosine-interacting	4	[illegible]
Acrdi.XM_015925329.1/1-867	Monca.adi2mcaRNA37269_R4/1-795	0.654207	0.0741916	Retrovirus-related Pol poly from transposon opus		
Acrdi.XM_015894584.1/1-531	Monca.augustus.g41389.t1/1-4872	0.0965454	0.074191	centrosomal of 89 kDa isoform X2	1	[illegible]
Acrdi.XM_015893559.1/1-702	Monca.augustus.g57581.t1/1-648	0.135723	0.0741868	Down syndrome critical region 3 homolog	3	[illegible]
Acrdi.XM_015904684.1/1-963	Monca.augustus.g10299.t1/1-1029	0.43991	0.0741851	pyroglutamylated RFamide peptide receptor-like isoform X2	2	[illegible]
Acrdi.XM_015908928.1/1-1071	Monca.augustus.g31860.t1/1-1167	0.116058	0.0741725	leucine-rich repeat extensin 3		
Acrdi.XM_015901502.1/1-873	Monca.adi2mcaRNA10825_R0/1-960	1.28326	0.0741707	RNA-directed DNA polymerase from mobile element jockey-like		
Acrdi.XM_015894839.1/1-735	Monca.augustus.g55764.t1/1-1440	0.084502	0.0741568	E3 ubiquitin- ligase ARIH2	9	[illegible]
Acrdi.XM_015923021.1/1-1221	Monca.augustus.g60610.t1/1-1215	0.76242	0.0741429	G2 M phase-specific E3 ubiquitin- ligase-like		
Acrdi.XM_015916595.1/1-486	Monca.adi2mcaRNA27416_R0/1-1341	0.65725	0.0741382	ATP-dependent DNA helicase -like	4	[illegible]
Acrdi.XM_015914170.1/1-1707	Monca.augustus.g24978.t1/1-1707	0.0892566	0.0741182	ankyrin repeat and SOCS box 3	3	[illegible]
Acrdi.XM_015921169.1/1-873	Monca.adi2mcaRNA32507_R1/1-795	0.762842	0.0741139	nuclease HARBI1		
Acrdi.XM_015912776.1/1-2358	Monca.augustus.g58409.t1/1-4239	0.108445	0.074104	transmembrane 94-like	2	[illegible]
Acrdi.XM_015898341.1/1-2424	Monca.augustus.g54261.t1/1-2436	0.0917672	0.0740871	tyrosine- kinase HTK16-like	10	[illegible]
Acrdi.XM_015901246.1/1-1710	Monca.augustus.g45968.t1/1-1758	0.101619	0.0740862	sodium- and chloride-dependent betaine transporter	5	[illegible]
Acrdi.XM_015904223.1/1-1437	Monca.augustus.g19648.t1/1-1488	0.0791815	0.0740829	PR domain zinc finger 14-like	2	[illegible]
Acrdi.XM_015910478.1/1-675	Monca.augustus.g41052.t1/1-702	0.0956845	0.0740699	Ankyrin repeat domain-containing 45		
Acrdi.XM_015910095.1/1-13083	Monca.augustus.g62062.t1/1-17628	0.375212	0.0740169	E3 ubiquitin- ligase RNF213-like	5	[illegible]
Acrdi.XM_015897258.1/1-1356	Monca.adi2mcaRNA6179_R0/1-1272	0.0656111	0.0740169	vam6 Vps39	4	[illegible]
Acrdi.XM_015909909.1/1-1041	Monca.augustus.g28078.t1/1-1023	0.126138	0.0740051	ribose-phosphate pyrophosphokinase 4-like	3	[illegible]
Acrdi.XM_015909258.1/1-1116	Monca.augustus.g35318.t1/1-1053	0.0951523	0.0739951	Ubiquitin carboxyl-terminal hydrolase isozyme L5	12	[illegible]
Acrdi.XM_015896237.1/1-372	Monca.augustus.g54598.t1/1-537	0.0903091	0.0739639	Programmed cell death 6	4	[illegible]
Acrdi.XM_015921511.1/1-1638	Monca.augustus.g23004.t1/1-2121	0.0790963	0.0738941	ATP-dependent RNA helicase DHX33	6	[illegible]
Acrdi.XM_015897057.1/1-1278	Monca.augustus.g48650.t1/1-1272	0.100307	0.0738677	E3 ubiquitin- ligase RNF38 isoform X5	7	[illegible]
Acrdi.XM_015909929.1/1-4227	Monca.augustus.g28102.t1/1-6003	0.10184	0.0738633	Golgi-specific brefeldin A-resistance guanine nucleotide exchange fact	6	[illegible]
Acrdi.XM_015897363.1/1-1668	Monca.augustus.g29316.t1/1-2328	0.111297	0.0738509	Neurogenic locus notch 3		
Acrdi.XM_015900503.1/1-846	Monca.augustus.g63125.t1/1-1038	0.076898	0.0738473	SWI SNF-related matrix-associated actin-dependent regulator of chromatin subfamily E member 1 isoform X2		
Acrdi.XM_015922058.1/1-969	Monca.augustus.g64136.t1/1-1014	0.0963428	0.0738458	histamine H2 receptor-like	3	[illegible]
Acrdi.XM_015897048.1/1-1440	Monca.adi2mcaRNA6018_R0/1-1737	0.109301	0.0738342	DNA-directed RNA polymerase II subunit RPB1-like		
Acrdi.XM_015896934.1/1-1710	Monca.augustus.g28910.t1/1-1953	0.122226	0.0738157	cleavage stimulation factor subunit 2 isoform X2	3	[illegible]
Acrdi.XM_015900770.1/1-1488	Monca.augustus.g61571.t1/1-1434	0.070048	0.0737465	adenylate kinase 8-like	3	[illegible]
Acrdi.XM_015916730.1/1-1296	Monca.augustus.g37100.t1/1-2760	0.0981139	0.0737374	homogentisate 1,2-dioxygenase	4	[illegible]
Acrdi.XM_015910586.1/1-1143	Monca.augustus.g36894.t1/1-2643	0.0623203	0.0737354	alpha- sarcomeric isoform X1	1	[illegible]
Acrdi.XM_015916739.1/1-2037	Monca.augustus.g17205.t1/1-3351	0.0884429	0.0737316	probable ATP-dependent RNA helicase DDX46 isoform X2	6	[illegible]
Acrdi.XM_015915116.1/1-900	Monca.augustus.g39761.t1/1-924	0.0889502	0.0736595	fibroblast growth factor 20	2	[illegible]
Acrdi.XM_015923639.1/1-1524	Monca.augustus.g56412.t1/1-1428	0.468197	0.0736559	PREDICTED: uncharacterized protein LOC107356998		
Acrdi.XM_015893371.1/1-1149	Monca.augustus.g41432.t1/1-1287	0.0910141	0.0736191	E3 ubiquitin- ligase TRAF7-like	3	[illegible]
Acrdi.XM_015898421.1/1-891	Monca.augustus.g2152.t1/1-954	0.109651	0.0736145	ester hydrolase C11orf54 homolog	2	[illegible]
Acrdi.XM_015900598.1/1-3141	Monca.augustus.g55763.t1/1-3711	0.0984756	0.0735903	unconventional myosin-VI-like	1	[illegible]
Acrdi.XM_015914783.1/1-3498	Monca.augustus.g47146.t1/1-3471	0.0879993	0.0735778	adenylate cyclase type 9-like	6	[illegible]
Acrdi.XM_015910143.1/1-816	Monca.augustus.g43752.t1/1-855	0.540705	0.0735286	THAP domain-containing 1-like	1	[illegible]
Acrdi.XM_015894578.1/1-654	Monca.augustus.g29094.t1/1-1038	0.0802586	0.0735192	ATP-dependent RNA helicase DDX46		
Acrdi.XM_015899432.1/1-531	Monca.adi2mcaRNA1047_R0/1-684	0.0523502	0.0735074	mRNA export factor	6	[illegible]
Acrdi.XM_015904108.1/1-1968	Monca.adi2mcaRNA13580_R0/1-1965	0.0932244	0.0734983	acyl-coenzyme A oxidase peroxisomal	9	[illegible]
Acrdi.XM_015906545.1/1-1329	Monca.augustus.g38394.t1/1-1332	0.0927392	0.0734685	tenascin-like isoform X1	2	[illegible]
Acrdi.XM_015897577.1/1-978	Monca.augustus.g69373.t1/1-585	0.0881405	0.0734012	Replication A 32 kDa subunit	5	[illegible]
Acrdi.XM_015902186.1/1-327	Monca.augustus.g55031.t1/1-327	0.0982935	0.0733897	archease-like	2	[illegible]
Acrdi.XM_015903803.1/1-912	Monca.augustus.g6559.t1/1-912	0.113471	0.0733196	tubulin polyglutamylase complex subunit 2 isoform X1		
Acrdi.XM_015922812.1/1-939	Monca.augustus.g379.t1/1-1758	0.103599	0.0733166	centrosomal of 78 kDa isoform X1	1	[illegible]
Acrdi.XM_015905357.1/1-1632	Monca.augustus.g61127.t1/1-1818	0.10522	0.0732565	MAU2 chromatid cohesion factor homolog	4	[illegible]
Acrdi.XM_015908852.1/1-714	Monca.adi2mcaRNA18811_R5/1-711	0.725354	0.0732555	reverse transcriptase		
Acrdi.XM_015896500.1/1-1305	Monca.augustus.g60645.t1/1-2112	0.0752793	0.073202	leucine-rich repeat-containing 74A-like isoform X2	1	[illegible]
Acrdi.XM_015920627.1/1-630	Monca.augustus.g36306.t1/1-648	0.126571	0.073186	NEDD4 family-interacting 1-like	5	[illegible]
Acrdi.XM_015924953.1/1-360	Monca.augustus.g60401.t1/1-507	0.0599028	0.0731829	small nuclear ribonucleo Sm D1	26	[illegible]
Acrdi.XM_015915471.1/1-531	Monca.augustus.g53450.t1/1-537	0.0967877	0.0731236	RING finger and CHY zinc finger domain-containing 1	1	[illegible]
Acrdi.XM_015900647.1/1-1071	Monca.augustus.g28772.t1/1-1068	0.120961	0.0731214	dnaJ homolog subfamily B member 5 isoform X2	1	[illegible]
Acrdi.XM_015900371.1/1-324	Monca.augustus.g54182.t1/1-846	0.122125	0.0731097	-L-isoaspartate O-methyltransferase domain-containing 1	3	[illegible]
Acrdi.XM_015910536.1/1-4002	Monca.adi2mcaRNA20723_R0/1-3594	0.0658397	0.0730993	rho GTPase-activating 190 isoform X2	2	[illegible]
Acrdi.XM_015900178.1/1-2040	Monca.augustus.g59992.t1/1-1980	0.0940617	0.0730651	Serine threonine- kinase PLK1	4	[illegible]
Acrdi.XM_015899762.1/1-582	Monca.adi2mcaRNA8876_R0/1-597	0.299903	0.07305	PREDICTED: uncharacterized protein LOC107334792		
Acrdi.XM_015897315.1/1-2022	Monca.augustus.g41216.t1/1-2991	0.0743407	0.0730487	extended synaptotagmin-1-like	2	[illegible]
Acrdi.XM_015897917.1/1-1176	Monca.augustus.g3109.t1/1-1179	0.10082	0.0730467	T family of potassium channels 7	4	[illegible]
Acrdi.XM_015917801.1/1-1611	Monca.augustus.g11194.t1/1-2334	0.0981085	0.0729709	leucine-zipper-like transcriptional regulator 1	2	[illegible]
Acrdi.XM_015898079.1/1-420	Monca.augustus.g2721.t1/1-1392	0.0848277	0.0729661	PREDICTED: uncharacterized protein LOC107333292		
Acrdi.XM_015922110.1/1-426	Monca.augustus.g39755.t1/1-498	0.0973416	0.0729555	short coiled-coil B-like		
Acrdi.XM_015910990.1/1-795	Monca.augustus.g63040.t1/1-783	0.125765	0.0729537	Calmodulin	2	[illegible]
Acrdi.XM_015918555.1/1-435	Monca.augustus.g19962.t1/1-435	0.13944	0.0729435	cytochrome c oxidase subunit 4 isoform mitochondrial-like	3	[illegible]
Acrdi.XM_015915336.1/1-345	Monca.augustus.g41794.t1/1-345	0.0718076	0.0729396	PREDICTED: uncharacterized protein LOC107349194		
Acrdi.XM_015914309.1/1-2985	Monca.adi2mcaRNA24854_R2/1-2985	0.338379	0.0729364	OTU domain-containing 6B isoform X1		
Acrdi.XM_015902769.1/1-2529	Monca.augustus.g5683.t1/1-2535	0.0825272	0.0728842	striatin-3-like isoform X1		
Acrdi.XM_015921766.1/1-1836	Monca.augustus.g70553.t1/1-2100	0.105255	0.072823	LMBR1 domain-containing 2-like	1	[illegible]
Acrdi.XM_015895725.1/1-540	Monca.augustus.g35427.t1/1-1056	0.118041	0.0727966	NAK MPSK kinase	6	[illegible]
Acrdi.XM_015921477.1/1-2106	Monca.augustus.g58048.t1/1-2385	0.0888591	0.0727949	H(+) Cl(-) exchange transporter 7	1	[illegible]
Acrdi.XM_015915442.1/1-912	Monca.augustus.g53738.t1/1-1476	0.0981152	0.07279	Histone deacetylase 11	1	[illegible]
Acrdi.XM_015899474.1/1-1290	Monca.augustus.g37235.t1/1-1368	0.113036	0.0727615	Coronin-6	3	[illegible]
Acrdi.XM_015900908.1/1-786	Monca.augustus.g27867.t1/1-783	0.101794	0.0727394	cold inducible RNA binding isoform 3	2	[illegible]
Acrdi.XM_015912975.1/1-702	Monca.augustus.g56211.t1/1-702	0.101425	0.0727201	homeobox HMX3-B-like	6	[illegible]
Acrdi.XM_015912786.1/1-951	Monca.augustus.g68782.t1/1-960	0.118763	0.0727096	Glycerophosphodiester phosphodiesterase 1	3	[illegible]
Acrdi.XM_015918437.1/1-1290	Monca.adi2mcaRNA29473_R0/1-1290	0.0695343	0.0726965	SH2 domain-containing 4B		
Acrdi.XM_015896195.1/1-531	Monca.adi2mcaRNA5137_R2/1-714	0.0358658	0.0726923	PREDICTED: uncharacterized protein LOC107331588		
Acrdi.XM_015921510.1/1-2607	Monca.augustus.g22993.t1/1-2691	0.104148	0.0726735	E3 ubiquitin- ligase MARCH6	6	[illegible]
Acrdi.XM_015908357.1/1-843	Monca.augustus.g40414.t1/1-750	0.0826909	0.0726662	endoplasmic reticulum resident 29-like	3	[illegible]
Acrdi.XM_015905869.1/1-492	Monca.augustus.g32223.t1/1-654	0.0432234	0.0726529	39S ribosomal mitochondrial	1	[illegible]
Acrdi.XM_015901955.1/1-1839	Monca.augustus.g45530.t1/1-1704	0.112485	0.072623	apoptosis-inducing factor 3-like isoform X1	7	[illegible]
Acrdi.XM_015896599.1/1-1098	Monca.augustus.g1589.t1/1-1062	0.109278	0.0725969	disulfide isomerase	4	[illegible]
Acrdi.XM_015899400.1/1-852	Monca.augustus.g48264.t1/1-1155	0.0874816	0.0725919	UDP-glucose 4-epimerase isoform X2	1	[illegible]
Acrdi.XM_015916774.1/1-669	Monca.augustus.g62793.t1/1-801	0.134017	0.0725873	methyltransferase 6 isoform X2	1	[illegible]
Acrdi.XM_015906743.1/1-936	Monca.augustus.g3897.t1/1-1017	0.086621	0.0725795	Malate mitochondrial	4	[illegible]
Acrdi.XM_015922206.1/1-567	Monca.augustus.g21732.t1/1-564	0.0715965	0.0725687	density-regulated homolog	2	[illegible]
Acrdi.XM_015892418.1/1-2358	Monca.adi2mcaRNA38363_R0/1-2178	0.0603361	0.0725685	Transient receptor potential cation channel subfamily A member 1	3	[illegible]
Acrdi.XM_015896050.1/1-2286	Monca.augustus.g14822.t1/1-2814	0.0826156	0.0725223	Pre-mRNA-processing factor 6	3	[illegible]
Acrdi.XM_015923383.1/1-1500	Monca.augustus.g67822.t1/1-1545	0.102383	0.07246	radial spoke head 4 homolog A-like		
Acrdi.XM_015912320.1/1-12810	Monca.augustus.g28485.t1/1-13332	0.365121	0.0724354	sacsin-like		
Acrdi.XM_015917554.1/1-954	Monca.augustus.g44190.t1/1-510	0.0896866	0.0724352	HBS1 isoform X1	5	[illegible]
Acrdi.XM_015902807.1/1-939	Monca.augustus.g27231.t1/1-939	0.0694977	0.0724145	testis-specific serine threonine- kinase 3	6	[illegible]
Acrdi.XM_015909990.1/1-2565	Monca.augustus.g44402.t1/1-3741	0.089947	0.0723946	transient receptor potential cation channel subfamily A member 1 hon	7	[illegible]
Acrdi.XM_015908035.1/1-954	Monca.augustus.g20214.t1/1-561	0.091656	0.0723933	major facilitator superfamily domain-containing 8-like	1	[illegible]
Acrdi.XM_015915045.1/1-3057	Monca.augustus.g58142.t1/1-3957	0.0830523	0.0723542	Ankyrin repeat and FYVE domain-containing	1	[illegible]
Acrdi.XM_015913116.1/1-369	Monca.augustus.g25044.t1/1-375	0.0839706	0.0723488	PREDICTED: uncharacterized protein LOC107347219		
Acrdi.XM_015902289.1/1-1302	Monca.augustus.g35096.t1/1-1413	0.0648599	0.0723458	General transcription factor IIE subunit 1	2	[illegible]
Acrdi.XM_015896648.1/1-405	Monca.augustus.g42397.t1/1-1020	0.124539	0.07234	alternative mitochondrial-like	3	[illegible]

Acrdi.XM_015910120.1/1-1569	Monca.augustus.g38320.t1/1-1761	0.0765495	0.0723291	9-divinyl ether synthase	4	F:GO:0016705; F:GO:0046872; F:GO:0004497; P:GO:0016125	F:oxidoreductase activity, acting on paired donors, with incorporation or reduction of molecular oxygen; F:metal ion binding; F:monooxygenase activity; P:sterol metabolic process	
Acrdi.XM_015900551.1/1-1686	Monca.augustus.g26471.t1/1-1686	0.0996749	0.0723212	kelch-like ECH-associated 1	3	C:GO:0043229; P:GO:0050794; P:GO:0051603	C:intracellular organelle; P:regulation of cellular process; P:proteolysis involved in cellular protein catabolic process	
Acrdi.XM_015903406.1/1-1215	Monca.augustus.g35982.t1/1-1200	0.0891024	0.0723116	Insulinoma-associated 1	4	F:GO:0003677; P:GO:0035987; P:GO:0048732; P:GO:0031018	F:DNA binding; P:endodermal cell differentiation; P:gland development; P:endocrine pancreas development	
Acrdi.XM_015910225.1/1-615	Monca.augustus.g25493.t1/1-1530	0.0820393	0.0722989	probable S-acyltransferase 23	1	F:GO:0003824	F:catalytic activity	
Acrdi.XM_015895720.1/1-756	Monca.augustus.g64090.t1/1-756	0.0667828	0.0722987	polyglutamine-binding 1-like				
Acrdi.XM_015915582.1/1-669	Monca.adi2mcaRNA26284_R0/1-642	0.132005	0.0722929	leucine-rich repeat-containing 34-like isoform X1				
Acrdi.XM_015898069.1/1-1353	Monca.adi2mcaRNA7216_R0/1-972	0.0976936	0.0722916	serine threonine- phosphatase 5	5	C:GO:0005634; F:GO:0004721; C:GO:0005737; P:GO:0006470;	C:nucleus; F:phosphoprotein phosphatase activity; C:cytoplasm; P:protein dephosphorylation; F:hydrolase activity	
Acrdi.XM_015899427.1/1-1938	Monca.adi2mcaRNA8493_R0/1-1860	0.100269	0.0722694	Replication A 70 kDa DNA-binding subunit	4	F:GO:0003677; P:GO:0006281; C:GO:0031981; P:GO:0006310	F:DNA binding; P:DNA repair; C:nuclear lumen; P:DNA recombination	
Acrdi.XM_015914355.1/1-912	Monca.augustus.g54639.t1/1-531	2.79339	0.0722112	RNA-directed DNA polymerase from transposon BS				
Acrdi.XM_015917736.1/1-4335	Monca.augustus.g1558.t1/1-4344	0.0765924	0.0721991	ankyrin and armadillo repeat-containing	2	C:GO:0005634; C:GO:0005737	C:nucleus; C:cytoplasm	
Acrdi.XM_015904293.1/1-1359	Monca.augustus.g32332.t1/1-1329	0.0744139	0.0721924	inositol hexakisphosphate kinase 1 isoform X1	3	F:GO:0008440; P:GO:0016310; F:GO:0016301	F:inositol-1,4,5-trisphosphate 3-kinase activity; P:phosphorylation; F:kinase activity	
Acrdi.XM_015911060.1/1-1095	Monca.augustus.g65545.t1/1-2271	0.101212	0.0721876	N-alpha-acetyltransferase auxiliary subunit	7	C:GO:0005737; P:GO:0001756; P:GO:0043066; P:GO:0008284;	C:cytoplasm; P:somitogenesis; P:negative regulation of apoptotic process; P:positive regulation of cell proliferation; P:blood vessel morphogenesis; C:macromolecular complex; P:regulation of TOR signaling	
Acrdi.XM_015906173.1/1-1197	Monca.adi2mcaRNA15974_R0/1-1062	0.0966479	0.0721238	short-chain specific acyl- mitochondrial	9	F:GO:0003995; P:GO:0033539; F:GO:0000062; F:GO:0050660;	F:acyl-CoA dehydrogenase activity; P:fatty acid beta-oxidation using acyl-CoA dehydrogenase; F:fatty-acyl-CoA binding; F:flavin adenine dinucleotide binding; F:butyryl-CoA de	EC:1.3.99.3; EC:1.3.8.1
Acrdi.XM_015912174.1/1-996	Monca.augustus.g57805.t1/1-1440	0.0894731	0.0721184	centrosomal of 57 kDa-like	3	C:GO:0005813; P:GO:0000070; F:GO:0008017	C:centrosome; P:mitotic sister chromatid segregation; F:microtubule binding	
Acrdi.XM_015902891.1/1-1131	Monca.augustus.g27536.t1/1-1704	0.0891309	0.0720875	MAM and LDL-receptor class A domain-containing 2				
Acrdi.XM_015915980.1/1-2235	Monca.adi2mcaRNA26735_R0/1-2196	0.640037	0.0720722	Macrophage scavenger receptor types I and II				
Acrdi.XM_015894592.1/1-612	Monca.augustus.g70677.t1/1-624	0.0750536	0.0720644	Ras-related Rab-24	4	F:GO:0005525; P:GO:0007264; C:GO:0005739; C:GO:0005776	F:GTP binding; P:small GTPase mediated signal transduction; C:mitochondrion; C:autophagosome	
Acrdi.XM_015905334.1/1-1119	Monca.augustus.g32877.t1/1-1596	0.0808332	0.0720627	Clusterin-associated 1	4	C:GO:0005813; P:GO:0001822; C:GO:0005929; C:GO:0030992	C:centrosome; P:kidney development; C:cilium; C:intraciliary transport particle B	
Acrdi.XM_015907556.1/1-942	Monca.augustus.g28028.t1/1-1035	0.099541	0.0720613	WD repeat-containing 5				
Acrdi.XM_015925092.1/1-1134	Monca.augustus.g51122.t1/1-1257	0.0717179	0.0720398	N-acylglucosamine 2-epimerase	9	F:GO:0005524; C:GO:0070062; F:GO:0004866; F:GO:0042803;	F:ATP binding; C:extracellular exosome; F:endopeptidase inhibitor activity; F:protein homodimerization activity; P:N-acetylneuraminate catabolic process; F:N-acylglucosamin	EC:5.1.3.8
Acrdi.XM_015918843.1/1-1317	Monca.augustus.g53729.t1/1-1305	0.0757546	0.0720192	neuronal acetylcholine receptor subunit alpha-7 isoform X2	8	F:GO:0005216; P:GO:0007271; F:GO:0015464; P:GO:0007165;	F:ion channel activity; P:synaptic transmission, cholinergic; F:acetylcholine receptor activity; P:signal transduction; P:ion transmembrane transport; F:acetylcholine binding; C:acetylcholine-gated channel complex; C:sy [illegible]	
Acrdi.XM_015900357.1/1-501	Monca.augustus.g69136.t1/1-732	0.075137	0.0720098	regulator of G- signaling 17-like isoform X4	4	C:GO:0005737; F:GO:0005096; C:GO:0005886; P:GO:0043547	C:cytoplasm; F:GTPase activator activity; C:plasma membrane; P:positive regulation of GTPase activity	
Acrdi.XM_015920180.1/1-342	Monca.augustus.g50788.t1/1-393	0.083379	0.072004	meprin A subunit beta-like	1	F:GO:0016787	F:hydrolase activity	
Acrdi.XM_015897507.1/1-522	Monca.augustus.g32444.t1/1-486	0.105804	0.071994	ER membrane complex subunit 7	2	C:GO:0016021; F:GO:0030246	C:integral component of membrane; F:carbohydrate binding	
Acrdi.XM_015902209.1/1-1665	Monca.augustus.g34039.t1/1-1665	0.123129	0.071987	kelch 17	3	F:GO:0004842; C:GO:0031463; P:GO:0016567	F:ubiquitin-protein transferase activity; C:Cul3-RING ubiquitin ligase complex; P:protein ubiquitination	
Acrdi.XM_015911529.1/1-1200	Monca.augustus.g45441.t1/1-1194	0.0846357	0.0719158	T-box transcription factor TBX20	38	P:GO:0060045; P:GO:0035162; P:GO:0003193; F:GO:0000978;	P:positive regulation of cardiac muscle cell proliferation; P:embryonic hemopoiesis; P:pulmonary valve formation; F:RNA polymerase II core promoter proximal region sequence-specific DNA binding; P:endocardial cu [illegible]	
Acrdi.XM_015911068.1/1-477	Monca.augustus.g35661.t1/1-339	0.745575	0.0718608	PREDICTED: uncharacterized protein LOC107345366				
Acrdi.XM_015910548.1/1-1329	Monca.adi2mcaRNA20756_R1/1-1293	0.0721474	0.0718563	Serine threonine kinase-like domain-containing STKLD1	1	F:GO:0004672	F:protein kinase activity	
Acrdi.XM_015923145.1/1-717	Monca.adi2mcaRNA34780_R0/1-585	0.178316	0.0718518	Transposon TX1 uncharacterized 149 kDa				
Acrdi.XM_015897304.1/1-525	Monca.augustus.g67506.t1/1-714	0.087672	0.0718445	bax inhibitor 1 isoform X1	1	C:GO:0016021	C:integral component of membrane	
Acrdi.XM_015900032.1/1-1110	Monca.augustus.g43406.t1/1-1032	0.113138	0.0718193	PREDICTED: uncharacterized protein C9orf172-like				
Acrdi.XM_015914488.1/1-4863	Monca.adi2mcaRNA25078_R0/1-4287	0.0921899	0.0718106	Cytoskeleton-associated 5	2	C:GO:0015630; C:GO:0044430	C:microtubule cytoskeleton; C:cytoskeletal part	
Acrdi.XM_015918256.1/1-1779	Monca.augustus.g48563.t1/1-1785	0.0973051	0.0718033	Calnexin	6	P:GO:0048916; F:GO:0005509; C:GO:0016021; C:GO:0005783;	P:posterior lateral line development; F:calcium ion binding; C:integral component of membrane; C:endoplasmic reticulum; F:unfolded protein binding; P:protein folding	
Acrdi.XM_015897476.1/1-1122	Monca.augustus.g2233.t1/1-1806	0.0776525	0.0717978	Dolichyl-diphosphooligosaccharide-- glycosyltransferase subunit 1	3	C:GO:0008250; F:GO:0004579; P:GO:0018279	C:oligosaccharyltransferase complex; F:dolichyl-diphosphooligosaccharide-protein glycotransferase activity; P:protein N-linked glycosylation via asparagine	EC:2.4.1.119
Acrdi.XM_015922920.1/1-1869	Monca.adi2mcaRNA34499_R0/1-1689	0.58351	0.0717672	Calcium-responsive transcription factor				
Acrdi.XM_015915320.1/1-1086	Monca.augustus.g25287.t1/1-1668	0.0957781	0.0717445	WD repeat-containing 26-like				
Acrdi.XM_015894902.1/1-705	Monca.adi2mcaRNA41252_R4/1-522	0.551082	0.0717416	P2X purinoceptor 7-like				
Acrdi.XM_015914650.1/1-846	Monca.adi2mcaRNA25240_R0/1-912	0.379293	0.0717294	PREDICTED: uncharacterized protein LOC107348595				
Acrdi.XM_015918920.1/1-1635	Monca.augustus.g55595.t1/1-1467	0.0698571	0.0716995	nicotinate phosphoribosyltransferase-like	2	P:GO:0019363; F:GO:0016763	P:pyridine nucleotide biosynthetic process; F:transferase activity, transferring pentosyl groups	
Acrdi.XM_015924283.1/1-528	Monca.augustus.g39147.t1/1-600	0.106137	0.0716755	1-phosphatidylinositol 4,5-bisphosphate phosphodiesterase gamma-1	2	P:GO:0035556; C:GO:0005622	P:intracellular signal transduction; C:intracellular	
Acrdi.XM_015915140.1/1-7008	Monca.adi2mcaRNA839_R0/1-5880	0.461817	0.0716576	PREDICTED: uncharacterized protein LOC107349046, partial				
Acrdi.XM_015916522.1/1-723	Monca.augustus.g38974.t1/1-717	0.0866485	0.0716423	Transmembrane 127	2	C:GO:0016020; P:GO:0048523	C:membrane; P:negative regulation of cellular process	
Acrdi.XM_015924009.1/1-1980	Monca.augustus.g55721.t1/1-2793	0.104829	0.0716328	von Willebrand factor A domain-containing 3A				
Acrdi.XM_015901880.1/1-2160	Monca.augustus.g35902.t1/1-2163	0.0884992	0.0716208	exostosin-1-like	6	P:GO:0009101; F:GO:0015020; C:GO:0016021; C:GO:0005783;	P:glycoprotein biosynthetic process; F:glucuronosyltransferase activity; C:integral component of membrane; C:endoplasmic reticulum; P:single-organism cellular process; P:si	EC:2.4.1.17
Acrdi.XM_015922455.1/1-1599	Monca.augustus.g40390.t1/1-1653	0.0884574	0.0716045	Splicing proline- and glutamine-rich	5	F:GO:0000166; C:GO:0005634; F:GO:0000976; P:GO:0006355;	F:nucleotide binding; C:nucleus; F:transcription regulatory region sequence-specific DNA binding; P:regulation of transcription, DNA-templated; P:mRNA splicing, via spliceosome	
Acrdi.XM_015914742.1/1-5475	Monca.augustus.g13127.t1/1-5469	0.0872152	0.0716041	Sodium channel 60E	2	F:GO:0005216; P:GO:0006811	F:ion channel activity; P:ion transport	
Acrdi.XM_015912690.1/1-1188	Monca.augustus.g43023.t1/1-2628	0.151627	0.0715963	chloride channel 2 isoform X2	5	P:GO:1903959; P:GO:0034220; C:GO:0016021; P:GO:0006821;	P:regulation of anion transmembrane transport; P:ion transmembrane transport; C:integral component of membrane; P:chloride transport; F:voltage-gated chloride channel activity	
Acrdi.XM_015911896.1/1-3720	Monca.augustus.g38089.t1/1-4440	0.101637	0.0715859	peroxidasin homolog				
Acrdi.XM_015903419.1/1-891	Monca.augustus.g18168.t1/1-891	0.113507	0.0715798	MEMO1				
Acrdi.XM_015903955.1/1-534	Monca.augustus.g45682.t1/1-537	0.130633	0.0715687	apoptosis regulator R1-like	5	F:GO:0042803; F:GO:0046982; P:GO:0008630; P:GO:0097192;	F:protein homodimerization activity; F:protein heterodimerization activity; P:intrinsic apoptotic signaling pathway in response to DNA damage; P:extrinsic apoptotic signaling pathway in absence of ligand; C:mitocho [illegible]	
Acrdi.XM_015904508.1/1-1488	Monca.adi2mcaRNA14019_R0/1-1485	0.0972405	0.0715546	tripartite motif-containing 2-like isoform X5	2	F:GO:0046872; C:GO:0005622	F:metal ion binding; C:intracellular	
Acrdi.XM_015914590.1/1-1866	Monca.augustus.g57107.t1/1-2184	0.0712343	0.071541	solute carrier family 12 member 8	2	P:GO:0055085; C:GO:0016021	P:transmembrane transport; C:integral component of membrane	
Acrdi.XM_015914693.1/1-792	Monca.augustus.g61425.t1/1-570	1.08488	0.0715406	methionine-R-sulfoxide reductase B3				
Acrdi.XM_015900232.1/1-1737	Monca.augustus.g12823.t1/1-1731	0.0924647	0.0715241	actin-binding IPP	2	F:GO:0004842; C:GO:0031463	F:ubiquitin-protein transferase activity; C:Cul3-RING ubiquitin ligase complex	
Acrdi.XM_015893746.1/1-378	Monca.augustus.g33535.t1/1-549	0.0648712	0.0715222	Transcription factor ETV6	5	C:GO:0005634; P:GO:0030154; F:GO:0043565; F:GO:0000981;	C:nucleus; P:cell differentiation; F:sequence-specific DNA binding; F:RNA polymerase II transcription factor activity, sequence-specific DNA binding; P:regulation of transcription from RNA polymerase II promoter	
Acrdi.XM_015913203.1/1-2040	Monca.adi2mcaRNA23639_R0/1-2091	0.0699882	0.0715172	nucleolar 4-	3	F:GO:0005515; C:GO:0005737; C:GO:0005654	F:protein binding; C:cytoplasm; C:nucleoplasm	
Acrdi.XM_015911197.1/1-1218	Monca.augustus.g37501.t1/1-1221	0.0965622	0.0715059	Forkhead box N4	5	C:GO:0005634; P:GO:0006351; F:GO:0043565; F:GO:0000981;	C:nucleus; P:transcription, DNA-templated; F:sequence-specific DNA binding; F:RNA polymerase II transcription factor activity, sequence-specific DNA binding; P:regulation of transcription from RNA polymerase II pro [illegible]	
Acrdi.XM_015903359.1/1-690	Monca.augustus.g18165.t1/1-1521	0.104046	0.0715031	WD repeat-containing				
Acrdi.XM_015910582.1/1-2775	Monca.adi2mcaRNA20779_R0/1-2556	0.0756935	0.0714971	unc-45 homolog B				
Acrdi.XM_015920951.1/1-393	Monca.augustus.g53586.t1/1-396	0.0645777	0.0714949	EF-hand calcium-binding domain-containing 10-like	1	F:GO:0005509	F:calcium ion binding	
Acrdi.XM_015907227.1/1-2442	Monca.adi2mcaRNA17128_R1/1-2112	0.0874135	0.0714844	probable E3 ubiquitin- ligase HERC4 isoform X1	5	C:GO:0005634; F:GO:0004842; C:GO:0005737; F:GO:0016874;	C:nucleus; F:ubiquitin-protein transferase activity; C:cytoplasm; F:ligase activity; P:protein ubiquitination involved in ubiquitin-dependent protein catabolic process	
Acrdi.XM_015922731.1/1-3717	Monca.augustus.g15992.t1/1-3600	0.0775838	0.0714384	intraflagellar transport 140 homolog	21	C:GO:0032391; C:GO:0036064; P:GO:0031076; P:GO:0060041;	C:photoreceptor connecting cilium; C:ciliary basal body; P:embryonic camera-type eye development; P:retina development in camera-type eye; P:regulation of cilium assembly; P:embryonic cranial skeleton morphoge [illegible]	
Acrdi.XM_015902777.1/1-735	Monca.augustus.g33291.t1/1-735	0.0822385	0.0714199	tubulin-folding cofactor B-like				
Acrdi.XM_015892574.1/1-1263	Monca.augustus.g36526.t1/1-2487	0.0858348	0.0714131	O- case-like isoform X2	1	F:GO:0016798	F:hydrolase activity, acting on glycosyl bonds	
Acrdi.XM_015899064.1/1-1356	Monca.augustus.g43399.t1/1-1539	0.084444	0.0714092	cilia- and flagella-associated 53-like	1	P:GO:0003341	P:cilium movement	
Acrdi.XM_015893704.1/1-654	Monca.augustus.g36731.t1/1-651	0.0721471	0.0714006	Phosphatidylcholine transfer	4	C:GO:0005829; P:GO:0015914; F:GO:0031210; F:GO:0008525	C:cytosol; P:phospholipid transport; F:phosphatidylcholine binding; F:phosphatidylcholine transporter activity	
Acrdi.XM_015917597.1/1-1053	Monca.adi2mcaRNA28550_R0/1-1008	0.178843	0.071384	basic proline-rich -like				
Acrdi.XM_015898204.1/1-1212	Monca.augustus.g29233.t1/1-1809	0.0956698	0.0713383	Potassium voltage-gated channel subfamily F member 1	7	F:GO:0005249; P:GO:0071805; C:GO:0008076; P:GO:0035235;	F:voltage-gated potassium channel activity; P:potassium ion transmembrane transport; C:voltage-gated potassium channel complex; P:ionotropic glutamate receptor signaling pathway; F:ionotropic glutamate recep [illegible]	
Acrdi.XM_015921528.1/1-714	Monca.adi2mcaRNA32880_R0/1-720	0.104565	0.0713182	Wilms tumor 1-interacting	14	P:GO:0060271; C:GO:0036064; C:GO:0045179; P:GO:0007067;	P:cilium assembly; C:ciliary basal body; C:apical cortex; P:mitotic nuclear division; F:binding; C:pericentriolar material; P:establishment of mitotic spindle orientation; C:adherens junction; C:cell-cell junction; P:ovarian [illegible]	
Acrdi.XM_015924204.1/1-738	Monca.augustus.g480.t1/1-825	0.120362	0.0712925	Exosome complex component RRP43	13	P:GO:0034475; P:GO:0034473; P:GO:0043928; P:GO:0000467;	P:U4 snRNA 3'-end processing; P:U1 snRNA 3'-end processing; P:exonucleolytic nuclear-transcribed mRNA catabolic process involved in deadenylation-dependent decay; P:exonucleolytic trimming to generate mature [illegible]	
Acrdi.XM_015910865.1/1-783	Monca.augustus.g70036.t1/1-789	0.0731349	0.0712835	isopentenyl-diphosphate Delta-isomerase 1	7	F:GO:0000287; P:GO:0009240; C:GO:0005777; C:GO:0005739;	F:magnesium ion binding; P:isopentenyl diphosphate biosynthetic process; C:peroxisome; C:mitochondrion; F:isopentenyl-diphosphate delta-isomerase activity; F:manganese	EC:5.3.3.2
Acrdi.XM_015895480.1/1-969	Monca.adi2mcaRNA4350_R0/1-840	0.119483	0.0712833	collagen alpha-2(XI) chain isoform X1				
Acrdi.XM_015897206.1/1-2091	Monca.adi2mcaRNA6074_R1/1-2091	0.0930792	0.0712731	tyrosine- kinase SYK-like	21	P:GO:0022407; P:GO:0007169; P:GO:0038083; P:GO:0045785;	P:regulation of cell-cell adhesion; P:transmembrane receptor protein tyrosine kinase signaling pathway; P:peptidyl-tyrosine autophosphorylation; P:positive regulation of cell	EC:2.7.10; EC:2.7.10.2
Acrdi.XM_015902457.1/1-3204	Monca.augustus.g41955.t1/1-3783	0.102022	0.071249	WD repeat-containing 11-like	1	C:GO:0005765	C:lysosomal membrane	
Acrdi.XM_015907959.1/1-1368	Monca.augustus.g31004.t1/1-1374	0.0685713	0.0712167	probable NADH dehydrogenase	2	F:GO:0016491; P:GO:0055114	F:oxidoreductase activity; P:oxidation-reduction process	
Acrdi.XM_015901753.1/1-1434	Monca.augustus.g6405.t1/1-1434	0.094675	0.0711844	E3 ubiquitin- ligase MYLIP				
Acrdi.XM_015898682.1/1-1377	Monca.augustus.g2534.t1/1-1776	0.0804029	0.0711811	kinase C iota type	6	F:GO:0005524; F:GO:0046872; P:GO:0018105; C:GO:0005622;	F:ATP binding; F:metal ion binding; P:peptidyl-serine phosphorylation; C:intracellular; P:intracellular signal transduction; F:protein kinase C activity	EC:2.7.11; EC:2.7.11.13
Acrdi.XM_015919128.1/1-450	Monca.augustus.g35159.t1/1-450	0.0881915	0.0711545	PREDICTED: protein FAM72A-like				
Acrdi.XM_015892834.1/1-1992	Monca.augustus.g25504.t1/1-3627	0.089109	0.0711453	Sterol regulatory element-binding 1	3	C:GO:0016020; C:GO:0016021; F:GO:0046983	C:membrane; C:integral component of membrane; F:protein dimerization activity	
Acrdi.XM_015923708.1/1-789	Monca.augustus.g34977.t1/1-666	0.096963	0.0711189	Uridine diphosphate glucose pyrophosphatase	1	F:GO:0016787	F:hydrolase activity	
Acrdi.XM_015916382.1/1-1035	Monca.adi2mcaRNA27166_R0/1-1122	0.103341	0.0710723	heterogeneous nuclear ribonucleo A B isoform X1	9	P:GO:0000122; F:GO:0003700; F:GO:0003723; F:GO:0005515;	P:negative regulation of transcription from RNA polymerase II promoter; F:transcription factor activity, sequence-specific DNA binding; F:RNA binding; F:protein binding; C:RNA polymerase II transcription factor comp [illegible]	
Acrdi.XM_015923129.1/1-1341	Monca.augustus.g32468.t1/1-1533	0.070256	0.0710668	amine oxidase [flavin-containing] B	3	F:GO:0016491; C:GO:0016020; P:GO:0055114	F:oxidoreductase activity; C:membrane; P:oxidation-reduction process	
Acrdi.XM_015909914.1/1-933	Monca.augustus.g28080.t1/1-1938	0.112833	0.0710624	graves disease carrier -like	5	P:GO:0055085; F:GO:0003735; C:GO:0016021; C:GO:0005743;	P:transmembrane transport; F:structural constituent of ribosome; C:integral component of membrane; C:mitochondrial inner membrane; P:translation	
Acrdi.XM_015906583.1/1-1302	Monca.adi2mcaRNA16379_R1/1-1263	0.0845987	0.0710083	L-fuconate dehydratase	2	P:GO:0009063; F:GO:0003824	P:cellular amino acid catabolic process; F:catalytic activity	
Acrdi.XM_015915129.1/1-702	Monca.augustus.g26561.t1/1-636	0.0900418	0.0709432	leucine-rich repeat-containing C10orf11 homolog	1	P:GO:0030318	P:melanocyte differentiation	
Acrdi.XM_015918154.1/1-660	Monca.augustus.g51062.t1/1-750	0.275057	0.0709319	G2 M phase-specific E3 ubiquitin- ligase				
Acrdi.XM_015896997.1/1-1197	Monca.augustus.g28905.t1/1-1191	0.102696	0.0709278	coiled-coil domain-containing 83-like				
Acrdi.XM_015909489.1/1-2094	Monca.adi2mcaRNA19555_R1/1-1896	0.139486	0.0709177	epithelial splicing regulatory 1 isoform X1	2	F:GO:0000166; F:GO:0003676	F:nucleotide binding; F:nucleic acid binding	
Acrdi.XM_015909585.1/1-2031	Monca.adi2mcaRNA2070_R4/1-1914	0.352616	0.0708859	Retrovirus-related Pol poly from transposon	4	F:GO:0003676; F:GO:0008270; F:GO:0046872; P:GO:0015074	F:nucleic acid binding; F:zinc ion binding; F:metal ion binding; P:DNA integration	
Acrdi.XM_015910703.1/1-462	Monca.augustus.g35869.t1/1-462	0.213011	0.0708825	V-type proton ATPase 16 kDa proteolipid subunit	8	C:GO:0000220; P:GO:0060036; P:GO:0015991; P:GO:0001878;	C:vacuolar proton-transporting V-type ATPase, V0 domain; P:notochord cell vacuolization; P:ATP hydrolysis coupled proton transport; P:response to yeast; P:lysosomal lumen ac	EC:3.6.1.3; EC:3.6.1.15
Acrdi.XM_015915180.1/1-1896	Monca.augustus.g29748.t1/1-1890	0.106275	0.0708367	SERAC1 isoform X1				
Acrdi.XM_015901304.1/1-480	Monca.augustus.g43371.t1/1-483	0.0655697	0.0708322	probable kinetochore SPC25				
Acrdi.XM_015898701.1/1-1989	Monca.augustus.g54240.t1/1-2133	0.101614	0.0708044	long-chain-fatty-acid-- ligase 3	5	F:GO:0016877; P:GO:0006629; C:GO:0016021; C:GO:0043226;	F:ligase activity, forming carbon-sulfur bonds; P:lipid metabolic process; C:integral component of membrane; C:organelle; C:intracellular part	
Acrdi.XM_015922169.1/1-1320	Monca.augustus.g9761.t1/1-1320	0.0985279	0.0707375	alpha-(1,3)-fucosyltransferase 11-like	5	F:GO:0008417; C:GO:0032580; P:GO:0036065; C:GO:0016021;	F:fucosyltransferase activity; C:Golgi cisterna membrane; P:fucosylation; C:integral component of membrane; P:protein glycosylation	
Acrdi.XM_015922358.1/1-1257	Monca.augustus.g27418.t1/1-1590	0.0713627	0.0707299	excitatory amino acid transporter 1 isoform X4	19	P:GO:0055085; P:GO:0007399; F:GO:0015370; P:GO:0042221;	P:transmembrane transport; P:nervous system development; F:solute:sodium symporter activity; P:response to chemical; P:cellular amino acid biosynthetic process; C:cell projection; P:response to stress; F:L-glutamat [illegible]	
Acrdi.XM_015908161.1/1-354	Monca.augustus.g10445.t1/1-555	0.071678	0.0707129	desumoylating isopeptidase 2-like				
Acrdi.XM_015920253.1/1-810	Monca.augustus.g5243.t1/1-750	0.0759255	0.0706961	PREDICTED: uncharacterized protein LOC107353864	7	C:GO:0005615; C:GO:0016020; C:GO:0016021; P:GO:0048513;	C:extracellular space; C:membrane; C:integral component of membrane; P:animal organ development; F:receptor binding; P:intracellular signal transduction; C:intracellular	
Acrdi.XM_015905506.1/1-1380	Monca.augustus.g28279.t1/1-1380	0.118916	0.0706929	cyclopropane-fatty-acyl-phospholipid synthase	4	C:GO:0005737; C:GO:0016020; F:GO:0008757; P:GO:0032259	C:cytoplasm; C:membrane; F:S-adenosylmethionine-dependent methyltransferase activity; P:methylation	
Acrdi.XM_015909613.1/1-1107	Monca.adi2mcaRNA2075_R0/1-1143	0.107148	0.070663	Transforming growth factor beta-2				
Acrdi.XM_015916908.1/1-1194	Monca.adi2mcaRNA27776_R0/1-1182	0.616055	0.0706601	Retrovirus-related Pol poly from transposon	5	F:GO:0003676; F:GO:0008270; F:GO:0004190; P:GO:0006508;	F:nucleic acid binding; F:zinc ion binding; F:aspartic-type endopeptidase activity; P:proteolysis; P:DNA integration	
Acrdi.XM_015899761.1/1-1707	Monca.adi2mcaRNA8874_R0/1-1407	0.297283	0.0706566	ATP-dependent DNA helicase Q1				
Acrdi.XM_015909187.1/1-1422	Monca.augustus.g59264.t1/1-2208	0.290075	0.070644	Mediator of RNA polymerase II transcription subunit 15				
Acrdi.XM_015905066.1/1-927	Monca.adi2mcaRNA14688_R0/1-774	0.0714527	0.0706426	mitogen-activated kinase 14 isoform X2	38	P:GO:0060045; P:GO:0090336; P:GO:0032495; P:GO:0002062;	P:positive regulation of cardiac muscle cell proliferation; P:positive regulation of brown fat cell differentiation; P:response to muramyl dipeptide; P:chondrocyte differentiatio	EC:2.7.11; EC:2.7.11.24
Acrdi.XM_015908034.1/1-1143	Monca.augustus.g5037.t1/1-1146	0.0765278	0.0706321	methylmalonic aciduria type A mitochondrial	5	F:GO:0005525; P:GO:0019626; C:GO:0005759; F:GO:0003924;	F:GTP binding; P:short-chain fatty acid catabolic process; C:mitochondrial matrix; F:GTPase activity; P:cobalamin metabolic process	EC:3.6.1.15
Acrdi.XM_015923204.1/1-525	Monca.augustus.g56636.t1/1-498	0.636306	0.0706271	UPF0746 DDB_G0281095-like				
Acrdi.XM_015903169.1/1-3018	Monca.augustus.g52463.t1/1-4677	0.104141	0.0706129	kinesin unc-104	6	P:GO:1902582; C:GO:0015630; P:GO:0051650; C:GO:0044430;	P:single-organism intracellular transport; C:microtubule cytoskeleton; P:establishment of vesicle localization; C:cytoskeletal part; F:binding; P:microtubule-based movement	
Acrdi.XM_015908973.1/1-1230	Monca.augustus.g59483.t1/1-1524	0.085823	0.0705961	Catalase	9	C:GO:0005777; F:GO:0046872; F:GO:0004096; C:GO:0005739;	C:peroxisome; F:metal ion binding; F:catalase activity; C:mitochondrion; P:cellular oxidant detoxification; P:oxidation-reduction process; P:response to hydrogen peroxide; F:he	EC:1.11.1.7; EC:1.11.1.6
Acrdi.XM_015894054.1/1-468	Monca.augustus.g21539.t1/1-1485	0.111007	0.0705813	glycylpeptide N-tetradecanoyltransferase	3	C:GO:0005737; F:GO:0004379; P:GO:0018008	C:cytoplasm; F:glycylpeptide N-tetradecanoyltransferase activity; P:N-terminal peptidyl-glycine N-myristoylation	EC:2.3.1.97
Acrdi.XM_015901902.1/1-378	Monca.augustus.g17433.t1/1-372	0.0695979	0.0705575	60S ribosomal L30	4	F:GO:0003723; F:GO:0003735; C:GO:0022625; P:GO:0006412	F:RNA binding; F:structural constituent of ribosome; C:cytosolic large ribosomal subunit; P:translation	
Acrdi.XM_015895325.1/1-483	Monca.augustus.g53116.t1/1-483	0.0871772	0.0705565	probable ribosome biogenesis RLP24	5	F:GO:0003735; C:GO:0022625; P:GO:0000027; P:GO:1902626;	F:structural constituent of ribosome; C:cytosolic large ribosomal subunit; P:ribosomal large subunit assembly; P:assembly of large subunit precursor of preribosome; P:translation	
Acrdi.XM_015899399.1/1-864	Monca.augustus.g37246.t1/1-1065	0.105713	0.0705515	GATS 3	5	F:GO:0034618; P:GO:1904262; C:GO:0005829; P:GO:1903577;	F:arginine binding; P:negative regulation of TORC1 signaling; C:cytosol; P:cellular response to L-arginine; C:GATOR2 complex	
Acrdi.XM_015923076.1/1-2607	Monca.augustus.g70888.t1/1-2907	0.0862797	0.07054	UPF0505 C16orf62 homolog				
Acrdi.XM_015909186.1/1-441	Monca.augustus.g44608.t1/1-495	0.0749895	0.0705136	striated muscle	1	F:GO:0046872	F:metal ion binding	
Acrdi.XM_015918674.1/1-732	Monca.augustus.g7583.t1/1-729	0.0770528	0.0705015	PREDICTED: uncharacterized protein LOC107352337				
Acrdi.XM_015915725.1/1-624	Monca.augustus.g29693.t1/1-987	0.113497	0.0704694	RING finger 175 isoform X2	6	C:GO:0005789; C:GO:0061630; F:GO:0046872; P:GO:0030968;	C:endoplasmic reticulum membrane; F:ubiquitin protein ligase activity; F:metal ion binding; P:endoplasmic reticulum unfolded protein response; C:Golgi membrane; P:ER-associated ubiquitin-dependent protein catab [illegible]	
Acrdi.XM_015907793.1/1-1431	Monca.augustus.g55792.t1/1-1446	0.0725538	0.0704343	potassium voltage-gated channel subfamily D member 2-like	5	P:GO:0071805; C:GO:0008076; F:GO:0005251; P:GO:0034765;	P:potassium ion transmembrane transport; C:voltage-gated potassium channel complex; F:delayed rectifier potassium channel activity; P:regulation of ion transmembrane transport; P:protein homooligomerization	
Acrdi.XM_015924797.1/1-3438	Monca.augustus.g68163.t1/1-4056	0.0863549	0.0704236	ATP-dependent RNA helicase TDRD9	6	C:GO:0043229; F:GO:0097159; F:GO:1901363; F:GO:0043167;	C:intracellular organelle; F:organic cyclic compound binding; F:heterocyclic compound binding; F:ion binding; C:macromolecular complex; F:helicase activity	EC:3.6.1.15
Acrdi.XM_015898053.1/1-2583	Monca.augustus.g2722.t1/1-2835	0.070301	0.0704015	vacuolar sorting-associated 18 homolog	15	P:GO:0007040; P:GO:0035542; P:GO:0060036; P:GO:0007032;	P:lysosome organization; P:regulation of SNARE complex assembly; P:notochord cell vacuolization; P:endosome organization; P:endosome to pigment granule transport; P:endosome to lysosome transport; P:intracellul [illegible]	
Acrdi.XM_015908024.1/1-3816	Monca.augustus.g5045.t1/1-3813	0.0929049	0.0704014	Ankyrin repeat domain-containing 50	2	P:GO:1990126; C:GO:0005622	P:retrograde transport, endosome to plasma membrane; C:intracellular	
Acrdi.XM_015916236.1/1-1014	Monca.augustus.g18678.t1/1-1020	0.0734753	0.0703883	octopamine receptor-like isoform X1	5	F:GO:0001588; P:GO:0001963; P:GO:0007186; F:GO:0035240;	F:dopamine neurotransmitter receptor activity, coupled via Gs; P:synaptic transmission, dopaminergic; P:G-protein coupled receptor signaling pathway; F:dopamine binding; C:integral component of membrane	
Acrdi.XM_015921442.1/1-2964	Monca.adi2mcaRNA32803_R0/1-2592	0.103315	0.0703314	Sorting nexin-13	1	F:GO:0035091	F:phosphatidylinositol binding	
Acrdi.XM_015896488.1/1-1272	Monca.augustus.g22662.t1/1-1329	0.104262	0.0703302	doublesex- and mab-3-related transcription factor A2-like isoform X2	6	C:GO:0005634; F:GO:0003700; P:GO:0007548; F:GO:0042803;	C:nucleus; F:transcription factor activity, sequence-specific DNA binding; P:sex differentiation; F:protein homodimerization activity; P:regulation of transcription, DNA-templated; F:core promoter proximal region sequ [illegible]	
Acrdi.XM_015914283.1/1-585	Monca.augustus.g40754.t1/1-585	0.0849772	0.0703118	Receptor expression-enhancing 5	1	C:GO:0016021	C:integral component of membrane	
Acrdi.XM_015922804.1/1-1002	Monca.augustus.g6737.t1/1-1005	0.0995801	0.0703059	Eukaryotic translation initiation factor 2 subunit 2	5	F:GO:0003743; F:GO:0005515; P:GO:0044699; P:GO:0006412;	F:translation initiation factor activity; F:protein binding; P:single-organism process; P:translation; P:multicellular organismal process	
Acrdi.XM_015899705.1/1-1827	Monca.augustus.g13800.t1/1-1695	0.0868072	0.0702802	Asparagine--tRNA cytoplasmic				
Acrdi.XM_015896836.1/1-999	Monca.augustus.g27646.t1/1-1005	0.0832528	0.0702758	glyceraldehyde-3-phosphate dehydrogenase	29	C:GO:0015630; C:GO:0043209; P:GO:0007275; F:GO:0004365;	C:microtubule cytoskeleton; C:myelin sheath; P:multicellular organism development; F:glyceraldehyde-3-phosphate dehydrogenase (NAD+) (phosphorylating) activity; C:extra	EC:1.2.1.59; EC:1.2.1.12
Acrdi.XM_015910575.1/1-897	Monca.adi2mcaRNA20757_R0/1-903	0.0989453	0.0702354	Mechanosensory 2	1	C:GO:0005887	C:integral component of plasma membrane	
Acrdi.XM_015912752.1/1-1902	Monca.adi2mcaRNA23170_R0/1-1671	0.0806197	0.070235	FAD-dependent oxidoreductase domain-containing 2	4	C:GO:0005788; P:GO:0055114; P:GO:0030433; F:GO:0004497	C:endoplasmic reticulum lumen; P:oxidation-reduction process; P:ER-associated ubiquitin-dependent protein catabolic process; F:monooxygenase activity	
Acrdi.XM_015923562.1/1-906	Monca.augustus.g58095.t1/1-906	0.117831	0.0702236	Cyclin-dependent kinase 5 activator 1	3	P:GO:0071902; C:GO:0016533; F:GO:0016534	P:positive regulation of protein serine/threonine kinase activity; C:cyclin-dependent protein kinase 5 holoenzyme complex; F:cyclin-dependent protein kinase 5 activator activity	
Acrdi.XM_015919106.1/1-780	Monca.augustus.g67791.t1/1-1101	0.111437	0.0701756	serine racemase isoform X1	8	F:GO:0003941; F:GO:0030170; C:GO:0005737; P:GO:0006563;	F:L-serine ammonia-lyase activity; F:pyridoxal phosphate binding; C:cytoplasm; P:L-serine metabolic process; F:cation binding; P:D-serine biosynthetic process; F:serine racem	EC:4.3.1.17; EC:4.3.1.18; EC:5.1.1.10
Acrdi.XM_015913167.1/1-474	Monca.adi2mcaRNA23577_R0/1-432	0.0775362	0.0701591	Centrosomal POC5				
Acrdi.XM_015917688.1/1-864	Monca.augustus.g42297.t1/1-1230	0.112254	0.0701554	transcription initiation SPT3 homolog isoform X2	12	F:GO:0004402; P:GO:0043966; C:GO:0030914; C:GO:0033276;	F:histone acetyltransferase activity; P:histone H3 acetylation; C:STAGA complex; C:transcription factor TFTC complex; F:transcription coactivator activity; P:transcription, DNA-	EC:2.3.1.48
Acrdi.XM_015916443.1/1-1242	Monca.adi2mcaRNA27258_R0/1-1218	0.0931017	0.0701344	aspartyl aminopeptidase isoform X2	4	F:GO:0008270; P:GO:0006508; F:GO:0004177; F:GO:0008237	F:zinc ion binding; P:proteolysis; F:aminopeptidase activity; F:metallopeptidase activity	EC:3.4.11
Acrdi.XM_015891797.1/1-2682	Monca.augustus.g39214.t1/1-3135	0.0780619	0.0700923	phosphatidylinositol 4,5-bisphosphate 3-kinase catalytic subunit delta i	6	C:GO:0016020; C:GO:0044464; F:GO:0016773; F:GO:0035004;	C:membrane; C:cell part; F:phosphotransferase activity, alcohol group as acceptor; F:phosphatidylinositol 3-kinase activity; P:single-organism process; P:phosphate-containing compound metabolic process	
Acrdi.XM_015908399.1/1-3150	Monca.augustus.g62716.t1/1-2799	0.320915	0.0700886	ankyrin repeat	3	F:GO:0004842; P:GO:0015969; P:GO:0016567	F:ubiquitin-protein transferase activity; P:guanosine tetraphosphate metabolic process; P:protein ubiquitination	
Acrdi.XM_015908869.1/1-1902	Monca.augustus.g56251.t1/1-1902	0.0873969	0.0700818	sodium- and chloride-dependent GABA transporter 1-like	4	C:GO:0005887; P:GO:0006836; F:GO:0005328; P:GO:0055085	C:integral component of plasma membrane; P:neurotransmitter transport; F:neurotransmitter:sodium symporter activity; P:transmembrane transport	
Acrdi.XM_015914437.1/1-2094	Monca.augustus.g56322.t1/1-2103	0.0847147	0.0700425	FAM160B1	3	P:GO:0008150; C:GO:0005575; F:GO:0003674	P:biological_process; C:cellular_component; F:molecular_function	
Acrdi.XM_015896058.1/1-2106	Monca.adi2mcaRNA4933_R0/1-2007	0.0574516	0.0700116	argonaute-2 isoform X2	1	F:GO:0003676	F:nucleic acid binding	
Acrdi.XM_015921080.1/1-1173	Monca.augustus.g34991.t1/1-1446	0.120603	0.0699914	Proton-coupled folate transporter	5	P:GO:0055085; C:GO:0016020; C:GO:0016021; F:GO:0005542;	P:transmembrane transport; C:membrane; C:integral component of membrane; F:folic acid binding; P:transport	
Acrdi.XM_015912491.1/1-870	Monca.augustus.g15083.t1/1-1113	0.0824102	0.0699802	PRKCA-binding -like	3	F:GO:0019904; C:GO:0005737; P:GO:0034315	F:protein domain specific binding; C:cytoplasm; P:regulation of Arp2/3 complex-mediated actin nucleation	
Acrdi.XM_015912064.1/1-1122	Monca.augustus.g38028.t1/1-1122	0.0806153	0.069929	very-long-chain (3R)-3-hydroxyacyl- dehydratase	1	C:GO:0016020	C:membrane	
Acrdi.XM_015916288.1/1-1311	Monca.augustus.g68774.t1/1-1317	0.0875227	0.0699242	sodium potassium calcium exchanger 3-like	9	C:GO:0005887; F:GO:0030955; P:GO:0098662; F:GO:0005262;	C:integral component of plasma membrane; F:potassium ion binding; P:inorganic cation transmembrane transport; F:calcium channel activity; F:calcium ion binding; F:calcium, potassium:sodium antiporter activity; P [illegible]	
Acrdi.XM_015898631.1/1-1011	Monca.augustus.g54252.t1/1-1002	0.116918	0.0699094	Growth hormone-regulated TBC	7	P:GO:0031338; P:GO:0090630; F:GO:0005096; C:GO:0012505;	P:regulation of vesicle fusion; P:activation of GTPase activity; F:GTPase activator activity; C:endomembrane system; P:intracellular protein transport; F:Rab GTPase binding; C:intracellular	
Acrdi.XM_015899384.1/1-453	Monca.augustus.g71141.t1/1-759	0.131524	0.0699029	Derlin-1	2	C:GO:0005789; C:GO:0016021	C:endoplasmic reticulum membrane; C:integral component of membrane	
Acrdi.XM_015905003.1/1-321	Monca.augustus.g55861.t1/1-318	0.78025	0.0698628	PREDICTED: uncharacterized protein LOC107339700				
Acrdi.XM_015898021.1/1-1374	Monca.augustus.g28149.t1/1-1683	0.0857147	0.0698236	Myotubularin-related 9	1	F:GO:0005515	F:protein binding	
Acrdi.XM_015910047.1/1-702	Monca.augustus.g36889.t1/1-678	0.0829631	0.0698159	Dr1-associated corepressor	3	P:GO:0008150; C:GO:0005634; F:GO:0046982	P:biological_process; C:nucleus; F:protein heterodimerization activity	
Acrdi.XM_015911959.1/1-1713	Monca.augustus.g30052.t1/1-1716	0.0796394	0.0698026	potassium voltage-gated channel Shaw-like	5	P:GO:0071805; C:GO:0008076; F:GO:0005251; P:GO:0034765;	P:potassium ion transmembrane transport; C:voltage-gated potassium channel complex; F:delayed rectifier potassium channel activity; P:regulation of ion transmembrane transport; P:protein homooligomerization	
Acrdi.XM_015897237.1/1-1188	Monca.augustus.g31738.t1/1-1206	0.104376	0.069796	Transcription factor Sp5	3	F:GO:0003676; F:GO:0046872; P:GO:0048332	F:nucleic acid binding; F:metal ion binding; P:mesoderm morphogenesis	
Acrdi.XM_015895649.1/1-1365	Monca.augustus.g35453.t1/1-1824	0.0584575	0.0697584	kelch 20	6	F:GO:0004842; C:GO:0005827; P:GO:0032465; C:GO:0031463;	F:ubiquitin-protein transferase activity; C:polar microtubule; P:regulation of cytokinesis; C:Cul3-RING ubiquitin ligase complex; P:chromosome passenger complex localization to spindle midzone; P:protein ubiquitinati [illegible]	
Acrdi.XM_015910881.1/1-1761	Monca.augustus.g54298.t1/1-2133	0.382553	0.0697403	ATP-dependent DNA helicase PIF1				
Acrdi.XM_015898450.1/1-1233	Monca.augustus.g3319.t1/1-1371	0.123482	0.0697393	cation channel sperm-associated 2-like	6	F:GO:0022836; P:GO:0034220; F:GO:0005261; P:GO:0006816;	F:gated channel activity; P:ion transmembrane transport; F:cation channel activity; P:calcium ion transport; P:membrane depolarization during action potential; C:plasma membrane	
Acrdi.XM_015906069.1/1-969	Monca.augustus.g34453.t1/1-960	0.111788	0.0697354	neuropeptide FF receptor 2-like	4	C:GO:0005887; F:GO:0004930; P:GO:0007186; P:GO:0007166	C:integral component of plasma membrane; F:G-protein coupled receptor activity; P:G-protein coupled receptor signaling pathway; P:cell surface receptor signaling pathway	
Acrdi.XM_015908069.1/1-498	Monca.augustus.g36066.t1/1-498	0.11562	0.0697311	prolyl-tRNA synthetase associated domain-containing 1-like	2	P:GO:0006450; F:GO:0002161	P:regulation of translational fidelity; F:aminoacyl-tRNA editing activity	EC:3.1.1.1
Acrdi.XM_015919378.1/1-2631	Monca.augustus.g62421.t1/1-2889	0.0903381	0.0697118	polycystic kidney disease 2-like 1 isoform X1	5	P:GO:0050982; F:GO:0005262; F:GO:0005509; C:GO:0016021;	P:detection of mechanical stimulus; F:calcium channel activity; F:calcium ion binding; C:integral component of membrane; P:calcium ion transmembrane transport	
Acrdi.XM_015898968.1/1-1056	Monca.augustus.g16784.t1/1-1056	0.0845231	0.0697074	calcium calmodulin-dependent kinase type II subunit delta isoform X11	3	F:GO:0000166; F:GO:0004672; P:GO:0016310	F:nucleotide binding; F:protein kinase activity; P:phosphorylation	
Acrdi.XM_015906244.1/1-615	Monca.augustus.g21675.t1/1-615	0.122336	0.0697025	meiotic nuclear division 1 homolog	3	C:GO:0005634; P:GO:0051321; P:GO:0006310	C:nucleus; P:meiotic cell cycle; P:DNA recombination	
Acrdi.XM_015904210.1/1-429	Monca.augustus.g33409.t1/1-1410	0.0829308	0.0696862	thimet oligopeptidase	5	C:GO:0005758; F:GO:0046872; P:GO:0006518; P:GO:0006508;	C:mitochondrial intermembrane space; F:metal ion binding; P:peptide metabolic process; P:proteolysis; F:metalloendopeptidase activity	EC:3.4.24
Acrdi.XM_015907434.1/1-2169	Monca.adi2mcaRNA17297_R0/1-1791	0.853425	0.0696725	PREDICTED: uncharacterized protein LOC107341966				
Acrdi.XM_015908740.1/1-2124	Monca.augustus.g55164.t1/1-2121	0.0911942	0.0696673	WD and tetratricopeptide repeats 1 isoform X1				
Acrdi.XM_015918364.1/1-765	Monca.augustus.g2901.t1/1-765	0.542621	0.0696425	PREDICTED: uncharacterized protein LOC107352062				
Acrdi.XM_015892441.1/1-855	Monca.augustus.g32805.t1/1-774	0.0556797	0.069636	Beta-1,4-galactosyltransferase 7	6	P:GO:0005975; F:GO:0035250; P:GO:0009100; C:GO:0016020;	P:carbohydrate metabolic process; F:UDP-galactosyltransferase activity; P:glycoprotein metabolic process; C:membrane; P:macromolecule biosynthetic process; P:carbohydrate derivative biosynthetic process	
Acrdi.XM_015909885.1/1-2451	Monca.adi2mcaRNA1960_R0/1-2574	0.0893166	0.0696352	Rho GTPase-activating 7	2	P:GO:0007165; F:GO:0008289	P:signal transduction; F:lipid binding	
Acrdi.XM_015923630.1/1-1083	Monca.augustus.g56413.t1/1-1077	0.801597	0.0696158	PREDICTED: uncharacterized protein LOC107356994				
Acrdi.XM_015914778.1/1-996	Monca.adi2mcaRNA25394_R1/1-897	0.123256	0.0696046	N-acetylglucosamine-6-phosphate deacetylase	3	C:GO:0005634; P:GO:0006046; F:GO:0008448	C:nucleus; P:N-acetylglucosamine catabolic process; F:N-acetylglucosamine-6-phosphate deacetylase activity	EC:3.5.1.25
Acrdi.XM_015897187.1/1-1584	Monca.adi2mcaRNA6045_R1/1-1506	0.200412	0.069587	PREDICTED: uncharacterized protein LOC107332453				
Acrdi.XM_015916481.1/1-1305	Monca.augustus.g2853.t1/1-1305	0.0653184	0.0695798	succinyl- ligase [GDP-forming] subunit mitochondrial	4	F:GO:0005524; F:GO:0046872; P:GO:0008152; F:GO:0016874	F:ATP binding; F:metal ion binding; P:metabolic process; F:ligase activity	
Acrdi.XM_015899822.1/1-1611	Monca.augustus.g53242.t1/1-2853	0.114299	0.0695746	glycosyl hydrolase family 31	7	P:GO:0005975; F:GO:0003824; P:GO:0008152; F:GO:0004553;	P:carbohydrate metabolic process; F:catalytic activity; P:metabolic process; F:hydrolase activity, hydrolyzing O-glycosyl compounds; F:carbohydrate binding; F:hydrolase activity; F:hydrolase activity, acting on glycosyl b [illegible]	
Acrdi.XM_015904340.1/1-555	Monca.adi2mcaRNA13842_R0/1-558	0.110772	0.0695686	Peroxiredoxin- mitochondrial	2	F:GO:0016491; P:GO:0055114	F:oxidoreductase activity; P:oxidation-reduction process	
Acrdi.XM_015921543.1/1-1218	Monca.augustus.g65975.t1/1-1281	0.0636748	0.0695445	Dipeptidase 1	4	F:GO:0046872; C:GO:0031224; F:GO:0008238; F:GO:0008237	F:metal ion binding; C:intrinsic component of membrane; F:exopeptidase activity; F:metallopeptidase activity	
Acrdi.XM_015897653.1/1-783	Monca.augustus.g56099.t1/1-1215	0.105068	0.0695219	dual specificity mitogen-activated kinase kinase 7-like	4	F:GO:0005524; F:GO:0004702; C:GO:0005737; P:GO:0023014	F:ATP binding; F:signal transducer, downstream of receptor, with serine/threonine kinase activity; C:cytoplasm; P:signal transduction by protein phosphorylation	EC:2.7.11
Acrdi.XM_015916716.1/1-942	Monca.augustus.g37095.t1/1-945	0.0871181	0.0695146	FAM76A isoform X2	1	F:GO:0070011	F:peptidase activity, acting on L-amino acid peptides	
Acrdi.XM_015922810.1/1-1509	Monca.augustus.g380.t1/1-1506	0.0849627	0.0695121	potassium voltage-gated channel Shaw-like	5	P:GO:0071805; C:GO:0008076; F:GO:0005251; P:GO:0034765;	P:potassium ion transmembrane transport; C:voltage-gated potassium channel complex; F:delayed rectifier potassium channel activity; P:regulation of ion transmembrane transport; P:protein homooligomerization	
Acrdi.XM_015903487.1/1-735	Monca.adi2mcaRNA1448_R2/1-561	0.830666	0.0694606	Transposon Tf2-6 poly				
Acrdi.XM_015908396.1/1-5238	Monca.adi2mcaRNA18329_R0/1-5178	0.0883055	0.0694327	pecanex 1	2	C:GO:0016020; C:GO:0016021	C:membrane; C:integral component of membrane	
Acrdi.XM_015912198.1/1-1104	Monca.augustus.g19808.t1/1-1074	0.111245	0.0694201	leucine-rich repeat-containing 58-like				
Acrdi.XM_015909926.1/1-726	Monca.augustus.g63655.t1/1-729	0.077135	0.069409	Ras-related Rab-33	6	F:GO:0005525; F:GO:0004767; P:GO:0007264; P:GO:2000785;	F:GTP binding; F:sphingomyelin phosphodiesterase activity; P:small GTPase mediated signal transduction; P:regulation of autophagosome assembly; C:intracellular; C:plasma	EC:3.1.4.12
Acrdi.XM_015911045.1/1-2697	Monca.adi2mcaRNA21269_R0/1-2355	0.0828664	0.0693878	lon protease mitochondrial	9	F:GO:0000166; F:GO:0070361; P:GO:0006515; C:GO:0005759;	F:nucleotide binding; F:mitochondrial light strand promoter anti-sense binding; P:misfolded or incompletely synthesized protein catabolic process; C:mitochondrial matrix; P:	EC:3.6.1.3; EC:3.6.1.15
Acrdi.XM_015918257.1/1-1683	Monca.adi2mcaRNA29288_R0/1-1674	0.0988723	0.0693525	alpha-1,3-mannosyl-glyco 4-beta-N-acetylglucosaminyltransferase B	4	P:GO:0005975; P:GO:0006491; C:GO:0016020; F:GO:0008454	P:carbohydrate metabolic process; P:N-glycan processing; C:membrane; F:alpha-1,3-mannosylglycoprotein 4-beta-N-acetylglucosaminyltransferase activity	EC:2.4.1.145
Acrdi.XM_015895890.1/1-1632	Monca.augustus.g58271.t1/1-2817	0.0775033	0.0693471	sodium calcium exchanger 2-like isoform X1	3	P:GO:0055085; C:GO:0016020; P:GO:0030001	P:transmembrane transport; C:membrane; P:metal ion transport	
Acrdi.XM_015899139.1/1-642	Monca.augustus.g68067.t1/1-1119	0.478653	0.0693424	PREDICTED: uncharacterized protein LOC107334212				
Acrdi.XM_015922559.1/1-1977	Monca.augustus.g25381.t1/1-1068	0.0735084	0.0693371	Eukaryotic translation initiation factor 3 subunit B	5	F:GO:0000166; F:GO:0003743; F:GO:0031369; P:GO:0006413;	F:nucleotide binding; F:translation initiation factor activity; F:translation initiation factor binding; P:translational initiation; C:eukaryotic translation initiation factor 3 complex	
Acrdi.XM_015916586.1/1-1167	Monca.adi2mcaRNA27396_R0/1-885	0.525987	0.069325	PREDICTED: uncharacterized protein LOC107350363				
Acrdi.XM_015923915.1/1-693	Monca.adi2mcaRNA35638_R3/1-600	0.438925	0.0693128	Zinc metallo ase nas-39	3	F:GO:0003677; P:GO:0015074; P:GO:0006310	F:DNA binding; P:DNA integration; P:DNA recombination	
Acrdi.XM_015901204.1/1-645	Monca.augustus.g58575.t1/1-645	0.083299	0.0692919	PREDICTED: uncharacterized protein C17orf105 homolog	11	C:GO:0005634; F:GO:0004842; C:GO:0005615; F:GO:0008270;	C:nucleus; F:ubiquitin-protein transferase activity; C:extracellular space; F:zinc ion binding; C:cytoplasm; F:metallocarboxypeptidase activity; F:calcium ion binding; P:peptide	EC:3.4.21; EC:3.4.17; EC:3.4.16
Acrdi.XM_015908984.1/1-648	Monca.augustus.g59495.t1/1-339	0.118653	0.069283	O-acetyl-ADP-ribose deacetylase 1				
Acrdi.XM_015904414.1/1-546	Monca.augustus.g48067.t1/1-546	0.0808864	0.0692415	PREDICTED: uncharacterized protein LOC107339167				
Acrdi.XM_015900014.1/1-1026	Monca.augustus.g51845.t1/1-924	0.0565225	0.0692414	phospholipid phosphatase 3-like	1	P:GO:0009987	P:cellular process	
Acrdi.XM_015924322.1/1-390	Monca.augustus.g2869.t1/1-375	0.89226	0.0692054	PREDICTED: uncharacterized protein LOC107357688				
Acrdi.XM_015896280.1/1-1104	Monca.augustus.g13697.t1/1-903	0.0907587	0.0691771	neuropeptide Y receptor type 2-like	4	C:GO:0005887; F:GO:0004930; P:GO:0007186; P:GO:0007166	C:integral component of plasma membrane; F:G-protein coupled receptor activity; P:G-protein coupled receptor signaling pathway; P:cell surface receptor signaling pathway	
Acrdi.XM_015914762.1/1-2418	Monca.augustus.g47150.t1/1-3783	0.0862529	0.0691598	C-Jun-amino-terminal kinase-interacting	7	P:GO:0016192; C:GO:0005737; F:GO:0019894; P:GO:0007257;	P:vesicle-mediated transport; C:cytoplasm; F:kinesin binding; P:activation of JUN kinase activity; F:JUN kinase binding; F:MAP-kinase scaffold activity; F:receptor signaling complex scaffold activity	
Acrdi.XM_015904016.1/1-501	Monca.augustus.g27725.t1/1-495	0.0655053	0.0691519	UPF0687 C20orf27 homolog				
Acrdi.XM_015916710.1/1-4872	Monca.adi2mcaRNA27568_R0/1-4581	0.0856594	0.0691504	androglobin isoform X1				
Acrdi.XM_015897274.1/1-711	Monca.augustus.g48411.t1/1-708	0.0785909	0.0691487	MORN repeat-containing 3				
Acrdi.XM_015895359.1/1-309	Monca.augustus.g25980.t1/1-4308	0.0796862	0.069139	breast cancer type 1 susceptibility homolog	18	P:GO:0031060; P:GO:0035066; P:GO:0045717; P:GO:0035067;	P:regulation of histone methylation; P:positive regulation of histone acetylation; P:negative regulation of fatty acid biosynthetic process; P:negative regulation of histone acetylation; F:zinc ion binding; P:double-strand [illegible]	
Acrdi.XM_015914489.1/1-1155	Monca.augustus.g27288.t1/1-1428	0.102403	0.0691106	disrupted in renal carcinoma 2	2	P:GO:0055085; C:GO:0016021	P:transmembrane transport; C:integral component of membrane	
Acrdi.XM_015896024.1/1-804	Monca.adi2mcaRNA4853_R0/1-774	0.375499	0.0690989	trihelix transcription factor GT-3a-like				
Acrdi.XM_015901732.1/1-687	Monca.augustus.g34402.t1/1-687	0.0473091	0.0690845	radial spoke head 1 homolog				
Acrdi.XM_015910871.1/1-2154	Monca.adi2mcaRNA21085_R0/1-1980	0.0906067	0.0690841	ubiquitin-like modifier-activating enzyme 6	4	F:GO:0019780; C:GO:0005829; P:GO:0006511; P:GO:0032446	F:FAT10 activating enzyme activity; C:cytosol; P:ubiquitin-dependent protein catabolic process; P:protein modification by small protein conjugation	
Acrdi.XM_015918058.1/1-633	Monca.augustus.g39960.t1/1-633	0.0720755	0.0690479	Serine threonine- kinase 35				
Acrdi.XM_015893412.1/1-2895	Monca.augustus.g34503.t1/1-3372	0.0948395	0.0690478	Trafficking particle complex subunit 11	2	P:GO:0060041; P:GO:0001889	P:retina development in camera-type eye; P:liver development	
Acrdi.XM_015908108.1/1-1056	Monca.augustus.g33243.t1/1-1155	0.0941662	0.0690337	RNA-binding Nova-2	1	F:GO:0003723	F:RNA binding	
Acrdi.XM_015911340.1/1-2157	Monca.augustus.g35293.t1/1-2703	0.0685863	0.0689471	anoctamin-4-like isoform X1	2	C:GO:0016021; F:GO:0046983	C:integral component of membrane; F:protein dimerization activity	
Acrdi.XM_015900524.1/1-672	Monca.augustus.g49618.t1/1-672	0.12185	0.0689445	Bcl-2-related ovarian killer	6	F:GO:0042803; F:GO:0046982; P:GO:0008630; P:GO:0042981;	F:protein homodimerization activity; F:protein heterodimerization activity; P:intrinsic apoptotic signaling pathway in response to DNA damage; P:regulation of apoptotic process; P:extrinsic apoptotic signaling pathwa [illegible]	
Acrdi.XM_015892851.1/1-2463	Monca.augustus.g40309.t1/1-2415	0.365259	0.0689236	TBC domain-containing kinase	4	P:GO:0008152; C:GO:0016020; P:GO:0006629; C:GO:0016021	P:metabolic process; C:membrane; P:lipid metabolic process; C:integral component of membrane	
Acrdi.XM_015914648.1/1-528	Monca.augustus.g58314.t1/1-375	0.216041	0.0688871	nuclease HARBI1				
Acrdi.XM_015903599.1/1-1320	Monca.augustus.g53513.t1/1-1212	0.127693	0.068867	alanyl-tRNA editing Aarsd1	6	F:GO:0005524; F:GO:0004813; P:GO:0006450; P:GO:0006419;	F:ATP binding; F:alanine-tRNA ligase activity; P:regulation of translational fidelity; P:alanyl-tRNA aminoacylation; F:Ser-tRNA(Ala) hydrolase activity; F:tRNA binding	EC:6.1.1.7; EC:3.1.1.1
Acrdi.XM_015892651.1/1-342	Monca.augustus.g51181.t1/1-657	0.110364	0.0688629	nucleoside diphosphate kinase homolog 5	11	P:GO:0021591; P:GO:0006228; P:GO:0006241; P:GO:0007286;	P:ventricular system development; P:UTP biosynthetic process; P:CTP biosynthetic process; P:spermatid development; P:GTP biosynthetic process; P:negative regulation of ox	EC:2.7.4.6
Acrdi.XM_015907074.1/1-1452	Monca.augustus.g43268.t1/1-1449	0.0799894	0.068858	Thrombospondin-type laminin G domain and EAR repeat-containing				
Acrdi.XM_015895539.1/1-798	Monca.augustus.g26011.t1/1-1494	0.128147	0.0688446	aladin isoform X2	2	P:GO:0006913; C:GO:0005643	P:nucleocytoplasmic transport; C:nuclear pore	
Acrdi.XM_015913525.1/1-531	Monca.augustus.g31848.t1/1-2163	0.269783	0.0688233	PREDICTED: uncharacterized protein LOC107347563				
Acrdi.XM_015906105.1/1-1227	Monca.augustus.g34421.t1/1-1209	0.100487	0.0688193	CTD small phosphatase 2	2	F:GO:0004721; P:GO:0016311	F:phosphoprotein phosphatase activity; P:dephosphorylation	EC:3.1.3.16; EC:3.1.3.41
Acrdi.XM_015919465.1/1-1506	Monca.augustus.g25231.t1/1-1479	0.0654147	0.068815	5 -AMP-activated kinase subunit gamma-2	19	F:GO:0019901; F:GO:0016208; F:GO:0030295; P:GO:0006110;	F:protein kinase binding; F:AMP binding; F:protein kinase activator activity; P:regulation of glycolytic process; F:ADP binding; F:cAMP-dependent protein kinase regulator activ	EC:2.7.11
Acrdi.XM_015897514.1/1-369	Monca.augustus.g32462.t1/1-450	0.090055	0.0688078	60S ribosomal L31	6	C:GO:0070062; C:GO:0005925; F:GO:0003735; P:GO:0002181;	C:extracellular exosome; C:focal adhesion; F:structural constituent of ribosome; P:cytoplasmic translation; C:membrane; C:cytosolic large ribosomal subunit	
Acrdi.XM_015909984.1/1-1302	Monca.augustus.g7103.t1/1-1290	0.0921683	0.0687958	cytochrome c oxidase assembly COX15 homolog	9	F:GO:0004129; F:GO:0016627; C:GO:0016021; P:GO:1902600;	F:cytochrome-c oxidase activity; F:oxidoreductase activity, acting on the CH-CH group of donors; C:integral component of membrane; P:hydrogen ion transmembrane transpor	EC:1.9.3.1
Acrdi.XM_015895823.1/1-1644	Monca.augustus.g63633.t1/1-1647	0.0881584	0.0687899	potassium voltage-gated channel Shaw-like	5	P:GO:0071805; C:GO:0008076; F:GO:0005251; P:GO:0034765;	P:potassium ion transmembrane transport; C:voltage-gated potassium channel complex; F:delayed rectifier potassium channel activity; P:regulation of ion transmembrane transport; P:protein homooligomerization	
Acrdi.XM_015905386.1/1-1215	Monca.augustus.g43322.t1/1-1215	0.138676	0.0687786	betaine--homocysteine S-methyltransferase 1	6	P:GO:0009086; F:GO:0008270; C:GO:0005829; F:GO:0047150;	P:methionine biosynthetic process; F:zinc ion binding; C:cytosol; F:betaine-homocysteine S-methyltransferase activity; F:S-adenosylmethionine-homocysteine S-methyltransfe	EC:2.1.1.5
Acrdi.XM_015924131.1/1-612	Monca.augustus.g3452.t1/1-660	0.115113	0.0687557	Cytochrome b5 domain-containing 1				
Acrdi.XM_015898019.1/1-1914	Monca.augustus.g28151.t1/1-1917	0.0710623	0.0686868	fem-1 homolog B				
Acrdi.XM_015898449.1/1-735	Monca.adi2mcaRNA7495_R0/1-762	0.104542	0.0686629	ribosome biogenesis NSA2 homolog	4	P:GO:0000460; P:GO:0000470; C:GO:0005730; C:GO:0030687	P:maturation of 5.8S rRNA; P:maturation of LSU-rRNA; C:nucleolus; C:preribosome, large subunit precursor	
Acrdi.XM_015899131.1/1-693	Monca.augustus.g34554.t1/1-1326	0.0982418	0.0686002	CCR4-NOT transcription complex subunit 11	11	P:GO:0006417; P:GO:0008283; C:GO:0005634; F:GO:0005515;	P:regulation of translation; P:cell proliferation; C:nucleus; F:protein binding; P:DNA damage response, signal transduction by p53 class mediator resulting in cell cycle arrest; P:transcription, DNA-templated; C:cytosol; [illegible]	
Acrdi.XM_015909568.1/1-591	Monca.augustus.g33552.t1/1-594	0.0807291	0.0685989	Contactin-associated -like 2	1	C:GO:0016021	C:integral component of membrane	
Acrdi.XM_015923000.1/1-1443	Monca.augustus.g67829.t1/1-1443	0.0822995	0.0685958	vesicular inhibitory amino acid transporter-like	3	C:GO:0016021; F:GO:0015171; P:GO:0003333	C:integral component of membrane; F:amino acid transmembrane transporter activity; P:amino acid transmembrane transport	
Acrdi.XM_015908348.1/1-1566	Monca.augustus.g36813.t1/1-1935	0.084275	0.0685898	bifunctional 3 -phosphoadenosine 5 -phosphosulfate synthase-like isof	6	F:GO:0005524; P:GO:0050428; F:GO:0004020; F:GO:0004781;	F:ATP binding; P:3'-phosphoadenosine 5'-phosphosulfate biosynthetic process; F:adenylylsulfate kinase activity; F:sulfate adenylyltransferase (ATP) activity; P:phosphorylatio	EC:2.7.7.4; EC:2.7.1.25
Acrdi.XM_015905842.1/1-2874	Monca.adi2mcaRNA15538_R0/1-1359	0.097277	0.0685574	alpha-catulin-like isoform X1	1	F:GO:0005515	F:protein binding	
Acrdi.XM_015922870.1/1-588	Monca.augustus.g31198.t1/1-588	0.0912395	0.0685513	UPF0573 C2orf70 homolog A-like				
Acrdi.XM_015902607.1/1-822	Monca.augustus.g22803.t1/1-1059	0.103733	0.0685478	Zinc-binding alcohol dehydrogenase domain-containing 2	3	F:GO:0008270; F:GO:0016491; P:GO:0055114	F:zinc ion binding; F:oxidoreductase activity; P:oxidation-reduction process	
Acrdi.XM_015920666.1/1-1968	Monca.augustus.g14350.t1/1-1935	0.606197	0.0685384	hemicentin-2-like isoform X1				
Acrdi.XM_015912998.1/1-819	Monca.augustus.g7090.t1/1-792	0.0683348	0.0685313	Cell division cycle	3	P:GO:0051301; P:GO:0030071; C:GO:0005680	P:cell division; P:regulation of mitotic metaphase/anaphase transition; C:anaphase-promoting complex	
Acrdi.XM_015896939.1/1-921	Monca.augustus.g28904.t1/1-918	0.101916	0.0685294	GATA-type zinc finger 1	4	F:GO:0003700; F:GO:0043565; C:GO:0044424; P:GO:0009987	F:transcription factor activity, sequence-specific DNA binding; F:sequence-specific DNA binding; C:intracellular part; P:cellular process	
Acrdi.XM_015922294.1/1-909	Monca.augustus.g33112.t1/1-2229	0.377822	0.0685279	RNA-directed DNA polymerase from transposon BS				
Acrdi.XM_015896157.1/1-720	Monca.augustus.g55045.t1/1-720	0.0608463	0.0685132	elongation of very long chain fatty acids 5	3	C:GO:0016020; P:GO:0030497; F:GO:0016740	C:membrane; P:fatty acid elongation; F:transferase activity	
Acrdi.XM_015902694.1/1-2040	Monca.adi2mcaRNA12045_R0/1-1989	0.0916503	0.0684997	serine threonine- kinase BRSK2-like isoform X8	15	P:GO:0061178; P:GO:1904152; P:GO:0048812; C:GO:0005783;	P:regulation of insulin secretion involved in cellular response to glucose stimulus; P:regulation of retrograde protein transport, ER to cytosol; P:neuron projection morphogene	EC:2.7.11; EC:2.7.11.26
Acrdi.XM_015910817.1/1-2694	Monca.augustus.g70015.t1/1-2607	0.0773427	0.0684843	oxysterol-binding -related 8 isoform X1	2	C:GO:0016020; P:GO:0006810	C:membrane; P:transport	
Acrdi.XM_015921465.1/1-1794	Monca.augustus.g13243.t1/1-1920	0.178022	0.0684642	PREDICTED: uncharacterized protein LOC107354962				
Acrdi.XM_015901017.1/1-1602	Monca.augustus.g25257.t1/1-2421	0.0823407	0.0684631	calpain-7 isoform X1	3	C:GO:0005737; P:GO:0006508; F:GO:0004198	C:cytoplasm; P:proteolysis; F:calcium-dependent cysteine-type endopeptidase activity	EC:3.4.22
Acrdi.XM_015908833.1/1-954	Monca.augustus.g45273.t1/1-1395	0.11022	0.068449	glutaminase liver mitochondrial-like isoform X1	1	F:GO:0016787	F:hydrolase activity	
Acrdi.XM_015909700.1/1-711	Monca.augustus.g55274.t1/1-711	0.108827	0.0684485	PREDICTED: uncharacterized protein LOC107344093	5	F:GO:0003677; C:GO:0016020; C:GO:0016021; P:GO:0015074;	F:DNA binding; C:membrane; C:integral component of membrane; P:DNA integration; P:DNA recombination	
Acrdi.XM_015908445.1/1-1437	Monca.augustus.g33950.t1/1-1455	0.0900644	0.0684437	myocyte-specific enhancer factor 2A-like isoform X1	5	C:GO:0005634; F:GO:0003677; P:GO:0006351; P:GO:0006355;	C:nucleus; F:DNA binding; P:transcription, DNA-templated; P:regulation of transcription, DNA-templated; F:protein dimerization activity	
Acrdi.XM_015911429.1/1-1227	Monca.augustus.g69039.t1/1-1227	0.0775423	0.0684397	Glucose-fructose oxidoreductase domain-containing 1	2	F:GO:0016491; P:GO:0055114	F:oxidoreductase activity; P:oxidation-reduction process	
Acrdi.XM_015919705.1/1-1062	Monca.augustus.g40781.t1/1-1059	0.0876052	0.0684225	endophilin-A3-like isoform X2	2	C:GO:0043229; C:GO:0005737	C:intracellular organelle; C:cytoplasm	
Acrdi.XM_015908198.1/1-1029	Monca.adi2mcaRNA18158_R6/1-1029	0.108327	0.0684174	TPA_exp: poly				
Acrdi.XM_015916758.1/1-1446	Monca.augustus.g12285.t1/1-1446	0.074675	0.0683906	RNA polymerase-associated LEO1	5	P:GO:0032968; C:GO:0005634; P:GO:0044260; F:GO:1990269;	P:positive regulation of transcription elongation from RNA polymerase II promoter; C:nucleus; P:cellular macromolecule metabolic process; F:RNA polymerase II C-terminal domain phosphoserine binding; P:primary m [illegible]	
Acrdi.XM_015898124.1/1-720	Monca.augustus.g18980.t1/1-786	0.109288	0.0683871	phospholipid scramblase 1 isoform X1	2	C:GO:0005886; F:GO:0017128	C:plasma membrane; F:phospholipid scramblase activity	
Acrdi.XM_015908359.1/1-1452	Monca.augustus.g9816.t1/1-1452	0.0919895	0.0683315	PREDICTED: uncharacterized protein LOC107342848 isoform X1				
Acrdi.XM_015907908.1/1-2046	Monca.adi2mcaRNA17852_R0/1-1953	0.0861062	0.0683021	Spermatogenesis-associated 20	1	F:GO:0003824	F:catalytic activity	
Acrdi.XM_015898176.1/1-1050	Monca.augustus.g29257.t1/1-1047	0.0748204	0.0682672	Wnt-3a	66	P:GO:0042659; P:GO:2000081; P:GO:0061098; F:GO:0005110;	P:regulation of cell fate specification; P:positive regulation of canonical Wnt signaling pathway involved in controlling type B pancreatic cell proliferation; P:positive regulation of protein tyrosine kinase activity; F:frizz [illegible]	
Acrdi.XM_015920720.1/1-648	Monca.adi2mcaRNA32012_R1/1-531	0.411952	0.0682383	nuclease HARBI1				
Acrdi.XM_015904338.1/1-1347	Monca.augustus.g32335.t1/1-1353	0.0978263	0.0682056	Leucine-rich repeat-containing 74A	1	F:GO:0005509	F:calcium ion binding	
Acrdi.XM_015893903.1/1-1269	Monca.adi2mcaRNA40047_R0/1-1362	0.0895735	0.0682029	Ankyrin repeat domain-containing 42				
Acrdi.XM_015898312.1/1-855	Monca.augustus.g20926.t1/1-936	0.0979537	0.0682025	SPRY domain-containing SOCS box 3	2	C:GO:0005622; P:GO:0035556	C:intracellular; P:intracellular signal transduction	
Acrdi.XM_015921992.1/1-684	Monca.augustus.g8352.t1/1-645	0.0860474	0.0681844	Ubiquitin carboxyl-terminal hydrolase isozyme L3	5	F:GO:0004843; P:GO:0016579; P:GO:0007219; C:GO:0005737;	F:thiol-dependent ubiquitin-specific protease activity; P:protein deubiquitination; P:Notch signaling pathway; C:cytoplasm; P:ubiquitin-dependent protein catabolic process	EC:3.4.19.12
Acrdi.XM_015923632.1/1-1008	Monca.augustus.g3270.t1/1-1089	0.378779	0.0681762	TNF receptor-associated factor 5-like	4	F:GO:0004842; F:GO:0008270; F:GO:0046872; P:GO:0016567	F:ubiquitin-protein transferase activity; F:zinc ion binding; F:metal ion binding; P:protein ubiquitination	
Acrdi.XM_015900218.1/1-1065	Monca.augustus.g10471.t1/1-1149	0.0901296	0.0681659	gamma-aminobutyric acid receptor alpha-like	4	P:GO:0034220; C:GO:0016021; F:GO:0005230; C:GO:0005886	P:ion transmembrane transport; C:integral component of membrane; F:extracellular ligand-gated ion channel activity; C:plasma membrane	
Acrdi.XM_015919845.1/1-528	Monca.augustus.g66734.t1/1-528	0.069825	0.0681318	tctex1 domain-containing 1-B-like				
Acrdi.XM_015899337.1/1-1785	Monca.augustus.g64415.t1/1-1785	0.0823589	0.0681302	TAF5-like RNA polymerase II p300 CBP-associated factor-associated fa	1	C:GO:0005634	C:nucleus	
Acrdi.XM_015918338.1/1-897	Monca.adi2mcaRNA29392_R0/1-804	0.479495	0.0681279	PREDICTED: uncharacterized protein LOC107352036				
Acrdi.XM_015923316.1/1-384	Monca.augustus.g3702.t1/1-444	0.800251	0.0681277	PREDICTED: uncharacterized protein LOC107356693				
Acrdi.XM_015906389.1/1-1257	Monca.augustus.g41973.t1/1-1413	0.101105	0.0680907	Major facilitator superfamily domain-containing 10	5	C:GO:0031526; P:GO:0055085; F:GO:0008493; C:GO:0016021;	C:brush border membrane; P:transmembrane transport; F:tetracycline transporter activity; C:integral component of membrane; P:tetracycline transport	
Acrdi.XM_015911160.1/1-1551	Monca.augustus.g24017.t1/1-2064	0.0650766	0.0680731	Cleavage and polyadenylation specificity factor subunit 3				
Acrdi.XM_015892835.1/1-1383	Monca.augustus.g25503.t1/1-1611	0.0756299	0.0680369	tetratricopeptide repeat 29-like				
Acrdi.XM_015902072.1/1-2325	Monca.adi2mcaRNA11403_R0/1-2130	0.0876279	0.0680198	kinesin KIF27	7	F:GO:0016887; F:GO:0005524; F:GO:0003777; C:GO:0005871;	F:ATPase activity; F:ATP binding; F:microtubule motor activity; C:kinesin complex; P:microtubule-based movement; C:microtubule; F:microtubule binding	EC:3.6.1.3; EC:3.6.1.15
Acrdi.XM_015914182.1/1-1125	Monca.adi2mcaRNA24713_R0/1-1062	0.109365	0.0679746	tyrosine aminotransferase	2	P:GO:0008152; F:GO:0016740	P:metabolic process; F:transferase activity	
Acrdi.XM_015906337.1/1-1773	Monca.augustus.g23849.t1/1-1950	0.0865442	0.0679414	ATP-dependent RNA helicase DDX3X isoform X2	7	P:GO:0044260; F:GO:0097159; F:GO:1901363; F:GO:0004386;	P:cellular macromolecule metabolic process; F:organic cyclic compound binding; F:heterocyclic compound binding; F:helicase activity; P:brain development; P:cellular nitroger	EC:3.6.1.15
Acrdi.XM_015906286.1/1-654	Monca.augustus.g26533.t1/1-567	0.122912	0.0678593	Transcription factor	5	C:GO:0005634; P:GO:0006366; F:GO:0001228; F:GO:0043565;	C:nucleus; P:transcription from RNA polymerase II promoter; F:transcriptional activator activity, RNA polymerase II transcription regulatory region sequence-specific binding; F:sequence-specific DNA binding; P:positiv [illegible]	
Acrdi.XM_015892078.1/1-1266	Monca.augustus.g23821.t1/1-1254	0.0922609	0.0678443	intraflagellar transport 57 homolog	12	P:GO:0060562; C:GO:0005813; P:GO:0042073; P:GO:0048731;	P:epithelial tube morphogenesis; C:centrosome; P:intraciliary transport; P:system development; C:ciliary basal body; P:embryonic morphogenesis; C:neuron part; P:apoptotic process; P:pattern specification process; C:c [illegible]	
Acrdi.XM_015921298.1/1-1005	Monca.augustus.g40033.t1/1-1176	0.092079	0.0678341	BTB POZ domain-containing 10				
Acrdi.XM_015913126.1/1-1158	Monca.adi2mcaRNA23498_R0/1-1161	0.074442	0.0678251	probable methyltransferase 15	2	P:GO:0070475; F:GO:0071424	P:rRNA base methylation; F:rRNA (cytosine-N4-)-methyltransferase activity	
Acrdi.XM_015918841.1/1-636	Monca.adi2mcaRNA29910_R0/1-636	0.234899	0.0678033	P2X purinoceptor 7-like				
Acrdi.XM_015898249.1/1-1569	Monca.augustus.g39435.t1/1-3003	0.0832475	0.0677853	integrator complex subunit 4-like	2	P:GO:0016180; C:GO:0032039	P:snRNA processing; C:integrator complex	
Acrdi.XM_015900779.1/1-2340	Monca.augustus.g42347.t1/1-2106	0.0988858	0.0677157	CAP-Gly domain-containing linker 4	2	C:GO:0016020; C:GO:0016021	C:membrane; C:integral component of membrane	
Acrdi.XM_015894719.1/1-300	Monca.augustus.g41125.t1/1-651	0.101463	0.0677096	TK-II propeptide	4	F:GO:0004965; P:GO:0007186; C:GO:0016020; C:GO:0016021	F:G-protein coupled GABA receptor activity; P:G-protein coupled receptor signaling pathway; C:membrane; C:integral component of membrane	
Acrdi.XM_015904514.1/1-1440	Monca.augustus.g33574.t1/1-1440	0.107265	0.067684	Membrane-bound transcription factor site-2 protease	4	C:GO:0005737; P:GO:0031293; C:GO:0016021; F:GO:0004222	C:cytoplasm; P:membrane protein intracellular domain proteolysis; C:integral component of membrane; F:metalloendopeptidase activity	EC:3.4.24
Acrdi.XM_015918530.1/1-1158	Monca.adi2mcaRNA29566_R0/1-1017	0.599315	0.0676671	PREDICTED: uncharacterized protein LOC107352197				
Acrdi.XM_015923451.1/1-570	Monca.adi2mcaRNA35119_R0/1-588	0.053878	0.0676661	Pre-mRNA-splicing factor SPF27	5	C:GO:0071013; C:GO:0071011; C:GO:0071012; C:GO:0000974;	C:catalytic step 2 spliceosome; C:precatalytic spliceosome; C:catalytic step 1 spliceosome; C:Prp19 complex; P:mRNA splicing, via spliceosome	
Acrdi.XM_015919218.1/1-1659	Monca.augustus.g40927.t1/1-1560	0.0592362	0.0676476	Transcription factor glial cells missing 2	10	P:GO:0008283; P:GO:0035165; C:GO:0005634; F:GO:0003677;	P:cell proliferation; P:embryonic crystal cell differentiation; C:nucleus; F:DNA binding; F:transcription factor activity, sequence-specific DNA binding; P:negative regulation of crystal cell differentiation; P:embryonic pla [illegible]	
Acrdi.XM_015907309.1/1-1245	Monca.augustus.g31789.t1/1-1512	0.0818551	0.0676461	RAC-alpha serine threonine- kinase	19	P:GO:0090201; P:GO:0008286; P:GO:0048009; P:GO:0060416;	P:negative regulation of release of cytochrome c from mitochondria; P:insulin receptor signaling pathway; P:insulin-like growth factor receptor signaling pathway; P:response	EC:2.7.11
Acrdi.XM_015897743.1/1-2694	Monca.augustus.g51506.t1/1-2565	0.0846237	0.0676215	DNA topoisomerase 3-beta-1	3	F:GO:0003677; F:GO:0003917; P:GO:0006265	F:DNA binding; F:DNA topoisomerase type I activity; P:DNA topological change	EC:5.99.1; EC:5.99.1.2
Acrdi.XM_015921021.1/1-2052	Monca.adi2mcaRNA32347_R0/1-1797	0.0794754	0.0676039	dystrobrevin beta-like	3	F:GO:0008270; C:GO:0005737; F:GO:0046872	F:zinc ion binding; C:cytoplasm; F:metal ion binding	
Acrdi.XM_015914664.1/1-741	Monca.adi2mcaRNA25245_R0/1-738	0.327099	0.0675825	PREDICTED: uncharacterized protein LOC107348606				
Acrdi.XM_015907272.1/1-1557	Monca.adi2mcaRNA17116_R1/1-1509	0.0842436	0.0675733	thioredoxin reductase mitochondrial				
Acrdi.XM_015910028.1/1-546	Monca.augustus.g31779.t1/1-549	0.0730841	0.0675655	60S ribosomal L11				
Acrdi.XM_015901758.1/1-699	Monca.augustus.g6412.t1/1-699	0.0766388	0.0675631	Guanidinoacetate N-methyltransferase	3	P:GO:0006601; F:GO:0030731; P:GO:0032259	P:creatine biosynthetic process; F:guanidinoacetate N-methyltransferase activity; P:methylation	EC:2.1.1.2
Acrdi.XM_015918902.1/1-1623	Monca.adi2mcaRNA30021_R0/1-1503	0.0761219	0.0675405	Nucleolar GTP-binding 1	3	F:GO:0005525; P:GO:0042254; C:GO:0005730	F:GTP binding; P:ribosome biogenesis; C:nucleolus	
Acrdi.XM_015915185.1/1-717	Monca.augustus.g29743.t1/1-717	0.127901	0.067528	Homeobox Nkx-	1	F:GO:0003677	F:DNA binding	
Acrdi.XM_015893787.1/1-1623	Monca.augustus.g31997.t1/1-2073	0.0747005	0.0675248	acetyl-coenzyme A cytoplasmic isoform X2	9	F:GO:0005524; F:GO:0003987; F:GO:0016208; C:GO:0005737;	F:ATP binding; F:acetate-CoA ligase activity; F:AMP binding; C:cytoplasm; C:integral component of membrane; P:propionate biosynthetic process; P:acetate biosynthetic proce	EC:6.2.1.1
Acrdi.XM_015911961.1/1-711	Monca.augustus.g30053.t1/1-885	0.0397376	0.0675206	reticulocalbin-3 isoform X1	1	F:GO:0005509	F:calcium ion binding	
Acrdi.XM_015919367.1/1-2655	Monca.adi2mcaRNA30514_R1/1-2655	0.102715	0.0675131	serine threonine- phosphatase 6 regulatory ankyrin repeat subunit B-li	2	F:GO:0004842; P:GO:0016567	F:ubiquitin-protein transferase activity; P:protein ubiquitination	
Acrdi.XM_015895592.1/1-1719	Monca.adi2mcaRNA4444_R0/1-1356	0.0818119	0.0675122	integrator complex subunit 9	5	P:GO:0016180; F:GO:0005515; C:GO:0005737; P:GO:0042795;	P:snRNA processing; F:protein binding; C:cytoplasm; P:snRNA transcription from RNA polymerase II promoter; C:integrator complex	
Acrdi.XM_015891930.1/1-2610	Monca.augustus.g22379.t1/1-2502	0.341262	0.0675057	ankyrin repeat				
Acrdi.XM_015900047.1/1-879	Monca.augustus.g6667.t1/1-879	0.118371	0.0674644	transmembrane 68 isoform X1	3	F:GO:0016746; P:GO:0008152; C:GO:0016021	F:transferase activity, transferring acyl groups; P:metabolic process; C:integral component of membrane	
Acrdi.XM_015907114.1/1-981	Monca.adi2mcaRNA16940_R0/1-987	0.127884	0.0674605	Nucleoporin Nup37	1	C:GO:0031080	C:nuclear pore outer ring	
Acrdi.XM_015922341.1/1-915	Monca.augustus.g60146.t1/1-930	0.083818	0.0674456	ubiA prenyltransferase domain-containing 1	6	F:GO:0004659; C:GO:0043231; C:GO:0016021; C:GO:0012505;	F:prenyltransferase activity; C:intracellular membrane-bounded organelle; C:integral component of membrane; C:endomembrane system; P:quinone biosynthetic process; C:cytoplasmic part	
Acrdi.XM_015896729.1/1-711	Monca.augustus.g18920.t1/1-2712	0.0664388	0.0674186	PHD finger 14-like				
Acrdi.XM_015909590.1/1-5370	Monca.adi2mcaRNA19686_R0/1-5304	0.0650444	0.0674147	Dedicator of cytokinesis 9				
Acrdi.XM_015922834.1/1-3504	Monca.adi2mcaRNA34384_R1/1-3309	0.0686691	0.0674049	nck-associated 1 isoform X4	2	P:GO:0050794; P:GO:0007155	P:regulation of cellular process; P:cell adhesion	
Acrdi.XM_015908321.1/1-1083	Monca.augustus.g36815.t1/1-3387	0.09461	0.0673864	FAM13A-like isoform X1				
Acrdi.XM_015908470.1/1-828	Monca.augustus.g33922.t1/1-828	0.0997505	0.0673782	glucose-6-phosphate 1-epimerase	4	P:GO:0005975; F:GO:0003824; F:GO:0030246; F:GO:0016853	P:carbohydrate metabolic process; F:catalytic activity; F:carbohydrate binding; F:isomerase activity	
Acrdi.XM_015925327.1/1-3327	Monca.adi2mcaRNA37268_R1/1-2793	0.311475	0.0673725	RNA-directed DNA polymerase from mobile element jockey				
Acrdi.XM_015917972.1/1-342	Monca.adi2mcaRNA28978_R0/1-309	0.995188	0.0673352	PREDICTED: uncharacterized protein LOC107351680				
Acrdi.XM_015896524.1/1-1989	Monca.adi2mcaRNA5478_R0/1-1503	0.097312	0.0673234	solute carrier family 28 member 3-like	2	F:GO:0005337; C:GO:0016021	F:nucleoside transmembrane transporter activity; C:integral component of membrane	
Acrdi.XM_015907101.1/1-1689	Monca.augustus.g14841.t1/1-2292	0.0913047	0.0673143	Carnitine O-palmitoyltransferase liver isoform	3	F:GO:0016746; C:GO:0016020; P:GO:0009987	F:transferase activity, transferring acyl groups; C:membrane; P:cellular process	
Acrdi.XM_015906032.1/1-882	Monca.augustus.g27809.t1/1-882	0.0650119	0.0673136	epimerase family SDR39U1	1	F:GO:0003824	F:catalytic activity	
Acrdi.XM_015914360.1/1-1551	Monca.adi2mcaRNA2497_R0/1-1506	0.0978842	0.0672982	N-sulphoglucosamine sulphohydrolase-like	2	F:GO:0008484; P:GO:0008152	F:sulfuric ester hydrolase activity; P:metabolic process	
Acrdi.XM_015913715.1/1-1278	Monca.augustus.g21642.t1/1-1263	0.0930759	0.0672866	phosphatidylserine decarboxylase proenzyme isoform X2	1	C:GO:0016020	C:membrane	
Acrdi.XM_015897925.1/1-672	Monca.augustus.g39406.t1/1-765	0.0931095	0.0672865	Electron transfer flavo subunit beta	1	F:GO:0009055	F:electron carrier activity	
Acrdi.XM_015896826.1/1-1749	Monca.augustus.g33188.t1/1-1791	0.0666688	0.067285	ATPase family AAA domain-containing 3A	3	C:GO:0044429; F:GO:0005524; C:GO:0016020	C:mitochondrial part; F:ATP binding; C:membrane	
Acrdi.XM_015924863.1/1-300	Monca.augustus.g37692.t1/1-438	0.149433	0.0672605	MORN repeat-containing 2				
Acrdi.XM_015915942.1/1-669	Monca.augustus.g61001.t1/1-669	0.122576	0.0672542	zinc finger FYVE domain-containing 21-like	1	F:GO:0046872	F:metal ion binding	
Acrdi.XM_015912004.1/1-591	Monca.adi2mcaRNA22286_R1/1-579	0.413884	0.0672532	PREDICTED: uncharacterized protein LOC107346235				
Acrdi.XM_015899344.1/1-582	Monca.augustus.g40290.t1/1-582	0.0446474	0.0672443	homeobox EMX1-like	3	C:GO:0005634; P:GO:0006355; F:GO:0043565	C:nucleus; P:regulation of transcription, DNA-templated; F:sequence-specific DNA binding	

Acrdi.XM_015919015.1/1-2358	Monca.adi2mcaRNA30173_R0/1-2349	0.0805907	0.0672232	ATP-dependent RNA helicase DHX36	7	F:GO:0000166; C:GO:0005634; F:GO:0003677; F:GO:0003723;	F:nucleotide binding; C:nucleus; F:DNA binding; F:RNA binding; C:cytoplasm; P:RNA processing; F:ATP-dependent RNA helicase activity	EC:3.[illegible]
Acrdi.XM_015894711.1/1-1335	Monca.augustus.g35419.t1/1-1587	0.103096	0.0672222	TBC1 domain family member 19				
Acrdi.XM_015901509.1/1-1791	Monca.augustus.g25802.t1/1-1779	0.101392	0.06722	sodium- and chloride-dependent taurine transporter isoform X2	4	C:GO:0005887; P:GO:0015734; F:GO:0005369; P:GO:0003333	C:integral component of plasma membrane; P:taurine transport; F:taurine:sodium symporter activity; P:amino acid transmembrane transport	
Acrdi.XM_015903635.1/1-1152	Monca.augustus.g70589.t1/1-1368	0.0741693	0.0672096	ras-related RGP2-like	6	F:GO:0005525; F:GO:0003677; F:GO:0008270; P:GO:0007264;	F:GTP binding; F:DNA binding; F:zinc ion binding; P:small GTPase mediated signal transduction; P:regulation of transcription, DNA-templated; C:intracellular	
Acrdi.XM_015908387.1/1-660	Monca.augustus.g34803.t1/1-657	0.0895991	0.0671784	PREDICTED: uncharacterized protein LOC107342873				
Acrdi.XM_015922324.1/1-738	Monca.augustus.g7328.t1/1-903	0.0982091	0.0671713	CD151 antigen	2	C:GO:0005887; P:GO:0007166	C:integral component of plasma membrane; P:cell surface receptor signaling pathway	
Acrdi.XM_015906976.1/1-828	Monca.augustus.g23311.t1/1-813	0.0614119	0.067126	structure-specific endonuclease subunit slx1-like	2	P:GO:0090304; F:GO:0004519	P:nucleic acid metabolic process; F:endonuclease activity	
Acrdi.XM_015897462.1/1-5664	Monca.augustus.g22831.t1/1-3009	0.0960043	0.0670974	myotubularin-related 13 isoform X1	3	F:GO:0046872; P:GO:0035556; C:GO:0005622	F:metal ion binding; P:intracellular signal transduction; C:intracellular	
Acrdi.XM_015908019.1/1-612	Monca.augustus.g5055.t1/1-615	0.0711025	0.0670933	Ras-related Rab-9A	4	F:GO:0005525; P:GO:0007264; C:GO:0005829; P:GO:0042147	F:GTP binding; P:small GTPase mediated signal transduction; C:cytosol; P:retrograde transport, endosome to Golgi	
Acrdi.XM_015906874.1/1-1191	Monca.augustus.g32954.t1/1-1185	0.108255	0.0670872	GPN-loop GTPase 1				
Acrdi.XM_015897362.1/1-3342	Monca.augustus.g29360.t1/1-10011	0.0990614	0.0670674	Neurogenic locus notch 3				
Acrdi.XM_015907888.1/1-402	Monca.augustus.g53164.t1/1-972	0.0932769	0.0670444	Retinol dehydrogenase 13	1	C:GO:0016020	C:membrane	
Acrdi.XM_015908304.1/1-2652	Monca.augustus.g34624.t1/1-2670	0.0871768	0.0670438	Kinesin KIF17				
Acrdi.XM_015919490.1/1-2940	Monca.augustus.g61031.t1/1-3354	0.0796225	0.0669832	Colorectal mutant cancer				
Acrdi.XM_015897961.1/1-1923	Monca.adi2mcaRNA6985_R1/1-1410	0.398738	0.0669566	RNA-directed DNA polymerase from transposon X-element				
Acrdi.XM_015901710.1/1-1278	Monca.augustus.g6411.t1/1-1449	0.0964547	0.0669516	CUGBP Elav-like family member 4 isoform X5	3	F:GO:0003676; P:GO:0048024; P:GO:0006396	F:nucleic acid binding; P:regulation of mRNA splicing, via spliceosome; P:RNA processing	
Acrdi.XM_015911722.1/1-1068	Monca.augustus.g30204.t1/1-885	0.086028	0.0669435	aristaless-related homeobox -like isoform X2	1	F:GO:0003677	F:DNA binding	
Acrdi.XM_015918375.1/1-2391	Monca.adi2mcaRNA29437_R0/1-2238	0.0752131	0.0669391	calpain-5-like	3	C:GO:0005737; P:GO:0006508; F:GO:0004198	C:cytoplasm; P:proteolysis; F:calcium-dependent cysteine-type endopeptidase activity	EC:3.[illegible]
Acrdi.XM_015905786.1/1-1449	Monca.adi2mcaRNA15472_R0/1-1458	0.246192	0.0669314	Contactin-associated -like 5	1	F:GO:0003676	F:nucleic acid binding	
Acrdi.XM_015902054.1/1-1656	Monca.augustus.g43517.t1/1-1614	0.0368842	0.0668954	ALK tyrosine kinase receptor	3	F:GO:0099600; C:GO:0016020; P:GO:0006810	F:transmembrane receptor activity; C:membrane; P:transport	
Acrdi.XM_015899123.1/1-711	Monca.augustus.g60420.t1/1-711	0.0613472	0.0668886	caltractin-like	2	F:GO:0005509; P:GO:0007049	F:calcium ion binding; P:cell cycle	
Acrdi.XM_015925309.1/1-327	Monca.augustus.g54926.t1/1-975	0.0939712	0.0668688	28S ribosomal mitochondrial	3	F:GO:0003735; C:GO:0016020; C:GO:0005763	F:structural constituent of ribosome; C:membrane; C:mitochondrial small ribosomal subunit	
Acrdi.XM_015918396.1/1-1551	Monca.augustus.g22399.t1/1-3540	0.103186	0.0668642	serine threonine- kinase SIK3 isoform X1	6	C:GO:0005634; F:GO:0005524; C:GO:0005737; F:GO:0004674;	C:nucleus; F:ATP binding; C:cytoplasm; F:protein serine/threonine kinase activity; P:protein phosphorylation; P:intracellular signal transduction	EC:2.[illegible]
Acrdi.XM_015897213.1/1-600	Monca.augustus.g63846.t1/1-696	0.0840229	0.0668436	ATP-dependent Clp protease proteolytic mitochondrial	2	P:GO:0006508; F:GO:0004252	P:proteolysis; F:serine-type endopeptidase activity	EC:3.[illegible]
Acrdi.XM_015908773.1/1-903	Monca.augustus.g24462.t1/1-1377	0.0790815	0.0668188	histone-lysine N-methyltransferase PRDM6	2	F:GO:0003676; F:GO:0046872	F:nucleic acid binding; F:metal ion binding	
Acrdi.XM_015907137.1/1-810	Monca.augustus.g36956.t1/1-801	0.0643763	0.0668178	apextrin 1	1	C:GO:0016021	C:integral component of membrane	
Acrdi.XM_015902534.1/1-633	Monca.adi2mcaRNA1155_R0/1-639	0.0686665	0.066807	transmembrane 41A	1	C:GO:0016020	C:membrane	
Acrdi.XM_015910868.1/1-654	Monca.augustus.g6322.t1/1-1146	0.0958002	0.0667825	probable isocitrate dehydrogenase [NAD] subunit mitochondrial	5	F:GO:0000287; F:GO:0004449; F:GO:0051287; C:GO:0005739;	F:magnesium ion binding; F:isocitrate dehydrogenase (NAD+) activity; F:NAD binding; C:mitochondrion; P:tricarboxylic acid cycle	EC:1.[illegible]
Acrdi.XM_015892631.1/1-1110	Monca.augustus.g63889.t1/1-1659	0.0920569	0.0667717	oxysterol-binding -related 9 isoform X5	1	P:GO:0006869	P:lipid transport	
Acrdi.XM_015902410.1/1-351	Monca.augustus.g57262.t1/1-543	0.0472475	0.0667659	DNA repair RAD50	34	P:GO:0046939; C:GO:0035861; P:GO:0006284; P:GO:0007131;	P:nucleotide phosphorylation; C:site of double-strand break; P:base-excision repair; P:reciprocal meiotic recombination; F:double-stranded telomeric DNA binding; P:regulatio[illegible]	EC:2.[illegible]
Acrdi.XM_015920426.1/1-1959	Monca.adi2mcaRNA31707_R2/1-1989	0.226683	0.0667542	ATP-dependent DNA helicase PIF1				
Acrdi.XM_015924985.1/1-1035	Monca.adi2mcaRNA36872_R0/1-1371	0.0840352	0.0667524	equilibrative nucleotide transporter 1-like	3	F:GO:0005337; C:GO:0016021; P:GO:1901642	F:nucleoside transmembrane transporter activity; C:integral component of membrane; P:nucleoside transmembrane transport	
Acrdi.XM_015904369.1/1-1800	Monca.augustus.g15811.t1/1-1770	0.102662	0.0667374	Sodium-independent sulfate anion transporter	12	C:GO:0005887; F:GO:0008271; F:GO:0015301; P:GO:0051453;	C:integral component of plasma membrane; F:secondary active sulfate transmembrane transporter activity; F:anion:anion antiporter activity; P:regulation of intracellular pH; P:[illegible]	
Acrdi.XM_015916708.1/1-822	Monca.augustus.g37091.t1/1-1065	0.0768663	0.0667247	sorbitol dehydrogenase-like	2	F:GO:0016491; F:GO:0046872	F:oxidoreductase activity; F:metal ion binding	
Acrdi.XM_015892036.1/1-2178	Monca.adi2mcaRNA37935_R4/1-2208	0.554305	0.0667141	Retrovirus-related Pol poly from transposon				

[illegible]

Acrdi.XM_015905059.1/1-531 | Monca.adi2mcaRNA14668_R1/1-504	0.585739	0.0619019	ATP-dependent DNA helicase Q-like 3	4	F:GO:0003676; C:GO:0043229; P:GO:0006281; F:GO:0003678
Acrdi.XM_015904968.1/1-594 | Monca.augustus.g55919.t1/1-591	0.0703909	0.0618727	COMM domain-containing 4		
Acrdi.XM_015913712.1/1-1695 | Monca.augustus.g26410.t1/1-1698	0.0745992	0.0618655	Phosphoglucomutase-1	11	F:GO:0000287; P:GO:0006094; F:GO:0004614; P:GO:0005978;
Acrdi.XM_015899614.1/1-1065 | Monca.augustus.g4245.t1/1-1278	0.0666025	0.0618649	saccharopine dehydrogenase-like oxidoreductase	1	C:GO:0016020
Acrdi.XM_015896775.1/1-471 | Monca.augustus.g12342.t1/1-468	0.101438	0.0618639	7,8-dihydro-8-oxoguanine triphosphatase	17	F:GO:0044714; F:GO:0050072; C:GO:0005886; F:GO:0044713;
Acrdi.XM_015914551.1/1-1875 | Monca.augustus.g29113.t1/1-1875	0.058949	0.0618535	potassium voltage-gated channel Shal isoform X1	5	P:GO:0071805; C:GO:0008076; F:GO:0005251; P:GO:0034765;
Acrdi.XM_015913576.1/1-1584 | Monca.adi2mcaRNA24050_R1/1-1470	0.707357	0.0618502	Pro-Pol poly		
Acrdi.XM_015899122.1/1-1356 | Monca.augustus.g60411.t1/1-1503	0.0575959	0.0618248	alpha-L-fucosidase-like	2	P:GO:0005975; F:GO:0016798
Acrdi.XM_015904468.1/1-726 | Monca.augustus.g6290.t1/1-1404	0.0663551	0.0618247	eukaryotic translation initiation factor 2 subunit 3	10	F:GO:0000166; C:GO:0005634; F:GO:0003743; P:GO:0001731;
Acrdi.XM_015904264.1/1-3471 | Monca.augustus.g62125.t1/1-5103	0.0755333	0.0618237	Tight junction ZO-1	1	P:GO:0042981
Acrdi.XM_015894003.1/1-2580 | Monca.adi2mcaRNA40167_R0/1-2871	0.0701635	0.0618119	Myosin-2 heavy chain	5	F:GO:0008061; P:GO:0006030; C:GO:0016020; C:GO:0016021;
Acrdi.XM_015913192.1/1-600 | Monca.augustus.g39287.t1/1-666	0.0754965	0.0617917	Allene oxide synthase-lipoxygenase	2	F:GO:0016491; F:GO:0005488
Acrdi.XM_015923402.1/1-3048 | Monca.augustus.g9020.t1/1-3378	0.435597	0.0617652	PREDICTED: uncharacterized protein K02A2.6-like		
Acrdi.XM_015900713.1/1-3078 | Monca.augustus.g71550.t1/1-3072	0.342334	0.0617636	gastrointestinal growth factor xP4	5	C:GO:0005856; P:GO:0007165; F:GO:0005509; C:GO:0016020;
Acrdi.XM_015909848.1/1-945 | Monca.augustus.g40677.t1/1-408	0.106455	0.0617628	E3 ubiquitin- ligase RING2	6	C:GO:0000151; F:GO:0003824; F:GO:0046872; C:GO:0031519;
Acrdi.XM_015898365.1/1-495 | Monca.adi2mcaRNA7402_R0/1-495	0.0806941	0.0617431	transmembrane channel 3	1	C:GO:0016021
Acrdi.XM_015906790.1/1-798 | Monca.augustus.g7738.t1/1-798	0.0978262	0.0617001	lysoplasmalogenase TMEM86A	1	C:GO:0016020
Acrdi.XM_015917210.1/1-1104 | Monca.augustus.g52212.t1/1-1104	0.0688009	0.0616936	beta-parvin-like isoform X4	3	F:GO:0003779; P:GO:0007155; P:GO:0031532
Acrdi.XM_015911190.1/1-1317 | Monca.augustus.g29786.t1/1-1485	0.524914	0.0616907	probable Werner syndrome ATP-dependent helicase homolog 1	2	P:GO:0006259; F:GO:0016787
Acrdi.XM_015895683.1/1-3216 | Monca.augustus.g63256.t1/1-3384	0.0906668	0.0616594	MBT domain-containing 1	3	C:GO:0005634; F:GO:0008270; P:GO:0006355
Acrdi.XM_015907662.1/1-561 | Monca.augustus.g29921.t1/1-2310	0.0818362	0.0616259	PREDICTED: uncharacterized protein LOC107342187		
Acrdi.XM_015923373.1/1-456 | Monca.augustus.g24896.t1/1-450	0.117662	0.0615627	Universal stress	1	P:GO:0006950
Acrdi.XM_015900018.1/1-1752 | Monca.augustus.g51847.t1/1-2346	0.0799607	0.0615383	epidermal growth factor receptor substrate 15-like 1 isoform X1		
Acrdi.XM_015910984.1/1-4239 | Monca.augustus.g5615.t1/1-5064	0.0668677	0.0615149	voltage-gated sodium channel	7	P:GO:0006814; P:GO:0034220; C:GO:0016021; P:GO:0086010;
Acrdi.XM_015900550.1/1-1320 | Monca.augustus.g26475.t1/1-1311	0.0749633	0.0615057	ankyrin repeat domain-containing 13C isoform X1	6	C:GO:0005789; P:GO:2000209; C:GO:0048471; P:GO:0010869;
Acrdi.XM_015923370.1/1-3360 | Monca.adi2mcaRNA35036_R0/1-3357	0.0864331	0.0614863	Adenylate cyclase type 5	11	F:GO:0000166; P:GO:0007193; P:GO:0006171; P:GO:0045471;
Acrdi.XM_015923513.1/1-1077 | Monca.augustus.g56464.t1/1-984	0.0647056	0.0614771	potassium channel subfamily K member 1-like isoform X2	4	C:GO:0005887; P:GO:0071805; P:GO:0030322; F:GO:0022841
Acrdi.XM_015904408.1/1-624 | Monca.augustus.g48074.t1/1-621	0.0795268	0.06147	ras-related Rab-10	3	F:GO:0005525; P:GO:0007264; C:GO:0005622
Acrdi.XM_015923844.1/1-1107 | Monca.adi2mcaRNA35549_R0/1-1113	0.0916975	0.0614363	Dual specificity phosphatase 7	3	P:GO:0000188; P:GO:0006470; F:GO:0017017
Acrdi.XM_015894659.1/1-1203 | Monca.adi2mcaRNA40969_R0/1-1128	0.823806	0.0614217	PREDICTED: uncharacterized protein LOC107330000		
Acrdi.XM_015913583.1/1-1242 | Monca.adi2mcaRNA22310_R9/1-1446	0.536498	0.0614138	RNA-directed DNA polymerase from mobile element jockey-like		
Acrdi.XM_015920926.1/1-1767 | Monca.augustus.g53566.t1/1-2016	0.0830683	0.0614017	arginine--tRNA cytoplasmic	4	F:GO:0005524; F:GO:0004814; C:GO:0005737; P:GO:0006420
Acrdi.XM_015909727.1/1-1839 | Monca.augustus.g41742.t1/1-1821	0.081153	0.0614013	ATP-dependent Clp protease ATP-binding subunit clpX- mitochondrial	7	F:GO:0005524; C:GO:0005759; P:GO:0030163; P:GO:0006508;
Acrdi.XM_015908900.1/1-1587 | Monca.augustus.g29211.t1/1-1647	0.0843332	0.0613781	armadillo repeat-containing 8 isoform X2		
Acrdi.XM_015903726.1/1-1887 | Monca.adi2mcaRNA13202_R0/1-1929	0.490953	0.0613345	nek6 si:ch211- NIMA (never in mitosis gene a)-related kinase		
Acrdi.XM_015892706.1/1-1116 | Monca.adi2mcaRNA38700_R0/1-1131	0.0678118	0.0612763	BTB POZ domain-containing 6	2	C:GO:0005737; P:GO:0006511
Acrdi.XM_015913764.1/1-2958 | Monca.augustus.g45560.t1/1-3072	0.0758608	0.0612683	small G signaling modulator 2 isoform X2	7	P:GO:0031338; P:GO:0090630; F:GO:0005096; C:GO:0012505;
Acrdi.XM_015912095.1/1-543 | Monca.augustus.g49197.t1/1-546	0.0830438	0.0612522	Group XIIA secretory phospholipase A2	4	F:GO:0004623; F:GO:0005509; P:GO:0016042; C:GO:0005576
Acrdi.XM_015909206.1/1-1698 | Monca.augustus.g49393.t1/1-1695	0.076419	0.0612509	WD repeat-containing 20 isoform X1	3	P:GO:0008150; C:GO:0005575; F:GO:0003674
Acrdi.XM_015904631.1/1-2445 | Monca.augustus.g31423.t1/1-2892	0.0909911	0.0612444	PTHB1 isoform X3	2	C:GO:0034464; C:GO:0016020
Acrdi.XM_015921439.1/1-1407 | Monca.adi2mcaRNA32792_R1/1-1104	0.629351	0.0612294	PREDICTED: uncharacterized protein LOC107354944		
Acrdi.XM_015925004.1/1-1038 | Monca.augustus.g53479.t1/1-1470	0.0816711	0.0612282	serine threonine- kinase 25	6	P:GO:0050794; C:GO:0000139; F:GO:0004672; P:GO:0044763;
Acrdi.XM_015895686.1/1-1710 | Monca.adi2mcaRNA4436_R0/1-1707	0.0723848	0.0612251	kelch 12	3	C:GO:0044424; P:GO:0044699; P:GO:0009987
Acrdi.XM_015910137.1/1-801 | Monca.adi2mcaRNA20306_R9/1-786	0.28887	0.0612119	PREDICTED: uncharacterized protein LOC107344481		
Acrdi.XM_015924312.1/1-2904 | Monca.augustus.g26371.t1/1-2160	1.0556	0.0611704	PREDICTED: uncharacterized protein LOC107357678, partial		
Acrdi.XM_015910818.1/1-1452 | Monca.augustus.g70022.t1/1-1887	0.0717439	0.0610953	aryl hydrocarbon receptor nuclear translocator 1	9	C:GO:0005667; P:GO:0045892; P:GO:0045893; P:GO:1901342;
Acrdi.XM_015902751.1/1-810 | Monca.augustus.g26643.t1/1-873	0.297709	0.0610902	PREDICTED: uncharacterized protein LOC107337552		
Acrdi.XM_015915038.1/1-1491 | Monca.augustus.g64703.t1/1-1542	0.0798482	0.0610567	leupaxin isoform X3	3	P:GO:0007160; F:GO:0008270; C:GO:0005856
Acrdi.XM_015907237.1/1-1101 | Monca.augustus.g7060.t1/1-1095	0.100843	0.0609836	Sperm flagellar 1	1	C:GO:0031514
Acrdi.XM_015898693.1/1-603 | Monca.augustus.g2076.t1/1-708	0.0731052	0.0609476	PREDICTED: uncharacterized protein C1orf158 homolog, partial		
Acrdi.XM_015892467.1/1-2421 | Monca.augustus.g38254.t1/1-2526	0.0750437	0.0609281	phosphofurin acidic cluster sorting 2-like isoform X3	2	P:GO:0072661; C:GO:0005622
Acrdi.XM_015907063.1/1-1725 | Monca.augustus.g29188.t1/1-1725	0.0596104	0.0609281	polypeptide N-acetylgalactosaminyltransferase	5	F:GO:0016757; C:GO:0016021; P:GO:0006486; C:GO:0000139;
Acrdi.XM_015900201.1/1-663 | Monca.augustus.g65829.t1/1-1047	0.0967242	0.0609234	Guanine nucleotide-binding subunit beta-5	2	P:GO:0007186; C:GO:0005886
Acrdi.XM_015899918.1/1-1566 | Monca.adi2mcaRNA9024_R1/1-1551	0.074853	0.0609194	vesicular glutamate transporter	4	P:GO:0055085; F:GO:0022857; C:GO:0016021; P:GO:0006820
Acrdi.XM_015900632.1/1-735 | Monca.augustus.g3622.t1/1-729	0.0543565	0.0609184	Pituitary-specific positive transcription factor 1	5	C:GO:0005634; F:GO:0003700; P:GO:0006351; P:GO:0006355;
Acrdi.XM_015894293.1/1-972 | Monca.augustus.g64050.t1/1-1215	0.0781778	0.0609041	PCI domain-containing 2	7	P:GO:0090267; P:GO:0045893; P:GO:0043066; P:GO:0048536;
Acrdi.XM_015914663.1/1-1554 | Monca.adi2mcaRNA25242_R0/1-1707	0.254726	0.0608997	SPRY domain-containing SOCS box 4	1	F:GO:0046872
Acrdi.XM_015894109.1/1-873 | Monca.augustus.g66766.t1/1-879	0.0883032	0.0608684	thioredoxin 1	9	F:GO:0015035; C:GO:0005739; P:GO:0006662; F:GO:0016671;
Acrdi.XM_015899578.1/1-1050 | Monca.augustus.g21740.t1/1-1056	0.0746248	0.0608684	L-threonine 3- mitochondrial-like	2	F:GO:0050662; F:GO:0003824
Acrdi.XM_015914199.1/1-912 | Monca.adi2mcaRNA24725_R0/1-918	0.10149	0.0608555	AKT-interacting isoform X1	4	F:GO:0061630; F:GO:0031625; C:GO:0005737; P:GO:0016567
Acrdi.XM_015901231.1/1-1362 | Monca.adi2mcaRNA10499_R1/1-1362	0.296146	0.0608199	Transposon Tf2-6 poly	2	F:GO:0003676; F:GO:0046872
Acrdi.XM_015900463.1/1-1857 | Monca.adi2mcaRNA9642_R5/1-1662	0.675154	0.0608164	Transposon Tf2-6 poly		
Acrdi.XM_015905519.1/1-1215 | Monca.augustus.g19908.t1/1-1182	0.0542791	0.0608141	carbamoyl phosphate synthase large subunit	3	F:GO:0005524; F:GO:0003824; F:GO:0046872
Acrdi.XM_015900155.1/1-1035 | Monca.augustus.g10473.t1/1-1299	0.0785652	0.0608117	Alpha-1,3 1,6-mannosyltransferase ALG2	2	C:GO:0016020; F:GO:0016740
Acrdi.XM_015918915.1/1-1425 | Monca.augustus.g48520.t1/1-1464	0.0745752	0.0607915	limb region 1 homolog	1	C:GO:0016020
Acrdi.XM_015918361.1/1-399 | Monca.augustus.g41669.t1/1-339	0.0886273	0.060783	Sulfiredoxin-1	4	F:GO:0005524; F:GO:0032542; P:GO:0098869; P:GO:0055114
Acrdi.XM_015896774.1/1-546 | Monca.augustus.g66645.t1/1-666	0.0712374	0.0607474	mediator complex subunit MED25 variant	4	C:GO:0005634; P:GO:0006351; P:GO:0060021; P:GO:0006355
Acrdi.XM_015923068.1/1-2493 | Monca.augustus.g4238.t1/1-3180	0.0692224	0.0607203	Membrane-bound transcription factor site-1 protease	3	P:GO:0006508; C:GO:0016021; F:GO:0004252
Acrdi.XM_015906239.1/1-1146 | Monca.augustus.g21683.t1/1-1572	0.0849547	0.0606884	Leucine-rich repeat-containing 74A	1	F:GO:0005509

Acrdi.XM_015916670.1/1-2262 | Monca.augustus.g41832.t1/1-2277	0.0527162	0.0559586	FACT complex subunit SSRP1	5	F:GO:0003676; P:GO:0090304; C:GO:0043229; P:GO:0044260;	[illegible]
Acrdi.XM_015900552.1/1-1512 | Monca.augustus.g26467.t1/1-1506	0.0696589	0.0559493	cell division cycle 20 homolog	4	F:GO:0097027; P:GO:0050790; F:GO:0010997; P:GO:0009987	[illegible]
Acrdi.XM_015911512.1/1-537 | Monca.augustus.g10122.t1/1-615	0.0807739	0.0559113	GTP-binding 6	3	F:GO:0005525; C:GO:0005737; F:GO:0043022	[illegible]
Acrdi.XM_015903238.1/1-744 | Monca.augustus.g7500.t1/1-747	0.0742205	0.0559073	radial spoke head 1 homolog	1	C:GO:0043229	[illegible]
Acrdi.XM_015897336.1/1-639 | Monca.augustus.g48412.t1/1-642	0.0803501	0.0558839	orai-2-like isoform X2	4	F:GO:0015279; P:GO:0002115; C:GO:0016021; P:GO:0070588	[illegible]
Acrdi.XM_015901723.1/1-1647 | Monca.augustus.g61709.t1/1-2091	0.0812237	0.0558719	Exostosin-2	15	P:GO:0015012; P:GO:0033692; F:GO:0015020; P:GO:0006024;	[illegible]
Acrdi.XM_015898058.1/1-1461 | Monca.augustus.g29264.t1/1-1473	0.0661104	0.055867	voltage-dependent L-type calcium channel subunit beta-2 isoform X10	6	P:GO:0007528; F:GO:0008331; P:GO:1901385; C:GO:0005891;	[illegible]
Acrdi.XM_015904991.1/1-2211 | Monca.augustus.g42967.t1/1-1593	0.415584	0.0558171	G2 M phase-specific E3 ubiquitin- ligase-like			
Acrdi.XM_015895782.1/1-1290 | Monca.augustus.g47030.t1/1-1218	0.0741481	0.0558056	tripartite motif-containing	3	F:GO:0008270; F:GO:0046872; C:GO:0005622	[illegible]
Acrdi.XM_015897339.1/1-753 | Monca.augustus.g48414.t1/1-1971	0.0533309	0.0558006	PREDICTED: uncharacterized protein LOC107332602, partial	1	P:GO:0060828	[illegible]
Acrdi.XM_015915654.1/1-600 | Monca.augustus.g16869.t1/1-600	0.116222	0.0557613	TEX261	5	P:GO:0006888; C:GO:0030173; C:GO:0030176; C:GO:0030134;	[illegible]
Acrdi.XM_015921596.1/1-618 | Monca.augustus.g41674.t1/1-618	0.0755362	0.0557613	Zinc finger matrin-type 2	5	F:GO:0008270; F:GO:0003723; F:GO:0005515; C:GO:0046540;	[illegible]
Acrdi.XM_015901335.1/1-678 | Monca.augustus.g48485.t1/1-678	0.0668409	0.0557582	V-type proton ATPase subunit E	7	C:GO:0000221; C:GO:0033181; P:GO:0015991; P:GO:0045176;	[illegible]
Acrdi.XM_015895165.1/1-489 | Monca.augustus.g36080.t1/1-528	0.685421	0.0557533	Transposon Ty3-G Gag-Pol poly			
Acrdi.XM_015918187.1/1-783 | Monca.augustus.g66560.t1/1-765	0.0728661	0.0557438	homeobox even-skipped homolog 2-like	3	C:GO:0005634; P:GO:0006355; F:GO:0043565	[illegible]
Acrdi.XM_015911080.1/1-2079 | Monca.augustus.g55132.t1/1-2565	0.0536162	0.0557397	Dipeptidyl peptidase 9	2	P:GO:0006508; F:GO:0008236	[illegible]
Acrdi.XM_015908619.1/1-1527 | Monca.augustus.g22366.t1/1-1917	0.0639091	0.0557304	Very long-chain specific acyl- mitochondrial	10	P:GO:0033539; P:GO:0060322; F:GO:0000062; F:GO:0050660;	[illegible]
Acrdi.XM_015899419.1/1-6483 | Monca.augustus.g46721.t1/1-6885	0.0918793	0.0557266	transmembrane FLJ37396 isoform X1			
Acrdi.XM_015895588.1/1-1701 | Monca.augustus.g63255.t1/1-1863	0.0643805	0.0556895	myotubularin-related 2 isoform X2	23	C:GO:0014069; F:GO:0042803; P:GO:0002091; C:GO:0008021;	[illegible]
Acrdi.XM_015909260.1/1-402 | Monca.augustus.g35320.t1/1-402	0.0936468	0.0556725	transmembrane 203	1	C:GO:0016021	[illegible]
Acrdi.XM_015914989.1/1-1485 | Monca.adi2mcaRNA25613_R0/1-1263	0.490891	0.0556578	PREDICTED: uncharacterized protein LOC107348900	1	F:GO:0003676	[illegible]
Acrdi.XM_015897264.1/1-957 | Monca.augustus.g41222.t1/1-1128	0.0338741	0.0556371	creatine kinase M-type	6	F:GO:0005524; C:GO:0005615; P:GO:0009408; C:GO:0005737;	[illegible]
Acrdi.XM_015896165.1/1-1209 | Monca.augustus.g23023.t1/1-1500	0.0541259	0.0555994	heat shock factor 1 isoform X5			
Acrdi.XM_015912834.1/1-1695 | Monca.adi2mcaRNA23232_R0/1-1665	0.059203	0.0555602	Armadillo repeat-containing 3	2	C:GO:0005634; C:GO:0005737	[illegible]
Acrdi.XM_015916307.1/1-549 | Monca.adi2mcaRNA27090_R0/1-534	0.0625823	0.0555557	lifeguard 4-like	2	C:GO:0016020; C:GO:0016021	[illegible]
Acrdi.XM_015897513.1/1-960 | Monca.augustus.g32463.t1/1-957	0.0666406	0.0555513	Transmembrane 165	1	C:GO:0016021	[illegible]
Acrdi.XM_015900074.1/1-843 | Monca.adi2mcaRNA9234_R2/1-990	0.289601	0.055549	PREDICTED: uncharacterized protein LOC107335106			
Acrdi.XM_015914427.1/1-1125 | Monca.augustus.g41422.t1/1-1206	0.0990954	0.0555143	Dihydroorotate dehydrogenase (quinone) mitochondrial	6	P:GO:0006207; C:GO:0016021; F:GO:0004152; P:GO:0055114;	[illegible]
Acrdi.XM_015916610.1/1-1155 | Monca.augustus.g8787.t1/1-1158	0.0928704	0.0555006	WD repeat-containing 88			
Acrdi.XM_015911317.1/1-630 | Monca.augustus.g67449.t1/1-630	0.0622181	0.0554759	transcription factor ETV7-like isoform X1	5	C:GO:0005634; P:GO:0030154; F:GO:0043565; F:GO:0000981;	[illegible]
Acrdi.XM_015907758.1/1-3009 | Monca.augustus.g16723.t1/1-2997	0.0809689	0.0554723	FAN-like	3	C:GO:0012505; C:GO:0019898; F:GO:0005543	[illegible]
Acrdi.XM_015912163.1/1-1245 | Monca.augustus.g6356.t1/1-1257	0.0691254	0.055449	Iron zinc purple acid phosphatase	3	F:GO:0046872; P:GO:0016311; F:GO:0003993	[illegible]
Acrdi.XM_015916986.1/1-957 | Monca.augustus.g10883.t1/1-1020	0.0616999	0.0554376	spermatogenesis-associated 6-like	2	P:GO:0007154; C:GO:0016021	[illegible]
Acrdi.XM_015914767.1/1-468 | Monca.augustus.g61016.t1/1-2277	0.0668666	0.0554277	TFIIH basal transcription factor complex helicase XPD subunit-like	4	F:GO:0097159; F:GO:1901363; F:GO:0004386; P:GO:0009987	[illegible]
Acrdi.XM_015916248.1/1-1716 | Monca.augustus.g70494.t1/1-2016	0.0759608	0.0553966	translation factor mitochondrial	7	F:GO:0005525; P:GO:0045727; C:GO:0005759; F:GO:0003924;	[illegible]
Acrdi.XM_015920335.1/1-1530 | Monca.augustus.g36359.t1/1-1974	0.0394901	0.0553774	O-linked-mannose beta-1,2-N-acetylglucosaminyltransferase 1	3	F:GO:0008375; C:GO:0016020; P:GO:0006486	[illegible]
Acrdi.XM_015910786.1/1-2271 | Monca.adi2mcaRNA20985_R0/1-2262	0.0734762	0.0553671	serine threonine- phosphatase 4 regulatory subunit 3A isoform X2			
Acrdi.XM_015914434.1/1-1245 | Monca.augustus.g57935.t1/1-1893	0.0743503	0.0553377	transcription factor COE1 isoform X4	3	C:GO:0005634; F:GO:0001077; P:GO:0045944	[illegible]
Acrdi.XM_015909519.1/1-735 | Monca.adi2mcaRNA19619_R1/1-735	0.104182	0.0553331	persulfide dioxygenase mitochondrial-like	1	F:GO:0016787	[illegible]
Acrdi.XM_015912195.1/1-378 | Monca.augustus.g3409.t1/1-2799	0.047315	0.0553298	splicing factor 3B subunit 2	1	C:GO:0005634	[illegible]
Acrdi.XM_015919615.1/1-519 | Monca.augustus.g7135.t1/1-519	0.0626938	0.0552858	NADH dehydrogenase [ubiquinone] 1 alpha subcomplex assembly facto	1	C:GO:0043231	[illegible]
Acrdi.XM_015907723.1/1-633 | Monca.augustus.g68020.t1/1-855	0.0741001	0.0552686	Type 1 phosphatidylinositol 4,5-bisphosphate 4-phosphatase	3	C:GO:0016021; F:GO:0034597; P:GO:0046856	[illegible]
Acrdi.XM_015904623.1/1-1299 | Monca.augustus.g22473.t1/1-1335	0.109217	0.0552547	probable ATP-dependent RNA helicase ddx6	8	F:GO:0003676; P:GO:0006417; F:GO:0005524; C:GO:0010494;	[illegible]
Acrdi.XM_015894310.1/1-1491 | Monca.augustus.g61979.t1/1-2040	0.0646196	0.0552343	dynein intermediate chain axonemal			
Acrdi.XM_015910359.1/1-753 | Monca.augustus.g36427.t1/1-756	0.0728248	0.0552316	carboxymethylenebutenolidase homolog isoform X2	1	F:GO:0016787	[illegible]
Acrdi.XM_015915339.1/1-942 | Monca.augustus.g25301.t1/1-1152	0.0675296	0.0552316	coiled-coil domain-containing 42 homolog			
Acrdi.XM_015907443.1/1-1485 | Monca.augustus.g3961.t1/1-1788	0.0574084	0.0552297	choline mitochondrial	5	P:GO:0019285; F:GO:0050660; C:GO:0005739; P:GO:0055114;	[illegible]
Acrdi.XM_015900540.1/1-495 | Monca.augustus.g37044.t1/1-495	0.0790956	0.0552094	coiled-coil domain-containing 12	3	P:GO:0008150; C:GO:0005575; F:GO:0003674	[illegible]
Acrdi.XM_015921667.1/1-774 | Monca.adi2mcaRNA33031_R1/1-774	0.187368	0.0551854	unc-13 homolog C-like	5	C:GO:0030529; P:GO:0032197; F:GO:0003727; F:GO:0019992;	[illegible]
Acrdi.XM_015907800.1/1-918 | Monca.augustus.g4425.t1/1-906	0.069497	0.055184	neuropeptide FF receptor 2-like isoform X1	3	F:GO:0004930; P:GO:0007186; C:GO:0016021	[illegible]
Acrdi.XM_015897619.1/1-1509 | Monca.augustus.g4548.t1/1-1518	0.0861878	0.0551478	neuronal acetylcholine receptor subunit alpha-10-like	5	C:GO:0045211; P:GO:0098655; C:GO:0016021; C:GO:0030054;	[illegible]
Acrdi.XM_015922261.1/1-5166 | Monca.augustus.g35173.t1/1-5391	0.355125	0.0551198	NFX1-type zinc finger-containing 1	4	C:GO:0005634; F:GO:0003700; F:GO:0008270; P:GO:0006355	[illegible]
Acrdi.XM_015903013.1/1-1278 | Monca.augustus.g47586.t1/1-1485	0.0724476	0.0551107	Transmembrane 180	1	C:GO:0016021	[illegible]
Acrdi.XM_015899466.1/1-498 | Monca.augustus.g43418.t1/1-963	0.0715076	0.0550732	THO complex subunit 3	2	P:GO:0006406; C:GO:0000445	[illegible]
Acrdi.XM_015923586.1/1-627 | Monca.augustus.g33338.t1/1-627	0.0497253	0.0550608	DNA replication complex GINS PSF3-like	2	C:GO:0005634; P:GO:0006260	[illegible]
Acrdi.XM_015919041.1/1-1932 | Monca.adi2mcaRNA30198_R0/1-2055	0.0752412	0.055042	U4 U6 small nuclear ribonucleo Prp3	3	C:GO:0046540; C:GO:0005654; P:GO:0000398	[illegible]
Acrdi.XM_015896301.1/1-1392 | Monca.adi2mcaRNA5169_R0/1-1392	0.0659153	0.0550387	methylcrotonoyl- carboxylase beta mitochondrial	1	F:GO:0016874	[illegible]
Acrdi.XM_015894182.1/1-1581 | Monca.augustus.g9810.t1/1-2346	0.061764	0.0550262	probable aconitate mitochondrial	3	P:GO:0006099; F:GO:0051539; F:GO:0003994	[illegible]
Acrdi.XM_015897149.1/1-1161 | Monca.augustus.g39404.t1/1-1203	0.0894765	0.0550243	intraflagellar transport 46 homolog	4	C:GO:0005813; P:GO:0042073; C:GO:0005929; C:GO:0030992	[illegible]
Acrdi.XM_015921517.1/1-981 | Monca.augustus.g42437.t1/1-987	0.0924654	0.055014	Tricarboxylate transport mitochondrial	4	P:GO:0055085; F:GO:0003735; C:GO:0016021; P:GO:0006412	[illegible]
Acrdi.XM_015921235.1/1-636 | Monca.augustus.g67780.t1/1-963	0.0773154	0.0549831	Eukaryotic translation initiation factor 3 subunit I	6	F:GO:0003743; P:GO:0001731; C:GO:0016282; C:GO:0033290;	[illegible]
Acrdi.XM_015910739.1/1-1659 | Monca.augustus.g48005.t1/1-2343	0.0604157	0.0549038	E3 ubiquitin- ligase TRIM71	4	F:GO:0008270; F:GO:0046872; P:GO:0010172; C:GO:0005622	[illegible]
Acrdi.XM_015919948.1/1-681 | Monca.adi2mcaRNA31194_R0/1-927	0.94041	0.0548957	serine threonine- kinase ULK4	8	F:GO:0000166; F:GO:0005524; F:GO:0004672; P:GO:0016310;	[illegible]
Acrdi.XM_015907235.1/1-1167 | Monca.adi2mcaRNA17072_R0/1-1164	0.0600664	0.0548767	intracellular transport USO1-like isoform X2			
Acrdi.XM_015914887.1/1-1623 | Monca.adi2mcaRNA25517_R0/1-1503	0.401579	0.0548753	PREDICTED: uncharacterized protein LOC107348808			
Acrdi.XM_015920459.1/1-1590 | Monca.augustus.g47231.t1/1-1593	0.0537156	0.0548086	bone morphogenetic receptor type-1A	72	P:GO:0060045; P:GO:0009950; P:GO:1904414; C:GO:0043025;	[illegible]
Acrdi.XM_015900720.1/1-726 | Monca.augustus.g13008.t1/1-720	0.13048	0.0548049	tgf(beta)-induced transcription	3	C:GO:0005634; F:GO:0003677; P:GO:0006355	[illegible]
Acrdi.XM_015919020.1/1-1089 | Monca.augustus.g32015.t1/1-1089	0.0921643	0.05478	Krueppel-like factor	1	P:GO:0050794	[illegible]
Acrdi.XM_015924484.1/1-2004 | Monca.adi2mcaRNA36293_R0/1-1617	0.263271	0.0547391	PREDICTED: uncharacterized protein LOC107357843			
Acrdi.XM_015922069.1/1-582 | Monca.augustus.g11595.t1/1-792	0.0886017	0.0546936	tetraspanin-5 isoform X1	6	C:GO:0005887; F:GO:0016491; P:GO:0090002; P:GO:0007166;	[illegible]
Acrdi.XM_015912995.1/1-522 | Monca.augustus.g56221.t1/1-1485	0.0593566	0.0546361	inositol 2-dehydrogenase-like	1	P:GO:0008152	[illegible]
Acrdi.XM_015924819.1/1-603 | Monca.augustus.g67348.t1/1-603	0.0653735	0.0546037	cysteine dioxygenase type 1	7	P:GO:0000097; F:GO:0017172; F:GO:0005506; P:GO:0019451;	[illegible]
Acrdi.XM_015913388.1/1-999 | Monca.adi2mcaRNA23846_R0/1-999	0.313659	0.0546027	cathepsin L1-like	4	C:GO:0005615; F:GO:0004197; P:GO:0051603; C:GO:0005764	[illegible]
Acrdi.XM_015916299.1/1-420 | Monca.augustus.g70711.t1/1-420	0.0926713	0.0545941	Baculoviral IAP repeat-containing 5	8	P:GO:1990001; C:GO:0005634; C:GO:0005876; F:GO:0004842;	[illegible]
Acrdi.XM_015923766.1/1-1839 | Monca.augustus.g71204.t1/1-2880	0.068416	0.0545904	coiled-coil domain-containing			
Acrdi.XM_015906033.1/1-741 | Monca.augustus.g27810.t1/1-1251	0.0637813	0.0545282	transmembrane prolyl 4-hydroxylase-like	2	F:GO:0016491; F:GO:0043167	[illegible]
Acrdi.XM_015919261.1/1-447 | Monca.augustus.g60216.t1/1-741	0.0437136	0.054524	F-box only 8	1	P:GO:0065007	[illegible]
Acrdi.XM_015918928.1/1-1008 | Monca.augustus.g48524.t1/1-1119	0.0700212	0.0544926	cysteine-rich secretory LCCL domain-containing 2-like	1	C:GO:0005576	[illegible]
Acrdi.XM_015896723.1/1-726 | Monca.augustus.g18688.t1/1-732	0.076234	0.0544889	Proteasome activator complex subunit 3	11	F:GO:0061133; C:GO:0005634; F:GO:0002039; P:GO:0061136;	[illegible]
Acrdi.XM_015896529.1/1-2760 | Monca.augustus.g22626.t1/1-2757	0.0854163	0.0544885	sodium calcium exchanger 1 isoform X3	5	P:GO:0007154; P:GO:0006816; C:GO:0016021; P:GO:0035725;	[illegible]
Acrdi.XM_015895714.1/1-2310 | Monca.augustus.g17452.t1/1-2310	0.0474333	0.0544875	bicaudal D homolog 2-like	2	C:GO:0005794; P:GO:0006810	[illegible]
Acrdi.XM_015903545.1/1-1194 | Monca.adi2mcaRNA12953_R1/1-1134	0.0469281	0.0544874	lymphocyte expansion molecule			
Acrdi.XM_015920943.1/1-1449 | Monca.augustus.g4097.t1/1-1449	0.0934765	0.0544413	Glutamate dehydrogenase	3	F:GO:0016639; P:GO:0006520; P:GO:0055114	[illegible]
Acrdi.XM_015921636.1/1-1833 | Monca.augustus.g49846.t1/1-1908	0.0837823	0.0543932	Cytoplasmic polyadenylation element-binding 2	13	F:GO:0000900; F:GO:0008135; C:GO:0045202; C:GO:0043005;	[illegible]
Acrdi.XM_015904909.1/1-924 | Monca.adi2mcaRNA14456_R2/1-885	0.129102	0.0543282	Transposon Ty3-G Gag-Pol poly			
Acrdi.XM_015916992.1/1-1248 | Monca.augustus.g26566.t1/1-1185	0.0571468	0.0543185	beta-N-acetylglucosaminidase	3	P:GO:0005975; F:GO:0004563; F:GO:0030247	[illegible]
Acrdi.XM_015898497.1/1-747 | Monca.augustus.g41767.t1/1-747	0.0682248	0.0542863	PREDICTED: uncharacterized protein LOC107333667			
Acrdi.XM_015914641.1/1-843 | Monca.augustus.g9593.t1/1-804	0.588272	0.0542602	DNA polymerase III polC-type	1	F:GO:0003676	[illegible]
Acrdi.XM_015901925.1/1-1341 | Monca.augustus.g7652.t1/1-1341	0.0797994	0.0542571	disulfide-isomerase A6	6	F:GO:0003756; P:GO:0043277; C:GO:0005783; P:GO:0045454;	[illegible]
Acrdi.XM_015923189.1/1-390 | Monca.augustus.g49867.t1/1-387	0.0479484	0.0542418	NADH dehydrogenase 1 beta subcomplex subunit	1	C:GO:0016020	[illegible]
Acrdi.XM_015916294.1/1-633 | Monca.augustus.g68766.t1/1-729	0.0700038	0.0542193	Thymidine cytosolic	6	F:GO:0005524; P:GO:0071897; P:GO:0009157; P:GO:0016310;	[illegible]
Acrdi.XM_015919939.1/1-810 | Monca.augustus.g33760.t1/1-810	0.0662809	0.0542032	dnaJ homolog subfamily C member 27	7	F:GO:0005525; C:GO:0005634; P:GO:0007264; F:GO:0003924;	[illegible]
Acrdi.XM_015902424.1/1-873 | Monca.augustus.g37587.t1/1-876	0.0898427	0.0541997	Eukaryotic translation initiation factor 3 subunit G	7	F:GO:0000166; F:GO:0003743; P:GO:0001731; C:GO:0016282;	[illegible]
Acrdi.XM_015892052.1/1-1230 | Monca.augustus.g8374.t1/1-1569	0.0651722	0.0541993	aldehyde dehydrogenase family 9 member A1-B-like	4	C:GO:0005737; F:GO:0019145; F:GO:0047105; P:GO:0055114	[illegible]
Acrdi.XM_015903067.1/1-621 | Monca.augustus.g56755.t1/1-2325	0.070307	0.0541433	transient receptor potential cation channel subfamily V member 4-like	3	F:GO:0005216; P:GO:0034220; C:GO:0016021	[illegible]
Acrdi.XM_015923889.1/1-2478 | Monca.adi2mcaRNA35621_R0/1-2151	0.0651491	0.0541301	chloride channel C-like	1	P:GO:0006811	[illegible]
Acrdi.XM_015897835.1/1-1269 | Monca.augustus.g49491.t1/1-1278	0.0869706	0.0541244	early growth response	1	F:GO:0005488	[illegible]
Acrdi.XM_015894325.1/1-582 | Monca.adi2mcaRNA40572_R3/1-474	0.614747	0.0541193	PREDICTED: uncharacterized protein LOC107329653			
Acrdi.XM_015916296.1/1-3531 | Monca.augustus.g70713.t1/1-5718	0.0768407	0.0541001	TATA-binding -associated factor 172	1	F:GO:0005524	[illegible]
Acrdi.XM_015905612.1/1-2412 | Monca.augustus.g65078.t1/1-2967	0.0614357	0.0540724	26S proteasome non-ATPase regulatory subunit 1	8	C:GO:0005634; P:GO:0043161; C:GO:0008540; P:GO:0050790;	[illegible]
Acrdi.XM_015910801.1/1-2265 | Monca.augustus.g48006.t1/1-2265	0.0477772	0.0540347	E3 ubiquitin- ligase TRIM71-like	1	F:GO:0046872	[illegible]
Acrdi.XM_015919431.1/1-573 | Monca.augustus.g26125.t1/1-798	1.61556	0.0539951	zinc finger and SCAN domain-containing 29-like			
Acrdi.XM_015918358.1/1-825 | Monca.augustus.g41663.t1/1-894	0.106169	0.0539926	Exosome complex component RRP4	13	P:GO:0034475; P:GO:0043928; P:GO:0000467; C:GO:0000177;	[illegible]
Acrdi.XM_015908419.1/1-2223 | Monca.adi2mcaRNA18414_R1/1-1845	0.67767	0.0539751	PREDICTED: uncharacterized protein LOC107342908 isoform X3			
Acrdi.XM_015921822.1/1-405 | Monca.augustus.g55981.t1/1-1026	0.099676	0.0539704	nuclear pore complex Nup155-like	3	P:GO:0006913; F:GO:0017056; C:GO:0005643	[illegible]
Acrdi.XM_015894403.1/1-558 | Monca.adi2mcaRNA40667_R0/1-948	0.707995	0.0539589	Ovarian otubain			
Acrdi.XM_015920793.1/1-2262 | Monca.adi2mcaRNA32115_R0/1-2109	0.0531339	0.0539587	Gamma-tubulin complex component 3	14	C:GO:0008275; C:GO:0000923; C:GO:0000922; F:GO:0005200;	[illegible]
Acrdi.XM_015921291.1/1-1353 | Monca.augustus.g64471.t1/1-1545	0.0662933	0.0539573	2,3-bisphosphoglycerate-independent phosphoglycerate mutase-like	4	C:GO:0005737; F:GO:0004619; F:GO:0030145; P:GO:0006007	[illegible]
Acrdi.XM_015898917.1/1-1347 | Monca.adi2mcaRNA8067_R3/1-1401	0.225275	0.0539555	PREDICTED: uncharacterized protein LOC107334045			
Acrdi.XM_015915270.1/1-1467 | Monca.augustus.g41285.t1/1-1482	0.0608257	0.0539182	phosphatase 1H-like	2	F:GO:0004722; P:GO:0006470	[illegible]
Acrdi.XM_015895719.1/1-1305 | Monca.augustus.g64085.t1/1-1302	0.044764	0.0538943	Splicing factor 45	2	F:GO:0097159; F:GO:1901363	[illegible]
Acrdi.XM_015897918.1/1-1524 | Monca.augustus.g23846.t1/1-2211	0.0497267	0.0538773	EF-hand domain-containing family member C2-like	1	F:GO:0005509	[illegible]
Acrdi.XM_015920896.1/1-375 | Monca.augustus.g30087.t1/1-1989	0.0576883	0.0538746	Lipase maturation factor 2	2	C:GO:0005783; C:GO:0044425	[illegible]
Acrdi.XM_015896107.1/1-951 | Monca.augustus.g63107.t1/1-951	0.0417428	0.0538633	potassium channel subfamily K member 9	4	C:GO:0005887; P:GO:0071805; P:GO:0030322; F:GO:0022841	[illegible]
Acrdi.XM_015923961.1/1-963 | Monca.adi2mcaRNA3566_R0/1-927	0.0936849	0.0538581	cell division cycle 2	4	P:GO:0051301; F:GO:0005524; F:GO:0004674; P:GO:0006468	[illegible]
Acrdi.XM_015908530.1/1-1437 | Monca.adi2mcaRNA18494_R0/1-1428	0.0789139	0.0538194	NADP-dependent malic mitochondrial	5	P:GO:0006108; F:GO:0046872; F:GO:0051287; F:GO:0004471;	[illegible]
Acrdi.XM_015923056.1/1-1461 | Monca.adi2mcaRNA34652_R1/1-1161	0.264113	0.053765	reverse transcriptase	2	F:GO:0003964; P:GO:0006278	[illegible]
Acrdi.XM_015923512.1/1-1713 | Monca.augustus.g47454.t1/1-1710	0.0689546	0.0537622	synaptotagmin-4-like isoform X2	8	P:GO:0048791; F:GO:0019905; P:GO:0006906; F:GO:0005509;	[illegible]
Acrdi.XM_015918342.1/1-1200 | Monca.augustus.g41662.t1/1-1191	0.0620358	0.0536952	PR domain zinc finger 12	11	F:GO:0003676; F:GO:0008171; P:GO:0016427; P:GO:0051574;	[illegible]
Acrdi.XM_015897179.1/1-2133 | Monca.adi2mcaRNA6034_R1/1-2280	0.255295	0.0536885	PREDICTED: uncharacterized protein LOC107332448			
Acrdi.XM_015913640.1/1-1257 | Monca.adi2mcaRNA24086_R2/1-1254	0.45954	0.0536834	RNA-directed DNA polymerase from mobile element jockey-like			
Acrdi.XM_015911906.1/1-1488 | Monca.augustus.g7927.t1/1-1491	0.0738586	0.0536814	High mobility group 20A	4	F:GO:0003677; C:GO:0005634; P:GO:0006351; P:GO:0006355	[illegible]
Acrdi.XM_015908847.1/1-474 | Monca.augustus.g26491.t1/1-477	0.0643972	0.0536637	U11 U12 small nuclear ribonucleo 25 kDa	1	C:GO:0044428	[illegible]
Acrdi.XM_015897372.1/1-2016 | Monca.augustus.g3798.t1/1-3735	0.0669328	0.0536493	flightless-1 homolog	3	P:GO:0051014; F:GO:0003779; P:GO:0030036	[illegible]
Acrdi.XM_015899856.1/1-1287 | Monca.augustus.g60334.t1/1-1296	0.0684282	0.0536211	MAPK MAK MRK overlapping kinase-like	3	F:GO:0005524; F:GO:0004672; P:GO:0006468	[illegible]
Acrdi.XM_015909545.1/1-2076 | Monca.augustus.g41987.t1/1-2286	0.0662751	0.0536054	kinesin KIF3B	7	F:GO:0000166; F:GO:0016887; F:GO:0003777; P:GO:0046530;	[illegible]
Acrdi.XM_015900146.1/1-969 | Monca.augustus.g59993.t1/1-1110	0.0695602	0.0535966	UV excision repair RAD23 homolog A isoform X2	1	P:GO:0009987	[illegible]
Acrdi.XM_015897072.1/1-2889 | Monca.augustus.g38098.t1/1-3030	0.0710186	0.0535944	E3 ubiquitin- ligase MIB1-like			
Acrdi.XM_015909655.1/1-3651 | Monca.adi2mcaRNA19752_R0/1-3588	0.0595508	0.0535853	vigilin isoform X2	1	F:GO:0003723	[illegible]
Acrdi.XM_015922513.1/1-1083 | Monca.augustus.g33871.t1/1-1083	0.087581	0.0535344	pyruvate dehydrogenase E1 component subunit mitochondrial	2	F:GO:0003824; P:GO:0008152	[illegible]
Acrdi.XM_015919118.1/1-903 | Monca.augustus.g35182.t1/1-978	0.0609668	0.053516	nuclear transcription factor Y subunit beta-like	1	F:GO:0005488	[illegible]
Acrdi.XM_015901360.1/1-1074 | Monca.adi2mcaRNA10632_R0/1-1017	0.384214	0.0535122	Retrovirus-related Pol poly from transposon 412			
Acrdi.XM_015912636.1/1-819 | Monca.augustus.g58010.t1/1-1755	0.0470508	0.0535061	mesoderm induction early response 1 isoform X1	2	F:GO:0003677; C:GO:0005634	[illegible]
Acrdi.XM_015897487.1/1-375 | Monca.augustus.g35239.t1/1-762	0.0557305	0.0534685	cytoplasmic phosphatidylinositol transfer 1 isoform X2	2	P:GO:0006810; C:GO:0005622	[illegible]
Acrdi.XM_015901831.1/1-681 | Monca.augustus.g56978.t1/1-1791	0.0459337	0.0534517	Splicing factor 1	3	F:GO:0008270; F:GO:0045131; P:GO:0000398	[illegible]
Acrdi.XM_015901361.1/1-1485 | Monca.adi2mcaRNA10633_R0/1-1422	0.359812	0.0534423	Retrovirus-related Pol poly from transposon 412			
Acrdi.XM_015908641.1/1-1314 | Monca.augustus.g53882.t1/1-1323	0.0780039	0.0533894	adenosine monophosphate- transferase FICD homolog	4	F:GO:0005524; C:GO:0016021; F:GO:0070733; P:GO:0018117	[illegible]
Acrdi.XM_015895397.1/1-372 | Monca.augustus.g24980.t1/1-852	0.0256806	0.0533644	Transmembrane 41B	1	C:GO:0016021	[illegible]
Acrdi.XM_015919315.1/1-2991 | Monca.augustus.g5206.t1/1-2883	0.0787649	0.053335	alanine--tRNA cytoplasmic	9	F:GO:0005524; F:GO:0008270; F:GO:0004813; P:GO:0006419;	[illegible]
Acrdi.XM_015892674.1/1-2295 | Monca.augustus.g58944.t1/1-2550	0.0678625	0.0533302	urease subunit alpha-like	4	P:GO:0043419; C:GO:0005737; F:GO:0016151; F:GO:0009039	[illegible]
Acrdi.XM_015908025.1/1-2136 | Monca.augustus.g5042.t1/1-2136	0.0640281	0.0533244	PC3-like endoprotease variant B	2	F:GO:0004252; P:GO:0016485	[illegible]
Acrdi.XM_015894901.1/1-615 | Monca.augustus.g45887.t1/1-558	0.613475	0.0532815	THAP domain-containing 2			
Acrdi.XM_015899696.1/1-747 | Monca.augustus.g33789.t1/1-747	0.0650391	0.0532593	probable 39S ribosomal mitochondrial isoform X3	4	F:GO:0003735; C:GO:0005739; C:GO:0005840; P:GO:0006412	[illegible]
Acrdi.XM_015922290.1/1-6966 | Monca.augustus.g22301.t1/1-7614	0.068188	0.0532519	probable ubiquitin carboxyl-terminal hydrolase FAF-X isoform X1	5	C:GO:0005737; P:GO:0006508; P:GO:0044267; P:GO:0016477;	[illegible]
Acrdi.XM_015911275.1/1-2265 | Monca.augustus.g18337.t1/1-2679	0.0589177	0.0532201	cGMP-dependent 3 ,5 -cyclic phosphodiesterase	1	F:GO:0016787	[illegible]
Acrdi.XM_015906419.1/1-1644 | Monca.augustus.g237.t1/1-1500	0.0846717	0.0531904	Negative elongation factor A	2	P:GO:0034244; C:GO:0032021	[illegible]
Acrdi.XM_015921709.1/1-726 | Monca.augustus.g47565.t1/1-2916	0.0814235	0.053187	exocyst complex component			
Acrdi.XM_015907543.1/1-552 | Monca.augustus.g4915.t1/1-744	0.242967	0.0531539	P2X purinoceptor 7-like	4	F:GO:0004931; C:GO:0005639; F:GO:0001614; P:GO:0050896	[illegible]
Acrdi.XM_015897554.1/1-2835 | Monca.adi2mcaRNA584_R0/1-2442	0.549545	0.0531439	RNA-directed DNA polymerase from mobile element jockey			
Acrdi.XM_015911374.1/1-774 | Monca.augustus.g35259.t1/1-774	0.0910687	0.053133	probable tRNA(His) guanylyltransferase	11	F:GO:0000287; F:GO:0005525; F:GO:0005524; F:GO:0008193;	[illegible]
Acrdi.XM_015924950.1/1-2466 | Monca.augustus.g4520.t1/1-2460	0.0805511	0.0530774	Neuroendocrine convertase 1	2	F:GO:0004252; P:GO:0016485	[illegible]
Acrdi.XM_015925429.1/1-747 | Monca.augustus.g23863.t1/1-732	0.045777	0.0530573	UBX domain-containing 7			
Acrdi.XM_015912168.1/1-2031 | Monca.augustus.g55527.t1/1-1851	0.061559	0.0530228	heterogeneous nuclear ribonucleo R	2	F:GO:0000166; F:GO:0003676	[illegible]
Acrdi.XM_015918393.1/1-2358 | Monca.augustus.g22396.t1/1-2277	0.0731645	0.0530178	serine threonine- kinase SIK2 isoform X2	7	C:GO:0005634; F:GO:0005524; C:GO:0005737; P:GO:0032870;	[illegible]
Acrdi.XM_015917754.1/1-1899 | Monca.augustus.g1573.t1/1-1893	0.050842	0.0530095	polypeptide N-acetylgalactosaminyltransferase 5 isoform X2	5	F:GO:0016757; C:GO:0016021; P:GO:0006486; C:GO:0000139;	[illegible]
Acrdi.XM_015913779.1/1-762 | Monca.augustus.g21665.t1/1-759	0.0703753	0.0530038	rab 3	3	F:GO:0005525; P:GO:0007264; C:GO:0005622	[illegible]
Acrdi.XM_015910268.1/1-7941 | Monca.augustus.g47618.t1/1-7977	0.0815044	0.0529887	neurofibromin isoform X4			
Acrdi.XM_015906778.1/1-1593 | Monca.augustus.g46210.t1/1-4836	0.249074	0.0529677	PREDICTED: uncharacterized protein LOC107341376			
Acrdi.XM_015921826.1/1-3768 | Monca.augustus.g33305.t1/1-3510	0.0551023	0.0529234	Intron-binding aquarius	2	C:GO:0005681; P:GO:0000398	[illegible]
Acrdi.XM_015922161.1/1-1254 | Monca.augustus.g196.t1/1-1254	0.0562911	0.0528855	Acetyl- cytosolic	3	F:GO:0003985; P:GO:0006635; C:GO:0005739	[illegible]
Acrdi.XM_015906023.1/1-402 | Monca.augustus.g41388.t1/1-381	0.0620302	0.0528525	programmed cell death 5	4	C:GO:0005634; F:GO:0003677; C:GO:0005829; P:GO:0012501	[illegible]
Acrdi.XM_015910866.1/1-2526 | Monca.adi2mcaRNA21067_R0/1-2481	0.0721572	0.0528425	mutS homolog 4	7	F:GO:0005524; P:GO:0007131; C:GO:0032300; C:GO:0000228;	[illegible]
Acrdi.XM_015899248.1/1-555 | Monca.augustus.g1496.t1/1-555	0.0589506	0.0527977	60S ribosomal L17	3	F:GO:0003735; C:GO:0015934; P:GO:0006412	[illegible]
Acrdi.XM_015915154.1/1-876 | Monca.augustus.g35526.t1/1-876	0.057276	0.0527125	transcription factor SOX-4-like	2	P:GO:0061386; P:GO:0045879	[illegible]
Acrdi.XM_015918172.1/1-1713 | Monca.adi2mcaRNA29187_R0/1-1728	0.0703218	0.0527029	Aspartate--tRNA cytoplasmic	8	F:GO:0003676; F:GO:0005524; C:GO:0070062; F:GO:0005515;	[illegible]
Acrdi.XM_015900588.1/1-1101 | Monca.adi2mcaRNA9828_R0/1-1155	0.0543511	0.0527001	katanin p60 ATPase-containing subunit A-like 1	7	F:GO:0000166; C:GO:0005634; C:GO:0005815; C:GO:0005819;	[illegible]
Acrdi.XM_015920392.1/1-618 | Monca.augustus.g47499.t1/1-1035	0.0546507	0.0526932	DNA-directed RNA polymerases I and III subunit RPAC1	8	F:GO:0001056; F:GO:0003677; C:GO:0005736; F:GO:0001054;	[illegible]
Acrdi.XM_015921586.1/1-1188 | Monca.augustus.g2830.t1/1-2403	0.0851175	0.0526818	Homeobox PKNOX2	3	F:GO:0003677; C:GO:0005634; P:GO:0006355	[illegible]
Acrdi.XM_015913858.1/1-2313 | Monca.adi2mcaRNA59_R0/1-1959	0.0890719	0.0526551	serine threonine- kinase TAO1	1	F:GO:0004672	[illegible]
Acrdi.XM_015899920.1/1-1590 | Monca.adi2mcaRNA9026_R0/1-1452	0.0666729	0.0526536	vesicular glutamate transporter 2-like	4	P:GO:0055085; F:GO:0022857; C:GO:0016021; P:GO:0006820	[illegible]
Acrdi.XM_015914711.1/1-5958 | Monca.adi2mcaRNA25302_R0/1-5538	0.449243	0.0526354	Gypsy retrotransposon integrase 1			
Acrdi.XM_015914260.1/1-1086 | Monca.augustus.g483.t1/1-1254	0.0679131	0.0526285	ankyrin repeat			
Acrdi.XM_015914892.1/1-477 | Monca.augustus.g11326.t1/1-735	0.629256	0.0525936	nuclease HARBI1			
Acrdi.XM_015919620.1/1-1944 | Monca.adi2mcaRNA30821_R0/1-1920	0.488572	0.0525903	RNA-directed DNA polymerase from mobile element jockey	5	F:GO:0003677; P:GO:0090305; C:GO:0005634; F:GO:0008270;	[illegible]
Acrdi.XM_015912945.1/1-1215 | Monca.augustus.g15084.t1/1-1221	0.0701214	0.0525739	leucine-rich repeat-containing 48 isoform X1	1	C:GO:0005737	[illegible]
Acrdi.XM_015897664.1/1-1641 | Monca.adi2mcaRNA6682_R0/1-1584	0.0679062	0.0525734	G- -signaling modulator 2-like	4	F:GO:0005092; P:GO:0050790; F:GO:0030695; P:GO:0008277	[illegible]
Acrdi.XM_015906586.1/1-630 | Monca.augustus.g31634.t1/1-489	0.605101	0.0525728	PREDICTED: uncharacterized protein LOC107341167			
Acrdi.XM_015924249.1/1-3084 | Monca.adi2mcaRNA3618_R0/1-3060	0.0690779	0.052543	1-phosphatidylinositol 4,5-bisphosphate phosphodiesterase gamma-1-	5	F:GO:0004435; P:GO:0016042; F:GO:0004871; C:GO:0005622;	[illegible]
Acrdi.XM_015908863.1/1-549 | Monca.augustus.g12211.t1/1-498	0.506071	0.0525406	PREDICTED: uncharacterized protein LOC107343295			
Acrdi.XM_015899078.1/1-417 | Monca.augustus.g6443.t1/1-600	0.0614561	0.0525397	N-terminal glutamine amidohydrolase-like	1	F:GO:0016811	[illegible]
Acrdi.XM_015903840.1/1-633 | Monca.augustus.g64138.t1/1-597	0.0563232	0.0524682	EF-hand calcium-binding domain-containing 9-like	1	F:GO:0005509	[illegible]
Acrdi.XM_015923296.1/1-561 | Monca.augustus.g35014.t1/1-561	0.072313	0.0524679	COMM domain-containing 1-like	6	P:GO:2000009; C:GO:0005768; P:GO:0031398; P:GO:0032434;	[illegible]
Acrdi.XM_015898410.1/1-1737 | Monca.augustus.g58548.t1/1-1737	0.0665143	0.0524267	Negative elongation factor B	6	F:GO:0005515; C:GO:0032021; P:GO:0034244; C:GO:0005737;	[illegible]
Acrdi.XM_015919574.1/1-873 | Monca.augustus.g13432.t1/1-906	0.0696109	0.0524093	Coiled-coil domain-containing 166			
Acrdi.XM_015922326.1/1-1179 | Monca.augustus.g7331.t1/1-1248	0.0733118	0.0523928	shaker-related potassium channel tsha2-like	5	P:GO:0071805; C:GO:0008076; F:GO:0005251; P:GO:0034765;	[illegible]
Acrdi.XM_015907218.1/1-780 | Monca.adi2mcaRNA17068_R0/1-561	0.0546849	0.052387	vacuolar membrane	7	P:GO:0007040; C:GO:0005768; P:GO:0035542; P:GO:0007032;	[illegible]
Acrdi.XM_015924585.1/1-834 | Monca.augustus.g2660.t1/1-837	0.406567	0.0523678	dimethylsulfonioproprionate lyase 7-like	1	F:GO:0016829	[illegible]
Acrdi.XM_015915331.1/1-3591 | Monca.adi2mcaRNA2605_R0/1-3558	0.0683194	0.0523547	probable phosphorylase b kinase regulatory subunit alpha isoform X1			
Acrdi.XM_015899407.1/1-2838 | Monca.augustus.g37216.t1/1-3024	0.0736869	0.0523162	sarcoplasmic endoplasmic reticulum calcium ATPase 3 isoform X2			
Acrdi.XM_015925375.1/1-462 | Monca.augustus.g37576.t1/1-807	0.550322	0.0523119	Coagulation factor V			
Acrdi.XM_015908014.1/1-897 | Monca.augustus.g6866.t1/1-900	0.0689818	0.0522956	5-hydroxytryptamine receptor-like	6	P:GO:0071482; C:GO:0005887; P:GO:0007186; P:GO:0009584;	[illegible]
Acrdi.XM_015916578.1/1-2394 | Monca.adi2mcaRNA27395_R1/1-1644	0.414378	0.0522839	PREDICTED: uncharacterized protein LOC107350351			
Acrdi.XM_015893837.1/1-840 | Monca.augustus.g53594.t1/1-2175	0.0601295	0.0522604	CSC1 ERD4	3	C:GO:0043231; C:GO:0016020; C:GO:0044444	[illegible]
Acrdi.XM_015892150.1/1-333 | Monca.augustus.g43941.t1/1-375	0.0702613	0.0522505	50S ribosomal L20	6	F:GO:0003735; C:GO:0016020; C:GO:0005761; F:GO:0019843;	[illegible]
Acrdi.XM_015917976.1/1-549 | Monca.augustus.g45174.t1/1-2568	0.0493433	0.0522254	Coiled-coil domain-containing R3HCC1L			
Acrdi.XM_015895856.1/1-696 | Monca.augustus.g41978.t1/1-591	0.0766651	0.0521966	synaptobrevin homolog YKT6-like	6	P:GO:0006906; C:GO:0016021; F:GO:0005484; C:GO:0031201;	[illegible]
Acrdi.XM_015902792.1/1-1179 | Monca.adi2mcaRNA12162_R1/1-1080	0.0827875	0.0521768	Bifunctional purine biosynthesis PURH	1	F:GO:0003824	[illegible]
Acrdi.XM_015900001.1/1-483 | Monca.augustus.g14107.t1/1-483	0.0331765	0.0521763	PFAM domain(EF-Hand)-containing	1	F:GO:0005509	[illegible]
Acrdi.XM_015892520.1/1-2595 | Monca.adi2mcaRNA38467_R5/1-2193	0.261984	0.0521695	reverse transcriptase			
Acrdi.XM_015922844.1/1-504 | Monca.augustus.g1403.t1/1-504	0.0839704	0.0521364	39S ribosomal mitochondrial	6	F:GO:0003735; F:GO:0003729; C:GO:0005761; P:GO:0070124;	[illegible]
Acrdi.XM_015910385.1/1-1494 | Monca.augustus.g51105.t1/1-1941	0.0710774	0.0520708	GTP-binding 1 isoform X3	8	C:GO:0000177; F:GO:0005525; P:GO:0061014; F:GO:0003746;	[illegible]
Acrdi.XM_015925293.1/1-1530 | Monca.augustus.g53769.t1/1-6420	0.0697257	0.0520338	Polyamine-modulated factor 1-binding 1			
Acrdi.XM_015912678.1/1-846 | Monca.augustus.g5182.t1/1-759	0.0577577	0.0520176	LIM homeobox Awh-like	1	F:GO:0046872	[illegible]
Acrdi.XM_015921018.1/1-6036 | Monca.augustus.g13202.t1/1-6504	0.0600896	0.0520133	rootletin-like isoform X3			
Acrdi.XM_015898366.1/1-357 | Monca.augustus.g58552.t1/1-1620	0.055461	0.0520075	Transmembrane channel 3			
Acrdi.XM_015919890.1/1-738 | Monca.augustus.g33623.t1/1-735	0.0871261	0.0519995	Transcription factor SOX-2	1	P:GO:0006355	[illegible]
Acrdi.XM_015921415.1/1-1116 | Monca.adi2mcaRNA3327_R0/1-1152	0.0821776	0.0519744	macrophage erythroblast attacher-like	4	P:GO:0065007; C:GO:0005856; C:GO:0044446; P:GO:0009987	[illegible]
Acrdi.XM_015907332.1/1-654 | Monca.augustus.g54011.t1/1-1932	0.294887	0.0519603	Choline O-acetyltransferase	3	F:GO:0016746; C:GO:0016020; C:GO:0016021	[illegible]
Acrdi.XM_015896721.1/1-870 | Monca.augustus.g11232.t1/1-954	0.0621542	0.0519514	mitochondrial substrate carrier family ucpB-like	4	F:GO:0003735; C:GO:0016020; P:GO:0006810; P:GO:0006412	[illegible]
Acrdi.XM_015915516.1/1-948 | Monca.augustus.g53737.t1/1-1137	0.0576123	0.0519465	RIB43A-like with coiled-coils 2			
Acrdi.XM_015906548.1/1-642 | Monca.augustus.g38393.t1/1-636	0.0756772	0.051942	Contactin-associated -like 2	1	C:GO:0016020	[illegible]
Acrdi.XM_015893081.1/1-1158 | Monca.augustus.g55289.t1/1-1509	0.357737	0.0519361	THAP domain-containing 3			
Acrdi.XM_015900586.1/1-1101 | Monca.adi2mcaRNA9821_R0/1-960	0.0633893	0.051933	transmembrane 181-like	2	C:GO:0016020; C:GO:0016021	[illegible]
Acrdi.XM_015913021.1/1-1011 | Monca.adi2mcaRNA23414_R0/1-1125	0.630029	0.0519319	Mitochondrial inner membrane protease subunit 1			
Acrdi.XM_015909607.1/1-897 | Monca.augustus.g43366.t1/1-1146	0.252007	0.0519319	trihelix transcription factor GTL1-like	2	P:GO:0006915; C:GO:0005622	[illegible]
Acrdi.XM_015918209.1/1-1095 | Monca.augustus.g53125.t1/1-1113	0.0798262	0.0519014	paired box Pax-3-B-like	6	F:GO:0003677; C:GO:0005634; P:GO:0007275; P:GO:0006351;	[illegible]
Acrdi.XM_015902279.1/1-1092 | Monca.augustus.g35112.t1/1-1092	0.0603949	0.0518855	nuclear inhibitor of phosphatase 1 isoform X1	11	P:GO:0008283; F:GO:0004864; P:GO:0016070; P:GO:0060255;	[illegible]
Acrdi.XM_015893244.1/1-906 | Monca.augustus.g55947.t1/1-906	0.807594	0.0518329	PREDICTED: uncharacterized protein LOC107328522			
Acrdi.XM_015901369.1/1-768 | Monca.augustus.g9018.t1/1-768	0.488442	0.0518223	RNA-directed DNA polymerase from transposon BS	2	F:GO:0003964; P:GO:0006278	[illegible]
Acrdi.XM_015903213.1/1-11448 | Monca.augustus.g51698.t1/1-13980	0.0747915	0.0517545	dynein heavy chain axonemal	5	F:GO:0016887; F:GO:0005524; F:GO:0003777; C:GO:0030286;	[illegible]
Acrdi.XM_015917957.1/1-795 | Monca.augustus.g61952.t1/1-1086	0.0623303	0.0516972	probable phosphatase 2C	3	F:GO:0004722; F:GO:0046872; P:GO:0006470	[illegible]
Acrdi.XM_015921974.1/1-723 | Monca.augustus.g23748.t1/1-723	0.0695884	0.0516456	Dihydropteridine reductase	4	C:GO:0005737; F:GO:0016491; P:GO:0055114; C:GO:0043227	[illegible]
Acrdi.XM_015907242.1/1-990 | Monca.augustus.g6147.t1/1-990	0.0768771	0.0516405	lipid droplet-associated hydrolase-like	2	C:GO:0005811; P:GO:0019915	[illegible]
Acrdi.XM_015906328.1/1-864 | Monca.adi2mcaRNA16127_R7/1-816	0.673646	0.0515788	Pro-Pol poly			
Acrdi.XM_015917016.1/1-885 | Monca.augustus.g67188.t1/1-768	0.0538445	0.0515432	Dual adapter for phosphotyrosine and 3-phosphotyrosine and 3-phosph	3	F:GO:0005547; F:GO:0043325; C:GO:0005886	[illegible]
Acrdi.XM_015918158.1/1-795 | Monca.augustus.g53132.t1/1-789	0.0635119	0.0515185	Methyl- -binding domain 2	2	F:GO:0003677; P:GO:0045892	[illegible]
Acrdi.XM_015924817.1/1-585 | Monca.augustus.g67355.t1/1-798	0.0467292	0.051508	PREDICTED: uncharacterized protein LOC107358193			
Acrdi.XM_015907683.1/1-879 | Monca.adi2mcaRNA17570_R2/1-876	0.656193	0.0514985	RNA-directed DNA polymerase from transposon X-element			
Acrdi.XM_015909434.1/1-2034 | Monca.augustus.g45715.t1/1-3306	0.0708698	0.0514949	NAD(P) mitochondrial	7	F:GO:0050661; P:GO:0015992; F:GO:0051287; C:GO:0016021;	[illegible]
Acrdi.XM_015898667.1/1-699 | Monca.augustus.g34610.t1/1-891	0.081209	0.0514807	pleckstrin homology domain-containing family F member 2	5	C:GO:0031901; F:GO:0046872; P:GO:0015031; C:GO:0005783;	[illegible]
Acrdi.XM_015922146.1/1-2109 | Monca.augustus.g26477.t1/1-4305	0.0793168	0.0514611	AP-3 complex subunit delta-1	17	P:GO:0061088; F:GO:0008565; P:GO:0048007; P:GO:0006886;	[illegible]
Acrdi.XM_015896175.1/1-1287 | Monca.adi2mcaRNA5049_R0/1-1281	0.0548517	0.0514603	cleavage and polyadenylation specificity factor subunit 2-like	3	C:GO:0005847; P:GO:0006378; P:GO:0006379	[illegible]
Acrdi.XM_015916529.1/1-1755 | Monca.augustus.g39001.t1/1-1767	0.0765317	0.0514555	hu-li tai shao-like isoform X16			
Acrdi.XM_015916629.1/1-483 | Monca.augustus.g40316.t1/1-1671	0.0482289	0.0514506	drebrin B	2	F:GO:0003779; C:GO:0005622	[illegible]
Acrdi.XM_015915579.1/1-2235 | Monca.adi2mcaRNA26279_R0/1-2178	0.256451	0.0514377	amine oxidase	1	F:GO:0003824	[illegible]
Acrdi.XM_015898257.1/1-1218 | Monca.augustus.g39438.t1/1-1224	0.099835	0.0514316	Transcriptional coactivator YAP1	1	P:GO:0048513	[illegible]
Acrdi.XM_015892952.1/1-528 | Monca.augustus.g24465.t1/1-528	0.0656498	0.0514145	MORN repeat-containing 5			
Acrdi.XM_015907358.1/1-750 | Monca.augustus.g63474.t1/1-750	0.0646339	0.0514064	outer dense fiber 3-like	3	P:GO:0008150; C:GO:0005737; F:GO:0003674	[illegible]
Acrdi.XM_015908561.1/1-510 | Monca.augustus.g930.t1/1-528	1.38551	0.0513672	nuclease HARBI1			
Acrdi.XM_015894762.1/1-1800 | Monca.augustus.g23280.t1/1-1800	0.0742511	0.0513646	Beta-glucuronidase	3	P:GO:0005975; C:GO:0043231; F:GO:0004566	[illegible]
Acrdi.XM_015913492.1/1-504 | Monca.augustus.g60181.t1/1-504	0.0785117	0.0513619	PREDICTED: uncharacterized protein C16orf52 homolog A-like	1	C:GO:0016021	[illegible]
Acrdi.XM_015903936.1/1-1104 | Monca.adi2mcaRNA13417_R5/1-1056	0.936741	0.0513545	Transposon Tf2-6 poly			
Acrdi.XM_015893175.1/1-963 | Monca.adi2mcaRNA39229_R0/1-963	0.491704	0.0513396	PREDICTED: uncharacterized protein K02A2.6-like			
Acrdi.XM_015919741.1/1-3828 | Monca.augustus.g17436.t1/1-4200	0.0632247	0.0513359	DNA-directed RNA polymerase III subunit RPC1	4	F:GO:0001056; F:GO:0003677; P:GO:0006383; C:GO:0005666	[illegible]
Acrdi.XM_015921778.1/1-1089 | Monca.adi2mcaRNA33155_R2/1-1050	0.293058	0.0513131	THAP domain-containing 1			
Acrdi.XM_015910016.1/1-1815 | Monca.adi2mcaRNA20144_R0/1-1179	0.085208	0.0512991	phosphoglucomutase-2	3	P:GO:0005975; F:GO:0016868; C:GO:0005829	[illegible]
Acrdi.XM_015897365.1/1-1875 | Monca.adi2mcaRNA6287_R0/1-1875	0.0752558	0.0512817	coiled-coil domain-containing 40-like			
Acrdi.XM_015894539.1/1-465 | Monca.adi2mcaRNA26008_R0/1-456	0.0375607	0.0512156	EF-hand_1 domain-containing	50	F:GO:0015276; P:GO:0022400; P:GO:0031954; C:GO:0034704;	[illegible]
Acrdi.XM_015895246.1/1-549 | Monca.augustus.g4765.t1/1-549	0.0561271	0.0512033	ras-related Rap-2a	16	P:GO:0035690; C:GO:0005886; P:GO:0048814; P:GO:0046328;	[illegible]
Acrdi.XM_015920184.1/1-1005 | Monca.augustus.g50167.t1/1-2112	0.991289	0.0511725	PREDICTED: uncharacterized protein LOC107353797			
Acrdi.XM_015899298.1/1-438 | Monca.augustus.g44801.t1/1-822	0.0694388	0.0511529	DNA-directed RNA polymerase II subunit RPB3	16	C:GO:0015630; P:GO:0006370; P:GO:0006283; P:GO:0008543;	[illegible]
Acrdi.XM_015891994.1/1-3801 | Monca.adi2mcaRNA37884_R1/1-3441	0.0590745	0.0510697	serine threonine- kinase MRCK alpha isoform X2			
Acrdi.XM_015913803.1/1-1161 | Monca.adi2mcaRNA24276_R0/1-1128	0.0689171	0.051043	isocitrate dehydrogenase [NAD] subunit mitochondrial-like	5	F:GO:0000287; F:GO:0004449; F:GO:0051287; C:GO:0005739;	[illegible]
Acrdi.XM_015907597.1/1-2802 | Monca.augustus.g50781.t1/1-2799	0.0592777	0.0510114	Dual serine threonine and tyrosine kinase	7	F:GO:0005524; P:GO:0007275; C:GO:0005829; C:GO:0098590;	[illegible]
Acrdi.XM_015914022.1/1-1290 | Monca.augustus.g14935.t1/1-1290	0.0776014	0.0510005	Serine threonine- kinase NIM1	8	F:GO:0000287; C:GO:0005634; F:GO:0005524; C:GO:0005737;	[illegible]
Acrdi.XM_015895379.1/1-1185 | Monca.augustus.g25994.t1/1-1281	0.0757869	0.0509934	26S proteasome non-ATPase regulatory subunit 11	5	P:GO:0048863; C:GO:0005737; C:GO:0022624; P:GO:0043248;	[illegible]
Acrdi.XM_015912354.1/1-741 | Monca.augustus.g62227.t1/1-831	0.431298	0.0509851	mitogen-activated kinase kinase kinase 14-like			
Acrdi.XM_015905565.1/1-768 | Monca.adi2mcaRNA15196_R1/1-768	0.283512	0.0509824	RNA-directed DNA polymerase from mobile element jockey-like			
Acrdi.XM_015918356.1/1-1932 | Monca.augustus.g41666.t1/1-1821	0.0636657	0.0509719	kinesin-associated 3 isoform X2	3	F:GO:0005515; C:GO:0016939; P:GO:0007017	[illegible]
Acrdi.XM_015904537.1/1-342 | Monca.augustus.g68202.t1/1-342	0.0725612	0.0509704	ATP synthase subunit mitochondrial	3	C:GO:0000276; F:GO:0015078; P:GO:0015986	[illegible]
Acrdi.XM_015913670.1/1-1017 | Monca.augustus.g30503.t1/1-1017	0.0693758	0.0509554	melatonin receptor type 1C-like	4	C:GO:0005887; P:GO:0007218; P:GO:0007166; F:GO:0004983	[illegible]
Acrdi.XM_015915134.1/1-927 | Monca.augustus.g51871.t1/1-885	0.676816	0.0509504	PREDICTED: uncharacterized protein LOC107349038			
Acrdi.XM_015918153.1/1-1581 | Monca.adi2mcaRNA29166_R2/1-1479	0.177976	0.0508772	RNA-directed DNA polymerase from mobile element jockey-like			
Acrdi.XM_015901069.1/1-1119 | Monca.adi2mcaRNA10307_R0/1-1119	0.0670618	0.0508742	Serine mitochondrial	21	C:GO:0015630; F:GO:0030170; F:GO:0042802; P:GO:0008284;	[illegible]
Acrdi.XM_015893478.1/1-1104 | Monca.augustus.g66993.t1/1-1119	0.0434361	0.0508439	TNF receptor-associated factor 4	1	F:GO:0008270	[illegible]
Acrdi.XM_015917002.1/1-924 | Monca.augustus.g48286.t1/1-924	0.0633565	0.0508099	UPF0602 C4orf47 homolog	1	C:GO:0005813	[illegible]
Acrdi.XM_015922795.1/1-1302 | Monca.adi2mcaRNA36621_R2/1-1617	0.0516868	0.0507279	vasa	4	F:GO:0003676; F:GO:0005524; F:GO:0008270; F:GO:0004386	[illegible]
Acrdi.XM_015901141.1/1-762 | Monca.augustus.g1878.t1/1-762	0.0691775	0.0507156	DNA damage-regulated autophagy modulator 1	2	C:GO:0016020; C:GO:0005764	[illegible]
Acrdi.XM_015899503.1/1-663 | Monca.augustus.g37211.t1/1-657	0.0725589	0.0507057	GTP-binding nuclear Ran	7	F:GO:0005525; C:GO:0005634; P:GO:0000054; C:GO:0005737;	[illegible]
Acrdi.XM_015916599.1/1-1674 | Monca.augustus.g22963.t1/1-1659	0.072495	0.0506703	beta-catenin 1	1	C:GO:0005681	[illegible]
Acrdi.XM_015922756.1/1-1164 | Monca.augustus.g69381.t1/1-1236	0.420247	0.0506625	PREDICTED: uncharacterized protein LOC107356115			
Acrdi.XM_015911506.1/1-1086 | Monca.augustus.g45455.t1/1-2964	0.0742414	0.0506621	serine threonine- kinase PLK4-like	15	F:GO:0042802; C:GO:0032154; P:GO:0007099; P:GO:0046601;	[illegible]
Acrdi.XM_015908291.1/1-1866 | Monca.augustus.g23848.t1/1-2685	0.04426	0.0506535	Mitogen-activated kinase kinase kinase	1	C:GO:0016021	[illegible]
Acrdi.XM_015910986.1/1-1092 | Monca.augustus.g63044.t1/1-1077	0.0637821	0.0506514	choline-phosphate cytidylyltransferase A	1	F:GO:0003824	[illegible]
Acrdi.XM_015898511.1/1-498 | Monca.augustus.g3329.t1/1-699	0.098518	0.0506474	molybdopterin synthase catalytic subunit-like	5	C:GO:0005829; C:GO:0030366; P:GO:0006777; P:GO:0032324;	[illegible]
Acrdi.XM_015921196.1/1-738 | Monca.augustus.g39898.t1/1-708	0.28703	0.050643	inositol 1,4,5-trisphosphate receptor type 3-like			
Acrdi.XM_015905704.1/1-1944 | Monca.adi2mcaRNA33157_R3/1-2025	0.205926	0.0505795	RNA-directed DNA polymerase from mobile element jockey-like			
Acrdi.XM_015897236.1/1-2118 | Monca.adi2mcaRNA716_R0/1-2013	0.0600361	0.0505572	Rho guanine nucleotide exchange factor 7	8	P:GO:0035023; F:GO:0005085; F:GO:0005089; C:GO:0042995;	[illegible]
Acrdi.XM_015903905.1/1-1026 | Monca.augustus.g8849.t1/1-945	0.402514	0.0504941	TNF receptor-associated factor 5-like	7	F:GO:0003677; F:GO:0004842; F:GO:0008270; F:GO:0046872;	[illegible]
Acrdi.XM_015898064.1/1-417 | Monca.augustus.g29247.t1/1-984	0.0621497	0.0504743	zinc finger 330 homolog	2	C:GO:0005634; F:GO:0008270	[illegible]
Acrdi.XM_015923948.1/1-4752 | Monca.augustus.g62275.t1/1-4899	0.0606265	0.0504729	transcription elongation factor SPT6	1	F:GO:0003676	[illegible]
Acrdi.XM_015897489.1/1-543 | Monca.augustus.g35235.t1/1-645	0.0549428	0.0504498	Mediator of RNA polymerase II transcription subunit 7	6	C:GO:0005667; P:GO:0019827; F:GO:0003713; C:GO:0016592;	[illegible]
Acrdi.XM_015898314.1/1-696 | Monca.augustus.g20927.t1/1-705	0.0812197	0.0504441	SPRY domain-containing SOCS box 3-like	2	P:GO:0035556; C:GO:0005622	[illegible]
Acrdi.XM_015906215.1/1-396 | Monca.adi2mcaRNA1598_R7/1-387	0.968462	0.0504205	PREDICTED: uncharacterized protein LOC107340822	8	F:GO:0000166; F:GO:0003677; P:GO:0006281; F:GO:0005524;	[illegible]
Acrdi.XM_015915653.1/1-1071 | Monca.augustus.g15821.t1/1-1068	0.0475088	0.0504051	G- coupled receptor 26	3	F:GO:0004930; P:GO:0007186; C:GO:0016021	[illegible]
Acrdi.XM_015917414.1/1-2412 | Monca.augustus.g66920.t1/1-2412	0.0675413	0.0503978	sodium calcium exchanger 1-like isoform X1	5	P:GO:0007154; P:GO:0006816; C:GO:0016021; P:GO:0035725;	[illegible]
Acrdi.XM_015925317.1/1-2661 | Monca.adi2mcaRNA37261_R1/1-2727	0.158772	0.0503572	Retrovirus-related Pol poly from transposon			
Acrdi.XM_015923025.1/1-1125 | Monca.augustus.g3821.t1/1-1125	0.435383	0.0503307	THAP domain-containing 10	1	F:GO:0003676	[illegible]
Acrdi.XM_015924676.1/1-1338 | Monca.adi2mcaRNA36520_R0/1-1269	0.684589	0.0503085	piggyBac transposable element-derived 4-like			
Acrdi.XM_015893272.1/1-1686 | Monca.adi2mcaRNA39343_R0/1-1887	0.446293	0.050306	PREDICTED: uncharacterized protein LOC107328549			
Acrdi.XM_015897831.1/1-1719 | Monca.augustus.g19247.t1/1-1719	0.0558631	0.0502983	kelch 17	3	F:GO:0004842; C:GO:0031463; P:GO:0016567	[illegible]
Acrdi.XM_015894911.1/1-486 | Monca.adi2mcaRNA41263_R2/1-372	0.233158	0.05029	PREDICTED: uncharacterized protein LOC107330276 isoform X1			
Acrdi.XM_015899225.1/1-540 | Monca.augustus.g61138.t1/1-516	0.0682283	0.0502885	Adenylate kinase isoenzyme 6	6	F:GO:0016887; F:GO:0004017; F:GO:0005524; P:GO:0046939;	[illegible]
Acrdi.XM_015906465.1/1-1305 | Monca.augustus.g52042.t1/1-2037	0.0600772	0.0502767	fragile X mental retardation syndrome-related 1-like isoform X2	5	C:GO:0005844; C:GO:0005634; C:GO:0005737; F:GO:0003729;	[illegible]
Acrdi.XM_015909888.1/1-3540 | Monca.augustus.g30583.t1/1-3615	0.0555341	0.0502566	pre-mRNA-splicing factor ATP-dependent RNA helicase PRP16	6	F:GO:0000166; F:GO:0003676; C:GO:0005737; C:GO:0005681;	[illegible]
Acrdi.XM_015923763.1/1-1218 | Monca.augustus.g71197.t1/1-2235	0.0614479	0.0502431	methylmalonyl- mitochondrial-like	5	F:GO:0046872; F:GO:0031419; C:GO:0005739; P:GO:0008152;	[illegible]
Acrdi.XM_015891690.1/1-1194 | Monca.augustus.g50017.t1/1-1179	0.453229	0.0502304	PREDICTED: uncharacterized protein LOC107326937 isoform X2			
Acrdi.XM_015900812.1/1-876 | Monca.adi2mcaRNA9997_R0/1-738	0.0747323	0.0502106	E3 ubiquitin- ligase CCNB1IP1	4	F:GO:0008270; F:GO:0061630; P:GO:0007131; P:GO:0016567	[illegible]
Acrdi.XM_015905314.1/1-1086 | Monca.augustus.g42660.t1/1-3450	0.0795118	0.0501938	plasma membrane calcium-transporting ATPase	8	C:GO:0005887; F:GO:0005524; F:GO:0046872; C:GO:0043231;	[illegible]
Acrdi.XM_015915117.1/1-1512 | Monca.adi2mcaRNA25779_R0/1-1293	0.445666	0.0501142	PREDICTED: uncharacterized protein LOC107349020			
Acrdi.XM_015915003.1/1-786 | Monca.augustus.g51618.t1/1-876	0.082595	0.0501021	BTB and MATH domain-containing 36-like			
Acrdi.XM_015919039.1/1-1131 | Monca.augustus.g7848.t1/1-1128	0.45453	0.0500711	52 kDa repressor of the inhibitor of the kinase-like	1	F:GO:0005488	[illegible]
Acrdi.XM_015901406.1/1-690 | Monca.adi2mcaRNA10668_R0/1-765	0.440017	0.0500711	TPA_exp: poly			
Acrdi.XM_015911177.1/1-1962 | Monca.augustus.g71196.t1/1-5592	0.0700956	0.0500708	unconventional myosin-X	4	C:GO:0016459; F:GO:0005524; F:GO:0003779; F:GO:0003774	[illegible]
Acrdi.XM_015911369.1/1-1215 | Monca.augustus.g35264.t1/1-1416	0.0478961	0.050705	serine mitochondrial isoform X4	7	F:GO:0030170; P:GO:0006563; F:GO:0004372; P:GO:0035999;	[illegible]
Acrdi.XM_015899728.1/1-957 | Monca.augustus.g71177.t1/1-891	0.17158	0.0500704	Integrase recombinase xerD-like	5	F:GO:0003677; C:GO:0016020; C:GO:0016021; P:GO:0015074;	[illegible]
Acrdi.XM_015918433.1/1-795 | Monca.augustus.g29193.t1/1-1749	0.082493	0.0500678	REST corepressor 3 isoform X1	6	C:GO:0005634; C:GO:0005667; F:GO:0003700; F:GO:0044212;	[illegible]
Acrdi.XM_015911837.1/1-507 | Monca.augustus.g71434.t1/1-507	0.0676457	0.0499873	PREDICTED: uncharacterized protein LOC107346070			
Acrdi.XM_015896285.1/1-651 | Monca.augustus.g33691.t1/1-651	0.0743161	0.0499772	PREDICTED: uncharacterized protein LOC107331672			
Acrdi.XM_015910794.1/1-1254 | Monca.augustus.g48003.t1/1-1254	0.0597995	0.0499627	BRO1 domain-containing BROX			
Acrdi.XM_015913983.1/1-663 | Monca.augustus.g59715.t1/1-585	0.0739391	0.0499437	Prefoldin subunit 3	7	C:GO:0005844; F:GO:0015631; P:GO:0007021; C:GO:0005737;	[illegible]

Acrdi.XM_015923987.1/1-414	Monca.augustus.g1829.t1/1-408	0.0682745	0.0499199	CDGSH iron-sulfur domain-containing 2	7	C:GO:0005789; F:GO:0042803; P:GO:0010506; P:GO:0010259;	C:endoplasmic reticulum membrane; F:protein homodimerization activity; P:regulation of autophagy; P:multicellular organism aging; C:mitochondrial outer membrane; F:2 iron, 2 [illegible]	
Acrdi.XM_015918244.1/1-1530	Monca.augustus.g48572.t1/1-1587	0.0650146	0.0499071	amidophosphoribosyltransferase-like	6	F:GO:0046872; P:GO:0006189; P:GO:0009116; F:GO:0004044;	F:metal ion binding; P:'de novo' IMP biosynthetic process; P:nucleoside metabolic process; F:amidophosphoribosyltransferase activity; P:purine nucleobase biosynthetic proce	EC:2.[illegible]
Acrdi.XM_015897208.1/1-942	Monca.augustus.g7165.t1/1-942	0.0615976	0.0498745	retinol dehydrogenase 12-like	1	C:GO:0016020	C:membrane	
Acrdi.XM_015907928.1/1-573	Monca.augustus.g53165.t1/1-618	0.0865156	0.0498511	PITH domain-containing CG6153				
Acrdi.XM_015908911.1/1-345	Monca.augustus.g15548.t1/1-345	0.0544916	0.0498271	histidine triad nucleotide-binding 1	2	P:GO:0016310; F:GO:0016301	P:phosphorylation; F:kinase activity	
Acrdi.XM_015920128.1/1-4521	Monca.adi2mcaRNA31375_R1/1-3957	0.053674	0.0498138	spectrin alpha non-erythrocytic 1	1	F:GO:0005509	F:calcium ion binding	
Acrdi.XM_015923773.1/1-1671	Monca.augustus.g54391.t1/1-1911	0.0663161	0.0498106	Asparagine synthetase [glutamine-hydrolyzing]	7	F:GO:0000166; F:GO:0042803; P:GO:0050794; P:GO:0006529;	F:nucleotide binding; F:protein homodimerization activity; P:regulation of cellular process; P:asparagine biosynthetic process; P:glutamine metabolic process; C:cytosol; F:asp	EC:6.[illegible]
Acrdi.XM_015901881.1/1-2100	Monca.augustus.g7721.t1/1-2100	0.0572231	0.0497986	probable ATP-dependent RNA helicase DHX35	6	F:GO:0003676; F:GO:0005524; C:GO:0071013; C:GO:0005737;	F:nucleic acid binding; F:ATP binding; C:catalytic step 2 spliceosome; C:cytoplasm; P:RNA processing; F:ATP-dependent RNA helicase activity	EC:3.[illegible]
Acrdi.XM_015900224.1/1-753	Monca.augustus.g14552.t1/1-753	0.077176	0.0497708	u2 small nuclear ribonucleo a	4	C:GO:0019013; C:GO:0005686; F:GO:0030620; P:GO:0000398	C:viral nucleocapsid; C:U2 snRNP; F:U2 snRNA binding; P:mRNA splicing, via spliceosome	
Acrdi.XM_015913862.1/1-345	Monca.augustus.g70082.t1/1-738	0.0575023	0.049768	UPF0568 C14orf166 homolog				
Acrdi.XM_015901960.1/1-1275	Monca.augustus.g45536.t1/1-1275	0.0760155	0.0497418	Isocitrate lyase	4	P:GO:0006097; C:GO:0005737; F:GO:0004451; F:GO:0046912	P:glyoxylate cycle; C:cytoplasm; F:isocitrate lyase activity; F:transferase activity, transferring acyl groups, acyl groups converted into alkyl on transfer	EC:4.[illegible]
Acrdi.XM_015906275.1/1-1509	Monca.augustus.g40445.t1/1-1509	0.0861011	0.049733	Trichoplein keratin filament-binding	3	C:GO:0005856; C:GO:0005737; C:GO:0044422	C:cytoskeleton; C:cytoplasm; C:organelle part	
Acrdi.XM_015898351.1/1-555	Monca.augustus.g44535.t1/1-1314	0.0636364	0.04972	trafficking particle complex subunit 13 isoform X2	1	F:GO:0005515	F:protein binding	
Acrdi.XM_015904566.1/1-453	Monca.augustus.g68205.t1/1-456	0.0520442	0.0496948	PREDICTED: uncharacterized protein CXorf65 homolog				
Acrdi.XM_015916983.1/1-705	Monca.adi2mcaRNA2843_R1/1-657	0.540033	0.0496882	U3 small nucleolar RNA-associated 6 homolog				
Acrdi.XM_015906480.1/1-1155	Monca.augustus.g22567.t1/1-2463	0.0624778	0.0496259	coiled-coil domain-containing 170-like				
Acrdi.XM_015896709.1/1-699	Monca.augustus.g21880.t1/1-1248	0.0406868	0.0496171	creatine kinase U- mitochondrial-like	1	F:GO:0003824	F:catalytic activity	
Acrdi.XM_015896603.1/1-1011	Monca.augustus.g1593.t1/1-1011	0.0811929	0.0495939	2-acylglycerol O-acyltransferase 2	2	F:GO:0016747; C:GO:0016021	F:transferase activity, transferring acyl groups other than amino-acyl groups; C:integral component of membrane	
Acrdi.XM_015905781.1/1-1752	Monca.augustus.g32754.t1/1-2697	0.0641227	0.0495789	pre-mRNA-processing factor 40 homolog A isoform X2	4	F:GO:0003723; C:GO:0071004; C:GO:0005685; P:GO:0000398	F:RNA binding; C:U2-type prespliceosome; C:U1 snRNP; P:mRNA splicing, via spliceosome	
Acrdi.XM_015907340.1/1-657	Monca.augustus.g31823.t1/1-654	0.0656923	0.0495725	ras-related Rab-11A	45	P:GO:0003091; C:GO:0032154; P:GO:0015031; P:GO:0000910;	P:renal water homeostasis; C:cleavage furrow; P:protein transport; P:cytokinesis; C:multivesicular body; P:regulation of vesicle-mediated transport; P:establishment of protein	EC:3.[illegible]
Acrdi.XM_015916419.1/1-468	Monca.adi2mcaRNA27224_R0/1-471	0.0685394	0.0495461	Prefoldin subunit 5	3	C:GO:0016272; F:GO:0051082; P:GO:0006457	C:prefoldin complex; F:unfolded protein binding; P:protein folding	
Acrdi.XM_015915821.1/1-3003	Monca.adi2mcaRNA26567_R1/1-2631	0.242893	0.0495321	Transposon Tf2-9 poly				
Acrdi.XM_015901470.1/1-471	Monca.augustus.g36351.t1/1-1767	0.0380369	0.0495308	calcium calmodulin-dependent 3 ,5 -cyclic nucleotide phosphodiestera	3	F:GO:0046872; P:GO:0007165; F:GO:0004114	F:metal ion binding; P:signal transduction; F:3',5'-cyclic-nucleotide phosphodiesterase activity	EC:3.[illegible]
Acrdi.XM_015903070.1/1-843	Monca.augustus.g48850.t1/1-831	0.0707154	0.0495195	Forkhead box L1	5	C:GO:0005634; P:GO:0006351; F:GO:0043565; F:GO:0000981;	C:nucleus; P:transcription, DNA-templated; F:sequence-specific DNA binding; F:RNA polymerase II transcription factor activity, sequence-specific DNA binding; P:regulation of tran [illegible]	
Acrdi.XM_015895853.1/1-1221	Monca.augustus.g65880.t1/1-1728	0.0623879	0.0495013	Phenylalanine--tRNA ligase beta subunit	6	F:GO:0000287; F:GO:0005524; F:GO:0003723; F:GO:0004826;	F:magnesium ion binding; F:ATP binding; F:RNA binding; F:phenylalanine-tRNA ligase activity; P:phenylalanyl-tRNA aminoacylation; C:phenylalanine-tRNA ligase complex	EC:6.[illegible]
Acrdi.XM_015905455.1/1-1722	Monca.adi2mcaRNA15103_R0/1-1683	0.0629106	0.049471	Major facilitator superfamily domain-containing 4	3	P:GO:0055085; C:GO:0016020; C:GO:0016021	P:transmembrane transport; C:membrane; C:integral component of membrane	
Acrdi.XM_015907710.1/1-1353	Monca.augustus.g55804.t1/1-1428	0.059216	0.0494624	vesicular inhibitory amino acid transporter-like	3	C:GO:0016021; F:GO:0015171; P:GO:0003333	C:integral component of membrane; F:amino acid transmembrane transporter activity; P:amino acid transmembrane transport	
Acrdi.XM_015894279.1/1-417	Monca.adi2mcaRNA40519_R8/1-366	0.45789	0.0494346	Mitochondrial inner membrane protease subunit 1				
Acrdi.XM_015915636.1/1-426	Monca.augustus.g36644.t1/1-426	0.0538453	0.0494344	D Chain Coupling Of Remote Alternating-access Transport Mechanisms For Protons And Substrates In The Multidrug Efflux Pump Acrb				
Acrdi.XM_015901493.1/1-402	Monca.adi2mcaRNA10809_R0/1-402	0.0771733	0.0494109	ATP synthase subunit mitochondrial isoform X2	1	C:GO:0005753	C:mitochondrial proton-transporting ATP synthase complex	
Acrdi.XM_015919165.1/1-492	Monca.adi2mcaRNA30316_R8/1-444	0.810934	0.0494092	PREDICTED: uncharacterized protein LOC107352846				
Acrdi.XM_015904502.1/1-1932	Monca.augustus.g33586.t1/1-1905	0.0926574	0.0493961	PR domain zinc finger 13	2	F:GO:0003676; F:GO:0046872	F:nucleic acid binding; F:metal ion binding	
Acrdi.XM_015894239.1/1-1533	Monca.adi2mcaRNA4062_R0/1-1545	0.0686133	0.0493865	TBC1 domain family member 23-like	1	P:GO:0050789	P:regulation of biological process	
Acrdi.XM_015923089.1/1-654	Monca.augustus.g70878.t1/1-654	0.0544758	0.0493838	autophagy-related 101	4	P:GO:0000045; F:GO:0042802; C:GO:0000407; F:GO:0032403	P:autophagosome assembly; F:identical protein binding; C:pre-autophagosomal structure; F:protein complex binding	
Acrdi.XM_015902292.1/1-1539	Monca.augustus.g35094.t1/1-1539	0.0531364	0.0493831	Tyrosine- kinase Src42A	3	F:GO:0005524; P:GO:0018108; F:GO:0004715	F:ATP binding; P:peptidyl-tyrosine phosphorylation; F:non-membrane spanning protein tyrosine kinase activity	EC:2.[illegible]
Acrdi.XM_015899438.1/1-699	Monca.augustus.g46731.t1/1-699	0.0746042	0.0493688	lysosomal-associated transmembrane 4A	1	C:GO:0016020	C:membrane	
Acrdi.XM_015893451.1/1-1350	Monca.augustus.g50902.t1/1-1701	0.0814675	0.0492712	glycerol kinase-like isoform X2	7	P:GO:0006071; P:GO:0046167; C:GO:0005739; F:GO:0004370;	P:glycerol metabolic process; P:glycerol-3-phosphate biosynthetic process; C:mitochondrion; F:glycerol kinase activity; C:integral component of membrane; P:triglyceride meta	EC:2.[illegible]
Acrdi.XM_015915169.1/1-1089	Monca.adi2mcaRNA25826_R0/1-1098	0.0842642	0.0492506	E3 ubiquitin- ligase KCMF1	1	F:GO:0003824	F:catalytic activity	
Acrdi.XM_015923999.1/1-468	Monca.augustus.g55722.t1/1-468	0.101826	0.0492122	A-kinase-interacting 1	1	P:GO:1901222	P:regulation of NIK/NF-kappaB signaling	
Acrdi.XM_015924021.1/1-828	Monca.augustus.g6258.t1/1-963	0.301465	0.0492104	PREDICTED: uncharacterized protein LOC107357385	2	C:GO:0016020; C:GO:0016021	C:membrane; C:integral component of membrane	
Acrdi.XM_015899352.1/1-726	Monca.augustus.g40312.t1/1-723	0.0651205	0.0492047	transcription factor SOX-14	10	P:GO:0000122; P:GO:0009649; F:GO:0046982; P:GO:0006351;	P:negative regulation of transcription from RNA polymerase II promoter; P:entrainment of circadian clock; F:protein heterodimerization activity; P:transcription, DNA-templated; F [illegible]	
Acrdi.XM_015893342.1/1-1227	Monca.augustus.g67208.t1/1-1239	0.152157	0.0491621	nuclease HARBI1				
Acrdi.XM_015896728.1/1-534	Monca.augustus.g18690.t1/1-531	0.0651796	0.0491557	fas apoptotic inhibitory molecule 1 isoform X2	1	P:GO:0043066	P:negative regulation of apoptotic process	
Acrdi.XM_015906681.1/1-849	Monca.adi2mcaRNA16448_R0/1-849	0.0636459	0.0491282	polyhomeotic 1 isoform X2				
Acrdi.XM_015918779.1/1-894	Monca.augustus.g49825.t1/1-894	0.412757	0.0491205	THAP domain-containing 2				
Acrdi.XM_015919329.1/1-1878	Monca.adi2mcaRNA30490_R0/1-1881	0.069622	0.0491083	arginine N-methyltransferase 5 isoform X1	26	P:GO:0008283; P:GO:0042118; F:GO:0035243; P:GO:0019918;	P:cell proliferation; P:endothelial cell activation; F:protein-arginine omega-N symmetric methyltransferase activity; P:peptidyl-arginine methylation, to symmetrical-dimethyl argi [illegible]	
Acrdi.XM_015918205.1/1-561	Monca.adi2mcaRNA29233_R5/1-570	1.09289	0.049108	PREDICTED: uncharacterized protein LOC107351899	1	P:GO:0009987	P:cellular process	
Acrdi.XM_015906065.1/1-1266	Monca.augustus.g34449.t1/1-1344	0.0426007	0.049067	cellular tumor antigen p53-like isoform X7	5	C:GO:0005634; F:GO:0003700; P:GO:0006915; F:GO:0044212;	C:nucleus; F:transcription factor activity, sequence-specific DNA binding; P:apoptotic process; F:transcription regulatory region DNA binding; P:regulation of transcription, DNA-tem [illegible]	
Acrdi.XM_015924739.1/1-438	Monca.augustus.g4188.t1/1-1512	0.0710852	0.0490595	probable aminopeptidase NPEPL1	5	C:GO:0005737; P:GO:0006508; F:GO:0008235; F:GO:0004177;	C:cytoplasm; P:proteolysis; F:metalloexopeptidase activity; F:aminopeptidase activity; F:manganese ion binding	EC:3.[illegible]
Acrdi.XM_015922159.1/1-435	Monca.augustus.g9770.t1/1-435	0.0641259	0.0490399	coiled-coil domain-containing 58 isoform X1	1	C:GO:0005739	C:mitochondrion	
Acrdi.XM_015897313.1/1-450	Monca.augustus.g41215.t1/1-450	0.0608721	0.0490245	myosin-2 essential light chain-like	1	F:GO:0005509	F:calcium ion binding	
Acrdi.XM_015923028.1/1-753	Monca.augustus.g3980.t1/1-759	0.267311	0.0489709	PREDICTED: uncharacterized protein LOC107356412				
Acrdi.XM_015900370.1/1-447	Monca.augustus.g54180.t1/1-1329	0.0641572	0.0489334	CDK5 and ABL1 enzyme substrate	1	P:GO:0051726	P:regulation of cell cycle	
Acrdi.XM_015911882.1/1-666	Monca.adi2mcaRNA22160_R0/1-675	0.428652	0.048918	nuclease HARBI1				
Acrdi.XM_015910779.1/1-348	Monca.augustus.g20679.t1/1-348	0.0628668	0.0489088	dpy-30 homolog	5	C:GO:0048188; F:GO:0042800; C:GO:0000781; P:GO:0006348;	C:Set1C/COMPASS complex; F:histone methyltransferase activity (H3-K4 specific); C:chromosome, telomeric region; P:chromatin silencing at telomere; P:histone H3-K4 meth	EC:2.[illegible]
Acrdi.XM_015912256.1/1-1755	Monca.augustus.g28985.t1/1-2349	0.0657366	0.0489045	Tyrosine- phosphatase non-receptor type	4	C:GO:0005737; F:GO:0004725; F:GO:0016740; P:GO:0035335	C:cytoplasm; F:protein tyrosine phosphatase activity; F:transferase activity; P:peptidyl-tyrosine dephosphorylation	EC:3.[illegible]
Acrdi.XM_015919732.1/1-3363	Monca.augustus.g11102.t1/1-3294	0.404245	0.0488809	ankyrin-1-like isoform X2				
Acrdi.XM_015899217.1/1-1041	Monca.adi2mcaRNA8343_R9/1-885	0.219539	0.0488759	Transposon Tf2-9 poly				
Acrdi.XM_015906653.1/1-1107	Monca.adi2mcaRNA16463_R2/1-930	0.392293	0.0488172	PREDICTED: uncharacterized protein K02A2.6-like				
Acrdi.XM_015901059.1/1-1755	Monca.adi2mcaRNA10298_R0/1-1767	0.0624329	0.048785	60 kDa heat shock mitochondrial-like	3	F:GO:0005524; C:GO:0005737; P:GO:0042026	F:ATP binding; C:cytoplasm; P:protein refolding	
Acrdi.XM_015911748.1/1-1299	Monca.augustus.g61977.t1/1-1011	0.0764276	0.0487244	Phenylalanine--tRNA mitochondrial	5	F:GO:0000166; C:GO:0005739; F:GO:0004826; F:GO:0043167;	F:nucleotide binding; C:mitochondrion; F:phenylalanine-tRNA ligase activity; F:ion binding; P:phenylalanyl-tRNA aminoacylation	EC:6.[illegible]
Acrdi.XM_015903019.1/1-1149	Monca.adi2mcaRNA12377_R0/1-1140	0.0633788	0.0486757	PREDICTED: uncharacterized protein LOC107337783 isoform X2	1	P:GO:0060828	P:regulation of canonical Wnt signaling pathway	
Acrdi.XM_015916041.1/1-2829	Monca.adi2mcaRNA26801_R0/1-2664	0.0597282	0.0486549	structural maintenance of chromosomes 3	1	C:GO:0044424	C:intracellular part	
Acrdi.XM_015908437.1/1-630	Monca.augustus.g33952.t1/1-633	0.0796165	0.0486402	osteoclast-stimulating factor 1-like	3	C:GO:0070062; C:GO:0005737; F:GO:0017124	C:extracellular exosome; C:cytoplasm; F:SH3 domain binding	
Acrdi.XM_015914206.1/1-537	Monca.augustus.g58340.t1/1-531	0.0841241	0.0486282	core-binding factor subunit beta isoform X1	11	C:GO:0016513; C:GO:0030018; F:GO:0003677; P:GO:0060047;	C:core-binding factor complex; C:Z disc; F:DNA binding; P:heart contraction; F:transcription coactivator activity; F:protein heterodimerization activity; P:ossification; P:hemopoiesis; [illegible]	
Acrdi.XM_015905770.1/1-564	Monca.augustus.g32748.t1/1-564	0.0605971	0.0486152	NADH dehydrogenase [ubiquinone] 1 beta subcomplex subunit mitochi	1	C:GO:0016020	C:membrane	
Acrdi.XM_015906085.1/1-3750	Monca.augustus.g34434.t1/1-6333	0.0753912	0.0486066	nipped-B isoform X2	10	P:GO:0048562; C:GO:0043229; P:GO:0007507; P:GO:0050793;	P:embryonic organ morphogenesis; C:intracellular organelle; P:heart development; P:regulation of developmental process; F:protein binding; P:regulation of transcription, DNA-te [illegible]	
Acrdi.XM_015901138.1/1-1233	Monca.adi2mcaRNA10361_R0/1-1233	0.0696141	0.0485942	UDP-N-acetylglucosamine--dolichyl-phosphate N-acetylglucosamineph	7	C:GO:0005789; F:GO:0003975; C:GO:0016021; P:GO:0006047;	C:endoplasmic reticulum membrane; F:UDP-N-acetylglucosamine-dolichyl-phosphate N-acetylglucosaminephosphotransferase activity; C:integral component of membrane; I	EC:2.[illegible]
Acrdi.XM_015899508.1/1-2907	Monca.augustus.g54409.t1/1-2910	0.314981	0.0485155	Kyphoscoliosis peptidase				
Acrdi.XM_015907276.1/1-1323	Monca.adi2mcaRNA17125_R0/1-1305	0.0697803	0.0485113	stress-activated kinase JNK isoform X2	4	F:GO:0005524; P:GO:0000165; F:GO:0004707; C:GO:0005622	F:ATP binding; P:MAPK cascade; F:MAP kinase activity; C:intracellular	EC:2.[illegible]
Acrdi.XM_015916653.1/1-1572	Monca.adi2mcaRNA27477_R0/1-1560	0.0412075	0.0484408	histone-lysine N-methyltransferase EZH2-like isoform X1				
Acrdi.XM_015924476.1/1-423	Monca.augustus.g67826.t1/1-681	0.074681	0.0484356	Exocyst complex component 2	2	P:GO:0006893; C:GO:0000145	P:Golgi to plasma membrane transport; C:exocyst	
Acrdi.XM_015908370.1/1-666	Monca.augustus.g9823.t1/1-735	0.0634475	0.0484296	TATA-box-binding -like	9	C:GO:0005634; F:GO:0003677; F:GO:0003700; P:GO:0045892;	C:nucleus; F:DNA binding; F:transcription factor activity, sequence-specific DNA binding; P:negative regulation of transcription, DNA-templated; C:cytoplasm; P:transcription from [illegible]	
Acrdi.XM_015896330.1/1-2586	Monca.adi2mcaRNA5145_R0/1-2280	0.312391	0.0484277	THAP domain-containing 10				
Acrdi.XM_015897465.1/1-684	Monca.augustus.g22835.t1/1-1998	0.0608555	0.0483981	Receptor-type tyrosine- phosphatase	2	F:GO:0004721; P:GO:0006470	F:phosphoprotein phosphatase activity; P:protein dephosphorylation	EC:3.[illegible]
Acrdi.XM_015895501.1/1-906	Monca.augustus.g32977.t1/1-900	0.10367	0.0483761	Mitochondrial dicarboxylate carrier	4	F:GO:0003735; C:GO:0016021; P:GO:0006810; P:GO:0006412	F:structural constituent of ribosome; C:integral component of membrane; P:transport; P:translation	
Acrdi.XM_015906943.1/1-1488	Monca.augustus.g49133.t1/1-1488	0.0890554	0.048358	T-box transcription factor TBX5-A-like	37	P:GO:0060044; P:GO:0060045; P:GO:0060980; P:GO:0003190;	P:negative regulation of cardiac muscle cell proliferation; P:positive regulation of cardiac muscle cell proliferation; P:cell migration involved in coronary vasculogenesis; P:atriovent [illegible]	
Acrdi.XM_015908028.1/1-684	Monca.augustus.g5041.t1/1-684	0.0631656	0.0483131	CRAL-TRIO domain-containing				
Acrdi.XM_015892675.1/1-651	Monca.augustus.g29486.t1/1-819	0.0411012	0.0483073	urease accessory -like	3	P:GO:0006807; F:GO:0003924; F:GO:0016151	P:nitrogen compound metabolic process; F:GTPase activity; F:nickel cation binding	EC:3.[illegible]
Acrdi.XM_015899208.1/1-1389	Monca.augustus.g62007.t1/1-1449	0.0618612	0.04829	Ubiquitin carboxyl-terminal hydrolase 3	4	F:GO:0004843; F:GO:0008270; P:GO:0006511; P:GO:0016578	F:thiol-dependent ubiquitin-specific protease activity; F:zinc ion binding; P:ubiquitin-dependent protein catabolic process; P:histone deubiquitination	EC:3.[illegible]
Acrdi.XM_015910576.1/1-948	Monca.augustus.g70622.t1/1-945	0.0723857	0.0482348	band 7 AGAP004871-like	4	P:GO:0007638; F:GO:0015485; C:GO:0016021; C:GO:0032589	P:mechanosensory behavior; F:cholesterol binding; C:integral component of membrane; C:neuron projection membrane	
Acrdi.XM_015923792.1/1-2055	Monca.augustus.g54326.t1/1-2325	0.0600653	0.0482166	exocyst complex component 6 isoform X3	2	P:GO:0006904; C:GO:0000145	P:vesicle docking involved in exocytosis; C:exocyst	
Acrdi.XM_015899708.1/1-447	Monca.augustus.g17502.t1/1-447	0.0559767	0.0481753	allograft inflammatory factor 1	1	F:GO:0005509	F:calcium ion binding	
Acrdi.XM_015901256.1/1-819	Monca.augustus.g61802.t1/1-963	0.0547651	0.0481501	Deoxyribose-phosphate aldolase	3	F:GO:0004139; P:GO:0009264; P:GO:0016052	F:deoxyribose-phosphate aldolase activity; P:deoxyribonucleotide catabolic process; P:carbohydrate catabolic process	EC:4.[illegible]
Acrdi.XM_015922002.1/1-1875	Monca.augustus.g60209.t1/1-1830	0.0520598	0.0481407	ATP-binding cassette sub-family E member 1	2	F:GO:0016887; F:GO:0005524	F:ATPase activity; F:ATP binding	EC:3.[illegible]
Acrdi.XM_015912820.1/1-471	Monca.augustus.g82.t1/1-2661	0.350645	0.0480895	PREDICTED: uncharacterized protein LOC107346951				
Acrdi.XM_015897205.1/1-2007	Monca.augustus.g39439.t1/1-2529	0.053836	0.0480828	ECT2 isoform X4				
Acrdi.XM_015918077.1/1-1431	Monca.augustus.g43975.t1/1-1926	0.0762077	0.048071	acyl-coenzyme A oxidase peroxisomal	9	F:GO:0003995; C:GO:0005777; F:GO:0003997; P:GO:0033539;	F:acyl-CoA dehydrogenase activity; C:peroxisome; F:acyl-CoA oxidase activity; P:fatty acid beta-oxidation using acyl-CoA dehydrogenase; F:fatty-acyl-CoA binding; F:flavin ade	EC:1.[illegible]
Acrdi.XM_015912364.1/1-2694	Monca.adi2mcaRNA22699_R6/1-2565	0.237886	0.0480658	Pao retrotransposon peptidase family				
Acrdi.XM_015915957.1/1-726	Monca.augustus.g18728.t1/1-726	0.0607531	0.0480644	Vacuolar-sorting SNF8	11	C:GO:0005634; P:GO:0043328; P:GO:0065008; F:GO:0005515;	C:nucleus; P:protein targeting to vacuole involved in ubiquitin-dependent protein catabolic process via the multivesicular body sorting pathway; P:regulation of biological quality; F [illegible]	
Acrdi.XM_015923563.1/1-888	Monca.augustus.g58092.t1/1-816	0.0640332	0.0480575	ras suppressor 1 isoform X2				
Acrdi.XM_015925460.1/1-2151	Monca.augustus.g43741.t1/1-2148	0.0633452	0.048051	Translin-associated factor X-interacting 1	1	F:GO:0005509	F:calcium ion binding	
Acrdi.XM_015902762.1/1-699	Monca.augustus.g5673.t1/1-699	0.0545441	0.0480431	complex I intermediate-associated mitochondrial				
Acrdi.XM_015909444.1/1-915	Monca.augustus.g45743.t1/1-972	0.0516391	0.0480313	WD repeat-containing 82	8	C:GO:0048188; F:GO:0042800; C:GO:0000781; F:GO:0003779;	C:Set1C/COMPASS complex; F:histone methyltransferase activity (H3-K4 specific); C:chromosome, telomeric region; F:actin binding; C:PTW/PP1 phosphatase complex; F:chro	EC:2.[illegible]
Acrdi.XM_015892945.1/1-2313	Monca.augustus.g60895.t1/1-2826	0.0719953	0.0480268	fem-1 homolog A				
Acrdi.XM_015902881.1/1-501	Monca.adi2mcaRNA12226_R0/1-444	0.0643599	0.0479402	Hypoxanthine-guanine phosphoribosyltransferase	9	F:GO:0000287; P:GO:0032263; P:GO:0032264; P:GO:0046100;	F:magnesium ion binding; P:GMP salvage; P:IMP salvage; P:hypoxanthine metabolic process; P:guanine salvage; C:cytosol; P:adenine salvage; F:hypoxanthine phosphoribosylt	EC:2.[illegible]
Acrdi.XM_015910945.1/1-363	Monca.augustus.g3089.t1/1-363	0.0661614	0.0478333	Transmembrane 218	6	P:GO:0008150; C:GO:0016020; C:GO:0016021; C:GO:0005929;	P:biological_process; C:membrane; C:integral component of membrane; C:cilium; C:cell projection; F:molecular_function	
Acrdi.XM_015918135.1/1-1623	Monca.adi2mcaRNA29133_R0/1-1626	0.0495907	0.0476928	Serine--tRNA cytoplasmic	5	F:GO:0005524; C:GO:0070062; C:GO:0005737; F:GO:0004828;	F:ATP binding; C:extracellular exosome; C:cytoplasm; F:serine-tRNA ligase activity; P:seryl-tRNA aminoacylation	EC:6.[illegible]
Acrdi.XM_015922468.1/1-1173	Monca.augustus.g11608.t1/1-1170	0.0634286	0.0476909	OSCP1 isoform X2				
Acrdi.XM_015918071.1/1-717	Monca.augustus.g34638.t1/1-717	0.0537156	0.0476875	Ras-related Rab-32	3	F:GO:0005525; P:GO:0007264; C:GO:0005776	F:GTP binding; P:small GTPase mediated signal transduction; C:autophagosome	
Acrdi.XM_015922746.1/1-1176	Monca.augustus.g30168.t1/1-3159	0.0619123	0.0476303	zinc finger RNA-binding -like	1	F:GO:0046872	F:metal ion binding	
Acrdi.XM_015907915.1/1-1389	Monca.augustus.g46316.t1/1-1389	0.058094	0.0475751	adenylosuccinate lyase	5	F:GO:0004018; P:GO:0044208; P:GO:0051262; P:GO:0006189;	F:N6-(1,2-dicarboxyethyl)AMP AMP-lyase (fumarate-forming) activity; P:'de novo' AMP biosynthetic process; P:protein tetramerization; P:'de novo' IMP biosynthetic process;	EC:4.[illegible]
Acrdi.XM_015925201.1/1-1020	Monca.adi2mcaRNA37117_R2/1-1635	0.5347	0.047571	zinc finger 502-like isoform X2				
Acrdi.XM_015901621.1/1-1464	Monca.adi2mcaRNA10932_R0/1-1458	0.212543	0.0475654	PREDICTED: uncharacterized protein LOC107336548				
Acrdi.XM_015924512.1/1-675	Monca.augustus.g28088.t1/1-675	0.0519194	0.0475621	programmed cell death 6-like	4	C:GO:0005737; F:GO:0005509; P:GO:0006508; F:GO:0004198	C:cytoplasm; F:calcium ion binding; P:proteolysis; F:calcium-dependent cysteine-type endopeptidase activity	EC:3.[illegible]
Acrdi.XM_015908031.1/1-345	Monca.augustus.g5046.t1/1-345	0.0450617	0.0475406	Anaphase-promoting complex subunit 15	2	C:GO:0005680; P:GO:0090266	C:anaphase-promoting complex; P:regulation of mitotic cell cycle spindle assembly checkpoint	
Acrdi.XM_015897443.1/1-1188	Monca.augustus.g35250.t1/1-1599	0.0436323	0.0475273	sorting nexin-8-like	5	C:GO:0005768; P:GO:0016050; F:GO:0035091; C:GO:0019898;	C:endosome; P:vesicle organization; F:phosphatidylinositol binding; C:extrinsic component of membrane; P:endocytosis	
Acrdi.XM_015909068.1/1-1155	Monca.augustus.g13101.t1/1-1158	0.068526	0.0475124	upstream stimulatory factor 2 isoform X2				
Acrdi.XM_015898918.1/1-1329	Monca.augustus.g37947.t1/1-1293	0.0549003	0.0474692	zinc finger DPF3-like isoform X2				
Acrdi.XM_015896234.1/1-537	Monca.augustus.g54597.t1/1-537	0.100976	0.0474442	programmed cell death 6-like isoform X2	4	C:GO:0005737; F:GO:0005509; P:GO:0006508; F:GO:0004198	C:cytoplasm; F:calcium ion binding; P:proteolysis; F:calcium-dependent cysteine-type endopeptidase activity	EC:3.[illegible]
Acrdi.XM_015903356.1/1-1266	Monca.augustus.g35988.t1/1-1842	0.0485951	0.0473821	kinesin light chain 1 isoform X4	2	F:GO:0003777; C:GO:0005871	F:microtubule motor activity; C:kinesin complex	EC:3.[illegible]
Acrdi.XM_015915985.1/1-402	Monca.augustus.g30919.t1/1-432	1.01269	0.047314	Zinc finger and SCAN domain-containing 29				
Acrdi.XM_015892333.1/1-591	Monca.augustus.g59165.t1/1-597	0.549044	0.0472758	PREDICTED: uncharacterized protein LOC107327590				
Acrdi.XM_015891675.1/1-1065	Monca.adi2mcaRNA37529_R0/1-987	0.252014	0.0472154	paired box Pax-5-like				
Acrdi.XM_015903192.1/1-696	Monca.augustus.g39409.t1/1-2022	0.0669806	0.0471747	IWS1 homolog	3	C:GO:0005634; F:GO:0003677; P:GO:0006351	C:nucleus; F:DNA binding; P:transcription, DNA-templated	
Acrdi.XM_015923800.1/1-720	Monca.augustus.g32996.t1/1-888	0.0471181	0.0471744	Glyoxalase domain-containing 4	3	P:GO:0008150; C:GO:0005575; F:GO:0003674	P:biological_process; C:cellular_component; F:molecular_function	
Acrdi.XM_015892070.1/1-519	Monca.augustus.g10911.t1/1-657	0.0649294	0.0471431	vesicle-associated membrane 7-like isoform X1	6	P:GO:0006906; C:GO:0016021; F:GO:0005484; C:GO:0031201;	P:vesicle fusion; C:integral component of membrane; F:SNAP receptor activity; C:SNARE complex; F:SNARE binding; P:exocytosis	
Acrdi.XM_015916915.1/1-2151	Monca.adi2mcaRNA27782_R1/1-1761	0.48866	0.0471356	PREDICTED: uncharacterized protein LOC107350682	1	F:GO:0004842	F:ubiquitin-protein transferase activity	
Acrdi.XM_015903603.1/1-1233	Monca.augustus.g32275.t1/1-1248	0.0645332	0.0470752	glycogen synthase kinase-3 beta isoform X2	3	F:GO:0005524; F:GO:0004674; P:GO:0006468	F:ATP binding; F:protein serine/threonine kinase activity; P:protein phosphorylation	EC:2.[illegible]
Acrdi.XM_015920289.1/1-2325	Monca.augustus.g39629.t1/1-2505	0.0747981	0.0470651	eyes absent homolog 1 isoform X6	9	P:GO:0048522; F:GO:0004725; F:GO:0046872; P:GO:0035295;	P:positive regulation of cellular process; F:protein tyrosine phosphatase activity; F:metal ion binding; P:tube development; P:inner ear morphogenesis; P:cell differentiation; P	EC:3.[illegible]
Acrdi.XM_015900933.1/1-927	Monca.augustus.g27890.t1/1-927	0.0621228	0.0470482	Transmembrane 231	1	C:GO:0016021	C:integral component of membrane	
Acrdi.XM_015923519.1/1-963	Monca.augustus.g47464.t1/1-954	0.0571591	0.0470439	mitochondrial substrate carrier family	4	F:GO:0003735; C:GO:0016020; P:GO:0006810; P:GO:0006412	F:structural constituent of ribosome; C:membrane; P:transport; P:translation	
Acrdi.XM_015895334.1/1-546	Monca.augustus.g42292.t1/1-1584	0.0625539	0.0470295	importin subunit alpha-7	6	P:GO:0006607; C:GO:0005829; F:GO:0008565; F:GO:0008139;	P:NLS-bearing protein import into nucleus; C:cytosol; F:protein transporter activity; F:nuclear localization sequence binding; C:nuclear pore; C:nucleoplasm	
Acrdi.XM_015898077.1/1-1335	Monca.augustus.g2723.t1/1-1335	0.0756956	0.0470071	molybdate-anion transporter	3	F:GO:0015098; C:GO:0016021; P:GO:0015689	F:molybdate ion transmembrane transporter activity; C:integral component of membrane; P:molybdate ion transport	
Acrdi.XM_015894135.1/1-1851	Monca.adi2mcaRNA40359_R6/1-1914	0.469923	0.0469714	PREDICTED: uncharacterized protein LOC107329445				
Acrdi.XM_015903962.1/1-2787	Monca.augustus.g43332.t1/1-2877	0.0681123	0.046938	Ras GTPase-activating 1	6	P:GO:0046580; C:GO:0005737; P:GO:0007165; F:GO:0005096;	P:negative regulation of Ras protein signal transduction; C:cytoplasm; P:signal transduction; F:GTPase activator activity; C:intrinsic component of the cytoplasmic side of the plasm [illegible]	
Acrdi.XM_015898888.1/1-699	Monca.augustus.g5285.t1/1-723	0.0542418	0.0469137	Ras-related Rab-23	12	F:GO:0005525; P:GO:0000045; P:GO:0006968; C:GO:0070062;	F:GTP binding; P:autophagosome assembly; P:cellular defense response; C:extracellular exosome; P:small GTPase mediated signal transduction; F:GTPase activity; C:phagocy	EC:3.[illegible]
Acrdi.XM_015923398.1/1-354	Monca.adi2mcaRNA35059_R2/1-474	0.617619	0.0469095	Transposon Tf2-6 poly				
Acrdi.XM_015916423.1/1-576	Monca.augustus.g64491.t1/1-585	0.0958011	0.0469089	cuticle collagen 34-like				
Acrdi.XM_015914015.1/1-1287	Monca.augustus.g44838.t1/1-1308	0.0540368	0.0469071	headcase isoform X1				
Acrdi.XM_015917702.1/1-1503	Monca.augustus.g42296.t1/1-1506	0.0869554	0.0469029	runt-related transcription factor 1 isoform X1	17	F:GO:0042803; F:GO:0000977; F:GO:0001228; F:GO:0008134;	F:protein homodimerization activity; F:RNA polymerase II regulatory region sequence-specific DNA binding; F:transcriptional activator activity, RNA polymerase II transcription reg [illegible]	
Acrdi.XM_015904270.1/1-330	Monca.adi2mcaRNA13751_R0/1-330	0.042458	0.0468988	Transcription initiation factor IIA subunit 2	10	P:GO:0051123; F:GO:0042803; F:GO:0003713; F:GO:0046982;	P:RNA polymerase II transcriptional preinitiation complex assembly; F:protein homodimerization activity; F:transcription coactivator activity; F:protein heterodimerization activity; [illegible]	
Acrdi.XM_015896384.1/1-1365	Monca.augustus.g38671.t1/1-1890	0.380457	0.0468289	RNA-directed DNA polymerase from mobile element jockey-like				
Acrdi.XM_015920298.1/1-2904	Monca.adi2mcaRNA31556_R0/1-2847	0.0670209	0.0467937	Exportin-2	6	C:GO:0005635; P:GO:0006606; C:GO:0005829; P:GO:0006611;	C:nuclear envelope; P:protein import into nucleus; C:cytosol; P:protein export from nucleus; F:Ran GTPase binding; F:nuclear export signal receptor activity	
Acrdi.XM_015924050.1/1-666	Monca.adi2mcaRNA35786_R0/1-537	0.0514684	0.0467796	replication factor C subunit 5-like	3	F:GO:0003677; F:GO:0005524; P:GO:0006260	F:DNA binding; F:ATP binding; P:DNA replication	
Acrdi.XM_015898269.1/1-927	Monca.augustus.g64607.t1/1-1215	0.0856939	0.0466967	SEC13 homolog	10	P:GO:0006888; P:GO:0036372; P:GO:0001889; P:GO:0010842;	P:ER to Golgi vesicle-mediated transport; P:opsin transport; P:liver development; P:retina layer formation; P:eye photoreceptor cell development; P:digestive system development; [illegible]	
Acrdi.XM_015905572.1/1-1362	Monca.augustus.g24094.t1/1-1362	0.0379763	0.0466459	mab-21-like 2				
Acrdi.XM_015894357.1/1-759	Monca.augustus.g62941.t1/1-1035	0.498612	0.0466404	PREDICTED: uncharacterized protein LOC107329691				
Acrdi.XM_015899631.1/1-408	Monca.augustus.g14871.t1/1-408	0.0584626	0.046622	PREDICTED: uncharacterized protein C20orf85-like				
Acrdi.XM_015903338.1/1-1806	Monca.augustus.g29880.t1/1-1851	0.055011	0.0465984	rac GTPase-activating 1	25	P:GO:0000915; C:GO:0032154; P:GO:0007275; F:GO:0005096;	P:actomyosin contractile ring assembly; C:cleavage furrow; P:multicellular organism development; F:GTPase activator activity; C:nucleoplasm; F:phosphatidylinositol-3,4,5-trispho [illegible]	
Acrdi.XM_015896698.1/1-723	Monca.augustus.g21907.t1/1-735	0.0557607	0.0465803	exosome complex component RRP41-like	12	C:GO:0000177; C:GO:0000176; P:GO:0090305; P:GO:0071051;	C:cytoplasmic exosome (RNase complex); C:nuclear exosome (RNase complex); P:nucleic acid phosphodiester bond hydrolysis; P:polyadenylation-dependent snoRNA 3'-end p	EC:2.[illegible]
Acrdi.XM_015922116.1/1-675	Monca.adi2mcaRNA33538_R4/1-675	0.430329	0.0465558	Pro-Pol poly				
Acrdi.XM_015919551.1/1-1575	Monca.augustus.g19276.t1/1-1881	0.0662336	0.0465508	ABC transport system ATP-binding	3	F:GO:0005524; F:GO:0004452; F:GO:0016853	F:ATP binding; F:isopentenyl-diphosphate delta-isomerase activity; F:isomerase activity	
Acrdi.XM_015905887.1/1-2310	Monca.augustus.g58498.t1/1-2403	0.0542415	0.0465477	histone acetyltransferase KAT2A				
Acrdi.XM_015903969.1/1-897	Monca.adi2mcaRNA13436_R0/1-897	0.730499	0.0465208	RNA-directed DNA polymerase from transposon BS				
Acrdi.XM_015915141.1/1-519	Monca.augustus.g40283.t1/1-465	0.557194	0.0465154	Proteasome subunit beta type-2	5	C:GO:0005634; C:GO:0005737; C:GO:0005839; F:GO:0004298;	C:nucleus; C:cytoplasm; C:proteasome core complex; F:threonine-type endopeptidase activity; P:proteolysis involved in cellular protein catabolic process	EC:3.[illegible]
Acrdi.XM_015891788.1/1-1122	Monca.adi2mcaRNA37650_R5/1-990	0.379993	0.0464795	endogenous retrovirus group K member 7 Pol -like				
Acrdi.XM_015921804.1/1-3288	Monca.augustus.g68733.t1/1-4743	0.0514602	0.0464763	global transcription activator	6	F:GO:0016887; C:GO:0016514; F:GO:0005524; P:GO:0006338;	F:ATPase activity; C:SWI/SNF complex; F:ATP binding; P:chromatin remodeling; F:histone binding; P:regulation of transcription, DNA-templated	EC:3.[illegible]
Acrdi.XM_015923281.1/1-681	Monca.augustus.g41209.t1/1-678	0.113836	0.0464163	homeobox otx5-A-like	3	C:GO:0005634; P:GO:0006355; F:GO:0043565	C:nucleus; P:regulation of transcription, DNA-templated; F:sequence-specific DNA binding	
Acrdi.XM_015922533.1/1-2376	Monca.adi2mcaRNA34008_R0/1-1833	0.547145	0.0463791	Retrovirus-related Pol poly from transposon 412				
Acrdi.XM_015923541.1/1-1479	Monca.adi2mcaRNA35207_R3/1-1590	0.289943	0.0463466	PREDICTED: uncharacterized protein LOC107356911				
Acrdi.XM_015919822.1/1-501	Monca.augustus.g69878.t1/1-963	0.0574151	0.0463252	SAGA-associated factor 29				
Acrdi.XM_015916428.1/1-1767	Monca.augustus.g64474.t1/1-2010	0.0506544	0.0463226	arachidonate 5-lipoxygenase-like	3	F:GO:0051213; F:GO:0046872; P:GO:0044710	F:dioxygenase activity; F:metal ion binding; P:single-organism metabolic process	
Acrdi.XM_015897195.1/1-1248	Monca.adi2mcaRNA6053_R1/1-1206	0.55156	0.0462962	PREDICTED: uncharacterized protein LOC107332460				
Acrdi.XM_015916245.1/1-2748	Monca.augustus.g35030.t1/1-2817	0.0624162	0.0462918	metastasis-associated MTA3-like	19	F:GO:0008270; P:GO:0006355; C:GO:0044444; F:GO:0003682;	F:zinc ion binding; P:regulation of transcription, DNA-templated; C:cytoplasmic part; F:chromatin binding; P:double-strand break repair; F:core promoter sequence-specific DNA bi [illegible]	
Acrdi.XM_015898169.1/1-2091	Monca.augustus.g29278.t1/1-1935	0.0621945	0.0462531	heat shock cognate 71 kDa	1	F:GO:0005524	F:ATP binding	
Acrdi.XM_015915863.1/1-750	Monca.augustus.g56364.t1/1-738	0.0825132	0.0462229	inhibitor of growth 4 isoform X3	17	P:GO:0007050; P:GO:0045926; P:GO:0043966; F:GO:0035064;	P:cell cycle arrest; P:negative regulation of growth; P:histone H3 acetylation; F:methylated histone binding; P:DNA replication; P:negative regulation of cell proliferation; P:histone [illegible]	
Acrdi.XM_015900455.1/1-603	Monca.adi2mcaRNA9627_R1/1-597	0.197447	0.0462206	PREDICTED: uncharacterized protein LOC107335436				
Acrdi.XM_015904595.1/1-1611	Monca.augustus.g35723.t1/1-1212	0.511124	0.0462172	THAP domain-containing 11				
Acrdi.XM_015918909.1/1-1365	Monca.adi2mcaRNA30034_R0/1-1356	0.724561	0.04618	ATP-dependent DNA helicase PIF1				
Acrdi.XM_015920823.1/1-612	Monca.augustus.g49851.t1/1-633	0.0581246	0.046176	Mediator of RNA polymerase II transcription subunit 20	4	F:GO:0003713; C:GO:0016592; F:GO:0001104; P:GO:0006357	F:transcription coactivator activity; C:mediator complex; F:RNA polymerase II transcription cofactor activity; P:regulation of transcription from RNA polymerase II promoter	
Acrdi.XM_015901756.1/1-657	Monca.augustus.g6409.t1/1-669	0.0832584	0.0461737	bcl-2 homologous antagonist killer	12	F:GO:0042803; P:GO:0048731; F:GO:0046982; P:GO:0048872;	F:protein homodimerization activity; P:system development; F:protein heterodimerization activity; P:homeostasis of number of cells; P:positive regulation of apoptotic process; C: [illegible]	
Acrdi.XM_015919978.1/1-495	Monca.augustus.g60886.t1/1-495	0.0672772	0.0461653	ATP synthase subunit mitochondrial	4	C:GO:0000275; F:GO:0046961; P:GO:0015986; F:GO:0046933	C:mitochondrial proton-transporting ATP synthase complex, catalytic core F(1); F:proton-transporting ATPase activity, rotational mechanism; P:ATP synthesis coupled proton t	EC:3.[illegible]
Acrdi.XM_015895394.1/1-603	Monca.augustus.g14603.t1/1-3078	0.0491414	0.0461078	histone acetyltransferase KAT6B-	8	C:GO:0005634; F:GO:0003677; F:GO:0004402; F:GO:0008270;	C:nucleus; F:DNA binding; F:histone acetyltransferase activity; F:zinc ion binding; P:regulation of transcription, DNA-templated; P:histone acetylation; C:nucleosome; P:nucleo	EC:2.[illegible]
Acrdi.XM_015917737.1/1-1251	Monca.augustus.g1559.t1/1-2550	0.0416935	0.0460918	tyrosine kinase receptor Cad96Ca-like	3	F:GO:0005524; P:GO:0018108; F:GO:0004713	F:ATP binding; P:peptidyl-tyrosine phosphorylation; F:protein tyrosine kinase activity	EC:2.[illegible]
Acrdi.XM_015905961.1/1-1650	Monca.augustus.g69494.t1/1-1647	0.0703881	0.0460779	major facilitator superfamily domain-containing 6	1	C:GO:0016020	C:membrane	
Acrdi.XM_015908384.1/1-684	Monca.augustus.g34805.t1/1-897	0.0792197	0.0460731	zinc finger C3H1 type-like 1	1	F:GO:0046872	F:metal ion binding	
Acrdi.XM_015924447.1/1-1686	Monca.adi2mcaRNA36240_R6/1-1422	0.263224	0.0460581	Gag-Pol poly				
Acrdi.XM_015893777.1/1-1350	Monca.augustus.g532.t1/1-1344	0.0398539	0.046046	transcriptional regulator ERG isoform X1	5	F:GO:0003677; C:GO:0005634; F:GO:0003700; P:GO:0006355;	F:DNA binding; C:nucleus; F:transcription factor activity, sequence-specific DNA binding; P:regulation of transcription, DNA-templated; F:sequence-specific DNA binding	
Acrdi.XM_015910141.1/1-396	Monca.augustus.g18226.t1/1-1266	0.0440816	0.0459949	BRCA1-A complex subunit Abraxas	7	P:GO:0045739; C:GO:0005634; F:GO:0031593; C:GO:0070531;	P:positive regulation of DNA repair; C:nucleus; F:polyubiquitin binding; C:BRCA1-A complex; P:G2 DNA damage checkpoint; P:response to ionizing radiation; P:double-strand break [illegible]	
Acrdi.XM_015901859.1/1-1686	Monca.adi2mcaRNA11158_R0/1-1695	0.0443688	0.0459831	E3 ubiquitin- ligase RNF19A	10	C:GO:0005813; F:GO:0031624; F:GO:0061630; C:GO:0000151;	C:centrosome; F:ubiquitin conjugating enzyme binding; F:ubiquitin protein ligase activity; C:ubiquitin ligase complex; C:cytoplasm; F:metal ion binding; P:protein polyubiquitination [illegible]	
Acrdi.XM_015910501.1/1-702	Monca.augustus.g25414.t1/1-861	0.0742412	0.0459654	YIPF5	1	C:GO:0016020	C:membrane	
Acrdi.XM_015918371.1/1-1944	Monca.adi2mcaRNA29406_R0/1-1752	0.0628766	0.0459446	Elongation factor mitochondrial	5	F:GO:0000166; F:GO:0003746; F:GO:0003924; C:GO:0005739;	F:nucleotide binding; F:translation elongation factor activity; F:GTPase activity; C:mitochondrion; P:mitochondrial translational elongation	EC:3.[illegible]
Acrdi.XM_015903610.1/1-1590	Monca.augustus.g24957.t1/1-1587	0.0634573	0.0459357	Major facilitator superfamily domain-containing 6	1	C:GO:0016021	C:integral component of membrane	
Acrdi.XM_015915363.1/1-1221	Monca.adi2mcaRNA26073_R0/1-1053	0.0445975	0.0458704	Vacuolar sorting-associated 45	2	P:GO:0016192; P:GO:0006904	P:vesicle-mediated transport; P:vesicle docking involved in exocytosis	
Acrdi.XM_015915367.1/1-1071	Monca.augustus.g30188.t1/1-1053	0.0563385	0.0458515	Insulin gene enhancer isl-1	57	F:GO:0016922; P:GO:0021559; P:GO:0055010; F:GO:0000978;	F:ligand-dependent nuclear receptor binding; P:trigeminal nerve development; P:ventricular cardiac muscle tissue morphogenesis; F:RNA polymerase II core promoter proximal re [illegible]	
Acrdi.XM_015904184.1/1-1203	Monca.augustus.g17131.t1/1-1362	0.0522039	0.0458487	fizzy-related homolog	3	F:GO:0097027; P:GO:0050790; F:GO:0010997	F:ubiquitin-protein transferase activator activity; P:regulation of catalytic activity; F:anaphase-promoting complex binding	
Acrdi.XM_015919260.1/1-4227	Monca.augustus.g17816.t1/1-3747	0.0525403	0.0458309	Centrosomal of 135 kDa				
Acrdi.XM_015925104.1/1-1500	Monca.augustus.g51155.t1/1-1500	0.0472318	0.0458096	TNF receptor-associated factor 4	3	F:GO:0004842; F:GO:0008270; P:GO:0016567	F:ubiquitin-protein transferase activity; F:zinc ion binding; P:protein ubiquitination	
Acrdi.XM_015909841.1/1-3975	Monca.augustus.g46894.t1/1-4419	0.0500926	0.0457967	PREDICTED: uncharacterized protein LOC107344197				
Acrdi.XM_015915033.1/1-1221	Monca.augustus.g8800.t1/1-1212	0.0646743	0.0457954	Prostaglandin E2 receptor EP4 subtype	13	P:GO:0007186; P:GO:0007165; F:GO:0009881; P:GO:0007166;	P:G-protein coupled receptor signaling pathway; P:signal transduction; F:photoreceptor activity; P:cell surface receptor signaling pathway; P:visual perception; F:signal transducer [illegible]	
Acrdi.XM_015924995.1/1-567	Monca.augustus.g69345.t1/1-990	0.40347	0.0457709	Transposon Ty3-G Gag-Pol poly				
Acrdi.XM_015913112.1/1-1761	Monca.adi2mcaRNA23502_R0/1-1737	0.0724539	0.045641	E3 ubiquitin- ligase TRIM71-like	1	F:GO:0046872	F:metal ion binding	
Acrdi.XM_015901071.1/1-2007	Monca.augustus.g57654.t1/1-1923	0.0554552	0.0455862	sodium myo-inositol cotransporter-like	3	C:GO:0005887; P:GO:0055085; F:GO:0015293	C:integral component of plasma membrane; P:transmembrane transport; F:symporter activity	
Acrdi.XM_015915699.1/1-1368	Monca.adi2mcaRNA26413_R0/1-1368	0.0493715	0.0455738	serine threonine- kinase 38-like	8	F:GO:0000287; F:GO:0005524; C:GO:0005737; C:GO:0015629;	F:magnesium ion binding; F:ATP binding; C:cytoplasm; C:actin cytoskeleton; F:protein serine/threonine kinase activity; P:peptidyl-serine phosphorylation; P:intracellular signal	EC:2.[illegible]
Acrdi.XM_015903246.1/1-1869	Monca.augustus.g51156.t1/1-1869	0.0583993	0.0455607	TNF receptor-associated factor 4	3	F:GO:0004842; F:GO:0008270; P:GO:0016567	F:ubiquitin-protein transferase activity; F:zinc ion binding; P:protein ubiquitination	
Acrdi.XM_015911563.1/1-450	Monca.adi2mcaRNA21801_R0/1-444	0.0557313	0.0455593	trafficking particle complex subunit 2	4	P:GO:0006888; C:GO:0043231; C:GO:0012505; C:GO:0044444	P:ER to Golgi vesicle-mediated transport; C:intracellular membrane-bounded organelle; C:endomembrane system; C:cytoplasmic part	
Acrdi.XM_015924855.1/1-2751	Monca.augustus.g6745.t1/1-3471	0.0643743	0.0455263	probable phospholipid-transporting ATPase IIB isoform X2	8	F:GO:0000287; F:GO:0005524; C:GO:0005802; C:GO:0048471;	F:magnesium ion binding; F:ATP binding; C:trans-Golgi network; C:perinuclear region of cytoplasm; P:phospholipid translocation; C:integral component of membrane; F:phosp	EC:3.[illegible]
Acrdi.XM_015899814.1/1-1836	Monca.augustus.g48632.t1/1-1869	0.0530536	0.0455222	dynein intermediate chain axonemal	5	C:GO:0005737; C:GO:0005929; C:GO:0030286; C:GO:0005874;	C:cytoplasm; C:cilium; C:dynein complex; C:microtubule; F:motor activity	EC:3.[illegible]
Acrdi.XM_015924149.1/1-1338	Monca.augustus.g62389.t1/1-1338	0.0690645	0.0455149	Dolichyl-diphosphooligosaccharide-- glycosyltransferase 48 kDa subuni	7	C:GO:0005789; F:GO:0004579; P:GO:0034097; C:GO:0016021;	C:endoplasmic reticulum membrane; F:dolichyl-diphosphooligosaccharide-protein glycotransferase activity; P:response to cytokine; C:integral component of membrane; P:T c	EC:2.[illegible]
Acrdi.XM_015919322.1/1-2307	Monca.augustus.g5825.t1/1-2304	0.0587323	0.045514	AFG3 2	11	P:GO:0045041; F:GO:0005524; F:GO:0008270; P:GO:0042407;	P:protein import into mitochondrial intermembrane space; F:ATP binding; F:zinc ion binding; P:cristae formation; P:protein complex assembly; P:mitochondrial fusion; C:integ	EC:3.[illegible]
Acrdi.XM_015894303.1/1-1053	Monca.adi2mcaRNA40549_R1/1-1134	0.25512	0.0454908	RNA-directed DNA polymerase from mobile element jockey-like				
Acrdi.XM_015918288.1/1-1029	Monca.adi2mcaRNA29304_R8/1-960	0.415404	0.0454517	CTD kinase subunit alpha-like				
Acrdi.XM_015911107.1/1-654	Monca.augustus.g7160.t1/1-654	0.076208	0.0454223	PREDICTED: uncharacterized protein LOC107345403				
Acrdi.XM_015910038.1/1-1098	Monca.augustus.g36876.t1/1-1761	0.0741498	0.0454136	polypeptide N-acetylgalactosaminyltransferase	6	F:GO:0016757; P:GO:0018193; P:GO:0006493; C:GO:0016021;	F:transferase activity, transferring glycosyl groups; P:peptidyl-amino acid modification; P:protein O-linked glycosylation; C:integral component of membrane; C:Golgi membrane; F [illegible]	
Acrdi.XM_015921348.1/1-8778	Monca.augustus.g49922.t1/1-15261	0.0644752	0.0453451	E3 ubiquitin- ligase UBR4				
Acrdi.XM_015922948.1/1-897	Monca.adi2mcaRNA34536_R0/1-1029	0.849475	0.0453202	PREDICTED: uncharacterized protein LOC107356332				
Acrdi.XM_015915856.1/1-1392	Monca.augustus.g56363.t1/1-2913	0.060474	0.0453063	rap1 GTPase-activating 1-like isoform X4				
Acrdi.XM_015910578.1/1-804	Monca.augustus.g10109.t1/1-807	0.0450849	0.045256	band 7 AGAP004871-like isoform X2	1	C:GO:0005887	C:integral component of plasma membrane	
Acrdi.XM_015911412.1/1-531	Monca.augustus.g4351.t1/1-537	0.0538606	0.0452195	PREDICTED: uncharacterized protein LOC107345674				
Acrdi.XM_015923187.1/1-1047	Monca.augustus.g49872.t1/1-1044	0.0609322	0.0452039	Fez family zinc finger 2	13	F:GO:0008270; P:GO:0006351; P:GO:0021879; P:GO:0008593;	F:zinc ion binding; P:transcription, DNA-templated; P:forebrain neuron differentiation; P:regulation of Notch signaling pathway; P:hypothalamus development; F:transcriptional re [illegible]	
Acrdi.XM_015895992.1/1-684	Monca.augustus.g51367.t1/1-684	0.0693334	0.0451641	GATA zinc finger domain-containing 1	4	F:GO:0003700; F:GO:0008270; P:GO:0006355; F:GO:0043565	F:transcription factor activity, sequence-specific DNA binding; F:zinc ion binding; P:regulation of transcription, DNA-templated; F:sequence-specific DNA binding	
Acrdi.XM_015919972.1/1-1245	Monca.augustus.g16008.t1/1-1245	0.0465869	0.0451364	phosphoglycerate kinase 1	3	F:GO:0005524; F:GO:0004618; P:GO:0006096	F:ATP binding; F:phosphoglycerate kinase activity; P:glycolytic process	EC:2.[illegible]
Acrdi.XM_015902283.1/1-1533	Monca.adi2mcaRNA11601_R0/1-1536	0.0474378	0.0450863	proto-oncogene tyrosine- kinase Yrk-like isoform X1	4	F:GO:0005524; P:GO:0018108; F:GO:0004715; P:GO:0045087	F:ATP binding; P:peptidyl-tyrosine phosphorylation; F:non-membrane spanning protein tyrosine kinase activity; P:innate immune response	EC:2.[illegible]
Acrdi.XM_015906922.1/1-354	Monca.augustus.g49156.t1/1-549	0.0404719	0.0450691	Vacuolar sorting-associated 29	6	C:GO:0005768; C:GO:0030904; C:GO:0005829; F:GO:0008565;	C:endosome; C:retromer complex; C:cytosol; F:protein transporter activity; P:intracellular protein transport; P:retrograde transport, endosome to Golgi	
Acrdi.XM_015913553.1/1-924	Monca.augustus.g58014.t1/1-924	0.0398862	0.0450654	probable dimethyladenosine transferase	3	C:GO:0005759; P:GO:0031167; F:GO:0000179	C:mitochondrial matrix; P:rRNA methylation; F:rRNA (adenine-N6,N6-)-dimethyltransferase activity	
Acrdi.XM_015915238.1/1-633	Monca.augustus.g30641.t1/1-1113	0.0598772	0.0450513	LIM domain-binding 2-like	4	C:GO:0005634; F:GO:0000989; F:GO:0030274; P:GO:0006357	C:nucleus; F:transcription factor activity, transcription factor binding; F:LIM domain binding; P:regulation of transcription from RNA polymerase II promoter	
Acrdi.XM_015895724.1/1-387	Monca.augustus.g64083.t1/1-810	0.0576416	0.0450246	Solute carrier family 35 member E3	4	F:GO:0005338; P:GO:1901264; C:GO:0016021; P:GO:1901679	F:nucleotide-sugar transmembrane transporter activity; P:carbohydrate derivative transport; C:integral component of membrane; P:nucleotide transmembrane transport	
Acrdi.XM_015914268.1/1-1383	Monca.adi2mcaRNA24840_R0/1-1383	0.067991	0.0449701	alpha-aminoadipic semialdehyde dehydrogenase isoform X2				
Acrdi.XM_015908429.1/1-630	Monca.adi2mcaRNA18407_R2/1-471	0.21595	0.0449565	Transposon Tf2-6 poly				
Acrdi.XM_015913682.1/1-750	Monca.augustus.g30464.t1/1-837	0.0659119	0.0448883	docking 3	1	F:GO:0005158	F:insulin receptor binding	
Acrdi.XM_015892159.1/1-453	Monca.augustus.g3421.t1/1-519	0.0515065	0.0448758	39S ribosomal mitochondrial isoform X1	5	F:GO:0003735; F:GO:0019843; P:GO:0000027; C:GO:0005762;	F:structural constituent of ribosome; F:rRNA binding; P:ribosomal large subunit assembly; C:mitochondrial large ribosomal subunit; P:translation	
Acrdi.XM_015909214.1/1-963	Monca.augustus.g49341.t1/1-1014	0.0561328	0.0448521	YIF1B isoform X1	1	C:GO:0016021	C:integral component of membrane	
Acrdi.XM_015902331.1/1-1077	Monca.adi2mcaRNA11693_R0/1-900	0.373417	0.0447762	Ankyrin repeat domain-containing 34B				
Acrdi.XM_015914145.1/1-1671	Monca.augustus.g24977.t1/1-1731	0.0636383	0.0447501	potassium voltage-gated channel subfamily A member 2-like	5	P:GO:0071805; C:GO:0008076; F:GO:0005251; P:GO:0034765;	P:potassium ion transmembrane transport; C:voltage-gated potassium channel complex; F:delayed rectifier potassium channel activity; P:regulation of ion transmembrane transp [illegible]	
Acrdi.XM_015906779.1/1-3018	Monca.augustus.g60651.t1/1-3090	0.0541881	0.0447277	importin-7 isoform X1	11	C:GO:0005635; F:GO:0046332; F:GO:0042393; P:GO:0007165;	C:nuclear envelope; F:SMAD binding; F:histone binding; P:signal transduction; P:protein import into nucleus; P:innate immune response; C:membrane; C:cytosol; F:protein transpor [illegible]	
Acrdi.XM_015911838.1/1-573	Monca.augustus.g6323.t1/1-1236	0.0485792	0.0446697	RAB6A-GEF complex partner 2-like isoform X1				
Acrdi.XM_015910218.1/1-2064	Monca.augustus.g25496.t1/1-2034	0.067489	0.0446209	PAB-dependent poly(A)-specific ribonuclease subunit PAN3-like isoforr	6	F:GO:0005524; C:GO:0031251; P:GO:0010606; C:GO:0000932;	F:ATP binding; C:PAN complex; P:positive regulation of cytoplasmic mRNA processing body assembly; C:P-body; P:mRNA processing; P:nuclear-transcribed mRNA poly(A) tail short [illegible]	
Acrdi.XM_015907105.1/1-402	Monca.augustus.g36985.t1/1-405	0.0586109	0.0445829	39S ribosomal mitochondrial	6	F:GO:0000166; F:GO:0003723; F:GO:0003735; P:GO:0032543;	F:nucleotide binding; F:RNA binding; F:structural constituent of ribosome; P:mitochondrial translation; C:nucleolus; C:mitochondrial large ribosomal subunit	
Acrdi.XM_015903506.1/1-2148	Monca.adi2mcaRNA1458_R3/1-2106	0.435218	0.0445186	RNA-directed DNA polymerase from mobile element jockey-like				
Acrdi.XM_015911690.1/1-783	Monca.augustus.g50294.t1/1-576	0.542075	0.0445126	UPF0746 DDB_G0281095-like				
Acrdi.XM_015899406.1/1-4644	Monca.augustus.g37223.t1/1-5373	0.0595632	0.0444947	intraflagellar transport 172 homolog	13	C:GO:0036064; P:GO:1902589; P:GO:0007166; P:GO:0003002;	C:ciliary basal body; P:single-organism organelle organization; P:cell surface receptor signaling pathway; P:regionalization; C:axoneme; P:determination of left/right symmetry; P [illegible]	
Acrdi.XM_015910692.1/1-690	Monca.augustus.g35883.t1/1-741	0.0597633	0.0444499	transcription cofactor vestigial 4 isoform X2	1	P:GO:0006355	P:regulation of transcription, DNA-templated	
Acrdi.XM_015915013.1/1-1761	Monca.augustus.g66532.t1/1-1704	0.0476066	0.0444218	cell division control 45 homolog	14	P:GO:0006270; P:GO:1900087; P:GO:0000727; C:GO:0031261;	P:DNA replication initiation; P:positive regulation of G1/S transition of mitotic cell cycle; P:double-strand break repair via break-induced replication; C:DNA replication preiniti	EC:3.[illegible]
Acrdi.XM_015925303.1/1-969	Monca.augustus.g53774.t1/1-1533	0.0723942	0.0444167	Bardet-Biedl syndrome 4	1	C:GO:0034464	C:BBSome	
Acrdi.XM_015908654.1/1-1275	Monca.adi2mcaRNA18655_R0/1-1275	0.0749873	0.0443898	lipoxygenase homology domain-containing 1-like	6	P:GO:0007605; P:GO:0050982; F:GO:0005262; C:GO:0016020;	P:sensory perception of sound; P:detection of mechanical stimulus; F:calcium channel activity; C:membrane; P:calcium ion transmembrane transport; C:stereocilium	
Acrdi.XM_015896590.1/1-1269	Monca.augustus.g62595.t1/1-1263	0.0729798	0.0443532	WD repeat and FYVE domain-containing 2	1	F:GO:0046872	F:metal ion binding	
Acrdi.XM_015913871.1/1-801	Monca.augustus.g61732.t1/1-1497	0.856779	0.0443496	Retrotransposable element Tf2 type 1	3	F:GO:0003676; P:GO:0015074; C:GO:0005575	F:nucleic acid binding; P:DNA integration; C:cellular_component	
Acrdi.XM_015918630.1/1-1491	Monca.adi2mcaRNA29697_R0/1-1539	0.0505317	0.0443434	kinesin KIF15	7	F:GO:0016887; F:GO:0005524; F:GO:0003777; C:GO:0005871;	F:ATPase activity; F:ATP binding; F:microtubule motor activity; C:kinesin complex; P:microtubule-based movement; C:microtubule; F:microtubule binding	EC:3.[illegible]
Acrdi.XM_015893351.1/1-966	Monca.augustus.g61758.t1/1-966	0.0493233	0.0443277	E3 ubiquitin- ligase NRDP1	15	F:GO:0008270; P:GO:1902531; P:GO:0000209; P:GO:0097191;	F:zinc ion binding; P:regulation of intracellular signal transduction; P:protein polyubiquitination; P:extrinsic apoptotic signaling pathway; P:negative regulation of cellular process; [illegible]	
Acrdi.XM_015891627.1/1-1740	Monca.adi2mcaRNA37471_R1/1-1590	0.250976	0.044323	ATP-dependent DNA helicase -like				
Acrdi.XM_015896555.1/1-1218	Monca.adi2mcaRNA5413_R0/1-1218	0.0435278	0.0443094	interleukin enhancer-binding factor 2	8	F:GO:0003677; F:GO:0005524; P:GO:0006955; F:GO:0003725;	F:DNA binding; F:ATP binding; P:immune response; F:double-stranded RNA binding; P:transcription, DNA-templated; P:positive regulation of transcription, DNA-templated; C:nucle [illegible]	
Acrdi.XM_015898099.1/1-549	Monca.augustus.g9832.t1/1-552	0.0746981	0.0443023	GTPase HRas	33	P:GO:0007050; P:GO:0008283; P:GO:0043406; P:GO:0007093;	P:cell cycle arrest; P:cell proliferation; P:positive regulation of MAP kinase activity; P:mitotic cell cycle checkpoint; F:protein C-terminus binding; P:negative regulation of cell p	EC:3.[illegible]
Acrdi.XM_015905957.1/1-1707	Monca.augustus.g69502.t1/1-1707	0.0568468	0.0442782	kelch 18	3	F:GO:0004842; C:GO:0031463; P:GO:0016567	F:ubiquitin-protein transferase activity; C:Cul3-RING ubiquitin ligase complex; P:protein ubiquitination	
Acrdi.XM_015920985.1/1-879	Monca.augustus.g42000.t1/1-876	0.05363	0.0442634	LIX1	2	P:GO:0097352; C:GO:0005737	P:autophagosome maturation; C:cytoplasm	
Acrdi.XM_015898730.1/1-381	Monca.augustus.g54236.t1/1-381	0.085455	0.044228	Profilin allergen	4	P:GO:0042989; C:GO:0005938; C:GO:0015629; F:GO:0003785	P:sequestering of actin monomers; C:cell cortex; C:actin cytoskeleton; F:actin monomer binding	
Acrdi.XM_015917504.1/1-1143	Monca.adi2mcaRNA28417_R0/1-1425	0.389214	0.0442236	PREDICTED: uncharacterized protein LOC107351207 isoform X2				
Acrdi.XM_015903598.1/1-1683	Monca.adi2mcaRNA13008_R0/1-1677	0.0578945	0.0442155	oocyte-specific F-box	2	F:GO:0042393; F:GO:0098769	F:histone binding; F:TIMP family protein binding	
Acrdi.XM_015908375.1/1-1572	Monca.augustus.g34820.t1/1-1548	0.116553	0.0441782	homeobox Meis1 isoform X2	4	C:GO:0005634; F:GO:0003677; P:GO:0006355; P:GO:0048513	C:nucleus; F:DNA binding; P:regulation of transcription, DNA-templated; P:animal organ development	
Acrdi.XM_015903249.1/1-2016	Monca.augustus.g65480.t1/1-3093	0.070846	0.044167	unconventional myosin-Ic-like isoform X1	4	C:GO:0016459; F:GO:0005524; F:GO:0003779; F:GO:0003774	C:myosin complex; F:ATP binding; F:actin binding; F:motor activity	EC:3.[illegible]
Acrdi.XM_015916357.1/1-795	Monca.augustus.g32902.t1/1-882	0.470357	0.0441664	PREDICTED: uncharacterized protein LOC107350133				
Acrdi.XM_015894032.1/1-375	Monca.augustus.g36766.t1/1-738	0.0700223	0.0441581	regulator complex LAMTOR3	6	F:GO:0032947; P:GO:0071230; F:GO:0005085; C:GO:0071986;	F:protein complex scaffold; P:cellular response to amino acid stimulus; F:guanyl-nucleotide exchange factor activity; C:Ragulator complex; P:positive regulation of TOR signaling; [illegible]	
Acrdi.XM_015925207.1/1-936	Monca.adi2mcaRNA37122_R1/1-1152	0.256348	0.0441468	RNA-directed DNA polymerase from mobile element jockey				
Acrdi.XM_015898303.1/1-1191	Monca.augustus.g61849.t1/1-1362	0.0661597	0.0441177	26S proteasome non-ATPase regulatory subunit 12	4	P:GO:0043161; C:GO:0005737; C:GO:0008541; C:GO:0031595	P:proteasome-mediated ubiquitin-dependent protein catabolic process; C:cytoplasm; C:proteasome regulatory particle, lid subcomplex; C:nuclear proteasome complex	
Acrdi.XM_015908756.1/1-372	Monca.augustus.g26278.t1/1-375	0.0462793	0.0441089	probable 28S ribosomal mitochondrial	4	F:GO:0003735; P:GO:0032543; C:GO:0005763; F:GO:0070181	F:structural constituent of ribosome; P:mitochondrial translation; C:mitochondrial small ribosomal subunit; F:small ribosomal subunit rRNA binding	
Acrdi.XM_015905456.1/1-2097	Monca.augustus.g29186.t1/1-1728	0.0640442	0.0440789	Major facilitator superfamily domain-containing 4	1	C:GO:0016021	C:integral component of membrane	
Acrdi.XM_015900814.1/1-411	Monca.augustus.g11796.t1/1-339	0.04967	0.0440734	homolog isoform X2	2	C:GO:0016021; P:GO:0010038	C:integral component of membrane; P:response to metal ion	
Acrdi.XM_015901024.1/1-417	Monca.augustus.g39843.t1/1-417	0.13372	0.0440729	40S ribosomal S12-like	3	F:GO:0003735; C:GO:0022627; P:GO:0006412	F:structural constituent of ribosome; C:cytosolic small ribosomal subunit; P:translation	
Acrdi.XM_015896885.1/1-1644	Monca.augustus.g2336.t1/1-1170	0.0422962	0.0440617	high affinity cGMP-specific 3 ,5 -cyclic phosphodiesterase 9A-like	3	F:GO:0046872; P:GO:0007165; F:GO:0004114	F:metal ion binding; P:signal transduction; F:3',5'-cyclic-nucleotide phosphodiesterase activity	EC:3.[illegible]
Acrdi.XM_015906336.1/1-2535	Monca.adi2mcaRNA16049_R0/1-2580	0.0616997	0.0440522	endoplasmin	5	F:GO:0005524; P:GO:0001666; P:GO:0035889; F:GO:0051082;	F:ATP binding; P:response to hypoxia; P:otolith tethering; F:unfolded protein binding; P:protein folding	
Acrdi.XM_015924780.1/1-2199	Monca.adi2mcaRNA36639_R0/1-2043	0.380648	0.044051	Transmembrane protease serine 13				
Acrdi.XM_015918441.1/1-2064	Monca.augustus.g55075.t1/1-2154	0.059895	0.0440504	Bardet-Biedl syndrome 7	16	P:GO:0060255; C:GO:0034464; C:GO:0036064; P:GO:0015031;	P:regulation of macromolecule metabolic process; C:BBSome; C:ciliary basal body; P:protein transport; P:cell projection organization; C:axoneme; P:gastrulation; C:cytoplasm; C:me [illegible]	
Acrdi.XM_015924747.1/1-1593	Monca.adi2mcaRNA36606_R4/1-1194	0.228266	0.0440178	Retrovirus-related Pol poly from transposon	3	P:GO:0043170; F:GO:0005488; P:GO:0044238	P:macromolecule metabolic process; F:binding; P:primary metabolic process	
Acrdi.XM_015918088.1/1-1365	Monca.augustus.g43974.t1/1-1362	0.0635082	0.0439659	Isocitrate dehydrogenase [NADP] mitochondrial	9	F:GO:0000287; C:GO:0005777; C:GO:0070062; F:GO:0051287;	F:magnesium ion binding; C:peroxisome; C:extracellular exosome; F:NAD binding; F:isocitrate dehydrogenase (NADP+) activity; C:cytosol; P:isocitrate metabolic process; P:tric	EC:1.[illegible]
Acrdi.XM_015912258.1/1-1446	Monca.augustus.g28991.t1/1-1527	0.0659194	0.0439583	6-phosphogluconate decarboxylating-like	3	F:GO:0004616; P:GO:0006098; P:GO:0055114	F:phosphogluconate dehydrogenase (decarboxylating) activity; P:pentose-phosphate shunt; P:oxidation-reduction process	EC:1.[illegible]
Acrdi.XM_015898150.1/1-3054	Monca.augustus.g29270.t1/1-2988	0.0539445	0.0439492	anion exchange 2-like isoform X1	5	C:GO:0005887; P:GO:0051453; P:GO:0098656; F:GO:0008509;	C:integral component of plasma membrane; P:regulation of intracellular pH; P:anion transmembrane transport; F:anion transmembrane transporter activity; P:bicarbonate transp [illegible]	
Acrdi.XM_015918451.1/1-2022	Monca.augustus.g55062.t1/1-2016	0.0665687	0.0438987	cyclin-dependent kinase-like 2 isoform X4	3	F:GO:0005524; F:GO:0004674; P:GO:0006468	F:ATP binding; F:protein serine/threonine kinase activity; P:protein phosphorylation	EC:2.[illegible]
Acrdi.XM_015924983.1/1-450	Monca.adi2mcaRNA36867_R1/1-468	0.543897	0.0438982	PREDICTED: uncharacterized protein LOC107358378 isoform X1				
Acrdi.XM_015924263.1/1-648	Monca.adi2mcaRNA36018_R0/1-651	0.0659653	0.0438388	UPF0428 CXorf56 homolog				
Acrdi.XM_015922841.1/1-675	Monca.augustus.g1402.t1/1-675	0.0639453	0.0438289	glucose-induced degradation 8 homolog	1	C:GO:0005634	C:nucleus	
Acrdi.XM_015919362.1/1-1701	Monca.augustus.g23943.t1/1-1701	0.0488339	0.043784	dihydropyrimidinase-like isoform X3	2	F:GO:0016810; C:GO:0005737	F:hydrolase activity, acting on carbon-nitrogen (but not peptide) bonds; C:cytoplasm	
Acrdi.XM_015917811.1/1-633	Monca.augustus.g62346.t1/1-918	0.0401922	0.0437765	phosphatase PTC7 homolog	1	F:GO:0003824	F:catalytic activity	
Acrdi.XM_015919806.1/1-516	Monca.augustus.g40329.t1/1-516	0.027864	0.0437068	myosin regulatory light polypeptide 9	1	F:GO:0005509	F:calcium ion binding	
Acrdi.XM_015918137.1/1-924	Monca.augustus.g30020.t1/1-924	0.0555214	0.0436399	ADP,ATP carrier mitochondrial-like	6	P:GO:0055085; F:GO:0003735; C:GO:0016021; C:GO:0005743;	P:transmembrane transport; F:structural constituent of ribosome; C:integral component of membrane; C:mitochondrial inner membrane; F:transporter activity; P:translation	
Acrdi.XM_015923997.1/1-1503	Monca.augustus.g54035.t1/1-1503	0.0563037	0.0436267	Vang 1	2	P:GO:0007275; C:GO:0016021	P:multicellular organism development; C:integral component of membrane	
Acrdi.XM_015910242.1/1-909	Monca.augustus.g25489.t1/1-912	0.0513286	0.0435912	mortality factor 4 1 isoform X2	11	P:GO:0008283; P:GO:0043967; P:GO:0006338; C:GO:0035267;	P:cell proliferation; P:histone H4 acetylation; P:chromatin remodeling; C:NuA4 histone acetyltransferase complex; P:histone H2A acetylation; C:Sin3 complex; F:protein N-terminus [illegible]	
Acrdi.XM_015896261.1/1-699	Monca.augustus.g33731.t1/1-699	0.0738042	0.0435711	hornerin-like isoform X1	2	C:GO:0042151; F:GO:0030246	C:nematocyst; F:carbohydrate binding	
Acrdi.XM_015902317.1/1-765	Monca.augustus.g63802.t1/1-750	0.0449669	0.0435704	Golgi SNAP receptor complex member 1-like	12	P:GO:0006888; C:GO:0005801; P:GO:0006906; P:GO:0015031;	P:ER to Golgi vesicle-mediated transport; C:cis-Golgi network; P:vesicle fusion; P:protein transport; C:integral component of membrane; F:SNAP receptor activity; C:SNARE comple [illegible]	
Acrdi.XM_015918814.1/1-765	Monca.augustus.g43824.t1/1-1002	0.0514886	0.0435507	photoreceptor-specific nuclear receptor-like	9	C:GO:0005634; F:GO:0008270; F:GO:0004879; P:GO:0006351;	C:nucleus; F:zinc ion binding; F:RNA polymerase II transcription factor activity, ligand-activated sequence-specific DNA binding; P:transcription, DNA-templated; F:steroid hormone [illegible]	
Acrdi.XM_015917201.1/1-540	Monca.adi2mcaRNA28113_R0/1-405	0.0602641	0.043534	inhibitor of growth 1 isoform X3	5	C:GO:0005737; F:GO:0046872; F:GO:0035064; P:GO:0008285;	C:cytoplasm; F:metal ion binding; F:methylated histone binding; P:negative regulation of cell proliferation; C:nucleoplasm	
Acrdi.XM_015901636.1/1-2298	Monca.augustus.g60598.t1/1-1983	0.568593	0.0435048	tigger transposable element-derived 6-like				
Acrdi.XM_015922579.1/1-4077	Monca.augustus.g54985.t1/1-4080	0.054475	0.0434956	regulatory-associated of mTOR isoform X2	2	P:GO:0031929; C:GO:0031931	P:TOR signaling; C:TORC1 complex	
Acrdi.XM_015925323.1/1-771	Monca.adi2mcaRNA37263_R2/1-1104	0.632822	0.0434931	transposase HI-				
Acrdi.XM_015899144.1/1-2934	Monca.adi2mcaRNA8270_R7/1-2787	0.911745	0.0434926	PREDICTED: uncharacterized protein LOC107334216	2	C:GO:0016020; C:GO:0016021	C:membrane; C:integral component of membrane	
Acrdi.XM_015908749.1/1-1134	Monca.augustus.g55166.t1/1-1137	0.067972	0.043491	FAM46C-like				
Acrdi.XM_015901505.1/1-615	Monca.augustus.g20058.t1/1-615	0.190951	0.0434877	G2 M phase-specific E3 ubiquitin- ligase-like	2	F:GO:0004842; P:GO:0016567	F:ubiquitin-protein transferase activity; P:protein ubiquitination	
Acrdi.XM_015924680.1/1-1023	Monca.augustus.g2600.t1/1-1047	0.0702	0.0434856	cAMP-dependent kinase catalytic subunit alpha	3	F:GO:0005524; F:GO:0004691; P:GO:0006468	F:ATP binding; F:cAMP-dependent protein kinase activity; P:protein phosphorylation	EC:2.[illegible]
Acrdi.XM_015908021.1/1-684	Monca.augustus.g5052.t1/1-681	0.0808081	0.0434694	Vesicle-associated membrane 7	8	P:GO:0006906; C:GO:0016021; F:GO:0005484; C:GO:0031201;	P:vesicle fusion; C:integral component of membrane; F:SNAP receptor activity; C:SNARE complex; P:endosome to lysosome transport; F:SNARE binding; P:endocytosis; P:exocytosi [illegible]	
Acrdi.XM_015916914.1/1-2325	Monca.adi2mcaRNA27781_R0/1-2004	0.0448057	0.0434353	structural maintenance of chromosomes 1A-like	6	C:GO:0005634; F:GO:0005524; P:GO:0006281; P:GO:0007064;	C:nucleus; F:ATP binding; P:DNA repair; P:mitotic sister chromatid cohesion; C:chromosome; F:chromatin binding	
Acrdi.XM_015898668.1/1-630	Monca.augustus.g34607.t1/1-630	0.0894817	0.0434329	dnaJ homolog subfamily C member 5				
Acrdi.XM_015902139.1/1-717	Monca.augustus.g46373.t1/1-720	0.0681116	0.0434302	Transcription factor SOX-14	1	P:GO:0006355	P:regulation of transcription, DNA-templated	
Acrdi.XM_015895635.1/1-1326	Monca.augustus.g22267.t1/1-1323	0.055258	0.0434224	Microspherule 1	4	P:GO:0043967; C:GO:1902494; F:GO:0010485; C:GO:0005654	P:histone H4 acetylation; C:catalytic complex; F:H4 histone acetyltransferase activity; C:nucleoplasm	EC:2.[illegible]
Acrdi.XM_015899760.1/1-2652	Monca.adi2mcaRNA8875_R1/1-2496	0.289749	0.0434222	Transposon TX1 uncharacterized 149 kDa				
Acrdi.XM_015901637.1/1-504	Monca.augustus.g34023.t1/1-504	0.380994	0.0434151	PREDICTED: uncharacterized protein LOC107336569				
Acrdi.XM_015896951.1/1-1989	Monca.augustus.g32356.t1/1-3036	0.208807	0.0434123	Mediator of RNA polymerase II transcription subunit 15				
Acrdi.XM_015915818.1/1-477	Monca.adi2mcaRNA26551_R0/1-636	0.412611	0.0433913	E3 ubiquitin- ligase RNF213-like				
Acrdi.XM_015921894.1/1-954	Monca.augustus.g3377.t1/1-801	0.717432	0.0433705	G2 M phase-specific E3 ubiquitin- ligase-like				
Acrdi.XM_015922057.1/1-441	Monca.adi2mcaRNA30733_R2/1-1017	0.489865	0.0433582	PREDICTED: uncharacterized protein LOC107355477				
Acrdi.XM_015903976.1/1-6261	Monca.adi2mcaRNA13466_R0/1-5520	0.053616	0.0433282	myoferlin isoform X4				
Acrdi.XM_015892979.1/1-1707	Monca.adi2mcaRNA3912_R1/1-1590	0.4776	0.0433151	RNA-directed DNA polymerase from transposon BS	11	C:GO:0005657; C:GO:0005634; F:GO:0043141; F:GO:0044806;	C:replication fork; C:nucleus; F:ATP-dependent 5'-3' DNA helicase activity; P:G-quadruplex DNA unwinding; P:DNA replication; P:DNA duplex unwinding; C:mitochondrion; P:re	EC:3.[illegible]
Acrdi.XM_015914011.1/1-2625	Monca.augustus.g44846.t1/1-2631	0.0532137	0.0432819	PC3-like endoprotease variant B	2	F:GO:0004252; P:GO:0016485	F:serine-type endopeptidase activity; P:protein processing	EC:3.[illegible]
Acrdi.XM_015922006.1/1-417	Monca.augustus.g8368.t1/1-741	0.0599206	0.0432263	Krueppel-like factor 12				
Acrdi.XM_015922568.1/1-1017	Monca.adi2mcaRNA3486_R2/1-915	0.494366	0.0432165	PREDICTED: uncharacterized protein LOC107355949	3	F:GO:0003677; P:GO:0015074; P:GO:0006310	F:DNA binding; P:DNA integration; P:DNA recombination	
Acrdi.XM_015925100.1/1-1206	Monca.augustus.g51153.t1/1-1206	0.0553567	0.0431871	3-methyl-2-oxobutanoate dehydrogenase [lipoamide] mitochondrial	2	P:GO:0016310; F:GO:0016301	P:phosphorylation; F:kinase activity	
Acrdi.XM_015893302.1/1-1014	Monca.augustus.g543.t1/1-1896	0.0593129	0.0431667	conserved oligomeric Golgi complex subunit 6				
Acrdi.XM_015893411.1/1-2766	Monca.augustus.g1257.t1/1-2832	0.35907	0.0431652	inner centromere A-like				
Acrdi.XM_015899448.1/1-1626	Monca.augustus.g48279.t1/1-1641	0.0555722	0.0431384	extracellular signal-regulated kinase 2-like	4	F:GO:0005524; P:GO:0000165; F:GO:0004707; C:GO:0005622	F:ATP binding; P:MAPK cascade; F:MAP kinase activity; C:intracellular	EC:2.[illegible]
Acrdi.XM_015906585.1/1-828	Monca.augustus.g70204.t1/1-951	0.0594427	0.0431095	tRNA selenocysteine 1-associated 1	2	F:GO:0000166; F:GO:0003676	F:nucleotide binding; F:nucleic acid binding	
Acrdi.XM_015914491.1/1-528	Monca.augustus.g27238.t1/1-528	0.0403714	0.0431058	calmodulin	6	F:GO:0016746; P:GO:0009847; F:GO:0005509; P:GO:0051726;	F:transferase activity, transferring acyl groups; P:spore germination; F:calcium ion binding; P:regulation of cell cycle; C:intracellular; C:spitzenkorper	
Acrdi.XM_015923637.1/1-909	Monca.adi2mcaRNA35308_R0/1-909	0.261165	0.0430529	TNF receptor-associated factor 5-like isoform X2				
Acrdi.XM_015924989.1/1-555	Monca.adi2mcaRNA40497_R0/1-486	0.0800652	0.0430342	Sugar phosphate exchanger 3	4	P:GO:0055085; F:GO:0022857; C:GO:0016021; P:GO:0006820	P:transmembrane transport; F:transmembrane transporter activity; C:integral component of membrane; P:anion transport	
Acrdi.XM_015915953.1/1-1545	Monca.augustus.g817.t1/1-2019	0.0647296	0.0429734	enhancer of polycomb homolog 1-like	3	P:GO:0006351; C:GO:0032777; P:GO:0006357	P:transcription, DNA-templated; C:Piccolo NuA4 histone acetyltransferase complex; P:regulation of transcription from RNA polymerase II promoter	
Acrdi.XM_015898282.1/1-495	Monca.augustus.g28196.t1/1-642	0.0721919	0.0429727	ras-related Rab-7L1-like	15	P:GO:0090316; P:GO:0007264; P:GO:0007030; P:GO:0006895;	P:positive regulation of intracellular protein transport; P:small GTPase mediated signal transduction; P:Golgi organization; P:Golgi to endosome transport; F:GTP binding; C:tr	EC:3.[illegible]
Acrdi.XM_015917280.1/1-723	Monca.augustus.g45713.t1/1-723	0.0702756	0.0428415	E3 ubiquitin- ligase MARCHII isoform X2	2	F:GO:0008270; C:GO:0016021	F:zinc ion binding; C:integral component of membrane	
Acrdi.XM_015907853.1/1-363	Monca.augustus.g3375.t1/1-363	0.0479325	0.0427608	PREDICTED: uncharacterized protein LOC107342357				
Acrdi.XM_015899490.1/1-939	Monca.augustus.g37214.t1/1-975	0.0556233	0.0427322	Phosphoenolpyruvate phosphomutase	6	F:GO:0050188; P:GO:0032923; P:GO:0006097; C:GO:0005737;	F:phosphoenolpyruvate mutase activity; P:organic phosphonate biosynthetic process; P:glyoxylate cycle; C:cytoplasm; F:metal ion binding; F:transferase activity, transferring a	EC:5.[illegible]
Acrdi.XM_015900499.1/1-1344	Monca.augustus.g26437.t1/1-2487	0.0347155	0.0427124	cAMP-specific 3 ,5 -cyclic phosphodiesterase 4D isoform X8	3	F:GO:0046872; P:GO:0007165; F:GO:0004114	F:metal ion binding; P:signal transduction; F:3',5'-cyclic-nucleotide phosphodiesterase activity	EC:3.[illegible]
Acrdi.XM_015913520.1/1-732	Monca.adi2mcaRNA23963_R0/1-732	0.0564762	0.0427114	L-xylulose reductase	2	F:GO:0016491; P:GO:0055114	F:oxidoreductase activity; P:oxidation-reduction process	
Acrdi.XM_015916866.1/1-378	Monca.adi2mcaRNA27760_R0/1-339	0.42641	0.0426863	PREDICTED: uncharacterized protein LOC107350630				
Acrdi.XM_015893613.1/1-1131	Monca.adi2mcaRNA39709_R0/1-1134	0.0699443	0.042668	Neurotransmitter-gated ion-channel transmembrane region	2	P:GO:0006811; C:GO:0016021	P:ion transport; C:integral component of membrane	
Acrdi.XM_015915474.1/1-813	Monca.augustus.g36663.t1/1-813	0.0693487	0.0426495	PCTP	1	F:GO:0008289	F:lipid binding	

Acrdi.XM_015912668.1/1-900 | Monca.augustus.g9557.t1/1-900	0.0525122	0.0426304	FAM166B-like			
Acrdi.XM_015908785.1/1-876 | Monca.adi2mcaRNA18784_R0/1-996	0.711618	0.0426138	ATP-dependent DNA helicase Q1	2	P:GO:0009987; F:GO:0016787	P:cellular process; F:hydrolase activity
Acrdi.XM_015922575.1/1-342 | Monca.augustus.g54972.t1/1-330	0.0508736	0.0425116	mediator of RNA polymerase II transcription subunit 11-like	3	C:GO:0016592; F:GO:0001104; P:GO:0006357	[illegible]
Acrdi.XM_015901620.1/1-969 | Monca.adi2mcaRNA10931_R0/1-954	0.190764	0.0425022	PREDICTED: uncharacterized protein LOC107336547	2	F:GO:0004842; P:GO:0016567	F:ubiquitin-protein transferase activity; P:protein ubiquitination
Acrdi.XM_015921062.1/1-780 | Monca.augustus.g52025.t1/1-612	0.0730723	0.0424736	cation channel sperm-associated 1	6	[illegible]	[illegible]
Acrdi.XM_015916065.1/1-660 | Monca.augustus.g5231.t1/1-660	0.0594302	0.0424444	transmembrane emp24 domain-containing 4-like	3	C:GO:0005789; C:GO:0016021; P:GO:0006810	C:endoplasmic reticulum membrane; C:integral component of membrane; P:transport
Acrdi.XM_015896508.1/1-1263 | Monca.adi2mcaRNA5394_R3/1-1398	0.833769	0.0423876	THAP domain-containing 6			
Acrdi.XM_015896520.1/1-3189 | Monca.augustus.g1595.t1/1-3294	0.0556817	0.0423316	importin-5			
Acrdi.XM_015901490.1/1-903 | Monca.augustus.g43360.t1/1-831	0.788864	0.0423122	THAP domain-containing 1			
Acrdi.XM_015921026.1/1-2319 | Monca.augustus.g65309.t1/1-2295	0.0459984	0.0422553	mitofusin-2	7	[illegible]	[illegible]
Acrdi.XM_015910323.1/1-726 | Monca.augustus.g26346.t1/1-726	0.0575156	0.0422548	ras-related Rab-39B-like	5	[illegible]	[illegible]
Acrdi.XM_015920818.1/1-3348 | Monca.adi2mcaRNA32128_R0/1-3924	0.505033	0.0422385	Pro-Pol poly			
Acrdi.XM_015904497.1/1-384 | Monca.augustus.g33595.t1/1-384	0.0589261	0.0422373	profilin-4-like isoform X5	5	[illegible]	[illegible]
Acrdi.XM_015898767.1/1-1791 | Monca.augustus.g55628.t1/1-1791	0.0492984	0.0421663	Bardet-Biedl syndrome 1	7	[illegible]	[illegible]
Acrdi.XM_015918300.1/1-501 | Monca.augustus.g29390.t1/1-639	0.0504641	0.0421423	ras-related Rab-13 isoform X1	3	F:GO:0005525; P:GO:0007264; C:GO:0005622	F:GTP binding; P:small GTPase mediated signal transduction; C:intracellular
Acrdi.XM_015907336.1/1-2139 | Monca.augustus.g35193.t1/1-2646	0.054351	0.0421261	coatomer subunit gamma-2-like	3	C:GO:0030117; P:GO:0015031; C:GO:0030663	C:membrane coat; P:protein transport; C:COPI-coated vesicle membrane
Acrdi.XM_015892164.1/1-315 | Monca.augustus.g53328.t1/1-1425	0.288361	0.042119	PREDICTED: uncharacterized protein LOC107327426			
Acrdi.XM_015897199.1/1-1281 | Monca.augustus.g63833.t1/1-1281	0.0605767	0.0421187	potassium voltage-gated channel Shal isoform X2	7	[illegible]	[illegible]
Acrdi.XM_015894402.1/1-1275 | Monca.adi2mcaRNA40668_R3/1-1620	0.396612	0.0420924	nuclease HARBI1			
Acrdi.XM_015896250.1/1-414 | Monca.augustus.g33753.t1/1-651	0.083264	0.0420306	ras-related Rab-31	6	[illegible]	[illegible]
Acrdi.XM_015916161.1/1-750 | Monca.augustus.g12402.t1/1-717	0.0485783	0.0420181	amyloid beta A4 precursor -binding family B member 1	5	[illegible]	[illegible]
Acrdi.XM_015897400.1/1-1344 | Monca.augustus.g51662.t1/1-1314	0.060493	0.0419903	Glutamate dehydrogenase	3	F:GO:0016639; P:GO:0006520; P:GO:0055114	[illegible]
Acrdi.XM_015904074.1/1-1152 | Monca.adi2mcaRNA13578_R0/1-753	0.0453424	0.0419504	pyruvate dehydrogenase (acetyl-transferring) mitochondrial	3	P:GO:0016310; F:GO:0016772; F:GO:0016301	P:phosphorylation; F:transferase activity, transferring phosphorus-containing groups; F:kinase activity
Acrdi.XM_015897024.1/1-543 | Monca.augustus.g46673.t1/1-2595	0.219006	0.0419469	G2 M phase-specific E3 ubiquitin- ligase-like	2	F:GO:0004842; P:GO:0016567	F:ubiquitin-protein transferase activity; P:protein ubiquitination
Acrdi.XM_015903809.1/1-1404 | Monca.augustus.g61997.t1/1-1392	0.0477716	0.0419468	casein kinase I isoform gamma-3 isoform X2	8	[illegible]	[illegible]
Acrdi.XM_015903842.1/1-810 | Monca.adi2mcaRNA13318_R2/1-951	1.48453	0.0418926	PREDICTED: uncharacterized protein LOC107338603			
Acrdi.XM_015908377.1/1-849 | Monca.augustus.g34818.t1/1-849	0.0557843	0.0418761	PREDICTED: uncharacterized protein C15orf41 homolog			
Acrdi.XM_015897662.1/1-2697 | Monca.augustus.g50732.t1/1-2535	0.0502156	0.0418332	Pre-mRNA-splicing factor SYF1	6	[illegible]	[illegible]
Acrdi.XM_015904357.1/1-2289 | Monca.augustus.g59957.t1/1-2316	0.0792729	0.0417532	E3 ubiquitin- ligase UNKL isoform X2	2	F:GO:0008270; F:GO:0046872	F:zinc ion binding; F:metal ion binding
Acrdi.XM_015920911.1/1-1212 | Monca.augustus.g54780.t1/1-2715	0.0532393	0.0417187	glutamate receptor	6	[illegible]	[illegible]
Acrdi.XM_015904597.1/1-1779 | Monca.augustus.g41299.t1/1-1584	0.638579	0.0416871	Eukaryotic translation initiation factor 3 subunit D			
Acrdi.XM_015908522.1/1-1065 | Monca.augustus.g65969.t1/1-1029	0.0767473	0.0416513	cysteine and histidine-rich 1	1	F:GO:0046872	F:metal ion binding
Acrdi.XM_015910015.1/1-4125 | Monca.adi2mcaRNA1976_R7/1-3954	0.35145	0.0416207	PREDICTED: uncharacterized protein LOC107344362			
Acrdi.XM_015915006.1/1-1836 | Monca.adi2mcaRNA25656_R0/1-1602	0.0515017	0.0415865	HIRA	3	C:GO:0005634; P:GO:0006351; P:GO:0006355	C:nucleus; P:transcription, DNA-templated; P:regulation of transcription, DNA-templated
Acrdi.XM_015919528.1/1-1221 | Monca.adi2mcaRNA30722_R3/1-1239	0.913942	0.0415782	nuclease HARBI1	1	C:GO:0005575	C:cellular_component
Acrdi.XM_015909550.1/1-1800 | Monca.adi2mcaRNA19635_R0/1-1677	0.0421326	0.0415596	recombining binding suppressor of hairless isoform X2	62	[illegible]	[illegible]
Acrdi.XM_015902247.1/1-1512 | Monca.augustus.g35098.t1/1-672	0.0456231	0.0415143	PREDICTED: uncharacterized protein LOC107337126, partial	2	C:GO:0005581; F:GO:0005201	C:collagen trimer; F:extracellular matrix structural constituent
Acrdi.XM_015900309.1/1-882 | Monca.augustus.g60611.t1/1-882	0.575118	0.0414929	ac transposable element-derived 4			
Acrdi.XM_015901899.1/1-1458 | Monca.augustus.g58311.t1/1-1458	0.0558715	0.0414911	tubulin epsilon chain-like	4	F:GO:0005525; F:GO:0003924; C:GO:0005874; P:GO:0007017	F:GTP binding; F:GTPase activity; C:microtubule; P:microtubule-based process
Acrdi.XM_015904113.1/1-2553 | Monca.adi2mcaRNA13589_R0/1-2487	0.059569	0.0414404	diacylglycerol kinase gamma isoform X1	4	F:GO:0000166; P:GO:0007165; F:GO:0043167; F:GO:0016301	F:nucleotide binding; P:signal transduction; F:ion binding; F:kinase activity
Acrdi.XM_015899496.1/1-357 | Monca.augustus.g37219.t1/1-357	0.056626	0.0413951	40S ribosomal S20	4	F:GO:0003723; F:GO:0003735; C:GO:0022627; P:GO:0006412	F:RNA binding; F:structural constituent of ribosome; C:cytosolic small ribosomal subunit; P:translation
Acrdi.XM_015895375.1/1-840 | Monca.adi2mcaRNA41817_R1/1-930	0.295058	0.0413711	PREDICTED: uncharacterized protein LOC107330819			
Acrdi.XM_015905645.1/1-852 | Monca.augustus.g43786.t1/1-834	0.0577519	0.0413629	eukaryotic translation initiation factor 3 subunit F-like	7	[illegible]	[illegible]
Acrdi.XM_015919317.1/1-1329 | Monca.augustus.g5209.t1/1-1368	0.0601798	0.0413417	NEDD8-activating enzyme E1 catalytic subunit-like isoform X1	9	[illegible]	[illegible]
Acrdi.XM_015906906.1/1-1716 | Monca.augustus.g8901.t1/1-1707	0.0426842	0.0413221	kelch 5 isoform X2	3	F:GO:0004842; C:GO:0031463; P:GO:0016567	F:ubiquitin-protein transferase activity; C:Cul3-RING ubiquitin ligase complex; P:protein ubiquitination
Acrdi.XM_015918242.1/1-621 | Monca.adi2mcaRNA29294_R0/1-657	0.307229	0.0412446	PREDICTED: uncharacterized protein LOC107351944			
Acrdi.XM_015897397.1/1-612 | Monca.augustus.g51658.t1/1-612	0.0536577	0.0411801	ubiquitin-conjugating enzyme E2 S-like	11	[illegible]	[illegible]
Acrdi.XM_015916846.1/1-423 | Monca.augustus.g55428.t1/1-1596	0.0461434	0.041111	Calcium-binding EF-hand family	5	[illegible]	[illegible]
Acrdi.XM_015921351.1/1-873 | Monca.adi2mcaRNA32699_R1/1-942	0.0718953	0.0410979	endophilin-B1 isoform X2	3	[illegible]	[illegible]
Acrdi.XM_015893549.1/1-1902 | Monca.adi2mcaRNA39638_R1/1-1644	0.0603578	0.041073	5-oxoprolinase-like	1	F:GO:0016787	[illegible]
Acrdi.XM_015905103.1/1-1878 | Monca.augustus.g29428.t1/1-1890	0.235236	0.0410635	choline dehydrogenase	6	[illegible]	[illegible]
Acrdi.XM_015914685.1/1-1287 | Monca.adi2mcaRNA25286_R0/1-1245	0.465864	0.0410526	THAP domain-containing 2			
Acrdi.XM_015905823.1/1-723 | Monca.augustus.g31759.t1/1-717	0.0706611	0.0410206	homeobox XENK-2-like	4	[illegible]	[illegible]
Acrdi.XM_015904539.1/1-1026 | Monca.adi2mcaRNA14061_R0/1-1020	0.0674942	0.0409477	probable palmitoyltransferase ZDHHC14 isoform X1	3	[illegible]	[illegible]
Acrdi.XM_015913935.1/1-870 | Monca.augustus.g66935.t1/1-873	0.0548083	0.0408878	homeobox six1-like	3	[illegible]	[illegible]
Acrdi.XM_015922212.1/1-972 | Monca.adi2mcaRNA33660_R1/1-1146	0.0439048	0.0408827	brahma-associated of 60 kDa isoform X1			
Acrdi.XM_015903640.1/1-903 | Monca.adi2mcaRNA13109_R0/1-858	0.0445815	0.0408776	Splicing factor 3A subunit 3	4	[illegible]	[illegible]
Acrdi.XM_015901469.1/1-921 | Monca.augustus.g31711.t1/1-921	0.0467833	0.0408765	mitochondrial substrate carrier family ucpB-like	4	[illegible]	[illegible]
Acrdi.XM_015923774.1/1-1227 | Monca.augustus.g54392.t1/1-1470	0.0524671	0.0408689	insulin-like growth factor 2 mRNA-binding 1 isoform X2	2	F:GO:0003676; F:GO:0003723	[illegible]
Acrdi.XM_015901751.1/1-2169 | Monca.adi2mcaRNA11052_R0/1-2184	0.0604088	0.0408678	Cleavage stimulation factor subunit 3	5	[illegible]	[illegible]
Acrdi.XM_015914367.1/1-1119 | Monca.augustus.g70646.t1/1-1086	0.303549	0.0408407	TNF receptor-associated factor 5-like	7	[illegible]	[illegible]
Acrdi.XM_015899662.1/1-981 | Monca.augustus.g39718.t1/1-981	0.0374019	0.0408185	partitioning defective 6 homolog gamma			
Acrdi.XM_015918246.1/1-1464 | Monca.adi2mcaRNA29280_R0/1-1497	0.0637849	0.0407942	Serine threonine- kinase	3	[illegible]	[illegible]
Acrdi.XM_015919328.1/1-654 | Monca.augustus.g15440.t1/1-675	0.0561372	0.0407658	PRELI domain containing 3B	3	[illegible]	[illegible]
Acrdi.XM_015907561.1/1-1011 | Monca.adi2mcaRNA17435_R8/1-810	0.339767	0.0407245	aspartyl asparaginyl beta-hydroxylase-like			
Acrdi.XM_015913043.1/1-624 | Monca.augustus.g8145.t1/1-624	0.0579603	0.0406662	transmembrane emp24 domain-containing 10	2	C:GO:0016021; P:GO:0006810	[illegible]
Acrdi.XM_015922523.1/1-1929 | Monca.adi2mcaRNA34014_R0/1-1932	0.421832	0.0406508	RNA-directed DNA polymerase from mobile element jockey-like			
Acrdi.XM_015918276.1/1-1878 | Monca.adi2mcaRNA29308_R1/1-1992	0.541568	0.0406383	PREDICTED: uncharacterized protein LOC107351974			
Acrdi.XM_015898476.1/1-795 | Monca.adi2mcaRNA7583_R5/1-624	0.646016	0.0406237	Tetratricopeptide repeat 28			
Acrdi.XM_015907316.1/1-2055 | Monca.augustus.g5324.t1/1-2025	0.0551146	0.0406098	transcription initiation factor TFIID subunit 5 isoform X2	12	[illegible]	[illegible]
Acrdi.XM_015924798.1/1-834 | Monca.augustus.g21509.t1/1-912	0.330053	0.0405391	PREDICTED: uncharacterized protein K02A2.6-like			
Acrdi.XM_015920130.1/1-1020 | Monca.augustus.g27079.t1/1-1599	0.0596032	0.0405302	parafibromin	14	[illegible]	[illegible]
Acrdi.XM_015896335.1/1-534 | Monca.augustus.g65176.t1/1-708	0.139301	0.0405149	PREDICTED: uncharacterized protein LOC107331717			
Acrdi.XM_015913248.1/1-879 | Monca.adi2mcaRNA23652_R1/1-828	0.276376	0.0405148	Retrovirus-related Pol poly from transposon 412	3	[illegible]	[illegible]
Acrdi.XM_015914269.1/1-702 | Monca.adi2mcaRNA24841_R0/1-705	0.0604111	0.0405055	Fructose-1,6-bisphosphatase 1	15	[illegible]	[illegible]
Acrdi.XM_015896557.1/1-327 | Monca.adi2mcaRNA5415_R0/1-315	0.0472455	0.040476	kinesin KIF23	8	[illegible]	[illegible]
Acrdi.XM_015904283.1/1-363 | Monca.augustus.g32331.t1/1-444	0.0449603	0.0404065	Diphosphoinositol polyphosphate phosphohydrolase 1	9	[illegible]	[illegible]
Acrdi.XM_015898298.1/1-636 | Monca.augustus.g61843.t1/1-582	0.0395117	0.0402541	anaphase-promoting complex subunit 10	7	[illegible]	[illegible]
Acrdi.XM_015903498.1/1-1125 | Monca.augustus.g20120.t1/1-1692	0.0378569	0.0402377	kinesin KIF2A			
Acrdi.XM_015923672.1/1-432 | Monca.augustus.g2793.t1/1-432	0.0552133	0.0402092	UPF0686 C11orf1 homolog	1	C:GO:0005634	[illegible]
Acrdi.XM_015898539.1/1-525 | Monca.augustus.g33996.t1/1-525	0.0660123	0.0402018	EF-hand calcium-binding domain-containing 2	1	F:GO:0005509	[illegible]
Acrdi.XM_015916503.1/1-744 | Monca.augustus.g38976.t1/1-1122	0.0475997	0.0402001	pantothenate kinase 3 isoform X1	4	[illegible]	[illegible]
Acrdi.XM_015901625.1/1-789 | Monca.adi2mcaRNA10906_R2/1-726	0.193539	0.0401651	LINE-1 type transposase domain-containing 1-like			
Acrdi.XM_015911214.1/1-1314 | Monca.augustus.g34669.t1/1-1494	0.0577361	0.040143	beta-1-syntrophin-like	3	[illegible]	[illegible]
Acrdi.XM_015910330.1/1-414 | Monca.adi2mcaRNA20459_R0/1-441	0.105509	0.0401031	FAM102B			
Acrdi.XM_015893539.1/1-3483 | Monca.augustus.g25399.t1/1-3486	0.0540044	0.0400924	WASH complex subunit 7	4	[illegible]	[illegible]
Acrdi.XM_015924098.1/1-1806 | Monca.adi2mcaRNA35850_R0/1-1284	0.368527	0.0400879	PREDICTED: uncharacterized protein LOC107357459			
Acrdi.XM_015900048.1/1-1281 | Monca.augustus.g13753.t1/1-1254	0.0524999	0.0400396	Mitochondrial chaperone BCS1	4	[illegible]	[illegible]
Acrdi.XM_015917167.1/1-1083 | Monca.augustus.g45678.t1/1-1074	0.38897	0.0400366	nuclease HARBI1			
Acrdi.XM_015896975.1/1-1374 | Monca.adi2mcaRNA5847_R0/1-1371	0.407328	0.0400331	type IIB DNA topoisomerase	9	[illegible]	[illegible]
Acrdi.XM_015908542.1/1-960 | Monca.augustus.g28024.t1/1-963	0.0526498	0.040023	Maspardin	3	[illegible]	[illegible]
Acrdi.XM_015912355.1/1-759 | Monca.adi2mcaRNA22698_R4/1-732	0.410122	0.0400001	transcription factor Adf-1-like	8	[illegible]	[illegible]
Acrdi.XM_015898343.1/1-1515 | Monca.adi2mcaRNA7369_R0/1-1467	0.0627571	0.0399779	E3 ubiquitin- ligase TRIM23	13	[illegible]	[illegible]
Acrdi.XM_015924123.1/1-1479 | Monca.augustus.g66956.t1/1-1491	0.0311152	0.0399736	Serine threonine- kinase 3	4	[illegible]	[illegible]
Acrdi.XM_015922824.1/1-1164 | Monca.augustus.g6742.t1/1-1164	0.0702934	0.03997	junctophilin-1 isoform X1			
Acrdi.XM_015899560.1/1-1197 | Monca.augustus.g46962.t1/1-2541	0.0421712	0.0399271	glutamine-dependent NAD(+) synthetase isoform X1	5	[illegible]	[illegible]
Acrdi.XM_015896105.1/1-1905 | Monca.adi2mcaRNA4923_R0/1-1911	0.0419231	0.0399271	Polyadenylate-binding 1	1	F:GO:0003676	[illegible]
Acrdi.XM_015900685.1/1-507 | Monca.adi2mcaRNA9889_R0/1-477	0.0236142	0.0399117	Peptidyl-prolyl cis-trans isomerase H	10	[illegible]	[illegible]
Acrdi.XM_015904274.1/1-519 | Monca.augustus.g8631.t1/1-519	0.0404783	0.0399036	uncharacterized HIT Sympcc7942_1390 isoform X2			
Acrdi.XM_015907830.1/1-2190 | Monca.augustus.g47395.t1/1-2190	0.0509019	0.0398806	Chondroitin sulfate synthase 1	2	C:GO:0032580; F:GO:0008376	[illegible]
Acrdi.XM_015903967.1/1-1401 | Monca.augustus.g435.t1/1-855	0.360525	0.0398363	RNA-directed DNA polymerase from mobile element jockey			
Acrdi.XM_015896172.1/1-3027 | Monca.augustus.g23049.t1/1-3090	0.0485312	0.039825	pre-mRNA-splicing factor ATP-dependent RNA helicase DHX16	7	[illegible]	[illegible]
Acrdi.XM_015906082.1/1-1488 | Monca.augustus.g34436.t1/1-1734	0.0537425	0.0398124	kelch 20 isoform X2	6	[illegible]	[illegible]
Acrdi.XM_015914519.1/1-447 | Monca.augustus.g27240.t1/1-447	0.0407967	0.0398065	neuronal acetylcholine receptor subunit alpha-7-like	12	[illegible]	[illegible]
Acrdi.XM_015916743.1/1-2514 | Monca.augustus.g62780.t1/1-2514	0.0438265	0.0398058	Tuftelin-interacting 11	4	[illegible]	[illegible]
Acrdi.XM_015893661.1/1-1032 | Monca.augustus.g56936.t1/1-1032	0.187371	0.0397968	PREDICTED: uncharacterized protein LOC107328915 isoform X2			
Acrdi.XM_015924813.1/1-1083 | Monca.augustus.g43720.t1/1-1302	0.0650828	0.0397925	Adenosylhomocysteinase	4	[illegible]	[illegible]
Acrdi.XM_015893671.1/1-2628 | Monca.augustus.g30721.t1/1-3399	0.0409424	0.0397608	kinesin heavy chain isoform 5C			
Acrdi.XM_015899232.1/1-2454 | Monca.adi2mcaRNA8373_R1/1-2793	0.636634	0.0397361	WDFY family member 4			
Acrdi.XM_015915327.1/1-1566 | Monca.augustus.g25262.t1/1-1566	0.0468406	0.0397115	DNA polymerase epsilon subunit 2	5	[illegible]	[illegible]
Acrdi.XM_015908012.1/1-615 | Monca.augustus.g6862.t1/1-612	0.0517905	0.0397103	trox-2 homeobox transcription factor	3	[illegible]	[illegible]
Acrdi.XM_015923412.1/1-450 | Monca.augustus.g42118.t1/1-447	0.336289	0.0397005	Nucleus accumbens-associated			
Acrdi.XM_015892747.1/1-1389 | Monca.augustus.g10432.t1/1-1275	0.355046	0.0396694	PREDICTED: uncharacterized protein LOC107328033	1	F:GO:0070492	[illegible]
Acrdi.XM_015906723.1/1-1878 | Monca.adi2mcaRNA16547_R0/1-1746	0.792726	0.039667	RNA-directed DNA polymerase from transposon BS			
Acrdi.XM_015924349.1/1-555 | Monca.augustus.g32408.t1/1-555	0.0657635	0.039626	GTP-binding Rheb homolog	4	[illegible]	[illegible]
Acrdi.XM_015905562.1/1-2013 | Monca.augustus.g30016.t1/1-1911	0.166363	0.0396132	PREDICTED: uncharacterized protein LOC107340202			
Acrdi.XM_015900095.1/1-2112 | Monca.adi2mcaRNA9223_R1/1-1698	0.248179	0.0395974	Mitochondrial inner membrane protease subunit 1			
Acrdi.XM_015908947.1/1-588 | Monca.augustus.g47244.t1/1-588	0.0562804	0.0395846	Hikeshi isoform X1	8	[illegible]	[illegible]
Acrdi.XM_015903189.1/1-798 | Monca.augustus.g71354.t1/1-837	0.0795594	0.0395483	proteasome subunit alpha type-1	5	[illegible]	[illegible]
Acrdi.XM_015897632.1/1-384 | Monca.augustus.g60805.t1/1-387	0.0488151	0.039522	PREDICTED: uncharacterized protein LOC107332878			
Acrdi.XM_015894091.1/1-750 | Monca.adi2mcaRNA40305_R3/1-702	0.735257	0.0395054	PREDICTED: uncharacterized protein LOC107329405			
Acrdi.XM_015895422.1/1-1260 | Monca.augustus.g6365.t1/1-1287	0.0434316	0.0394659	transmembrane 184B-like	3	[illegible]	[illegible]
Acrdi.XM_015921503.1/1-945 | Monca.augustus.g58053.t1/1-1332	0.0570421	0.0394115	WD repeat domain phosphoinositide-interacting	10	[illegible]	[illegible]
Acrdi.XM_015913993.1/1-1605 | Monca.adi2mcaRNA24508_R2/1-1491	0.446704	0.0394055	PREDICTED: uncharacterized protein LOC107347995	1	F:GO:0003676	[illegible]
Acrdi.XM_015921666.1/1-954 | Monca.adi2mcaRNA3301_R0/1-855	0.518183	0.039359	PREDICTED: uncharacterized protein LOC107355137 isoform X2			
Acrdi.XM_015899257.1/1-3360 | Monca.augustus.g68355.t1/1-3252	0.056846	0.039348	transcription initiation factor TFIID subunit 2 isoform X1	11	[illegible]	[illegible]
Acrdi.XM_015919459.1/1-3300 | Monca.augustus.g25246.t1/1-3303	0.0506861	0.0393338	ATP-citrate synthase isoform X1	11	[illegible]	[illegible]
Acrdi.XM_015898199.1/1-1092 | Monca.augustus.g29228.t1/1-1092	0.0422816	0.0393328	proto-oncogene Wnt-1	5	[illegible]	[illegible]
Acrdi.XM_015892677.1/1-948 | Monca.augustus.g58958.t1/1-2103	0.0455885	0.0392879	GMP synthase [glutamine-hydrolyzing]	6	[illegible]	[illegible]
Acrdi.XM_015912922.1/1-6387 | Monca.adi2mcaRNA23326_R0/1-6339	0.0499519	0.0392858	talin-1 isoform X1	3	[illegible]	[illegible]
Acrdi.XM_015917505.1/1-747 | Monca.augustus.g31635.t1/1-768	0.409415	0.0392665	G2 M phase-specific E3 ubiquitin- ligase			
Acrdi.XM_015909948.1/1-1788 | Monca.augustus.g49963.t1/1-1704	0.390121	0.0392547	Carboxypeptidase D			
Acrdi.XM_015919564.1/1-369 | Monca.augustus.g52098.t1/1-339	0.0470476	0.0392544	PREDICTED: uncharacterized protein LOC107353241			
Acrdi.XM_015917575.1/1-498 | Monca.augustus.g33492.t1/1-534	0.523927	0.0392474	PREDICTED: uncharacterized protein LOC107351283			
Acrdi.XM_015908305.1/1-330 | Monca.augustus.g34625.t1/1-348	0.0716171	0.0392396	BET1	1	C:GO:0016021	[illegible]
Acrdi.XM_015901254.1/1-2868 | Monca.augustus.g17164.t1/1-2907	0.0462142	0.0392244	gamma-tubulin complex component 2	14	[illegible]	[illegible]
Acrdi.XM_015921111.1/1-1518 | Monca.augustus.g64936.t1/1-1779	0.24574	0.0392176	Zinc finger 653			
Acrdi.XM_015896996.1/1-1179 | Monca.adi2mcaRNA5879_R0/1-1176	0.045926	0.0391931	Nuclear receptor subfamily 2 group E member 1	15	[illegible]	[illegible]
Acrdi.XM_015915136.1/1-840 | Monca.adi2mcaRNA25784_R0/1-666	0.995563	0.0391681	PREDICTED: uncharacterized protein LOC107349040			
Acrdi.XM_015908647.1/1-1770 | Monca.augustus.g53876.t1/1-1917	0.0482299	0.0391552	ring canal kelch homolog	4	[illegible]	[illegible]
Acrdi.XM_015897795.1/1-738 | Monca.augustus.g49482.t1/1-987	0.0556681	0.0391431	Succinyl- ligase [ADP GDP-forming] subunit mitochondrial	9	[illegible]	[illegible]
Acrdi.XM_015923558.1/1-543 | Monca.augustus.g15158.t1/1-543	0.040139	0.0391199	ADP-ribosylation factor 1	3	[illegible]	[illegible]
Acrdi.XM_015893219.1/1-864 | Monca.augustus.g41328.t1/1-990	0.0600588	0.0391179	ankyrin PH and SEC7 domain containing secG-like			
Acrdi.XM_015910934.1/1-1176 | Monca.adi2mcaRNA21133_R0/1-1185	0.262866	0.0390653	Zinc finger 653			
Acrdi.XM_015900426.1/1-1596 | Monca.adi2mcaRNA9609_R0/1-1719	0.316456	0.0390121	Retrovirus-related Pol poly from transposon opus			
Acrdi.XM_015918025.1/1-696 | Monca.augustus.g65787.t1/1-807	0.850024	0.038949	RNA-directed DNA polymerase from mobile element jockey-like			
Acrdi.XM_015909274.1/1-1605 | Monca.adi2mcaRNA1975_R0/1-1920	0.366662	0.0389384	cytosolic carboxypeptidase 2			
Acrdi.XM_015922947.1/1-960 | Monca.adi2mcaRNA34534_R0/1-1101	0.330601	0.038907	PREDICTED: uncharacterized protein LOC107356330			
Acrdi.XM_015924507.1/1-1290 | Monca.augustus.g28544.t1/1-1287	0.0809938	0.0388922	Zinc finger ZIC 1	7	[illegible]	[illegible]
Acrdi.XM_015923401.1/1-420 | Monca.augustus.g59807.t1/1-1083	0.20448	0.0388904	PREDICTED: uncharacterized protein LOC107356773	1	F:GO:0070492	[illegible]
Acrdi.XM_015908546.1/1-702 | Monca.augustus.g25866.t1/1-636	0.195789	0.0388443	inositol 1,4,5-trisphosphate receptor type 3-like			
Acrdi.XM_015892366.1/1-1215 | Monca.adi2mcaRNA38296_R1/1-1176	0.66318	0.0388106	RNA-directed DNA polymerase from mobile element jockey-like			
Acrdi.XM_015921476.1/1-861 | Monca.adi2mcaRNA3359_R0/1-897	0.0883151	0.0387851	PREDICTED: uncharacterized protein LOC107354970			
Acrdi.XM_015900921.1/1-942 | Monca.augustus.g53999.t1/1-945	0.0764109	0.0387429	Eukaryotic translation initiation factor 2 subunit 1	31	[illegible]	[illegible]
Acrdi.XM_015908720.1/1-1302 | Monca.adi2mcaRNA18704_R1/1-1155	0.309735	0.0387421	Macrophage scavenger receptor types I and II			
Acrdi.XM_015918164.1/1-345 | Monca.augustus.g53131.t1/1-345	0.0723607	0.0387385	endosulfine alpha	6	[illegible]	[illegible]
Acrdi.XM_015914841.1/1-1185 | Monca.augustus.g40127.t1/1-1134	0.074862	0.0387267	Guanine nucleotide-binding G(s) subunit alpha	6	[illegible]	[illegible]
Acrdi.XM_015925458.1/1-1446 | Monca.adi2mcaRNA37443_R0/1-1620	0.0611825	0.0386855	high-affinity choline transporter 1-like	6	[illegible]	[illegible]
Acrdi.XM_015906212.1/1-1074 | Monca.augustus.g21702.t1/1-2760	0.0469525	0.0386509	peptidyl-prolyl cis-trans isomerase-like 2	9	[illegible]	[illegible]
Acrdi.XM_015920604.1/1-1899 | Monca.adi2mcaRNA31892_R0/1-1806	0.378769	0.0386493	ATP-dependent DNA helicase -like	11	[illegible]	[illegible]
Acrdi.XM_015900169.1/1-1344 | Monca.augustus.g59987.t1/1-1338	0.0333806	0.0386486	calreticulin	4	[illegible]	[illegible]
Acrdi.XM_015898095.1/1-378 | Monca.augustus.g9829.t1/1-378	0.0291719	0.0386427	microtubule-associated s 1A 1B light chain 3A	8	[illegible]	[illegible]
Acrdi.XM_015921724.1/1-1281 | Monca.augustus.g26711.t1/1-1296	0.372213	0.0386373	PREDICTED: uncharacterized protein LOC107355195			
Acrdi.XM_015921464.1/1-2244 | Monca.adi2mcaRNA32822_R2/1-2214	0.20241	0.0386319	HMG domain-containing 3			
Acrdi.XM_015905224.1/1-951 | Monca.augustus.g52847.t1/1-951	0.0587601	0.0386238	Serine-threonine kinase receptor-associated	15	[illegible]	[illegible]
Acrdi.XM_015924521.1/1-450 | Monca.augustus.g31185.t1/1-333	0.0521969	0.038617	transmembrane 170A	1	C:GO:0016021	[illegible]
Acrdi.XM_015914243.1/1-531 | Monca.augustus.g24193.t1/1-1080	0.0488085	0.0385645	Sulfatase-modifying factor 1			
Acrdi.XM_015906969.1/1-561 | Monca.augustus.g49158.t1/1-564	0.0513325	0.0385531	Mitochondrial import inner membrane translocase subunit Tim22	5	[illegible]	[illegible]
Acrdi.XM_015899512.1/1-900 | Monca.augustus.g15184.t1/1-900	0.0559822	0.038535	cyclin N-terminal domain-containing 1-like			
Acrdi.XM_015899368.1/1-462 | Monca.augustus.g44802.t1/1-462	0.0468895	0.0384733	ubiquitin-conjugating enzyme X	9	[illegible]	[illegible]
Acrdi.XM_015902010.1/1-1083 | Monca.augustus.g70387.t1/1-1575	0.039395	0.0384727	phosphatidylserine synthase 1-like	2	C:GO:0016021; P:GO:0008654	[illegible]
Acrdi.XM_015909816.1/1-1074 | Monca.augustus.g47076.t1/1-1080	0.0549402	0.0384594	PREDICTED: uncharacterized protein LOC107344182	1	C:GO:0016020	[illegible]
Acrdi.XM_015912795.1/1-2034 | Monca.augustus.g26427.t1/1-3627	0.0436155	0.0384399	Cilia- and flagella-associated 57			
Acrdi.XM_015912839.1/1-912 | Monca.augustus.g49295.t1/1-909	0.0341563	0.0384151	Solute carrier family 25 member 35	5	[illegible]	[illegible]
Acrdi.XM_015900457.1/1-3267 | Monca.augustus.g49629.t1/1-3150	0.0619597	0.038386	Valine--tRNA ligase	6	[illegible]	[illegible]
Acrdi.XM_015910050.1/1-681 | Monca.augustus.g38593.t1/1-681	0.0554555	0.0383762	DGCR6-like isoform X1			
Acrdi.XM_015913195.1/1-4875 | Monca.adi2mcaRNA23608_R0/1-4794	0.0450267	0.0383598	CAD	12	[illegible]	[illegible]
Acrdi.XM_015904366.1/1-552 | Monca.augustus.g59955.t1/1-552	0.0485318	0.0383536	ADP-ribosylation factor 3-like	5	[illegible]	[illegible]
Acrdi.XM_015901500.1/1-2289 | Monca.adi2mcaRNA10822_R2/1-1953	0.337804	0.0383408	SURP and G-patch domain-containing 1	1	F:GO:0046872	[illegible]
Acrdi.XM_015893439.1/1-768 | Monca.augustus.g64075.t1/1-1464	0.0518024	0.0383063	solute carrier family 41 member 1-like	3	[illegible]	[illegible]
Acrdi.XM_015899558.1/1-1236 | Monca.augustus.g46960.t1/1-1245	0.0640005	0.0383018	proteasomal ubiquitin receptor ADRM1 isoform X1	2	C:GO:0005634; C:GO:0005737	[illegible]
Acrdi.XM_015899027.1/1-5814 | Monca.augustus.g19578.t1/1-4770	0.64978	0.0382701	PREDICTED: uncharacterized protein LOC107334125			
Acrdi.XM_015919343.1/1-417 | Monca.augustus.g15439.t1/1-417	0.0717423	0.0382127	Mediator of RNA polymerase II transcription subunit 10	4	[illegible]	[illegible]
Acrdi.XM_015912482.1/1-1641 | Monca.adi2mcaRNA22857_R3/1-1464	0.719361	0.0381827	zinc finger 862-like			
Acrdi.XM_015921594.1/1-2022 | Monca.augustus.g41675.t1/1-2028	0.0653797	0.038124	F-box WD repeat-containing 7 isoform X1	12	[illegible]	[illegible]
Acrdi.XM_015921007.1/1-1335 | Monca.adi2mcaRNA32317_R8/1-1149	0.544715	0.0381204	TPA: gag-pol poly			
Acrdi.XM_015916664.1/1-3285 | Monca.augustus.g59158.t1/1-4905	0.0325442	0.0381122	kalirin isoform X5	3	[illegible]	[illegible]
Acrdi.XM_015907849.1/1-753 | Monca.augustus.g3367.t1/1-753	0.0386969	0.0380723	outer dense fiber 3-like			
Acrdi.XM_015909179.1/1-1323 | Monca.adi2mcaRNA19239_R1/1-1323	0.0377593	0.0380512	WD repeat-containing WRAP73			
Acrdi.XM_015920182.1/1-858 | Monca.adi2mcaRNA31432_R6/1-798	0.394381	0.0380262	piggyBac transposable element-derived 4-like	1	C:GO:0016020	[illegible]
Acrdi.XM_015919460.1/1-1008 | Monca.augustus.g25244.t1/1-1008	0.0357898	0.0380018	Serine threonine- kinase 17A	8	[illegible]	[illegible]
Acrdi.XM_015907231.1/1-1626 | Monca.augustus.g15333.t1/1-1629	0.08195	0.0379856	YTH domain-containing family 1	5	[illegible]	[illegible]
Acrdi.XM_015917257.1/1-672 | Monca.augustus.g55541.t1/1-672	0.0515434	0.0379821	PREDICTED: uncharacterized protein C9orf135-like			
Acrdi.XM_015914691.1/1-864 | Monca.adi2mcaRNA25291_R0/1-924	0.825933	0.0379585	RNA-directed DNA polymerase from transposon BS			
Acrdi.XM_015905655.1/1-1638 | Monca.augustus.g5889.t1/1-1647	0.0601736	0.0379495	Propionyl- carboxylase beta mitochondrial	1	F:GO:0016874	[illegible]
Acrdi.XM_015892785.1/1-699 | Monca.augustus.g3817.t1/1-699	0.39048	0.0379435	PREDICTED: uncharacterized protein LOC107328072			
Acrdi.XM_015896277.1/1-1560 | Monca.augustus.g33698.t1/1-1566	0.0414223	0.0379254	beta,beta-carotene 9 ,10 -oxygenase-like isoform X1	2	F:GO:0016702; P:GO:0055114	[illegible]
Acrdi.XM_015898020.1/1-372 | Monca.augustus.g28150.t1/1-372	0.0537116	0.0378616	ubiquinol-cytochrome-c reductase complex assembly factor 2	2	C:GO:0044429; P:GO:0050794	[illegible]
Acrdi.XM_015907539.1/1-1707 | Monca.augustus.g48968.t1/1-1701	0.307862	0.037812	Transposon TX1 uncharacterized 149 kDa			
Acrdi.XM_015911106.1/1-588 | Monca.augustus.g55133.t1/1-588	0.0592182	0.0378082	isochorismatase domain-containing mitochondrial-like	2	F:GO:0003824; P:GO:0008152	[illegible]
Acrdi.XM_015915405.1/1-564 | Monca.augustus.g56854.t1/1-564	0.0416973	0.0377752	PREDICTED: uncharacterized protein LOC107349285	1	F:GO:0070492	[illegible]
Acrdi.XM_015901517.1/1-1362 | Monca.augustus.g25809.t1/1-1344	0.0724434	0.0377519	ETS translocation variant 1 isoform X6	5	[illegible]	[illegible]
Acrdi.XM_015896746.1/1-582 | Monca.augustus.g42407.t1/1-1227	0.0647051	0.0377228	lanC 2	11	[illegible]	[illegible]
Acrdi.XM_015895855.1/1-588 | Monca.adi2mcaRNA4639_R1/1-600	1.2851	0.0376821	ATP-dependent DNA helicase PIF1			
Acrdi.XM_015899464.1/1-753 | Monca.augustus.g37260.t1/1-753	0.0575727	0.0376247	ribosome maturation SBDS	5	[illegible]	[illegible]
Acrdi.XM_015909437.1/1-1068 | Monca.augustus.g45722.t1/1-1539	0.0570607	0.03758	inosine-5 -monophosphate dehydrogenase 1b isoform X2			
Acrdi.XM_015908674.1/1-1782 | Monca.augustus.g53868.t1/1-1980	0.0697933	0.0375778	probable RNA-binding 46 isoform X1	5	[illegible]	[illegible]
Acrdi.XM_015910014.1/1-1122 | Monca.augustus.g34717.t1/1-1122	0.442064	0.0375331	serine threonine- kinase			
Acrdi.XM_015905231.1/1-585 | Monca.augustus.g52843.t1/1-618	0.0458037	0.0375275	cGMP-dependent kinase 1			
Acrdi.XM_015895987.1/1-3528 | Monca.adi2mcaRNA4811_R0/1-2109	0.0491422	0.0375213	ATP-dependent RNA helicase DHX8			
Acrdi.XM_015909450.1/1-570 | Monca.augustus.g56414.t1/1-1098	0.162211	0.0375112	PREDICTED: uncharacterized protein LOC107343862			
Acrdi.XM_015892124.1/1-1257 | Monca.adi2mcaRNA38026_R3/1-936	0.436224	0.0375091	nuclease HARBI1			
Acrdi.XM_015904692.1/1-1041 | Monca.augustus.g41115.t1/1-1494	0.0626651	0.0374332	Ribosomal S6 kinase beta-1	6	[illegible]	[illegible]
Acrdi.XM_015916934.1/1-1452 | Monca.augustus.g55279.t1/1-2730	0.0524098	0.0374219	tubulin polyglutamylase TTLL13-like	1	P:GO:0006464	[illegible]
Acrdi.XM_015910488.1/1-3825 | Monca.augustus.g25426.t1/1-8169	0.0482385	0.0374189	Inositol 1,4,5-trisphosphate receptor type 1	7	[illegible]	[illegible]
Acrdi.XM_015895854.1/1-774 | Monca.augustus.g25524.t1/1-969	2.38784	0.0373871	PREDICTED: uncharacterized protein LOC107331282	4	[illegible]	[illegible]
Acrdi.XM_015911416.1/1-834 | Monca.augustus.g9159.t1/1-846	0.0456572	0.0373803	Calcium-dependent kinase 5	1	F:GO:0005509	[illegible]
Acrdi.XM_015901644.1/1-1125 | Monca.augustus.g40563.t1/1-1125	0.636937	0.0373421	PREDICTED: uncharacterized protein LOC107336575			
Acrdi.XM_015896660.1/1-720 | Monca.augustus.g12336.t1/1-1587	0.0668309	0.0373363	epsin-2 isoform X2			
Acrdi.XM_015911238.1/1-1482 | Monca.augustus.g41212.t1/1-2778	0.0444255	0.0373332	F-box only 11-like	3	[illegible]	[illegible]
Acrdi.XM_015911478.1/1-975 | Monca.augustus.g19672.t1/1-978	0.0337207	0.0372892	calretinin	3	[illegible]	[illegible]
Acrdi.XM_015914368.1/1-921 | Monca.adi2mcaRNA24915_R2/1-888	0.264006	0.0372755	TNF receptor-associated factor 5-like isoform X2			
Acrdi.XM_015915734.1/1-1149 | Monca.augustus.g41831.t1/1-1146	0.0701287	0.0372713	signal peptide peptidase-like 3	8	[illegible]	[illegible]
Acrdi.XM_015919392.1/1-831 | Monca.augustus.g23336.t1/1-1755	0.303988	0.0372119	AP1 endonuclease			
Acrdi.XM_015921342.1/1-768 | Monca.adi2mcaRNA3297_R9/1-960	1.30036	0.0371877	Pro-Pol poly			
Acrdi.XM_015903567.1/1-360 | Monca.augustus.g53523.t1/1-669	0.0351321	0.0371837	Charged multivesicular body 5	2	P:GO:0007034; C:GO:0005622	[illegible]
Acrdi.XM_015916678.1/1-2934 | Monca.augustus.g67110.t1/1-3243	0.0508279	0.0371222	Armadillo repeat-containing 4	4	[illegible]	[illegible]
Acrdi.XM_015912673.1/1-741 | Monca.augustus.g53964.t1/1-744	0.0530787	0.0371178	retinal homeobox Rx1-like	3	[illegible]	[illegible]
Acrdi.XM_015893283.1/1-726 | Monca.augustus.g67442.t1/1-729	0.0519206	0.0371168	BTB POZ domain-containing KCTD16	1	P:GO:0051260	[illegible]
Acrdi.XM_015922215.1/1-1791 | Monca.augustus.g3042.t1/1-1689	0.0478358	0.0371084	Coiled-coil domain-containing 93			
Acrdi.XM_015920419.1/1-1536 | Monca.augustus.g61323.t1/1-1548	0.489558	0.0371019	PREDICTED: uncharacterized protein LOC107354018			
Acrdi.XM_015925186.1/1-894 | Monca.adi2mcaRNA37098_R0/1-852	0.481269	0.0370854	PREDICTED: uncharacterized protein LOC107358577			
Acrdi.XM_015911873.1/1-939 | Monca.augustus.g17904.t1/1-939	0.0494803	0.0370822	Mitochondrial carnitine acylcarnitine carrier	4	[illegible]	[illegible]
Acrdi.XM_015896251.1/1-366 | Monca.augustus.g33745.t1/1-366	0.0333283	0.0370808	Sjoegren syndrome nuclear autoantigen 1 homolog			
Acrdi.XM_015911451.1/1-645 | Monca.augustus.g53639.t1/1-1122	0.0307924	0.0370525	Casein kinase II subunit alpha	3	[illegible]	[illegible]
Acrdi.XM_015915086.1/1-1215 | Monca.augustus.g64357.t1/1-1218	0.0571962	0.0369123	dnaJ homolog subfamily A member 2	10	[illegible]	[illegible]
Acrdi.XM_015905878.1/1-768 | Monca.augustus.g32228.t1/1-774	0.0429286	0.0368886	haloacid dehalogenase-like hydrolase domain-containing 2	2	F:GO:0016791; P:GO:0016311	[illegible]
Acrdi.XM_015910318.1/1-723 | Monca.augustus.g36436.t1/1-750	0.0670066	0.0368764	Syntaxin-6	9	[illegible]	[illegible]
Acrdi.XM_015917994.1/1-690 | Monca.augustus.g56732.t1/1-693	0.0792963	0.0368737	nuclear transcription factor Y subunit alpha-like isoform X2	4	[illegible]	[illegible]
Acrdi.XM_015897358.1/1-2034 | Monca.augustus.g29322.t1/1-2346	0.0615252	0.0367373	HID1	4	[illegible]	[illegible]
Acrdi.XM_015892155.1/1-963 | Monca.adi2mcaRNA38058_R0/1-969	0.0450286	0.0367037	breast cancer metastasis-suppressor 1 -A	5	[illegible]	[illegible]
Acrdi.XM_015919989.1/1-1494 | Monca.augustus.g16010.t1/1-1491	0.0462186	0.0366604	Serine palmitoyltransferase 1	4	[illegible]	[illegible]
Acrdi.XM_015924049.1/1-705 | Monca.adi2mcaRNA35780_R2/1-660	0.246457	0.036626	P2X purinoceptor 7-like	2	C:GO:0016020; C:GO:0016021	[illegible]
Acrdi.XM_015908463.1/1-501 | Monca.augustus.g33940.t1/1-501	0.0431472	0.0366166	RNA-binding 8A	6	[illegible]	[illegible]
Acrdi.XM_015919767.1/1-1347 | Monca.augustus.g48087.t1/1-1347	0.0408877	0.0366045	PREDICTED: T-complex-associated testis-expressed protein 1-like			
Acrdi.XM_015916204.1/1-1311 | Monca.augustus.g2725.t1/1-2718	0.0462293	0.0365554	26S proteasome non-ATPase regulatory subunit 2	8	[illegible]	[illegible]
Acrdi.XM_015900073.1/1-1707 | Monca.adi2mcaRNA9233_R6/1-2022	0.274772	0.0365543	RNA-directed DNA polymerase from mobile element jockey			
Acrdi.XM_015921499.1/1-471 | Monca.augustus.g66251.t1/1-423	0.612233	0.036512	G2 M phase-specific E3 ubiquitin- ligase-like			
Acrdi.XM_015913930.1/1-1395 | Monca.augustus.g59527.t1/1-1398	0.0566854	0.036502	Regulator of G- signaling 7	7	[illegible]	[illegible]
Acrdi.XM_015895222.1/1-705 | Monca.augustus.g40346.t1/1-753	0.0483705	0.0364899	bridging integrator 3	2	F:GO:0019904; C:GO:0005737	[illegible]
Acrdi.XM_015919775.1/1-975 | Monca.adi2mcaRNA30986_R3/1-975	0.686934	0.0364457	unc-13-like C	5	[illegible]	[illegible]
Acrdi.XM_015915523.1/1-1056 | Monca.augustus.g42677.t1/1-1008	1.01371	0.0363124	PREDICTED: uncharacterized protein LOC107349375	9	[illegible]	[illegible]
Acrdi.XM_015907901.1/1-1380 | Monca.adi2mcaRNA17847_R1/1-1380	0.0448937	0.0362982	vacuolar fusion CCZ1 homolog	4	[illegible]	[illegible]
Acrdi.XM_015893262.1/1-2715 | Monca.adi2mcaRNA39334_R0/1-2556	0.0469102	0.0362842	RNA polymerase-associated CTR9 homolog	8	[illegible]	[illegible]
Acrdi.XM_015910966.1/1-1536 | Monca.augustus.g3082.t1/1-1539	0.0546703	0.0362659	retinoblastoma-binding 5 homolog	5	[illegible]	[illegible]
Acrdi.XM_015919843.1/1-363 | Monca.adi2mcaRNA31067_R8/1-348	0.235713	0.0362416	PREDICTED: uncharacterized protein LOC107353524			
Acrdi.XM_015922066.1/1-1443 | Monca.augustus.g11602.t1/1-1446	0.0439868	0.0362324	calcium calmodulin-dependent kinase type II alpha chain isoform X6	18	[illegible]	[illegible]
Acrdi.XM_015923247.1/1-891 | Monca.adi2mcaRNA34878_R0/1-774	0.50588	0.036084	PREDICTED: uncharacterized protein LOC107356638			
Acrdi.XM_015907886.1/1-951 | Monca.augustus.g53169.t1/1-951	0.0501778	0.036079	glutathione S-transferase omega-like 2			
Acrdi.XM_015911435.1/1-375 | Monca.augustus.g7456.t1/1-375	0.0503084	0.0360496	NADH dehydrogenase [ubiquinone] 1 beta subcomplex subunit 9	3	[illegible]	[illegible]
Acrdi.XM_015904620.1/1-2487 | Monca.adi2mcaRNA14136_R0/1-1668	0.627372	0.0360278	52 kDa repressor of the inhibitor of the kinase-like			
Acrdi.XM_015906904.1/1-1998 | Monca.augustus.g8903.t1/1-2073	0.0432409	0.0360004	probable ATP-dependent RNA helicase DDX5 isoform X1	5	[illegible]	[illegible]
Acrdi.XM_015906764.1/1-1026 | Monca.adi2mcaRNA16564_R8/1-1998	0.605447	0.0359923	RNA-directed DNA polymerase from mobile element jockey-like			
Acrdi.XM_015924525.1/1-1356 | Monca.augustus.g40900.t1/1-1356	0.0412461	0.0359528	WD repeat and SOCS box-containing 1-like	2	C:GO:0005622; P:GO:0035556	[illegible]
[illegible]	0.0306928	0.0359048	6-phosphofructo-2-kinase fructose-2,6-bisphosphatase-like isoform X1	5	[illegible]	[illegible]
[illegible]	[illegible]	[illegible]	[illegible]	[illegible]	[illegible]	[illegible]

Acrdi.XM_015923177.1/1-948	Monca.augustus.g49864.t1/1-1086	0.0375129	0.0348119	calcium calmodulin-dependent kinase type 1-like isoform X1	3
Acrdi.XM_015909697.1/1-1131	Monca.augustus.g55278.t1/1-1131	0.0464185	0.034777	Ubiquitin-conjugating enzyme E2 Q1	1
Acrdi.XM_015895805.1/1-1239	Monca.augustus.g47627.t1/1-1350	0.0518397	0.0347252	Elongation factor mitochondrial	5
Acrdi.XM_015897862.1/1-951	Monca.augustus.g39399.t1/1-963	0.0435471	0.0346794	ubiquitin-like-conjugating enzyme ATG3	7
Acrdi.XM_015919532.1/1-2040	Monca.adi2mcaRNA30718_R0/1-2049	0.0494823	0.0346738	fermitin family homolog 2-like	6
Acrdi.XM_015924816.1/1-900	Monca.augustus.g67354.t1/1-906	0.0380996	0.0346697	acidic leucine-rich nuclear phospho 32 family member B-like	
Acrdi.XM_015925173.1/1-1359	Monca.adi2mcaRNA37083_R0/1-1338	0.0316479	0.0346637	EF-hand domain-containing 1-like	1
Acrdi.XM_015909744.1/1-6150	Monca.adi2mcaRNA19804_R0/1-6234	0.421721	0.0346396	Tetratricopeptide repeat 28	
Acrdi.XM_015924505.1/1-1413	Monca.augustus.g28542.t1/1-1413	0.0692009	0.0346133	zinc finger ZIC 4-like	2
Acrdi.XM_015912909.1/1-6081	Monca.adi2mcaRNA2531_R0/1-6312	0.264277	0.0345654	PREDICTED: uncharacterized protein LOC107347040	1
Acrdi.XM_015907111.1/1-402	Monca.augustus.g36980.t1/1-402	0.0534212	0.0345648	achaete-scute homolog 4	5
Acrdi.XM_015898607.1/1-3939	Monca.adi2mcaRNA7616_R0/1-2625	0.585682	0.0345595	tetratricopeptide repeat 28-like	
Acrdi.XM_015910503.1/1-666	Monca.augustus.g25424.t1/1-666	0.04796	0.0345517	thioredoxin domain-containing 9	5
Acrdi.XM_015909154.1/1-795	Monca.adi2mcaRNA19197_R1/1-762	0.229126	0.0345326	nucleoporin GLE1-like	
Acrdi.XM_015904534.1/1-1626	Monca.augustus.g16841.t1/1-1731	0.0526449	0.0345225	pre-mRNA-processing factor 17	5
Acrdi.XM_015905311.1/1-1008	Monca.adi2mcaRNA1536_R6/1-891	0.532644	0.0345029	RNA-directed DNA polymerase from mobile element jockey	
Acrdi.XM_015917728.1/1-2694	Monca.augustus.g19447.t1/1-3084	0.505064	0.0344408	Gypsy retrotransposon integrase 1	1
Acrdi.XM_015892254.1/1-1257	Monca.adi2mcaRNA38168_R0/1-1182	0.285639	0.034415	ATP-dependent DNA helicase Q1	
Acrdi.XM_015895706.1/1-1329	Monca.augustus.g68548.t1/1-1524	0.0336996	0.0343588	tRNA-splicing ligase homolog	5
Acrdi.XM_015905234.1/1-1218	Monca.augustus.g1762.t1/1-1218	0.0486378	0.0343134	COP9 signalosome complex subunit 4 isoform X1	11
Acrdi.XM_015911872.1/1-1383	Monca.augustus.g17903.t1/1-1527	0.0575111	0.0342947	dihydrolipoyl mitochondrial isoform X3	28
Acrdi.XM_015896255.1/1-438	Monca.augustus.g33735.t1/1-1140	0.129844	0.0342825	nematogalectin	2
Acrdi.XM_015895624.1/1-651	Monca.augustus.g22289.t1/1-651	0.0593349	0.0342325	neuralized 4	2
Acrdi.XM_015897996.1/1-915	Monca.augustus.g30590.t1/1-975	0.0486504	0.034229	sideroflexin-1	6
Acrdi.XM_015893273.1/1-996	Monca.augustus.g20820.t1/1-2466	0.274789	0.0342083	PREDICTED: uncharacterized protein LOC107328551	
Acrdi.XM_015909393.1/1-1557	Monca.augustus.g32246.t1/1-2160	0.0421866	0.0341343	glycerol-3-phosphate mitochondrial-like isoform X1	5
Acrdi.XM_015905997.1/1-990	Monca.augustus.g39216.t1/1-990	0.418694	0.0341131	PREDICTED: uncharacterized protein LOC107340631	
Acrdi.XM_015903741.1/1-2052	Monca.augustus.g41344.t1/1-2067	0.0351882	0.0341093	segment polarity dishevelled homolog DVL-3 isoform X2	19
Acrdi.XM_015919429.1/1-1161	Monca.adi2mcaRNA30593_R0/1-1224	0.43348	0.0340478	nuclease HARBI1	
Acrdi.XM_015920021.1/1-1356	Monca.adi2mcaRNA31266_R0/1-1389	1.56449	0.0340354	PREDICTED: uncharacterized protein LOC107353679	3
Acrdi.XM_015904948.1/1-549	Monca.augustus.g17419.t1/1-549	0.0445085	0.0339579	Dynactin subunit 6	2
Acrdi.XM_015915119.1/1-1071	Monca.augustus.g51869.t1/1-762	0.566626	0.0339321	PREDICTED: uncharacterized protein LOC107349022	
Acrdi.XM_015910641.1/1-2928	Monca.augustus.g37493.t1/1-3048	0.0445579	0.0339074	transcription elongation factor SPT5 isoform X1	8
Acrdi.XM_015923469.1/1-795	Monca.adi2mcaRNA35138_R1/1-795	0.99204	0.0339041	UPF0746 DDB_G0281095-like	
Acrdi.XM_015909193.1/1-3354	Monca.augustus.g57521.t1/1-1851	0.0345983	0.0338784	coatomer subunit beta	6
Acrdi.XM_015913521.1/1-384	Monca.augustus.g69413.t1/1-663	0.0612881	0.03386	programmed cell death 6-like	4
Acrdi.XM_015909884.1/1-477	Monca.adi2mcaRNA29302_R8/1-1005	1.87379	0.0338235	zinc finger MYM-type 3-	
Acrdi.XM_015906903.1/1-780	Monca.augustus.g8909.t1/1-1008	0.0382882	0.0338085	nuclear distribution nudE homolog 1	16
Acrdi.XM_015909814.1/1-1896	Monca.adi2mcaRNA19932_R0/1-1929	0.0410868	0.0337661	Bardet-Biedl syndrome 2	6
Acrdi.XM_015917719.1/1-1143	Monca.augustus.g42293.t1/1-1161	0.0329636	0.0337657	PC3-like endoprotease variant B	3
Acrdi.XM_015901443.1/1-1107	Monca.augustus.g6349.t1/1-1038	0.0386778	0.0337241	Cytoplasmic tRNA 2-thiolation 1	6
Acrdi.XM_015893410.1/1-855	Monca.augustus.g34510.t1/1-1086	0.0469348	0.0335347	Fructose-bisphosphate aldolase C	2
Acrdi.XM_015916011.1/1-1212	Monca.augustus.g70002.t1/1-2007	0.0404876	0.0334748	grainyhead 2 homolog	4
Acrdi.XM_015919342.1/1-585	Monca.augustus.g15438.t1/1-594	0.0630032	0.0333534	RNA-binding -like	1
Acrdi.XM_015923539.1/1-2481	Monca.adi2mcaRNA35205_R2/1-3042	0.616479	0.0333386	Transposon Tf2-6 poly	
Acrdi.XM_015917718.1/1-999	Monca.adi2mcaRNA2894_R0/1-996	1.82479	0.0332929	52 kDa repressor of the inhibitor of the kinase-like	3
Acrdi.XM_015902099.1/1-1476	Monca.augustus.g4705.t1/1-2715	0.0382238	0.0332643	Glycoside family 63	4
Acrdi.XM_015893082.1/1-1662	Monca.adi2mcaRNA39119_R0/1-1653	0.225271	0.0331902	ac transposable element-derived 4	
Acrdi.XM_015912794.1/1-438	Monca.augustus.g12460.t1/1-954	0.0468998	0.0331331	cytosolic Fe-S cluster assembly factor nubp1	5
Acrdi.XM_015896184.1/1-1107	Monca.adi2mcaRNA483_R1/1-1092	0.0417581	0.0331056	adenylosuccinate synthetase isozyme 2-like	6
Acrdi.XM_015917565.1/1-342	Monca.augustus.g44188.t1/1-342	0.0460285	0.0330881	Transcription elongation factor SPT4	9
Acrdi.XM_015908203.1/1-318	Monca.augustus.g4571.t1/1-324	0.0379268	0.0330792	Asterix	1
Acrdi.XM_015897730.1/1-1041	Monca.augustus.g50756.t1/1-1284	0.048544	0.0330407	flotillin-1 isoform X2	
Acrdi.XM_015918178.1/1-564	Monca.augustus.g66566.t1/1-1422	0.04467	0.0329053	myc box-dependent-interacting 1 isoform X3	1
Acrdi.XM_015912062.1/1-624	Monca.augustus.g38035.t1/1-624	0.0449165	0.0328942	lethal(2)essential for life	
Acrdi.XM_015912994.1/1-924	Monca.augustus.g64760.t1/1-924	0.0427933	0.0328925	potassium channel subfamily K member 16-like	4
Acrdi.XM_015895504.1/1-363	Monca.augustus.g25938.t1/1-576	0.037999	0.0328827	Sorcin	4
Acrdi.XM_015919047.1/1-744	Monca.adi2mcaRNA30197_R7/1-780	0.454827	0.0328804	RNA-directed DNA polymerase from mobile element jockey	
Acrdi.XM_015895348.1/1-846	Monca.augustus.g51319.t1/1-1296	0.321179	0.0328639	zinc finger MYM-type 1-like	
Acrdi.XM_015919085.1/1-828	Monca.augustus.g53478.t1/1-2142	0.355756	0.0328522	PREDICTED: uncharacterized protein LOC107352772	
Acrdi.XM_015911264.1/1-597	Monca.adi2mcaRNA21478_R0/1-594	0.557702	0.0328185	ATP-dependent DNA helicase PIF1	7
Acrdi.XM_015903181.1/1-1386	Monca.augustus.g71360.t1/1-1386	0.0467979	0.0328016	tyrosine- kinase CSK-like	
Acrdi.XM_015897071.1/1-1182	Monca.augustus.g70185.t1/1-1662	0.046651	0.0327473	CCR4-NOT transcription complex subunit 6-like isoform X1	
Acrdi.XM_015899899.1/1-2874	Monca.augustus.g44169.t1/1-3678	0.0544019	0.032715	Coatomer subunit alpha	7
Acrdi.XM_015900467.1/1-1404	Monca.adi2mcaRNA934_R0/1-1410	0.0587822	0.0326822	General transcription factor IIH subunit 4	10
Acrdi.XM_015905325.1/1-624	Monca.adi2mcaRNA14963_R0/1-609	0.39078	0.0326816	PREDICTED: uncharacterized protein LOC107339967	
Acrdi.XM_015920602.1/1-2034	Monca.adi2mcaRNA31891_R0/1-1974	0.267291	0.0326546	PREDICTED: uncharacterized protein LOC107354160	3
Acrdi.XM_015899955.1/1-951	Monca.augustus.g10900.t1/1-945	0.036602	0.0326493	guanine nucleotide-binding subunit beta-2-like 1	64
Acrdi.XM_015913286.1/1-363	Monca.augustus.g70500.t1/1-363	0.0168837	0.0326307	Serine threonine- kinase Nek1	
Acrdi.XM_015906923.1/1-1140	Monca.augustus.g23310.t1/1-621	0.0438787	0.0326076	NADH dehydrogenase [ubiquinone] iron-sulfur mitochondrial	4
Acrdi.XM_015897015.1/1-11436	Monca.adi2mcaRNA628_R0/1-11199	0.0450408	0.0326035	dynein heavy chain axonemal	5
Acrdi.XM_015909935.1/1-510	Monca.augustus.g5528.t1/1-504	1.10083	0.0325683	PREDICTED: uncharacterized protein LOC107344282	
Acrdi.XM_015893628.1/1-1962	Monca.adi2mcaRNA568_R2/1-1983	0.405073	0.0324889	RNA-directed DNA polymerase from mobile element jockey-like	
Acrdi.XM_015892422.1/1-1092	Monca.augustus.g32811.t1/1-1095	0.0250689	0.0324783	developmentally-regulated GTP-binding 2	1
Acrdi.XM_015892212.1/1-900	Monca.augustus.g10168.t1/1-879	0.225168	0.0324471	UPF0746 DDB_G0281095-like	
Acrdi.XM_015891674.1/1-2229	Monca.augustus.g22644.t1/1-2619	0.405536	0.0323721	zinc finger 862-like	
Acrdi.XM_015918202.1/1-948	Monca.augustus.g63761.t1/1-1389	0.174888	0.0323569	THAP domain-containing 3	
Acrdi.XM_015921966.1/1-1170	Monca.adi2mcaRNA33372_R1/1-888	0.197139	0.0323296	Pro-Pol poly	
Acrdi.XM_015892503.1/1-1821	Monca.adi2mcaRNA38448_R0/1-1821	0.0448473	0.0322768	Electron transfer flavo -ubiquinone mitochondrial	12
Acrdi.XM_015904266.1/1-588	Monca.augustus.g32325.t1/1-588	0.0401612	0.0322025	neurocalcin homolog	1
Acrdi.XM_015919905.1/1-930	Monca.adi2mcaRNA31159_R0/1-1086	0.256559	0.032139	PREDICTED: uncharacterized protein LOC107353576	
Acrdi.XM_015899518.1/1-516	Monca.augustus.g26774.t1/1-516	0.0420305	0.0321364	tubulin polymerization-promoting family member 2	
Acrdi.XM_015919885.1/1-315	Monca.augustus.g33616.t1/1-315	0.0231531	0.0321345	C-Myc-binding -like	3
Acrdi.XM_015902783.1/1-1191	Monca.augustus.g33296.t1/1-3210	0.0480477	0.0320885	SNW domain-containing 1 isoform X1	22
Acrdi.XM_015919917.1/1-1860	Monca.adi2mcaRNA31158_R1/1-1698	0.258298	0.0320734	Chromatin modification-related png1	
Acrdi.XM_015903121.1/1-588	Monca.adi2mcaRNA12469_R2/1-588	1.04479	0.0320692	aspartyl asparaginyl beta-hydroxylase-like	
Acrdi.XM_015901966.1/1-1101	Monca.augustus.g45525.t1/1-1092	0.0707712	0.0320392	LIM homeobox Lhx1	26
Acrdi.XM_015906788.1/1-1050	Monca.augustus.g40433.t1/1-1221	0.0227903	0.0320198	Septin-2	3
Acrdi.XM_015924164.1/1-903	Monca.augustus.g63776.t1/1-903	0.255845	0.0319965	REM2- and Rab-like small GTPase 1	
Acrdi.XM_015913625.1/1-471	Monca.augustus.g48692.t1/1-471	0.036283	0.0319405	trafficking particle complex subunit 6B-like	4
Acrdi.XM_015922515.1/1-795	Monca.adi2mcaRNA3473_R3/1-768	5.89689	0.0319403	PREDICTED: uncharacterized protein LOC107355898	
Acrdi.XM_015899810.1/1-402	Monca.augustus.g48638.t1/1-1713	0.0771642	0.0318332	HORMA domain-containing 1-like	
Acrdi.XM_015906974.1/1-1206	Monca.augustus.g23303.t1/1-1206	0.0416356	0.0318016	tektin-1 isoform X1	1
Acrdi.XM_015920719.1/1-699	Monca.augustus.g44239.t1/1-1101	0.929087	0.0317748	Werner syndrome ATP-dependent helicase	1
Acrdi.XM_015900260.1/1-3261	Monca.adi2mcaRNA9480_R0/1-3360	0.487171	0.0317407	Retrovirus-related Pol poly from transposon	
Acrdi.XM_015896546.1/1-1317	Monca.augustus.g29576.t1/1-1317	0.0354883	0.031702	vesicular inhibitory amino acid transporter-like	3
Acrdi.XM_015902077.1/1-2208	Monca.adi2mcaRNA11417_R7/1-2349	0.396415	0.0316967	Retrovirus-related Pol poly from transposon	
Acrdi.XM_015918531.1/1-636	Monca.augustus.g50499.t1/1-630	0.350885	0.0316901	PREDICTED: uncharacterized protein LOC107352198	
Acrdi.XM_015907603.1/1-1947	Monca.augustus.g8664.t1/1-2064	0.0426712	0.0316842	anoctamin-8-like isoform X1	1
Acrdi.XM_015921598.1/1-645	Monca.augustus.g41677.t1/1-645	0.0585155	0.0316774	vesicle-trafficking SEC22b	13
Acrdi.XM_015910215.1/1-1170	Monca.adi2mcaRNA20397_R5/1-1098	0.68333	0.0316704	ATP-binding cassette sub-family B member mitochondrial	
Acrdi.XM_015919042.1/1-1317	Monca.augustus.g56506.t1/1-1230	0.0493108	0.0316379	Tryptophan--tRNA cytoplasmic	10
Acrdi.XM_015908791.1/1-1209	Monca.augustus.g4693.t1/1-4830	0.0527679	0.031636	serine threonine- kinase WNK3-like	
Acrdi.XM_015903255.1/1-663	Monca.augustus.g51690.t1/1-663	0.0533687	0.0316238	unc-119 homolog B-like	1
Acrdi.XM_015918250.1/1-1284	Monca.augustus.g48573.t1/1-1284	0.0563946	0.0316148	multifunctional ADE2-like	6
Acrdi.XM_015919156.1/1-2160	Monca.adi2mcaRNA30308_R0/1-2082	0.511923	0.0315913	PREDICTED: uncharacterized protein LOC107352838	
Acrdi.XM_015907045.1/1-1404	Monca.augustus.g43306.t1/1-2061	0.0305721	0.0315806	kinase C alpha type	6
Acrdi.XM_015908858.1/1-2700	Monca.adi2mcaRNA18830_R0/1-2463	0.0391176	0.0315762	orofacial cleft 1 candidate gene 1	1
Acrdi.XM_015899364.1/1-4677	Monca.adi2mcaRNA331_R0/1-4167	0.0516503	0.0315742	otoferlin isoform X1	1
Acrdi.XM_015925005.1/1-1575	Monca.augustus.g53481.t1/1-1758	0.0529154	0.0315617	Serine threonine- phosphatase 2A 65 kDa regulatory subunit A alpha is	1
Acrdi.XM_015900556.1/1-525	Monca.augustus.g26466.t1/1-564	0.0500167	0.0315119	Trafficking particle complex subunit 5	
Acrdi.XM_015914531.1/1-11847	Monca.adi2mcaRNA25134_R0/1-6165	0.034078	0.0314845	dynein heavy chain axonemal	6
Acrdi.XM_015905580.1/1-498	Monca.augustus.g24100.t1/1-1023	0.0551791	0.0314774	transmembrane 185A	1
Acrdi.XM_015903176.1/1-3270	Monca.augustus.g71349.t1/1-5103	0.0428264	0.0314634	unconventional myosin-Ie-like	
Acrdi.XM_015919828.1/1-657	Monca.adi2mcaRNA31064_R9/1-657	0.360864	0.0314397	PREDICTED: uncharacterized protein K02A2.6-like	
Acrdi.XM_015903479.1/1-6453	Monca.adi2mcaRNA1443_R0/1-6264	0.0421054	0.0313879	DNA polymerase epsilon catalytic subunit A	15
Acrdi.XM_015923286.1/1-1416	Monca.adi2mcaRNA34947_R6/1-1557	0.439337	0.0313587	RNA-directed DNA polymerase from transposon BS	
Acrdi.XM_015905728.1/1-11199	Monca.augustus.g32746.t1/1-5184	0.0383246	0.031297	dynein heavy chain axonemal	
Acrdi.XM_015897668.1/1-522	Monca.augustus.g51286.t1/1-522	1.31492	0.0312898	PREDICTED: uncharacterized protein LOC107332915	
Acrdi.XM_015903170.1/1-1977	Monca.augustus.g32272.t1/1-2259	0.0392892	0.0312641	Cullin-3	34
Acrdi.XM_015908744.1/1-861	Monca.augustus.g55162.t1/1-840	0.0454199	0.031245	quaking-B isoform X2	2
Acrdi.XM_015922459.1/1-3990	Monca.adi2mcaRNA3449_R0/1-3876	0.0516358	0.0312198	cleavage and polyadenylation specificity factor subunit 1	4
Acrdi.XM_015916200.1/1-954	Monca.augustus.g55490.t1/1-1257	0.0420394	0.0311959	isovaleryl- mitochondrial	8
Acrdi.XM_015923825.1/1-384	Monca.augustus.g33429.t1/1-384	0.0468379	0.0311889	DNA-directed RNA polymerases and III subunit RPABC2	4
Acrdi.XM_015903160.1/1-432	Monca.augustus.g14566.t1/1-432	0.748249	0.0311797	PREDICTED: uncharacterized protein LOC107337912	
Acrdi.XM_015892772.1/1-450	Monca.augustus.g33463.t1/1-450	0.0496071	0.0311353	NADH dehydrogenase [ubiquinone] 1 alpha subcomplex subunit 11	1
Acrdi.XM_015909322.1/1-1566	Monca.adi2mcaRNA19396_R0/1-1785	0.467677	0.0311255	reverse	2
Acrdi.XM_015900863.1/1-423	Monca.augustus.g68341.t1/1-423	0.0443307	0.0311043	Ribonuclease UK114	21
Acrdi.XM_015904068.1/1-1314	Monca.adi2mcaRNA13570_R0/1-1353	0.302689	0.0310629	ATP-dependent DNA helicase Q1	
Acrdi.XM_015904067.1/1-900	Monca.augustus.g33560.t1/1-711	0.969479	0.0309129	zinc finger 862-like	
Acrdi.XM_015896888.1/1-366	Monca.augustus.g33169.t1/1-447	0.0321485	0.0309001	Retinal rod rhodopsin-sensitive cGMP 3 ,5 -cyclic phosphodiesterase s	4
Acrdi.XM_015898447.1/1-1032	Monca.augustus.g41766.t1/1-1008	0.0496632	0.0308666	LIM and senescent cell antigen-like-containing domain 1	1
Acrdi.XM_015892069.1/1-1971	Monca.augustus.g1134.t1/1-1950	0.0456485	0.0308465	kinesin KIF25	4
Acrdi.XM_015895443.1/1-411	Monca.augustus.g12571.t1/1-1551	0.0319166	0.0307798	Pleiotropic regulator 1	6
Acrdi.XM_015908105.1/1-2178	Monca.augustus.g33244.t1/1-2190	0.0226736	0.0307404	heat shock HSP 90-alpha	8
Acrdi.XM_015904518.1/1-1197	Monca.augustus.g33573.t1/1-1203	0.0420007	0.0307339	radial spoke head 3 homolog	
Acrdi.XM_015897966.1/1-885	Monca.augustus.g63449.t1/1-885	0.779405	0.0306918	exonuclease 1-like	1
Acrdi.XM_015922866.1/1-453	Monca.adi2mcaRNA34406_R0/1-480	0.827396	0.0306732	PREDICTED: uncharacterized protein LOC107356229	
Acrdi.XM_015919185.1/1-2694	Monca.augustus.g70410.t1/1-2685	0.312376	0.0306684	PREDICTED: uncharacterized protein LOC107353071	
Acrdi.XM_015897186.1/1-1932	Monca.adi2mcaRNA6041_R0/1-1797	0.277104	0.0306589	PREDICTED: uncharacterized protein LOC107332451	
Acrdi.XM_015897261.1/1-1434	Monca.augustus.g13129.t1/1-1491	0.260562	0.0306391	PREDICTED: uncharacterized protein LOC107332521	
Acrdi.XM_015896928.1/1-948	Monca.augustus.g28890.t1/1-1107	0.0317091	0.0306356	Photoreceptor-specific nuclear receptor	9
Acrdi.XM_015919651.1/1-462	Monca.augustus.g32524.t1/1-462	0.0391861	0.0305949	nudC domain-containing 2	
Acrdi.XM_015900652.1/1-603	Monca.augustus.g28781.t1/1-600	0.05041	0.0305661	charged multivesicular body 1b	2
Acrdi.XM_015918391.1/1-2499	Monca.augustus.g22402.t1/1-2436	0.0375473	0.0305242	cullin-4B isoform X2	14
Acrdi.XM_015913176.1/1-672	Monca.augustus.g10870.t1/1-672	0.0559261	0.0305078	ropporin-1	
Acrdi.XM_015905803.1/1-1716	Monca.adi2mcaRNA15474_R5/1-2100	0.775912	0.0305034	Pao retrotransposon peptidase family	
Acrdi.XM_015899710.1/1-1761	Monca.augustus.g68164.t1/1-2358	0.0523478	0.0304856	serine threonine- kinase MARK1-like	3
Acrdi.XM_015911400.1/1-834	Monca.augustus.g53225.t1/1-855	0.0540842	0.0304456	transcription factor SOX-14-like	1
Acrdi.XM_015914018.1/1-1629	Monca.augustus.g44837.t1/1-1629	0.0452184	0.0304392	T-complex 1 subunit theta isoform X2	5
Acrdi.XM_015909363.1/1-840	Monca.adi2mcaRNA19450_R0/1-870	0.0526111	0.0303837	carnitine-acylcarnitine carrier	4
Acrdi.XM_015897661.1/1-654	Monca.augustus.g50735.t1/1-669	0.0344529	0.030355	casein kinase II subunit beta isoform X1	19
Acrdi.XM_015896240.1/1-570	Monca.augustus.g54599.t1/1-540	0.0407213	0.0303506	ER membrane complex subunit 4	1
Acrdi.XM_015920404.1/1-582	Monca.augustus.g46002.t1/1-582	0.0401996	0.0303384	ras rasD	4
Acrdi.XM_015919108.1/1-606	Monca.adi2mcaRNA30234_R9/1-513	0.211861	0.0302765	TNF receptor-associated factor 5-like isoform X2	
Acrdi.XM_015901226.1/1-3519	Monca.adi2mcaRNA10485_R1/1-3513	0.363617	0.0302597	Transposon Tf2-6 poly	
Acrdi.XM_015918289.1/1-972	Monca.adi2mcaRNA29305_R8/1-978	0.328359	0.0302493	Transposon Ty3-G Gag-Pol poly	
Acrdi.XM_015901609.1/1-8415	Monca.adi2mcaRNA10914_R0/1-8073	0.486722	0.0302398	bark beetle-like	8
Acrdi.XM_015916657.1/1-4134	Monca.adi2mcaRNA27480_R1/1-4026	0.0357141	0.0302203	Carbamoyl-phosphate synthase [ammonia] mitochondrial	
Acrdi.XM_015894061.1/1-723	Monca.augustus.g50905.t1/1-2115	0.035972	0.0302031	leucine-rich repeat and calponin homology domain-containing 1-like	
Acrdi.XM_015915855.1/1-846	Monca.augustus.g56360.t1/1-852	0.0788783	0.0301975	E3 ubiquitin- ligase BRE1A-like	
Acrdi.XM_015919323.1/1-1416	Monca.augustus.g15441.t1/1-1713	0.0505026	0.0301957	negative elongation factor C D	2
Acrdi.XM_015923038.1/1-567	Monca.augustus.g57884.t1/1-642	0.448729	0.0300795	PREDICTED: uncharacterized protein LOC107356421	
Acrdi.XM_015908094.1/1-318	Monca.augustus.g33231.t1/1-1527	0.0300443	0.0299982	Tetratricopeptide repeat 8	
Acrdi.XM_015905368.1/1-2418	Monca.adi2mcaRNA15014_R2/1-2172	0.421141	0.0299567	Transposable element Tcb2	
Acrdi.XM_015894120.1/1-657	Monca.adi2mcaRNA40345_R0/1-654	0.187059	0.0299247	PREDICTED: uncharacterized protein LOC107329432	1
Acrdi.XM_015901943.1/1-894	Monca.augustus.g45547.t1/1-894	0.0460064	0.0298007	Translation initiation factor eIF-2B subunit alpha	8
Acrdi.XM_015920700.1/1-444	Monca.augustus.g31372.t1/1-372	1.40819	0.0297823	PREDICTED: uncharacterized protein LOC107354245	
Acrdi.XM_015922949.1/1-1200	Monca.adi2mcaRNA3472_R1/1-1284	0.727676	0.029772	PREDICTED: uncharacterized protein LOC107356331	
Acrdi.XM_015914450.1/1-717	Monca.augustus.g56328.t1/1-714	0.0284521	0.0297127	Polycomb group RING finger 3	2
Acrdi.XM_015917618.1/1-384	Monca.augustus.g9236.t1/1-384	0.0223099	0.0297116	NADH dehydrogenase 1 alpha subcomplex subunit 8	3
Acrdi.XM_015904381.1/1-6207	Monca.augustus.g48064.t1/1-7437	0.0396397	0.0297004	Serine threonine- kinase mTOR	6
Acrdi.XM_015916628.1/1-732	Monca.adi2mcaRNA27445_R0/1-549	0.0394837	0.0296376	pre-mRNA-splicing factor ATP-dependent RNA helicase DHX15	3
Acrdi.XM_015892814.1/1-1338	Monca.augustus.g8564.t1/1-1254	0.26672	0.0296367	piggyBac transposable element-derived 4-like	
Acrdi.XM_015905290.1/1-819	Monca.augustus.g23141.t1/1-819	0.0353999	0.0296156	pyrroline-5-carboxylate reductase mitochondrial-like	3
Acrdi.XM_015923471.1/1-9255	Monca.augustus.g42216.t1/1-12999	0.0390266	0.0295754	Cytoplasmic dynein 2 heavy chain 1	7
Acrdi.XM_015901928.1/1-1044	Monca.augustus.g7650.t1/1-1044	0.0514346	0.0295559	U5 small nuclear ribonucleo 40 kDa	7
Acrdi.XM_015915426.1/1-375	Monca.augustus.g32702.t1/1-375	0.0178155	0.0294934	NADH dehydrogenase [ubiquinone] 1 alpha subcomplex subunit 6	2
Acrdi.XM_015923106.1/1-588	Monca.augustus.g49071.t1/1-585	0.0456777	0.0294929	mitochondrial import inner membrane translocase subunit tim23-like	4
Acrdi.XM_015915417.1/1-1062	Monca.augustus.g32692.t1/1-1062	0.0359244	0.0294703	COUP transcription factor	11
Acrdi.XM_015901048.1/1-948	Monca.augustus.g57619.t1/1-951	0.0489028	0.0294424	cAMP-responsive element modulator isoform X1	5
Acrdi.XM_015923012.1/1-498	Monca.adi2mcaRNA3484_R0/1-522	0.19795	0.0294229	G2 M phase-specific E3 ubiquitin- ligase-like	
Acrdi.XM_015914885.1/1-1434	Monca.augustus.g36843.t1/1-1860	0.443955	0.0293464	THAP domain-containing 2	
Acrdi.XM_015914807.1/1-597	Monca.augustus.g66648.t1/1-588	0.0490103	0.0292153	Ubiquitin-conjugating enzyme E2-22 kDa	7
Acrdi.XM_015901000.1/1-1173	Monca.augustus.g56181.t1/1-1248	0.162809	0.0291821	PREDICTED: uncharacterized protein LOC107335949	
Acrdi.XM_015902768.1/1-711	Monca.adi2mcaRNA12124_R0/1-612	0.122839	0.0291492	nuclear polyadenylated RNA-binding 3-like	
Acrdi.XM_015903663.1/1-1215	Monca.augustus.g17910.t1/1-981	0.025813	0.0291268	peptidyl-prolyl cis-trans isomerase-like 4	5
Acrdi.XM_015908164.1/1-714	Monca.augustus.g36917.t1/1-1269	0.0416164	0.0291245	COP9 signalosome complex subunit 3	5
Acrdi.XM_015914974.1/1-384	Monca.augustus.g42423.t1/1-402	0.0334355	0.0291144	transcription factor 15-like	1
Acrdi.XM_015891802.1/1-879	Monca.augustus.g39213.t1/1-885	0.0290081	0.0290921	Lysine-specific histone demethylase 1B	6
Acrdi.XM_015894925.1/1-2979	Monca.adi2mcaRNA41284_R0/1-2931	0.471219	0.0290788	Transposon Tf2-6 poly	1
Acrdi.XM_015893171.1/1-315	Monca.augustus.g61989.t1/1-426	0.0326401	0.0290573	40S ribosomal S17	2
Acrdi.XM_015891680.1/1-1740	Monca.adi2mcaRNA37536_R0/1-1701	0.897627	0.0289809	PREDICTED: uncharacterized protein LOC107326928	9
Acrdi.XM_015904011.1/1-504	Monca.augustus.g32301.t1/1-504	0.0543485	0.0289521	ubiquitin-fold modifier-conjugating enzyme 1	5
Acrdi.XM_015904289.1/1-750	Monca.adi2mcaRNA13788_R1/1-891	0.553048	0.0289518	PREDICTED: uncharacterized protein LOC107339047	3
Acrdi.XM_015906344.1/1-1047	Monca.augustus.g45325.t1/1-1047	0.0433429	0.0289411	pre-mRNA-splicing factor 18 isoform X2	6
Acrdi.XM_015901624.1/1-705	Monca.augustus.g30905.t1/1-915	0.25422	0.02892	nuclease HARBI1	
Acrdi.XM_015900075.1/1-2013	Monca.adi2mcaRNA9235_R1/1-1953	0.434476	0.0289116	PREDICTED: uncharacterized protein LOC107335107	
Acrdi.XM_015919030.1/1-564	Monca.augustus.g32030.t1/1-831	0.052994	0.0287511	GPN-loop GTPase 3	
Acrdi.XM_015897056.1/1-579	Monca.augustus.g48648.t1/1-600	0.0345578	0.0287426	lin-7 homolog C	7
Acrdi.XM_015918542.1/1-933	Monca.adi2mcaRNA29589_R3/1-534	1.26108	0.02872	PREDICTED: uncharacterized protein LOC107352206	
Acrdi.XM_015918084.1/1-837	Monca.augustus.g34653.t1/1-1038	0.046729	0.0286928	probable tRNA N6-adenosine threonylcarbamoyltransferase	7
Acrdi.XM_015917968.1/1-795	Monca.adi2mcaRNA28977_R1/1-789	0.97242	0.0286856	nuclease HARBI1	
Acrdi.XM_015919008.1/1-1350	Monca.adi2mcaRNA30141_R1/1-1380	0.38856	0.0286815	nuclease HARBI1	1
Acrdi.XM_015894472.1/1-1014	Monca.adi2mcaRNA40747_R5/1-1032	0.688969	0.0286324	PREDICTED: uncharacterized protein LOC107329799	
Acrdi.XM_015900999.1/1-936	Monca.augustus.g52573.t1/1-348	0.194197	0.0285855	endoplasmic reticulum oxidoreductin-1-like	
Acrdi.XM_015899150.1/1-2604	Monca.augustus.g63379.t1/1-2610	0.0338561	0.0285769	phosphatidylinositol 3-kinase catalytic subunit type 3 isoform X2	17
Acrdi.XM_015922361.1/1-507	Monca.augustus.g66199.t1/1-1125	0.0415049	0.0285692	zinc transporter 7-like	3
Acrdi.XM_015910384.1/1-897	Monca.augustus.g70897.t1/1-1233	0.0325932	0.0285425	neuronal-specific septin-3-like isoform X2	1
Acrdi.XM_015918668.1/1-474	Monca.augustus.g7585.t1/1-606	0.0471299	0.0285346	dickkopf-related 3-like	3
Acrdi.XM_015903254.1/1-1554	Monca.adi2mcaRNA12628_R0/1-1548	0.0410049	0.028534	Pre-mRNA-processing factor 19	25
Acrdi.XM_015917040.1/1-546	Monca.augustus.g37435.t1/1-546	0.253339	0.0285276	RNA-binding 25-like isoform X1	
Acrdi.XM_015892856.1/1-768	Monca.augustus.g43163.t1/1-768	0.0375985	0.0285266	unc-50 homolog	2
Acrdi.XM_015913127.1/1-1134	Monca.augustus.g25045.t1/1-1134	0.0513956	0.0285007	rho GTPase-activating 100F-like	1
Acrdi.XM_015902733.1/1-939	Monca.augustus.g27724.t1/1-978	0.346634	0.0284927	retinol dehydrogenase 7-like	4
Acrdi.XM_015897601.1/1-1578	Monca.adi2mcaRNA6526_R1/1-1581	0.64778	0.02848	PREDICTED: uncharacterized protein LOC107332848	
Acrdi.XM_015923616.1/1-1419	Monca.adi2mcaRNA35285_R2/1-1620	0.54291	0.0284591	RNA-directed DNA polymerase from transposon X-element	
Acrdi.XM_015920708.1/1-1401	Monca.adi2mcaRNA32014_R0/1-1440	0.200231	0.0283628	PREDICTED: uncharacterized protein LOC107354259	1
Acrdi.XM_015908898.1/1-369	Monca.augustus.g29212.t1/1-369	0.0299083	0.0283597	multifunctional methyltransferase subunit TRM112	3
Acrdi.XM_015910878.1/1-834	Monca.adi2mcaRNA2223_R0/1-807	0.0333239	0.0283267	26S protease regulatory subunit 6A-B	10
Acrdi.XM_015914890.1/1-1755	Monca.adi2mcaRNA2756_R6/1-2997	0.418076	0.0282834	RNA-directed DNA polymerase from mobile element jockey-like	
Acrdi.XM_015902803.1/1-1824	Monca.augustus.g27233.t1/1-1470	0.027885	0.0282754	sodium hydrogen exchanger 10-like	5
Acrdi.XM_015900125.1/1-1308	Monca.augustus.g65813.t1/1-1308	0.0363201	0.0282709	BTB POZ domain-containing 2-like isoform X1	
Acrdi.XM_015903183.1/1-1374	Monca.adi2mcaRNA12557_R0/1-1374	0.0485715	0.0282105	Golgi pH regulator	6
Acrdi.XM_015919148.1/1-900	Monca.augustus.g35150.t1/1-900	0.0580459	0.0281965	ATP synthase subunit mitochondrial isoform X1	7
Acrdi.XM_015905984.1/1-1782	Monca.adi2mcaRNA15695_R0/1-1776	0.0422549	0.0281511	vacuolar sorting-associated 35	6
Acrdi.XM_015904209.1/1-402	Monca.augustus.g16308.t1/1-402	0.014376	0.0281396	FAM183A-like isoform X2	
Acrdi.XM_015896998.1/1-501	Monca.augustus.g28906.t1/1-501	0.0380642	0.0280813	sorting nexin-12	5
Acrdi.XM_015921602.1/1-3642	Monca.augustus.g67240.t1/1-3684	0.0372303	0.028005	splicing factor 3B subunit 3	5
Acrdi.XM_015897188.1/1-1302	Monca.augustus.g37652.t1/1-3903	0.318119	0.0279885	nuclease HARBI1	
Acrdi.XM_015920546.1/1-3237	Monca.augustus.g38793.t1/1-3240	0.0366775	0.0279605	Ubiquitin carboxyl-terminal hydrolase 7	3
Acrdi.XM_015891693.1/1-1338	Monca.augustus.g65233.t1/1-1431	0.466629	0.0278924	ATP-dependent DNA helicase -like	2
Acrdi.XM_015902151.1/1-780	Monca.augustus.g17201.t1/1-774	0.0375849	0.027844	Splicing factor 3A subunit 2	6
Acrdi.XM_015917647.1/1-918	Monca.augustus.g23027.t1/1-1080	0.39122	0.0278179	TNF receptor-associated factor 5-like	5
Acrdi.XM_015901269.1/1-1470	Monca.adi2mcaRNA10579_R0/1-1134	0.0394671	0.0277864	transcriptional enhancer factor TEF-1 isoform X1	5
Acrdi.XM_015893995.1/1-864	Monca.adi2mcaRNA40150_R2/1-750	0.533414	0.0277507	piggyBac transposable element-derived 4-like	
Acrdi.XM_015906505.1/1-498	Monca.augustus.g22542.t1/1-495	0.0287257	0.0277385	Calmodulin	36
Acrdi.XM_015916293.1/1-1041	Monca.augustus.g68767.t1/1-1833	0.0379265	0.0277252	Forkhead box K1	1
Acrdi.XM_015919135.1/1-825	Monca.augustus.g35169.t1/1-1008	0.0331842	0.027671	guanine nucleotide-binding subunit beta A	5
Acrdi.XM_015908799.1/1-1878	Monca.adi2mcaRNA37473_R9/1-1146	0.585196	0.0274871	Transposon Tf2-6 poly	
Acrdi.XM_015901063.1/1-975	Monca.adi2mcaRNA1021_R0/1-882	0.0591761	0.0274555	Trifunctional enzyme subunit mitochondrial	2
Acrdi.XM_015925206.1/1-951	Monca.adi2mcaRNA37121_R4/1-891	0.158875	0.0274539	PREDICTED: uncharacterized protein LOC107358608	
Acrdi.XM_015924205.1/1-546	Monca.augustus.g39157.t1/1-546	0.0231771	0.0273865	secretory immunoglobulin A-binding -like	1
Acrdi.XM_015892928.1/1-924	Monca.adi2mcaRNA38950_R1/1-1026	0.373053	0.0272579	nuclease HARBI1	
Acrdi.XM_015916746.1/1-693	Monca.augustus.g62783.t1/1-678	0.0417222	0.0272552	juxtaposed with another zinc finger 1	1
Acrdi.XM_015898765.1/1-420	Monca.augustus.g55629.t1/1-423	0.0281484	0.0272458	DNA-directed RNA polymerase II subunit RPB4	12
Acrdi.XM_015898268.1/1-951	Monca.augustus.g39434.t1/1-954	0.0312992	0.0272414	COP9 signalosome complex subunit 6	1
Acrdi.XM_015899901.1/1-456	Monca.augustus.g43425.t1/1-459	0.0429981	0.0272259	LIM domain transcription factor -like isoform X1	1
Acrdi.XM_015894384.1/1-1179	Monca.adi2mcaRNA40649_R2/1-1179	0.921874	0.0271847	Pro-Pol poly	
Acrdi.XM_015908416.1/1-2406	Monca.adi2mcaRNA18412_R1/1-1878	0.378577	0.0270926	PREDICTED: uncharacterized protein LOC107342907	
Acrdi.XM_015918636.1/1-2076	Monca.augustus.g33676.t1/1-2937	0.0353094	0.0270854	kinesin KIF15	1
Acrdi.XM_015918100.1/1-2061	Monca.adi2mcaRNA29114_R1/1-2037	0.307745	0.0270765	RNA-directed DNA polymerase from transposon X-element	
Acrdi.XM_015911156.1/1-897	Monca.augustus.g24018.t1/1-849	0.0372259	0.0270362	E3 ubiquitin- ligase SIAH1	9
Acrdi.XM_015900231.1/1-459	Monca.augustus.g14548.t1/1-459	0.0241031	0.0269958	calmodulin-like isoform X1	1
Acrdi.XM_015916696.1/1-1857	Monca.adi2mcaRNA27522_R5/1-2127	1.33802	0.0269816	zinc finger 862-like	2
Acrdi.XM_015901186.1/1-660	Monca.augustus.g58599.t1/1-660	0.0265901	0.0269053	spliceosome-associated CWC15 homolog	5
Acrdi.XM_015919205.1/1-321	Monca.augustus.g54935.t1/1-648	0.0284724	0.0267804	ras-related Rab-21-like	5
Acrdi.XM_015922143.1/1-966	Monca.augustus.g26479.t1/1-1098	0.0496385	0.0267667	NIPA 2	3
Acrdi.XM_015914244.1/1-411	Monca.augustus.g24194.t1/1-411	0.0354352	0.0267538	NTF2-related export 1	2
Acrdi.XM_015894880.1/1-1158	Monca.augustus.g53680.t1/1-2514	0.457582	0.0267465	RNA-directed DNA polymerase from transposon X-element	1
Acrdi.XM_015919134.1/1-1215	Monca.augustus.g35166.t1/1-1233	0.0341826	0.0266922	E3 ubiquitin- ligase Hakai	3
Acrdi.XM_015923101.1/1-1410	Monca.adi2mcaRNA34714_R2/1-1410	0.43605	0.0266484	RNA-directed DNA polymerase from mobile element jockey-like	
Acrdi.XM_015906121.1/1-2133	Monca.adi2mcaRNA15837_R2/1-1878	0.360969	0.0265912	zinc finger 862-like	
Acrdi.XM_015921083.1/1-2412	Monca.augustus.g27612.t1/1-2079	0.0365124	0.0265756	Glycogen phosphorylase	4
Acrdi.XM_015915322.1/1-675	Monca.augustus.g25286.t1/1-1401	0.0334555	0.0265705	Dihydrolipoyllysine-residue succinyltransferase component of 2-oxoglu	12
Acrdi.XM_015898501.1/1-1677	Monca.augustus.g3317.t1/1-1701	0.0293069	0.0265124	DET1 homolog	
Acrdi.XM_015907506.1/1-651	Monca.augustus.g55653.t1/1-651	0.0276669	0.0265101	charged multivesicular body 3 isoform X2	
Acrdi.XM_015922845.1/1-558	Monca.augustus.g1405.t1/1-555	0.0434525	0.0264969	PHD finger 13-like	2
Acrdi.XM_015913020.1/1-1083	Monca.augustus.g46539.t1/1-1083	0.231075	0.0264739	PREDICTED: uncharacterized protein LOC107347148	
Acrdi.XM_015911385.1/1-462	Monca.adi2mcaRNA21649_R0/1-462	0.0385651	0.0264501	Proteasome subunit alpha type-7	5
Acrdi.XM_015907104.1/1-540	Monca.augustus.g36984.t1/1-540	0.0924048	0.0264455	achaete-scute homolog 1a-like	6
Acrdi.XM_015910404.1/1-1002	Monca.augustus.g51117.t1/1-3306	0.0308655	0.0264412	kinesin KIF19	9
Acrdi.XM_015897185.1/1-1215	Monca.augustus.g2763.t1/1-1194	0.476921	0.0264121	PREDICTED: uncharacterized protein LOC107332450	
Acrdi.XM_015891695.1/1-837	Monca.augustus.g43733.t1/1-837	0.0381221	0.0263993	proteasome subunit beta type-5-like	5
Acrdi.XM_015917891.1/1-636	Monca.augustus.g39681.t1/1-996	0.0287747	0.0263823	Transcription factor E2F4	6
Acrdi.XM_015901212.1/1-489	Monca.augustus.g58571.t1/1-471	0.0331727	0.0263287	AP-1 complex subunit sigma-2-like	4
Acrdi.XM_015913992.1/1-1446	Monca.augustus.g1521.t1/1-1821	0.874581	0.0263151	RNA-directed DNA polymerase from mobile element jockey	
Acrdi.XM_015916315.1/1-660	Monca.augustus.g64069.t1/1-852	0.0287181	0.0262505	Methionine aminopeptidase 2	5
Acrdi.XM_015913132.1/1-360	Monca.augustus.g9265.t1/1-300	0.0540722	0.02624	U6 snRNA-associated Sm LSm7	10
Acrdi.XM_015917403.1/1-516	Monca.augustus.g23808.t1/1-468	0.0316195	0.0261694	39S ribosomal mitochondrial	7
Acrdi.XM_015902857.1/1-1080	Monca.augustus.g54822.t1/1-1494	0.0279685	0.0259778	fumarate mitochondrial	8
Acrdi.XM_015895642.1/1-1287	Monca.augustus.g35445.t1/1-1287	0.0259065	0.0259615	septin-6 isoform X3	1
Acrdi.XM_015908130.1/1-4500	Monca.adi2mcaRNA18093_R2/1-4458	0.221607	0.0259316	Pro-Pol poly	
Acrdi.XM_015914058.1/1-2268	Monca.augustus.g24060.t1/1-3081	0.0306085	0.0259179	SWI SNF-related matrix-associated actin-dependent regulator of chror	6
Acrdi.XM_015900259.1/1-2070	Monca.adi2mcaRNA9478_R5/1-1524	0.531358	0.0258698	Retrovirus-related Pol poly from transposon	2
Acrdi.XM_015905342.1/1-735	Monca.augustus.g70902.t1/1-699	0.697146	0.0258398	UPF0746 DDB_G0281095-like	
Acrdi.XM_015909930.1/1-1101	Monca.augustus.g28077.t1/1-1134	0.0386085	0.0257701	methionine aminopeptidase 1	5
Acrdi.XM_015895943.1/1-840	Monca.augustus.g36103.t1/1-840	0.0547571	0.0257521	surfeit locus 4 homolog	1
Acrdi.XM_015916782.1/1-1083	Monca.augustus.g62779.t1/1-1215	0.0340133	0.0257355	Glycerol-3-phosphate dehydrogenase [NAD(+)] cytoplasmic	7
Acrdi.XM_015914843.1/1-1239	Monca.adi2mcaRNA25493_R2/1-3345	0.564885	0.0257347	RNA-directed DNA polymerase from mobile element jockey-like	
Acrdi.XM_015903812.1/1-2265	Monca.adi2mcaRNA13250_R0/1-2262	0.0334934	0.0257039	E3 ubiquitin- ligase TRIM71	2
Acrdi.XM_015914433.1/1-1269	Monca.augustus.g71046.t1/1-1488	0.0451274	0.025614	V-type proton ATPase subunit brain isoform	5
Acrdi.XM_015918176.1/1-702	Monca.augustus.g66570.t1/1-702	0.0488709	0.0255383	proteasome subunit alpha type-2	24
Acrdi.XM_015916744.1/1-717	Monca.augustus.g62781.t1/1-840	0.0334303	0.0254783	Ubiquitin-conjugating enzyme E2 Z	7
Acrdi.XM_015920692.1/1-801	Monca.augustus.g4863.t1/1-819	0.0563548	0.0254721	Transcriptional activator Pur-alpha	
Acrdi.XM_015916702.1/1-12942	Monca.augustus.g42254.t1/1-13854	0.033019	0.025417	dynein heavy chain axonemal-like	5
Acrdi.XM_015915795.1/1-1095	Monca.adi2mcaRNA26527_R0/1-1068	0.282708	0.025381	PREDICTED: uncharacterized protein LOC107349618	
Acrdi.XM_015903887.1/1-837	Monca.adi2mcaRNA21145_R9/1-552	0.132909	0.0253369	TNF receptor-associated factor 5-like isoform X2	
Acrdi.XM_015904241.1/1-816	Monca.augustus.g8621.t1/1-3963	0.0289994	0.0252385	tyrosine- phosphatase non-receptor type 13 isoform X1	6
Acrdi.XM_015922121.1/1-1596	Monca.adi2mcaRNA3372_R0/1-1245	0.0315675	0.0251495	probable ATP-dependent RNA helicase DDX41	6
Acrdi.XM_015892278.1/1-1089	Monca.augustus.g1826.t1/1-1398	0.0217245	0.025115	Glutamine synthetase	4
Acrdi.XM_015904661.1/1-609	Monca.augustus.g7184.t1/1-1269	0.026267	0.025087	testis-expressed sequence 9	
Acrdi.XM_015923658.1/1-1557	Monca.adi2mcaRNA35333_R0/1-1602	0.264779	0.0249569	PREDICTED: uncharacterized protein LOC107357015 isoform X2	
Acrdi.XM_015906307.1/1-744	Monca.adi2mcaRNA16033_R0/1-627	0.025089	0.0249563	PREDICTED: uncharacterized protein C4orf22 homolog	
Acrdi.XM_015902230.1/1-987	Monca.augustus.g58166.t1/1-984	0.0374215	0.0249505	tribbles homolog 2	7
Acrdi.XM_015902093.1/1-2586	Monca.adi2mcaRNA11477_R1/1-2298	0.0287156	0.0249153	DNA-directed RNA polymerase III subunit RPC2	
Acrdi.XM_015912145.1/1-2496	Monca.adi2mcaRNA22449_R1/1-2340	0.225426	0.0249036	Retrovirus-related Pol poly from transposon	5
Acrdi.XM_015897940.1/1-597	Monca.augustus.g39405.t1/1-597	0.0375058	0.0248999	coiled-coil domain-containing 153-like	
Acrdi.XM_015922243.1/1-486	Monca.augustus.g59147.t1/1-486	0.0280468	0.0248767	calmodulin 3	1
Acrdi.XM_015899417.1/1-1299	Monca.adi2mcaRNA8631_R7/1-1299	0.555984	0.0248739	PREDICTED: uncharacterized protein LOC107334477	
Acrdi.XM_015923062.1/1-876	Monca.augustus.g45662.t1/1-1347	0.617617	0.0248526	RNA-directed DNA polymerase from mobile element jockey-like	5

Acrdi.XM_015907533.1/1-2034 | Monca.augustus.g45770.t1/1-2643	0.0303771	0.0248169	Cilia- and flagella-associated 58			
Acrdi.XM_015901183.1/1-774 | Monca.augustus.g58597.t1/1-771	0.0465502	0.024815	DCN1 1	6	F:GO:0032182; F:GO:0031624; P:GO:0051443; C:GO:0000151;	[illegible]
Acrdi.XM_015915099.1/1-1617 | Monca.adi2mcaRNA25756_R0/1-1617	0.305619	0.024794	PREDICTED: uncharacterized protein LOC107349000	3	F:GO:0003676; C:GO:0016020; C:GO:0016021	F:nucleic acid binding; C:membrane; C:integral component of membrane
Acrdi.XM_015921029.1/1-846 | Monca.adi2mcaRNA32348_R0/1-849	0.0253266	0.0247747	CTD nuclear envelope phosphatase 1	8	C:GO:0005789; F:GO:0004722; C:GO:0031965; P:GO:0006998;	[illegible]
Acrdi.XM_015922491.1/1-933 | Monca.augustus.g60688.t1/1-933	0.0355819	0.0247654	coiled-coil domain-containing 42 homolog	1	C:GO:0016020	C:membrane
Acrdi.XM_015924957.1/1-1125 | Monca.augustus.g4522.t1/1-1125	0.033485	0.0247367	coiled-coil domain-containing 113-like			
Acrdi.XM_015922909.1/1-882 | Monca.adi2mcaRNA34469_R3/1-972	0.524524	0.0246924	RNA-directed DNA polymerase from transposon BS			
Acrdi.XM_015914190.1/1-1953 | Monca.augustus.g50915.t1/1-2334	0.0352103	0.0244998	C-Maf-inducing -like			
Acrdi.XM_015917440.1/1-2028 | Monca.augustus.g69702.t1/1-2400	0.0286048	0.0244888	probable ATP-dependent RNA helicase DDX23	9	F:GO:0003676; P:GO:0000375; F:GO:0005524; C:GO:0070062;	[illegible]
Acrdi.XM_015891814.1/1-588 | Monca.augustus.g43711.t1/1-606	0.0329763	0.0244732	charged multivesicular body 1a-like	5	P:GO:0016192; P:GO:0007076; C:GO:0000815; C:GO:0016363;	P:vesicle-mediated transport; P:mitotic chromosome condensation; C:ESCRT III complex; C:nuclear matrix; P:vacuolar transport
Acrdi.XM_015892786.1/1-1788 | Monca.adi2mcaRNA38803_R0/1-1698	0.345645	0.0244561	THAP domain-containing 10			
Acrdi.XM_015909134.1/1-981 | Monca.augustus.g26059.t1/1-981	0.764218	0.0244436	PREDICTED: uncharacterized protein LOC107343551 isoform X1			
Acrdi.XM_015904694.1/1-1131 | Monca.augustus.g12719.t1/1-1131	0.0363052	0.0244429	Cyclin-dependent kinase 9	8	C:GO:0005634; F:GO:0005524; F:GO:0008353; P:GO:0006351;	[illegible]
Acrdi.XM_015907299.1/1-1131 | Monca.augustus.g31778.t1/1-1500	0.0328179	0.0244015	signal recognition particle 54 kDa	5	F:GO:0005525; P:GO:0006614; C:GO:0048500; F:GO:0003924;	F:GTP binding; P:SRP-dependent cotranslational protein targeting to membrane; C:signal recognition particle; F:GTPase activity; F:7S RNA binding
Acrdi.XM_015908129.1/1-1617 | Monca.adi2mcaRNA18092_R3/1-1515	1.40037	0.0244005	zinc finger 862-like	2	F:GO:0004842; P:GO:0016567	F:ubiquitin-protein transferase activity; P:protein ubiquitination
Acrdi.XM_015907536.1/1-1503 | Monca.adi2mcaRNA1829_R1/1-1479	0.0267521	0.0243993	78 kDa glucose-regulated	10	F:GO:0016887; P:GO:0007281; C:GO:0005788; F:GO:0005524;	[illegible]
Acrdi.XM_015920855.1/1-1692 | Monca.augustus.g30750.t1/1-1689	0.033434	0.0243525	acid-sensing ion channel 2 isoform X1	9	P:GO:0065008; F:GO:0022836; F:GO:0005272; C:GO:0016021;	[illegible]
Acrdi.XM_015901765.1/1-534 | Monca.augustus.g12065.t1/1-948	0.038792	0.0243272	RNA-binding with multiple splicing-like	2	F:GO:0000166; F:GO:0003676	F:nucleotide binding; F:nucleic acid binding
Acrdi.XM_015910227.1/1-1077 | Monca.augustus.g25443.t1/1-1749	0.0319207	0.0242423	Transmembrane 9 superfamily member 3	1	C:GO:0016021	C:integral component of membrane
Acrdi.XM_015894309.1/1-714 | Monca.adi2mcaRNA40554_R1/1-1104	0.457546	0.0242353	Integrase recombinase xerD-like			
Acrdi.XM_015916139.1/1-1047 | Monca.augustus.g24446.t1/1-1047	0.0328172	0.024139	Cyclin-dependent kinase 20	10	P:GO:0051301; C:GO:0005634; F:GO:0005524; P:GO:0060271;	[illegible]
Acrdi.XM_015907458.1/1-318 | Monca.augustus.g43827.t1/1-318	0.0303429	0.0241022	Membrane magnesium transporter 1	1	C:GO:0016021	C:integral component of membrane
Acrdi.XM_015894678.1/1-663 | Monca.augustus.g49920.t1/1-546	0.0381504	0.0241009	transmembrane and coiled-coil domain-containing 1	1	C:GO:0016021	C:integral component of membrane
Acrdi.XM_015911890.1/1-1632 | Monca.augustus.g7925.t1/1-1641	0.0420787	0.0240959	von Willebrand factor type EGF and pentraxin domain-containing	4	P:GO:0007165; C:GO:0005739; P:GO:0051607; F:GO:0004871	P:signal transduction; C:mitochondrion; P:defense response to virus; F:signal transducer activity
Acrdi.XM_015914924.1/1-2553 | Monca.adi2mcaRNA25564_R0/1-2553	0.620895	0.0240636	PREDICTED: uncharacterized protein LOC107348864	2	F:GO:0003676; F:GO:0046872	F:nucleic acid binding; F:metal ion binding
Acrdi.XM_015913716.1/1-1275 | Monca.augustus.g21635.t1/1-1275	0.0483904	0.0240627	flotillin-2-like isoform X2			
Acrdi.XM_015914965.1/1-1182 | Monca.adi2mcaRNA25615_R4/1-1467	0.207963	0.0238968	PREDICTED: uncharacterized protein LOC107348881	1	F:GO:0003676	F:nucleic acid binding
Acrdi.XM_015921254.1/1-1446 | Monca.augustus.g42829.t1/1-1467	0.0296717	0.0238685	nuclear respiratory factor 1 isoform X1	2	F:GO:0000976; C:GO:0043227	F:transcription regulatory region sequence-specific DNA binding; C:membrane-bounded organelle
Acrdi.XM_015907360.1/1-354 | Monca.augustus.g63473.t1/1-354	0.0225047	0.0238238	lin-52 homolog isoform X2	3	P:GO:0006351; C:GO:0070176; P:GO:0007049	P:transcription, DNA-templated; C:DRM complex; P:cell cycle
Acrdi.XM_015905113.1/1-639 | Monca.adi2mcaRNA14707_R3/1-639	0.263697	0.0237948	PREDICTED: uncharacterized protein LOC107339800			
Acrdi.XM_015916690.1/1-1065 | Monca.adi2mcaRNA27525_R0/1-1026	0.480777	0.0236414	piggyBac transposable element-derived 4-like			
Acrdi.XM_015896744.1/1-597 | Monca.augustus.g42410.t1/1-594	0.044673	0.0235779	ras-related Ral-A	5	F:GO:0005525; F:GO:0003924; P:GO:0007265; C:GO:0005622;	F:GTP binding; F:GTPase activity; P:Ras protein signal transduction; C:intracellular; C:plasma membrane
Acrdi.XM_015898723.1/1-813 | Monca.augustus.g2536.t1/1-813	0.036137	0.023526	F-actin-capping subunit beta	9	F:GO:0051015; P:GO:0010591; P:GO:0051016; C:GO:0005737;	[illegible]
Acrdi.XM_015892526.1/1-897 | Monca.adi2mcaRNA38478_R0/1-573	0.253548	0.0234533	PREDICTED: uncharacterized protein LOC107327782	1	F:GO:0003676	F:nucleic acid binding
Acrdi.XM_015898474.1/1-3825 | Monca.adi2mcaRNA7579_R9/1-3831	0.15554	0.0233926	Transposon Ty3-G Gag-Pol poly			
Acrdi.XM_015916581.1/1-1215 | Monca.augustus.g22861.t1/1-990	1.268	0.0233107	nuclease HARBI1			
Acrdi.XM_015897967.1/1-1533 | Monca.augustus.g57924.t1/1-1488	0.366156	0.023204	PREDICTED: uncharacterized protein LOC107333199	2	C:GO:0016020; C:GO:0016021	C:membrane; C:integral component of membrane
Acrdi.XM_015906767.1/1-1179 | Monca.adi2mcaRNA16568_R6/1-1116	0.477651	0.0231951	RNA-directed DNA polymerase from mobile element jockey			
Acrdi.XM_015909189.1/1-477 | Monca.augustus.g21530.t1/1-819	0.389089	0.0231922	zinc finger MYM-type 2-like	3	F:GO:0003676; P:GO:0006355; C:GO:0005622	[illegible]
Acrdi.XM_015893964.1/1-546 | Monca.adi2mcaRNA40110_R3/1-540	0.3832	0.0231365	Transposable element P transposase			
Acrdi.XM_015917523.1/1-543 | Monca.augustus.g56365.t1/1-543	0.0501024	0.0230832	PREDICTED: uncharacterized protein LOC107351225	1	C:GO:0016020	C:membrane
Acrdi.XM_015904686.1/1-2022 | Monca.augustus.g64798.t1/1-2022	0.341425	0.0230325	G2 M phase-specific E3 ubiquitin- ligase-like	1	F:GO:0004842	F:ubiquitin-protein transferase activity
Acrdi.XM_015907896.1/1-300 | Monca.augustus.g46272.t1/1-300	0.0614988	0.023024	UBA-like domain-containing 2			
Acrdi.XM_015923868.1/1-540 | Monca.augustus.g50390.t1/1-552	0.0242647	0.0229594	Pancreas transcription factor 1 subunit alpha	14	P:GO:0009790; F:GO:0043565; F:GO:0046983; P:GO:0048699;	[illegible]
Acrdi.XM_015909838.1/1-969 | Monca.augustus.g15380.t1/1-834	0.226991	0.022941	PREDICTED: uncharacterized protein LOC107344194			
Acrdi.XM_015906936.1/1-1095 | Monca.augustus.g11632.t1/1-1098	0.0342617	0.0229128	GTP-binding 128up	1	F:GO:0005525	F:GTP binding
Acrdi.XM_015910074.1/1-3996 | Monca.adi2mcaRNA24919_R3/1-3045	0.639708	0.0229	Retrovirus-related Pol poly from transposon			
Acrdi.XM_015922985.1/1-516 | Monca.augustus.g67845.t1/1-516	0.00980526	0.0228775	myosin regulatory light polypeptide 9	1	F:GO:0005509	F:calcium ion binding
Acrdi.XM_015911502.1/1-681 | Monca.augustus.g10124.t1/1-681	0.0362357	0.022837	MOB kinase activator-like 3	2	P:GO:0016310; F:GO:0016301	P:phosphorylation; F:kinase activity
Acrdi.XM_015903294.1/1-2043 | Monca.augustus.g65500.t1/1-2046	0.0341098	0.0227988	Beta-adrenergic receptor kinase 2	23	F:GO:0019912; P:GO:0043009; F:GO:0034211; F:GO:0004690;	[illegible]
Acrdi.XM_015903091.1/1-702 | Monca.augustus.g45209.t1/1-702	0.0286055	0.0227981	Ubiquitin domain-containing 2	1	F:GO:0043130	F:ubiquitin binding
Acrdi.XM_015903867.1/1-423 | Monca.augustus.g32296.t1/1-423	0.0223046	0.0227772	trafficking particle complex subunit 1	5	C:GO:0030008; P:GO:0006888; C:GO:0005737; F:GO:0017112;	[illegible]
Acrdi.XM_015918640.1/1-735 | Monca.augustus.g33668.t1/1-735	0.0454664	0.0227369	eukaryotic translation initiation factor 6	9	F:GO:0003743; P:GO:0007229; C:GO:0005737; F:GO:0043022;	[illegible]
Acrdi.XM_015903483.1/1-1782 | Monca.adi2mcaRNA12883_R0/1-1740	0.65387	0.0226715	ATP-dependent DNA helicase -like	2	P:GO:0006259; F:GO:0016787	P:DNA metabolic process; F:hydrolase activity
Acrdi.XM_015899734.1/1-807 | Monca.augustus.g66172.t1/1-807	0.0313504	0.0226537	26S proteasome non-ATPase regulatory subunit 8	5	C:GO:0005634; P:GO:0043161; C:GO:0005829; C:GO:0008541;	[illegible]
Acrdi.XM_015909172.1/1-1122 | Monca.augustus.g57519.t1/1-876	0.0624327	0.0226183	forkhead box L2	5	C:GO:0005634; F:GO:0003700; P:GO:0006355; P:GO:0006366;	[illegible]
Acrdi.XM_015893102.1/1-855 | Monca.augustus.g63396.t1/1-1782	0.350618	0.022576	52 kDa repressor of the inhibitor of the kinase-like	7	F:GO:0003676; F:GO:0003677; C:GO:0005737; F:GO:0046983;	[illegible]
Acrdi.XM_015923442.1/1-2895 | Monca.adi2mcaRNA35109_R5/1-2715	0.464415	0.0225216	OTU domain-containing 6B isoform X1			
Acrdi.XM_015894170.1/1-1629 | Monca.adi2mcaRNA40399_R5/1-2121	0.96874	0.0224694	Transposon Tf2-6 poly			
Acrdi.XM_015915524.1/1-981 | Monca.augustus.g12950.t1/1-981	1.08129	0.0224208	ATP-dependent DNA helicase			
Acrdi.XM_015905458.1/1-474 | Monca.augustus.g28268.t1/1-726	0.0396112	0.0224068	Proteasome subunit alpha type-5	6	C:GO:0070062; C:GO:0005737; C:GO:0019773; F:GO:0004298;	[illegible]
Acrdi.XM_015910889.1/1-633 | Monca.augustus.g6317.t1/1-633	0.0344064	0.0223982	GTP-binding Rit2	4	F:GO:0005525; P:GO:0007264; C:GO:0016020; C:GO:0005622	[illegible]
Acrdi.XM_015910475.1/1-1758 | Monca.augustus.g41057.t1/1-1785	0.0208499	0.0223768	kelch 20 isoform X2	12	F:GO:0004842; C:GO:0005802; P:GO:0043161; C:GO:0016605;	[illegible]
Acrdi.XM_015910967.1/1-651 | Monca.augustus.g3079.t1/1-651	0.0367037	0.0223483	Trafficking particle complex subunit 4	9	C:GO:0030008; C:GO:0030425; P:GO:0006888; C:GO:0008021;	[illegible]
Acrdi.XM_015922092.1/1-3651 | Monca.augustus.g48047.t1/1-2898	0.53018	0.0223251	Transposon Ty3-G Gag-Pol poly			
Acrdi.XM_015904594.1/1-537 | Monca.adi2mcaRNA14104_R2/1-489	0.200405	0.0223095	PREDICTED: uncharacterized protein LOC107339321	9	C:GO:0005634; F:GO:0030170; F:GO:0046872; F:GO:0003824;	[illegible]
Acrdi.XM_015923838.1/1-1389 | Monca.adi2mcaRNA35545_R0/1-1389	0.0374676	0.022257	POC1 centriolar homolog A-like isoform X1			
Acrdi.XM_015922710.1/1-1818 | Monca.augustus.g6736.t1/1-1818	0.0248254	0.0222189	kelch 18	3	F:GO:0004842; P:GO:0046529; C:GO:0031463	[illegible]
Acrdi.XM_015908840.1/1-1038 | Monca.augustus.g45265.t1/1-1773	0.0288916	0.0221605	transmembrane 9 superfamily member 1 isoform X2	3	C:GO:0016021; P:GO:0007034; C:GO:0005622	C:integral component of membrane; P:vacuolar transport; C:intracellular
Acrdi.XM_015914237.1/1-2952 | Monca.augustus.g57432.t1/1-2952	0.0285193	0.0220474	UDP-N-acetylglucosamine--peptide N-acetylglucosaminyltransferase 110 kDa subunit isoform X2			
Acrdi.XM_015901721.1/1-2331 | Monca.adi2mcaRNA11090_R0/1-2289	0.0374802	0.0220239	Coatomer subunit beta	9	P:GO:0009790; P:GO:0006888; F:GO:0005515; P:GO:0006890;	[illegible]
Acrdi.XM_015913606.1/1-1119 | Monca.adi2mcaRNA24078_R0/1-1128	0.993657	0.022003	PREDICTED: uncharacterized protein LOC107347647			
Acrdi.XM_015910476.1/1-381 | Monca.augustus.g41056.t1/1-459	0.0300551	0.0219826	40S ribosomal S18	6	F:GO:0003735; C:GO:0005829; P:GO:0042254; C:GO:0015935;	[illegible]
Acrdi.XM_015905537.1/1-2400 | Monca.augustus.g28245.t1/1-2541	0.0318941	0.0219463	IQ and AAA domain-containing 1	1	F:GO:0005524	F:ATP binding
Acrdi.XM_015893417.1/1-429 | Monca.augustus.g34515.t1/1-855	0.0309188	0.0219325	nap homolog 1	3	C:GO:0005739; C:GO:0016020; P:GO:0019233	C:mitochondrion; C:membrane; P:sensory perception of pain
Acrdi.XM_015920712.1/1-3357 | Monca.augustus.g52406.t1/1-3141	0.891736	0.0217768	PREDICTED: uncharacterized protein LOC107354263 isoform X1	4	C:GO:0005634; F:GO:0046872; F:GO:0003682; P:GO:0006357	[illegible]
Acrdi.XM_015909943.1/1-6369 | Monca.adi2mcaRNA20056_R1/1-6162	0.41126	0.0217327	OTU domain-containing 6B isoform X1			
Acrdi.XM_015902524.1/1-3510 | Monca.adi2mcaRNA11876_R0/1-3510	0.593303	0.0215955	tetratricopeptide repeat 28-like			
Acrdi.XM_015923655.1/1-1722 | Monca.adi2mcaRNA35337_R3/1-1710	0.164274	0.0215879	SPRY domain-containing SOCS box 4			
Acrdi.XM_015904001.1/1-399 | Monca.augustus.g69790.t1/1-1368	0.0411838	0.0214402	short branched chain specific acyl- mitochondrial	8	F:GO:0003995; P:GO:0033539; F:GO:0000062; F:GO:0050660;	[illegible]
Acrdi.XM_015903134.1/1-975 | Monca.augustus.g12309.t1/1-975	0.0271979	0.0213411	mitotic checkpoint BUB3 isoform X2	16	P:GO:0007094; P:GO:0007062; C:GO:0000777; P:GO:0042787;	[illegible]
Acrdi.XM_015916215.1/1-5514 | Monca.augustus.g40341.t1/1-6411	0.0227664	0.0213193	U5 small nuclear ribonucleo 200 kDa helicase	3	F:GO:0003676; F:GO:0005524; F:GO:0004386	F:nucleic acid binding; F:ATP binding; F:helicase activity
Acrdi.XM_015914121.1/1-852 | Monca.augustus.g8020.t1/1-849	0.0555805	0.021302	Krueppel-like factor 7	1	F:GO:0005488	F:binding
Acrdi.XM_015924820.1/1-1059 | Monca.augustus.g67353.t1/1-1059	0.0460891	0.0212427	guanine nucleotide-binding G(q) subunit alpha	44	F:GO:0005096; P:GO:0009791; P:GO:0007213; P:GO:0043267;	[illegible]
Acrdi.XM_015895759.1/1-582 | Monca.augustus.g69462.t1/1-582	0.0218794	0.0211527	calcineurin B homologous 1	40	C:GO:0015630; P:GO:0090314; P:GO:0022406; C:GO:0000139;	[illegible]
Acrdi.XM_015901973.1/1-1830 | Monca.augustus.g35961.t1/1-2130	0.0271145	0.0211473	dolichyl-diphosphooligosaccharide-- glycosyltransferase subunit STT3A	5	C:GO:0008250; F:GO:0004579; C:GO:0016021; F:GO:0018279;	[illegible]
Acrdi.XM_015925311.1/1-381 | Monca.augustus.g10736.t1/1-918	0.0267261	0.0211448	Serine threonine- phosphatase 6 catalytic subunit	2	F:GO:0004721; P:GO:0006470	F:phosphoprotein phosphatase activity; P:protein dephosphorylation
Acrdi.XM_015922546.1/1-2349 | Monca.augustus.g23471.t1/1-2349	0.0333174	0.0211309	Multiple C2 and transmembrane domain-containing 1	2	C:GO:0016021; C:GO:0005783	C:integral component of membrane; C:endoplasmic reticulum
Acrdi.XM_015900413.1/1-396 | Monca.augustus.g49644.t1/1-636	0.024322	0.0211138	NADH dehydrogenase [ubiquinone] iron-sulfur mitochondrial	5	F:GO:0048038; F:GO:0046872; F:GO:0008137; P:GO:0055114;	[illegible]
Acrdi.XM_015899191.1/1-1263 | Monca.augustus.g24887.t1/1-1380	0.02898	0.0210595	probable tubulin polyglutamylase TTLL1	1	P:GO:0018095	P:protein polyglutamylation
Acrdi.XM_015912913.1/1-1431 | Monca.adi2mcaRNA23299_R6/1-1563	0.304488	0.0210322	RNA-directed DNA polymerase from mobile element jockey-like			
Acrdi.XM_015917646.1/1-1338 | Monca.adi2mcaRNA28589_R1/1-1344	0.222305	0.0209856	PREDICTED: uncharacterized protein LOC107351359			
Acrdi.XM_015919632.1/1-1269 | Monca.augustus.g5420.t1/1-1362	0.721422	0.0209498	PREDICTED: uncharacterized protein LOC107353291	2	F:GO:0004842; P:GO:0016567	F:ubiquitin-protein transferase activity; P:protein ubiquitination
Acrdi.XM_015913680.1/1-1188 | Monca.augustus.g40035.t1/1-1191	0.60493	0.0209449	galactoside 3(4)-L-fucosyltransferase-like	9	F:GO:0016757; F:GO:0008417; C:GO:0032580; P:GO:0036065;	[illegible]
Acrdi.XM_015920452.1/1-564 | Monca.augustus.g51640.t1/1-885	0.028212	0.0209422	Enoyl- mitochondrial	2	F:GO:0003824; P:GO:0008152	F:catalytic activity; P:metabolic process
Acrdi.XM_015898467.1/1-1806 | Monca.augustus.g33976.t1/1-2322	0.0267061	0.0209387	transducin-like enhancer 1 isoform X2	2	C:GO:0005634; P:GO:0006355	C:nucleus; P:regulation of transcription, DNA-templated
Acrdi.XM_015911881.1/1-543 | Monca.adi2mcaRNA22159_R0/1-501	0.154443	0.0209288	nuclease HARBI1			
Acrdi.XM_015913740.1/1-807 | Monca.augustus.g21662.t1/1-828	0.0317676	0.020922	serine arginine-rich splicing factor 1B	5	F:GO:0000166; C:GO:0005634; P:GO:0008380; F:GO:0003723;	[illegible]
Acrdi.XM_015912531.1/1-462 | Monca.augustus.g14875.t1/1-456	0.0232463	0.020821	ubiquitin-conjugating enzyme E2 N	30	F:GO:0061630; P:GO:0033182; F:GO:0061631; P:GO:0038095;	[illegible]
Acrdi.XM_015905022.1/1-507 | Monca.augustus.g40462.t1/1-507	0.0538859	0.020806	ubiquitin-conjugating enzyme E2 G1	10	F:GO:0005524; F:GO:0061630; C:GO:0070062; F:GO:0031625;	[illegible]
Acrdi.XM_015923104.1/1-3552 | Monca.adi2mcaRNA34711_R0/1-3753	0.398473	0.0207316	RNA-directed DNA polymerase from mobile element jockey	2	C:GO:0016020; C:GO:0016021	C:membrane; C:integral component of membrane
Acrdi.XM_015906304.1/1-648 | Monca.augustus.g18200.t1/1-675	0.789487	0.0206982	RNA-binding 25-like isoform X1			
Acrdi.XM_015908091.1/1-1395 | Monca.augustus.g33232.t1/1-1395	0.0235184	0.020643	uncharacterized F-box LRR-repeat -like			
Acrdi.XM_015915494.1/1-1467 | Monca.augustus.g68534.t1/1-3978	0.488541	0.0206419	Pro-Pol poly			
Acrdi.XM_015911863.1/1-843 | Monca.augustus.g43167.t1/1-852	0.0341202	0.0206148	CCR4-NOT transcription complex subunit 7-like	6	F:GO:0003676; F:GO:0004535; C:GO:0005634; P:GO:0090503;	[illegible]
Acrdi.XM_015898775.1/1-384 | Monca.augustus.g54232.t1/1-384	0.0533089	0.0205603	Profilin	5	P:GO:0042989; C:GO:0005938; C:GO:0015629; P:GO:0030036;	[illegible]
Acrdi.XM_015910937.1/1-1041 | Monca.adi2mcaRNA21139_R3/1-1041	0.710889	0.0205498	Integrase recombinase xerD-like			
Acrdi.XM_015904372.1/1-636 | Monca.augustus.g62180.t1/1-636	0.0369107	0.020451	ER lumen -retaining receptor 2	5	C:GO:0005789; P:GO:0015031; C:GO:0016021; F:GO:0046923;	[illegible]
Acrdi.XM_015893177.1/1-3036 | Monca.adi2mcaRNA39233_R0/1-3063	0.497525	0.0204437	OTU domain-containing 6B isoform X1			
Acrdi.XM_015916793.1/1-615 | Monca.augustus.g3859.t1/1-615	0.0345169	0.0204272	Ras-related Rab-18-B	8	F:GO:0005525; P:GO:0043009; P:GO:0001654; P:GO:0007264;	[illegible]
Acrdi.XM_015913184.1/1-681 | Monca.augustus.g2109.t1/1-681	0.0298778	0.0204257	Ras-related Rab-27A	30	P:GO:0036257; P:GO:0043320; C:GO:0005794; P:GO:0007596;	[illegible]
Acrdi.XM_015902778.1/1-528 | Monca.augustus.g33290.t1/1-528	0.0248435	0.0203253	B9 domain-containing 2-like	4	P:GO:0036372; P:GO:0001736; P:GO:0007163; C:GO:0005929	[illegible]
Acrdi.XM_015914549.1/1-891 | Monca.augustus.g12261.t1/1-891	0.0308627	0.0203001	boule-like	2	F:GO:0000166; F:GO:0003676	F:nucleotide binding; F:nucleic acid binding
Acrdi.XM_015904433.1/1-636 | Monca.adi2mcaRNA1389_R0/1-636	0.0442808	0.0202772	Kinase DC2	3	F:GO:0005524; F:GO:0004674; P:GO:0006468	[illegible]
Acrdi.XM_015921150.1/1-828 | Monca.adi2mcaRNA32484_R0/1-771	1.45247	0.0202233	zinc finger 862-like			
Acrdi.XM_015902879.1/1-1452 | Monca.augustus.g27564.t1/1-1449	0.0191868	0.0202212	U4 U6 small nuclear ribonucleo Prp31	13	P:GO:0060041; C:GO:0016607; C:GO:0005690; C:GO:0015030;	[illegible]
Acrdi.XM_015892702.1/1-1269 | Monca.augustus.g58968.t1/1-1251	0.389699	0.0202073	RNA-directed DNA polymerase from mobile element jockey-like			
Acrdi.XM_015917015.1/1-2685 | Monca.adi2mcaRNA2762_R0/1-2649	0.76762	0.0201497	PREDICTED: uncharacterized protein LOC107350773	3	C:GO:0005634; F:GO:0003682; P:GO:0006357	[illegible]
Acrdi.XM_015918974.1/1-2160 | Monca.adi2mcaRNA30108_R0/1-2166	0.0240676	0.0200995	vacuolar sorting-associated 52 homolog			
Acrdi.XM_015904288.1/1-333 | Monca.augustus.g4774.t1/1-360	0.177236	0.0200778	PREDICTED: uncharacterized protein LOC107339046			
Acrdi.XM_015918136.1/1-1542 | Monca.augustus.g53133.t1/1-1539	0.0452334	0.0200557	RNA-binding MEX3B-like	1	F:GO:0005488	F:binding
Acrdi.XM_015909048.1/1-990 | Monca.augustus.g42083.t1/1-1068	0.512656	0.0200285	PREDICTED: uncharacterized protein LOC107343480	3	F:GO:0003676; C:GO:0016020; C:GO:0016021	F:nucleic acid binding; C:membrane; C:integral component of membrane
Acrdi.XM_015920566.1/1-414 | Monca.augustus.g47224.t1/1-651	0.0188715	0.0199206	MOB kinase activator-like 1	2	P:GO:0016310; F:GO:0016301	P:phosphorylation; F:kinase activity
Acrdi.XM_015915869.1/1-1173 | Monca.adi2mcaRNA26575_R5/1-1044	0.87559	0.01989	52 kDa repressor of the inhibitor of the kinase-like			
Acrdi.XM_015912570.1/1-702 | Monca.augustus.g50573.t1/1-708	0.568598	0.0198803	G2 M phase-specific E3 ubiquitin- ligase-like			
Acrdi.XM_015914020.1/1-1176 | Monca.augustus.g18746.t1/1-1176	0.0411257	0.0198609	POU class transcription factor	5	C:GO:0005634; F:GO:0003700; P:GO:0006351; P:GO:0006355;	[illegible]
Acrdi.XM_015905706.1/1-1053 | Monca.adi2mcaRNA15369_R3/1-1014	0.51878	0.0198333	Pro-Pol poly			
Acrdi.XM_015896241.1/1-1314 | Monca.augustus.g54602.t1/1-1314	0.0388283	0.0197825	hepatocyte nuclear factor 4-gamma-like isoform X2	8	C:GO:0005634; F:GO:0003700; F:GO:0008270; P:GO:0006351;	[illegible]
Acrdi.XM_015912572.1/1-462 | Monca.augustus.g50570.t1/1-435	0.365392	0.0197523	PREDICTED: uncharacterized protein LOC107346752			
Acrdi.XM_015910398.1/1-768 | Monca.augustus.g26334.t1/1-768	0.0281178	0.0196993	parkin coregulated gene	18	P:GO:0007286; P:GO:0060548; F:GO:0043014; C:GO:0043005;	[illegible]
Acrdi.XM_015923036.1/1-639 | Monca.augustus.g57885.t1/1-639	0.165343	0.0196594	P2X purinoceptor 7-like	2	C:GO:0016020; C:GO:0016021	C:membrane; C:integral component of membrane
Acrdi.XM_015897502.1/1-1065 | Monca.augustus.g18031.t1/1-1065	0.0197826	0.0196475	Replication factor C subunit 3	8	C:GO:0031390; C:GO:0005634; F:GO:0003689; F:GO:0043142;	[illegible]
Acrdi.XM_015919180.1/1-1569 | Monca.augustus.g62826.t1/1-1599	0.030645	0.0194839	T-complex 1 subunit beta	4	F:GO:0005524; C:GO:0005737; F:GO:0051082; P:GO:0006457	F:ATP binding; C:cytoplasm; F:unfolded protein binding; P:protein folding
Acrdi.XM_015913505.1/1-603 | Monca.augustus.g9930.t1/1-603	0.0367159	0.0194803	BTB POZ domain-containing KCTD5	1	P:GO:0051260	P:protein homooligomerization
Acrdi.XM_015914465.1/1-3261 | Monca.adi2mcaRNA25005_R0/1-3237	0.0267979	0.0193737	Exportin-7	6	C:GO:0005737; C:GO:0016021; P:GO:0006611; F:GO:0008536;	[illegible]
Acrdi.XM_015900256.1/1-1374 | Monca.adi2mcaRNA9475_R4/1-714	0.300318	0.0193543	PREDICTED: uncharacterized protein LOC107335261			
Acrdi.XM_015891713.1/1-465 | Monca.augustus.g52055.t1/1-636	0.0207567	0.0192471	AMME syndrome candidate gene 1 isoform X2			
Acrdi.XM_015906472.1/1-666 | Monca.augustus.g22529.t1/1-834	0.0280774	0.0192227	radial spoke head 9 homolog	3	P:GO:0060294; P:GO:0035082; P:GO:0044458	[illegible]
Acrdi.XM_015899451.1/1-1650 | Monca.augustus.g48271.t1/1-1626	0.0303967	0.0191739	elongator complex 3	10	F:GO:0004402; C:GO:0008023; C:GO:0005737; C:GO:0033588;	[illegible]
Acrdi.XM_015897880.1/1-1089 | Monca.augustus.g71069.t1/1-663	0.264827	0.0191728	piggyBac transposable element-derived 4-like			
Acrdi.XM_015903911.1/1-1719 | Monca.augustus.g30300.t1/1-1722	0.0270677	0.0190754	Dual specificity tyrosine-phosphorylation-regulated kinase 2	8	C:GO:0005634; F:GO:0005524; F:GO:0046872; P:GO:0007165;	[illegible]
Acrdi.XM_015904570.1/1-723 | Monca.adi2mcaRNA14099_R2/1-855	0.548	0.0190675	zinc finger and SCAN domain-containing 29-like			
Acrdi.XM_015905510.1/1-552 | Monca.augustus.g28265.t1/1-783	0.0299628	0.0190211	ras-related Rap-1A	7	F:GO:0005525; P:GO:0007507; P:GO:0003428; P:GO:0007264;	[illegible]
Acrdi.XM_015906567.1/1-468 | Monca.adi2mcaRNA16318_R0/1-321	0.0265956	0.0189019	structural maintenance of chromosomes 1A-like	6	C:GO:0005634; F:GO:0005524; P:GO:0006281; P:GO:0007064;	[illegible]
Acrdi.XM_015912476.1/1-537 | Monca.augustus.g64806.t1/1-540	0.0156319	0.018829	PREDICTED: uncharacterized protein LOC107346672			
Acrdi.XM_015892884.1/1-663 | Monca.augustus.g14314.t1/1-663	0.0284625	0.0187978	vacuolar sorting-associated 28 homolog	3	P:GO:0043328; C:GO:0000813; F:GO:0032403	[illegible]
Acrdi.XM_015925102.1/1-387 | Monca.augustus.g51150.t1/1-387	0.0399233	0.0187863	intraflagellar transport 20 homolog	26	C:GO:0032391; P:GO:0036372; P:GO:0061351; P:GO:1902017;	[illegible]
Acrdi.XM_015893481.1/1-2346 | Monca.adi2mcaRNA35599_R4/1-3813	0.401004	0.0187406	ATP-dependent DNA helicase PIF1			
Acrdi.XM_015914827.1/1-624 | Monca.augustus.g41785.t1/1-1125	0.0197822	0.0187216	Replication factor C subunit 2			
Acrdi.XM_015892098.1/1-543 | Monca.augustus.g32184.t1/1-645	0.0298963	0.0187089	ras-related Rab-5C	6	F:GO:0005525; C:GO:0030139; C:GO:0005769; P:GO:0007264;	[illegible]
Acrdi.XM_015899733.1/1-477 | Monca.adi2mcaRNA8863_R1/1-489	0.252616	0.0187005	PREDICTED: uncharacterized protein LOC107334760	5	F:GO:0003676; F:GO:0046872; C:GO:0005739; F:GO:0052856;	[illegible]
Acrdi.XM_015896344.1/1-1053 | Monca.adi2mcaRNA5182_R2/1-987	0.791003	0.0186617	tripartite motif-containing 16-like			
Acrdi.XM_015915460.1/1-2289 | Monca.adi2mcaRNA26168_R0/1-2223	0.0243973	0.0186211	DNA-directed RNA polymerase II subunit RPB2	5	F:GO:0003677; F:GO:0001055; F:GO:0032549; P:GO:0006366;	[illegible]
Acrdi.XM_015908885.1/1-1122 | Monca.adi2mcaRNA18895_R3/1-1029	0.433314	0.0185981	Retrovirus-related Pol poly from transposon			
Acrdi.XM_015906624.1/1-453 | Monca.augustus.g70213.t1/1-453	0.0295198	0.0185967	ubiquitin-conjugating enzyme E2 W	19	P:GO:0006281; F:GO:0061630; P:GO:0070979; F:GO:0008565;	[illegible]
Acrdi.XM_015904691.1/1-612 | Monca.augustus.g1245.t1/1-1017	0.25199	0.018578	PREDICTED: uncharacterized protein LOC107339409 isoform X3			
Acrdi.XM_015917684.1/1-672 | Monca.adi2mcaRNA28641_R3/1-621	0.479567	0.0185547	PREDICTED: uncharacterized protein LOC107351398			
Acrdi.XM_015905345.1/1-2220 | Monca.augustus.g17918.t1/1-3219	0.949757	0.0185397	zinc finger 862-like			
Acrdi.XM_015910927.1/1-1635 | Monca.adi2mcaRNA21144_R7/1-1560	0.745632	0.0184687	zinc finger 862-like			
Acrdi.XM_015899025.1/1-906 | Monca.augustus.g47843.t1/1-906	0.688444	0.0184548	nuclease HARBI1			
Acrdi.XM_015910089.1/1-2607 | Monca.augustus.g16727.t1/1-4428	0.361451	0.0184013	Pro-Pol poly			
Acrdi.XM_015908016.1/1-504 | Monca.augustus.g6865.t1/1-504	0.029474	0.0183724	Cysteine-rich hydrophobic domain-containing 2	3	C:GO:0016021; P:GO:0006810; C:GO:0005622	C:integral component of membrane; P:transport; C:intracellular
Acrdi.XM_015908941.1/1-2532 | Monca.augustus.g31858.t1/1-2919	0.0217042	0.0183513	116 kDa U5 small nuclear ribonucleo component	6	F:GO:0005525; C:GO:0030529; F:GO:0003746; F:GO:0003924;	[illegible]
Acrdi.XM_015923883.1/1-1590 | Monca.augustus.g10658.t1/1-3126	0.202081	0.0183048	PREDICTED: uncharacterized protein LOC107357246			
Acrdi.XM_015913775.1/1-603 | Monca.augustus.g45557.t1/1-603	0.023317	0.0183008	Ras-related Rab-35	25	P:GO:0036010; P:GO:1990090; P:GO:0015031; P:GO:0000910;	[illegible]
Acrdi.XM_015922506.1/1-1641 | Monca.augustus.g33029.t1/1-1764	0.917925	0.018297	PREDICTED: uncharacterized protein LOC107355887			
Acrdi.XM_015896891.1/1-1056 | Monca.augustus.g33184.t1/1-1059	0.0229663	0.0182895	casein kinase I isoform alpha isoform X4	26	P:GO:0008360; C:GO:0016607; P:GO:0007030; P:GO:0045104;	[illegible]
Acrdi.XM_015902749.1/1-885 | Monca.augustus.g54276.t1/1-876	0.00823698	0.0182601	Hepatocyte nuclear factor 3-beta	12	P:GO:0007418; C:GO:0005634; F:GO:0019904; P:GO:0021779;	[illegible]
Acrdi.XM_015909635.1/1-573 | Monca.augustus.g53334.t1/1-573	0.0242862	0.0182305	cell division control 42 homolog isoform X1	15	P:GO:0060271; P:GO:0007264; P:GO:0060041; P:GO:0002040;	[illegible]
Acrdi.XM_015893996.1/1-456 | Monca.augustus.g23247.t1/1-792	0.330834	0.0182234	Gag-Pol poly			
Acrdi.XM_015908861.1/1-2556 | Monca.adi2mcaRNA18871_R0/1-2502	0.164602	0.0181767	52 kDa repressor of the inhibitor of the kinase			
Acrdi.XM_015912788.1/1-741 | Monca.augustus.g29502.t1/1-741	0.161064	0.0181721	UPF0746 DDB_G0281095-like			
Acrdi.XM_015896961.1/1-1548 | Monca.adi2mcaRNA613_R0/1-1548	0.317467	0.0181678	PREDICTED: uncharacterized protein LOC107332231			
Acrdi.XM_015899575.1/1-642 | Monca.augustus.g6975.t1/1-663	0.229595	0.0180903	GFP-like chromo	4	P:GO:0018298; P:GO:0010117; P:GO:0008218; C:GO:0018995	[illegible]
Acrdi.XM_015892525.1/1-873 | Monca.augustus.g36522.t1/1-1128	0.0274111	0.0179877	alpha-centractin	2	F:GO:0005524; C:GO:0005869	F:ATP binding; C:dynactin complex
Acrdi.XM_015896260.1/1-789 | Monca.augustus.g33732.t1/1-786	0.0500794	0.0178834	nematogalectin-related isoform X1	3	C:GO:0042151; C:GO:0016021; F:GO:0030246	C:nematocyst; C:integral component of membrane; F:carbohydrate binding
Acrdi.XM_015901271.1/1-981 | Monca.adi2mcaRNA10592_R8/1-1257	1.41387	0.0178624	C2H2 zinc finger	3	F:GO:0003676; F:GO:0003677; F:GO:0046872	F:nucleic acid binding; F:DNA binding; F:metal ion binding
Acrdi.XM_015903140.1/1-1287 | Monca.augustus.g70483.t1/1-1287	0.0312733	0.0178607	spliceosome RNA helicase Ddx39b	27	P:GO:0045727; C:GO:0016607; P:GO:0006369; F:GO:0043008;	[illegible]
Acrdi.XM_015915559.1/1-495 | Monca.augustus.g31917.t1/1-495	0.0196034	0.0178195	Ubiquitin-conjugating enzyme E2 G2	6	F:GO:0005524; F:GO:0061630; F:GO:0031625; C:GO:0005737;	[illegible]
Acrdi.XM_015917165.1/1-2709 | Monca.augustus.g45677.t1/1-3153	0.258018	0.0176721	PREDICTED: uncharacterized protein LOC107350910			
Acrdi.XM_015918067.1/1-699 | Monca.augustus.g34633.t1/1-1590	0.028126	0.0176167	CBFA2T1-like isoform X5	4	C:GO:0005634; F:GO:0003700; P:GO:0045892; F:GO:0003714	[illegible]
Acrdi.XM_015901611.1/1-702 | Monca.augustus.g4707.t1/1-855	0.0338276	0.0176161	Cell cycle checkpoint RAD1	5	F:GO:0008408; P:GO:0000077; P:GO:0006281; F:GO:0003684;	[illegible]
Acrdi.XM_015907427.1/1-4296 | Monca.adi2mcaRNA17264_R0/1-4116	0.377117	0.0174792	Pro-Pol poly			
Acrdi.XM_015904558.1/1-468 | Monca.augustus.g68207.t1/1-468	0.0222344	0.017477	tat pathway signal sequence	1	F:GO:0046872	F:metal ion binding
Acrdi.XM_015918796.1/1-897 | Monca.augustus.g69769.t1/1-1416	0.238137	0.0174732	THAP domain-containing 11			
Acrdi.XM_015915184.1/1-870 | Monca.augustus.g29744.t1/1-870	0.0657157	0.0174514	Homeobox Nkx-	1	F:GO:0003677	F:DNA binding
Acrdi.XM_015906956.1/1-1149 | Monca.augustus.g49145.t1/1-1128	0.0221668	0.0174166	SWI SNF-related matrix-associated actin-dependent regulator of chror	36	P:GO:0043923; P:GO:0006281; P:GO:0016569; P:GO:0008285;	[illegible]
Acrdi.XM_015908235.1/1-1593 | Monca.adi2mcaRNA18213_R0/1-1596	0.0211088	0.0173191	T-complex 1 subunit zeta	4	F:GO:0005524; C:GO:0005737; F:GO:0051082; P:GO:0006457	F:ATP binding; C:cytoplasm; F:unfolded protein binding; P:protein folding
Acrdi.XM_015909752.1/1-816 | Monca.adi2mcaRNA12993_R1/1-1494	0.189509	0.0172162	RNA-directed DNA polymerase from transposon BS			
Acrdi.XM_015903159.1/1-930 | Monca.augustus.g19996.t1/1-930	0.0271998	0.0172139	Transcription initiation factor IIB	6	F:GO:0003743; F:GO:0008270; P:GO:0006355; P:GO:0006352;	[illegible]
Acrdi.XM_015902148.1/1-540 | Monca.augustus.g46367.t1/1-540	0.0231509	0.0171368	ADP-ribosylation factor 6	6	F:GO:0005525; P:GO:0007264; P:GO:0055113; C:GO:0005622;	[illegible]
Acrdi.XM_015916305.1/1-1626 | Monca.augustus.g3591.t1/1-2010	0.025977	0.0171119	E3 ubiquitin- ligase TRAF7	5	F:GO:0004842; F:GO:0008270; P:GO:0043410; C:GO:0044424;	[illegible]
Acrdi.XM_015920543.1/1-312 | Monca.augustus.g38789.t1/1-405	0.0234642	0.0170394	NADH dehydrogenase [ubiquinone] 1 beta subcomplex subunit 10			
Acrdi.XM_015925278.1/1-786 | Monca.adi2mcaRNA37208_R1/1-759	0.83535	0.0169313	nucleoporin GLE1-like			
Acrdi.XM_015909718.1/1-711 | Monca.augustus.g41732.t1/1-711	0.0392134	0.0167942	Ubiquitin-conjugating enzyme E2 R2	9	F:GO:0005524; F:GO:0061630; P:GO:0043161; F:GO:0031625;	[illegible]
Acrdi.XM_015896297.1/1-1587 | Monca.augustus.g135.t1/1-3228	0.50084	0.016772	52 kDa repressor of the inhibitor of the kinase-like			
Acrdi.XM_015919800.1/1-1707 | Monca.augustus.g41937.t1/1-1875	0.0238463	0.0166478	poly(U)-binding-splicing factor PUF60 isoform X3	2	F:GO:0000166; F:GO:0003676	F:nucleotide binding; F:nucleic acid binding
Acrdi.XM_015910884.1/1-444 | Monca.augustus.g34118.t1/1-1680	0.176317	0.0165707	PREDICTED: uncharacterized protein LOC107345175			
Acrdi.XM_015897564.1/1-762 | Monca.augustus.g60806.t1/1-3186	0.024637	0.0164846	E3 ubiquitin- ligase	2	F:GO:0008270; C:GO:0005622	F:zinc ion binding; C:intracellular
Acrdi.XM_015918608.1/1-1641 | Monca.augustus.g23476.t1/1-1641	0.0279378	0.0164308	T-complex 1 subunit gamma-like	6	F:GO:0005524; C:GO:0005737; P:GO:0048048; P:GO:0046671;	[illegible]
Acrdi.XM_015913604.1/1-1068 | Monca.augustus.g35757.t1/1-1251	0.573686	0.0164071	PREDICTED: uncharacterized protein LOC107347645			
Acrdi.XM_015896762.1/1-990 | Monca.augustus.g52112.t1/1-1032	0.0159106	0.0163576	WD repeat domain phosphoinositide-interacting 3	9	P:GO:0044804; P:GO:0034497; C:GO:0034045; P:GO:0006497;	[illegible]
Acrdi.XM_015902887.1/1-378 | Monca.augustus.g27547.t1/1-378	0.0222612	0.0162866	Microtubule-associated s 1A 1B light chain 3C	6	P:GO:0000045; C:GO:0005829; P:GO:0000422; C:GO:0000421;	[illegible]
Acrdi.XM_015892259.1/1-996 | Monca.adi2mcaRNA3911_R1/1-951	0.347307	0.016266	PREDICTED: uncharacterized protein LOC107327509	1	F:GO:0003676	F:nucleic acid binding
Acrdi.XM_015903484.1/1-1221 | Monca.augustus.g11501.t1/1-1191	0.270794	0.0161784	PREDICTED: uncharacterized protein LOC107338249	1	F:GO:0005488	F:binding
Acrdi.XM_015922980.1/1-753 | Monca.augustus.g47696.t1/1-762	0.147128	0.0161783	UPF0746 DDB_G0281095-like			
Acrdi.XM_015916613.1/1-1074 | Monca.augustus.g32917.t1/1-1074	0.0365412	0.0161564	Guanine nucleotide-binding subunit alpha-12	8	F:GO:0005525; F:GO:0001664; C:GO:0005834; P:GO:0007264;	[illegible]
Acrdi.XM_015922028.1/1-2097 | Monca.augustus.g10499.t1/1-4014	0.599615	0.0161491	zinc finger MYM-type 1-like			
Acrdi.XM_015903881.1/1-627 | Monca.adi2mcaRNA13343_R0/1-513	0.0207913	0.0161308	l(2)37Cc	1	C:GO:0016020	C:membrane
Acrdi.XM_015898014.1/1-1917 | Monca.adi2mcaRNA6992_R0/1-2124	0.75985	0.016109	Transposon Tf2-6 poly	5	C:GO:0005813; C:GO:0016020; C:GO:0016021; P:GO:0034454;	[illegible]
Acrdi.XM_015925105.1/1-1032 | Monca.adi2mcaRNA37010_R0/1-600	0.263983	0.0160148	zinc finger 862-like			
Acrdi.XM_015917704.1/1-939 | Monca.adi2mcaRNA28657_R0/1-942	0.0304094	0.0159536	dnaJ homolog subfamily B member 13-like	2	F:GO:0051082; P:GO:0006457	F:unfolded protein binding; P:protein folding
Acrdi.XM_015904292.1/1-684 | Monca.augustus.g16969.t1/1-738	0.0182268	0.0158369	Proteasome subunit alpha type-6	10	C:GO:0005844; F:GO:0051059; C:GO:0070062; F:GO:0003723;	[illegible]
Acrdi.XM_015895052.1/1-693 | Monca.augustus.g48681.t1/1-885	0.862861	0.0157716	octopamine receptor-like	1	F:GO:0004871	F:signal transducer activity
Acrdi.XM_015900759.1/1-600 | Monca.augustus.g5458.t1/1-600	0.0279201	0.0157579	RNA polymerase II subunit A C-terminal domain phosphatase SSU72	7	F:GO:0005515; C:GO:0005737; C:GO:0005847; P:GO:0070940;	[illegible]
Acrdi.XM_015922827.1/1-768 | Monca.augustus.g1651.t1/1-765	0.0267085	0.015736	serine arginine-rich splicing factor 6 isoform X1	2	F:GO:0097159; F:GO:1901363	F:organic cyclic compound binding; F:heterocyclic compound binding
Acrdi.XM_015916170.1/1-336 | Monca.augustus.g12398.t1/1-336	0.0197922	0.0156386	E3 ubiquitin- ligase RBX1	38	P:GO:0000717; P:GO:0000715; F:GO:0034450; P:GO:0006283;	[illegible]
Acrdi.XM_015920163.1/1-591 | Monca.augustus.g27120.t1/1-591	0.0251677	0.0155182	neuronal calcium sensor 1-like	1	F:GO:0005509	F:calcium ion binding
Acrdi.XM_015914781.1/1-573 | Monca.adi2mcaRNA25401_R0/1-522	0.041408	0.0154121	Histone deacetylase complex subunit SAP30L	6	F:GO:0003712; F:GO:0004407; C:GO:0000118; P:GO:0001947;	[illegible]
Acrdi.XM_015924235.1/1-1290 | Monca.adi2mcaRNA36004_R1/1-1290	0.32892	0.0153847	nuclease HARBI1			
Acrdi.XM_015914514.1/1-489 | Monca.augustus.g27244.t1/1-489	0.0116832	0.0152744	Calmodulin	2	F:GO:0005509; F:GO:0019899	F:calcium ion binding; F:enzyme binding
Acrdi.XM_015903172.1/1-1560 | Monca.augustus.g52466.t1/1-1929	0.0332956	0.0152741	pre-B-cell leukemia transcription factor 1 isoform X2			
Acrdi.XM_015904592.1/1-1338 | Monca.augustus.g70388.t1/1-1371	0.72167	0.0152013	nuclease HARBI1			
Acrdi.XM_015914797.1/1-954 | Monca.augustus.g47152.t1/1-954	0.0194402	0.0151335	ribose-phosphate pyrophosphokinase 2	9	F:GO:0000287; F:GO:0005524; F:GO:0042803; P:GO:0009156;	[illegible]
Acrdi.XM_015925055.1/1-3000 | Monca.augustus.g69070.t1/1-1671	0.512318	0.0151304	ATP-dependent RNA helicase DDX51			
Acrdi.XM_015916440.1/1-987 | Monca.adi2mcaRNA27239_R0/1-1020	0.903214	0.0150452	nuclease HARBI1			
Acrdi.XM_015916103.1/1-429 | Monca.augustus.g12572.t1/1-429	0.0324964	0.0149223	40S ribosomal S23	13	C:GO:0022627; P:GO:0006364; C:GO:0005730; P:GO:0006413;	[illegible]
Acrdi.XM_015917096.1/1-681 | Monca.augustus.g7464.t1/1-681	2.18236	0.0148434	PREDICTED: uncharacterized protein LOC107350848	3	F:GO:0097159; F:GO:1901363; P:GO:0009987	[illegible]
Acrdi.XM_015904887.1/1-450 | Monca.augustus.g66113.t1/1-450	0.0202151	0.0147555	myosin-2 essential light chain	9	F:GO:0032440; F:GO:0005509; C:GO:0016460; C:GO:0031475;	[illegible]
Acrdi.XM_015901522.1/1-2307 | Monca.augustus.g25823.t1/1-2310	0.0205443	0.0145776	Cullin-1	10	C:GO:1990452; P:GO:0008283; F:GO:0061630; F:GO:0031625;	[illegible]
Acrdi.XM_015913198.1/1-1266 | Monca.augustus.g32068.t1/1-1359	0.0238925	0.0145686	tubulin alpha-1A chain	15	C:GO:0036464; F:GO:0019904; C:GO:0043209; F:GO:0005200;	[illegible]
Acrdi.XM_015909049.1/1-2472 | Monca.augustus.g37706.t1/1-2472	0.699842	0.0144878	RNA-directed DNA polymerase from mobile element jockey	5	P:GO:0090305; F:GO:0003677; C:GO:0005634; F:GO:0008270;	[illegible]
Acrdi.XM_015892334.1/1-1110 | Monca.adi2mcaRNA38258_R0/1-1107	0.217097	0.0143041	nuclease HARBI1			
Acrdi.XM_015906965.1/1-426 | Monca.augustus.g49152.t1/1-426	0.0188672	0.0142945	thioredoxin 4A isoform X1	10	P:GO:0051301; F:GO:0005515; C:GO:0005737; C:GO:0046540;	[illegible]
Acrdi.XM_015916205.1/1-795 | Monca.augustus.g43600.t1/1-933	0.0149501	0.0142387	proliferating cell nuclear antigen	44	C:GO:0043626; P:GO:0008283; P:GO:0016925; P:GO:0032077;	[illegible]
Acrdi.XM_015905405.1/1-654 | Monca.augustus.g9648.t1/1-657	0.0225975	0.0142362	Ras-related Rab-11A	47	P:GO:0003091; C:GO:0032154; P:GO:0015031; P:GO:0000910;	[illegible]
Acrdi.XM_015894133.1/1-2538 | Monca.augustus.g50757.t1/1-2121	0.204103	0.0141593	exonuclease R569			
Acrdi.XM_015917308.1/1-1560 | Monca.augustus.g62351.t1/1-2208	0.0194131	0.0140297	tRNA (uracil-5-)-methyltransferase homolog A	4	F:GO:0003676; P:GO:0016070; F:GO:0008168; P:GO:0032259	[illegible]
Acrdi.XM_015896999.1/1-1371 | Monca.augustus.g28909.t1/1-1143	0.00524672	0.014022	expressed conserved	7	C:GO:0005634; C:GO:0005615; C:GO:0043209; C:GO:0070062;	[illegible]
Acrdi.XM_015914415.1/1-1080 | Monca.augustus.g60708.t1/1-1080	0.0402528	0.0137896	ELAV-like	10	C:GO:0005634; C:GO:0036477; P:GO:0007626; C:GO:0043232;	[illegible]
Acrdi.XM_015924203.1/1-1320 | Monca.augustus.g478.t1/1-1317	0.0165343	0.0137808	mothers against decapentaplegic homolog 5	21	P:GO:0007281; P:GO:0006351; F:GO:0000979; P:GO:0060395;	[illegible]
Acrdi.XM_015893380.1/1-360 | Monca.adi2mcaRNA40027_R0/1-474	0.0042324	0.0137135	S-phase kinase-associated 1	7	C:GO:0005829; P:GO:0031146; C:GO:0019005; P:GO:0016310;	[illegible]
Acrdi.XM_015924577.1/1-1344 | Monca.augustus.g41304.t1/1-1434	0.0172447	0.0136996	F-box LRR-repeat 2-like			
Acrdi.XM_015921009.1/1-2673 | Monca.augustus.g41192.t1/1-2673	0.0156018	0.0135195	transportin-1 isoform X1	11	C:GO:0031965; C:GO:0070062; C:GO:0005737; P:GO:0000060;	[illegible]
Acrdi.XM_015913726.1/1-348 | Monca.augustus.g21649.t1/1-348	0.0265757	0.0135065	Transcription elongation factor B polypeptide 2	3	C:GO:0030891; C:GO:0070449; P:GO:0006368	[illegible]
Acrdi.XM_015898532.1/1-351 | Monca.augustus.g34002.t1/1-351	0.0233948	0.0134778	DNA-directed RNA polymerase II subunit RPB11-a	5	F:GO:0003677; F:GO:0001055; P:GO:0006366; F:GO:0046983;	[illegible]
Acrdi.XM_015917353.1/1-2613 | Monca.adi2mcaRNA28284_R0/1-3114	0.303011	0.013311	cytosolic carboxypeptidase 2			
Acrdi.XM_015896823.1/1-1236 | Monca.augustus.g13500.t1/1-1236	0.0213102	0.0132764	26S protease regulatory subunit 6B	10	F:GO:0005524; C:GO:0008540; C:GO:0031597; C:GO:0031595;	[illegible]
Acrdi.XM_015922987.1/1-462 | Monca.augustus.g67842.t1/1-462	0.00940734	0.0132317	ORM1 3	5	P:GO:1900060; F:GO:0005515; C:GO:0016021; C:GO:0035339;	[illegible]
Acrdi.XM_015915785.1/1-2190 | Monca.augustus.g61011.t1/1-2190	0.0192073	0.0132236	DNA replication licensing factor mcm5	17	F:GO:0016887; P:GO:0006270; P:GO:0043009; P:GO:0030174;	[illegible]
Acrdi.XM_015922051.1/1-420 | Monca.augustus.g2996.t1/1-420	0.0161282	0.013055	60S ribosomal L23	15	C:GO:0022625; P:GO:0006364; C:GO:0005730; F:GO:0070180;	[illegible]
Acrdi.XM_015903310.1/1-732 | Monca.adi2mcaRNA12669_R0/1-729	0.0189464	0.0130267	40S ribosomal S3	59	P:GO:0006281; P:GO:0043507; P:GO:1902231; P:GO:1990090;	[illegible]
Acrdi.XM_015922174.1/1-1356 | Monca.adi2mcaRNA33626_R0/1-1356	0.180718	0.0130034	nuclease HARBI1	1	F:GO:0003676	F:nucleic acid binding
Acrdi.XM_015919621.1/1-1044 | Monca.adi2mcaRNA30829_R0/1-1272	0.793133	0.0129459	E3 ubiquitin- ligase TOM1-	2	F:GO:0004842; P:GO:0016567	F:ubiquitin-protein transferase activity; P:protein ubiquitination
Acrdi.XM_015896550.1/1-1923 | Monca.adi2mcaRNA5402_R1/1-1905	0.775746	0.0129195	mediator of RNA polymerase II transcription subunit 34-like	2	P:GO:0006259; F:GO:0016787	P:DNA metabolic process; F:hydrolase activity
Acrdi.XM_015921956.1/1-741 | Monca.adi2mcaRNA25546_R8/1-1989	1.07766	0.0128197	RNA-directed DNA polymerase from transposon BS			
Acrdi.XM_015911593.1/1-4188 | Monca.adi2mcaRNA21838_R2/1-4146	0.314917	0.012726	TPA_exp: gag-pol			
Acrdi.XM_015916637.1/1-894 | Monca.adi2mcaRNA27446_R0/1-897	0.0221099	0.0127004	pre-mRNA-splicing factor ATP-dependent RNA helicase DHX15	6	F:GO:0003676; F:GO:0005524; C:GO:0005737; C:GO:0005681;	[illegible]
Acrdi.XM_015922567.1/1-477 | Monca.augustus.g54977.t1/1-477	0.013245	0.012638	SUMO-conjugating enzyme UBC9-B	8	C:GO:0005634; F:GO:0005524; F:GO:0061630; F:GO:0031625;	[illegible]
Acrdi.XM_015923197.1/1-1410 | Monca.augustus.g20025.t1/1-1374	0.358738	0.0125961	G2 M phase-specific E3 ubiquitin- ligase-like			
Acrdi.XM_015894179.1/1-1059 | Monca.augustus.g56706.t1/1-1269	0.0119345	0.0125639	AP-1 complex subunit mu-1	5	P:GO:0016192; P:GO:0048565; C:GO:0030131; P:GO:0007420;	[illegible]
Acrdi.XM_015891686.1/1-1752 | Monca.adi2mcaRNA37539_R7/1-1698	0.956651	0.0123689	toll-like receptor 6	2	F:GO:0003676; C:GO:0044424	F:nucleic acid binding; C:intracellular part
Acrdi.XM_015904559.1/1-621 | Monca.adi2mcaRNA14108_R3/1-621	0.496843	0.0123374	PREDICTED: uncharacterized protein LOC107339294			
Acrdi.XM_015925101.1/1-858 | Monca.augustus.g51151.t1/1-858	0.0224082	0.0122486	BTB POZ domain-containing adapter for CUL3-mediated degradation 1	1	P:GO:0051260	P:protein homooligomerization
Acrdi.XM_015923505.1/1-2256 | Monca.augustus.g47470.t1/1-2322	0.0136883	0.0122341	transport Sec23A	4	F:GO:0008270; P:GO:0006888; P:GO:0006886; C:GO:0030127	[illegible]
Acrdi.XM_015919455.1/1-696 | Monca.augustus.g25233.t1/1-1620	0.0170744	0.0121717	ATP synthase subunit mitochondrial	5	C:GO:0045261; F:GO:0005524; P:GO:0015991; P:GO:0015986;	[illegible]
Acrdi.XM_015891867.1/1-702 | Monca.adi2mcaRNA37742_R8/1-702	0.546901	0.0121273	RNA-directed DNA polymerase from mobile element jockey-like			
Acrdi.XM_015902150.1/1-525 | Monca.augustus.g46366.t1/1-525	0.0212792	0.0121227	ADP-ribosylation factor 6	3	F:GO:0005525; P:GO:0007264; C:GO:0005622	[illegible]
Acrdi.XM_015912934.1/1-1674 | Monca.augustus.g51634.t1/1-1674	0.0170731	0.0118753	intraflagellar transport 56 isoform X1			
Acrdi.XM_015907492.1/1-1332 | Monca.augustus.g23755.t1/1-1332	0.0222262	0.0118579	serine threonine- phosphatase 2A 55 kDa regulatory subunit B alpha is	1	C:GO:0000159	C:protein phosphatase type 2A complex
Acrdi.XM_015910863.1/1-1413 | Monca.augustus.g70038.t1/1-1416	0.0144633	0.0117846	ruvB-like 2	15	P:GO:0006281; P:GO:0060420; P:GO:0006351; C:GO:0000812;	[illegible]
Acrdi.XM_015903885.1/1-687 | Monca.adi2mcaRNA13346_R0/1-666	0.251416	0.0117625	UPF0746 DDB_G0281095-like			
Acrdi.XM_015909133.1/1-1578 | Monca.adi2mcaRNA19166_R0/1-1542	0.398022	0.0117042	PREDICTED: uncharacterized protein LOC107343550			
Acrdi.XM_015905888.1/1-1047 | Monca.adi2mcaRNA15601_R0/1-843	0.0230559	0.0116536	F-box LRR-repeat 20			
Acrdi.XM_015921926.1/1-429 | Monca.augustus.g16883.t1/1-747	0.291527	0.0116377	PREDICTED: uncharacterized protein LOC107355348			
Acrdi.XM_015908187.1/1-906 | Monca.augustus.g31228.t1/1-906	0.64327	0.011561	nuclease HARBI1			
Acrdi.XM_015913000.1/1-579 | Monca.augustus.g56204.t1/1-588	0.0189918	0.0115455	Hematopoietically-expressed homeobox hhex	13	P:GO:0009790; P:GO:0030097; P:GO:0009952; P:GO:0006355;	[illegible]
Acrdi.XM_015913393.1/1-615 | Monca.augustus.g15037.t1/1-615	0.0158655	0.0115123	Proteasome subunit beta type-3	5	C:GO:0005634; C:GO:0005737; C:GO:0005839; F:GO:0004298;	[illegible]
Acrdi.XM_015917648.1/1-600 | Monca.adi2mcaRNA28592_R0/1-600	0.154454	0.0115047	TNF receptor-associated factor 5-like isoform X2			
Acrdi.XM_015916329.1/1-543 | Monca.augustus.g64078.t1/1-543	0.0131245	0.0114781	ubiquitin-conjugating enzyme E2 H isoform X1	8	F:GO:0005524; F:GO:0061630; P:GO:0043161; P:GO:0070979;	[illegible]
Acrdi.XM_015902067.1/1-756 | Monca.augustus.g10207.t1/1-1158	0.0141262	0.0114189	ras-related GTP-binding C-like	10	F:GO:0005525; C:GO:0005634; P:GO:0071230; F:GO:0003924;	[illegible]
Acrdi.XM_015903056.1/1-1026 | Monca.augustus.g10956.t1/1-1116	0.0166868	0.0113832	casein kinase I isoform alpha isoform X5	29	P:GO:0008360; C:GO:0016607; P:GO:0007030; P:GO:0045104;	[illegible]
Acrdi.XM_015914270.1/1-861 | Monca.augustus.g5907.t1/1-861	0.0191206	0.0113411	Alpha beta hydrolase domain-containing 17C	1	F:GO:0016787	F:hydrolase activity
Acrdi.XM_015900334.1/1-900 | Monca.adi2mcaRNA9599_R0/1-900	0.0124076	0.0111464	Mitogen-activated kinase 1	83	P:GO:0060440; P:GO:0070849; P:GO:0043627; P:GO:0045727;	[illegible]
Acrdi.XM_015901491.1/1-924 | Monca.adi2mcaRNA10777_R0/1-987	0.524479	0.0110394	THAP domain-containing 6			
Acrdi.XM_015924248.1/1-1002 | Monca.augustus.g24323.t1/1-1005	0.269007	0.0109415	tripartite motif-containing 16-like			
Acrdi.XM_015902981.1/1-1260 | Monca.augustus.g600.t1/1-1257	0.014523	0.0109395	mothers against decapentaplegic homolog 3	81	P:GO:0007050; P:GO:0007492; F:GO:0000978; P:GO:0007179;	[illegible]
Acrdi.XM_015914830.1/1-2820 | Monca.adi2mcaRNA2721_R0/1-2676	0.0179487	0.0108783	exportin-1 isoform X1			
Acrdi.XM_015905714.1/1-498 | Monca.augustus.g1927.t1/1-498	0.0131738	0.0107162	60S ribosomal L12-like	5	F:GO:0003735; C:GO:0022625; F:GO:0019843; P:GO:0000027;	[illegible]
Acrdi.XM_015920678.1/1-900 | Monca.augustus.g32651.t1/1-933	0.0201912	0.0106631	prohibitin-2 isoform X1	1	C:GO:0016020	C:membrane
Acrdi.XM_015892027.1/1-402 | Monca.augustus.g4881.t1/1-402	1.20873	0.0106534	G2 M phase-specific E3 ubiquitin- ligase-like			
Acrdi.XM_015895290.1/1-2130 | Monca.augustus.g26320.t1/1-2130	1.20764	0.0105824	zinc finger 862-like			
Acrdi.XM_015921065.1/1-945 | Monca.augustus.g34992.t1/1-1020	0.0147767	0.0105745	DNA repair RAD51 homolog 1	7	F:GO:0005524; F:GO:0008094; F:GO:0000150; P:GO:1990426;	[illegible]
Acrdi.XM_015915870.1/1-918 | Monca.augustus.g20433.t1/1-918	1.23996	0.0105088	F-box LRR-repeat 14			
Acrdi.XM_015921522.1/1-1062 | Monca.augustus.g22999.t1/1-1062	0.0198281	0.0104463	guanine nucleotide-binding G(o) subunit alpha isoform X1	15	C:GO:0043209; P:GO:0007212; P:GO:0008016; P:GO:0007188;	[illegible]
Acrdi.XM_015917007.1/1-558 | Monca.augustus.g50848.t1/1-549	0.0138165	0.0104243	tyrosine phosphatase type IVA 2-like	10	C:GO:0005634; C:GO:0005769; F:GO:0004725; P:GO:0007275;	[illegible]
Acrdi.XM_015891668.1/1-1047 | Monca.augustus.g34619.t1/1-1047	0.0126136	0.0102088	DDB1- and CUL4-associated factor 7	3	C:GO:0005634; F:GO:0005515; P:GO:0051216	C:nucleus; F:protein binding; P:cartilage development
Acrdi.XM_015891941.1/1-771 | Monca.adi2mcaRNA37822_R4/1-714	0.267663	0.010058	RNA-directed DNA polymerase from mobile element jockey-like			
Acrdi.XM_015906675.1/1-2148 | Monca.augustus.g60586.t1/1-2583	0.0164541	0.0100504	anoctamin-7-like isoform X1	2	C:GO:0016021; F:GO:0046983	C:integral component of membrane; F:protein dimerization activity
Acrdi.XM_015912534.1/1-642 | Monca.augustus.g26724.t1/1-642	0.393432	0.00987878	P2X purinoceptor 7-like			
Acrdi.XM_015907119.1/1-405 | Monca.augustus.g36974.t1/1-594	0.0220493	0.0098618	ras-related R-Ras2	4	F:GO:0005525; P:GO:0007264; C:GO:0005622; C:GO:0005886	[illegible]
Acrdi.XM_015904971.1/1-951 | Monca.augustus.g261.t1/1-1305	0.250627	0.00985664	serine threonine- kinase	3	F:GO:0005524; F:GO:0004672; P:GO:0006468	F:ATP binding; F:protein kinase activity; P:protein phosphorylation
Acrdi.XM_015903711.1/1-972 | Monca.augustus.g41346.t1/1-1047	0.0155411	0.00984283	guanine nucleotide-binding G(I) G(S) G(T) subunit beta-1	3	F:GO:0004402; P:GO:0007165; P:GO:0016573	[illegible]
Acrdi.XM_015898074.1/1-675 | Monca.augustus.g2720.t1/1-969	0.00994899	0.00969042	vacuolar sorting-associated 26B-like	6	C:GO:0005768; C:GO:0030904; C:GO:0005829; F:GO:0008565;	[illegible]
Acrdi.XM_015898333.1/1-606 | Monca.augustus.g51104.t1/1-606	0.0165363	0.00968353	glucose-induced degradation 4 homolog	3	P:GO:0008150; C:GO:0005575; F:GO:0003674	P:biological_process; C:cellular_component; F:molecular_function
Acrdi.XM_015924352.1/1-543 | Monca.augustus.g32417.t1/1-1989	0.0184466	0.00939404	cyclin-T1 isoform X2	15	F:GO:0019901; P:GO:0045737; F:GO:0016538; P:GO:0006351;	[illegible]
Acrdi.XM_015898883.1/1-597 | Monca.augustus.g5115.t1/1-597	0.0122586	0.00916906	ras-related Rab-10	5	F:GO:0005525; P:GO:0032869; C:GO:0032593; P:GO:0007264;	[illegible]
Acrdi.XM_015895743.1/1-1065 | Monca.adi2mcaRNA4641_R0/1-960	0.821205	0.00909697	PREDICTED: uncharacterized protein LOC107331191			
Acrdi.XM_015913910.1/1-711 | Monca.augustus.g66934.t1/1-708	0.0189846	0.00906711	Homeobox SIX3	7	C:GO:0005634; F:GO:0003677; P:GO:0001654; P:GO:0007420;	[illegible]
Acrdi.XM_015907702.1/1-486 | Monca.augustus.g14805.t1/1-684	0.0240521	0.00893802	cleavage and polyadenylation specificity factor subunit 5	10	F:GO:0017091; C:GO:0005813; F:GO:0042803; F:GO:0042826;	[illegible]
Acrdi.XM_015914350.1/1-882 | Monca.adi2mcaRNA24873_R5/1-585	1.17145	0.00888483	PREDICTED: uncharacterized protein LOC107348322	7	F:GO:0003677; P:GO:0090305; C:GO:0005634; F:GO:0008270;	[illegible]

Acrdi.XM_015909640.1/1-1302	Monca.augustus.g53335.t1/1-1302	0.0105809	0.00879721	26S protease regulatory subunit 7	31	C:GO:0008540; P:GO:0038095; C:GO:0000932; P:GO:0043488;	C:proteasome regulatory particle, base subcomplex; P:Fc-epsilon receptor signaling pathway; C:P-body; P:regulation of mRNA stability; P:positive regulation of proteasomal p	EC:3.6.1.3; EC:3.6.1.15	Adenosinetriphosphatase; Nucleoside-triphosphate phosphatase
Acrdi.XM_015923083.1/1-1089	Monca.augustus.g70883.t1/1-1089	0.0136122	0.00878552	phosphoribosyl pyrophosphate synthase-associated 2	6	F:GO:0000287; P:GO:0009165; F:GO:0004749; C:GO:0016021;	F:magnesium ion binding; P:nucleotide biosynthetic process; F:ribose phosphate diphosphokinase activity; C:integral component of membrane; P:oxidation-reduction process;	EC:2.7.6.1	Ribose-phosphate diphosphokinase
Acrdi.XM_015902458.1/1-1044	Monca.augustus.g36352.t1/1-1044	0.00825199	0.0087569	Cyclin-dependent kinase-like 1	3	F:GO:0005524; F:GO:0004674; P:GO:0006468	F:ATP binding; F:protein serine/threonine kinase activity; P:protein phosphorylation	EC:2.7.11	Transferring phosphorus-containing groups
Acrdi.XM_015909167.1/1-954	Monca.adi2mcaRNA19209_R0/1-978	0.328738	0.00869166	PREDICTED: uncharacterized protein LOC107343583 isoform X2	4	P:GO:0007154; C:GO:0016021; P:GO:0051716; P:GO:0044700	P:cell communication; C:integral component of membrane; P:cellular response to stimulus; P:single organism signaling		
Acrdi.XM_015895781.1/1-1329	Monca.augustus.g47029.t1/1-1329	0.00849712	0.00867259	COP9 signalosome complex subunit 2	6	P:GO:0008283; P:GO:0035914; P:GO:0045892; C:GO:0005737;	P:cell proliferation; P:skeletal muscle cell differentiation; P:negative regulation of transcription, DNA-templated; C:cytoplasm; F:transcription corepressor activity; C:COP9 signalosome		
Acrdi.XM_015921084.1/1-633	Monca.augustus.g27609.t1/1-633	0.0171463	0.00864192	ras-related Rab-4B	6	F:GO:0005525; C:GO:0005768; C:GO:0032593; P:GO:0007264;	F:GTP binding; C:endosome; C:insulin-responsive compartment; P:small GTPase mediated signal transduction; P:regulation of endocytosis; F:hydrolase activity		
Acrdi.XM_015892197.1/1-840	Monca.augustus.g68066.t1/1-840	0.196228	0.00857737	enoyl- hydratase	2	P:GO:0009062; F:GO:0016853	P:fatty acid catabolic process; F:isomerase activity		
Acrdi.XM_015893004.1/1-1074	Monca.augustus.g17712.t1/1-1074	0.191961	0.00854008	Ankyrin repeat domain-containing 34B					
Acrdi.XM_015912943.1/1-504	Monca.augustus.g60137.t1/1-504	0.0141669	0.00836535	Centrin-3	1	F:GO:0005509	F:calcium ion binding		
Acrdi.XM_015918716.1/1-480	Monca.augustus.g71012.t1/1-2829	0.0131764	0.00836287	structural maintenance of chromosomes 4-like	4	C:GO:0005634; F:GO:0005524; P:GO:0051276; C:GO:0005694	C:nucleus; F:ATP binding; P:chromosome organization; C:chromosome		
Acrdi.XM_015923501.1/1-717	Monca.adi2mcaRNA35177_R0/1-723	0.0162224	0.00828809	cell differentiation RCD1 homolog	17	F:GO:0019904; F:GO:0042803; P:GO:0006351; C:GO:0000932;	F:protein domain specific binding; F:protein homodimerization activity; P:transcription, DNA-templated; C:P-body; P:regulation of transcription, DNA-templated; P:nuclear-transcribed mRNA poly(A) tail shortening; C:nucleus; P:sex differentiation; P:DNA damage response, signal transduction by p53 class mediator resulting in cell cycle arrest; P:positive regulation of ligand-dependent nuclear receptor transcription coactivator activity; P:cytokine-mediated signaling pathway; C:membrane; C:cytosol; P:gene silencing by RNA; P:negative regulation of intracellular estrogen receptor signaling pathway; C:CCR4-NOT core complex; P:negative regulation of translation		
Acrdi.XM_015919037.1/1-462	Monca.augustus.g32029.t1/1-462	0.0107758	0.0081228	Ubiquitin-conjugating enzyme E2 L3	1	F:GO:0005524	F:ATP binding		
Acrdi.XM_015905304.1/1-993	Monca.augustus.g23104.t1/1-993	0.0220489	0.0081193	POU class transcription factor 3	8	C:GO:0005634; F:GO:0003700; P:GO:0060113; P:GO:0006351;	C:nucleus; F:transcription factor activity, sequence-specific DNA binding; P:inner ear receptor cell differentiation; P:transcription, DNA-templated; P:axonogenesis; F:sequence-specific DNA binding; P:neurological system process; P:regulation of transcription from RNA polymerase II promoter		
Acrdi.XM_015898725.1/1-675	Monca.augustus.g2532.t1/1-855	0.012161	0.00800255	succinate dehydrogenase [ubiquinone] iron-sulfur mitochondrial	9	C:GO:0005749; F:GO:0008177; F:GO:0046872; F:GO:0009055;	C:mitochondrial respiratory chain complex II, succinate dehydrogenase complex (ubiquinone); F:succinate dehydrogenase (ubiquinone) activity; F:metal ion binding; F:electror	EC:1.3.99.1; EC:1.3.5.1	Deleted entry; Succinate dehydrogenase (quinone)
Acrdi.XM_015899061.1/1-546	Monca.adi2mcaRNA8109_R0/1-546	0.39369	0.00798604	Transposable element P transposase					
Acrdi.XM_015921252.1/1-1212	Monca.adi2mcaRNA32600_R0/1-1551	0.013623	0.00787325	Adenosylhomocysteinase 3	4	P:GO:0033353; C:GO:0005829; F:GO:0004013; P:GO:0006730	P:S-adenosylmethionine cycle; C:cytosol; F:adenosylhomocysteinase activity; P:one-carbon metabolic process	EC:3.3.1.1	Adenosylhomocysteinase
Acrdi.XM_015924496.1/1-771	Monca.augustus.g31183.t1/1-2595	0.0113277	0.0078259	NEDD4-like E3 ubiquitin- ligase WWP1	5	C:GO:0005634; F:GO:0004842; C:GO:0005737; F:GO:0016874;	C:nucleus; F:ubiquitin-protein transferase activity; C:cytoplasm; F:ligase activity; P:protein ubiquitination involved in ubiquitin-dependent protein catabolic process		
Acrdi.XM_015924305.1/1-789	Monca.augustus.g63506.t1/1-2376	0.429624	0.00772179	PREDICTED: uncharacterized protein LOC107357665					
Acrdi.XM_015900935.1/1-351	Monca.augustus.g27888.t1/1-351	0.00980533	0.00762124	gamma-aminobutyric acid receptor-associated -like 2	5	P:GO:0000045; C:GO:0005829; P:GO:0000422; C:GO:0000421;	P:autophagosome assembly; C:cytosol; P:mitophagy; C:autophagosome membrane; P:cellular response to nitrogen starvation		
Acrdi.XM_015920769.1/1-369	Monca.augustus.g35961.t1/1-369	0.0089715	0.00754508	Splicing factor 3B subunit 6	11	F:GO:0000166; F:GO:0003676; C:GO:0005689; C:GO:0071013;	F:nucleotide binding; F:nucleic acid binding; C:U12-type spliceosomal complex; C:catalytic step 2 spliceosome; C:precatalytic spliceosome; C:integral component of membrane; C:U2-type spliceosomal complex; C:U2 snRNP; F:transferase activity; P:fatty acid biosynthetic process; P:mRNA splicing, via spliceosome		
Acrdi.XM_015895715.1/1-549	Monca.augustus.g17451.t1/1-555	0.0136623	0.00742632	ADP-ribosylation factor 8B-A	3	F:GO:0005525; P:GO:0007264; C:GO:0005622	F:GTP binding; P:small GTPase mediated signal transduction; C:intracellular		
Acrdi.XM_015917352.1/1-4503	Monca.adi2mcaRNA28282_R6/1-4653	1.15509	0.00716867	PREDICTED: uncharacterized protein LOC107351088					
Acrdi.XM_015923267.1/1-609	Monca.adi2mcaRNA34909_R0/1-612	0.00720796	0.00699698	homeobox otx5-A-like	6	C:GO:0005634; P:GO:0042706; P:GO:0009952; F:GO:0043565;	C:nucleus; P:eye photoreceptor cell fate commitment; P:anterior/posterior pattern specification; F:sequence-specific DNA binding; P:positive regulation of rhodopsin gene expression; P:positive regulation of transcription from RNA polymerase II promoter		
Acrdi.XM_015892521.1/1-1614	Monca.augustus.g47456.t1/1-807	0.124626	0.00693913	PREDICTED: uncharacterized protein LOC107327773					
Acrdi.XM_015895784.1/1-1209	Monca.augustus.g47027.t1/1-1209	0.0113243	0.00692627	eukaryotic initiation factor 4A-III	7	F:GO:0003676; F:GO:0005524; P:GO:0008380; C:GO:0071013;	F:nucleic acid binding; F:ATP binding; P:RNA splicing; C:catalytic step 2 spliceosome; P:regulation of gene expression; P:RNA secondary structure unwinding; F:ATP-dependent	EC:3.6.1.3; EC:3.6.1.15	Adenosinetriphosphatase; Nucleoside-triphosphate phosphatase
Acrdi.XM_015903780.1/1-1176	Monca.augustus.g32291.t1/1-1176	0.0116352	0.00682456	26S protease regulatory subunit 10B	10	F:GO:0005524; C:GO:0008540; C:GO:0031597; C:GO:0031595;	F:ATP binding; C:proteasome regulatory particle, base subcomplex; C:cytosolic proteasome complex; C:nuclear proteasome complex; F:peptidase activity; F:TBP-class protein	EC:3.6.1.3; EC:3.6.1.15	Adenosinetriphosphatase; Nucleoside-triphosphate phosphatase
Acrdi.XM_015901963.1/1-393	Monca.augustus.g45540.t1/1-393	0.00858054	0.00682041	Small nuclear ribonucleo Sm D3	31	P:GO:0051170; C:GO:0005697; P:GO:0006369; C:GO:0005654;	P:nuclear import; C:telomerase holoenzyme complex; P:termination of RNA polymerase II transcription; C:nucleoplasm; C:commitment complex; C:U12-type spliceosomal complex; C:prespliceosome; C:extracellular exosome; C:catalytic step 2 spliceosome; F:U1 snRNP binding; C:methylosome; C:precatalytic spliceosome; C:spliceosomal tri-snRNP complex; C:cytosol; F:U2 snRNA binding; C:U5 snRNP; P:histone mRNA metabolic process; C:U7 snRNP; C:U2 snRNP; P:protein methylation; C:U1 snRNP; P:spliceosomal complex assembly; C:U4 snRNP; P:spliceosomal snRNP assembly; C:pICln-Sm protein complex; F:telomerase RNA binding; C:SMN-Sm protein complex; C:viral nucleocapsid; F:histone pre-mRNA DCP binding; F:U7 snRNA binding; F:enzyme binding		
Acrdi.XM_015924549.1/1-1398	Monca.augustus.g26869.t1/1-1320	0.00771778	0.00676467	26S protease regulatory subunit 4	7	F:GO:0016887; F:GO:0005524; P:GO:0043161; C:GO:0008540;	F:ATPase activity; F:ATP binding; P:proteasome-mediated ubiquitin-dependent protein catabolic process; C:proteasome regulatory particle, base subcomplex; C:cytoplasm; F:	EC:3.6.1.3; EC:3.6.1.15	Adenosinetriphosphatase; Nucleoside-triphosphate phosphatase
Acrdi.XM_015894674.1/1-1956	Monca.augustus.g35358.t1/1-3117	0.92229	0.00655913	zinc finger 862-like					
Acrdi.XM_015894168.1/1-447	Monca.augustus.g69056.t1/1-414	0.0103927	0.00618697	trafficking particle complex subunit 2	2	P:GO:0006888; C:GO:0005737	P:ER to Golgi vesicle-mediated transport; C:cytoplasm		
Acrdi.XM_015923979.1/1-441	Monca.augustus.g1833.t1/1-441	0.0142342	0.0060744	ubiquitin-conjugating enzyme E2-17 kDa	25	F:GO:0061630; F:GO:0061631; P:GO:0040035; P:GO:0009792;	F:ubiquitin protein ligase activity; F:ubiquitin conjugating enzyme activity; P:hermaphrodite genitalia development; P:embryo development ending in birth or egg hatching; C:microtubule associated complex; F:ATP binding; P:nematode larval development; P:apoptotic process; P:cell migration; P:spermatid development; P:protein polyubiquitination; P:male meiosis; P:regulation of R7 cell differentiation; P:mitotic nuclear division; P:protein ubiquitination involved in ubiquitin-dependent protein catabolic process; P:transposition, DNA-mediated; P:compound eye photoreceptor cell differentiation; P:female germ-line stem cell asymmetric division; F:ubiquitin protein ligase binding; P:regulation of protein stability; F:transposase activity; P:chromosome organization; P:germ-line stem cell population maintenance; P:anaphase-promoting complex-dependent catabolic process; P:neuron remodeling		
Acrdi.XM_015906451.1/1-450	Monca.augustus.g9540.t1/1-450	0.00677658	0.00606668	DNA-directed RNA polymerases and III subunit RPABC3	24	P:GO:0045815; P:GO:0006361; P:GO:0006383; P:GO:0006362;	P:positive regulation of gene expression, epigenetic; P:transcription initiation from RNA polymerase I promoter; P:transcription from RNA polymerase III promoter; P:transcri	EC:2.7.7.6	DNA-directed RNA polymerase
Acrdi.XM_015898394.1/1-474	Monca.augustus.g17850.t1/1-474	0.353277	0.00605499	PREDICTED: uncharacterized protein LOC107333563					
Acrdi.XM_015891967.1/1-1341	Monca.augustus.g41435.t1/1-1341	0.00688421	0.00603408	tubulin beta-4B chain	6	F:GO:0005525; C:GO:0005737; F:GO:0003924; F:GO:0005200;	F:GTP binding; C:cytoplasm; F:GTPase activity; F:structural constituent of cytoskeleton; C:microtubule; P:microtubule-based process	EC:3.6.1.15	Nucleoside-triphosphate phosphatase
Acrdi.XM_015924296.1/1-6270	Monca.adi2mcaRNA36063_R2/1-6270	0.454495	0.0058636	PREDICTED: uncharacterized protein LOC107357656					
Acrdi.XM_015914227.1/1-504	Monca.augustus.g50930.t1/1-504	0.0103521	0.00582963	actin-related 2 3 complex subunit 4	9	F:GO:0051015; C:GO:0070062; C:GO:0005737; P:GO:0030041;	F:actin filament binding; C:extracellular exosome; C:cytoplasm; P:actin filament polymerization; P:Arp2/3 complex-mediated actin nucleation; F:structural constituent of cytoskeleton; F:protein binding, bridging; C:Arp2/3 protein complex; F:enzyme binding		
Acrdi.XM_015903412.1/1-516	Monca.augustus.g35993.t1/1-516	0.00613909	0.00548754	DNA-directed RNA polymerase II subunit RPB7	20	P:GO:0045948; P:GO:0006370; P:GO:0006283; C:GO:0000932;	P:positive regulation of translational initiation; P:7-methylguanosine mRNA capping; P:transcription-coupled nucleotide-excision repair; C:P-body; P:fibroblast growth factor n	EC:2.7.7.6	DNA-directed RNA polymerase
Acrdi.XM_015904978.1/1-1050	Monca.augustus.g68302.t1/1-1056	1.58037	0.00523068	trihelix transcription factor ASIL1-like					
Acrdi.XM_015922375.1/1-1215	Monca.augustus.g63529.t1/1-1575	1.21764	0.00517341	ATP-dependent DNA helicase	2	P:GO:0006259; F:GO:0016787	P:DNA metabolic process; F:hydrolase activity		
Acrdi.XM_015905664.1/1-513	Monca.augustus.g1926.t1/1-702	0.00638466	0.00512662	U1 small nuclear ribonucleo A	4	F:GO:0030619; F:GO:0035614; C:GO:0005685; P:GO:0000398	F:U1 snRNA binding; F:snRNA stem-loop binding; C:U1 snRNP; P:mRNA splicing, via spliceosome		
Acrdi.XM_015903363.1/1-354	Monca.augustus.g4659.t1/1-921	0.00690827	0.00505604	presenilin-1-like					
Acrdi.XM_015922858.1/1-579	Monca.augustus.g31191.t1/1-579	0.010351	0.00498491	cilia- and flagella-associated 20	11	C:GO:0031514; P:GO:0018095; C:GO:0070062; P:GO:0060271;	C:motile cilium; P:protein polyglutamylation; C:extracellular exosome; P:cilium assembly; C:centriole; C:ciliary basal body; P:regulation of cilium beat frequency involved in ciliary motility; P:positive regulation of feeding behavior; C:nucleoplasm; C:microtubule; P:positive regulation of cell motility		
Acrdi.XM_015904813.1/1-1137	Monca.augustus.g29901.t1/1-1293	0.00747422	0.00492022	26S protease regulatory subunit 8	38	C:GO:0008540; P:GO:0038095; P:GO:0006366; P:GO:0043488;	C:proteasome regulatory particle, base subcomplex; P:Fc-epsilon receptor signaling pathway; P:transcription from RNA polymerase II promoter; P:regulation of mRNA stabili	EC:3.6.1.3; EC:3.6.1.15	Adenosinetriphosphatase; Nucleoside-triphosphate phosphatase
Acrdi.XM_015914985.1/1-1383	Monca.augustus.g42436.t1/1-1383	0.00885416	0.00472499	Tubulin gamma-1 chain	19	C:GO:0031252; C:GO:0036064; P:GO:0007020; F:GO:0005200;	C:cell leading edge; C:ciliary basal body; P:microtubule nucleation; F:structural constituent of cytoskeleton; C:gamma-tubulin complex; C:cytoplasmic microtubule; P:meiotic s	EC:3.6.1.15	Nucleoside-triphosphate phosphatase
Acrdi.XM_015903424.1/1-609	Monca.augustus.g18170.t1/1-609	0.00673755	0.00462462	ras-related Rab-1A	29	P:GO:0090557; P:GO:1903020; P:GO:0007030; P:GO:0090110;	P:establishment of endothelial intestinal barrier; P:positive regulation of glycoprotein metabolic process; P:Golgi organization; P:cargo loading into COPII-coated vesicle; C:Go	EC:3.6.1.15	Nucleoside-triphosphate phosphatase
Acrdi.XM_015896363.1/1-534	Monca.augustus.g54401.t1/1-534	0.00756487	0.0045264	ADP-ribosylation factor 5B	3	F:GO:0005525; P:GO:0007264; C:GO:0005622	F:GTP binding; P:small GTPase mediated signal transduction; C:intracellular		
Acrdi.XM_015919209.1/1-939	Monca.augustus.g54944.t1/1-987	0.00766783	0.00448806	serine threonine- phosphatase 2A catalytic subunit beta isoform	2	F:GO:0004721; P:GO:0006470	F:phosphoprotein phosphatase activity; P:protein dephosphorylation	EC:3.1.3.16; EC:3.1.3.41	Protein-serine/threonine phosphatase; 4-nitrophenylphosphatase
Acrdi.XM_015916826.1/1-1248	Monca.augustus.g14615.t1/1-1248	0.114316	0.00443248	PREDICTED: uncharacterized protein LOC107350583					
Acrdi.XM_015908431.1/1-2283	Monca.augustus.g53299.t1/1-2559	0.804273	0.00431504	zinc finger 862-like					
Acrdi.XM_015921093.1/1-987	Monca.adi2mcaRNA32413_R2/1-987	0.323704	0.00404665	52 kDa repressor of the inhibitor of the kinase-like					
Acrdi.XM_015916827.1/1-1062	Monca.adi2mcaRNA33626_R3/1-1371	0.231137	0.00379014	nuclease HARBI1	1	F:GO:0003676	F:nucleic acid binding		
Acrdi.XM_015898301.1/1-330	Monca.augustus.g61845.t1/1-330	0.00493567	0.00362299	dynein light chain axonemal	2	C:GO:0030286; P:GO:0007017	C:dynein complex; P:microtubule-based process		
Acrdi.XM_015913704.1/1-774	Monca.augustus.g21650.t1/1-927	0.00534051	0.00360892	serine threonine- phosphatase 4 catalytic subunit isoform X2	6	F:GO:0004722; C:GO:0005634; C:GO:0005737; P:GO:0006470;	F:protein serine/threonine phosphatase activity; C:nucleus; C:cytoplasm; P:protein dephosphorylation; P:regulation of double-strand break repair via homologous recombinati	EC:3.1.3.16; EC:3.1.3.41	Protein-serine/threonine phosphatase; 4-nitrophenylphosphatase
Acrdi.XM_015910968.1/1-468	Monca.augustus.g3076.t1/1-387	0.00621432	0.00346873	ubiquitin-60S ribosomal L40	7	F:GO:0003735; P:GO:0002181; C:GO:0022625; F:GO:0031386;	F:structural constituent of ribosome; P:cytoplasmic translation; C:cytosolic large ribosomal subunit; F:protein tag; C:microtubule associated complex; P:ubiquitin-dependent protein catabolic process; P:protein ubiquitination		
Acrdi.XM_015901590.1/1-426	Monca.augustus.g55982.t1/1-426	0.00475242	0.00344033	RING finger 11	1	F:GO:0008270	F:zinc ion binding		
Acrdi.XM_015915868.1/1-1623	Monca.adi2mcaRNA26580_R4/1-1623	0.34385	0.0029471	PREDICTED: uncharacterized protein LOC107349688	2	C:GO:0016020; C:GO:0016021	C:membrane; C:integral component of membrane		
Acrdi.XM_015913735.1/1-987	Monca.augustus.g21658.t1/1-987	0.00399124	0.00276215	serine threonine- phosphatase alpha-2 isoform	3	F:GO:0004721; F:GO:0046872; P:GO:0006470	F:phosphoprotein phosphatase activity; F:metal ion binding; P:protein dephosphorylation	EC:3.1.3.16; EC:3.1.3.41	Protein-serine/threonine phosphatase; 4-nitrophenylphosphatase
Acrdi.XM_015904589.1/1-942	Monca.augustus.g43005.t1/1-942	0.15435	0.00271481	G2 M phase-specific E3 ubiquitin- ligase					
Acrdi.XM_015905569.1/1-456	Monca.augustus.g24090.t1/1-456	0.00287084	0.00266455	ubiquitin-conjugating enzyme E2-17 kDa isoform X1	11	F:GO:0005524; P:GO:0006281; F:GO:0061630; P:GO:0043161;	F:ATP binding; P:DNA repair; F:ubiquitin protein ligase activity; P:proteasome-mediated ubiquitin-dependent protein catabolic process; C:nuclear chromatin; F:ubiquitin protein ligase binding; C:cytoplasm; P:protein polyubiquitination; P:histone ubiquitination; C:HULC complex; F:ligase activity		
Acrdi.XM_015904878.1/1-1092	Monca.augustus.g29904.t1/1-1395	0.00300871	0.00259957	speckle-type POZ -like					
Acrdi.XM_015915150.1/1-513	Monca.augustus.g39762.t1/1-513	0.00304533	0.00235564	caltractin isoform X1	1	F:GO:0005509	F:calcium ion binding		
Acrdi.XM_015914988.1/1-1695	Monca.augustus.g24367.t1/1-1695	0.0598035	0.00231629	Inhibitor of growth 3					
Acrdi.XM_015909477.1/1-636	Monca.augustus.g35542.t1/1-636	0.00486342	0.0020712	ras-related Rab-2	4	F:GO:0005525; F:GO:0004767; P:GO:0007264; C:GO:0005622	F:GTP binding; F:sphingomyelin phosphodiesterase activity; P:small GTPase mediated signal transduction; C:intracellular	EC:3.1.4.12	Sphingomyelin phosphodiesterase
Acrdi.XM_015910299.1/1-852	Monca.augustus.g60387.t1/1-1656	0.0027285	0.00164544	ATP synthase subunit mitochondrial	6	C:GO:0045261; F:GO:0005524; P:GO:0015991; F:GO:0046961;	C:proton-transporting ATP synthase complex, catalytic core F(1); F:ATP binding; P:ATP hydrolysis coupled proton transport; F:proton-transporting ATPase activity, rotational m	EC:3.6.1.3; EC:3.6.1.15	Adenosinetriphosphatase; Nucleoside-triphosphate phosphatase
Acrdi.XM_015901872.1/1-324	Monca.augustus.g56965.t1/1-324	0.001	0.0016277	DNA-directed RNA polymerase III subunit RPC10	6	F:GO:0003676; F:GO:0001056; F:GO:0008270; P:GO:0006386;	F:nucleic acid binding; F:RNA polymerase III activity; F:zinc ion binding; P:termination of RNA polymerase III transcription; C:nucleolus; C:DNA-directed RNA polymerase III cor	EC:2.7.7.6	DNA-directed RNA polymerase
Acrdi.XM_015919255.1/1-822	Monca.augustus.g1060.t1/1-2151	0.261066	0.00155833	G2 M phase-specific E3 ubiquitin- ligase-like					
Acrdi.XM_015924150.1/1-1074	Monca.augustus.g62390.t1/1-1317	0.00223394	0.00141816	AP-2 complex subunit mu isoform X2	22	P:GO:1903077; C:GO:0036020; C:GO:0030141; F:GO:0008289;	P:negative regulation of protein localization to plasma membrane; C:endolysosome membrane; C:secretory granule; F:lipid binding; F:signal sequence binding; P:intracellular protein transport; P:microtubule-based movement; P:negative regulation of epidermal growth factor receptor signaling pathway; F:ion channel binding; F:transporter activity; P:clathrin-dependent endocytosis; C:extracellular exosome; F:low-density lipoprotein particle receptor binding; C:terminal bouton; P:regulation of defense response to virus by virus; C:mitochondrion; P:ephrin receptor signaling pathway; C:cytosol; F:clathrin adaptor activity; P:Wnt signaling pathway, planar cell polarity pathway; C:AP-2 adaptor complex; P:antigen processing and presentation of exogenous peptide antigen via MHC class II		
Acrdi.XM_015908149.1/1-3138	Monca.adi2mcaRNA18105_R0/1-3138	0.217039	0.00118972	Chromatin modification-related YNG2					
Acrdi.XM_015909185.1/1-447	Monca.augustus.g44605.t1/1-447	0.001	0.00107316	calmodulin	31	P:GO:0046716; P:GO:0007099; P:GO:0006468; C:GO:0005875;	P:muscle cell cellular homeostasis; P:centriole replication; P:protein phosphorylation; C:microtubule associated complex; C:nucleoplasm; P:sensory perception of sound; F:calcium ion binding; P:kinetochore organization; P:sensory perception of smell; C:myosin V complex; C:cytosol; C:myosin VI complex; P:actin filament-based movement; P:regulation of response to DNA damage stimulus; P:salivary gland cell autophagic cell death; C:plasma membrane; P:positive regulation of NFAT protein import into nucleus; C:mitotic spindle pole; P:metarhodopsin inactivation; P:regulation of light-activated channel activity; P:photoreceptor cell axon guidance; C:centriole; P:adaptation of rhodopsin mediated signaling; P:rhabdomere development; P:lateral inhibition; P:positive regulation of cation channel activity; C:midbody; F:kinase activity; C:rhabdomere; F:myosin V binding; F:myosin VI head/neck binding		
Acrdi.XM_015899007.1/1-4434	Monca.augustus.g7727.t1/1-4434	0.452877	0.000880035	Retrovirus-related Pol poly from type-2 retrotransposable element R2DM					
Acrdi.XM_015896425.1/1-360	Monca.augustus.g69680.t1/1-354	0.001	0.00069935	gamma-aminobutyric acid receptor-associated	5	P:GO:0000045; C:GO:0005829; P:GO:0000422; C:GO:0000421;	P:autophagosome assembly; C:cytosol; P:mitophagy; C:autophagosome membrane; P:cellular response to nitrogen starvation		
Acrdi.XM_015898773.1/1-339	Monca.augustus.g29487.t1/1-453	0.001	0.000600851	dynein light chain Tctex-type 1	4	P:GO:0019060; F:GO:0042802; C:GO:0005622; C:GO:0043657	P:intracellular transport of viral protein in host cell; F:identical protein binding; C:intracellular; C:host cell		
Acrdi.XM_015897468.1/1-501	Monca.augustus.g7252.t1/1-540	0.001	0.000340802	NEDD8-conjugating enzyme Ubc12	12	F:GO:0005524; F:GO:0061630; C:GO:0070062; F:GO:0031625;	F:ATP binding; F:ubiquitin protein ligase activity; C:extracellular exosome; F:ubiquitin protein ligase binding; P:transforming growth factor beta receptor signaling pathway; C:cytosol; P:NIK/NF-kappaB signaling; P:protein neddylation; F:ribosomal S6-glutamic acid ligase activity; F:NEDD8 transferase activity; P:positive regulation of neuron apoptotic process; P:protein ubiquitination		

Metabolism	
**Global and overview maps**	**26**
01100 Metabolic pathways	14
01110 Biosynthesis of secondary metabolites	5
01120 Microbial metabolism in diverse environments	1
01130 Biosynthesis of antibiotics	3
01200 Carbon metabolism	1
01230 Biosynthesis of amino acids	2
**Carbohydrate metabolism**	**2**
00010 Glycolysis / Gluconeogenesis	1
00520 Amino sugar and nucleotide sugar metabolism	1
**Energy metabolism**	**2**
00680 Methane metabolism	1
00910 Nitrogen metabolism	1
**Lipid metabolism**	**11**
00564 Glycerophospholipid metabolism	2
00565 Ether lipid metabolism	2
00590 Arachidonic acid metabolism	3
00591 Linoleic acid metabolism	2
00592 alpha-Linolenic acid metabolism	2
**Nucleotide metabolism**	**2**
00230 Purine metabolism	2
**Amino acid metabolism**	**5**
00260 Glycine, serine and threonine metabolism	1
00310 Lysine degradation	2
00220 Arginine biosynthesis	1
00330 Arginine and proline metabolism	1
**Metabolism of other amino acids**	**1**
00480 Glutathione metabolism	1
**Glycan biosynthesis and metabolism**	**10**
00513 Various types of N-glycan biosynthesis	2
00531 Glycosaminoglycan degradation	2
00563 Glycosylphosphatidylinositol GPI-anchor biosynt	2

00601 Glycosphingolipid biosynthesis - lacto and neola	1
00603 Glycosphingolipid biosynthesis - globo and isogl	1
00604 Glycosphingolipid biosynthesis - ganglio series	1
00511 Other glycan degradation	1
**Metabolism of cofactors and vitamins**	**3**
00770 Pantothenate and CoA biosynthesis	1
00670 One carbon pool by folate	1
00830 Retinol metabolism	1
**Xenobiotics biodegradation and metabolism**	**2**
00980 Metabolism of xenobiotics by cytochrome P450	1
00982 Drug metabolism - cytochrome P450	1
**Genetic Information Processing**	
**Transcription**	**5**
03022 Basal transcription factors	1
03040 Spliceosome	4
**Translation**	**14**
03010 Ribosome	4
03013 RNA transport	7
03015 mRNA surveillance pathway	1
03008 Ribosome biogenesis in eukaryotes	2
**Folding, sorting and degradation**	**6**
04141 Protein processing in endoplasmic reticulum	1
04130 SNARE interactions in vesicular transport	1
04120 Ubiquitin mediated proteolysis	1
03050 Proteasome	1
03018 RNA degradation	2
**Replication and repair**	**9**
03030 DNA replication	2
03410 Base excision repair	2
03420 Nucleotide excision repair	1
03430 Mismatch repair	1
03440 Homologous recombination	1
03460 Fanconi anemia pathway	2

Environmental Information Processing	
**Membrane transport**	**1**
02010 ABC transporters	1
**Signal transduction**	**62**
04151 PI3K-Akt signaling pathway	8
04015 Rap1 signaling pathway	7
04014 Ras signaling pathway	6
04010 MAPK signaling pathway	6
04310 Wnt signaling pathway	3
04330 Notch signaling pathway	3
04066 HIF-1 signaling pathway	3
04024 cAMP signaling pathway	3
04012 ErbB signaling pathway	2
04390 Hippo signaling pathway	2
04370 VEGF signaling pathway	2
04630 Jak-STAT signaling pathway	2
04668 TNF signaling pathway	2
04068 FoxO signaling pathway	2
04020 Calcium signaling pathway	2
04072 Phospholipase D signaling pathway	2
04150 mTOR signaling pathway	2
04013 MAPK signaling pathway - fly	1
04340 Hedgehog signaling pathway	1
04371 Apelin signaling pathway	1
04064 NF-kappa B signaling pathway	1
04022 cGMP-PKG signaling pathway	1
**Signaling molecules and interaction**	**11**
04080 Neuroactive ligand-receptor interaction	4
04060 Cytokine-cytokine receptor interaction	3
04512 ECM-receptor interaction	4
**Cellular Processes**	
**Transport and catabolism**	**21**
04144 Endocytosis	8

04145 Phagosome	2
04142 Lysosome	6
04146 Peroxisome	1
04140 Autophagy - animal	2
04138 Autophagy - yeast	1
04137 Mitophagy - animal	1
**Cell growth and death**	**8**
04110 Cell cycle	1
04210 Apoptosis	1
04214 Apoptosis - fly	1
04215 Apoptosis - multiple species	1
04217 Necroptosis	3
04115 p53 signaling pathway	1
**Cellular community - eukaryotes**	**17**
04510 Focal adhesion	5
04520 Adherens junction	3
04530 Tight junction	3
04540 Gap junction	3
04550 Signaling pathways regulating pluripotency of s	3
**Cell motility**	**6**
04810 Regulation of actin cytoskeleton	6
**Organismal Systems**	
**Immune system**	**18**
04611 Platelet activation	2
04620 Toll-like receptor signaling pathway	1
04621 NOD-like receptor signaling pathway	4
04622 RIG-I-like receptor signaling pathway	1
04623 Cytosolic DNA-sensing pathway	1
04650 Natural killer cell mediated cytotoxicity	1
04658 Th1 and Th2 cell differentiation	2
04657 IL-17 signaling pathway	2
04664 Fc epsilon RI signaling pathway	1
04666 Fc gamma R-mediated phagocytosis	1

04062 Chemokine signaling pathway	2
**Endocrine system**	**17**
04911 Insulin secretion	1
04912 GnRH signaling pathway	2
04913 Ovarian steroidogenesis	1
04915 Estrogen signaling pathway	1
04917 Prolactin signaling pathway	2
04921 Oxytocin signaling pathway	2
04926 Relaxin signaling pathway	2
04919 Thyroid hormone signaling pathway	2
04916 Melanogenesis	1
04924 Renin secretion	1
04925 Aldosterone synthesis and secretion	2
**Circulatory system**	**3**
04270 Vascular smooth muscle contraction	3
**Digestive system**	**7**
04972 Pancreatic secretion	1
04976 Bile secretion	1
04973 Carbohydrate digestion and absorption	1
04974 Protein digestion and absorption	1
04975 Fat digestion and absorption	1
04977 Vitamin digestion and absorption	1
04978 Mineral absorption	1
**Nervous system**	**9**
04724 Glutamatergic synapse	1
04727 GABAergic synapse	1
04725 Cholinergic synapse	1
04728 Dopaminergic synapse	1
04726 Serotonergic synapse	2
04730 Long-term depression	1
04722 Neurotrophin signaling pathway	2
**Sensory system**	**4**
04750 Inflammatory mediator regulation of TRP chan	4

**Development**	**4**
04360 Axon guidance	4
**Aging**	**1**
04211 Longevity regulating pathway	1
**Environmental adaptation**	**3**
04710 Circadian rhythm	1
04711 Circadian rhythm - fly	1
04626 Plant-pathogen interaction	1

GO ID	GO Description / Protein ID	GO Category / NCBI Annotation	P-value	# Test set	# Reference set	# Non-annot Test	# Non-annot Reference	GO Terms
**GO:0005578**	**proteinaceous extracellular matrix GO:0005578**	**CELLULAR_COMPONENT**	**0.006102648**	**10**	**31**	**1212**	**10943**	
	Acrdi.XM_015920238.1	Collagen alpha-6(VI) chain						F:GO:0004867; C:GO:0005581; C:GO:0005576; C:GO:0005578
	Acrdi.XM_015903020.1	metallo ase inhibitor 3-like						P:GO:0034097; P:GO:0010033; C:GO:0005615; P:GO:0043086; P:GO:0
	Acrdi.XM_015912043.1	collectin-12-like isoform X2						C:GO:0016020; C:GO:0016021; C:GO:0005578
	Acrdi.XM_015893569.1	collagen alpha-1(II) chain						C:GO:0005581; F:GO:0005201; C:GO:0005578
	Acrdi.XM_015916799.1	ADAMTS 4						C:GO:0005614; P:GO:0043065; P:GO:0006508; P:GO:0030198; F:GO:0
	Acrdi.XM_015904404.1	nidogen-2 isoform X1						P:GO:0007160; P:GO:0006955; F:GO:0005509; F:GO:0005044; F:GO:0
	Acrdi.XM_015908096.1	Mucin						P:GO:0007160; F:GO:0005509; C:GO:0016020; C:GO:0016021; F:GO:0
	Acrdi.XM_015909298.1	low affinity immunoglobulin epsilon Fc receptor						F:GO:0008270; C:GO:0005581; C:GO:0016020; P:GO:0006508; C:GO:0
	Acrdi.XM_015906432.1	FRAS1-related extracellular matrix 1 isoform X1						P:GO:0007160; C:GO:0005604; P:GO:0007154; F:GO:0005509; C:GO:0
	Acrdi.XM_015912038.1	early endosome antigen 1-like isoform X3						C:GO:0016020; C:GO:0016021; C:GO:0005578
**GO:0008083**	**growth factor activity GO:0008083**	**MOLECULAR_FUNCTION**	**0.006434499**	**6**	**12**	**1216**	**10962**	
	Acrdi.XM_015906470.1	balbiani ring 3-like isoform X1						F:GO:0008083; C:GO:0016020
	Acrdi.XM_015906467.1	balbiani ring 3-like isoform X1						C:GO:0005615; F:GO:0008083; P:GO:0031954; P:GO:0008284; C:GO:0
	Acrdi.XM_015915718.1	trefoil factor 2-like						F:GO:0008083; C:GO:0005576
	Acrdi.XM_015919102.1	balbiani ring 3-like isoform X1						C:GO:0005615; F:GO:0008083; P:GO:0031954; P:GO:0008284; C:GO:0
	Acrdi.XM_015902132.1	bone morphogenetic 2-like						F:GO:0008083; C:GO:0005576
	Acrdi.XM_015923775.1	growth differentiation factor 8						C:GO:0005615; F:GO:0008083; P:GO:0043408; F:GO:0005160; P:GO:0
**GO:0005581**	**collagen trimer GO:0005581**	**CELLULAR_COMPONENT**	**0.008362856**	**4**	**5**	**1218**	**10969**	
	Acrdi.XM_015920238.1	Collagen alpha-6(VI) chain						F:GO:0004867; C:GO:0005581; C:GO:0005576; C:GO:0005578
	Acrdi.XM_015909298.1	low affinity immunoglobulin epsilon Fc receptor						F:GO:0008270; C:GO:0005581; C:GO:0016020; P:GO:0006508; C:GO:0
	Acrdi.XM_015912880.1	secreted containing alpha beta gamma chain						F:GO:0005509; C:GO:0005581; F:GO:0005201
	Acrdi.XM_015893569.1	collagen alpha-1(II) chain						C:GO:0005581; F:GO:0005201; C:GO:0005578
**GO:0000120**	**RNA polymerase I transcription factor complex GO:0000120**	**CELLULAR_COMPONENT**	**0.010032003**	**2**	**0**	**1220**	**10974**	
	Acrdi.XM_015919820.1	PREDICTED: uncharacterized protein LOC107353490						P:GO:0001189; C:GO:0005668; F:GO:0001187; F:GO:0001164; C:GO:0
	Acrdi.XM_015920782.1	TATA box-binding -associated factor RNA polymerase I subunit C						C:GO:0000120; P:GO:0006360
**GO:0007130**	**synaptonemal complex assembly GO:0007130**	**BIOLOGICAL_PROCESS**	**0.028088621**	**2**	**1**	**1220**	**10973**	
	Acrdi.XM_015896073.1	structural maintenance of chromosomes 1A-like						P:GO:0070193; P:GO:0007130; C:GO:0000795
	Acrdi.XM_015910987.1	translationally-controlled tumor homolog						P:GO:0070193; P:GO:0007130; C:GO:0000795
**GO:0019236**	**response to pheromone GO:0019236**	**BIOLOGICAL_PROCESS**	**0.028088621**	**2**	**1**	**1220**	**10973**	
	Acrdi.XM_015914752.1	transmembrane 145-like						P:GO:0007186; C:GO:0016020; C:GO:0016021; P:GO:0019236
	Acrdi.XM_015901918.1	rRNA-processing EFG1						P:GO:0000462; C:GO:0005634; P:GO:0006364; C:GO:0005730; P:GO:0
**GO:0005375**	**copper ion transmembrane transporter activity GO:0005375**	**MOLECULAR_FUNCTION**	**0.028088621**	**2**	**1**	**1220**	**10973**	
	Acrdi.XM_015892057.1	high affinity copper uptake 1						C:GO:0016020; C:GO:0016021; F:GO:0005375; P:GO:0035434
	Acrdi.XM_015897761.1	high affinity copper uptake 1 isoform X2						C:GO:0016020; C:GO:0016021; F:GO:0005375; P:GO:0035434
**GO:0035434**	**copper ion transmembrane transport GO:0035434**	**BIOLOGICAL_PROCESS**	**0.028088621**	**2**	**1**	**1220**	**10973**	
	Acrdi.XM_015892057.1	high affinity copper uptake 1						C:GO:0016020; C:GO:0016021; F:GO:0005375; P:GO:0035434
	Acrdi.XM_015897761.1	high affinity copper uptake 1 isoform X2						C:GO:0016020; C:GO:0016021; F:GO:0005375; P:GO:0035434
**GO:0051731**	**polynucleotide 5'-hydroxyl-kinase activity GO:0051731**	**MOLECULAR_FUNCTION**	**0.028088621**	**2**	**1**	**1220**	**10973**	
	Acrdi.XM_015914757.1	NEDD4-binding 2 isoform X2						P:GO:0090305; F:GO:0005524; F:GO:0004519; C:GO:0005829; P:GO:0
	Acrdi.XM_015911575.1	Polynucleotide 5 -hydroxyl-kinase NOL9						F:GO:0000166; F:GO:0051731; C:GO:0005634; F:GO:0005524; C:GO:0
**GO:0007160**	**cell-matrix adhesion GO:0007160**	**BIOLOGICAL_PROCESS**	**0.034297186**	**4**	**9**	**1218**	**10965**	
	Acrdi.XM_015904404.1	nidogen-2 isoform X1						P:GO:0007160; P:GO:0006955; F:GO:0005509; F:GO:0005044; F:GO:0
	Acrdi.XM_015913856.1	mammalian ependymin-related 1-like						P:GO:0007160; F:GO:0005509; C:GO:0005576
	Acrdi.XM_015908096.1	Mucin						P:GO:0007160; F:GO:0005509; C:GO:0016020; C:GO:0016021; F:GO:0
	Acrdi.XM_015906432.1	FRAS1-related extracellular matrix 1 isoform X1						P:GO:0007160; C:GO:0005604; P:GO:0007154; F:GO:0005509; C:GO:0

GO Annotations
F:serine-type endopeptidase inhibitor activity; C:collagen trimer; C:extracellular region; C:proteinaceous extracellular matrix
P:response to cytokine; P:response to organic substance; C:extracellular space; P:negative regulation of catalytic activity; P:negative regulation of membrane protein ectodomain proteolysis; P:negative regulation of endopeptidase activity; F:protease binding; P:response to hormone; C:extracellular region; F:metalloendopeptidase inhibitor activity; C:proteinaceous extracellular matrix
C:membrane; C:integral component of membrane; C:proteinaceous extracellular matrix
C:collagen trimer; F:extracellular matrix structural constituent; C:proteinaceous extracellular matrix
C:interstitial matrix; P:positive regulation of apoptotic process; P:proteolysis; P:extracellular matrix organization; F:protease binding; F:peptidase activity; C:extracellular matrix; F:metalloendopeptidase activity; C:proteinaceous extracellular matrix
P:cell-matrix adhesion; P:immune response; F:calcium ion binding; F:scavenger receptor activity; F:extracellular matrix structural constituent; P:receptor-mediated endocytosis; C:proteinaceous extracellular matrix; F:polysaccharide binding
P:cell-matrix adhesion; F:calcium ion binding; C:membrane; C:integral component of membrane; F:extracellular matrix structural constituent; C:proteinaceous extracellular matrix
F:zinc ion binding; C:collagen trimer; C:membrane; P:proteolysis; C:integral component of membrane; F:metalloendopeptidase activity; C:proteinaceous extracellular matrix
P:cell-matrix adhesion; C:basement membrane; P:cell communication; F:calcium ion binding; C:integral component of membrane
C:membrane; C:integral component of membrane; C:proteinaceous extracellular matrix
F:growth factor activity; C:membrane
C:extracellular space; F:growth factor activity; P:positive regulation of protein autophosphorylation; P:positive regulation of cell proliferation; C:membrane; C:integral component of membrane; F:growth factor receptor binding
F:growth factor activity; C:extracellular region
C:extracellular space; F:growth factor activity; P:positive regulation of protein autophosphorylation; P:positive regulation of cell proliferation; C:membrane; C:integral component of membrane; F:growth factor receptor binding
F:growth factor activity; C:extracellular region
C:extracellular space; F:growth factor activity; P:regulation of MAPK cascade; F:transforming growth factor beta receptor binding; P:SMAD protein signal transduction; P:regulation of apoptotic process; P:cell development; F:cytokine activity; C:extracellular region; P:growth; P:positive regulation of pathway-restricted SMAD protein phosphorylation
F:serine-type endopeptidase inhibitor activity; C:collagen trimer; C:extracellular region; C:proteinaceous extracellular matrix
F:zinc ion binding; C:collagen trimer; C:membrane; P:proteolysis; C:integral component of membrane; F:metalloendopeptidase activity; C:proteinaceous extracellular matrix
F:calcium ion binding; C:collagen trimer; F:extracellular matrix structural constituent
C:collagen trimer; F:extracellular matrix structural constituent; C:proteinaceous extracellular matrix
P:RNA polymerase I transcriptional preinitiation complex assembly at the promoter for the nuclear large rRNA transcript; C:RNA polymerase transcription factor SL1 complex; F:transcription factor activity, RNA polymerase I CORE element binding transcription factor recruiting; F:RNA polymerase I CORE element sequence-specific DNA binding; C:RNA polymerase I core factor complex
C:RNA polymerase I transcription factor complex; P:transcription from RNA polymerase I promoter
P:synaptonemal complex organization; P:synaptonemal complex assembly; C:synaptonemal complex
P:synaptonemal complex organization; P:synaptonemal complex assembly; C:synaptonemal complex
P:G-protein coupled receptor signaling pathway; C:membrane; C:integral component of membrane; P:response to pheromone
P:maturation of SSU-rRNA from tricistronic rRNA transcript (SSU-rRNA, 5.8S rRNA, LSU-rRNA); C:nucleus; P:rRNA processing; C:nucleolus; P:re-entry into mitotic cell cycle after pheromone arrest
C:membrane; C:integral component of membrane; F:copper ion transmembrane transporter activity; P:copper ion transmembrane transport
C:membrane; C:integral component of membrane; F:copper ion transmembrane transporter activity; P:copper ion transmembrane transport
C:membrane; C:integral component of membrane; F:copper ion transmembrane transporter activity; P:copper ion transmembrane transport
C:membrane; C:integral component of membrane; F:copper ion transmembrane transporter activity; P:copper ion transmembrane transport
P:nucleic acid phosphodiester bond hydrolysis; F:ATP binding; F:endonuclease activity; C:cytosol; P:phosphorylation; F:ATP-dependent polydeoxyribonucleotide 5'-hydroxyl-kinase activity
F:nucleotide binding; F:polynucleotide 5'-hydroxyl-kinase activity; C:nucleus; F:ATP binding; C:membrane; P:cleavage in ITS2 between 5.8S rRNA and LSU-rRNA of tricistronic rRNA transcript (SSU-rRNA, 5.8S rRNA, LSU-rRNA); P:phosphorylation
P:cell-matrix adhesion; P:immune response; F:calcium ion binding; F:scavenger receptor activity; F:extracellular matrix structural constituent; P:receptor-mediated endocytosis; C:proteinaceous extracellular matrix; F:polysaccharide binding
P:cell-matrix adhesion; F:calcium ion binding; C:extracellular region
P:cell-matrix adhesion; F:calcium ion binding; C:membrane; C:integral component of membrane; F:extracellular matrix structural constituent; C:proteinaceous extracellular matrix
P:cell-matrix adhesion; C:basement membrane; P:cell communication; F:calcium ion binding; C:integral component of membrane

Table S5. Results of the network analysis.

Significantly enriched (Fisher's exact test, *p* -value < 0.05, see Methods) KEGG ortholog IDs in the modules identified using WGCNA. The Interpro and GO annotations are also shown.

ModuleId	KEGG ortholog ID	Description	Pathways Ids	Pathways descriptions	Enrichment	p-value
blue	K06564	IGF2R, CD222; insulin-like growth factor 2 receptor	ko04144#map04144#map04142#	NA#Endocytosis#Lysosome#	17.03125	0.030627
blue	K14312	NUP155, NUP170, NUP157; nuclear pore complex protein Nup155	ko03013#map03013	NA#RNA transport#	100000000	0.010967
blue	K02868	**RP-L11e, RPL11; large subunit ribosomal protein L11e**	map03010#ko03010	Ribosome#NA#	100000000	0.010967
blue	K05096	FLT1, VEGFR1; FMS-like tyrosine kinase 1 [EC:2.7.10.1]	ko04060#ko05202#map04510#m	NA#NA#Focal adhesion#Tran	100000000	0.010967
blue	K02891	**RP-L22e, RPL22; large subunit ribosomal protein L22e**	map03010#ko03010	Ribosome#NA#	100000000	0.010967
brown	K01939	purA, ADSS; adenylosuccinate synthase [EC:6.3.4.4]	map01100#map00230#ko01100#	Metabolic pathways#Purine m	100000000	0.004157
**green**	K16311	SIK2; serine/threonine-protein kinase SIK2 [EC:2.7.11.1]	ko04922#map04922	NA#Glucagon signaling pathw	100000000	0.044335
**green**	K05022	CLIC2; chloride intracellular channel protein 2	NA		100000000	0.044335
**green**	K09050	CREBN; cAMP response element-binding protein, invertebrate	NA		100000000	0.044335
**green**	K09408	FOXO3; forkhead box protein O3	map05213#ko04062#map04213#	Endometrial cancer#NA#Long	100000000	0.044335
**green**	K16687	YAP1, Yki; transcriptional coactivator YAP1	map04392#map04390#map04391	Hippo signaling pathway -mul	100000000	0.044335
**green**	K19603	MAPK15; mitogen-activated protein kinase 15 [EC:2.7.11.24]	NA		100000000	0.044335
**green**	K19473	SIX3_6, OPTIX; homeobox protein SIX3/6	NA		100000000	0.044335
**green**	K08814	TRIB1_2; tribbles homolog 1/2	NA		100000000	0.044335
**green**	K18754	LIN28; protein lin-28	NA		100000000	0.044335
**green**	K04237	PARD3; partitioning defective protein 3	map04530#map04390#ko04080#	Tight junction#Hippo signalin	100000000	0.044335
**green**	K17341	HMCN; hemicentin	NA		8.622222222	0.035119
**green**	K09510	DNAJB4; DnaJ homolog subfamily B member 4	NA		100000000	0.001931
**green**	K08367	RUNX1, AML1; runt-related transcription factor 1	map05202#ko05220#map05200#	Transcriptional misregulation	100000000	0.044335
**green**	K09441	GABPA; GA-binding protein transcription factor, alpha	NA		100000000	0.044335
**green**	K20232	EDL; ETS-domain lacking	ko04013#map04013	NA#MAPK signaling pathway -	100000000	0.044335
**green**	K19495	JAZF1; juxtaposed with another zinc finger protein 1	NA		100000000	0.044335
**green**	K16342	PLA2G4, CPLA2; cytosolic phospholipase A2 [EC:3.1.1.4]	map01100#ko00564#ko00565#m	Metabolic pathways#NA#NA#	100000000	0.044335
**green**	K10343	SPSB1_4, SSB1, SSB4; SPRY domain-containing SOCS box protein 1/4	NA		100000000	0.044335
**green**	K07936	RAN; GTP-binding nuclear protein Ran	map05166#map05169#ko03008#	HTLV-I infection#Epstein-Bar	100000000	0.044335
**green**	K07917	RAB30; Ras-related protein Rab-30	NA		100000000	0.044335
**green**	K17494	CSRNP; cysteine/serine-rich nuclear protein	NA		100000000	0.044335
**green**	K04506	SIAH1; E3 ubiquitin-protein ligase SIAH1 [EC:2.3.2.27]	map04115#map04013#ko04013#	p53 signaling pathway#MAPK	100000000	0.044335
**green**	K01809	manA, MPI; mannose-6-phosphate isomerase [EC:5.3.1.8]	map01100#map01110#ko01110#	Metabolic pathways#Biosyntl	100000000	0.044335
**green**	K09381	PAX3_7; paired box protein 3/7	NA		64.66666667	0.00032
**green**	K18733	LARP6; la-related protein 6	NA		100000000	0.044335
**green**	K09431	ETV1; ets translocation variant 1	map05202#ko05202	Transcriptional misregulation	100000000	0.044335
**green**	K09309	NKX1; homeobox protein Nkx-1	NA		100000000	0.044335
**green**	K08826	HIPK; homeodomain interacting protein kinase [EC:2.7.11.1]	NA		100000000	0.044335
**green**	K18753	ZFP36L; butyrate response factor 1	NA		100000000	0.044335
**green**	K14443	TOB; protein Tob/BTG	map03018#ko03018	RNA degradation#NA#	43.11111111	0.005627
**green**	K18798	AFG1, LACE1; peroxisome-assembly ATPase [EC:3.6.4.7]	NA		100000000	0.044335
**green**	K09360	BARHL; BarH-like	NA		100000000	0.044335
**green**	K19041	RNF38_44; E3 ubiquitin-protein ligase RNF38/44 [EC:2.3.2.27]	NA		100000000	0.044335
**green**	K09203	EGR1; early growth response protein 1	map05166#ko05020#ko05166#k	HTLV-I infection#NA#NA#NA	100000000	0.001931
**green**	K09207	KLF6_7; krueppel-like factor 6/7	NA		100000000	0.044335
**green**	K14544	UTP22, NOL6; U3 small nucleolar RNA-associated protein 22	ko03008#map03008	NA#Ribosome biogenesis in eu	100000000	0.044335
**green**	K19907	SYT7; synaptotagmin-7	NA		100000000	0.044335
**green**	K04459	DUSP, MKP; dual specificity MAP kinase phosphatase [EC:3.1.3.16 3.1.3.48]	map04010#ko04010	MAPK signaling pathway#NA#	100000000	0.044335
**green**	K08596	SENP7; sentrin-specific protease 7 [EC:3.4.22.68]	NA		100000000	0.044335
**grey**	K14216	OAS; 2'-5'-oligoadenylate synthetase [EC:2.7.7.84]	map05164#map05162#map05160	Influenza A#Measles#Hepatiti	100000000	0.047027
**grey**	K21853	NA	NA		100000000	0.047027
magenta	K14857	SPB1, FTSJ3; AdoMet-dependent rRNA methyltransferase SPB1 [EC:2.1.1.-]	NA		100000000	0.025452
magenta	K10573	UBE2A, UBC2, RAD6A; ubiquitin-conjugating enzyme E2 A [EC:2.3.2.23]	map04120#ko04120	Ubiquitin mediated proteolysi	100000000	0.025452
magenta	K20365	ERGIC1; endoplasmic reticulum-Golgi intermediate compartment protein 1	NA		100000000	0.025452
magenta	K19269	PGP, PGLP; phosphoglycolate phosphatase [EC:3.1.3.18 3.1.3.48]	map01100#map00630#map01110	Metabolic pathways#Glyoxyla	100000000	0.025452
magenta	K09256	NFKBIL1; NF-kappa-B inhibitor-like protein 1	NA		100000000	0.025452
magenta	K13107	RBMX2, IST3; RNA-binding motif protein, X-linked 2	NA		100000000	0.025452
magenta	K02727	PSMA3; 20S proteasome subunit alpha 7 [EC:3.4.25.1]	map03050#ko03050	Proteasome#NA#	100000000	0.025452
magenta	K15703	RNF139, TRC8; E3 ubiquitin-protein ligase RNF139 [EC:2.3.2.27]	NA		100000000	0.025452
magenta	K03687	GRPE; molecular chaperone GrpE	NA		100000000	0.025452
magenta	K16185	RRAGA_B; Ras-related GTP-binding protein A/B	map04150#ko04150	mTOR signaling pathway#NA#	100000000	0.025452
magenta	K12386	CTNS; cystinosin	map04142#ko04142	Lysosome#NA#	100000000	0.025452
magenta	K01106	E3.1.3.56; inositol-1,4,5-trisphosphate 5-phosphatase [EC:3.1.3.56]	map01100#ko00562#map00562#	Metabolic pathways#NA#Inos	100000000	0.025452
magenta	K06171	NCSTN; nicastrin	map04330#ko05010#map05010#	Notch signaling pathway#NA#	100000000	0.025452
magenta	K06170	PSENEN, PEN2; presenilin enhancer 2	map04330#ko05010#map05010#	Notch signaling pathway#NA#	100000000	0.025452
magenta	K06172	APH1A; gamma-secretase subunit APH-1A	map04330#ko05010#map05010#	Notch signaling pathway#NA#	100000000	0.025452
magenta	K08515	VAMP7; vesicle-associated membrane protein 7	ko04130#map04130	NA#SNARE interactions in ves	100000000	0.025452
magenta	K03038	PSMD7, RPN8; 26S proteasome regulatory subunit N8	ko05169#map05169#map03050#	NA#Epstein-Barr virus infecti	100000000	0.025452
magenta	K07890	RAB21; Ras-related protein Rab-21	NA		100000000	0.025452
magenta	K07893	RAB6A; Ras-related protein Rab-6A	NA		100000000	0.025452
magenta	K03031	PSMD8, RPN12; 26S proteasome regulatory subunit N12	ko05169#map05169#map03050#	NA#Epstein-Barr virus infecti	100000000	0.025452
magenta	K15284	SLC35E2; solute carrier family 35, member E2	NA		100000000	0.025452
magenta	K08496	GOSR2, BOS1; golgi SNAP receptor complex member 2	ko04130#map04130	NA#SNARE interactions in ves	100000000	0.025452
magenta	K19177	NUS1; dehydrodolichyl diphosphate syntase complex subunit NUS1 [EC:2.5.1.87]	ko01110#map01110#ko00900#m	NA#Biosynthesis of secondary	100000000	0.025452
magenta	K00733	B4GALT7; xylosylprotein 4-beta-galactosyltransferase [EC:2.4.1.133]	map01100#ko00534#ko01100#k	Metabolic pathways#NA#NA#	100000000	0.025452
magenta	K02736	PSMB4; 20S proteasome subunit beta 7 [EC:3.4.25.1]	map03050#ko03050	Proteasome#NA#	100000000	0.025452
magenta	K11827	AP2S1; AP-2 complex subunit sigma-1	ko05016#ko04144#map04961#k	NA#NA#Endocrine and other	100000000	0.025452
magenta	K08378	GPR103; G protein-coupled receptor 103	NA		100000000	0.025452
magenta	K10435	MAP1LC; microtubule-associated protein 1 light chain	NA		100000000	0.025452
red	K08873	SMG1; serine/threonine-protein kinase SMG1 [EC:2.7.11.1]	map03015#ko03015	mRNA surveillance pathway#	100000000	0.022989
red	K01115	PLD1_2; phospholipase D1/2 [EC:3.1.4.4]	map01100#ko00564#ko00565#m	Metabolic pathways#NA#NA#	100000000	0.022989
red	K18627	KIF25; kinesin family member 25	NA		100000000	0.022989
red	K12968	ADAR, ADAR1; double-stranded RNA-specific adenosine deaminase [EC:3.5.4.37]	map05164#map05162#ko05164#	Influenza A#Measles#NA#Cyt	100000000	0.022989
red	K10691	UBR4, ZUBR1; E3 ubiquitin-protein ligase UBR4 [EC:2.3.2.27]	map05203#ko05203	Viral carcinogenesis#NA#	100000000	0.022989
red	K09553	STIP1; stress-induced-phosphoprotein 1	ko05020#map05020	NA#Prion diseases#	100000000	0.022989
red	K09214	GL; glass	NA		100000000	0.022989
red	K16573	TUBGCP6, GCP6; gamma-tubulin complex component 6	NA		100000000	0.022989
red	K10408	DNAH; dynein heavy chain, axonemal	map05016#ko05016	Huntington's disease#NA#	21.25	0.007224
red	K04424	ZAK, MLTK; sterile alpha motif and leucine zipper containing kinase AZK [EC:2.7.11.25]	ko04530#map04530#map04010#	NA#Tight junction#MAPK sig	100000000	0.022989
red	K05695	PTPRF, LAR; receptor-type tyrosine-protein phosphatase F [EC:3.1.3.48]	map04514#ko04910#ko04514#k	Cell adhesion molecules (CAM	100000000	0.022989
red	K16685	WWC1; protein KIBRA	map04392#map04390#map04391	Hippo signaling pathway -mul	100000000	0.022989
red	K00922	PIK3C; phosphatidylinositol-4,5-bisphosphate 3-kinase [EC:2.7.1.153]	ko00562#ko05100#ko04062#ko	NA#NA#NA#NA#NA#NA#NA	100000000	0.022989
red	K09654	B3GALNT2; beta-1,3-N-acetylgalactosaminyltransferase 2 [EC:2.4.1.313]	map01100#ko01100#map00515#	Metabolic pathways#NA#Mar	100000000	0.022989
red	K19526	VPS13B; vacuolar protein sorting-associated protein 13B	NA		100000000	0.022989
red	K08333	PIK3R4, VPS15; phosphoinositide-3-kinase, regulatory subunit 4 [EC:2.7.11.1]	ko04140#map04140	NA#Autophagy#	42.5	0.045467
red	K13708	DOCK1; dedicator of cytokinesis protein 1	map04510#map04810#ko05100#	Focal adhesion#Regulation of	100000000	0.022989
red	K08117	APLP2; amyloid-like protein 2	NA		42.5	0.045467
red	K11990	DHH; desert hedgehog	map04340#ko04340	Hedgehog signaling pathway#	100000000	0.022989
red	K10358	MYO6; myosin VI	NA		100000000	0.022989
red	K17277	EPS8; epidermal growth factor receptor kinase substrate 8	NA		100000000	0.022989
red	K12231	HECTD1; E3 ubiquitin-protein ligase HECTD1 [EC:2.3.2.26]	NA		100000000	0.022989
red	K05766	SSH; protein phosphatase slingshot [EC:3.1.3.16 3.1.3.48]	ko04360#map04810#ko04810#m	NA#Regulation of actin cytosk	100000000	0.022989
**turquoise**	K08834	MYO3, DFNB30; myosin III [EC:2.7.11.1]	map04745#ko04745	Phototransduction - fly#NA#	100000000	0.024226
**turquoise**	K16727	ARHGEF10; Rho guanine nucleotide exchange factor 10	NA		100000000	0.024226
**turquoise**	K13864	SLC7A2, ATRC2; solute carrier family 7 (cationic amino acid transporter), member 2	NA		100000000	0.003745
**turquoise**	K14317	NUP214, CAN; nuclear pore complex protein Nup214	ko03013#map03013#ko05169#m	NA#RNA transport#NA#Epst	100000000	0.024226
**turquoise**	K09542	CRYAB; crystallin, alpha B	map04213#ko04213#ko04141#m	Longevity regulating pathway	100000000	0.024226
yellow	K18402	PHF20; PHD finger protein 20	NA		100000000	0.003948
yellow	K10408	DNAH; dynein heavy chain, axonemal	map05016#ko05016	Huntington's disease#NA#	14.81818182	0.004234
yellow	K14676	NTE, NRE; lysophospholipid hydrolase [EC:3.1.1.5]	map00564#ko00564	Glycerophospholipid metabol	100000000	0.003948
yellow	K10587	UBE3A, E6AP; ubiquitin-protein ligase E3 A [EC:2.3.2.26]	map04120#ko04120#map05203#	Ubiquitin mediated proteolysi	100000000	0.003948

ModuleId	InterPro ID	Description	Enrichment	p-value
blue	IPR023393	START-like domain	7.48600509	0.04957148
blue	IPR014717	Translation elongation factor EF1B/ribosomal protein S6	100000000	0.003910601
blue	IPR001163	LSM domain, eukaryotic/archaea-type	100000000	1.51E-05
blue	IPR000626	Ubiquitin domain	9.98134012	0.03445601
blue	IPR011331	Ribosomal protein L37ae/L37e	100000000	0.003910601
blue	IPR009072	Histone-fold	7.48600509	0.04957148
blue	IPR005824	KOW	100000000	0.003910601
blue	IPR000218	Ribosomal protein L14P	100000000	0.003910601
blue	IPR018181	Heat shock protein 70, conserved site	9.98134012	0.03445601
blue	IPR020929	Ribosomal protein L5, conserved site	100000000	0.003910601
blue	IPR003594	Histidine kinase-like ATPase, C-terminal domain	9.98134012	0.03445601
blue	IPR000479	Cation-independent mannose-6-phosphate receptor	29.9440204	0.01124446
blue	IPR002942	RNA-binding S4 domain	100000000	0.003910601
blue	IPR009011	Mannose-6-phosphate receptor binding domain	4.99067006	0.03508166
blue	IPR016130	Protein-tyrosine phosphatase, active site	14.9720102	0.02155967
blue	IPR031309	Ribosomal protein L5, C-terminal	100000000	0.003910601
blue	IPR019956	Ubiquitin	14.9720102	0.02155967
blue	IPR019954	Ubiquitin conserved site	29.9440204	0.01124446
blue	IPR011511	Variant SH3 domain	7.48600509	0.04957148
blue	IPR031310	Ribosomal protein L5, N-terminal	100000000	0.003910601
blue	IPR014908	Nucleoporin, Nup133/Nup155-like, N-terminal	100000000	0.003910601
blue	IPR002671	Ribosomal protein L22e	100000000	0.003910601
blue	IPR013126	Heat shock protein 70 family	9.98134012	0.03445601
blue	IPR022803	Ribosomal protein L5 domain	100000000	0.003910601
blue	IPR013098	Immunoglobulin I-set	3.11916879	0.03205506
brown	IPR027469	Cation efflux protein transmembrane domain	34.494186	0.008658945
brown	IPR002524	Cation efflux protein	34.494186	0.008658945
brown	IPR001452	SH3 domain	5.17412791	0.03095725
brown	IPR001114	Adenylosuccinate synthetase	100000000	0.002995143
brown	IPR003439	ABC transporter-like	4.10645072	0.01217322
brown	IPR017871	ABC transporter, conserved site	5.07267442	0.005832653
brown	IPR029063	S-adenosyl-L-methionine-dependent methyltransferase	3.4494186	0.03948101
brown	IPR023753	FAD/NAD(P)-binding domain	7.66537468	0.004269313
brown	IPR013216	Methyltransferase type 11	17.247093	0.01669154
brown	IPR001368	TNFR/NGFR cysteine-rich region	7.83958774	0.001269309
brown	IPR001394	Peptidase C19, ubiquitin carboxyl-terminal hydrolase	11.498062	0.02681776
brown	IPR003137	PA domain	11.498062	0.02681776
**green**	IPR020479	Homeodomain, metazoa	21.2588652	0.01136293
**green**	IPR002087	Anti-proliferative protein	42.5177305	0.005854992
**green**	IPR003102	Coactivator CBP, pKID	100000000	0.04492592
**green**	IPR009917	Steroid receptor RNA activator-protein/coat protein complex II, Sec31	100000000	0.04492592
**green**	IPR019169	Transmembrane protein 26	100000000	0.04492592
**green**	IPR018499	Tetraspanin/Peripherin	14.1725768	0.01837905
**green**	IPR013087	Zinc finger C2H2-type/integrase DNA-binding domain	9.30075355	4.52E-05
**green**	IPR022750	Interferon regulatory factor 2-binding protein 1 & 2, zinc finger	100000000	0.04492592
**green**	IPR000585	Hemopexin-like domain	100000000	0.04492592
**green**	IPR021190	Peptidase M10A	100000000	0.04492592
**green**	IPR001523	Paired domain	63.7765957	0.000347051
**green**	IPR011991	Winged helix-turn-helix DNA-binding domain	7.97207447	9.77E-06
**green**	IPR005654	ATPase, AFG1-like	100000000	0.04492592
**green**	IPR000232	Heat shock factor (HSF)-type, DNA-binding	100000000	0.04492592
**green**	IPR001356	Homeobox domain	21.2588652	5.73E-06
**green**	IPR001452	SH3 domain	6.37765957	0.01834907
**green**	IPR029399	TMEM192 family	100000000	0.04492592
**green**	IPR018050	Phosphomannose isomerase, type I, conserved site	100000000	0.04492592
**green**	IPR011511	Variant SH3 domain	10.6294326	0.02675768
**green**	IPR005225	Small GTP-binding protein domain	3.54314421	0.03488968
**green**	IPR005554	Nrap protein	100000000	0.04492592
**green**	IPR009565	Protein of unknown function DUF1180	100000000	0.04492592
**green**	IPR016305	Mannose-6-phosphate isomerase	100000000	0.04492592
**green**	IPR028002	Myb/SANT-like DNA-binding domain	100000000	0.04492592
**green**	IPR017979	GPCR, family 3, conserved site	100000000	0.04492592
**green**	IPR002939	Chaperone DnaJ, C-terminal	14.1725768	0.01837905
**green**	IPR003812	Fido domain	100000000	0.002011502
**green**	IPR020683	Ankyrin repeat-containing domain	3.46074551	0.006513987
**green**	IPR009057	Homeodomain-like	14.1725768	2.77E-05
**green**	IPR018487	Hemopexin-like repeats	100000000	0.04492592
**green**	IPR018888	Uncharacterised protein family UPF0561	100000000	0.04492592
**green**	IPR031701	Homeobox protein SIX1, N-terminal SD domain	100000000	0.04492592
**green**	IPR001806	Small GTPase superfamily	3.69719396	0.03096417
**green**	IPR000040	Acute myeloid leukemia 1 protein (AML1)/Runt	100000000	0.04492592
**green**	IPR003417	Core binding factor, beta subunit	100000000	0.04492592
**green**	IPR010542	Vertebrate heat shock transcription factor, C-terminal domain	100000000	0.04492592
**green**	IPR011500	GPCR, family 3, nine cysteines domain	100000000	0.04492592
**green**	IPR000162	GPCR, family 3, metabotropic glutamate receptor	100000000	0.04492592
**green**	IPR008653	Immediate early response	100000000	0.04492592
**green**	IPR023260	Cysteine/serine-rich nuclear protein family	100000000	0.04492592
**green**	IPR006630	RNA-binding protein Lupus La	100000000	0.04492592
**green**	IPR000770	SAND domain	100000000	0.04492592
**green**	IPR018121	Seven-in-absentia protein, TRAF-like domain	100000000	0.04492592
**green**	IPR001818	Peptidase M10, metallopeptidase	100000000	0.04492592
**green**	IPR013524	Runt domain	100000000	0.04492592
**green**	IPR001565	Synaptotagmin	100000000	0.04492592
**green**	IPR000276	G protein-coupled receptor, rhodopsin-like	5.9960902	9.66E-06
**green**	IPR031972	Cysteine/serine-rich nuclear protein, N-terminal domain	100000000	0.04492592
**green**	IPR002642	Lysophospholipase, catalytic domain	100000000	0.04492592
**green**	IPR007635	Tis11B-like protein, N-terminal	100000000	0.04492592
**green**	IPR002404	IRS-type PTB domain	100000000	0.04492592
**green**	IPR017970	Homeobox, conserved site	14.1725768	0.000675495
**green**	IPR001820	Protease inhibitor I35 (TIMP)	21.2588652	0.01136293
**green**	IPR000418	Ets domain	15.9441489	0.002744118
**green**	IPR001496	SOCS box domain	100000000	0.002011502
**green**	IPR000301	Tetraspanin	14.1725768	0.01837905
**green**	IPR007087	Zinc finger, C2H2	5.31471631	0.001857767
**green**	IPR001250	Mannose-6-phosphate isomerase, type I	100000000	0.04492592
**green**	IPR002344	Lupus La protein	100000000	0.04492592
**green**	IPR006715	PEA3-type ETS-domain transcription factor, N-terminal	100000000	0.04492592
**green**	IPR021922	Domain of unknown function DUF3534	100000000	0.04492592
**green**	IPR001630	cAMP response element binding (CREB) protein	100000000	0.04492592
**green**	IPR003118	Pointed domain	42.5177305	0.005854992
grey	IPR014729	Rossmann-like alpha/beta/alpha sandwich fold	5.62584596	0.0100362
grey	IPR017448	SRCR-like domain	8.18304866	0.01792997
grey	IPR006652	Kelch repeat type 1	7.16016758	0.03266737
grey	IPR000884	Thrombospondin type-1 (TSP1) repeat	3.58008379	0.01357718
grey	IPR016186	C-type lectin-like	3.40960361	0.04216629
grey	IPR016054	Ly-6 antigen/uPA receptor-like	100000000	0.01455846
grey	IPR001611	Leucine-rich repeat	2.1736223	0.04762368
grey	IPR015422	Pyridoxal phosphate-dependent transferase, major region, subdomain 2	7.16016758	0.03266737
grey	IPR002934	Polymerase, nucleotidyl transferase domain	100000000	0.02947406
grey	IPR011705	BTB/Kelch-associated	3.75056397	0.0259012
grey	IPR001875	Death effector domain	100000000	0.02947406
grey	IPR001878	Zinc finger, CCHC-type	2.65949082	0.04328249
grey	IPR005018	DOMON domain	100000000	0.01455846
grey	IPR001190	SRCR domain	7.16016758	0.03266737
grey	IPR004344	Tubulin-tyrosine ligase/Tubulin polyglutamylase	100000000	0.02947406
grey	IPR002035	von Willebrand factor, type A	4.60296487	0.02996234
grey	IPR000210	BTB/POZ domain	2.3380139	0.04123504
grey	IPR001781	Zinc finger, LIM-type	100000000	0.007189849
magenta	IPR006762	Gtr1/RagA G protein	100000000	0.01736498
magenta	IPR019379	Gamma-secretase aspartyl protease complex, presenilin enhancer-2 subunit	100000000	0.01736498
magenta	IPR009294	Gamma-secretase subunit Aph-1	100000000	0.01736498
magenta	IPR006573	Neuralized homology repeat (NHR) domain	100000000	0.01736498
magenta	IPR001441	Decaprenyl diphosphate synthase-like	100000000	0.01736498
magenta	IPR004241	Autophagy protein Atg8 ubiquitin-like	113.174312	0.00088628
magenta	IPR001353	Proteasome, subunit alpha/beta	16.1677588	0.009933441
magenta	IPR000740	GrpE nucleotide exchange factor	100000000	0.01736498
magenta	IPR001666	Phosphatidylinositol transfer protein	56.587156	0.03443114
magenta	IPR010400	PITH domain	100000000	0.01736498
magenta	IPR006349	2-phosphoglycolate phosphatase, eukaryotic	100000000	0.01736498
magenta	IPR005225	Small GTP-binding protein domain	6.79045872	0.01217107
magenta	IPR018628	Cytochrome c oxidase assembly factor 3, mitochondrial	100000000	0.01736498
magenta	IPR005282	Lysosomal cystine transporter	100000000	0.01736498
magenta	IPR024969	Rpn11/EIF3F, C-terminal	56.587156	0.03443114
magenta	IPR027791	Galactosyltransferase, C-terminal	100000000	0.01736498
magenta	IPR006357	HAD-superfamily hydrolase, subfamily IIA	100000000	0.01736498
magenta	IPR006603	PQ-loop repeat	100000000	0.01736498
magenta	IPR016021	MIF4G-like domain	56.587156	0.03443114
magenta	IPR001806	Small GTPase superfamily	7.0733945	0.01100391
magenta	IPR013766	Thioredoxin domain	28.293578	0.004282377
magenta	IPR027421	DNA polymerase family X lyase domain	56.587156	0.03443114
magenta	IPR009012	GrpE nucleotide exchange factor, head	100000000	0.01736498
magenta	IPR008710	Nicastrin	100000000	0.01736498
magenta	IPR025754	TRC8 N-terminal domain	100000000	0.01736498
magenta	IPR021720	Malectin	100000000	0.01736498
magenta	IPR003307	W2 domain	56.587156	0.03443114
magenta	IPR029398	DNA polymerase beta, thumb domain	56.587156	0.03443114
magenta	IPR023215	Nitrophenylphosphatase-like domain	100000000	0.01736498
magenta	IPR022775	AP complex, mu/sigma subunit	100000000	0.01736498
magenta	IPR033464	CSN8/PSMD8/EIF3K	100000000	0.01736498
magenta	IPR000804	Clathrin adaptor complex, small chain	100000000	0.01736498
magenta	IPR027995	Galactosyltransferase, N-terminal	100000000	0.01736498
magenta	IPR029055	Nucleophile aminohydrolases, N-terminal	16.1677588	0.009933441
magenta	IPR004853	Sugar phosphate transporter domain	56.587156	0.03443114
magenta	IPR018790	Protein of unknown function DUF2358	100000000	0.01736498
magenta	IPR004932	Retrieval of early ER protein Rer1	100000000	0.01736498
magenta	IPR013805	GrpE nucleotide exchange factor, coiled-coil	100000000	0.01736498
magenta	IPR002877	Ribosomal RNA methyltransferase FtsJ domain	100000000	0.01736498
magenta	IPR003859	Beta-1,4-galactosyltransferase	100000000	0.01736498
magenta	IPR012936	Endoplasmic reticulum vesicle transporter, C-terminal	100000000	0.01736498
magenta	IPR022564	Protein of unknown function DUF2678	100000000	0.01736498
red	IPR000001	Kringle	100000000	0.0324996
red	IPR015943	WD40/YVTN repeat-like-containing domain	3.84123972	0.02580197
red	IPR000488	Death domain	6.8699095	0.01385471
red	IPR024491	Selenoprotein SelK/SelG	100000000	0.0324996
red	IPR020067	Frizzled domain	100000000	0.0324996
red	IPR002857	Zinc finger, CXXC-type	100000000	0.0324996
red	IPR024317	Dynein heavy chain, P-loop containing D4 domain	19.8464052	0.009851466
red	IPR002420	Phosphatidylinositol 3-kinase, C2 domain	100000000	0.0324996
red	IPR018936	Phosphatidylinositol 3/4-kinase, conserved site	59.5392157	0.003085962
red	IPR003126	Zinc finger, UBR-type	100000000	0.0324996
red	IPR013625	Tensin phosphotyrosine-binding domain	100000000	0.0324996
red	IPR000341	Phosphatidylinositol 3-kinase Ras-binding (PI3K RBD) domain	100000000	0.0324996
red	IPR018056	Kringle, conserved site	100000000	0.0324996
red	IPR004273	Dynein heavy chain domain	22.3272059	0.001074841
red	IPR014876	DEK, C-terminal	100000000	0.0324996
red	IPR000560	Histidine phosphatase superfamily, clade-2	100000000	0.0324996
red	IPR019594	Ionotropic glutamate receptor, L-glutamate and glycine-binding domain	100000000	0.0324996
red	IPR003152	FATC domain	100000000	0.0324996
red	IPR004009	Myosin, N-terminal, SH3-like	100000000	0.0324996
red	IPR001263	Phosphoinositide 3-kinase, accessory (PIK) domain	100000000	0.0324996
red	IPR018863	Fragile site-associated protein, C-terminal	100000000	0.0324996
red	IPR013602	Dynein heavy chain, domain-2	14.8848039	0.01446258
red	IPR011704	ATPase, dynein-related, AAA domain	100000000	0.0324996
red	IPR013594	Dynein heavy chain, domain-1	100000000	0.001051214
red	IPR009543	Vacuolar protein sorting-associated protein 13, SHR-binding domain	100000000	0.0324996
red	IPR032412	Myosin VI, cargo binding domain	100000000	0.0324996
red	IPR024779	2OGFeDO, oxygenase domain	100000000	0.0324996
red	IPR010580	Stress-associated endoplasmic reticulum protein	100000000	0.0324996
red	IPR001657	Hedgehog protein	100000000	0.0324996
red	IPR001245	Serine-threonine/tyrosine-protein kinase catalytic domain	4.57993967	0.01530777
red	IPR031645	Vacuolar protein sorting-associated protein 13, C-terminal	100000000	0.0324996
red	IPR031646	Vacuolar protein sorting-associated protein 13, second N-terminal domain	100000000	0.0324996
red	IPR002466	Adenosine deaminase/editase	100000000	0.0324996
red	IPR032376	Dedicator of cytokinesis, N-terminal domain	100000000	0.0324996
red	IPR007259	Gamma-tubulin complex component protein	100000000	0.0324996
red	IPR031559	Serine/threonine-protein kinase SMG1	100000000	0.0324996
red	IPR000716	Thyroglobulin type-1	100000000	0.0324996
red	IPR013651	ATP-grasp fold, RimK-type	100000000	0.0324996
red	IPR001736	Phospholipase D/Transphosphatidylase	100000000	0.0324996
red	IPR000403	Phosphatidylinositol 3-/4-kinase, catalytic domain	59.5392157	0.003085962
red	IPR003113	Phosphatidylinositol 3-kinase adaptor-binding (PI3K ABD) domain	100000000	0.0324996
**turquoise**	**IPR015943**	**WD40/YVTN repeat-like-containing domain (may form propellers)**	**2.297374**	**0.02252717**
**turquoise**	**IPR002293**	**Amino acid/polyamine transporter I**	**25.06226**	**0.000652984**
**turquoise**	IPR005552	Scramblase	100000000	0.02764074
**turquoise**	IPR002151	Kinesin light chain	100000000	0.004589905
**turquoise**	IPR008978	HSP20-like chaperone	5.01245211	0.0303501

**turquoise**	IPR019826	Carboxylesterase type B, active site	7.51867816	0.03523306
**turquoise**	IPR012561	Ferlin B-domain	100000000	0.02764074
**turquoise**	IPR007632	Anoctamin	15.0373563	0.01607491
**turquoise**	**IPR025139**	**Domain of unknown function DUF4062**	**25.06226**	**0.000652984**
**turquoise**	IPR002347	Short-chain dehydrogenase/reductase SDR	2.73406479	0.0497091
**turquoise**	IPR004843	Calcineurin-like phosphoesterase domain, apaH type	7.51867816	0.03523306
**turquoise**	IPR017907	Zinc finger, RING-type, conserved site	2.27838732	0.03013884
**turquoise**	IPR000082	SEA domain	5.01245211	0.007783187
**turquoise**	IPR002068	Alpha crystallin/Hsp20 domain	15.0373563	0.01607491
**turquoise**	IPR000330	SNF2-related, N-terminal domain involved in transcription regulation, DNA repair and recor	15.0373563	0.01607491
**turquoise**	IPR020904	Short-chain dehydrogenase/reductase, conserved site	5.01245211	0.01525013
**turquoise**	IPR023827	Peptidase S8, subtilisin, Asp-active site	100000000	0.02764074
**turquoise**	**IPR029063**	**S-adenosyl-L-methionine-dependent methyltransferase**	**3.580323**	**0.003225657**
**turquoise**	IPR002018	Carboxylesterase, type B	7.51867816	0.03523306
**turquoise**	IPR032362	Ferlin, C-terminal domain	100000000	0.02764074
**turquoise**	IPR019819	Carboxylesterase type B, conserved site	7.51867816	0.03523306
**turquoise**	IPR001436	Alpha crystallin/Heat shock protein	100000000	0.004589905
**turquoise**	**IPR000008**	**C2 domain (Ca2+-dependent membrane-targeting module involved in signal transd**	**4.177043**	**0.001461918**
**turquoise**	IPR006076	FAD dependent oxidoreductase	100000000	0.02764074
**turquoise**	**IPR000504**	**RNA recognition motif domain (heterogeneous nuclear ribonucleoproteins (hnRNPs)**	**3.341635**	**0.02697838**
**turquoise**	IPR001214	SET domain	15.0373563	0.01607491
**turquoise**	IPR004012	RUN domain	100000000	0.02764074
**turquoise**	IPR011032	GroES-like chaperonin 10	15.0373563	0.01607491
**turquoise**	IPR025946	CABIT domain	100000000	0.02764074
**turquoise**	IPR013149	Alcohol dehydrogenase, C-terminal	15.0373563	0.01607491
**turquoise**	IPR001132	SMAD domain, Dwarfin-type, control of cell growth	100000000	0.02764074
**turquoise**	IPR019775	WD40 repeat, conserved site (may form propellers)	3.00747126	0.03724798
**turquoise**	**IPR029485**	**Cationic amino acid transporter, C-terminal**	**20.04981**	**0.003302808**
**turquoise**	IPR000219	Dbl homology (DH) domain	7.51867816	0.03523306
**turquoise**	IPR013154	Alcohol dehydrogenase, N-terminal	15.0373563	0.01607491
**turquoise**	IPR016040	NAD(P)-binding domain	3.10294654	0.002066015
yellow	IPR002017	Spectrin repeat	27.0601852	0.0135313
yellow	IPR023780	Chromo domain	100000000	0.004726351
yellow	IPR011990	Tetratricopeptide-like helical domain	2.75188324	0.002996717
yellow	IPR000595	Cyclic nucleotide-binding domain	13.5300926	0.02583352
yellow	IPR001202	WW domain	6.7650463	0.01989414
yellow	IPR001206	Diacylglycerol kinase, catalytic domain	13.5300926	0.02583352
yellow	IPR024317	Dynein heavy chain, P-loop containing D4 domain	9.02006173	0.0411121
yellow	IPR001440	Tetratricopeptide repeat 1	9.02006173	0.0411121
yellow	IPR000742	EGF-like domain	3.38252315	0.04444683
yellow	IPR002049	Laminin EGF domain	20.2951389	0.002915652
yellow	IPR000198	Rho GTPase-activating protein domain	27.0601852	0.0135313
yellow	IPR001589	Actinin-type actin-binding domain, conserved site	27.0601852	0.0135313
yellow	IPR013602	Dynein heavy chain, domain-2	13.5300926	0.00553435
yellow	IPR000571	Zinc finger, CCCH-type	9.02006173	0.0411121
yellow	IPR019787	Zinc finger, PHD-finger	10.1475694	0.009193679
yellow	IPR012317	Poly(ADP-ribose) polymerase, catalytic domain	9.02006173	0.0411121
yellow	IPR001715	Calponin homology domain	13.5300926	0.00553435
yellow	IPR024983	CHAT domain	18.0401235	9.81E-07
yellow	IPR024743	Dynein heavy chain, coiled coil stalk	13.5300926	0.02583352
yellow	IPR032171	C-terminal of Roc (COR) domain	10.1475694	0.009193679
yellow	IPR019786	Zinc finger, PHD-type, conserved site	9.02006173	0.0411121
yellow	IPR032353	Ubiquitin-protein ligase E3A, N-terminal zinc-binding domain	100000000	0.004726351
yellow	IPR001791	Laminin G domain	13.5300926	0.02583352
yellow	IPR000253	Forkhead-associated (FHA) domain	13.5300926	0.02583352
yellow	IPR000159	Ras-associating (RA) domain	13.5300926	0.02583352
yellow	IPR019734	Tetratricopeptide repeat	4.92003367	0.000716237

ModuleId	GO Id	Description	Enrichmen	p-value
blue	GO:002262	cytosolic small ribosomal subunit	1E+08	1.20E-07
blue	GO:002262	cytosolic large ribosomal subunit	19.35972	2.31E-05
blue	GO:007018	large ribosomal subunit rRNA binding	1E+08	0.02928
blue	GO:000373	structural constituent of ribosome	31.94355	5.02E-21
blue	GO:000093	P-body	1E+08	0.02928
blue	GO:000641	translation	29.03959	7.40E-19
blue	GO:000592	focal adhesion	12.90648	7.19E-05
blue	GO:000573	small nucleolar ribonucleoprotein complex	1E+08	0.02928
blue	GO:000573	nucleolus	7.74389	0.000406
blue	GO:001984	rRNA binding	1E+08	2.47E-05
blue	GO:008000	Cul4-RING E3 ubiquitin ligase complex	1E+08	0.02928
blue	GO:000584	ribosome	58.07917	6.35E-09
blue	GO:001593	small ribosomal subunit	1E+08	0.004999
blue	GO:000372	RNA binding	11.29317	0.000311
blue	GO:000372	mRNA binding	1E+08	0.000852
brown	GO:000526	calcium channel activity	16.29111	0.033186
brown	GO:003555	intracellular signal transduction	6.109164	0.032442
brown	GO:004262	ATPase activity, coupled to transmembrane movement of substances	1E+08	0.011927
brown	GO:005153	4 iron, 4 sulfur cluster binding	16.29111	0.033186
brown	GO:003130	integral component of mitochondrial inner membrane	1E+08	0.011927
brown	GO:000194	heart looping	1E+08	0.011927
brown	GO:000551	calmodulin binding	16.29111	0.033186
brown	GO:000697	response to oxidative stress	16.29111	0.033186
brown	GO:000487	signal transducer activity	1E+08	0.011927
**green**	GO:005068	regulation of defense response to virus	1E+08	0.037135
**green**	GO:003198	Golgi cisterna	1E+08	0.037135
**green**	GO:001060	negative regulation of macromolecule metabolic process	1E+08	0.037135
**green**	GO:004851	circadian behavior	1E+08	0.037135
**green**	GO:004851	animal organ development	10.37143	0.025421
**green**	GO:003016	regulation of proteolysis	1E+08	0.037135
**green**	GO:007073	protein adenylyltransferase activity	1E+08	0.037135
**green**	GO:002184	cell proliferation in forebrain	1E+08	0.037135
**green**	GO:009740	neuroblast migration	1E+08	0.037135
**green**	GO:000340	optic vesicle morphogenesis	1E+08	0.037135
**green**	GO:000098	RNA polymerase II distal enhancer sequence-specific DNA binding	1E+08	0.037135
**green**	GO:003264	regulation of interferon-beta production	1E+08	0.037135
**green**	GO:005510	ubiquitin-protein transferase regulator activity	1E+08	0.037135
**green**	GO:001401	neuroblast differentiation	1E+08	0.037135
**green**	GO:000994	proximal/distal axis specification	1E+08	0.037135
**green**	GO:006500	biological regulation	9.723214	0.00663
**green**	GO:190198	regulation of cell cycle phase transition	1E+08	0.037135
**green**	GO:006107	regulation of neural retina development	1E+08	0.037135
**green**	GO:001811	protein adenylylation	1E+08	0.037135
**green**	GO:003208	negative regulation of NF-kappaB transcription factor activity	1E+08	0.037135
**green**	GO:000635	regulation of transcription, DNA-templated	5.983516	0.019649
**green**	GO:003563	CD40 receptor complex	1E+08	0.037135
**green**	GO:003320	tumor necrosis factor-mediated signaling pathway	1E+08	0.037135
**green**	GO:004340	regulation of MAP kinase activity	1E+08	0.037135
**green**	GO:002179	forebrain dorsal/ventral pattern formation	1E+08	0.037135
**green**	GO:000222	toll-like receptor signaling pathway	1E+08	0.037135
**green**	GO:002179	forebrain anterior/posterior pattern specification	1E+08	0.037135
**green**	GO:007030	lens fiber cell differentiation	1E+08	0.037135
**green**	GO:190350	regulation of nucleic acid-templated transcription	1E+08	0.037135
**green**	GO:004559	negative regulation of fat cell differentiation	1E+08	0.037135
**green**	GO:000703	Golgi organization	1E+08	0.037135
**green**	GO:000122	transcription corepressor binding	1E+08	0.037135
**green**	GO:003009	hemopoiesis	1E+08	0.037135
**green**	GO:003132	negative regulation of cellular metabolic process	1E+08	0.037135
**green**	GO:002198	pituitary gland development	1E+08	0.037135
**green**	GO:003143	mitogen-activated protein kinase kinase binding	1E+08	0.037135
**green**	GO:003017	negative regulation of Wnt signaling pathway	1E+08	0.037135
**green**	GO:190269	regulation of neuroblast proliferation	1E+08	0.037135
**green**	GO:190015	regulation of nuclear-transcribed mRNA catabolic process, deadenylation-depender	1E+08	0.037135
**green**	GO:199008	lens fiber cell apoptotic process	1E+08	0.037135
**green**	GO:002197	telencephalon regionalization	1E+08	0.037135
**green**	GO:000646	protein phosphorylation	8.642857	0.03308
**green**	GO:003023	myofibril assembly	1E+08	0.037135
**green**	GO:004508	negative regulation of interleukin-10 biosynthetic process	1E+08	0.037135
**green**	GO:006023	lens induction in camera-type eye	1E+08	0.037135
**green**	GO:005117	negative regulation of nitrogen compound metabolic process	1E+08	0.037135
**green**	GO:000120	transcriptional activator activity, RNA polymerase II distal enhancer sequence-spec	1E+08	0.037135
**green**	GO:000580	cis-Golgi network	1E+08	0.037135
**green**	GO:190274	apoptotic process involved in development	1E+08	0.037135
grey	GO:000429	threonine-type endopeptidase activity	8.698917	0.020946
grey	GO:004444	cytoplasmic part	2.658002	0.04023
magenta	GO:000810	oocyte microtubule cytoskeleton polarization	1E+08	0.046272
magenta	GO:004546	R7 cell development	1E+08	0.046272
magenta	GO:000729	germarium-derived egg chamber formation	1E+08	0.046272
magenta	GO:006032	head development	1E+08	0.046272
magenta	GO:004826	positive regulation of receptor-mediated endocytosis	1E+08	0.046272
magenta	GO:009000	positive regulation of protein localization to plasma membrane	1E+08	0.046272
magenta	GO:004545	cell redox homeostasis	1E+08	0.046272
magenta	GO:000045	enzyme-directed rRNA 2'-O-methylation	1E+08	0.046272
magenta	GO:000583	proteasome regulatory particle	1E+08	0.002128
magenta	GO:000000	reproduction	1E+08	0.046272
magenta	GO:000579	endoplasmic reticulum-Golgi intermediate compartment	1E+08	0.046272
magenta	GO:000392	GTPase activity	13.74098	0.019401
magenta	GO:000760	phototransduction	1E+08	0.046272
magenta	GO:005083	defense response to fungus	1E+08	0.046272
magenta	GO:000079	nuclear chromatin	1E+08	0.046272
magenta	GO:003525	UDP-galactosyltransferase activity	1E+08	0.046272
magenta	GO:007003	rRNA (guanosine-2'-O-)-methyltransferase activity	1E+08	0.046272
magenta	GO:003017	integral component of Golgi membrane	1E+08	0.046272
magenta	GO:001017	body morphogenesis	1E+08	0.046272
magenta	GO:000038	first spliceosomal transesterification activity	1E+08	0.046272
magenta	GO:001657	histone ubiquitination	1E+08	0.046272
magenta	GO:200064	positive regulation of early endosome to late endosome transport	1E+08	0.046272
magenta	GO:000552	GTP binding	5.152866	0.029346
magenta	GO:000662	protein targeting to vacuole	1E+08	0.046272
magenta	GO:003248	Rab protein signal transduction	1E+08	0.046272
magenta	GO:003027	clathrin binding	1E+08	0.046272
magenta	GO:001250	ER to Golgi transport vesicle membrane	1E+08	0.046272
magenta	GO:006007	regulation of postsynaptic membrane potential	1E+08	0.046272
magenta	GO:000689	intra-Golgi vesicle-mediated transport	1E+08	0.002128
magenta	GO:000689	retrograde vesicle-mediated transport, Golgi to ER	1E+08	0.002128
magenta	GO:000689	Golgi to vacuole transport	1E+08	0.046272
magenta	GO:000211	nematode larval development	1E+08	0.046272
magenta	GO:007134	skeletal muscle acetylcholine-gated channel clustering	1E+08	0.046272
magenta	GO:007027	RES complex	1E+08	0.046272
magenta	GO:001670	oxidoreductase activity, acting on paired donors, with incorporation or reduction o	1E+08	0.046272
magenta	GO:004214	retrograde transport, endosome to Golgi	1E+08	0.002128
magenta	GO:007258	clathrin-dependent endocytosis	1E+08	0.046272
magenta	GO:000979	embryo development ending in birth or egg hatching	1E+08	0.046272
magenta	GO:000741	axon guidance	1E+08	0.046272
magenta	GO:003001	Z disc	1E+08	0.046272
magenta	GO:000202	regulation of the force of heart contraction	1E+08	0.046272
magenta	GO:004300	neuron projection	1E+08	0.046272
magenta	GO:000650	proteolysis	10.30573	0.028224
magenta	GO:000550	iron ion binding	1E+08	0.046272
magenta	GO:004280	protein homodimerization activity	10.30573	0.028224
magenta	GO:003012	AP-2 adaptor complex	1E+08	0.046272
magenta	GO:003258	Golgi cisterna membrane	1E+08	0.046272
magenta	GO:004545	pole plasm oskar mRNA localization	1E+08	0.046272
magenta	GO:005077	positive regulation of dendrite morphogenesis	1E+08	0.046272
magenta	GO:190113	carbohydrate derivative biosynthetic process	1E+08	0.046272
magenta	GO:000865	rRNA (uridine-2'-O-)-methyltransferase activity	1E+08	0.046272
magenta	GO:001715	regulation of exocytosis	1E+08	0.046272
magenta	GO:000417	endopeptidase activity	1E+08	0.046272
magenta	GO:003313	acetylcholine receptor binding	1E+08	0.046272
magenta	GO:004520	synapse	1E+08	0.046272
magenta	GO:001518	L-cystine transmembrane transporter activity	1E+08	0.046272
magenta	GO:009855	cytoplasmic side of early endosome membrane	1E+08	0.046272
magenta	GO:000910	glycoprotein metabolic process	1E+08	0.046272
magenta	GO:003350	HULC complex	1E+08	0.046272
magenta	GO:000640	mRNA export from nucleus	1E+08	0.046272
magenta	GO:000905	macromolecule biosynthetic process	1E+08	0.046272
magenta	GO:001061	negative regulation of cardiac muscle hypertrophy	1E+08	0.046272
magenta	GO:004828	vesicle fusion with Golgi apparatus	1E+08	0.046272
magenta	GO:000568	U2 snRNP	1E+08	0.046272
magenta	GO:000077	adenyl-nucleotide exchange factor activity	1E+08	0.046272
red	GO:000647	protein acetylation	1E+08	0.017683
red	GO:003500	phosphatidylinositol 3-kinase activity	1E+08	0.017683
red	GO:000648	protein glycosylation	55.55	0.035059
red	GO:001602	membrane	3.560897	0.014777
red	GO:000961	response to wounding	1E+08	0.017683
red	GO:001607	RNA metabolic process	1E+08	0.017683
red	GO:001563	microtubule cytoskeleton	55.55	0.035059
red	GO:001711	nucleoside-triphosphatase activity	55.55	0.035059
red	GO:004476	developmental process	15.87143	0.010222
red	GO:004476	cellular process	6.172222	0.047646
red	GO:004443	cytoskeletal part	55.55	0.001804
red	GO:004323	protein-containing complex	55.55	0.035059
red	GO:004470	multicellular organismal process	22.22	0.0061
red	GO:001677	phosphotransferase activity, alcohol group as acceptor	1E+08	0.017683
red	GO:004423	cellular metabolic process	55.55	0.000101
red	GO:000679	phosphate-containing compound metabolic process	55.55	0.035059
red	GO:001656	covalent chromatin modification	1E+08	0.017683
**turquoise**	GO:190136	heterocyclic compound binding	6.438559	0.028628
**turquoise**	GO:004471	metabolic process	5.150847	0.042327
**turquoise**	GO:190156	organonitrogen compound metabolic process	1E+08	0.010858
**turquoise**	GO:000548	binding	3.090508	0.006493
**turquoise**	GO:004322	membrane-bounded organelle	4.905569	0.021203
**turquoise**	GO:001678	hydrolase activity	4.905569	0.021203
**turquoise**	GO:000815	metabolic process	6.867797	0.000222
**turquoise**	GO:009715	organic cyclic compound binding	6.438559	0.028628
**turquoise**	GO:004316	ion binding	5.723164	0.014679
**turquoise**	GO:001649	oxidoreductase activity	6.009322	0.000975
**turquoise**	GO:001682	lyase activity	17.16949	0.030319
**turquoise**	GO:007170	organic substance metabolic process	6.438559	0.028628
**turquoise**	GO:004423	primary metabolic process	4.905569	0.021203
**turquoise**	GO:000701	microtubule-based movement	25.75424	0.004158
yellow	GO:001684	phosphorus-oxygen lyase activity	1E+08	0.046861
yellow	GO:000150	skeletal system development	1E+08	0.046861
yellow	GO:000334	cilium movement	1E+08	0.046861
yellow	GO:007254	T-helper 17 cell lineage commitment	1E+08	0.046861
yellow	GO:001602	membrane	2.16956	0.038786
yellow	GO:000471	non-membrane spanning protein tyrosine kinase activity	1E+08	0.046861
yellow	GO:000471	protein serine/threonine/tyrosine kinase activity	1E+08	0.046861
yellow	GO:004409	positive regulation of molecular function	1E+08	0.046861
yellow	GO:000444	iodide peroxidase activity	1E+08	0.046861
yellow	GO:000660	NLS-bearing protein import into nucleus	1E+08	0.046861
yellow	GO:003004	actin filament polymerization	1E+08	0.046861
yellow	GO:007191	determination of liver left/right asymmetry	1E+08	0.046861
yellow	GO:000997	cyclase activity	1E+08	0.046861
yellow	GO:001656	protein ubiquitination	13.55975	0.019876
yellow	GO:001688	ATPase activity	8.135849	0.039235
yellow	GO:200031	regulation of T-helper 17 type immune response	1E+08	0.046861
yellow	GO:000661	ribosomal protein import into nucleus	1E+08	0.046861
yellow	GO:190223	regulation of endoplasmic reticulum stress-induced intrinsic apoptotic signaling pat	1E+08	0.046861
yellow	GO:000716	transmembrane receptor protein tyrosine kinase signaling pathway	1E+08	0.046861
yellow	GO:190293	phosphatidylinositol bisphosphate binding	1E+08	0.046861
yellow	GO:000377	actin binding	1E+08	0.046861
yellow	GO:009031	positive regulation of protein deacetylation	1E+08	0.046861
yellow	GO:000377	microtubule motor activity	20.33962	0.012302
yellow	GO:003439	nuclear periphery	1E+08	0.046861
yellow	GO:004815	tau protein binding	1E+08	0.046861
yellow	GO:005079	regulation of cellular process	3.874214	0.027203
yellow	GO:000926	cellular response to starvation	1E+08	0.046861
yellow	GO:004802	negative regulation of mRNA splicing, via spliceosome	1E+08	0.046861
yellow	GO:001810	peptidyl-threonine phosphorylation	1E+08	0.046861
yellow	GO:003101	pancreas development	1E+08	0.046861
yellow	GO:006157	Lys63-specific deubiquitinase activity	1E+08	0.046861
yellow	GO:001099	regulation of translational initiation by eIF2 alpha phosphorylation	1E+08	0.046861
yellow	GO:007261	interleukin-21 secretion	1E+08	0.046861
yellow	GO:003205	negative regulation of translation in response to stress	1E+08	0.046861
yellow	GO:001648	cytosolic transport	1E+08	0.046861
yellow	GO:000005	obsolete protein import into nucleus, docking	1E+08	0.046861
yellow	GO:000919	cyclic nucleotide biosynthetic process	1E+08	0.046861
yellow	GO:000551	protein binding	3.589345	0.009838
yellow	GO:004337	CD8-positive, alpha-beta T cell differentiation	1E+08	0.046861
yellow	GO:003374	peptide-methionine (R)-S-oxide reductase activity	1E+08	0.046861
yellow	GO:007260	interleukin-9 secretion	1E+08	0.046861
yellow	GO:001810	peptidyl-tyrosine phosphorylation	1E+08	0.046861
yellow	GO:190507	negative regulation of interleukin-17 secretion	1E+08	0.046861
yellow	GO:007194	cell periphery	1E+08	0.046861
yellow	GO:199038	Lys48-specific deubiquitinase activity	1E+08	0.046861
yellow	GO:000813	nuclear localization sequence binding	1E+08	0.046861
yellow	GO:003196	nuclear membrane	1E+08	0.046861
yellow	GO:004508	innate immune response	1E+08	0.046861
yellow	GO:004351	negative regulation of DNA damage response, signal transduction by p53 class media	1E+08	0.046861
yellow	GO:003649	regulation of translation in response to endoplasmic reticulum stress	1E+08	0.046861
yellow	GO:004362	protein self-association	1E+08	0.046861
